# Inter‐lab concordance of variant classifications establishes clinical validity of expanded carrier screening

**DOI:** 10.1111/cge.13582

**Published:** 2019-07-01

**Authors:** Kristjan E. Kaseniit, Elizabeth Collins, Christine Lo, Krista Moyer, Rebecca Mar‐Heyming, Hyunseok P. Kang, Dale Muzzey

**Affiliations:** ^1^ Myriad Women's Health (formerly Counsyl) South San Francisco California; ^2^ Myriad Genetics Salt Lake City Utah

**Keywords:** clinical validity, ClinVar, expanded carrier screening, variant classification, variant interpretation

## Abstract

Expanded carrier screening (ECS) panels that use next‐generation sequencing aim to identify pathogenic variants in coding and clinically relevant non‐coding regions of hundreds of genes, each associated with a serious recessive condition. ECS has established analytical validity and clinical utility, meaning that variants are accurately identified and pathogenic variants tend to alter patients' clinical management, respectively. However, the clinical validity of ECS, that is, correct discernment of whether an identified variant is indeed pathogenic, has only been shown for single conditions, not for panels. Here, we evaluate the clinical validity of a >170‐condition ECS panel by assessing concordance between >12 000 variant interpretations classified with guideline‐based criteria to their corresponding per‐variant combined classifications in ClinVar. We observe 99% concordance at the level of unique variants. A more clinically relevant frequency‐weighted analysis reveals that fewer than 1 in 500 patients are expected to receive a report with a variant that has a discordant classification. Importantly, gene‐level concordance is not diminished for rare ECS conditions, suggesting that large panels do not balloon the panel‐wide false‐positive rate. Finally, because ECS is intended to serve all reproductive‐age couples, we show that classification of novel variants is feasible and scales predictably for a large population.

## Graphical abstract

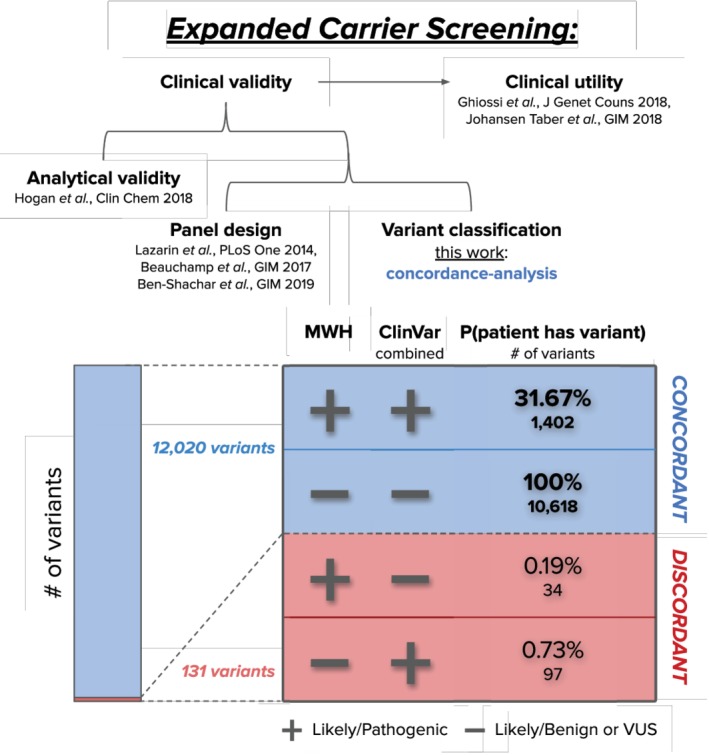

## 1 INTRODUCTION

A genetic test is described as having “clinical validity” if it yields a positive result when the clinical condition of interest is present and a negative result otherwise.^1^ In the context of expanded carrier screening (ECS), which tests for tens to hundreds of Mendelian conditions simultaneously, patients are not typically affected with the screened conditions; rather, they are most often asymptomatic carriers who are at high risk of having an affected child if their reproductive partners are also carriers for any of the same conditions.

For an ECS panel to be clinically valid, it must correctly identify pathogenic variants in the gene associated with each screened condition. This requirement can be broken down into three steps to assess whether it is satisfied: (a) demonstration that the screened genes are associated with the conditions of interest, (b) evaluation of whether the test accurately discovers variants in those genes (“analytical validity”), and (c) correct discernment of which variants are pathogenic and which are benign. The first step has been satisfied for many of the most prevalent conditions screened by ECS (eg, defects in the *CFTR*, *SMN1/2*, *FMR1*, and *HEXA* genes cause cystic fibrosis, spinal muscular atrophy, fragile X syndrome, and Tay Sachs Disease, respectively^2^, ^3^, ^4^, ^5^). Efforts are currently underway to apply established criteria^6^ for gene‐disease association to the less‐common conditions on larger ECS panels (manuscript in preparation). The second step, which describes the analytical validity of ECS, is well established in the literature.^7^ We recently validated that next‐generation sequencing (NGS) coupled with software‐assisted manual call review can detect single‐nucleotide variants (SNVs), short insertions and deletions (“indels”), and copy‐number variants (CNVs) with >99% sensitivity and specificity on a >170‐condition ECS panel.^8^, ^9^

The third step, correct discernment of variant pathogenicity, has been investigated for some commonly tested ECS conditions,^10^ but has not been well established for whole ECS panels. Nevertheless, it is critically important because the sequencing of full exons performed on many ECS offerings can discover novel variants whose pathogenicity must be assessed prior to reporting.^11^ Here and elsewhere, we refer to novel variants as those that have not been previously detected and classified by the observing institution. In an attempt to make variant interpretation more systematic, the American College of Medical Genetics and Genomics (ACMG) and the Association of Molecular Pathologists (AMP) issued joint guidelines that specify combinations of evidence (eg, enrichment in cases relative to controls, clinical impact in animal models, etc.) that can yield the following classifications: benign, likely benign, variant of uncertain significance (VUS), likely pathogenic, and pathogenic.^12^ These guidelines recommend that variant classification criteria be applied differently for ECS than for tests performed in an affected population: more stringent criteria must be met for pathogenicity because ECS patients are often asymptomatic, leading to rare variants with limited evidence often being classified as VUS. ECS laboratories typically only report pathogenic and likely pathogenic alleles (VUSs are not reported in ECS^11^).

One way to assess the proficiency of discerning variant pathogenicity is through comparison to the consensus among submitters to public databases like ClinVar^13^ (see Section 4). For instance, the clinical validity of hereditary cancer screening was explored through analysis of ClinVar concordance.^14^ Other studies of ClinVar data reveal inter‐lab disparity in variant classification that manifests as observed discordances.^13^, ^15^, ^16^ Because ClinVar submissions often include the evidence underlying each classification, it may be possible to adjudicate discordances and understand their origin (eg, laboratories performing different types of testing may weigh the age of disease onset differently).

We investigated the clinical validity of a >170‐condition ECS at the level of variant classification through concordance between our classifications and those from other laboratories with submissions in ClinVar. We count the number of variants with concordant or discordant interpretations, classify the reasons for discordance, calculate the frequency with which patients' reports contain a variant with disputed interpretation, and assess gene‐level concordance rates. Finally, because clinical uptake of ECS is growing and the number of variants requiring classification will increase proportionately, we explore the resources required to maintain the clinical validity of ECS as testing volume scales.

## 2 METHODS

### 2.1 Variant classification

We retrospectively queried variant classifications used internally at Myriad Women's Health (“MWH”; previously Counsyl, South San Francisco, California) for the Foresight ECS, which uses NGS of full exons or specialized assays to detect SNVs, indels, and CNVs in genes that cause 176 different recessive conditions. These classifications were generated in a manner consistent with the ACMG/AMP variant interpretation guidelines either manually or using software‐assisted classification (for variants without literature references), and classifications are routinely re‐evaluated as new data are obtained.

### 2.2 ClinVar submission filtering

MWH classifications were compared against those from the April 2018 release of ClinVar, which underwent filtering to remove artifacts. Classifications from submitters with fewer than 100 submissions for genes implicated in any of the 176 conditions of interest were excluded. Three of the 176 conditions (associated with mutations in *FMR1*, *HBA1/2*, *SMN1/2*) were excluded due to use of specialized assays that do not require interpretation of novel variants. Assertions with a “date last evaluated” before March 5th 2015 were ignored because they preceded publication of the ACMG/AMP variant interpretation guidelines. Additionally, assertions from before January 1st 2016 by one submitter were excluded after personal communication. Assertions by MWH were not included in the ClinVar dataset. Only rare variants (allele frequency ≤5%) were included in the analysis, as those with higher frequency are expected to be benign according to ACMG guidelines (with specific exceptions, for example, the NM_004004.5:c.109G>A(p.Val37Ile) variant in *GJB2* 17). ClinVar assertions explicitly mentioning that the interpretation was in the context of hereditary cancer‐predisposing syndromes were excluded. ClinVar assertions that could not be categorized as pathogenic, VUS, or benign (eg, “other”) were excluded. Variants where the interpretation depends on the presence of other variants were excluded. Variants that have always been observed in *cis* with other variants were excluded. The *ASL* gene did not have any ClinVar submissions passing the above filters and was not included in further analyses.

### 2.3 Concordance analysis

We evaluated and categorized differences between MWH and ClinVar classifications for the 172 genes of interest, simplifying assertions to “reportable” (eg, pathogenic or likely pathogenic) *vs* “not reportable” (eg, benign, likely benign, or VUS). ClinVar assertions were combined per variant using a majority rule. In the case of a tie, the variant was considered non‐reportable. The combined entry was used for concordance analysis. The latest MWH interpretations for variants for which either the combined ClinVar interpretation or the MWH interpretation at the time of this study was “reportable” have been submitted to ClinVar under the name Counsyl (https://www.ncbi.nlm.nih.gov/clinvar/submitters/320494/).^18^

ClinVar entries were classified as either concordant with MWH interpretations, or falling into one of seven discordance categories: (a) homozygotes observed in population studies; (b) unclear submitter classification; (c) no published cases; (d) adult onset; (e) dependent variant (where the interpretation depends on the presence of other variants); (f) reduced penetrance; (g) variable expressivity. Any discordant variant not falling into one of the seven categories was considered an “uncategorized discordance” and was considered to count toward false positives or false negatives (see below). Each discordant variant was evaluated by an expert variant interpreter to categorize the discordance.

Alleles were weighted by their population frequency (described below) to estimate the frequencies of different variant categories and to establish the respective rates at which a patient is expected to receive a report with concordant or discordant variants.

### 2.4 Cohort and allele frequency analysis

Variant observations in 265 189 patients with a “routine carrier screening” test indication sequenced between January 1st 2014 and April 18th 2018 at MWH were used for variant frequency analysis according to WIRB protocol #1‐1134598‐1. For genes on chromosome X, the variants were analyzed in the context of two chromosomes per individual (eg, screening women for carrier status).

### 2.5 Estimating clinical sensitivity, specificity, positive predictive value, and negative predictive value

The aggregate clinical sensitivity, specificity, positive predictive value (PPV), and negative predictive value (NPV) were estimated at the level of variant classification. To model the minimum of these values for our laboratory, we assumed that the combined ClinVar interpretations were always correct. Specifically, true positives were concordant reportable variants (ie, pathogenic and likely pathogenic); true negatives were concordant non‐reportable variants (ie, benign, likely benign, and VUS); false positives were variants that MWH considered reportable but combined ClinVar submissions considered non‐reportable, and false negatives were variants that MWH considered non‐reportable but combined ClinVar submissions considered reportable. When calculating sensitivity, specificity, PPV, and NPV, we used the aggregate probability of having a variant of each different type (eg, the carrier rate of true positive variants) rather than the number of such variants (eg, 42 true positives) to obtain clinically relevant performance values. The probabilities of individuals having variants in two or more categories were also evaluated and included in the performance calculations according to Table S1. Sensitivity and PPV could not be evaluated for 21 conditions due to the absence of any variants that were both (a) classified as reportable in ClinVar and (b) observed and classified in the MWH patient cohort. Similarly, specificity and NPV could not be evaluated for three conditions due to the absence of variants that were both (a) classified as non‐reportable in ClinVar and (b) observed and classified in the MWH patient cohort.

To establish the clinical performance of the combined ECS panel, the per‐gene performance metrics were combined in a weighted manner proportional to the gene's share of the panel. For example, the per‐gene sensitivity values were weighted by the approximate proportion of all carriers due to the given gene.

### 2.6 Variant interpretation load analysis

To assess the workload required to interpret novel variants, we estimated the rate of observing novel alleles in the patient population and measured the time needed to interpret new variants. First, we determined how many previously unobserved alleles were found in groups of 1000 consecutive patients and then calculated the rate of novel alleles per patient. For ease of representation of this fractional number, we multiplied the number by 100 to obtain the number of novel alleles per 100 patients. Second, we analyzed the self‐reported time expended on variant interpretation. Variant interpreters routinely provided the itemized time consumed for different parts of the variant interpretation process (searching databases, searching for literature, analyzing literature, taking notes, and other activities) using self‐reporting tools available in the variant‐interpretation interface. The self‐reported times were not inclusive of the whole variant‐interpretation process; for example, it did not include laboratory‐director review time.

## 3 RESULTS

**Figure 1:**
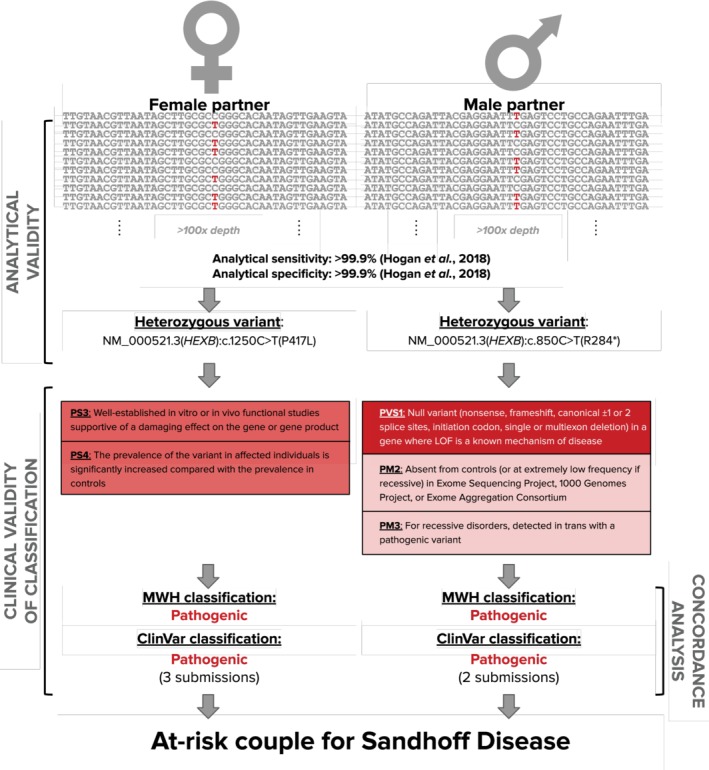
Variant interpretation case study. Expanded carrier screening typically employs next‐generation sequencing to identify variants in genes associated with severe and profound disorders to determine the risk status of reproductive couples for these diseases. A variant‐classification process based on American College of Medical Genetics and Genomics/Association of Molecular Pathologists guidelines is used to gather evidence for and against the pathogenicity of the variants. The clinical validity of variant classification can be analyzed through concordance with databases such as ClinVar [Colour figure can be viewed at http://wileyonlinelibrary.com]

Our approach to assessing the clinical validity of a >170‐condition ECS panel is best illustrated through an example (Figure 1). For each partner in a couple, NGS was used to discover variants (Figure 1, top). Importantly, in this study, we did not explore the efficacy of variant identification, as the analytical validity of ECS has been established previously.^8^ Instead we focused on variant interpretation, which follows a guideline‐based workflow to gather various forms of evidence that collectively yield a classification (Figure 1, middle). In particular, we performed an investigation of the concordance between our variant classifications and the combined classifications in ClinVar (see Section 4; Figure 1, bottom). Consistency of variant classifications across laboratories is important to verify because when both partners are carriers because of pathogenic variants in the same autosomal gene, they have a 25% risk of an affected child and often pursue alternative reproductive options.^19^

### 3.1 Most ECS variant interpretations are concordant

**Figure 2:**
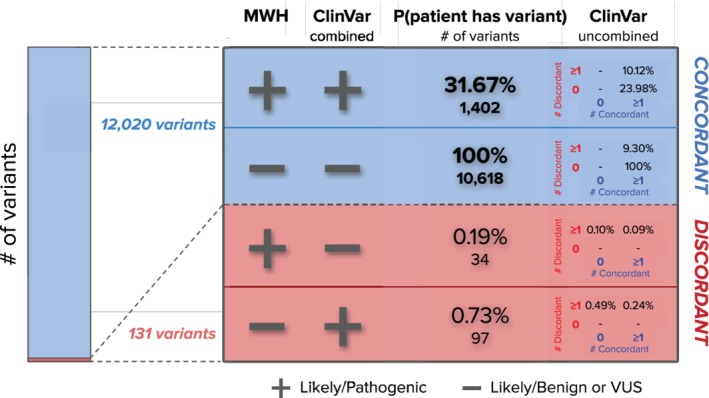
Foresight variant interpretations are highly concordant with ClinVar interpretations. Over 12 000 variants were analyzed for interpretation concordance with ClinVar submissions. The majority of variants were concordant. Discordant variants are expected to be carried by 0.91% of individuals, while concordant reportable variants are expected to be carried by 31.67% of individuals. The “ClinVar uncombined” column shows the carrier rate of concordant and discordant assessments from single submitters [Colour figure can be viewed at http://wileyonlinelibrary.com]

For 12 257 unique variants observed during routine ECS on 265 189 patients, we evaluated the concordance between classifications from MWH and ClinVar. Of these, 12 020 (98.1%) were concordant between the MWH interpretation and the combined ClinVar interpretation (Figure 2), and 131 (1.07%) variants were discordant with no clear explanation after expert review (described in more detail below). Table S2 lists details on the 12 151 variants, and Table S3 lists details on the remaining 106 variants for which discordances had a clear explanation. Of the concordant variants, 1402 variants were reportable (pathogenic or likely pathogenic); based on their frequencies, 31.67% of patients are expected to be carriers for at least one of these variants (Figure 2). The remaining 10 618 concordant variants were non‐reportable (benign, likely benign, or VUS), and 100% of patients are expected to be heterozygous for at least one such variant in the genome (note that VUSs are not reported in ECS). MWH was the only ClinVar submitter for 1006 reportable variants with a 1.86% aggregate carrier rate, but these variants were not included in subsequent analyses of concordance.

### 3.2 Discordant interpretations arise partly due to ECS‐specific evidence requirements

**Figure 3:**
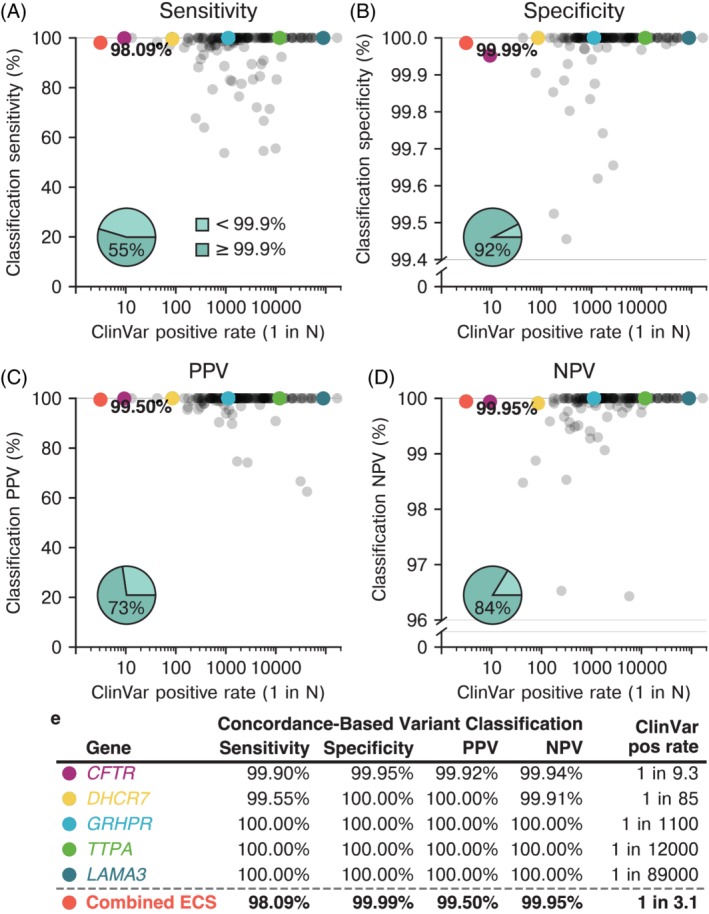
Clinical variant classification performance**.** The variant classification performance metrics were calculated based on the assumption that ClinVar classifications are correct and using a probabilistic approach accounting for the allele frequencies of variants under consideration. The “ClinVar positive rate” is the probability of carrying a variant analyzed here considered reportable by the combined ClinVar classifications. **(A)**, **(B), (C), (D)** Inset pie charts indicate the proportion of diseases above (dark teal) or below (light teal) a 99.9% threshold for the given metric with the number indicating the proportion of genes above the threshold. The subset of conditions that could not be evaluated (see Section 2) are indicated in the pie plots as being below the 99.9% threshold. (E) Example genes across a range of ClinVar positive rates [Colour figure can be viewed at http://wileyonlinelibrary.com]

We sought to elucidate common themes underlying the observed discordant variant classifications by categorizing their causes. For instance, because there is more of a premium on specificity in screening tests as compared to diagnostic tests, we expected that our ECS classification workflow would tend to favor non‐reportable VUS and benign/likely‐benign classifications relative to diagnostic tests that may have relatively more reportable pathogenic/likely‐pathogenic classifications. Of the 237 “raw discordances,” 76.8% were because of reportable assertions in ClinVar for variants that we did not consider meeting criteria for being reportable (ie, were considered VUS or benign/likely benign). After expert review, 44.7% of discordant variants had a clear explanation that warranted removal from further analyses in an ECS context: 25.7% of all discordances were because of variants with seemingly unreliable classifications based on sparse data and a hedged description (eg, an LP classification without case reports, based only on in silico analysis and low allele frequency, and accompanied by free‐text stating “[the variant] is a strong candidate for a disease‐causing variant, however, the possibility it may be a rare benign variant cannot be excluded”). Eleven percent were variants with no published cases and no other lines of evidence to support pathogenic classification, 3.4% were variants with homozygotes observed in the population (suggesting that the variant is either benign or low penetrance), and 4.6% were due to categories where reporting of variants in a carrier‐screening setting might not be appropriate compared to a diagnostic setting (eg, variants with an adult‐onset phenotype, variants whose pathogenicity is contingent on the presence or absence of a second variant in the same gene, and variants with reduced penetrance or variable expressivity). These variants with clearly explained discordances were not counted in the clinical performance analysis (Figures 2, 3) because they did not meet MWH's definition of ECS‐level evidence for pathogenicity or were not appropriate for reporting in ECS (see Table S3 for details on each of the 106 excluded variants). A remaining 131 (55.3%) of raw discordances could not be clearly categorized by an expert, the majority (74%) of those being cases where MWH did not consider there to be sufficient available evidence to interpret the variant as reportable (Figure 2) (eg, other labs may be privy to additional patient data that enable the reportable interpretation with confidence). These 131 discordances were used in further analyses.

### 3.3 The probability of carrying a variant with a legitimate interpretation difference is low, yielding high clinical sensitivity and PPV

We investigated the probability of an ECS patient receiving a report with at least one disputed variant call because prior exploration of variant discordance suggested that interpretation discordance was common.^13^ One or more of the 131 discordant ECS alleles are carried by 0.91% of individuals; put differently, >99% of patient reports do not contain a variant with disputed classification. While 0.19% of all individuals are expected to carry at least one of the 34 variants that we consider reportable but that are VUS or benign/likely benign in the combined ClinVar interpretation, the consensus in ClinVar is not unanimous: nine of these variants have at least one ClinVar submission concordant with our interpretation, accounting for 48% of the individuals with these types of discordant results (Figure 2, +/− row). Therefore, fewer than 1 in 1000 individuals carry a discordant reportable variant that was not also classified as reportable by another ClinVar submitter. Conversely, 0.73% carry at least one of the 97 discordant variants that MWH considers VUS or benign/likely benign; seven of these variants (carried by 33% of these individuals) have at least one concordant ClinVar submission (Figure 2, −/+ row).

To assess the clinical sensitivity, specificity, PPV, and NPV of MWH variant classifications, comparison to a truth set was needed. However, for discordant variants, it was unclear whether the interpretation from MWH or ClinVar was correct. Therefore, we approximated the worst‐case scenario for MWH by assuming that ClinVar is always the correct source of truth. Under this assumption, the aggregate clinical sensitivity of our ECS panel—based on variant interpretation concordance—is estimated to be >98%, the PPV >99%, the specificity >99.9% and the NPV >99.9% (Figure 3, red points). These data suggest that there is broad agreement among variant interpretations for an ECS panel with >170 genes.

We calculated the estimated clinical‐performance metrics individually for every gene on the panel to test whether interpretation efficacy decreases for rare conditions (Table S4). For a common disease like cystic fibrosis, the estimated clinical sensitivity was 99.90%, with PPV, specificity, and NPV all >99.9% (Figure 3), comparable to levels of clinical validity reported for hereditary cancer screening of *BRCA1/2*.^14^ The metrics were high for most genes individually, with 55% of genes having a sensitivity of >99.9%, 92% having a specificity of >99.9%, 73% having a PPV of >99.9%, and 84% having an NPV of >99.9% (Figure 3, pie charts). While 73% of genes had a sensitivity of >95%, 15% of genes had a sensitivity between 54% and 95% due to a small number of relatively high‐frequency discordant variants that consequently have large impact on the calculation of gene‐level sensitivity. In addition, 12% of genes could not be analyzed for sensitivity due to no reportable ClinVar variants observed by another laboratory that passed our filtering criteria (see Section 2). Notably, performance did not diminish for rare disorders: it remained high across the range of carrier rates (Figure 3).

### 3.4 Variant interpretation can be performed at scale

**Figure 4:**
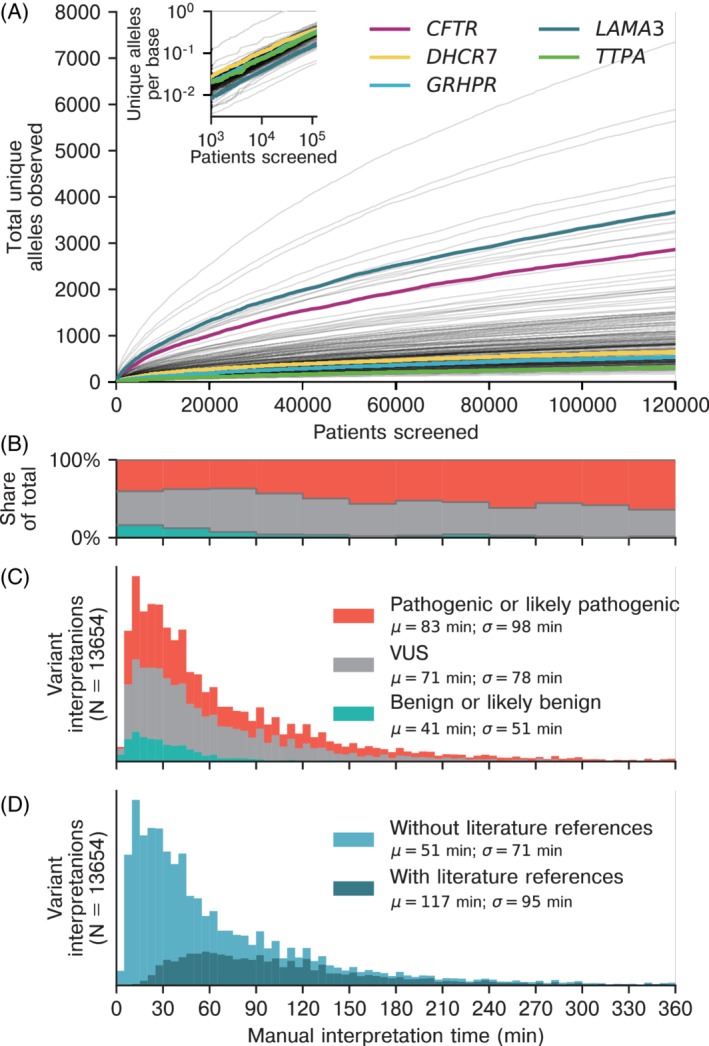
Variant interpretation at scale. (A) The total number of unique alleles observed as a function of the number of patients sequenced. Inset shows the same data on a log‐log scale with the total number of alleles normalized by the coding length of the gene. (B), (C) Benign and likely benign variants tended to take less time to interpret compared to variant of uncertain significance, pathogenic, or likely pathogenic variants, while time‐consuming interpretations were mostly for reportable variants (ie, pathogenic or likely pathogenic). (D) Variants with literature references took a longer and more varied time to interpret compared to variants without literature references [Colour figure can be viewed at http://wileyonlinelibrary.com]

NGS‐based ECS with interpretation of novel variants is only clinically viable for a laboratory if its classification workflow can handle a large volume of tests and has well‐understood behavior over time. Therefore, we next investigated the relationship between the number of patients tested and novel variants identified, as well as the drivers of time needed for classification. The number of observed novel variants exhibited a power‐law‐type behavior (Figure 4A) as evidenced by the straight lines when graphed on a log‐log scale (Figure 4A**, inset**). The decreasing rate of novel alleles observed as more patients are screened can facilitate ECS laboratory workflow in two manners: (a) laboratories can pre‐interpret a set of the more common variants in order to obtain a reduced and predictable rate of novel alleles as patients undergo screening, and (b) laboratories can better predict the labor required for ongoing variant interpretation.

The time required to interpret variants was tracked as part of the classification‐software interface. Each operator followed a standardized operating procedure for the variant‐interpretation process, and each interpretation required approval by a certified clinical molecular geneticist. The mean time to interpret a variant was 1 hour and 13 minutes (median time 45 minutes) based on self‐reported timing by the operators. The largest contribution to this time was the identification and analysis of literature related to the variant in question. Generally, benign/likely benign variants tended to consume less time for interpretation than VUS or reportable variants did (Figure 4B, C). Variants with literature references tended to take more time to interpret than those without such evidence (Figure 4D), with the time also being more variable.

## 4 DISCUSSION

Here we evaluated a key aspect of ECS clinical validity: the correct discernment of variant pathogenicity. Following the convention for this type of analysis, we assessed concordance with respect to ClinVar and found that 99% of evaluated variants for a >170‐gene ECS had concordant classifications in our database and ClinVar. Discordant variants were rare and also had low frequency; indeed, fewer than 1 in 1000 patients would be expected to receive a report with a reportable variant not also considered reportable by at least one other established ClinVar submitter. Elevated classification discordance for low‐frequency variants is expected, as they are likely to have less available evidence for pathogenicity, for example, fewer literature case reports, or laboratory‐specific access to unpublished data. Discordances were more common among variants classified as reportable in ClinVar but VUS, benign, or likely benign (ie, non‐ reportable) by our laboratory, consistent with an adaptation of ACMG criteria that accounts for screening a large asymptomatic population.

**Figure 5:**
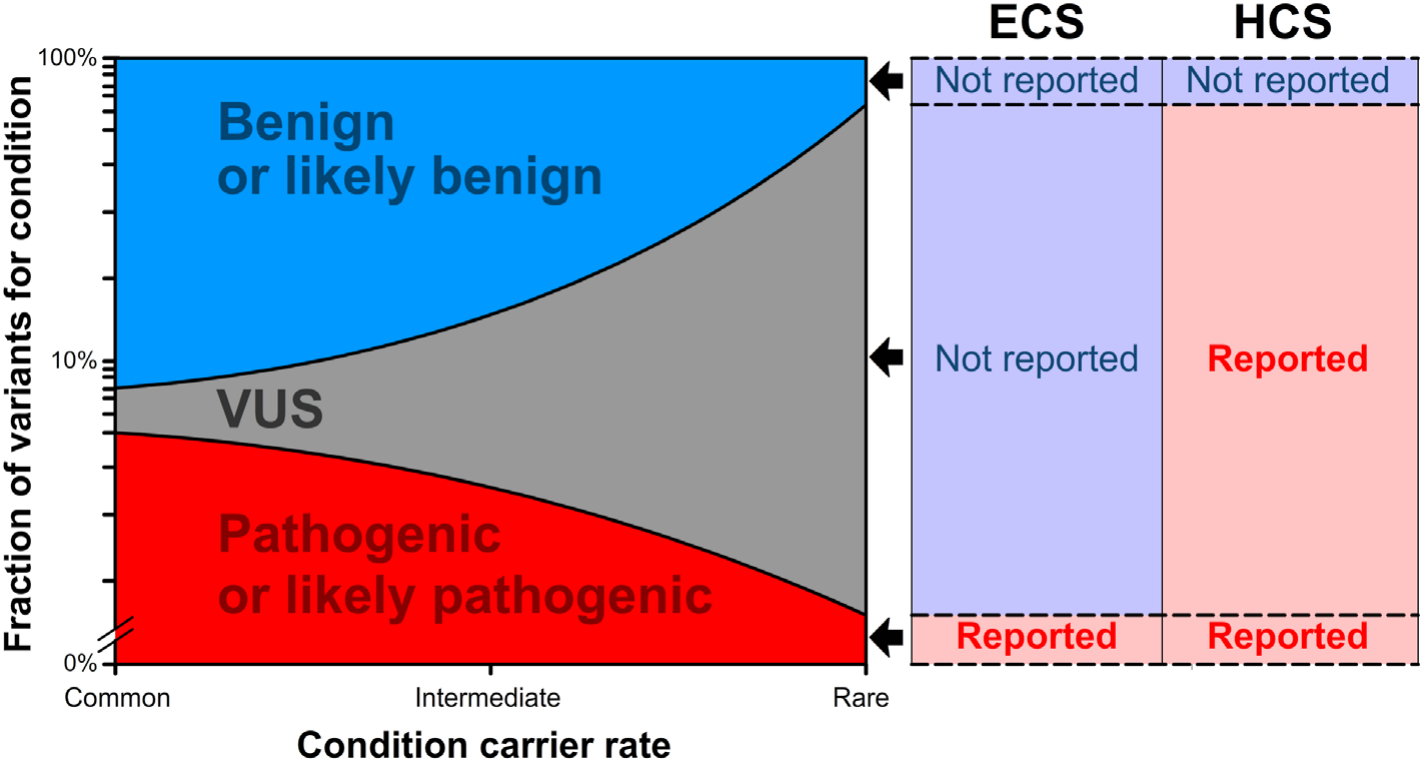
Schematic of how the number of reported variants scales with panel size if progressively rarer conditions are added. The schematic illustrates the general relationship between carrier rate and variant classifications (left), as well as the types of variants reported for expanded carrier screening (ECS) and hereditary cancer screening (“HCS”; right). For progressively rarer conditions, variant of uncertain significance (VUS) classifications become more common because evidence is too scarce to yield pathogenic, likely‐pathogenic, benign or likely‐benign classifications. For HCS, where VUSs are reported, the fraction of variants that are reportable for a given disease is expected to rise. In contrast, as rarer diseases are added to an ECS panel, the fraction of reportable variants for each marginal disease is expected to fall because only those variants that have sufficient evidence to yield a pathogenic or likely pathogenic classification are reported. Note that the *y*‐axis in this schematic is on a log scale [Colour figure can be viewed at http://wileyonlinelibrary.com]

The transformation of ECS in recent years—expansion of panel size and transition from genotyping to NGS technologies—has engendered concern that ECS panels will yield an abundance of false positives that unnecessarily cause anxiety for patients, increase clinical burden for providers, and escalate testing costs for payers. This intuition fails to account for three mitigating factors specific to ECS: (a) each severe disease added to an ECS panel tends to have progressively lower carrier frequency, (b) a higher level of evidence for pathogenicity is required for interpretation in ECS than in other screening contexts, and (c) VUSs are not reported in ECS. Consequently, variants in low‐frequency diseases will likely not have spurious evidence sufficient to yield a pathogenic or likely pathogenic classification (eg, from observations in affected patients), and are more likely to fall into the non‐reportable VUS category. Indeed, the high frequency of a variant relative to the incidence of a disease may be used as evidence against pathogenicity in the ACMG variant‐interpretation framework, so misclassified variants in low‐prevalence diseases are not likely to significantly increase the number of incorrect reports. In Figure 5, we provide a schematic for the expected scaling relationship between reported variants and panel size if progressively rarer conditions are added to HCS and ECS panels. Whereas HCS panel size must be mindful of reporting of VUSs, ECS panel size should not be dictated by a concern over the aggregate specificity of the test as it is expected to be high (see above), but rather by the point at which marginal sensitivity for each additional gene becomes insufficient.^20^

A limitation of the current work is that classification concordance with ClinVar is an imperfect proxy for correct variant interpretation. For instance, classifications for a variant could be 100% concordant but also clinically incorrect (eg, a variant with the pathogenic effect that all laboratories have classified as being benign). Furthermore, it would be trivially possible for a laboratory that lacks a variant‐interpretation system altogether to achieve 100% concordance simply by duplicating the classifications in ClinVar. Ideally, pathogenicity is assessed through identification of enrichment of the variant in cases, depletion in controls, impact in animal models, supporting functional assays, and more. But, these are precisely the factors incorporated into the ACMG criteria; thus, the burden of clinical‐validity proof shifts to establishing that laboratories consistently applied these criteria, which—coming full circle, and as demonstrated here—can be evaluated by concordance among independently generated classifications.

In this study, we differentiated between “raw discordances” and final discordances, and this analysis required the involvement of a variant‐interpretation expert. For instance, we observed 61 discordances involving unclear ClinVar submissions that would be hard to parse in an automated manner (eg, where a variant's classification and description were mutually inconsistent); therefore, reconciliation by a trained expert was needed to generate a final accounting of concordant and discordant variants. Further, discordances were excluded when there was a clear difference in the level of evidence required to consider a variant reportable in ECS or in an affected population, with the latter commonly the case for the many diagnostic laboratories that submit to ClinVar. The differences in patient population for various laboratories are often challenging to programmatically identify (such annotation in ClinVar would facilitate these analyses), necessitating the involvement of an expert.

Widespread clinical uptake of ECS panels that use NGS to interrogate many genes could oversubscribe variant‐interpretation resources if the determinants of the time required for classification are poorly understood. We showed that the demand for interpretation of novel variants can be anticipated from the size of the test's region of interest and/or the variant frequencies observed in a relatively small sample population. Many variants could be enumerated and interpreted in advance of clinical testing to buffer against fluctuations in the workload of expert interpreters. Automated tools, for example, for database and literature searches, further streamline variant interpretation such that most variants can be interpreted by an expert in less than an hour using an SOP. Additionally, many variants can be interpreted using the SOP through automated methods, for example, in the case of common intronic variants without literature references. Our results show that variant interpretation can be scaled to population‐screening levels without sacrificing clinical performance.

Based on the variant‐interpretation concordance analyses presented here and in several previous studies describing the genes selected for inclusion on the panel,^6^, ^21^, ^22^ we conclude that the ECS investigated here has sufficient clinical validity for widespread screening.

## CONFLICT OF INTEREST

All authors are former employees of Counsyl, a company providing ECS. Since the acquisition of Counsyl by Myriad, the Counsyl ECS is offered by Myriad Women's Health, whose employees include the co‐authors Elizabeth Collins, Krista Moyer, Rebecca Mar‐Heyming, and Hyunseok P. Kang. Co‐authors Kristjan E. Kaseniit, Christine Lo, and Dale Muzzey are employed by Myriad.

5

## Supporting information


**TABLE S1** Details of sensitivity, specificity, positive predictive value, and negative predictive value calculations

**TABLE S2** Details of the 12 151 variants used in the clinical variant classification performance analysis

**TABLE S3** Details of the 106 variants excluded from the clinical variant classification performance analysis

**TABLE S4** Per‐gene clinical variant classification performance

## Data Availability

The data that support the findings of the study are openly available as part of the supplemental materials. Variant interpretations are also submitted on an ongoing basis to ClinVar (submitter Counsyl, https://www.ncbi.nlm.nih.gov/clinvar/submitters/320494/).
