# Supplementary material for: Inter‐lab concordance of variant classifications establishes clinical validity of expanded carrier screening
Source: Clin Genet. 2019 Jul 1;96(3):236–45. doi: 10.1111/cge.13582 (PMC6852020; doi:10.1111/cge.13582)
Supplement: Supplementary file 2 — TABLE S2 Details of the 12 151 variants used in the clinical variant classification performance analysis [file CGE-96-236-s002.pdf]

Supplemental Table 2

standard_name	category	MWH	ClinVar	clinvar_id	n_reportable_cv	n_unreportable_cv	accession	raw_clinical_significance	submitter	submission_date	gene	chrom	start	stop	gnomad_link	allele_frequency	gnomad_POPMAX	gnomad_AF_POPMAX	gnomad_Hom	gnomad_Hemi	MWH_Hom	MWH_Hemi	clinvar_link
NM_000352.3(ABCC8):c.4178G>A	discordant	unreportable	reportable	446776	1	0	SCV000612218	Likely pathogenic	Athena Diagnostics	8/17/2017	ABCC8	chr11	17417419	17417419		3.10E-05					0	0	https://www.ncbi.nlm.nih.gov/clinvar/variation/446776/
NM_000017.2(ACADS):c.1156C>T	discordant	unreportable	reportable	203566	1	0	SCV000238624	Likely pathogenic	GeneDx	11/28/2017	ACADS	chr12	121177168	121177168	http://gnomad.b	2.55E-05	SAS	0.0001951979939	0		0	0	https://www.ncbi.nlm.nih.gov/clinvar/variation/203566/
NM_000017.2(ACADS):c.1112G>T	discordant	unreportable	reportable	203563	1	0	SCV000238618	Pathogenic	GeneDx	11/28/2017	ACADS	chr12	121177124	121177124		1.42E-05					0	0	https://www.ncbi.nlm.nih.gov/clinvar/variation/203563/
NM_000018.3(ACADVL):c.1375C>T	discordant	unreportable	reportable	203584	1	0	SCV000602370	Likely pathogenic	ARUP Laboratories	6/30/2017	ACADVL	chr17	7127329	7127329	http://gnomad.b	8.50E-06	AMR	8.93E-05	0		0	0	https://www.ncbi.nlm.nih.gov/clinvar/variation/203584/
NM_000018.3(ACADVL):c.637G>A	discordant	unreportable	reportable	198683	1	0	SCV000406315	Likely pathogenic	Illumina Clinical Se	10/18/2016	ACADVL	chr17	7125285	7125285	http://gnomad.b	2.83E-05	AMR	5.96E-05	0		0	0	https://www.ncbi.nlm.nih.gov/clinvar/variation/198683/
NM_000018.3(ACADVL):c.1096C>T	discordant	unreportable	reportable	203579	1	0	SCV000602375	Pathogenic	ARUP Laboratories	6/30/2017	ACADVL	chr17	7126470	7126470	http://gnomad.b	1.70E-05	AFR	6.53E-05	0		0	0	https://www.ncbi.nlm.nih.gov/clinvar/variation/203579/
NM_000018.3(ACADVL):c.1468G>C	discordant	reportable	unreportable	474888	0	1	SCV000654935	Uncertain significance	Invitae	10/5/2017	ACADVL	chr17	7127498	7127498	http://gnomad.b	1.70E-05	NFE	1.79E-05	0		0	0	https://www.ncbi.nlm.nih.gov/clinvar/variation/474888/
NM_000022.2(ADA):c.58G>A	discordant	unreportable	reportable	68267	1	0	SCV000582055	Likely pathogenic	GeneDx	11/28/2017	ADA	chr20	43264905	43264905		1.60E-05					0	0	https://www.ncbi.nlm.nih.gov/clinvar/variation/68267/
NM_000478.4(ALPL):c.815G>A	discordant	unreportable	reportable	495882	1	0	SCV000696803	Likely pathogenic	Laboratory Corpora	1/25/2018	ALPL	chr1	21896820	21896820	http://gnomad.b	2.82E-06	SAS	3.25E-05	0		0	0	https://www.ncbi.nlm.nih.gov/clinvar/variation/495882/
NM_000487.5(ARSA):c.979G>A	discordant	unreportable	reportable	372303	1	0	SCV000490407	Pathogenic	GeneDx	12/21/2016	ARSA	chr22	51064582	51064582	http://gnomad.b	1.41E-05	AFR	7.61E-05	0		0	0	https://www.ncbi.nlm.nih.gov/clinvar/variation/372303/
NM_000487.5(ARSA):c.1174C>T	discordant	reportable	unreportable	3085	0	1	SCV000330911	Uncertain significance	EGL Genetic Diagn	6/30/2017	ARSA	chr22	51064043	51064043	http://gnomad.b	1.97E-05	SAS	6.50E-05	0		0	0	https://www.ncbi.nlm.nih.gov/clinvar/variation/3085/
NM_000050.4(ASS1):c.929A>G	discordant	unreportable	reportable	365260	2	1	SCV000602548;S	Likely pathogenic;Likely pa	ARUP Laboratories	2017-06-30;2016-1	ASS1	chr9	133364810	133364810	http://gnomad.b	1.11E-05	NFE	4.48E-05	0		0	0	https://www.ncbi.nlm.nih.gov/clinvar/variation/365260/
NM_000050.4(ASS1):c.805G>A	discordant	unreportable	reportable	193968	1	0	SCV000593462	Likely pathogenic	Genetic Services La	7/5/2017	ASS1	chr9	133355803	133355803	http://gnomad.b	3.34E-05	NFE	0.0001074829997	0		0	0	https://www.ncbi.nlm.nih.gov/clinvar/variation/193968/
NM_000051.3(ATM):c.7926A>C	discordant	unreportable	reportable	215980	2	0	SCV000520897;S	Likely pathogenic;Likely pa	GeneDx;Invitae	2017-11-28;2015-0	ATM	chr11	108203626	108203626		5.57E-06					0	0	https://www.ncbi.nlm.nih.gov/clinvar/variation/215980/
NM_000051.3(ATM):c.9023G>A	discordant	unreportable	reportable	141634	3	0	SCV000687872;S	Likely pathogenic;Likely pa	Color Genomics, In	2017-12-21;2017-1	ATM	chr11	108236087	108236087	http://gnomad.b	8.35E-06	FIN	4.48E-05	0		0	0	https://www.ncbi.nlm.nih.gov/clinvar/variation/141634/
NM_000051.3(ATM):c.2849T>G	discordant	unreportable	reportable	186574	1	0	SCV000321411	Likely pathogenic	GeneDx	11/28/2017	ATM	chr11	108141801	108141801		5.57E-06					0	0	https://www.ncbi.nlm.nih.gov/clinvar/variation/186574/
NM_000053.3(ATP7B):c.2905C>T	discordant	unreportable	reportable	381534	1	0	SCV000520884	Likely pathogenic	GeneDx	11/28/2017	ATP7B	chr13	52520575	52520575	http://gnomad.b	3.65E-05	SAS	9.75E-05	0		0	0	https://www.ncbi.nlm.nih.gov/clinvar/variation/381534/
NM_000053.3(ATP7B):c.2827G>A	discordant	unreportable	reportable	3856	1	0	SCV000694432	Pathogenic	Laboratory Corpora	1/25/2018	ATP7B	chr13	52523836	52523836	http://gnomad.b	1.40E-05	AFR	6.55E-05	0		0	0	https://www.ncbi.nlm.nih.gov/clinvar/variation/3856/
NM_000053.3(ATP7B):c.3452G>A	discordant	reportable	unreportable	495415	0	1	SCV000694447	Uncertain significance	Laboratory Corpora	1/25/2018	ATP7B	chr13	52515321	52515321	http://gnomad.b	8.42E-06	AFR	6.54E-05	0		0	0	https://www.ncbi.nlm.nih.gov/clinvar/variation/495415/
NM_000053.3(ATP7B):c.1877G>C	discordant	reportable	unreportable	157930	1	1	SCV000589556;S	Uncertain significance;Path	GeneDx;Laborator	2017-11-28;2018-0	ATP7B	chr13	52536042	52536042	http://gnomad.b	0.0001262654605	ASJ	0.000913706026	0		0	0	https://www.ncbi.nlm.nih.gov/clinvar/variation/157930/
NM_000053.3(ATP7B):c.3992A>C	discordant	unreportable	reportable	430024	1	0	SCV000582735	Likely pathogenic	GeneDx	11/28/2017	ATP7B	chr13	52511441	52511441		2.81E-06					0	0	https://www.ncbi.nlm.nih.gov/clinvar/variation/430024/
NM_000709.3(BCKDHA):c.1037G>A	discordant	unreportable	reportable	93336	1	0	SCV000232574	Likely pathogenic	EGL Genetic Diagn	6/30/2017	BCKDHA	chr19	41928944	41928944		2.37E-05					0	0	https://www.ncbi.nlm.nih.gov/clinvar/variation/93336/
NM_183050.2(BCKDHB):c.503G>A	discordant	unreportable	reportable	457149	1	0	SCV000627819	Likely pathogenic	Invitae	10/5/2017	BCKDHB	chr6	80878617	80878617		1.03E-05					0	0	https://www.ncbi.nlm.nih.gov/clinvar/variation/457149/
NM_000060.2(BTD):c.469C>T	discordant	unreportable	reportable	25012	1	0	SCV000491032	Likely pathogenic	GeneDx	12/21/2016	BTD	chr3	15685832	15685832	http://gnomad.b	1.15E-05	AFR	6.55E-05	0		0	0	https://www.ncbi.nlm.nih.gov/clinvar/variation/25012/
NM_000071.2(CBS):c.700G>A	discordant	unreportable	reportable	212852	2	0	SCV000249690;S	Pathogenic;Pathogenic	GeneDx;Laborator	2017-11-28;2018-0	CBS	chr21	44485349	44485349	http://gnomad.b	8.44E-06	SAS	6.50E-05	0		0	0	https://www.ncbi.nlm.nih.gov/clinvar/variation/212852/
NM_000492.3(CFTR):c.1853T>C	discordant	reportable	unreportable	53404	1	1	SCV000330920;S	Pathogenic;Uncertain signi	EGL Genetic Diagn	2017-06-30;2018-0	CFTR	chr7	117232074	117232074	http://gnomad.b	4.62E-05	AFR	0.0003361119889	0		0	0	https://www.ncbi.nlm.nih.gov/clinvar/variation/53404/
NM_000492.3(CFTR):c.330C>A	discordant	unreportable	reportable	53714	2	0	SCV000708644;S	Pathogenic;Pathogenic	EGL Genetic Diagn	2017-06-30;2018-0	CFTR	chr7	117171009	117171009	http://gnomad.b	1.16E-05	NFE	2.69E-05	0		0	0	https://www.ncbi.nlm.nih.gov/clinvar/variation/53714/
NM_000492.3(CFTR):c.4426C>T	discordant	unreportable	reportable	53947	2	0	SCV000602994;S	Pathogenic;Pathogenic	ARUP Laboratories	2017-06-30;2018-0	CFTR	chr7	117307145	117307145	http://gnomad.b	3.85E-05	AFR	6.54E-05	0		0	0	https://www.ncbi.nlm.nih.gov/clinvar/variation/53947/
NM_006493.2(CLN5):c.1121A>G	discordant	unreportable	reportable	2570	1	0	SCV000581862	Likely pathogenic	GeneDx	11/28/2017	CLN5	chr13	77575001	77575001	http://gnomad.b	3.36E-05	NFE	1.90E-05	0		0	0	https://www.ncbi.nlm.nih.gov/clinvar/variation/2570/
NM_018941.3(CLN8):c.470A>G	discordant	unreportable	reportable	210736	1	0	SCV000247046	Likely pathogenic	Genetic Services La	9/15/2015	CLN8	chr8	1719690	1719690	http://gnomad.b	2.24E-05	NFE	2.69E-05	0		0	0	https://www.ncbi.nlm.nih.gov/clinvar/variation/210736/
NM_018941.3(CLN8):c.209G>A	discordant	reportable	unreportable	56704	0	1	SCV000549256	Uncertain significance	Invitae	3/14/2017	CLN8	chr8	1719429	1719429	http://gnomad.b	2.80E-06	EAS	0.0001159550011	0		0	0	https://www.ncbi.nlm.nih.gov/clinvar/variation/56704/
NM_000098.2(CPT2):c.680C>T	discordant	reportable	unreportable	8964	1	1	SCV000331529;S	Uncertain significance;Path	EGL Genetic Diagn	2017-06-30;2017-1	CPT2	chr1	53676026	53676026	http://gnomad.b	0.000100731422	AFR	0.0007875050069	0		0	0	https://www.ncbi.nlm.nih.gov/clinvar/variation/8964/
NM_001360.2(DHCR7):c.970T>C	discordant	unreportable	reportable	458678	1	0	SCV000630078	Pathogenic	Invitae	10/5/2017	DHCR7	chr11	71146879	71146879	http://gnomad.b	8.08E-06	AFR	7.12E-05	0		0	0	https://www.ncbi.nlm.nih.gov/clinvar/variation/458678/
NM_000152.3(GAA):c.971C>T	discordant	unreportable	reportable	431990	1	0	SCV000589627	Likely pathogenic	GeneDx	11/28/2017	GAA	chr17	78082104	78082104	http://gnomad.b	2.22E-05	SAS	3.25E-05	0		0	0	https://www.ncbi.nlm.nih.gov/clinvar/variation/431990/
NM_000152.3(GAA):c.670C>T	discordant	reportable	unreportable	189188	0	1	SCV000339005	Uncertain significance	EGL Genetic Diagn	6/30/2017	GAA	chr17	78079671	78079671	http://gnomad.b	1.95E-05	NFE	3.62E-05	0		0	0	https://www.ncbi.nlm.nih.gov/clinvar/variation/189188/
NM_000153.3(GALC):c.331G>A	discordant	unreportable	reportable	211058	1	0	SCV000247446	Pathogenic	Genetic Services La	9/15/2015	GALC	chr14	88452944	88452944	http://gnomad.b	2.80E-06	AMR	5.97E-05	0		0	0	https://www.ncbi.nlm.nih.gov/clinvar/variation/211058/
NM_000155.3(GALT):c.552C>A	discordant	unreportable	reportable	25210	1	0	SCV000582003	Likely pathogenic	GeneDx	11/28/2017	GALT	chr9	34648156	34648156		5.50E-06					0	0	https://www.ncbi.nlm.nih.gov/clinvar/variation/25210/
NM_000155.3(GALT):c.772C>T	discordant	unreportable	reportable	92522	1	0	SCV000582974	Likely pathogenic	GeneDx	11/28/2017	GALT	chr9	34648843	34648843	http://gnomad.b	2.75E-05	NFE	6.27E-05	0		0	0	https://www.ncbi.nlm.nih.gov/clinvar/variation/92522/
NM_000155.3(GALT):c.958G>A	discordant	reportable	unreportable	25298	0	1	SCV000340778	Uncertain significance	EGL Genetic Diagn	6/30/2017	GALT	chr9	34649460	34649460	http://gnomad.b	8.25E-06	NFE	2.69E-05	0		0	0	https://www.ncbi.nlm.nih.gov/clinvar/variation/25298/
NM_000155.3(GALT):c.776G>A	discordant	unreportable	reportable	280989	2	0	SCV000330999;S	Likely pathogenic;Likely pa	EGL Genetic Diagn	2017-06-30;2018-0	GALT	chr9	34648847	34648847		8.25E-06					0	0	https://www.ncbi.nlm.nih.gov/clinvar/variation/280989/
NM_000155.3(GALT):c.100T>A	discordant	unreportable	reportable	25125	1	0	SCV000341275	Pathogenic	EGL Genetic Diagn	6/30/2017	GALT	chr9	34647103	34647103		2.75E-06					0	0	https://www.ncbi.nlm.nih.gov/clinvar/variation/25125/
NM_000155.3(GALT):c.601C>T	discordant	unreportable	reportable	25226	2	0	SCV000603796;S	Pathogenic;Pathogenic	ARUP Laboratories	2017-06-30;2017-0	GALT	chr9	34648367	34648367	http://gnomad.b	1.10E-05	EAS	5.80E-05	0		0	0	https://www.ncbi.nlm.nih.gov/clinvar/variation/25226/
NM_000155.3(GALT):c.379A>G	discordant	reportable	unreportable	25167	1	1	SCV000331278;S	Uncertain significance;Path	EGL Genetic Diagn	2017-06-30;2018-0	GALT	chr9	34647830	34647830		5.50E-06					0	0	https://www.ncbi.nlm.nih.gov/clinvar/variation/25167/
NM_000159.2(GCDH):c.395G>A	discordant	unreportable	reportable	377917	2	0	SCV000513123;S	Pathogenic;Likely pathoger	GeneDx;Invitae	2017-11-28;2017-1	GCDH	chr19	13004357	13004357		8.43E-06					0	0	https://www.ncbi.nlm.nih.gov/clinvar/variation/377917/
NM_000159.2(GCDH):c.1168G>C	discordant	unreportable	reportable	193799	1	0	SCV000521170	Pathogenic	GeneDx	11/28/2017	GCDH	chr19	13008602	13008602	http://gnomad.b	1.97E-05	AFR	6.54E-05	0		0	0	https://www.ncbi.nlm.nih.gov/clinvar/variation/193799/
NM_000159.2(GCDH):c.997C>G	discordant	unreportable	reportable	193561	1	0	SCV000521169	Likely pathogenic	GeneDx	11/28/2017	GCDH	chr19	13008157	13008157		5.62E-06					0	0	https://www.ncbi.nlm.nih.gov/clinvar/variation/193561/
NM_004004.5(GJB2):c.571T>C	discordant	unreportable	reportable	44760	1	0	SCV000603821	Likely pathogenic	ARUP Laboratories	6/30/2017	GJB2	chr13	20763150	20763150	http://gnomad.b	0.0001927902051	EAS	0.002029220108	0		0	0	https://www.ncbi.nlm.nih.gov/clinvar/variation/44760/
NM_001128227.2(GNE):c.1891G>A	discordant	reportable	unreportable	286014	1	1	SCV000339267;S	Uncertain significance;Likel	EGL Genetic Diagn	2017-06-30;2017-1	GNE	chr9	36219853	36219853		8.40E-06					0	0	https://www.ncbi.nlm.nih.gov/clinvar/variation/286014/
NM_000191.2(HMGCL):c.602C>A	discordant	unreportable	reportable	449033	1	0	SCV000616740	Pathogenic	GeneDx	11/28/2017	HMGCL	chr1	24134773	24134773	http://gnomad.b	8.00E-06	FIN	8.97E-05	0		0	0	https://www.ncbi.nlm.nih.gov/clinvar/variation/449033/
NM_000203.3(IDUA):c.1091C>T	discordant	unreportable	reportable	11925	1	0	SCV000695965	Pathogenic	Laboratory Corpora	1/25/2018	IDUA	chr4	996175	996175		5.55E-06					0	0	https://www.ncbi.nlm.nih.gov/clinvar/variation/11925/
NM_002225.3(IVD):c.605G>T	discordant	unreportable	reportable	3563	1	0	SCV000521033	Pathogenic	GeneDx	11/28/2017	IVD	chr15	40703799	40703799	http://gnomad.b	8.41E-06	NFE	1.79E-05	0		0	0	https://www.ncbi.nlm.nih.gov/clinvar/variation/3563/
NM_000243.2(MEFV):c.800C>T	discordant	unreportable	reportable	2544	1	0	SCV000696080	Pathogenic	Laboratory Corpora	1/25/2018	MEFV	chr16	3304268	3304268	http://gnomad.b	0.0001903162608	SAS	0.0003248649882	0		0	0	https://www.ncbi.nlm.nih.gov/clinvar/variation/2544/
NM_052845.3(MMAB):c.568C>T	discordant	unreportable	reportable	96244	1	0	SCV000700308	Pathogenic	EGL Genetic Diagn	6/30/2017	MMAB	chr12	109998861	109998861	http://gnomad.b	8.00E-06	NFE	2.69E-05	0		0	0	https://www.ncbi.nlm.nih.gov/clinvar/variation/96244/
NM_000271.4(NPC1):c.1210C>T	discordant	unreportable	reportable	499939	1	0	SCV000705676	Likely pathogenic	EGL Genetic Diagn	6/30/2017	NPC1	chr18	21136323	21136323		2.86E-06					0	0	https://www.ncbi.nlm.nih.gov/clinvar/variation/499939/
NM_000277.1(PAH):c.805A>C	discordant	unreportable	reportable	102842	1	0	SCV000330984	Pathogenic	EGL Genetic Diagn	6/30/2017	PAH	chr12	103246630	103246630	http://gnomad.b	0.0001232829426	AMR	0.0001787200017	0		0	0	https://www.ncbi.nlm.nih.gov/clinvar/variation/102842/
NM_000277.1(PAH):c.520A>G	discordant	reportable	unreportable	102720	0	1	SCV000203187	Uncertain significance	EGL Genetic Diagn	6/30/2017	PAH	chr12	103249100	103249100		1.37E-05					0	0	https://www.ncbi.nlm.nih.gov/clinvar/variation/102720/
NM_000277.1(PAH):c.283A>T	discordant	unreportable	reportable	102645	1	0	SCV000329447	Pathogenic	GeneDx	11/28/2017	PAH	chr12	103288582	103288582	http://gnomad.b	7.12E-05	ASJ	0.0005076139933	0		0	0	https://www.ncbi.nlm.nih.gov/clinvar/variation/102645/
NM_000277.1(PAH):c.464G>C	discordant	reportable	unreportable	102687	0	1	SCV000230889	Uncertain significance	EGL Genetic Diagn	6/30/2017	PAH	chr12	103260419	103260419		2.74E-06					0	0	https://www.ncbi.nlm.nih.gov/clinvar/variation/102687/
NM_138694.3(PKHD1):c.8411T>A	discordant	unreportable	reportable	197958	1	0	SCV000332131	Likely pathogenic	EGL Genetic Diagn	6/30/2017	PKHD1	chr6	51656063	51656063		5.56E-06					0	0	https://www.ncbi.nlm.nih.gov/clinvar/variation/197958/
NM_138694.3(PKHD1):c.5372C>T	discordant	unreportable	reportable	283652	1	0	SCV000335884	Likely pathogenic	EGL Genetic Diagn	6/30/2017	PKHD1	chr6	51887607	51887607		2.78E-06					0	0	https://www.ncbi.nlm.nih.gov/clinvar/variation/283652/
NM_003060.3(SLC22A5):c.845G>A	discordant	unreportable	reportable	25401	1	0	SCV000490805	Pathogenic	GeneDx	12/21/2016	SLC22A5	chr5	131722737	131722737		3.11E-05					0	0	https://www.ncbi.nlm.nih.gov/clinvar/variation/25401/
NM_003060.3(SLC22A5):c.1412G>A	discordant	unreportable	reportable	25426	1	0	SCV000632530	Pathogenic	Invitae	10/5/2017	SLC22A5	chr5	131728269	131728269	http://gnomad.b	5.65E-06			0		0	0	https://www.ncbi.nlm.nih.gov/clinvar/variation/25426/
NM_003060.3(SLC22A5):c.364G>T	discordant	unreportable	reportable	25371	2	1	SCV000224396;S	Pathogenic;Pathogenic;Un	EGL Genetic Diagn	2017-06-30;2017-1	SLC22A5	chr5	131706028	131706028	http://gnomad.b	0.0004772878752	NFE	0.0006171200075	0		0	0	https://www.ncbi.nlm.nih.gov/clinvar/variation/25371/
NM_000543.4(SMPD1):c.742G>A	discordant	unreportable	reportable	496823	1	0	SCV000700336	Pathogenic	EGL Genetic Diagn	6/30/2017	SMPD1	chr11	6413037	6413037	http://gnomad.b	7.14E-06			0		0	0	https://www.ncbi.nlm.nih.gov/clinvar/variation/496823/
NM_000543.4(SMPD1):c.757G>C	discordant	unreportable	reportable	93320	2	0	SCV000227150;S	Pathogenic;Pathogenic	EGL Genetic Diagn	2017-06-30;2016-1	SMPD1	chr11	6413052	6413052	http://gnomad.b	3.09E-05	NFE	4.50E-05	0		0	0	https://www.ncbi.nlm.nih.gov/clinvar/variation/93320/
NM_000518.4(HBB):c.-75G>C	discordant	unreportable	reportable	36294	1	0	SCV000605835	Likely pathogenic	Quest Diagnostics	8/1/2017	HBB	chr11	5248326	5248326		9.33E-06					0	0	https://www.ncbi.nlm.nih.gov/clinvar/variation/36294/
NM_000016.4(ACADM):c.430_432del	discordant	reportable	unreportable	226062	0	1	SCV000330908	Uncertain significance	EGL Genetic Diagn	6/30/2017	ACADM	chr1	76200511	76200514	http://gnomad.b	2.72E-06	NFE	8.96E-06	0		0	0	https://www.ncbi.nlm.nih.gov/clinvar/variation/226062/
NM_000016.4(ACADM):c.387+1delG	discordant	reportable	unreportable	188719	0	1	SCV000331022	Uncertain significance	EGL Genetic Diagn	6/30/2017	ACADM	chr1	76199309	76199310	http://gnomad.b	3.26E-05	EAS	5.80E-05	0		0	0	https://www.ncbi.nlm.nih.gov/clinvar/variation/188719/
NM_000017.2(ACADS):c.310_312delG	discordant	unreportable	reportable	3833	1	0	SCV000632501	Likely pathogenic	Invitae	10/5/2017	ACADS	chr12	121174883	121174886	http://gnomad.b	2.27E-05	NFE	3.03E-05	0		0	0	https://www.ncbi.nlm.nih.gov/clinvar/variation/3833/
NM_000271.4(NPC1):c.813_815delCA	discordant	unreportable	reportable	449503	1	0	SCV000617718	Likely pathogenic	GeneDx	11/28/2017	NPC1	chr18	21140260	21140263		5.72E-06					0	0	https://www.ncbi.nlm.nih.gov/clinvar/variation/449503/
NM_000112.3(SLC26A2):c.1650delG	discordant	reportable	unreportable	56016	0	1	SCV000454817	Uncertain significance	Illumina Clinical Se	10/18/2016	SLC26A2	chr5	149360805	149360806	http://gnomad.b	3.07E-05	NFE	1.79E-05	0		0	0	https://www.ncbi.nlm.nih.gov/clinvar/variation/56016/
NM_003060.3(SLC22A5):c.1324_1325	discordant	unreportable	reportable	6428	2	0	SCV000239183;S	Pathogenic;Likely pathoger	GeneDx;Invitae	2017-11-28;2017-1	SLC22A5	chr5	131728180	131728182		3.67E-05					0	0	https://www.ncbi.nlm.nih.gov/clinvar/variation/6428/
NM_000027.3(AGA):c.677G>A	discordant	unreportable	reportable	55950	1	0	SCV000329847	Pathogenic	GeneDx	11/28/2017	AGA	chr4	178357451	178357451	http://gnomad.b	2.96E-06	NFE	1.79E-05	0		0	0	https://www.ncbi.nlm.nih.gov/clinvar/variation/55950/
NM_000060.2(BTD):c.459G>A	discordant	unreportable	reportable	25009	1	0	SCV000630333	Pathogenic	Invitae	10/5/2017	BTD	chr3	15683564	15683564	http://gnomad.b	8.65E-06	NFE	8.96E-06	0		0	0	https://www.ncbi.nlm.nih.gov/clinvar/variation/25009/
NM_000152.3(GAA):c.546G>A	discordant	unreportable	reportable	280955	2	0	SCV000330940;S	Pathogenic;Pathogenic	EGL Genetic Diagn	2017-06-30;2018-0	GAA	chr17	78078931	78078931	http://gnomad.b	7.23E-05	EAS	0.0001764500048	0		0	0	https://www.ncbi.nlm.nih.gov/clinvar/variation/280955/
NM_017739.3(POMGNT1):c.1895+1G	discordant	reportable	unreportable	56593	1	1	SCV000577451;S	Pathogenic;Uncertain signi	GeneDx;Illumina C	2017-11-28;2016-1	POMGNT1	chr1	46655129	46655129	http://gnomad.b	0.0001007844389	FIN	0.000225611002	0		0	0	https://www.ncbi.nlm.nih.gov/clinvar/variation/56593/
NM_000441.1(SLC26A4):c.1708G>A	discordant	unreportable	reportable	43521	1	0	SCV000589698	Likely pathogenic	GeneDx	11/28/2017	SLC26A4	chr7	107341546	107341546	http://gnomad.b	0.000136811128	ASJ	0.0008136699907	0		0	0	https://www.ncbi.nlm.nih.gov/clinvar/variation/43521/
NM_000642.2(AGL):c.1078C>T	discordant	reportable	unreportable	371296	0	1	SCV000626663	Uncertain significance	Invitae	10/5/2017	AGL	chr1	100340362	100340362	http://gnomad.b	2.51E-05	SAS	6.50E-05	0		0	0	https://www.ncbi.nlm.nih.gov/clinvar/variation/371296/
NM_138694.3(PKHD1):c.4292G>A	discordant	reportable	unreportable	287201	0	1	SCV000340904	Uncertain significance	EGL Genetic Diagn	6/30/2017	PKHD1	chr6	51890316	51890316	http://gnomad.b	1.67E-05	AMR	0.0002680970065	0		0	0	https://www.ncbi.nlm.nih.gov/clinvar/variation/287201/
NM_000642.2(AGL):c.1027C>T	discordant	unreportable	reportable	429532	1	0	SCV000582123	Likely pathogenic	GeneDx	11/28/2017	AGL	chr1	100340311	100340311	http://gnomad.b	5.58E-06	AMR	2.98E-05	0		0	0	https://www.ncbi.nlm.nih.gov/clinvar/variation/429532/
NM_000352.3(ABCC8):c.4376T>G	discordant	unreportable	reportable	434058	1	0	SCV000592999	Pathogenic	Genetic Services La	7/5/2017	ABCC8	chr11	17416754	17416754		1.03E-05					0	0	https://www.ncbi.nlm.nih.gov/clinvar/variation/434058/
NM_000481.3(AMT):c.217C>T	discordant	reportable	unreportable	56228	0	1	SCV000636425	Uncertain significance	Invitae	10/5/2017	AMT	chr3	49459578	49459578	http://gnomad.b	4.80E-05	EAS	5.80E-05	0		0	0	https://www.ncbi.nlm.nih.gov/clinvar/variation/56228/
NM_000481.3(AMT):c.887G>A	discordant	reportable	unreportable	56239	0	1	SCV000512023	Uncertain significance	GeneDx	11/28/2017	AMT	chr3	49455397	49455397	http://gnomad.b	1.60E-05	AMR	9.14E-05	0		0	0	https://www.ncbi.nlm.nih.gov/clinvar/variation/56239/
NM_017890.4(VPS13B):c.8697-9A>G	discordant	unreportable	reportable	56697	1	0	SCV000491142	Pathogenic	GeneDx	12/21/2016	VPS13B	chr8	100831631	100831631	http://gnomad.b	2.80E-06	SAS	3.25E-05	0		0	0	https://www.ncbi.nlm.nih.gov/clinvar/variation/56697/
NM_000260.3(MYO7A):c.5618G>A	discordant	reportable	unreportable	43292	0	1	SCV000706352	Uncertain significance	EGL Genetic Diagn	6/30/2017	MYO7A	chr11	76916644	76916644	http://gnomad.b	7.20E-05	FIN	9.04E-05	0		0	0	https://www.ncbi.nlm.nih.gov/clinvar/variation/43292/
NM_206933.2(USH2A):c.653T>A	discordant	reportable	unreportable	48564	0	1	SCV000484497	Uncertain significance	EGL Genetic Diagn	6/30/2017	USH2A	chr1	216538426	216538426	http://gnomad.b	2.41E-05	NFE	7.21E-05	0		0	0	https://www.ncbi.nlm.nih.gov/clinvar/variation/48564/
NM_001164277.1(SLC37A4):c.81T>A	discordant	reportable	unreportable	68290	0	1	SCV000367678	Uncertain significance	Illumina Clinical Se	10/18/2016	SLC37A4	chr11	118899999	118899999	http://gnomad.b	2.79E-06	NFE	1.79E-05	0		0	0	https://www.ncbi.nlm.nih.gov/clinvar/variation/68290/
NM_000441.1(SLC26A4):c.706C>G	discordant	reportable	unreportable	43565	1	1	SCV000605149;S	Uncertain significance;Likel	ARUP Laboratories	2017-06-30;2016-0	SLC26A4	chr7	107315495	107315495	http://gnomad.b	5.03E-05	AMR	0.0002680170001	0		0	0	https://www.ncbi.nlm.nih.gov/clinvar/variation/43565/
NM_000784.3(CYP27A1):c.1184G>A	discordant	unreportable	reportable	65834	1	0	SCV000709264	Likely pathogenic	EGL Genetic Diagn	6/30/2017	CYP27A1	chr2	219678910	219678910	http://gnomad.b	2.40E-05	ASJ	0.0004065039975	0		0	0	https://www.ncbi.nlm.nih.gov/clinvar/variation/65834/
NM_000317.2(PTS):c.347A>G	discordant	unreportable	reportable	483	1	0	SCV000596632	Likely pathogenic	Genetic Services La	7/5/2017	PTS	chr11	112104187	112104187		8.00E-06					0	0	https://www.ncbi.nlm.nih.gov/clinvar/variation/483/
NM_024301.4(FKRP):c.1387A>G	discordant	unreportable	reportable	4235	2	1	SCV000230413;S	Pathogenic;Pathogenic;Un	EGL Genetic Diagn	2017-06-30;2015-0	FKRP	chr19	47260094	47260094	http://gnomad.b	9.60E-05	AMR	0.0008617389831	0		0	0	https://www.ncbi.nlm.nih.gov/clinvar/variation/4235/
NM_003494.3(DYSF):c.1555G>A	discordant	reportable	unreportable	6679	1	1	SCV000332008;S	Uncertain significance;Path	EGL Genetic Diagn	2017-06-30;2017-1	DYSF	chr2	71778203	71778203		8.08E-06					0	0	https://www.ncbi.nlm.nih.gov/clinvar/variation/6679/
NM_000426.3(LAMA2):c.9253C>T	discordant	reportable	unreportable	14291	0	1	SCV000617518	Uncertain significance	GeneDx	11/28/2017	LAMA2	chr6	129837376	129837376	http://gnomad.b	8.00E-06	NFE	8.97E-06	0		0	0	https://www.ncbi.nlm.nih.gov/clinvar/variation/14291/
NM_003060.3(SLC22A5):c.529A>G	discordant	unreportable	reportable	379230	1	0	SCV000515915	Likely pathogenic	GeneDx	11/28/2017	SLC22A5	chr5	131719870	131719870	http://gnomad.b	9.60E-05	AFR	0.001176159945	0		0	0	https://www.ncbi.nlm.nih.gov/clinvar/variation/379230/
NM_000136.2(FANCC):c.1663C>T	discordant	unreportable	reportable	234905	1	0	SCV000279991	Likely pathogenic	GeneDx	11/28/2017	FANCC	chr9	97864003	97864003	http://gnomad.b	3.33E-05	AFR	0.0001309929939	0		0	0	https://www.ncbi.nlm.nih.gov/clinvar/variation/234905/
NM_000017.2(ACADS):c.814C>T	discordant	unreportable	reportable	377422	1	0	SCV000511939	Likely pathogenic	GeneDx	11/28/2017	ACADS	chr12	121176354	121176354	http://gnomad.b	6.23E-05	AFR	6.56E-05	0		0	0	https://www.ncbi.nlm.nih.gov/clinvar/variation/377422/

Supplemental Table 2

NM_000060.2(BTD):c.626G>A	discordant	unreportable	reportable	92400	2	0	SCV000602898;S	Pathogenic;Likely pathogen	ARUP Laboratories	2017-06-30;2017-0	BTD	chr3	15685989	15685989	http://gnomad.b	4.90E-05	AFR	0.0001306850027	0		0	0	https://www.ncbi.nlm.nih.gov/clinvar/variation/92400/
NM_000152.3(GAA):c.761C>T	discordant	unreportable	reportable	325782	2	1	SCV000590492;S	Pathogenic;Likely pathogen	GeneDx;Illumina C	2017-11-28;2016-1	GAA	chr17	78081424	78081424	http://gnomad.b	0.0001945514477	EAS	0.002784220036	0		0	0	https://www.ncbi.nlm.nih.gov/clinvar/variation/325782/
NM_000152.3(GAA):c.752C>T	discordant	unreportable	reportable	325781	2	1	SCV000590491;S	Pathogenic;Likely pathogen	GeneDx;Illumina C	2017-11-28;2016-1	GAA	chr17	78081415	78081415	http://gnomad.b	0.0002001100605	EAS	0.002784549957	0		0	0	https://www.ncbi.nlm.nih.gov/clinvar/variation/325781/
NM_000492.3(CFTR):c.2909-15T>G	discordant	unreportable	reportable	53592	1	0	SCV000601082	Likely pathogenic	Quest Diagnostics	8/1/2017	CFTR	chr7	117246713	117246713	http://gnomad.b	7.70E-06	SAS	3.27E-05	0		0	0	https://www.ncbi.nlm.nih.gov/clinvar/variation/53592/
NM_000016.4(ACADM):c.1257C>A	discordant	unreportable	reportable	488675	1	0	SCV000680494	Pathogenic	GeneDx	2/7/2018	ACADM	chr1	76228439	76228439	http://gnomad.b	6.53E-05	ASJ	0.0008200079901	0		0	0	https://www.ncbi.nlm.nih.gov/clinvar/variation/488675/
NM_000277.1(PAH):c.1200-8G>A	discordant	unreportable	reportable	102565	1	0	SCV000629179	Likely pathogenic	Invitae	10/5/2017	PAH	chr12	103234301	103234301	http://gnomad.b	2.74E-06	NFE	8.95E-06	0		0	0	https://www.ncbi.nlm.nih.gov/clinvar/variation/102565/
NM_001360.2(DHCR7):c.1348delC	discordant	unreportable	reportable	282268	1	0	SCV000333636	Likely pathogenic	EGL Genetic Diagn	6/30/2017	DHCR7	chr11	71146500	71146501		1.88E-05					0	0	https://www.ncbi.nlm.nih.gov/clinvar/variation/282268/
NM_000017.2(ACADS):c.974G>A	discordant	unreportable	reportable	450631	1	0	SCV000619237	Likely pathogenic	GeneDx	11/28/2017	ACADS	chr12	121176663	121176663	http://gnomad.b	1.13E-05	NFE	8.97E-06	0		0	0	https://www.ncbi.nlm.nih.gov/clinvar/variation/450631/
NM_000169.2(GLA):c.593T>C	discordant	unreportable	reportable	167142	1	0	SCV000202803	Likely pathogenic	EGL Genetic Diagn	6/30/2017	GLA	chrX	100655700	100655700	http://gnomad.b	2.91E-05	NFE	2.52E-05	0	1	0	0	https://www.ncbi.nlm.nih.gov/clinvar/variation/167142/
NM_004006.2(DMD):c.1724T>C	discordant	unreportable	reportable	161220	2	1	SCV000613109;S	Likely pathogenic;Uncertain	Athena Diagnostics	2017-08-17;2017-0	DMD	chrX	32591735	32591735	http://gnomad.b	1.90E-05	NFE	1.25E-05	0	0	0	0	https://www.ncbi.nlm.nih.gov/clinvar/variation/161220/
NM_003494.3(DYSF):c.5444G>T	discordant	unreportable	reportable	283243	1	0	SCV000700927	Likely pathogenic	EGL Genetic Diagn	6/30/2017	DYSF	chr2	71896256	71896256		2.43E-05					0	0	https://www.ncbi.nlm.nih.gov/clinvar/variation/283243/
NM_000199.3(SGSH):c.130G>A	discordant	unreportable	reportable	381685	1	0	SCV000521211	Likely pathogenic	GeneDx	7/13/2017	SGSH	chr17	78190950	78190950		8.00E-06					0	0	https://www.ncbi.nlm.nih.gov/clinvar/variation/381685/
NM_000018.3(ACADVL):c.1183-15A>G	discordant	unreportable	reportable	439363	1	0	SCV000602382	Likely pathogenic	ARUP Laboratories	6/30/2017	ACADVL	chr17	7126948	7126948	http://gnomad.b	2.83E-06	SAS	9.75E-05	0		0	0	https://www.ncbi.nlm.nih.gov/clinvar/variation/439363/
NM_000228.2(LAMB3):c.1132+5G>A	discordant	unreportable	reportable	265229	1	0	SCV000321861	Pathogenic	GeneDx	11/28/2017	LAMB3	chr1	209803077	209803077	http://gnomad.b	2.80E-06	NFE	5.38E-05	0		0	0	https://www.ncbi.nlm.nih.gov/clinvar/variation/265229/
NM_000282.3(PCCA):c.893A>G	discordant	unreportable	reportable	449078	1	0	SCV000616817	Likely pathogenic	GeneDx	11/28/2017	PCCA	chr13	100921016	100921016	http://gnomad.b	1.60E-05	NFE	8.97E-06	0		0	0	https://www.ncbi.nlm.nih.gov/clinvar/variation/449078/
NM_000255.3(MUT):c.1084-10A>G	discordant	reportable	unreportable	203845	0	1	SCV000239009	Uncertain significance	GeneDx	7/13/2017	MUT	chr6	49419437	49419437	http://gnomad.b	7.99E-06	SAS	0.0001628449972	0		0	0	https://www.ncbi.nlm.nih.gov/clinvar/variation/203845/
NM_000532.4(PCCB):c.683C>T	discordant	unreportable	reportable	198428	4	0	SCV000604601;S	Pathogenic;Pathogenic;Pat	ARUP Laboratories	2017-06-30;2017-0	PCCB	chr3	136012626	136012626	http://gnomad.b	6.39E-05	NFE	9.87E-05	0		0	0	https://www.ncbi.nlm.nih.gov/clinvar/variation/198428/
NM_031885.3(BBS2):c.1895G>C	discordant	unreportable	reportable	4578	2	0	SCV000398050;S	Pathogenic;Pathogenic	Illumina Clinical Se	2016-10-18;2018-0	BBS2	chr16	56530894	56530894	http://gnomad.b	0.000248015873	ASJ	0.002639590064	0		0	0	https://www.ncbi.nlm.nih.gov/clinvar/variation/4578/
NM_000070.2(CAPN3):c.1505T>C	discordant	unreportable	reportable	282512	2	1	SCV000612634;S	Likely pathogenic;Uncertain	Athena Diagnostics	2017-08-17;2017-0	CAPN3	chr15	42693989	42693989	http://gnomad.b	0.000192027652	NFE	0.000261158013	0		0	0	https://www.ncbi.nlm.nih.gov/clinvar/variation/282512/
NM_000070.2(CAPN3):c.2092C>T	discordant	reportable	unreportable	285572	0	1	SCV000338651	Uncertain significance	EGL Genetic Diagn	6/30/2017	CAPN3	chr15	42702170	42702170	http://gnomad.b	8.00E-06	AMR	2.98E-05	0		0	0	https://www.ncbi.nlm.nih.gov/clinvar/variation/285572/
NM_000070.2(CAPN3):c.1257T>G	discordant	unreportable	reportable	282873	1	0	SCV000334587	Likely pathogenic	EGL Genetic Diagn	6/30/2017	CAPN3	chr15	42691753	42691753	http://gnomad.b	1.60E-05	AFR	6.54E-05	0		0	0	https://www.ncbi.nlm.nih.gov/clinvar/variation/282873/
NM_000070.2(CAPN3):c.640G>A	discordant	reportable	unreportable	496915	0	1	SCV000700630	Uncertain significance	EGL Genetic Diagn	6/30/2017	CAPN3	chr15	42681133	42681133	http://gnomad.b	6.40E-05	NFE	0.0001880100026	0		0	0	https://www.ncbi.nlm.nih.gov/clinvar/variation/496915/
NM_000359.2(TGM1):c.967C>T	discordant	unreportable	reportable	449108	1	0	SCV000616891	Likely pathogenic	GeneDx	11/28/2017	TGM1	chr14	24728927	24728927		8.02E-06					0	0	https://www.ncbi.nlm.nih.gov/clinvar/variation/449108/
NM_017882.2(CLN6):c.794_796delCC	discordant	reportable	unreportable	205182	1	1	SCV000708915;S	Uncertain significance;Path	EGL Genetic Diagn	2017-06-30;2015-0	CLN6	chr15	68500617	68500620	http://gnomad.b	7.99E-06	AFR	0.0001306850027	0		0	0	https://www.ncbi.nlm.nih.gov/clinvar/variation/205182/
NM_000092.4(COL4A4):c.1396G>A	discordant	unreportable	reportable	447180	1	0	SCV000612959	Pathogenic	Athena Diagnostics	8/17/2017	COL4A4	chr2	227954647	227954647	http://gnomad.b	1.60E-05	AFR	6.54E-05	0		0	0	https://www.ncbi.nlm.nih.gov/clinvar/variation/447180/
NM_032957.4(RTEL1):c.442C>T	discordant	unreportable	reportable	449904	1	0	SCV000618367	Likely pathogenic	GeneDx	11/28/2017	RTEL1	chr20	62293873	62293873	http://gnomad.b	8.00E-06	SAS	0.0001967599965	0		0	0	https://www.ncbi.nlm.nih.gov/clinvar/variation/449904/
NM_000155.3(GALT):c.752_753delAC	discordant	unreportable	reportable	286433	1	0	SCV000339869	Likely pathogenic	EGL Genetic Diagn	6/30/2017	GALT	chr9	34648822	34648824		5.50E-06					0	0	https://www.ncbi.nlm.nih.gov/clinvar/variation/286433/
NM_002485.4(NBN):c.163_171+3del1	discordant	unreportable	reportable	370793	1	0	SCV000690670	Likely pathogenic	Color Genomics, In	12/21/2017	NBN	chr8	90994946	90994958		2.79E-06					0	0	https://www.ncbi.nlm.nih.gov/clinvar/variation/370793/
NM_000282.3(PCCA):c.2119-9A>G	discordant	unreportable	reportable	459938	1	0	SCV000631905	Likely pathogenic	Invitae	10/5/2017	PCCA	chr13	101182343	101182343		2.40E-05					0	0	https://www.ncbi.nlm.nih.gov/clinvar/variation/459938/
NM_000135.2(FANCA):c.971T>G	discordant	unreportable	reportable	456147	1	0	SCV000626227	Likely pathogenic	Invitae	10/5/2017	FANCA	chr16	89862349	89862349		1.60E-05					0	0	https://www.ncbi.nlm.nih.gov/clinvar/variation/456147/
NM_206933.2(USH2A):c.15520-1G>A	discordant	unreportable	reportable	287641	1	0	SCV000341466	Likely pathogenic	EGL Genetic Diagn	6/30/2017	USH2A	chr1	215799213	215799213	http://gnomad.b	2.41E-05	SAS	9.75E-05	0		0	0	https://www.ncbi.nlm.nih.gov/clinvar/variation/287641/
NM_000091.4(COL4A3):c.1367_1369d	discordant	reportable	unreportable	334760	0	1	SCV000428161	Uncertain significance	Illumina Clinical Se	10/18/2016	COL4A3	chr2	228131183	228131186	http://gnomad.b	3.20E-05	EAS	5.81E-05	0		0	0	https://www.ncbi.nlm.nih.gov/clinvar/variation/334760/
NM_003640.3(IKBKAP):c.312T>A	discordant	reportable	unreportable	501334	0	1	SCV000707658	Uncertain significance	EGL Genetic Diagn	6/30/2017	IKBKAP	chr9	111689725	111689725	http://gnomad.b	4.73E-06	NFE	1.79E-05	0		0	0	https://www.ncbi.nlm.nih.gov/clinvar/variation/501334/
NM_003640.3(IKBKAP):c.1461-2A>G	discordant	reportable	unreportable	498695	0	1	SCV000703836	Uncertain significance	EGL Genetic Diagn	6/30/2017	IKBKAP	chr9	111668767	111668767		2.37E-06					0	0	https://www.ncbi.nlm.nih.gov/clinvar/variation/498695/
NM_001271208.1(NEB):c.25319delG	discordant	unreportable	reportable	465589	1	0	SCV000640754	Pathogenic	Invitae	10/5/2017	NEB	chr2	152346968	152346969		2.58E-06					0	0	https://www.ncbi.nlm.nih.gov/clinvar/variation/465589/
NM_000352.3(ABCC8):c.4160_4162de	concordant	reportable	reportable	196880	3	0	SCV000256808;S	Pathogenic;Pathogenic;Pat	Division of Genomi	2015-10-28;2017-0	ABCC8	chr11	17417434	17417437	http://gnomad.b	6.19E-05	ASJ	0.0003360970004	0		0	0	https://www.ncbi.nlm.nih.gov/clinvar/variation/196880/
NM_000352.3(ABCC8):c.3989-9G>A	concordant	reportable	reportable	9088	3	0	SCV000612213;S	Pathogenic;Pathogenic;Pat	Athena Diagnostics	2017-08-17;2015-1	ABCC8	chr11	17418602	17418602	http://gnomad.b	0.0007769065183	ASJ	0.005685280077	0		0	0	https://www.ncbi.nlm.nih.gov/clinvar/variation/9088/
NM_014363.4(SACS):c.8844delT	concordant	reportable	reportable	5512	2	0	SCV000329911;S	Pathogenic;Pathogenic	GeneDx;Invitae	2017-11-28;2017-1	SACS	chr13	23909170	23909171	http://gnomad.b	1.19E-05	NFE	0.0001257340045	0		0	0	https://www.ncbi.nlm.nih.gov/clinvar/variation/5512/
NM_000023.2(SGCA):c.229C>T	concordant	reportable	reportable	9437	3	0	SCV000228756;S	Pathogenic;Pathogenic;Pat	EGL Genetic Diagn	2017-06-30;2017-1	SGCA	chr17	48245014	48245014	http://gnomad.b	0.0002966495394	FIN	0.001985739917	0		0	0	https://www.ncbi.nlm.nih.gov/clinvar/variation/9437/
NM_000383.3(AIRE):c.769C>T	concordant	reportable	reportable	3307	1	0	SCV000329055	Pathogenic	GeneDx	11/28/2017	AIRE	chr21	45709656	45709656	http://gnomad.b	0.0002617990609	FIN	0.004986519925	1		0	0	https://www.ncbi.nlm.nih.gov/clinvar/variation/3307/
NM_000232.4(SGCB):c.341C>T	concordant	reportable	reportable	42035	5	0	SCV000255839;S	Pathogenic;Pathogenic;Pat	Athena Diagnostics	2015-10-06;2017-0	SGCB	chr4	52895932	52895932	http://gnomad.b	0.000179120184	NFE	0.0005550779752	0		0	0	https://www.ncbi.nlm.nih.gov/clinvar/variation/42035/
NM_000518.4(HBB):c.47G>A	concordant	reportable	reportable	15403	3	0	SCV000603905;S	Pathogenic;Pathogenic;Pat	ARUP Laboratories	2017-06-30;2018-0	HBB	chr11	5248205	5248205	http://gnomad.b	4.67E-05	SAS	0.0006497729919	0		0	0	https://www.ncbi.nlm.nih.gov/clinvar/variation/15403/
NM_000518.4(HBB):c.118C>T	concordant	reportable	reportable	15402	5	0	SCV000603892;S	Pathogenic;Pathogenic;Pat	ARUP Laboratories	2017-06-30;2017-0	HBB	chr11	5248004	5248004	http://gnomad.b	0.0003942224254	NFE	0.000671826012	0		0	0	https://www.ncbi.nlm.nih.gov/clinvar/variation/15402/
NM_000518.4(HBB):c.51delC	concordant	reportable	reportable	15414	1	0	SCV000697137	Pathogenic	Laboratory Corpor	1/25/2018	HBB	chr11	5248200	5248201	http://gnomad.b	1.63E-05	SAS	0.0002923790016	0		0	0	https://www.ncbi.nlm.nih.gov/clinvar/variation/15414/
NM_000518.4(HBB):c.20delA	concordant	reportable	reportable	15418	2	0	SCV000603925;S	Pathogenic;Pathogenic	ARUP Laboratories	2017-06-30;2018-0	HBB	chr11	5248231	5248232	http://gnomad.b	4.20E-05	AFR	6.54E-05	0		0	0	https://www.ncbi.nlm.nih.gov/clinvar/variation/15418/
NM_000518.4(HBB):c.126_129delCTT	concordant	reportable	reportable	15417	2	0	SCV000697076;S	Pathogenic;Pathogenic	Laboratory Corpor	2018-01-25;2017-0	HBB	chr11	5247992	5247996	http://gnomad.b	0.0002495964469	EAS	0.002031340031	0		0	0	https://www.ncbi.nlm.nih.gov/clinvar/variation/15417/
NM_000518.4(HBB):c.25_26delAA	concordant	reportable	reportable	15413	1	0	SCV000601261	Pathogenic	Quest Diagnostics	8/1/2017	HBB	chr11	5248225	5248227	http://gnomad.b	2.10E-05	SAS	6.52E-05	0		0	0	https://www.ncbi.nlm.nih.gov/clinvar/variation/15413/
NM_000518.4(HBB):c.27dupG	concordant	reportable	reportable	36308	2	0	SCV000568518;S	Pathogenic;Pathogenic	GeneDx;Illumina C	2017-07-13;2016-1	HBB	chr11	5248224	5248224	http://gnomad.b	8.86E-05	SAS	0.002046779962	0		0	0	https://www.ncbi.nlm.nih.gov/clinvar/variation/36308/
NM_000518.4(HBB):c.316-106C>G	concordant	reportable	reportable	15457	3	0	SCV000603910;S	Pathogenic;Pathogenic;Pat	ARUP Laboratories	2017-06-30;2017-0	HBB	chr11	5247062	5247062		5.37E-05					0	0	https://www.ncbi.nlm.nih.gov/clinvar/variation/15457/
NM_000518.4(HBB):c.315+1G>A	concordant	reportable	reportable	15438	4	0	SCV000603893;S	Pathogenic;Pathogenic;Pat	ARUP Laboratories	2017-06-30;2017-1	HBB	chr11	5247806	5247806	http://gnomad.b	0.0001212992078	AFR	0.0001960270019	0		0	0	https://www.ncbi.nlm.nih.gov/clinvar/variation/15438/
NM_000518.4(HBB):c.75T>A	concordant	reportable	reportable	15459	2	0	SCV000603908;S	Pathogenic;Pathogenic	ARUP Laboratories	2017-06-30;2017-0	HBB	chr11	5248177	5248177	http://gnomad.b	1.63E-05	AFR	6.54E-05	0		0	0	https://www.ncbi.nlm.nih.gov/clinvar/variation/15459/
NM_000518.4(HBB):c.-79A>G	concordant	reportable	reportable	15469	1	0	SCV000697149	Pathogenic	Laboratory Corpor	1/25/2018	HBB	chr11	5248330	5248330		0.0002122736137					0	0	https://www.ncbi.nlm.nih.gov/clinvar/variation/15469/
NM_000518.4(HBB):c.-137C>G	concordant	reportable	reportable	15464	1	0	SCV000601245	Pathogenic	Quest Diagnostics	8/1/2017	HBB	chr11	5248388	5248388		9.33E-06					0	0	https://www.ncbi.nlm.nih.gov/clinvar/variation/15464/
NM_000060.2(BTD):c.755A>G	concordant	reportable	reportable	1901	1	0	SCV000600947	Pathogenic	Quest Diagnostics	8/1/2017	BTD	chr3	15686118	15686118	http://gnomad.b	5.77E-05	AMR	8.93E-05	0		0	0	https://www.ncbi.nlm.nih.gov/clinvar/variation/1901/
NM_000492.3(CFTR):c.178G>T	concordant	reportable	reportable	38730	2	0	SCV000071535;S	Pathogenic;Pathogenic	CFTR2;Laboratory	2017-06-29;2018-0	CFTR	chr7	117149101	117149101	http://gnomad.b	2.50E-05	NFE	5.39E-05	0		0	0	https://www.ncbi.nlm.nih.gov/clinvar/variation/38730/
NM_000492.3(CFTR):c.223C>T	concordant	reportable	reportable	48672	1	0	SCV000071554	Pathogenic	CFTR2	6/29/2017	CFTR	chr7	117149146	117149146	http://gnomad.b	3.27E-05	AMR	8.94E-05	0		0	0	https://www.ncbi.nlm.nih.gov/clinvar/variation/48672/
NM_000492.3(CFTR):c.254G>A	concordant	reportable	reportable	7143	2	0	SCV000071502;S	Pathogenic;Pathogenic	CFTR2;EGL Genetic	2017-06-29;2017-0	CFTR	chr7	117149177	117149177	http://gnomad.b	5.58E-05	NFE	9.88E-05	0		0	0	https://www.ncbi.nlm.nih.gov/clinvar/variation/7143/
NM_000492.3(CFTR):c.274G>T	concordant	reportable	reportable	7186	2	0	SCV000071538;S	Pathogenic;Pathogenic	CFTR2;Laboratory	2017-06-29;2018-0	CFTR	chr7	117170953	117170953	http://gnomad.b	5.78E-06	NFE	8.98E-06	0		0	0	https://www.ncbi.nlm.nih.gov/clinvar/variation/7186/
NM_000492.3(CFTR):c.350G>A	concordant	reportable	reportable	7109	5	1	SCV000602967;S	Pathogenic;Pathogenic;Pat	ARUP Laboratories	2017-06-30;2014-0	CFTR	chr7	117171029	117171029	http://gnomad.b	0.001480965073	NFE	0.002576299943	1		2	0	https://www.ncbi.nlm.nih.gov/clinvar/variation/7109/
NM_000492.3(CFTR):c.532G>A	concordant	reportable	reportable	48692	2	0	SCV000071500;S	Pathogenic;Pathogenic	CFTR2;Quest Diag	2017-06-29;2017-0	CFTR	chr7	117174372	117174372	http://gnomad.b	1.93E-06	NFE	2.69E-05	0		0	0	https://www.ncbi.nlm.nih.gov/clinvar/variation/48692/
NM_000492.3(CFTR):c.617T>G	concordant	reportable	reportable	7190	5	0	SCV000603055;S	Pathogenic;Pathogenic;Pat	ARUP Laboratories	2017-06-30;2017-0	CFTR	chr7	117175339	117175339	http://gnomad.b	0.0001386599288	AMR	0.0005360650248	0		0	0	https://www.ncbi.nlm.nih.gov/clinvar/variation/7190/
NM_000492.3(CFTR):c.988G>T	concordant	reportable	reportable	48708	1	0	SCV000071539	Pathogenic	CFTR2	6/29/2017	CFTR	chr7	117180272	117180272		5.78E-06					0	0	https://www.ncbi.nlm.nih.gov/clinvar/variation/48708/
NM_000492.3(CFTR):c.1013C>T	concordant	reportable	reportable	7193	3	0	SCV000603069;S	Pathogenic;Pathogenic;Pat	ARUP Laboratories	2017-06-30;2017-0	CFTR	chr7	117180297	117180297	http://gnomad.b	2.31E-05	NFE	5.38E-05	0		0	0	https://www.ncbi.nlm.nih.gov/clinvar/variation/7193/
NM_000492.3(CFTR):c.1040G>A	concordant	reportable	reportable	7182	4	0	SCV000603045;S	Pathogenic;Pathogenic;Pat	ARUP Laboratories	2017-06-30;2017-0	CFTR	chr7	117180324	117180324	http://gnomad.b	5.78E-05	NFE	3.59E-05	0		0	0	https://www.ncbi.nlm.nih.gov/clinvar/variation/7182/
NM_000492.3(CFTR):c.1040G>C	concordant	reportable	reportable	7110	3	0	SCV000603032;S	Pathogenic;Pathogenic;Pat	ARUP Laboratories	2017-06-30;2017-0	CFTR	chr7	117180324	117180324	http://gnomad.b	3.47E-05	NFE	5.39E-05	0		0	0	https://www.ncbi.nlm.nih.gov/clinvar/variation/7110/
NM_000492.3(CFTR):c.1477C>T	concordant	reportable	reportable	7107	2	0	SCV000071546;S	Pathogenic;Pathogenic	CFTR2;Laboratory	2017-06-29;2018-0	CFTR	chr7	117199602	117199602	http://gnomad.b	2.31E-05	NFE	4.48E-05	0		0	0	https://www.ncbi.nlm.nih.gov/clinvar/variation/7107/
NM_000492.3(CFTR):c.1558G>T	concordant	reportable	reportable	7150	1	0	SCV000071532	Pathogenic	CFTR2	6/29/2017	CFTR	chr7	117199683	117199683	http://gnomad.b	2.12E-05	NFE	8.97E-06	0		0	0	https://www.ncbi.nlm.nih.gov/clinvar/variation/7150/
NM_000492.3(CFTR):c.1624G>T	concordant	reportable	reportable	7115	5	0	SCV000603003;S	Pathogenic;Pathogenic;Pat	ARUP Laboratories	2017-06-30;2017-0	CFTR	chr7	117227832	117227832	http://gnomad.b	0.000452570601	ASJ	0.001422479982	0		0	0	https://www.ncbi.nlm.nih.gov/clinvar/variation/7115/
NM_000492.3(CFTR):c.1647T>G	concordant	reportable	reportable	40190	3	0	SCV000087508;S	Pathogenic;Pathogenic;Pat	CFTR2;Laboratory	2017-06-29;2018-0	CFTR	chr7	117227855	117227855	http://gnomad.b	5.78E-06	AMR	5.98E-05	0		0	0	https://www.ncbi.nlm.nih.gov/clinvar/variation/40190/
NM_000492.3(CFTR):c.1652G>A	concordant	reportable	reportable	7120	6	0	SCV000603048;S	Pathogenic;Pathogenic;Pat	ARUP Laboratories	2017-06-30;2017-0	CFTR	chr7	117227860	117227860	http://gnomad.b	0.0002965781811	NFE	0.0003953140113	0		0	0	https://www.ncbi.nlm.nih.gov/clinvar/variation/7120/
NM_000492.3(CFTR):c.1657C>T	concordant	reportable	reportable	7122	4	0	SCV000071552;S	Pathogenic;Pathogenic;Pat	CFTR2;EGL Genetic	2017-06-29;2017-0	CFTR	chr7	117227865	117227865	http://gnomad.b	0.000105920779	NFE	0.000134810005	0		0	0	https://www.ncbi.nlm.nih.gov/clinvar/variation/7122/
NM_000492.3(CFTR):c.1679G>C	concordant	reportable	reportable	7113	1	0	SCV000071524	Pathogenic	CFTR2	6/29/2017	CFTR	chr7	117227887	117227887	http://gnomad.b	4.43E-05	NFE	4.50E-05	0		0	0	https://www.ncbi.nlm.nih.gov/clinvar/variation/7113/
NM_000492.3(CFTR):c.2290C>T	concordant	reportable	reportable	53471	1	0	SCV000071555	Pathogenic	CFTR2	6/29/2017	CFTR	chr7	117232511	117232511	http://gnomad.b	1.93E-06	FIN	4.80E-05	0		0	0	https://www.ncbi.nlm.nih.gov/clinvar/variation/53471/
NM_000492.3(CFTR):c.3266G>A	concordant	reportable	reportable	7194	4	0	SCV000602987;S	Pathogenic;Pathogenic;Pat	ARUP Laboratories	2017-06-30;2017-0	CFTR	chr7	117251761	117251761	http://gnomad.b	5.78E-06	AMR	0.0001792539988	0		0	0	https://www.ncbi.nlm.nih.gov/clinvar/variation/7194/
NM_000492.3(CFTR):c.3276C>A	concordant	reportable	reportable	38728	2	0	SCV000071565;S	Pathogenic;Pathogenic	CFTR2;GeneDx	2017-06-29;2017-1	CFTR	chr7	117251771	117251771	http://gnomad.b	2.70E-05	NFE	2.69E-05	0		0	0	https://www.ncbi.nlm.nih.gov/clinvar/variation/38728/
NM_000492.3(CFTR):c.3302T>A	concordant	reportable	reportable	39516	2	0	SCV000071511;S	Pathogenic;Pathogenic	CFTR2;EGL Genetic	2017-06-29;2017-0	CFTR	chr7	117251797	117251797	http://gnomad.b	5.78E-06	NFE	3.59E-05	0		0	0	https://www.ncbi.nlm.nih.gov/clinvar/variation/39516/
NM_000492.3(CFTR):c.3454G>C	concordant	reportable	reportable	35867	6	0	SCV000603066;S	Pathogenic;Pathogenic;Pat	ARUP Laboratories	2017-06-30;2017-0	CFTR	chr7	117254753	117254753	http://gnomad.b	0.0005719722064	ASJ	0.002845529933	0		0	0	https://www.ncbi.nlm.nih.gov/clinvar/variation/35867/
NM_000492.3(CFTR):c.2012delT	concordant	reportable	reportable	35836	3	0	SCV000071470;S	Pathogenic;Pathogenic;Pat	CFTR2;Invitae;Lab	2017-06-29;2017-0	CFTR	chr7	117232231	117232232	http://gnomad.b	5.78E-06	NFE	9.04E-06	0		0	0	https://www.ncbi.nlm.nih.gov/clinvar/variation/35836/
NM_000492.3(CFTR):c.3067_3072del	concordant	reportable	reportable	38480	2	0	SCV000074788;S	Pathogenic;Pathogenic	Invitae;Laboratory	2017-06-19;2018-0	CFTR	chr7	117250646	117250652	http://gnomad.b	1.54E-05	AMR	0.000149325002	0		0	0	https://www.ncbi.nlm.nih.gov/clinvar/variation/38480/
NM_000492.3(CFTR):c.1521_1523del	concordant	reportable	reportable	7105	7	0	SCV000602968;S	Pathogenic;Pathogenic;Pat	ARUP Laboratories	2017-06-30;2017-0	CFTR	chr7	117199644	117199647	http://gnomad.b	0.008731723851	NFE	0.01203559991	1		26	0	https://www.ncbi.nlm.nih.gov/clinvar/variation/7105/
NM_000492.3(CFTR):c.935_937delTCT	concordant	reportable	reportable	48704	1	0	SCV000697058	Pathogenic	Laboratory Corpor	1/25/2018	CFTR	chr7	117180210	117180213	http://gnomad.b	5.97E-05	AMR	0.0003578669857	1		0	0	https://www.ncbi.nlm.nih.gov/clinvar/variation/48704/
NM_000492.3(CFTR):c.262_263delTT	concordant	reportable	reportable	7232	3	0	SCV000603009;S	Pathogenic;Pathogenic;Pat	ARUP Laboratories	2017-06-30;2017-0	CFTR	chr7	117149181	117149183	http://gnomad.b	2.50E-05	FIN	0.00140107004	0		0	0	https://www.ncbi.nlm.nih.gov/clinvar/variation/7232/
NM_000492.3(CFTR):c.313delA	concordant	reportable	reportable	48684	1	0	SCV000245889	Pathogenic	CFTR2	6/29/2017	CFTR	chr7	117170990	117170991	http://gnomad.b	1.93E-06	AFR	6.54E-05	0		0	0	https://www.ncbi.nlm.nih.gov/clinvar/variation/48684/
NM_000492.3(CFTR):c.1519_1521del	concordant	reportable	reportable	7106	1	0	SCV000071494	Pathogenic	CFTR2	6/29/2017	CFTR	chr7	117199640	117199643	http://gnomad.b	6.16E-05	AFR	0.0001307019993	0		0	0	https://www.ncbi.nlm.nih.gov/clinvar/variation/7106/
NM_000492.3(CFTR):c.531delT	concordant	reportable	reportable	53991	1	0	SCV000071489	Pathogenic	CFTR2	6/29/2017	CFTR	chr7	117174369	117174370		7.70E-06					0	0	https://www.ncbi.nlm.nih.gov/clinvar/variation/53991/
NM_000492.3(CFTR):c.803delA	concordant	reportable	reportable	48700	1	0	SCV000245931	Pathogenic	CFTR2	6/29/2017	CFTR	chr7	117176657	117176658	http://gnomad.b	1.16E-05	AMR	0.0001202650019	0		0	0	https://www.ncbi.nlm.nih.gov/clinvar/variation/48700/
NM_000492.3(CFTR):c.948delT	concordant	reportable	reportable	7163	3	0	SCV000071464;S	Pathogenic;Pathogenic;Pat	CFTR2;Laboratory	2017-06-29;2018-0	CFTR	chr7	117180229	117180230	http://gnomad.b	2.12E-05	AFR	6.54E-05	0		0	0	https://www.ncbi.nlm.nih.gov/clinvar/variation/7163/
NM_000492.3(CFTR):c.1545_1546delT	concordant	reportable	reportable	7140	3	0	SCV000071469;S	Pathogenic;Pathogenic;Pat	CFTR2;Invitae;Lab	2017-06-29;2017-0	CFTR	chr7	117199666	117199668		1.16E-05					0	0	https://www.ncbi.nlm.nih.gov/clinvar/variation/7140/
NM_000492.3(CFTR):c.1911delG	concordant	reportable	reportable	35834	1	0	SCV000052140	Pathogenic	Laboratory Corpor	11/20/2015	CFTR	chr7	117232131	117232132		1.93E-06					0	0	https://www.ncbi.nlm.nih.gov/clinvar/variation/35834/
NM_000492.3(CFTR):c.3528delC	concordant	reportable	reportable	35868	4	0	SCV000603072;S	Pathogenic;Pathogenic;Pat	ARUP Laboratories	2017-06-30;2017-0	CFTR	chr7	117267633	117267634	http://gnomad.b	2.70E-05	FIN	0.0003590660053	0		0	0	https://www.ncbi.nlm.nih.gov/clinvar/variation/35868/
NM_000492.3(CFTR):c.1155_1156dup	concordant	reportable	reportable	487393	2	0	SCV000245887;S	Pathogenic;Pathogenic	CFTR2;Laboratory	2017-06-29;2018-0	CFTR	chr7	117182104	117182104	http://gnomad.b	3.85E-06	AMR	8.95E-05	0		0	0	https://www.ncbi.nlm.nih.gov/clinvar/variation/487393/
NM_000492.3(CFTR):c.2052dupA	concordant	reportable	reportable	35838	3	0	SCV000071473;S	Pathogenic;Pathogenic;Pat	CFTR2;Invitae;Lab	2017-06-29;2017-0	CFTR	chr7	117232266	117232266	http://gnomad.b	6.93E-05	SAS	6.51E-05	0		0	0	https://www.ncbi.nlm.nih.gov/clinvar/variation/35838/
NM_000492.3(CFTR):c.3773dupT	concordant	reportable	reportable	38726	2	0	SCV000071482;S	Pathogenic;Pathogenic	CFTR2;Invitae	2017-06-29;2017-0	CFTR	chr7	117282541	117282541	http://gnomad.b	1.73E-05	NFE	1.80E-05	0		0	0	https://www.ncbi.nlm.nih.gov/clinvar/variation/38726/
NM_000492.3(CFTR):c.3889dupT	concordant	reportable	reportable	53841	3	0	SCV000071484;S	Pathogenic;Pathogenic;Pat	CFTR2;Laboratory	2017-06-29;2018-0	CFTR	chr7	117292905	117292905	http://gnomad.b	3.85E-06	AFR	6.57E-05	0		0	0	https://www.ncbi.nlm.nih.gov/clinvar/variation/53841/
NM_000492.3(CFTR):c.273+1G>A	concordant	reportable	reportable	53549	1	0	SCV000071459	Pathogenic	CFTR2	6/29/2017	CFTR	chr7	117149197	117149197	http://gnomad.b	3.85E-06	NFE	9.00E-06	0		0	0	https://www.ncbi.nlm.nih.gov/clinvar/variation/53549/
NM_000492.3(CFTR):c.274-1G>A	concordant	reportable	reportable	48680	2	0	SCV000071460;S	Pathogenic;Pathogenic	CFTR2;Laboratory	2017-06-29;2018-0	CFTR	chr7	117170952	117170952	http://gnomad.b	5.78E-06	AMR	0.0001199109975	0		0	0	https://www.ncbi.nlm.nih.gov/clinvar/variation/48680/
NM_000492.3(CFTR):c.489+1G>T	concordant	reportable	reportable	38799	3	0	SCV000071461;S	Pathogenic;Pathogenic;Pat	CFTR2;EGL Genetic	2017-06-29;2017-0	CFTR	chr7	117171169	117171169	http://gnomad.b	0.0001097724437	NFE	0.0001756300044	0		0	0	https://www.ncbi.nlm.nih.gov/clinvar/variation/38799/

Supplemental Table 2

NM_000492.3(CFTR):c.1766+1G>A	concordant	reportable	reportable	7168	1	0	SCV000071454	Pathogenic	CFTR2	6/29/2017	CFTR	chr7	117230494	117230494	http://gnomad.b	3.85E-05	NFE	7.21E-05	0		0	0	https://www.ncbi.nlm.nih.gov/clinvar/variation/7168/
NM_000492.3(CFTR):c.2657+5G>A	concordant	reportable	reportable	38497	3	0	SCV000603041;S	Pathogenic;Pathogenic;Pat	ARUP Laboratories	2017-06-30;2017-0	CFTR	chr7	117242922	117242922	http://gnomad.b	9.05E-05	NFE	0.0001521800004	0		0	0	https://www.ncbi.nlm.nih.gov/clinvar/variation/38497/
NM_000492.3(CFTR):c.2988+1G>A	concordant	reportable	reportable	7224	5	0	SCV000603044;S	Pathogenic;Pathogenic;Pat	ARUP Laboratories	2017-06-30;2017-0	CFTR	chr7	117246808	117246808	http://gnomad.b	0.0001020691143	AFR	0.001373809995	0		0	0	https://www.ncbi.nlm.nih.gov/clinvar/variation/7224/
NM_000492.3(CFTR):c.3140-26A>G	concordant	reportable	reportable	35864	4	0	SCV000071572;S	Pathogenic;Pathogenic;Pat	CFTR2;EGL Genetic	2017-06-29;2017-0	CFTR	chr7	117251609	117251609	http://gnomad.b	4.43E-05	AMR	9.30E-05	0		0	0	https://www.ncbi.nlm.nih.gov/clinvar/variation/35864/
NM_000492.3(CFTR):c.3718-2477C>T	concordant	reportable	reportable	7166	5	0	SCV000603001;S	Pathogenic;Pathogenic;Pat	ARUP Laboratories	2017-06-30;2017-0	CFTR	chr7	117280015	117280015		0.0002310998814					0	0	https://www.ncbi.nlm.nih.gov/clinvar/variation/7166/
NM_000492.3(CFTR):c.2988G>A	concordant	reportable	reportable	35857	3	0	SCV000071571;S	Pathogenic;Pathogenic;Pat	CFTR2;EGL Genetic	2017-06-29;2017-0	CFTR	chr7	117246807	117246807	http://gnomad.b	1.54E-05	NFE	8.98E-06	0		0	0	https://www.ncbi.nlm.nih.gov/clinvar/variation/35857/
NM_000492.3(CFTR):c.2051_2052delA	concordant	reportable	reportable	35837	3	0	SCV000071471;S	Pathogenic;Pathogenic;Pat	CFTR2;EGL Genetic	2017-06-29;2017-0	CFTR	chr7	117232271	117232273		5.01E-05					0	0	https://www.ncbi.nlm.nih.gov/clinvar/variation/35837/
NM_006493.2(CLN5):c.1175_1176delA	concordant	reportable	reportable	2564	1	0	SCV000568354	Pathogenic	GeneDx	11/28/2017	CLN5	chr13	77575052	77575054	http://gnomad.b	2.80E-06	FIN	0.0008274340071	0		0	0	https://www.ncbi.nlm.nih.gov/clinvar/variation/2564/
NM_000049.2(ASPA):c.914C>A	concordant	reportable	reportable	2607	3	0	SCV000577185;S	Pathogenic;Pathogenic;Pat	GeneDx;Invitae;La	2017-11-28;2017-0	ASPA	chr17	3402354	3402354	http://gnomad.b	0.0002649683457	NFE	0.000385685009	0		0	0	https://www.ncbi.nlm.nih.gov/clinvar/variation/2607/
NM_000049.2(ASPA):c.854A>C	concordant	reportable	reportable	2605	4	0	SCV000231463;S	Pathogenic;Pathogenic;Pat	EGL Genetic Diagn	2017-06-30;2017-1	ASPA	chr17	3402294	3402294	http://gnomad.b	0.001057507594	ASJ	0.008730960079	0		0	0	https://www.ncbi.nlm.nih.gov/clinvar/variation/2605/
NM_000049.2(ASPA):c.693C>A	concordant	reportable	reportable	2609	3	0	SCV000402018;S	Pathogenic;Pathogenic;Pat	Illumina Clinical Se	2016-10-18;2017-1	ASPA	chr17	3397702	3397702	http://gnomad.b	0.0001182894401	ASJ	0.001218519988	0		0	0	https://www.ncbi.nlm.nih.gov/clinvar/variation/2609/
NM_000098.2(CPT2):c.1239_1240delG	concordant	reportable	reportable	92430	3	0	SCV000238786;S	Pathogenic;Pathogenic;Pat	GeneDx;Invitae;La	2017-11-28;2017-1	CPT2	chr1	53676583	53676585	http://gnomad.b	0.0004365028289	ASJ	0.004670049995	0		0	0	https://www.ncbi.nlm.nih.gov/clinvar/variation/92430/
NM_000098.2(CPT2):c.338C>T	concordant	reportable	reportable	8953	5	0	SCV000611187;S	Pathogenic;Pathogenic;Pat	Fulgent Genetics,F	2017-05-23;2017-0	CPT2	chr1	53668099	53668099	http://gnomad.b	0.002207697	ASJ	0.006903550122	3		3	0	https://www.ncbi.nlm.nih.gov/clinvar/variation/8953/
NM_000098.2(CPT2):c.370C>T	concordant	reportable	reportable	203659	1	0	SCV000238778	Pathogenic	GeneDx	11/28/2017	CPT2	chr1	53675716	53675716	http://gnomad.b	1.68E-05	NFE	8.95E-06	0		0	0	https://www.ncbi.nlm.nih.gov/clinvar/variation/203659/
NM_000303.2(PMM2):c.357C>A	concordant	reportable	reportable	7711	1	0	SCV000399673	Pathogenic	Illumina Clinical Se	10/18/2016	PMM2	chr16	8904945	8904945		4.73E-05					0	0	https://www.ncbi.nlm.nih.gov/clinvar/variation/7711/
NM_004646.3(NPHS1):c.121_122delC	concordant	reportable	reportable	56431	1	0	SCV000698495	Pathogenic	Laboratory Corpor	1/25/2018	NPHS1	chr19	36342510	36342512	http://gnomad.b	4.46E-05	FIN	0.01119130012	0		0	0	https://www.ncbi.nlm.nih.gov/clinvar/variation/56431/
NM_004646.3(NPHS1):c.3325C>T	concordant	reportable	reportable	6867	1	0	SCV000698506	Pathogenic	Laboratory Corpor	1/25/2018	NPHS1	chr19	36322260	36322260	http://gnomad.b	3.90E-05	FIN	0.0006279150257	0		0	0	https://www.ncbi.nlm.nih.gov/clinvar/variation/6867/
NM_004937.2(CTNS):c.414G>A	concordant	reportable	reportable	4443	1	0	SCV000232215	Pathogenic	EGL Genetic Diagn	6/30/2017	CTNS	chr17	3558599	3558599	http://gnomad.b	6.22E-05	NFE	8.95E-05	0		0	0	https://www.ncbi.nlm.nih.gov/clinvar/variation/4443/
NM_000228.2(LAMB3):c.727C>T	concordant	reportable	reportable	35480	1	0	SCV000680542	Pathogenic	GeneDx	2/7/2018	LAMB3	chr1	209806023	209806023	http://gnomad.b	5.60E-06	NFE	2.69E-05	0		0	0	https://www.ncbi.nlm.nih.gov/clinvar/variation/35480/
NM_000228.2(LAMB3):c.1903C>T	concordant	reportable	reportable	14539	2	0	SCV000321863;S	Pathogenic;Pathogenic	GeneDx;Laborator	2017-11-28;2018-0	LAMB3	chr1	209799066	209799066	http://gnomad.b	0.0004815202602	NFE	0.00105712004	0		0	0	https://www.ncbi.nlm.nih.gov/clinvar/variation/14539/
NM_000136.2(FANCC):c.67delG	concordant	reportable	reportable	12049	3	0	SCV000149267;S	Pathogenic;Pathogenic;Pat	GeneDx;Invitae;La	2017-11-28;2017-1	FANCC	chr9	98011506	98011507	http://gnomad.b	9.51E-05	NFE	0.0002871190081	0		0	0	https://www.ncbi.nlm.nih.gov/clinvar/variation/12049/
NM_000136.2(FANCC):c.456+4A>T	concordant	reportable	reportable	12045	4	0	SCV000230847;S	Pathogenic;Pathogenic;Pat	EGL Genetic Diagn	2017-06-30;2017-1	FANCC	chr9	97934315	97934315	http://gnomad.b	0.0006682489025	ASJ	0.00589550985	0		0	0	https://www.ncbi.nlm.nih.gov/clinvar/variation/12045/
NM_000243.2(MEFV):c.2040G>A	concordant	reportable	reportable	2550	3	0	SCV000604195;S	Pathogenic;Pathogenic;Pat	ARUP Laboratories	2017-06-30;2017-1	MEFV	chr16	3293447	3293447		3.36E-05					1	0	https://www.ncbi.nlm.nih.gov/clinvar/variation/2550/
NM_000243.2(MEFV):c.2040G>C	concordant	reportable	reportable	36507	4	0	SCV000604173;S	Pathogenic;Pathogenic;Pat	ARUP Laboratories	2017-06-30;2017-0	MEFV	chr16	3293447	3293447	http://gnomad.b	0.0002294990204	NFE	0.0002148380008	0		1	0	https://www.ncbi.nlm.nih.gov/clinvar/variation/36507/
NM_004004.5(GJB2):c.71G>A	concordant	reportable	reportable	17002	5	0	SCV000599727;S	Pathogenic;Pathogenic;Pat	Division of Genomi	2017-05-17;2017-0	GJB2	chr13	20763650	20763650	http://gnomad.b	0.0005085190918	SAS	0.004450649954	1		0	0	https://www.ncbi.nlm.nih.gov/clinvar/variation/17002/
NM_004004.5(GJB2):c.229T>C	concordant	reportable	reportable	17003	2	0	SCV000700561;S	Pathogenic;Pathogenic	EGL Genetic Diagn	2017-06-30;2017-1	GJB2	chr13	20763492	20763492	http://gnomad.b	4.19E-05	AMR	0.0002084449952	0		0	0	https://www.ncbi.nlm.nih.gov/clinvar/variation/17003/
NM_005476.5(GNE):c.2135T>C	concordant	reportable	reportable	6025	2	0	SCV000331555;S	Pathogenic;Pathogenic	EGL Genetic Diagn	2017-06-30;2017-1	GNE	chr9	36217396	36217396	http://gnomad.b	0.0001316747913	NFE	4.48E-05	0		0	0	https://www.ncbi.nlm.nih.gov/clinvar/variation/6025/
NM_000155.3(GALT):c.253-2A>G	concordant	reportable	reportable	25142	4	0	SCV000228800;S	Pathogenic;Pathogenic;Pat	EGL Genetic Diagn	2017-06-30;2017-1	GALT	chr9	34647487	34647487	http://gnomad.b	1.65E-05	AMR	0.0003275559866	0		0	0	https://www.ncbi.nlm.nih.gov/clinvar/variation/25142/
NM_000155.3(GALT):c.512T>C	concordant	reportable	reportable	3616	3	0	SCV000110063;S	Pathogenic;Pathogenic;Pat	EGL Genetic Diagn	2017-06-30;2017-1	GALT	chr9	34648116	34648116	http://gnomad.b	2.75E-06	AFR	6.53E-05	0		0	0	https://www.ncbi.nlm.nih.gov/clinvar/variation/3616/
NM_000155.3(GALT):c.584T>C	concordant	reportable	reportable	25222	1	0	SCV000110068	Pathogenic	EGL Genetic Diagn	6/30/2017	GALT	chr9	34648350	34648350	http://gnomad.b	6.87E-05	NFE	0.0001521770027	0		0	0	https://www.ncbi.nlm.nih.gov/clinvar/variation/25222/
NM_000155.3(GALT):c.626A>G	concordant	reportable	reportable	25231	2	0	SCV000110069;S	Pathogenic;Pathogenic	EGL Genetic Diagn	2017-06-30;2017-1	GALT	chr9	34648392	34648392	http://gnomad.b	2.47E-05	SAS	3.25E-05	0		0	0	https://www.ncbi.nlm.nih.gov/clinvar/variation/25231/
NM_000155.3(GALT):c.855G>T	concordant	reportable	reportable	3621	2	0	SCV000603795;S	Pathogenic;Pathogenic	ARUP Laboratories	2017-06-30;2017-0	GALT	chr9	34649029	34649029	http://gnomad.b	0.0002171319888	AMR	0.0002382230014	0		0	0	https://www.ncbi.nlm.nih.gov/clinvar/variation/3621/
NM_001005741.2(GBA):c.1297G>T	concordant	reportable	reportable	4292	1	0	SCV000697579	Pathogenic	Laboratory Corpor	1/25/2018	GBA	chr1	155205563	155205563	http://gnomad.b	5.47E-05	ASJ	0.0007108040154	0		0	0	https://www.ncbi.nlm.nih.gov/clinvar/variation/4292/
NM_001005741.2(GBA):c.1342G>C	concordant	reportable	reportable	4293	3	0	SCV000224864;S	Pathogenic;Pathogenic;Pat	EGL Genetic Diagn	2017-06-30;2017-1	GBA	chr1	155205518	155205518	http://gnomad.b	9.51E-05	AMR	0.0002091929928	0		0	0	https://www.ncbi.nlm.nih.gov/clinvar/variation/4293/
NM_001005741.2(GBA):c.1604G>A	concordant	reportable	reportable	4311	2	0	SCV000225538;S	Pathogenic;Pathogenic	EGL Genetic Diagn	2017-06-30;2018-0	GBA	chr1	155204793	155204793	http://gnomad.b	0.0002972665744	ASJ	0.002267299918	0		0	0	https://www.ncbi.nlm.nih.gov/clinvar/variation/4311/
NM_001005741.2(GBA):c.115+1G>A	concordant	reportable	reportable	93445	2	0	SCV000228922;S	Pathogenic;Pathogenic	EGL Genetic Diagn	2017-06-30;2018-0	GBA	chr1	155210420	155210420	http://gnomad.b	0.0001593348839	ASJ	0.0002030460018	0		0	0	https://www.ncbi.nlm.nih.gov/clinvar/variation/93445/
NM_000159.2(GCDH):c.1204C>T	concordant	reportable	reportable	2085	4	0	SCV000225200;S	Pathogenic;Pathogenic;Pat	EGL Genetic Diagn	2017-06-30;2017-1	GCDH	chr19	13008638	13008638	http://gnomad.b	0.0002473967119	FIN	0.0007174889906	0		0	0	https://www.ncbi.nlm.nih.gov/clinvar/variation/2085/
NM_000151.3(G6PC):c.379_380dupTA	concordant	reportable	reportable	11997	1	0	SCV000402979	Pathogenic	Illumina Clinical Se	10/18/2016	G6PC	chr17	41059575	41059575	http://gnomad.b	4.09E-05	AMR	0.0007148390287	0		0	0	https://www.ncbi.nlm.nih.gov/clinvar/variation/11997/
NM_000151.3(G6PC):c.248G>A	concordant	reportable	reportable	38300	1	0	SCV000695637	Pathogenic	Laboratory Corpor	1/25/2018	G6PC	chr17	41055965	41055965	http://gnomad.b	6.39E-05	EAS	0.0004638220125	0		0	0	https://www.ncbi.nlm.nih.gov/clinvar/variation/38300/
NM_000151.3(G6PC):c.247C>T	concordant	reportable	reportable	11998	5	0	SCV000227025;S	Pathogenic;Pathogenic;Pat	EGL Genetic Diagn	2017-06-30;2017-1	G6PC	chr17	41055964	41055964	http://gnomad.b	0.001142421935	ASJ	0.006802029908	1		0	0	https://www.ncbi.nlm.nih.gov/clinvar/variation/11998/
NM_000151.3(G6PC):c.562G>C	concordant	reportable	reportable	12008	1	0	SCV000695639	Pathogenic	Laboratory Corpor	1/25/2018	G6PC	chr17	41061435	41061435	http://gnomad.b	5.11E-05	NFE	0.0001164100031	0		0	0	https://www.ncbi.nlm.nih.gov/clinvar/variation/12008/
NM_001164277.1(SLC37A4):c.1015G>	concordant	reportable	reportable	6921	1	0	SCV000252293	Pathogenic	GeneDx	11/28/2017	SLC37A4	chr11	118896009	118896009	http://gnomad.b	7.54E-05	NFE	0.0001284030004	0		0	0	https://www.ncbi.nlm.nih.gov/clinvar/variation/6921/
NM_000152.3(GAA):c.2560C>T	concordant	reportable	reportable	4034	4	0	SCV000331031;S	Pathogenic;Pathogenic;Pat	EGL Genetic Diagn	2017-06-30;2017-1	GAA	chr17	78092070	78092070	http://gnomad.b	0.0001167308686	AFR	0.002033849945	0		0	0	https://www.ncbi.nlm.nih.gov/clinvar/variation/4034/
NM_000642.2(AGL):c.18_19delGA	concordant	reportable	reportable	195097	2	0	SCV000346051;S	Likely pathogenic;Pathogen	Illumina Clinical Se	2016-10-18;2018-0	AGL	chr1	100316614	100316616	http://gnomad.b	8.93E-05	NFE	8.06E-05	0		0	0	https://www.ncbi.nlm.nih.gov/clinvar/variation/195097/
NM_000642.2(AGL):c.4456delT	concordant	reportable	reportable	1098	1	0	SCV000626759	Pathogenic	Invitae	10/5/2017	AGL	chr1	100382259	100382260		1.67E-05					0	0	https://www.ncbi.nlm.nih.gov/clinvar/variation/1098/
NM_004328.4(BCS1L):c.232A>G	concordant	reportable	reportable	6167	2	0	SCV000617082;S	Pathogenic;Pathogenic	GeneDx;Laborator	2017-11-28;2018-0	BCS1L	chr2	219525942	219525942	http://gnomad.b	5.58E-05	FIN	0.004306090064	0		0	0	https://www.ncbi.nlm.nih.gov/clinvar/variation/6167/
NM_000518.4(HBB):c.79G>A	concordant	reportable	reportable	15161	3	0	SCV000603907;S	Pathogenic;Pathogenic;Pat	ARUP Laboratories	2017-06-30;2017-1	HBB	chr11	5248173	5248173	http://gnomad.b	0.0008584251631	SAS	0.001137029962	1		29	0	https://www.ncbi.nlm.nih.gov/clinvar/variation/15161/
NM_000518.4(HBB):c.364G>C	concordant	reportable	reportable	15152	3	1	SCV000603898;S	Pathogenic;Pathogenic;Pat	ARUP Laboratories	2017-06-30;2017-0	HBB	chr11	5246908	5246908	http://gnomad.b	0.0003219094361	SAS	0.004970440175	4		0	0	https://www.ncbi.nlm.nih.gov/clinvar/variation/15152/
NM_000520.4(HEXA):c.1274_1277dup	concordant	reportable	reportable	3889	4	0	SCV000225230;S	Pathogenic;Pathogenic;Pat	EGL Genetic Diagn	2016-11-03;2017-1	HEXA	chr15	72638920	72638920	http://gnomad.b	0.001554216811	ASJ	0.01350250002	0		0	0	https://www.ncbi.nlm.nih.gov/clinvar/variation/3889/
NM_000071.2(CBS):c.919G>A	concordant	reportable	reportable	117	4	0	SCV000249696;S	Pathogenic;Pathogenic;Pat	GeneDx;Illumina C	2017-11-28;2016-1	CBS	chr21	44483098	44483098	http://gnomad.b	0.0002502685466	NFE	0.0002864410053	0		1	0	https://www.ncbi.nlm.nih.gov/clinvar/variation/117/
NM_000030.2(AGXT):c.731T>C	concordant	reportable	reportable	5646	1	0	SCV000693980	Pathogenic	Laboratory Corpor	1/25/2018	AGXT	chr2	241814576	241814576	http://gnomad.b	5.67E-05	AMR	0.0002382800012	0		0	0	https://www.ncbi.nlm.nih.gov/clinvar/variation/5646/
NM_012203.1(GRHPR):c.103delG	concordant	reportable	reportable	5636	1	0	SCV000331674	Pathogenic	EGL Genetic Diagn	6/30/2017	GRHPR	chr9	37424858	37424859	http://gnomad.b	0.0003349382458	NFE	0.000500774011	0		0	0	https://www.ncbi.nlm.nih.gov/clinvar/variation/5636/
NM_000478.4(ALPL):c.979T>C	concordant	reportable	reportable	13673	2	0	SCV000331658;S	Pathogenic;Pathogenic	EGL Genetic Diagn	2017-06-30;2018-0	ALPL	chr1	21900274	21900274	http://gnomad.b	7.89E-05	EAS	0.00104360003	0		0	0	https://www.ncbi.nlm.nih.gov/clinvar/variation/13673/
NM_000478.4(ALPL):c.1133A>T	concordant	reportable	reportable	13671	1	0	SCV000696798	Pathogenic	Laboratory Corpor	1/25/2018	ALPL	chr1	21902361	21902361	http://gnomad.b	2.82E-06	NFE	8.95E-06	0		0	0	https://www.ncbi.nlm.nih.gov/clinvar/variation/13671/
NM_002225.3(IVD):c.941C>T	concordant	reportable	reportable	100060	4	0	SCV000611275;S	Pathogenic;Pathogenic;Pat	Fulgent Genetics,F	2017-05-23;2017-1	IVD	chr15	40707653	40707653	http://gnomad.b	0.0007565865059	NFE	0.001244719955	0		0	0	https://www.ncbi.nlm.nih.gov/clinvar/variation/100060/
NM_020533.2(MCOLN1):c.406-2A>G	concordant	reportable	reportable	5131	3	0	SCV000230384;S	Pathogenic;Pathogenic;Pat	EGL Genetic Diagn	2017-06-30;2017-1	MCOLN1	chr19	7591645	7591645	http://gnomad.b	0.0004067670818	ASJ	0.004265690222	0		0	0	https://www.ncbi.nlm.nih.gov/clinvar/variation/5131/
NM_000203.3(IDUA):c.208C>T	concordant	reportable	reportable	11909	2	0	SCV000451736;S	Pathogenic;Pathogenic	Illumina Clinical Se	2016-10-18;2018-0	IDUA	chr4	981646	981646	http://gnomad.b	0.0002859331971	FIN	0.002006019931	0		0	0	https://www.ncbi.nlm.nih.gov/clinvar/variation/11909/
NM_017739.3(POMGNT1):c.1539+1G>	concordant	reportable	reportable	56582	4	0	SCV000331070;S	Pathogenic;Pathogenic;Pat	EGL Genetic Diagn	2017-06-30;2017-1	POMGNT1	chr1	46657769	46657769	http://gnomad.b	0.0002043684455	FIN	0.001884759986	0		0	0	https://www.ncbi.nlm.nih.gov/clinvar/variation/56582/
NM_000543.4(SMPD1):c.996delC	concordant	reportable	reportable	2990	1	0	SCV000697420	Pathogenic	Laboratory Corpor	1/25/2018	SMPD1	chr11	6413285	6413286	http://gnomad.b	0.0001569933397	ASJ	0.00132032996	0		0	0	https://www.ncbi.nlm.nih.gov/clinvar/variation/2990/
NM_000543.4(SMPD1):c.911T>C	concordant	reportable	reportable	2989	1	0	SCV000697419	Pathogenic	Laboratory Corpor	1/25/2018	SMPD1	chr11	6413206	6413206	http://gnomad.b	5.95E-05	ASJ	0.0004060910142	0		0	0	https://www.ncbi.nlm.nih.gov/clinvar/variation/2989/
NM_000543.4(SMPD1):c.1493G>T	concordant	reportable	reportable	2980	4	0	SCV000231559;S	Pathogenic;Pathogenic;Pat	EGL Genetic Diagn	2017-06-30;2016-1	SMPD1	chr11	6415434	6415434	http://gnomad.b	0.0002188392008	ASJ	0.002746139886	0		0	0	https://www.ncbi.nlm.nih.gov/clinvar/variation/2980/
NM_002485.4(NBN):c.657_661del5	concordant	reportable	reportable	6940	3	0	SCV000149712;S	Pathogenic;Pathogenic;Pat	GeneDx;Invitae;La	2017-11-28;2017-1	NBN	chr8	90983441	90983446	http://gnomad.b	0.0003402119353	NFE	0.0003771150077	0		0	0	https://www.ncbi.nlm.nih.gov/clinvar/variation/6940/
NM_000310.3(PPT1):c.223A>C	concordant	reportable	reportable	8900	1	0	SCV000242333	Pathogenic	GeneDx	11/28/2017	PPT1	chr1	40558081	40558081	http://gnomad.b	6.41E-05	AFR	6.53E-05	0		0	0	https://www.ncbi.nlm.nih.gov/clinvar/variation/8900/
NM_000310.3(PPT1):c.364A>T	concordant	reportable	reportable	8899	1	0	SCV000242348	Pathogenic	GeneDx	11/28/2017	PPT1	chr1	40557070	40557070	http://gnomad.b	6.41E-05	FIN	0.006418889854	0		0	0	https://www.ncbi.nlm.nih.gov/clinvar/variation/8899/
NM_000441.1(SLC26A4):c.1001+1G>A	concordant	reportable	reportable	4819	3	0	SCV000296920;S	Pathogenic;Pathogenic;Pat	Division of Genomi	2016-01-06;2017-1	SLC26A4	chr7	107323983	107323983	http://gnomad.b	0.0002289492347	NFE	0.0003882760066	0		0	0	https://www.ncbi.nlm.nih.gov/clinvar/variation/4819/
NM_000277.1(PAH):c.1066-11G>A	concordant	reportable	reportable	607	5	0	SCV000110356;S	Pathogenic;Pathogenic;Pat	EGL Genetic Diagn	2017-06-30;2017-0	PAH	chr12	103237568	103237568	http://gnomad.b	0.0003260148926	NFE	0.0004043930094	0		0	0	https://www.ncbi.nlm.nih.gov/clinvar/variation/607/
NM_000277.1(PAH):c.1315+1G>A	concordant	reportable	reportable	576	5	0	SCV000110366;S	Pathogenic;Pathogenic;Pat	EGL Genetic Diagn	2017-06-30;2017-0	PAH	chr12	103234177	103234177	http://gnomad.b	0.0005150487379	NFE	0.0008236640133	0		0	0	https://www.ncbi.nlm.nih.gov/clinvar/variation/576/
NM_000277.1(PAH):c.143T>C	concordant	reportable	reportable	608	3	0	SCV000239058;S	Pathogenic;Pathogenic;Pat	GeneDx;Invitae;La	2017-11-28;2017-1	PAH	chr12	103306594	103306594	http://gnomad.b	0.0001451999101	AMR	0.0002680170001	0		0	0	https://www.ncbi.nlm.nih.gov/clinvar/variation/608/
NM_000277.1(PAH):c.814G>T	concordant	reportable	reportable	596	1	0	SCV000239074	Pathogenic	GeneDx	7/13/2017	PAH	chr12	103246621	103246621	http://gnomad.b	3.29E-05	NFE	6.27E-05	0		0	0	https://www.ncbi.nlm.nih.gov/clinvar/variation/596/
NM_000277.1(PAH):c.1222C>T	concordant	reportable	reportable	577	6	0	SCV000110363;S	Pathogenic;Pathogenic;Pat	EGL Genetic Diagn	2017-06-30;2017-0	PAH	chr12	103234271	103234271	http://gnomad.b	0.001095848378	NFE	0.001495160046	0		1	0	https://www.ncbi.nlm.nih.gov/clinvar/variation/577/
NM_000277.1(PAH):c.1241A>G	concordant	reportable	reportable	593	6	0	SCV000110364;S	Pathogenic;Pathogenic;Pat	EGL Genetic Diagn	2017-06-30;2017-0	PAH	chr12	103234252	103234252	http://gnomad.b	0.0002684828527	NFE	0.0007341090241	0		0	0	https://www.ncbi.nlm.nih.gov/clinvar/variation/593/
NM_138694.3(PKHD1):c.5895dupA	concordant	reportable	reportable	167486	2	0	SCV000229843;S	Pathogenic;Pathogenic	EGL Genetic Diagn	2016-11-03;2017-1	PKHD1	chr6	51824680	51824680		0.000125098689					0	0	https://www.ncbi.nlm.nih.gov/clinvar/variation/167486/
NM_138694.3(PKHD1):c.107C>T	concordant	reportable	reportable	4108	3	0	SCV000229254;S	Pathogenic;Pathogenic;Pat	EGL Genetic Diagn	2017-06-30;2017-1	PKHD1	chr6	51947999	51947999	http://gnomad.b	0.0004809349598	NFE	0.0008156899712	0		0	0	https://www.ncbi.nlm.nih.gov/clinvar/variation/4108/
NM_000017.2(ACADS):c.319C>T	concordant	reportable	reportable	3826	4	0	SCV000228753;S	Pathogenic;Pathogenic;Pat	EGL Genetic Diagn	2017-06-30;2017-1	ACADS	chr12	121174897	121174897	http://gnomad.b	0.001931376658	ASJ	0.01941110007	2		4	0	https://www.ncbi.nlm.nih.gov/clinvar/variation/3826/
NM_001360.2(DHCR7):c.964-1G>C	concordant	reportable	reportable	93725	8	0	SCV000613096;S	Pathogenic;Pathogenic;Pat	Athena Diagnostics	2017-08-17;2017-0	DHCR7	chr11	71146886	71146886	http://gnomad.b	0.005177817508	ASJ	0.01175460033	0		0	0	https://www.ncbi.nlm.nih.gov/clinvar/variation/93725/
NM_001360.2(DHCR7):c.278C>T	concordant	reportable	reportable	6783	3	0	SCV000512799;S	Pathogenic;Pathogenic;Pat	GeneDx;Invitae;La	2017-11-28;2017-1	DHCR7	chr11	71155082	71155082	http://gnomad.b	5.39E-05	NFE	6.31E-05	0		0	0	https://www.ncbi.nlm.nih.gov/clinvar/variation/6783/
NM_001360.2(DHCR7):c.1055G>A	concordant	reportable	reportable	6795	2	0	SCV000331534;S	Pathogenic;Pathogenic	EGL Genetic Diagn	2017-06-30;2017-0	DHCR7	chr11	71146794	71146794	http://gnomad.b	1.88E-05	AFR	0.0001322750031	0		0	0	https://www.ncbi.nlm.nih.gov/clinvar/variation/6795/
NM_001360.2(DHCR7):c.1054C>T	concordant	reportable	reportable	6787	2	0	SCV000700668;S	Pathogenic;Pathogenic	EGL Genetic Diagn	2017-06-30;2017-1	DHCR7	chr11	71146795	71146795	http://gnomad.b	2.15E-05	SAS	3.25E-05	0		0	0	https://www.ncbi.nlm.nih.gov/clinvar/variation/6787/
NM_000112.3(SLC26A2):c.532C>T	concordant	reportable	reportable	4092	2	0	SCV000491205;S	Pathogenic;Pathogenic	GeneDx;Laborator	2016-12-21;2018-0	SLC26A2	chr5	149357747	149357747	http://gnomad.b	0.0001338158137	AMR	0.0002979210112	0		0	0	https://www.ncbi.nlm.nih.gov/clinvar/variation/4092/
NM_000112.3(SLC26A2):c.835C>T	concordant	reportable	reportable	4089	3	0	SCV000329646;S	Pathogenic;Pathogenic;Pat	GeneDx;Illumina C	2017-11-28;2016-1	SLC26A2	chr5	149359991	149359991	http://gnomad.b	0.00116810054	ASJ	0.001931680017	0		0	0	https://www.ncbi.nlm.nih.gov/clinvar/variation/4089/
NM_000391.3(TPP1):c.851G>T	concordant	reportable	reportable	2647	1	0	SCV000628919	Pathogenic	Invitae	10/5/2017	TPP1	chr11	6637927	6637927	http://gnomad.b	5.60E-06	NFE	1.79E-05	0		0	0	https://www.ncbi.nlm.nih.gov/clinvar/variation/2647/
NM_000391.3(TPP1):c.509-1G>C	concordant	reportable	reportable	2644	7	0	SCV000262910;S	Pathogenic;Pathogenic;Pat	Ambry Genetics;EG	2017-11-14;2017-0	TPP1	chr11	6638385	6638385	http://gnomad.b	0.0004672609555	NFE	0.0008510099724	0		0	0	https://www.ncbi.nlm.nih.gov/clinvar/variation/2644/
NM_199292.2(TH):c.698G>A	concordant	reportable	reportable	12327	1	0	SCV000700791	Pathogenic	EGL Genetic Diagn	6/30/2017	TH	chr11	2189135	2189135	http://gnomad.b	0.0001183158583	EAS	0.0003513290139	0		0	0	https://www.ncbi.nlm.nih.gov/clinvar/variation/12327/
NM_000137.2(FAH):c.1062+5G>A	concordant	reportable	reportable	11870	3	0	SCV000329344;S	Pathogenic;Pathogenic;Pat	GeneDx;Illumina C	2017-11-28;2016-1	FAH	chr15	80472572	80472572	http://gnomad.b	0.000219163185	FIN	0.0006706009735	0		0	0	https://www.ncbi.nlm.nih.gov/clinvar/variation/11870/
NM_000137.2(FAH):c.192G>T	concordant	reportable	reportable	21054	1	0	SCV000695443	Pathogenic	Laboratory Corpor	1/25/2018	FAH	chr15	80450512	80450512	http://gnomad.b	4.44E-05	SAS	0.000454811001	0		0	0	https://www.ncbi.nlm.nih.gov/clinvar/variation/21054/
NM_000137.2(FAH):c.782C>T	concordant	reportable	reportable	21058	2	0	SCV000321620;S	Pathogenic;Pathogenic	GeneDx;Laborator	2017-11-28;2018-0	FAH	chr15	80465431	80465431	http://gnomad.b	0.0004827138506	ASJ	0.003857869888	0		0	0	https://www.ncbi.nlm.nih.gov/clinvar/variation/21058/
NM_000137.2(FAH):c.1069G>T	concordant	reportable	reportable	11871	2	0	SCV000631826;S	Pathogenic;Pathogenic	Invitae;Laboratory	2017-10-05;2018-0	FAH	chr15	80473390	80473390	http://gnomad.b	5.83E-05	NFE	5.37E-05	0		0	0	https://www.ncbi.nlm.nih.gov/clinvar/variation/11871/
NM_000053.3(ATP7B):c.3207C>A	concordant	reportable	reportable	3848	6	0	SCV000602583;S	Pathogenic;Pathogenic;Pat	ARUP Laboratories	2017-06-30;2017-0	ATP7B	chr13	52518281	52518281	http://gnomad.b	0.001386114166	ASJ	0.005281330086	0		0	0	https://www.ncbi.nlm.nih.gov/clinvar/variation/3848/
NM_024649.4(BBS1):c.1169T>G	concordant	reportable	reportable	12143	7	0	SCV000225702;S	Pathogenic;Pathogenic;Pat	EGL Genetic Diagn	2017-06-30;2017-0	BBS1	chr11	66293652	66293652	http://gnomad.b	0.001772278883	NFE	0.002732089954	0		0	0	https://www.ncbi.nlm.nih.gov/clinvar/variation/12143/
NM_000370.3(TTPA):c.744delA	concordant	reportable	reportable	9136	1	0	SCV000696612	Pathogenic	Laboratory Corpor	1/25/2018	TTPA	chr8	63973903	63973904	http://gnomad.b	1.80E-05	AMR	0.0001488980051	0		0	0	https://www.ncbi.nlm.nih.gov/clinvar/variation/9136/
NM_006261.4(PROP1):c.301_302delA	concordant	reportable	reportable	8098	1	0	SCV000456757	Pathogenic	Illumina Clinical Se	10/18/2016	PROP1	chr5	177421146	177421148	http://gnomad.b	0.0003211339974	NFE	0.0003048070066	0		0	0	https://www.ncbi.nlm.nih.gov/clinvar/variation/8098/
NM_000492.3(CFTR):c.1645A>C	concordant	reportable	reportable	38733	1	0	SCV000071529	Pathogenic	CFTR2	6/29/2017	CFTR	chr7	117227853	117227853	http://gnomad.b	1.93E-06	NFE	8.98E-06	0		0	0	https://www.ncbi.nlm.nih.gov/clinvar/variation/38733/
NM_000492.3(CFTR):c.3717+4A>G	concordant	reportable	reportable	7138	1	0	SCV000245988	Pathogenic	CFTR2	6/29/2017	CFTR	chr7	117267828	117267828		1.93E-06					0	0	https://www.ncbi.nlm.nih.gov/clinvar/variation/7138/
NM_000543.4(SMPD1):c.1829_1831d	concordant	reportable	reportable	198093	2	0	SCV000490822;S	Pathogenic;Pathogenic	GeneDx;Invitae	2016-12-21;2017-1	SMPD1	chr11	6415766	6415769	http://gnomad.b	0.0001498572788	ASJ	0.0004066689871	0		0	0	https://www.ncbi.nlm.nih.gov/clinvar/variation/198093/
NM_000051.3(ATM):c.5908C>T	concordant	reportable	reportable	141404	3	0	SCV000293233;S	Pathogenic;Pathogenic;Pat	GeneDx;Invitae;La	2017-11-28;2017-1	ATM	chr11	108181032	108181032	http://gnomad.b	2.23E-05	AMR	0.0002087309986	0		0	0	https://www.ncbi.nlm.nih.gov/clinvar/variation/141404/
NM_000051.3(ATM):c.7517_7520delG	concordant	reportable	reportable	141647	4	0	SCV000687776;S	Pathogenic;Pathogenic;Pat	Color Genomics, In	2017-12-21;2017-1	ATM	chr11	108202168	108202172	http://gnomad.b	8.35E-06	SAS	3.26E-05	0		0	0	https://www.ncbi.nlm.nih.gov/clinvar/variation/141647/
NM_000303.2(PMM2):c.691G>A	concordant	reportable	reportable	7719	3	0	SCV000232516;S	Pathogenic;Pathogenic;Pat	EGL Genetic Diagn	2017-06-30;2017-1	PMM2	chr16	8941632	8941632	http://gnomad.b	6.13E-05	EAS	0.0001159960011	0		0	0	https://www.ncbi.nlm.nih.gov/clinvar/variation/7719/

Supplemental Table 2

NM_000303.2(PMM2):c.338C>T	concordant	reportable	reportable	7723	1	0	SCV000230063	Pathogenic	EGL Genetic Diagn	6/30/2017	PMM2	chr16	8900255	8900255	http://gnomad.b	1.39E-05	SAS	9.75E-05	0		0	0	https://www.ncbi.nlm.nih.gov/clinvar/variation/7723/
NM_000152.3(GAA):c.525delT	concordant	reportable	reportable	4033	4	0	SCV000227027;S	Pathogenic;Pathogenic;Pat	EGL Genetic Diagn	2017-06-30;2017-1	GAA	chr17	78078909	78078910	http://gnomad.b	0.0001111722559	NFE	0.0001994640043	0		0	0	https://www.ncbi.nlm.nih.gov/clinvar/variation/4033/
NM_000487.5(ARSA):c.1210+1G>A	concordant	reportable	reportable	3058	1	0	SCV000627137	Pathogenic	Invitae	10/5/2017	ARSA	chr22	51064006	51064006	http://gnomad.b	5.63E-06	NFE	8.97E-06	0		0	0	https://www.ncbi.nlm.nih.gov/clinvar/variation/3058/
NM_000487.5(ARSA):c.542T>G	concordant	reportable	reportable	3057	2	0	SCV000627144;S	Pathogenic;Pathogenic	Invitae;Laboratory	2017-10-05;2018-0	ARSA	chr22	51065404	51065404	http://gnomad.b	0.0002307376288	FIN	0.0004056250036	0		0	0	https://www.ncbi.nlm.nih.gov/clinvar/variation/3057/
NM_000441.1(SLC26A4):c.2168A>G	concordant	reportable	reportable	4825	1	0	SCV000568753	Pathogenic	GeneDx	11/28/2017	SLC26A4	chr7	107350577	107350577	http://gnomad.b	0.0001675238303	EAS	0.001681550057	0		0	0	https://www.ncbi.nlm.nih.gov/clinvar/variation/4825/
NM_000277.1(PAH):c.838G>A	concordant	reportable	reportable	580	4	0	SCV000110388;S	Pathogenic;Pathogenic;Pat	EGL Genetic Diagn	2017-06-30;2017-0	PAH	chr12	103246597	103246597	http://gnomad.b	0.0001041055959	NFE	7.17E-05	0		0	0	https://www.ncbi.nlm.nih.gov/clinvar/variation/580/
NM_000277.1(PAH):c.1045T>C	concordant	reportable	reportable	615	4	0	SCV000611227;S	Pathogenic;Pathogenic;Pat	Fulgent Genetics,F	2017-05-23;2017-1	PAH	chr12	103238134	103238134	http://gnomad.b	6.85E-05	AMR	0.0001492180018	0		0	0	https://www.ncbi.nlm.nih.gov/clinvar/variation/615/
NM_000288.3(PEX7):c.903+1G>C	concordant	reportable	reportable	7785	3	0	SCV000331543;S	Pathogenic;Pathogenic;Pat	EGL Genetic Diagn	2017-06-30;2018-0	PEX7	chr6	137219380	137219380	http://gnomad.b	0.0001361156483	NFE	0.0001166020011	0		0	0	https://www.ncbi.nlm.nih.gov/clinvar/variation/7785/
NM_001360.2(DHCR7):c.724C>T	concordant	reportable	reportable	21275	4	0	SCV000331535;S	Pathogenic;Pathogenic;Pat	EGL Genetic Diagn	2017-06-30;2017-1	DHCR7	chr11	71150032	71150032	http://gnomad.b	0.0001480915044	AFR	0.0001960780064	0		0	0	https://www.ncbi.nlm.nih.gov/clinvar/variation/21275/
NM_001360.2(DHCR7):c.725G>A	concordant	reportable	reportable	21276	1	0	SCV000697857	Pathogenic	Laboratory Corpor	1/25/2018	DHCR7	chr11	71150031	71150031	http://gnomad.b	4.85E-05	EAS	5.80E-05	0		0	0	https://www.ncbi.nlm.nih.gov/clinvar/variation/21276/
NM_001360.2(DHCR7):c.1228G>A	concordant	reportable	reportable	21272	2	0	SCV000564934;S	Pathogenic;Pathogenic	GeneDx;Laborator	2017-11-28;2018-0	DHCR7	chr11	71146621	71146621	http://gnomad.b	3.23E-05	AMR	0.0003282990074	0		0	0	https://www.ncbi.nlm.nih.gov/clinvar/variation/21272/
NM_000137.2(FAH):c.554-1G>T	concordant	reportable	reportable	11874	4	0	SCV000321623;S	Pathogenic;Pathogenic;Pat	GeneDx;Illumina C	2017-11-28;2016-1	FAH	chr15	80460605	80460605	http://gnomad.b	0.0001803241396	AMR	0.0003573560098	0		0	0	https://www.ncbi.nlm.nih.gov/clinvar/variation/11874/
NM_133647.1(SLC12A6):c.3031C>T	concordant	reportable	reportable	5326	1	0	SCV000390300	Pathogenic	Illumina Clinical Se	10/18/2016	SLC12A6	chr15	34528920	34528920	http://gnomad.b	2.96E-06	NFE	8.96E-06	0		0	0	https://www.ncbi.nlm.nih.gov/clinvar/variation/5326/
NM_000051.3(ATM):c.5763-1050A>G	concordant	reportable	reportable	3021	2	0	SCV000700719;S	Pathogenic;Pathogenic	EGL Genetic Diagn	2017-06-30;2017-1	ATM	chr11	108179837	108179837	http://gnomad.b	2.78E-05	NFE	8.97E-05	0		0	0	https://www.ncbi.nlm.nih.gov/clinvar/variation/3021/
NM_000051.3(ATM):c.7638_7646del9	concordant	reportable	reportable	3019	4	0	SCV000682427;S	Pathogenic;Pathogenic;Pat	Color Genomics, In	2017-10-26;2017-0	ATM	chr11	108202611	108202620	http://gnomad.b	4.46E-05	AFR	6.54E-05	0		0	0	https://www.ncbi.nlm.nih.gov/clinvar/variation/3019/
NM_000051.3(ATM):c.3245_3247delA	concordant	reportable	reportable	3033	3	0	SCV000682111;S	Pathogenic;Pathogenic;Pat	Color Genomics, In	2017-10-26;2017-1	ATM	chr11	108143539	108143542		1.95E-05					0	0	https://www.ncbi.nlm.nih.gov/clinvar/variation/3033/
NM_004004.5(GJB2):c.269T>C	concordant	reportable	reportable	17016	6	0	SCV000603829;S	Pathogenic;Pathogenic;Pat	ARUP Laboratories	2017-06-30;2017-0	GJB2	chr13	20763452	20763452	http://gnomad.b	0.0008354242223	NFE	0.001236469951	0		0	0	https://www.ncbi.nlm.nih.gov/clinvar/variation/17016/
NM_005476.5(GNE):c.1714G>C	concordant	reportable	reportable	6033	1	0	SCV000224876	Pathogenic	EGL Genetic Diagn	6/30/2017	GNE	chr9	36219937	36219937	http://gnomad.b	1.12E-05	EAS	0.0001739330037	0		0	0	https://www.ncbi.nlm.nih.gov/clinvar/variation/6033/
NM_000414.3(HSD17B4):c.46G>A	concordant	reportable	reportable	7655	3	0	SCV000331675;S	Pathogenic;Pathogenic;Pat	EGL Genetic Diagn	2017-06-30;2017-1	HSD17B4	chr5	118788316	118788316	http://gnomad.b	0.0002422958376	NFE	0.0004165009886	0		0	0	https://www.ncbi.nlm.nih.gov/clinvar/variation/7655/
NM_000414.3(HSD17B4):c.1369A>T	concordant	reportable	reportable	7656	2	0	SCV000329591;S	Pathogenic;Pathogenic	GeneDx;Invitae	2017-11-28;2017-1	HSD17B4	chr5	118844871	118844871	http://gnomad.b	3.10E-05	AMR	5.96E-05	0		0	0	https://www.ncbi.nlm.nih.gov/clinvar/variation/7656/
NM_000018.3(ACADVL):c.848T>C	concordant	reportable	reportable	21025	8	0	SCV000602354;S	Pathogenic;Pathogenic;Pat	ARUP Laboratories	2017-06-30;2017-0	ACADVL	chr17	7125591	7125591	http://gnomad.b	0.001078892224	NFE	0.002354480093	2		1	0	https://www.ncbi.nlm.nih.gov/clinvar/variation/21025/
NM_003060.3(SLC22A5):c.760C>T	concordant	reportable	reportable	6426	3	0	SCV000605136;S	Pathogenic;Pathogenic;Pat	ARUP Laboratories	2017-06-30;2017-1	SLC22A5	chr5	131721127	131721127	http://gnomad.b	9.32E-05	EAS	0.001333490014	0		0	0	https://www.ncbi.nlm.nih.gov/clinvar/variation/6426/
NM_000492.3(CFTR):c.1210-7_1210-6	concordant	reportable	reportable	7189	3	2	SCV000602990;S	Pathogenic;Pathogenic;Pat	ARUP Laboratories	2017-06-30;2017-0	CFTR	chr7	117188682	117188684		0.04168849277					543	0	https://www.ncbi.nlm.nih.gov/clinvar/variation/7189/
NM_000352.3(ABCC8):c.1384A>G	concordant	unreportable	unreportable	210068	0	2	SCV000246288;S	Uncertain significance;Likel	Genetic Services La	2015-09-15;2016-1	ABCC8	chr11	17464808	17464808	http://gnomad.b	0.001027271742	NFE	0.001783350017	0		2	0	https://www.ncbi.nlm.nih.gov/clinvar/variation/210068/
NM_000352.3(ABCC8):c.2500C>T	concordant	unreportable	unreportable	303772	0	1	SCV000369322	Likely benign	Illumina Clinical Se	10/18/2016	ABCC8	chr11	17434269	17434269	http://gnomad.b	2.58E-05	EAS	0.0002935309894	0		0	0	https://www.ncbi.nlm.nih.gov/clinvar/variation/303772/
NM_000352.3(ABCC8):c.2797C>T	concordant	reportable	reportable	188864	1	0	SCV000256802	Pathogenic	Division of Genomi	10/28/2015	ABCC8	chr11	17429962	17429962	http://gnomad.b	1.29E-05	SAS	3.25E-05	0		0	0	https://www.ncbi.nlm.nih.gov/clinvar/variation/188864/
NM_000352.3(ABCC8):c.1858C>T	concordant	unreportable	unreportable	210071	0	1	SCV000246291	Likely benign	Genetic Services La	9/15/2015	ABCC8	chr11	17450177	17450177	http://gnomad.b	0.0008001362813	AFR	0.008821220137	1		1	0	https://www.ncbi.nlm.nih.gov/clinvar/variation/210071/
NM_000352.3(ABCC8):c.4411G>A	concordant	reportable	reportable	188931	1	0	SCV000696591	Pathogenic	Laboratory Corpor	1/25/2018	ABCC8	chr11	17416719	17416719		2.32E-05					0	0	https://www.ncbi.nlm.nih.gov/clinvar/variation/188931/
NM_000352.3(ABCC8):c.221G>A	concordant	reportable	reportable	495834	1	0	SCV000696585	Pathogenic	Laboratory Corpor	1/25/2018	ABCC8	chr11	17496502	17496502	http://gnomad.b	1.55E-05	SAS	3.25E-05	0		0	0	https://www.ncbi.nlm.nih.gov/clinvar/variation/495834/
NM_000352.3(ABCC8):c.1678G>A	concordant	unreportable	unreportable	188919	0	1	SCV000369370	Likely benign	Illumina Clinical Se	10/18/2016	ABCC8	chr11	17452500	17452500	http://gnomad.b	0.0008775688246	EAS	0.008603650145	1		0	0	https://www.ncbi.nlm.nih.gov/clinvar/variation/188919/
NM_000352.3(ABCC8):c.4628T>C	concordant	reportable	reportable	188836	1	0	SCV000592989	Likely pathogenic	Genetic Services La	7/5/2017	ABCC8	chr11	17414656	17414656	http://gnomad.b	1.55E-05	NFE	2.69E-05	0		0	0	https://www.ncbi.nlm.nih.gov/clinvar/variation/188836/
NM_000352.3(ABCC8):c.2506C>T	concordant	reportable	reportable	188915	3	0	SCV000612205;S	Pathogenic;Pathogenic;Pat	Athena Diagnostics	2017-08-17;2015-1	ABCC8	chr11	17434263	17434263	http://gnomad.b	1.29E-05	AMR	0.000329795992	0		0	0	https://www.ncbi.nlm.nih.gov/clinvar/variation/188915/
NM_000352.3(ABCC8):c.2992C>T	concordant	reportable	reportable	434053	2	0	SCV000612206;S	Pathogenic;Pathogenic	Athena Diagnostics	2017-08-17;2017-0	ABCC8	chr11	17428605	17428605		2.84E-05					0	0	https://www.ncbi.nlm.nih.gov/clinvar/variation/434053/
NM_000352.3(ABCC8):c.683G>A	concordant	reportable	reportable	217846	2	0	SCV000256806;S	Pathogenic;Pathogenic	Division of Genomi	2015-10-28;2018-0	ABCC8	chr11	17483269	17483269		7.74E-06					0	0	https://www.ncbi.nlm.nih.gov/clinvar/variation/217846/
NM_000352.3(ABCC8):c.62T>A	concordant	reportable	reportable	495835	1	0	SCV000696592	Pathogenic	Laboratory Corpor	1/25/2018	ABCC8	chr11	17498262	17498262	http://gnomad.b	1.81E-05	NFE	0.0001039639974	0		0	0	https://www.ncbi.nlm.nih.gov/clinvar/variation/495835/
NM_000352.3(ABCC8):c.1063G>A	concordant	unreportable	unreportable	210066	0	2	SCV000246286;S	Uncertain significance;Likel	Genetic Services La	2015-09-15;2016-1	ABCC8	chr11	17474779	17474779	http://gnomad.b	0.0004568520058	NFE	0.0009221620276	0		0	0	https://www.ncbi.nlm.nih.gov/clinvar/variation/210066/
NM_000016.4(ACADM):c.616C>T	concordant	reportable	reportable	92267	2	0	SCV000268479;S	Likely pathogenic;Pathoger	ARUP Laboratories	2017-06-28;2017-1	ACADM	chr1	76211507	76211507	http://gnomad.b	5.17E-05	SAS	6.50E-05	0		0	0	https://www.ncbi.nlm.nih.gov/clinvar/variation/92267/
NM_000016.4(ACADM):c.362C>T	concordant	reportable	reportable	3599	2	0	SCV000238594;S	Pathogenic;Pathogenic	GeneDx;Invitae	2017-11-28;2017-1	ACADM	chr1	76199288	76199288	http://gnomad.b	2.99E-05	FIN	4.49E-05	0		0	0	https://www.ncbi.nlm.nih.gov/clinvar/variation/3599/
NM_000016.4(ACADM):c.526G>A	concordant	unreportable	unreportable	495343	0	1	SCV000693958	Uncertain significance	Laboratory Corpor	1/25/2018	ACADM	chr1	76205722	76205722	http://gnomad.b	8.16E-06	AFR	6.53E-05	0		0	0	https://www.ncbi.nlm.nih.gov/clinvar/variation/495343/
NM_000016.4(ACADM):c.580A>G	concordant	reportable	reportable	226100	1	0	SCV000630290	Pathogenic	Invitae	10/5/2017	ACADM	chr1	76205776	76205776	http://gnomad.b	1.09E-05	AMR	0.0001489069982	0		0	0	https://www.ncbi.nlm.nih.gov/clinvar/variation/226100/
NM_000016.4(ACADM):c.583G>A	concordant	reportable	reportable	3594	2	0	SCV000511932;S	Pathogenic;Pathogenic	GeneDx;Invitae	2017-11-28;2017-1	ACADM	chr1	76205779	76205779	http://gnomad.b	5.17E-05	NFE	7.17E-05	0		0	0	https://www.ncbi.nlm.nih.gov/clinvar/variation/3594/
NM_000016.4(ACADM):c.797A>G	concordant	unreportable	unreportable	203540	1	1	SCV000238582;S	Pathogenic;Uncertain signi	GeneDx;Invitae	2017-11-28;2017-1	ACADM	chr1	76215192	76215192	http://gnomad.b	0.0001468620476	AMR	0.0006253349711	0		0	0	https://www.ncbi.nlm.nih.gov/clinvar/variation/203540/
NM_000016.4(ACADM):c.157C>T	concordant	reportable	reportable	92258	1	0	SCV000329673	Pathogenic	GeneDx	11/28/2017	ACADM	chr1	76198367	76198367	http://gnomad.b	1.36E-05	SAS	0.0001624330034	0		0	0	https://www.ncbi.nlm.nih.gov/clinvar/variation/92258/
NM_000017.2(ACADS):c.529T>C	concordant	reportable	reportable	3828	4	0	SCV000703978;S	Pathogenic;Pathogenic;Pat	EGL Genetic Diagn	2017-06-30;2017-0	ACADS	chr12	121175696	121175696	http://gnomad.b	0.0006088650755	AFR	0.007056979928	0		1	0	https://www.ncbi.nlm.nih.gov/clinvar/variation/3828/
NM_000017.2(ACADS):c.1095G>T	concordant	reportable	reportable	203560	1	0	SCV000238615	Pathogenic	GeneDx	11/28/2017	ACADS	chr12	121177107	121177107	http://gnomad.b	7.65E-05	AFR	6.81E-05	0		0	0	https://www.ncbi.nlm.nih.gov/clinvar/variation/203560/
NM_000017.2(ACADS):c.1147C>T	concordant	reportable	reportable	3829	2	0	SCV000238621;S	Pathogenic;Likely pathoger	GeneDx;Invitae	2017-11-28;2017-1	ACADS	chr12	121177159	121177159	http://gnomad.b	0.0001557561821	NFE	0.0001367840014	0		0	0	https://www.ncbi.nlm.nih.gov/clinvar/variation/3829/
NM_000017.2(ACADS):c.136C>T	concordant	reportable	reportable	3825	1	0	SCV000238627	Pathogenic	GeneDx	11/28/2017	ACADS	chr12	121164918	121164918	http://gnomad.b	0.0001387645986	SAS	0.0007796760183	0		0	0	https://www.ncbi.nlm.nih.gov/clinvar/variation/3825/
NM_000017.2(ACADS):c.511C>T	concordant	unreportable	unreportable	3830	1	3	SCV000602350;S	Benign;Pathogenic;Likely b	ARUP Laboratories	2017-06-30;2016-0	ACADS	chr12	121175678	121175678	http://gnomad.b	0.02915472536	FIN	0.06404270232	183		192	0	https://www.ncbi.nlm.nih.gov/clinvar/variation/3830/
NM_000017.2(ACADS):c.425C>G	concordant	unreportable	unreportable	420094	0	1	SCV000568665	Uncertain significance	GeneDx	11/28/2017	ACADS	chr12	121175223	121175223	http://gnomad.b	1.13E-05	NFE	2.69E-05	0		0	0	https://www.ncbi.nlm.nih.gov/clinvar/variation/420094/
NM_000017.2(ACADS):c.988C>T	concordant	reportable	reportable	203557	1	0	SCV000238611	Pathogenic	GeneDx	11/28/2017	ACADS	chr12	121176677	121176677	http://gnomad.b	5.66E-06	AFR	0.001770489966	0		0	0	https://www.ncbi.nlm.nih.gov/clinvar/variation/203557/
NM_000017.2(ACADS):c.1138C>T	concordant	reportable	reportable	3837	1	0	SCV000238620	Pathogenic	GeneDx	11/28/2017	ACADS	chr12	121177150	121177150	http://gnomad.b	4.53E-05	AMR	0.0005069780163	0		0	0	https://www.ncbi.nlm.nih.gov/clinvar/variation/3837/
NM_000017.2(ACADS):c.1058C>T	concordant	reportable	reportable	3836	1	0	SCV000238614	Likely pathogenic	GeneDx	7/13/2017	ACADS	chr12	121176971	121176971	http://gnomad.b	0.0001500923209	NFE	0.0004325899936	0		0	0	https://www.ncbi.nlm.nih.gov/clinvar/variation/3836/
NM_000018.3(ACADVL):c.1153C>T	concordant	unreportable	unreportable	193786	0	1	SCV000225144	Uncertain significance	EGL Genetic Diagn	6/30/2017	ACADVL	chr17	7126527	7126527	http://gnomad.b	4.81E-05	AMR	0.0002084449952	0		0	0	https://www.ncbi.nlm.nih.gov/clinvar/variation/193786/
NM_000018.3(ACADVL):c.652G>A	concordant	unreportable	unreportable	474901	0	1	SCV000654961	Uncertain significance	Invitae	10/5/2017	ACADVL	chr17	7125300	7125300	http://gnomad.b	8.50E-06	SAS	3.25E-05	0		0	0	https://www.ncbi.nlm.nih.gov/clinvar/variation/474901/
NM_000018.3(ACADVL):c.481G>A	concordant	unreportable	unreportable	324987	0	1	SCV000406312	Uncertain significance	Illumina Clinical Se	10/18/2016	ACADVL	chr17	7124860	7124860	http://gnomad.b	4.25E-05	AMR	0.0001786670036	0		0	0	https://www.ncbi.nlm.nih.gov/clinvar/variation/324987/
NM_000018.3(ACADVL):c.664G>A	concordant	reportable	reportable	92289	1	0	SCV000491232	Likely pathogenic	GeneDx	12/21/2016	ACADVL	chr17	7125312	7125312	http://gnomad.b	1.13E-05	SAS	3.25E-05	0		0	0	https://www.ncbi.nlm.nih.gov/clinvar/variation/92289/
NM_000018.3(ACADVL):c.865G>A	concordant	reportable	reportable	370981	1	0	SCV000576558	Pathogenic	GeneDx	11/28/2017	ACADVL	chr17	7125608	7125608	http://gnomad.b	0.000116101263	NFE	0.0002149840002	0		0	0	https://www.ncbi.nlm.nih.gov/clinvar/variation/370981/
NM_000018.3(ACADVL):c.553G>A	concordant	reportable	reportable	203595	2	0	SCV000238670;S	Pathogenic;Likely pathoger	GeneDx;Invitae	2017-11-28;2017-1	ACADVL	chr17	7124932	7124932	http://gnomad.b	1.13E-05	EAS	5.80E-05	0		0	0	https://www.ncbi.nlm.nih.gov/clinvar/variation/203595/
NM_000018.3(ACADVL):c.520G>A	concordant	reportable	reportable	92286	2	0	SCV000231986;S	Pathogenic;Likely pathoger	EGL Genetic Diagn	2017-06-30;2017-1	ACADVL	chr17	7124899	7124899	http://gnomad.b	2.27E-05	AFR	0.0001306850027	0		0	0	https://www.ncbi.nlm.nih.gov/clinvar/variation/92286/
NM_000018.3(ACADVL):c.1322G>A	concordant	reportable	reportable	21016	2	0	SCV000602359;S	Pathogenic;Pathogenic	ARUP Laboratories	2017-06-30;2017-1	ACADVL	chr17	7127184	7127184	http://gnomad.b	3.11E-05	NFE	5.37E-05	0		0	0	https://www.ncbi.nlm.nih.gov/clinvar/variation/21016/
NM_000018.3(ACADVL):c.881G>A	concordant	unreportable	unreportable	203575	0	1	SCV000654973	Uncertain significance	Invitae	10/5/2017	ACADVL	chr17	7125988	7125988	http://gnomad.b	1.98E-05	NFE	9.01E-06	0		0	0	https://www.ncbi.nlm.nih.gov/clinvar/variation/203575/
NM_000018.3(ACADVL):c.1837C>T	concordant	reportable	reportable	1623	2	0	SCV000602366;S	Pathogenic;Pathogenic	ARUP Laboratories	2017-06-30;2017-0	ACADVL	chr17	7128285	7128285	http://gnomad.b	3.40E-05	NFE	8.97E-05	0		0	0	https://www.ncbi.nlm.nih.gov/clinvar/variation/1623/
NM_000022.2(ADA):c.845G>A	concordant	unreportable	unreportable	402341	0	1	SCV000538235	Uncertain significance	Laboratory for Mol	4/3/2017	ADA	chr20	43251229	43251229	http://gnomad.b	8.00E-06	NFE	8.96E-06	0		0	0	https://www.ncbi.nlm.nih.gov/clinvar/variation/402341/
NM_000022.2(ADA):c.320T>C	concordant	reportable	reportable	1965	3	0	SCV000704676;S	Pathogenic;Pathogenic;Pat	EGL Genetic Diagn	2017-06-30;2017-1	ADA	chr20	43255139	43255139	http://gnomad.b	2.40E-05	AMR	0.0002680009929	0		0	0	https://www.ncbi.nlm.nih.gov/clinvar/variation/1965/
NM_000642.2(AGL):c.118C>T	concordant	reportable	reportable	189045	1	0	SCV000680764	Pathogenic	GeneDx	2/7/2018	AGL	chr1	100327094	100327094	http://gnomad.b	5.58E-06	EAS	5.80E-05	0		0	0	https://www.ncbi.nlm.nih.gov/clinvar/variation/189045/
NM_000642.2(AGL):c.3652C>T	concordant	reportable	reportable	456492	1	0	SCV000626733	Pathogenic	Invitae	10/5/2017	AGL	chr1	100368302	100368302	http://gnomad.b	2.79E-06	SAS	3.25E-05	0		0	0	https://www.ncbi.nlm.nih.gov/clinvar/variation/456492/
NM_000642.2(AGL):c.1481G>A	concordant	unreportable	unreportable	256723	0	5	SCV000331433;S	Benign;Benign;Likely benig	EGL Genetic Diagn	2017-06-30;2017-1	AGL	chr1	100343254	100343254	http://gnomad.b	0.005461994898	FIN	0.0313594006	25		9	0	https://www.ncbi.nlm.nih.gov/clinvar/variation/256723/
NM_000642.2(AGL):c.3980G>A	concordant	reportable	reportable	1108	2	0	SCV000331819;S	Pathogenic;Pathogenic	EGL Genetic Diagn	2017-06-30;2017-1	AGL	chr1	100379113	100379113	http://gnomad.b	8.09E-05	AMR	0.0001194959987	0		0	0	https://www.ncbi.nlm.nih.gov/clinvar/variation/1108/
NM_000383.3(AIRE):c.1322C>T	concordant	unreportable	unreportable	35660	0	1	SCV000593079	Uncertain significance	Genetic Services La	7/5/2017	AIRE	chr21	45713715	45713715	http://gnomad.b	0.001832593426	ASJ	0.003579460084	0		2	0	https://www.ncbi.nlm.nih.gov/clinvar/variation/35660/
NM_000383.3(AIRE):c.232T>C	concordant	reportable	reportable	189060	1	0	SCV000696652	Pathogenic	Laboratory Corpor	1/25/2018	AIRE	chr21	45706539	45706539	http://gnomad.b	4.18E-05	NFE	1.79E-05	0		0	0	https://www.ncbi.nlm.nih.gov/clinvar/variation/189060/
NM_000383.3(AIRE):c.415C>T	concordant	reportable	reportable	3310	1	0	SCV000490396	Pathogenic	GeneDx	12/21/2016	AIRE	chr21	45706968	45706968	http://gnomad.b	1.95E-05	AFR	7.18E-05	0		0	0	https://www.ncbi.nlm.nih.gov/clinvar/variation/3310/
NM_000382.2(ALDH3A2):c.733G>A	concordant	reportable	reportable	265459	1	0	SCV000322393	Pathogenic	GeneDx	11/28/2017	ALDH3A2	chr17	19561110	19561110	http://gnomad.b	1.12E-05	NFE	8.95E-06	0		0	0	https://www.ncbi.nlm.nih.gov/clinvar/variation/265459/
NM_000382.2(ALDH3A2):c.529C>T	concordant	reportable	reportable	265027	2	1	SCV000339070;S	Pathogenic;Pathogenic;Un	EGL Genetic Diagn	2017-06-30;2017-1	ALDH3A2	chr17	19559736	19559736	http://gnomad.b	2.80E-06	AMR	0.0004168899904	0		0	0	https://www.ncbi.nlm.nih.gov/clinvar/variation/265027/
NM_000478.4(ALPL):c.1250A>G	concordant	reportable	reportable	13679	1	0	SCV000332907	Likely pathogenic	EGL Genetic Diagn	6/30/2017	ALPL	chr1	21903075	21903075	http://gnomad.b	2.82E-05	NFE	4.48E-05	0		0	0	https://www.ncbi.nlm.nih.gov/clinvar/variation/13679/
NM_000478.4(ALPL):c.455G>A	concordant	unreportable	unreportable	197679	0	5	SCV000602471;S	Benign;Benign;Benign;Likel	ARUP Laboratories	2017-06-30;2017-0	ALPL	chr1	21889760	21889760	http://gnomad.b	0.01206024205	SAS	0.02919539995	44		41	0	https://www.ncbi.nlm.nih.gov/clinvar/variation/197679/
NM_000478.4(ALPL):c.1349G>A	concordant	unreportable	unreportable	429390	0	1	SCV000581949	Uncertain significance	GeneDx	11/28/2017	ALPL	chr1	21903915	21903915	http://gnomad.b	9.30E-05	SAS	0.0004235910019	1		0	0	https://www.ncbi.nlm.nih.gov/clinvar/variation/429390/
NM_000478.4(ALPL):c.668G>A	concordant	reportable	reportable	381586	1	0	SCV000521021	Likely pathogenic	GeneDx	11/28/2017	ALPL	chr1	21894616	21894616	http://gnomad.b	3.10E-05	SAS	3.25E-05	0		0	0	https://www.ncbi.nlm.nih.gov/clinvar/variation/381586/
NM_000478.4(ALPL):c.407G>A	concordant	reportable	reportable	13675	1	0	SCV000354296	Pathogenic	Illumina Clinical Se	10/18/2016	ALPL	chr1	21889712	21889712	http://gnomad.b	0.0001098690586	NFE	0.0002424830018	0		0	0	https://www.ncbi.nlm.nih.gov/clinvar/variation/13675/
NM_000487.5(ARSA):c.827C>T	concordant	reportable	reportable	3075	2	0	SCV000627146;S	Pathogenic;Pathogenic	Invitae;Laboratory	2017-10-05;2018-0	ARSA	chr22	51065046	51065046	http://gnomad.b	3.10E-05			0		0	0	https://www.ncbi.nlm.nih.gov/clinvar/variation/3075/
NM_000487.5(ARSA):c.847G>T	concordant	unreportable	unreportable	68151	0	1	SCV000110814	Uncertain significance	EGL Genetic Diagn	6/30/2017	ARSA	chr22	51065026	51065026		2.81E-06					0	0	https://www.ncbi.nlm.nih.gov/clinvar/variation/68151/
NM_000487.5(ARSA):c.346C>T	concordant	reportable	reportable	280950	1	0	SCV000330912	Pathogenic	EGL Genetic Diagn	6/30/2017	ARSA	chr22	51065713	51065713	http://gnomad.b	5.63E-06	FIN	5.62E-05	0		0	0	https://www.ncbi.nlm.nih.gov/clinvar/variation/280950/
NM_000487.5(ARSA):c.937C>T	concordant	reportable	reportable	434394	1	0	SCV000593421	Pathogenic	Genetic Services La	7/5/2017	ARSA	chr22	51064624	51064624	http://gnomad.b	2.25E-05	NFE	2.79E-05	0		0	0	https://www.ncbi.nlm.nih.gov/clinvar/variation/434394/
NM_000487.5(ARSA):c.511G>A	concordant	unreportable	unreportable	3069	0	1	SCV000627143	Likely benign	Invitae	10/5/2017	ARSA	chr22	51065435	51065435	http://gnomad.b	0.0003376648226	EAS	0.002280439949	0		0	0	https://www.ncbi.nlm.nih.gov/clinvar/variation/3069/
NM_000487.5(ARSA):c.656G>A	concordant	unreportable	unreportable	196276	0	1	SCV000228889	Uncertain significance	EGL Genetic Diagn	6/30/2017	ARSA	chr22	51065290	51065290	http://gnomad.b	8.16E-05	AFR	0.0005236970028	0		0	0	https://www.ncbi.nlm.nih.gov/clinvar/variation/196276/
NM_000487.5(ARSA):c.1115G>A	concordant	unreportable	unreportable	3082	0	1	SCV000627135	Uncertain significance	Invitae	10/5/2017	ARSA	chr22	51064102	51064102	http://gnomad.b	1.13E-05	NFE	8.99E-06	0		0	0	https://www.ncbi.nlm.nih.gov/clinvar/variation/3082/
NM_000487.5(ARSA):c.370G>A	concordant	unreportable	unreportable	3063	0	1	SCV000110806	Uncertain significance	EGL Genetic Diagn	6/30/2017	ARSA	chr22	51065689	51065689	http://gnomad.b	8.44E-06	EAS	6.57E-05	0		0	0	https://www.ncbi.nlm.nih.gov/clinvar/variation/3063/
NM_000487.5(ARSA):c.763G>A	concordant	reportable	reportable	3091	1	0	SCV000329854	Pathogenic	GeneDx	11/28/2017	ARSA	chr22	51065110	51065110	http://gnomad.b	2.81E-06	NFE	8.96E-06	0		0	0	https://www.ncbi.nlm.nih.gov/clinvar/variation/3091/
NM_000487.5(ARSA):c.769G>C	concordant	reportable	reportable	21187	3	0	SCV000331188;S	Pathogenic;Pathogenic;Pat	EGL Genetic Diagn	2017-06-30;2017-1	ARSA	chr22	51065104	51065104	http://gnomad.b	3.94E-05	AMR	5.96E-05	0		0	0	https://www.ncbi.nlm.nih.gov/clinvar/variation/21187/
NM_000487.5(ARSA):c.302G>A	concordant	reportable	reportable	3053	1	0	SCV000700718	Likely pathogenic	EGL Genetic Diagn	6/30/2017	ARSA	chr22	51065757	51065757		8.44E-06					0	0	https://www.ncbi.nlm.nih.gov/clinvar/variation/3053/
NM_000487.5(ARSA):c.960G>A	concordant	reportable	reportable	495883	1	0	SCV000696812	Pathogenic	Laboratory Corpor	1/25/2018	ARSA	chr22	51064601	51064601	http://gnomad.b	1.69E-05	EAS	0.0002495319932	0		0	0	https://www.ncbi.nlm.nih.gov/clinvar/variation/495883/
NM_000487.5(ARSA):c.746T>C	concordant	reportable	reportable	68149	1	0	SCV000627145	Likely pathogenic	Invitae	10/5/2017	ARSA	chr22	51065127	51065127	http://gnomad.b	5.63E-06	SAS	3.25E-05	0		0	0	https://www.ncbi.nlm.nih.gov/clinvar/variation/68149/
NM_000049.2(ASPA):c.212G>A	concordant	reportable	reportable	2616	1	0	SCV000401980	Likely pathogenic	Illumina Clinical Se	10/18/2016	ASPA	chr17	3379665	3379665	http://gnomad.b	7.10E-05	FIN	0.002071700059	0		0	0	https://www.ncbi.nlm.nih.gov/clinvar/variation/2616/
NM_000049.2(ASPA):c.859G>A	concordant	reportable	reportable	188803	1	0	SCV000694158	Pathogenic	Laboratory Corpor	1/25/2018	ASPA	chr17	3402299	3402299		9.46E-06					0	0	https://www.ncbi.nlm.nih.gov/clinvar/variation/188803/
NM_000049.2(ASPA):c.79G>A	concordant	reportable	reportable	188888	1	0	SCV000577172	Pathogenic	GeneDx	11/28/2017	ASPA	chr17	3379532	3379532	http://gnomad.b	2.37E-05	AMR	0.0002084449952	0		0	0	https://www.ncbi.nlm.nih.gov/clinvar/variation/188888/
NM_000050.4(ASS1):c.256C>T	concordant	reportable	reportable	6332	1	0	SCV000630054	Pathogenic	Invitae	10/5/2017	ASS1	chr9	133333869	133333869	http://gnomad.b	1.67E-05	AMR	0.0002084570006	0		0	0	https://www.ncbi.nlm.nih.gov/clinvar/variation/6332/
NM_000050.4(ASS1):c.814C>T	concordant	reportable	reportable	371132	1	0	SCV000630064	Pathogenic	Invitae	10/5/2017	ASS1	chr9	133355812	133355812	http://gnomad.b	8.36E-06	AMR	2.98E-05	0		0	0	https://www.ncbi.nlm.nih.gov/clinvar/variation/371132/
NM_000050.4(ASS1):c.1087C>T	concordant	reportable	reportable	6328	3	0	SCV000602545;S	Likely pathogenic;Pathoger	ARUP Laboratories	2017-06-30;2017-1	ASS1	chr9	133370370	133370370	http://gnomad.b	7.24E-05	AFR	6.56E-05	0		0	0	https://www.ncbi.nlm.nih.gov/clinvar/variation/6328/

Supplemental Table 2

NM_000050.4(ASS1):c.919C>T	concordant	unreportable	unreportable	265044	1	1	SCV000321410;S	Likely pathogenic;Uncertai	GeneDx;Invitae	2017-11-28;2017-1	ASS1	chr9	133364800	133364800	http://gnomad.b	0.0001169740485	AMR	0.0002680009929	0		0	0	https://www.ncbi.nlm.nih.gov/clinvar/variation/265044/
NM_000050.4(ASS1):c.535T>C	concordant	reportable	reportable	6335	1	0	SCV000329824	Pathogenic	GeneDx	11/28/2017	ASS1	chr9	133346260	133346260	http://gnomad.b	6.41E-05	NFE	0.0001342879987	0		0	0	https://www.ncbi.nlm.nih.gov/clinvar/variation/6335/
NM_000051.3(ATM):c.4362A>C	concordant	unreportable	unreportable	127386	0	3	SCV000682199;S	Uncertain significance;Unc	Color Genomics, In	2017-10-26;2017-1	ATM	chr11	108160454	108160454	http://gnomad.b	0.0002339155231	NFE	0.0006275780033	0		0	0	https://www.ncbi.nlm.nih.gov/clinvar/variation/127386/
NM_000051.3(ATM):c.670A>G	concordant	unreportable	unreportable	142522	0	6	SCV000682362;S	Uncertain significance;Unc	Color Genomics, In	2017-10-26;2017-0	ATM	chr11	108115522	108115522	http://gnomad.b	9.19E-05	NFE	0.0001974189945	0		0	0	https://www.ncbi.nlm.nih.gov/clinvar/variation/142522/
NM_000051.3(ATM):c.4066A>G	concordant	unreportable	unreportable	127380	0	4	SCV000149094;S	Uncertain significance;Unc	GeneDx;Genetic Se	2017-11-28;2017-0	ATM	chr11	108158399	108158399	http://gnomad.b	0.0002534084833	NFE	0.0002657430014	0		0	0	https://www.ncbi.nlm.nih.gov/clinvar/variation/127380/
NM_000051.3(ATM):c.9008A>G	concordant	unreportable	unreportable	181905	0	2	SCV000209670;S	Uncertain significance;Unc	GeneDx;Invitae	2017-11-28;2017-1	ATM	chr11	108236072	108236072		1.39E-05					0	0	https://www.ncbi.nlm.nih.gov/clinvar/variation/181905/
NM_000051.3(ATM):c.5262G>T	concordant	unreportable	unreportable	236734	0	3	SCV000682258;S	Uncertain significance;Unc	Color Genomics, In	2017-10-26;2017-1	ATM	chr11	108172459	108172459	http://gnomad.b	1.39E-05	NFE	2.69E-05	0		0	0	https://www.ncbi.nlm.nih.gov/clinvar/variation/236734/
NM_000051.3(ATM):c.2924A>G	concordant	unreportable	unreportable	181937	0	1	SCV000547050	Uncertain significance	Invitae	10/5/2017	ATM	chr11	108141980	108141980	http://gnomad.b	8.35E-06	EAS	5.80E-05	0		0	0	https://www.ncbi.nlm.nih.gov/clinvar/variation/181937/
NM_000051.3(ATM):c.3467C>T	concordant	unreportable	unreportable	183918	0	2	SCV000566875;S	Uncertain significance;Unc	GeneDx;Invitae	2017-11-28;2017-1	ATM	chr11	108151786	108151786	http://gnomad.b	2.23E-05	NFE	2.69E-05	0		0	0	https://www.ncbi.nlm.nih.gov/clinvar/variation/183918/
NM_000051.3(ATM):c.1369C>T	concordant	reportable	reportable	219999	2	0	SCV000687305;S	Pathogenic;Pathogenic	Color Genomics, In	2017-12-21;2017-1	ATM	chr11	108121561	108121561	http://gnomad.b	5.57E-06	NFE	8.96E-06	0		0	0	https://www.ncbi.nlm.nih.gov/clinvar/variation/219999/
NM_000051.3(ATM):c.6100C>T	concordant	reportable	reportable	127417	3	0	SCV000292138;S	Pathogenic;Pathogenic;Pat	Color Genomics, In	2016-07-05;2017-1	ATM	chr11	108186742	108186742	http://gnomad.b	1.39E-05	SAS	3.25E-05	0		0	0	https://www.ncbi.nlm.nih.gov/clinvar/variation/127417/
NM_000051.3(ATM):c.8977C>T	concordant	reportable	reportable	186330	5	0	SCV000687865;S	Pathogenic;Pathogenic;Pat	Color Genomics, In	2017-12-21;2017-0	ATM	chr11	108235935	108235935	http://gnomad.b	3.34E-05	NFE	2.69E-05	0		0	0	https://www.ncbi.nlm.nih.gov/clinvar/variation/186330/
NM_000051.3(ATM):c.6679C>T	concordant	reportable	reportable	181981	3	0	SCV000687719;S	Pathogenic;Pathogenic;Pat	Color Genomics, In	2017-12-21;2017-1	ATM	chr11	108196143	108196143		5.57E-06					0	0	https://www.ncbi.nlm.nih.gov/clinvar/variation/181981/
NM_000051.3(ATM):c.2158C>T	concordant	unreportable	unreportable	407475	0	2	SCV000566507;S	Uncertain significance;Unc	GeneDx;Invitae	2017-11-28;2017-1	ATM	chr11	108126975	108126975	http://gnomad.b	2.78E-06	AFR	0.0001307530038	0		0	0	https://www.ncbi.nlm.nih.gov/clinvar/variation/407475/
NM_000051.3(ATM):c.9022C>T	concordant	reportable	reportable	142187	2	0	SCV000209774;S	Pathogenic;Pathogenic	GeneDx;Invitae	2017-11-28;2017-1	ATM	chr11	108236086	108236086	http://gnomad.b	5.57E-06	AFR	6.53E-05	0		0	0	https://www.ncbi.nlm.nih.gov/clinvar/variation/142187/
NM_000051.3(ATM):c.6095G>A	concordant	reportable	reportable	181974	4	0	SCV000682303;S	Pathogenic;Pathogenic;Pat	Color Genomics, In	2017-10-26;2017-1	ATM	chr11	108186638	108186638	http://gnomad.b	1.67E-05	NFE	6.27E-05	0		0	0	https://www.ncbi.nlm.nih.gov/clinvar/variation/181974/
NM_000051.3(ATM):c.4279G>A	concordant	unreportable	unreportable	141884	0	2	SCV000278825;S	Uncertain significance;Unc	GeneDx;Invitae	2017-11-28;2017-1	ATM	chr11	108160371	108160371	http://gnomad.b	5.29E-05	AFR	0.000131216002	0		0	0	https://www.ncbi.nlm.nih.gov/clinvar/variation/141884/
NM_000051.3(ATM):c.6315G>C	concordant	unreportable	unreportable	135767	0	4	SCV000537592;S	Uncertain significance;Unc	Color Genomics, In	2017-12-21;2017-1	ATM	chr11	108188216	108188216	http://gnomad.b	0.0001893601854	ASJ	0.001117659966	0		0	0	https://www.ncbi.nlm.nih.gov/clinvar/variation/135767/
NM_000051.3(ATM):c.2276G>A	concordant	unreportable	unreportable	185556	0	3	SCV000687373;S	Uncertain significance;Unc	Color Genomics, In	2017-12-21;2017-1	ATM	chr11	108128233	108128233		2.78E-05					0	0	https://www.ncbi.nlm.nih.gov/clinvar/variation/185556/
NM_000051.3(ATM):c.5821G>C	concordant	unreportable	unreportable	127412	0	4	SCV000537538;S	Uncertain significance;Unc	Color Genomics, In	2017-12-21;2017-0	ATM	chr11	108180945	108180945	http://gnomad.b	0.0001002495099	NFE	0.0002422310063	0		0	0	https://www.ncbi.nlm.nih.gov/clinvar/variation/127412/
NM_000051.3(ATM):c.2T>C	concordant	reportable	reportable	187275	3	0	SCV000616967;S	Pathogenic;Pathogenic;Pat	GeneDx;Invitae;La	2017-11-28;2017-1	ATM	chr11	108098353	108098353	http://gnomad.b	1.11E-05	NFE	8.96E-06	0		0	0	https://www.ncbi.nlm.nih.gov/clinvar/variation/187275/
NM_000051.3(ATM):c.2336T>C	concordant	unreportable	unreportable	133606	0	1	SCV000617367	Uncertain significance	GeneDx	11/28/2017	ATM	chr11	108128293	108128293	http://gnomad.b	1.11E-05	SAS	3.25E-05	0		0	0	https://www.ncbi.nlm.nih.gov/clinvar/variation/133606/
NM_000051.3(ATM):c.7592T>C	concordant	unreportable	unreportable	140910	0	3	SCV000344511;S	Uncertain significance;Unc	EGL Genetic Diagn	2017-06-30;2017-1	ATM	chr11	108202247	108202247	http://gnomad.b	4.46E-05	NFE	8.06E-05	0		0	0	https://www.ncbi.nlm.nih.gov/clinvar/variation/140910/
NM_000051.3(ATM):c.8000T>C	concordant	unreportable	unreportable	407502	0	2	SCV000546743;S	Uncertain significance;Unc	Invitae;Laboratory	2017-03-14;2018-0	ATM	chr11	108204685	108204685	http://gnomad.b	1.67E-05	NFE	8.97E-06	0		0	0	https://www.ncbi.nlm.nih.gov/clinvar/variation/407502/
NM_000051.3(ATM):c.3G>A	concordant	reportable	reportable	188901	4	0	SCV000682174;S	Pathogenic;Pathogenic;Pat	Color Genomics, In	2017-10-26;2017-1	ATM	chr11	108098354	108098354	http://gnomad.b	1.39E-05	AFR	6.54E-05	0		0	0	https://www.ncbi.nlm.nih.gov/clinvar/variation/188901/
NM_000051.3(ATM):c.3630G>A	concordant	unreportable	unreportable	133616	0	4	SCV000682153;S	Uncertain significance;Unc	Color Genomics, In	2017-10-26;2017-1	ATM	chr11	108153490	108153490	http://gnomad.b	4.18E-05	NFE	3.59E-05	0		0	0	https://www.ncbi.nlm.nih.gov/clinvar/variation/133616/
NM_000051.3(ATM):c.692A>G	concordant	unreportable	unreportable	142096	0	3	SCV000682377;S	Uncertain significance;Unc	Color Genomics, In	2017-10-26;2017-1	ATM	chr11	108115544	108115544	http://gnomad.b	1.95E-05	NFE	2.69E-05	0		0	0	https://www.ncbi.nlm.nih.gov/clinvar/variation/142096/
NM_000051.3(ATM):c.6332A>G	concordant	unreportable	unreportable	229957	0	3	SCV000682325;S	Uncertain significance;Unc	Color Genomics, In	2017-10-26;2017-1	ATM	chr11	108188233	108188233		1.11E-05					0	0	https://www.ncbi.nlm.nih.gov/clinvar/variation/229957/
NM_000051.3(ATM):c.5644C>T	concordant	reportable	reportable	188737	1	0	SCV000261294	Pathogenic	Invitae	3/14/2017	ATM	chr11	108175549	108175549		1.11E-05					0	0	https://www.ncbi.nlm.nih.gov/clinvar/variation/188737/
NM_000051.3(ATM):c.7456C>T	concordant	reportable	reportable	127445	2	0	SCV000149160;S	Pathogenic;Pathogenic	GeneDx;Invitae	2017-11-28;2017-1	ATM	chr11	108201089	108201089	http://gnomad.b	2.78E-06	SAS	6.50E-05	0		0	0	https://www.ncbi.nlm.nih.gov/clinvar/variation/127445/
NM_000051.3(ATM):c.9139C>T	concordant	reportable	reportable	3029	3	0	SCV000682541;S	Likely pathogenic;Pathogen	Color Genomics, In	2017-10-26;2017-1	ATM	chr11	108236203	108236203	http://gnomad.b	1.67E-05	SAS	9.75E-05	0		0	0	https://www.ncbi.nlm.nih.gov/clinvar/variation/3029/
NM_000051.3(ATM):c.37C>T	concordant	unreportable	unreportable	185977	0	3	SCV000293426;S	Uncertain significance;Unc	GeneDx;Invitae;La	2017-11-28;2017-0	ATM	chr11	108098388	108098388	http://gnomad.b	2.78E-05	EAS	0.0001739330037	0		0	0	https://www.ncbi.nlm.nih.gov/clinvar/variation/185977/
NM_000051.3(ATM):c.3161C>G	concordant	unreportable	unreportable	132695	0	2	SCV000602551;S	Benign;Benign	ARUP Laboratories	2017-06-30;2017-1	ATM	chr11	108143456	108143456	http://gnomad.b	0.01953473089	NFE	0.02663370036	59		103	0	https://www.ncbi.nlm.nih.gov/clinvar/variation/132695/
NM_000051.3(ATM):c.749G>A	concordant	unreportable	unreportable	140915	0	2	SCV000209681;S	Uncertain significance;Unc	GeneDx;Invitae	2017-11-28;2017-1	ATM	chr11	108115601	108115601	http://gnomad.b	7.24E-05	AMR	8.94E-05	1		0	0	https://www.ncbi.nlm.nih.gov/clinvar/variation/140915/
NM_000051.3(ATM):c.2414G>A	concordant	unreportable	unreportable	142128	0	2	SCV000564618;S	Uncertain significance;Unc	GeneDx;Invitae	2017-11-28;2017-1	ATM	chr11	108129750	108129750	http://gnomad.b	4.18E-05	SAS	0.0001624540018	0		0	0	https://www.ncbi.nlm.nih.gov/clinvar/variation/142128/
NM_000051.3(ATM):c.2771G>A	concordant	unreportable	unreportable	142194	0	3	SCV000682076;S	Uncertain significance;Unc	Color Genomics, In	2017-10-26;2017-1	ATM	chr11	108139269	108139269	http://gnomad.b	3.06E-05	SAS	0.0003898380091	0		0	0	https://www.ncbi.nlm.nih.gov/clinvar/variation/142194/
NM_000051.3(ATM):c.8156G>A	concordant	unreportable	unreportable	127456	0	4	SCV000682464;S	Uncertain significance;Unc	Color Genomics, In	2017-10-26;2017-1	ATM	chr11	108206576	108206576	http://gnomad.b	0.0001169577615	AMR	0.000507039018	1		0	0	https://www.ncbi.nlm.nih.gov/clinvar/variation/127456/
NM_000051.3(ATM):c.3848T>C	concordant	unreportable	unreportable	181994	0	1	SCV000546658	Uncertain significance	Invitae	10/5/2017	ATM	chr11	108155055	108155055		5.57E-06					0	0	https://www.ncbi.nlm.nih.gov/clinvar/variation/181994/
NM_000051.3(ATM):c.6200C>A	concordant	reportable	reportable	39749	1	0	SCV000547142	Pathogenic	Invitae	10/5/2017	ATM	chr11	108188101	108188101	http://gnomad.b	1.11E-05	NFE	8.96E-06	0		0	0	https://www.ncbi.nlm.nih.gov/clinvar/variation/39749/
NM_000051.3(ATM):c.1339C>T	concordant	reportable	reportable	127337	4	0	SCV000537674;S	Pathogenic;Pathogenic;Pat	Color Genomics, In	2017-01-26;2017-1	ATM	chr11	108121531	108121531	http://gnomad.b	3.06E-05	AFR	6.54E-05	0		0	0	https://www.ncbi.nlm.nih.gov/clinvar/variation/127337/
NM_000051.3(ATM):c.2413C>T	concordant	reportable	reportable	216021	3	0	SCV000682052;S	Pathogenic;Pathogenic;Pat	Color Genomics, In	2017-10-26;2017-1	ATM	chr11	108129749	108129749	http://gnomad.b	5.57E-06	EAS	0.000347986992	0		0	0	https://www.ncbi.nlm.nih.gov/clinvar/variation/216021/
NM_000051.3(ATM):c.7327C>T	concordant	reportable	reportable	3036	4	0	SCV000687767;S	Pathogenic;Pathogenic;Pat	Color Genomics, In	2017-12-21;2017-1	ATM	chr11	108200960	108200960	http://gnomad.b	2.78E-06	NFE	8.98E-06	0		0	0	https://www.ncbi.nlm.nih.gov/clinvar/variation/3036/
NM_000051.3(ATM):c.8287C>T	concordant	reportable	reportable	232612	2	0	SCV000622814;S	Pathogenic;Pathogenic	Invitae;Laboratory	2017-10-05;2018-0	ATM	chr11	108213967	108213967		2.78E-06					0	0	https://www.ncbi.nlm.nih.gov/clinvar/variation/232612/
NM_000051.3(ATM):c.8560C>T	concordant	unreportable	unreportable	142147	0	4	SCV000537553;S	Uncertain significance;Unc	Color Genomics, In	2017-12-21;2017-0	ATM	chr11	108216611	108216611	http://gnomad.b	0.0001448048476	AMR	0.000477041991	0		0	0	https://www.ncbi.nlm.nih.gov/clinvar/variation/142147/
NM_000051.3(ATM):c.590G>A	concordant	unreportable	unreportable	420008	0	1	SCV000568313	Uncertain significance	GeneDx	11/28/2017	ATM	chr11	108114773	108114773	http://gnomad.b	2.78E-06	SAS	0.0002275239967	0		0	0	https://www.ncbi.nlm.nih.gov/clinvar/variation/420008/
NM_000051.3(ATM):c.902G>A	concordant	unreportable	unreportable	188359	0	2	SCV000564611;S	Uncertain significance;Unc	GeneDx;Invitae	2017-11-28;2017-1	ATM	chr11	108117691	108117691	http://gnomad.b	6.68E-05	NFE	0.0001167719965	0		0	0	https://www.ncbi.nlm.nih.gov/clinvar/variation/188359/
NM_000051.3(ATM):c.7271T>G	concordant	reportable	reportable	3023	6	0	SCV000602560;S	Pathogenic;Pathogenic;Pat	ARUP Laboratories	2017-06-30;2016-0	ATM	chr11	108199929	108199929	http://gnomad.b	3.62E-05	NFE	8.97E-05	0		0	0	https://www.ncbi.nlm.nih.gov/clinvar/variation/3023/
NM_000051.3(ATM):c.1229T>C	concordant	unreportable	unreportable	127332	0	6	SCV000681959;S	Benign;Likely benign;Benig	Color Genomics, In	2017-10-26;2017-0	ATM	chr11	108119823	108119823	http://gnomad.b	0.002250044555	ASJ	0.004168360028	3		0	0	https://www.ncbi.nlm.nih.gov/clinvar/variation/127332/
NM_000051.3(ATM):c.4709T>C	concordant	unreportable	unreportable	127394	0	4	SCV000149108;S	Uncertain significance;Unc	GeneDx;Genetic Se	2017-07-13;2017-0	ATM	chr11	108164137	108164137	http://gnomad.b	0.0005068169667	NFE	0.0007275009993	0		0	0	https://www.ncbi.nlm.nih.gov/clinvar/variation/127394/
NM_000051.3(ATM):c.8147T>C	concordant	reportable	reportable	142700	3	0	SCV000682458;S	Likely pathogenic;Likely pat	Color Genomics, In	2017-10-26;2017-1	ATM	chr11	108205832	108205832	http://gnomad.b	3.90E-05	NFE	5.37E-05	0		0	0	https://www.ncbi.nlm.nih.gov/clinvar/variation/142700/
NM_000051.3(ATM):c.7291A>G	concordant	unreportable	unreportable	220522	0	2	SCV000617373;S	Uncertain significance;Unc	GeneDx;Invitae	2017-11-28;2016-0	ATM	chr11	108199949	108199949		1.39E-05					0	0	https://www.ncbi.nlm.nih.gov/clinvar/variation/220522/
NM_000051.3(ATM):c.4318A>T	concordant	reportable	reportable	407482	1	0	SCV000546697	Pathogenic	Invitae	3/14/2017	ATM	chr11	108160410	108160410		5.57E-06					0	0	https://www.ncbi.nlm.nih.gov/clinvar/variation/407482/
NM_000051.3(ATM):c.5890A>G	concordant	unreportable	unreportable	142126	0	5	SCV000537568;S	Uncertain significance;Unc	Color Genomics, In	2017-12-21;2017-0	ATM	chr11	108181014	108181014	http://gnomad.b	0.0001642978079	AMR	0.000208655998	1		0	0	https://www.ncbi.nlm.nih.gov/clinvar/variation/142126/
NM_000051.3(ATM):c.8266A>T	concordant	reportable	reportable	135780	4	0	SCV000682472;S	Pathogenic;Pathogenic;Pat	Color Genomics, In	2017-10-26;2017-1	ATM	chr11	108206686	108206686	http://gnomad.b	4.73E-05	NFE	3.60E-05	0		0	0	https://www.ncbi.nlm.nih.gov/clinvar/variation/135780/
NM_000051.3(ATM):c.7311C>A	concordant	reportable	reportable	453669	1	0	SCV000622727	Pathogenic	Invitae	10/5/2017	ATM	chr11	108200944	108200944		5.57E-06					0	0	https://www.ncbi.nlm.nih.gov/clinvar/variation/453669/
NM_000051.3(ATM):c.3372C>G	concordant	reportable	reportable	127371	3	0	SCV000611164;S	Pathogenic;Pathogenic;Pat	Fulgent Genetics,F	2017-05-23;2017-1	ATM	chr11	108150305	108150305	http://gnomad.b	1.67E-05	NFE	8.99E-06	0		0	0	https://www.ncbi.nlm.nih.gov/clinvar/variation/127371/
NM_000051.3(ATM):c.295A>G	concordant	unreportable	unreportable	127364	0	4	SCV000611354;S	Uncertain significance;Unc	Fulgent Genetics,F	2017-05-23;2017-1	ATM	chr11	108100014	108100014	http://gnomad.b	0.0004399839601	ASJ	0.003861000063	0		0	0	https://www.ncbi.nlm.nih.gov/clinvar/variation/127364/
NM_000051.3(ATM):c.5882A>G	concordant	unreportable	unreportable	127413	0	4	SCV000292183;S	Uncertain significance;Unc	Color Genomics, In	2017-12-21;2017-1	ATM	chr11	108181006	108181006	http://gnomad.b	3.62E-05	NFE	6.28E-05	0		0	0	https://www.ncbi.nlm.nih.gov/clinvar/variation/127413/
NM_000051.3(ATM):c.1648A>G	concordant	unreportable	unreportable	185780	0	2	SCV000512139;S	Likely benign;Uncertain sig	GeneDx;Invitae	2017-11-28;2017-1	ATM	chr11	108122604	108122604	http://gnomad.b	7.52E-05	SAS	9.75E-05	0		0	0	https://www.ncbi.nlm.nih.gov/clinvar/variation/185780/
NM_000051.3(ATM):c.802C>T	concordant	reportable	reportable	188961	2	0	SCV000617365;S	Pathogenic;Likely pathogen	GeneDx;Laborator	2017-11-28;2018-0	ATM	chr11	108115654	108115654	http://gnomad.b	2.78E-06	NFE	8.97E-06	0		0	0	https://www.ncbi.nlm.nih.gov/clinvar/variation/188961/
NM_000051.3(ATM):c.1960C>A	concordant	unreportable	unreportable	135739	0	2	SCV000322091;S	Uncertain significance;Unc	GeneDx;Invitae	2017-11-28;2016-0	ATM	chr11	108124602	108124602	http://gnomad.b	5.29E-05	SAS	6.50E-05	0		0	0	https://www.ncbi.nlm.nih.gov/clinvar/variation/135739/
NM_000051.3(ATM):c.3886C>T	concordant	unreportable	unreportable	220775	0	2	SCV000682168;S	Uncertain significance;Unc	Color Genomics, In	2017-10-26;2016-0	ATM	chr11	108155093	108155093		2.78E-06					0	0	https://www.ncbi.nlm.nih.gov/clinvar/variation/220775/
NM_000051.3(ATM):c.4396C>T	concordant	reportable	reportable	236716	2	0	SCV000282958;S	Pathogenic;Pathogenic	Invitae;Laboratory	2017-10-05;2018-0	ATM	chr11	108160488	108160488	http://gnomad.b	2.78E-06	SAS	6.50E-05	0		0	0	https://www.ncbi.nlm.nih.gov/clinvar/variation/236716/
NM_000051.3(ATM):c.4852C>T	concordant	reportable	reportable	187207	3	0	SCV000682226;S	Pathogenic;Pathogenic;Pat	Color Genomics, In	2017-10-26;2017-1	ATM	chr11	108165729	108165729	http://gnomad.b	5.57E-06	SAS	3.25E-05	0		0	0	https://www.ncbi.nlm.nih.gov/clinvar/variation/187207/
NM_000051.3(ATM):c.5188C>T	concordant	reportable	reportable	189054	2	0	SCV000682254;S	Pathogenic;Pathogenic	Color Genomics, In	2017-10-26;2017-1	ATM	chr11	108172385	108172385	http://gnomad.b	2.78E-06	NFE	1.80E-05	0		0	0	https://www.ncbi.nlm.nih.gov/clinvar/variation/189054/
NM_000051.3(ATM):c.7792C>T	concordant	reportable	reportable	265383	1	0	SCV000322214	Likely pathogenic	GeneDx	11/28/2017	ATM	chr11	108203492	108203492	http://gnomad.b	2.78E-06	AMR	2.98E-05	0		0	0	https://www.ncbi.nlm.nih.gov/clinvar/variation/265383/
NM_000051.3(ATM):c.8071C>T	concordant	unreportable	unreportable	133636	0	4	SCV000682453;S	Uncertain significance;Unc	Color Genomics, In	2017-10-26;2017-1	ATM	chr11	108205756	108205756	http://gnomad.b	0.0001030342185	EAS	0.0006384950248	0		0	0	https://www.ncbi.nlm.nih.gov/clinvar/variation/133636/
NM_000051.3(ATM):c.1009C>T	concordant	unreportable	unreportable	127327	0	4	SCV000681943;S	Uncertain significance;Unc	Color Genomics, In	2017-10-26;2017-0	ATM	chr11	108117798	108117798	http://gnomad.b	8.91E-05	AMR	0.0001789020025	0		0	0	https://www.ncbi.nlm.nih.gov/clinvar/variation/127327/
NM_000051.3(ATM):c.2941C>T	concordant	unreportable	unreportable	135747	0	1	SCV000166095	Uncertain significance	Invitae	1/6/2016	ATM	chr11	108141997	108141997	http://gnomad.b	5.57E-06	NFE	1.80E-05	0		0	0	https://www.ncbi.nlm.nih.gov/clinvar/variation/135747/
NM_000051.3(ATM):c.7775C>G	concordant	unreportable	unreportable	186713	0	3	SCV000687787;S	Uncertain significance;Unc	Color Genomics, In	2017-12-21;2017-1	ATM	chr11	108202751	108202751	http://gnomad.b	1.39E-05	NFE	3.60E-05	0		0	0	https://www.ncbi.nlm.nih.gov/clinvar/variation/186713/
NM_000051.3(ATM):c.6814G>A	concordant	unreportable	unreportable	265315	0	4	SCV000708587;S	Uncertain significance;Unc	EGL Genetic Diagn	2017-06-30;2017-1	ATM	chr11	108196791	108196791		5.57E-06					0	0	https://www.ncbi.nlm.nih.gov/clinvar/variation/265315/
NM_000051.3(ATM):c.170G>A	concordant	reportable	reportable	127341	3	0	SCV000292162;S	Pathogenic;Pathogenic;Pat	Color Genomics, In	2016-07-05;2017-1	ATM	chr11	108098600	108098600		5.57E-06					0	0	https://www.ncbi.nlm.nih.gov/clinvar/variation/127341/
NM_000051.3(ATM):c.6067G>A	concordant	unreportable	unreportable	127416	0	3	SCV000149130;S	Uncertain significance;Likel	GeneDx;Invitae;La	2017-11-28;2017-1	ATM	chr11	108186610	108186610	http://gnomad.b	0.001475895562	NFE	0.002462520031	1		0	0	https://www.ncbi.nlm.nih.gov/clinvar/variation/127416/
NM_000051.3(ATM):c.8307G>A	concordant	reportable	reportable	189104	2	0	SCV000682476;S	Pathogenic;Pathogenic	Color Genomics, In	2017-10-26;2017-1	ATM	chr11	108213987	108213987	http://gnomad.b	5.57E-06	EAS	5.80E-05	0		0	0	https://www.ncbi.nlm.nih.gov/clinvar/variation/189104/
NM_000051.3(ATM):c.1837G>T	concordant	unreportable	unreportable	181922	0	3	SCV000682004;S	Uncertain significance;Unc	Color Genomics, In	2017-10-26;2017-1	ATM	chr11	108123578	108123578	http://gnomad.b	3.90E-05	NFE	4.49E-05	0		0	0	https://www.ncbi.nlm.nih.gov/clinvar/variation/181922/
NM_000051.3(ATM):c.2789T>G	concordant	reportable	reportable	186898	1	0	SCV000622360	Pathogenic	Invitae	10/5/2017	ATM	chr11	108139287	108139287		2.78E-06					0	0	https://www.ncbi.nlm.nih.gov/clinvar/variation/186898/
NM_000051.3(ATM):c.1880T>G	concordant	unreportable	unreportable	232928	0	3	SCV000682010;S	Uncertain significance;Unc	Color Genomics, In	2017-10-26;2017-1	ATM	chr11	108123621	108123621	http://gnomad.b	2.51E-05	NFE	2.69E-05	0		0	0	https://www.ncbi.nlm.nih.gov/clinvar/variation/232928/
NM_000051.3(ATM):c.4388T>G	concordant	unreportable	unreportable	127388	0	5	SCV000602567;S	Likely benign;Likely benign,	ARUP Laboratories	2017-06-30;2017-0	ATM	chr11	108160480	108160480	http://gnomad.b	0.003274817323	ASJ	0.02702699974	6		11	0	https://www.ncbi.nlm.nih.gov/clinvar/variation/127388/
NM_000053.3(ATP7B):c.2921C>T	concordant	unreportable	unreportable	289465	0	1	SCV000343834	Uncertain significance	EGL Genetic Diagn	6/30/2017	ATP7B	chr13	52520559	52520559	http://gnomad.b	0.0002497250219	AFR	0.0003269260051	0		0	0	https://www.ncbi.nlm.nih.gov/clinvar/variation/289465/
NM_000053.3(ATP7B):c.2930C>T	concordant	reportable	reportable	35710	4	0	SCV000225744;S	Pathogenic;Pathogenic;Pat	EGL Genetic Diagn	2017-06-30;2017-0	ATP7B	chr13	52520550	52520550	http://gnomad.b	0.0001010123684	SAS	0.0001624849974	0		0	0	https://www.ncbi.nlm.nih.gov/clinvar/variation/35710/
NM_000053.3(ATP7B):c.2972C>T	concordant	unreportable	unreportable	35712	1	3	SCV000602616;S	Uncertain significance;Unc	ARUP Laboratories	2017-06-30;2017-0	ATP7B	chr13	52520508	52520508	http://gnomad.b	0.001453455745	NFE	0.002417410025	0		3	0	https://www.ncbi.nlm.nih.gov/clinvar/variation/35712/
NM_000053.3(ATP7B):c.3247C>T	concordant	reportable	reportable	495412	1	0	SCV000694441	Pathogenic	Laboratory Corpor	1/25/2018	ATP7B	chr13	52516687	52516687	http://gnomad.b	8.42E-06	EAS	0.0001159550011	0		0	0	https://www.ncbi.nlm.nih.gov/clinvar/variation/495412/
NM_000053.3(ATP7B):c.2570T>C	concordant	reportable	reportable	377538	1	0	SCV000512210	Likely pathogenic	GeneDx	11/28/2017	ATP7B	chr13	52524413	52524413		2.81E-06					0	0	https://www.ncbi.nlm.nih.gov/clinvar/variation/377538/
NM_000053.3(ATP7B):c.1995G>A	concordant	unreportable	unreportable	157933	1	4	SCV000331190;S	Uncertain significance;Likel	EGL Genetic Diagn	2017-06-30;2017-1	ATP7B	chr13	52534410	52534410	http://gnomad.b	0.00207917125	ASJ	0.006294419989	2		0	0	https://www.ncbi.nlm.nih.gov/clinvar/variation/157933/
NM_000053.3(ATP7B):c.3305T>C	concordant	reportable	reportable	430725	1	0	SCV000694443	Pathogenic	Laboratory Corpor	1/25/2018	ATP7B	chr13	52516629	52516629	http://gnomad.b	5.61E-06	SAS	0.0001299459982	0		0	0	https://www.ncbi.nlm.nih.gov/clinvar/variation/430725/
NM_000053.3(ATP7B):c.3443T>C	concordant	reportable	reportable	37122	1	0	SCV000709306	Pathogenic	EGL Genetic Diagn	6/30/2017	ATP7B	chr13	52515330	52515330	http://gnomad.b	3.93E-05	EAS	0.0004058440099	0		0	0	https://www.ncbi.nlm.nih.gov/clinvar/variation/37122/
NM_000053.3(ATP7B):c.1969A>C	concordant	unreportable	unreportable	157932	0	1	SCV000694409	Uncertain significance	Laboratory Corpor	1/25/2018	ATP7B	chr13	52534436	52534436	http://gnomad.b	5.61E-06	NFE	1.80E-05	0		0	0	https://www.ncbi.nlm.nih.gov/clinvar/variation/157932/
NM_000053.3(ATP7B):c.2305A>G	concordant	reportable	reportable	35706	3	0	SCV000232468;S	Pathogenic;Pathogenic;Pat	EGL Genetic Diagn	2017-06-30;2017-1	ATP7B	chr13	52532497	52532497	http://gnomad.b	0.0001431008552	NFE	0.000134307993	0		0	0	https://www.ncbi.nlm.nih.gov/clinvar/variation/35706/
NM_000053.3(ATP7B):c.2519C>T	concordant	reportable	reportable	188879	2	0	SCV000329796;S	Pathogenic;Pathogenic	GeneDx;Laborator	2017-11-28;2018-0	ATP7B	chr13	52524464	52524464		5.61E-06					0	0	https://www.ncbi.nlm.nih.gov/clinvar/variation/188879/
NM_000053.3(ATP7B):c.3818C>T	concordant	reportable	reportable	189139	1	0	SCV000694455	Pathogenic	Laboratory Corpor	1/25/2018	ATP7B	chr13	52511697	52511697	http://gnomad.b	1.96E-05	AFR	0.0001307530038	0		0	0	https://www.ncbi.nlm.nih.gov/clinvar/variation/189139/
NM_000053.3(ATP7B):c.3955C>T	concordant	reportable	reportable	35728	4	0	SCV000602617;S	Pathogenic;Pathogenic;Pat	ARUP Laboratories	2017-06-30;2017-0	ATP7B	chr13	52511478	52511478	http://gnomad.b	0.0001094300658	AMR	0.0001488889975	0		0	0	https://www.ncbi.nlm.nih.gov/clinvar/variation/35728/
NM_000053.3(ATP7B):c.2755C>G	concordant	reportable	reportable	3857	2	0	SCV000626846;S	Pathogenic;Pathogenic	Invitae;Laboratory	2017-10-05;2018-0	ATP7B	chr13	52523908	52523908	http://gnomad.b	4.77E-05	EAS	0.000348230009	0		0	0	https://www.ncbi.nlm.nih.gov/clinvar/variation/3857/
NM_000053.3(ATP7B):c.2332C>T	concordant	reportable	reportable	456553	2	0	SCV000626843;S	Pathogenic;Pathogenic	Invitae;Laboratory	2017-10-05;2018-0	ATP7B	chr13	52532470	52532470	http://gnomad.b	2.53E-05	AFR	0.0001962069946	0		0	0	https://www.ncbi.nlm.nih.gov/clinvar/variation/456553/
NM_000053.3(ATP7B):c.3895C>T	concordant	reportable	reportable	189149	1	0	SCV000626861	Pathogenic	Invitae	10/5/2017	ATP7B	chr13	52511620	52511620	http://gnomad.b	1.68E-05	SAS	0.0002598919964	0		0	0	https://www.ncbi.nlm.nih.gov/clinvar/variation/189149/
NM_000053.3(ATP7B):c.1621G>A	concordant	unreportable	unreportable	92386	0	1	SCV000694401	Uncertain significance	Laboratory Corpor	1/25/2018	ATP7B	chr13	52542666	52542666	http://gnomad.b	0.0001431008552	NFE	0.0001881549979	0		0	0	https://www.ncbi.nlm.nih.gov/clinvar/variation/92386/
NM_000053.3(ATP7B):c.3517G>A	concordant	reportable	reportable	189050	2	0	SCV000626855;S	Pathogenic;Pathogenic	Invitae;Laboratory	2017-10-05;2018-0	ATP7B	chr13	52515256	52515256	http://gnomad.b	1.12E-05	ASJ	0.0001015230009	0		0	0	https://www.ncbi.nlm.nih.gov/clinvar/variation/189050/
NM_000053.3(ATP7B):c.3007G>A	concordant	reportable	reportable	188802	1	0	SCV000626849	Pathogenic	Invitae	10/5/2017	ATP7B	chr13	52520473	52520473	http://gnomad.b	2.53E-05	NFE	6.54E-05	0		0	0	https://www.ncbi.nlm.nih.gov/clinvar/variation/188802/

Supplemental Table 2

NM_000053.3(ATP7B):c.2128G>A	concordant	reportable	reportable	156281	4	0	SCV000702263;S(	Pathogenic;Pathogenic;Pat	EGL Genetic Diagn	2017-06-30;2017-1	ATP7B	chr13	52532674	52532674	http://gnomad.b	5.33E-05	NFE	3.60E-05	0		0	0	https://www.ncbi.nlm.nih.gov/clinvar/variation/156281/
NM_000053.3(ATP7B):c.3646G>A	concordant	reportable	reportable	188859	1	0	SCV000334638	Likely pathogenic	EGL Genetic Diagn	6/30/2017	ATP7B	chr13	52513240	52513240	http://gnomad.b	1.12E-05	FIN	0.000762331998	0		0	0	https://www.ncbi.nlm.nih.gov/clinvar/variation/188859/
NM_000053.3(ATP7B):c.3191A>C	concordant	reportable	reportable	370081	1	0	SCV000694439	Pathogenic	Laboratory Corpora	1/25/2018	ATP7B	chr13	52518297	52518297	http://gnomad.b	0.000274978114	ASJ	0.003250049893	0		0	0	https://www.ncbi.nlm.nih.gov/clinvar/variation/370081/
NM_000053.3(ATP7B):c.4051C>T	concordant	reportable	reportable	188947	1	0	SCV000702098	Pathogenic	EGL Genetic Diagn	6/30/2017	ATP7B	chr13	52509802	52509802	http://gnomad.b	1.12E-05	NFE	9.15E-06	0		0	0	https://www.ncbi.nlm.nih.gov/clinvar/variation/188947/
NM_000053.3(ATP7B):c.2621C>T	concordant	reportable	reportable	3851	1	0	SCV000626845	Pathogenic	Invitae	10/5/2017	ATP7B	chr13	52524252	52524252	http://gnomad.b	7.86E-05	EAS	0.0004638220125	0		0	0	https://www.ncbi.nlm.nih.gov/clinvar/variation/3851/
NM_000053.3(ATP7B):c.2479C>T	concordant	unreportable	unreportable	285881	0	1	SCV000339092	Uncertain significance	EGL Genetic Diagn	6/30/2017	ATP7B	chr13	52524504	52524504	http://gnomad.b	5.61E-05	AFR	0.0001961300004	0		0	0	https://www.ncbi.nlm.nih.gov/clinvar/variation/285881/
NM_000053.3(ATP7B):c.3121C>T	concordant	reportable	reportable	312384	1	0	SCV000384660	Likely pathogenic	Illumina Clinical Se	10/18/2016	ATP7B	chr13	52518367	52518367	http://gnomad.b	3.37E-05	NFE	7.18E-05	0		0	0	https://www.ncbi.nlm.nih.gov/clinvar/variation/312384/
NM_000053.3(ATP7B):c.2828G>A	concordant	reportable	reportable	189058	1	0	SCV000694433	Pathogenic	Laboratory Corpora	1/25/2018	ATP7B	chr13	52523835	52523835	http://gnomad.b	2.24E-05	EAS	0.000289921998	0		0	0	https://www.ncbi.nlm.nih.gov/clinvar/variation/189058/
NM_000053.3(ATP7B):c.1607T>C	concordant	unreportable	unreportable	35703	0	4	SCV000331695;S(	Benign;Uncertain significar	EGL Genetic Diagn	2017-06-30;2017-1	ATP7B	chr13	52542680	52542680	http://gnomad.b	0.002407461447	FIN	0.0113003999	1		1	0	https://www.ncbi.nlm.nih.gov/clinvar/variation/35703/
NM_000053.3(ATP7B):c.3688A>G	concordant	unreportable	unreportable	312379	0	2	SCV000384649;S(	Uncertain significance;Unc	Illumina Clinical Se	2016-10-18;2017-0	ATP7B	chr13	52513198	52513198	http://gnomad.b	0.0004012435745	NFE	0.0005729219993	0		1	0	https://www.ncbi.nlm.nih.gov/clinvar/variation/312379/
NM_000053.3(ATP7B):c.865C>T	concordant	reportable	reportable	3864	1	0	SCV000520886	Pathogenic	GeneDx	11/28/2017	ATP7B	chr13	52548491	52548491	http://gnomad.b	8.42E-06	NFE	5.43E-05	0		0	0	https://www.ncbi.nlm.nih.gov/clinvar/variation/3864/
NM_000053.3(ATP7B):c.1915C>T	concordant	unreportable	unreportable	500690	0	1	SCV000706740	Uncertain significance	EGL Genetic Diagn	6/30/2017	ATP7B	chr13	52536004	52536004	http://gnomad.b	0.0001066241666	NFE	0.0001074129977	0		0	0	https://www.ncbi.nlm.nih.gov/clinvar/variation/500690/
NM_000053.3(ATP7B):c.314C>A	concordant	reportable	reportable	189193	2	0	SCV000329798;S(	Pathogenic;Pathogenic	GeneDx;Laborator	2017-11-28;2018-0	ATP7B	chr13	52549042	52549042	http://gnomad.b	1.12E-05	EAS	5.80E-05	0		0	0	https://www.ncbi.nlm.nih.gov/clinvar/variation/189193/
NM_000053.3(ATP7B):c.813C>A	concordant	reportable	reportable	188930	1	0	SCV000700720	Pathogenic	EGL Genetic Diagn	6/30/2017	ATP7B	chr13	52548543	52548543	http://gnomad.b	3.37E-05	SAS	0.0007476760075	0		0	0	https://www.ncbi.nlm.nih.gov/clinvar/variation/188930/
NM_000053.3(ATP7B):c.1922T>C	concordant	unreportable	unreportable	420002	0	2	SCV000568298;S(	Uncertain significance;Unc	GeneDx;Laborator	2017-11-28;2018-0	ATP7B	chr13	52535997	52535997	http://gnomad.b	0.0005948506139	AMR	0.0008040019893	0		0	0	https://www.ncbi.nlm.nih.gov/clinvar/variation/420002/
NM_024649.4(BBS1):c.157A>G	concordant	unreportable	unreportable	462953	0	1	SCV000636515	Uncertain significance	Invitae	10/5/2017	BBS1	chr11	66278708	66278708	http://gnomad.b	5.57E-06	EAS	0.0001739330037	0		0	0	https://www.ncbi.nlm.nih.gov/clinvar/variation/462953/
NM_024649.4(BBS1):c.1285C>T	concordant	reportable	reportable	188983	1	0	SCV000699513	Likely pathogenic	Laboratory Corpora	1/25/2018	BBS1	chr11	66294224	66294224	http://gnomad.b	1.67E-05	SAS	3.25E-05	0		0	0	https://www.ncbi.nlm.nih.gov/clinvar/variation/188983/
NM_024649.4(BBS1):c.24T>C	concordant	unreportable	unreportable	193457	0	2	SCV000224650;S(	Benign;Benign	EGL Genetic Diagn	2017-06-30;2016-0	BBS1	chr11	66278154	66278154	http://gnomad.b	0.005099481692	ASJ	0.01555829961	5		7	0	https://www.ncbi.nlm.nih.gov/clinvar/variation/193457/
NM_024685.3(BBS10):c.1736A>G	concordant	unreportable	unreportable	35751	0	1	SCV000290895	Uncertain significance	Invitae	6/10/2016	BBS10	chr12	76740029	76740029	http://gnomad.b	0.000436461306	AFR	0.004773740191	0		0	0	https://www.ncbi.nlm.nih.gov/clinvar/variation/35751/
NM_024685.3(BBS10):c.145C>T	concordant	reportable	reportable	225010	2	0	SCV000262879;S(	Pathogenic;Pathogenic	Ambry Genetics;Ill	2016-04-08;2016-1	BBS10	chr12	76741994	76741994	http://gnomad.b	8.39E-05	NFE	9.98E-05	0		0	0	https://www.ncbi.nlm.nih.gov/clinvar/variation/225010/
NM_000709.3(BCKDHA):c.793C>T	concordant	unreportable	unreportable	2379	1	1	SCV000706771;S(	Uncertain significance;Path	EGL Genetic Diagn	2017-06-30;2017-1	BCKDHA	chr19	41928215	41928215	http://gnomad.b	2.37E-05	SAS	3.25E-05	0		0	0	https://www.ncbi.nlm.nih.gov/clinvar/variation/2379/
NM_000709.3(BCKDHA):c.1312T>A	concordant	reportable	reportable	100009	3	0	SCV000238735;S(	Pathogenic;Pathogenic;Pat	GeneDx;Genetic Se	2017-11-28;2017-0	BCKDHA	chr19	41930487	41930487	http://gnomad.b	5.54E-05	NFE	0.0001457170001	0		0	0	https://www.ncbi.nlm.nih.gov/clinvar/variation/100009/
NM_000709.3(BCKDHA):c.761C>A	concordant	unreportable	unreportable	93371	0	1	SCV000231570	Uncertain significance	EGL Genetic Diagn	6/30/2017	BCKDHA	chr19	41928183	41928183		1.58E-05					0	0	https://www.ncbi.nlm.nih.gov/clinvar/variation/93371/
NM_183050.2(BCKDHB):c.970C>T	concordant	reportable	reportable	96621	2	0	SCV000233196;S(	Pathogenic;Likely pathoger	EGL Genetic Diagn	2017-06-30;2016-1	BCKDHB	chr6	80982870	80982870	http://gnomad.b	2.31E-05	AMR	0.0002089049958	0		0	0	https://www.ncbi.nlm.nih.gov/clinvar/variation/96621/
NM_183050.2(BCKDHB):c.1016C>T	concordant	reportable	reportable	96563	1	0	SCV000233195	Pathogenic	EGL Genetic Diagn	6/30/2017	BCKDHB	chr6	80982916	80982916		1.79E-05					0	0	https://www.ncbi.nlm.nih.gov/clinvar/variation/96563/
NM_183050.2(BCKDHB):c.502C>T	concordant	unreportable	unreportable	96586	0	1	SCV000114788	Uncertain significance	EGL Genetic Diagn	6/30/2017	BCKDHB	chr6	80878616	80878616	http://gnomad.b	7.69E-06	NFE	8.96E-06	0		0	0	https://www.ncbi.nlm.nih.gov/clinvar/variation/96586/
NM_000057.2(BLM):c.3847C>T	concordant	reportable	reportable	42089	1	0	SCV000623319	Pathogenic	Invitae	10/5/2017	BLM	chr15	91352462	91352462	http://gnomad.b	4.76E-06	AMR	2.98E-05	0		0	0	https://www.ncbi.nlm.nih.gov/clinvar/variation/42089/
NM_000057.2(BLM):c.2015A>G	concordant	unreportable	unreportable	188963	0	1	SCV000623256	Uncertain significance	Invitae	10/5/2017	BLM	chr15	91306328	91306328	http://gnomad.b	2.38E-05	NFE	5.38E-05	0		0	0	https://www.ncbi.nlm.nih.gov/clinvar/variation/188963/
NM_000057.2(BLM):c.1642C>T	concordant	reportable	reportable	127478	4	0	SCV000331817;S(	Pathogenic;Pathogenic;Pat	EGL Genetic Diagn	2017-06-30;2017-1	BLM	chr15	91304245	91304245	http://gnomad.b	0.0001593492841	NFE	0.0002832190075	0		0	0	https://www.ncbi.nlm.nih.gov/clinvar/variation/127478/
NM_000057.2(BLM):c.1933C>T	concordant	reportable	reportable	454091	2	0	SCV000623255;S(	Pathogenic;Pathogenic	Invitae;Laboratory	2017-10-05;2018-0	BLM	chr15	91306246	91306246	http://gnomad.b	5.95E-05	NFE	8.98E-05	0		0	0	https://www.ncbi.nlm.nih.gov/clinvar/variation/454091/
NM_000057.2(BLM):c.2098C>T	concordant	reportable	reportable	42067	2	0	SCV000329113;S(	Pathogenic;Pathogenic	GeneDx;Invitae	2017-11-28;2017-0	BLM	chr15	91308549	91308549	http://gnomad.b	2.38E-05	NFE	8.95E-06	0		0	0	https://www.ncbi.nlm.nih.gov/clinvar/variation/42067/
NM_000060.2(BTD):c.1466A>C	concordant	unreportable	unreportable	1903	0	1	SCV000441834	Uncertain significance	Illumina Clinical Se	10/18/2016	BTD	chr3	15686829	15686829	http://gnomad.b	2.31E-05	EAS	0.0002319380001	0		0	0	https://www.ncbi.nlm.nih.gov/clinvar/variation/1903/
NM_000060.2(BTD):c.641A>G	concordant	unreportable	unreportable	38579	0	1	SCV000617652	Uncertain significance	GeneDx	11/28/2017	BTD	chr3	15686004	15686004	http://gnomad.b	2.59E-05	NFE	0.0001074210013	0		0	0	https://www.ncbi.nlm.nih.gov/clinvar/variation/38579/
NM_000060.2(BTD):c.528G>T	concordant	reportable	reportable	25018	4	0	SCV000230011;S(	Pathogenic;Pathogenic;Pat	EGL Genetic Diagn	2017-06-30;2016-1	BTD	chr3	15685891	15685891	http://gnomad.b	3.75E-05	AMR	0.0007444460061	0		0	0	https://www.ncbi.nlm.nih.gov/clinvar/variation/25018/
NM_000060.2(BTD):c.1205A>G	concordant	unreportable	unreportable	25062	0	1	SCV000335421	Uncertain significance	EGL Genetic Diagn	6/30/2017	BTD	chr3	15686568	15686568	http://gnomad.b	0.0002191224722	NFE	0.0003403800074	0		0	0	https://www.ncbi.nlm.nih.gov/clinvar/variation/25062/
NM_000060.2(BTD):c.424C>A	concordant	reportable	reportable	25004	1	0	SCV000521148	Likely pathogenic	GeneDx	11/28/2017	BTD	chr3	15683529	15683529		2.88E-06					0	0	https://www.ncbi.nlm.nih.gov/clinvar/variation/25004/
NM_000060.2(BTD):c.631C>T	concordant	reportable	reportable	25028	1	0	SCV000600945	Pathogenic	Quest Diagnostics I	8/1/2017	BTD	chr3	15685994	15685994	http://gnomad.b	1.15E-05	AFR	6.53E-05	0		0	0	https://www.ncbi.nlm.nih.gov/clinvar/variation/25028/
NM_000060.2(BTD):c.470G>A	concordant	reportable	reportable	38290	2	0	SCV000230012;S(	Pathogenic;Pathogenic	EGL Genetic Diagn	2017-06-30;2017-1	BTD	chr3	15685833	15685833	http://gnomad.b	0.0001412763307	NFE	0.0002067190071	0		0	0	https://www.ncbi.nlm.nih.gov/clinvar/variation/38290/
NM_000060.2(BTD):c.133G>A	concordant	unreportable	unreportable	24973	0	4	SCV000227009;S(	Uncertain significance;Likel	EGL Genetic Diagn	2017-06-30;2017-1	BTD	chr3	15677019	15677019	http://gnomad.b	0.008808146743	FIN	0.02390350029	20		31	0	https://www.ncbi.nlm.nih.gov/clinvar/variation/24973/
NM_000060.2(BTD):c.632G>A	concordant	unreportable	unreportable	25029	0	1	SCV000109915	Uncertain significance	EGL Genetic Diagn	6/30/2017	BTD	chr3	15685995	15685995	http://gnomad.b	4.61E-05	EAS	0.0001739330037	0		0	0	https://www.ncbi.nlm.nih.gov/clinvar/variation/25029/
NM_000060.2(BTD):c.212T>C	concordant	unreportable	unreportable	38482	0	2	SCV000521147;S(	Likely benign;Uncertain sig	GeneDx;Genetic Se	2017-11-28;2017-0	BTD	chr3	15677098	15677098	http://gnomad.b	0.0006689001782	SAS	0.0124423001	12		2	0	https://www.ncbi.nlm.nih.gov/clinvar/variation/38482/
NM_000060.2(BTD):c.1489C>T	concordant	reportable	reportable	25094	2	0	SCV000490443;S(	Pathogenic;Pathogenic	GeneDx;Invitae	2016-12-21;2017-1	BTD	chr3	15686852	15686852	http://gnomad.b	2.88E-05	AFR	0.0003267120046	0		0	0	https://www.ncbi.nlm.nih.gov/clinvar/variation/25094/
NM_000060.2(BTD):c.557G>A	concordant	reportable	reportable	25020	1	0	SCV000630337	Pathogenic	Invitae	10/5/2017	BTD	chr3	15685920	15685920	http://gnomad.b	1.15E-05	SAS	0.0001624330034	1		0	0	https://www.ncbi.nlm.nih.gov/clinvar/variation/25020/
NM_000060.2(BTD):c.734G>A	concordant	reportable	reportable	38281	1	0	SCV000230003	Likely pathogenic	EGL Genetic Diagn	6/30/2017	BTD	chr3	15686097	15686097	http://gnomad.b	2.88E-06	NFE	1.79E-05	0		0	0	https://www.ncbi.nlm.nih.gov/clinvar/variation/38281/
NM_000071.2(CBS):c.1058C>T	concordant	reportable	reportable	131	1	0	SCV000249706	Pathogenic	GeneDx	11/28/2017	CBS	chr21	44480638	44480638	http://gnomad.b	2.25E-05	AFR	0.0004153400077	0		0	0	https://www.ncbi.nlm.nih.gov/clinvar/variation/131/
NM_000071.2(CBS):c.146C>T	concordant	reportable	reportable	212872	3	0	SCV000317366;S(	Pathogenic;Likely pathoger	Ambry Genetics;Ge	2017-11-14;2017-1	CBS	chr21	44492158	44492158	http://gnomad.b	0.0002109004606	NFE	0.0003239680082	0		0	0	https://www.ncbi.nlm.nih.gov/clinvar/variation/212872/
NM_000071.2(CBS):c.1265C>T	concordant	unreportable	unreportable	129	0	1	SCV000617613	Uncertain significance	GeneDx	11/28/2017	CBS	chr21	44479037	44479037		1.97E-05					0	0	https://www.ncbi.nlm.nih.gov/clinvar/variation/129/
NM_000071.2(CBS):c.52C>T	concordant	unreportable	unreportable	263858	0	4	SCV000319320;S(	Benign;Benign;Likely benig	Ambry Genetics;EG	2017-11-14;2017-0	CBS	chr21	44492252	44492252	http://gnomad.b	0.001085434371	EAS	0.0112327002	0		4	0	https://www.ncbi.nlm.nih.gov/clinvar/variation/263858/
NM_000071.2(CBS):c.1330G>A	concordant	reportable	reportable	126	2	0	SCV000249732;S(	Pathogenic;Pathogenic	GeneDx;Invitae	2017-11-28;2017-1	CBS	chr21	44478972	44478972	http://gnomad.b	0.0002165244729	AMR	0.001387130003	0		0	0	https://www.ncbi.nlm.nih.gov/clinvar/variation/126/
NM_000071.2(CBS):c.904G>A	concordant	unreportable	unreportable	496864	0	1	SCV000700449	Uncertain significance	EGL Genetic Diagn	6/30/2017	CBS	chr21	44483113	44483113	http://gnomad.b	5.62E-06	AFR	6.53E-05	0		0	0	https://www.ncbi.nlm.nih.gov/clinvar/variation/496864/
NM_000071.2(CBS):c.374G>A	concordant	reportable	reportable	197625	1	0	SCV000649834	Pathogenic	Invitae	10/5/2017	CBS	chr21	44486430	44486430	http://gnomad.b	1.69E-05	EAS	5.80E-05	0		0	0	https://www.ncbi.nlm.nih.gov/clinvar/variation/197625/
NM_000071.2(CBS):c.502G>A	concordant	unreportable	unreportable	127	0	1	SCV000543513	Uncertain significance	Invitae	3/14/2017	CBS	chr21	44485755	44485755		1.41E-05					0	0	https://www.ncbi.nlm.nih.gov/clinvar/variation/127/
NM_000071.2(CBS):c.304A>C	concordant	unreportable	unreportable	136677	0	3	SCV000436230;S(	Likely benign;Benign;Benig	Illumina Clinical Se	2016-10-18;2017-1	CBS	chr21	44488631	44488631	http://gnomad.b	0.003096018762	AFR	0.03954249993	15		18	0	https://www.ncbi.nlm.nih.gov/clinvar/variation/136677/
NM_000071.2(CBS):c.373C>T	concordant	reportable	reportable	340089	1	0	SCV000436227	Likely pathogenic	Illumina Clinical Se	10/18/2016	CBS	chr21	44486431	44486431		1.12E-05					0	0	https://www.ncbi.nlm.nih.gov/clinvar/variation/340089/
NM_000071.2(CBS):c.325T>C	concordant	reportable	reportable	212878	3	0	SCV000700264;S(	Pathogenic;Pathogenic;Pat	EGL Genetic Diagn	2017-06-30;2017-1	CBS	chr21	44486479	44486479	http://gnomad.b	1.12E-05	NFE	2.69E-05	0		0	0	https://www.ncbi.nlm.nih.gov/clinvar/variation/212878/
NM_000492.3(CFTR):c.859A>T	concordant	unreportable	unreportable	54069	0	1	SCV000697049	Uncertain significance	Laboratory Corpora	1/25/2018	CFTR	chr7	117176717	117176717	http://gnomad.b	2.12E-05	NFE	2.70E-05	0		0	0	https://www.ncbi.nlm.nih.gov/clinvar/variation/54069/
NM_000492.3(CFTR):c.861C>G	concordant	unreportable	unreportable	54071	0	1	SCV000700268	Uncertain significance	EGL Genetic Diagn	6/30/2017	CFTR	chr7	117176719	117176719	http://gnomad.b	0.0001636957493	EAS	0.001510750037	0		1	0	https://www.ncbi.nlm.nih.gov/clinvar/variation/54071/
NM_000492.3(CFTR):c.1253A>G	concordant	unreportable	unreportable	53224	0	1	SCV000603035	Uncertain significance	ARUP Laboratories	6/30/2017	CFTR	chr7	117188738	117188738		4.62E-05					0	0	https://www.ncbi.nlm.nih.gov/clinvar/variation/53224/
NM_000492.3(CFTR):c.3151A>G	concordant	unreportable	unreportable	495927	0	1	SCV000696951	Uncertain significance	Laboratory Corpora	1/25/2018	CFTR	chr7	117251646	117251646	http://gnomad.b	8.67E-05	ASJ	0.000203500007	0		0	0	https://www.ncbi.nlm.nih.gov/clinvar/variation/495927/
NM_000492.3(CFTR):c.535C>A	concordant	unreportable	unreportable	53993	0	1	SCV000697034	Uncertain significance	Laboratory Corpora	1/25/2018	CFTR	chr7	117174375	117174375	http://gnomad.b	2.31E-05	NFE	1.80E-05	0		0	0	https://www.ncbi.nlm.nih.gov/clinvar/variation/53993/
NM_000492.3(CFTR):c.292C>T	concordant	reportable	reportable	53600	1	0	SCV000071549	Pathogenic	CFTR2	6/29/2017	CFTR	chr7	117170971	117170971	http://gnomad.b	3.85E-06	AFR	0.0001307530038	0		0	0	https://www.ncbi.nlm.nih.gov/clinvar/variation/53600/
NM_000492.3(CFTR):c.658C>T	concordant	reportable	reportable	54036	1	0	SCV000071544	Pathogenic	CFTR2	6/29/2017	CFTR	chr7	117175380	117175380	http://gnomad.b	1.73E-05	NFE	8.96E-06	0		0	0	https://www.ncbi.nlm.nih.gov/clinvar/variation/54036/
NM_000492.3(CFTR):c.1163C>T	concordant	unreportable	unreportable	495887	0	1	SCV000696822	Uncertain significance	Laboratory Corpora	1/25/2018	CFTR	chr7	117182116	117182116	http://gnomad.b	0.0001752507434	SAS	0.0003576070012	0		0	0	https://www.ncbi.nlm.nih.gov/clinvar/variation/495887/
NM_000492.3(CFTR):c.2374C>G	concordant	unreportable	unreportable	35840	0	2	SCV000700817;S(	Uncertain significance;Unc	EGL Genetic Diagn	2017-06-30;2015-1	CFTR	chr7	117232595	117232595	http://gnomad.b	9.44E-05	AMR	0.0008656340069	0		0	0	https://www.ncbi.nlm.nih.gov/clinvar/variation/35840/
NM_000492.3(CFTR):c.349C>G	concordant	unreportable	unreportable	53762	0	1	SCV000601103	Uncertain significance	Quest Diagnostics I	8/1/2017	CFTR	chr7	117171028	117171028	http://gnomad.b	2.50E-05	NFE	3.59E-05	0		0	0	https://www.ncbi.nlm.nih.gov/clinvar/variation/53762/
NM_000492.3(CFTR):c.3208C>T	concordant	reportable	reportable	53685	2	0	SCV000271348;S(	Pathogenic;Likely pathoger	Laboratory for Mol	2016-04-26;2017-0	CFTR	chr7	117251703	117251703	http://gnomad.b	5.20E-05	AFR	0.0001307360071	0		0	0	https://www.ncbi.nlm.nih.gov/clinvar/variation/53685/
NM_000492.3(CFTR):c.3158C>T	concordant	unreportable	unreportable	53666	0	2	SCV000074813;S(	Uncertain significance;Unc	Invitae;Laboratory	2015-09-11;2018-0	CFTR	chr7	117251653	117251653	http://gnomad.b	3.27E-05	NFE	1.80E-05	0		0	0	https://www.ncbi.nlm.nih.gov/clinvar/variation/53666/
NM_000492.3(CFTR):c.1399C>T	concordant	unreportable	unreportable	53246	0	1	SCV000696841	Likely benign	Laboratory Corpora	1/25/2018	CFTR	chr7	117199524	117199524	http://gnomad.b	3.27E-05	NFE	8.96E-05	0		0	0	https://www.ncbi.nlm.nih.gov/clinvar/variation/53246/
NM_000492.3(CFTR):c.166G>A	concordant	reportable	reportable	53325	2	0	SCV000245908;S(	Pathogenic;Likely pathoger	CFTR2;Laboratory (	2017-06-29;2018-0	CFTR	chr7	117149089	117149089	http://gnomad.b	9.63E-06	NFE	2.70E-05	0		0	0	https://www.ncbi.nlm.nih.gov/clinvar/variation/53325/
NM_000492.3(CFTR):c.3310G>T	concordant	reportable	reportable	53715	1	0	SCV000071533	Pathogenic	CFTR2	6/29/2017	CFTR	chr7	117251805	117251805		3.85E-06					0	0	https://www.ncbi.nlm.nih.gov/clinvar/variation/53715/
NM_000492.3(CFTR):c.1680A>C	concordant	reportable	reportable	53339	1	0	SCV000677623	Pathogenic	CFTR2	6/29/2017	CFTR	chr7	117230407	117230407	http://gnomad.b	1.93E-06	SAS	3.25E-05	0		0	0	https://www.ncbi.nlm.nih.gov/clinvar/variation/53339/
NM_000492.3(CFTR):c.1766G>A	concordant	unreportable	unreportable	53382	0	1	SCV000601061	Uncertain significance	Quest Diagnostics I	8/1/2017	CFTR	chr7	117230493	117230493		3.85E-06					0	0	https://www.ncbi.nlm.nih.gov/clinvar/variation/53382/
NM_000492.3(CFTR):c.2017G>T	concordant	reportable	reportable	53427	1	0	SCV000245943	Pathogenic	CFTR2	6/29/2017	CFTR	chr7	117232238	117232238	http://gnomad.b	1.93E-06	NFE	9.05E-06	0		0	0	https://www.ncbi.nlm.nih.gov/clinvar/variation/53427/
NM_000492.3(CFTR):c.3705T>G	concordant	unreportable	unreportable	35872	0	5	SCV000602980;S(	Uncertain significance;Likel	ARUP Laboratories	2017-06-30;2014-0	CFTR	chr7	117267812	117267812	http://gnomad.b	0.005842975334	ASJ	0.008654040284	4		15	0	https://www.ncbi.nlm.nih.gov/clinvar/variation/35872/
NM_000492.3(CFTR):c.1007T>A	concordant	reportable	reportable	53162	2	0	SCV000071505;S(	Pathogenic;Pathogenic	CFTR2;EGL Genetic	2017-06-29;2017-0	CFTR	chr7	117180291	117180291	http://gnomad.b	7.70E-06	NFE	8.97E-06	0		0	0	https://www.ncbi.nlm.nih.gov/clinvar/variation/53162/
NM_000492.3(CFTR):c.374T>C	concordant	unreportable	unreportable	53802	0	3	SCV000706922;S(	Likely benign;Likely benign	EGL Genetic Diagn	2017-06-30;2017-1	CFTR	chr7	117171053	117171053	http://gnomad.b	0.0006663379913	EAS	0.009513859637	1		3	0	https://www.ncbi.nlm.nih.gov/clinvar/variation/53802/
NM_000492.3(CFTR):c.1019T>A	concordant	unreportable	unreportable	495884	0	1	SCV000696816	Uncertain significance	Laboratory Corpora	1/25/2018	CFTR	chr7	117180303	117180303		9.63E-06					0	0	https://www.ncbi.nlm.nih.gov/clinvar/variation/495884/
NM_000492.3(CFTR):c.1043T>A	concordant	unreportable	unreportable	53173	0	2	SCV000345968;S(	Uncertain significance;Beni	EGL Genetic Diagn	2017-06-30;2016-0	CFTR	chr7	117180327	117180327	http://gnomad.b	0.0002985040134	NFE	0.0002064	0		0	0	https://www.ncbi.nlm.nih.gov/clinvar/variation/53173/
NM_000492.3(CFTR):c.842T>C	concordant	unreportable	unreportable	54067	0	1	SCV000697047	Uncertain significance	Laboratory Corpora	1/25/2018	CFTR	chr7	117176700	117176700	http://gnomad.b	5.01E-05	AMR	2.99E-05	0		0	0	https://www.ncbi.nlm.nih.gov/clinvar/variation/54067/
NM_000492.3(CFTR):c.2855T>C	concordant	unreportable	unreportable	53579	0	3	SCV000702493;S(	Uncertain significance;Unc	EGL Genetic Diagn	2017-06-30;2017-1	CFTR	chr7	117243783	117243783	http://gnomad.b	0.0002002865639	NFE	0.0005553369992	1		0	0	https://www.ncbi.nlm.nih.gov/clinvar/variation/53579/
NM_000492.3(CFTR):c.794T>G	concordant	unreportable	unreportable	54058	0	1	SCV000603052	Uncertain significance	ARUP Laboratories	6/30/2017	CFTR	chr7	117176652	117176652	http://gnomad.b	2.12E-05	NFE	2.75E-05	0		0	0	https://www.ncbi.nlm.nih.gov/clinvar/variation/54058/
NM_000492.3(CFTR):c.443T>A	concordant	unreportable	unreportable	53949	0	1	SCV000706678	Uncertain significance	EGL Genetic Diagn	6/30/2017	CFTR	chr7	117171122	117171122		1.93E-06					0	0	https://www.ncbi.nlm.nih.gov/clinvar/variation/53949/
NM_000492.3(CFTR):c.3080T>C	concordant	unreportable	unreportable	35860	0	3	SCV000603024;S(	Benign;Likely benign;Likely	ARUP Laboratories	2017-06-30;2017-1	CFTR	chr7	117250664	117250664	http://gnomad.b	0.0003389464927	NFE	0.0005024040001	0		0	0	https://www.ncbi.nlm.nih.gov/clinvar/variation/35860/
NM_000492.3(CFTR):c.571T>G	concordant	unreportable	unreportable	495953	1	1	SCV000700410;S(	Likely pathogenic;Uncertai	EGL Genetic Diagn	2017-06-30;2018-0	CFTR	chr7	117174411	117174411	http://gnomad.b	3.47E-05	AFR	0.0001309759973	0		0	0	https://www.ncbi.nlm.nih.gov/clinvar/variation/495953/
NM_000492.3(CFTR):c.3872A>G	concordant	unreportable	unreportable	53827	0	1	SCV000601109	Uncertain significance	Quest Diagnostics I	8/1/2017	CFTR	chr7	117282646	117282646		3.85E-06					0	0	https://www.ncbi.nlm.nih.gov/clinvar/variation/53827/
NM_000492.3(CFTR):c.4056G>C	concordant	unreportable	unreportable	7237	1	4	SCV000603061;S(	Pathogenic;Uncertain signi	ARUP Laboratories	2017-06-30;2017-0	CFTR	chr7	117304834	117304834	http://gnomad.b	0.001247939359	EAS	0.01346180029	4		11	0	https://www.ncbi.nlm.nih.gov/clinvar/variation/7237/
NM_000492.3(CFTR):c.1826A>G	concordant	reportable	reportable	53398	1	0	SCV000696876	Pathogenic	Laboratory Corpora	1/25/2018	CFTR	chr7	117232047	117232047		1.16E-05					0	0	https://www.ncbi.nlm.nih.gov/clinvar/variation/53398/
NM_000492.3(CFTR):c.3409A>G	concordant	unreportable	unreportable	53733	0	1	SCV000696968	Uncertain significance	Laboratory Corpora	1/25/2018	CFTR	chr7	117254708	117254708	http://gnomad.b	6.16E-05	NFE	4.49E-05	0		0	0	https://www.ncbi.nlm.nih.gov/clinvar/variation/53733/
NM_000492.3(CFTR):c.115C>T	concordant	reportable	reportable	53204	1	0	SCV000071545	Pathogenic	CFTR2	6/29/2017	CFTR	chr7	117144368	117144368		9.63E-06					0	0	https://www.ncbi.nlm.nih.gov/clinvar/variation/53204/
NM_000492.3(CFTR):c.4144C>T	concordant	reportable	reportable	53902	2	0	SCV000245970;S(	Pathogenic;Pathogenic	CFTR2;Laboratory (	2017-06-29;2018-0	CFTR	chr7	117305520	117305520		1.93E-06					0	0	https://www.ncbi.nlm.nih.gov/clinvar/variation/53902/
NM_000492.3(CFTR):c.4115C>T	concordant	unreportable	unreportable	53893	0	1	SCV000601116	Uncertain significance	Quest Diagnostics I	8/1/2017	CFTR	chr7	117304893	117304893	http://gnomad.b	9.63E-06	AMR	5.96E-05	0		0	0	https://www.ncbi.nlm.nih.gov/clinvar/variation/53893/
NM_000492.3(CFTR):c.332C>T	concordant	unreportable	unreportable	53720	0	2	SCV000074881;S(	Uncertain significance;Unc	Invitae;Laboratory	2017-03-14;2018-0	CFTR	chr7	117171011	117171011	http://gnomad.b	9.05E-05	NFE	0.0001165939975	0		0	0	https://www.ncbi.nlm.nih.gov/clinvar/variation/53720/
NM_000492.3(CFTR):c.2353C>T	concordant	reportable	reportable	53480	2	0	SCV000245881;S(	Pathogenic;Pathogenic	CFTR2;Laboratory (	2017-06-29;2018-0	CFTR	chr7	117232574	117232574	http://gnomad.b	7.70E-06	AFR	6.92E-05	0		0	0	https://www.ncbi.nlm.nih.gov/clinvar/variation/53480/
NM_000492.3(CFTR):c.508C>T	concordant	unreportable	unreportable	455782	0	2	SCV000625754;S(	Uncertain significance;Unc	Invitae;Laboratory	2017-10-05;2018-0	CFTR	chr7	117174348	117174348	http://gnomad.b	7.90E-05	SAS	0.0002276129962	0		0	0	https://www.ncbi.nlm.nih.gov/clinvar/variation/455782/
NM_000492.3(CFTR):c.2002C>T	concordant	unreportable	unreportable	35835	0	8	SCV000603020;S(	Uncertain significance;Unc	ARUP Laboratories	2017-06-30;2017-0	CFTR	chr7	117232223	117232223	http://gnomad.b	0.006008596916	NFE	0.009488009848	6		11	0	https://www.ncbi.nlm.nih.gov/clinvar/variation/35835/
NM_000492.3(CFTR):c.2249C>T	concordant	unreportable	unreportable	53460	0	2	SCV000603030;S(	Uncertain significance;Unc	ARUP Laboratories	2017-06-30;2018-0	CFTR	chr7	117232470	117232470	http://gnomad.b	0.0004313864452	NFE	0.0005674039712	0		0	0	https://www.ncbi.nlm.nih.gov/clinvar/variation/53460/
NM_000492.3(CFTR):c.2173G>A	concordant	unreportable	unreportable	53448	0	2	SCV000704855;S(	Uncertain significance;Unc	EGL Genetic Diagn	2017-06-30;2018-0	CFTR	chr7	117232394	117232394	http://gnomad.b	9.05E-05	NFE	0.000179249997	0		0	0	https://www.ncbi.nlm.nih.gov/clinvar/variation/53448/

Supplemental Table 2

NM_000492.3(CFTR):c.2770G>A	concordant	unreportable	unreportable	53565	0	2	SCV000704123;S(	Uncertain significance;Unc	EGL Genetic Diagn	2017-06-30;2018-0	CFTR		chr7	117243698	117243698	http://gnomad.b	9.63E-05	NFE	0.0001074460015	0		0	0	https://www.ncbi.nlm.nih.gov/clinvar/variation/53565/
NM_000492.3(CFTR):c.3025G>A	concordant	unreportable	unreportable	53631	0	1	SCV000226877	Uncertain significance	EGL Genetic Diagn	6/30/2017	CFTR		chr7	117250609	117250609	http://gnomad.b	7.32E-05	AMR	0.0003284369886	0		0	0	https://www.ncbi.nlm.nih.gov/clinvar/variation/53631/
NM_000492.3(CFTR):c.221G>A	concordant	unreportable	unreportable	53456	0	4	SCV000602983;S(	Uncertain significance;Unc	ARUP Laboratories	2017-06-30;2017-0	CFTR		chr7	117149144	117149144	http://gnomad.b	0.0003042815105	NFE	0.0005297369789	0		0	0	https://www.ncbi.nlm.nih.gov/clinvar/variation/53456/
NM_000492.3(CFTR):c.1001G>A	concordant	unreportable	unreportable	53159	1	2	SCV000603057;S(	Likely pathogenic;Uncertai	ARUP Laboratories	2017-06-30;2017-1	CFTR		chr7	117180285	117180285	http://gnomad.b	0.0001887315698	ASJ	0.0008125129971	0		1	0	https://www.ncbi.nlm.nih.gov/clinvar/variation/53159/
NM_000492.3(CFTR):c.3209G>A	concordant	reportable	reportable	35866	2	0	SCV000484489;S(	Pathogenic;Pathogenic	EGL Genetic Diagn	2017-06-30;2015-1	CFTR		chr7	117251704	117251704	http://gnomad.b	0.0003370206603	SAS	0.004582980182	3		2	0	https://www.ncbi.nlm.nih.gov/clinvar/variation/35866/
NM_000492.3(CFTR):c.509G>A	concordant	unreportable	unreportable	53983	1	4	SCV000230936;S(	Uncertain significance;Unc	EGL Genetic Diagn	2017-06-30;2017-1	CFTR		chr7	117174349	117174349	http://gnomad.b	0.0009860261605	ASJ	0.006914789788	0		1	0	https://www.ncbi.nlm.nih.gov/clinvar/variation/53983/
NM_000492.3(CFTR):c.3197G>A	concordant	reportable	reportable	7158	3	0	SCV000603049;S(	Pathogenic;Pathogenic;Pat	ARUP Laboratories	2017-06-30;2017-0	CFTR		chr7	117251692	117251692	http://gnomad.b	2.12E-05	AFR	6.54E-05	0		0	0	https://www.ncbi.nlm.nih.gov/clinvar/variation/7158/
NM_000492.3(CFTR):c.1558G>A	concordant	unreportable	unreportable	35825	1	3	SCV000603040;S(	Pathogenic;Uncertain signi	ARUP Laboratories	2017-06-30;2017-0	CFTR		chr7	117199683	117199683	http://gnomad.b	0.0006335988414	EAS	0.0008116880199	0		0	0	https://www.ncbi.nlm.nih.gov/clinvar/variation/35825/
NM_000492.3(CFTR):c.601G>A	concordant	unreportable	unreportable	54022	1	3	SCV000602985;S(	Pathogenic;Uncertain signi	ARUP Laboratories	2017-06-30;2017-0	CFTR		chr7	117175323	117175323	http://gnomad.b	0.0004833839185	AMR	0.0006849719794	1		1	0	https://www.ncbi.nlm.nih.gov/clinvar/variation/54022/
NM_000492.3(CFTR):c.2260G>A	concordant	unreportable	unreportable	53465	0	5	SCV000603060;S(	Uncertain significance;Unc	ARUP Laboratories	2017-06-30;2017-0	CFTR		chr7	117232481	117232481	http://gnomad.b	0.001506000894	FIN	0.002671380062	1		2	0	https://www.ncbi.nlm.nih.gov/clinvar/variation/53465/
NM_000492.3(CFTR):c.2758G>T	concordant	unreportable	unreportable	53562	0	1	SCV000700347	Uncertain significance	EGL Genetic Diagn	6/30/2017	CFTR		chr7	117243686	117243686	http://gnomad.b	3.85E-05	NFE	5.37E-05	0		0	0	https://www.ncbi.nlm.nih.gov/clinvar/variation/53562/
NM_000492.3(CFTR):c.250T>C	concordant	unreportable	unreportable	498176	0	1	SCV000703058	Uncertain significance	EGL Genetic Diagn	6/30/2017	CFTR		chr7	117149173	117149173	http://gnomad.b	2.70E-05	NFE	2.69E-05	0		0	0	https://www.ncbi.nlm.nih.gov/clinvar/variation/498176/
NM_000492.3(CFTR):c.3230T>C	concordant	reportable	reportable	53690	2	0	SCV000071507;S(	Pathogenic;Pathogenic	CFTR2;Laboratory (	2017-06-29;2018-0	CFTR		chr7	117251725	117251725	http://gnomad.b	1.35E-05	NFE	8.97E-06	0		0	0	https://www.ncbi.nlm.nih.gov/clinvar/variation/53690/
NM_000492.3(CFTR):c.1400T>C	concordant	reportable	reportable	35823	4	0	SCV000071509;S(	Pathogenic;Pathogenic;Pat	CFTR2;EGL Genetic	2017-06-29;2017-0	CFTR		chr7	117199525	117199525	http://gnomad.b	1.54E-05	AFR	0.0001308039937	0		0	0	https://www.ncbi.nlm.nih.gov/clinvar/variation/35823/
NM_000492.3(CFTR):c.837A>T	concordant	unreportable	unreportable	501133	0	1	SCV000707380	Uncertain significance	EGL Genetic Diagn	6/30/2017	CFTR		chr7	117176695	117176695	http://gnomad.b	2.89E-05	FIN	0.0002794080065	0		0	0	https://www.ncbi.nlm.nih.gov/clinvar/variation/501133/
NM_000492.3(CFTR):c.1841A>G	concordant	reportable	reportable	53403	1	0	SCV000696877	Pathogenic	Laboratory Corpor	1/25/2018	CFTR		chr7	117232062	117232062	http://gnomad.b	1.93E-05	AMR	0.0001064960015	0		0	0	https://www.ncbi.nlm.nih.gov/clinvar/variation/53403/
NM_000492.3(CFTR):c.3503A>G	concordant	unreportable	unreportable	53763	0	1	SCV000708957	Uncertain significance	EGL Genetic Diagn	6/30/2017	CFTR		chr7	117267610	117267610	http://gnomad.b	1.16E-05	AFR	6.54E-05	0		0	0	https://www.ncbi.nlm.nih.gov/clinvar/variation/53763/
NM_000492.3(CFTR):c.650A>G	concordant	unreportable	unreportable	7238	0	5	SCV000700761;S(	Uncertain significance;Likel	EGL Genetic Diagn	2017-06-30;2016-1	CFTR		chr7	117175372	117175372	http://gnomad.b	0.001242161862	FIN	0.0358777009	30		3	0	https://www.ncbi.nlm.nih.gov/clinvar/variation/7238/
NM_000492.3(CFTR):c.454A>T	concordant	unreportable	unreportable	439479	0	1	SCV000603013	Uncertain significance	ARUP Laboratories	6/30/2017	CFTR		chr7	117171133	117171133	http://gnomad.b	3.85E-05	AFR	6.55E-05	0		0	0	https://www.ncbi.nlm.nih.gov/clinvar/variation/439479/
NM_000492.3(CFTR):c.853A>T	concordant	unreportable	unreportable	35892	0	2	SCV000700536;S(	Likely benign;Likely benign	EGL Genetic Diagn	2017-06-30;2018-0	CFTR		chr7	117176711	117176711	http://gnomad.b	0.0003832406366	AFR	0.00450032996	0		1	0	https://www.ncbi.nlm.nih.gov/clinvar/variation/35892/
NM_000492.3(CFTR):c.1450C>T	concordant	unreportable	unreportable	53257	0	2	SCV000707884;S(	Uncertain significance;Unc	EGL Genetic Diagn	2017-06-30;2017-0	CFTR		chr7	117199575	117199575	http://gnomad.b	1.54E-05	AMR	8.93E-05	0		0	0	https://www.ncbi.nlm.nih.gov/clinvar/variation/53257/
NM_000492.3(CFTR):c.926C>G	concordant	unreportable	unreportable	54086	0	1	SCV000697056	Uncertain significance	Laboratory Corpor	1/25/2018	CFTR		chr7	117180210	117180210	http://gnomad.b	2.89E-05	SAS	0.0001949440048	0		0	0	https://www.ncbi.nlm.nih.gov/clinvar/variation/54086/
NM_000492.3(CFTR):c.1196C>T	concordant	unreportable	unreportable	495889	0	1	SCV000696824	Uncertain significance	Laboratory Corpor	1/25/2018	CFTR		chr7	117182149	117182149	http://gnomad.b	0.0001001432819	EAS	0.0006386439782	0		0	0	https://www.ncbi.nlm.nih.gov/clinvar/variation/495889/
NM_000492.3(CFTR):c.3200C>T	concordant	unreportable	unreportable	53683	0	2	SCV000339600;S(	Uncertain significance;Unc	EGL Genetic Diagn	2017-06-30;2018-0	CFTR		chr7	117251695	117251695	http://gnomad.b	3.66E-05	NFE	2.69E-05	0		0	0	https://www.ncbi.nlm.nih.gov/clinvar/variation/53683/
NM_000492.3(CFTR):c.3854C>T	concordant	unreportable	unreportable	53823	0	1	SCV000696994	Uncertain significance	Laboratory Corpor	1/25/2018	CFTR		chr7	117282628	117282628	http://gnomad.b	0.0003678339778	SAS	0.005265899934	1		0	0	https://www.ncbi.nlm.nih.gov/clinvar/variation/53823/
NM_000492.3(CFTR):c.3731G>A	concordant	reportable	reportable	53797	2	0	SCV000071499;S(	Pathogenic;Pathogenic	CFTR2;Laboratory (	2017-06-29;2018-0	CFTR		chr7	117282505	117282505	http://gnomad.b	1.93E-05	NFE	1.80E-05	0		0	0	https://www.ncbi.nlm.nih.gov/clinvar/variation/53797/
NM_000492.3(CFTR):c.2491G>T	concordant	reportable	reportable	53501	1	0	SCV000071537	Pathogenic	CFTR2	6/29/2017	CFTR		chr7	117234984	117234984	http://gnomad.b	1.93E-05	NFE	5.38E-05	0		0	0	https://www.ncbi.nlm.nih.gov/clinvar/variation/53501/
NM_000492.3(CFTR):c.4129G>C	concordant	unreportable	unreportable	53896	0	1	SCV000342707	Uncertain significance	EGL Genetic Diagn	6/30/2017	CFTR		chr7	117304907	117304907	http://gnomad.b	1.16E-05	NFE	8.97E-06	0		0	0	https://www.ncbi.nlm.nih.gov/clinvar/variation/53896/
NM_000492.3(CFTR):c.592G>C	concordant	unreportable	unreportable	54017	0	1	SCV000340479	Uncertain significance	EGL Genetic Diagn	6/30/2017	CFTR		chr7	117175314	117175314	http://gnomad.b	5.78E-06	EAS	5.80E-05	0		0	0	https://www.ncbi.nlm.nih.gov/clinvar/variation/54017/
NM_000492.3(CFTR):c.638G>A	concordant	unreportable	unreportable	411118	0	1	SCV000552131	Uncertain significance	Invitae	3/14/2017	CFTR		chr7	117175360	117175360	http://gnomad.b	2.12E-05	NFE	5.37E-05	0		0	0	https://www.ncbi.nlm.nih.gov/clinvar/variation/411118/
NM_000492.3(CFTR):c.2909G>A	concordant	reportable	reportable	35854	1	0	SCV000052160	Likely pathogenic	Laboratory Corpor	11/20/2015	CFTR		chr7	117246728	117246728	http://gnomad.b	3.85E-05	EAS	0.0001739330037	0		0	0	https://www.ncbi.nlm.nih.gov/clinvar/variation/35854/
NM_000492.3(CFTR):c.3274T>C	concordant	unreportable	unreportable	495930	0	1	SCV000696961	Uncertain significance	Laboratory Corpor	1/25/2018	CFTR		chr7	117251769	117251769	http://gnomad.b	7.51E-05	NFE	6.27E-05	0		0	0	https://www.ncbi.nlm.nih.gov/clinvar/variation/495930/
NM_000492.3(CFTR):c.950T>C	concordant	unreportable	unreportable	495964	0	1	SCV000697061	Uncertain significance	Laboratory Corpor	1/25/2018	CFTR		chr7	117180234	117180234		5.78E-06					0	0	https://www.ncbi.nlm.nih.gov/clinvar/variation/495964/
NM_000492.3(CFTR):c.2813T>G	concordant	unreportable	unreportable	35850	1	1	SCV000052156;S(	Pathogenic;Uncertain signi	Laboratory Corpor	2015-11-20;2017-0	CFTR		chr7	117243741	117243741	http://gnomad.b	4.24E-05	NFE	2.69E-05	0		0	0	https://www.ncbi.nlm.nih.gov/clinvar/variation/35850/
NM_000492.3(CFTR):c.202A>G	concordant	unreportable	unreportable	53428	0	2	SCV000703035;S(	Uncertain significance;Unc	EGL Genetic Diagn	2017-06-30;2018-0	CFTR		chr7	117149125	117149125	http://gnomad.b	0.0001463632582	SAS	0.0007796760183	1		1	0	https://www.ncbi.nlm.nih.gov/clinvar/variation/53428/
NM_000492.3(CFTR):c.3041A>G	concordant	unreportable	unreportable	53638	0	2	SCV000696943;S(	Uncertain significance;Unc	Laboratory Corpor	2018-01-25;2017-0	CFTR		chr7	117250625	117250625	http://gnomad.b	0.0004756805892	ASJ	0.003758630017	0		0	0	https://www.ncbi.nlm.nih.gov/clinvar/variation/53638/
NM_000492.3(CFTR):c.3275A>G	concordant	unreportable	unreportable	500634	0	1	SCV000706667	Uncertain significance	EGL Genetic Diagn	6/30/2017	CFTR		chr7	117251770	117251770	http://gnomad.b	5.78E-06	SAS	3.25E-05	0		0	0	https://www.ncbi.nlm.nih.gov/clinvar/variation/500634/
NM_000492.3(CFTR):c.3169A>G	concordant	unreportable	unreportable	53668	0	1	SCV000334857	Uncertain significance	EGL Genetic Diagn	6/30/2017	CFTR		chr7	117251664	117251664	http://gnomad.b	3.47E-05	AMR	0.0001197320016	0		0	0	https://www.ncbi.nlm.nih.gov/clinvar/variation/53668/
NM_000492.3(CFTR):c.825C>G	concordant	reportable	reportable	35890	2	0	SCV000677629;S(	Pathogenic;Pathogenic	CFTR2;Laboratory (	2017-06-29;2015-1	CFTR		chr7	117176683	117176683		5.78E-06					0	0	https://www.ncbi.nlm.nih.gov/clinvar/variation/35890/
NM_000492.3(CFTR):c.365A>G	concordant	unreportable	unreportable	411120	0	2	SCV000552134;S(	Uncertain significance;Unc	Invitae;Laboratory	2017-03-14;2018-0	CFTR		chr7	117171044	117171044	http://gnomad.b	1.73E-05	NFE	8.98E-06	0		0	0	https://www.ncbi.nlm.nih.gov/clinvar/variation/411120/
NM_000492.3(CFTR):c.355A>G	concordant	unreportable	unreportable	35870	0	1	SCV000589584	Uncertain significance	GeneDx	11/28/2017	CFTR		chr7	117171034	117171034	http://gnomad.b	2.89E-05	NFE	6.28E-05	0		0	0	https://www.ncbi.nlm.nih.gov/clinvar/variation/35870/
NM_000492.3(CFTR):c.1516A>G	concordant	unreportable	unreportable	7131	0	4	SCV000603019;S(	Uncertain significance;Beni	ARUP Laboratories	2017-06-30;2017-0	CFTR		chr7	117199641	117199641	http://gnomad.b	0.0006124146856	AMR	0.0006552300183	0		0	0	https://www.ncbi.nlm.nih.gov/clinvar/variation/7131/
NM_000492.3(CFTR):c.3415A>G	concordant	unreportable	unreportable	53736	0	2	SCV000603071;S(	Uncertain significance;Unc	ARUP Laboratories	2017-06-30;2017-0	CFTR		chr7	117254714	117254714	http://gnomad.b	0.000117475773	NFE	0.0002243119961	0		0	0	https://www.ncbi.nlm.nih.gov/clinvar/variation/53736/
NM_000492.3(CFTR):c.2739T>A	concordant	reportable	reportable	53552	1	0	SCV000245922	Pathogenic	CFTR2	6/29/2017	CFTR		chr7	117243667	117243667	http://gnomad.b	1.16E-05	AFR	0.0001307189959	0		0	0	https://www.ncbi.nlm.nih.gov/clinvar/variation/53552/
NM_000492.3(CFTR):c.4364C>G	concordant	reportable	reportable	7223	1	0	SCV000697025	Pathogenic	Laboratory Corpor	1/25/2018	CFTR		chr7	117307083	117307083	http://gnomad.b	2.31E-05	SAS	3.25E-05	0		0	0	https://www.ncbi.nlm.nih.gov/clinvar/variation/7223/
NM_000492.3(CFTR):c.3937C>T	concordant	reportable	reportable	7180	1	0	SCV000071543	Pathogenic	CFTR2	6/29/2017	CFTR		chr7	117292959	117292959		5.78E-06					0	0	https://www.ncbi.nlm.nih.gov/clinvar/variation/7180/
NM_000492.3(CFTR):c.4123C>A	concordant	unreportable	unreportable	53894	0	1	SCV000075092	Uncertain significance	Invitae	10/5/2017	CFTR		chr7	117304901	117304901	http://gnomad.b	9.44E-05	NFE	0.000143562007	0		0	0	https://www.ncbi.nlm.nih.gov/clinvar/variation/53894/
NM_000492.3(CFTR):c.4277C>T	concordant	unreportable	unreportable	455780	0	1	SCV000625751	Uncertain significance	Invitae	10/5/2017	CFTR		chr7	117306996	117306996	http://gnomad.b	3.66E-05	EAS	0.0002907309972	0		0	0	https://www.ncbi.nlm.nih.gov/clinvar/variation/455780/
NM_000492.3(CFTR):c.613C>T	concordant	reportable	reportable	54026	3	0	SCV000071514;S(	Pathogenic;Pathogenic;Pat	CFTR2;EGL Genetic	2017-06-29;2017-0	CFTR		chr7	117175335	117175335	http://gnomad.b	1.35E-05	AMR	2.98E-05	0		0	0	https://www.ncbi.nlm.nih.gov/clinvar/variation/54026/
NM_000492.3(CFTR):c.1315C>T	concordant	unreportable	unreportable	53226	0	1	SCV000074284	Uncertain significance	Invitae	6/10/2016	CFTR		chr7	117188800	117188800		9.63E-06					0	0	https://www.ncbi.nlm.nih.gov/clinvar/variation/53226/
NM_000492.3(CFTR):c.2551C>T	concordant	reportable	reportable	7141	1	0	SCV000071556	Pathogenic	CFTR2	6/29/2017	CFTR		chr7	117235044	117235044		1.16E-05					0	0	https://www.ncbi.nlm.nih.gov/clinvar/variation/7141/
NM_000492.3(CFTR):c.1054C>T	concordant	unreportable	unreportable	35816	1	4	SCV000603070;S(	Pathogenic;Uncertain signi	ARUP Laboratories	2017-06-30;2017-0	CFTR		chr7	117180338	117180338	http://gnomad.b	9.24E-05	AMR	0.003010609886	0		0	0	https://www.ncbi.nlm.nih.gov/clinvar/variation/35816/
NM_000492.3(CFTR):c.125C>T	concordant	unreportable	unreportable	35819	0	2	SCV000338741;S(	Uncertain significance;Unc	EGL Genetic Diagn	2017-06-30;2018-0	CFTR		chr7	117144378	117144378	http://gnomad.b	0.000167547414	NFE	0.0002607869974	1		0	0	https://www.ncbi.nlm.nih.gov/clinvar/variation/35819/
NM_000492.3(CFTR):c.577G>T	concordant	reportable	reportable	54007	2	0	SCV000602969;S(	Pathogenic;Pathogenic	ARUP Laboratories	2017-06-30;2017-0	CFTR		chr7	117174417	117174417		7.70E-06					0	0	https://www.ncbi.nlm.nih.gov/clinvar/variation/54007/
NM_000492.3(CFTR):c.4111G>T	concordant	reportable	reportable	53891	1	0	SCV000245913	Pathogenic	CFTR2	6/29/2017	CFTR		chr7	117304889	117304889	http://gnomad.b	1.93E-06	NFE	8.97E-06	0		0	0	https://www.ncbi.nlm.nih.gov/clinvar/variation/53891/
NM_000492.3(CFTR):c.2506G>T	concordant	unreportable	unreportable	53505	0	3	SCV000343987;S(	Uncertain significance;Unc	EGL Genetic Diagn	2017-06-30;2017-1	CFTR		chr7	117234999	117234999	http://gnomad.b	0.0003755373072	AMR	0.001697940053	0		0	0	https://www.ncbi.nlm.nih.gov/clinvar/variation/53505/
NM_000492.3(CFTR):c.2597G>A	concordant	unreportable	unreportable	35843	0	1	SCV000696909	Uncertain significance	Laboratory Corpor	1/25/2018	CFTR		chr7	117235090	117235090	http://gnomad.b	7.70E-05	EAS	0.000173952998	0		0	0	https://www.ncbi.nlm.nih.gov/clinvar/variation/35843/
NM_000492.3(CFTR):c.828C>A	concordant	reportable	reportable	54064	1	0	SCV000245895	Pathogenic	CFTR2	6/29/2017	CFTR		chr7	117176686	117176686		3.85E-06					0	0	https://www.ncbi.nlm.nih.gov/clinvar/variation/54064/
NM_000492.3(CFTR):c.2537G>A	concordant	reportable	reportable	53508	1	0	SCV000071564	Pathogenic	CFTR2	6/29/2017	CFTR		chr7	117235030	117235030		7.70E-06					0	0	https://www.ncbi.nlm.nih.gov/clinvar/variation/53508/
NM_000492.3(CFTR):c.2538G>A	concordant	reportable	reportable	7127	1	0	SCV000087509	Pathogenic	CFTR2	6/29/2017	CFTR		chr7	117235031	117235031	http://gnomad.b	5.78E-06	NFE	8.96E-06	0		0	0	https://www.ncbi.nlm.nih.gov/clinvar/variation/7127/
NM_000492.3(CFTR):c.715G>A	concordant	unreportable	unreportable	54046	0	1	SCV000704648	Uncertain significance	EGL Genetic Diagn	6/30/2017	CFTR		chr7	117175437	117175437	http://gnomad.b	8.86E-05	AMR	0.000178731003	0		0	0	https://www.ncbi.nlm.nih.gov/clinvar/variation/54046/
NM_000492.3(CFTR):c.171G>A	concordant	reportable	reportable	7179	1	0	SCV000245965	Pathogenic	CFTR2	6/29/2017	CFTR		chr7	117149094	117149094		3.85E-06					0	0	https://www.ncbi.nlm.nih.gov/clinvar/variation/7179/
NM_000492.3(CFTR):c.1270G>A	concordant	unreportable	unreportable	286406	0	3	SCV000339832;S(	Uncertain significance;Unc	EGL Genetic Diagn	2017-06-30;2017-1	CFTR		chr7	117188755	117188755		0.0001155499407					0	0	https://www.ncbi.nlm.nih.gov/clinvar/variation/286406/
NM_000492.3(CFTR):c.2563G>A	concordant	unreportable	unreportable	53513	0	1	SCV000696908	Uncertain significance	Laboratory Corpor	1/25/2018	CFTR		chr7	117235056	117235056	http://gnomad.b	1.93E-06	SAS	0.0005847570137	0		0	0	https://www.ncbi.nlm.nih.gov/clinvar/variation/53513/
NM_000492.3(CFTR):c.2195T>G	concordant	reportable	reportable	53451	2	0	SCV000071542;S(	Pathogenic;Pathogenic	CFTR2;Laboratory (	2017-06-29;2018-0	CFTR		chr7	117232416	117232416	http://gnomad.b	3.85E-06	NFE	8.96E-06	0		0	0	https://www.ncbi.nlm.nih.gov/clinvar/variation/53451/
NM_000492.3(CFTR):c.2981T>G	concordant	unreportable	unreportable	53612	0	1	SCV000696940	Uncertain significance	Laboratory Corpor	1/25/2018	CFTR		chr7	117246800	117246800	http://gnomad.b	2.50E-05	NFE	8.97E-06	0		0	0	https://www.ncbi.nlm.nih.gov/clinvar/variation/53612/
NM_000492.3(CFTR):c.3297C>A	concordant	unreportable	unreportable	286681	0	2	SCV000340215;S(	Uncertain significance;Unc	EGL Genetic Diagn	2017-06-30;2018-0	CFTR		chr7	117251792	117251792	http://gnomad.b	2.89E-05	AFR	0.0001961039961	0		0	0	https://www.ncbi.nlm.nih.gov/clinvar/variation/286681/
NM_000492.3(CFTR):c.2900T>C	concordant	unreportable	unreportable	219537	1	4	SCV000603067;S(	Pathogenic;Uncertain signi	ARUP Laboratories	2017-06-30;2017-0	CFTR		chr7	117243828	117243828	http://gnomad.b	0.0009783228311	AMR	0.001341999974	0		0	0	https://www.ncbi.nlm.nih.gov/clinvar/variation/219537/
NM_000492.3(CFTR):c.1523T>G	concordant	unreportable	unreportable	7126	1	4	SCV000608288;S(	Pathogenic;Benign;Benign;	ARUP Laboratories	2017-10-26-04:00;2	CFTR		chr7	117199648	117199648	http://gnomad.b	0.001072688616	NFE	0.001762990025	0		0	0	https://www.ncbi.nlm.nih.gov/clinvar/variation/7126/
NM_000492.3(CFTR):c.3154T>G	concordant	unreportable	unreportable	35865	2	2	SCV000700272;S(	Uncertain significance;Unc	EGL Genetic Diagn	2017-06-30;2017-1	CFTR		chr7	117251649	117251649	http://gnomad.b	0.0008916603756	ASJ	0.002034999896	1		0	0	https://www.ncbi.nlm.nih.gov/clinvar/variation/35865/
NM_000492.3(CFTR):c.3222T>A	concordant	reportable	reportable	53688	1	0	SCV000617533	Pathogenic	GeneDx	11/28/2017	CFTR		chr7	117251717	117251717	http://gnomad.b	1.73E-05	NFE	8.97E-06	0		0	0	https://www.ncbi.nlm.nih.gov/clinvar/variation/53688/
NM_000492.3(CFTR):c.2079T>G	concordant	unreportable	unreportable	194336	0	1	SCV000226032	Uncertain significance	EGL Genetic Diagn	6/30/2017	CFTR		chr7	117232300	117232300	http://gnomad.b	0.000117475773	AFR	0.0009273979813	1		1	0	https://www.ncbi.nlm.nih.gov/clinvar/variation/194336/
NM_000492.3(CFTR):c.2502T>G	concordant	unreportable	unreportable	455769	0	2	SCV000625737;S(	Uncertain significance;Unc	Invitae;Laboratory	2017-10-05;2018-0	CFTR		chr7	117234995	117234995	http://gnomad.b	0.0001136241083	FIN	0.001166549977	0		0	0	https://www.ncbi.nlm.nih.gov/clinvar/variation/455769/
NM_001042432.1(CLN3):c.1000C>T	concordant	reportable	reportable	56243	1	0	SCV000697651	Likely pathogenic	Laboratory Corpor	1/25/2018	CLN3		chr16	28493482	28493482		5.56E-06					0	0	https://www.ncbi.nlm.nih.gov/clinvar/variation/56243/
NM_001042432.1(CLN3):c.1054C>T	concordant	reportable	reportable	56246	1	0	SCV000680509	Pathogenic	GeneDx	2/7/2018	CLN3		chr16	28493428	28493428	http://gnomad.b	2.78E-06	NFE	1.83E-05	0		0	0	https://www.ncbi.nlm.nih.gov/clinvar/variation/56246/
NM_006493.2(CLN5):c.61C>T	concordant	unreportable	unreportable	193342	0	4	SCV000612844;S(	Likely benign;Likely benign	Athena Diagnostics	2017-08-17;2017-0	CLN5		chr13	77566147	77566147	http://gnomad.b	0.003082741682	NFE	0.005911659915	3		3	0	https://www.ncbi.nlm.nih.gov/clinvar/variation/193342/
NM_006493.2(CLN5):c.223T>C	concordant	unreportable	unreportable	205127	0	2	SCV000700766;S(	Likely benign;Likely benign	EGL Genetic Diagn	2017-06-30;2017-1	CLN5		chr13	77566309	77566309	http://gnomad.b	0.003572287775	ASJ	0.01965020038	3		9	0	https://www.ncbi.nlm.nih.gov/clinvar/variation/205127/
NM_006493.2(CLN5):c.671G>A	concordant	reportable	reportable	56544	1	0	SCV000240646	Likely pathogenic	GeneDx	11/28/2017	CLN5		chr13	77570221	77570221	http://gnomad.b	3.64E-05	NFE	4.48E-05	0		0	0	https://www.ncbi.nlm.nih.gov/clinvar/variation/56544/
NM_018941.3(CLN8):c.374A>G	concordant	unreportable	unreportable	205207	0	4	SCV000342696;S(	Uncertain significance;Unc	EGL Genetic Diagn	2017-06-30;2017-1	CLN8		chr8	1719594	1719594	http://gnomad.b	0.001015782404	SAS	0.002209079918	0		1	0	https://www.ncbi.nlm.nih.gov/clinvar/variation/205207/
NM_018941.3(CLN8):c.46C>A	concordant	unreportable	unreportable	56709	0	1	SCV000334637	Uncertain significance	EGL Genetic Diagn	6/30/2017	CLN8		chr8	1719266	1719266	http://gnomad.b	1.68E-05	ASJ	0.0001015230009	0		0	0	https://www.ncbi.nlm.nih.gov/clinvar/variation/56709/
NM_018941.3(CLN8):c.709G>A	concordant	reportable	reportable	188917	1	0	SCV000240702	Likely pathogenic	GeneDx	11/28/2017	CLN8		chr8	1728581	1728581	http://gnomad.b	8.39E-06	AFR	6.53E-05	0		0	0	https://www.ncbi.nlm.nih.gov/clinvar/variation/188917/
NM_018941.3(CLN8):c.661G>A	concordant	unreportable	unreportable	56717	0	1	SCV000707192	Uncertain significance	EGL Genetic Diagn	6/30/2017	CLN8		chr8	1728533	1728533	http://gnomad.b	3.64E-05	AFR	0.0001306850027	0		1	0	https://www.ncbi.nlm.nih.gov/clinvar/variation/56717/
NM_018941.3(CLN8):c.806A>T	concordant	unreportable	unreportable	196493	0	3	SCV000611460;S(	Uncertain significance;Likel	Fulgent Genetics,F	2017-05-23;2017-1	CLN8		chr8	1728678	1728678	http://gnomad.b	0.0005372733378	SAS	0.0005848329747	2		2	0	https://www.ncbi.nlm.nih.gov/clinvar/variation/196493/
NM_174878.2(CLRN1):c.118T>G	concordant	unreportable	unreportable	4399	0	1	SCV000337866	Uncertain significance	EGL Genetic Diagn	6/30/2017	CLRN1		chr3	150690378	150690378	http://gnomad.b	4.63E-05	NFE	0.0001611659973	0		0	0	https://www.ncbi.nlm.nih.gov/clinvar/variation/4399/
NM_001875.4(CPS1):c.3643A>G	concordant	unreportable	unreportable	235659	0	1	SCV000577569	Uncertain significance	GeneDx	11/28/2017	CPS1		chr2	211521333	211521333	http://gnomad.b	0.002584951262	ASJ	0.01563449949	1		2	0	https://www.ncbi.nlm.nih.gov/clinvar/variation/235659/
NM_001875.4(CPS1):c.2407C>T	concordant	unreportable	unreportable	477851	0	1	SCV000659343	Uncertain significance	Invitae	10/5/2017	CPS1		chr2	211476856	211476856	http://gnomad.b	8.00E-06	AFR	6.54E-05	0		0	0	https://www.ncbi.nlm.nih.gov/clinvar/variation/477851/
NM_001876.3(CPT1A):c.946C>G	concordant	unreportable	unreportable	65659	0	1	SCV000332900	Uncertain significance	EGL Genetic Diagn	6/30/2017	CPT1A		chr11	68560804	68560804		8.45E-06					0	0	https://www.ncbi.nlm.nih.gov/clinvar/variation/65659/
NM_000098.2(CPT2):c.149C>A	concordant	reportable	reportable	8954	4	0	SCV000700664;S(	Pathogenic;Pathogenic;Pat	EGL Genetic Diagn	2017-06-30;2017-1	CPT2		chr1	53662764	53662764	http://gnomad.b	0.0001259142776	FIN	0.001589599997	0		0	0	https://www.ncbi.nlm.nih.gov/clinvar/variation/8954/
NM_000098.2(CPT2):c.886C>T	concordant	reportable	reportable	166953	1	0	SCV000632615	Pathogenic	Invitae	10/5/2017	CPT2		chr1	53676232	53676232	http://gnomad.b	1.68E-05	AFR	6.54E-05	0		0	0	https://www.ncbi.nlm.nih.gov/clinvar/variation/166953/
NM_000098.2(CPT2):c.452G>A	concordant	reportable	reportable	130889	1	0	SCV000583282	Likely pathogenic	GeneDx	11/28/2017	CPT2		chr1	53675798	53675798	http://gnomad.b	5.60E-06	EAS	5.80E-05	0		0	0	https://www.ncbi.nlm.nih.gov/clinvar/variation/130889/
NM_000098.2(CPT2):c.1438G>A	concordant	unreportable	unreportable	447218	0	1	SCV000613021	Uncertain significance	Athena Diagnostics	8/17/2017	CPT2		chr1	53676784	53676784	http://gnomad.b	0.0002210495095	NFE	0.0004218580143	0		0	0	https://www.ncbi.nlm.nih.gov/clinvar/variation/447218/
NM_000098.2(CPT2):c.1646G>A	concordant	unreportable	unreportable	130890	0	1	SCV000700665	Uncertain significance	EGL Genetic Diagn	6/30/2017	CPT2		chr1	53678936	53678936	http://gnomad.b	1.68E-05	AMR	5.96E-05	0		0	0	https://www.ncbi.nlm.nih.gov/clinvar/variation/130890/
NM_000098.2(CPT2):c.1763C>G	concordant	unreportable	unreportable	203664	0	2	SCV000707881;S(	Likely benign;Benign	EGL Genetic Diagn	2017-06-30;2017-1	CPT2		chr1	53679053	53679053	http://gnomad.b	0.0009037847034	AFR	0.01084680017	1		2	0	https://www.ncbi.nlm.nih.gov/clinvar/variation/203664/
NM_001360.2(DHCR7):c.1A>G	concordant	reportable	reportable	6794	2	0	SCV000568697;S(	Likely pathogenic;Likely pat	GeneDx;Laborator	2017-11-28;2018-0	DHCR7		chr11	71155998	71155998	http://gnomad.b	3.50E-05	AMR	2.98E-05	0		0	0	https://www.ncbi.nlm.nih.gov/clinvar/variation/6794/
NM_001360.2(DHCR7):c.866C>T	concordant	reportable	reportable	6789	1	0	SCV000490514	Likely pathogenic	GeneDx	12/21/2016	DHCR7		chr11	71148955	71148955	http://gnomad.b	2.15E-05	NFE	3.58E-05	0		0	0	https://www.ncbi.nlm.nih.gov/clinvar/variation/6789/
NM_001360.2(DHCR7):c.461C>G	concordant	reportable	reportable	166988	1	0	SCV000231620	Likely pathogenic	EGL Genetic Diagn	6/30/2017	DHCR7		chr11	71152438	71152438	http://gnomad.b	9.15E-05	NFE	0.0001632390049	0		0	0	https://www.ncbi.nlm.nih.gov/clinvar/variation/166988/
NM_001360.2(DHCR7):c.1396G>A	concordant	reportable	reportable	265097	2	0	SCV000321551;S(	Pathogenic;Likely pathoger	GeneDx;Invitae	2017-11-28;2017-1	DHCR7		chr11	71146453	71146453	http://gnomad.b	8.08E-06	NFE	2.76E-05	0		0	0	https://www.ncbi.nlm.nih.gov/clinvar/variation/265097/

Supplemental Table 2

NM_001360.2(DHCR7):c.292C>T	concordant	reportable	reportable	188723	1	0	SCV000576842	Pathogenic	GeneDx	11/28/2017	DHCR7	chr11	71155068	71155068	http://gnomad.b	2.96E-05	AMR	8.96E-05	0		0	0	https://www.ncbi.nlm.nih.gov/clinvar/variation/188723/
NM_001360.2(DHCR7):c.670G>A	concordant	unreportable	unreportable	499993	0	1	SCV000705746	Uncertain significance	EGL Genetic Diagn	6/30/2017	DHCR7	chr11	71150086	71150086	http://gnomad.b	2.69E-06	NFE	3.58E-05	0		0	0	https://www.ncbi.nlm.nih.gov/clinvar/variation/499993/
NM_001360.2(DHCR7):c.1406G>C	concordant	unreportable	unreportable	449476	0	1	SCV000617666	Uncertain significance	GeneDx	11/28/2017	DHCR7	chr11	71146443	71146443	http://gnomad.b	2.69E-06			0		0	0	https://www.ncbi.nlm.nih.gov/clinvar/variation/449476/
NM_001360.2(DHCR7):c.841G>A	concordant	reportable	reportable	93724	1	0	SCV000321548	Pathogenic	GeneDx	11/28/2017	DHCR7	chr11	71148980	71148980	http://gnomad.b	7.81E-05	AMR	0.00068529899	0		0	0	https://www.ncbi.nlm.nih.gov/clinvar/variation/93724/
NM_001360.2(DHCR7):c.470T>C	concordant	reportable	reportable	488492	1	0	SCV000700795	Likely pathogenic	EGL Genetic Diagn	6/30/2017	DHCR7	chr11	71152429	71152429	http://gnomad.b	3.23E-05	NFE	9.95E-05	0		0	0	https://www.ncbi.nlm.nih.gov/clinvar/variation/488492/
NM_001360.2(DHCR7):c.742T>C	concordant	unreportable	unreportable	290579	0	1	SCV000345155	Uncertain significance	EGL Genetic Diagn	6/30/2017	DHCR7	chr11	71150014	71150014	http://gnomad.b	2.69E-06	AFR	6.54E-05	0		0	0	https://www.ncbi.nlm.nih.gov/clinvar/variation/290579/
NM_001360.2(DHCR7):c.1138T>C	concordant	reportable	reportable	284527	1	0	SCV000337184	Likely pathogenic	EGL Genetic Diagn	6/30/2017	DHCR7	chr11	71146711	71146711	http://gnomad.b	5.39E-06	AFR	6.59E-05	0		0	0	https://www.ncbi.nlm.nih.gov/clinvar/variation/284527/
NM_000108.3(DLD):c.1123G>A	concordant	reportable	reportable	11971	2	0	SCV000238810;S	Pathogenic;Likely pathoger	GeneDx;Laborator	2017-11-28;2018-0	DLD	chr7	107557794	107557794	http://gnomad.b	0.0002648140071	ASJ	0.002335970057	0		0	0	https://www.ncbi.nlm.nih.gov/clinvar/variation/11971/
NM_000137.2(FAH):c.1A>G	concordant	reportable	reportable	372766	1	0	SCV000491239	Pathogenic	GeneDx	12/21/2016	FAH	chr15	80445397	80445397	http://gnomad.b	1.39E-05	AMR	2.98E-05	0		0	0	https://www.ncbi.nlm.nih.gov/clinvar/variation/372766/
NM_000137.2(FAH):c.1210G>A	concordant	unreportable	unreportable	459903	0	1	SCV000631828	Uncertain significance	Invitae	10/5/2017	FAH	chr15	80478501	80478501	http://gnomad.b	1.66E-05	EAS	5.80E-05	0		0	0	https://www.ncbi.nlm.nih.gov/clinvar/variation/459903/
NM_000136.2(FANCC):c.1661T>C	concordant	reportable	reportable	12043	1	0	SCV000512973	Pathogenic	GeneDx	11/28/2017	FANCC	chr9	97864005	97864005	http://gnomad.b	7.13E-06	NFE	2.69E-05	0		0	0	https://www.ncbi.nlm.nih.gov/clinvar/variation/12043/
NM_000136.2(FANCC):c.584A>T	concordant	unreportable	unreportable	134305	0	5	SCV000257632;S	Uncertain significance;Beni	Division of Genomi	2015-08-10;2017-0	FANCC	chr9	97912307	97912307	http://gnomad.b	0.00360759283	NFE	0.004676579963	4		4	0	https://www.ncbi.nlm.nih.gov/clinvar/variation/134305/
NM_000136.2(FANCC):c.37C>T	concordant	reportable	reportable	12046	2	0	SCV000149260;S	Pathogenic;Pathogenic	GeneDx;Invitae	2017-11-28;2017-0	FANCC	chr9	98011537	98011537	http://gnomad.b	1.43E-05	AMR	2.98E-05	0		0	0	https://www.ncbi.nlm.nih.gov/clinvar/variation/12046/
NM_000136.2(FANCC):c.65G>A	concordant	reportable	reportable	188926	1	0	SCV000695435	Pathogenic	Laboratory Corpora	1/25/2018	FANCC	chr9	98011509	98011509	http://gnomad.b	1.90E-05	AFR	6.54E-05	0		0	0	https://www.ncbi.nlm.nih.gov/clinvar/variation/188926/
NM_001079802.1(FKTN):c.919C>T	concordant	reportable	reportable	3216	2	0	SCV000589561;S	Pathogenic;Likely pathoger	GeneDx;Laborator	2017-11-28;2018-0	FKTN	chr9	108380248	108380248	http://gnomad.b	2.13E-05	EAS	0.0001161850014	0		0	0	https://www.ncbi.nlm.nih.gov/clinvar/variation/3216/
NM_000151.3(G6PC):c.562G>A	concordant	reportable	reportable	214465	2	0	SCV000251527;S	Pathogenic;Pathogenic	GeneDx;Laborator	2017-11-28;2018-0	G6PC	chr17	41061435	41061435	http://gnomad.b	1.28E-05	FIN	4.49E-05	0		0	0	https://www.ncbi.nlm.nih.gov/clinvar/variation/214465/
NM_000151.3(G6PC):c.113A>T	concordant	reportable	reportable	12004	1	0	SCV000603763	Pathogenic	ARUP Laboratories	6/30/2017	G6PC	chr17	41053006	41053006	http://gnomad.b	3.58E-05	NFE	1.79E-05	0		0	0	https://www.ncbi.nlm.nih.gov/clinvar/variation/12004/
NM_000152.3(GAA):c.2132C>G	concordant	unreportable	unreportable	381006	0	1	SCV000520045	Likely benign	GeneDx	11/28/2017	GAA	chr17	78087108	78087108	http://gnomad.b	2.78E-06	EAS	0.00140809	0		0	0	https://www.ncbi.nlm.nih.gov/clinvar/variation/381006/
NM_000152.3(GAA):c.1841C>A	concordant	reportable	reportable	167113	1	0	SCV000700459	Likely pathogenic	EGL Genetic Diagn	6/30/2017	GAA	chr17	78086463	78086463	http://gnomad.b	3.34E-05	NFE	3.68E-05	0		0	0	https://www.ncbi.nlm.nih.gov/clinvar/variation/167113/
NM_000152.3(GAA):c.1556T>C	concordant	unreportable	unreportable	189124	0	1	SCV000703062	Uncertain significance	EGL Genetic Diagn	6/30/2017	GAA	chr17	78084744	78084744	http://gnomad.b	2.78E-06	SAS	3.25E-05	0		0	0	https://www.ncbi.nlm.nih.gov/clinvar/variation/189124/
NM_000152.3(GAA):c.2173C>T	concordant	reportable	reportable	4024	1	0	SCV000695648	Pathogenic	Laboratory Corpora	1/25/2018	GAA	chr17	78087149	78087149	http://gnomad.b	5.00E-05	ASJ	0.0006996269803	0		0	0	https://www.ncbi.nlm.nih.gov/clinvar/variation/4024/
NM_000152.3(GAA):c.1735G>A	concordant	reportable	reportable	495664	1	0	SCV000695643	Pathogenic	Laboratory Corpora	1/25/2018	GAA	chr17	78085880	78085880	http://gnomad.b	8.34E-06	NFE	8.95E-06	0		0	0	https://www.ncbi.nlm.nih.gov/clinvar/variation/495664/
NM_000152.3(GAA):c.2284G>A	concordant	unreportable	unreportable	456399	0	1	SCV000626560	Uncertain significance	Invitae	10/5/2017	GAA	chr17	78090861	78090861	http://gnomad.b	2.22E-05	SAS	3.25E-05	0		0	0	https://www.ncbi.nlm.nih.gov/clinvar/variation/456399/
NM_000152.3(GAA):c.1375G>A	concordant	unreportable	unreportable	188480	1	1	SCV000702471;S	Uncertain significance;Likel	EGL Genetic Diagn	2017-06-30;2017-1	GAA	chr17	78083792	78083792	http://gnomad.b	1.95E-05	AMR	0.0004172630142	0		0	0	https://www.ncbi.nlm.nih.gov/clinvar/variation/188480/
NM_000152.3(GAA):c.1933G>A	concordant	reportable	reportable	188728	1	0	SCV000568677	Pathogenic	GeneDx	11/28/2017	GAA	chr17	78086719	78086719	http://gnomad.b	2.50E-05	AFR	6.94E-05	0		0	0	https://www.ncbi.nlm.nih.gov/clinvar/variation/188728/
NM_000152.3(GAA):c.784G>A	concordant	reportable	reportable	188806	1	0	SCV000626642	Likely pathogenic	Invitae	10/5/2017	GAA	chr17	78081447	78081447	http://gnomad.b	1.11E-05	EAS	5.80E-05	0		0	0	https://www.ncbi.nlm.nih.gov/clinvar/variation/188806/
NM_000152.3(GAA):c.1799G>A	concordant	reportable	reportable	370130	1	0	SCV000617644	Pathogenic	GeneDx	11/28/2017	GAA	chr17	78086421	78086421	http://gnomad.b	1.39E-05	SAS	3.25E-05	0		0	0	https://www.ncbi.nlm.nih.gov/clinvar/variation/370130/
NM_000152.3(GAA):c.2105G>A	concordant	reportable	reportable	426278	4	0	SCV000709614;S	Likely pathogenic;Pathoger	EGL Genetic Diagn	2017-06-30;2017-1	GAA	chr17	78087081	78087081	http://gnomad.b	3.61E-05	NFE	9.23E-05	0		0	0	https://www.ncbi.nlm.nih.gov/clinvar/variation/426278/
NM_000152.3(GAA):c.503G>A	concordant	unreportable	unreportable	325778	0	2	SCV000407260;S	Uncertain significance;Unc	Illumina Clinical Se	2016-10-18;2017-1	GAA	chr17	78078888	78078888	http://gnomad.b	8.89E-05	EAS	0.001517810044	0		0	0	https://www.ncbi.nlm.nih.gov/clinvar/variation/325778/
NM_000152.3(GAA):c.1942G>A	concordant	reportable	reportable	188902	1	0	SCV000626535	Likely pathogenic	Invitae	10/5/2017	GAA	chr17	78086728	78086728	http://gnomad.b	2.78E-05	SAS	0.0003345820005	0		0	0	https://www.ncbi.nlm.nih.gov/clinvar/variation/188902/
NM_000152.3(GAA):c.655G>A	concordant	reportable	reportable	189065	4	0	SCV000330942;S	Pathogenic;Pathogenic;Like	EGL Genetic Diagn	2017-06-30;2017-1	GAA	chr17	78079656	78079656		1.39E-05					0	0	https://www.ncbi.nlm.nih.gov/clinvar/variation/189065/
NM_000152.3(GAA):c.925G>A	concordant	reportable	reportable	188797	1	0	SCV000700458	Pathogenic	EGL Genetic Diagn	6/30/2017	GAA	chr17	78081665	78081665	http://gnomad.b	3.61E-05	ASJ	0.0001030290005	0		0	0	https://www.ncbi.nlm.nih.gov/clinvar/variation/188797/
NM_000152.3(GAA):c.1324G>A	concordant	unreportable	unreportable	497377	0	1	SCV000701854	Uncertain significance	EGL Genetic Diagn	6/30/2017	GAA	chr17	78082625	78082625		3.61E-05					0	0	https://www.ncbi.nlm.nih.gov/clinvar/variation/497377/
NM_000152.3(GAA):c.1655T>C	concordant	reportable	reportable	279811	3	0	SCV000331328;S	Pathogenic;Pathogenic;Pat	EGL Genetic Diagn	2017-06-30;2017-1	GAA	chr17	78085800	78085800	http://gnomad.b	3.61E-05	NFE	6.27E-05	0		0	0	https://www.ncbi.nlm.nih.gov/clinvar/variation/279811/
NM_000152.3(GAA):c.2014C>T	concordant	reportable	reportable	188773	2	0	SCV000336054;S	Pathogenic;Likely pathoger	EGL Genetic Diagn	2017-06-30;2016-1	GAA	chr17	78086800	78086800		1.67E-05					0	0	https://www.ncbi.nlm.nih.gov/clinvar/variation/188773/
NM_000152.3(GAA):c.2297A>G	concordant	unreportable	unreportable	285197	0	1	SCV000338123	Uncertain significance	EGL Genetic Diagn	6/30/2017	GAA	chr17	78090874	78090874	http://gnomad.b	1.95E-05	SAS	6.50E-05	0		0	0	https://www.ncbi.nlm.nih.gov/clinvar/variation/285197/
NM_000153.3(GALC):c.1186C>T	concordant	reportable	reportable	456712	1	0	SCV000627123	Pathogenic	Invitae	10/5/2017	GALC	chr14	88417068	88417068		4.48E-05					0	0	https://www.ncbi.nlm.nih.gov/clinvar/variation/456712/
NM_000153.3(GALC):c.1403C>G	concordant	unreportable	unreportable	92495	0	1	SCV000627126	Benign	Invitae	10/5/2017	GALC	chr14	88414158	88414158	http://gnomad.b	0.001219525839	AFR	0.0159115009	1		2	0	https://www.ncbi.nlm.nih.gov/clinvar/variation/92495/
NM_000153.3(GALC):c.749T>C	concordant	reportable	reportable	265479	1	0	SCV000322420	Pathogenic	GeneDx	11/28/2017	GALC	chr14	88442705	88442705	http://gnomad.b	2.80E-06	NFE	1.79E-05	0		0	0	https://www.ncbi.nlm.nih.gov/clinvar/variation/265479/
NM_000153.3(GALC):c.205C>T	concordant	reportable	reportable	195020	1	0	SCV000695676	Pathogenic	Laboratory Corpora	1/25/2018	GALC	chr14	88454858	88454858	http://gnomad.b	5.59E-06	SAS	3.25E-05	0		0	0	https://www.ncbi.nlm.nih.gov/clinvar/variation/195020/
NM_000153.3(GALC):c.1592G>A	concordant	reportable	reportable	167119	1	0	SCV000617677	Pathogenic	GeneDx	11/28/2017	GALC	chr14	88411975	88411975	http://gnomad.b	0.0001174772598	EAS	0.0005218600272	0		0	0	https://www.ncbi.nlm.nih.gov/clinvar/variation/167119/
NM_000153.3(GALC):c.850G>A	concordant	reportable	reportable	374024	1	0	SCV000695673	Pathogenic	Laboratory Corpora	1/25/2018	GALC	chr14	88434737	88434737	http://gnomad.b	1.68E-05	NFE	1.79E-05	0		0	0	https://www.ncbi.nlm.nih.gov/clinvar/variation/374024/
NM_000154.1(GALK1):c.593C>T	concordant	unreportable	unreportable	5633	0	1	SCV000631404	Uncertain significance	Invitae	10/5/2017	GALK1	chr17	73759113	73759113	http://gnomad.b	0.00193947554	EAS	0.01554519963	2		2	0	https://www.ncbi.nlm.nih.gov/clinvar/variation/5633/
NM_000155.3(GALT):c.241G>A	concordant	unreportable	unreportable	25141	0	1	SCV000227033	Uncertain significance	EGL Genetic Diagn	6/30/2017	GALT	chr9	34647244	34647244		5.50E-06					0	0	https://www.ncbi.nlm.nih.gov/clinvar/variation/25141/
NM_000155.3(GALT):c.341A>C	concordant	unreportable	unreportable	280963	0	1	SCV000330950	Uncertain significance	EGL Genetic Diagn	6/30/2017	GALT	chr9	34647666	34647666		2.75E-06					0	0	https://www.ncbi.nlm.nih.gov/clinvar/variation/280963/
NM_000155.3(GALT):c.1132A>G	concordant	unreportable	unreportable	25334	0	1	SCV000603800	Uncertain significance	ARUP Laboratories	6/30/2017	GALT	chr9	34650438	34650438	http://gnomad.b	0.0001814014083	NFE	0.0001701930014	0		0	0	https://www.ncbi.nlm.nih.gov/clinvar/variation/25334/
NM_000155.3(GALT):c.997C>T	concordant	reportable	reportable	3610	3	0	SCV000110084;S	Pathogenic;Pathogenic;Pat	EGL Genetic Diagn	2017-06-30;2017-0	GALT	chr9	34649499	34649499	http://gnomad.b	3.85E-05	SAS	3.25E-05	0		0	0	https://www.ncbi.nlm.nih.gov/clinvar/variation/3610/
NM_000155.3(GALT):c.292G>C	concordant	reportable	reportable	25149	2	0	SCV000330952;S	Pathogenic;Pathogenic	EGL Genetic Diagn	2017-06-30;2018-0	GALT	chr9	34647528	34647528		2.47E-05					0	0	https://www.ncbi.nlm.nih.gov/clinvar/variation/25149/
NM_000155.3(GALT):c.602G>A	concordant	reportable	reportable	25227	1	0	SCV000330954	Pathogenic	EGL Genetic Diagn	6/30/2017	GALT	chr9	34648368	34648368	http://gnomad.b	2.47E-05	SAS	0.0001624330034	0		0	0	https://www.ncbi.nlm.nih.gov/clinvar/variation/25227/
NM_000155.3(GALT):c.652C>G	concordant	unreportable	unreportable	25239	0	1	SCV000331030	Uncertain significance	EGL Genetic Diagn	6/30/2017	GALT	chr9	34648418	34648418		2.75E-06					0	0	https://www.ncbi.nlm.nih.gov/clinvar/variation/25239/
NM_000155.3(GALT):c.1030C>A	concordant	reportable	reportable	25320	2	0	SCV000521168;S	Likely pathogenic;Pathoger	GeneDx;Laborator	2017-11-28;2015-1	GALT	chr9	34649532	34649532		2.20E-05					0	0	https://www.ncbi.nlm.nih.gov/clinvar/variation/25320/
NM_000155.3(GALT):c.961C>T	concordant	unreportable	unreportable	25299	0	1	SCV000695710	Uncertain significance	Laboratory Corpora	1/25/2018	GALT	chr9	34649463	34649463	http://gnomad.b	1.65E-05	AFR	6.53E-05	0		0	0	https://www.ncbi.nlm.nih.gov/clinvar/variation/25299/
NM_000155.3(GALT):c.775C>T	concordant	reportable	reportable	189191	1	0	SCV000232473	Pathogenic	EGL Genetic Diagn	6/30/2017	GALT	chr9	34648846	34648846	http://gnomad.b	5.50E-06	AMR	2.98E-05	1		0	0	https://www.ncbi.nlm.nih.gov/clinvar/variation/189191/
NM_000155.3(GALT):c.389G>A	concordant	unreportable	unreportable	25168	0	1	SCV000331243	Uncertain significance	EGL Genetic Diagn	6/30/2017	GALT	chr9	34647840	34647840		2.75E-06					0	0	https://www.ncbi.nlm.nih.gov/clinvar/variation/25168/
NM_000159.2(GCDH):c.572T>C	concordant	reportable	reportable	198396	2	0	SCV000232017;S	Likely pathogenic;Likely pat	EGL Genetic Diagn	2017-06-30;2017-1	GCDH	chr19	13006872	13006872	http://gnomad.b	0.0001068303983	NFE	0.0001342739997	0		0	0	https://www.ncbi.nlm.nih.gov/clinvar/variation/198396/
NM_000159.2(GCDH):c.1213A>G	concordant	reportable	reportable	193798	2	0	SCV000225199;S	Pathogenic;Pathogenic	EGL Genetic Diagn	2017-06-30;2016-1	GCDH	chr19	13008647	13008647	http://gnomad.b	7.03E-05	AFR	0.0004575760104	0		0	0	https://www.ncbi.nlm.nih.gov/clinvar/variation/193798/
NM_000159.2(GCDH):c.382C>T	concordant	reportable	reportable	371176	1	0	SCV000695720	Pathogenic	Laboratory Corpora	1/25/2018	GCDH	chr19	13004344	13004344		2.81E-06					0	0	https://www.ncbi.nlm.nih.gov/clinvar/variation/371176/
NM_000159.2(GCDH):c.1063C>T	concordant	reportable	reportable	430222	1	0	SCV000582975	Likely pathogenic	GeneDx	11/28/2017	GCDH	chr19	13008223	13008223	http://gnomad.b	2.53E-05	EAS	0.0001740949956	0		0	0	https://www.ncbi.nlm.nih.gov/clinvar/variation/430222/
NM_000159.2(GCDH):c.877G>A	concordant	reportable	reportable	2083	1	0	SCV000695723	Pathogenic	Laboratory Corpora	1/25/2018	GCDH	chr19	13007748	13007748	http://gnomad.b	4.22E-05	AFR	0.0004586550058	0		0	0	https://www.ncbi.nlm.nih.gov/clinvar/variation/2083/
NM_000159.2(GCDH):c.482G>A	concordant	reportable	reportable	188789	1	0	SCV000339588	Pathogenic	EGL Genetic Diagn	6/30/2017	GCDH	chr19	13004444	13004444	http://gnomad.b	2.25E-05	NFE	6.27E-05	0		0	0	https://www.ncbi.nlm.nih.gov/clinvar/variation/188789/
NM_000159.2(GCDH):c.680G>C	concordant	reportable	reportable	2089	3	0	SCV000321706;S	Pathogenic;Pathogenic;Pat	GeneDx;Illumina C	2017-11-28;2016-1	GCDH	chr19	13007063	13007063	http://gnomad.b	0.0001911701864	NFE	0.0003405020107	0		0	0	https://www.ncbi.nlm.nih.gov/clinvar/variation/2089/
NM_000159.2(GCDH):c.1198G>A	concordant	reportable	reportable	2088	1	0	SCV000329357	Pathogenic	GeneDx	11/28/2017	GCDH	chr19	13008632	13008632	http://gnomad.b	0.0001152643771	NFE	0.0001612380001	0		0	0	https://www.ncbi.nlm.nih.gov/clinvar/variation/2088/
NM_000159.2(GCDH):c.1262C>T	concordant	reportable	reportable	2082	3	0	SCV000321708;S	Pathogenic;Pathogenic;Pat	GeneDx;Illumina C	2017-11-28;2016-1	GCDH	chr19	13010300	13010300	http://gnomad.b	0.0001012077458	NFE	0.0002330120042	0		0	0	https://www.ncbi.nlm.nih.gov/clinvar/variation/2082/
NM_000159.2(GCDH):c.769C>T	concordant	reportable	reportable	188872	1	0	SCV000568702	Likely pathogenic	GeneDx	11/28/2017	GCDH	chr19	13007152	13007152	http://gnomad.b	1.97E-05	SAS	9.75E-05	0		0	0	https://www.ncbi.nlm.nih.gov/clinvar/variation/188872/
NM_000159.2(GCDH):c.262C>T	concordant	reportable	reportable	189150	2	0	SCV000321704;S	Pathogenic;Pathogenic	GeneDx;Laborator	2017-11-28;2018-0	GCDH	chr19	13002779	13002779	http://gnomad.b	4.22E-05	NFE	0.0001074559987	0		0	0	https://www.ncbi.nlm.nih.gov/clinvar/variation/189150/
NM_004004.5(GJB2):c.44A>C	concordant	reportable	reportable	44752	2	0	SCV000599724;S	Likely pathogenic;Pathoger	Division of Genomi	2017-05-17;2017-1	GJB2	chr13	20763677	20763677	http://gnomad.b	2.79E-05	NFE	1.82E-05	0		0	0	https://www.ncbi.nlm.nih.gov/clinvar/variation/44752/
NM_004004.5(GJB2):c.503A>G	concordant	unreportable	unreportable	44756	0	2	SCV000599755;S	Uncertain significance;Likel	Division of Genomi	2017-05-17;2018-0	GJB2	chr13	20763218	20763218	http://gnomad.b	0.0003604338618	AMR	0.0003575049923	0		0	0	https://www.ncbi.nlm.nih.gov/clinvar/variation/44756/
NM_004004.5(GJB2):c.617A>G	concordant	reportable	reportable	44763	3	0	SCV000603822;S	Pathogenic;Pathogenic;Pat	ARUP Laboratories	2017-06-30;2017-0	GJB2	chr13	20763104	20763104	http://gnomad.b	0.0001341149253	AMR	0.0004765309859	0		0	0	https://www.ncbi.nlm.nih.gov/clinvar/variation/44763/
NM_004004.5(GJB2):c.298C>T	concordant	reportable	reportable	158607	2	0	SCV000603836;S	Pathogenic;Pathogenic	ARUP Laboratories	2017-06-30;2016-0	GJB2	chr13	20763423	20763423	http://gnomad.b	1.40E-05	AFR	6.54E-05	0		0	0	https://www.ncbi.nlm.nih.gov/clinvar/variation/158607/
NM_004004.5(GJB2):c.23C>T	concordant	unreportable	unreportable	379889	0	1	SCV000517380	Uncertain significance	GeneDx	11/28/2017	GJB2	chr13	20763698	20763698	http://gnomad.b	0.0001201446206	SAS	0.0004223250144	0		0	0	https://www.ncbi.nlm.nih.gov/clinvar/variation/379889/
NM_004004.5(GJB2):c.56G>C	concordant	unreportable	unreportable	21389	0	1	SCV000599725	Uncertain significance	Division of Genomi	5/17/2017	GJB2	chr13	20763665	20763665	http://gnomad.b	1.68E-05	NFE	2.73E-05	0		0	0	https://www.ncbi.nlm.nih.gov/clinvar/variation/21389/
NM_004004.5(GJB2):c.339T>G	concordant	unreportable	unreportable	21385	0	1	SCV000383018	Uncertain significance	Illumina Clinical Se	10/18/2016	GJB2	chr13	20763382	20763382	http://gnomad.b	2.79E-05	ASJ	0.0003049400111	0		0	0	https://www.ncbi.nlm.nih.gov/clinvar/variation/21385/
NM_004004.5(GJB2):c.608T>C	concordant	unreportable	unreportable	44762	0	2	SCV000599764;S	Benign;Benign	Division of Genomi	2017-05-17;2016-1	GJB2	chr13	20763113	20763113	http://gnomad.b	0.004076534917	EAS	0.05828789994	27		34	0	https://www.ncbi.nlm.nih.gov/clinvar/variation/44762/
NM_004004.5(GJB2):c.488T>C	concordant	unreportable	unreportable	449488	0	1	SCV000617685	Uncertain significance	GeneDx	11/28/2017	GJB2	chr13	20763233	20763233		2.79E-06					0	0	https://www.ncbi.nlm.nih.gov/clinvar/variation/449488/
NM_004004.5(GJB2):c.279G>A	concordant	reportable	reportable	44733	1	0	SCV000617688	Likely pathogenic	GeneDx	11/28/2017	GJB2	chr13	20763442	20763442	http://gnomad.b	8.38E-06	AFR	6.54E-05	0		0	0	https://www.ncbi.nlm.nih.gov/clinvar/variation/44733/
NM_004004.5(GJB2):c.60T>G	concordant	unreportable	unreportable	438615	0	1	SCV000599726	Uncertain significance	Division of Genomi	5/17/2017	GJB2	chr13	20763661	20763661	http://gnomad.b	7.82E-05	ASJ	0.0008125129971	0		0	0	https://www.ncbi.nlm.nih.gov/clinvar/variation/438615/
NM_004004.5(GJB2):c.583A>G	concordant	reportable	reportable	225375	1	0	SCV000599761	Likely pathogenic	Division of Genomi	5/17/2017	GJB2	chr13	20763138	20763138	http://gnomad.b	5.03E-05	EAS	0.0002319110063	0		0	0	https://www.ncbi.nlm.nih.gov/clinvar/variation/225375/
NM_004004.5(GJB2):c.427C>T	concordant	reportable	reportable	17009	6	0	SCV000599751;S	Pathogenic;Pathogenic;Pat	Division of Genomi	2017-05-17;2017-0	GJB2	chr13	20763294	20763294	http://gnomad.b	0.0001089683768	AFR	0.0006554800202	0		0	0	https://www.ncbi.nlm.nih.gov/clinvar/variation/17009/
NM_004004.5(GJB2):c.475G>A	concordant	unreportable	unreportable	438618	0	1	SCV000599754	Uncertain significance	Division of Genomi	5/17/2017	GJB2	chr13	20763246	20763246	http://gnomad.b	5.87E-05	NFE	0.0001705140021	0		0	0	https://www.ncbi.nlm.nih.gov/clinvar/variation/438618/
NM_004004.5(GJB2):c.355G>A	concordant	unreportable	unreportable	188488	0	1	SCV000383010	Likely benign	Illumina Clinical Se	10/18/2016	GJB2	chr13	20763366	20763366	http://gnomad.b	0.0001229386815	NFE	0.0001621450065	0		0	0	https://www.ncbi.nlm.nih.gov/clinvar/variation/188488/
NM_004004.5(GJB2):c.95G>T	concordant	reportable	reportable	499513	1	0	SCV000705030	Likely pathogenic	EGL Genetic Diagn	6/30/2017	GJB2	chr13	20763626	20763626	http://gnomad.b	1.40E-05	AMR	0.0002382800012	0		0	0	https://www.ncbi.nlm.nih.gov/clinvar/variation/499513/
NM_004004.5(GJB2):c.380G>T	concordant	unreportable	unreportable	195204	0	2	SCV000227300;S	Uncertain significance;Unc	EGL Genetic Diagn	2017-06-30;2018-0	GJB2	chr13	20763341	20763341	http://gnomad.b	0.0001285268034	AMR	0.001846999978	0		1	0	https://www.ncbi.nlm.nih.gov/clinvar/variation/195204/
NM_004004.5(GJB2):c.445G>A	concordant	unreportable	unreportable	44751	0	1	SCV000599752	Uncertain significance	Division of Genomi	5/17/2017	GJB2	chr13	20763276	20763276	http://gnomad.b	9.22E-05	NFE	8.08E-05	0		0	0	https://www.ncbi.nlm.nih.gov/clinvar/variation/44751/
NM_004004.5(GJB2):c.511G>A	concordant	unreportable	unreportable	44758	0	1	SCV000599757	Uncertain significance	Division of Genomi	5/17/2017	GJB2	chr13	20763210	20763210	http://gnomad.b	0.000201172388	NFE	0.0001794819982	0		0	0	https://www.ncbi.nlm.nih.gov/clinvar/variation/44758/
NM_004004.5(GJB2):c.551G>C	concordant	reportable	reportable	17007	2	0	SCV000599760;S	Pathogenic;Pathogenic	Division of Genomi	2017-05-17;2018-0	GJB2	chr13	20763170	20763170	http://gnomad.b	6.71E-05	AMR	8.93E-05	0		0	0	https://www.ncbi.nlm.nih.gov/clinvar/variation/17007/
NM_004004.5(GJB2):c.250G>A	concordant	reportable	reportable	17036	1	0	SCV000322426	Pathogenic	GeneDx	11/28/2017	GJB2	chr13	20763471	20763471	http://gnomad.b	1.12E-05	ASJ	0.0001015230009	0		0	0	https://www.ncbi.nlm.nih.gov/clinvar/variation/17036/
NM_004004.5(GJB2):c.283G>A	concordant	reportable	reportable	44735	3	0	SCV000603835;S	Pathogenic;Pathogenic;Pat	ARUP Laboratories	2017-06-30;2017-0	GJB2	chr13	20763438	20763438	http://gnomad.b	4.19E-05	AMR	0.0001488980051	0		0	0	https://www.ncbi.nlm.nih.gov/clinvar/variation/44735/
NM_004004.5(GJB2):c.250G>C	concordant	reportable	reportable	17032	2	0	SCV000599740;S	Pathogenic;Pathogenic	Division of Genomi	2017-05-17;2018-0	GJB2	chr13	20763471	20763471	http://gnomad.b	2.24E-05	AFR	0.0001306850027	0		0	0	https://www.ncbi.nlm.nih.gov/clinvar/variation/17032/
NM_004004.5(GJB2):c.187G>T	concordant	unreportable	unreportable	447443	0	1	SCV000613507	Uncertain significance	Athena Diagnostics	8/17/2017	GJB2	chr13	20763534	20763534	http://gnomad.b	2.51E-05	EAS	0.000115969	0		0	0	https://www.ncbi.nlm.nih.gov/clinvar/variation/447443/
NM_004004.5(GJB2):c.250G>T	concordant	reportable	reportable	167134	1	0	SCV000603824	Pathogenic	ARUP Laboratories	6/30/2017	GJB2	chr13	20763471	20763471	http://gnomad.b	5.59E-06	AMR	0.0001191190022	0		0	0	https://www.ncbi.nlm.nih.gov/clinvar/variation/167134/
NM_004004.5(GJB2):c.109G>A	concordant	reportable	reportable	17023	5	1	SCV000603818;S	Pathogenic;Pathogenic;Unc	ARUP Laboratories	2017-06-30;2017-0	GJB2	chr13	20763612	20763612	http://gnomad.b	0.008717470145	EAS	0.08076299727	82		90	0	https://www.ncbi.nlm.nih.gov/clinvar/variation/17023/
NM_004004.5(GJB2):c.1A>G	concordant	reportable	reportable	44729	4	0	SCV000603820;S	Pathogenic;Pathogenic;Pat	ARUP Laboratories	2017-06-30;2017-0	GJB2	chr13	20763720	20763720	http://gnomad.b	4.47E-05	SAS	0.0001624330034	0		0	0	https://www.ncbi.nlm.nih.gov/clinvar/variation/44729/
NM_004004.5(GJB2):c.19C>T	concordant	reportable	reportable	44728	1	0	SCV000698233	Pathogenic	Laboratory Corpora	1/25/2018	GJB2	chr13	20763702	20763702	http://gnomad.b	8.38E-06	AMR	5.96E-05	0		0	0	https://www.ncbi.nlm.nih.gov/clinvar/variation/44728/
NM_004004.5(GJB2):c.169C>T	concordant	reportable	reportable	44725	2	0	SCV000603833;S	Pathogenic;Pathogenic	ARUP Laboratories	2017-06-30;2017-0	GJB2	chr13	20763552	20763552	http://gnomad.b	4.75E-05	NFE	8.08E-05	0		0	0	https://www.ncbi.nlm.nih.gov/clinvar/variation/44725/
NM_004004.5(GJB2):c.146C>T	concordant	unreportable	unreportable	372771	0	1	SCV000491248	Uncertain significance	GeneDx	12/21/2016	GJB2	chr13	20763575	20763575		5.59E-06					0	0	https://www.ncbi.nlm.nih.gov/clinvar/variation/372771/
NM_004004.5(GJB2):c.241C>G	concordant	unreportable	unreportable	311372	0	1	SCV000383028	Likely benign	Illumina Clinical Se	10/18/2016	GJB2	chr13	20763480	20763480	http://gnomad.b	5.03E-05	AFR	0.0003267120046	0		1	0	https://www.ncbi.nlm.nih.gov/clinvar/variation/311372/
NM_004004.5(GJB2):c.358G>A	concordant	unreportable	unreportable	447444	0	2	SCV000613515;S	Uncertain significance;Unc	Athena Diagnostics	2017-08-17;2018-0	GJB2	chr13	20763363	20763363	http://gnomad.b	5.59E-05	AFR	6.55E-05	0		0	0	https://www.ncbi.nlm.nih.gov/clinvar/variation/447444/

Supplemental Table 2

NM_004004.5(GJB2):c.34G>T	concordant	reportable	reportable	44740	6	1	SCV000603834;S(	Likely pathogenic;Likely pat	ARUP Laboratories	2017-06-30;2017-0	GJB2	chr13	20763687	20763687	http://gnomad.b	0.0001089683768	AMR	0.003787650028	1	0	0	https://www.ncbi.nlm.nih.gov/clinvar/variation/44740/
NM_004004.5(GJB2):c.110T>C	concordant	unreportable	unreportable	449490	1	1	SCV000617689;S(	Likely pathogenic;Uncertai	GeneDx;Laborator	2017-11-28;2018-0	GJB2	chr13	20763611	20763611	http://gnomad.b	8.10E-05	AFR	0.0003920540039	0	0	0	https://www.ncbi.nlm.nih.gov/clinvar/variation/449490/
NM_004004.5(GJB2):c.677T>G	concordant	unreportable	unreportable	447450	0	1	SCV000613524	Uncertain significance	Athena Diagnostics	8/17/2017	GJB2	chr13	20763044	20763044	http://gnomad.b	3.63E-05	AMR	2.98E-05	0	0	0	https://www.ncbi.nlm.nih.gov/clinvar/variation/447450/
NM_004004.5(GJB2):c.456C>A	concordant	reportable	reportable	44753	1	0	SCV000680740	Likely pathogenic	GeneDx	2/7/2018	GJB2	chr13	20763265	20763265	http://gnomad.b	2.79E-06	AMR	2.98E-05	0	0	0	https://www.ncbi.nlm.nih.gov/clinvar/variation/44753/
NM_004004.5(GJB2):c.94C>T	concordant	reportable	reportable	188758	1	0	SCV000700804	Likely pathogenic	EGL Genetic Diagn	6/30/2017	GJB2	chr13	20763627	20763627	http://gnomad.b	5.31E-05	AFR	0.0003267120046	0	0	0	https://www.ncbi.nlm.nih.gov/clinvar/variation/188758/
NM_004004.5(GJB2):c.131G>A	concordant	reportable	reportable	188830	5	0	SCV000613505;S(	Pathogenic;Pathogenic;Pat	Athena Diagnostics	2017-08-17;2017-0	GJB2	chr13	20763590	20763590	http://gnomad.b	1.12E-05	AMR	0.0001787100045	0	0	0	https://www.ncbi.nlm.nih.gov/clinvar/variation/188830/
NM_004004.5(GJB2):c.132G>A	concordant	reportable	reportable	158605	1	0	SCV000700809	Pathogenic	EGL Genetic Diagn	6/30/2017	GJB2	chr13	20763589	20763589	http://gnomad.b	2.24E-05	AFR	0.0002614039986	0	0	0	https://www.ncbi.nlm.nih.gov/clinvar/variation/158605/
NM_000170.2(GLDC):c.1229G>A	concordant	unreportable	unreportable	225378	0	2	SCV000480516;S(	Likely benign;Benign	Illumina Clinical Se	2016-10-18;2017-1	GLDC	chr9	6595046	6595046	http://gnomad.b	0.002616963042	AMR	0.02629379928	13	7	0	https://www.ncbi.nlm.nih.gov/clinvar/variation/225378/
NM_000170.2(GLDC):c.1545G>C	concordant	reportable	reportable	11985	1	0	SCV000636358	Pathogenic	Invitae	10/5/2017	GLDC	chr9	6589230	6589230	http://gnomad.b	8.00E-05	NFE	0.0001879899937	0	0	0	https://www.ncbi.nlm.nih.gov/clinvar/variation/11985/
NM_000170.2(GLDC):c.1009C>T	concordant	reportable	reportable	56036	2	0	SCV000329726;S(	Pathogenic;Pathogenic	GeneDx;Laborator	2017-11-28;2018-0	GLDC	chr9	6604637	6604637	http://gnomad.b	4.00E-05	SAS	3.25E-05	0	0	0	https://www.ncbi.nlm.nih.gov/clinvar/variation/56036/
NM_000170.2(GLDC):c.1525C>G	concordant	unreportable	unreportable	462855	0	1	SCV000636356	Uncertain significance	Invitae	10/5/2017	GLDC	chr9	6589250	6589250	http://gnomad.b	0.0002480912976	SAS	0.002403999912	0	0	0	https://www.ncbi.nlm.nih.gov/clinvar/variation/462855/
NM_000170.2(GLDC):c.499G>T	concordant	reportable	reportable	370365	2	0	SCV000636407;S(	Pathogenic;Pathogenic	Invitae;Laboratory	2017-10-05;2018-0	GLDC	chr9	6610328	6610328	http://gnomad.b	3.20E-05	AMR	8.93E-05	0	0	0	https://www.ncbi.nlm.nih.gov/clinvar/variation/370365/
NM_001128227.2(GNE):c.410T>C	concordant	unreportable	unreportable	288301	0	1	SCV000342374	Uncertain significance	EGL Genetic Diagn	6/30/2017	GNE	chr9	36246327	36246327	http://gnomad.b	8.40E-06	AMR	2.98E-05	0	0	0	https://www.ncbi.nlm.nih.gov/clinvar/variation/288301/
NM_005476.5(GNE):c.1760T>C	concordant	reportable	reportable	188882	1	0	SCV000701266	Pathogenic	EGL Genetic Diagn	6/30/2017	GNE	chr9	36219891	36219891	http://gnomad.b	2.80E-05	NFE	8.95E-06	0	0	0	https://www.ncbi.nlm.nih.gov/clinvar/variation/188882/
NM_001128227.2(GNE):c.304A>T	concordant	unreportable	unreportable	290713	0	2	SCV000345325;S(	Uncertain significance;Unc	EGL Genetic Diagn	2017-06-30;2017-1	GNE	chr9	36246433	36246433		2.24E-05				0	0	https://www.ncbi.nlm.nih.gov/clinvar/variation/290713/
NM_001128227.2(GNE):c.577C>T	concordant	reportable	reportable	290196	1	0	SCV000701417	Pathogenic	EGL Genetic Diagn	6/30/2017	GNE	chr9	36246160	36246160	http://gnomad.b	1.12E-05	NFE	2.69E-05	0	0	0	https://www.ncbi.nlm.nih.gov/clinvar/variation/290196/
NM_001128227.2(GNE):c.1096C>T	concordant	unreportable	unreportable	281291	0	1	SCV000332005	Uncertain significance	EGL Genetic Diagn	6/30/2017	GNE	chr9	36229085	36229085	http://gnomad.b	2.24E-05	EAS	0.0001159820022	0	0	0	https://www.ncbi.nlm.nih.gov/clinvar/variation/281291/
NM_001128227.2(GNE):c.479G>A	concordant	reportable	reportable	189156	1	0	SCV000638335	Pathogenic	Invitae	10/5/2017	GNE	chr9	36246258	36246258	http://gnomad.b	2.80E-06	NFE	8.96E-06	0	0	0	https://www.ncbi.nlm.nih.gov/clinvar/variation/189156/
NM_001128227.2(GNE):c.1225G>T	concordant	reportable	reportable	283278	2	0	SCV000700931;S(	Pathogenic;Pathogenic	EGL Genetic Diagn	2017-06-30;2017-1	GNE	chr9	36227394	36227394	http://gnomad.b	0.0001708970695	NFE	0.0003492810065	0	0	0	https://www.ncbi.nlm.nih.gov/clinvar/variation/283278/
NM_001128227.2(GNE):c.1864G>A	concordant	unreportable	unreportable	498575	0	1	SCV000703655	Uncertain significance	EGL Genetic Diagn	6/30/2017	GNE	chr9	36219880	36219880	http://gnomad.b	1.40E-05	NFE	1.79E-05	0	0	0	https://www.ncbi.nlm.nih.gov/clinvar/variation/498575/
NM_001128227.2(GNE):c.1768G>A	concordant	unreportable	unreportable	288129	0	1	SCV000342146	Uncertain significance	EGL Genetic Diagn	6/30/2017	GNE	chr9	36219976	36219976	http://gnomad.b	2.24E-05	EAS	0.000173952998	0	0	0	https://www.ncbi.nlm.nih.gov/clinvar/variation/288129/
NM_005476.5(GNE):c.527A>T	concordant	reportable	reportable	41233	2	0	SCV000703658;S(	Pathogenic;Pathogenic	EGL Genetic Diagn	2017-06-30;2016-1	GNE	chr9	36246117	36246117	http://gnomad.b	0.0001204684261	EAS	0.0005797769991	0	0	0	https://www.ncbi.nlm.nih.gov/clinvar/variation/41233/
NM_001128227.2(GNE):c.1985C>T	concordant	reportable	reportable	6035	4	0	SCV000613535;S(	Pathogenic;Pathogenic;Pat	Athena Diagnostics	2017-08-17;2017-0	GNE	chr9	36218221	36218221	http://gnomad.b	0.0001372779739	NFE	0.0001879899937	0	0	0	https://www.ncbi.nlm.nih.gov/clinvar/variation/6035/
NM_005476.5(GNE):c.736C>T	concordant	reportable	reportable	197184	1	0	SCV000700836	Pathogenic	EGL Genetic Diagn	6/30/2017	GNE	chr9	36236862	36236862	http://gnomad.b	1.12E-05	AMR	2.98E-05	0	0	0	https://www.ncbi.nlm.nih.gov/clinvar/variation/197184/
NM_001128227.2(GNE):c.1900C>T	concordant	unreportable	unreportable	283229	0	1	SCV000335207	Uncertain significance	EGL Genetic Diagn	6/30/2017	GNE	chr9	36219844	36219844	http://gnomad.b	8.40E-06	NFE	1.79E-05	0	0	0	https://www.ncbi.nlm.nih.gov/clinvar/variation/283229/
NM_001128227.2(GNE):c.740T>C	concordant	reportable	reportable	218297	3	0	SCV000613536;S(	Pathogenic;Likely pathoger	Athena Diagnostics	2017-08-17;2017-0	GNE	chr9	36236951	36236951	http://gnomad.b	3.64E-05	NFE	5.37E-05	0	0	0	https://www.ncbi.nlm.nih.gov/clinvar/variation/218297/
NM_001128227.2(GNE):c.1937C>G	concordant	reportable	reportable	286439	1	0	SCV000701168	Pathogenic	EGL Genetic Diagn	6/30/2017	GNE	chr9	36218269	36218269	http://gnomad.b	8.40E-06	AFR	6.53E-05	0	0	0	https://www.ncbi.nlm.nih.gov/clinvar/variation/286439/
NM_001128227.2(GNE):c.131G>C	concordant	reportable	reportable	496947	1	0	SCV000700793	Pathogenic	EGL Genetic Diagn	6/30/2017	GNE	chr9	36249315	36249315		1.40E-05				0	0	https://www.ncbi.nlm.nih.gov/clinvar/variation/496947/
NM_001128227.2(GNE):c.1676T>G	concordant	unreportable	unreportable	497075	0	1	SCV000701257	Uncertain significance	EGL Genetic Diagn	6/30/2017	GNE	chr9	36222824	36222824	http://gnomad.b	2.80E-06	NFE	8.95E-06	0	0	0	https://www.ncbi.nlm.nih.gov/clinvar/variation/497075/
NM_012203.1(GRHPR):c.494G>A	concordant	reportable	reportable	204235	1	0	SCV000699330	Pathogenic	Laboratory Corpora	1/25/2018	GRHPR	chr9	37429729	37429729	http://gnomad.b	6.58E-05	SAS	0.000844648981	0	0	0	https://www.ncbi.nlm.nih.gov/clinvar/variation/204235/
NM_000182.4(HADHA):c.914T>A	concordant	reportable	reportable	8736	1	0	SCV000519097	Likely pathogenic	GeneDx	11/28/2017	HADHA	chr2	26437316	26437316	http://gnomad.b	1.40E-05	NFE	1.80E-05	0	0	0	https://www.ncbi.nlm.nih.gov/clinvar/variation/8736/
NM_000182.4(HADHA):c.871C>T	concordant	reportable	reportable	8737	1	0	SCV000695943	Pathogenic	Laboratory Corpora	1/25/2018	HADHA	chr2	26437359	26437359		1.96E-05				0	0	https://www.ncbi.nlm.nih.gov/clinvar/variation/8737/
NM_000518.4(HBB):c.263C>A	concordant	unreportable	unreportable	15150	0	1	SCV000601264	Likely benign	Quest Diagnostics I	8/1/2017	HBB	chr11	5247859	5247859		2.33E-06				0	0	https://www.ncbi.nlm.nih.gov/clinvar/variation/15150/
NM_000518.4(HBB):c.364G>T	concordant	reportable	reportable	15404	1	0	SCV000601299	Pathogenic	Quest Diagnostics I	8/1/2017	HBB	chr11	5246908	5246908		2.33E-06				0	0	https://www.ncbi.nlm.nih.gov/clinvar/variation/15404/
NM_000518.4(HBB):c.208G>A	concordant	unreportable	unreportable	15138	1	1	SCV000333005;S(	Likely pathogenic;Uncertai	EGL Genetic Diagn	2017-06-30;2018-0	HBB	chr11	5247914	5247914	http://gnomad.b	0.001875472367	ASJ	0.01787530072	0	3	0	https://www.ncbi.nlm.nih.gov/clinvar/variation/15138/
NM_000518.4(HBB):c.34G>A	concordant	unreportable	unreportable	15189	0	1	SCV000601294	Uncertain significance	Quest Diagnostics I	8/1/2017	HBB	chr11	5248218	5248218	http://gnomad.b	5.60E-05	AFR	0.0001307019993	0	0	0	https://www.ncbi.nlm.nih.gov/clinvar/variation/15189/
NM_000518.4(HBB):c.44T>C	concordant	unreportable	unreportable	15341	0	1	SCV000605836	Uncertain significance	Quest Diagnostics I	8/1/2017	HBB	chr11	5248208	5248208		7.00E-06				0	0	https://www.ncbi.nlm.nih.gov/clinvar/variation/15341/
NM_000518.4(HBB):c.68A>C	concordant	unreportable	unreportable	15171	0	1	SCV000697143	Likely benign	Laboratory Corpora	1/25/2018	HBB	chr11	5248184	5248184	http://gnomad.b	1.17E-05	SAS	6.50E-05	0	0	0	https://www.ncbi.nlm.nih.gov/clinvar/variation/15171/
NM_000518.4(HBB):c.389C>T	concordant	unreportable	unreportable	15245	0	1	SCV000697128	Uncertain significance	Laboratory Corpora	1/25/2018	HBB	chr11	5246883	5246883	http://gnomad.b	2.57E-05	AFR	0.000326754991	0	0	0	https://www.ncbi.nlm.nih.gov/clinvar/variation/15245/
NM_000518.4(HBB):c.274C>T	concordant	unreportable	unreportable	439144	0	1	SCV000601265	Uncertain significance	Quest Diagnostics I	8/1/2017	HBB	chr11	5247848	5247848	http://gnomad.b	5.60E-05	EAS	0.0003480279993	0	0	0	https://www.ncbi.nlm.nih.gov/clinvar/variation/439144/
NM_000518.4(HBB):c.410G>A	concordant	unreportable	unreportable	15202	0	2	SCV000603900;S(	Likely benign;Likely benign	ARUP Laboratories	2017-06-30;2018-0	HBB	chr11	5246862	5246862	http://gnomad.b	4.67E-06	AFR	0.0001960780064	0	0	0	https://www.ncbi.nlm.nih.gov/clinvar/variation/15202/
NM_000518.4(HBB):c.67G>C	concordant	unreportable	unreportable	15151	0	2	SCV000603917;S(	Likely benign;Uncertain sig	ARUP Laboratories	2017-06-30;2018-0	HBB	chr11	5248185	5248185	http://gnomad.b	1.87E-05	SAS	0.0004873290018	0	0	0	https://www.ncbi.nlm.nih.gov/clinvar/variation/15151/
NM_000518.4(HBB):c.220G>A	concordant	unreportable	unreportable	15244	0	2	SCV000603915;S(	Likely benign;Likely benign	ARUP Laboratories	2017-06-30;2018-0	HBB	chr11	5247902	5247902	http://gnomad.b	1.63E-05	AFR	0.0001306850027	0	0	0	https://www.ncbi.nlm.nih.gov/clinvar/variation/15244/
NM_000520.4(HEXA):c.1495C>T	concordant	reportable	reportable	3915	1	0	SCV000697165	Pathogenic	Laboratory Corpora	1/25/2018	HEXA	chr15	72637818	72637818	http://gnomad.b	4.70E-06			0	0	0	https://www.ncbi.nlm.nih.gov/clinvar/variation/3915/
NM_000520.4(HEXA):c.2T>C	concordant	reportable	reportable	189126	1	0	SCV000629534	Pathogenic	Invitae	10/5/2017	HEXA	chr15	72668312	72668312		2.35E-06				0	0	https://www.ncbi.nlm.nih.gov/clinvar/variation/189126/
NM_000520.4(HEXA):c.409C>T	concordant	reportable	reportable	3904	2	0	SCV000331825;S(	Pathogenic;Pathogenic	EGL Genetic Diagn	2017-06-30;2017-1	HEXA	chr15	72647903	72647903	http://gnomad.b	3.29E-05	NFE	4.48E-05	0	0	0	https://www.ncbi.nlm.nih.gov/clinvar/variation/3904/
NM_000520.4(HEXA):c.1496G>A	concordant	reportable	reportable	3899	2	0	SCV000331828;S(	Pathogenic;Pathogenic	EGL Genetic Diagn	2017-06-30;2017-1	HEXA	chr15	72637817	72637817	http://gnomad.b	5.17E-05	SAS	0.0003248860012	0	0	0	https://www.ncbi.nlm.nih.gov/clinvar/variation/3899/
NM_000520.4(HEXA):c.1177C>T	concordant	reportable	reportable	3905	1	0	SCV000617693	Pathogenic	GeneDx	11/28/2017	HEXA	chr15	72639021	72639021	http://gnomad.b	1.17E-05	AMR	2.98E-05	0	0	0	https://www.ncbi.nlm.nih.gov/clinvar/variation/3905/
NM_000520.4(HEXA):c.1528C>T	concordant	reportable	reportable	188954	1	0	SCV000521178	Pathogenic	GeneDx	11/28/2017	HEXA	chr15	72636480	72636480		9.39E-06				0	0	https://www.ncbi.nlm.nih.gov/clinvar/variation/188954/
NM_000520.4(HEXA):c.1490A>G	concordant	unreportable	unreportable	445397	0	1	SCV000697164	Uncertain significance	Laboratory Corpora	1/25/2018	HEXA	chr15	72637823	72637823	http://gnomad.b	6.57E-05	NFE	8.95E-05	0	0	0	https://www.ncbi.nlm.nih.gov/clinvar/variation/445397/
NM_000411.6(HLCS):c.1693C>T	concordant	reportable	reportable	426486	1	0	SCV000576938	Pathogenic	GeneDx	11/28/2017	HLCS	chr21	38132130	38132130	http://gnomad.b	8.04E-06	AFR	6.53E-05	0	0	0	https://www.ncbi.nlm.nih.gov/clinvar/variation/426486/
NM_000411.6(HLCS):c.1648G>A	concordant	reportable	reportable	1911	1	0	SCV000238919	Pathogenic	GeneDx	11/28/2017	HLCS	chr21	38137345	38137345	http://gnomad.b	1.61E-05	AMR	2.98E-05	0	0	0	https://www.ncbi.nlm.nih.gov/clinvar/variation/1911/
NM_000191.2(HMGCL):c.494G>A	concordant	unreportable	unreportable	501099	0	1	SCV000707331	Uncertain significance	EGL Genetic Diagn	6/30/2017	HMGCL	chr1	24140683	24140683	http://gnomad.b	8.00E-06	AFR	6.54E-05	0	0	0	https://www.ncbi.nlm.nih.gov/clinvar/variation/501099/
NM_000191.2(HMGCL):c.109G>T	concordant	reportable	reportable	195033	1	0	SCV000329363	Pathogenic	GeneDx	11/28/2017	HMGCL	chr1	24147035	24147035	http://gnomad.b	4.00E-05	AMR	0.0002084449952	0	0	0	https://www.ncbi.nlm.nih.gov/clinvar/variation/195033/
NM_000414.3(HSD17B4):c.1516C>T	concordant	reportable	reportable	495866	1	0	SCV000696690	Pathogenic	Laboratory Corpora	1/25/2018	HSD17B4	chr5	118860923	118860923	http://gnomad.b	1.41E-05	AMR	2.99E-05	0	0	0	https://www.ncbi.nlm.nih.gov/clinvar/variation/495866/
NM_000203.3(IDUA):c.1598C>G	concordant	reportable	reportable	11910	2	0	SCV000700446;S(	Pathogenic;Pathogenic	EGL Genetic Diagn	2017-06-30;2017-1	IDUA	chr4	997206	997206	http://gnomad.b	5.55E-05	AMR	0.0001706049952	0	0	0	https://www.ncbi.nlm.nih.gov/clinvar/variation/11910/
NM_000206.2(IL2RG):c.676C>T	concordant	reportable	reportable	225195	2	0	SCV000321776;S(	Pathogenic;Pathogenic	GeneDx;Invitae	2017-11-28;2017-1	IL2RG	chrX	70329159	70329159		9.68E-06				0	0	https://www.ncbi.nlm.nih.gov/clinvar/variation/225195/
NM_002225.3(IVD):c.367G>A	concordant	reportable	reportable	167199	4	0	SCV000230190;S(	Pathogenic;Likely pathoger	EGL Genetic Diagn	2017-06-30;2017-0	IVD	chr15	40702898	40702898	http://gnomad.b	6.16E-05	AFR	0.0001306850027	0	0	0	https://www.ncbi.nlm.nih.gov/clinvar/variation/167199/
NM_000228.2(LAMB3):c.430C>T	concordant	reportable	reportable	488703	1	0	SCV000680541	Pathogenic	GeneDx	2/7/2018	LAMB3	chr1	209807926	209807926	http://gnomad.b	2.80E-06	AMR	2.98E-05	0	0	0	https://www.ncbi.nlm.nih.gov/clinvar/variation/488703/
NM_000228.2(LAMB3):c.1978C>T	concordant	reportable	reportable	189034	1	0	SCV000680543	Pathogenic	GeneDx	2/7/2018	LAMB3	chr1	209797344	209797344	http://gnomad.b	2.52E-05	AFR	0.0001310619991	0	0	0	https://www.ncbi.nlm.nih.gov/clinvar/variation/189034/
NM_000528.3(MAN2B1):c.1383C>G	concordant	reportable	reportable	189068	1	0	SCV000697258	Pathogenic	Laboratory Corpora	1/25/2018	MAN2B1	chr19	12768296	12768296		1.13E-05				0	0	https://www.ncbi.nlm.nih.gov/clinvar/variation/189068/
NM_000528.3(MAN2B1):c.2278C>T	concordant	reportable	reportable	1685	1	0	SCV000697260	Pathogenic	Laboratory Corpora	1/25/2018	MAN2B1	chr19	12760232	12760232		1.13E-05				0	0	https://www.ncbi.nlm.nih.gov/clinvar/variation/1685/
NM_020533.2(MCOLN1):c.304C>T	concordant	reportable	reportable	5136	3	0	SCV000331818;S(	Pathogenic;Pathogenic;Pat	EGL Genetic Diagn	2017-06-30;2017-1	MCOLN1	chr19	7591391	7591391	http://gnomad.b	1.19E-05	AFR	6.54E-05	0	0	0	https://www.ncbi.nlm.nih.gov/clinvar/variation/5136/
NM_000243.2(MEFV):c.775A>G	concordant	unreportable	unreportable	97545	0	1	SCV000696079	Uncertain significance	Laboratory Corpora	1/25/2018	MEFV	chr16	3304293	3304293	http://gnomad.b	6.16E-05	SAS	6.50E-05	0	0	0	https://www.ncbi.nlm.nih.gov/clinvar/variation/97545/
NM_000243.2(MEFV):c.926C>T	concordant	unreportable	unreportable	97554	0	2	SCV000279043;S(	Uncertain significance;Unc	GeneDx;Laborator	2017-11-28;2018-0	MEFV	chr16	3299765	3299765	http://gnomad.b	0.0003302546879	SAS	0.002797479974	1	1	0	https://www.ncbi.nlm.nih.gov/clinvar/variation/97554/
NM_000243.2(MEFV):c.1772T>C	concordant	unreportable	unreportable	36506	0	6	SCV000604178;S(	Uncertain significance;Likel	ARUP Laboratories	2017-06-30;2017-0	MEFV	chr16	3293880	3293880	http://gnomad.b	0.009809683739	FIN	0.0207754001	20	26	0	https://www.ncbi.nlm.nih.gov/clinvar/variation/36506/
NM_000243.2(MEFV):c.848C>G	concordant	unreportable	unreportable	97548	0	1	SCV000279042	Uncertain significance	GeneDx	11/28/2017	MEFV	chr16	3304220	3304220	http://gnomad.b	5.60E-05	NFE	2.69E-05	0	0	0	https://www.ncbi.nlm.nih.gov/clinvar/variation/97548/
NM_000243.2(MEFV):c.124C>T	concordant	unreportable	unreportable	234347	0	1	SCV000279020	Uncertain significance	GeneDx	11/28/2017	MEFV	chr16	3306464	3306464	http://gnomad.b	0.0003582423734	AFR	0.004117109813	0	1	0	https://www.ncbi.nlm.nih.gov/clinvar/variation/234347/
NM_000243.2(MEFV):c.688G>A	concordant	unreportable	unreportable	97537	0	1	SCV000279040	Uncertain significance	GeneDx	11/28/2017	MEFV	chr16	3304380	3304380	http://gnomad.b	0.0002826756227	SAS	0.004678360187	1	0	0	https://www.ncbi.nlm.nih.gov/clinvar/variation/97537/
NM_000243.2(MEFV):c.1958G>A	concordant	reportable	reportable	2553	2	0	SCV000321877;S(	Pathogenic;Pathogenic	GeneDx;Laborator	2017-11-28;2018-0	MEFV	chr16	3293529	3293529	http://gnomad.b	6.16E-05	AFR	0.0001306850027	0	0	0	https://www.ncbi.nlm.nih.gov/clinvar/variation/2553/
NM_000243.2(MEFV):c.2282G>A	concordant	reportable	reportable	2549	5	0	SCV000331538;S(	Pathogenic;Pathogenic;Pat	EGL Genetic Diagn	2017-06-30;2017-1	MEFV	chr16	3293205	3293205	http://gnomad.b	0.0003162608452	EAS	0.001855290029	0	0	0	https://www.ncbi.nlm.nih.gov/clinvar/variation/2549/
NM_000243.2(MEFV):c.1318C>G	concordant	unreportable	unreportable	36499	0	5	SCV000604179;S(	Likely benign;Uncertain sig	ARUP Laboratories	2017-06-30;2017-0	MEFV	chr16	3298947	3298947	http://gnomad.b	0.001080324657	AFR	0.01372190006	1	1	0	https://www.ncbi.nlm.nih.gov/clinvar/variation/36499/
NM_000243.2(MEFV):c.2338C>A	concordant	unreportable	unreportable	97511	0	1	SCV000279570	Uncertain significance	GeneDx	11/28/2017	MEFV	chr16	3293149	3293149	http://gnomad.b	4.48E-05	NFE	6.27E-05	0	0	0	https://www.ncbi.nlm.nih.gov/clinvar/variation/97511/
NM_000243.2(MEFV):c.866C>T	concordant	unreportable	unreportable	97550	0	1	SCV000702665	Uncertain significance	EGL Genetic Diagn	6/30/2017	MEFV	chr16	3304202	3304202	http://gnomad.b	0.0001427371956	NFE	0.0002151390072	0	0	0	https://www.ncbi.nlm.nih.gov/clinvar/variation/97550/
NM_000243.2(MEFV):c.1370C>T	concordant	unreportable	unreportable	97441	0	3	SCV000279049;S(	Uncertain significance;Unc	GeneDx;Invitae;Lal	2017-11-28;2017-1	MEFV	chr16	3297233	3297233	http://gnomad.b	0.0005569549398	ASJ	0.005179769825	0	1	0	https://www.ncbi.nlm.nih.gov/clinvar/variation/97441/
NM_000243.2(MEFV):c.2033G>A	concordant	unreportable	unreportable	97479	0	1	SCV000491138	Uncertain significance	GeneDx	12/21/2016	MEFV	chr16	3293454	3293454	http://gnomad.b	5.60E-05	NFE	8.06E-05	0	0	0	https://www.ncbi.nlm.nih.gov/clinvar/variation/97479/
NM_000243.2(MEFV):c.1508C>G	concordant	unreportable	unreportable	457996	0	1	SCV000629025	Uncertain significance	Invitae	10/5/2017	MEFV	chr16	3297095	3297095	http://gnomad.b	0.0001819199552	EAS	0.0009856220568	0	0	0	https://www.ncbi.nlm.nih.gov/clinvar/variation/457996/
NM_000243.2(MEFV):c.2246C>G	concordant	unreportable	unreportable	97505	0	1	SCV000617280	Uncertain significance	GeneDx	11/28/2017	MEFV	chr16	3293241	3293241	http://gnomad.b	1.96E-05	FIN	0.0008071750053	1	0	0	https://www.ncbi.nlm.nih.gov/clinvar/variation/97505/
NM_000243.2(MEFV):c.1016C>T	concordant	unreportable	unreportable	36497	0	2	SCV000329420;S(	Uncertain significance;Unc	GeneDx;Laborator	2017-11-28;2015-1	MEFV	chr16	3299675	3299675	http://gnomad.b	0.0001903162608	ASJ	0.0006153850118	0	0	0	https://www.ncbi.nlm.nih.gov/clinvar/variation/36497/
NM_000243.2(MEFV):c.1894G>A	concordant	reportable	reportable	97467	1	0	SCV000696055	Likely pathogenic	Laboratory Corpora	1/25/2018	MEFV	chr16	3293593	3293593	http://gnomad.b	8.68E-05	NFE	5.53E-05	0	0	0	https://www.ncbi.nlm.nih.gov/clinvar/variation/97467/
NM_015166.3(MLC1):c.65G>A	concordant	unreportable	unreportable	225412	0	1	SCV000565135	Uncertain significance	GeneDx	11/28/2017	MLC1	chr22	50523267	50523267	http://gnomad.b	0.0005201982347	EAS	0.004588219803	0	1	0	https://www.ncbi.nlm.nih.gov/clinvar/variation/225412/
NM_172250.2(MMAA):c.586C>T	concordant	reportable	reportable	466217	1	0	SCV000641767	Pathogenic	Invitae	10/5/2017	MMAA	chr4	146567161	146567161	http://gnomad.b	8.00E-06	NFE	1.79E-05	0	0	0	https://www.ncbi.nlm.nih.gov/clinvar/variation/466217/
NM_052845.3(MMAB):c.556C>T	concordant	reportable	reportable	3095	2	0	SCV000375743;S(	Pathogenic;Pathogenic	Illumina Clinical Se	2016-10-18;2018-0	MMAB	chr12	109998873	109998873	http://gnomad.b	0.0001839941122	NFE	0.0002063709981	0	0	0	https://www.ncbi.nlm.nih.gov/clinvar/variation/3095/
NM_052845.3(MMAB):c.571C>T	concordant	reportable	reportable	218324	2	0	SCV000331820;S(	Pathogenic;Pathogenic	EGL Genetic Diagn	2017-06-30;2016-1	MMAB	chr12	109998858	109998858	http://gnomad.b	1.60E-05	AFR	6.56E-05	0	0	0	https://www.ncbi.nlm.nih.gov/clinvar/variation/218324/
NM_015506.2(MMACHC):c.440G>C	concordant	reportable	reportable	203828	2	0	SCV000357927;S(	Likely pathogenic;Pathoger	Illumina Clinical Se	2016-10-18;2018-0	MMACHC	chr1	45974478	45974478	http://gnomad.b	0.0003797708716	AMR	0.0006265660049	0	0	0	https://www.ncbi.nlm.nih.gov/clinvar/variation/203828/
NM_015506.2(MMACHC):c.389A>G	concordant	unreportable	unreportable	297484	1	1	SCV000595813;S(	Likely pathogenic;Uncertai	Genetic Services La	2017-07-05;2016-1	MMACHC	chr1	45973996	45973996	http://gnomad.b	0.00015032597	NFE	0.0002241070033	0	0	0	https://www.ncbi.nlm.nih.gov/clinvar/variation/297484/
NM_015506.2(MMACHC):c.666C>A	concordant	reportable	reportable	496436	1	0	SCV000699404	Pathogenic	Laboratory Corpora	1/25/2018	MMACHC	chr1	45974704	45974704	http://gnomad.b	2.37E-05	SAS	6.50E-05	0	0	0	https://www.ncbi.nlm.nih.gov/clinvar/variation/496436/
NM_002435.1(MPI):c.656G>A	concordant	reportable	reportable	14345	1	0	SCV000393945	Pathogenic	Illumina Clinical Se	10/18/2016	MPI	chr15	75185647	75185647	http://gnomad.b	0.0001063698761	NFE	0.0005224660272	0	0	0	https://www.ncbi.nlm.nih.gov/clinvar/variation/14345/
NM_000255.3(MUT):c.160A>T	concordant	reportable	reportable	466219	1	0	SCV000641773	Pathogenic	Invitae	10/5/2017	MUT	chr6	49427020	49427020		7.99E-06				0	0	https://www.ncbi.nlm.nih.gov/clinvar/variation/466219/
NM_000255.3(MUT):c.323G>A	concordant	reportable	reportable	100707	1	0	SCV000595871	Pathogenic	Genetic Services La	7/5/2017	MUT	chr6	49426857	49426857	http://gnomad.b	7.99E-06	AFR	6.53E-05	0	0	0	https://www.ncbi.nlm.nih.gov/clinvar/variation/100707/
NM_000255.3(MUT):c.572C>A	concordant	reportable	reportable	203854	3	0	SCV000239018;S(	Pathogenic;Pathogenic;Pat	GeneDx;Invitae;Lal	2017-11-28;2017-1	MUT	chr6	49425585	49425585	http://gnomad.b	1.60E-05	NFE	8.07E-05	0	0	0	https://www.ncbi.nlm.nih.gov/clinvar/variation/203854/
NM_000255.3(MUT):c.1889G>A	concordant	reportable	reportable	426995	1	0	SCV000577600	Likely pathogenic	GeneDx	11/28/2017	MUT	chr6	49407986	49407986	http://gnomad.b	2.40E-05	NFE	1.79E-05	0	0	0	https://www.ncbi.nlm.nih.gov/clinvar/variation/426995/
NM_000255.3(MUT):c.1867G>A	concordant	reportable	reportable	1884	1	0	SCV000520995	Pathogenic	GeneDx	11/28/2017	MUT	chr6	49408008	49408008	http://gnomad.b	1.60E-05	AFR	6.54E-05	0	0	0	https://www.ncbi.nlm.nih.gov/clinvar/variation/1884/
NM_000255.3(MUT):c.1280G>A	concordant	reportable	reportable	218990	2	0	SCV000330976;S(	Pathogenic;Pathogenic	EGL Genetic Diagn	2017-06-30;2018-0	MUT	chr6	49419231	49419231	http://gnomad.b	7.99E-06	EAS	0.000347986992	0	0	0	https://www.ncbi.nlm.nih.gov/clinvar/variation/218990/
NM_000255.3(MUT):c.205A>G	concordant	unreportable	unreportable	235300	0	2	SCV000331463;S(	Uncertain significance;Likel	EGL Genetic Diagn	2017-06-30;2017-1	MUT	chr6	49426975	49426975	http://gnomad.b	0.002118000607	NFE	0.003491620068	2	1	0	https://www.ncbi.nlm.nih.gov/clinvar/variation/235300/
NM_000255.3(MUT):c.454C>T	concordant	reportable	reportable	426467	1	0	SCV000576917	Pathogenic	GeneDx	11/28/2017	MUT	chr6	49425703	49425703	http://gnomad.b	7.99E-06	SAS	9.76E-05	0	0	0	https://www.ncbi.nlm.nih.gov/clinvar/variation/426467/
NM_000255.3(MUT):c.1399C>T	concordant	reportable	reportable	495778	1	0	SCV000696305	Pathogenic	Laboratory Corpora	1/25/2018	MUT	chr6	49416574	49416574	http://gnomad.b	0.0001358717371	ASJ	0.001320059993	0	0	0	https://www.ncbi.nlm.nih.gov/clinvar/variation/495778/

Supplemental Table 2

NM_000255.3(MUT):c.1718T>C	concordant	reportable	reportable	284235	1	0	SCV000336766	Likely pathogenic	EGL Genetic Diagn	6/30/2017	MUT	chr6	49409643	49409643	http://gnomad.b	1.60E-05	AFR	6.54E-05	0		0	0	https://www.ncbi.nlm.nih.gov/clinvar/variation/284235/
NM_002485.4(NBN):c.595C>T	concordant	unreportable	unreportable	127874	0	3	SCV000611490;S(	Uncertain significance;Unc	Fulgent Genetics,F	2017-05-23;2017-1	NBN	chr8	90983508	90983508	http://gnomad.b	6.41E-05	ASJ	0.0004066689871	0		0	0	https://www.ncbi.nlm.nih.gov/clinvar/variation/127874/
NM_002485.4(NBN):c.797C>T	concordant	unreportable	unreportable	134878	0	2	SCV000261615;S(	Benign;Benign	Invitae;Quest Diag	2017-10-05;2017-0	NBN	chr8	90982691	90982691	http://gnomad.b	0.002479085332	AFR	0.02980390005	9		9	0	https://www.ncbi.nlm.nih.gov/clinvar/variation/134878/
NM_002485.4(NBN):c.664T>C	concordant	unreportable	unreportable	136046	0	3	SCV000292199;S(	Uncertain significance;Unc	Color Genomics, In	2017-12-21;2017-1	NBN	chr8	90983439	90983439	http://gnomad.b	1.95E-05	NFE	2.69E-05	0		0	0	https://www.ncbi.nlm.nih.gov/clinvar/variation/136046/
NM_002485.4(NBN):c.628G>T	concordant	unreportable	unreportable	127014	0	5	SCV000611491;S(	Uncertain significance;Unc	Fulgent Genetics,F	2017-05-23;2017-1	NBN	chr8	90983475	90983475	http://gnomad.b	0.000510317903	NFE	0.0007628240273	0		0	0	https://www.ncbi.nlm.nih.gov/clinvar/variation/127014/
NM_001271208.1(NEB):c.19097G>T	concordant	reportable	reportable	496132	1	0	SCV000697809	Pathogenic	Laboratory Corpora	1/25/2018	NEB	chr2	152417726	152417726	http://gnomad.b	2.58E-06	FIN	0.0005999080022	0		0	0	https://www.ncbi.nlm.nih.gov/clinvar/variation/496132/
NM_000271.4(NPC1):c.3614C>A	concordant	reportable	reportable	188747	2	0	SCV000339052;S(	Likely pathogenic;Pathogen	EGL Genetic Diagn	2017-06-30;2017-1	NPC1	chr18	21113459	21113459	http://gnomad.b	2.00E-05	NFE	8.95E-06	0		0	0	https://www.ncbi.nlm.nih.gov/clinvar/variation/188747/
NM_000271.4(NPC1):c.709C>T	concordant	unreportable	unreportable	21142	0	2	SCV000604562;S(	Benign;Benign	ARUP Laboratories	2017-06-30;2017-0	NPC1	chr18	21140367	21140367	http://gnomad.b	0.009015062273	FIN	0.01803180017	29		27	0	https://www.ncbi.nlm.nih.gov/clinvar/variation/21142/
NM_000271.4(NPC1):c.3019C>G	concordant	reportable	reportable	2966	5	0	SCV000611221;S(	Pathogenic;Pathogenic;Pat	Fulgent Genetics,F	2017-05-23;2017-0	NPC1	chr18	21118528	21118528	http://gnomad.b	0.0001915664993	NFE	0.0002327370021	0		0	0	https://www.ncbi.nlm.nih.gov/clinvar/variation/2966/
NM_000271.4(NPC1):c.1628C>T	concordant	reportable	reportable	181455	2	0	SCV000611220;S(	Pathogenic;Likely pathogen	Fulgent Genetics,F	2017-05-23;2017-1	NPC1	chr18	21131617	21131617	http://gnomad.b	8.58E-06	EAS	0.0001159820022	0		0	0	https://www.ncbi.nlm.nih.gov/clinvar/variation/181455/
NM_000271.4(NPC1):c.3557G>A	concordant	reportable	reportable	189174	2	0	SCV000339684;S(	Likely pathogenic;Pathogen	EGL Genetic Diagn	2017-06-30;2017-1	NPC1	chr18	21114444	21114444	http://gnomad.b	1.43E-05	AFR	0.0001307360071	0		0	0	https://www.ncbi.nlm.nih.gov/clinvar/variation/189174/
NM_000271.4(NPC1):c.1211G>A	concordant	reportable	reportable	188794	3	0	SCV000321923;S(	Pathogenic;Pathogenic;Pat	GeneDx;Genetic Se	2017-11-28;2017-0	NPC1	chr18	21136322	21136322	http://gnomad.b	3.43E-05	AMR	8.93E-05	0		0	0	https://www.ncbi.nlm.nih.gov/clinvar/variation/188794/
NM_000271.4(NPC1):c.2621A>T	concordant	reportable	reportable	194810	2	0	SCV000226730;S(	Pathogenic;Likely pathogen	EGL Genetic Diagn	2017-06-30;2017-0	NPC1	chr18	21119949	21119949	http://gnomad.b	0.0001258048652	NFE	9.85E-05	0		0	0	https://www.ncbi.nlm.nih.gov/clinvar/variation/194810/
NM_000271.4(NPC1):c.2974G>A	concordant	reportable	reportable	21137	1	0	SCV000491260	Pathogenic	GeneDx	12/21/2016	NPC1	chr18	21118573	21118573	http://gnomad.b	1.14E-05	SAS	3.25E-05	0		0	0	https://www.ncbi.nlm.nih.gov/clinvar/variation/21137/
NM_000271.4(NPC1):c.2974G>C	concordant	reportable	reportable	2969	3	0	SCV000331815;S(	Pathogenic;Pathogenic;Pat	EGL Genetic Diagn	2017-06-30;2017-1	NPC1	chr18	21118573	21118573	http://gnomad.b	4.86E-05	SAS	0.0002274059952	0		0	0	https://www.ncbi.nlm.nih.gov/clinvar/variation/2969/
NM_000271.4(NPC1):c.2848G>A	concordant	reportable	reportable	2971	1	0	SCV000208908	Pathogenic	GeneDx	11/28/2017	NPC1	chr18	21119382	21119382	http://gnomad.b	5.72E-06	AFR	6.59E-05	0		0	0	https://www.ncbi.nlm.nih.gov/clinvar/variation/2971/
NM_000271.4(NPC1):c.2861C>T	concordant	reportable	reportable	181457	1	0	SCV000208909	Pathogenic	GeneDx	11/28/2017	NPC1	chr18	21119369	21119369	http://gnomad.b	8.86E-05	FIN	0.0002691789996	0		0	0	https://www.ncbi.nlm.nih.gov/clinvar/variation/181457/
NM_000271.4(NPC1):c.3011C>T	concordant	unreportable	unreportable	326253	0	2	SCV000704662;S(	Uncertain significance;Unc	EGL Genetic Diagn	2017-06-30;2016-1	NPC1	chr18	21118536	21118536	http://gnomad.b	0.0004717682446	FIN	0.00183872995	0		0	0	https://www.ncbi.nlm.nih.gov/clinvar/variation/326253/
NM_000271.4(NPC1):c.3503G>A	concordant	reportable	reportable	502274	1	0	SCV000708945	Likely pathogenic	EGL Genetic Diagn	6/30/2017	NPC1	chr18	21114498	21114498		5.72E-06					0	0	https://www.ncbi.nlm.nih.gov/clinvar/variation/502274/
NM_000271.4(NPC1):c.1561G>T	concordant	unreportable	unreportable	500953	0	1	SCV000707128	Uncertain significance	EGL Genetic Diagn	6/30/2017	NPC1	chr18	21131684	21131684	http://gnomad.b	0.0001887072978	AFR	0.002555370098	0		0	0	https://www.ncbi.nlm.nih.gov/clinvar/variation/500953/
NM_004646.3(NPHS1):c.881C>T	concordant	unreportable	unreportable	259508	0	1	SCV000340194	Benign	EGL Genetic Diagn	6/30/2017	NPHS1	chr19	36340009	36340009	http://gnomad.b	0.008460213303	NFE	0.01316179987	10		11	0	https://www.ncbi.nlm.nih.gov/clinvar/variation/259508/
NM_004646.3(NPHS1):c.2928G>T	concordant	reportable	reportable	188761	1	0	SCV000698505	Pathogenic	Laboratory Corpora	1/25/2018	NPHS1	chr19	36330320	36330320	http://gnomad.b	4.74E-05	AMR	0.0001191260017	0		0	0	https://www.ncbi.nlm.nih.gov/clinvar/variation/188761/
NM_004646.3(NPHS1):c.3478C>T	concordant	reportable	reportable	6873	1	0	SCV000698507	Pathogenic	Laboratory Corpora	1/25/2018	NPHS1	chr19	36321958	36321958	http://gnomad.b	8.92E-05	SAS	0.0003898380091	0		0	0	https://www.ncbi.nlm.nih.gov/clinvar/variation/6873/
NM_004646.3(NPHS1):c.895C>T	concordant	unreportable	unreportable	328874	0	1	SCV000411580	Uncertain significance	Illumina Clinical Se	10/18/2016	NPHS1	chr19	36339995	36339995	http://gnomad.b	1.12E-05	SAS	3.25E-05	0		0	0	https://www.ncbi.nlm.nih.gov/clinvar/variation/328874/
NM_004646.3(NPHS1):c.1868G>T	concordant	reportable	reportable	56453	1	0	SCV000411567	Pathogenic	Illumina Clinical Se	10/18/2016	NPHS1	chr19	36336332	36336332	http://gnomad.b	6.69E-05	NFE	8.08E-05	0		0	0	https://www.ncbi.nlm.nih.gov/clinvar/variation/56453/
NM_014625.2(NPHS2):c.503G>A	concordant	reportable	reportable	188730	1	0	SCV000614350	Pathogenic	Athena Diagnostics	8/17/2017	NPHS2	chr1	179528845	179528845		5.63E-06					0	0	https://www.ncbi.nlm.nih.gov/clinvar/variation/188730/
NM_014625.2(NPHS2):c.538G>A	concordant	reportable	reportable	5368	1	0	SCV000614351	Pathogenic	Athena Diagnostics	8/17/2017	NPHS2	chr1	179526362	179526362	http://gnomad.b	3.66E-05	SAS	3.25E-05	0		0	0	https://www.ncbi.nlm.nih.gov/clinvar/variation/5368/
NM_014625.2(NPHS2):c.779T>A	concordant	reportable	reportable	447882	2	0	SCV000614352;S(	Pathogenic;Pathogenic	Athena Diagnostics	2017-08-17;2018-0	NPHS2	chr1	179523626	179523626	http://gnomad.b	2.53E-05	AFR	0.0002613700053	0		0	0	https://www.ncbi.nlm.nih.gov/clinvar/variation/447882/
NM_000531.5(OTC):c.140A>C	concordant	unreportable	unreportable	97112	0	1	SCV000539982	Uncertain significance	Laboratory for Mol	4/3/2017	OTC	chrX	38226606	38226606	http://gnomad.b	0.000154971185	FIN	0.0003649409919	0	14	0	2	https://www.ncbi.nlm.nih.gov/clinvar/variation/97112/
NM_000277.1(PAH):c.960G>C	concordant	reportable	reportable	102910	1	0	SCV000329865	Pathogenic	GeneDx	11/28/2017	PAH	chr12	103240682	103240682		1.10E-05					0	0	https://www.ncbi.nlm.nih.gov/clinvar/variation/102910/
NM_000277.1(PAH):c.916A>G	concordant	reportable	reportable	618	2	0	SCV000611226;S(	Likely pathogenic;Pathogen	Fulgent Genetics,F	2017-05-23;2018-0	PAH	chr12	103240726	103240726	http://gnomad.b	3.29E-05	NFE	2.69E-05	0		0	0	https://www.ncbi.nlm.nih.gov/clinvar/variation/618/
NM_000277.1(PAH):c.331C>T	concordant	reportable	reportable	581	1	0	SCV000629190	Pathogenic	Invitae	10/5/2017	PAH	chr12	103288534	103288534	http://gnomad.b	3.84E-05	AFR	0.0001960270019	0		0	0	https://www.ncbi.nlm.nih.gov/clinvar/variation/581/
NM_000277.1(PAH):c.472C>T	concordant	reportable	reportable	102693	1	0	SCV000581901	Pathogenic	GeneDx	11/28/2017	PAH	chr12	103260411	103260411	http://gnomad.b	5.48E-06	AFR	0.0001307189959	0		0	0	https://www.ncbi.nlm.nih.gov/clinvar/variation/102693/
NM_000277.1(PAH):c.809G>A	concordant	reportable	reportable	102846	2	0	SCV000629217;S(	Pathogenic;Pathogenic	Invitae;Laboratory	2017-10-05;2018-0	PAH	chr12	103246626	103246626	http://gnomad.b	2.47E-05	AMR	8.94E-05	0		0	0	https://www.ncbi.nlm.nih.gov/clinvar/variation/102846/
NM_000277.1(PAH):c.529G>A	concordant	reportable	reportable	102726	2	0	SCV000629200;S(	Pathogenic;Pathogenic	Invitae;Laboratory	2017-10-05;2018-0	PAH	chr12	103249091	103249091	http://gnomad.b	0.000101365975	AMR	2.98E-05	0		0	0	https://www.ncbi.nlm.nih.gov/clinvar/variation/102726/
NM_000277.1(PAH):c.529G>C	concordant	reportable	reportable	102727	1	0	SCV000490679	Pathogenic	GeneDx	12/21/2016	PAH	chr12	103249091	103249091	http://gnomad.b	2.19E-05	NFE	6.28E-05	0		0	0	https://www.ncbi.nlm.nih.gov/clinvar/variation/102727/
NM_000277.1(PAH):c.847A>T	concordant	reportable	reportable	102876	2	0	SCV000700368;S(	Pathogenic;Pathogenic	EGL Genetic Diagn	2017-06-30;2017-1	PAH	chr12	103245530	103245530	http://gnomad.b	1.37E-05	NFE	2.70E-05	0		0	0	https://www.ncbi.nlm.nih.gov/clinvar/variation/102876/
NM_000277.1(PAH):c.1A>G	concordant	reportable	reportable	586	2	0	SCV000331821;S(	Pathogenic;Pathogenic	EGL Genetic Diagn	2017-06-30;2018-0	PAH	chr12	103310908	103310908	http://gnomad.b	5.48E-06	NFE	1.79E-05	0		0	0	https://www.ncbi.nlm.nih.gov/clinvar/variation/586/
NM_000277.1(PAH):c.490A>G	concordant	unreportable	unreportable	102698	0	1	SCV000629194	Uncertain significance	Invitae	10/5/2017	PAH	chr12	103260393	103260393	http://gnomad.b	1.92E-05	SAS	6.50E-05	0		0	0	https://www.ncbi.nlm.nih.gov/clinvar/variation/102698/
NM_000277.1(PAH):c.727C>T	concordant	reportable	reportable	588	4	0	SCV000490681;S(	Pathogenic;Pathogenic;Pat	GeneDx;Illumina C	2016-12-21;2016-1	PAH	chr12	103246708	103246708	http://gnomad.b	7.94E-05	NFE	8.98E-05	0		0	0	https://www.ncbi.nlm.nih.gov/clinvar/variation/588/
NM_000277.1(PAH):c.781C>T	concordant	reportable	reportable	610	1	0	SCV000581840	Pathogenic	GeneDx	11/28/2017	PAH	chr12	103246654	103246654	http://gnomad.b	2.74E-05	AMR	2.98E-05	0		0	0	https://www.ncbi.nlm.nih.gov/clinvar/variation/610/
NM_000277.1(PAH):c.1042C>G	concordant	reportable	reportable	92727	3	0	SCV000490682;S(	Pathogenic;Pathogenic;Pat	GeneDx;Invitae;Qu	2016-12-21;2017-1	PAH	chr12	103238137	103238137	http://gnomad.b	0.0001424602892	NFE	0.0002150609944	0		0	0	https://www.ncbi.nlm.nih.gov/clinvar/variation/92727/
NM_000277.1(PAH):c.728G>A	concordant	reportable	reportable	591	3	0	SCV000700317;S(	Pathogenic;Pathogenic;Pat	EGL Genetic Diagn	2017-06-30;2017-1	PAH	chr12	103246707	103246707	http://gnomad.b	8.49E-05	EAS	0.000348149013	0		0	0	https://www.ncbi.nlm.nih.gov/clinvar/variation/591/
NM_000277.1(PAH):c.527G>T	concordant	reportable	reportable	631	3	0	SCV000333001;S(	Pathogenic;Pathogenic;Pat	EGL Genetic Diagn	2017-06-30;2017-1	PAH	chr12	103249093	103249093	http://gnomad.b	9.04E-05	AMR	0.0002384499967	0		0	0	https://www.ncbi.nlm.nih.gov/clinvar/variation/631/
NM_000277.1(PAH):c.158G>A	concordant	unreportable	unreportable	102601	0	1	SCV000696438	Uncertain significance	Laboratory Corpora	1/25/2018	PAH	chr12	103306579	103306579	http://gnomad.b	0.002043757226	EAS	0.01414659992	3		6	0	https://www.ncbi.nlm.nih.gov/clinvar/variation/102601/
NM_000277.1(PAH):c.722G>A	concordant	reportable	reportable	102804	3	0	SCV000239067;S(	Pathogenic;Pathogenic;Pat	GeneDx;Laborator	2017-11-28;2018-0	PAH	chr12	103246713	103246713	http://gnomad.b	0.0002712224737	ASJ	0.002846689895	0		0	0	https://www.ncbi.nlm.nih.gov/clinvar/variation/102804/
NM_000277.1(PAH):c.1162G>A	concordant	reportable	reportable	619	2	0	SCV000203178;S(	Pathogenic;Pathogenic	EGL Genetic Diagn	2017-06-30;2017-1	PAH	chr12	103237461	103237461	http://gnomad.b	7.40E-05	AMR	0.0004474410089	0		0	0	https://www.ncbi.nlm.nih.gov/clinvar/variation/619/
NM_000277.1(PAH):c.638T>C	concordant	reportable	reportable	92747	1	0	SCV000239064	Pathogenic	GeneDx	11/28/2017	PAH	chr12	103248982	103248982		1.37E-05					0	0	https://www.ncbi.nlm.nih.gov/clinvar/variation/92747/
NM_000277.1(PAH):c.533A>G	concordant	reportable	reportable	92746	2	0	SCV000110382;S(	Pathogenic;Pathogenic	EGL Genetic Diagn	2017-06-30;2017-1	PAH	chr12	103249087	103249087	http://gnomad.b	7.40E-05	NFE	0.0001255609968	0		0	0	https://www.ncbi.nlm.nih.gov/clinvar/variation/92746/
NM_000277.1(PAH):c.434A>T	concordant	reportable	reportable	102667	3	0	SCV000490677;S(	Pathogenic;Pathogenic;Pat	GeneDx;Invitae;Lal	2016-12-21;2017-1	PAH	chr12	103271247	103271247	http://gnomad.b	8.49E-05	AMR	0.0001191109986	0		0	0	https://www.ncbi.nlm.nih.gov/clinvar/variation/102667/
NM_000277.1(PAH):c.1114A>T	concordant	reportable	reportable	102528	1	0	SCV000375563	Likely pathogenic	Illumina Clinical Se	10/18/2016	PAH	chr12	103237509	103237509	http://gnomad.b	2.74E-06	NFE	8.96E-06	0		0	0	https://www.ncbi.nlm.nih.gov/clinvar/variation/102528/
NM_000277.1(PAH):c.926C>T	concordant	reportable	reportable	92753	2	0	SCV000581842;S(	Pathogenic;Pathogenic	GeneDx;Invitae	2017-06-08;2017-1	PAH	chr12	103240716	103240716	http://gnomad.b	1.92E-05	SAS	3.25E-05	0		0	0	https://www.ncbi.nlm.nih.gov/clinvar/variation/92753/
NM_000277.1(PAH):c.1208C>T	concordant	reportable	reportable	92731	4	0	SCV000110362;S(	Pathogenic;Pathogenic;Pat	EGL Genetic Diagn	2017-06-30;2017-1	PAH	chr12	103234285	103234285	http://gnomad.b	0.00127392374	ASJ	0.00497562997	0		0	0	https://www.ncbi.nlm.nih.gov/clinvar/variation/92731/
NM_000277.1(PAH):c.745C>T	concordant	reportable	reportable	102821	3	0	SCV000239070;S(	Pathogenic;Pathogenic;Pat	GeneDx;Invitae;Lal	2017-11-28;2017-1	PAH	chr12	103246690	103246690	http://gnomad.b	5.75E-05	FIN	0.0001346019999	0		0	0	https://www.ncbi.nlm.nih.gov/clinvar/variation/102821/
NM_000277.1(PAH):c.169G>A	concordant	unreportable	unreportable	92737	0	1	SCV000110371	Uncertain significance	EGL Genetic Diagn	6/30/2017	PAH	chr12	103288696	103288696	http://gnomad.b	5.48E-06	SAS	3.25E-05	0		0	0	https://www.ncbi.nlm.nih.gov/clinvar/variation/92737/
NM_000277.1(PAH):c.716G>T	concordant	unreportable	unreportable	102800	0	1	SCV000203184	Uncertain significance	EGL Genetic Diagn	6/30/2017	PAH	chr12	103246719	103246719		2.74E-06					0	0	https://www.ncbi.nlm.nih.gov/clinvar/variation/102800/
NM_000277.1(PAH):c.829T>G	concordant	reportable	reportable	603	2	0	SCV000239075;S(	Pathogenic;Pathogenic	GeneDx;Quest Diag	2017-11-28;2017-0	PAH	chr12	103246606	103246606	http://gnomad.b	2.19E-05	AMR	2.98E-05	0		0	0	https://www.ncbi.nlm.nih.gov/clinvar/variation/603/
NM_000277.1(PAH):c.734T>C	concordant	reportable	reportable	632	3	0	SCV000331506;S(	Pathogenic;Pathogenic;Pat	EGL Genetic Diagn	2017-06-30;2017-0	PAH	chr12	103246701	103246701	http://gnomad.b	0.0004109431419	NFE	0.0009508940275	0		0	0	https://www.ncbi.nlm.nih.gov/clinvar/variation/632/
NM_000277.1(PAH):c.1068C>A	concordant	reportable	reportable	92729	1	0	SCV000239085	Pathogenic	GeneDx	11/28/2017	PAH	chr12	103237555	103237555	http://gnomad.b	2.47E-05	EAS	0.0001739940053	0		0	0	https://www.ncbi.nlm.nih.gov/clinvar/variation/92729/
NM_000277.1(PAH):c.1068C>G	concordant	reportable	reportable	595	1	0	SCV000629171	Pathogenic	Invitae	10/5/2017	PAH	chr12	103237555	103237555	http://gnomad.b	2.47E-05	NFE	3.59E-05	0		0	0	https://www.ncbi.nlm.nih.gov/clinvar/variation/595/
NM_000277.1(PAH):c.611A>G	concordant	reportable	reportable	590	1	0	SCV000329866	Pathogenic	GeneDx	11/28/2017	PAH	chr12	103249009	103249009	http://gnomad.b	3.84E-05	EAS	0.0001740949956	0		0	0	https://www.ncbi.nlm.nih.gov/clinvar/variation/590/
NM_000277.1(PAH):c.526C>T	concordant	reportable	reportable	102723	2	0	SCV000629197;S(	Pathogenic;Pathogenic	Invitae;Laboratory	2017-10-05;2018-0	PAH	chr12	103249094	103249094	http://gnomad.b	1.64E-05	AFR	6.54E-05	0		0	0	https://www.ncbi.nlm.nih.gov/clinvar/variation/102723/
NM_000277.1(PAH):c.754C>T	concordant	reportable	reportable	584	3	0	SCV000700469;S(	Pathogenic;Pathogenic;Pat	EGL Genetic Diagn	2017-06-30;2017-1	PAH	chr12	103246681	103246681	http://gnomad.b	5.75E-05	EAS	5.80E-05	0		0	0	https://www.ncbi.nlm.nih.gov/clinvar/variation/584/
NM_000277.1(PAH):c.561G>A	concordant	reportable	reportable	102736	1	0	SCV000680747	Pathogenic	GeneDx	2/7/2018	PAH	chr12	103249059	103249059	http://gnomad.b	1.37E-05	NFE	1.79E-05	0		0	0	https://www.ncbi.nlm.nih.gov/clinvar/variation/102736/
NM_000277.1(PAH):c.117C>G	concordant	reportable	reportable	605	5	0	SCV000110360;S(	Pathogenic;Pathogenic;Pat	EGL Genetic Diagn	2017-06-30;2017-1	PAH	chr12	103306620	103306620	http://gnomad.b	0.0001588980149	NFE	0.0001791410032	0		0	0	https://www.ncbi.nlm.nih.gov/clinvar/variation/605/
NM_000277.1(PAH):c.896T>G	concordant	reportable	reportable	613	1	0	SCV000239077	Pathogenic	GeneDx	11/28/2017	PAH	chr12	103245481	103245481	http://gnomad.b	6.03E-05	NFE	0.0001885810052	0		0	0	https://www.ncbi.nlm.nih.gov/clinvar/variation/613/
NM_000277.1(PAH):c.165T>G	concordant	reportable	reportable	92734	2	0	SCV000611224;S(	Likely pathogenic;Pathogen	Fulgent Genetics,F	2017-05-23;2018-0	PAH	chr12	103306572	103306572	http://gnomad.b	0.0001451999101	AMR	0.0009529480012	0		0	0	https://www.ncbi.nlm.nih.gov/clinvar/variation/92734/
NM_033056.3(PCDH15):c.7C>T	concordant	reportable	reportable	4931	1	0	SCV000699764	Pathogenic	Laboratory Corpora	1/25/2018	PCDH15	chr10	56424016	56424016	http://gnomad.b	5.14E-06	SAS	6.57E-05	0		0	0	https://www.ncbi.nlm.nih.gov/clinvar/variation/4931/
NM_033056.3(PCDH15):c.3316C>T	concordant	reportable	reportable	46464	1	0	SCV000611293	Pathogenic	Fulgent Genetics,F	5/23/2017	PCDH15	chr10	55698632	55698632	http://gnomad.b	1.03E-05	NFE	4.49E-05	0		0	0	https://www.ncbi.nlm.nih.gov/clinvar/variation/46464/
NM_000466.2(PEX1):c.2614C>T	concordant	reportable	reportable	265395	1	0	SCV000322246	Pathogenic	GeneDx	11/28/2017	PEX1	chr7	92129122	92129122	http://gnomad.b	1.39E-05	SAS	3.26E-05	0		0	0	https://www.ncbi.nlm.nih.gov/clinvar/variation/265395/
NM_000466.2(PEX1):c.2992C>T	concordant	reportable	reportable	495880	1	0	SCV000696796	Likely pathogenic	Laboratory Corpora	1/25/2018	PEX1	chr7	92123645	92123645	http://gnomad.b	1.39E-05	NFE	1.80E-05	0		0	0	https://www.ncbi.nlm.nih.gov/clinvar/variation/495880/
NM_000288.3(PEX7):c.694C>T	concordant	reportable	reportable	7783	1	0	SCV000322290	Pathogenic	GeneDx	11/28/2017	PEX7	chr6	137191088	137191088	http://gnomad.b	1.11E-05	SAS	3.25E-05	0		0	0	https://www.ncbi.nlm.nih.gov/clinvar/variation/7783/
NM_000288.3(PEX7):c.120C>G	concordant	reportable	reportable	7788	2	0	SCV000460525;S(	Likely pathogenic;Pathogen	Illumina Clinical Se	2016-10-18;2018-0	PEX7	chr6	137143923	137143923	http://gnomad.b	3.89E-05	AMR	0.0004061739892	0		0	0	https://www.ncbi.nlm.nih.gov/clinvar/variation/7788/
NM_138694.3(PKHD1):c.7921A>G	concordant	unreportable	unreportable	96426	0	2	SCV000297372;S(	Likely benign;Benign	Division of Genomi	2016-01-06;2017-1	PKHD1	chr6	51712759	51712759	http://gnomad.b	0.002265676256	AFR	0.0255622007	12		9	0	https://www.ncbi.nlm.nih.gov/clinvar/variation/96426/
NM_138694.3(PKHD1):c.1877A>G	concordant	unreportable	unreportable	188736	0	2	SCV000227727;S(	Benign;Benign	EGL Genetic Diagn	2017-06-30;2017-1	PKHD1	chr6	51918923	51918923	http://gnomad.b	0.003655661689	EAS	0.03509689868	13		17	0	https://www.ncbi.nlm.nih.gov/clinvar/variation/188736/
NM_138694.3(PKHD1):c.9241A>G	concordant	unreportable	unreportable	501331	0	1	SCV000707652	Uncertain significance	EGL Genetic Diagn	6/30/2017	PKHD1	chr6	51613173	51613173	http://gnomad.b	0.0002112777858	AMR	0.0003876890114	0		0	0	https://www.ncbi.nlm.nih.gov/clinvar/variation/501331/
NM_138694.3(PKHD1):c.2341C>T	concordant	reportable	reportable	96387	1	0	SCV000228089	Pathogenic	EGL Genetic Diagn	6/30/2017	PKHD1	chr6	51913356	51913356	http://gnomad.b	4.17E-05	AFR	6.53E-05	0		0	0	https://www.ncbi.nlm.nih.gov/clinvar/variation/96387/
NM_138694.3(PKHD1):c.10666C>T	concordant	unreportable	unreportable	502327	0	1	SCV000709017	Uncertain significance	EGL Genetic Diagn	6/30/2017	PKHD1	chr6	51524258	51524258	http://gnomad.b	2.50E-05	AFR	0.0001308219944	0		0	0	https://www.ncbi.nlm.nih.gov/clinvar/variation/502327/
NM_138694.3(PKHD1):c.9866G>T	concordant	unreportable	unreportable	167475	0	3	SCV000203261;S(	Benign;Likely benign;Benig	EGL Genetic Diagn	2017-06-30;2017-0	PKHD1	chr6	51611651	51611651	http://gnomad.b	0.003005148506	FIN	0.004914780147	3		2	0	https://www.ncbi.nlm.nih.gov/clinvar/variation/167475/
NM_138694.3(PKHD1):c.6992T>A	concordant	reportable	reportable	188813	3	0	SCV000331822;S(	Pathogenic;Pathogenic;Pat	EGL Genetic Diagn	2017-06-30;2016-1	PKHD1	chr6	51768399	51768399	http://gnomad.b	0.0001556783685	FIN	0.00300447992	0		0	0	https://www.ncbi.nlm.nih.gov/clinvar/variation/188813/
NM_138694.3(PKHD1):c.8870T>C	concordant	reportable	reportable	357423	4	0	SCV000617329;S(	Pathogenic;Pathogenic;Pat	GeneDx;Illumina C	2017-11-28;2016-1	PKHD1	chr6	51618079	51618079	http://gnomad.b	9.17E-05	NFE	0.0001166630027	0		0	0	https://www.ncbi.nlm.nih.gov/clinvar/variation/357423/
NM_138694.3(PKHD1):c.8407T>C	concordant	reportable	reportable	96432	1	0	SCV000231334	Likely pathogenic	EGL Genetic Diagn	6/30/2017	PKHD1	chr6	51656067	51656067	http://gnomad.b	6.39E-05	AFR	6.53E-05	0		0	0	https://www.ncbi.nlm.nih.gov/clinvar/variation/96432/
NM_138694.3(PKHD1):c.10926G>A	concordant	unreportable	unreportable	167473	0	2	SCV000203259;S(	Uncertain significance;Unc	EGL Genetic Diagn	2017-06-30;2017-0	PKHD1	chr6	51523998	51523998	http://gnomad.b	0.0008451111432	NFE	0.001266460051	0		0	0	https://www.ncbi.nlm.nih.gov/clinvar/variation/167473/
NM_138694.3(PKHD1):c.664A>G	concordant	reportable	reportable	406891	2	0	SCV000545850;S(	Pathogenic;Pathogenic	Invitae;Laboratory	2017-03-14;2018-0	PKHD1	chr6	51935807	51935807	http://gnomad.b	0.000166798252	ASJ	0.0004069179995	0		0	0	https://www.ncbi.nlm.nih.gov/clinvar/variation/406891/
NM_138694.3(PKHD1):c.2027C>G	concordant	unreportable	unreportable	96383	0	3	SCV000520871;S(	Likely benign;Benign;Likely	GeneDx;Invitae;Lal	2017-11-28;2017-1	PKHD1	chr6	51917987	51917987	http://gnomad.b	0.001954319519	AFR	0.02443809994	4		7	0	https://www.ncbi.nlm.nih.gov/clinvar/variation/96383/
NM_138694.3(PKHD1):c.2264C>T	concordant	reportable	reportable	371292	1	0	SCV000709051	Pathogenic	EGL Genetic Diagn	6/30/2017	PKHD1	chr6	51914970	51914970		5.56E-06					0	0	https://www.ncbi.nlm.nih.gov/clinvar/variation/371292/
NM_138694.3(PKHD1):c.1480C>T	concordant	reportable	reportable	189026	2	0	SCV000338570;S(	Pathogenic;Pathogenic	EGL Genetic Diagn	2017-06-30;2018-0	PKHD1	chr6	51923153	51923153		1.67E-05					0	0	https://www.ncbi.nlm.nih.gov/clinvar/variation/189026/
NM_138694.3(PKHD1):c.274C>T	concordant	unreportable	unreportable	286684	0	1	SCV000340220	Uncertain significance	EGL Genetic Diagn	6/30/2017	PKHD1	chr6	51947197	51947197	http://gnomad.b	1.11E-05	AFR	6.53E-05	0		0	0	https://www.ncbi.nlm.nih.gov/clinvar/variation/286684/
NM_138694.3(PKHD1):c.4870C>T	concordant	reportable	reportable	188369	5	0	SCV000229571;S(	Pathogenic;Pathogenic;Pat	EGL Genetic Diagn	2017-06-30;2017-0	PKHD1	chr6	51889738	51889738	http://gnomad.b	0.0001334386016	NFE	0.0002418179938	0		0	0	https://www.ncbi.nlm.nih.gov/clinvar/variation/188369/
NM_138694.3(PKHD1):c.334G>A	concordant	unreportable	unreportable	499278	0	1	SCV000704700	Uncertain significance	EGL Genetic Diagn	6/30/2017	PKHD1	chr6	51944754	51944754	http://gnomad.b	0.0004531352511	NFE	0.0006629279815	0		0	0	https://www.ncbi.nlm.nih.gov/clinvar/variation/499278/
NM_138694.3(PKHD1):c.3367G>A	concordant	reportable	reportable	188959	1	0	SCV000629914	Likely pathogenic	Invitae	10/5/2017	PKHD1	chr6	51893147	51893147	http://gnomad.b	1.67E-05	NFE	1.79E-05	0		0	0	https://www.ncbi.nlm.nih.gov/clinvar/variation/188959/
NM_138694.3(PKHD1):c.8068T>C	concordant	reportable	reportable	357427	2	0	SCV000617330;S(	Likely pathogenic;Likely pat	GeneDx;Illumina C	2017-11-28;2016-1	PKHD1	chr6	51712612	51712612	http://gnomad.b	2.78E-05	AFR	6.54E-05	0		0	0	https://www.ncbi.nlm.nih.gov/clinvar/variation/357427/
NM_138694.3(PKHD1):c.10036T>C	concordant	unreportable	unreportable	198306	0	2	SCV000231850;S(	Uncertain significance;Unc	EGL Genetic Diagn	2017-06-30;2018-0	PKHD1	chr6	51609303	51609303	http://gnomad.b	0.0003697361252	ASJ	0.001117200009	1		0	0	https://www.ncbi.nlm.nih.gov/clinvar/variation/198306/
NM_138694.3(PKHD1):c.9215C>T	concordant	unreportable	unreportable	96440	0	1	SCV000114632	Benign	EGL Genetic Diagn	6/30/2017	PKHD1	chr6	51613199	51613199	http://gnomad.b	0.002830010341	ASJ	0.01301340014	2		5	0	https://www.ncbi.nlm.nih.gov/clinvar/variation/96440/
NM_138694.3(PKHD1):c.10444C>T	concordant	reportable	reportable	188896	1	0	SCV000344513	Pathogenic	EGL Genetic Diagn	6/30/2017	PKHD1	chr6	51524480	51524480	http://gnomad.b	3.61E-05	SAS	0.0002274199942	0		0	0	https://www.ncbi.nlm.nih.gov/clinvar/variation/188896/
NM_138694.3(PKHD1):c.5410C>T	concordant	unreportable	unreportable	501154	0	1	SCV000707407	Uncertain significance	EGL Genetic Diagn	6/30/2017	PKHD1	chr6	51882398	51882398	http://gnomad.b	0.000227957611	SAS	0.0007797779981	0		0	0	https://www.ncbi.nlm.nih.gov/clinvar/variation/501154/
NM_138694.3(PKHD1):c.652G>A	concordant	unreportable	unreportable	199185	0	1	SCV000233182	Uncertain significance	EGL Genetic Diagn	6/30/2017	PKHD1	chr6	51935819	51935819	http://gnomad.b	0.0008034115802	AFR	0.005298269913	1		1	0	https://www.ncbi.nlm.nih.gov/clinvar/variation/199185/

Supplemental Table 2

NM_138694.3(PKHD1):c.1342G>C	concordant	unreportable	unreportable	374479	0	1	SCV000704763	Uncertain significance	EGL Genetic Diagn	6/30/2017	PKHD1	chr6	51923291	51923291	http://gnomad.b	0.001398325346	NFE	0.002603749977	0		0	0	https://www.ncbi.nlm.nih.gov/clinvar/variation/374479/
NM_138694.3(PKHD1):c.5498C>T	concordant	unreportable	unreportable	496898	0	1	SCV000700545	Uncertain significance	EGL Genetic Diagn	6/30/2017	PKHD1	chr6	51882310	51882310	http://gnomad.b	0.0001473384559	NFE	0.0003142619971	0		0	0	https://www.ncbi.nlm.nih.gov/clinvar/variation/496898/
NM_138694.3(PKHD1):c.7717C>T	concordant	unreportable	unreportable	499806	0	1	SCV000705484	Uncertain significance	EGL Genetic Diagn	6/30/2017	PKHD1	chr6	51732677	51732677	http://gnomad.b	1.39E-05	EAS	5.81E-05	0		0	0	https://www.ncbi.nlm.nih.gov/clinvar/variation/499806/
NM_138694.3(PKHD1):c.11612G>A	concordant	reportable	reportable	458588	1	0	SCV000629902	Pathogenic	Invitae	10/5/2017	PKHD1	chr6	51497416	51497416		2.78E-06					0	0	https://www.ncbi.nlm.nih.gov/clinvar/variation/458588/
NM_000303.2(PMM2):c.710C>G	concordant	reportable	reportable	7716	2	0	SCV000399682;S	Pathogenic;Pathogenic	Illumina Clinical Se	2016-10-18;2018-0	PMM2	chr16	8941651	8941651	http://gnomad.b	0.0002227283104	NFE	0.0002866300056	0		0	0	https://www.ncbi.nlm.nih.gov/clinvar/variation/7716/
NM_000303.2(PMM2):c.385G>A	concordant	reportable	reportable	7708	3	0	SCV000331816;S	Likely pathogenic;Pathogen	EGL Genetic Diagn	2017-06-30;2017-1	PMM2	chr16	8904973	8904973		1.39E-05					0	0	https://www.ncbi.nlm.nih.gov/clinvar/variation/7708/
NM_000303.2(PMM2):c.200T>G	concordant	unreportable	unreportable	285370	0	1	SCV000338360	Uncertain significance	EGL Genetic Diagn	6/30/2017	PMM2	chr16	8898645	8898645	http://gnomad.b	3.62E-05	NFE	4.48E-05	0		0	0	https://www.ncbi.nlm.nih.gov/clinvar/variation/285370/
NM_000303.2(PMM2):c.430T>C	concordant	reportable	reportable	197658	2	0	SCV000700607;S	Likely pathogenic;Likely pat	EGL Genetic Diagn	2017-06-30;2016-1	PMM2	chr16	8905018	8905018	http://gnomad.b	0.0001280687785	EAS	0.001930500031	0		0	0	https://www.ncbi.nlm.nih.gov/clinvar/variation/197658/
NM_000303.2(PMM2):c.722G>C	concordant	reportable	reportable	7717	2	0	SCV000633728;S	Pathogenic;Pathogenic	Invitae;Laboratory	2017-10-05;2018-0	PMM2	chr16	8941663	8941663	http://gnomad.b	7.24E-05	AFR	0.0005892370245	0		0	0	https://www.ncbi.nlm.nih.gov/clinvar/variation/7717/
NM_000303.2(PMM2):c.470T>C	concordant	reportable	reportable	188763	3	0	SCV000568193;S	Pathogenic;Pathogenic;Pat	GeneDx;Illumina C	2017-11-28;2016-1	PMM2	chr16	8905517	8905517	http://gnomad.b	0.0002589216609	NFE	0.0005554059753	0		0	0	https://www.ncbi.nlm.nih.gov/clinvar/variation/188763/
NM_017739.3(POMGNT1):c.643C>T	concordant	reportable	reportable	56604	1	0	SCV000680688	Pathogenic	GeneDx	2/7/2018	POMGNT1	chr1	46660525	46660525	http://gnomad.b	2.52E-05	AMR	5.96E-05	0		0	0	https://www.ncbi.nlm.nih.gov/clinvar/variation/56604/
NM_017739.3(POMGNT1):c.931C>T	concordant	reportable	reportable	56610	1	0	SCV000617417	Pathogenic	GeneDx	11/28/2017	POMGNT1	chr1	46659546	46659546		5.60E-06					0	0	https://www.ncbi.nlm.nih.gov/clinvar/variation/56610/
NM_017739.3(POMGNT1):c.1324C>T	concordant	reportable	reportable	3992	2	0	SCV000331827;S	Pathogenic;Pathogenic	EGL Genetic Diagn	2017-06-30;2017-1	POMGNT1	chr1	46658069	46658069		1.96E-05					0	0	https://www.ncbi.nlm.nih.gov/clinvar/variation/3992/
NM_017739.3(POMGNT1):c.1666G>A	concordant	unreportable	unreportable	3997	0	4	SCV000614740;S	Likely benign;Benign;Benig	Athena Diagnostics	2017-08-17;2017-1	POMGNT1	chr1	46655645	46655645	http://gnomad.b	0.0112234671	ASJ	0.03152329847	15		25	0	https://www.ncbi.nlm.nih.gov/clinvar/variation/3997/
NM_000310.3(PPT1):c.3G>A	concordant	reportable	reportable	56193	1	0	SCV000696512	Likely pathogenic	Laboratory Corpora	1/25/2018	PPT1	chr1	40562908	40562908		1.11E-05					0	0	https://www.ncbi.nlm.nih.gov/clinvar/variation/56193/
NM_000330.3(RS1):c.590G>A	concordant	reportable	reportable	98997	1	0	SCV000589355	Pathogenic	GeneDx	11/28/2017	RS1	chrX	18660209	18660209	http://gnomad.b	3.70E-06	FIN	6.08E-05	0	0	0	0	https://www.ncbi.nlm.nih.gov/clinvar/variation/98997/
NM_000330.3(RS1):c.305G>A	concordant	reportable	reportable	9896	1	0	SCV000490780	Pathogenic	GeneDx	12/21/2016	RS1	chrX	18665332	18665332		7.39E-06					0	1	https://www.ncbi.nlm.nih.gov/clinvar/variation/9896/
NM_000330.3(RS1):c.208G>A	concordant	reportable	reportable	372496	1	0	SCV000490779	Pathogenic	GeneDx	12/21/2016	RS1	chrX	18665429	18665429		3.70E-06					0	0	https://www.ncbi.nlm.nih.gov/clinvar/variation/372496/
NM_014363.4(SACS):c.8393C>A	concordant	unreportable	unreportable	212115	0	3	SCV000614988;S	Uncertain significance;Unc	Athena Diagnostics	2017-08-17;2017-0	SACS	chr13	23909622	23909622	http://gnomad.b	0.001781395239	NFE	0.003035160014	0		1	0	https://www.ncbi.nlm.nih.gov/clinvar/variation/212115/
NM_014363.4(SACS):c.2182C>T	concordant	reportable	reportable	280095	1	0	SCV000329912	Pathogenic	GeneDx	11/28/2017	SACS	chr13	23927927	23927927	http://gnomad.b	2.07E-05	ASJ	0.000101812002	0		0	0	https://www.ncbi.nlm.nih.gov/clinvar/variation/280095/
NM_014363.4(SACS):c.11374C>T	concordant	reportable	reportable	370283	1	0	SCV000699370	Pathogenic	Laboratory Corpora	1/25/2018	SACS	chr13	23906641	23906641	http://gnomad.b	2.96E-06	SAS	3.25E-05	0		0	0	https://www.ncbi.nlm.nih.gov/clinvar/variation/370283/
NM_000023.2(SGCA):c.371T>C	concordant	reportable	reportable	188733	1	0	SCV000229992	Pathogenic	EGL Genetic Diagn	6/30/2017	SGCA	chr17	48245366	48245366	http://gnomad.b	3.92E-05	AMR	5.96E-05	0		0	0	https://www.ncbi.nlm.nih.gov/clinvar/variation/188733/
NM_000023.2(SGCA):c.89C>T	concordant	unreportable	unreportable	285929	0	1	SCV000339153	Uncertain significance	EGL Genetic Diagn	6/30/2017	SGCA	chr17	48244780	48244780	http://gnomad.b	8.40E-06	NFE	1.79E-05	0		0	0	https://www.ncbi.nlm.nih.gov/clinvar/variation/285929/
NM_000023.2(SGCA):c.100C>T	concordant	unreportable	unreportable	217250	1	1	SCV000332844;S	Uncertain significance;Likel	EGL Genetic Diagn	2017-06-30;2017-1	SGCA	chr17	48244791	48244791	http://gnomad.b	2.80E-05	NFE	8.95E-06	0		0	0	https://www.ncbi.nlm.nih.gov/clinvar/variation/217250/
NM_000023.2(SGCA):c.850C>T	concordant	reportable	reportable	9439	4	0	SCV000231990;S	Pathogenic;Pathogenic;Pat	EGL Genetic Diagn	2017-06-30;2017-1	SGCA	chr17	48247606	48247606	http://gnomad.b	0.000209893542	ASJ	0.0007106600096	0		0	0	https://www.ncbi.nlm.nih.gov/clinvar/variation/9439/
NM_000023.2(SGCA):c.614C>A	concordant	unreportable	unreportable	289794	1	1	SCV000344211;S	Uncertain significance;Likel	EGL Genetic Diagn	2017-06-30;2017-1	SGCA	chr17	48246482	48246482	http://gnomad.b	1.12E-05	AFR	6.53E-05	0		0	0	https://www.ncbi.nlm.nih.gov/clinvar/variation/289794/
NM_000023.2(SGCA):c.409G>A	concordant	reportable	reportable	286049	2	0	SCV000339307;S	Pathogenic;Likely pathogen	EGL Genetic Diagn	2017-06-30;2017-1	SGCA	chr17	48245758	48245758		5.32E-05					0	0	https://www.ncbi.nlm.nih.gov/clinvar/variation/286049/
NM_000023.2(SGCA):c.662G>A	concordant	unreportable	unreportable	254721	0	1	SCV000331580	Likely benign	EGL Genetic Diagn	6/30/2017	SGCA	chr17	48246530	48246530	http://gnomad.b	0.0008703585541	EAS	0.007189239841	2		1	0	https://www.ncbi.nlm.nih.gov/clinvar/variation/254721/
NM_000023.2(SGCA):c.101G>A	concordant	reportable	reportable	92301	2	0	SCV000226997;S	Pathogenic;Pathogenic	EGL Genetic Diagn	2017-06-30;2017-1	SGCA	chr17	48244792	48244792	http://gnomad.b	2.52E-05	NFE	6.27E-05	0		0	0	https://www.ncbi.nlm.nih.gov/clinvar/variation/92301/
NM_000023.2(SGCA):c.293G>A	concordant	reportable	reportable	9435	2	0	SCV000337400;S	Pathogenic;Pathogenic	EGL Genetic Diagn	2017-06-30;2017-1	SGCA	chr17	48245078	48245078	http://gnomad.b	3.36E-05	NFE	4.59E-05	0		0	0	https://www.ncbi.nlm.nih.gov/clinvar/variation/9435/
NM_000023.2(SGCA):c.421C>A	concordant	unreportable	unreportable	197618	0	3	SCV000230831;S	Uncertain significance;Unc	EGL Genetic Diagn	2017-06-30;2017-1	SGCA	chr17	48245770	48245770	http://gnomad.b	0.0008311784263	AMR	0.001053699991	0		0	0	https://www.ncbi.nlm.nih.gov/clinvar/variation/197618/
NM_000023.2(SGCA):c.241C>T	concordant	unreportable	unreportable	92302	0	1	SCV000109767	Uncertain significance	EGL Genetic Diagn	6/30/2017	SGCA	chr17	48245026	48245026	http://gnomad.b	2.80E-06	SAS	3.25E-05	0		0	0	https://www.ncbi.nlm.nih.gov/clinvar/variation/92302/
NM_000023.2(SGCA):c.292C>T	concordant	reportable	reportable	284708	2	0	SCV000337437;S	Likely pathogenic;Likely pat	EGL Genetic Diagn	2017-06-30;2017-1	SGCA	chr17	48245077	48245077	http://gnomad.b	3.08E-05	AFR	0.0004661690036	0		0	0	https://www.ncbi.nlm.nih.gov/clinvar/variation/284708/
NM_000232.4(SGCB):c.452C>G	concordant	reportable	reportable	8712	1	0	SCV000701300	Pathogenic	EGL Genetic Diagn	6/30/2017	SGCB	chr4	52895065	52895065		5.60E-06					0	0	https://www.ncbi.nlm.nih.gov/clinvar/variation/8712/
NM_000232.4(SGCB):c.31C>G	concordant	unreportable	unreportable	282248	0	3	SCV000333606;S	Uncertain significance;Unc	EGL Genetic Diagn	2017-06-30;2016-1	SGCB	chr4	52904395	52904395	http://gnomad.b	0.0001063526093	SAS	0.001017180039	0		0	0	https://www.ncbi.nlm.nih.gov/clinvar/variation/282248/
NM_000232.4(SGCB):c.31C>T	concordant	reportable	reportable	284502	2	0	SCV000337151;S	Pathogenic;Likely pathogen	EGL Genetic Diagn	2017-06-30;2016-1	SGCB	chr4	52904395	52904395		1.40E-05					0	0	https://www.ncbi.nlm.nih.gov/clinvar/variation/284502/
NM_000232.4(SGCB):c.355A>T	concordant	unreportable	unreportable	282249	0	2	SCV000333607;S	Uncertain significance;Unc	EGL Genetic Diagn	2017-06-30;2017-1	SGCB	chr4	52895918	52895918	http://gnomad.b	5.60E-06	SAS	0.0001299459982	0		0	0	https://www.ncbi.nlm.nih.gov/clinvar/variation/282249/
NM_000232.4(SGCB):c.271C>T	concordant	reportable	reportable	499193	1	0	SCV000704557	Likely pathogenic	EGL Genetic Diagn	6/30/2017	SGCB	chr4	52896002	52896002	http://gnomad.b	3.36E-05	SAS	9.75E-05	0		0	0	https://www.ncbi.nlm.nih.gov/clinvar/variation/499193/
NM_012434.4(SLC17A5):c.918T>G	concordant	reportable	reportable	440272	1	0	SCV000605124	Pathogenic	ARUP Laboratories	6/30/2017	SLC17A5	chr6	74331587	74331587	http://gnomad.b	8.60E-05	AMR	0.0004467479885	0		0	0	https://www.ncbi.nlm.nih.gov/clinvar/variation/440272/
NM_003060.3(SLC22A5):c.95A>G	concordant	reportable	reportable	25355	4	0	SCV000605132;S	Pathogenic;Pathogenic;Pat	ARUP Laboratories	2017-06-30;2017-0	SLC22A5	chr5	131705759	131705759	http://gnomad.b	1.98E-05	SAS	9.76E-05	0		0	0	https://www.ncbi.nlm.nih.gov/clinvar/variation/25355/
NM_003060.3(SLC22A5):c.791C>G	concordant	unreportable	unreportable	460416	0	1	SCV000632571	Uncertain significance	Invitae	10/5/2017	SLC22A5	chr5	131721158	131721158	http://gnomad.b	2.26E-05	NFE	8.06E-05	0		0	0	https://www.ncbi.nlm.nih.gov/clinvar/variation/460416/
NM_003060.3(SLC22A5):c.695C>T	concordant	reportable	reportable	25386	4	0	SCV000111953;S	Pathogenic;Pathogenic;Pat	EGL Genetic Diagn	2017-06-30;2017-1	SLC22A5	chr5	131721062	131721062	http://gnomad.b	0.0001129675444	AMR	0.0001488889975	0		0	0	https://www.ncbi.nlm.nih.gov/clinvar/variation/25386/
NM_003060.3(SLC22A5):c.865C>T	concordant	reportable	reportable	25404	1	0	SCV000632575	Pathogenic	Invitae	10/5/2017	SLC22A5	chr5	131722757	131722757	http://gnomad.b	8.47E-06	AMR	5.96E-05	0		0	0	https://www.ncbi.nlm.nih.gov/clinvar/variation/25404/
NM_003060.3(SLC22A5):c.904A>G	concordant	unreportable	unreportable	460419	0	1	SCV000632577	Likely benign	Invitae	10/5/2017	SLC22A5	chr5	131722796	131722796	http://gnomad.b	0.0006156731171	EAS	0.006493510213	1		0	0	https://www.ncbi.nlm.nih.gov/clinvar/variation/460419/
NM_003060.3(SLC22A5):c.1193C>T	concordant	reportable	reportable	25411	1	0	SCV000111942	Pathogenic	EGL Genetic Diagn	6/30/2017	SLC22A5	chr5	131726522	131726522	http://gnomad.b	9.04E-05	AFR	0.0002613700053	0		0	0	https://www.ncbi.nlm.nih.gov/clinvar/variation/25411/
NM_003060.3(SLC22A5):c.136C>T	concordant	reportable	reportable	193250	5	0	SCV000605131;S	Pathogenic;Pathogenic;Pat	ARUP Laboratories	2017-06-30;2017-0	SLC22A5	chr5	131705800	131705800	http://gnomad.b	0.0005196507044	NFE	0.0008516940288	0		0	0	https://www.ncbi.nlm.nih.gov/clinvar/variation/193250/
NM_003060.3(SLC22A5):c.1462C>T	concordant	unreportable	unreportable	460399	0	1	SCV000632534	Uncertain significance	Invitae	10/5/2017	SLC22A5	chr5	131729379	131729379	http://gnomad.b	5.65E-05	AMR	0.0001191260017	0		0	0	https://www.ncbi.nlm.nih.gov/clinvar/variation/460399/
NM_003060.3(SLC22A5):c.505C>T	concordant	reportable	reportable	6422	1	0	SCV000632554	Pathogenic	Invitae	10/5/2017	SLC22A5	chr5	131719846	131719846	http://gnomad.b	1.41E-05	NFE	8.95E-06	0		0	0	https://www.ncbi.nlm.nih.gov/clinvar/variation/6422/
NM_003060.3(SLC22A5):c.248G>T	concordant	reportable	reportable	25361	4	0	SCV000111947;S	Pathogenic;Pathogenic;Pat	EGL Genetic Diagn	2017-06-30;2017-1	SLC22A5	chr5	131705912	131705912	http://gnomad.b	0.0002061657686	SAS	0.001865540049	0		2	0	https://www.ncbi.nlm.nih.gov/clinvar/variation/25361/
NM_003060.3(SLC22A5):c.506G>A	concordant	reportable	reportable	6421	1	0	SCV000239159	Pathogenic	GeneDx	11/28/2017	SLC22A5	chr5	131719847	131719847	http://gnomad.b	3.11E-05	EAS	0.0001739330037	0		0	0	https://www.ncbi.nlm.nih.gov/clinvar/variation/6421/
NM_003060.3(SLC22A5):c.641C>T	concordant	unreportable	unreportable	38279	1	1	SCV000331244;S	Uncertain significance;Likel	EGL Genetic Diagn	2017-06-30;2016-0	SLC22A5	chr5	131719982	131719982	http://gnomad.b	0.0006213214943	SAS	0.005847570021	3		1	0	https://www.ncbi.nlm.nih.gov/clinvar/variation/38279/
NM_003060.3(SLC22A5):c.424G>T	concordant	reportable	reportable	25372	4	0	SCV000605126;S	Pathogenic;Pathogenic;Pat	ARUP Laboratories	2017-06-30;2017-0	SLC22A5	chr5	131714100	131714100	http://gnomad.b	0.0001044949786	NFE	9.85E-05	0		0	0	https://www.ncbi.nlm.nih.gov/clinvar/variation/25372/
NM_003060.3(SLC22A5):c.43G>T	concordant	reportable	reportable	6429	3	0	SCV000239176;S	Pathogenic;Pathogenic;Pat	GeneDx;Invitae;Lal	2017-11-28;2017-1	SLC22A5	chr5	131705707	131705707	http://gnomad.b	6.78E-05	SAS	0.000813007995	0		0	0	https://www.ncbi.nlm.nih.gov/clinvar/variation/6429/
NM_003060.3(SLC22A5):c.1345T>G	concordant	unreportable	unreportable	25420	1	3	SCV000111944;S	Uncertain significance;Path	EGL Genetic Diagn	2017-06-30;2017-1	SLC22A5	chr5	131728202	131728202	http://gnomad.b	0.0003417268219	AFR	0.003789860057	0		0	0	https://www.ncbi.nlm.nih.gov/clinvar/variation/25420/
NM_003060.3(SLC22A5):c.632A>G	concordant	reportable	reportable	6420	2	0	SCV000339309;S	Likely pathogenic;Likely pat	EGL Genetic Diagn	2017-06-30;2017-1	SLC22A5	chr5	131719973	131719973	http://gnomad.b	1.98E-05	SAS	3.25E-05	0		0	0	https://www.ncbi.nlm.nih.gov/clinvar/variation/6420/
NM_003060.3(SLC22A5):c.1085C>T	concordant	unreportable	unreportable	281066	0	1	SCV000331201	Uncertain significance	EGL Genetic Diagn	6/30/2017	SLC22A5	chr5	131726414	131726414		8.47E-06					0	0	https://www.ncbi.nlm.nih.gov/clinvar/variation/281066/
NM_000441.1(SLC26A4):c.2162C>T	concordant	reportable	reportable	4826	1	0	SCV000204013	Pathogenic	Laboratory for Mol	4/26/2016	SLC26A4	chr7	107350571	107350571	http://gnomad.b	3.63E-05	AMR	0.0002982229926	0		0	0	https://www.ncbi.nlm.nih.gov/clinvar/variation/4826/
NM_000441.1(SLC26A4):c.200C>G	concordant	unreportable	unreportable	43531	0	2	SCV000521212;S	Likely benign;Likely benign	GeneDx;Laboratory	2017-11-28;2016-0	SLC26A4	chr7	107303776	107303776	http://gnomad.b	4.75E-05	EAS	0.0001159550011	0		0	0	https://www.ncbi.nlm.nih.gov/clinvar/variation/43531/
NM_000441.1(SLC26A4):c.554G>C	concordant	reportable	reportable	188878	1	0	SCV000271454	Pathogenic	Laboratory for Mol	4/26/2016	SLC26A4	chr7	107314747	107314747	http://gnomad.b	5.30E-05	NFE	0.0001611860062	0		0	0	https://www.ncbi.nlm.nih.gov/clinvar/variation/188878/
NM_000441.1(SLC26A4):c.84C>A	concordant	reportable	reportable	188998	1	0	SCV000605155	Pathogenic	ARUP Laboratories	6/30/2017	SLC26A4	chr7	107302170	107302170		1.12E-05					0	0	https://www.ncbi.nlm.nih.gov/clinvar/variation/188998/
NM_000441.1(SLC26A4):c.441G>A	concordant	unreportable	unreportable	229258	0	1	SCV000272441	Uncertain significance	Laboratory for Mol	4/26/2016	SLC26A4	chr7	107314634	107314634	http://gnomad.b	0.0001396031919	FIN	0.005381170195	1		0	0	https://www.ncbi.nlm.nih.gov/clinvar/variation/229258/
NM_000441.1(SLC26A4):c.3G>C	concordant	reportable	reportable	188721	1	0	SCV000680757	Pathogenic	GeneDx	2/7/2018	SLC26A4	chr7	107302089	107302089		8.38E-06					0	0	https://www.ncbi.nlm.nih.gov/clinvar/variation/188721/
NM_000441.1(SLC26A4):c.1541A>G	concordant	reportable	reportable	43511	2	0	SCV000605151;S	Pathogenic;Pathogenic	ARUP Laboratories	2017-06-30;2017-1	SLC26A4	chr7	107336481	107336481	http://gnomad.b	3.35E-05	AFR	0.0001307189959	0		0	0	https://www.ncbi.nlm.nih.gov/clinvar/variation/43511/
NM_000441.1(SLC26A4):c.85G>C	concordant	reportable	reportable	4839	1	0	SCV000060164	Pathogenic	Laboratory for Mol	4/26/2016	SLC26A4	chr7	107302171	107302171	http://gnomad.b	0.0001144746173	FIN	0.000200480994	0		0	0	https://www.ncbi.nlm.nih.gov/clinvar/variation/4839/
NM_000441.1(SLC26A4):c.1489G>A	concordant	reportable	reportable	43510	1	0	SCV000577405	Pathogenic	GeneDx	11/28/2017	SLC26A4	chr7	107336429	107336429	http://gnomad.b	3.07E-05	EAS	5.80E-05	0		0	0	https://www.ncbi.nlm.nih.gov/clinvar/variation/43510/
NM_000441.1(SLC26A4):c.841G>A	concordant	unreportable	unreportable	179723	0	1	SCV000466084	Uncertain significance	Illumina Clinical Se	10/18/2016	SLC26A4	chr7	107323722	107323722	http://gnomad.b	0.0001228508089	AMR	0.001311479951	0		0	0	https://www.ncbi.nlm.nih.gov/clinvar/variation/179723/
NM_000441.1(SLC26A4):c.2171A>G	concordant	reportable	reportable	228396	1	0	SCV000271452	Pathogenic	Laboratory for Mol	4/26/2016	SLC26A4	chr7	107350580	107350580	http://gnomad.b	3.35E-05	AFR	6.53E-05	0		0	0	https://www.ncbi.nlm.nih.gov/clinvar/variation/228396/
NM_000441.1(SLC26A4):c.28C>A	concordant	unreportable	unreportable	43550	0	1	SCV000700620	Uncertain significance	EGL Genetic Diagn	6/30/2017	SLC26A4	chr7	107302114	107302114	http://gnomad.b	0.0001284349365	AFR	0.002110149944	0		0	0	https://www.ncbi.nlm.nih.gov/clinvar/variation/43550/
NM_000441.1(SLC26A4):c.716T>A	concordant	reportable	reportable	43566	1	0	SCV000331645	Pathogenic	EGL Genetic Diagn	6/30/2017	SLC26A4	chr7	107315505	107315505	http://gnomad.b	0.000120058745	SAS	0.001656810055	0		1	0	https://www.ncbi.nlm.nih.gov/clinvar/variation/43566/
NM_000441.1(SLC26A4):c.898A>C	concordant	unreportable	unreportable	43570	0	1	SCV000060166	Benign	Laboratory for Mol	4/26/2016	SLC26A4	chr7	107323779	107323779	http://gnomad.b	0.001038647748	AFR	0.01418669987	3		2	0	https://www.ncbi.nlm.nih.gov/clinvar/variation/43570/
NM_000441.1(SLC26A4):c.796G>A	concordant	unreportable	unreportable	440275	0	1	SCV000605147	Uncertain significance	ARUP Laboratories	6/30/2017	SLC26A4	chr7	107323677	107323677	http://gnomad.b	4.19E-05	AFR	6.54E-05	0		0	0	https://www.ncbi.nlm.nih.gov/clinvar/variation/440275/
NM_000441.1(SLC26A4):c.1334T>G	concordant	reportable	reportable	4829	1	0	SCV000060092	Pathogenic	Laboratory for Mol	4/26/2016	SLC26A4	chr7	107334918	107334918	http://gnomad.b	0.0001116825535	NFE	0.0001620190014	0		0	0	https://www.ncbi.nlm.nih.gov/clinvar/variation/4829/
NM_000543.4(SMPD1):c.557C>T	concordant	reportable	reportable	371341	2	0	SCV000700333;S	Pathogenic;Pathogenic	EGL Genetic Diagn	2017-06-30;2018-0	SMPD1	chr11	6412852	6412852		1.67E-05					0	0	https://www.ncbi.nlm.nih.gov/clinvar/variation/371341/
NM_000543.4(SMPD1):c.1430C>T	concordant	reportable	reportable	189075	1	0	SCV000617730	Likely pathogenic	GeneDx	11/28/2017	SMPD1	chr11	6415215	6415215	http://gnomad.b	1.43E-05	AMR	8.93E-05	0		0	0	https://www.ncbi.nlm.nih.gov/clinvar/variation/189075/
NM_000543.4(SMPD1):c.689G>A	concordant	unreportable	unreportable	288072	0	1	SCV000342070	Uncertain significance	EGL Genetic Diagn	6/30/2017	SMPD1	chr11	6412984	6412984	http://gnomad.b	0.0001593720266	AFR	0.002893669996	1		0	0	https://www.ncbi.nlm.nih.gov/clinvar/variation/288072/
NM_000543.4(SMPD1):c.887G>A	concordant	unreportable	unreportable	195091	0	2	SCV000227155;S	Benign;Likely benign	EGL Genetic Diagn	2017-06-30;2016-1	SMPD1	chr11	6413182	6413182	http://gnomad.b	0.003813035205	AFR	0.04966669902	21		31	0	https://www.ncbi.nlm.nih.gov/clinvar/variation/195091/
NM_000543.4(SMPD1):c.872G>A	concordant	unreportable	unreportable	195085	0	1	SCV000227144	Uncertain significance	EGL Genetic Diagn	6/30/2017	SMPD1	chr11	6413167	6413167	http://gnomad.b	0.001274976213	NFE	0.002194120083	0		0	0	https://www.ncbi.nlm.nih.gov/clinvar/variation/195085/
NM_000543.4(SMPD1):c.1550A>T	concordant	unreportable	unreportable	198094	0	2	SCV000605228;S	Uncertain significance;Likel	ARUP Laboratories	2017-06-30;2017-0	SMPD1	chr11	6415491	6415491	http://gnomad.b	0.002847288297	NFE	0.003701650072	1		3	0	https://www.ncbi.nlm.nih.gov/clinvar/variation/198094/
NM_000543.4(SMPD1):c.1460C>T	concordant	unreportable	unreportable	235490	0	2	SCV000343585;S	Benign;Benign	EGL Genetic Diagn	2017-06-30;2017-1	SMPD1	chr11	6415245	6415245	http://gnomad.b	0.002545195052	NFE	0.004762330092	1		2	0	https://www.ncbi.nlm.nih.gov/clinvar/variation/235490/
NM_000543.4(SMPD1):c.1267C>T	concordant	reportable	reportable	2992	1	0	SCV000700331	Pathogenic	EGL Genetic Diagn	6/30/2017	SMPD1	chr11	6414850	6414850	http://gnomad.b	9.51E-06	NFE	9.15E-06	0		0	0	https://www.ncbi.nlm.nih.gov/clinvar/variation/2992/
NM_000543.4(SMPD1):c.739G>A	concordant	reportable	reportable	100731	1	0	SCV000700337	Pathogenic	EGL Genetic Diagn	6/30/2017	SMPD1	chr11	6413034	6413034	http://gnomad.b	2.14E-05	AFR	6.61E-05	0		0	0	https://www.ncbi.nlm.nih.gov/clinvar/variation/100731/
NM_000543.4(SMPD1):c.1106A>G	concordant	unreportable	unreportable	288073	0	1	SCV000342071	Uncertain significance	EGL Genetic Diagn	6/30/2017	SMPD1	chr11	6414460	6414460		7.14E-06					0	0	https://www.ncbi.nlm.nih.gov/clinvar/variation/288073/
NM_199292.2(TH):c.1493A>G	concordant	reportable	reportable	304067	1	0	SCV000369908	Likely pathogenic	Illumina Clinical Se	10/18/2016	TH	chr11	2185557	2185557	http://gnomad.b	2.82E-05	NFE	0.0001207120004	0		0	0	https://www.ncbi.nlm.nih.gov/clinvar/variation/304067/
NM_199292.2(TH):c.457C>T	concordant	reportable	reportable	449110	1	0	SCV000616894	Pathogenic	GeneDx	11/28/2017	TH	chr11	2189844	2189844	http://gnomad.b	3.66E-05	EAS	0.000645691005	0		0	0	https://www.ncbi.nlm.nih.gov/clinvar/variation/449110/
NM_000391.3(TPP1):c.380G>A	concordant	reportable	reportable	68746	1	0	SCV000583376	Pathogenic	GeneDx	11/28/2017	TPP1	chr11	6638857	6638857		8.39E-06					0	0	https://www.ncbi.nlm.nih.gov/clinvar/variation/68746/
NM_000391.3(TPP1):c.797G>A	concordant	unreportable	unreportable	207580	0	2	SCV000243420;S	Uncertain significance;Unc	GeneDx;Invitae	2017-11-28;2017-1	TPP1	chr11	6637981	6637981	http://gnomad.b	3.08E-05	EAS	0.0001159550011	0		0	0	https://www.ncbi.nlm.nih.gov/clinvar/variation/207580/
NM_000391.3(TPP1):c.1094G>A	concordant	reportable	reportable	2642	3	0	SCV000243429;S	Likely pathogenic;Pathogen	GeneDx;Invitae;Lal	2017-11-28;2017-1	TPP1	chr11	6637287	6637287	http://gnomad.b	2.80E-05	NFE	3.58E-05	0		0	0	https://www.ncbi.nlm.nih.gov/clinvar/variation/2642/
NM_000391.3(TPP1):c.311T>A	concordant	reportable	reportable	207561	1	0	SCV000628909	Pathogenic	Invitae	10/5/2017	TPP1	chr11	6638926	6638926	http://gnomad.b	1.96E-05	NFE	8.95E-06	0		0	0	https://www.ncbi.nlm.nih.gov/clinvar/variation/207561/
NM_000370.3(TTPA):c.2T>C	concordant	reportable	reportable	189186	1	0	SCV000616204	Pathogenic	Athena Diagnostics	8/17/2017	TTPA	chr8	63998579	63998579		2.40E-05					0	0	https://www.ncbi.nlm.nih.gov/clinvar/variation/189186/
NM_017890.4(VPS13B):c.3427C>T	concordant	reportable	reportable	56658	1	0	SCV000520846	Pathogenic	GeneDx	11/28/2017	VPS13B	chr8	100454845	100454845	http://gnomad.b	1.40E-05	SAS	0.0001299629948	0		0	0	https://www.ncbi.nlm.nih.gov/clinvar/variation/56658/
NM_017890.4(VPS13B):c.8515C>T	concordant	reportable	reportable	56694	1	0	SCV000680675	Pathogenic	GeneDx	2/7/2018	VPS13B	chr8	100830757	100830757	http://gnomad.b	1.40E-05	AMR	2.98E-05	0		0	0	https://www.ncbi.nlm.nih.gov/clinvar/variation/56694/
NM_017890.4(VPS13B):c.3866C>G	concordant	unreportable	unreportable	95851	0	1	SCV000630874	Likely benign	Invitae	10/5/2017	VPS13B	chr8	100494026	100494026	http://gnomad.b	0.0004031467846	AFR	0.005429090001	0		0	0	https://www.ncbi.nlm.nih.gov/clinvar/variation/95851/
NM_017890.4(VPS13B):c.5920C>T	concordant	reportable	reportable	68089	2	0	SCV000616274;S	Pathogenic;Pathogenic	Athena Diagnostics	2017-08-17;2018-0	VPS13B	chr8	100654663	100654663	http://gnomad.b	5.60E-06	AMR	5.97E-05	0		0	0	https://www.ncbi.nlm.nih.gov/clinvar/variation/68089/
NM_017890.4(VPS13B):c.11314C>T	concordant	reportable	reportable	56635	1	0	SCV000709905	Pathogenic	GeneDx	3/26/2018	VPS13B	chr8	100880540	100880540		2.80E-06					0	0	https://www.ncbi.nlm.nih.gov/clinvar/variation/56635/
NM_017890.4(VPS13B):c.4471G>T	concordant	reportable	reportable	2822	1	0	SCV000699447	Pathogenic	Laboratory Corpora	1/25/2018	VPS13B	chr8	100523503	100523503	http://gnomad.b	1.40E-05	NFE	8.98E-06	0		0	0	https://www.ncbi.nlm.nih.gov/clinvar/variation/2822/
NM_017890.4(VPS13B):c.1768G>A	concordant	unreportable	unreportable	194256	0	3	SCV000611527;S	Uncertain significance;Unc	Fulgent Genetics,F	2017-05-23;2017-1	VPS13B	chr8	100155318	100155318	http://gnomad.b	0.001080657353	NFE	0.001407059957	2		1	0	https://www.ncbi.nlm.nih.gov/clinvar/variation/194256/
NM_017890.4(VPS13B):c.10946G>A	concordant	reportable	reportable	56631	1	0	SCV000520847	Pathogenic	GeneDx	11/28/2017	VPS13B	chr8	100871535	100871535		2.80E-06					0	0	https://www.ncbi.nlm.nih.gov/clinvar/variation/56631/

Supplemental Table 2

NM_017890.4(VPS13B):c.2889G>A	concordant	reportable	reportable	56655	1	0	SCV000329732	Pathogenic	GeneDx	11/28/2017	VPS13B	chr8	100396500	100396500		1.12E-05					0	0	https://www.ncbi.nlm.nih.gov/clinvar/variation/56655/
NM_000035.3(ALDOB):c.-214G>A	concordant	unreportable	unreportable	500745	0	1	SCV000706817	Uncertain significance	EGL Genetic Diagn	6/30/2017	ALDOB	chr9	104198194	104198194		0.005697652116					12	0	https://www.ncbi.nlm.nih.gov/clinvar/variation/500745/
NM_000050.4(ASS1):c.-4C>T	concordant	unreportable	unreportable	203632	1	1	SCV000238726;S(	Likely pathogenic;Uncertai	GeneDx;Invitae	2017-11-28;2017-1	ASS1	chr9	133327612	133327612	http://gnomad.b	0.0005653745676	SAS	0.001886670012	2		1	0	https://www.ncbi.nlm.nih.gov/clinvar/variation/203632/
NM_000492.3(CFTR):c.*2G>A	concordant	unreportable	unreportable	53155	0	1	SCV000228426	Uncertain significance	EGL Genetic Diagn	6/30/2017	CFTR	chr7	117307164	117307164	http://gnomad.b	0.0002060640609	AFR	0.00196721009	0		0	0	https://www.ncbi.nlm.nih.gov/clinvar/variation/53155/
NM_000492.3(CFTR):c.-226G>T	concordant	unreportable	unreportable	51031	0	3	SCV000706119;S(	Uncertain significance;Unc	EGL Genetic Diagn	2017-06-30;2016-0	CFTR	chr7	117119923	117119923		0.001382747624					2	0	https://www.ncbi.nlm.nih.gov/clinvar/variation/51031/
NM_000518.4(HBB):c.*110T>C	concordant	reportable	reportable	36332	2	0	SCV000603914;S(	Pathogenic;Pathogenic	ARUP Laboratories	2017-06-30;2017-0	HBB	chr11	5246718	5246718		3.97E-05					0	0	https://www.ncbi.nlm.nih.gov/clinvar/variation/36332/
NM_000518.4(HBB):c.*111A>G	concordant	reportable	reportable	15488	1	0	SCV000601230	Pathogenic	Quest Diagnostics I	8/1/2017	HBB	chr11	5246717	5246717		4.67E-06					0	0	https://www.ncbi.nlm.nih.gov/clinvar/variation/15488/
NM_000518.4(HBB):c.-151C>T	concordant	reportable	reportable	15461	1	0	SCV000697087	Pathogenic	Laboratory Corpora	1/25/2018	HBB	chr11	5248402	5248402		0.0001166338537					2	0	https://www.ncbi.nlm.nih.gov/clinvar/variation/15461/
NM_000518.4(HBB):c.-138C>A	concordant	reportable	reportable	393701	1	0	SCV000601246	Pathogenic	Quest Diagnostics I	8/1/2017	HBB	chr11	5248389	5248389		9.33E-06					0	0	https://www.ncbi.nlm.nih.gov/clinvar/variation/393701/
NM_000518.4(HBB):c.-31C>T	concordant	unreportable	unreportable	36291	0	2	SCV000603911;S(	Likely benign;Uncertain sig	ARUP Laboratories	2017-06-30;2017-0	HBB	chr11	5248282	5248282	http://gnomad.b	5.60E-05	NFE	0.0001349670056	0		0	0	https://www.ncbi.nlm.nih.gov/clinvar/variation/36291/
NM_000518.4(HBB):c.-78A>C	concordant	reportable	reportable	15470	2	0	SCV000697148;S(	Pathogenic;Pathogenic	Laboratory Corpora	2018-01-25;2017-0	HBB	chr11	5248329	5248329		9.33E-06					0	0	https://www.ncbi.nlm.nih.gov/clinvar/variation/15470/
NM_000518.4(HBB):c.-82C>T	concordant	unreportable	unreportable	439163	1	1	SCV000697151;S(	Uncertain significance;Path	Laboratory Corpora	2018-01-25;2017-0	HBB	chr11	5248333	5248333		7.00E-06					0	0	https://www.ncbi.nlm.nih.gov/clinvar/variation/439163/
NM_000518.4(HBB):c.-50A>C	concordant	reportable	reportable	36292	1	0	SCV000605834	Pathogenic	Quest Diagnostics I	8/1/2017	HBB	chr11	5248301	5248301	http://gnomad.b	1.87E-05	SAS	0.0008475679788	0		0	0	https://www.ncbi.nlm.nih.gov/clinvar/variation/36292/
NM_000518.4(HBB):c.-140C>T	concordant	reportable	reportable	15514	1	0	SCV000052609	Pathogenic	Laboratory Corpora	11/20/2015	HBB	chr11	5248391	5248391		1.17E-05					0	0	https://www.ncbi.nlm.nih.gov/clinvar/variation/15514/
NM_000243.2(MEFV):c.*9C>T	concordant	unreportable	unreportable	445530	0	2	SCV000629015;S(	Benign;Uncertain significar	Invitae;Laboratory	2017-10-05;2018-0	MEFV	chr16	3293132	3293132	http://gnomad.b	0.002065491184	AFR	0.02497380041	2		7	0	https://www.ncbi.nlm.nih.gov/clinvar/variation/445530/
NM_000277.1(PAH):c.*19G>T	concordant	unreportable	unreportable	255733	0	3	SCV000521187;S(	Likely benign;Benign;Likely	GeneDx;Invitae;Qu	2017-11-28;2017-1	PAH	chr12	103232934	103232934	http://gnomad.b	0.002071153435	SAS	0.0081232097	8		7	0	https://www.ncbi.nlm.nih.gov/clinvar/variation/255733/
NR_003051.3(RMRP):n.147G>A	concordant	reportable	reportable	379208	1	0	SCV000640108	Likely pathogenic	Invitae	10/5/2017	RMRP	chr9	35657869	35657869	http://gnomad.b	3.90E-05	ASJ	0.0001270969951	0		0	0	https://www.ncbi.nlm.nih.gov/clinvar/variation/379208/
NM_000441.1(SLC26A4):c.-103T>C	concordant	unreportable	unreportable	4838	1	2	SCV000491274;S(	Likely pathogenic;Uncertai	GeneDx;Illumina C	2016-12-21;2016-1	SLC26A4	chr7	107301201	107301201		0.001870682771					1	0	https://www.ncbi.nlm.nih.gov/clinvar/variation/4838/
NM_000352.3(ABCC8):c.1332+4delC	concordant	unreportable	unreportable	157682	0	2	SCV000592988;S(	Uncertain significance;Likel	Genetic Services La	2017-07-05;2016-1	ABCC8	chr11	17470058	17470059	http://gnomad.b	0.0008543390616	FIN	0.002556049963	0		0	0	https://www.ncbi.nlm.nih.gov/clinvar/variation/157682/
NM_000352.3(ABCC8):c.1879delC	concordant	reportable	reportable	370909	1	0	SCV000592995	Pathogenic	Genetic Services La	7/5/2017	ABCC8	chr11	17450155	17450156		1.03E-05					0	0	https://www.ncbi.nlm.nih.gov/clinvar/variation/370909/
NM_000016.4(ACADM):c.449_452del(	concordant	reportable	reportable	189036	2	0	SCV000700258;S(	Pathogenic;Pathogenic	EGL Genetic Diagn	2017-06-30;2017-1	ACADM	chr1	76200533	76200537	http://gnomad.b	2.72E-05	EAS	0.000289921998	0		0	0	https://www.ncbi.nlm.nih.gov/clinvar/variation/189036/
NM_000018.3(ACADVL):c.298_299del(	concordant	reportable	reportable	189116	1	0	SCV000654949	Pathogenic	Invitae	10/5/2017	ACADVL	chr17	7124101	7124103		1.13E-05					0	0	https://www.ncbi.nlm.nih.gov/clinvar/variation/189116/
NM_000018.3(ACADVL):c.887_888del(	concordant	reportable	reportable	189008	1	0	SCV000693968	Likely pathogenic	Laboratory Corpora	1/25/2018	ACADVL	chr17	7125993	7125995	http://gnomad.b	2.83E-06	NFE	8.99E-06	0		0	0	https://www.ncbi.nlm.nih.gov/clinvar/variation/189008/
NM_000018.3(ACADVL):c.343delG	concordant	reportable	reportable	1624	1	0	SCV000576837	Pathogenic	GeneDx	11/28/2017	ACADVL	chr17	7124241	7124242	http://gnomad.b	4.53E-05	NFE	8.95E-06	0		0	0	https://www.ncbi.nlm.nih.gov/clinvar/variation/1624/
NM_000018.3(ACADVL):c.708_709del(	concordant	reportable	reportable	495345	1	0	SCV000693966	Likely pathogenic	Laboratory Corpora	1/25/2018	ACADVL	chr17	7125355	7125357		5.66E-06					0	0	https://www.ncbi.nlm.nih.gov/clinvar/variation/495345/
NM_000022.2(ADA):c.956_960del5	concordant	reportable	reportable	193544	2	0	SCV000321381;S(	Pathogenic;Pathogenic	GeneDx;Laborator	2017-11-28;2018-0	ADA	chr20	43249673	43249678	http://gnomad.b	0.0001120322653	NFE	0.0002416759962	0		0	0	https://www.ncbi.nlm.nih.gov/clinvar/variation/193544/
NM_000642.2(AGL):c.3216_3217delG/	concordant	reportable	reportable	432008	3	0	SCV000701400;S(	Pathogenic;Pathogenic;Pat	EGL Genetic Diagn	2017-06-30;2017-1	AGL	chr1	100358117	100358119	http://gnomad.b	1.67E-05	AMR	5.96E-05	0		0	0	https://www.ncbi.nlm.nih.gov/clinvar/variation/432008/
NM_000383.3(AIRE):c.1265delC	concordant	reportable	reportable	265456	1	0	SCV000322390	Pathogenic	GeneDx	11/28/2017	AIRE	chr21	45713043	45713044	http://gnomad.b	8.36E-06	NFE	3.95E-05	0		0	0	https://www.ncbi.nlm.nih.gov/clinvar/variation/265456/
NM_000382.2(ALDH3A2):c.471+1delG	concordant	reportable	reportable	189163	1	0	SCV000696650	Pathogenic	Laboratory Corpora	1/25/2018	ALDH3A2	chr17	19555944	19555945	http://gnomad.b	1.96E-05	NFE	2.69E-05	0		0	0	https://www.ncbi.nlm.nih.gov/clinvar/variation/189163/
NM_000050.4(ASS1):c.450_451delCT	concordant	reportable	reportable	189044	2	0	SCV000630058;S(	Pathogenic;Likely pathoger	Invitae;Laboratory	2017-10-05;2018-0	ASS1	chr9	133342139	133342141	http://gnomad.b	5.57E-06	NFE	8.95E-06	0		0	0	https://www.ncbi.nlm.nih.gov/clinvar/variation/189044/
NM_000051.3(ATM):c.450_453delTTC	concordant	reportable	reportable	490572	1	0	SCV000687555	Pathogenic	Color Genomics, In	12/21/2017	ATM	chr11	108106510	108106514	http://gnomad.b	2.78E-06	NFE	8.96E-06	0		0	0	https://www.ncbi.nlm.nih.gov/clinvar/variation/490572/
NM_000051.3(ATM):c.3850delA	concordant	reportable	reportable	234124	2	0	SCV000568320;S(	Pathogenic;Pathogenic	GeneDx;Invitae	2017-11-28;2017-0	ATM	chr11	108155055	108155056		2.78E-06					0	0	https://www.ncbi.nlm.nih.gov/clinvar/variation/234124/
NM_000051.3(ATM):c.1564_1565del(	concordant	reportable	reportable	127340	3	0	SCV000681982;S(	Pathogenic;Pathogenic;Pat	Color Genomics, In	2017-10-26;2017-1	ATM	chr11	108121752	108121754	http://gnomad.b	3.06E-05	NFE	9.86E-05	0		0	0	https://www.ncbi.nlm.nih.gov/clinvar/variation/127340/
NM_000051.3(ATM):c.8876_8879delA	concordant	reportable	reportable	189140	2	0	SCV000293443;S(	Pathogenic;Pathogenic	GeneDx;Invitae	2017-11-28;2017-0	ATM	chr11	108235831	108235835	http://gnomad.b	2.78E-06	AMR	5.96E-05	0		0	0	https://www.ncbi.nlm.nih.gov/clinvar/variation/189140/
NM_000051.3(ATM):c.3802delG	concordant	reportable	reportable	127374	3	0	SCV000682163;S(	Pathogenic;Pathogenic;Pat	Color Genomics, In	2017-10-26;2017-1	ATM	chr11	108155007	108155008	http://gnomad.b	3.34E-05	AMR	8.94E-05	0		0	0	https://www.ncbi.nlm.nih.gov/clinvar/variation/127374/
NM_000051.3(ATM):c.790delT	concordant	reportable	reportable	141742	4	0	SCV000687801;S(	Pathogenic;Pathogenic;Pat	Color Genomics, In	2017-12-21;2017-1	ATM	chr11	108115639	108115640	http://gnomad.b	5.57E-06	NFE	1.80E-05	0		0	0	https://www.ncbi.nlm.nih.gov/clinvar/variation/141742/
NM_000051.3(ATM):c.8418+5_8418+8	concordant	reportable	reportable	181866	2	0	SCV000209610;S(	Pathogenic;Pathogenic	GeneDx;Invitae	2017-11-28;2017-1	ATM	chr11	108214098	108214102	http://gnomad.b	1.67E-05	NFE	8.98E-06	0		0	0	https://www.ncbi.nlm.nih.gov/clinvar/variation/181866/
NM_000051.3(ATM):c.5290delC	concordant	reportable	reportable	127405	3	0	SCV000682261;S(	Pathogenic;Pathogenic;Pat	Color Genomics, In	2017-10-26;2017-1	ATM	chr11	108172486	108172487		1.11E-05					0	0	https://www.ncbi.nlm.nih.gov/clinvar/variation/127405/
NM_000051.3(ATM):c.1355delC	concordant	reportable	reportable	141474	3	0	SCV000687303;S(	Pathogenic;Pathogenic;Pat	Color Genomics, In	2017-12-21;2017-1	ATM	chr11	108121546	108121547		8.35E-06					0	0	https://www.ncbi.nlm.nih.gov/clinvar/variation/141474/
NM_000051.3(ATM):c.1402_1403delA	concordant	reportable	reportable	140889	3	0	SCV000681977;S(	Pathogenic;Pathogenic;Pat	Color Genomics, In	2017-10-26;2017-1	ATM	chr11	108121593	108121595	http://gnomad.b	4.73E-05	EAS	0.0002319110063	0		0	0	https://www.ncbi.nlm.nih.gov/clinvar/variation/140889/
NM_000051.3(ATM):c.1290_1291delT	concordant	reportable	reportable	141241	1	0	SCV000547149	Pathogenic	Invitae	10/5/2017	ATM	chr11	108121479	108121481	http://gnomad.b	5.57E-06	SAS	3.25E-05	0		0	0	https://www.ncbi.nlm.nih.gov/clinvar/variation/141241/
NM_000051.3(ATM):c.1027_1030del(	concordant	reportable	reportable	135731	4	0	SCV000681947;S(	Pathogenic;Pathogenic;Pat	Color Genomics, In	2017-10-26;2017-1	ATM	chr11	108117812	108117816	http://gnomad.b	3.90E-05	ASJ	0.0004063390079	0		0	0	https://www.ncbi.nlm.nih.gov/clinvar/variation/135731/
NM_000051.3(ATM):c.8833_8834delC	concordant	reportable	reportable	186546	1	0	SCV000568343	Pathogenic	GeneDx	11/28/2017	ATM	chr11	108225581	108225583	http://gnomad.b	2.78E-06	NFE	8.96E-06	0		0	0	https://www.ncbi.nlm.nih.gov/clinvar/variation/186546/
NM_000051.3(ATM):c.381delA	concordant	reportable	reportable	141546	3	0	SCV000602573;S(	Pathogenic;Pathogenic;Pat	ARUP Laboratories	2017-06-30;2017-1	ATM	chr11	108106445	108106446	http://gnomad.b	2.78E-06	NFE	8.96E-06	0		0	0	https://www.ncbi.nlm.nih.gov/clinvar/variation/141546/
NM_000051.3(ATM):c.6228delT	concordant	reportable	reportable	186516	5	0	SCV000682314;S(	Pathogenic;Pathogenic;Pat	Color Genomics, In	2017-10-26;2017-0	ATM	chr11	108188127	108188128		2.23E-05					0	0	https://www.ncbi.nlm.nih.gov/clinvar/variation/186516/
NM_000051.3(ATM):c.7705_7706del(	concordant	reportable	reportable	220550	3	0	SCV000682432;S(	Pathogenic;Pathogenic;Pat	Color Genomics, In	2017-10-26;2017-1	ATM	chr11	108202677	108202679	http://gnomad.b	1.11E-05	AMR	0.0001489160059	0		0	0	https://www.ncbi.nlm.nih.gov/clinvar/variation/220550/
NM_000051.3(ATM):c.5910delA	concordant	reportable	reportable	142051	1	0	SCV000568327	Pathogenic	GeneDx	11/28/2017	ATM	chr11	108181032	108181033		2.78E-06					0	0	https://www.ncbi.nlm.nih.gov/clinvar/variation/142051/
NM_000051.3(ATM):c.4632_4635delC	concordant	reportable	reportable	420011	1	0	SCV000568324	Pathogenic	GeneDx	11/28/2017	ATM	chr11	108164057	108164061		2.78E-06					0	0	https://www.ncbi.nlm.nih.gov/clinvar/variation/420011/
NM_000051.3(ATM):c.1524delT	concordant	reportable	reportable	189158	2	0	SCV000293432;S(	Likely pathogenic;Pathoger	GeneDx;Invitae	2017-11-28;2017-1	ATM	chr11	108121714	108121715		5.57E-06					0	0	https://www.ncbi.nlm.nih.gov/clinvar/variation/189158/
NM_000053.3(ATP7B):c.4092_4093de	concordant	reportable	reportable	371438	2	0	SCV000593530;S(	Pathogenic;Pathogenic	Genetic Services La	2017-07-05;2018-0	ATP7B	chr13	52509759	52509761	http://gnomad.b	2.81E-05	EAS	5.81E-05	0		0	0	https://www.ncbi.nlm.nih.gov/clinvar/variation/371438/
NM_000053.3(ATP7B):c.2009_2015de	concordant	reportable	reportable	188862	1	0	SCV000694412	Pathogenic	Laboratory Corpora	1/25/2018	ATP7B	chr13	52534389	52534396	http://gnomad.b	1.96E-05	NFE	4.49E-05	0		0	0	https://www.ncbi.nlm.nih.gov/clinvar/variation/188862/
NM_000053.3(ATP7B):c.3402delC	concordant	reportable	reportable	88958	2	0	SCV000617353;S(	Pathogenic;Pathogenic	GeneDx;Illumina C	2017-11-28;2016-1	ATP7B	chr13	52516531	52516532	http://gnomad.b	5.33E-05	NFE	0.0001433900034	0		0	0	https://www.ncbi.nlm.nih.gov/clinvar/variation/88958/
NM_000053.3(ATP7B):c.2532delA	concordant	reportable	reportable	188883	1	0	SCV000602597	Pathogenic	ARUP Laboratories	6/30/2017	ATP7B	chr13	52524450	52524451	http://gnomad.b	3.37E-05	NFE	8.95E-06	0		0	0	https://www.ncbi.nlm.nih.gov/clinvar/variation/188883/
NM_000053.3(ATP7B):c.1745_1746de	concordant	reportable	reportable	189112	1	0	SCV000341737	Pathogenic	EGL Genetic Diagn	6/30/2017	ATP7B	chr13	52539130	52539132	http://gnomad.b	5.61E-06	NFE	3.58E-05	0		0	0	https://www.ncbi.nlm.nih.gov/clinvar/variation/189112/
NM_024685.3(BBS10):c.1677delC	concordant	reportable	reportable	496471	1	0	SCV000699622	Pathogenic	Laboratory Corpora	1/25/2018	BBS10	chr12	76740087	76740088	http://gnomad.b	8.39E-06	AMR	2.98E-05	0		0	0	https://www.ncbi.nlm.nih.gov/clinvar/variation/496471/
NM_024685.3(BBS10):c.909_912delT(	concordant	reportable	reportable	432013	1	0	SCV000589675	Pathogenic	GeneDx	11/28/2017	BBS10	chr12	76740852	76740856	http://gnomad.b	5.88E-05	NFE	3.59E-05	0		0	0	https://www.ncbi.nlm.nih.gov/clinvar/variation/432013/
NM_024685.3(BBS10):c.687delT	concordant	reportable	reportable	216123	2	0	SCV000332733;S(	Pathogenic;Pathogenic	EGL Genetic Diagn	2017-06-30;2016-0	BBS10	chr12	76741077	76741078	http://gnomad.b	5.04E-05	NFE	3.58E-05	0		0	0	https://www.ncbi.nlm.nih.gov/clinvar/variation/216123/
NM_024685.3(BBS10):c.1091delA	concordant	reportable	reportable	166723	1	0	SCV000699619	Pathogenic	Laboratory Corpora	1/25/2018	BBS10	chr12	76740673	76740674	http://gnomad.b	0.0001398914442	ASJ	0.001523770043	0		0	0	https://www.ncbi.nlm.nih.gov/clinvar/variation/166723/
NM_024685.3(BBS10):c.2119_2120de	concordant	reportable	reportable	406221	2	0	SCV000544764;S(	Pathogenic;Pathogenic	Invitae;Laboratory	2017-03-14;2018-0	BBS10	chr12	76739644	76739646	http://gnomad.b	3.92E-05	NFE	0.0001255429961	0		0	0	https://www.ncbi.nlm.nih.gov/clinvar/variation/406221/
NM_000709.3(BCKDHA):c.117delC	concordant	reportable	reportable	93342	1	0	SCV000566136	Pathogenic	GeneDx	11/28/2017	BCKDHA	chr19	41916543	41916544	http://gnomad.b	3.96E-05	SAS	3.25E-05	0		0	0	https://www.ncbi.nlm.nih.gov/clinvar/variation/93342/
NM_183050.2(BCKDHB):c.595_596del	concordant	reportable	reportable	96599	1	0	SCV000627820	Pathogenic	Invitae	10/5/2017	BCKDHB	chr6	80878706	80878708	http://gnomad.b	2.82E-05	NFE	5.38E-05	0		0	0	https://www.ncbi.nlm.nih.gov/clinvar/variation/96599/
NM_000057.2(BLM):c.3028delG	concordant	reportable	reportable	189048	1	0	SCV000623297	Pathogenic	Invitae	10/5/2017	BLM	chr15	91337404	91337405	http://gnomad.b	9.51E-06	NFE	3.61E-05	0		0	0	https://www.ncbi.nlm.nih.gov/clinvar/variation/189048/
NM_000057.2(BLM):c.2923delC	concordant	reportable	reportable	42076	3	0	SCV000336681;S(	Pathogenic;Pathogenic;Pat	EGL Genetic Diagn	2017-06-30;2017-1	BLM	chr15	91333976	91333977	http://gnomad.b	3.09E-05	FIN	8.97E-05	0		0	0	https://www.ncbi.nlm.nih.gov/clinvar/variation/42076/
NM_000060.2(BTD):c.1052delC	concordant	reportable	reportable	25057	2	0	SCV000329855;S(	Pathogenic;Pathogenic	GeneDx;Quest Dia	2017-11-28;2017-0	BTD	chr3	15686414	15686415		5.77E-06					0	0	https://www.ncbi.nlm.nih.gov/clinvar/variation/25057/
NM_000071.2(CBS):c.536_553del18	concordant	reportable	reportable	198388	1	0	SCV000491080	Likely pathogenic	GeneDx	12/21/2016	CBS	chr21	44485609	44485627	http://gnomad.b	1.12E-05	NFE	2.73E-05	0		0	0	https://www.ncbi.nlm.nih.gov/clinvar/variation/198388/
NM_000492.3(CFTR):c.174_177delTA(	concordant	reportable	reportable	53373	1	0	SCV000677598	Pathogenic	CFTR2	6/29/2017	CFTR	chr7	117149094	117149098		1.93E-06					0	0	https://www.ncbi.nlm.nih.gov/clinvar/variation/53373/
NM_000492.3(CFTR):c.1029delC	concordant	reportable	reportable	53170	1	0	SCV000245932	Pathogenic	CFTR2	6/29/2017	CFTR	chr7	117180312	117180313	http://gnomad.b	1.54E-05	SAS	0.0005197839928	0		0	0	https://www.ncbi.nlm.nih.gov/clinvar/variation/53170/
NM_000492.3(CFTR):c.3002_3003delT	concordant	reportable	reportable	53623	1	0	SCV000245904	Pathogenic	CFTR2	6/29/2017	CFTR	chr7	117250583	117250585		1.93E-06					0	0	https://www.ncbi.nlm.nih.gov/clinvar/variation/53623/
NM_000492.3(CFTR):c.2875delG	concordant	reportable	reportable	53583	1	0	SCV000071478	Pathogenic	CFTR2	6/29/2017	CFTR	chr7	117243802	117243803	http://gnomad.b	1.93E-06	NFE	8.96E-06	0		0	0	https://www.ncbi.nlm.nih.gov/clinvar/variation/53583/
NM_000492.3(CFTR):c.2463_2464delT	concordant	reportable	reportable	455767	2	0	SCV000245879;S(	Pathogenic;Pathogenic	CFTR2;Invitae	2017-06-29;2017-1	CFTR	chr7	117232682	117232684		1.93E-06					0	0	https://www.ncbi.nlm.nih.gov/clinvar/variation/455767/
NM_000492.3(CFTR):c.2015_2017del/	concordant	unreportable	unreportable	439062	0	1	SCV000601065	Uncertain significance	Quest Diagnostics I	8/1/2017	CFTR	chr7	117232233	117232236	http://gnomad.b	3.85E-06			0		0	0	https://www.ncbi.nlm.nih.gov/clinvar/variation/439062/
NM_006493.2(CLN5):c.924_925delAT	concordant	reportable	reportable	189038	2	0	SCV000491175;S(	Likely pathogenic;Pathoger	GeneDx;Invitae	2016-12-21;2017-0	CLN5	chr13	77574801	77574803	http://gnomad.b	1.40E-05	AMR	0.0002383079991	0		0	0	https://www.ncbi.nlm.nih.gov/clinvar/variation/189038/
NM_004937.2(CTNS):c.18_21delGACT	concordant	reportable	reportable	188834	1	0	SCV000229059	Pathogenic	EGL Genetic Diagn	6/30/2017	CTNS	chr17	3543515	3543519	http://gnomad.b	2.67E-05	SAS	0.0001299459982	0		0	0	https://www.ncbi.nlm.nih.gov/clinvar/variation/188834/
NM_000152.3(GAA):c.1827delC	concordant	reportable	reportable	188936	1	0	SCV000341591	Pathogenic	EGL Genetic Diagn	6/30/2017	GAA	chr17	78086448	78086449	http://gnomad.b	5.56E-05	NFE	7.40E-05	0		0	0	https://www.ncbi.nlm.nih.gov/clinvar/variation/188936/
NM_000152.3(GAA):c.1051delG	concordant	reportable	reportable	188841	1	0	SCV000339223	Pathogenic	EGL Genetic Diagn	6/30/2017	GAA	chr17	78082182	78082183	http://gnomad.b	8.34E-06	NFE	8.97E-06	0		0	0	https://www.ncbi.nlm.nih.gov/clinvar/variation/188841/
NM_000152.3(GAA):c.2501_2502delC.	concordant	reportable	reportable	286229	1	0	SCV000339572	Pathogenic	EGL Genetic Diagn	6/30/2017	GAA	chr17	78092008	78092010	http://gnomad.b	1.11E-05	AMR	5.97E-05	0		0	0	https://www.ncbi.nlm.nih.gov/clinvar/variation/286229/
NM_000153.3(GALC):c.955delT	concordant	reportable	reportable	188767	1	0	SCV000321694	Pathogenic	GeneDx	11/28/2017	GALC	chr14	88431926	88431927	http://gnomad.b	1.12E-05	NFE	8.99E-06	0		0	0	https://www.ncbi.nlm.nih.gov/clinvar/variation/188767/
NM_004004.5(GJB2):c.645delT	concordant	reportable	reportable	449487	1	0	SCV000617684	Likely pathogenic	GeneDx	11/28/2017	GJB2	chr13	20763075	20763076		2.79E-06					0	0	https://www.ncbi.nlm.nih.gov/clinvar/variation/449487/
NM_004004.5(GJB2):c.176_191del16	concordant	reportable	reportable	284906	2	0	SCV000599736;S(	Pathogenic;Pathogenic	Division of Genomi	2017-05-17;2017-0	GJB2	chr13	20763529	20763545	http://gnomad.b	4.19E-05	EAS	0.0001739330037	0		0	0	https://www.ncbi.nlm.nih.gov/clinvar/variation/284906/
NM_004004.5(GJB2):c.299_300delAT	concordant	reportable	reportable	44736	1	0	SCV000322425	Pathogenic	GeneDx	11/28/2017	GJB2	chr13	20763420	20763422	http://gnomad.b	0.0001229386815	EAS	0.0008697669837	0		0	0	https://www.ncbi.nlm.nih.gov/clinvar/variation/44736/
NM_012203.1(GRHPR):c.864_865delT(	concordant	reportable	reportable	162020	1	0	SCV000699331	Pathogenic	Laboratory Corpora	1/25/2018	GRHPR	chr9	37432131	37432133	http://gnomad.b	4.49E-05	EAS	0.0005218000151	0		0	0	https://www.ncbi.nlm.nih.gov/clinvar/variation/162020/
NM_000182.4(HADHA):c.1967delT	concordant	reportable	reportable	188712	2	0	SCV000322387;S(	Pathogenic;Pathogenic	GeneDx;Laborator	2017-11-28;2018-0	HADHA	chr2	26415211	26415212	http://gnomad.b	1.12E-05	NFE	8.95E-06	0		0	0	https://www.ncbi.nlm.nih.gov/clinvar/variation/188712/
NM_000518.4(HBB):c.251delG	concordant	reportable	reportable	36306	2	0	SCV000697101;S(	Pathogenic;Pathogenic	Laboratory Corpora	2018-01-25;2017-0	HBB	chr11	5247870	5247871		2.33E-06					0	0	https://www.ncbi.nlm.nih.gov/clinvar/variation/36306/
NM_000518.4(HBB):c.203_204delTG	concordant	reportable	reportable	36303	2	0	SCV000697091;S(	Likely pathogenic;Pathoger	Laboratory Corpora	2018-01-25;2017-0	HBB	chr11	5247917	5247919		9.33E-06					0	0	https://www.ncbi.nlm.nih.gov/clinvar/variation/36303/
NM_000518.4(HBB):c.46delT	concordant	reportable	reportable	38647	2	0	SCV000697134;S(	Pathogenic;Pathogenic	Laboratory Corpora	2018-01-25;2017-0	HBB	chr11	5248205	5248206		2.33E-06					0	0	https://www.ncbi.nlm.nih.gov/clinvar/variation/38647/
NM_000518.4(HBB):c.135delC	concordant	reportable	reportable	15415	3	0	SCV000603916;S(	Pathogenic;Pathogenic;Pat	ARUP Laboratories	2017-06-30;2018-0	HBB	chr11	5247986	5247987	http://gnomad.b	9.33E-06	NFE	2.69E-05	0		0	0	https://www.ncbi.nlm.nih.gov/clinvar/variation/15415/
NM_000411.6(HLCS):c.782delG	concordant	reportable	reportable	203777	1	0	SCV000238927	Pathogenic	GeneDx	11/28/2017	HLCS	chr21	38308962	38308963	http://gnomad.b	4.82E-05	EAS	0.0002319380001	0		0	0	https://www.ncbi.nlm.nih.gov/clinvar/variation/203777/
NM_000203.3(IDUA):c.1893delC	concordant	reportable	reportable	495733	1	0	SCV000695970	Likely pathogenic	Laboratory Corpora	1/25/2018	IDUA	chr4	998108	998109		2.78E-06					0	0	https://www.ncbi.nlm.nih.gov/clinvar/variation/495733/
NM_000228.2(LAMB3):c.2842delG	concordant	reportable	reportable	372403	1	0	SCV000490597	Pathogenic	GeneDx	12/21/2016	LAMB3	chr1	209791863	209791864	http://gnomad.b	3.36E-05	NFE	3.58E-05	0		0	0	https://www.ncbi.nlm.nih.gov/clinvar/variation/372403/
NM_000228.2(LAMB3):c.1365_1366de	concordant	reportable	reportable	189004	1	0	SCV000695998	Pathogenic	Laboratory Corpora	1/25/2018	LAMB3	chr1	209800846	209800848	http://gnomad.b	2.80E-05	NFE	5.38E-05	0		0	0	https://www.ncbi.nlm.nih.gov/clinvar/variation/189004/
NM_000243.2(MEFV):c.2076_2078del	concordant	reportable	reportable	97485	1	0	SCV000565123	Pathogenic	GeneDx	11/28/2017	MEFV	chr16	3293408	3293411	http://gnomad.b	2.80E-06			0		0	0	https://www.ncbi.nlm.nih.gov/clinvar/variation/97485/
NM_015506.2(MMACHC):c.328_331de	concordant	reportable	reportable	203835	2	0	SCV000640287;S(	Pathogenic;Pathogenic	Invitae;Laboratory	2017-10-05;2018-0	MMACHC	chr1	45973932	45973936	http://gnomad.b	2.37E-05	AMR	0.0003871119989	0		0	0	https://www.ncbi.nlm.nih.gov/clinvar/variation/203835/
NM_002485.4(NBN):c.698_701delAAC	concordant	reportable	reportable	127878	4	0	SCV000685816;S(	Pathogenic;Pathogenic;Pat	Color Genomics, In	2017-10-26;2017-1	NBN	chr8	90983401	90983405	http://gnomad.b	2.79E-05	AFR	6.57E-05	0		0	0	https://www.ncbi.nlm.nih.gov/clinvar/variation/127878/
NM_000271.4(NPC1):c.3734_3735del(	concordant	reportable	reportable	419641	1	0	SCV000706895	Likely pathogenic	EGL Genetic Diagn	6/30/2017	NPC1	chr18	21113337	21113339		5.72E-06					0	0	https://www.ncbi.nlm.nih.gov/clinvar/variation/419641/
NM_004646.3(NPHS1):c.1020delT	concordant	reportable	reportable	496269	1	0	SCV000698494	Likely pathogenic	Laboratory Corpora	1/25/2018	NPHS1	chr19	36339688	36339689		2.79E-06					0	0	https://www.ncbi.nlm.nih.gov/clinvar/variation/496269/
NM_014625.2(NPHS2):c.948delT	concordant	reportable	reportable	188990	1	0	SCV000614355	Pathogenic	Athena Diagnostics	8/17/2017	NPHS2	chr1	179520511	179520512	http://gnomad.b	1.69E-05	NFE	2.70E-05	0		0	0	https://www.ncbi.nlm.nih.gov/clinvar/variation/188990/
NM_000277.1(PAH):c.967_969delACA	concordant	reportable	reportable	102913	1	0	SCV000696474	Pathogenic	Laboratory Corpora	1/25/2018	PAH	chr12	103240672	103240675	http://gnomad.b	1.10E-05	ASJ	0.0002031689946	0		0	0	https://www.ncbi.nlm.nih.gov/clinvar/variation/102913/

Supplemental Table 2

NM_000277.1(PAH):c.806delT	concordant	reportable	reportable	102844	2	0	SCV000203181;S	Pathogenic;Pathogenic	EGL Genetic Diagn	2017-06-30;2017-1	PAH	chr12	103246628	103246629		2.74E-06					0	0	https://www.ncbi.nlm.nih.gov/clinvar/variation/102844/
NM_000277.1(PAH):c.284_286delTCA	concordant	reportable	reportable	604	3	0	SCV000239098;S	Pathogenic;Pathogenic;Pat	GeneDx;Invitae;Qu	2017-11-28;2017-1	PAH	chr12	103288578	103288581	http://gnomad.b	6.58E-05	EAS	5.80E-05	0		1	0	https://www.ncbi.nlm.nih.gov/clinvar/variation/604/
NM_000277.1(PAH):c.165delT	concordant	reportable	reportable	611	3	0	SCV000239100;S	Pathogenic;Pathogenic;Pat	GeneDx;Laborator	2017-11-28;2018-0	PAH	chr12	103306571	103306572	http://gnomad.b	3.29E-05	NFE	2.69E-05	0		0	0	https://www.ncbi.nlm.nih.gov/clinvar/variation/611/
NM_000466.2(PEX1):c.2636_2638delT	concordant	unreportable	unreportable	288706	0	1	SCV000342896	Uncertain significance	EGL Genetic Diagn	6/30/2017	PEX1	chr7	92129097	92129100		2.77E-06					0	0	https://www.ncbi.nlm.nih.gov/clinvar/variation/288706/
NM_138694.3(PKHD1):c.711_714delA	concordant	reportable	reportable	188960	1	0	SCV000340221	Pathogenic	EGL Genetic Diagn	6/30/2017	PKHD1	chr6	51934318	51934322	http://gnomad.b	1.11E-05	NFE	1.79E-05	0		0	0	https://www.ncbi.nlm.nih.gov/clinvar/variation/188960/
NM_000303.2(PMM2):c.324delG	concordant	reportable	reportable	503676	1	0	SCV000709891	Pathogenic	GeneDx	3/26/2018	PMM2	chr16	8900240	8900241		2.78E-06					0	0	https://www.ncbi.nlm.nih.gov/clinvar/variation/503676/
NM_012434.4(SLC17A5):c.802_816del	concordant	reportable	reportable	56558	2	0	SCV000568752;S	Pathogenic;Pathogenic	GeneDx;Laborator	2017-11-28;2018-0	SLC17A5	chr6	74345107	74345122	http://gnomad.b	5.63E-05	NFE	6.29E-05	0		0	0	https://www.ncbi.nlm.nih.gov/clinvar/variation/56558/
NM_012434.4(SLC17A5):c.533delC	concordant	reportable	reportable	167693	1	0	SCV000699340	Pathogenic	Laboratory Corpora	1/25/2018	SLC17A5	chr6	74348214	74348215	http://gnomad.b	6.52E-05	NFE	0.0001164619971	0		0	0	https://www.ncbi.nlm.nih.gov/clinvar/variation/167693/
NM_012434.4(SLC17A5):c.507delA	concordant	reportable	reportable	56556	1	0	SCV000699339	Likely pathogenic	Laboratory Corpora	1/25/2018	SLC17A5	chr6	74351431	74351432	http://gnomad.b	2.67E-05	NFE	3.59E-05	0		0	0	https://www.ncbi.nlm.nih.gov/clinvar/variation/56556/
NM_003060.3(SLC22A5):c.67_69delTT	concordant	reportable	reportable	25353	1	0	SCV000632563	Pathogenic	Invitae	10/5/2017	SLC22A5	chr5	131705725	131705728	http://gnomad.b	2.54E-05	AMR	8.95E-05	0		0	0	https://www.ncbi.nlm.nih.gov/clinvar/variation/25353/
NM_003060.3(SLC22A5):c.458_459del	concordant	reportable	reportable	25376	2	0	SCV000704164;S	Pathogenic;Pathogenic	EGL Genetic Diagn	2017-06-30;2018-0	SLC22A5	chr5	131714130	131714132	http://gnomad.b	2.54E-05	FIN	4.48E-05	0		0	0	https://www.ncbi.nlm.nih.gov/clinvar/variation/25376/
NM_000112.3(SLC26A2):c.1020_1022	concordant	reportable	reportable	65558	2	0	SCV000329831;S	Pathogenic;Pathogenic	GeneDx;Laborator	2017-11-28;2018-0	SLC26A2	chr5	149360165	149360168	http://gnomad.b	6.97E-05	EAS	0.0002898889943	0		0	0	https://www.ncbi.nlm.nih.gov/clinvar/variation/65558/
NM_000441.1(SLC26A4):c.279delT	concordant	reportable	reportable	188715	1	0	SCV000605148	Pathogenic	ARUP Laboratories	6/30/2017	SLC26A4	chr7	107303854	107303855	http://gnomad.b	2.79E-05	AMR	2.98E-05	0		0	0	https://www.ncbi.nlm.nih.gov/clinvar/variation/188715/
NM_000543.4(SMPD1):c.573delT	concordant	reportable	reportable	167710	1	0	SCV000697418	Pathogenic	Laboratory Corpora	1/25/2018	SMPD1	chr11	6412867	6412868		7.14E-06					0	0	https://www.ncbi.nlm.nih.gov/clinvar/variation/167710/
NM_000543.4(SMPD1):c.538_539delT	concordant	reportable	reportable	189096	1	0	SCV000697416	Pathogenic	Laboratory Corpora	1/25/2018	SMPD1	chr11	6412831	6412833	http://gnomad.b	2.14E-05	NFE	2.99E-05	0		0	0	https://www.ncbi.nlm.nih.gov/clinvar/variation/189096/
NM_000016.4(ACADM):c.244dupT	concordant	reportable	reportable	188934	1	0	SCV000344747	Pathogenic	EGL Genetic Diagn	11/3/2016	ACADM	chr1	76198564	76198564	http://gnomad.b	4.90E-05	NFE	4.48E-05	0		0	0	https://www.ncbi.nlm.nih.gov/clinvar/variation/188934/
NM_000642.2(AGL):c.3911dupA	concordant	reportable	reportable	370832	1	0	SCV000626744	Pathogenic	Invitae	10/5/2017	AGL	chr1	100378027	100378027		4.47E-05					0	0	https://www.ncbi.nlm.nih.gov/clinvar/variation/370832/
NM_000383.3(AIRE):c.1249dupC	concordant	reportable	reportable	188935	1	0	SCV000329057	Pathogenic	GeneDx	11/28/2017	AIRE	chr21	45713024	45713024	http://gnomad.b	1.39E-05	NFE	4.79E-05	0		0	0	https://www.ncbi.nlm.nih.gov/clinvar/variation/188935/
NM_000487.5(ARSA):c.418dupC	concordant	reportable	reportable	370305	1	0	SCV000710122	Pathogenic	GeneDx	3/26/2018	ARSA	chr22	51065640	51065640		4.22E-05					0	0	https://www.ncbi.nlm.nih.gov/clinvar/variation/370305/
NM_000051.3(ATM):c.1139_1142dup	concordant	reportable	reportable	280044	3	0	SCV000329804;S	Likely pathogenic;Pathoger	GeneDx;Invitae;La	2017-11-28;2017-1	ATM	chr11	108119732	108119732	http://gnomad.b	2.78E-06	NFE	1.79E-05	0		0	0	https://www.ncbi.nlm.nih.gov/clinvar/variation/280044/
NM_000051.3(ATM):c.3085dupA	concordant	reportable	reportable	230318	2	0	SCV000293435;S	Pathogenic;Pathogenic	GeneDx;Invitae	2017-11-28;2017-0	ATM	chr11	108143264	108143264		5.57E-06					0	0	https://www.ncbi.nlm.nih.gov/clinvar/variation/230318/
NM_000051.3(ATM):c.6997dupA	concordant	reportable	reportable	140818	3	0	SCV000682383;S	Pathogenic;Pathogenic;Pat	Color Genomics, In	2017-10-26;2017-1	ATM	chr11	108198392	108198392	http://gnomad.b	1.67E-05	NFE	1.80E-05	0		0	0	https://www.ncbi.nlm.nih.gov/clinvar/variation/140818/
NM_000051.3(ATM):c.5712dupA	concordant	reportable	reportable	141416	3	0	SCV000682282;S	Pathogenic;Pathogenic;Pat	Color Genomics, In	2017-10-26;2017-1	ATM	chr11	108178655	108178655	http://gnomad.b	1.39E-05	NFE	1.79E-05	0		0	0	https://www.ncbi.nlm.nih.gov/clinvar/variation/141416/
NM_000053.3(ATP7B):c.2304dupC	concordant	reportable	reportable	456552	2	0	SCV000626841;S	Pathogenic;Pathogenic	Invitae;Laboratory	2017-10-05;2018-0	ATP7B	chr13	52532497	52532497	http://gnomad.b	0.0001431008552	NFE	0.0001701230067	0		0	0	https://www.ncbi.nlm.nih.gov/clinvar/variation/456552/
NM_000053.3(ATP7B):c.778dupC	concordant	reportable	reportable	188938	1	0	SCV000694470	Pathogenic	Laboratory Corpora	1/25/2018	ATP7B	chr13	52548577	52548577	http://gnomad.b	3.65E-05	NFE	9.02E-06	0		0	0	https://www.ncbi.nlm.nih.gov/clinvar/variation/188938/
NM_000053.3(ATP7B):c.525dupA	concordant	reportable	reportable	370306	1	0	SCV000694468	Pathogenic	Laboratory Corpora	1/25/2018	ATP7B	chr13	52548830	52548830	http://gnomad.b	2.24E-05	EAS	0.0002899559913	0		0	0	https://www.ncbi.nlm.nih.gov/clinvar/variation/370306/
NM_000709.3(BCKDHA):c.117dupC	concordant	reportable	reportable	166741	2	0	SCV000700309;S	Pathogenic;Pathogenic	EGL Genetic Diagn	2017-06-30;2017-1	BCKDHA	chr19	41916543	41916543	http://gnomad.b	7.91E-06	NFE	3.59E-05	0		0	0	https://www.ncbi.nlm.nih.gov/clinvar/variation/166741/
NM_183050.2(BCKDHB):c.93_103dup	concordant	reportable	reportable	189101	1	0	SCV000700013	Likely pathogenic	Laboratory Corpora	1/25/2018	BCKDHB	chr6	80816489	80816489	http://gnomad.b	1.54E-05			0		0	0	https://www.ncbi.nlm.nih.gov/clinvar/variation/189101/
NM_000057.2(BLM):c.1968dupG	concordant	reportable	reportable	495425	1	0	SCV000694475	Likely pathogenic	Laboratory Corpora	1/25/2018	BLM	chr15	91306280	91306280	http://gnomad.b	1.66E-05	NFE	1.79E-05	0		0	0	https://www.ncbi.nlm.nih.gov/clinvar/variation/495425/
NM_000057.3(BLM):c.2407dupT	concordant	reportable	reportable	42177	1	0	SCV000709964	Pathogenic	GeneDx	3/26/2018	BLM	chr15	91312667	91312667	http://gnomad.b	2.38E-06	ASJ	0.0001015640009	0		0	0	https://www.ncbi.nlm.nih.gov/clinvar/variation/42177/
NM_000492.3(CFTR):c.3532_3535dup	concordant	reportable	reportable	7222	1	0	SCV000245890	Pathogenic	CFTR2	6/29/2017	CFTR	chr7	117267638	117267638		3.85E-06					0	0	https://www.ncbi.nlm.nih.gov/clinvar/variation/7222/
NM_000492.3(CFTR):c.1021_1022dup	concordant	reportable	reportable	7134	1	0	SCV000071465	Pathogenic	CFTR2	6/29/2017	CFTR	chr7	117180302	117180302	http://gnomad.b	3.27E-05	NFE	6.28E-05	0		0	0	https://www.ncbi.nlm.nih.gov/clinvar/variation/7134/
NM_000492.3(CFTR):c.1327_1330dup	concordant	reportable	reportable	188958	2	0	SCV000071468;S	Pathogenic;Pathogenic	CFTR2;Quest Diagr	2017-06-29;2017-0	CFTR	chr7	117188810	117188810		9.63E-06					0	0	https://www.ncbi.nlm.nih.gov/clinvar/variation/188958/
NM_000492.3(CFTR):c.2554dupT	concordant	reportable	reportable	371181	1	0	SCV000696906	Pathogenic	Laboratory Corpora	1/25/2018	CFTR	chr7	117235046	117235046		1.93E-06					0	0	https://www.ncbi.nlm.nih.gov/clinvar/variation/371181/
NM_001042432.1(CLN3):c.944dupA	concordant	reportable	reportable	56292	1	0	SCV000549229	Pathogenic	Invitae	3/14/2017	CLN3	chr16	28493665	28493665	http://gnomad.b	2.78E-06	NFE	8.97E-06	0		0	0	https://www.ncbi.nlm.nih.gov/clinvar/variation/56292/
NM_000136.2(FANCC):c.455dupA	concordant	reportable	reportable	409657	2	0	SCV000549961;S	Pathogenic;Likely pathoger	Invitae;Laboratory	2017-10-05;2018-0	FANCC	chr9	97934319	97934319	http://gnomad.b	9.51E-06	NFE	2.69E-05	0		0	0	https://www.ncbi.nlm.nih.gov/clinvar/variation/409657/
NM_000152.3(GAA):c.2242dupG	concordant	reportable	reportable	370651	1	0	SCV000617645	Pathogenic	GeneDx	11/28/2017	GAA	chr17	78090813	78090813	http://gnomad.b	1.67E-05	NFE	1.80E-05	0		0	0	https://www.ncbi.nlm.nih.gov/clinvar/variation/370651/
NM_004004.5(GJB2):c.290dupA	concordant	reportable	reportable	188821	1	0	SCV000599743	Pathogenic	Division of Genomi	5/17/2017	GJB2	chr13	20763430	20763430	http://gnomad.b	1.68E-05	NFE	2.69E-05	0		0	0	https://www.ncbi.nlm.nih.gov/clinvar/variation/188821/
NM_004004.5(GJB2):c.269dupT	concordant	reportable	reportable	177737	2	0	SCV000698240;S	Pathogenic;Pathogenic	Laboratory Corpora	2018-01-25;2016-0	GJB2	chr13	20763451	20763451	http://gnomad.b	3.07E-05	NFE	3.58E-05	0		0	0	https://www.ncbi.nlm.nih.gov/clinvar/variation/177737/
NM_002225.3(IVD):c.879dupG	concordant	reportable	reportable	459932	1	0	SCV000631892	Pathogenic	Invitae	10/5/2017	IVD	chr15	40707167	40707167	http://gnomad.b	1.12E-05	FIN	4.57E-05	0		0	0	https://www.ncbi.nlm.nih.gov/clinvar/variation/459932/
NM_015506.2(MMACHC):c.271dupA	concordant	reportable	reportable	1421	6	0	SCV000604238;S	Pathogenic;Pathogenic;Pat	ARUP Laboratories	2017-06-30;2016-1	MMACHC	chr1	45973216	45973216	http://gnomad.b	0.001345021837	ASJ	0.002438029973	0		0	0	https://www.ncbi.nlm.nih.gov/clinvar/variation/1421/
NM_000271.4(NPC1):c.423_424dupG	concordant	reportable	reportable	503633	1	0	SCV000709826	Pathogenic	GeneDx	3/26/2018	NPC1	chr18	21148825	21148825	http://gnomad.b	8.58E-06	NFE	1.79E-05	0		0	0	https://www.ncbi.nlm.nih.gov/clinvar/variation/503633/
NM_000271.4(NPC1):c.973_974dupG	concordant	reportable	reportable	420124	2	0	SCV000345567;S	Pathogenic;Pathogenic	EGL Genetic Diagn	2016-11-03;2017-1	NPC1	chr18	21136558	21136558		5.72E-06					0	0	https://www.ncbi.nlm.nih.gov/clinvar/variation/420124/
NM_138694.3(PKHD1):c.2180dupA	concordant	reportable	reportable	496517	1	0	SCV000699855	Likely pathogenic	Laboratory Corpora	1/25/2018	PKHD1	chr6	51915053	51915053		2.78E-06					0	0	https://www.ncbi.nlm.nih.gov/clinvar/variation/496517/
NM_000310.3(PPT1):c.169dupA	concordant	reportable	reportable	56182	1	0	SCV000284561	Pathogenic	Invitae	6/10/2016	PPT1	chr1	40558134	40558134	http://gnomad.b	3.06E-05	NFE	6.27E-05	0		0	0	https://www.ncbi.nlm.nih.gov/clinvar/variation/56182/
NM_000232.4(SGCB):c.21_23dupGGC	concordant	unreportable	unreportable	193064	0	1	SCV000224170	Uncertain significance	EGL Genetic Diagn	11/3/2016	SGCB	chr4	52904402	52904402		0.0005037755176					1	0	https://www.ncbi.nlm.nih.gov/clinvar/variation/193064/
NM_000383.3(AIRE):c.132+1_132+3de	concordant	reportable	reportable	279973	1	0	SCV000329648	Pathogenic	GeneDx	11/28/2017	AIRE	chr21	45706021	45706024		5.57E-06					0	0	https://www.ncbi.nlm.nih.gov/clinvar/variation/279973/
NM_000478.4(ALPL):c.400_401delAC	concordant	reportable	reportable	188877	1	0	SCV000329063	Pathogenic	GeneDx	11/28/2017	ALPL	chr1	21889704	21889706		5.63E-05					0	0	https://www.ncbi.nlm.nih.gov/clinvar/variation/188877/
NM_000492.3(CFTR):c.4077_4080delT	concordant	reportable	reportable	53883	2	0	SCV000071485;S	Pathogenic;Pathogenic	CFTR2;Laboratory	2017-06-29;2018-0	CFTR	chr7	117304854	117304858		9.63E-06					0	0	https://www.ncbi.nlm.nih.gov/clinvar/variation/53883/
NM_000303.2(PMM2):c.95_96delTAin	concordant	reportable	reportable	92807	1	0	SCV000696505	Pathogenic	Laboratory Corpora	1/25/2018	PMM2	chr16	8895683	8895685		1.95E-05					0	0	https://www.ncbi.nlm.nih.gov/clinvar/variation/92807/
NM_000018.3(ACADVL):c.1182+1G>A	concordant	reportable	reportable	1622	2	0	SCV000238647;S	Pathogenic;Pathogenic	GeneDx;Invitae	2017-11-28;2017-1	ACADVL	chr17	7126557	7126557	http://gnomad.b	3.11E-05	AFR	6.54E-05	0		0	0	https://www.ncbi.nlm.nih.gov/clinvar/variation/1622/
NM_000018.3(ACADVL):c.1679-6G>A	concordant	reportable	reportable	21019	2	0	SCV000238656;S	Pathogenic;Pathogenic	GeneDx;Invitae	2017-11-28;2017-1	ACADVL	chr17	7127955	7127955		5.66E-06					0	0	https://www.ncbi.nlm.nih.gov/clinvar/variation/21019/
NM_000018.3(ACADVL):c.753-2A>C	concordant	reportable	reportable	92290	2	0	SCV000616632;S	Pathogenic;Pathogenic	GeneDx;Invitae	2017-11-28;2017-1	ACADVL	chr17	7125494	7125494	http://gnomad.b	2.27E-05	NFE	8.95E-06	0		0	0	https://www.ncbi.nlm.nih.gov/clinvar/variation/92290/
NM_000642.2(AGL):c.2309-1G>A	concordant	reportable	reportable	188804	1	0	SCV000697530	Pathogenic	Laboratory Corpora	1/25/2018	AGL	chr1	100349675	100349675	http://gnomad.b	5.58E-06	NFE	1.80E-05	0		0	0	https://www.ncbi.nlm.nih.gov/clinvar/variation/188804/
NM_000642.2(AGL):c.2681+1G>A	concordant	reportable	reportable	281229	2	0	SCV000331829;S	Pathogenic;Pathogenic	EGL Genetic Diagn	2017-06-30;2017-1	AGL	chr1	100350260	100350260	http://gnomad.b	2.23E-05	EAS	0.0001159820022	0		0	0	https://www.ncbi.nlm.nih.gov/clinvar/variation/281229/
NM_000642.2(AGL):c.4260-12A>G	concordant	reportable	reportable	1099	1	0	SCV000626749	Pathogenic	Invitae	10/5/2017	AGL	chr1	100381954	100381954	http://gnomad.b	3.63E-05	AFR	0.0002635050041	0		0	0	https://www.ncbi.nlm.nih.gov/clinvar/variation/1099/
NM_000642.2(AGL):c.664+3A>G	concordant	reportable	reportable	188994	1	0	SCV000697535	Pathogenic	Laboratory Corpora	1/25/2018	AGL	chr1	100330148	100330148	http://gnomad.b	1.95E-05	NFE	1.80E-05	0		0	0	https://www.ncbi.nlm.nih.gov/clinvar/variation/188994/
NM_000383.3(AIRE):c.463+2T>C	concordant	reportable	reportable	188800	1	0	SCV000490397	Pathogenic	GeneDx	12/21/2016	AIRE	chr21	45707018	45707018	http://gnomad.b	1.11E-05	NFE	1.86E-05	0		0	0	https://www.ncbi.nlm.nih.gov/clinvar/variation/188800/
NM_000050.4(ASS1):c.1194-1G>C	concordant	reportable	reportable	166704	1	0	SCV000694162	Pathogenic	Laboratory Corpora	1/25/2018	ASS1	chr9	133376362	133376362	http://gnomad.b	2.51E-05	NFE	2.69E-05	0		0	0	https://www.ncbi.nlm.nih.gov/clinvar/variation/166704/
NM_000051.3(ATM):c.2921+1G>A	concordant	reportable	reportable	141182	4	0	SCV000602557;S	Pathogenic;Pathogenic;Pat	ARUP Laboratories	2017-06-30;2017-1	ATM	chr11	108141874	108141874	http://gnomad.b	2.23E-05	AFR	0.000130786997	0		0	0	https://www.ncbi.nlm.nih.gov/clinvar/variation/141182/
NM_000051.3(ATM):c.4776+2T>C	concordant	reportable	reportable	141672	1	0	SCV000293433	Pathogenic	GeneDx	11/28/2017	ATM	chr11	108164206	108164206	http://gnomad.b	2.78E-06	AMR	2.99E-05	0		0	0	https://www.ncbi.nlm.nih.gov/clinvar/variation/141672/
NM_000051.3(ATM):c.5497-2A>C	concordant	reportable	reportable	449345	2	0	SCV000617369;S	Pathogenic;Likely pathoger	GeneDx;Invitae	2017-11-28;2017-1	ATM	chr11	108175400	108175400		2.78E-06					0	0	https://www.ncbi.nlm.nih.gov/clinvar/variation/449345/
NM_000051.3(ATM):c.6154G>A	concordant	unreportable	unreportable	181975	1	1	SCV000209758;S	Uncertain significance;Likel	GeneDx;Invitae	2017-11-28;2017-1	ATM	chr11	108186796	108186796	http://gnomad.b	4.73E-05	SAS	0.0003898889991	0		0	0	https://www.ncbi.nlm.nih.gov/clinvar/variation/181975/
NM_000051.3(ATM):c.7789-3T>G	concordant	reportable	reportable	219629	2	0	SCV000682436;S	Pathogenic;Pathogenic	Color Genomics, In	2017-10-26;2017-1	ATM	chr11	108203486	108203486		5.57E-06					0	0	https://www.ncbi.nlm.nih.gov/clinvar/variation/219629/
NM_000051.3(ATM):c.8786+1G>A	concordant	reportable	reportable	127463	3	0	SCV000682516;S	Pathogenic;Pathogenic;Pat	Color Genomics, In	2017-10-26;2017-1	ATM	chr11	108224608	108224608	http://gnomad.b	1.11E-05	NFE	1.79E-05	0		0	0	https://www.ncbi.nlm.nih.gov/clinvar/variation/127463/
NM_000051.3(ATM):c.901+1G>A	concordant	reportable	reportable	186761	2	0	SCV000611170;S	Pathogenic;Pathogenic	Fulgent Genetics,F	2017-05-23;2017-0	ATM	chr11	108115754	108115754	http://gnomad.b	1.39E-05	AMR	8.98E-05	0		0	0	https://www.ncbi.nlm.nih.gov/clinvar/variation/186761/
NM_000053.3(ATP7B):c.2575+1G>C	concordant	reportable	reportable	188749	1	0	SCV000694425	Likely pathogenic	Laboratory Corpora	1/25/2018	ATP7B	chr13	52524407	52524407	http://gnomad.b	8.42E-06	NFE	1.79E-05	0		0	0	https://www.ncbi.nlm.nih.gov/clinvar/variation/188749/
NM_000053.3(ATP7B):c.3556+1G>A	concordant	reportable	reportable	189055	1	0	SCV000694449	Likely pathogenic	Laboratory Corpora	1/25/2018	ATP7B	chr13	52515216	52515216	http://gnomad.b	1.40E-05	EAS	5.80E-05	0		0	0	https://www.ncbi.nlm.nih.gov/clinvar/variation/189055/
NM_024685.3(BBS10):c.197+1G>T	concordant	reportable	reportable	283884	1	0	SCV000336240	Pathogenic	EGL Genetic Diagn	6/30/2017	BBS10	chr12	76741941	76741941		2.80E-06					0	0	https://www.ncbi.nlm.nih.gov/clinvar/variation/283884/
NM_000709.3(BCKDHA):c.647-1G>C	concordant	reportable	reportable	431985	1	0	SCV000589622	Pathogenic	GeneDx	11/28/2017	BCKDHA	chr19	41928068	41928068	http://gnomad.b	1.58E-05	NFE	9.08E-06	0		0	0	https://www.ncbi.nlm.nih.gov/clinvar/variation/431985/
NM_004328.4(BCS1L):c.320+1G>T	concordant	reportable	reportable	56412	1	0	SCV000576973	Likely pathogenic	GeneDx	11/28/2017	BCS1L	chr2	219526031	219526031	http://gnomad.b	3.35E-05	AFR	6.54E-05	0		0	0	https://www.ncbi.nlm.nih.gov/clinvar/variation/56412/
NM_000071.2(CBS):c.1224-2A>C	concordant	reportable	reportable	128	3	0	SCV000225996;S	Pathogenic;Pathogenic;Pat	EGL Genetic Diagn	2017-06-30;2017-1	CBS	chr21	44479080	44479080	http://gnomad.b	0.0002052764483	ASJ	0.001412739977	0		0	0	https://www.ncbi.nlm.nih.gov/clinvar/variation/128/
NM_000492.3(CFTR):c.53+1G>T	concordant	reportable	reportable	53988	1	0	SCV000245957	Pathogenic	CFTR2	6/29/2017	CFTR	chr7	117120202	117120202	http://gnomad.b	1.93E-06	SAS	3.25E-05	0		0	0	https://www.ncbi.nlm.nih.gov/clinvar/variation/53988/
NM_000492.3(CFTR):c.1766+3A>G	concordant	reportable	reportable	53379	3	0	SCV000071569;S	Pathogenic;Pathogenic;Pat	CFTR2;EGL Genetic	2017-06-29;2017-0	CFTR	chr7	117230496	117230496		1.93E-06					0	0	https://www.ncbi.nlm.nih.gov/clinvar/variation/53379/
NM_000492.3(CFTR):c.2490+1G>A	concordant	reportable	reportable	7175	3	0	SCV000071455;S	Pathogenic;Pathogenic;Pat	CFTR2;Invitae;Labc	2017-06-29;2017-0	CFTR	chr7	117232712	117232712	http://gnomad.b	2.50E-05	NFE	2.18E-05	0		0	0	https://www.ncbi.nlm.nih.gov/clinvar/variation/7175/
NM_000492.3(CFTR):c.2989-1G>A	concordant	reportable	reportable	53613	1	0	SCV000071457	Pathogenic	CFTR2	6/29/2017	CFTR	chr7	117250572	117250572	http://gnomad.b	3.85E-06	NFE	8.97E-06	0		0	0	https://www.ncbi.nlm.nih.gov/clinvar/variation/53613/
NM_000492.3(CFTR):c.3368-2A>G	concordant	reportable	reportable	188783	1	0	SCV000245940	Pathogenic	CFTR2	6/29/2017	CFTR	chr7	117254665	117254665	http://gnomad.b	1.16E-05	AMR	2.98E-05	0		0	0	https://www.ncbi.nlm.nih.gov/clinvar/variation/188783/
NM_000492.3(CFTR):c.3718-2530A>G	concordant	unreportable	unreportable	237856	0	2	SCV000703051;S	Benign;Benign	EGL Genetic Diagn	2017-06-30;2016-0	CFTR	chr7	117279962	117279962		0.001359637635					7	0	https://www.ncbi.nlm.nih.gov/clinvar/variation/237856/
NM_000492.3(CFTR):c.165-1G>A	concordant	reportable	reportable	53314	3	0	SCV000245916;S	Pathogenic;Pathogenic;Pat	CFTR2;EGL Genetic	2017-06-29;2017-0	CFTR	chr7	117149087	117149087		1.93E-06					0	0	https://www.ncbi.nlm.nih.gov/clinvar/variation/53314/
NM_000492.3(CFTR):c.3873+1G>A	concordant	reportable	reportable	7160	1	0	SCV000071458	Pathogenic	CFTR2	6/29/2017	CFTR	chr7	117282648	117282648		3.85E-06					0	0	https://www.ncbi.nlm.nih.gov/clinvar/variation/7160/
NM_000492.3(CFTR):c.1393-2A>G	concordant	reportable	reportable	53243	2	0	SCV000245956;S	Pathogenic;Pathogenic	CFTR2;Invitae	2017-06-29;2017-0	CFTR	chr7	117199516	117199516	http://gnomad.b	1.93E-06	AFR	6.54E-05	0		0	0	https://www.ncbi.nlm.nih.gov/clinvar/variation/53243/
NM_001360.2(DHCR7):c.964-1G>T	concordant	reportable	reportable	305956	1	0	SCV000373913	Likely pathogenic	Illumina Clinical Se	10/18/2016	DHCR7	chr11	71146886	71146886	http://gnomad.b	5.12E-05	FIN	0.0005883739796	0		0	0	https://www.ncbi.nlm.nih.gov/clinvar/variation/305956/
NM_000151.3(G6PC):c.648G>T	concordant	reportable	reportable	12003	1	0	SCV000695640	Pathogenic	Laboratory Corpora	1/25/2018	G6PC	chr17	41063017	41063017	http://gnomad.b	7.41E-05	EAS	0.00121767004	0		0	0	https://www.ncbi.nlm.nih.gov/clinvar/variation/12003/
NM_000152.3(GAA):c.1552-3C>G	concordant	reportable	reportable	419722	1	0	SCV000701459	Likely pathogenic	EGL Genetic Diagn	6/30/2017	GAA	chr17	78084737	78084737	http://gnomad.b	0.0001500825454	NFE	0.0002776139881	0		0	0	https://www.ncbi.nlm.nih.gov/clinvar/variation/419722/
NM_000152.3(GAA):c.1438-1G>C	concordant	reportable	reportable	189184	1	0	SCV000708298	Pathogenic	EGL Genetic Diagn	6/30/2017	GAA	chr17	78084525	78084525		2.78E-05					0	0	https://www.ncbi.nlm.nih.gov/clinvar/variation/189184/
NM_000159.2(GCDH):c.1244-2A>C	concordant	reportable	reportable	193976	2	0	SCV000617681;S	Pathogenic;Pathogenic	GeneDx;Laborator	2017-11-28;2018-0	GCDH	chr19	13010280	13010280	http://gnomad.b	3.65E-05	EAS	0.00115955004	0		0	0	https://www.ncbi.nlm.nih.gov/clinvar/variation/193976/
NM_004004.5(GJB2):c.-23+1G>A	concordant	reportable	reportable	17029	4	0	SCV000599720;S	Pathogenic;Pathogenic;Pat	Division of Genomi	2017-05-17;2017-1	GJB2	chr13	20766921	20766921		0.0004945487871					0	0	https://www.ncbi.nlm.nih.gov/clinvar/variation/17029/
NM_004004.5(GJB2):c.-23G>T	concordant	reportable	reportable	189155	1	0	SCV000271226	Likely pathogenic	Laboratory for Mol	4/26/2016	GJB2	chr13	20766922	20766922		2.79E-05					0	0	https://www.ncbi.nlm.nih.gov/clinvar/variation/189155/
NM_000170.2(GLDC):c.2316-1G>A	concordant	reportable	reportable	56073	1	0	SCV000636382	Pathogenic	Invitae	10/5/2017	GLDC	chr9	6553510	6553510	http://gnomad.b	8.00E-06	NFE	7.25E-05	0		0	0	https://www.ncbi.nlm.nih.gov/clinvar/variation/56073/
NM_000182.4(HADHA):c.180+3A>G	concordant	reportable	reportable	8731	1	0	SCV000238890	Pathogenic	GeneDx	11/28/2017	HADHA	chr2	26461799	26461799	http://gnomad.b	3.93E-05	NFE	9.85E-05	0		0	0	https://www.ncbi.nlm.nih.gov/clinvar/variation/8731/
NM_000182.4(HADHA):c.919-2A>G	concordant	reportable	reportable	193564	1	0	SCV000238885	Pathogenic	GeneDx	11/28/2017	HADHA	chr2	26435497	26435497	http://gnomad.b	1.40E-05	NFE	8.96E-05	0		0	0	https://www.ncbi.nlm.nih.gov/clinvar/variation/193564/
NM_000518.4(HBB):c.33C>A	concordant	unreportable	unreportable	439155	0	1	SCV000601292	Uncertain significance	Quest Diagnostics I	8/1/2017	HBB	chr11	5248219	5248219	http://gnomad.b	1.63E-05	SAS	0.0002923790016	0		0	0	https://www.ncbi.nlm.nih.gov/clinvar/variation/439155/
NM_000518.4(HBB):c.92G>C	concordant	reportable	reportable	15234	3	0	SCV000603906;S	Pathogenic;Pathogenic;Pat	ARUP Laboratories	2017-06-30;2018-0	HBB	chr11	5248160	5248160	http://gnomad.b	3.03E-05	SAS	0.0007797270082	0		0	0	https://www.ncbi.nlm.nih.gov/clinvar/variation/15234/
NM_000518.4(HBB):c.92+1G>T	concordant	reportable	reportable	15437	3	0	SCV000603902;S	Pathogenic;Pathogenic;Pat	ARUP Laboratories	2017-06-30;2018-0	HBB	chr11	5248159	5248159	http://gnomad.b	3.97E-05	SAS	0.0005847950233	0		0	0	https://www.ncbi.nlm.nih.gov/clinvar/variation/15437/
NM_000518.4(HBB):c.92+5G>T	concordant	reportable	reportable	15448	2	0	SCV000603942;S	Pathogenic;Pathogenic	ARUP Laboratories	2017-06-30;2017-0	HBB	chr11	5248155	5248155	http://gnomad.b	1.40E-05	NFE	8.97E-06	0		0	0	https://www.ncbi.nlm.nih.gov/clinvar/variation/15448/
NM_000518.4(HBB):c.316-3C>A	concordant	reportable	reportable	15451	1	0	SCV000601284	Pathogenic	Quest Diagnostics I	8/1/2017	HBB	chr11	5246959	5246959	http://gnomad.b	9.33E-06			0		0	0	https://www.ncbi.nlm.nih.gov/clinvar/variation/15451/
NM_000520.4(HEXA):c.460-1G>T	concordant	reportable	reportable	496011	1	0	SCV000697169	Pathogenic	Laboratory Corpora	1/25/2018	HEXA	chr15	72645520	72645520	http://gnomad.b	1.64E-05	AFR	0.0001992299949	0		0	0	https://www.ncbi.nlm.nih.gov/clinvar/variation/496011/

Supplemental Table 2

NM_002225.3(IVD):c.465+2T>C	concordant	reportable	reportable	94055	1	0	SCV000631888	Pathogenic	Invitae	10/5/2017	IVD	chr15	40702998	40702998	http://gnomad.b	1.96E-05	AMR	5.96E-05	0		0	0	https://www.ncbi.nlm.nih.gov/clinvar/variation/94055/
NM_052845.3(MMAB):c.197-1G>T	concordant	reportable	reportable	219008	1	0	SCV000699779	Pathogenic	Laboratory Corpora	1/25/2018	MMAB	chr12	110006669	110006669	http://gnomad.b	8.00E-06	SAS	0.000259908993	0		0	0	https://www.ncbi.nlm.nih.gov/clinvar/variation/219008/
NM_000271.4(NPC1):c.1553G>A	concordant	reportable	reportable	132890	1	0	SCV000339053	Pathogenic	EGL Genetic Diagn	6/30/2017	NPC1	chr18	21134722	21134722	http://gnomad.b	2.86E-06	NFE	8.98E-06	0		0	0	https://www.ncbi.nlm.nih.gov/clinvar/variation/132890/
NM_004646.3(NPHS1):c.2335-1G>A	concordant	reportable	reportable	188734	2	0	SCV000709982;S	Pathogenic;Pathogenic	GeneDx;Laborator	2018-03-26;2018-0	NPHS1	chr19	36333453	36333453	http://gnomad.b	0.0001393774844	NFE	0.0001883410005	0		0	0	https://www.ncbi.nlm.nih.gov/clinvar/variation/188734/
NM_000531.5(OTC):c.298+5G>C	concordant	unreportable	unreportable	97162	0	4	SCV000334662;S	Benign;Benign;Likely benig	EGL Genetic Diagn	2017-06-30;2017-1	OTC	chrX	38229135	38229135	http://gnomad.b	0.001820911424	EAS	0.02128490061	0	98	2	30	https://www.ncbi.nlm.nih.gov/clinvar/variation/97162/
NM_000277.1(PAH):c.1198A>C	concordant	unreportable	unreportable	102553	0	1	SCV000331126	Uncertain significance	EGL Genetic Diagn	6/30/2017	PAH	chr12	103237425	103237425	http://gnomad.b	5.48E-06	AFR	6.54E-05	0		0	0	https://www.ncbi.nlm.nih.gov/clinvar/variation/102553/
NM_000277.1(PAH):c.1197A>T	concordant	reportable	reportable	601	2	0	SCV000708636;S	Pathogenic;Pathogenic	EGL Genetic Diagn	2017-06-30;2018-0	PAH	chr12	103237426	103237426	http://gnomad.b	2.74E-05	EAS	5.80E-05	0		0	0	https://www.ncbi.nlm.nih.gov/clinvar/variation/601/
NM_000277.1(PAH):c.1199+1G>C	concordant	reportable	reportable	102557	1	0	SCV000629177	Pathogenic	Invitae	10/5/2017	PAH	chr12	103237423	103237423	http://gnomad.b	5.48E-06	SAS	3.25E-05	0		0	0	https://www.ncbi.nlm.nih.gov/clinvar/variation/102557/
NM_000277.1(PAH):c.441+1G>A	concordant	reportable	reportable	102671	1	0	SCV000696448	Pathogenic	Laboratory Corpora	1/25/2018	PAH	chr12	103271239	103271239	http://gnomad.b	1.10E-05	NFE	8.95E-06	0		0	0	https://www.ncbi.nlm.nih.gov/clinvar/variation/102671/
NM_000277.1(PAH):c.842+1G>A	concordant	reportable	reportable	599	1	0	SCV000629220	Pathogenic	Invitae	10/5/2017	PAH	chr12	103246592	103246592	http://gnomad.b	4.11E-05	NFE	8.07E-05	0		0	0	https://www.ncbi.nlm.nih.gov/clinvar/variation/599/
NM_000277.1(PAH):c.912+1G>A	concordant	reportable	reportable	92752	3	0	SCV000110393;S	Pathogenic;Pathogenic;Pat	EGL Genetic Diagn	2017-06-30;2017-1	PAH	chr12	103245464	103245464	http://gnomad.b	1.64E-05	SAS	3.25E-05	0		0	0	https://www.ncbi.nlm.nih.gov/clinvar/variation/92752/
NM_000277.1(PAH):c.913-7A>G	concordant	reportable	reportable	102894	2	0	SCV000703341;S	Pathogenic;Pathogenic	EGL Genetic Diagn	2017-06-30;2018-0	PAH	chr12	103240736	103240736	http://gnomad.b	1.10E-05	NFE	2.69E-05	0		0	0	https://www.ncbi.nlm.nih.gov/clinvar/variation/102894/
NM_000466.2(PEX1):c.2926+1G>A	concordant	reportable	reportable	188729	2	0	SCV000340724;S	Pathogenic;Likely pathoger	EGL Genetic Diagn	2017-06-30;2018-0	PEX1	chr7	92123800	92123800	http://gnomad.b	1.94E-05	NFE	4.48E-05	0		0	0	https://www.ncbi.nlm.nih.gov/clinvar/variation/188729/
NM_000303.2(PMM2):c.255+2T>C	concordant	reportable	reportable	321216	1	0	SCV000399670	Likely pathogenic	Illumina Clinical Se	10/18/2016	PMM2	chr16	8898702	8898702	http://gnomad.b	6.68E-05	AMR	0.0001488980051	0		0	0	https://www.ncbi.nlm.nih.gov/clinvar/variation/321216/
NM_017739.3(POMGNT1):c.636C>T	concordant	reportable	reportable	265399	1	0	SCV000322258	Pathogenic	GeneDx	11/28/2017	POMGNT1	chr1	46660532	46660532	http://gnomad.b	4.20E-05	NFE	6.27E-05	0		0	0	https://www.ncbi.nlm.nih.gov/clinvar/variation/265399/
NM_000441.1(SLC26A4):c.1614C>T	concordant	unreportable	unreportable	43517	0	1	SCV000333398	Uncertain significance	EGL Genetic Diagn	6/30/2017	SLC26A4	chr7	107338556	107338556	http://gnomad.b	0.0002177809793	AFR	0.002549020108	0		0	0	https://www.ncbi.nlm.nih.gov/clinvar/variation/43517/
NM_000441.1(SLC26A4):c.1614+1G>A	concordant	reportable	reportable	43516	2	0	SCV000329897;S	Pathogenic;Pathogenic	GeneDx;Laborator	2017-11-28;2016-0	SLC26A4	chr7	107338557	107338557	http://gnomad.b	2.79E-05	NFE	4.49E-05	0		0	0	https://www.ncbi.nlm.nih.gov/clinvar/variation/43516/
NM_000441.1(SLC26A4):c.2089+1G>A	concordant	reportable	reportable	165263	1	0	SCV000582142	Pathogenic	GeneDx	11/28/2017	SLC26A4	chr7	107344831	107344831	http://gnomad.b	1.40E-05	AFR	0.0001489870047	0		0	0	https://www.ncbi.nlm.nih.gov/clinvar/variation/165263/
NM_000441.1(SLC26A4):c.416-1G>A	concordant	reportable	reportable	371079	1	0	SCV000680758	Pathogenic	GeneDx	2/7/2018	SLC26A4	chr7	107314608	107314608	http://gnomad.b	8.38E-06	EAS	5.80E-05	0		0	0	https://www.ncbi.nlm.nih.gov/clinvar/variation/371079/
NM_000441.1(SLC26A4):c.919-2A>G	concordant	reportable	reportable	4840	1	0	SCV000700797	Pathogenic	EGL Genetic Diagn	6/30/2017	SLC26A4	chr7	107323898	107323898	http://gnomad.b	0.0004076413203	EAS	0.004466359969	0		1	0	https://www.ncbi.nlm.nih.gov/clinvar/variation/4840/
NM_000391.3(TPP1):c.381-2A>G	concordant	reportable	reportable	495859	1	0	SCV000696664	Likely pathogenic	Laboratory Corpora	1/25/2018	TPP1	chr11	6638661	6638661		2.80E-06					0	0	https://www.ncbi.nlm.nih.gov/clinvar/variation/495859/
NM_017890.4(VPS13B):c.6732+1G>A	concordant	reportable	reportable	56683	3	0	SCV000329305;S	Pathogenic;Pathogenic;Pat	GeneDx;Genetic Se	2017-11-28;2017-0	VPS13B	chr8	100729602	100729602	http://gnomad.b	7.28E-05	NFE	7.19E-05	0		0	0	https://www.ncbi.nlm.nih.gov/clinvar/variation/56683/
NM_000310.3(PPT1):c.535C>T	concordant	unreportable	unreportable	206647	0	1	SCV000614762	Uncertain significance	Athena Diagnostics	8/17/2017	PPT1	chr1	40555083	40555083	http://gnomad.b	0.0003510746786	SAS	0.002501460025	1		4	0	https://www.ncbi.nlm.nih.gov/clinvar/variation/206647/
NM_000391.3(TPP1):c.1253G>A	concordant	unreportable	unreportable	137703	0	2	SCV000615824;S	Likely benign;Benign	Athena Diagnostics	2017-08-17;2017-1	TPP1	chr11	6636686	6636686	http://gnomad.b	0.001594842782	AFR	0.01489809994	1		1	0	https://www.ncbi.nlm.nih.gov/clinvar/variation/137703/
NM_138694.3(PKHD1):c.4035C>A	concordant	unreportable	unreportable	196803	0	1	SCV000260369	Benign	Invitae	10/5/2017	PKHD1	chr6	51890573	51890573	http://gnomad.b	0.00988835637	SAS	0.03622480109	41		39	0	https://www.ncbi.nlm.nih.gov/clinvar/variation/196803/
NM_000520.4(HEXA):c.1435G>A	concordant	unreportable	unreportable	287080	0	1	SCV000340739	Benign	EGL Genetic Diagn	6/30/2017	HEXA	chr15	72637878	72637878	http://gnomad.b	0.001394569163	EAS	0.008174859919	3		5	0	https://www.ncbi.nlm.nih.gov/clinvar/variation/287080/
NM_000466.2(PEX1):c.2088A>G	concordant	unreportable	unreportable	129882	0	2	SCV000152174;S	Likely benign;Likely benign	Genetic Services La	2014-06-27;2016-1	PEX1	chr7	92132493	92132493	http://gnomad.b	0.03200281782	ASJ	0.0430806987	121		219	0	https://www.ncbi.nlm.nih.gov/clinvar/variation/129882/
NM_014363.4(SACS):c.1373C>T	concordant	unreportable	unreportable	282385	0	4	SCV000614939;S	Likely benign;Uncertain sig	Athena Diagnostics	2017-08-17;2017-0	SACS	chr13	23929378	23929378	http://gnomad.b	0.004360120459	ASJ	0.02223349921	1		9	0	https://www.ncbi.nlm.nih.gov/clinvar/variation/282385/
NM_000060.2(BTD):c.645C>T	concordant	unreportable	unreportable	25032	0	2	SCV000441825;S	Uncertain significance;Beni	Illumina Clinical Se	2016-10-18;2017-1	BTD	chr3	15686008	15686008	http://gnomad.b	0.01011711519	ASJ	0.03310320154	12		42	0	https://www.ncbi.nlm.nih.gov/clinvar/variation/25032/
NM_000383.3(AIRE):c.1296G>A	concordant	unreportable	unreportable	35659	0	2	SCV000593078;S	Likely benign;Benign	Genetic Services La	2017-07-05;2017-1	AIRE	chr21	45713689	45713689	http://gnomad.b	0.0006043659171	AFR	0.006073140074	0		3	0	https://www.ncbi.nlm.nih.gov/clinvar/variation/35659/
NM_138694.3(PKHD1):c.6499C>A	concordant	unreportable	unreportable	286801	0	1	SCV000340366	Uncertain significance	EGL Genetic Diagn	6/30/2017	PKHD1	chr6	51774264	51774264	http://gnomad.b	0.000102858922	EAS	0.0002899559913	0		0	0	https://www.ncbi.nlm.nih.gov/clinvar/variation/286801/
NM_000360.3(TH):c.684G>A	concordant	unreportable	unreportable	242255	0	3	SCV000342333;S	Benign;Uncertain significar	EGL Genetic Diagn	2017-06-30;2016-1	TH	chr11	2188676	2188676	http://gnomad.b	0.005828464542	FIN	0.00932576973	5		6	0	https://www.ncbi.nlm.nih.gov/clinvar/variation/242255/
NM_001079802.1(FKTN):c.42G>A	concordant	unreportable	unreportable	93521	0	2	SCV000111315;S	Benign;Benign	EGL Genetic Diagn	2017-06-30;2017-1	FKTN	chr9	108337355	108337355	http://gnomad.b	0.002584904724	AFR	0.03262289986	7		9	0	https://www.ncbi.nlm.nih.gov/clinvar/variation/93521/
NM_002225.3(IVD):c.97C>T	concordant	unreportable	unreportable	258592	0	3	SCV000330969;S	Benign;Benign;Likely benig	EGL Genetic Diagn	2017-06-30;2017-1	IVD	chr15	40698116	40698116	http://gnomad.b	0.002339813824	AFR	0.03059850074	9		11	0	https://www.ncbi.nlm.nih.gov/clinvar/variation/258592/
NM_000051.3(ATM):c.735C>T	concordant	unreportable	unreportable	183707	0	3	SCV000537370;S	Benign;Likely benign;Benig	Color Genomics, In	2017-01-26;2016-1	ATM	chr11	108115587	108115587	http://gnomad.b	0.007067590447	FIN	0.04170409963	41		18	0	https://www.ncbi.nlm.nih.gov/clinvar/variation/183707/
NM_001079802.1(FKTN):c.1336A>G	concordant	unreportable	unreportable	36138	0	3	SCV000247383;S	Likely benign;Benign;Benig	Genetic Services La	2015-09-15;2017-1	FKTN	chr9	108397495	108397495	http://gnomad.b	0.01150264873	SAS	0.02329589985	29		28	0	https://www.ncbi.nlm.nih.gov/clinvar/variation/36138/
NM_000182.4(HADHA):c.-1G>T	concordant	unreportable	unreportable	92593	0	2	SCV000110179;S	Likely benign;Likely benign	EGL Genetic Diagn	2017-06-30;2017-1	HADHA	chr2	26467465	26467465	http://gnomad.b	0.002428001077	NFE	0.004504620098	0		4	0	https://www.ncbi.nlm.nih.gov/clinvar/variation/92593/
NM_000098.2(CPT2):c.1767G>A	concordant	unreportable	unreportable	92432	0	1	SCV000632596	Benign	Invitae	10/5/2017	CPT2	chr1	53679057	53679057	http://gnomad.b	0.005778066292	AFR	0.02594090067	8		24	0	https://www.ncbi.nlm.nih.gov/clinvar/variation/92432/
NM_138694.3(PKHD1):c.11525G>T	concordant	unreportable	unreportable	96371	0	2	SCV000557643;S	Benign;Benign	Invitae;Laboratory	2017-10-05;2018-0	PKHD1	chr6	51497503	51497503	http://gnomad.b	0.01894272148	FIN	0.04176760092	65		96	0	https://www.ncbi.nlm.nih.gov/clinvar/variation/96371/
NM_000071.2(CBS):c.1643G>A	concordant	unreportable	unreportable	212869	0	3	SCV000317729;S	Likely benign;Likely benign,	Ambry Genetics;Ge	2017-11-14;2017-1	CBS	chr21	44474003	44474003	http://gnomad.b	0.001673143654	NFE	0.002472290071	1		0	0	https://www.ncbi.nlm.nih.gov/clinvar/variation/212869/
NM_000642.2(AGL):c.1908A>G	concordant	unreportable	unreportable	291334	0	3	SCV000527195;S	Likely benign;Uncertain sig	GeneDx;Illumina C	2017-11-28;2016-1	AGL	chr1	100346640	100346640	http://gnomad.b	0.000390740565	FIN	0.002737639938	1		0	0	https://www.ncbi.nlm.nih.gov/clinvar/variation/291334/
NM_000136.2(FANCC):c.29G>A	concordant	unreportable	unreportable	127538	0	3	SCV000345144;S	Uncertain significance;Unc	EGL Genetic Diagn	2017-06-30;2017-1	FANCC	chr9	98011545	98011545	http://gnomad.b	0.0003876319257	SAS	0.001494570053	0		0	0	https://www.ncbi.nlm.nih.gov/clinvar/variation/127538/
NM_004937.2(CTNS):c.444G>A	concordant	unreportable	unreportable	322840	0	1	SCV000402224	Uncertain significance	Illumina Clinical Se	10/18/2016	CTNS	chr17	3558629	3558629	http://gnomad.b	0.0001185213279	FIN	0.0009865469765	0		0	0	https://www.ncbi.nlm.nih.gov/clinvar/variation/322840/
NM_000152.3(GAA):c.859-21G>A	concordant	unreportable	unreportable	495670	0	1	SCV000695668	Benign	Laboratory Corpora	1/25/2018	GAA	chr17	78081578	78081578	http://gnomad.b	0.001331287764	AFR	0.01659660041	1		2	0	https://www.ncbi.nlm.nih.gov/clinvar/variation/495670/
NM_020533.2(MCOLN1):c.782C>T	concordant	unreportable	unreportable	283177	0	2	SCV000335116;S	Benign;Uncertain significar	EGL Genetic Diagn	2017-06-30;2016-1	MCOLN1	chr19	7593048	7593048	http://gnomad.b	0.003463467083	AMR	0.005121789873	3		5	0	https://www.ncbi.nlm.nih.gov/clinvar/variation/283177/
NM_000391.3(TPP1):c.840G>C	concordant	unreportable	unreportable	92885	0	2	SCV000373331;S	Uncertain significance;Beni	Illumina Clinical Se	2016-10-18;2017-1	TPP1	chr11	6637938	6637938	http://gnomad.b	0.002753202276	NFE	0.003893940011	3		5	0	https://www.ncbi.nlm.nih.gov/clinvar/variation/92885/
NM_001173990.2(TMEM216):c.253C>	concordant	unreportable	unreportable	282947	0	2	SCV000334696;S	Benign;Benign	EGL Genetic Diagn	2017-06-30;2017-1	TMEM216	chr11	61165269	61165269	http://gnomad.b	0.002815300839	EAS	0.03756960109	18		16	0	https://www.ncbi.nlm.nih.gov/clinvar/variation/282947/
NM_000153.3(GALC):c.96G>T	concordant	unreportable	unreportable	314760	0	2	SCV000700288;S	Benign;Uncertain significar	EGL Genetic Diagn	2017-06-30;2016-1	GALC	chr14	88459413	88459413	http://gnomad.b	0.001292249857	AFR	0.01075149979	1		0	0	https://www.ncbi.nlm.nih.gov/clinvar/variation/314760/
NM_000098.2(CPT2):c.236A>C	concordant	unreportable	unreportable	460433	0	1	SCV000632603	Uncertain significance	Invitae	10/5/2017	CPT2	chr1	53667997	53667997	http://gnomad.b	0.0003693485475	ASJ	0.001015229966	1		0	0	https://www.ncbi.nlm.nih.gov/clinvar/variation/460433/
NM_014363.4(SACS):c.4076T>C	concordant	unreportable	unreportable	193706	0	3	SCV000614954;S	Uncertain significance;Unc	Athena Diagnostics	2017-08-17;2017-0	SACS	chr13	23913939	23913939	http://gnomad.b	0.001502774353	NFE	0.003421579953	3		0	0	https://www.ncbi.nlm.nih.gov/clinvar/variation/193706/
NM_000487.5(ARSA):c.1002C>T	concordant	unreportable	unreportable	280986	0	2	SCV000330993;S	Benign;Uncertain significar	EGL Genetic Diagn	2017-06-30;2016-1	ARSA	chr22	51064469	51064469	http://gnomad.b	0.0043783872	SAS	0.02050619945	17		14	0	https://www.ncbi.nlm.nih.gov/clinvar/variation/280986/
NM_000478.4(ALPL):c.1542G>T	concordant	unreportable	unreportable	256229	0	1	SCV000354318	Likely benign	Illumina Clinical Se	10/18/2016	ALPL	chr1	21904108	21904108	http://gnomad.b	0.00814721327	FIN	0.01762410067	17		23	0	https://www.ncbi.nlm.nih.gov/clinvar/variation/256229/
NM_000051.3(ATM):c.6919C>T	concordant	unreportable	unreportable	127430	0	4	SCV000342311;S	Uncertain significance;Likel	EGL Genetic Diagn	2017-06-30;2017-0	ATM	chr11	108196896	108196896	http://gnomad.b	0.002935082873	ASJ	0.03077390045	3		12	0	https://www.ncbi.nlm.nih.gov/clinvar/variation/127430/
NM_000053.3(ATP7B):c.2175G>A	concordant	unreportable	unreportable	157936	0	4	SCV000345552;S	Likely benign;Likely benign,	EGL Genetic Diagn	2017-06-30;2017-1	ATP7B	chr13	52532627	52532627	http://gnomad.b	0.002890076096	FIN	0.006008970086	3		4	0	https://www.ncbi.nlm.nih.gov/clinvar/variation/157936/
NM_015166.3(MLC1):c.279G>A	concordant	unreportable	unreportable	342108	0	1	SCV000439242	Uncertain significance	Illumina Clinical Se	10/18/2016	MLC1	chr22	50518815	50518815	http://gnomad.b	0.006829699404	ASJ	0.01512609981	10		14	0	https://www.ncbi.nlm.nih.gov/clinvar/variation/342108/
NM_000053.3(ATP7B):c.4302G>A	concordant	unreportable	unreportable	35731	0	2	SCV000526109;S	Benign;Benign	GeneDx;Invitae	2017-11-28;2017-1	ATP7B	chr13	52508988	52508988	http://gnomad.b	0.003462479517	AFR	0.03803920001	14		24	0	https://www.ncbi.nlm.nih.gov/clinvar/variation/35731/
NM_000360.3(TH):c.1035G>T	concordant	unreportable	unreportable	304073	0	3	SCV000615802;S	Benign;Likely benign;Benig	Athena Diagnostics	2017-08-17;2016-1	TH	chr11	2187722	2187722	http://gnomad.b	0.004650940048	AFR	0.05285039917	17		29	0	https://www.ncbi.nlm.nih.gov/clinvar/variation/304073/
NM_033056.3(PCDH15):c.2625G>A	concordant	unreportable	unreportable	46454	0	2	SCV000340195;S	Likely benign;Uncertain sig	EGL Genetic Diagn	2016-11-03;2016-1	PCDH15	chr10	55780078	55780078	http://gnomad.b	0.003754564625	NFE	0.005793309771	2		3	0	https://www.ncbi.nlm.nih.gov/clinvar/variation/46454/
NM_133647.1(SLC12A6):c.318C>T	concordant	unreportable	unreportable	382639	0	1	SCV000522634	Likely benign	GeneDx	11/28/2017	SLC12A6	chr15	34553220	34553220	http://gnomad.b	0.001665402304	AFR	0.01725500077	1		3	0	https://www.ncbi.nlm.nih.gov/clinvar/variation/382639/
NM_003060.3(SLC22A5):c.1434C>T	concordant	unreportable	unreportable	384574	0	2	SCV000525455;S	Likely benign;Benign	GeneDx;Invitae	2017-11-28;2017-1	SLC22A5	chr5	131728291	131728291	http://gnomad.b	0.0005620135335	AFR	0.006599579938	1		0	0	https://www.ncbi.nlm.nih.gov/clinvar/variation/384574/
NM_017890.4(VPS13B):c.3203C>T	concordant	unreportable	unreportable	95844	0	2	SCV000470789;S	Likely benign;Benign	Illumina Clinical Se	2016-10-18;2017-1	VPS13B	chr8	100443885	100443885	http://gnomad.b	0.009571936504	SAS	0.03328130022	34		32	0	https://www.ncbi.nlm.nih.gov/clinvar/variation/95844/
NM_000136.2(FANCC):c.816C>T	concordant	unreportable	unreportable	137277	0	4	SCV000594673;S	Likely benign;Uncertain sig	Genetic Services La	2017-07-05;2016-1	FANCC	chr9	97897655	97897655	http://gnomad.b	0.001669433201	EAS	0.01542390045	2		8	0	https://www.ncbi.nlm.nih.gov/clinvar/variation/137277/
NM_014363.4(SACS):c.10896A>G	concordant	unreportable	unreportable	311508	0	2	SCV000614926;S	Benign;Uncertain significar	Athena Diagnostics	2017-08-17;2016-1	SACS	chr13	23907119	23907119	http://gnomad.b	0.00163319264	AFR	0.01925089955	5		3	0	https://www.ncbi.nlm.nih.gov/clinvar/variation/311508/
NM_000303.2(PMM2):c.441C>T	concordant	unreportable	unreportable	290565	0	1	SCV000345138	Uncertain significance	EGL Genetic Diagn	6/30/2017	PMM2	chr16	8905029	8905029	http://gnomad.b	0.0006654008274	ASJ	0.005811300129	1		0	0	https://www.ncbi.nlm.nih.gov/clinvar/variation/290565/
NM_000152.3(GAA):c.852G>A	concordant	unreportable	unreportable	188477	0	1	SCV000626645	Benign	Invitae	10/5/2017	GAA	chr17	78081515	78081515	http://gnomad.b	0.007923802536	NFE	0.01024250034	5		14	0	https://www.ncbi.nlm.nih.gov/clinvar/variation/188477/
NM_005562.2(LAMC2):c.3546A>G	concordant	unreportable	unreportable	294024	0	1	SCV000351701	Uncertain significance	Illumina Clinical Se	10/18/2016	LAMC2	chr1	183212499	183212499	http://gnomad.b	0.006150925859	FIN	0.02377109975	12		19	0	https://www.ncbi.nlm.nih.gov/clinvar/variation/294024/
NM_017890.4(VPS13B):c.4197T>C	concordant	unreportable	unreportable	95852	0	2	SCV000470802;S	Likely benign;Benign	Illumina Clinical Se	2016-10-18;2017-1	VPS13B	chr8	100520037	100520037	http://gnomad.b	0.003941879672	AMR	0.07470030338	123		34	0	https://www.ncbi.nlm.nih.gov/clinvar/variation/95852/
NM_000057.2(BLM):c.254G>C	concordant	unreportable	unreportable	127488	0	1	SCV000283124	Benign	Invitae	10/5/2017	BLM	chr15	91292752	91292752	http://gnomad.b	0.0005184797603	AFR	0.006603030022	2		0	0	https://www.ncbi.nlm.nih.gov/clinvar/variation/127488/
NM_000071.2(CBS):c.215A>T	concordant	unreportable	unreportable	212883	0	2	SCV000249734;S	Uncertain significance;Likel	GeneDx;Invitae	2017-11-28;2017-1	CBS	chr21	44488720	44488720	http://gnomad.b	0.0008801579223	EAS	0.007136229891	0		0	0	https://www.ncbi.nlm.nih.gov/clinvar/variation/212883/
NM_033056.3(PCDH15):c.2885G>T	concordant	unreportable	unreportable	46458	0	2	SCV000203210;S	Uncertain significance;Unc	EGL Genetic Diagn	2017-06-30;2016-1	PCDH15	chr10	55721636	55721636	http://gnomad.b	0.002689914108	AMR	0.005097480025	1		2	0	https://www.ncbi.nlm.nih.gov/clinvar/variation/46458/
NM_000414.3(HSD17B4):c.948A>G	concordant	unreportable	unreportable	283769	0	1	SCV000336078	Benign	EGL Genetic Diagn	6/30/2017	HSD17B4	chr5	118832317	118832317	http://gnomad.b	0.0009776355307	EAS	0.0138582997	6		0	0	https://www.ncbi.nlm.nih.gov/clinvar/variation/283769/
NM_000303.2(PMM2):c.552T>C	concordant	unreportable	unreportable	321218	0	1	SCV000399676	Uncertain significance	Illumina Clinical Se	10/18/2016	PMM2	chr16	8906876	8906876	http://gnomad.b	4.18E-05	SAS	0.0006172439898	0		1	0	https://www.ncbi.nlm.nih.gov/clinvar/variation/321218/
NM_006493.2(CLN5):c.606G>A	concordant	unreportable	unreportable	205145	0	4	SCV000332348;S	Uncertain significance;Unc	EGL Genetic Diagn	2017-06-30;2017-1	CLN5	chr13	77570156	77570156	http://gnomad.b	0.0005650760615	NFE	0.0009936620481	0		0	0	https://www.ncbi.nlm.nih.gov/clinvar/variation/205145/
NM_000027.3(AGA):c.60A>G	concordant	unreportable	unreportable	348238	0	1	SCV000448741	Uncertain significance	Illumina Clinical Se	10/18/2016	AGA	chr4	178363470	178363470	http://gnomad.b	0.001603590145	AFR	0.01565369964	1		1	0	https://www.ncbi.nlm.nih.gov/clinvar/variation/348238/
NM_133647.1(SLC12A6):c.1284C>T	concordant	unreportable	unreportable	159887	0	2	SCV000516091;S	Benign;Uncertain significar	GeneDx;Illumina C	2017-11-28;2016-1	SLC12A6	chr15	34544420	34544420	http://gnomad.b	0.002326229197	AFR	0.02718240023	3		9	0	https://www.ncbi.nlm.nih.gov/clinvar/variation/159887/
NM_000057.2(BLM):c.1315A>G	concordant	unreportable	unreportable	127475	0	1	SCV000283112	Benign	Invitae	10/5/2017	BLM	chr15	91303918	91303918	http://gnomad.b	0.0005351281929	ASJ	0.004774480127	0		1	0	https://www.ncbi.nlm.nih.gov/clinvar/variation/127475/
NM_000271.4(NPC1):c.966C>T	concordant	unreportable	unreportable	326274	0	2	SCV000407882;S	Likely benign;Benign	Illumina Clinical Se	2016-10-18;2017-1	NPC1	chr18	21136567	21136567	http://gnomad.b	0.00389709162	AFR	0.03916539997	11		21	0	https://www.ncbi.nlm.nih.gov/clinvar/variation/326274/
NM_000057.2(BLM):c.2268A>G	concordant	unreportable	unreportable	136514	0	3	SCV000339654;S	Likely benign;Uncertain sig	EGL Genetic Diagn	2017-06-30;2016-1	BLM	chr15	91310214	91310214	http://gnomad.b	0.002544831851	ASJ	0.004782250151	0		3	0	https://www.ncbi.nlm.nih.gov/clinvar/variation/136514/
NM_017890.4(VPS13B):c.3386A>G	concordant	unreportable	unreportable	95845	0	4	SCV000297278;S	Likely benign;Likely benign,	Division of Genomi	2016-01-06;2017-1	VPS13B	chr8	100454804	100454804	http://gnomad.b	0.004409417957	NFE	0.006850299891	5		5	0	https://www.ncbi.nlm.nih.gov/clinvar/variation/95845/
NM_002485.4(NBN):c.425A>G	concordant	unreportable	unreportable	127871	0	6	SCV000611488;S	Uncertain significance;Unc	Fulgent Genetics,F	2017-05-23;2017-0	NBN	chr8	90993017	90993017	http://gnomad.b	0.0004127161182	AMR	0.0003573980066	0		1	0	https://www.ncbi.nlm.nih.gov/clinvar/variation/127871/
NM_005476.5(GNE):c.1770C>T	concordant	unreportable	unreportable	95282	0	4	SCV000345897;S	Benign;Benign;Likely benig	EGL Genetic Diagn	2016-11-03;2017-1	GNE	chr9	36219881	36219881	http://gnomad.b	0.01728021516	FIN	0.03710180148	69		78	0	https://www.ncbi.nlm.nih.gov/clinvar/variation/95282/
NM_138694.3(PKHD1):c.6211A>C	concordant	unreportable	unreportable	262408	0	1	SCV000702217	Benign	EGL Genetic Diagn	6/30/2017	PKHD1	chr6	51777285	51777285	http://gnomad.b	0.001298246394	AFR	0.00928346999	2		2	0	https://www.ncbi.nlm.nih.gov/clinvar/variation/262408/
NM_138694.3(PKHD1):c.8673C>G	concordant	unreportable	unreportable	167480	0	2	SCV000291343;S	Benign;Likely benign	Invitae;Laboratory	2017-10-05;2018-0	PKHD1	chr6	51619706	51619706	http://gnomad.b	0.00378910029	AFR	0.04720190167	14		34	0	https://www.ncbi.nlm.nih.gov/clinvar/variation/167480/
NM_000492.3(CFTR):c.4272C>T	concordant	unreportable	unreportable	93156	0	3	SCV000602995;S	Benign;Likely benign;Benig	ARUP Laboratories	2017-06-30;2016-1	CFTR	chr7	117306991	117306991	http://gnomad.b	0.008637358066	ASJ	0.02471020073	13		33	0	https://www.ncbi.nlm.nih.gov/clinvar/variation/93156/
NM_001042432.1(CLN3):c.1210C>A	concordant	unreportable	unreportable	128775	0	3	SCV000150722;S	Likely benign;Uncertain sig	Genetic Services La	2014-06-27;2016-1	CLN3	chr16	28488944	28488944	http://gnomad.b	0.003077709384	AFR	0.02666670084	6		7	0	https://www.ncbi.nlm.nih.gov/clinvar/variation/128775/
NM_002225.3(IVD):c.900G>A	concordant	unreportable	unreportable	94058	0	3	SCV000111893;S	Likely benign;Uncertain sig	EGL Genetic Diagn	2017-06-30;2016-1	IVD	chr15	40707612	40707612	http://gnomad.b	0.001784983719	SAS	0.004256289918	1		2	0	https://www.ncbi.nlm.nih.gov/clinvar/variation/94058/
NM_002485.4(NBN):c.381T>C	concordant	unreportable	unreportable	132744	0	6	SCV000537390;S	Benign;Benign;Likely benig	Color Genomics, In	2017-01-26;2017-0	NBN	chr8	90993061	90993061	http://gnomad.b	0.003070273285	SAS	0.007764279842	4		2	0	https://www.ncbi.nlm.nih.gov/clinvar/variation/132744/
NM_033056.3(PCDH15):c.475-3C>T	concordant	unreportable	unreportable	46485	0	1	SCV000363245	Uncertain significance	Illumina Clinical Se	10/18/2016	PCDH15	chr10	56106247	56106247	http://gnomad.b	0.01065164841	ASJ	0.02945359983	18		27	0	https://www.ncbi.nlm.nih.gov/clinvar/variation/46485/
NM_138694.3(PKHD1):c.3686G>C	concordant	unreportable	unreportable	196802	0	2	SCV000291331;S	Benign;Likely benign	Invitae;Laboratory	2017-03-14;2018-0	PKHD1	chr6	51890922	51890922	http://gnomad.b	0.003071867807	AFR	0.03306649998	12		19	0	https://www.ncbi.nlm.nih.gov/clinvar/variation/196802/
NM_000543.4(SMPD1):c.138G>T	concordant	unreportable	unreportable	193116	0	1	SCV000224251	Uncertain significance	EGL Genetic Diagn	6/30/2017	SMPD1	chr11	6411966	6411966	http://gnomad.b	2.38E-06	ASJ	0.0001039720009	0		0	0	https://www.ncbi.nlm.nih.gov/clinvar/variation/193116/
NM_000277.1(PAH):c.1242C>T	concordant	unreportable	unreportable	102577	0	2	SCV000515909;S	Likely benign;Benign	GeneDx;Invitae	2017-11-28;2017-1	PAH	chr12	103234251	103234251	http://gnomad.b	0.008024349751	NFE	0.01397470012	17		26	0	https://www.ncbi.nlm.nih.gov/clinvar/variation/102577/
NM_000382.2(ALDH3A2):c.28C>G	concordant	unreportable	unreportable	495853	0	1	SCV000696649	Benign	Laboratory Corpora	1/25/2018	ALDH3A2	chr17	19552312	19552312	http://gnomad.b	0.003681060593	NFE	0.006815080065	2		3	0	https://www.ncbi.nlm.nih.gov/clinvar/variation/495853/
NM_000016.4(ACADM):c.900C>T	concordant	unreportable	unreportable	136257	0	3	SCV000630298;S	Benign;Benign;Benign	Invitae;Laboratory	2017-10-05;2018-0	ACADM	chr1	76216186	76216186	http://gnomad.b	0.003706906868	AFR	0.0420438014	14		21	0	https://www.ncbi.nlm.nih.gov/clinvar/variation/136257/
NM_138694.3(PKHD1):c.3703C>T	concordant	unreportable	unreportable	497967	0	1	SCV000702747	Uncertain significance	EGL Genetic Diagn	6/30/2017	PKHD1	chr6	51890905	51890905	http://gnomad.b	7.23E-05	NFE	0.0001452879951	0		0	0	https://www.ncbi.nlm.nih.gov/clinvar/variation/497967/
NM_000303.2(PMM2):c.324G>A	concordant	unreportable	unreportable	129976	0	5	SCV000257665;S	Benign;Benign;Likely benig	Division of Genomi	2015-08-10;2017-1	PMM2	chr16	8900241	8900241	http://gnomad.b	0.01902656592	ASJ	0.04741119966	62		94	0	https://www.ncbi.nlm.nih.gov/clinvar/variation/129976/
NM_138694.3(PKHD1):c.11878G>A	concordant	unreportable	unreportable	96375	0	2	SCV000114567;S	Benign;Benign	EGL Genetic Diagn	2017-06-30;2017-1	PKHD1	chr6	51484226	51484226	http://gnomad.b	0.01325490109	AMR	0.0212011002	32		36	0	https://www.ncbi.nlm.nih.gov/clinvar/variation/96375/
NM_017890.4(VPS13B):c.7227G>A	concordant	unreportable	unreportable	95864	0	2	SCV000249418;S	Likely benign;Benign	Genetic Services La	2017-10-04;2017-1	VPS13B	chr8	100779103	100779103	http://gnomad.b	0.00574204205	ASJ	0.01076800004	6		13	0	https://www.ncbi.nlm.nih.gov/clinvar/variation/95864/

Supplemental Table 2

NM_001164277.1(SLC37A4):c.593A>T	concordant	unreportable	unreportable	139188	0	3	SCV000705610;S(	Likely benign;Benign;Benig	EGL Genetic Diagn(	2017-06-30;2017-1	SLC37A4	chr11	118898370	118898370	http://gnomad.b	0.004257577874	NFE	0.005518230144	2		2	0	https://www.ncbi.nlm.nih.gov/clinvar/variation/139188/
NM_000137.2(FAH):c.139A>G	concordant	unreportable	unreportable	317206	0	2	SCV000394041;S(	Uncertain significance;Beni	Illumina Clinical Se	2016-10-18;2017-1	FAH	chr15	80450459	80450459	http://gnomad.b	0.001961371795	AFR	0.02084420063	7		8	0	https://www.ncbi.nlm.nih.gov/clinvar/variation/317206/
NM_000642.2(AGL):c.1155G>T	concordant	unreportable	unreportable	256719	0	3	SCV000518636;S(	Benign;Likely benign;Benig	GeneDx;Illumina C	2017-11-28;2016-1	AGL	chr1	100340782	100340782	http://gnomad.b	0.01357265263	FIN	0.03403890133	36		42	0	https://www.ncbi.nlm.nih.gov/clinvar/variation/256719/
NM_000035.3(ALDOB):c.543T>G	concordant	unreportable	unreportable	364311	0	1	SCV000476063	Uncertain significance	Illumina Clinical Se	10/18/2016	ALDOB	chr9	104188918	104188918	http://gnomad.b	0.001779811131	ASJ	0.01788979955	2		2	0	https://www.ncbi.nlm.nih.gov/clinvar/variation/364311/
NM_000051.3(ATM):c.4578C>T	concordant	unreportable	unreportable	128457	0	5	SCV000537351;S(	Benign;Likely benign;Likely	Color Genomics, In	2017-01-26;2014-0	ATM	chr11	108163487	108163487		0.03267855552					255	0	https://www.ncbi.nlm.nih.gov/clinvar/variation/128457/
NM_000382.2(ALDH3A2):c.1270C>T	concordant	unreportable	unreportable	283733	0	2	SCV000336031;S(	Uncertain significance;Unc	EGL Genetic Diagn(	2017-06-30;2016-1	ALDH3A2	chr17	19575096	19575096	http://gnomad.b	0.004523645566	NFE	0.006455250084	9		4	0	https://www.ncbi.nlm.nih.gov/clinvar/variation/283733/
NM_000098.2(CPT2):c.1025T>C	concordant	unreportable	unreportable	376799	0	1	SCV000632582	Uncertain significance	Invitae	10/5/2017	CPT2	chr1	53676371	53676371	http://gnomad.b	0.0009457561292	FIN	0.002242150018	0		1	0	https://www.ncbi.nlm.nih.gov/clinvar/variation/376799/
NM_000018.3(ACADVL):c.1434+14T>A	concordant	unreportable	unreportable	194313	0	2	SCV000525460;S(	Likely benign;Uncertain sig	GeneDx;Illumina C	2017-11-28;2016-1	ACADVL	chr17	7127402	7127402	http://gnomad.b	0.001322421702	NFE	0.002291609999	1		3	0	https://www.ncbi.nlm.nih.gov/clinvar/variation/194313/
NM_000227.3(LAMA3):c.3277A>G	concordant	unreportable	unreportable	326326	0	1	SCV000407980	Likely benign	Illumina Clinical Se	10/18/2016	LAMA3	chr18	21501476	21501476	http://gnomad.b	0.003239244979	AFR	0.03836100176	12		26	0	https://www.ncbi.nlm.nih.gov/clinvar/variation/326326/
NM_000642.2(AGL):c.207T>C	concordant	unreportable	unreportable	256726	0	3	SCV000518545;S(	Benign;Likely benign;Benig	GeneDx;Illumina C	2017-11-28;2016-1	AGL	chr1	100327183	100327183	http://gnomad.b	0.0166650851	ASJ	0.04214910045	48		75	0	https://www.ncbi.nlm.nih.gov/clinvar/variation/256726/
NM_014625.2(NPHS2):c.87C>G	concordant	unreportable	unreportable	260431	0	2	SCV000614354;S(	Benign;Uncertain significar	Athena Diagnostics	2017-08-17;2016-1	NPHS2	chr1	179544913	179544913	http://gnomad.b	0.004120392	FIN	0.008267349564	1		6	0	https://www.ncbi.nlm.nih.gov/clinvar/variation/260431/
NM_015166.3(MLC1):c.597A>G	concordant	unreportable	unreportable	129615	0	2	SCV000151858;S(	Likely benign;Likely benign	Genetic Services La	2014-06-27;2016-1	MLC1	chr22	50515270	50515270		0.005996263522					147	0	https://www.ncbi.nlm.nih.gov/clinvar/variation/129615/
NM_000352.3(ABCC8):c.1958G>A	concordant	unreportable	unreportable	303779	0	1	SCV000369350	Likely benign	Illumina Clinical Se	10/18/2016	ABCC8	chr11	17449918	17449918	http://gnomad.b	0.0003303788516	SAS	0.004711159971	1		1	0	https://www.ncbi.nlm.nih.gov/clinvar/variation/303779/
NM_000271.4(NPC1):c.3441C>T	concordant	unreportable	unreportable	326249	0	2	SCV000407846;S(	Uncertain significance;Beni	Illumina Clinical Se	2016-10-18;2017-1	NPC1	chr18	21115469	21115469	http://gnomad.b	0.00100357972	AFR	0.01096520014	1		1	0	https://www.ncbi.nlm.nih.gov/clinvar/variation/326249/
NM_000528.3(MAN2B1):c.2260G>A	concordant	unreportable	unreportable	328262	0	2	SCV000709515;S(	Uncertain significance;Unc	EGL Genetic Diagn(	2017-06-30;2016-1	MAN2B1	chr19	12760734	12760734	http://gnomad.b	0.001816921881	ASJ	0.003045690013	2		2	0	https://www.ncbi.nlm.nih.gov/clinvar/variation/328262/
NM_033056.3(PCDH15):c.2885G>A	concordant	unreportable	unreportable	46457	0	2	SCV000514044;S(	Uncertain significance;Unc	GeneDx;Illumina C	2017-11-28;2016-1	PCDH15	chr10	55721636	55721636	http://gnomad.b	0.001265236846	AFR	0.007066209801	1		2	0	https://www.ncbi.nlm.nih.gov/clinvar/variation/46457/
NM_000487.5(ARSA):c.624T>C	concordant	unreportable	unreportable	256246	0	2	SCV000512102;S(	Benign;Uncertain significar	GeneDx;Illumina C	2017-11-28;2016-1	ARSA	chr22	51065322	51065322	http://gnomad.b	0.008756774401	FIN	0.02781270072	21		35	0	https://www.ncbi.nlm.nih.gov/clinvar/variation/256246/
NM_000360.3(TH):c.252G>A	concordant	unreportable	unreportable	304084	0	1	SCV000369929	Uncertain significance	Illumina Clinical Se	10/18/2016	TH	chr11	2190940	2190940	http://gnomad.b	0.0001295840352	AMR	0.002829399891	0		0	0	https://www.ncbi.nlm.nih.gov/clinvar/variation/304084/
NM_138694.3(PKHD1):c.7307C>T	concordant	unreportable	unreportable	197555	0	2	SCV000230727;S(	Uncertain significance;Unc	EGL Genetic Diagn(	2017-06-30;2016-1	PKHD1	chr6	51747934	51747934	http://gnomad.b	0.0004420153677	AFR	0.004055470228	0		0	0	https://www.ncbi.nlm.nih.gov/clinvar/variation/197555/
NM_033056.3(PCDH15):c.5359C>T	concordant	unreportable	unreportable	46498	0	1	SCV000363113	Uncertain significance	Illumina Clinical Se	10/18/2016	PCDH15	chr10	55582127	55582127	http://gnomad.b	0.00907524559	FIN	0.07245390117	70		17	0	https://www.ncbi.nlm.nih.gov/clinvar/variation/46498/
NM_000023.2(SGCA):c.528C>T	concordant	unreportable	unreportable	130293	0	4	SCV000534385;S(	Benign;Likely benign;Likely	GeneDx;Genetic Se	2017-11-28;2014-0	SGCA	chr17	48245877	48245877	http://gnomad.b	0.003817263884	AFR	0.02925970033	4		12	0	https://www.ncbi.nlm.nih.gov/clinvar/variation/130293/
NM_000271.4(NPC1):c.2775C>T	concordant	unreportable	unreportable	326257	0	1	SCV000407857	Uncertain significance	Illumina Clinical Se	10/18/2016	NPC1	chr18	21119795	21119795	http://gnomad.b	0.0001944257008	ASJ	0.00142161001	0		0	0	https://www.ncbi.nlm.nih.gov/clinvar/variation/326257/
NM_006261.4(PROP1):c.425C>T	concordant	unreportable	unreportable	196432	0	2	SCV000229096;S(	Likely benign;Uncertain sig	EGL Genetic Diagn(	2017-06-30;2016-1	PROP1	chr5	177419966	177419966	http://gnomad.b	0.003197255149	ASJ	0.006906359922	1		3	0	https://www.ncbi.nlm.nih.gov/clinvar/variation/196432/
NM_138694.3(PKHD1):c.2463C>G	concordant	unreportable	unreportable	357443	0	1	SCV000464104	Uncertain significance	Illumina Clinical Se	10/18/2016	PKHD1	chr6	51910931	51910931	http://gnomad.b	0.0009090504732	AFR	0.01189390011	0		1	0	https://www.ncbi.nlm.nih.gov/clinvar/variation/357443/
NM_000642.2(AGL):c.3431T>A	concordant	unreportable	unreportable	291342	0	2	SCV000346084;S(	Uncertain significance;Beni	Illumina Clinical Se	2016-10-18;2017-1	AGL	chr1	100366260	100366260	http://gnomad.b	0.001068954546	NFE	0.002434829948	1		0	0	https://www.ncbi.nlm.nih.gov/clinvar/variation/291342/
NM_000543.4(SMPD1):c.1071C>T	concordant	unreportable	unreportable	93310	0	1	SCV000372937	Uncertain significance	Illumina Clinical Se	10/18/2016	SMPD1	chr11	6413366	6413366	http://gnomad.b	0.006976688868	ASJ	0.01036159974	9		18	0	https://www.ncbi.nlm.nih.gov/clinvar/variation/93310/
NM_000492.3:c.-288G>C	concordant	unreportable	unreportable	237853	0	2	SCV000706996;S(	Likely benign;Uncertain sig	EGL Genetic Diagn(	2017-06-30;2016-0	CFTR	chr7	117119861	117119861		0.001848799051					4	0	https://www.ncbi.nlm.nih.gov/clinvar/variation/237853/
NM_001360.2(DHCR7):c.1341C>T	concordant	unreportable	unreportable	93709	0	2	SCV000630072;S(	Benign;Likely benign	Invitae;Laboratory	2017-10-05;2018-0	DHCR7	chr11	71146508	71146508	http://gnomad.b	0.006354471825	ASJ	0.03520410135	8		30	0	https://www.ncbi.nlm.nih.gov/clinvar/variation/93709/
NM_000310.3(PPT1):c.334A>T	concordant	unreportable	unreportable	423512	0	1	SCV000573219	Uncertain significance	GeneDx	11/28/2017	PPT1	chr1	40557745	40557745	http://gnomad.b	2.79E-06	NFE	1.79E-05	0		0	0	https://www.ncbi.nlm.nih.gov/clinvar/variation/423512/
NM_000271.4(NPC1):c.2501T>C	concordant	unreportable	unreportable	326260	0	2	SCV000706848;S(	Likely benign;Uncertain sig	EGL Genetic Diagn(	2017-06-30;2016-1	NPC1	chr18	21121045	21121045	http://gnomad.b	0.0006147283187	ASJ	0.004873100203	0		0	0	https://www.ncbi.nlm.nih.gov/clinvar/variation/326260/
NM_004543.4(NEB):c.15941C>G	concordant	unreportable	unreportable	129724	0	5	SCV000614167;S(	Likely benign;Benign;Benig	Athena Diagnostics	2017-08-17;2017-1	NEB	chr2	152394444	152394444	http://gnomad.b	0.008343745805	FIN	0.02320259996	19		15	0	https://www.ncbi.nlm.nih.gov/clinvar/variation/129724/
NM_000271.4(NPC1):c.445G>A	concordant	unreportable	unreportable	497771	0	1	SCV000702479	Benign	EGL Genetic Diagn(	6/30/2017	NPC1	chr18	21148805	21148805	http://gnomad.b	0.0005518258861	SAS	0.005458089989	2		4	0	https://www.ncbi.nlm.nih.gov/clinvar/variation/497771/
NM_000049.2(ASPA):c.831C>T	concordant	unreportable	unreportable	198037	0	2	SCV000231465;S(	Benign;Benign	EGL Genetic Diagn(	2017-06-30;2017-1	ASPA	chr17	3402271	3402271	http://gnomad.b	0.00374977525	AMR	0.03923380002	32		24	0	https://www.ncbi.nlm.nih.gov/clinvar/variation/198037/
NM_000137.2(FAH):c.565G>A	concordant	unreportable	unreportable	496929	0	1	SCV000700711	Likely benign	EGL Genetic Diagn(	6/30/2017	FAH	chr15	80460617	80460617	http://gnomad.b	0.0007518129512	AFR	0.002810090082	0		0	0	https://www.ncbi.nlm.nih.gov/clinvar/variation/496929/
NM_000543.4(SMPD1):c.813T>C	concordant	unreportable	unreportable	280985	0	2	SCV000330990;S(	Benign;Uncertain significar	EGL Genetic Diagn(	2017-06-30;2016-1	SMPD1	chr11	6413108	6413108	http://gnomad.b	0.003891531874	FIN	0.007568249945	4		7	0	https://www.ncbi.nlm.nih.gov/clinvar/variation/280985/
NM_000060.2(BTD):c.1171C>T	concordant	unreportable	unreportable	38570	0	4	SCV000602900;S(	Benign;Benign;Likely benig	ARUP Laboratories	2017-06-30;2017-0	BTD	chr3	15686534	15686534	http://gnomad.b	0.01505890358	NFE	0.02384190075	45		60	0	https://www.ncbi.nlm.nih.gov/clinvar/variation/38570/
NM_000228.2(LAMB3):c.3479A>G	concordant	unreportable	unreportable	295060	0	1	SCV000353577	Uncertain significance	Illumina Clinical Se	10/18/2016	LAMB3	chr1	209788656	209788656	http://gnomad.b	0.000895851647	ASJ	0.006194150075	0		1	0	https://www.ncbi.nlm.nih.gov/clinvar/variation/295060/
NM_000271.4(NPC1):c.1503C>T	concordant	unreportable	unreportable	199002	0	1	SCV000407880	Likely benign	Illumina Clinical Se	10/18/2016	NPC1	chr18	21134772	21134772	http://gnomad.b	0.004303098231	AFR	0.05338469893	19		33	0	https://www.ncbi.nlm.nih.gov/clinvar/variation/199002/
NM_000136.2(FANCC):c.416G>A	concordant	unreportable	unreportable	134304	0	3	SCV000481135;S(	Likely benign;Benign;Benig	Illumina Clinical Se	2016-10-18;2017-1	FANCC	chr9	97934359	97934359	http://gnomad.b	0.004275841732	AFR	0.04690570012	16		50	0	https://www.ncbi.nlm.nih.gov/clinvar/variation/134304/
NM_005562.2(LAMC2):c.1899G>C	concordant	unreportable	unreportable	287517	0	1	SCV000341306	Uncertain significance	EGL Genetic Diagn(	6/30/2017	LAMC2	chr1	183201363	183201363	http://gnomad.b	0.003703994042	ASJ	0.0144191999	0		7	0	https://www.ncbi.nlm.nih.gov/clinvar/variation/287517/
NM_000520.4(HEXA):c.1107C>T	concordant	unreportable	unreportable	317043	0	1	SCV000393835	Uncertain significance	Illumina Clinical Se	10/18/2016	HEXA	chr15	72640066	72640066	http://gnomad.b	0.000779456165	AMR	0.001846230007	2		0	0	https://www.ncbi.nlm.nih.gov/clinvar/variation/317043/
NM_004543.4(NEB):c.3986A>C	concordant	unreportable	unreportable	331513	0	3	SCV000513912;S(	Likely benign;Uncertain sig	GeneDx;Illumina C	2017-11-28;2016-1	NEB	chr2	152530992	152530992	http://gnomad.b	0.001143650802	NFE	0.001548710046	0		1	0	https://www.ncbi.nlm.nih.gov/clinvar/variation/331513/
NM_033056.3(PCDH15):c.3795A>T	concordant	unreportable	unreportable	46474	0	2	SCV000514046;S(	Benign;Uncertain significar	GeneDx;Illumina C	2017-11-28;2016-1	PCDH15	chr10	55616946	55616946	http://gnomad.b	0.004155737283	AFR	0.05090960115	25		27	0	https://www.ncbi.nlm.nih.gov/clinvar/variation/46474/
NM_000060.2(BTD):c.1101C>T	concordant	unreportable	unreportable	282920	0	3	SCV000334665;S(	Benign;Uncertain significar	EGL Genetic Diagn(	2017-06-30;2016-1	BTD	chr3	15686464	15686464	http://gnomad.b	0.001678016826	EAS	0.01756929979	3		3	0	https://www.ncbi.nlm.nih.gov/clinvar/variation/282920/
NM_000492.3(CFTR):c.1312A>G	concordant	unreportable	unreportable	193594	0	1	SCV000466512	Uncertain significance	Illumina Clinical Se	10/18/2016	CFTR	chr7	117188797	117188797		1.93E-06					0	0	https://www.ncbi.nlm.nih.gov/clinvar/variation/193594/
NM_004543.4(NEB):c.8592T>C	concordant	unreportable	unreportable	129760	0	5	SCV000343562;S(	Benign;Benign;Likely benig	EGL Genetic Diagn(	2017-06-30;2017-1	NEB	chr2	152496962	152496962	http://gnomad.b	0.003392228338	AFR	0.04272999987	20		20	0	https://www.ncbi.nlm.nih.gov/clinvar/variation/129760/
NM_005562.2(LAMC2):c.2387C>T	concordant	unreportable	unreportable	294008	0	1	SCV000351681	Uncertain significance	Illumina Clinical Se	10/18/2016	LAMC2	chr1	183204796	183204796	http://gnomad.b	0.00113667542	NFE	0.001650220016	1		0	0	https://www.ncbi.nlm.nih.gov/clinvar/variation/294008/
NM_000243.2(MEFV):c.2118G>A	concordant	unreportable	unreportable	36508	0	1	SCV000629036	Benign	Invitae	10/5/2017	MEFV	chr16	3293369	3293369	http://gnomad.b	0.00458718164	AFR	0.02084420063	3		10	0	https://www.ncbi.nlm.nih.gov/clinvar/variation/36508/
NM_000023.2(SGCA):c.648C>T	concordant	unreportable	unreportable	324043	0	1	SCV000403950	Uncertain significance	Illumina Clinical Se	10/18/2016	SGCA	chr17	48246516	48246516	http://gnomad.b	5.60E-05	NFE	6.27E-05	0		0	0	https://www.ncbi.nlm.nih.gov/clinvar/variation/324043/
NM_000060.2(BTD):c.1237G>A	concordant	unreportable	unreportable	38577	0	1	SCV000630321	Uncertain significance	Invitae	10/5/2017	BTD	chr3	15686600	15686600	http://gnomad.b	2.02E-05	NFE	2.69E-05	0		0	0	https://www.ncbi.nlm.nih.gov/clinvar/variation/38577/
NM_017890.4(VPS13B):c.1041A>G	concordant	unreportable	unreportable	198887	0	1	SCV000470763	Uncertain significance	Illumina Clinical Se	10/18/2016	VPS13B	chr8	100133508	100133508	http://gnomad.b	0.001063859571	ASJ	0.007209590171	0		2	0	https://www.ncbi.nlm.nih.gov/clinvar/variation/198887/
NM_001164277.1(SLC37A4):c.1062C>T	concordant	unreportable	unreportable	139191	0	2	SCV000631376;S(	Benign;Benign	Invitae;Laboratory	2017-10-05;2018-0	SLC37A4	chr11	118895962	118895962	http://gnomad.b	0.01201005727	ASJ	0.01530919969	18		34	0	https://www.ncbi.nlm.nih.gov/clinvar/variation/139191/
NM_000051.3(ATM):c.1986T>C	concordant	unreportable	unreportable	135740	0	4	SCV000602570;S(	Likely benign;Likely benign,	ARUP Laboratories	2017-06-30;2017-0	ATM	chr11	108124628	108124628	http://gnomad.b	0.0006098511852	ASJ	0.001117200009	0		0	0	https://www.ncbi.nlm.nih.gov/clinvar/variation/135740/
NM_000152.3(GAA):c.271G>A	concordant	unreportable	unreportable	4020	0	1	SCV000151258	Likely benign	Genetic Services La	6/27/2014	GAA	chr17	78078656	78078656	http://gnomad.b	0.02212605822	NFE	0.03269949928	77		113	0	https://www.ncbi.nlm.nih.gov/clinvar/variation/4020/
NM_005476.5(GNE):c.1872G>A	concordant	unreportable	unreportable	286172	0	3	SCV000339502;S(	Benign;Likely benign;Likely	EGL Genetic Diagn(	2017-06-30;2017-1	GNE	chr9	36218241	36218241	http://gnomad.b	0.003981061243	ASJ	0.03239240125	5		22	0	https://www.ncbi.nlm.nih.gov/clinvar/variation/286172/
NM_017890.4(VPS13B):c.2760A>G	concordant	unreportable	unreportable	361049	0	3	SCV000616268;S(	Likely benign;Likely benign,	Athena Diagnostics	2017-08-17;2017-0	VPS13B	chr8	100287418	100287418	http://gnomad.b	0.002990005319	ASJ	0.01411450002	4		3	0	https://www.ncbi.nlm.nih.gov/clinvar/variation/361049/
NM_000466.2(PEX1):c.2442C>T	concordant	unreportable	unreportable	93106	0	1	SCV000470510	Uncertain significance	Illumina Clinical Se	10/18/2016	PEX1	chr7	92130962	92130962	http://gnomad.b	0.003644310826	ASJ	0.003861000063	0		7	0	https://www.ncbi.nlm.nih.gov/clinvar/variation/93106/
NM_004543.4(NEB):c.2739C>T	concordant	unreportable	unreportable	331525	0	1	SCV000417028	Uncertain significance	Illumina Clinical Se	10/18/2016	NEB	chr2	152541388	152541388	http://gnomad.b	0.0001445698531	EAS	0.0006406520261	0		0	0	https://www.ncbi.nlm.nih.gov/clinvar/variation/331525/
NM_017739.3(POMGNT1):c.1257G>A	concordant	unreportable	unreportable	130004	0	2	SCV000152346;S(	Likely benign;Benign	Genetic Services La	2014-06-27;2017-1	POMGNT1	chr1	46658217	46658217	http://gnomad.b	0.01067195225	NFE	0.01632039994	13		34	0	https://www.ncbi.nlm.nih.gov/clinvar/variation/130004/
NM_000271.4(NPC1):c.547G>A	concordant	unreportable	unreportable	181450	0	1	SCV000339988	Uncertain significance	EGL Genetic Diagn(	6/30/2017	NPC1	chr18	21141408	21141408	http://gnomad.b	0.0001572560815	AFR	0.0008494509966	0		1	0	https://www.ncbi.nlm.nih.gov/clinvar/variation/181450/
NM_000050.4(ASS1):c.675C>T	concordant	unreportable	unreportable	203630	0	1	SCV000630063	Benign	Invitae	10/5/2017	ASS1	chr9	133352335	133352335	http://gnomad.b	0.001980203535	AFR	0.02352330089	5		7	0	https://www.ncbi.nlm.nih.gov/clinvar/variation/203630/
NM_003640.3(IKBKAP):c.2855A>T	concordant	unreportable	unreportable	259109	0	1	SCV000563267	Benign	Invitae	10/5/2017	IKBKAP	chr9	111656228	111656228	http://gnomad.b	0.01491594551	EAS	0.02963010035	46		70	0	https://www.ncbi.nlm.nih.gov/clinvar/variation/259109/
NM_000136.2(FANCC):c.1156T>C	concordant	unreportable	unreportable	134296	0	1	SCV000253074	Likely benign	Invitae	10/5/2017	FANCC	chr9	97873918	97873918	http://gnomad.b	0.0006159304831	AFR	0.007713430095	1		1	0	https://www.ncbi.nlm.nih.gov/clinvar/variation/134296/
NM_138694.3(PKHD1):c.275G>A	concordant	unreportable	unreportable	357457	0	2	SCV000700533;S(	Uncertain significance;Unc	EGL Genetic Diagn(	2017-06-30;2016-1	PKHD1	chr6	51947196	51947196	http://gnomad.b	0.0007589320464	AFR	0.00124166999	1		0	0	https://www.ncbi.nlm.nih.gov/clinvar/variation/357457/
NM_183050.2(BCKDHB):c.51A>G	concordant	unreportable	unreportable	358168	0	2	SCV000525789;S(	Likely benign;Uncertain sig	GeneDx;Illumina C	2017-11-28;2016-1	BCKDHB	chr6	80816461	80816461	http://gnomad.b	0.001112284335	NFE	0.001837649965	0		0	0	https://www.ncbi.nlm.nih.gov/clinvar/variation/358168/
NM_000528.3(MAN2B1):c.2701C>A	concordant	unreportable	unreportable	328255	0	1	SCV000410775	Uncertain significance	Illumina Clinical Se	10/18/2016	MAN2B1	chr19	12758376	12758376	http://gnomad.b	0.0003098626463	NFE	0.0003834289964	0		0	0	https://www.ncbi.nlm.nih.gov/clinvar/variation/328255/
NM_000382.2(ALDH3A2):c.563C>T	concordant	unreportable	unreportable	322210	0	1	SCV000401253	Likely benign	Illumina Clinical Se	10/18/2016	ALDH3A2	chr17	19559770	19559770	http://gnomad.b	0.003451519172	AFR	0.03914010152	13		28	0	https://www.ncbi.nlm.nih.gov/clinvar/variation/322210/
NM_000053.3(ATP7B):c.1620C>T	concordant	unreportable	unreportable	254760	0	5	SCV000602599;S(	Benign;Uncertain significar	ARUP Laboratories	2017-06-30;2017-0	ATP7B	chr13	52542667	52542667	http://gnomad.b	0.00134402568	NFE	0.002508870093	1		0	0	https://www.ncbi.nlm.nih.gov/clinvar/variation/254760/
NM_000050.4(ASS1):c.766G>A	concordant	unreportable	unreportable	235312	0	1	SCV000520638	Benign	GeneDx	11/28/2017	ASS1	chr9	133355180	133355180	http://gnomad.b	0.002514942042	AFR	0.02862000093	7		8	0	https://www.ncbi.nlm.nih.gov/clinvar/variation/235312/
NM_017890.4(VPS13B):c.3811A>T	concordant	unreportable	unreportable	195900	0	4	SCV000228257;S(	Uncertain significance;Unc	EGL Genetic Diagn(	2017-06-30;2017-0	VPS13B	chr8	100493971	100493971	http://gnomad.b	0.0006495142641	AMR	0.001996899955	0		2	0	https://www.ncbi.nlm.nih.gov/clinvar/variation/195900/
NM_000108.3(DLD):c.249T>C	concordant	unreportable	unreportable	137092	0	1	SCV000466215	Uncertain significance	Illumina Clinical Se	10/18/2016	DLD	chr7	107542820	107542820	http://gnomad.b	0.002718237896	AFR	0.03189539909	10		14	0	https://www.ncbi.nlm.nih.gov/clinvar/variation/137092/
NM_033056.3(PCDH15):c.4334C>G	concordant	unreportable	unreportable	46480	0	2	SCV000363144;S(	Uncertain significance;Beni	Illumina Clinical Se	2016-10-18;2016-0	PCDH15	chr10	55587186	55587186	http://gnomad.b	0.0008280615131	AMR	0.01848760061	12		1	0	https://www.ncbi.nlm.nih.gov/clinvar/variation/46480/
NM_017890.4(VPS13B):c.7787C>T	concordant	unreportable	unreportable	197472	0	4	SCV000230597;S(	Uncertain significance;Unc	EGL Genetic Diagn(	2017-06-30;2017-0	VPS13B	chr8	100791192	100791192	http://gnomad.b	0.0006495142641	AMR	0.001997610088	0		2	0	https://www.ncbi.nlm.nih.gov/clinvar/variation/197472/
NM_000159.2(GCDH):c.1011A>G	concordant	unreportable	unreportable	193562	0	2	SCV000631927;S(	Benign;Benign	Invitae;Laboratory	2017-10-05;2018-0	GCDH	chr19	13008171	13008171	http://gnomad.b	0.003114949509	EAS	0.03254060075	5		16	0	https://www.ncbi.nlm.nih.gov/clinvar/variation/193562/
NM_000108.3(DLD):c.543A>T	concordant	unreportable	unreportable	137095	0	1	SCV000466225	Uncertain significance	Illumina Clinical Se	10/18/2016	DLD	chr7	107545910	107545910	http://gnomad.b	0.004372027333	NFE	0.005552770104	5		8	0	https://www.ncbi.nlm.nih.gov/clinvar/variation/137095/
NM_001876.3(CPT1A):c.1941C>G	concordant	unreportable	unreportable	203653	0	1	SCV000700360	Benign	EGL Genetic Diagn(	6/30/2017	CPT1A	chr11	68529090	68529090	http://gnomad.b	0.002576666385	AFR	0.02803190053	14		9	0	https://www.ncbi.nlm.nih.gov/clinvar/variation/203653/
NM_000528.3(MAN2B1):c.2562C>G	concordant	unreportable	unreportable	328258	0	2	SCV000410778;S(	Likely benign;Benign	Illumina Clinical Se	2016-10-18;2017-1	MAN2B1	chr19	12759091	12759091	http://gnomad.b	0.003123978862	AFR	0.03750550002	12		10	0	https://www.ncbi.nlm.nih.gov/clinvar/variation/328258/
NM_003060.3(SLC22A5):c.1590G>T	concordant	unreportable	unreportable	460403	0	1	SCV000632539	Benign	Invitae	10/5/2017	SLC22A5	chr5	131729880	131729880	http://gnomad.b	0.0009884660137	AFR	0.0114364	1		1	0	https://www.ncbi.nlm.nih.gov/clinvar/variation/460403/
NM_000053.3(ATP7B):c.3015C>T	concordant	unreportable	unreportable	35715	0	3	SCV000602604;S(	Benign;Likely benign;Benig	ARUP Laboratories	2017-06-30;2017-1	ATP7B	chr13	52520465	52520465	http://gnomad.b	0.001052212171	AFR	0.01313129999	3		2	0	https://www.ncbi.nlm.nih.gov/clinvar/variation/35715/
NM_000030.2(AGXT):c.866G>A	concordant	unreportable	unreportable	198982	0	2	SCV000232896;S(	Uncertain significance;Unc	EGL Genetic Diagn(	2017-06-30;2018-0	AGXT	chr2	241816973	241816973	http://gnomad.b	0.001256476375	ASJ	0.006496690214	0		1	0	https://www.ncbi.nlm.nih.gov/clinvar/variation/198982/
NM_004543.4(NEB):c.8466C>T	concordant	unreportable	unreportable	129759	0	4	SCV000519527;S(	Benign;Likely benign;Likely	GeneDx;Genetic Se	2017-11-28;2014-0	NEB	chr2	152497088	152497088	http://gnomad.b	0.02423352162	ASJ	0.04711620137	79		166	0	https://www.ncbi.nlm.nih.gov/clinvar/variation/129759/
NM_002485.4(NBN):c.613A>G	concordant	unreportable	unreportable	182714	0	3	SCV000685803;S(	Uncertain significance;Unc	Color Genomics, In	2017-10-26;2017-1	NBN	chr8	90983490	90983490	http://gnomad.b	4.18E-05	AFR	0.0001309759973	0		0	0	https://www.ncbi.nlm.nih.gov/clinvar/variation/182714/
NM_000391.3(TPP1):c.1396G>A	concordant	unreportable	unreportable	137704	0	1	SCV000559670	Benign	Invitae	10/5/2017	TPP1	chr11	6636431	6636431	http://gnomad.b	0.0008589767265	AFR	0.01045479998	0		1	0	https://www.ncbi.nlm.nih.gov/clinvar/variation/137704/
NM_000518.4(HBB):c.402G>C	concordant	unreportable	unreportable	36327	0	1	SCV000603932	Likely benign	ARUP Laboratories	6/30/2017	HBB	chr11	5246870	5246870	http://gnomad.b	0.003275078611	NFE	0.004489489831	4		4	0	https://www.ncbi.nlm.nih.gov/clinvar/variation/36327/
NM_012203.1(GRHPR):c.16C>T	concordant	unreportable	unreportable	366851	0	1	SCV000480129	Uncertain significance	Illumina Clinical Se	10/18/2016	GRHPR	chr9	37422763	37422763	http://gnomad.b	0.003014444212	SAS	0.01760309935	6		7	0	https://www.ncbi.nlm.nih.gov/clinvar/variation/366851/
NM_017890.4(VPS13B):c.9424A>G	concordant	unreportable	unreportable	95874	0	2	SCV000470837;S(	Likely benign;Benign	Illumina Clinical Se	2016-10-18;2017-1	VPS13B	chr8	100844615	100844615	http://gnomad.b	0.008510876564	SAS	0.03015179932	25		23	0	https://www.ncbi.nlm.nih.gov/clinvar/variation/95874/
NM_000466.2(PEX1):c.330C>G	concordant	unreportable	unreportable	93111	0	1	SCV000470527	Uncertain significance	Illumina Clinical Se	10/18/2016	PEX1	chr7	92148336	92148336	http://gnomad.b	0.002509970546	NFE	0.003474340076	5		2	0	https://www.ncbi.nlm.nih.gov/clinvar/variation/93111/
NM_000391.3(TPP1):c.185C>T	concordant	unreportable	unreportable	137698	0	1	SCV000559662	Benign	Invitae	10/5/2017	TPP1	chr11	6640051	6640051	http://gnomad.b	0.00149411587	AFR	0.01927600056	4		6	0	https://www.ncbi.nlm.nih.gov/clinvar/variation/137698/
NM_000153.3(GALC):c.1006G>A	concordant	unreportable	unreportable	314752	0	1	SCV000389250	Uncertain significance	Illumina Clinical Se	10/18/2016	GALC	chr14	88431876	88431876	http://gnomad.b	0.0008279349735	AFR	0.01204190031	1		0	0	https://www.ncbi.nlm.nih.gov/clinvar/variation/314752/
NM_000035.3(ALDOB):c.759C>T	concordant	unreportable	unreportable	287107	0	3	SCV000340776;S(	Uncertain significance;Likel	EGL Genetic Diagn(	2017-06-30;2017-1	ALDOB	chr9	104187775	104187775	http://gnomad.b	0.002231110309	ASJ	0.01391149964	1		4	0	https://www.ncbi.nlm.nih.gov/clinvar/variation/287107/
NM_000182.4(HADHA):c.1212G>C	concordant	unreportable	unreportable	335381	0	2	SCV000526892;S(	Likely benign;Uncertain sig	GeneDx;Illumina C	2017-11-28;2016-1	HADHA	chr2	26426939	26426939	http://gnomad.b	0.0007233536695	ASJ	0.001319800038	0		0	0	https://www.ncbi.nlm.nih.gov/clinvar/variation/335381/
NM_004937.2(CTNS):c.108C>T	concordant	unreportable	unreportable	322835	0	1	SCV000402209	Uncertain significance	Illumina Clinical Se	10/18/2016	CTNS	chr17	3550784	3550784	http://gnomad.b	0.0043645479	FIN	0.02048519999	21		9	0	https://www.ncbi.nlm.nih.gov/clinvar/variation/322835/
NM_000228.2(LAMB3):c.1051G>A	concordant	unreportable	unreportable	295116	0	1	SCV000353643	Uncertain significance	Illumina Clinical Se	10/18/2016	LAMB3	chr1	209803163	209803163	http://gnomad.b	0.001097418268	NFE	0.001763459994	0		0	0	https://www.ncbi.nlm.nih.gov/clinvar/variation/295116/
NM_003060.3(SLC22A5):c.1368A>G	concordant	unreportable	unreportable	384573	0	2	SCV000525454;S(	Likely benign;Benign	GeneDx;Invitae	2017-11-28;2017-1	SLC22A5	chr5	131728225	131728225	http://gnomad.b	0.0009884660137	AFR	0.01143489964	1		1	0	https://www.ncbi.nlm.nih.gov/clinvar/variation/384573/
NM_020533.2(MCOLN1):c.1200C>T	concordant	unreportable	unreportable	459179	0	1	SCV000630740	Benign	Invitae	10/5/2017	MCOLN1	chr19	7594052	7594052	http://gnomad.b	0.005582937667	AFR	0.04052820057	31		37	0	https://www.ncbi.nlm.nih.gov/clinvar/variation/459179/
NM_000152.3(GAA):c.858+8G>A	concordant	unreportable	unreportable	129122	0	1	SCV000626648	Benign	Invitae	10/5/2017	GAA	chr17	78081529	78081529	http://gnomad.b	0.0005503026665	AFR	0.01527400035	216		0	0	https://www.ncbi.nlm.nih.gov/clinvar/variation/129122/
NM_000153.3(GALC):c.1302C>T	concordant	unreportable	unreportable	255370	0	2	SCV000389246;S(	Likely benign;Benign	Illumina Clinical Se	2016-10-18;2017-1	GALC	chr14	88416225	88416225	http://gnomad.b	0.008889112655	EAS	0.0386984013	29		42	0	https://www.ncbi.nlm.nih.gov/clinvar/variation/255370/

Supplemental Table 2

NM_014363.4(SACS):c.3129A>G	concordant	unreportable	unreportable	240898	0		4	SCV000614949;S	Likely benign;Likely benign	Athena Diagnostics	2017-08-17;2017-0	SACS	chr13	23914886	23914886	http://gnomad.b	0.003210068292	NFE	0.005442759953	3		3	0	https://www.ncbi.nlm.nih.gov/clinvar/variation/240898/
NM_014363.4(SACS):c.4936C>A	concordant	unreportable	unreportable	311543	0		2	SCV000614958;S	Uncertain significance;Unc	Athena Diagnostics	2017-08-17;2016-1	SACS	chr13	23913079	23913079	http://gnomad.b	9.19E-05	FIN	0.0002702219936	0		0	0	https://www.ncbi.nlm.nih.gov/clinvar/variation/311543/
NM_001164277.1(SLC37A4):c.872C>T	concordant	unreportable	unreportable	459625	0		1	SCV000631386	Likely benign	Invitae	10/5/2017	SLC37A4	chr11	118896789	118896789	http://gnomad.b	0.000435815058	AFR	0.004733920097	0		0	0	https://www.ncbi.nlm.nih.gov/clinvar/variation/459625/
NM_000137.2(FAH):c.165C>T	concordant	unreportable	unreportable	380509	0		2	SCV000518666;S	Benign;Benign	GeneDx;Invitae	2017-11-28;2017-1	FAH	chr15	80450485	80450485	http://gnomad.b	0.002388601295	AFR	0.02528749965	7		12	0	https://www.ncbi.nlm.nih.gov/clinvar/variation/380509/
NM_004543.4(NEB):c.2640C>T	concordant	unreportable	unreportable	129737	0		3	SCV000531255;S	Likely benign;Likely benign	GeneDx;Genetic Se	2017-11-28;2014-0	NEB	chr2	152541487	152541487	http://gnomad.b	0.00118237487	AFR	0.01301280037	2		6	0	https://www.ncbi.nlm.nih.gov/clinvar/variation/129737/
NM_133647.1(SLC12A6):c.1412G>C	concordant	unreportable	unreportable	284439	0		3	SCV000337069;S	Benign;Benign;Uncertain si	EGL Genetic Diagn	2017-06-30;2017-1	SLC12A6	chr15	34543180	34543180	http://gnomad.b	0.006196363378	NFE	0.00990046002	5		6	0	https://www.ncbi.nlm.nih.gov/clinvar/variation/284439/
NM_000492.3(CFTR):c.1210-13G>T	concordant	unreportable	unreportable	162962	0		2	SCV000602974;S	Benign;Likely benign	ARUP Laboratories	2017-06-30;2016-1	CFTR	chr7	117188682	117188682		0.003699523934					75	0	https://www.ncbi.nlm.nih.gov/clinvar/variation/162962/
NM_001271208.1(NEB):c.11729A>G	concordant	unreportable	unreportable	129707	0		3	SCV000522834;S	Benign;Likely benign;Benig	GeneDx;Genetic Se	2017-11-28;2014-0	NEB	chr2	152468776	152468776	http://gnomad.b	0.0126240461	FIN	0.02731190063	43		59	0	https://www.ncbi.nlm.nih.gov/clinvar/variation/129707/
NM_000642.2(AGL):c.3849T>C	concordant	unreportable	unreportable	256745	0		3	SCV000518641;S	Benign;Likely benign;Benig	GeneDx;Illumina C	2017-11-28;2016-1	AGL	chr1	100377973	100377973	http://gnomad.b	0.01387408106	FIN	0.03442240134	39		43	0	https://www.ncbi.nlm.nih.gov/clinvar/variation/256745/
NM_001079802.1(FKTN):c.285T>C	concordant	unreportable	unreportable	379745	0		2	SCV000517097;S	Likely benign;Likely benign	GeneDx;Invitae	2017-11-28;2017-1	FKTN	chr9	108363545	108363545	http://gnomad.b	0.0001028288573	AFR	0.002287579933	0		0	0	https://www.ncbi.nlm.nih.gov/clinvar/variation/379745/
NM_000051.3(ATM):c.4042T>C	concordant	unreportable	unreportable	132715	0		2	SCV000682179;S	Benign;Benign	Color Genomics, In	2017-10-26;2017-1	ATM	chr11	108158375	108158375	http://gnomad.b	0.001662471039	AFR	0.01804159954	1		3	0	https://www.ncbi.nlm.nih.gov/clinvar/variation/132715/
NM_017890.4(VPS13B):c.10640C>T	concordant	unreportable	unreportable	361091	0		1	SCV000470854	Uncertain significance	Illumina Clinical Se	10/18/2016	VPS13B	chr8	100866182	100866182	http://gnomad.b	4.20E-05	NFE	9.85E-05	0		0	0	https://www.ncbi.nlm.nih.gov/clinvar/variation/361091/
NM_000271.4(NPC1):c.612C>T	concordant	unreportable	unreportable	197651	0		3	SCV000230885;S	Benign;Uncertain significar	EGL Genetic Diagn	2017-06-30;2016-1	NPC1	chr18	21141343	21141343	http://gnomad.b	0.004057206903	SAS	0.02985510044	21		26	0	https://www.ncbi.nlm.nih.gov/clinvar/variation/197651/
NM_004543.4(NEB):c.10419G>C	concordant	unreportable	unreportable	129706	0		4	SCV000523861;S	Benign;Likely benign;Likely	GeneDx;Genetic Se	2017-11-28;2014-0	NEB	chr2	152473911	152473911	http://gnomad.b	0.01323588637	FIN	0.02925099991	28		49	0	https://www.ncbi.nlm.nih.gov/clinvar/variation/129706/
NM_000228.2(LAMB3):c.1015T>C	concordant	unreportable	unreportable	295118	0		1	SCV000353645	Uncertain significance	Illumina Clinical Se	10/18/2016	LAMB3	chr1	209803199	209803199	http://gnomad.b	0.01403967503	SAS	0.01760770008	40		54	0	https://www.ncbi.nlm.nih.gov/clinvar/variation/295118/
NM_005562.2(LAMC2):c.407A>T	concordant	unreportable	unreportable	293991	0		1	SCV000351657	Likely benign	Illumina Clinical Se	10/18/2016	LAMC2	chr1	183187527	183187527	http://gnomad.b	0.004689486032	EAS	0.05169409886	34		27	0	https://www.ncbi.nlm.nih.gov/clinvar/variation/293991/
NM_020533.2(MCOLN1):c.405+13G>A	concordant	unreportable	unreportable	330489	0		1	SCV000415604	Likely benign	Illumina Clinical Se	10/18/2016	MCOLN1	chr19	7591505	7591505	http://gnomad.b	0.0135184639	FIN	0.06598360091	106		60	0	https://www.ncbi.nlm.nih.gov/clinvar/variation/330489/
NM_000098.2(CPT2):c.1806T>C	concordant	unreportable	unreportable	460427	0		2	SCV000702018;S	Uncertain significance;Beni	EGL Genetic Diagn	2017-06-30;2017-1	CPT2	chr1	53679096	53679096	http://gnomad.b	0.004135584494	ASJ	0.01340099983	3		5	0	https://www.ncbi.nlm.nih.gov/clinvar/variation/460427/
NM_000071.2(CBS):c.573G>A	concordant	unreportable	unreportable	136667	0		2	SCV000602916;S	Benign;Benign	ARUP Laboratories	2017-06-30;2017-1	CBS	chr21	44485590	44485590	http://gnomad.b	0.00847538651	ASJ	0.03000199981	12		24	0	https://www.ncbi.nlm.nih.gov/clinvar/variation/136667/
NM_000152.3(GAA):c.2668G>C	concordant	unreportable	unreportable	255361	0		4	SCV000334977;S	Benign;Benign;Uncertain si	EGL Genetic Diagn	2017-06-30;2017-1	GAA	chr17	78092473	78092473	http://gnomad.b	0.002551403272	SAS	0.02647650056	13		17	0	https://www.ncbi.nlm.nih.gov/clinvar/variation/255361/
NM_004543.4(NEB):c.539A>G	concordant	unreportable	unreportable	198810	0		2	SCV000417054;S	Uncertain significance;Beni	Illumina Clinical Se	2016-10-18;2017-1	NEB	chr2	152580847	152580847	http://gnomad.b	0.0007693181466	SAS	0.008555199951	4		0	0	https://www.ncbi.nlm.nih.gov/clinvar/variation/198810/
NM_138694.3(PKHD1):c.11340T>C	concordant	unreportable	unreportable	262384	0		1	SCV000557658	Benign	Invitae	10/5/2017	PKHD1	chr6	51512887	51512887	http://gnomad.b	0.03474407588	AFR	0.04391580075	170		262	0	https://www.ncbi.nlm.nih.gov/clinvar/variation/262384/
NM_138694.3(PKHD1):c.5199C>A	concordant	unreportable	unreportable	241846	0		3	SCV000331172;S	Benign;Benign;Benign	EGL Genetic Diagn	2017-06-30;2017-1	PKHD1	chr6	51889409	51889409	http://gnomad.b	0.004486872978	AFR	0.05088960007	17		29	0	https://www.ncbi.nlm.nih.gov/clinvar/variation/241846/
NM_000391.3(TPP1):c.1117C>G	concordant	unreportable	unreportable	137701	0		1	SCV000284804	Benign	Invitae	10/5/2017	TPP1	chr11	6637264	6637264	http://gnomad.b	0.00230552711	AFR	0.02679390088	3		8	0	https://www.ncbi.nlm.nih.gov/clinvar/variation/137701/
NM_017890.4(VPS13B):c.2451T>C	concordant	unreportable	unreportable	262659	0		2	SCV000616267;S	Benign;Uncertain significar	Athena Diagnostics	2017-08-17;2016-1	VPS13B	chr8	100205221	100205221	http://gnomad.b	0.003908284106	AFR	0.01686719991	4		7	0	https://www.ncbi.nlm.nih.gov/clinvar/variation/262659/
NM_000228.2(LAMB3):c.689G>C	concordant	unreportable	unreportable	295131	0		1	SCV000353659	Uncertain significance	Illumina Clinical Se	10/18/2016	LAMB3	chr1	209806061	209806061	http://gnomad.b	0.002102451834	AFR	0.02287280001	1		13	0	https://www.ncbi.nlm.nih.gov/clinvar/variation/295131/
NM_138694.3(PKHD1):c.888A>T	concordant	unreportable	unreportable	220081	0		1	SCV000260341	Benign	Invitae	10/5/2017	PKHD1	chr6	51929841	51929841	http://gnomad.b	0.002563133138	AFR	0.0316339992	6		17	0	https://www.ncbi.nlm.nih.gov/clinvar/variation/220081/
NM_000642.2(AGL):c.334A>G	concordant	unreportable	unreportable	291324	0		2	SCV000346056;S	Uncertain significance;Unc	Illumina Clinical Se	2016-10-18;2017-1	AGL	chr1	100327853	100327853	http://gnomad.b	0.0006782139807	NFE	0.001038379967	1		0	0	https://www.ncbi.nlm.nih.gov/clinvar/variation/291324/
NM_138694.3(PKHD1):c.6184C>T	concordant	unreportable	unreportable	262407	0		1	SCV000702215	Benign	EGL Genetic Diagn	6/30/2017	PKHD1	chr6	51777312	51777312	http://gnomad.b	0.001298246394	AFR	0.009282260202	2		2	0	https://www.ncbi.nlm.nih.gov/clinvar/variation/262407/
NM_000271.4(NPC1):c.3717C>T	concordant	unreportable	unreportable	282371	0		2	SCV000333819;S	Uncertain significance;Likel	EGL Genetic Diagn	2017-06-30;2017-1	NPC1	chr18	21113356	21113356	http://gnomad.b	0.0008806340565	ASJ	0.002741670003	1		2	0	https://www.ncbi.nlm.nih.gov/clinvar/variation/282371/
NM_012203.1(GRHPR):c.801A>C	concordant	unreportable	unreportable	366859	0		1	SCV000480141	Uncertain significance	Illumina Clinical Se	10/18/2016	GRHPR	chr9	37432071	37432071	http://gnomad.b	0.002362510841	ASJ	0.0220305007	2		3	0	https://www.ncbi.nlm.nih.gov/clinvar/variation/366859/
NM_001360.2(DHCR7):c.1012G>A	concordant	unreportable	unreportable	93706	0		2	SCV000111521;S	Uncertain significance;Unc	EGL Genetic Diagn	2017-06-30;2018-0	DHCR7	chr11	71146837	71146837	http://gnomad.b	0.0009558633465	EAS	0.003152000019	1		0	0	https://www.ncbi.nlm.nih.gov/clinvar/variation/93706/
NM_000642.2(AGL):c.2885C>G	concordant	unreportable	unreportable	256732	0		2	SCV000522560;S	Benign;Benign	GeneDx;Invitae	2017-11-28;2017-1	AGL	chr1	100356848	100356848	http://gnomad.b	0.004041373844	AFR	0.04706500098	12		37	0	https://www.ncbi.nlm.nih.gov/clinvar/variation/256732/
NM_000271.4(NPC1):c.873G>T	concordant	unreportable	unreportable	289805	0		1	SCV000344223	Uncertain significance	EGL Genetic Diagn	6/30/2017	NPC1	chr18	21140203	21140203	http://gnomad.b	0.0001258048652	AFR	0.00112003996	0		0	0	https://www.ncbi.nlm.nih.gov/clinvar/variation/289805/
NM_004543.4(NEB):c.2832C>T	concordant	unreportable	unreportable	167340	0		3	SCV000522873;S	Benign;Uncertain significar	GeneDx;Illumina C	2017-11-28;2016-1	NEB	chr2	152541295	152541295	http://gnomad.b	0.007553774822	NFE	0.01045370009	9		11	0	https://www.ncbi.nlm.nih.gov/clinvar/variation/167340/
NM_017890.4(VPS13B):c.10124C>T	concordant	unreportable	unreportable	130717	0		2	SCV000470848;S	Uncertain significance;Beni	Illumina Clinical Se	2016-10-18;2017-1	VPS13B	chr8	100861110	100861110	http://gnomad.b	0.009079201545	ASJ	0.01705930009	9		17	0	https://www.ncbi.nlm.nih.gov/clinvar/variation/130717/
NM_000112.3(SLC26A2):c.1410A>G	concordant	unreportable	unreportable	281117	0		3	SCV000331422;S	Benign;Likely benign;Benig	EGL Genetic Diagn	2017-06-30;2016-1	SLC26A2	chr5	149360566	149360566	http://gnomad.b	0.002726497204	SAS	0.03567250073	20		13	0	https://www.ncbi.nlm.nih.gov/clinvar/variation/281117/
NM_000352.3(ABCC8):c.4728C>T	concordant	unreportable	unreportable	157706	0		1	SCV000483251	Likely benign	Illumina Clinical Se	10/18/2016	ABCC8	chr11	17414556	17414556	http://gnomad.b	0.00337089672	AFR	0.03803420067	7		18	0	https://www.ncbi.nlm.nih.gov/clinvar/variation/157706/
NM_000053.3(ATP7B):c.4311G>A	concordant	unreportable	unreportable	35732	0		3	SCV000602613;S	Benign;Benign;Benign	ARUP Laboratories	2017-06-30;2017-1	ATP7B	chr13	52508979	52508979	http://gnomad.b	0.007118566073	NFE	0.0114601003	16		18	0	https://www.ncbi.nlm.nih.gov/clinvar/variation/35732/
NM_005562.2(LAMC2):c.2570G>C	concordant	unreportable	unreportable	294009	0		1	SCV000351682	Uncertain significance	Illumina Clinical Se	10/18/2016	LAMC2	chr1	183205708	183205708	http://gnomad.b	0.004135146788	NFE	0.006441080011	4		5	0	https://www.ncbi.nlm.nih.gov/clinvar/variation/294009/
NM_000053.3(ATP7B):c.1707+9T>C	concordant	unreportable	unreportable	35704	0		2	SCV000521844;S	Benign;Benign	GeneDx;Invitae	2017-11-28;2017-1	ATP7B	chr13	52542571	52542571	http://gnomad.b	0.003585939078	AFR	0.04258240014	17		23	0	https://www.ncbi.nlm.nih.gov/clinvar/variation/35704/
NM_138694.3(PKHD1):c.1675C>T	concordant	unreportable	unreportable	496515	0		1	SCV000699851	Benign	Laboratory Corpora	1/25/2018	PKHD1	chr6	51921514	51921514	http://gnomad.b	0.001868140422	EAS	0.02169629931	5		10	0	https://www.ncbi.nlm.nih.gov/clinvar/variation/496515/
NM_000030.2(AGXT):c.705G>A	concordant	unreportable	unreportable	204047	0		1	SCV000429368	Likely benign	Illumina Clinical Se	10/18/2016	AGXT	chr2	241814550	241814550	http://gnomad.b	0.004052957047	AFR	0.05050969869	23		25	0	https://www.ncbi.nlm.nih.gov/clinvar/variation/204047/
NM_014363.4(SACS):c.10982C>T	concordant	unreportable	unreportable	212109	0		3	SCV000614927;S	Uncertain significance;Unc	Athena Diagnostics	2017-08-17;2016-1	SACS	chr13	23907033	23907033	http://gnomad.b	0.001327895286	AFR	0.004530529957	1		1	0	https://www.ncbi.nlm.nih.gov/clinvar/variation/212109/
NM_000053.3(ATP7B):c.3160A>C	concordant	unreportable	unreportable	388652	0		1	SCV000531002	Likely benign	GeneDx	11/28/2017	ATP7B	chr13	52518328	52518328	http://gnomad.b	0.0002385014254	ASJ	0.0008126780158	0		0	0	https://www.ncbi.nlm.nih.gov/clinvar/variation/388652/
NM_002435.1(MPI):c.414G>A	concordant	unreportable	unreportable	449251	0		1	SCV000617147	Uncertain significance	GeneDx	11/28/2017	MPI	chr15	75185070	75185070	http://gnomad.b	0.0006186248055	AFR	0.007318350021	0		1	0	https://www.ncbi.nlm.nih.gov/clinvar/variation/449251/
NM_001164277.1(SLC37A4):c.467C>T	concordant	unreportable	unreportable	215177	0		1	SCV000252288	Uncertain significance	GeneDx	11/28/2017	SLC37A4	chr11	118898497	118898497	http://gnomad.b	0.001530940075	FIN	0.003323619952	2		0	0	https://www.ncbi.nlm.nih.gov/clinvar/variation/215177/
NM_014363.4(SACS):c.4466A>G	concordant	unreportable	unreportable	212114	0		2	SCV000704701;S	Benign;Benign	EGL Genetic Diagn	2017-06-30;2017-1	SACS	chr13	23913549	23913549	http://gnomad.b	0.01155387461	ASJ	0.04315720126	32		47	0	https://www.ncbi.nlm.nih.gov/clinvar/variation/212114/
NM_138694.3(PKHD1):c.6245C>T	concordant	unreportable	unreportable	197049	0		2	SCV000464075;S	Likely benign;Benign	Illumina Clinical Se	2016-10-18;2017-0	PKHD1	chr6	51777251	51777251	http://gnomad.b	0.001751381646	FIN	0.06085199863	43		3	0	https://www.ncbi.nlm.nih.gov/clinvar/variation/197049/
NM_017890.4(VPS13B):c.2485G>A	concordant	unreportable	unreportable	95839	0		2	SCV000153486;S	Likely benign;Benign	Genetic Services La	2014-06-27;2017-1	VPS13B	chr8	100205255	100205255	http://gnomad.b	0.008788039979	NFE	0.01220390014	13		19	0	https://www.ncbi.nlm.nih.gov/clinvar/variation/95839/
NM_024649.4(BBS1):c.235G>A	concordant	unreportable	unreportable	305458	0		1	SCV000373259	Uncertain significance	Illumina Clinical Se	10/18/2016	BBS1	chr11	66281952	66281952	http://gnomad.b	0.000799754779	NFE	0.001317420043	0		0	0	https://www.ncbi.nlm.nih.gov/clinvar/variation/305458/
NM_025136.3(OPA3):c.478C>T	concordant	unreportable	unreportable	449077	0		2	SCV000709437;S	Uncertain significance;Unc	EGL Genetic Diagn	2017-06-30;2017-1	OPA3	chr19	46056834	46056834	http://gnomad.b	0.0003621512901	AFR	0.004396049771	0		0	0	https://www.ncbi.nlm.nih.gov/clinvar/variation/449077/
NM_033056.3(PCDH15):c.5414C>T	concordant	unreportable	unreportable	46500	0		1	SCV000203201	Likely benign	EGL Genetic Diagn	6/30/2017	PCDH15	chr10	55582072	55582072	http://gnomad.b	0.001254950368	AFR	0.006974840071	0		3	0	https://www.ncbi.nlm.nih.gov/clinvar/variation/46500/
NM_000027.3(AGA):c.1023A>G	concordant	unreportable	unreportable	348228	0		1	SCV000448728	Uncertain significance	Illumina Clinical Se	10/18/2016	AGA	chr4	178352880	178352880	http://gnomad.b	0.008474425553	FIN	0.0188424997	19		12	0	https://www.ncbi.nlm.nih.gov/clinvar/variation/348228/
NM_000057.2(BLM):c.3128C>A	concordant	unreportable	unreportable	136516	0		3	SCV000340424;S	Benign;Likely benign;Benig	EGL Genetic Diagn	2017-06-30;2017-0	BLM	chr15	91337505	91337505	http://gnomad.b	0.0012010655	AFR	0.01165680029	1		3	0	https://www.ncbi.nlm.nih.gov/clinvar/variation/136516/
NM_017890.4(VPS13B):c.4247G>A	concordant	unreportable	unreportable	130725	0		2	SCV000153491;S	Likely benign;Likely benign	Genetic Services La	2014-06-27;2016-1	VPS13B	chr8	100520087	100520087	http://gnomad.b	0.00324477169	AFR	0.03855710104	10		24	0	https://www.ncbi.nlm.nih.gov/clinvar/variation/130725/
NM_000108.3(DLD):c.74A>C	concordant	unreportable	unreportable	358564	0		1	SCV000466204	Uncertain significance	Illumina Clinical Se	10/18/2016	DLD	chr7	107533679	107533679	http://gnomad.b	0.000127214572	SAS	0.0005543599837	1		0	0	https://www.ncbi.nlm.nih.gov/clinvar/variation/358564/
NM_000053.3(ATP7B):c.-54G>T	concordant	unreportable	unreportable	312402	0		2	SCV000602602;S	Uncertain significance;Unc	ARUP Laboratories	2017-06-30;2016-1	ATP7B	chr13	52585527	52585527		0.001731239758					14	0	https://www.ncbi.nlm.nih.gov/clinvar/variation/312402/
NM_000360.3(TH):c.627C>G	concordant	unreportable	unreportable	304077	0		1	SCV000369921	Uncertain significance	Illumina Clinical Se	10/18/2016	TH	chr11	2189113	2189113	http://gnomad.b	0.0007972235212	EAS	0.006501949858	2		0	0	https://www.ncbi.nlm.nih.gov/clinvar/variation/304077/
NM_000352.3(ABCC8):c.2538C>T	concordant	unreportable	unreportable	157692	0		1	SCV000369319	Likely benign	Illumina Clinical Se	10/18/2016	ABCC8	chr11	17434231	17434231	http://gnomad.b	0.003071490886	AFR	0.03304380178	15		20	0	https://www.ncbi.nlm.nih.gov/clinvar/variation/157692/
NM_000360.3(TH):c.267G>A	concordant	unreportable	unreportable	263254	0		3	SCV000338376;S	Benign;Likely benign;Benig	EGL Genetic Diagn	2017-06-30;2016-1	TH	chr11	2190925	2190925	http://gnomad.b	0.01021178539	SAS	0.04484599829	52		55	0	https://www.ncbi.nlm.nih.gov/clinvar/variation/263254/
NM_000155.3(GALT):c.564+15G>A	concordant	unreportable	unreportable	25216	0		2	SCV000330958;S	Likely benign;Likely benign	EGL Genetic Diagn	2017-06-30;2018-0	GALT	chr9	34648183	34648183	http://gnomad.b	0.0002226290011	AMR	0.004496459849	0		0	0	https://www.ncbi.nlm.nih.gov/clinvar/variation/25216/
NM_015166.3(MLC1):c.594C>T	concordant	unreportable	unreportable	129614	0		2	SCV000151857;S	Likely benign;Likely benign	Genetic Services La	2014-06-27;2016-1	MLC1	chr22	50515273	50515273	http://gnomad.b	0.005954312052	SAS	0.2228289992	2131		147	0	https://www.ncbi.nlm.nih.gov/clinvar/variation/129614/
NM_033056.3(PCDH15):c.546A>G	concordant	unreportable	unreportable	46501	0		1	SCV000363242	Uncertain significance	Illumina Clinical Se	10/18/2016	PCDH15	chr10	56106173	56106173	http://gnomad.b	0.01701640693	ASJ	0.02640129998	33		79	0	https://www.ncbi.nlm.nih.gov/clinvar/variation/46501/
NM_000051.3(ATM):c.2442C>A	concordant	unreportable	unreportable	133607	0		1	SCV000262027	Benign	Invitae	10/5/2017	ATM	chr11	108129778	108129778	http://gnomad.b	0.002094100873	AFR	0.02474470064	8		11	0	https://www.ncbi.nlm.nih.gov/clinvar/variation/133607/
NM_012434.4(SLC17A5):c.1324G>A	concordant	unreportable	unreportable	235209	0		1	SCV000334655	Benign	EGL Genetic Diagn	6/30/2017	SLC17A5	chr6	74310100	74310100	http://gnomad.b	0.001917696156	EAS	0.006842169911	1		1	0	https://www.ncbi.nlm.nih.gov/clinvar/variation/235209/
NM_000271.4(NPC1):c.1819C>A	concordant	unreportable	unreportable	501524	0		1	SCV000707921	Uncertain significance	EGL Genetic Diagn	6/30/2017	NPC1	chr18	21125052	21125052	http://gnomad.b	5.15E-05	AFR	0.0003267120046	0		0	0	https://www.ncbi.nlm.nih.gov/clinvar/variation/501524/
NM_017890.4(VPS13B):c.11688C>T	concordant	unreportable	unreportable	95828	0		2	SCV000597903;S	Likely benign;Uncertain sig	Genetic Services La	2017-07-05;2016-1	VPS13B	chr8	100883793	100883793	http://gnomad.b	0.0008258909824	AMR	0.001518759993	0		0	0	https://www.ncbi.nlm.nih.gov/clinvar/variation/95828/
NM_000152.3(GAA):c.2415G>A	concordant	unreportable	unreportable	194697	0		4	SCV000226557;S	Uncertain significance;Likel	EGL Genetic Diagn	2017-06-30;2017-1	GAA	chr17	78091482	78091482	http://gnomad.b	0.0009338469492	NFE	0.001440210035	0		1	0	https://www.ncbi.nlm.nih.gov/clinvar/variation/194697/
NM_000051.3(ATM):c.5793T>C	concordant	unreportable	unreportable	128459	0		6	SCV000602553;S	Benign;Benign;Benign;Likel	ARUP Laboratories	2017-06-30;2017-0	ATM	chr11	108180917	108180917	http://gnomad.b	0.005811686865	EAS	0.01114340033	5		11	0	https://www.ncbi.nlm.nih.gov/clinvar/variation/128459/
NM_005562.2(LAMC2):c.889A>G	concordant	unreportable	unreportable	290575	0		2	SCV000345151;S	Uncertain significance;Unc	EGL Genetic Diagn	2017-06-30;2016-1	LAMC2	chr1	183192395	183192395	http://gnomad.b	0.001542630928	NFE	0.002642659936	0		0	0	https://www.ncbi.nlm.nih.gov/clinvar/variation/290575/
NM_000136.2(FANCC):c.1162G>A	concordant	unreportable	unreportable	220210	0		2	SCV000564979;S	Uncertain significance;Unc	GeneDx;Invitae	2017-11-28;2017-1	FANCC	chr9	97873912	97873912	http://gnomad.b	2.62E-05	AFR	6.54E-05	0		0	0	https://www.ncbi.nlm.nih.gov/clinvar/variation/220210/
NM_017890.4(VPS13B):c.10140G>T	concordant	unreportable	unreportable	459245	0		1	SCV000630854	Benign	Invitae	10/5/2017	VPS13B	chr8	100865682	100865682	http://gnomad.b	0.02000335956	NFE	0.02607320063	48		100	0	https://www.ncbi.nlm.nih.gov/clinvar/variation/459245/
NM_024685.3(BBS10):c.1158G>A	concordant	unreportable	unreportable	310492	0		2	SCV000381151;S	Uncertain significance;Likel	Illumina Clinical Se	2016-10-18;2018-0	BBS10	chr12	76740607	76740607	http://gnomad.b	0.001964075877	ASJ	0.01361509971	0		4	0	https://www.ncbi.nlm.nih.gov/clinvar/variation/310492/
NM_017890.4(VPS13B):c.7753G>A	concordant	unreportable	unreportable	95867	0		3	SCV000512679;S	Likely benign;Likely benign	GeneDx;Genetic Se	2017-11-28;2017-1	VPS13B	chr8	100791158	100791158	http://gnomad.b	0.003787899997	NFE	0.005302510224	3		2	0	https://www.ncbi.nlm.nih.gov/clinvar/variation/95867/
NM_138694.3(PKHD1):c.1736C>T	concordant	unreportable	unreportable	96381	0		1	SCV000557657	Benign	Invitae	10/5/2017	PKHD1	chr6	51920485	51920485	http://gnomad.b	0.02572029045	NFE	0.03619299829	104		145	0	https://www.ncbi.nlm.nih.gov/clinvar/variation/96381/
NM_006493.2(CLN5):c.1166A>C	concordant	unreportable	unreportable	205133	0		2	SCV000240630;S	Uncertain significance;Unc	GeneDx;Invitae	2017-11-28;2017-1	CLN5	chr13	77575046	77575046	http://gnomad.b	0.0001202884685	ASJ	0.0001139730011	0		0	0	https://www.ncbi.nlm.nih.gov/clinvar/variation/205133/
NM_000277.1(PAH):c.*79G>A	concordant	unreportable	unreportable	306912	0		1	SCV000375558	Uncertain significance	Illumina Clinical Se	10/18/2016	PAH	chr12	103232874	103232874		0.0003397129973					0	0	https://www.ncbi.nlm.nih.gov/clinvar/variation/306912/
NM_000137.2(FAH):c.1259G>A	concordant	unreportable	unreportable	291101	0		2	SCV000345799;S	Benign;Likely benign	EGL Genetic Diagn	2017-06-30;2017-1	FAH	chr15	80478550	80478550	http://gnomad.b	0.001625691474	FIN	0.008926169947	4		0	0	https://www.ncbi.nlm.nih.gov/clinvar/variation/291101/
NM_006261.4(PROP1):c.471C>T	concordant	unreportable	unreportable	286283	0		2	SCV000339645;S	Uncertain significance;Unc	EGL Genetic Diagn	2017-06-30;2017-0	PROP1	chr5	177419920	177419920	http://gnomad.b	0.0012676342	ASJ	0.00417090999	0		0	0	https://www.ncbi.nlm.nih.gov/clinvar/variation/286283/
NM_138694.3(PKHD1):c.10354A>G	concordant	unreportable	unreportable	498158	0		1	SCV000703032	Uncertain significance	EGL Genetic Diagn	6/30/2017	PKHD1	chr6	51524570	51524570	http://gnomad.b	0.0001223187181	AMR	0.0005078570102	0		0	0	https://www.ncbi.nlm.nih.gov/clinvar/variation/498158/
NM_003060.3(SLC22A5):c.1249A>G	concordant	unreportable	unreportable	460395	0		1	SCV000632519	Benign	Invitae	10/5/2017	SLC22A5	chr5	131726578	131726578	http://gnomad.b	0.001081664238	AFR	0.01215530001	1		2	0	https://www.ncbi.nlm.nih.gov/clinvar/variation/460395/
NM_000051.3(ATM):c.3150T>C	concordant	unreportable	unreportable	135748	0		2	SCV000682105;S	Likely benign;Benign	Color Genomics, In	2017-10-26;2017-1	ATM	chr11	108143331	108143331	http://gnomad.b	0.0004984628408	FIN	0.009240150452	1		2	0	https://www.ncbi.nlm.nih.gov/clinvar/variation/135748/
NM_000492.3(CFTR):c.2898G>A	concordant	unreportable	unreportable	35853	0		3	SCV000602992;S	Benign;Likely benign;Benig	ARUP Laboratories	2017-06-30;2016-1	CFTR	chr7	117243826	117243826	http://gnomad.b	0.007466452001	ASJ	0.01604389958	5		19	0	https://www.ncbi.nlm.nih.gov/clinvar/variation/35853/
NM_004646.3(NPHS1):c.2961T>C	concordant	unreportable	unreportable	328859	0		1	SCV000411558	Uncertain significance	Illumina Clinical Se	10/18/2016	NPHS1	chr19	36330287	36330287	http://gnomad.b	0.0002007035775	ASJ	0.001624369994	0		0	0	https://www.ncbi.nlm.nih.gov/clinvar/variation/328859/
NM_004328.4(BCS1L):c.613G>A	concordant	unreportable	unreportable	214159	0		1	SCV000602631	Benign	ARUP Laboratories	6/30/2017	BCS1L	chr2	219526634	219526634	http://gnomad.b	0.003084181995	AMR	0.05403229967	59		19	0	https://www.ncbi.nlm.nih.gov/clinvar/variation/214159/
NM_000642.2(AGL):c.2390A>G	concordant	unreportable	unreportable	456470	0		1	SCV000626694	Uncertain significance	Invitae	10/5/2017	AGL	chr1	100349757	100349757	http://gnomad.b	0.00204022395	NFE	0.002803080017	1		2	0	https://www.ncbi.nlm.nih.gov/clinvar/variation/456470/
NM_000528.3(MAN2B1):c.1068C>G	concordant	unreportable	unreportable	328274	0		1	SCV000410800	Uncertain significance	Illumina Clinical Se	10/18/2016	MAN2B1	chr19	12769283	12769283	http://gnomad.b	0.005966264409	ASJ	0.02467009984	5		8	0	https://www.ncbi.nlm.nih.gov/clinvar/variation/328274/
NM_133647.1(SLC12A6):c.2925G>A	concordant	unreportable	unreportable	159894	0		2	SCV000521486;S	Benign;Likely benign	GeneDx;Genetic Se	2017-11-28;2014-0	SLC12A6	chr15	34529629	34529629	http://gnomad.b	0.002029894268	AFR	0.02136700042	2		3	0	https://www.ncbi.nlm.nih.gov/clinvar/variation/159894/
NM_002485.4(NBN):c.279G>A	concordant	unreportable	unreportable	136044	0		2	SCV000685769;S	Benign;Benign	Color Genomics, In	2017-10-26;2017-1	NBN	chr8	90993644	90993644	http://gnomad.b	5.86E-05	AMR	0.0008935490041	1		0	0	https://www.ncbi.nlm.nih.gov/clinvar/variation/136044/
NM_001360.2(DHCR7):c.25A>T	concordant	unreportable	unreportable	439595	0		1	SCV000603314	Benign	ARUP Laboratories	6/30/2017	DHCR7	chr11	71155974	71155974	http://gnomad.b	0.002765272273	AFR	0.03182170168	8		15	0	https://www.ncbi.nlm.nih.gov/clinvar/variation/439595/
NM_138694.3(PKHD1):c.10515C>A	concordant	unreportable	unreportable	167474	0		1	SCV000260345	Benign	Invitae	10/5/2017	PKHD1	chr6	51524409	51524409	http://gnomad.b	0.007358582882	NFE	0.0113597	7		18	0	https://www.ncbi.nlm.nih.gov/clinvar/variation/167474/
NM_000153.3(GALC):c.330C>T	concordant	unreportable	unreportable	92502	0		2	SCV000389261;S	Likely benign;Benign	Illumina Clinical Se	2016-10-18;2017-1	GALC	chr14	88452945	88452945	http://gnomad.b	0.03470054487	FIN	0.05653579906	191		291	0	https://www.ncbi.nlm.nih.gov/clinvar/variation/92502/
NM_138694.3(PKHD1):c.2168G>T	concordant	unreportable	unreportable	498428	0		1	SCV000703433	Benign	EGL Genetic Diagn	6/30/2017	PKHD1	chr6	51915066	51915066	http://gnomad.b	0.001128668172	SAS	0.01004359964	4		1	0	https://www.ncbi.nlm.nih.gov/clinvar/variation/498428/
NM_014363.4(SACS):c.4302A>G	concordant	unreportable	unreportable	311544	0		2	SCV000614956;S	Likely benign;Uncertain sig	Athena Diagnostics	2017-08-17;2016-1	SACS	chr13	23913713	23913713	http://gnomad.b	0.001197476999	SAS	0.008524759673	5		5	0	https://www.ncbi.nlm.nih.gov/clinvar/variation/311544/
NM_000492.3(CFTR):c.1210-7_1210-6	concordant	unreportable	unreportable	161188	0		1	SCV000466509	Likely benign	Illumina Clinical Se	10/18/2016	CFTR	chr7	117188682	117188682		0.0008184787465					25	0	https://www.ncbi.nlm.nih.gov/clinvar/variation/161188/
NM_000466.2(PEX1):c.2718+3A>G	concordant	unreportable	unreportable	360920	0		1	SCV000470505	Uncertain significance	Illumina Clinical Se	10/18/2016	PEX1	chr7	92129015	92129015	http://gnomad.b	0.001611373356	SAS	0.01495159976	4		7	0	https://www.ncbi.nlm.nih.gov/clinvar/variation/360920/

Supplemental Table 2

NM_000520.4(HEXA):c.1074-43C>G	concordant	unreportable	unreportable	256349	0		1	SCV000700303	Benign	EGL Genetic Diagn	6/30/2017	HEXA	chr15	72640142	72640142	http://gnomad.b	0.009224347205	AFR	0.1760459989	418		238	0	https://www.ncbi.nlm.nih.gov/clinvar/variation/256349/
NM_000153.3(GALC):c.1072C>T	concordant	unreportable	unreportable	314751	0		3	SCV000513101;S	Benign;Likely benign;Benig	GeneDx;Illumina C	2017-11-28;2016-1	GALC	chr14	88429817	88429817	http://gnomad.b	0.004645945916	AFR	0.05621730164	20		41	0	https://www.ncbi.nlm.nih.gov/clinvar/variation/314751/
NM_020533.2(MCOLN1):c.771C>A	concordant	unreportable	unreportable	330493	0		1	SCV000415608	Uncertain significance	Illumina Clinical Se	10/18/2016	MCOLN1	chr19	7592840	7592840	http://gnomad.b	0.002205105759	AFR	0.02719309926	8		11	0	https://www.ncbi.nlm.nih.gov/clinvar/variation/330493/
NM_000051.3(ATM):c.4853G>A	concordant	unreportable	unreportable	184966	0		1	SCV000282970	Uncertain significance	Invitae	10/5/2017	ATM	chr11	108165730	108165730	http://gnomad.b	4.73E-05	SAS	0.0002598919964	0		0	0	https://www.ncbi.nlm.nih.gov/clinvar/variation/184966/
NM_000053.3(ATP7B):c.3045G>A	concordant	unreportable	unreportable	35716	0		3	SCV000602586;S	Benign;Benign;Likely benig	ARUP Laboratories	2017-06-30;2017-1	ATP7B	chr13	52520435	52520435	http://gnomad.b	0.02858088846	NFE	0.03943039849	112		216	0	https://www.ncbi.nlm.nih.gov/clinvar/variation/35716/
NM_005562.2(LAMC2):c.493C>T	concordant	unreportable	unreportable	290568	0		1	SCV000345142	Likely benign	EGL Genetic Diagn	6/30/2017	LAMC2	chr1	183187613	183187613	http://gnomad.b	0.002505725372	NFE	0.003818759928	0		4	0	https://www.ncbi.nlm.nih.gov/clinvar/variation/290568/
NM_138694.3(PKHD1):c.6777C>T	concordant	unreportable	unreportable	167484	0		2	SCV000464072;S	Uncertain significance;Beni	Illumina Clinical Se	2016-10-18;2017-0	PKHD1	chr6	51771044	51771044	http://gnomad.b	0.004000378076	FIN	0.01449989993	8		8	0	https://www.ncbi.nlm.nih.gov/clinvar/variation/167484/
NM_000271.4(NPC1):c.2073G>A	concordant	unreportable	unreportable	194158	0		3	SCV000225764;S	Benign;Benign;Likely benig	EGL Genetic Diagn	2017-06-30;2017-1	NPC1	chr18	21124365	21124365	http://gnomad.b	0.006696249871	SAS	0.009973360226	10		14	0	https://www.ncbi.nlm.nih.gov/clinvar/variation/194158/
NM_024649.4(BBS1):c.569A>T	concordant	unreportable	unreportable	389028	0		1	SCV000531445	Uncertain significance	GeneDx	11/28/2017	BBS1	chr11	66283382	66283382	http://gnomad.b	3.90E-05	AMR	0.001072000014	0		1	0	https://www.ncbi.nlm.nih.gov/clinvar/variation/389028/
NM_000136.2(FANCC):c.1407G>A	concordant	unreportable	unreportable	137284	0		1	SCV000259562	Benign	Invitae	10/5/2017	FANCC	chr9	97869474	97869474	http://gnomad.b	0.0004803782146	EAS	0.005509159993	1		2	0	https://www.ncbi.nlm.nih.gov/clinvar/variation/137284/
NM_001079802.1(FKTN):c.166C>T	concordant	unreportable	unreportable	93517	0		5	SCV000613316;S	Benign;Likely benign;Uncer	Athena Diagnostics	2017-08-17;2014-0	FKTN	chr9	108363426	108363426	http://gnomad.b	0.01979987377	FIN	0.03137850016	75		83	0	https://www.ncbi.nlm.nih.gov/clinvar/variation/93517/
NM_004543.4(NEB):c.6159G>A	concordant	unreportable	unreportable	129754	0		3	SCV000527132;S	Benign;Likely benign;Benig	GeneDx;Genetic Se	2017-11-28;2014-0	NEB	chr2	152514521	152514521	http://gnomad.b	0.01025413315	ASJ	0.01589740068	12		28	0	https://www.ncbi.nlm.nih.gov/clinvar/variation/129754/
NM_000642.2(AGL):c.3619G>A	concordant	unreportable	unreportable	256741	0		2	SCV000528007;S	Benign;Benign	GeneDx;Invitae	2017-11-28;2017-1	AGL	chr1	100368269	100368269	http://gnomad.b	0.002425382507	AFR	0.03076010011	11		14	0	https://www.ncbi.nlm.nih.gov/clinvar/variation/256741/
NM_000057.2(BLM):c.1122T>C	concordant	unreportable	unreportable	136510	0		2	SCV000394413;S	Likely benign;Benign	Illumina Clinical Se	2016-10-18;2017-1	BLM	chr15	91303411	91303411	http://gnomad.b	0.004171621557	FIN	0.0315275006	29		23	0	https://www.ncbi.nlm.nih.gov/clinvar/variation/136510/
NM_017890.4(VPS13B):c.56A>G	concordant	unreportable	unreportable	195336	0		2	SCV000227469;S	Uncertain significance;Unc	EGL Genetic Diagn	2017-06-30;2016-1	VPS13B	chr8	100026072	100026072	http://gnomad.b	7.56E-05	NFE	0.0001342640026	0		0	0	https://www.ncbi.nlm.nih.gov/clinvar/variation/195336/
NM_001042432.1(CLN3):c.45G>A	concordant	unreportable	unreportable	136783	0		2	SCV000202484;S	Benign;Benign	EGL Genetic Diagn	2017-06-30;2017-1	CLN3	chr16	28503036	28503036	http://gnomad.b	0.001986066976	SAS	0.01443149988	9		8	0	https://www.ncbi.nlm.nih.gov/clinvar/variation/136783/
NM_000271.4(NPC1):c.3331C>T	concordant	unreportable	unreportable	92711	0		1	SCV000407847	Uncertain significance	Illumina Clinical Se	10/18/2016	NPC1	chr18	21115579	21115579	http://gnomad.b	0.001452474353	NFE	0.002220189897	1		3	0	https://www.ncbi.nlm.nih.gov/clinvar/variation/92711/
NM_001079802.1(FKTN):c.1297A>G	concordant	unreportable	unreportable	36137	0		5	SCV000613314;S	Uncertain significance;Likel	Athena Diagnostics	2017-08-17;2017-0	FKTN	chr9	108397456	108397456	http://gnomad.b	0.001698449057	FIN	0.007713700179	2		0	0	https://www.ncbi.nlm.nih.gov/clinvar/variation/36137/
NM_000153.3(GALC):c.1573G>A	concordant	unreportable	unreportable	314747	0		1	SCV000389242	Uncertain significance	Illumina Clinical Se	10/18/2016	GALC	chr14	88411994	88411994	http://gnomad.b	0.0004727061166	EAS	0.002725270111	0		0	0	https://www.ncbi.nlm.nih.gov/clinvar/variation/314747/
NM_005562.2(LAMC2):c.3000T>A	concordant	unreportable	unreportable	294014	0		1	SCV000351689	Uncertain significance	Illumina Clinical Se	10/18/2016	LAMC2	chr1	183208629	183208629	http://gnomad.b	0.001867395333	AFR	0.02170789987	2		9	0	https://www.ncbi.nlm.nih.gov/clinvar/variation/294014/
NM_000520.4(HEXA):c.759G>A	concordant	unreportable	unreportable	256352	0		3	SCV000603955;S	Benign;Benign;Benign	ARUP Laboratories	2017-06-30;2017-0	HEXA	chr15	72642905	72642905	http://gnomad.b	0.004836384638	FIN	0.006863450166	9		5	0	https://www.ncbi.nlm.nih.gov/clinvar/variation/256352/
NM_138694.3(PKHD1):c.11400G>A	concordant	unreportable	unreportable	287103	0		1	SCV000340771	Uncertain significance	EGL Genetic Diagn	6/30/2017	PKHD1	chr6	51503753	51503753	http://gnomad.b	0.0007589320464	ASJ	0.009756100364	0		1	0	https://www.ncbi.nlm.nih.gov/clinvar/variation/287103/
NM_000492.3(CFTR):c.3285A>T	concordant	unreportable	unreportable	195464	0		5	SCV000603000;S	Benign;Benign;Likely benig	ARUP Laboratories	2017-06-30;2017-0	CFTR	chr7	117251780	117251780	http://gnomad.b	0.005504028841	NFE	0.008123669773	4		10	0	https://www.ncbi.nlm.nih.gov/clinvar/variation/195464/
NM_000137.2(FAH):c.483C>T	concordant	unreportable	unreportable	381848	0		2	SCV000521526;S	Benign;Benign	GeneDx;Invitae	2017-11-28;2017-1	FAH	chr15	80460421	80460421	http://gnomad.b	0.002507892649	AFR	0.03116830066	4		13	0	https://www.ncbi.nlm.nih.gov/clinvar/variation/381848/
NM_000051.3(ATM):c.-186G>A	concordant	unreportable	unreportable	302239	0		1	SCV000367015	Uncertain significance	Illumina Clinical Se	10/18/2016	ATM	chr11	108093758	108093758		4.46E-05					0	0	https://www.ncbi.nlm.nih.gov/clinvar/variation/302239/
NM_000642.2(AGL):c.4300A>G	concordant	unreportable	unreportable	456503	0		1	SCV000626752	Uncertain significance	Invitae	10/5/2017	AGL	chr1	100382006	100382006	http://gnomad.b	5.58E-05	NFE	0.0001169169991	0		0	0	https://www.ncbi.nlm.nih.gov/clinvar/variation/456503/
NM_001128227.2(GNE):c.2179G>T	concordant	unreportable	unreportable	194025	0		4	SCV000225561;S	Uncertain significance;Unc	EGL Genetic Diagn	2017-06-30;2017-1	GNE	chr9	36217445	36217445	http://gnomad.b	0.0009385330868	NFE	0.001450369949	0		1	0	https://www.ncbi.nlm.nih.gov/clinvar/variation/194025/
NM_000053.3(ATP7B):c.3366A>G	concordant	unreportable	unreportable	254767	0		2	SCV000526107;S	Benign;Benign	GeneDx;Invitae	2017-11-28;2017-1	ATP7B	chr13	52516568	52516568	http://gnomad.b	0.003184695504	AFR	0.03678970039	13		19	0	https://www.ncbi.nlm.nih.gov/clinvar/variation/254767/
NM_000543.4(SMPD1):c.1749G>A	concordant	unreportable	unreportable	256593	0		1	SCV000372947	Uncertain significance	Illumina Clinical Se	10/18/2016	SMPD1	chr11	6415690	6415690	http://gnomad.b	0.0177188392	FIN	0.03864860162	71		98	0	https://www.ncbi.nlm.nih.gov/clinvar/variation/256593/
NM_000151.3(G6PC):c.910G>A	concordant	unreportable	unreportable	214463	0		1	SCV000251524	Uncertain significance	GeneDx	11/28/2017	G6PC	chr17	41063279	41063279	http://gnomad.b	6.39E-05	EAS	0.0001159550011	0		0	0	https://www.ncbi.nlm.nih.gov/clinvar/variation/214463/
NM_000271.4(NPC1):c.3598A>G	concordant	unreportable	unreportable	284662	0		1	SCV000337368	Benign	EGL Genetic Diagn	6/30/2017	NPC1	chr18	21113475	21113475	http://gnomad.b	0.001017875728	AFR	0.01183629967	1		2	0	https://www.ncbi.nlm.nih.gov/clinvar/variation/284662/
NM_003060.3(SLC22A5):c.287G>C	concordant	unreportable	unreportable	25364	0		1	SCV000632545	Uncertain significance	Invitae	10/5/2017	SLC22A5	chr5	131705951	131705951	http://gnomad.b	5.08E-05	ASJ	0.006505160127	0		0	0	https://www.ncbi.nlm.nih.gov/clinvar/variation/25364/
NM_138694.3(PKHD1):c.3876C>T	concordant	unreportable	unreportable	241845	0		2	SCV000291332;S	Benign;Benign	Invitae;Laboratory	2017-03-14;2018-0	PKHD1	chr6	51890732	51890732	http://gnomad.b	0.003080207719	AFR	0.03308819979	12		19	0	https://www.ncbi.nlm.nih.gov/clinvar/variation/241845/
NM_000227.3(LAMA3):c.2678A>G	concordant	unreportable	unreportable	285244	0		2	SCV000338185;S	Uncertain significance;Unc	EGL Genetic Diagn	2017-06-30;2016-1	LAMA3	chr18	21494685	21494685	http://gnomad.b	0.0009966907627	ASJ	0.008541789837	0		0	0	https://www.ncbi.nlm.nih.gov/clinvar/variation/285244/
NM_000136.2(FANCC):c.1394A>G	concordant	unreportable	unreportable	134297	0		3	SCV000247342;S	Likely benign;Benign;Benig	Genetic Services La	2015-09-15;2017-1	FANCC	chr9	97869487	97869487	http://gnomad.b	0.001017831069	AFR	0.009873149917	0		4	0	https://www.ncbi.nlm.nih.gov/clinvar/variation/134297/
NM_017890.4(VPS13B):c.983A>G	concordant	unreportable	unreportable	198886	0		2	SCV000616281;S	Uncertain significance;Unc	Athena Diagnostics	2017-08-17;2017-0	VPS13B	chr8	100133450	100133450	http://gnomad.b	0.0006243175901	AMR	0.001043900033	0		0	0	https://www.ncbi.nlm.nih.gov/clinvar/variation/198886/
NM_003060.3(SLC22A5):c.59T>A	concordant	unreportable	unreportable	25352	0		1	SCV000632558	Benign	Invitae	10/5/2017	SLC22A5	chr5	131705723	131705723	http://gnomad.b	0.001073191672	AFR	0.01250830013	1		3	0	https://www.ncbi.nlm.nih.gov/clinvar/variation/25352/
NM_000152.3(GAA):c.1551+42G>A	concordant	unreportable	unreportable	496841	0		1	SCV000700380	Benign	EGL Genetic Diagn	6/30/2017	GAA	chr17	78084681	78084681	http://gnomad.b	0.002948844086	AFR	0.02772689983	8		11	0	https://www.ncbi.nlm.nih.gov/clinvar/variation/496841/
NM_000057.2(BLM):c.3625T>A	concordant	unreportable	unreportable	127501	0		3	SCV000343878;S	Uncertain significance;Unc	EGL Genetic Diagn	2017-06-30;2017-0	BLM	chr15	91347463	91347463	http://gnomad.b	0.0007111259097	ASJ	0.004163280129	0		0	0	https://www.ncbi.nlm.nih.gov/clinvar/variation/127501/
NM_133647.1(SLC12A6):c.963C>T	concordant	unreportable	unreportable	139118	0		1	SCV000390321	Likely benign	Illumina Clinical Se	10/18/2016	SLC12A6	chr15	34546704	34546704	http://gnomad.b	0.01395441183	AMR	0.0794024989	186		68	0	https://www.ncbi.nlm.nih.gov/clinvar/variation/139118/
NM_000271.4(NPC1):c.540C>T	concordant	unreportable	unreportable	471944	0		1	SCV000650850	Benign	Invitae	10/5/2017	NPC1	chr18	21141415	21141415	http://gnomad.b	0.002964991937	AMR	0.02251210064	9		3	0	https://www.ncbi.nlm.nih.gov/clinvar/variation/471944/
NM_000053.3(ATP7B):c.670A>T	concordant	unreportable	unreportable	312398	0		1	SCV000384685	Uncertain significance	Illumina Clinical Se	10/18/2016	ATP7B	chr13	52548686	52548686	http://gnomad.b	0.0007211160744	ASJ	0.008326560259	0		1	0	https://www.ncbi.nlm.nih.gov/clinvar/variation/312398/
NM_000051.3(ATM):c.2260C>A	concordant	unreportable	unreportable	141496	0		3	SCV000209698;S	Uncertain significance;Unc	GeneDx;Invitae;La	2017-11-28;2017-1	ATM	chr11	108128217	108128217		2.78E-05					0	0	https://www.ncbi.nlm.nih.gov/clinvar/variation/141496/
NM_000051.3(ATM):c.94C>T	concordant	unreportable	unreportable	142913	0		2	SCV000292845;S	Uncertain significance;Unc	GeneDx;Invitae	2017-11-28;2017-0	ATM	chr11	108098524	108098524	http://gnomad.b	8.35E-05	SAS	0.000552485988	0		1	0	https://www.ncbi.nlm.nih.gov/clinvar/variation/142913/
NM_002225.3(IVD):c.473G>A	concordant	unreportable	unreportable	315798	0		1	SCV000390859	Uncertain significance	Illumina Clinical Se	10/18/2016	IVD	chr15	40703466	40703466	http://gnomad.b	8.97E-05	EAS	0.0006957330042	0		0	0	https://www.ncbi.nlm.nih.gov/clinvar/variation/315798/
NM_001042432.1(CLN3):c.768C>T	concordant	unreportable	unreportable	93505	0		4	SCV000111296;S	Likely benign;Likely benign	EGL Genetic Diagn	2017-06-30;2017-0	CLN3	chr16	28495349	28495349	http://gnomad.b	0.001066642963	ASJ	0.007346849889	0		2	0	https://www.ncbi.nlm.nih.gov/clinvar/variation/93505/
NM_000243.2(MEFV):c.1467G>A	concordant	unreportable	unreportable	97446	0		3	SCV000604169;S	Benign;Benign;Likely benig	ARUP Laboratories	2017-06-30;2017-1	MEFV	chr16	3297136	3297136	http://gnomad.b	0.001886369997	FIN	0.01466370001	5		1	0	https://www.ncbi.nlm.nih.gov/clinvar/variation/97446/
NM_001876.3(CPT1A):c.863G>A	concordant	unreportable	unreportable	203655	0		3	SCV000603219;S	Benign;Benign;Benign	ARUP Laboratories	2017-06-30;2017-1	CPT1A	chr11	68562288	68562288	http://gnomad.b	0.004316972206	NFE	0.009577520192	7		2	0	https://www.ncbi.nlm.nih.gov/clinvar/variation/203655/
NM_000035.3(ALDOB):c.538C>A	concordant	unreportable	unreportable	501678	0		1	SCV000708150	Uncertain significance	EGL Genetic Diagn	6/30/2017	ALDOB	chr9	104189766	104189766	http://gnomad.b	0.0002510351675	SAS	0.003021250013	2		0	0	https://www.ncbi.nlm.nih.gov/clinvar/variation/501678/
NM_000155.3(GALT):c.510C>A	concordant	unreportable	unreportable	281074	0		2	SCV000331218;S	Benign;Uncertain significar	EGL Genetic Diagn	2017-06-30;2016-1	GALT	chr9	34648114	34648114	http://gnomad.b	0.001976175948	FIN	0.01201900002	5		0	0	https://www.ncbi.nlm.nih.gov/clinvar/variation/281074/
NM_000071.2(CBS):c.531+11G>A	concordant	unreportable	unreportable	136666	0		1	SCV000436226	Uncertain significance	Illumina Clinical Se	10/18/2016	CBS	chr21	44485715	44485715	http://gnomad.b	0.004234881249	NFE	0.007925660349	1		5	0	https://www.ncbi.nlm.nih.gov/clinvar/variation/136666/
NM_000153.3(GALC):c.997G>A	concordant	unreportable	unreportable	314753	0		1	SCV000389251	Uncertain significance	Illumina Clinical Se	10/18/2016	GALC	chr14	88431885	88431885	http://gnomad.b	0.0006209512302	AFR	0.007264399901	0		1	0	https://www.ncbi.nlm.nih.gov/clinvar/variation/314753/
NM_017890.4(VPS13B):c.11146G>A	concordant	unreportable	unreportable	95826	0		5	SCV000616264;S	Uncertain significance;Likel	Athena Diagnostics	2017-08-17;2016-0	VPS13B	chr8	100874030	100874030	http://gnomad.b	0.003286766147	NFE	0.004366630223	4		1	0	https://www.ncbi.nlm.nih.gov/clinvar/variation/95826/
NM_000396.3(CTSK):c.169A>G	concordant	unreportable	unreportable	292587	0		1	SCV000348282	Uncertain significance	Illumina Clinical Se	10/18/2016	CTSK	chr1	150778652	150778652	http://gnomad.b	0.0003462893307	ASJ	0.0036607699	0		0	0	https://www.ncbi.nlm.nih.gov/clinvar/variation/292587/
NM_002485.4(NBN):c.1690G>A	concordant	unreportable	unreportable	138427	0		4	SCV000685731;S	Likely benign;Benign;Benig	Color Genomics, In	2017-10-26;2017-1	NBN	chr8	90965627	90965627	http://gnomad.b	0.001201896263	EAS	0.008812620305	0		2	0	https://www.ncbi.nlm.nih.gov/clinvar/variation/138427/
NM_138694.3(PKHD1):c.11738G>A	concordant	unreportable	unreportable	241840	0		1	SCV000291324	Benign	Invitae	6/10/2016	PKHD1	chr6	51491842	51491842	http://gnomad.b	0.001423345083	AFR	0.0158149004	3		3	0	https://www.ncbi.nlm.nih.gov/clinvar/variation/241840/
NM_000227.3(LAMA3):c.2970C>T	concordant	unreportable	unreportable	326323	0		1	SCV000407973	Uncertain significance	Illumina Clinical Se	10/18/2016	LAMA3	chr18	21496533	21496533	http://gnomad.b	0.004762278054	FIN	0.01116789971	2		8	0	https://www.ncbi.nlm.nih.gov/clinvar/variation/326323/
NM_000057.2(BLM):c.816G>A	concordant	unreportable	unreportable	454166	0		1	SCV000623353	Likely benign	Invitae	10/5/2017	BLM	chr15	91295033	91295033	http://gnomad.b	0.0001094039861	AFR	0.001307529979	0		0	0	https://www.ncbi.nlm.nih.gov/clinvar/variation/454166/
NM_000016.4(ACADM):c.994C>T	concordant	unreportable	unreportable	298072	0		3	SCV000705268;S	Uncertain significance;Likel	EGL Genetic Diagn	2017-06-30;2017-1	ACADM	chr1	76226855	76226855	http://gnomad.b	0.001593725183	ASJ	0.002945360029	0		0	0	https://www.ncbi.nlm.nih.gov/clinvar/variation/298072/
NM_000023.2(SGCA):c.1120C>T	concordant	unreportable	unreportable	167678	0		2	SCV000519932;S	Likely benign;Benign	GeneDx;Invitae	2017-11-28;2017-1	SGCA	chr17	48252754	48252754	http://gnomad.b	0.001690342658	AFR	0.0195109006	1		5	0	https://www.ncbi.nlm.nih.gov/clinvar/variation/167678/
NM_004543.4(NEB):c.571G>C	concordant	unreportable	unreportable	129748	0		2	SCV000519544;S	Benign;Benign	GeneDx;Invitae	2017-11-28;2017-1	NEB	chr2	152580815	152580815	http://gnomad.b	0.01587428619	NFE	0.02545160055	69		63	0	https://www.ncbi.nlm.nih.gov/clinvar/variation/129748/
NM_000391.3(TPP1):c.1494C>T	concordant	unreportable	unreportable	130607	0		3	SCV000153065;S	Likely benign;Uncertain sig	Genetic Services La	2014-06-27;2016-1	TPP1	chr11	6636154	6636154	http://gnomad.b	0.007630063626	FIN	0.01660240069	16		13	0	https://www.ncbi.nlm.nih.gov/clinvar/variation/130607/
NM_033056.3(PCDH15):c.5565C>T	concordant	unreportable	unreportable	46503	0		3	SCV000706730;S	Uncertain significance;Unc	EGL Genetic Diagn	2017-06-30;2016-1	PCDH15	chr10	55581921	55581921	http://gnomad.b	0.001656123026	NFE	0.003046160098	1		1	0	https://www.ncbi.nlm.nih.gov/clinvar/variation/46503/
NM_000057.2(BLM):c.968A>G	concordant	unreportable	unreportable	127518	0		1	SCV000283158	Uncertain significance	Invitae	10/5/2017	BLM	chr15	91298049	91298049	http://gnomad.b	0.0006017219236	NFE	0.0007096789777	0		0	0	https://www.ncbi.nlm.nih.gov/clinvar/variation/127518/
NM_138694.3(PKHD1):c.11130C>T	concordant	unreportable	unreportable	198316	0		1	SCV000231866	Uncertain significance	EGL Genetic Diagn	6/30/2017	PKHD1	chr6	51523794	51523794	http://gnomad.b	0.0003669561543	AFR	0.003399139969	0		0	0	https://www.ncbi.nlm.nih.gov/clinvar/variation/198316/
NM_005562.2(LAMC2):c.880C>T	concordant	unreportable	unreportable	290574	0		2	SCV000345149;S	Benign;Uncertain significar	EGL Genetic Diagn	2017-06-30;2016-1	LAMC2	chr1	183192386	183192386	http://gnomad.b	0.00523822589	ASJ	0.01300549973	6		11	0	https://www.ncbi.nlm.nih.gov/clinvar/variation/290574/
NM_004543.4(NEB):c.8072G>A	concordant	unreportable	unreportable	95139	0		2	SCV000113053;S	Likely benign;Likely benign	EGL Genetic Diagn	2017-06-30;2016-0	NEB	chr2	152499752	152499752	http://gnomad.b	0.0004543623953	AFR	0.004579950124	0		1	0	https://www.ncbi.nlm.nih.gov/clinvar/variation/95139/
NM_017890.4(VPS13B):c.3075G>A	concordant	unreportable	unreportable	212573	0		3	SCV000249404;S	Uncertain significance;Unc	Genetic Services La	2015-09-15;2016-1	VPS13B	chr8	100403925	100403925	http://gnomad.b	0.000629916851	ASJ	0.002134149894	0		3	0	https://www.ncbi.nlm.nih.gov/clinvar/variation/212573/
NM_005562.2(LAMC2):c.1147A>G	concordant	unreportable	unreportable	294002	0		1	SCV000351672	Uncertain significance	Illumina Clinical Se	10/18/2016	LAMC2	chr1	183195913	183195913	http://gnomad.b	0.0006663269706	ASJ	0.003147209994	0		0	0	https://www.ncbi.nlm.nih.gov/clinvar/variation/294002/
NM_004543.4(NEB):c.5567G>A	concordant	unreportable	unreportable	129747	0		4	SCV000614180;S	Likely benign;Benign;Likely	Athena Diagnostics	2017-08-17;2017-1	NEB	chr2	152520258	152520258	http://gnomad.b	0.01090469749	NFE	0.0170169007	23		37	0	https://www.ncbi.nlm.nih.gov/clinvar/variation/129747/
NM_000243.2(MEFV):c.1759+11C>T	concordant	unreportable	unreportable	439888	0		1	SCV000604197	Likely benign	ARUP Laboratories	6/30/2017	MEFV	chr16	3294243	3294243	http://gnomad.b	2.80E-05	AMR	0.000446880993	0		0	0	https://www.ncbi.nlm.nih.gov/clinvar/variation/439888/
NM_000492.3(CFTR):c.1680-871A>G	concordant	unreportable	unreportable	227244	0		1	SCV000270057	Likely benign	Laboratory for Mol	4/26/2016	CFTR	chr7	117229536	117229536		0.0002002865639					1	0	https://www.ncbi.nlm.nih.gov/clinvar/variation/227244/
NM_004646.3(NPHS1):c.1926A>G	concordant	unreportable	unreportable	328865	0		1	SCV000411566	Uncertain significance	Illumina Clinical Se	10/18/2016	NPHS1	chr19	36336274	36336274	http://gnomad.b	0.001438375639	AFR	0.01275509968	4		1	0	https://www.ncbi.nlm.nih.gov/clinvar/variation/328865/
NM_000391.3(TPP1):c.1033A>C	concordant	unreportable	unreportable	305509	0		2	SCV000514961;S	Likely benign;Uncertain sig	GeneDx;Illumina C	2017-11-28;2016-1	TPP1	chr11	6637588	6637588	http://gnomad.b	0.001026854914	SAS	0.01260480005	4		3	0	https://www.ncbi.nlm.nih.gov/clinvar/variation/305509/
NM_000642.2(AGL):c.4164C>G	concordant	unreportable	unreportable	387976	0		1	SCV000530175	Likely benign	GeneDx	11/28/2017	AGL	chr1	100380947	100380947	http://gnomad.b	0.0001367591978	AMR	0.0003582299978	0		0	0	https://www.ncbi.nlm.nih.gov/clinvar/variation/387976/
NM_000152.3(GAA):c.1273C>T	concordant	unreportable	unreportable	499089	0		1	SCV000704405	Uncertain significance	EGL Genetic Diagn	6/30/2017	GAA	chr17	78082574	78082574		1.11E-05					0	0	https://www.ncbi.nlm.nih.gov/clinvar/variation/499089/
NM_005562.2(LAMC2):c.1318A>G	concordant	unreportable	unreportable	283044	0		2	SCV000334855;S	Uncertain significance;Unc	EGL Genetic Diagn	2017-06-30;2016-1	LAMC2	chr1	183196682	183196682	http://gnomad.b	0.003020868913	ASJ	0.01868019998	3		5	0	https://www.ncbi.nlm.nih.gov/clinvar/variation/283044/
NM_017890.4(VPS13B):c.3744A>G	concordant	unreportable	unreportable	130723	0		2	SCV000153489;S	Likely benign;Likely benign	Genetic Services La	2014-06-27;2016-1	VPS13B	chr8	100493904	100493904	http://gnomad.b	0.002945211232	AFR	0.03483200073	15		18	0	https://www.ncbi.nlm.nih.gov/clinvar/variation/130723/
NM_000051.3(ATM):c.3154-4G>A	concordant	unreportable	unreportable	142254	0		3	SCV000537432;S	Likely benign;Likely benign	Color Genomics, In	2017-01-26;2017-1	ATM	chr11	108143445	108143445	http://gnomad.b	0.000348088576	NFE	0.0003048669896	0		0	0	https://www.ncbi.nlm.nih.gov/clinvar/variation/142254/
NM_000466.2(PEX1):c.3031G>A	concordant	unreportable	unreportable	288256	0		3	SCV000342312;S	Uncertain significance;Unc	EGL Genetic Diagn	2017-06-30;2017-1	PEX1	chr7	92122443	92122443	http://gnomad.b	0.001331255096	ASJ	0.008448380046	3		2	0	https://www.ncbi.nlm.nih.gov/clinvar/variation/288256/
NM_000057.2(BLM):c.2119C>T	concordant	unreportable	unreportable	127482	0		2	SCV000337707;S	Likely benign;Benign	EGL Genetic Diagn	2017-06-30;2017-1	BLM	chr15	91308570	91308570	http://gnomad.b	0.00175759882	NFE	0.002462350065	1		1	0	https://www.ncbi.nlm.nih.gov/clinvar/variation/127482/
NM_138694.3(PKHD1):c.5100C>T	concordant	unreportable	unreportable	196804	0		1	SCV000229577	Likely benign	EGL Genetic Diagn	6/30/2017	PKHD1	chr6	51889508	51889508	http://gnomad.b	0.0004420153677	AFR	0.006012289785	1		1	0	https://www.ncbi.nlm.nih.gov/clinvar/variation/196804/
NM_000528.3(MAN2B1):c.743C>T	concordant	unreportable	unreportable	197684	0		2	SCV000230942;S	Likely benign;Likely benign	EGL Genetic Diagn	2017-06-30;2017-1	MAN2B1	chr19	12774537	12774537	http://gnomad.b	0.002371857711	NFE	0.003599090036	3		0	0	https://www.ncbi.nlm.nih.gov/clinvar/variation/197684/
NM_003060.3(SLC22A5):c.1341C>T	concordant	unreportable	unreportable	460396	0		1	SCV000632522	Likely benign	Invitae	10/5/2017	SLC22A5	chr5	131728198	131728198	http://gnomad.b	0.0001242642989	AFR	0.001894930028	1		0	0	https://www.ncbi.nlm.nih.gov/clinvar/variation/460396/
NM_000466.2(PEX1):c.1579A>G	concordant	unreportable	unreportable	493456	0		1	SCV000703814	Likely benign	EGL Genetic Diagn	6/30/2017	PEX1	chr7	92140266	92140266	http://gnomad.b	0.002559892612	NFE	0.003509340109	0		1	0	https://www.ncbi.nlm.nih.gov/clinvar/variation/493456/
NM_001360.2(DHCR7):c.199G>A	concordant	unreportable	unreportable	93714	0		1	SCV000111531	Uncertain significance	EGL Genetic Diagn	6/30/2017	DHCR7	chr11	71155161	71155161	http://gnomad.b	0.0009316301913	AMR	0.001146720024	0		0	0	https://www.ncbi.nlm.nih.gov/clinvar/variation/93714/
NM_003060.3(SLC22A5):c.522C>T	concordant	unreportable	unreportable	350812	0		1	SCV000452729	Uncertain significance	Illumina Clinical Se	10/18/2016	SLC22A5	chr5	131719863	131719863	http://gnomad.b	4.80E-05	NFE	0.0001074250031	0		0	0	https://www.ncbi.nlm.nih.gov/clinvar/variation/350812/
NM_018941.3(CLN8):c.53A>T	concordant	unreportable	unreportable	205202	0		1	SCV000240712	Uncertain significance	GeneDx	11/28/2017	CLN8	chr8	1719273	1719273	http://gnomad.b	0.0001902843071	NFE	0.0003669889993	0		0	0	https://www.ncbi.nlm.nih.gov/clinvar/variation/205202/
NM_024685.3(BBS10):c.1545T>C	concordant	unreportable	unreportable	261754	0		3	SCV000331801;S	Benign;Likely benign;Benig	EGL Genetic Diagn	2017-06-30;2016-1	BBS10	chr12	76740220	76740220	http://gnomad.b	0.003721112417	EAS	0.03241330013	13		32	0	https://www.ncbi.nlm.nih.gov/clinvar/variation/261754/
NM_000543.4(SMPD1):c.1763C>T	concordant	unreportable	unreportable	235264	0		2	SCV000342072;S	Benign;Likely benign	EGL Genetic Diagn	2017-06-30;2016-1	SMPD1	chr11	6415704	6415704	http://gnomad.b	0.003748810657	AFR	0.04954250157	20		34	0	https://www.ncbi.nlm.nih.gov/clinvar/variation/235264/
NM_000112.3(SLC26A2):c.655A>G	concordant	unreportable	unreportable	352021	0		1	SCV000454761	Likely benign	Illumina Clinical Se	10/18/2016	SLC26A2	chr5	149357870	149357870	http://gnomad.b	0.002099235577	AFR	0.02600969933	10		13	0	https://www.ncbi.nlm.nih.gov/clinvar/variation/352021/
NM_000277.1(PAH):c.963C>T	concordant	unreportable	unreportable	102911	0		1	SCV000601720	Benign	Quest Diagnostics	8/1/2017	PAH	chr12	103240679	103240679	http://gnomad.b	0.004331340716	AFR	0.05320959911	27		42	0	https://www.ncbi.nlm.nih.gov/clinvar/variation/102911/
NM_138694.3(PKHD1):c.7298A>T	concordant	unreportable	unreportable	167483	0		2	SCV000291339;S	Benign;Likely benign	Invitae;Laboratory	2017-10-05;2018-0	PKHD1	chr6	51747943	51747943	http://gnomad.b	0.002971788856	AFR	0.03565819934	11		18	0	https://www.ncbi.nlm.nih.gov/clinvar/variation/167483/
NM_000051.3(ATM):c.1176C>G	concordant	unreportable	unreportable	142140	0		3	SCV000681955;S	Benign;Likely benign;Benig	Color Genomics, In	2017-10-26;2016-1	ATM	chr11	108119770	108119770	http://gnomad.b	0.004132507574	AFR	0.05208469927	25		35	0	https://www.ncbi.nlm.nih.gov/clinvar/variation/142140/
NM_002485.4(NBN):c.426T>C	concordant	unreportable	unreportable	184528	0		3	SCV000685783;S	Likely benign;Likely benign	Color Genomics, In	2017-10-26;2017-1	NBN	chr8	90993016	90993016	http://gnomad.b	5.30E-05	NFE	0.0001254530071	0		0	0	https://www.ncbi.nlm.nih.gov/clinvar/variation/184528/
NM_133647.1(SLC12A6):c.408T>C	concordant	unreportable	unreportable	315623	0		1	SCV000390326	Uncertain significance	Illumina Clinical Se	10/18/2016	SLC12A6	chr15	34553130	34553130	http://gnomad.b	0.008199587502	FIN	0.0271773003	24		20	0	https://www.ncbi.nlm.nih.gov/clinvar/variation/315623/
NM_000030.2(AGXT):c.489G>A	concordant	unreportable	unreportable	204040	0		1	SCV000429362	Uncertain significance	Illumina Clinical Se	10/18/2016	AGXT	chr2	241810831	241810831	http://gnomad.b	0.0002894969319	AFR	0.0003110899997	0		0	0	https://www.ncbi.nlm.nih.gov/clinvar/variation/204040/

Supplemental Table 2

NM_000391.3(TPP1):c.1241A>T	concordant	unreportable	unreportable	193583	0		3	SCV000243434;S(	Uncertain significance;Unc	GeneDx;Illumina C	2017-11-28;2016-1	TPP1	chr11	6636698	6636698	http://gnomad.b	0.0005176244117	NFE	0.0005371149746	0		0	0	https://www.ncbi.nlm.nih.gov/clinvar/variation/193583/
NM_000303.2(PMM2):c.713G>A	concordant	unreportable	unreportable	198714	0		2	SCV000232517;S(	Uncertain significance;Unc	EGL Genetic Diagn	2017-06-30;2017-1	PMM2	chr16	8941654	8941654	http://gnomad.b	0.001049607163	ASJ	0.004873100203	0		1	0	https://www.ncbi.nlm.nih.gov/clinvar/variation/198714/
NM_000642.2(AGL):c.1875G>T	concordant	unreportable	unreportable	382454	0		2	SCV000522400;S(	Likely benign;Likely benign	GeneDx;Invitae	2017-11-28;2017-1	AGL	chr1	100346327	100346327	http://gnomad.b	0.001962075837	ASJ	0.005687590223	0		1	0	https://www.ncbi.nlm.nih.gov/clinvar/variation/382454/
NM_001360.2(DHCR7):c.399C>T	concordant	unreportable	unreportable	197726	0		2	SCV000231007;S(	Likely benign;Uncertain sig	EGL Genetic Diagn	2017-06-30;2016-1	DHCR7	chr11	71153322	71153322	http://gnomad.b	0.002835279166	NFE	0.004777099937	3		4	0	https://www.ncbi.nlm.nih.gov/clinvar/variation/197726/
NM_000478.4(ALPL):c.818C>T	concordant	unreportable	unreportable	288247	0		3	SCV000342299;S(	Uncertain significance;Unc	EGL Genetic Diagn	2017-06-30;2017-1	ALPL	chr1	21896823	21896823	http://gnomad.b	0.003805976877	ASJ	0.03462630138	5		27	0	https://www.ncbi.nlm.nih.gov/clinvar/variation/288247/
NM_000051.3(ATM):c.5488A>G	concordant	unreportable	unreportable	141271	0		3	SCV000292471;S(	Uncertain significance;Unc	GeneDx;Invitae;Lal	2017-11-28;2017-1	ATM	chr11	108173748	108173748	http://gnomad.b	3.62E-05	EAS	5.80E-05	0		0	0	https://www.ncbi.nlm.nih.gov/clinvar/variation/141271/
NM_001164277.1(SLC37A4):c.1275C>	concordant	unreportable	unreportable	139193	0		1	SCV000631379	Benign	Invitae	10/5/2017	SLC37A4	chr11	118895635	118895635	http://gnomad.b	0.005081715323	AFR	0.03557829931	26		38	0	https://www.ncbi.nlm.nih.gov/clinvar/variation/139193/
NM_000152.3(GAA):c.1552-13G>A	concordant	unreportable	unreportable	255353	0		2	SCV000338203;S(	Likely benign;Uncertain sig	EGL Genetic Diagn	2017-06-30;2016-1	GAA	chr17	78084727	78084727	http://gnomad.b	0.002957182006	NFE	0.004504750017	2		2	0	https://www.ncbi.nlm.nih.gov/clinvar/variation/255353/
NM_000441.1(SLC26A4):c.2190G>T	concordant	unreportable	unreportable	43543	0		1	SCV000334674	Likely benign	EGL Genetic Diagn	6/30/2017	SLC26A4	chr7	107350599	107350599	http://gnomad.b	0.0003294635329	AFR	0.003724520095	1		0	0	https://www.ncbi.nlm.nih.gov/clinvar/variation/43543/
NM_000543.4(SMPD1):c.807C>T	concordant	unreportable	unreportable	286248	0		2	SCV000339598;S(	Benign;Likely benign	EGL Genetic Diagn	2017-06-30;2016-1	SMPD1	chr11	6413102	6413102	http://gnomad.b	0.004293529971	AFR	0.05689249933	24		44	0	https://www.ncbi.nlm.nih.gov/clinvar/variation/286248/
NM_005562.2(LAMC2):c.3158T>A	concordant	unreportable	unreportable	294015	0		1	SCV000351690	Uncertain significance	Illumina Clinical Se	10/18/2016	LAMC2	chr1	183209263	183209263	http://gnomad.b	0.0002295748386	SAS	0.00224171998	2		0	0	https://www.ncbi.nlm.nih.gov/clinvar/variation/294015/
NM_000228.2(LAMB3):c.2542C>T	concordant	unreportable	unreportable	295087	0		1	SCV000353610	Likely benign	Illumina Clinical Se	10/18/2016	LAMB3	chr1	209796341	209796341	http://gnomad.b	0.0031074854	AFR	0.04173250124	8		24	0	https://www.ncbi.nlm.nih.gov/clinvar/variation/295087/
NM_033056.3(PCDH15):c.3018G>T	concordant	unreportable	unreportable	46461	0		2	SCV000203208;S(	Benign;Uncertain significar	EGL Genetic Diagn	2017-06-30;2016-1	PCDH15	chr10	55719596	55719596	http://gnomad.b	0.002959934167	SAS	0.01572450064	9		8	0	https://www.ncbi.nlm.nih.gov/clinvar/variation/46461/
NM_017890.4(VPS13B):c.10204C>T	concordant	unreportable	unreportable	437244	0		1	SCV000597896	Uncertain significance	Genetic Services La	7/5/2017	VPS13B	chr8	100865746	100865746	http://gnomad.b	3.92E-05	SAS	0.0004223519936	1		0	0	https://www.ncbi.nlm.nih.gov/clinvar/variation/437244/
NM_012203.1(GRHPR):c.963G>A	concordant	unreportable	unreportable	204229	0		1	SCV000480144	Uncertain significance	Illumina Clinical Se	10/18/2016	GRHPR	chr9	37436755	37436755	http://gnomad.b	0.007326774126	AFR	0.02567959949	21		18	0	https://www.ncbi.nlm.nih.gov/clinvar/variation/204229/
NM_000642.2(AGL):c.2771A>C	concordant	unreportable	unreportable	291338	0		2	SCV000346077;S(	Uncertain significance;Unc	Illumina Clinical Se	2016-10-18;2017-1	AGL	chr1	100353623	100353623	http://gnomad.b	9.77E-05	NFE	0.0001075079999	0		0	0	https://www.ncbi.nlm.nih.gov/clinvar/variation/291338/
NM_000018.3(ACADVL):c.205-7T>C	concordant	unreportable	unreportable	324985	0		1	SCV000406310	Uncertain significance	Illumina Clinical Se	10/18/2016	ACADVL	chr17	7123916	7123916	http://gnomad.b	3.68E-05	ASJ	0.003756349906	0		0	0	https://www.ncbi.nlm.nih.gov/clinvar/variation/324985/
NM_000642.2(AGL):c.2522C>T	concordant	unreportable	unreportable	256727	0		2	SCV000521968;S(	Likely benign;Benign	GeneDx;Invitae	2017-11-28;2017-1	AGL	chr1	100349983	100349983	http://gnomad.b	0.004284191195	ASJ	0.01560910046	3		6	0	https://www.ncbi.nlm.nih.gov/clinvar/variation/256727/
NM_017890.4(VPS13B):c.5576C>T	concordant	unreportable	unreportable	95857	0		2	SCV000470813;S(	Likely benign;Benign	Illumina Clinical Se	2016-10-18;2017-1	VPS13B	chr8	100654319	100654319	http://gnomad.b	0.002900417145	AMR	0.06132499874	83		21	0	https://www.ncbi.nlm.nih.gov/clinvar/variation/95857/
NM_000466.2(PEX1):c.1725G>A	concordant	unreportable	unreportable	497965	0		1	SCV000702745	Uncertain significance	EGL Genetic Diagn	6/30/2017	PEX1	chr7	92136386	92136386	http://gnomad.b	0.00042711101	ASJ	0.003351609921	0		0	0	https://www.ncbi.nlm.nih.gov/clinvar/variation/497965/
NM_000543.4(SMPD1):c.559C>T	concordant	unreportable	unreportable	195087	0		1	SCV000372926	Likely benign	Illumina Clinical Se	10/18/2016	SMPD1	chr11	6412854	6412854		0.004769267364					29	0	https://www.ncbi.nlm.nih.gov/clinvar/variation/195087/
NM_004543.4(NEB):c.2343G>A	concordant	unreportable	unreportable	195882	0		1	SCV000417032	Uncertain significance	Illumina Clinical Se	10/18/2016	NEB	chr2	152544878	152544878	http://gnomad.b	0.0009448672539	EAS	0.009401110001	2		1	0	https://www.ncbi.nlm.nih.gov/clinvar/variation/195882/
NM_005562.2(LAMC2):c.344G>A	concordant	unreportable	unreportable	293989	0		1	SCV000351655	Likely benign	Illumina Clinical Se	10/18/2016	LAMC2	chr1	183184663	183184663	http://gnomad.b	0.007354793915	FIN	0.04135270044	31		15	0	https://www.ncbi.nlm.nih.gov/clinvar/variation/293989/
NM_000159.2(GCDH):c.1143C>T	concordant	unreportable	unreportable	382438	0		3	SCV000706049;S(	Uncertain significance;Likel	EGL Genetic Diagn	2017-06-30;2017-1	GCDH	chr19	13008577	13008577	http://gnomad.b	0.001644625869	ASJ	0.002437039977	1		0	0	https://www.ncbi.nlm.nih.gov/clinvar/variation/382438/
NM_014363.4(SACS):c.1885G>A	concordant	unreportable	unreportable	448197	0		1	SCV000614943	Uncertain significance	Athena Diagnostics	8/17/2017	SACS	chr13	23928866	23928866	http://gnomad.b	0.0004801764204	NFE	0.000836029998	2		0	0	https://www.ncbi.nlm.nih.gov/clinvar/variation/448197/
NM_024685.3(BBS10):c.1631A>G	concordant	unreportable	unreportable	166720	0		2	SCV000261859;S(	Benign;Likely benign	Invitae;Laboratory	2017-10-05;2018-0	BBS10	chr12	76740134	76740134	http://gnomad.b	0.009112528678	SAS	0.01566259935	13		30	0	https://www.ncbi.nlm.nih.gov/clinvar/variation/166720/
NM_000228.2(LAMB3):c.67C>T	concordant	unreportable	unreportable	295151	0		1	SCV000353682	Uncertain significance	Illumina Clinical Se	10/18/2016	LAMB3	chr1	209823425	209823425	http://gnomad.b	0.0006998840992	AMR	0.001787310001	0		1	0	https://www.ncbi.nlm.nih.gov/clinvar/variation/295151/
NM_138694.3(PKHD1):c.12110T>C	concordant	unreportable	unreportable	216821	0		1	SCV000255209	Uncertain significance	Invitae	6/10/2016	PKHD1	chr6	51483994	51483994	http://gnomad.b	0.0001278786598	NFE	0.0002967840119	0		0	0	https://www.ncbi.nlm.nih.gov/clinvar/variation/216821/
NM_002485.4(NBN):c.1489A>G	concordant	unreportable	unreportable	134872	0		4	SCV000604436;S(	Benign;Likely benign;Benig	ARUP Laboratories	2017-06-30;2017-0	NBN	chr8	90965828	90965828	http://gnomad.b	0.001882320134	AFR	0.02473499998	7		11	0	https://www.ncbi.nlm.nih.gov/clinvar/variation/134872/
NM_002435.1(MPI):c.982C>T	concordant	unreportable	unreportable	94072	0		3	SCV000522331;S(	Likely benign;Uncertain sig	GeneDx;Illumina C	2017-11-28;2016-1	MPI	chr15	75189489	75189489	http://gnomad.b	0.005869937634	NFE	0.0108489003	6		11	0	https://www.ncbi.nlm.nih.gov/clinvar/variation/94072/
NM_000018.3(ACADVL):c.1284G>A	concordant	unreportable	unreportable	21014	0		1	SCV000523265	Likely benign	GeneDx	11/28/2017	ACADVL	chr17	7127146	7127146	http://gnomad.b	0.0007645692926	NFE	0.001208400005	0		1	0	https://www.ncbi.nlm.nih.gov/clinvar/variation/21014/
NM_000243.2(MEFV):c.1173T>C	concordant	unreportable	unreportable	319114	0		1	SCV000396772	Uncertain significance	Illumina Clinical Se	10/18/2016	MEFV	chr16	3299518	3299518	http://gnomad.b	0.0001147495102	FIN	0.02175280079	8		0	0	https://www.ncbi.nlm.nih.gov/clinvar/variation/319114/
NM_004646.3(NPHS1):c.1110T>C	concordant	unreportable	unreportable	328872	0		1	SCV000411578	Uncertain significance	Illumina Clinical Se	10/18/2016	NPHS1	chr19	36339599	36339599		0.00143001299					1	0	https://www.ncbi.nlm.nih.gov/clinvar/variation/328872/
NM_000391.3(TPP1):c.1044C>T	concordant	unreportable	unreportable	130605	0		2	SCV000153063;S(	Likely benign;Benign	Genetic Services La	2014-06-27;2017-1	TPP1	chr11	6637577	6637577	http://gnomad.b	0.01021538771	NFE	0.01390330028	14		14	0	https://www.ncbi.nlm.nih.gov/clinvar/variation/130605/
NM_033056.3(PCDH15):c.4850A>G	concordant	unreportable	unreportable	46484	0		2	SCV000297035;S(	Likely benign;Uncertain sig	Division of Genomi	2016-01-06;2016-1	PCDH15	chr10	55582636	55582636	http://gnomad.b	0.005662706372	NFE	0.009036360309	4		7	0	https://www.ncbi.nlm.nih.gov/clinvar/variation/46484/
NM_000152.3(GAA):c.862G>A	concordant	unreportable	unreportable	456441	0		1	SCV000626650	Uncertain significance	Invitae	10/5/2017	GAA	chr17	78081602	78081602	http://gnomad.b	4.45E-05	EAS	0.0005273640272	0		0	0	https://www.ncbi.nlm.nih.gov/clinvar/variation/456441/
NM_138694.3(PKHD1):c.2255C>T	concordant	unreportable	unreportable	498544	0		1	SCV000703608	Uncertain significance	EGL Genetic Diagn	6/30/2017	PKHD1	chr6	51914979	51914979	http://gnomad.b	0.0002613172614	AMR	0.0003574189905	0		0	0	https://www.ncbi.nlm.nih.gov/clinvar/variation/498544/
NM_003640.3(IKBKAP):c.2825G>A	concordant	unreportable	unreportable	242293	0		2	SCV000291942;S(	Uncertain significance;Unc	GeneDx;Invitae	2017-11-28;2017-1	IKBKAP	chr9	111656258	111656258	http://gnomad.b	0.0005630444143	NFE	0.0008884820272	1		0	0	https://www.ncbi.nlm.nih.gov/clinvar/variation/242293/
NM_001173990.2(TMEM216):c.5T>C	concordant	unreportable	unreportable	193189	0		1	SCV000372625	Uncertain significance	Illumina Clinical Se	10/18/2016	TMEM216	chr11	61160108	61160108	http://gnomad.b	0.0007610310943	SAS	0.008113860153	3		2	0	https://www.ncbi.nlm.nih.gov/clinvar/variation/193189/
NM_000383.3(AIRE):c.1404G>A	concordant	unreportable	unreportable	35661	0		1	SCV000629947	Benign	Invitae	10/5/2017	AIRE	chr21	45714287	45714287	http://gnomad.b	0.003002333911	AFR	0.01434590016	5		4	0	https://www.ncbi.nlm.nih.gov/clinvar/variation/35661/
NM_001876.3(CPT1A):c.1461C>T	concordant	unreportable	unreportable	389615	0		1	SCV000532207	Likely benign	GeneDx	11/28/2017	CPT1A	chr11	68542898	68542898	http://gnomad.b	6.76E-05	SAS	9.75E-05	0		0	0	https://www.ncbi.nlm.nih.gov/clinvar/variation/389615/
NM_000271.4(NPC1):c.3561G>T	concordant	unreportable	unreportable	211683	0		2	SCV000700540;S(	Likely benign;Benign	EGL Genetic Diagn	2017-06-30;2017-1	NPC1	chr18	21114440	21114440	http://gnomad.b	0.00325948969	ASJ	0.009543149732	4		5	0	https://www.ncbi.nlm.nih.gov/clinvar/variation/211683/
NM_000071.2(CBS):c.1287C>T	concordant	unreportable	unreportable	471357	0		1	SCV000649826	Likely benign	Invitae	10/5/2017	CBS	chr21	44479015	44479015	http://gnomad.b	6.47E-05	AMR	0.0006796040107	0		0	0	https://www.ncbi.nlm.nih.gov/clinvar/variation/471357/
NM_033056.3(PCDH15):c.5254_5256c	concordant	unreportable	unreportable	46488	0		2	SCV000570036;S(	Likely benign;Uncertain sig	GeneDx;Illumina C	2017-11-28;2016-1	PCDH15	chr10	55582229	55582232		0.001517255568					3	0	https://www.ncbi.nlm.nih.gov/clinvar/variation/46488/
NM_000057.2(BLM):c.1722A>G	concordant	unreportable	unreportable	136511	0		1	SCV000283116	Benign	Invitae	10/5/2017	BLM	chr15	91304325	91304325	http://gnomad.b	0.006354944585	SAS	0.02595910057	16		27	0	https://www.ncbi.nlm.nih.gov/clinvar/variation/136511/
NM_000035.3(ALDOB):c.-1C>T	concordant	unreportable	unreportable	364317	0		1	SCV000476071	Uncertain significance	Illumina Clinical Se	10/18/2016	ALDOB	chr9	104193170	104193170	http://gnomad.b	0.0009336251735	SAS	0.01104899962	2		1	0	https://www.ncbi.nlm.nih.gov/clinvar/variation/364317/
NM_000152.3(GAA):c.915G>A	concordant	unreportable	unreportable	188478	0		3	SCV000330943;S(	Uncertain significance;Likel	EGL Genetic Diagn	2017-06-30;2017-1	GAA	chr17	78081655	78081655	http://gnomad.b	0.001517501292	NFE	0.001823149971	0		3	0	https://www.ncbi.nlm.nih.gov/clinvar/variation/188478/
NM_001876.3(CPT1A):c.518G>A	concordant	unreportable	unreportable	381410	0		1	SCV000520649	Benign	GeneDx	11/28/2017	CPT1A	chr11	68571505	68571505	http://gnomad.b	0.001636112754	SAS	0.0147508001	6		11	0	https://www.ncbi.nlm.nih.gov/clinvar/variation/381410/
NM_000136.2(FANCC):c.178G>A	concordant	unreportable	unreportable	127537	0		2	SCV000149256;S(	Likely benign;Uncertain sig	GeneDx;Invitae	2017-11-28;2017-1	FANCC	chr9	98009786	98009786	http://gnomad.b	0.001262776396	NFE	0.001429780037	0		0	0	https://www.ncbi.nlm.nih.gov/clinvar/variation/127537/
NM_000051.3(ATM):c.5529A>G	concordant	unreportable	unreportable	233190	0		1	SCV000558418	Likely benign	Invitae	10/5/2017	ATM	chr11	108175434	108175434		2.78E-06					0	0	https://www.ncbi.nlm.nih.gov/clinvar/variation/233190/
NM_000098.2(CPT2):c.588T>C	concordant	unreportable	unreportable	297603	0		3	SCV000512763;S(	Likely benign;Uncertain sig	GeneDx;Illumina C	2017-11-28;2016-1	CPT2	chr1	53675934	53675934	http://gnomad.b	0.0009877275551	EAS	0.009392390028	0		7	0	https://www.ncbi.nlm.nih.gov/clinvar/variation/297603/
NM_000057.2(BLM):c.43C>T	concordant	unreportable	unreportable	236821	0		1	SCV000283150	Likely benign	Invitae	10/5/2017	BLM	chr15	91290665	91290665	http://gnomad.b	0.0002854017029	AMR	0.0003592600115	0		0	0	https://www.ncbi.nlm.nih.gov/clinvar/variation/236821/
NM_000155.3(GALT):c.508-17G>A	concordant	unreportable	unreportable	36148	0		1	SCV000331123	Benign	EGL Genetic Diagn	6/30/2017	GALT	chr9	34648095	34648095	http://gnomad.b	0.003487854351	AFR	0.04515159875	15		23	0	https://www.ncbi.nlm.nih.gov/clinvar/variation/36148/
NM_000232.4(SGCB):c.799C>T	concordant	unreportable	unreportable	283043	0		4	SCV000615221;S(	Likely benign;Benign;Uncer	Athena Diagnostics	2017-08-17;2017-0	SGCB	chr4	52890281	52890281	http://gnomad.b	0.001374187662	SAS	0.01530210022	11		2	0	https://www.ncbi.nlm.nih.gov/clinvar/variation/283043/
NM_183050.2(BCKDHB):c.122C>T	concordant	unreportable	unreportable	377557	0		1	SCV000512256	Likely benign	GeneDx	7/13/2017	BCKDHB	chr6	80816532	80816532	http://gnomad.b	0.001291685034	AFR	0.01167939976	1		2	0	https://www.ncbi.nlm.nih.gov/clinvar/variation/377557/
NM_000057.2(BLM):c.615G>A	concordant	unreportable	unreportable	136509	0		2	SCV000593632;S(	Likely benign;Benign	Genetic Services La	2017-07-05;2017-1	BLM	chr15	91293113	91293113	http://gnomad.b	0.001407981734	AFR	0.01785250008	2		3	0	https://www.ncbi.nlm.nih.gov/clinvar/variation/136509/
NM_002485.4(NBN):c.286G>A	concordant	unreportable	unreportable	183076	0		1	SCV000211927	Uncertain significance	Invitae	10/5/2017	NBN	chr8	90993637	90993637	http://gnomad.b	1.39E-05	AMR	5.96E-05	0		0	0	https://www.ncbi.nlm.nih.gov/clinvar/variation/183076/
NM_000152.3(GAA):c.664G>A	concordant	unreportable	unreportable	290223	0		1	SCV000344733	Benign	EGL Genetic Diagn	6/30/2017	GAA	chr17	78079665	78079665	http://gnomad.b	3.34E-05	SAS	0.005427009892	4		0	0	https://www.ncbi.nlm.nih.gov/clinvar/variation/290223/
NM_000360.3(TH):c.1278G>A	concordant	unreportable	unreportable	304069	0		2	SCV000483270;S(	Likely benign;Benign	Illumina Clinical Se	2016-10-18;2017-0	TH	chr11	2186518	2186518	http://gnomad.b	0.003560743925	AFR	0.04559170082	15		26	0	https://www.ncbi.nlm.nih.gov/clinvar/variation/304069/
NM_014363.4(SACS):c.9846A>G	concordant	unreportable	unreportable	416834	0		2	SCV000614996;S(	Likely benign;Benign	Athena Diagnostics	2017-08-17;2017-1	SACS	chr13	23908169	23908169	http://gnomad.b	0.002673574884	NFE	0.004764220212	1		7	0	https://www.ncbi.nlm.nih.gov/clinvar/variation/416834/
NM_017890.4(VPS13B):c.1832G>A	concordant	unreportable	unreportable	95835	0		4	SCV000113817;S(	Uncertain significance;Unc	EGL Genetic Diagn	2017-06-30;2017-0	VPS13B	chr8	100155382	100155382	http://gnomad.b	0.001265432963	SAS	0.003118709894	2		1	0	https://www.ncbi.nlm.nih.gov/clinvar/variation/95835/
NM_000182.4(HADHA):c.1981C>T	concordant	unreportable	unreportable	92595	0		3	SCV000429497;S(	Uncertain significance;Beni	Illumina Clinical Se	2016-10-18;2017-1	HADHA	chr2	26415198	26415198	http://gnomad.b	0.004581239907	NFE	0.007125210017	7		6	0	https://www.ncbi.nlm.nih.gov/clinvar/variation/92595/
NM_000071.2(CBS):c.625C>A	concordant	unreportable	unreportable	413349	0		1	SCV000555940	Likely benign	Invitae	3/14/2017	CBS	chr21	44485538	44485538	http://gnomad.b	0.0001434123132	SAS	0.000943335006	0		0	0	https://www.ncbi.nlm.nih.gov/clinvar/variation/413349/
NM_017890.4(VPS13B):c.11007C>T	concordant	unreportable	unreportable	361093	0		1	SCV000470857	Uncertain significance	Illumina Clinical Se	10/18/2016	VPS13B	chr8	100871596	100871596	http://gnomad.b	0.0002603656317	ASJ	0.002436550101	0		0	0	https://www.ncbi.nlm.nih.gov/clinvar/variation/361093/
NM_000051.3(ATM):c.1020C>T	concordant	unreportable	unreportable	236664	0		2	SCV000681946;S(	Likely benign;Likely benign	Color Genomics, In	2017-10-26;2016-0	ATM	chr11	108117809	108117809	http://gnomad.b	8.35E-06	SAS	3.25E-05	0		0	0	https://www.ncbi.nlm.nih.gov/clinvar/variation/236664/
NM_000051.3(ATM):c.4673C>T	concordant	unreportable	unreportable	141389	0		2	SCV000209739;S(	Uncertain significance;Unc	GeneDx;Invitae	2017-11-28;2017-1	ATM	chr11	108164101	108164101	http://gnomad.b	1.95E-05	AFR	0.0002617459977	0		0	0	https://www.ncbi.nlm.nih.gov/clinvar/variation/141389/
NM_001042432.1(CLN3):c.649C>A	concordant	unreportable	unreportable	205112	0		2	SCV000396329;S(	Uncertain significance;Unc	Illumina Clinical Se	2016-10-18;2017-1	CLN3	chr16	28497696	28497696	http://gnomad.b	5.56E-05	NFE	0.0001094249965	0		0	0	https://www.ncbi.nlm.nih.gov/clinvar/variation/205112/
NM_000018.3(ACADVL):c.205-8C>G	concordant	unreportable	unreportable	324984	0		1	SCV000406309	Uncertain significance	Illumina Clinical Se	10/18/2016	ACADVL	chr17	7123915	7123915	http://gnomad.b	3.68E-05	ASJ	0.003756349906	0		0	0	https://www.ncbi.nlm.nih.gov/clinvar/variation/324984/
NM_000016.4(ACADM):c.678A>G	concordant	unreportable	unreportable	226082	0		2	SCV000358940;S(	Uncertain significance;Beni	Illumina Clinical Se	2016-10-18;2017-1	ACADM	chr1	76211569	76211569	http://gnomad.b	0.001550210502	AMR	0.03010720015	20		9	0	https://www.ncbi.nlm.nih.gov/clinvar/variation/226082/
NM_000108.3(DLD):c.675T>C	concordant	unreportable	unreportable	203670	0		1	SCV000700372	Benign	EGL Genetic Diagn	6/30/2017	DLD	chr7	107546804	107546804	http://gnomad.b	0.003437389661	AFR	0.04224979877	18		22	0	https://www.ncbi.nlm.nih.gov/clinvar/variation/203670/
NM_000112.3(SLC26A2):c.987T>C	concordant	unreportable	unreportable	196211	0		3	SCV000228784;S(	Likely benign;Likely benign	EGL Genetic Diagn	2017-06-30;2017-1	SLC26A2	chr5	149360143	149360143	http://gnomad.b	0.003373273637	ASJ	0.009447379969	2		3	0	https://www.ncbi.nlm.nih.gov/clinvar/variation/196211/
NM_001173990.2(TMEM216):c.420T>	concordant	unreportable	unreportable	305081	0		2	SCV000372639;S(	Uncertain significance;Likel	Illumina Clinical Se	2016-10-18;2017-0	TMEM216	chr11	61165436	61165436	http://gnomad.b	0.0003162392723	ASJ	0.002030459931	0		0	0	https://www.ncbi.nlm.nih.gov/clinvar/variation/305081/
NM_000271.4(NPC1):c.3198C>T	concordant	unreportable	unreportable	92709	0		1	SCV000110334	Uncertain significance	EGL Genetic Diagn	6/30/2017	NPC1	chr18	21116684	21116684	http://gnomad.b	0.0006633347439	NFE	0.001620499999	1		0	0	https://www.ncbi.nlm.nih.gov/clinvar/variation/92709/
NM_000153.3(GALC):c.1731C>T	concordant	unreportable	unreportable	314745	0		1	SCV000389237	Uncertain significance	Illumina Clinical Se	10/18/2016	GALC	chr14	88407842	88407842	http://gnomad.b	0.001499233601	SAS	0.01442500018	10		8	0	https://www.ncbi.nlm.nih.gov/clinvar/variation/314745/
NM_000057.2(BLM):c.174T>C	concordant	unreportable	unreportable	136507	0		1	SCV000623236	Likely benign	Invitae	10/5/2017	BLM	chr15	91292672	91292672	http://gnomad.b	3.09E-05	AFR	0.000721785007	0		0	0	https://www.ncbi.nlm.nih.gov/clinvar/variation/136507/
NM_017890.4(VPS13B):c.1248G>T	concordant	unreportable	unreportable	95831	0		1	SCV000597873	Uncertain significance	Genetic Services La	7/5/2017	VPS13B	chr8	100146901	100146901	http://gnomad.b	0.00149780229	ASJ	0.004579689819	0		1	0	https://www.ncbi.nlm.nih.gov/clinvar/variation/95831/
NM_014363.4(SACS):c.2983G>T	concordant	unreportable	unreportable	218707	0		4	SCV000614948;S(	Uncertain significance;Likel	Athena Diagnostics	2017-08-17;2015-0	SACS	chr13	23915032	23915032	http://gnomad.b	0.002214146827	NFE	0.002789889928	4		1	0	https://www.ncbi.nlm.nih.gov/clinvar/variation/218707/
NM_012203.1(GRHPR):c.336C>T	concordant	unreportable	unreportable	366854	0		1	SCV000480133	Uncertain significance	Illumina Clinical Se	10/18/2016	GRHPR	chr9	37426583	37426583	http://gnomad.b	0.000116630282	AFR	0.0001960270019	0		0	0	https://www.ncbi.nlm.nih.gov/clinvar/variation/366854/
NM_000152.3(GAA):c.447G>A	concordant	unreportable	unreportable	129121	0		5	SCV000227030;S(	Benign;Benign;Benign;Unc	EGL Genetic Diagn	2017-06-30;2017-1	GAA	chr17	78078832	78078832	http://gnomad.b	0.005986625978	EAS	0.02966200002	10		18	0	https://www.ncbi.nlm.nih.gov/clinvar/variation/129121/
NM_017890.4(VPS13B):c.3751A>G	concordant	unreportable	unreportable	95849	0		2	SCV000113831;S(	Benign;Uncertain significar	EGL Genetic Diagn	2017-06-30;2016-1	VPS13B	chr8	100493911	100493911	http://gnomad.b	0.001388616703	SAS	0.008869400248	8		8	0	https://www.ncbi.nlm.nih.gov/clinvar/variation/95849/
NM_138694.3(PKHD1):c.7942G>A	concordant	unreportable	unreportable	96427	0		2	SCV000114619;S(	Benign;Benign	EGL Genetic Diagn	2017-06-30;2017-0	PKHD1	chr6	51712738	51712738	http://gnomad.b	0.002552013255	AMR	0.03640589863	20		14	0	https://www.ncbi.nlm.nih.gov/clinvar/variation/96427/
NM_000478.4(ALPL):c.1381G>A	concordant	unreportable	unreportable	295552	0		1	SCV000354314	Likely benign	Illumina Clinical Se	10/18/2016	ALPL	chr1	21903947	21903947	http://gnomad.b	0.00238049627	AFR	0.02812919952	6		14	0	https://www.ncbi.nlm.nih.gov/clinvar/variation/295552/
NM_000051.3(ATM):c.2021A>G	concordant	unreportable	unreportable	141183	0		3	SCV000611347;S(	Uncertain significance;Unc	Fulgent Genetics,F	2017-05-23;2017-1	ATM	chr11	108124663	108124663	http://gnomad.b	0.00021999198	SAS	0.000844648981	0		0	0	https://www.ncbi.nlm.nih.gov/clinvar/variation/141183/
NM_014363.4(SACS):c.12649A>G	concordant	unreportable	unreportable	235607	0		3	SCV000614934;S(	Benign;Uncertain significar	Athena Diagnostics	2017-08-17;2016-1	SACS	chr13	23905366	23905366	http://gnomad.b	0.002223038983	AFR	0.02799949981	7		8	0	https://www.ncbi.nlm.nih.gov/clinvar/variation/235607/
NM_000642.2(AGL):c.1319C>T	concordant	unreportable	unreportable	256721	0		2	SCV000528000;S(	Likely benign;Benign	GeneDx;Invitae	2017-11-28;2017-1	AGL	chr1	100342049	100342049	http://gnomad.b	0.0009991794448	AFR	0.01138589997	1		1	0	https://www.ncbi.nlm.nih.gov/clinvar/variation/256721/
NM_138694.3(PKHD1):c.9478C>T	concordant	unreportable	unreportable	285245	0		2	SCV000338186;S(	Uncertain significance;Unc	EGL Genetic Diagn	2017-06-30;2016-1	PKHD1	chr6	51612936	51612936	http://gnomad.b	0.0008451111432	ASJ	0.007224259898	0		0	0	https://www.ncbi.nlm.nih.gov/clinvar/variation/285245/
NM_033056.3(PCDH15):c.243G>A	concordant	unreportable	unreportable	46452	0		2	SCV000363250;S(	Uncertain significance;Likel	Illumina Clinical Se	2016-10-18;2018-0	PCDH15	chr10	56138617	56138617	http://gnomad.b	0.002203878002	ASJ	0.01533930004	2		3	0	https://www.ncbi.nlm.nih.gov/clinvar/variation/46452/
NM_017890.4(VPS13B):c.3516A>G	concordant	unreportable	unreportable	195833	0		2	SCV000228163;S(	Uncertain significance;Likel	EGL Genetic Diagn	2017-06-30;2017-1	VPS13B	chr8	100479712	100479712	http://gnomad.b	0.0008958817436	NFE	0.001830250025	0		0	0	https://www.ncbi.nlm.nih.gov/clinvar/variation/195833/
NM_000228.2(LAMB3):c.3068G>A	concordant	unreportable	unreportable	295071	0		1	SCV000353589	Uncertain significance	Illumina Clinical Se	10/18/2016	LAMB3	chr1	209790915	209790915	http://gnomad.b	0.0002575573485	AMR	0.0005955569795	0		1	0	https://www.ncbi.nlm.nih.gov/clinvar/variation/295071/
NM_014363.4(SACS):c.973G>A	concordant	unreportable	unreportable	448230	0		1	SCV000614995	Uncertain significance	Athena Diagnostics	8/17/2017	SACS	chr13	23929778	23929778	http://gnomad.b	0.0007410129944	ASJ	0.007106599864	0		1	0	https://www.ncbi.nlm.nih.gov/clinvar/variation/448230/
NM_000492.3(CFTR):c.2559T>C	concordant	unreportable	unreportable	358732	0		3	SCV000702203;S(	Uncertain significance;Unc	EGL Genetic Diagn	2017-06-30;2016-1	CFTR	chr7	117235052	117235052	http://gnomad.b	0.0004660514274	AMR	0.0008638150175	0		0	0	https://www.ncbi.nlm.nih.gov/clinvar/variation/358732/
NM_000051.3(ATM):c.4949A>G	concordant	unreportable	unreportable	127395	0		5	SCV000682231;S(	Benign;Benign;Likely benig	Color Genomics, In	2017-10-26;2017-0	ATM	chr11	108168053	108168053	http://gnomad.b	0.000952370344	EAS	0.00684932014	0		2	0	https://www.ncbi.nlm.nih.gov/clinvar/variation/127395/
NM_000050.4(ASS1):c.240C>T	concordant	unreportable	unreportable	365251	0		1	SCV000477618	Uncertain significance	Illumina Clinical Se	10/18/2016	ASS1	chr9	133333853	133333853	http://gnomad.b	6.68E-05	AFR	0.0004575760104	0		0	0	https://www.ncbi.nlm.nih.gov/clinvar/variation/365251/
NM_004543.4(NEB):c.5555T>G	concordant	unreportable	unreportable	197524	0		5	SCV000230687;S(	Likely benign;Likely benign	EGL Genetic Diagn	2017-06-30;2017-1	NEB	chr2	152520270	152520270	http://gnomad.b	0.004303534733	NFE	0.00548553979	4		2	0	https://www.ncbi.nlm.nih.gov/clinvar/variation/197524/
NM_000288.3(PEX7):c.377A>C	concordant	unreportable	unreportable	255746	0		4	SCV000604618;S(	Benign;Likely benign;Benig	ARUP Laboratories	2017-06-30;2017-0	PEX7	chr6	137166790	137166790	http://gnomad.b	0.00371956843	FIN	0.01228919998	3		4	0	https://www.ncbi.nlm.nih.gov/clinvar/variation/255746/
NM_000414.3(HSD17B4):c.1471G>A	concordant	unreportable	unreportable	194706	0		2	SCV000226571;S(	Likely benign;Uncertain sig	EGL Genetic Diagn	2017-06-30;2016-1	HSD17B4	chr5	118850709	118850709	http://gnomad.b	0.004189464075	NFE	0.005531250034	3		9	0	https://www.ncbi.nlm.nih.gov/clinvar/variation/194706/
NM_002485.4(NBN):c.2146A>G	concordant	unreportable	unreportable	134874	0		5	SCV000595913;S(	Benign;Uncertain significar	Genetic Services La	2017-07-05;2016-1	NBN	chr8	90955519	90955519	http://gnomad.b	0.002013385388	AFR	0.02666670084	8		8	0	https://www.ncbi.nlm.nih.gov/clinvar/variation/134874/

Supplemental Table 2

NM_002225.3(IVD):c.238C>T	concordant	unreportable	unreportable	382411	0	2	SCV000522341;S	Likely benign;Benign	GeneDx;Invitae	2017-11-28;2017-1	IVD	chr15	40699921	40699921	http://gnomad.b	0.0007958169173	SAS	0.004710549954	2		1	0	https://www.ncbi.nlm.nih.gov/clinvar/variation/382411/
NM_004543.4(NEB):c.2823G>A	concordant	unreportable	unreportable	331524	0	1	SCV000417027	Uncertain significance	Illumina Clinical Se	10/18/2016	NEB	chr2	152541304	152541304	http://gnomad.b	5.94E-05	AFR	0.0005314910086	0		0	0	https://www.ncbi.nlm.nih.gov/clinvar/variation/331524/
NM_000642.2(AGL):c.3203A>G	concordant	unreportable	unreportable	281333	0	2	SCV000332077;S	Benign;Benign	EGL Genetic Diagn	2017-06-30;2017-1	AGL	chr1	100358107	100358107	http://gnomad.b	0.0005274997628	AFR	0.005493069999	1		0	0	https://www.ncbi.nlm.nih.gov/clinvar/variation/281333/
NM_017890.4(VPS13B):c.1639A>G	concordant	unreportable	unreportable	374535	0	1	SCV000597914	Uncertain significance	Genetic Services La	7/5/2017	VPS13B	chr8	100148968	100148968	http://gnomad.b	0.000411545676	ASJ	0.0009140769835	1		1	0	https://www.ncbi.nlm.nih.gov/clinvar/variation/374535/
NM_000182.4(HADHA):c.1843A>G	concordant	unreportable	unreportable	449769	0	1	SCV000618157	Uncertain significance	GeneDx	11/28/2017	HADHA	chr2	26416488	26416488	http://gnomad.b	0.000905593935	AFR	0.01123890001	3		2	0	https://www.ncbi.nlm.nih.gov/clinvar/variation/449769/
NM_033056.3(PCDH15):c.5398G>A	concordant	unreportable	unreportable	46499	0	2	SCV000297036;S	Likely benign;Benign	Division of Genomi	2016-01-06;2016-0	PCDH15	chr10	55582088	55582088	http://gnomad.b	0.001272951705	SAS	0.006345179863	3		0	0	https://www.ncbi.nlm.nih.gov/clinvar/variation/46499/
NM_033056.3(PCDH15):c.3450C>A	concordant	unreportable	unreportable	300182	0	1	SCV000363164	Uncertain significance	Illumina Clinical Se	10/18/2016	PCDH15	chr10	55663054	55663054	http://gnomad.b	0.0009489276346	ASJ	0.003250709968	0		1	0	https://www.ncbi.nlm.nih.gov/clinvar/variation/300182/
NM_000018.3(ACADVL):c.1581G>A	concordant	unreportable	unreportable	324995	0	1	SCV000406327	Uncertain significance	Illumina Clinical Se	10/18/2016	ACADVL	chr17	7127688	7127688	http://gnomad.b	0.0005238715524	ASJ	0.002439520089	0		1	0	https://www.ncbi.nlm.nih.gov/clinvar/variation/324995/
NM_000152.3(GAA):c.687C>T	concordant	unreportable	unreportable	456428	0	1	SCV000626625	Likely benign	Invitae	10/5/2017	GAA	chr17	78079688	78079688	http://gnomad.b	4.45E-05	ASJ	0.0003110739926	0		0	0	https://www.ncbi.nlm.nih.gov/clinvar/variation/456428/
NM_000098.2(CPT2):c.577C>T	concordant	unreportable	unreportable	287483	0	1	SCV000341267	Likely benign	EGL Genetic Diagn	6/30/2017	CPT2	chr1	53675923	53675923	http://gnomad.b	0.0004197142585	EAS	0.001797519973	2		1	0	https://www.ncbi.nlm.nih.gov/clinvar/variation/287483/
NM_000243.2(MEFV):c.2163C>T	concordant	unreportable	unreportable	458000	0	2	SCV000629037;S	Benign;Likely benign	Invitae;Laboratory	2017-10-05;2018-0	MEFV	chr16	3293324	3293324	http://gnomad.b	0.001684858662	AFR	0.01875329949	2		7	0	https://www.ncbi.nlm.nih.gov/clinvar/variation/458000/
NM_005562.2(LAMC2):c.2035C>T	concordant	unreportable	unreportable	259783	0	1	SCV000351678	Likely benign	Illumina Clinical Se	10/18/2016	LAMC2	chr1	183201807	183201807	http://gnomad.b	0.003636801407	AFR	0.04215139896	10		19	0	https://www.ncbi.nlm.nih.gov/clinvar/variation/259783/
NM_000352.3(ABCC8):c.105G>A	concordant	unreportable	unreportable	303789	0	1	SCV000369425	Uncertain significance	Illumina Clinical Se	10/18/2016	ABCC8	chr11	17498219	17498219	http://gnomad.b	0.00023229763	NFE	0.0005004450213	0		0	0	https://www.ncbi.nlm.nih.gov/clinvar/variation/303789/
NM_000057.2(BLM):c.419A>G	concordant	unreportable	unreportable	133708	0	3	SCV000394409;S	Likely benign;Benign;Benig	Illumina Clinical Se	2016-10-18;2017-1	BLM	chr15	91292917	91292917	http://gnomad.b	0.002651857489	AFR	0.03255749866	8		17	0	https://www.ncbi.nlm.nih.gov/clinvar/variation/133708/
NM_000271.4(NPC1):c.1300C>T	concordant	unreportable	unreportable	92700	0	3	SCV000513948;S	Likely benign;Likely benign	GeneDx;Illumina C	2017-11-28;2016-1	NPC1	chr18	21136233	21136233	http://gnomad.b	0.003233756876	AFR	0.04652990028	16		22	0	https://www.ncbi.nlm.nih.gov/clinvar/variation/92700/
NM_001079802.1(FKTN):c.681G>A	concordant	unreportable	unreportable	264299	0	4	SCV000320135;S	Likely benign;Uncertain sig	Ambry Genetics;EG	2017-11-14;2017-0	FKTN	chr9	108370133	108370133	http://gnomad.b	0.0003049407493	AFR	0.003202199936	0		0	0	https://www.ncbi.nlm.nih.gov/clinvar/variation/264299/
NM_000152.3(GAA):c.257C>G	concordant	unreportable	unreportable	325776	0	2	SCV000702184;S	Benign;Uncertain significar	EGL Genetic Diagn	2017-06-30;2016-1	GAA	chr17	78078642	78078642	http://gnomad.b	0.0005780957304	EAS	0.007564300206	0		0	0	https://www.ncbi.nlm.nih.gov/clinvar/variation/325776/
NM_000053.3(ATP7B):c.3588C>T	concordant	unreportable	unreportable	35723	0	1	SCV000532827	Likely benign	GeneDx	11/28/2017	ATP7B	chr13	52513298	52513298	http://gnomad.b	0.0009147231139	AFR	0.01006800029	1		1	0	https://www.ncbi.nlm.nih.gov/clinvar/variation/35723/
NM_000051.3(ATM):c.3118A>G	concordant	unreportable	unreportable	3027	0	5	SCV000602561;S	Benign;Likely benign;Likely	ARUP Laboratories	2017-06-30;2014-0	ATM	chr11	108143299	108143299	http://gnomad.b	0.003297094992	AFR	0.04310569912	19		26	0	https://www.ncbi.nlm.nih.gov/clinvar/variation/3027/
NM_000057.2(BLM):c.1928G>A	concordant	unreportable	unreportable	127480	0	5	SCV000331364;S	Likely benign;Likely benign	EGL Genetic Diagn	2017-06-30;2017-1	BLM	chr15	91306241	91306241	http://gnomad.b	0.003360605052	NFE	0.004572319798	2		7	0	https://www.ncbi.nlm.nih.gov/clinvar/variation/127480/
NM_017890.4(VPS13B):c.1293T>G	concordant	unreportable	unreportable	130722	0	3	SCV000153484;S	Likely benign;Likely benign	Genetic Services La	2014-06-27;2016-1	VPS13B	chr8	100146946	100146946	http://gnomad.b	0.003785100367	AFR	0.04418120161	19		25	0	https://www.ncbi.nlm.nih.gov/clinvar/variation/130722/
NM_002435.1(MPI):c.10C>T	concordant	unreportable	unreportable	282926	0	3	SCV000334671;S	Uncertain significance;Likel	EGL Genetic Diagn	2017-06-30;2017-0	MPI	chr15	75182424	75182424	http://gnomad.b	0.003465418593	ASJ	0.0105777001	3		9	0	https://www.ncbi.nlm.nih.gov/clinvar/variation/282926/
NM_001360.2(DHCR7):c.570C>T	concordant	unreportable	unreportable	305957	0	2	SCV000512801;S	Benign;Uncertain significar	GeneDx;Illumina C	2017-11-28;2016-1	DHCR7	chr11	71152329	71152329	http://gnomad.b	0.002864897467	AFR	0.03456610069	10		18	0	https://www.ncbi.nlm.nih.gov/clinvar/variation/305957/
NM_003060.3(SLC22A5):c.1377A>G	concordant	unreportable	unreportable	350817	0	2	SCV000452739;S	Uncertain significance;Likel	Illumina Clinical Se	2016-10-18;2017-1	SLC22A5	chr5	131728234	131728234	http://gnomad.b	0.0006410908146	ASJ	0.00294416002	0		2	0	https://www.ncbi.nlm.nih.gov/clinvar/variation/350817/
NM_000271.4(NPC1):c.3343G>T	concordant	unreportable	unreportable	92712	0	1	SCV000492076	Uncertain significance	GeneDx	12/21/2016	NPC1	chr18	21115567	21115567	http://gnomad.b	0.0007090819676	AFR	0.009999999776	1		1	0	https://www.ncbi.nlm.nih.gov/clinvar/variation/92712/
NM_000271.4(NPC1):c.2103C>T	concordant	unreportable	unreportable	194159	0	1	SCV000407868	Likely benign	Illumina Clinical Se	10/18/2016	NPC1	chr18	21124335	21124335	http://gnomad.b	0.003167995242	AFR	0.04026670009	14		16	0	https://www.ncbi.nlm.nih.gov/clinvar/variation/194159/
NM_002485.4(NBN):c.1903A>T	concordant	reportable	reportable	142559	3	0	SCV000537637;S	Pathogenic;Pathogenic;Pat	Color Genomics, In	2017-01-26;2017-1	NBN	chr8	90960063	90960063	http://gnomad.b	8.64E-05	ASJ	0.000764025026	0		0	0	https://www.ncbi.nlm.nih.gov/clinvar/variation/142559/
NM_000414.3(HSD17B4):c.1566T>A	concordant	unreportable	unreportable	226666	0	2	SCV000452134;S	Uncertain significance;Beni	Illumina Clinical Se	2016-10-18;2016-0	HSD17B4	chr5	118860973	118860973	http://gnomad.b	0.0004789568882	EAS	0.005971710198	1		0	0	https://www.ncbi.nlm.nih.gov/clinvar/variation/226666/
NM_000271.4(NPC1):c.2661G>A	concordant	unreportable	unreportable	326259	0	2	SCV000407859;S	Uncertain significance;Beni	Illumina Clinical Se	2016-10-18;2017-1	NPC1	chr18	21119909	21119909	http://gnomad.b	0.001947116209	AFR	0.02058289945	3		14	0	https://www.ncbi.nlm.nih.gov/clinvar/variation/326259/
NM_000018.3(ACADVL):c.393C>G	concordant	unreportable	unreportable	474897	0	1	SCV000654953	Likely benign	Invitae	10/5/2017	ACADVL	chr17	7124293	7124293	http://gnomad.b	4.25E-05	NFE	6.27E-05	0		0	0	https://www.ncbi.nlm.nih.gov/clinvar/variation/474897/
NM_000152.3(GAA):c.1830C>T	concordant	unreportable	unreportable	255356	0	3	SCV000333920;S	Likely benign;Likely benign	EGL Genetic Diagn	2017-06-30;2017-1	GAA	chr17	78086452	78086452	http://gnomad.b	0.001439680713	ASJ	0.003191930009	2		1	0	https://www.ncbi.nlm.nih.gov/clinvar/variation/255356/
NM_000018.3(ACADVL):c.663C>T	concordant	unreportable	unreportable	383052	0	1	SCV000523289	Likely benign	GeneDx	11/28/2017	ACADVL	chr17	7125311	7125311	http://gnomad.b	0.0003029959789	AFR	0.003399580019	0		0	0	https://www.ncbi.nlm.nih.gov/clinvar/variation/383052/
NM_000023.2(SGCA):c.158-11G>A	concordant	unreportable	unreportable	324041	0	2	SCV000522747;S	Likely benign;Uncertain sig	GeneDx;Illumina C	2017-11-28;2016-1	SGCA	chr17	48244932	48244932	http://gnomad.b	0.001662356853	ASJ	0.005287779961	0		3	0	https://www.ncbi.nlm.nih.gov/clinvar/variation/324041/
NM_033056.3(PCDH15):c.4831_4834c	concordant	unreportable	unreportable	227010	0	2	SCV000577447;S	Uncertain significance;Beni	GeneDx;Laborator	2017-11-28;2016-0	PCDH15	chr10	55582651	55582651	http://gnomad.b	0.0008769222857	AFR	0.01026009955	1		3	0	https://www.ncbi.nlm.nih.gov/clinvar/variation/227010/
NM_000152.3(GAA):c.841C>T	concordant	unreportable	unreportable	283894	0	2	SCV000336261;S	Uncertain significance;Unc	EGL Genetic Diagn	2017-06-30;2017-1	GAA	chr17	78081504	78081504	http://gnomad.b	0.00030850301	NFE	0.0004019149928	0		0	0	https://www.ncbi.nlm.nih.gov/clinvar/variation/283894/
NM_000478.4(ALPL):c.1119C>T	concordant	unreportable	unreportable	295548	0	1	SCV000354310	Likely benign	Illumina Clinical Se	10/18/2016	ALPL	chr1	21902347	21902347	http://gnomad.b	0.002366410493	AFR	0.02783589996	6		14	0	https://www.ncbi.nlm.nih.gov/clinvar/variation/295548/
NM_000310.3(PPT1):c.*3C>A	concordant	unreportable	unreportable	138793	0	1	SCV000357388	Uncertain significance	Illumina Clinical Se	10/18/2016	PPT1	chr1	40539730	40539730	http://gnomad.b	0.004107016478	AFR	0.02588240057	7		12	0	https://www.ncbi.nlm.nih.gov/clinvar/variation/138793/
NM_004937.2(CTNS):c.695G>A	concordant	unreportable	unreportable	322842	0	1	SCV000402235	Uncertain significance	Illumina Clinical Se	10/18/2016	CTNS	chr17	3561312	3561312	http://gnomad.b	0.0002518578218	AFR	0.001767019974	1		0	0	https://www.ncbi.nlm.nih.gov/clinvar/variation/322842/
NM_005562.2(LAMC2):c.1637C>A	concordant	unreportable	unreportable	294005	0	1	SCV000351676	Uncertain significance	Illumina Clinical Se	10/18/2016	LAMC2	chr1	183197677	183197677	http://gnomad.b	0.002169762194	NFE	0.002934100106	0		4	0	https://www.ncbi.nlm.nih.gov/clinvar/variation/294005/
NM_000227.3(LAMA3):c.4071C>T	concordant	unreportable	unreportable	326341	0	1	SCV000408020	Likely benign	Illumina Clinical Se	10/18/2016	LAMA3	chr18	21519222	21519222	http://gnomad.b	0.002760497449	EAS	0.03136960045	8		9	0	https://www.ncbi.nlm.nih.gov/clinvar/variation/326341/
NM_000543.4(SMPD1):c.99G>A	concordant	unreportable	unreportable	285418	0	1	SCV000338427	Benign	EGL Genetic Diagn	6/30/2017	SMPD1	chr11	6411927	6411927	http://gnomad.b	0.0007873453853	AFR	0.01033630036	1		3	0	https://www.ncbi.nlm.nih.gov/clinvar/variation/285418/
NM_000391.3(TPP1):c.299A>G	concordant	unreportable	unreportable	68745	0	4	SCV000230076;S	Benign;Likely benign;Benig	EGL Genetic Diagn	2017-06-30;2014-0	TPP1	chr11	6638938	6638938	http://gnomad.b	0.002515374844	AFR	0.03142970055	6		16	0	https://www.ncbi.nlm.nih.gov/clinvar/variation/68745/
NM_000352.3(ABCC8):c.423G>A	concordant	unreportable	unreportable	157702	0	1	SCV000369407	Likely benign	Illumina Clinical Se	10/18/2016	ABCC8	chr11	17485141	17485141	http://gnomad.b	0.00623331974	AFR	0.02789779939	16		20	0	https://www.ncbi.nlm.nih.gov/clinvar/variation/157702/
NM_000227.3(LAMA3):c.110T>G	concordant	unreportable	unreportable	326292	0	1	SCV000407906	Likely benign	Illumina Clinical Se	10/18/2016	LAMA3	chr18	21453118	21453118	http://gnomad.b	0.005523794592	EAS	0.03896100074	36		27	0	https://www.ncbi.nlm.nih.gov/clinvar/variation/326292/
NM_138694.3(PKHD1):c.2853C>T	concordant	unreportable	unreportable	241843	0	3	SCV000331013;S	Benign;Uncertain significar	EGL Genetic Diagn	2017-06-30;2016-1	PKHD1	chr6	51907901	51907901	http://gnomad.b	0.001417785142	FIN	0.02422690019	7		0	0	https://www.ncbi.nlm.nih.gov/clinvar/variation/241843/
NM_000492.3(CFTR):c.2245C>T	concordant	unreportable	unreportable	256251	0	3	SCV000703406;S	Uncertain significance;Likel	EGL Genetic Diagn	2017-06-30;2017-1	CFTR	chr7	117232466	117232466	http://gnomad.b	0.000675967153	NFE	0.001169060008	0		0	0	https://www.ncbi.nlm.nih.gov/clinvar/variation/256251/
NM_000023.2(SGCA):c.328C>T	concordant	unreportable	unreportable	92303	0	2	SCV000109768;S	Uncertain significance;Unc	EGL Genetic Diagn	2017-06-30;2016-1	SGCA	chr17	48245323	48245323	http://gnomad.b	0.0003722112145	ASJ	0.00284322002	0		0	0	https://www.ncbi.nlm.nih.gov/clinvar/variation/92303/
NM_000071.2(CBS):c.531+34G>T	concordant	unreportable	unreportable	496832	0	1	SCV000700357	Benign	EGL Genetic Diagn	6/30/2017	CBS	chr21	44485692	44485692	http://gnomad.b	0.005382179755	FIN	0.0139367003	7		10	0	https://www.ncbi.nlm.nih.gov/clinvar/variation/496832/
NM_002485.4(NBN):c.1809C>A	concordant	unreportable	unreportable	185174	0	5	SCV000707503;S	Likely benign;Likely benign	EGL Genetic Diagn	2017-06-30;2017-1	NBN	chr8	90965508	90965508	http://gnomad.b	0.0005326268823	EAS	0.004929250106	0		0	0	https://www.ncbi.nlm.nih.gov/clinvar/variation/185174/
NM_000182.4(HADHA):c.2113G>A	concordant	unreportable	unreportable	335374	0	1	SCV000429496	Uncertain significance	Illumina Clinical Se	10/18/2016	HADHA	chr2	26414385	26414385	http://gnomad.b	0.0001654180872	FIN	0.0004932740121	0		0	0	https://www.ncbi.nlm.nih.gov/clinvar/variation/335374/
NM_133647.1(SLC12A6):c.1601A>G	concordant	unreportable	unreportable	373219	0	1	SCV000491797	Uncertain significance	GeneDx	12/21/2016	SLC12A6	chr15	34542547	34542547	http://gnomad.b	0.0001748376085	AMR	0.004556010012	0		0	0	https://www.ncbi.nlm.nih.gov/clinvar/variation/373219/
NM_017890.4(VPS13B):c.2275G>C	concordant	unreportable	unreportable	95838	0	3	SCV000153485;S	Benign;Likely benign;Benig	Genetic Services La	2017-10-04;2016-1	VPS13B	chr8	100182333	100182333	http://gnomad.b	0.003936280411	AMR	0.07803089917	142		33	0	https://www.ncbi.nlm.nih.gov/clinvar/variation/95838/
NM_017890.4(VPS13B):c.9667C>T	concordant	unreportable	unreportable	95876	0	3	SCV000616280;S	Likely benign;Likely benign	Athena Diagnostics	2017-08-17;2015-0	VPS13B	chr8	100844858	100844858	http://gnomad.b	0.003810297041	ASJ	0.006410260219	3		4	0	https://www.ncbi.nlm.nih.gov/clinvar/variation/95876/
NM_000543.4(SMPD1):c.1625G>A	concordant	unreportable	unreportable	305207	0	1	SCV000372946	Uncertain significance	Illumina Clinical Se	10/18/2016	SMPD1	chr11	6415566	6415566	http://gnomad.b	0.00154376784	AFR	0.02247780003	5		3	0	https://www.ncbi.nlm.nih.gov/clinvar/variation/305207/
NM_133647.1(SLC12A6):c.1752A>C	concordant	unreportable	unreportable	315613	0	1	SCV000390309	Uncertain significance	Illumina Clinical Se	10/18/2016	SLC12A6	chr15	34537966	34537966	http://gnomad.b	0.002023967569	EAS	0.0222079996	6		8	0	https://www.ncbi.nlm.nih.gov/clinvar/variation/315613/
NM_005562.2(LAMC2):c.3459G>A	concordant	unreportable	unreportable	294022	0	1	SCV000351699	Uncertain significance	Illumina Clinical Se	10/18/2016	LAMC2	chr1	183212412	183212412	http://gnomad.b	0.001579026939	NFE	0.002431889996	1		0	0	https://www.ncbi.nlm.nih.gov/clinvar/variation/294022/
NM_000414.3(HSD17B4):c.1670A>T	concordant	unreportable	unreportable	286628	0	1	SCV000340140	Uncertain significance	EGL Genetic Diagn	6/30/2017	HSD17B4	chr5	118861708	118861708	http://gnomad.b	0.0003127306741	AFR	0.003856709925	0		0	0	https://www.ncbi.nlm.nih.gov/clinvar/variation/286628/
NM_000151.3(G6PC):c.954C>T	concordant	unreportable	unreportable	390692	0	1	SCV000533590	Likely benign	GeneDx	11/28/2017	G6PC	chr17	41063323	41063323	http://gnomad.b	3.07E-05	SAS	3.25E-05	0		0	0	https://www.ncbi.nlm.nih.gov/clinvar/variation/390692/
NM_000051.3(ATM):c.6503C>T	concordant	unreportable	unreportable	220531	0	4	SCV000537533;S	Uncertain significance;Unc	Color Genomics, In	2017-12-21;2017-1	ATM	chr11	108192078	108192078	http://gnomad.b	6.40E-05	EAS	0.0006959749735	0		0	0	https://www.ncbi.nlm.nih.gov/clinvar/variation/220531/
NM_000136.2(FANCC):c.915C>T	concordant	unreportable	unreportable	220578	0	1	SCV000261227	Likely benign	Invitae	1/6/2016	FANCC	chr9	97887449	97887449	http://gnomad.b	2.14E-05	AMR	2.98E-05	0		0	0	https://www.ncbi.nlm.nih.gov/clinvar/variation/220578/
NM_000053.3(ATP7B):c.3101A>G	concordant	unreportable	unreportable	35719	0	3	SCV000602605;S	Likely benign;Likely benign	ARUP Laboratories	2017-06-30;2017-0	ATP7B	chr13	52518387	52518387	http://gnomad.b	0.001043794474	AFR	0.01374750026	4		2	0	https://www.ncbi.nlm.nih.gov/clinvar/variation/35719/
NM_000051.3(ATM):c.6235G>A	concordant	unreportable	unreportable	133629	0	2	SCV000593487;S	Likely benign;Benign	Genetic Services La	2017-07-05;2017-1	ATM	chr11	108188136	108188136	http://gnomad.b	0.003945932098	AFR	0.01784309931	5		12	0	https://www.ncbi.nlm.nih.gov/clinvar/variation/133629/
NM_012203.1(GRHPR):c.286C>T	concordant	unreportable	unreportable	366852	0	1	SCV000480130	Uncertain significance	Illumina Clinical Se	10/18/2016	GRHPR	chr9	37425990	37425990	http://gnomad.b	0.0009210801758	ASJ	0.004568529781	0		0	0	https://www.ncbi.nlm.nih.gov/clinvar/variation/366852/
NM_033056.3(PCDH15):c.5450C>T	concordant	unreportable	unreportable	300170	0	1	SCV000363110	Uncertain significance	Illumina Clinical Se	10/18/2016	PCDH15	chr10	55582036	55582036	http://gnomad.b	4.89E-05	NFE	5.43E-05	0		0	0	https://www.ncbi.nlm.nih.gov/clinvar/variation/300170/
NM_000543.4(SMPD1):c.297C>G	concordant	unreportable	unreportable	305196	0	1	SCV000372923	Uncertain significance	Illumina Clinical Se	10/18/2016	SMPD1	chr11	6412125	6412125	http://gnomad.b	0.0006850618459	NFE	0.001462219981	0		1	0	https://www.ncbi.nlm.nih.gov/clinvar/variation/305196/
NM_138694.3(PKHD1):c.333C>T	concordant	unreportable	unreportable	500501	0	1	SCV000706488	Uncertain significance	EGL Genetic Diagn	6/30/2017	PKHD1	chr6	51944755	51944755	http://gnomad.b	8.62E-05	AFR	0.0009151520208	1		0	0	https://www.ncbi.nlm.nih.gov/clinvar/variation/500501/
NM_002485.4(NBN):c.2220T>C	concordant	unreportable	unreportable	136043	0	2	SCV000685762;S	Likely benign;Benign	Color Genomics, In	2017-10-26;2017-1	NBN	chr8	90949268	90949268	http://gnomad.b	0.0006609035137	EAS	0.007603019942	0		0	0	https://www.ncbi.nlm.nih.gov/clinvar/variation/136043/
NM_017890.4(VPS13B):c.11959C>G	concordant	unreportable	unreportable	283776	0	2	SCV000336089;S	Benign;Uncertain significar	EGL Genetic Diagn	2017-06-30;2016-1	VPS13B	chr8	100887784	100887784	http://gnomad.b	0.0008034939388	EAS	0.01136500016	0		1	0	https://www.ncbi.nlm.nih.gov/clinvar/variation/283776/
NM_000108.3(DLD):c.34G>A	concordant	unreportable	unreportable	137103	0	1	SCV000466199	Uncertain significance	Illumina Clinical Se	10/18/2016	DLD	chr7	107531729	107531729	http://gnomad.b	0.001793985087	AFR	0.02072980069	2		5	0	https://www.ncbi.nlm.nih.gov/clinvar/variation/137103/
NM_000352.3(ABCC8):c.-8G>T	concordant	unreportable	unreportable	255928	0	2	SCV000592969;S	Uncertain significance;Likel	Genetic Services La	2017-07-05;2016-1	ABCC8	chr11	17498331	17498331	http://gnomad.b	0.002774666137	NFE	0.005082820077	2		6	0	https://www.ncbi.nlm.nih.gov/clinvar/variation/255928/
NM_014363.4(SACS):c.8339T>G	concordant	unreportable	unreportable	240902	0	4	SCV000614985;S	Likely benign;Uncertain sig	Athena Diagnostics	2017-08-17;2017-0	SACS	chr13	23909676	23909676	http://gnomad.b	0.002889950678	NFE	0.004488890059	4		2	0	https://www.ncbi.nlm.nih.gov/clinvar/variation/240902/
NM_000051.3(ATM):c.6925C>T	concordant	unreportable	unreportable	183966	0	3	SCV000682375;S	Likely benign;Likely benign	Color Genomics, In	2017-10-26;2017-1	ATM	chr11	108196902	108196902	http://gnomad.b	0.0001002495099	ASJ	0.0003046299971	0		0	0	https://www.ncbi.nlm.nih.gov/clinvar/variation/183966/
NM_000466.2(PEX1):c.473-3C>T	concordant	unreportable	unreportable	167446	0	1	SCV000203230	Benign	EGL Genetic Diagn	6/30/2017	PEX1	chr7	92147359	92147359	http://gnomad.b	0.003508411868	AMR	0.01980940066	15		2	0	https://www.ncbi.nlm.nih.gov/clinvar/variation/167446/
NM_000051.3(ATM):c.7290T>G	concordant	unreportable	unreportable	489587	0	1	SCV000682402	Uncertain significance	Color Genomics, In	10/26/2017	ATM	chr11	108199948	108199948	http://gnomad.b	2.78E-06	NFE	9.01E-06	0		0	0	https://www.ncbi.nlm.nih.gov/clinvar/variation/489587/
NM_000051.3(ATM):c.6437G>C	concordant	unreportable	unreportable	133630	0	2	SCV000593488;S	Likely benign;Benign	Genetic Services La	2017-07-05;2017-1	ATM	chr11	108190770	108190770	http://gnomad.b	0.001194639993	AFR	0.01216800045	1		1	0	https://www.ncbi.nlm.nih.gov/clinvar/variation/133630/
NM_000528.3(MAN2B1):c.1744C>G	concordant	unreportable	unreportable	457139	0	1	SCV000627790	Benign	Invitae	10/5/2017	MAN2B1	chr19	12766594	12766594	http://gnomad.b	0.002194390923	AFR	0.02623319998	5		14	0	https://www.ncbi.nlm.nih.gov/clinvar/variation/457139/
NM_000271.4(NPC1):c.2131-6_2131-4	concordant	unreportable	unreportable	282052	0	1	SCV000333225	Benign	EGL Genetic Diagn	6/30/2017	NPC1	chr18	21123536	21123539		0.0001029312534					0	0	https://www.ncbi.nlm.nih.gov/clinvar/variation/282052/
NM_006493.2(CLN5):c.595C>T	concordant	reportable	reportable	205144	1	0	SCV000628964	Pathogenic	Invitae	10/5/2017	CLN5	chr13	77570145	77570145	http://gnomad.b	3.08E-05	AMR	5.96E-05	0		0	0	https://www.ncbi.nlm.nih.gov/clinvar/variation/205144/
NM_017739.3(POMGNT1):c.452C>T	concordant	unreportable	unreportable	290156	0	1	SCV000344660	Uncertain significance	EGL Genetic Diagn	6/30/2017	POMGNT1	chr1	46661565	46661565	http://gnomad.b	5.60E-06	AMR	2.98E-05	0		0	0	https://www.ncbi.nlm.nih.gov/clinvar/variation/290156/
NM_000466.2(PEX1):c.2842C>T	concordant	unreportable	unreportable	282809	0	2	SCV000334463;S	Uncertain significance;Unc	EGL Genetic Diagn	2017-06-30;2016-1	PEX1	chr7	92123885	92123885	http://gnomad.b	0.0004853534205	ASJ	0.003453880083	0		0	0	https://www.ncbi.nlm.nih.gov/clinvar/variation/282809/
NM_000466.2(PEX1):c.3720C>T	concordant	unreportable	unreportable	256222	0	1	SCV000470492	Uncertain significance	Illumina Clinical Se	10/18/2016	PEX1	chr7	92118654	92118654	http://gnomad.b	0.00138395061	AFR	0.01622189954	2		3	0	https://www.ncbi.nlm.nih.gov/clinvar/variation/256222/
NM_000228.2(LAMB3):c.867T>C	concordant	unreportable	unreportable	295126	0	1	SCV000353653	Uncertain significance	Illumina Clinical Se	10/18/2016	LAMB3	chr1	209804036	209804036	http://gnomad.b	0.0007390776087	AFR	0.008690539747	0		1	0	https://www.ncbi.nlm.nih.gov/clinvar/variation/295126/
NM_000023.2(SGCA):c.1109G>A	concordant	unreportable	unreportable	282905	0	1	SCV000334645	Uncertain significance	EGL Genetic Diagn	6/30/2017	SGCA	chr17	48252743	48252743	http://gnomad.b	5.60E-06	SAS	3.26E-05	0		0	0	https://www.ncbi.nlm.nih.gov/clinvar/variation/282905/
NM_001042432.1(CLN3):c.313A>G	concordant	unreportable	unreportable	128777	0	3	SCV000150724;S	Likely benign;Likely benign	Genetic Services La	2014-06-27;2016-1	CLN3	chr16	28499044	28499044	http://gnomad.b	0.005680429324	AMR	0.05039960146	65		49	0	https://www.ncbi.nlm.nih.gov/clinvar/variation/128777/
NM_133647.1(SLC12A6):c.21C>T	concordant	unreportable	unreportable	315626	0	1	SCV000390329	Uncertain significance	Illumina Clinical Se	10/18/2016	SLC12A6	chr15	34628861	34628861	http://gnomad.b	0.0001896543549	EAS	0.003420690075	0		0	0	https://www.ncbi.nlm.nih.gov/clinvar/variation/315626/
NM_000642.2(AGL):c.3668G>A	concordant	unreportable	unreportable	456493	0	1	SCV000626734	Likely benign	Invitae	10/5/2017	AGL	chr1	100368318	100368318	http://gnomad.b	0.0002567723713	EAS	0.003018340096	0		2	0	https://www.ncbi.nlm.nih.gov/clinvar/variation/456493/
NM_000153.3(GALC):c.525C>T	concordant	unreportable	unreportable	496798	0	1	SCV000700290	Uncertain significance	EGL Genetic Diagn	6/30/2017	GALC	chr14	88450795	88450795	http://gnomad.b	0.001882433234	FIN	0.00385685009	0		1	0	https://www.ncbi.nlm.nih.gov/clinvar/variation/496798/
NM_000112.3(SLC26A2):c.1046T>A	concordant	unreportable	unreportable	352025	0	2	SCV000706729;S	Uncertain significance;Unc	EGL Genetic Diagn	2017-06-30;2016-1	SLC26A2	chr5	149360202	149360202	http://gnomad.b	0.0007973192232	ASJ	0.001118569984	0		0	0	https://www.ncbi.nlm.nih.gov/clinvar/variation/352025/
NM_000466.2(PEX1):c.1797A>G	concordant	unreportable	unreportable	290906	0	1	SCV000345574	Likely benign	EGL Genetic Diagn	6/30/2017	PEX1	chr7	92136314	92136314	http://gnomad.b	0.0004631658356	AFR	0.004966020118	0		1	0	https://www.ncbi.nlm.nih.gov/clinvar/variation/290906/
NM_017890.4(VPS13B):c.10687G>A	concordant	unreportable	unreportable	167836	0	1	SCV000597899	Uncertain significance	Genetic Services La	7/5/2017	VPS13B	chr8	100866229	100866229	http://gnomad.b	8.96E-05	AFR	0.0006534240092	0		0	0	https://www.ncbi.nlm.nih.gov/clinvar/variation/167836/
NM_014363.4(SACS):c.6781C>A	concordant	unreportable	unreportable	240901	0	3	SCV000614973;S	Likely benign;Uncertain sig	Athena Diagnostics	2017-08-17;2016-1	SACS	chr13	23911234	23911234	http://gnomad.b	0.002809921275	FIN	0.02119060047	7		2	0	https://www.ncbi.nlm.nih.gov/clinvar/variation/240901/
NM_000492.3(CFTR):c.4436G>A	concordant	unreportable	unreportable	502054	0	1	SCV000708649	Uncertain significance	EGL Genetic Diagn	6/30/2017	CFTR	chr7	117307155	117307155	http://gnomad.b	1.54E-05	AMR	5.97E-05	0		0	0	https://www.ncbi.nlm.nih.gov/clinvar/variation/502054/
NM_000243.2(MEFV):c.932C>T	concordant	unreportable	unreportable	279848	0	2	SCV000335716;S	Uncertain significance;Unc	EGL Genetic Diagn	2017-06-30;2017-1	MEFV	chr16	3299759	3299759	http://gnomad.b	0.0001791211867	AFR	0.002230969956	0		0	0	https://www.ncbi.nlm.nih.gov/clinvar/variation/279848/
NM_000492.3(CFTR):c.4095G>A	concordant	unreportable	unreportable	455779	0	1	SCV000625750	Likely benign	Invitae	10/5/2017	CFTR	chr7	117304873	117304873	http://gnomad.b	5.78E-06	SAS	9.75E-05	0		0	0	https://www.ncbi.nlm.nih.gov/clinvar/variation/455779/
NM_000518.4(HBB):c.246C>A	concordant	unreportable	unreportable	439778	0	1	SCV000603927	Likely benign	ARUP Laboratories	6/30/2017	HBB	chr11	5247876	5247876	http://gnomad.b	0.0002402657386	EAS	0.003944769967	0		0	0	https://www.ncbi.nlm.nih.gov/clinvar/variation/439778/
NM_000098.2(CPT2):c.1941G>A	concordant	unreportable	unreportable	460431	0	1	SCV000632601	Uncertain significance	Invitae	10/5/2017	CPT2	chr1	53679231	53679231	http://gnomad.b	8.39E-05	AFR	0.00137218996	0		0	0	https://www.ncbi.nlm.nih.gov/clinvar/variation/460431/
NM_024649.4(BBS1):c.1194C>A	concordant	unreportable	unreportable	305469	0	1	SCV000373276	Uncertain significance	Illumina Clinical Se	10/18/2016	BBS1	chr11	66294133	66294133	http://gnomad.b	0.0004012706905	EAS	0.00295686	0		2	0	https://www.ncbi.nlm.nih.gov/clinvar/variation/305469/

Supplemental Table 2

NM_000543.4(SMPD1):c.8G>A	concordant	unreportable	unreportable	281886	0	1	SCV000332919	Uncertain significance	EGL Genetic Diagn	6/30/2017	SMPD1	chr11	6411836	6411836	http://gnomad.b	0.0005637488107	NFE	0.001088469988	0		0	0	https://www.ncbi.nlm.nih.gov/clinvar/variation/281886/
NM_000098.2(CPT2):c.1578T>C	concordant	unreportable	unreportable	297609	0	2	SCV000358107;S	Likely benign;Benign	Illumina Clinical Se	2016-10-18;2017-1	CPT2	chr1	53676924	53676924	http://gnomad.b	0.00219090843	FIN	0.0245446004	10		1	0	https://www.ncbi.nlm.nih.gov/clinvar/variation/297609/
NM_000108.3(DLD):c.321A>G	concordant	unreportable	unreportable	382483	0	1	SCV000522431	Likely benign	GeneDx	11/28/2017	DLD	chr7	107543976	107543976	http://gnomad.b	0.000425779384	NFE	0.0005740839988	0		0	0	https://www.ncbi.nlm.nih.gov/clinvar/variation/382483/
NM_014363.4(SACS):c.810T>G	concordant	unreportable	unreportable	448222	0	1	SCV000614982	Likely benign	Athena Diagnostics	8/17/2017	SACS	chr13	23929941	23929941	http://gnomad.b	0.0004979607322	FIN	0.01058300026	3		0	0	https://www.ncbi.nlm.nih.gov/clinvar/variation/448222/
NM_001173990.2(TMEM216):c.211G>	concordant	unreportable	unreportable	196320	0	2	SCV000536311;S	Uncertain significance;Likel	GeneDx;Invitae	2017-11-28;2017-1	TMEM216	chr11	61161430	61161430	http://gnomad.b	0.0007327495334	AFR	0.007781850174	0		1	0	https://www.ncbi.nlm.nih.gov/clinvar/variation/196320/
NM_017890.4(VPS13B):c.8073T>C	concordant	unreportable	unreportable	437240	0	1	SCV000597889	Likely benign	Genetic Services La	7/5/2017	VPS13B	chr8	100821659	100821659	http://gnomad.b	0.000599120916	ASJ	0.006501419935	0		0	0	https://www.ncbi.nlm.nih.gov/clinvar/variation/437240/
NM_004543.4(NEB):c.3348C>T	concordant	unreportable	unreportable	196865	0	2	SCV000417021;S	Uncertain significance;Beni	Illumina Clinical Se	2016-10-18;2017-1	NEB	chr2	152534609	152534609	http://gnomad.b	0.003090180609	EAS	0.03463279828	7		17	0	https://www.ncbi.nlm.nih.gov/clinvar/variation/196865/
NM_004646.3(NPHS1):c.1802G>C	concordant	unreportable	unreportable	180464	0	1	SCV000411569	Uncertain significance	Illumina Clinical Se	10/18/2016	NPHS1	chr19	36336398	36336398	http://gnomad.b	0.001909471536	EAS	0.02306249924	7		5	0	https://www.ncbi.nlm.nih.gov/clinvar/variation/180464/
NM_001079802.1(FKTN):c.1312C>T	concordant	unreportable	unreportable	291125	0	1	SCV000345826	Uncertain significance	EGL Genetic Diagn	6/30/2017	FKTN	chr9	108397471	108397471	http://gnomad.b	2.48E-05	EAS	0.0001159550011	0		0	0	https://www.ncbi.nlm.nih.gov/clinvar/variation/291125/
NM_000051.3(ATM):c.2685A>G	concordant	unreportable	unreportable	140770	0	3	SCV000682070;S	Benign;Likely benign;Benig	Color Genomics, In	2017-10-26;2016-1	ATM	chr11	108139183	108139183	http://gnomad.b	0.003993272144	AFR	0.04862109944	24		30	0	https://www.ncbi.nlm.nih.gov/clinvar/variation/140770/
NM_000352.3(ABCC8):c.1926C>G	concordant	unreportable	unreportable	157685	0	1	SCV000369356	Likely benign	Illumina Clinical Se	10/18/2016	ABCC8	chr11	17449950	17449950	http://gnomad.b	0.0006891496358	AFR	0.008905190043	0		2	0	https://www.ncbi.nlm.nih.gov/clinvar/variation/157685/
NM_024649.4(BBS1):c.1059A>G	concordant	unreportable	unreportable	497902	0	1	SCV000702653	Uncertain significance	EGL Genetic Diagn	6/30/2017	BBS1	chr11	66291302	66291302	http://gnomad.b	0.0001309702948	NFE	0.0002329919953	0		0	0	https://www.ncbi.nlm.nih.gov/clinvar/variation/497902/
NM_017890.4(VPS13B):c.11256A>G	concordant	unreportable	unreportable	283557	0	3	SCV000335742;S	Benign;Likely benign;Benig	EGL Genetic Diagn	2017-06-30;2016-1	VPS13B	chr8	100874140	100874140	http://gnomad.b	0.001937344271	AFR	0.02506140061	4		4	0	https://www.ncbi.nlm.nih.gov/clinvar/variation/283557/
NM_000152.3(GAA):c.2400C>T	concordant	unreportable	unreportable	167115	0	2	SCV000526400;S	Benign;Benign	GeneDx;Invitae	2017-11-28;2017-1	GAA	chr17	78091467	78091467	http://gnomad.b	0.002048348814	AFR	0.02473800071	7		11	0	https://www.ncbi.nlm.nih.gov/clinvar/variation/167115/
NM_005562.2(LAMC2):c.884G>A	concordant	unreportable	unreportable	293998	0	1	SCV000351667	Uncertain significance	Illumina Clinical Se	10/18/2016	LAMC2	chr1	183192390	183192390	http://gnomad.b	0.001696614051	ASJ	0.003963409923	1		0	0	https://www.ncbi.nlm.nih.gov/clinvar/variation/293998/
NM_002435.1(MPI):c.303C>T	concordant	unreportable	unreportable	196348	0	1	SCV000527186	Likely benign	GeneDx	11/28/2017	MPI	chr15	75183878	75183878	http://gnomad.b	0.002516487331	NFE	0.003715180093	1		0	0	https://www.ncbi.nlm.nih.gov/clinvar/variation/196348/
NM_014363.4(SACS):c.13717A>C	concordant	unreportable	unreportable	193707	0	3	SCV000614938;S	Uncertain significance;Likel	Athena Diagnostics	2017-08-17;2017-0	SACS	chr13	23904298	23904298	http://gnomad.b	0.003897728351	NFE	0.00513203023	1		3	0	https://www.ncbi.nlm.nih.gov/clinvar/variation/193707/
NM_000227.3(LAMA3):c.3456G>A	concordant	unreportable	unreportable	326332	0	1	SCV000407992	Uncertain significance	Illumina Clinical Se	10/18/2016	LAMA3	chr18	21508192	21508192	http://gnomad.b	0.000218376066	AFR	0.0005886969739	0		0	0	https://www.ncbi.nlm.nih.gov/clinvar/variation/326332/
NM_000383.3(AIRE):c.564C>G	concordant	unreportable	unreportable	287809	0	1	SCV000341717	Uncertain significance	EGL Genetic Diagn	6/30/2017	AIRE	chr21	45708253	45708253	http://gnomad.b	0.000298005314	EAS	0.0006960559986	0		0	0	https://www.ncbi.nlm.nih.gov/clinvar/variation/287809/
NM_014363.4(SACS):c.6336A>C	concordant	unreportable	unreportable	448215	0	1	SCV000614971	Likely benign	Athena Diagnostics	8/17/2017	SACS	chr13	23911679	23911679	http://gnomad.b	0.0009899933605	AFR	0.01124619972	0		3	0	https://www.ncbi.nlm.nih.gov/clinvar/variation/448215/
NM_000051.3(ATM):c.2127T>C	concordant	unreportable	unreportable	135742	0	3	SCV000682029;S	Benign;Uncertain significar	Color Genomics, In	2017-10-26;2016-1	ATM	chr11	108126944	108126944	http://gnomad.b	0.001386784887	EAS	0.01870369911	0		1	0	https://www.ncbi.nlm.nih.gov/clinvar/variation/135742/
NM_183050.2(BCKDHB):c.987C>T	concordant	unreportable	unreportable	358171	0	2	SCV000465669;S	Uncertain significance;Beni	Illumina Clinical Se	2016-10-18;2017-1	BCKDHB	chr6	80982887	80982887	http://gnomad.b	0.001048212656	SAS	0.008154120296	4		2	0	https://www.ncbi.nlm.nih.gov/clinvar/variation/358171/
NM_000023.2(SGCA):c.350G>A	concordant	unreportable	unreportable	283118	0	1	SCV000335023	Uncertain significance	EGL Genetic Diagn	6/30/2017	SGCA	chr17	48245345	48245345	http://gnomad.b	5.60E-05	SAS	0.0002923790016	0		0	0	https://www.ncbi.nlm.nih.gov/clinvar/variation/283118/
NM_017890.4(VPS13B):c.7783G>A	concordant	unreportable	unreportable	95868	0	1	SCV000491577	Uncertain significance	GeneDx	12/21/2016	VPS13B	chr8	100791188	100791188	http://gnomad.b	0.0003695512192	AFR	0.005032679997	0		0	0	https://www.ncbi.nlm.nih.gov/clinvar/variation/95868/
NM_000152.3(GAA):c.2580C>T	concordant	unreportable	unreportable	281751	0	3	SCV000332714;S	Benign;Likely benign;Benig	EGL Genetic Diagn	2017-06-30;2017-1	GAA	chr17	78092090	78092090	http://gnomad.b	0.001292377474	AFR	0.01510949992	0		4	0	https://www.ncbi.nlm.nih.gov/clinvar/variation/281751/
NM_000051.3(ATM):c.6095+15T>C	concordant	unreportable	unreportable	140766	0	2	SCV000682301;S	Benign;Likely benign	Color Genomics, In	2017-10-26;2016-1	ATM	chr11	108186653	108186653	http://gnomad.b	0.003996056853	AFR	0.04862109944	24		30	0	https://www.ncbi.nlm.nih.gov/clinvar/variation/140766/
NM_000152.3(GAA):c.2423C>T	concordant	unreportable	unreportable	281274	0	1	SCV000331972	Uncertain significance	EGL Genetic Diagn	6/30/2017	GAA	chr17	78091490	78091490	http://gnomad.b	5.56E-06	AFR	6.65E-05	0		0	0	https://www.ncbi.nlm.nih.gov/clinvar/variation/281274/
NM_000051.3(ATM):c.6995T>C	concordant	unreportable	unreportable	133631	0	2	SCV000703941;S	Benign;Benign	EGL Genetic Diagn	2017-06-30;2017-1	ATM	chr11	108198391	108198391	http://gnomad.b	0.001810060595	AFR	0.02196650021	6		11	0	https://www.ncbi.nlm.nih.gov/clinvar/variation/133631/
NM_000112.3(SLC26A2):c.2220A>G	concordant	unreportable	unreportable	255133	0	1	SCV000454853	Likely benign	Illumina Clinical Se	10/18/2016	SLC26A2	chr5	149361376	149361376	http://gnomad.b	0.003886234256	AFR	0.04667399824	16		23	0	https://www.ncbi.nlm.nih.gov/clinvar/variation/255133/
NM_000051.3(ATM):c.370A>G	concordant	unreportable	unreportable	127373	0	2	SCV000149087;S	Likely benign;Benign	GeneDx;Invitae	2017-11-28;2017-1	ATM	chr11	108106435	108106435	http://gnomad.b	0.0005318793441	AFR	0.00673379004	0		0	0	https://www.ncbi.nlm.nih.gov/clinvar/variation/127373/
NM_000492.3(CFTR):c.663G>A	concordant	unreportable	unreportable	290978	0	2	SCV000345650;S	Uncertain significance;Likel	EGL Genetic Diagn	2017-06-30;2017-0	CFTR	chr7	117175385	117175385	http://gnomad.b	5.20E-05	NFE	8.06E-05	0		0	0	https://www.ncbi.nlm.nih.gov/clinvar/variation/290978/
NM_000352.3(ABCC8):c.-72G>A	concordant	unreportable	unreportable	303791	0	2	SCV000592970;S	Uncertain significance;Likel	Genetic Services La	2017-07-05;2016-1	ABCC8	chr11	17498395	17498395	http://gnomad.b	0.005378980678	AMR	0.1011240035	0		7	0	https://www.ncbi.nlm.nih.gov/clinvar/variation/303791/
NM_000053.3(ATP7B):c.3006C>T	concordant	unreportable	unreportable	495409	0	1	SCV000694434	Uncertain significance	Laboratory Corpora	1/25/2018	ATP7B	chr13	52520474	52520474	http://gnomad.b	0.0001739657456	AMR	0.0002122110018	0		0	0	https://www.ncbi.nlm.nih.gov/clinvar/variation/495409/
NM_000057.2(BLM):c.465T>C	concordant	unreportable	unreportable	136508	0	1	SCV000555816	Likely benign	Invitae	10/5/2017	BLM	chr15	91292963	91292963	http://gnomad.b	0.0003591304761	NFE	0.0006627619732	0		0	0	https://www.ncbi.nlm.nih.gov/clinvar/variation/136508/
NM_000018.3(ACADVL):c.693T>A	concordant	unreportable	unreportable	136261	0	2	SCV000406316;S	Uncertain significance;Beni	Illumina Clinical Se	2016-10-18;2017-1	ACADVL	chr17	7125341	7125341	http://gnomad.b	0.0021096449	AFR	0.02026139945	5		10	0	https://www.ncbi.nlm.nih.gov/clinvar/variation/136261/
NM_000051.3(ATM):c.2193C>T	concordant	unreportable	unreportable	140761	0	3	SCV000682039;S	Benign;Likely benign;Benig	Color Genomics, In	2017-10-26;2016-1	ATM	chr11	108127010	108127010	http://gnomad.b	0.004466672607	AFR	0.05269350111	26		36	0	https://www.ncbi.nlm.nih.gov/clinvar/variation/140761/
NM_006493.2(CLN5):c.571G>C	concordant	unreportable	unreportable	373760	0	1	SCV000492381	Uncertain significance	GeneDx	12/21/2016	CLN5	chr13	77570121	77570121	http://gnomad.b	0.000137072906	EAS	0.0006377549726	0		0	0	https://www.ncbi.nlm.nih.gov/clinvar/variation/373760/
NM_000112.3(SLC26A2):c.1512G>A	concordant	unreportable	unreportable	282910	0	2	SCV000334650;S	Uncertain significance;Unc	EGL Genetic Diagn	2017-06-30;2016-1	SLC26A2	chr5	149360668	149360668	http://gnomad.b	0.0003233882164	EAS	0.003710580058	0		0	0	https://www.ncbi.nlm.nih.gov/clinvar/variation/282910/
NM_000057.2(BLM):c.645C>T	concordant	unreportable	unreportable	182061	0	1	SCV000623344	Likely benign	Invitae	10/5/2017	BLM	chr15	91293143	91293143	http://gnomad.b	0.000145079199	AFR	0.001569040003	0		0	0	https://www.ncbi.nlm.nih.gov/clinvar/variation/182061/
NM_017890.4(VPS13B):c.5980A>G	concordant	unreportable	unreportable	95858	0	1	SCV000249414	Likely benign	Genetic Services La	10/4/2017	VPS13B	chr8	100654723	100654723	http://gnomad.b	0.001699375682	NFE	0.002649510046	1		2	0	https://www.ncbi.nlm.nih.gov/clinvar/variation/95858/
NM_174878.2(CLRN1):c.9C>A	concordant	unreportable	unreportable	48149	0	2	SCV000702941;S	Uncertain significance;Beni	EGL Genetic Diagn	2017-06-30;2016-0	CLRN1	chr3	150690487	150690487	http://gnomad.b	0.0007946427194	EAS	0.007595079951	1		0	0	https://www.ncbi.nlm.nih.gov/clinvar/variation/48149/
NM_000159.2(GCDH):c.1210G>A	concordant	unreportable	unreportable	328333	0	2	SCV000492199;S	Uncertain significance;Unc	GeneDx;Illumina Cl	2016-12-21;2016-1	GCDH	chr19	13008644	13008644	http://gnomad.b	0.0001405663136	NFE	0.0001792889962	0		0	0	https://www.ncbi.nlm.nih.gov/clinvar/variation/328333/
NM_000153.3(GALC):c.1632T>C	concordant	unreportable	unreportable	495671	0	1	SCV000695670	Likely benign	Laboratory Corpora	1/25/2018	GALC	chr14	88411935	88411935	http://gnomad.b	0.0004978798152	AFR	0.006617739797	0		0	0	https://www.ncbi.nlm.nih.gov/clinvar/variation/495671/
NM_000053.3(ATP7B):c.1993A>G	concordant	unreportable	unreportable	495404	0	1	SCV000694410	Uncertain significance	Laboratory Corpora	1/25/2018	ATP7B	chr13	52534412	52534412	http://gnomad.b	0.0007940694516	ASJ	0.004874090198	0		0	0	https://www.ncbi.nlm.nih.gov/clinvar/variation/495404/
NM_000057.2(BLM):c.410A>G	concordant	unreportable	unreportable	133707	0	2	SCV000331616;S	Benign;Benign	EGL Genetic Diagn	2017-06-30;2017-1	BLM	chr15	91292908	91292908	http://gnomad.b	0.001196308805	AFR	0.01425400004	1		4	0	https://www.ncbi.nlm.nih.gov/clinvar/variation/133707/
NM_000492.3(CFTR):c.2973A>G	concordant	unreportable	unreportable	358733	0	1	SCV000466521	Uncertain significance	Illumina Clinical Se	10/18/2016	CFTR	chr7	117246792	117246792	http://gnomad.b	2.50E-05	NFE	2.69E-05	0		0	0	https://www.ncbi.nlm.nih.gov/clinvar/variation/358733/
NM_000051.3(ATM):c.3014A>G	concordant	unreportable	unreportable	127366	0	6	SCV000296850;S	Uncertain significance;Unc	Division of Genomi	2016-01-06;2017-0	ATM	chr11	108142070	108142070	http://gnomad.b	0.0008270584566	ASJ	0.00660568988	0		1	0	https://www.ncbi.nlm.nih.gov/clinvar/variation/127366/
NM_000232.4(SGCB):c.496A>G	concordant	unreportable	unreportable	285961	0	3	SCV000615220;S	Uncertain significance;Unc	Athena Diagnostics	2017-08-17;2017-0	SGCB	chr4	52895021	52895021	http://gnomad.b	0.0003694353796	NFE	0.0007431950071	0		0	0	https://www.ncbi.nlm.nih.gov/clinvar/variation/285961/
NM_017739.3(POMGNT1):c.1298C>T	concordant	unreportable	unreportable	162586	0	5	SCV000614739;S	Likely benign;Benign;Likely	Athena Diagnostics	2017-08-17;2017-0	POMGNT1	chr1	46658095	46658095	http://gnomad.b	0.001005044821	FIN	0.01834739931	8		1	0	https://www.ncbi.nlm.nih.gov/clinvar/variation/162586/
NM_000303.2(PMM2):c.366C>T	concordant	unreportable	unreportable	281078	0	2	SCV000331235;S	Likely benign;Likely benign	EGL Genetic Diagn	2017-06-30;2017-1	PMM2	chr16	8904954	8904954	http://gnomad.b	0.001809667522	EAS	0.008631950244	1		15	0	https://www.ncbi.nlm.nih.gov/clinvar/variation/281078/
NM_000051.3(ATM):c.660G>A	concordant	unreportable	unreportable	302243	0	1	SCV000367023	Uncertain significance	Illumina Clinical Se	10/18/2016	ATM	chr11	108114843	108114843	http://gnomad.b	1.67E-05	NFE	3.59E-05	0		0	0	https://www.ncbi.nlm.nih.gov/clinvar/variation/302243/
NM_006493.2(CLN5):c.152C>T	concordant	unreportable	unreportable	205125	0	3	SCV000701539;S	Uncertain significance;Likel	EGL Genetic Diagn	2017-06-30;2017-1	CLN5	chr13	77566238	77566238	http://gnomad.b	0.001465840872	ASJ	0.01297069993	0		0	0	https://www.ncbi.nlm.nih.gov/clinvar/variation/205125/
NM_012203.1(GRHPR):c.512G>A	concordant	unreportable	unreportable	366856	0	1	SCV000480136	Uncertain significance	Illumina Clinical Se	10/18/2016	GRHPR	chr9	37429747	37429747	http://gnomad.b	0.0011633123	EAS	0.01130700018	3		3	0	https://www.ncbi.nlm.nih.gov/clinvar/variation/366856/
NM_017890.4(VPS13B):c.3297A>C	concordant	unreportable	unreportable	361056	0	1	SCV000470792	Uncertain significance	Illumina Clinical Se	10/18/2016	VPS13B	chr8	100454715	100454715	http://gnomad.b	0.0002463674795	EAS	0.002034409903	0		0	0	https://www.ncbi.nlm.nih.gov/clinvar/variation/361056/
NM_000228.2(LAMB3):c.671T>C	concordant	unreportable	unreportable	295132	0	1	SCV000353660	Uncertain significance	Illumina Clinical Se	10/18/2016	LAMB3	chr1	209806079	209806079	http://gnomad.b	9.80E-05	SAS	0.0002598919964	0		1	0	https://www.ncbi.nlm.nih.gov/clinvar/variation/295132/
NM_000227.3(LAMA3):c.2391C>T	concordant	unreportable	unreportable	326314	0	1	SCV000407954	Uncertain significance	Illumina Clinical Se	10/18/2016	LAMA3	chr18	21492734	21492734	http://gnomad.b	0.001147874193	NFE	0.002812460065	2		3	0	https://www.ncbi.nlm.nih.gov/clinvar/variation/326314/
NM_005562.2(LAMC2):c.1066A>G	concordant	unreportable	unreportable	294000	0	1	SCV000351670	Uncertain significance	Illumina Clinical Se	10/18/2016	LAMC2	chr1	183194855	183194855	http://gnomad.b	2.52E-05	NFE	6.27E-05	0		0	0	https://www.ncbi.nlm.nih.gov/clinvar/variation/294000/
NM_000310.3(PPT1):c.904A>G	concordant	unreportable	unreportable	199009	0	5	SCV000232942;S	Uncertain significance;Unc	EGL Genetic Diagn	2017-06-30;2017-0	PPT1	chr1	40539750	40539750	http://gnomad.b	0.0009194813011	NFE	0.00173785002	0		0	0	https://www.ncbi.nlm.nih.gov/clinvar/variation/199009/
NM_000136.2(FANCC):c.383A>G	concordant	unreportable	unreportable	134303	0	1	SCV000570538	Uncertain significance	GeneDx	11/28/2017	FANCC	chr9	97934392	97934392	http://gnomad.b	3.09E-05	SAS	0.0001949949947	1		0	0	https://www.ncbi.nlm.nih.gov/clinvar/variation/134303/
NM_000051.3(ATM):c.2289T>A	concordant	unreportable	unreportable	127347	0	4	SCV000611351;S	Uncertain significance;Unc	Fulgent Genetics,F	2017-05-23;2017-0	ATM	chr11	108128246	108128246	http://gnomad.b	0.0008688290857	AFR	0.00202587992	1		1	0	https://www.ncbi.nlm.nih.gov/clinvar/variation/127347/
NM_000057.2(BLM):c.2619G>C	concordant	unreportable	unreportable	405297	0	1	SCV000543360	Uncertain significance	Invitae	3/14/2017	BLM	chr15	91326115	91326115	http://gnomad.b	7.37E-05	NFE	0.0001164770001	0		0	0	https://www.ncbi.nlm.nih.gov/clinvar/variation/405297/
NM_003060.3(SLC22A5):c.1380C>T	concordant	unreportable	unreportable	460397	0	1	SCV000632527	Likely benign	Invitae	10/5/2017	SLC22A5	chr5	131728237	131728237	http://gnomad.b	0.002380790999	ASJ	0.01999999955	1		1	0	https://www.ncbi.nlm.nih.gov/clinvar/variation/460397/
NM_138694.3(PKHD1):c.4920A>G	concordant	unreportable	unreportable	196805	0	2	SCV000229578;S	Likely benign;Benign	EGL Genetic Diagn	2017-06-30;2017-0	PKHD1	chr6	51889688	51889688	http://gnomad.b	0.003060747923	NFE	0.004122450016	0		7	0	https://www.ncbi.nlm.nih.gov/clinvar/variation/196805/
NM_138694.3(PKHD1):c.12106C>T	concordant	unreportable	unreportable	406885	0	2	SCV000700488;S	Uncertain significance;Unc	EGL Genetic Diagn	2017-06-30;2017-0	PKHD1	chr6	51483998	51483998	http://gnomad.b	0.0001556783685	ASJ	0.0009150059777	0		1	0	https://www.ncbi.nlm.nih.gov/clinvar/variation/406885/
NM_000642.2(AGL):c.2883A>G	concordant	unreportable	unreportable	256731	0	2	SCV000534232;S	Likely benign;Benign	GeneDx;Invitae	2017-11-28;2017-1	AGL	chr1	100356846	100356846	http://gnomad.b	0.0009824334206	AFR	0.01261440013	1		0	0	https://www.ncbi.nlm.nih.gov/clinvar/variation/256731/
NM_000227.3(LAMA3):c.3455C>T	concordant	unreportable	unreportable	326331	0	1	SCV000407989	Uncertain significance	Illumina Clinical Se	10/18/2016	LAMA3	chr18	21508191	21508191	http://gnomad.b	0.0004143545867	ASJ	0.004061739892	0		0	0	https://www.ncbi.nlm.nih.gov/clinvar/variation/326331/
NM_000466.2(PEX1):c.934A>G	concordant	unreportable	unreportable	283861	0	1	SCV000336207	Likely benign	EGL Genetic Diagn	6/30/2017	PEX1	chr7	92146895	92146895	http://gnomad.b	0.000782112369	AFR	0.008760459721	1		1	0	https://www.ncbi.nlm.nih.gov/clinvar/variation/283861/
NM_138694.3(PKHD1):c.158A>G	concordant	unreportable	unreportable	197367	0	3	SCV000230450;S	Likely benign;Uncertain sig	EGL Genetic Diagn	2017-06-30;2016-1	PKHD1	chr6	51947313	51947313	http://gnomad.b	0.001459484705	NFE	0.001863430021	0		0	0	https://www.ncbi.nlm.nih.gov/clinvar/variation/197367/
NM_001360.2(DHCR7):c.126C>T	concordant	unreportable	unreportable	166620	0	1	SCV000202100	Benign	EGL Genetic Diagn	6/30/2017	DHCR7	chr11	71155234	71155234	http://gnomad.b	0.001513225918	SAS	0.01336709969	4		3	0	https://www.ncbi.nlm.nih.gov/clinvar/variation/166620/
NM_000492.3(CFTR):c.1584+12T>C	concordant	unreportable	unreportable	35827	0	1	SCV000052130	Uncertain significance	Laboratory Corpora	11/20/2015	CFTR	chr7	117199721	117199721	http://gnomad.b	0.0003312431633	NFE	0.0003786309971	0		0	0	https://www.ncbi.nlm.nih.gov/clinvar/variation/35827/
NM_002485.4(NBN):c.1999T>C	concordant	unreportable	unreportable	127863	0	3	SCV000611486;S	Uncertain significance;Unc	Fulgent Genetics,F	2017-05-23;2017-0	NBN	chr8	90958439	90958439	http://gnomad.b	7.25E-05	AMR	8.94E-05	0		0	0	https://www.ncbi.nlm.nih.gov/clinvar/variation/127863/
NM_000051.3(ATM):c.162T>C	concordant	unreportable	unreportable	132757	0	5	SCV000681988;S	Benign;Likely benign;Likely	Color Genomics, In	2017-10-26;2017-0	ATM	chr11	108098592	108098592	http://gnomad.b	0.002277891641	NFE	0.002751389984	0		0	0	https://www.ncbi.nlm.nih.gov/clinvar/variation/132757/
NM_001042432.1(CLN3):c.242C>T	concordant	unreportable	unreportable	205105	0	4	SCV000339352;S	Uncertain significance;Unc	EGL Genetic Diagn	2017-06-30;2017-1	CLN3	chr16	28499964	28499964	http://gnomad.b	0.0008305370992	ASJ	0.002639590064	0		0	0	https://www.ncbi.nlm.nih.gov/clinvar/variation/205105/
NM_000153.3(GALC):c.41C>G	concordant	unreportable	unreportable	314763	0	1	SCV000389272	Uncertain significance	Illumina Clinical Se	10/18/2016	GALC	chr14	88459468	88459468	http://gnomad.b	0.001569160541	AMR	0.002013989957	1		2	0	https://www.ncbi.nlm.nih.gov/clinvar/variation/314763/
NM_000228.2(LAMB3):c.1439C>T	concordant	unreportable	unreportable	295108	0	1	SCV000353633	Uncertain significance	Illumina Clinical Se	10/18/2016	LAMB3	chr1	209800774	209800774	http://gnomad.b	0.002298419382	AFR	0.01087529957	1		5	0	https://www.ncbi.nlm.nih.gov/clinvar/variation/295108/
NM_000383.3(AIRE):c.497C>T	concordant	unreportable	unreportable	235694	0	1	SCV000629955	Benign	Invitae	10/5/2017	AIRE	chr21	45707433	45707433	http://gnomad.b	0.001386978003	AFR	0.01679709926	1		7	0	https://www.ncbi.nlm.nih.gov/clinvar/variation/235694/
NM_000057.2(BLM):c.2838A>G	concordant	unreportable	unreportable	236807	0	2	SCV000339649;S	Uncertain significance;Beni	EGL Genetic Diagn	2017-06-30;2017-1	BLM	chr15	91333893	91333893	http://gnomad.b	0.0001593492841	AMR	0.0005360330106	1		0	0	https://www.ncbi.nlm.nih.gov/clinvar/variation/236807/
NM_017739.3(POMGNT1):c.301G>A	concordant	unreportable	unreportable	95759	0	5	SCV000614742;S	Likely benign;Likely benign	Athena Diagnostics	2017-08-17;2017-1	POMGNT1	chr1	46662456	46662456	http://gnomad.b	0.006117055527	ASJ	0.04609140009	10		41	0	https://www.ncbi.nlm.nih.gov/clinvar/variation/95759/
NM_000057.2(BLM):c.191A>T	concordant	unreportable	unreportable	127479	0	3	SCV000228762;S	Uncertain significance;Unc	EGL Genetic Diagn	2017-06-30;2017-0	BLM	chr15	91292689	91292689	http://gnomad.b	0.000294915093	NFE	0.0005112109939	1		0	0	https://www.ncbi.nlm.nih.gov/clinvar/variation/127479/
NM_000137.2(FAH):c.296A>G	concordant	unreportable	unreportable	317211	0	2	SCV000709543;S	Likely benign;Uncertain sig	EGL Genetic Diagn	2017-06-30;2016-1	FAH	chr15	80452201	80452201	http://gnomad.b	0.0004105841947	SAS	0.005945030134	1		1	0	https://www.ncbi.nlm.nih.gov/clinvar/variation/317211/
NM_001360.2(DHCR7):c.1092G>A	concordant	unreportable	unreportable	166985	0	1	SCV000373909	Uncertain significance	Illumina Clinical Se	10/18/2016	DHCR7	chr11	71146757	71146757	http://gnomad.b	0.001556307083	AFR	0.0197158996	4		4	0	https://www.ncbi.nlm.nih.gov/clinvar/variation/166985/
NM_002485.4(NBN):c.1720T>A	concordant	unreportable	unreportable	127861	0	6	SCV000604437;S	Uncertain significance;Likel	ARUP Laboratories	2017-06-30;2017-1	NBN	chr8	90965597	90965597	http://gnomad.b	0.0004656999442	FIN	0.001396649983	2		0	0	https://www.ncbi.nlm.nih.gov/clinvar/variation/127861/
NM_017890.4(VPS13B):c.8645C>T	concordant	unreportable	unreportable	95872	0	3	SCV000113854;S	Likely benign;Uncertain sig	EGL Genetic Diagn	2017-06-30;2017-0	VPS13B	chr8	100831065	100831065	http://gnomad.b	0.002096923206	ASJ	0.01356870029	0		1	0	https://www.ncbi.nlm.nih.gov/clinvar/variation/95872/
NM_000227.3(LAMA3):c.4002G>A	concordant	unreportable	unreportable	290582	0	2	SCV000345159;S	Uncertain significance;Unc	EGL Genetic Diagn	2017-06-30;2016-1	LAMA3	chr18	21513866	21513866	http://gnomad.b	0.0008847030365	FIN	0.003184709931	1		2	0	https://www.ncbi.nlm.nih.gov/clinvar/variation/290582/
NM_000303.2(PMM2):c.426T>G	concordant	unreportable	unreportable	495798	0	1	SCV000696498	Uncertain significance	Laboratory Corpora	1/25/2018	PMM2	chr16	8905014	8905014	http://gnomad.b	3.34E-05	AFR	9.23E-05	0		0	0	https://www.ncbi.nlm.nih.gov/clinvar/variation/495798/
NM_000018.3(ACADVL):c.1894C>T	concordant	unreportable	unreportable	474894	0	1	SCV000654947	Uncertain significance	Invitae	10/5/2017	ACADVL	chr17	7128342	7128342	http://gnomad.b	0.0003199864077	NFE	0.0005823330139	0		0	0	https://www.ncbi.nlm.nih.gov/clinvar/variation/474894/
NM_002485.4(NBN):c.284A>G	concordant	unreportable	unreportable	141816	0	3	SCV000685771;S	Uncertain significance;Unc	Color Genomics, In	2017-10-26;2017-1	NBN	chr8	90993639	90993639	http://gnomad.b	2.51E-05	NFE	6.27E-05	0		0	0	https://www.ncbi.nlm.nih.gov/clinvar/variation/141816/
NM_000642.2(AGL):c.3384G>A	concordant	unreportable	unreportable	256739	0	2	SCV000534233;S	Likely benign;Benign	GeneDx;Invitae	2017-11-28;2017-1	AGL	chr1	100366213	100366213	http://gnomad.b	0.001021507477	AFR	0.01261110045	1		0	0	https://www.ncbi.nlm.nih.gov/clinvar/variation/256739/
NM_033056.3(PCDH15):c.1362C>T	concordant	unreportable	unreportable	46443	0	1	SCV000363210	Uncertain significance	Illumina Clinical Se	10/18/2016	PCDH15	chr10	55944972	55944972	http://gnomad.b	0.0008794939053	AFR	0.008039220236	1		0	0	https://www.ncbi.nlm.nih.gov/clinvar/variation/46443/
NM_000228.2(LAMB3):c.2873G>A	concordant	unreportable	unreportable	295077	0	1	SCV000353596	Uncertain significance	Illumina Clinical Se	10/18/2016	LAMB3	chr1	209791833	209791833	http://gnomad.b	0.0003723383408	NFE	0.0006457630079	0		0	0	https://www.ncbi.nlm.nih.gov/clinvar/variation/295077/
NM_133647.1(SLC12A6):c.374G>T	concordant	unreportable	unreportable	450842	0	1	SCV000619464	Uncertain significance	GeneDx	11/28/2017	SLC12A6	chr15	34553164	34553164	http://gnomad.b	0.0001096439239	NFE	1.79E-05	0		1	0	https://www.ncbi.nlm.nih.gov/clinvar/variation/450842/
NM_000487.5(ARSA):c.1447G>A	concordant	unreportable	unreportable	198734	0	2	SCV000232555;S	Uncertain significance;Unc	EGL Genetic Diagn	2017-06-30;2016-1	ARSA	chr22	51063656	51063656	http://gnomad.b	0.001072085812	AFR	0.007088330109	1		2	0	https://www.ncbi.nlm.nih.gov/clinvar/variation/198734/
NM_000352.3(ABCC8):c.1920G>A	concordant	unreportable	unreportable	303780	0	2	SCV000369359;S	Likely benign;Benign	Illumina Clinical Se	2016-10-18;2018-0	ABCC8	chr11	17450115	17450115	http://gnomad.b	0.0004465276666	AFR	0.00248333998	0		0	0	https://www.ncbi.nlm.nih.gov/clinvar/variation/303780/
NM_001360.2(DHCR7):c.1368C>T	concordant	unreportable	unreportable	93710	0	2	SCV000111527;S	Uncertain significance;Unc	EGL Genetic Diagn	2017-06-30;2016-1	DHCR7	chr11	71146481	71146481	http://gnomad.b	0.0007619981044	NFE	0.00141199003	1		2	0	https://www.ncbi.nlm.nih.gov/clinvar/variation/93710/
NM_000642.2(AGL):c.4539C>T	concordant	unreportable	unreportable	391085	0	1	SCV000534068	Likely benign	GeneDx	11/28/2017	AGL	chr1	100387147	100387147	http://gnomad.b	1.95E-05	NFE	8.97E-06	0		0	0	https://www.ncbi.nlm.nih.gov/clinvar/variation/391085/
NM_000030.2(AGXT):c.836T>C	concordant	unreportable	unreportable	198686	0	1	SCV000232462	Benign	EGL Genetic Diagn	6/30/2017	AGXT	chr2	241815411	241815411	http://gnomad.b	0.001999617983	NFE	0.002991710091	0		0	0	https://www.ncbi.nlm.nih.gov/clinvar/variation/198686/
NM_002435.1(MPI):c.678C>A	concordant	unreportable	unreportable	94070	0	2	SCV000111908;S	Uncertain significance;Unc	EGL Genetic Diagn	2017-06-30;2016-1	MPI	chr15	75188500	75188500	http://gnomad.b	0.0006018295619	ASJ	0.005691059865	0		1	0	https://www.ncbi.nlm.nih.gov/clinvar/variation/94070/

Supplemental Table 2

NM_005562.2(LAMC2):c.196G>A	concordant	unreportable	unreportable	195246	0	2	SCV000227366;S(	Uncertain significance;Unc(	EGL Genetic Diagn(	2017-06-30;2016-1	LAMC2	chr1	183177132	183177132	http://gnomad.b	0.001534231848	SAS	0.002728869906	2	1	0	https://www.ncbi.nlm.nih.gov/clinvar/variation/195246/
NM_138694.3(PKHD1):c.11338C>T	concordant	unreportable	unreportable	216820	0	2	SCV000343921;S(	Likely benign;Likely benign	EGL Genetic Diagn(	2017-06-30;2017-1	PKHD1	chr6	51512889	51512889	http://gnomad.b	0.001720801966	NFE	0.002559769899	1	0	0	https://www.ncbi.nlm.nih.gov/clinvar/variation/216820/
NM_000243.2(MEFV):c.42G>A	concordant	unreportable	unreportable	138208	0	2	SCV000629045;S(	Benign;Likely benign	Invitae;Laboratory	2017-10-05;2018-0	MEFV	chr16	3306546	3306546	http://gnomad.b	0.0008816120907	AFR	0.01176160015	1	3	0	https://www.ncbi.nlm.nih.gov/clinvar/variation/138208/
NM_000303.2(PMM2):c.110A>T	concordant	unreportable	unreportable	321215	0	2	SCV000399669;S(	Uncertain significance;Beni	Illumina Clinical Se	2016-10-18;2017-1	PMM2	chr16	8895699	8895699	http://gnomad.b	0.001450518122	EAS	0.01478980016	2	5	0	https://www.ncbi.nlm.nih.gov/clinvar/variation/321215/
NM_001876.3(CPT1A):c.1908G>A	concordant	unreportable	unreportable	287115	0	1	SCV000340789	Benign	EGL Genetic Diagn(	6/30/2017	CPT1A	chr11	68529123	68529123	http://gnomad.b	0.002106389569	AFR	0.02561420016	3	11	0	https://www.ncbi.nlm.nih.gov/clinvar/variation/287115/
NM_000098.2(CPT2):c.365C>T	concordant	unreportable	unreportable	447219	0	2	SCV000613022;S(	Uncertain significance;Likel	Athena Diagnostics	2017-08-17;2017-1	CPT2	chr1	53675711	53675711	http://gnomad.b	0.0005092533004	EAS	0.003362709889	0	1	0	https://www.ncbi.nlm.nih.gov/clinvar/variation/447219/
NM_000642.2(AGL):c.4430C>G	concordant	unreportable	unreportable	377192	0	1	SCV000626757	Benign	Invitae	10/5/2017	AGL	chr1	100382236	100382236	http://gnomad.b	0.001322935913	AFR	0.01687600091	1	10	0	https://www.ncbi.nlm.nih.gov/clinvar/variation/377192/
NM_014363.4(SACS):c.1519A>G	concordant	unreportable	unreportable	448194	0	1	SCV000614940	Uncertain significance	Athena Diagnostics	8/17/2017	SACS	chr13	23929232	23929232	http://gnomad.b	9.48E-05	AFR	0.001312509994	0	0	0	https://www.ncbi.nlm.nih.gov/clinvar/variation/448194/
NM_000243.2(MEFV):c.585G>A	concordant	unreportable	unreportable	282968	0	4	SCV000334728;S(	Benign;Uncertain significar	EGL Genetic Diagn(	2017-06-30;2016-1	MEFV	chr16	3304483	3304483	http://gnomad.b	0.001399384271	AFR	0.0177880004	1	3	0	https://www.ncbi.nlm.nih.gov/clinvar/variation/282968/
NM_003060.3(SLC22A5):c.1451G>T	concordant	unreportable	unreportable	203931	1	1	SCV000239172;S(	Likely pathogenic;Uncertai	GeneDx;Invitae	2017-11-28;2017-1	SLC22A5	chr5	131729368	131729368	http://gnomad.b	0.0003982105941	FIN	0.001210980001	0	0	0	https://www.ncbi.nlm.nih.gov/clinvar/variation/203931/
NM_000151.3(G6PC):c.132C>T	concordant	unreportable	unreportable	377897	0	1	SCV000513087	Likely benign	GeneDx	11/28/2017	G6PC	chr17	41053025	41053025	http://gnomad.b	0.0008970695727	SAS	0.007829249837	3	4	0	https://www.ncbi.nlm.nih.gov/clinvar/variation/377897/
NM_000227.3(LAMA3):c.4414C>T	concordant	unreportable	unreportable	326344	0	1	SCV000408026	Uncertain significance	Illumina Clinical Se	10/18/2016	LAMA3	chr18	21526138	21526138	http://gnomad.b	0.0003415625647	AFR	0.002548350021	0	0	0	https://www.ncbi.nlm.nih.gov/clinvar/variation/326344/
NM_000136.2(FANCC):c.77C>T	concordant	unreportable	unreportable	134301	0	3	SCV000296873;S(	Benign;Likely benign;Benig	Division of Genomi	2016-01-06;2017-0	FANCC	chr9	98011497	98011497	http://gnomad.b	0.005961921703	ASJ	0.009042879567	6	9	0	https://www.ncbi.nlm.nih.gov/clinvar/variation/134301/
NM_000035.3(ALDOB):c.619G>C	concordant	unreportable	unreportable	364310	0	2	SCV000529368;S(	Benign;Uncertain significar	GeneDx;Illumina C	2017-11-28;2016-1	ALDOB	chr9	104188842	104188842	http://gnomad.b	0.001858788487	EAS	0.01710340008	9	10	0	https://www.ncbi.nlm.nih.gov/clinvar/variation/364310/
NM_000071.2(CBS):c.394C>T	concordant	unreportable	unreportable	212843	0	3	SCV000344004;S(	Uncertain significance;Unc(	EGL Genetic Diagn(	2017-06-30;2017-1	CBS	chr21	44486410	44486410	http://gnomad.b	0.0003430647493	EAS	0.0005222239997	0	0	0	https://www.ncbi.nlm.nih.gov/clinvar/variation/212843/
NM_000228.2(LAMB3):c.1090C>T	concordant	unreportable	unreportable	295115	0	1	SCV000353642	Uncertain significance	Illumina Clinical Se	10/18/2016	LAMB3	chr1	209803124	209803124	http://gnomad.b	0.0006214970801	ASJ	0.005177659914	0	1	0	https://www.ncbi.nlm.nih.gov/clinvar/variation/295115/
NM_000051.3(ATM):c.6176C>T	concordant	unreportable	unreportable	127421	0	4	SCV000149135;S(	Uncertain significance;Unc(	GeneDx;Genetic Se	2017-07-13;2017-0	ATM	chr11	108186818	108186818	http://gnomad.b	0.0003508732846	AFR	0.003659169888	0	1	0	https://www.ncbi.nlm.nih.gov/clinvar/variation/127421/
NM_014363.4(SACS):c.11884A>T	concordant	unreportable	unreportable	373338	0	1	SCV000491925	Uncertain significance	GeneDx	12/21/2016	SACS	chr13	23906131	23906131	http://gnomad.b	0.0002252679503	ASJ	0.00284322002	0	0	0	https://www.ncbi.nlm.nih.gov/clinvar/variation/373338/
NM_004646.3(NPHS1):c.697A>G	concordant	unreportable	unreportable	259506	0	1	SCV000614342	Uncertain significance	Athena Diagnostics	8/17/2017	NPHS1	chr19	36340467	36340467	http://gnomad.b	0.0008390524561	AFR	0.01071619987	0	0	0	https://www.ncbi.nlm.nih.gov/clinvar/variation/259506/
NM_000352.3(ABCC8):c.-19A>G	concordant	unreportable	unreportable	35602	0	1	SCV000369433	Likely benign	Illumina Clinical Se	10/18/2016	ABCC8	chr11	17498342	17498342	http://gnomad.b	0.004607236329	AFR	0.04478900135	4	40	0	https://www.ncbi.nlm.nih.gov/clinvar/variation/35602/
NM_000352.3(ABCC8):c.1572G>A	concordant	unreportable	unreportable	35604	0	1	SCV000369379	Likely benign	Illumina Clinical Se	10/18/2016	ABCC8	chr11	17464325	17464325	http://gnomad.b	0.002903720376	AFR	0.03263999894	7	20	0	https://www.ncbi.nlm.nih.gov/clinvar/variation/35604/
NM_000383.3(AIRE):c.589G>A	concordant	unreportable	unreportable	495854	0	1	SCV000696654	Uncertain significance	Laboratory Corpora	1/25/2018	AIRE	chr21	45708278	45708278	http://gnomad.b	0.0001058336629	AFR	0.001049729995	0	0	0	https://www.ncbi.nlm.nih.gov/clinvar/variation/495854/
NM_000243.2(MEFV):c.333G>A	concordant	unreportable	unreportable	97517	0	2	SCV000709301;S(	Benign;Benign	EGL Genetic Diagn(	2017-06-30;2017-1	MEFV	chr16	3304735	3304735	http://gnomad.b	0.0009123985446	AFR	0.009857989848	0	4	0	https://www.ncbi.nlm.nih.gov/clinvar/variation/97517/
NM_000018.3(ACADVL):c.1824C>T	concordant	unreportable	unreportable	324996	0	1	SCV000406330	Uncertain significance	Illumina Clinical Se	10/18/2016	ACADVL	chr17	7128200	7128200	http://gnomad.b	0.001897264541	ASJ	0.005686429795	1	2	0	https://www.ncbi.nlm.nih.gov/clinvar/variation/324996/
NM_000232.4(SGCB):c.495C>T	concordant	unreportable	unreportable	288286	0	1	SCV000342353	Uncertain significance	EGL Genetic Diagn(	6/30/2017	SGCB	chr4	52895022	52895022	http://gnomad.b	0.0002714790289	NFE	0.0003581659985	0	0	0	https://www.ncbi.nlm.nih.gov/clinvar/variation/288286/
NM_000051.3(ATM):c.4473C>T	concordant	unreportable	unreportable	135754	0	5	SCV000682210;S(	Benign;Benign;Uncertain si	Color Genomics, In	2017-10-26;2017-0	ATM	chr11	108163382	108163382	http://gnomad.b	0.001559436821	ASJ	0.002233959967	0	0	0	https://www.ncbi.nlm.nih.gov/clinvar/variation/135754/
NM_000051.3(ATM):c.2921+16C>A	concordant	unreportable	unreportable	381297	0	1	SCV000520488	Likely benign	GeneDx	11/28/2017	ATM	chr11	108141889	108141889	http://gnomad.b	2.23E-05	NFE	5.38E-05	0	0	0	https://www.ncbi.nlm.nih.gov/clinvar/variation/381297/
NM_017890.4(VPS13B):c.10230G>A	concordant	unreportable	unreportable	130718	0	3	SCV000153479;S(	Likely benign;Likely benign	Genetic Services La	2014-06-27;2016-1	VPS13B	chr8	100865772	100865772	http://gnomad.b	0.003015201993	AFR	0.03986410052	14	9	0	https://www.ncbi.nlm.nih.gov/clinvar/variation/130718/
NM_000271.4(NPC1):c.2257G>A	concordant	unreportable	unreportable	287771	0	1	SCV000341671	Uncertain significance	EGL Genetic Diagn(	6/30/2017	NPC1	chr18	21121386	21121386	http://gnomad.b	0.0001686928875	NFE	0.0003761620028	0	0	0	https://www.ncbi.nlm.nih.gov/clinvar/variation/287771/
NM_004543.4(NEB):c.8490G>A	concordant	unreportable	unreportable	331486	0	1	SCV000416954	Uncertain significance	Illumina Clinical Se	10/18/2016	NEB	chr2	152497064	152497064	http://gnomad.b	0.0005886058303	ASJ	0.004670049995	0	0	0	https://www.ncbi.nlm.nih.gov/clinvar/variation/331486/
NM_138694.3(PKHD1):c.3783C>T	concordant	unreportable	unreportable	262400	0	3	SCV000342236;S(	Uncertain significance;Unc(	EGL Genetic Diagn(	2017-06-30;2016-1	PKHD1	chr6	51890825	51890825	http://gnomad.b	0.000725572396	NFE	0.001143109985	0	0	0	https://www.ncbi.nlm.nih.gov/clinvar/variation/262400/
NM_000243.2(MEFV):c.297C>T	concordant	unreportable	unreportable	97514	0	2	SCV000396785;S(	Uncertain significance;Unc(	Illumina Clinical Se	2016-10-18;2018-0	MEFV	chr16	3304771	3304771	http://gnomad.b	0.001105513574	ASJ	0.009983530268	1	3	0	https://www.ncbi.nlm.nih.gov/clinvar/variation/97514/
NM_000137.2(FAH):c.554-20T>G	concordant	unreportable	unreportable	382952	0	1	SCV000523148	Likely benign	GeneDx	11/28/2017	FAH	chr15	80460586	80460586		0.003204221249				1	0	https://www.ncbi.nlm.nih.gov/clinvar/variation/382952/
NM_000228.2(LAMB3):c.1188C>T	concordant	unreportable	unreportable	282914	0	2	SCV000334656;S(	Benign;Likely benign	EGL Genetic Diagn(	2017-06-30;2016-1	LAMB3	chr1	209801480	209801480	http://gnomad.b	0.001945677796	EAS	0.01250580046	1	2	0	https://www.ncbi.nlm.nih.gov/clinvar/variation/282914/
NM_000352.3(ABCC8):c.1707C>T	concordant	unreportable	unreportable	210070	0	2	SCV000246290;S(	Uncertain significance;Likel	Genetic Services La	2015-09-15;2016-1	ABCC8	chr11	17452471	17452471	http://gnomad.b	0.002452030539	ASJ	0.01777010039	2	7	0	https://www.ncbi.nlm.nih.gov/clinvar/variation/210070/
NM_000360.3(TH):c.44G>A	concordant	unreportable	unreportable	304086	0	1	SCV000369934	Uncertain significance	Illumina Clinical Se	10/18/2016	TH	chr11	2192973	2192973	http://gnomad.b	0.0002084612741	ASJ	0.001632650034	0	0	0	https://www.ncbi.nlm.nih.gov/clinvar/variation/304086/
NM_004543.4(NEB):c.5623A>G	concordant	unreportable	unreportable	331504	0	1	SCV000416988	Uncertain significance	Illumina Clinical Se	10/18/2016	NEB	chr2	152520202	152520202	http://gnomad.b	0.0001755491073	ASJ	0.00193011004	0	1	0	https://www.ncbi.nlm.nih.gov/clinvar/variation/331504/
NM_004004.5(GJB2):c.78C>T	concordant	unreportable	unreportable	227385	0	1	SCV000270219	Likely benign	Laboratory for Mol	4/26/2016	GJB2	chr13	20763643	20763643	http://gnomad.b	0.0002542595459	ASJ	0.001929330057	0	0	0	https://www.ncbi.nlm.nih.gov/clinvar/variation/227385/
NM_000360.3(TH):c.676G>A	concordant	unreportable	unreportable	304076	0	1	SCV000369920	Uncertain significance	Illumina Clinical Se	10/18/2016	TH	chr11	2188684	2188684		3.10E-05				0	0	https://www.ncbi.nlm.nih.gov/clinvar/variation/304076/
NM_006493.2(CLN5):c.50G>C	concordant	unreportable	unreportable	420530	0	1	SCV000569402	Uncertain significance	GeneDx	11/28/2017	CLN5	chr13	77566136	77566136	http://gnomad.b	2.80E-05	ASJ	0.0004397539888	0	0	0	https://www.ncbi.nlm.nih.gov/clinvar/variation/420530/
NM_017739.3(POMGNT1):c.486A>G	concordant	unreportable	unreportable	283314	0	2	SCV000335329;S(	Uncertain significance;Unc(	EGL Genetic Diagn(	2017-06-30;2016-1	POMGNT1	chr1	46661531	46661531	http://gnomad.b	0.00108063315	ASJ	0.01003479958	0	1	0	https://www.ncbi.nlm.nih.gov/clinvar/variation/283314/
NM_000642.2(AGL):c.39C>T	concordant	unreportable	unreportable	287104	0	3	SCV000340772;S(	Likely benign;Uncertain sig	EGL Genetic Diagn(	2017-06-30;2016-1	AGL	chr1	100316637	100316637	http://gnomad.b	0.00137596499	SAS	0.007666820195	2	3	0	https://www.ncbi.nlm.nih.gov/clinvar/variation/287104/
NM_017890.4(VPS13B):c.1463C>T	concordant	unreportable	unreportable	95832	0	2	SCV000597875;S(	Uncertain significance;Unc(	Genetic Services La	2017-07-05;2016-1	VPS13B	chr8	100147861	100147861	http://gnomad.b	0.001173045158	ASJ	0.007215450052	0	2	0	https://www.ncbi.nlm.nih.gov/clinvar/variation/95832/
NM_000053.3(ATP7B):c.3396C>T	concordant	unreportable	unreportable	312381	0	2	SCV000528337;S(	Likely benign;Uncertain sig	GeneDx;Illumina C	2017-11-28;2016-1	ATP7B	chr13	52516538	52516538	http://gnomad.b	0.0003563491885	ASJ	0.003248729976	0	0	0	https://www.ncbi.nlm.nih.gov/clinvar/variation/312381/
NM_138694.3(PKHD1):c.9493G>A	concordant	unreportable	unreportable	357417	0	2	SCV000704006;S(	Uncertain significance;Unc(	EGL Genetic Diagn(	2017-06-30;2016-1	PKHD1	chr6	51612921	51612921	http://gnomad.b	0.0006894327747	ASJ	0.00508646993	1	0	0	https://www.ncbi.nlm.nih.gov/clinvar/variation/357417/
NM_012434.4(SLC17A5):c.1192A>G	concordant	unreportable	unreportable	357923	0	1	SCV000464774	Uncertain significance	Illumina Clinical Se	10/18/2016	SLC17A5	chr6	74320190	74320190	http://gnomad.b	0.0002163706637	ASJ	0.001928930054	0	0	0	https://www.ncbi.nlm.nih.gov/clinvar/variation/357923/
NM_000136.2(FANCC):c.906C>G	concordant	unreportable	unreportable	377847	0	2	SCV000512971;S(	Likely benign;Likely benign	GeneDx;Invitae	2017-11-28;2017-1	FANCC	chr9	97887458	97887458	http://gnomad.b	0.0002116517876	EAS	0.001681350055	1	7	0	https://www.ncbi.nlm.nih.gov/clinvar/variation/377847/
NM_014363.4(SACS):c.7140T>A	concordant	unreportable	unreportable	285241	0	2	SCV000614974;S(	Uncertain significance;Unc(	Athena Diagnostics	2017-08-17;2017-0	SACS	chr13	23910875	23910875	http://gnomad.b	0.0003645783932	ASJ	0.003658540081	0	0	0	https://www.ncbi.nlm.nih.gov/clinvar/variation/285241/
NM_138694.3(PKHD1):c.1913T>C	concordant	unreportable	unreportable	497603	0	1	SCV000702214	Uncertain significance	EGL Genetic Diagn(	6/30/2017	PKHD1	chr6	51918887	51918887	http://gnomad.b	0.0003224766204	ASJ	0.001727640047	0	0	0	https://www.ncbi.nlm.nih.gov/clinvar/variation/497603/
NM_000492.3(CFTR):c.2475C>T	concordant	unreportable	unreportable	455768	0	1	SCV000625736	Likely benign	Invitae	10/5/2017	CFTR	chr7	117232696	117232696	http://gnomad.b	0.0001405857612	ASJ	0.0005617979914	0	0	0	https://www.ncbi.nlm.nih.gov/clinvar/variation/455768/
NM_000352.3(ABCC8):c.2610C>T	concordant	unreportable	unreportable	195647	0	1	SCV000369316	Likely benign	Illumina Clinical Se	10/18/2016	ABCC8	chr11	17432147	17432147	http://gnomad.b	0.0007020550597	AFR	0.003463150002	1	1	0	https://www.ncbi.nlm.nih.gov/clinvar/variation/195647/
NM_138694.3(PKHD1):c.2194G>T	concordant	unreportable	unreportable	288720	0	2	SCV000342912;S(	Uncertain significance;Beni	EGL Genetic Diagn(	2017-06-30;2017-1	PKHD1	chr6	51915040	51915040	http://gnomad.b	0.001754161616	ASJ	0.01210329961	1	3	0	https://www.ncbi.nlm.nih.gov/clinvar/variation/288720/
NM_000228.2(LAMB3):c.547C>T	concordant	unreportable	unreportable	295138	0	1	SCV000353666	Uncertain significance	Illumina Clinical Se	10/18/2016	LAMB3	chr1	209807809	209807809	http://gnomad.b	0.0009350451565	ASJ	0.004162440076	0	0	0	https://www.ncbi.nlm.nih.gov/clinvar/variation/295138/
NM_000478.4(ALPL):c.413G>A	concordant	unreportable	unreportable	295542	0	2	SCV000593124;S(	Uncertain significance;Unc(	Genetic Services La	2017-07-05;2016-1	ALPL	chr1	21889718	21889718	http://gnomad.b	0.0008169750513	ASJ	0.005998369772	0	0	0	https://www.ncbi.nlm.nih.gov/clinvar/variation/295542/
NM_000288.3(PEX7):c.615C>T	concordant	unreportable	unreportable	355532	0	2	SCV000704387;S(	Benign;Uncertain significar	EGL Genetic Diagn(	2017-06-30;2016-1	PEX7	chr6	137187853	137187853	http://gnomad.b	0.002391746392	ASJ	0.01868779957	1	8	0	https://www.ncbi.nlm.nih.gov/clinvar/variation/355532/
NM_000152.3(GAA):c.2109C>T	concordant	unreportable	unreportable	291223	0	2	SCV000345935;S(	Uncertain significance;Likel	EGL Genetic Diagn(	2017-06-30;2017-1	GAA	chr17	78087085	78087085	http://gnomad.b	0.0001778756094	AFR	0.0008709420217	0	0	0	https://www.ncbi.nlm.nih.gov/clinvar/variation/291223/
NM_000152.3(GAA):c.2152G>A	concordant	unreportable	unreportable	281232	0	4	SCV000331842;S(	Uncertain significance;Likel	EGL Genetic Diagn(	2017-06-30;2017-1	GAA	chr17	78087128	78087128	http://gnomad.b	0.0006725921479	ASJ	0.005604399834	0	1	0	https://www.ncbi.nlm.nih.gov/clinvar/variation/281232/
NM_138694.3(PKHD1):c.7744C>T	concordant	unreportable	unreportable	288719	0	2	SCV000342911;S(	Likely benign;Benign	EGL Genetic Diagn(	2017-06-30;2017-1	PKHD1	chr6	51720858	51720858	http://gnomad.b	0.001837560742	ASJ	0.01157599967	1	3	0	https://www.ncbi.nlm.nih.gov/clinvar/variation/288719/
NM_138694.3(PKHD1):c.2006G>A	concordant	unreportable	unreportable	357445	0	2	SCV000707781;S(	Uncertain significance;Unc(	EGL Genetic Diagn(	2017-06-30;2016-1	PKHD1	chr6	51918008	51918008	http://gnomad.b	0.0004420153677	ASJ	0.00122049998	0	0	0	https://www.ncbi.nlm.nih.gov/clinvar/variation/357445/
NM_014363.4(SACS):c.3868G>T	concordant	unreportable	unreportable	311546	0	2	SCV000596918;S(	Uncertain significance;Unc(	Genetic Services La	2017-07-05;2016-1	SACS	chr13	23914147	23914147	http://gnomad.b	5.04E-05	ASJ	0.0006122449995	0	0	0	https://www.ncbi.nlm.nih.gov/clinvar/variation/311546/
NM_000051.3(ATM):c.198A>G	concordant	unreportable	unreportable	135741	0	2	SCV000682020;S(	Likely benign;Likely benign	Color Genomics, In	2017-10-26;2017-1	ATM	chr11	108099917	108099917	http://gnomad.b	0.0001977143112	ASJ	0.002251330065	0	0	0	https://www.ncbi.nlm.nih.gov/clinvar/variation/135741/
NM_003060.3(SLC22A5):c.934A>G	concordant	unreportable	unreportable	381879	0	2	SCV000521561;S(	Likely benign;Likely benign	GeneDx;Invitae	2017-11-28;2017-1	SLC22A5	chr5	131722826	131722826	http://gnomad.b	0.00109860937	NFE	0.001647060039	0	0	0	https://www.ncbi.nlm.nih.gov/clinvar/variation/381879/
NM_000030.2(AGXT):c.1111G>A	concordant	unreportable	unreportable	288249	0	1	SCV000342301	Uncertain significance	EGL Genetic Diagn(	6/30/2017	AGXT	chr2	241818170	241818170	http://gnomad.b	0.000414846119	ASJ	0.003016760107	0	0	0	https://www.ncbi.nlm.nih.gov/clinvar/variation/288249/
NM_017890.4(VPS13B):c.358A>G	concordant	unreportable	unreportable	212575	0	2	SCV000249408;S(	Likely benign;Uncertain sig	Genetic Services La	2015-09-15;2016-1	VPS13B	chr8	100108606	100108606	http://gnomad.b	0.001153447745	SAS	0.007342909928	4	2	0	https://www.ncbi.nlm.nih.gov/clinvar/variation/212575/
NM_033056.3(PCDH15):c.4812G>T	concordant	unreportable	unreportable	227009	0	2	SCV000363136;S(	Uncertain significance;Beni	Illumina Clinical Se	2016-10-18;2016-0	PCDH15	chr10	55582674	55582674	http://gnomad.b	0.002332458983	EAS	0.02835110016	10	11	0	https://www.ncbi.nlm.nih.gov/clinvar/variation/227009/
NM_000518.4(HBB):c.294C>T	concordant	unreportable	unreportable	439783	0	2	SCV000603947;S(	Likely benign;Uncertain sig	ARUP Laboratories	2017-06-30;2018-0	HBB	chr11	5247828	5247828	http://gnomad.b	0.0008211023299	ASJ	0.009547020309	1	1	0	https://www.ncbi.nlm.nih.gov/clinvar/variation/439783/
NM_000528.3(MAN2B1):c.135C>T	concordant	unreportable	unreportable	328283	0	1	SCV000410813	Likely benign	Illumina Clinical Se	10/18/2016	MAN2B1	chr19	12777381	12777381	http://gnomad.b	0.001940866939	EAS	0.01605219953	3	8	0	https://www.ncbi.nlm.nih.gov/clinvar/variation/328283/
NM_000383.3(AIRE):c.348G>A	concordant	unreportable	unreportable	458618	0	1	SCV000629953	Benign	Invitae	10/5/2017	AIRE	chr21	45706901	45706901	http://gnomad.b	0.001228227509	SAS	0.002698129974	0	2	0	https://www.ncbi.nlm.nih.gov/clinvar/variation/458618/
NM_000023.2(SGCA):c.320C>T	concordant	unreportable	unreportable	197094	0	1	SCV000403946	Uncertain significance	Illumina Clinical Se	10/18/2016	SGCA	chr17	48245315	48245315	http://gnomad.b	0.0003890026978	EAS	0.003768549999	0	0	0	https://www.ncbi.nlm.nih.gov/clinvar/variation/197094/
NM_000051.3(ATM):c.8730C>G	concordant	unreportable	unreportable	135785	0	3	SCV000682510;S(	Likely benign;Benign;Likely	Color Genomics, In	2017-10-26;2017-1	ATM	chr11	108224551	108224551	http://gnomad.b	0.0002673320264	SAS	0.004905459937	1	1	0	https://www.ncbi.nlm.nih.gov/clinvar/variation/135785/
NM_000352.3(ABCC8):c.3989-10C>T	concordant	unreportable	unreportable	303755	0	1	SCV000369256	Likely benign	Illumina Clinical Se	10/18/2016	ABCC8	chr11	17418603	17418603	http://gnomad.b	0.0004129735645	SAS	0.00341107999	0	2	0	https://www.ncbi.nlm.nih.gov/clinvar/variation/303755/
NM_002225.3(IVD):c.173A>T	concordant	unreportable	unreportable	315797	0	2	SCV000617988;S(	Uncertain significance;Unc(	GeneDx;Illumina C	2017-11-28;2016-1	IVD	chr15	40699856	40699856	http://gnomad.b	0.0008770799123	NFE	0.002470589941	0	0	0	https://www.ncbi.nlm.nih.gov/clinvar/variation/315797/
NM_000023.2(SGCA):c.329G>A	concordant	unreportable	unreportable	497191	0	1	SCV000701535	Benign	EGL Genetic Diagn(	6/30/2017	SGCA	chr17	48245324	48245324	http://gnomad.b	0.0003134410227	FIN	0.006592520047	2	0	0	https://www.ncbi.nlm.nih.gov/clinvar/variation/497191/
NM_000352.3(ABCC8):c.3435C>T	concordant	unreportable	unreportable	303763	0	1	SCV000369284	Likely benign	Illumina Clinical Se	10/18/2016	ABCC8	chr11	17426181	17426181	http://gnomad.b	0.0006555955337	SAS	0.006239790004	0	2	0	https://www.ncbi.nlm.nih.gov/clinvar/variation/303763/
NM_000352.3(ABCC8):c.4431C>T	concordant	unreportable	unreportable	157703	0	1	SCV000369233	Likely benign	Illumina Clinical Se	10/18/2016	ABCC8	chr11	17415927	17415927	http://gnomad.b	0.0001884191888	NFE	0.0003491310054	0	0	0	https://www.ncbi.nlm.nih.gov/clinvar/variation/157703/
NM_000151.3(G6PC):c.*3G>A	concordant	unreportable	unreportable	212778	0	1	SCV000402986	Likely benign	Illumina Clinical Se	10/18/2016	G6PC	chr17	41063446	41063446	http://gnomad.b	0.003667506658	SAS	0.04350509867	46	45	0	https://www.ncbi.nlm.nih.gov/clinvar/variation/212778/
NM_000053.3(ATP7B):c.4361T>C	concordant	unreportable	unreportable	434448	0	1	SCV000593532	Uncertain significance	Genetic Services La	7/5/2017	ATP7B	chr13	52508929	52508929	http://gnomad.b	2.81E-06			0	0	0	https://www.ncbi.nlm.nih.gov/clinvar/variation/434448/
NM_002485.4(NBN):c.1036G>A	concordant	unreportable	unreportable	127854	0	4	SCV000341709;S(	Uncertain significance;Unc(	EGL Genetic Diagn(	2017-06-30;2017-1	NBN	chr8	90971041	90971041	http://gnomad.b	0.0002509760178	EAS	0.003304729937	0	0	0	https://www.ncbi.nlm.nih.gov/clinvar/variation/127854/
NM_138694.3(PKHD1):c.3984C>T	concordant	unreportable	unreportable	96401	0	1	SCV000557655	Benign	Invitae	3/14/2017	PKHD1	chr6	51890624	51890624	http://gnomad.b	0.0009646498905	EAS	0.006207940169	0	4	0	https://www.ncbi.nlm.nih.gov/clinvar/variation/96401/
NM_033056.3(PCDH15):c.1360G>A	concordant	unreportable	unreportable	46442	0	3	SCV000514042;S(	Benign;Uncertain significar	GeneDx;Illumina C	2017-11-28;2016-1	PCDH15	chr10	55944974	55944974	http://gnomad.b	0.003121946202	AFR	0.03738560155	8	22	0	https://www.ncbi.nlm.nih.gov/clinvar/variation/46442/
NM_000370.3(TTPA):c.48G>A	concordant	unreportable	unreportable	286666	0	1	SCV000340197	Benign	EGL Genetic Diagn(	6/30/2017	TTPA	chr8	63998533	63998533	http://gnomad.b	0.0007055743375	AFR	0.01139940042	0	0	0	https://www.ncbi.nlm.nih.gov/clinvar/variation/286666/
NM_000016.4(ACADM):c.1091T>C	concordant	unreportable	unreportable	92253	0	3	SCV000109705;S(	Uncertain significance;Unc(	EGL Genetic Diagn(	2017-06-30;2016-1	ACADM	chr1	76226952	76226952	http://gnomad.b	0.0006037661956	SAS	0.001331949956	2	0	0	https://www.ncbi.nlm.nih.gov/clinvar/variation/92253/
NM_000383.3(AIRE):c.1215G>A	concordant	unreportable	unreportable	458613	0	1	SCV000629944	Likely benign	Invitae	10/5/2017	AIRE	chr21	45712995	45712995	http://gnomad.b	0.0004762514831	NFE	0.0009451230289	0	0	0	https://www.ncbi.nlm.nih.gov/clinvar/variation/458613/
NM_001079802.1(FKTN):c.1023G>A	concordant	unreportable	unreportable	36136	0	2	SCV000338031;S(	Likely benign;Benign	EGL Genetic Diagn(	2017-06-30;2017-0	FKTN	chr9	108380352	108380352	http://gnomad.b	0.000287211636	FIN	0.008343050256	1	0	0	https://www.ncbi.nlm.nih.gov/clinvar/variation/36136/
NM_000016.4(ACADM):c.582C>T	concordant	unreportable	unreportable	386599	0	1	SCV000528329	Likely benign	GeneDx	11/28/2017	ACADM	chr1	76205778	76205778	http://gnomad.b	0.0002094144012	SAS	0.0007797270082	0	0	0	https://www.ncbi.nlm.nih.gov/clinvar/variation/386599/
NM_133647.1(SLC12A6):c.1250A>G	concordant	unreportable	unreportable	315615	0	1	SCV000390314	Uncertain significance	Illumina Clinical Se	10/18/2016	SLC12A6	chr15	34544454	34544454	http://gnomad.b	0.0004356123465	ASJ	0.001421320019	0	0	0	https://www.ncbi.nlm.nih.gov/clinvar/variation/315615/
NM_000414.3(HSD17B4):c.666C>G	concordant	unreportable	unreportable	199018	0	1	SCV000452112	Uncertain significance	Illumina Clinical Se	10/18/2016	HSD17B4	chr5	118824930	118824930	http://gnomad.b	0.001008626859	AFR	0.01163700037	1	2	0	https://www.ncbi.nlm.nih.gov/clinvar/variation/199018/
NM_000227.3(LAMA3):c.3101G>T	concordant	unreportable	unreportable	326324	0	1	SCV000407976	Uncertain significance	Illumina Clinical Se	10/18/2016	LAMA3	chr18	21500903	21500903	http://gnomad.b	0.00176660638	SAS	0.00675720023	2	0	0	https://www.ncbi.nlm.nih.gov/clinvar/variation/326324/
NM_138694.3(PKHD1):c.1925C>T	concordant	unreportable	unreportable	167495	0	1	SCV000203282	Uncertain significance	EGL Genetic Diagn(	6/30/2017	PKHD1	chr6	51918875	51918875	http://gnomad.b	0.0002112777858	AFR	0.002744740108	0	0	0	https://www.ncbi.nlm.nih.gov/clinvar/variation/167495/
NM_138694.3(PKHD1):c.4343A>G	concordant	unreportable	unreportable	96403	0	2	SCV000291333;S(	Benign;Benign	Invitae;Laboratory	2017-10-05;2018-0	PKHD1	chr6	51890265	51890265	http://gnomad.b	0.001770841442	AFR	0.02184149995	3	5	0	https://www.ncbi.nlm.nih.gov/clinvar/variation/96403/
NM_000243.2(MEFV):c.1245C>T	concordant	unreportable	unreportable	97435	0	1	SCV000696043	Uncertain significance	Laboratory Corpora	1/25/2018	MEFV	chr16	3299446	3299446	http://gnomad.b	0.0003554436048	SAS	0.002275829902	0	0	0	https://www.ncbi.nlm.nih.gov/clinvar/variation/97435/
NM_001164277.1(SLC37A4):c.1067G>	concordant	unreportable	unreportable	199062	0	2	SCV000533600;S(	Likely benign;Benign	GeneDx;Invitae	2017-11-28;2017-1	SLC37A4	chr11	118895957	118895957	http://gnomad.b	0.0008576616846	SAS	0.00868599955	3	2	0	https://www.ncbi.nlm.nih.gov/clinvar/variation/199062/
NM_000271.4(NPC1):c.2731G>A	concordant	unreportable	unreportable	181452	0	1	SCV000331143	Benign	EGL Genetic Diagn(	6/30/2017	NPC1	chr18	21119839	21119839	http://gnomad.b	0.001152258197	AFR	0.01032540016	1	2	0	https://www.ncbi.nlm.nih.gov/clinvar/variation/181452/
NM_000051.3(ATM):c.-222T>A	concordant	unreportable	unreportable	302237	0	1	SCV000367013	Uncertain significance	Illumina Clinical Se	10/18/2016	ATM	chr11	108093722	108093722		0.0001197424701				1	0	https://www.ncbi.nlm.nih.gov/clinvar/variation/302237/
NM_000152.3(GAA):c.1195-15G>A	concordant	unreportable	unreportable	325786	0	2	SCV000527907;S(	Likely benign;Uncertain sig	GeneDx;Illumina C	2017-11-28;2016-1	GAA	chr17	78082481	78082481	http://gnomad.b	0.0003724270571	ASJ	0.00265956996	1	0	0	https://www.ncbi.nlm.nih.gov/clinvar/variation/325786/
NM_000642.2(AGL):c.3696C>T	concordant	unreportable	unreportable	384564	0	1	SCV000525444	Likely benign	GeneDx	11/28/2017	AGL	chr1	100368346	100368346	http://gnomad.b	4.74E-05	AFR	0.0001987279975	0	0	0	https://www.ncbi.nlm.nih.gov/clinvar/variation/384564/
NM_001173990.2(TMEM216):c.358A>	concordant	unreportable	unreportable	305080	0	1	SCV000372636	Uncertain significance	Illumina Clinical Se	10/18/2016	TMEM216	chr11	61165374	61165374	http://gnomad.b	0.0001105551928	SAS	0.00126696995	2	0	0	https://www.ncbi.nlm.nih.gov/clinvar/variation/305080/

Supplemental Table 2

NM_138694.3(PKHD1):c.9553G>A	concordant	unreportable	unreportable	458608	0		2	SCV000702120;S	Uncertain significance;Likel	EGL Genetic Diagn	2017-06-30;2017-1	PKHD1	chr6	51612861	51612861	http://gnomad.b	0.0002390774945	AFR	0.002745819977	0		0	0	https://www.ncbi.nlm.nih.gov/clinvar/variation/458608/
NM_000112.3(SLC26A2):c.782C>G	concordant	unreportable	unreportable	426387	0		1	SCV000576810	Uncertain significance	GeneDx	11/28/2017	SLC26A2	chr5	149359938	149359938	http://gnomad.b	0.0007582896109	FIN	0.00134625996	0		1	0	https://www.ncbi.nlm.nih.gov/clinvar/variation/426387/
NM_000018.3(ACADVL):c.753-27C>T	concordant	unreportable	unreportable	474903	0		1	SCV000654966	Benign	Invitae	10/5/2017	ACADVL	chr17	7125469	7125469	http://gnomad.b	0.001965226256	NFE	0.002891209908	0		1	0	https://www.ncbi.nlm.nih.gov/clinvar/variation/474903/
NM_138694.3(PKHD1):c.3303A>G	concordant	unreportable	unreportable	287087	0		2	SCV000340747;S	Likely benign;Uncertain sig	EGL Genetic Diagn	2017-06-30;2016-1	PKHD1	chr6	51897889	51897889	http://gnomad.b	0.0007005526582	AFR	0.006928099785	0		0	0	https://www.ncbi.nlm.nih.gov/clinvar/variation/287087/
NM_138694.3(PKHD1):c.7977G>A	concordant	unreportable	unreportable	281377	0		1	SCV000332144	Likely benign	EGL Genetic Diagn	6/30/2017	PKHD1	chr6	51712703	51712703	http://gnomad.b	0.0001362185724	AMR	0.003693549894	1		0	0	https://www.ncbi.nlm.nih.gov/clinvar/variation/281377/
NM_000352.3(ABCC8):c.1158C>T	concordant	unreportable	unreportable	157681	0		2	SCV000192012;S	Likely benign;Likely benign	Genetic Services La	2014-09-11;2016-1	ABCC8	chr11	17474684	17474684	http://gnomad.b	0.003450910349	AFR	0.04763460159	20		22	0	https://www.ncbi.nlm.nih.gov/clinvar/variation/157681/
NM_000228.2(LAMB3):c.476G>A	concordant	unreportable	unreportable	290567	0		1	SCV000345141	Uncertain significance	EGL Genetic Diagn	6/30/2017	LAMB3	chr1	209807880	209807880	http://gnomad.b	0.0001735712566	SAS	0.0005198179861	0		0	0	https://www.ncbi.nlm.nih.gov/clinvar/variation/290567/
NM_138694.3(PKHD1):c.5725C>T	concordant	unreportable	unreportable	96411	0		2	SCV000291336;S	Benign;Likely benign	Invitae;Laboratory	2017-10-05;2018-0	PKHD1	chr6	51875133	51875133	http://gnomad.b	0.00154010386	AFR	0.01868790016	3		3	0	https://www.ncbi.nlm.nih.gov/clinvar/variation/96411/
NM_000053.3(ATP7B):c.3422C>G	concordant	unreportable	unreportable	312380	0		1	SCV000384653	Uncertain significance	Illumina Clinical Se	10/18/2016	ATP7B	chr13	52515351	52515351	http://gnomad.b	1.96E-05	ASJ	0.005177659914	0		0	0	https://www.ncbi.nlm.nih.gov/clinvar/variation/312380/
NM_000053.3(ATP7B):c.1278C>T	concordant	unreportable	unreportable	312391	0		2	SCV000384677;S	Uncertain significance;Unc	Illumina Clinical Se	2016-10-18;2018-0	ATP7B	chr13	52548078	52548078	http://gnomad.b	0.001487126535	NFE	0.001884859987	0		0	0	https://www.ncbi.nlm.nih.gov/clinvar/variation/312391/
NM_001042432.1(CLN3):c.1230G>A	concordant	unreportable	unreportable	136785	0		1	SCV000628925	Likely benign	Invitae	10/5/2017	CLN3	chr16	28488924	28488924	http://gnomad.b	0.0003722139508	FIN	0.0008142589941	0		0	0	https://www.ncbi.nlm.nih.gov/clinvar/variation/136785/
NM_017739.3(POMGNT1):c.1738C>A	concordant	unreportable	unreportable	500726	0		1	SCV000706795	Uncertain significance	EGL Genetic Diagn	6/30/2017	POMGNT1	chr1	46655573	46655573		1.12E-05					0	0	https://www.ncbi.nlm.nih.gov/clinvar/variation/500726/
NM_000023.2(SGCA):c.690G>C	concordant	unreportable	unreportable	198032	0		2	SCV000231456;S	Uncertain significance;Likel	EGL Genetic Diagn	2017-06-30;2017-1	SGCA	chr17	48246558	48246558	http://gnomad.b	0.0005821047565	NFE	0.001012020046	0		1	0	https://www.ncbi.nlm.nih.gov/clinvar/variation/198032/
NM_138694.3(PKHD1):c.4105C>T	concordant	unreportable	unreportable	288588	0		1	SCV000342750	Uncertain significance	EGL Genetic Diagn	6/30/2017	PKHD1	chr6	51890503	51890503	http://gnomad.b	3.89E-05	AMR	0.0002084699954	0		0	0	https://www.ncbi.nlm.nih.gov/clinvar/variation/288588/
NM_000492.3(CFTR):c.3558A>G	concordant	unreportable	unreportable	35869	0		3	SCV000603064;S	Likely benign;Uncertain sig	ARUP Laboratories	2017-06-30;2017-0	CFTR	chr7	117267665	117267665	http://gnomad.b	0.0003004298458	NFE	0.0004141080135	0		0	0	https://www.ncbi.nlm.nih.gov/clinvar/variation/35869/
NM_000232.4(SGCB):c.151C>T	concordant	unreportable	unreportable	92659	0		1	SCV000110256	Uncertain significance	EGL Genetic Diagn	6/30/2017	SGCB	chr4	52899689	52899689	http://gnomad.b	0.0003974229084	ASJ	0.001015429967	0		0	0	https://www.ncbi.nlm.nih.gov/clinvar/variation/92659/
NM_004937.2(CTNS):c.356G>A	concordant	unreportable	unreportable	198524	0		2	SCV000232216;S	Benign;Uncertain significar	EGL Genetic Diagn	2017-06-30;2016-1	CTNS	chr17	3558541	3558541	http://gnomad.b	0.001505220864	SAS	0.02319540083	9		7	0	https://www.ncbi.nlm.nih.gov/clinvar/variation/198524/
NM_001164277.1(SLC37A4):c.991A>G	concordant	unreportable	unreportable	215181	0		1	SCV000252292	Uncertain significance	GeneDx	11/28/2017	SLC37A4	chr11	118896033	118896033	http://gnomad.b	0.000550356195	SAS	0.008777240291	3		2	0	https://www.ncbi.nlm.nih.gov/clinvar/variation/215181/
NM_000155.3(GALT):c.498T>C	concordant	unreportable	unreportable	36147	0		1	SCV000603790	Likely benign	ARUP Laboratories	6/30/2017	GALT	chr9	34647949	34647949	http://gnomad.b	0.0003408147672	FIN	0.001255610026	0		0	0	https://www.ncbi.nlm.nih.gov/clinvar/variation/36147/
NM_004937.2(CTNS):c.970G>A	concordant	unreportable	unreportable	322844	0		1	SCV000402241	Uncertain significance	Illumina Clinical Se	10/18/2016	CTNS	chr17	3563269	3563269	http://gnomad.b	0.0006014957392	FIN	0.00130080001	0		1	0	https://www.ncbi.nlm.nih.gov/clinvar/variation/322844/
NM_000382.2(ALDH3A2):c.-3G>A	concordant	unreportable	unreportable	322206	0		1	SCV000401248	Uncertain significance	Illumina Clinical Se	10/18/2016	ALDH3A2	chr17	19552282	19552282	http://gnomad.b	0.0002603335629	SAS	0.002530480037	0		0	0	https://www.ncbi.nlm.nih.gov/clinvar/variation/322206/
NM_005476.5(GNE):c.1442G>A	concordant	unreportable	unreportable	451929	0		3	SCV000706495;S	Uncertain significance;Unc	EGL Genetic Diagn	2017-06-30;2017-1	GNE	chr9	36222965	36222965	http://gnomad.b	0.0002717543565	AFR	0.0001960270019	0		0	0	https://www.ncbi.nlm.nih.gov/clinvar/variation/451929/
NM_000051.3(ATM):c.2922-21T>G	concordant	unreportable	unreportable	136438	0		1	SCV000682089	Benign	Color Genomics, In	10/26/2017	ATM	chr11	108141957	108141957		0.0005318793441					0	0	https://www.ncbi.nlm.nih.gov/clinvar/variation/136438/
NM_000060.2(BTD):c.1284C>T	concordant	unreportable	unreportable	38593	0		1	SCV000630322	Benign	Invitae	10/5/2017	BTD	chr3	15686647	15686647	http://gnomad.b	0.003586688886	AFR	0.03836100176	14		19	0	https://www.ncbi.nlm.nih.gov/clinvar/variation/38593/
NM_000288.3(PEX7):c.86C>T	concordant	unreportable	unreportable	498141	0		1	SCV000703010	Uncertain significance	EGL Genetic Diagn	6/30/2017	PEX7	chr6	137143889	137143889	http://gnomad.b	3.61E-05	NFE	6.08E-05	0		0	0	https://www.ncbi.nlm.nih.gov/clinvar/variation/498141/
NM_033056.3(PCDH15):c.5550C>A	concordant	unreportable	unreportable	46502	0		1	SCV000528426	Likely benign	GeneDx	11/28/2017	PCDH15	chr10	55581936	55581936	http://gnomad.b	0.001452965077	AFR	0.01502879988	1		4	0	https://www.ncbi.nlm.nih.gov/clinvar/variation/46502/
NM_000060.2(BTD):c.262C>G	concordant	unreportable	unreportable	385308	0		1	SCV000526539	Uncertain significance	GeneDx	11/28/2017	BTD	chr3	15677148	15677148	http://gnomad.b	0.001011999839	FIN	0.009506730363	0		1	0	https://www.ncbi.nlm.nih.gov/clinvar/variation/385308/
NM_014363.4(SACS):c.2681C>T	concordant	unreportable	unreportable	311554	0		2	SCV000576741;S	Uncertain significance;Unc	GeneDx;Illumina C	2017-11-28;2016-1	SACS	chr13	23915334	23915334	http://gnomad.b	0.0003734705492	ASJ	0.003452480072	0		0	0	https://www.ncbi.nlm.nih.gov/clinvar/variation/311554/
NM_014363.4(SACS):c.6051G>C	concordant	unreportable	unreportable	448212	0		1	SCV000614967	Likely benign	Athena Diagnostics	8/17/2017	SACS	chr13	23911964	23911964	http://gnomad.b	0.001434601157	AFR	0.01864740066	5		2	0	https://www.ncbi.nlm.nih.gov/clinvar/variation/448212/
NM_000018.3(ACADVL):c.636C>T	concordant	unreportable	unreportable	324988	0		3	SCV000522360;S	Benign;Uncertain significar	GeneDx;Illumina C	2017-11-28;2016-1	ACADVL	chr17	7125284	7125284	http://gnomad.b	0.001724528516	AFR	0.01647699997	3		6	0	https://www.ncbi.nlm.nih.gov/clinvar/variation/324988/
NM_018941.3(CLN8):c.274C>T	concordant	unreportable	unreportable	128788	0		3	SCV000612850;S	Benign;Likely benign;Benig	Athena Diagnostics	2017-08-17;2014-0	CLN8	chr8	1719494	1719494	http://gnomad.b	0.003153682561	AFR	0.0377026014	12		15	0	https://www.ncbi.nlm.nih.gov/clinvar/variation/128788/
NM_000152.3(GAA):c.2417C>T	concordant	unreportable	unreportable	252467	0		3	SCV000296876;S	Uncertain significance;Unc	Division of Genomi	2016-01-06;2017-0	GAA	chr17	78091484	78091484	http://gnomad.b	0.0003529719123	AMR	0.001282509998	0		0	0	https://www.ncbi.nlm.nih.gov/clinvar/variation/252467/
NM_000060.2(BTD):c.880A>G	concordant	unreportable	unreportable	25047	0		1	SCV000441826	Likely benign	Illumina Clinical Se	10/18/2016	BTD	chr3	15686243	15686243	http://gnomad.b	0.003393515128	AFR	0.04240719974	16		23	0	https://www.ncbi.nlm.nih.gov/clinvar/variation/25047/
NM_000271.4(NPC1):c.2304C>T	concordant	unreportable	unreportable	326263	0		2	SCV000705867;S	Uncertain significance;Unc	EGL Genetic Diagn	2017-06-30;2016-1	NPC1	chr18	21121339	21121339	http://gnomad.b	0.0001143680593	EAS	0.0006958139711	0		0	0	https://www.ncbi.nlm.nih.gov/clinvar/variation/326263/
NM_000466.2(PEX1):c.3426C>T	concordant	unreportable	unreportable	286992	0		1	SCV000340615	Likely benign	EGL Genetic Diagn	6/30/2017	PEX1	chr7	92120598	92120598	http://gnomad.b	0.0003882827364	AFR	0.004966670182	0		0	0	https://www.ncbi.nlm.nih.gov/clinvar/variation/286992/
NM_000051.3(ATM):c.7618G>A	concordant	unreportable	unreportable	141579	0		2	SCV000292819;S	Uncertain significance;Unc	GeneDx;Invitae	2017-11-28;2017-1	ATM	chr11	108202273	108202273	http://gnomad.b	5.85E-05	NFE	8.07E-05	0		0	0	https://www.ncbi.nlm.nih.gov/clinvar/variation/141579/
NM_000396.3(CTSK):c.812G>A	concordant	unreportable	unreportable	292581	0		1	SCV000348276	Uncertain significance	Illumina Clinical Se	10/18/2016	CTSK	chr1	150771722	150771722	http://gnomad.b	0.0002029971939	NFE	0.0003222459927	0		0	0	https://www.ncbi.nlm.nih.gov/clinvar/variation/292581/
NM_000018.3(ACADVL):c.1239A>G	concordant	unreportable	unreportable	474879	0		1	SCV000654922	Likely benign	Invitae	10/5/2017	ACADVL	chr17	7127019	7127019	http://gnomad.b	0.0003596307413	ASJ	0.003045690013	0		0	0	https://www.ncbi.nlm.nih.gov/clinvar/variation/474879/
NM_000642.2(AGL):c.4214A>G	concordant	unreportable	unreportable	456501	0		1	SCV000626748	Uncertain significance	Invitae	10/5/2017	AGL	chr1	100380997	100380997	http://gnomad.b	0.0001702512462	AFR	0.002092869952	0		0	0	https://www.ncbi.nlm.nih.gov/clinvar/variation/456501/
NM_000182.4(HADHA):c.1655C>T	concordant	unreportable	unreportable	335380	0		1	SCV000429511	Uncertain significance	Illumina Clinical Se	10/18/2016	HADHA	chr2	26417473	26417473	http://gnomad.b	0.0001289700341	NFE	0.0001432489953	0		0	0	https://www.ncbi.nlm.nih.gov/clinvar/variation/335380/
NM_006493.2(CLN5):c.852G>A	concordant	unreportable	unreportable	136792	0		2	SCV000230319;S	Likely benign;Benign	EGL Genetic Diagn	2017-06-30;2017-1	CLN5	chr13	77574732	77574732	http://gnomad.b	0.0004923434991	AFR	0.005816990044	0		0	0	https://www.ncbi.nlm.nih.gov/clinvar/variation/136792/
NM_000466.2(PEX1):c.637C>T	concordant	unreportable	unreportable	285000	0		1	SCV000337835	Likely benign	EGL Genetic Diagn	6/30/2017	PEX1	chr7	92147192	92147192	http://gnomad.b	0.0004160172176	AFR	0.006463150028	0		0	0	https://www.ncbi.nlm.nih.gov/clinvar/variation/285000/
NM_002225.3(IVD):c.244-18G>A	concordant	unreportable	unreportable	382073	0		1	SCV000521859	Benign	GeneDx	11/28/2017	IVD	chr15	40700120	40700120	http://gnomad.b	0.002586404981	AFR	0.03574230149	10		11	0	https://www.ncbi.nlm.nih.gov/clinvar/variation/382073/
NM_133647.1(SLC12A6):c.2106T>C	concordant	unreportable	unreportable	315611	0		1	SCV000390306	Uncertain significance	Illumina Clinical Se	10/18/2016	SLC12A6	chr15	34534359	34534359	http://gnomad.b	0.001206083163	EAS	0.01026210003	1		4	0	https://www.ncbi.nlm.nih.gov/clinvar/variation/315611/
NM_000051.3(ATM):c.609C>T	concordant	unreportable	unreportable	135764	0		2	SCV000682306;S	Benign;Benign	Color Genomics, In	2017-10-26;2017-1	ATM	chr11	108114792	108114792	http://gnomad.b	0.001459187311	NFE	0.002267959993	0		0	0	https://www.ncbi.nlm.nih.gov/clinvar/variation/135764/
NM_000152.3(GAA):c.2307G>C	concordant	unreportable	unreportable	325791	0		2	SCV000407294;S	Uncertain significance;Unc	Illumina Clinical Se	2016-10-18;2017-1	GAA	chr17	78090884	78090884	http://gnomad.b	6.39E-05	NFE	6.31E-05	0		0	0	https://www.ncbi.nlm.nih.gov/clinvar/variation/325791/
NM_000227.3(LAMA3):c.30C>T	concordant	unreportable	unreportable	326291	0		1	SCV000407903	Uncertain significance	Illumina Clinical Se	10/18/2016	LAMA3	chr18	21453038	21453038	http://gnomad.b	0.001248663147	NFE	0.001414580038	1		0	0	https://www.ncbi.nlm.nih.gov/clinvar/variation/326291/
NM_000352.3(ABCC8):c.3112G>A	concordant	unreportable	unreportable	303766	0		1	SCV000369296	Uncertain significance	Illumina Clinical Se	10/18/2016	ABCC8	chr11	17428485	17428485	http://gnomad.b	0.0002245543757	ASJ	0.001729049953	0		0	0	https://www.ncbi.nlm.nih.gov/clinvar/variation/303766/
NM_033056.3(PCDH15):c.4080G>A	concordant	unreportable	unreportable	46478	0		1	SCV000363150	Uncertain significance	Illumina Clinical Se	10/18/2016	PCDH15	chr10	55591197	55591197	http://gnomad.b	0.0002340173841	ASJ	0.0004060910142	0		0	0	https://www.ncbi.nlm.nih.gov/clinvar/variation/46478/
NM_024649.4(BBS1):c.1338C>T	concordant	unreportable	unreportable	285380	0		1	SCV000338375	Uncertain significance	EGL Genetic Diagn	6/30/2017	BBS1	chr11	66294277	66294277	http://gnomad.b	0.0001755559271	SAS	0.003183679888	0		0	0	https://www.ncbi.nlm.nih.gov/clinvar/variation/285380/
NM_004646.3(NPHS1):c.1638T>G	concordant	unreportable	unreportable	328869	0		2	SCV000411572;S	Uncertain significance;Likel	Illumina Clinical Se	2016-10-18;2018-0	NPHS1	chr19	36336690	36336690	http://gnomad.b	0.001892746238	NFE	0.002792390063	3		0	0	https://www.ncbi.nlm.nih.gov/clinvar/variation/328869/
NM_004543.4(NEB):c.3443C>T	concordant	unreportable	unreportable	331519	0		1	SCV000417017	Uncertain significance	Illumina Clinical Se	10/18/2016	NEB	chr2	152534514	152534514	http://gnomad.b	0.0001497330621	EAS	0.002030400094	0		0	0	https://www.ncbi.nlm.nih.gov/clinvar/variation/331519/
NM_000053.3(ATP7B):c.-122G>A	concordant	unreportable	unreportable	312407	0		1	SCV000384696	Uncertain significance	Illumina Clinical Se	10/18/2016	ATP7B	chr13	52585595	52585595		0.0004910323464					0	0	https://www.ncbi.nlm.nih.gov/clinvar/variation/312407/
NM_017890.4(VPS13B):c.6802G>T	concordant	reportable	reportable	279780	1		0	SCV000329306	Pathogenic	GeneDx	11/28/2017	VPS13B	chr8	100732642	100732642	http://gnomad.b	5.04E-05	ASJ	0.0003048779909	0		0	0	https://www.ncbi.nlm.nih.gov/clinvar/variation/279780/
NM_000152.3(GAA):c.2561G>A	concordant	unreportable	unreportable	286210	0		3	SCV000339549;S	Uncertain significance;Unc	EGL Genetic Diagn	2017-06-30;2017-1	GAA	chr17	78092071	78092071	http://gnomad.b	0.0006809300671	ASJ	0.00646970002	0		1	0	https://www.ncbi.nlm.nih.gov/clinvar/variation/286210/
NM_000152.3(GAA):c.2481+16G>A	concordant	unreportable	unreportable	92478	0		2	SCV000110011;S	Uncertain significance;Likel	EGL Genetic Diagn	2017-06-30;2017-1	GAA	chr17	78091564	78091564	http://gnomad.b	0.002431893097	NFE	0.003783120075	2		3	0	https://www.ncbi.nlm.nih.gov/clinvar/variation/92478/
NM_024649.4(BBS1):c.1744G>A	concordant	unreportable	unreportable	305473	0		1	SCV000373283	Uncertain significance	Illumina Clinical Se	10/18/2016	BBS1	chr11	66299470	66299470	http://gnomad.b	4.74E-05	AMR	0.0006409870111	0		0	0	https://www.ncbi.nlm.nih.gov/clinvar/variation/305473/
NM_000492.3(CFTR):c.1731C>T	concordant	unreportable	unreportable	35832	0		1	SCV000052138	Uncertain significance	Laboratory Corpora	11/20/2015	CFTR	chr7	117230458	117230458	http://gnomad.b	0.0001540665876	NFE	0.0003775889927	0		0	0	https://www.ncbi.nlm.nih.gov/clinvar/variation/35832/
NM_000492.3(CFTR):c.1052C>G	concordant	unreportable	unreportable	53174	0		3	SCV000705166;S	Uncertain significance;Unc	EGL Genetic Diagn	2017-06-30;2017-1	CFTR	chr7	117180336	117180336	http://gnomad.b	0.0003119848398	ASJ	0.0007115270128	0		0	0	https://www.ncbi.nlm.nih.gov/clinvar/variation/53174/
NM_000466.2(PEX1):c.1441G>A	concordant	unreportable	unreportable	360927	0		2	SCV000703303;S	Uncertain significance;Unc	EGL Genetic Diagn	2017-06-30;2016-1	PEX1	chr7	92140936	92140936	http://gnomad.b	0.0001525396464	ASJ	0.002234860091	1		0	0	https://www.ncbi.nlm.nih.gov/clinvar/variation/360927/
NM_000053.3(ATP7B):c.1122C>G	concordant	unreportable	unreportable	312395	0		1	SCV000384682	Uncertain significance	Illumina Clinical Se	10/18/2016	ATP7B	chr13	52548234	52548234	http://gnomad.b	0.0001711598465	EAS	0.0008116880199	0		0	0	https://www.ncbi.nlm.nih.gov/clinvar/variation/312395/
NM_014363.4(SACS):c.9286G>A	concordant	unreportable	unreportable	426329	0		1	SCV000576738	Uncertain significance	GeneDx	11/28/2017	SACS	chr13	23908729	23908729	http://gnomad.b	8.89E-06	NFE	8.99E-06	0		0	0	https://www.ncbi.nlm.nih.gov/clinvar/variation/426329/
NM_138694.3(PKHD1):c.7221T>C	concordant	unreportable	unreportable	357431	0		2	SCV000707653;S	Uncertain significance;Unc	EGL Genetic Diagn	2017-06-30;2016-1	PKHD1	chr6	51748020	51748020	http://gnomad.b	0.0001306586307	NFE	0.0002783809905	0		0	0	https://www.ncbi.nlm.nih.gov/clinvar/variation/357431/
NM_006493.2(CLN5):c.49G>C	concordant	unreportable	unreportable	205123	0		4	SCV000704084;S	Benign;Likely benign;Uncer	EGL Genetic Diagn	2017-06-30;2017-1	CLN5	chr13	77566135	77566135	http://gnomad.b	0.0008196400298	AFR	0.005036939867	0		0	0	https://www.ncbi.nlm.nih.gov/clinvar/variation/205123/
NM_006493.2(CLN5):c.22G>C	concordant	unreportable	unreportable	205122	0		1	SCV000240618	Uncertain significance	GeneDx	11/28/2017	CLN5	chr13	77566108	77566108	http://gnomad.b	0.0003664602181	ASJ	0.002077080077	0		0	0	https://www.ncbi.nlm.nih.gov/clinvar/variation/205122/
NM_006493.2(CLN5):c.716A>G	concordant	unreportable	unreportable	205147	0		2	SCV000240648;S	Uncertain significance;Unc	GeneDx;Invitae	2017-11-28;2017-1	CLN5	chr13	77574596	77574596	http://gnomad.b	9.23E-05	AMR	0.0001794370037	0		0	0	https://www.ncbi.nlm.nih.gov/clinvar/variation/205147/
NM_000383.3(AIRE):c.1083C>T	concordant	unreportable	unreportable	458612	0		1	SCV000629942	Benign	Invitae	10/5/2017	AIRE	chr21	45712272	45712272	http://gnomad.b	0.0004149793624	AFR	0.003825549968	1		0	0	https://www.ncbi.nlm.nih.gov/clinvar/variation/458612/
NM_000391.3(TPP1):c.1426-10A>G	concordant	unreportable	unreportable	137705	0		1	SCV000628901	Likely benign	Invitae	10/5/2017	TPP1	chr11	6636232	6636232	http://gnomad.b	0.0001790700668	NFE	0.0002327499969	0		0	0	https://www.ncbi.nlm.nih.gov/clinvar/variation/137705/
NM_000543.4(SMPD1):c.901G>A	concordant	unreportable	unreportable	305201	0		2	SCV000704670;S	Benign;Uncertain significar	EGL Genetic Diagn	2017-06-30;2016-1	SMPD1	chr11	6413196	6413196	http://gnomad.b	0.001115604186	AFR	0.01261110045	0		3	0	https://www.ncbi.nlm.nih.gov/clinvar/variation/305201/
NM_000051.3(ATM):c.3342G>A	concordant	unreportable	unreportable	135751	0		2	SCV000166100;S	Benign;Likely benign	Invitae;Laboratory	2017-10-05;2018-0	ATM	chr11	108150275	108150275	http://gnomad.b	0.001035911602	ASJ	0.009558670223	1		2	0	https://www.ncbi.nlm.nih.gov/clinvar/variation/135751/
NM_138694.3(PKHD1):c.2265G>A	concordant	unreportable	unreportable	284051	0		1	SCV000336516	Uncertain significance	EGL Genetic Diagn	6/30/2017	PKHD1	chr6	51914969	51914969	http://gnomad.b	0.0001223187181	ASJ	0.000305249996	0		0	0	https://www.ncbi.nlm.nih.gov/clinvar/variation/284051/
NM_000466.2(PEX1):c.939T>C	concordant	unreportable	unreportable	291249	0		1	SCV000345964	Uncertain significance	EGL Genetic Diagn	6/30/2017	PEX1	chr7	92146890	92146890	http://gnomad.b	0.0004687127318	ASJ	0.003250049893	3		0	0	https://www.ncbi.nlm.nih.gov/clinvar/variation/291249/
NM_133647.1(SLC12A6):c.940A>T	concordant	unreportable	unreportable	159899	0		1	SCV000390322	Uncertain significance	Illumina Clinical Se	10/18/2016	SLC12A6	chr15	34546727	34546727	http://gnomad.b	4.74E-05	ASJ	0.0004060910142	0		0	0	https://www.ncbi.nlm.nih.gov/clinvar/variation/159899/
NM_024685.3(BBS10):c.440A>G	concordant	unreportable	unreportable	406223	0		1	SCV000544766	Uncertain significance	Invitae	3/14/2017	BBS10	chr12	76741325	76741325	http://gnomad.b	0.0004112808461	ASJ	0.002863569884	0		1	0	https://www.ncbi.nlm.nih.gov/clinvar/variation/406223/
NM_001876.3(CPT1A):c.2142G>A	concordant	unreportable	unreportable	377755	0		1	SCV000512761	Likely benign	GeneDx	11/28/2017	CPT1A	chr11	68527693	68527693	http://gnomad.b	0.0006054461998	NFE	0.001285400009	0		0	0	https://www.ncbi.nlm.nih.gov/clinvar/variation/377755/
NM_138694.3(PKHD1):c.9402G>A	concordant	unreportable	unreportable	357418	0		3	SCV000700539;S	Uncertain significance;Unc	EGL Genetic Diagn	2017-06-30;2016-1	PKHD1	chr6	51613012	51613012	http://gnomad.b	0.0005976937362	ASJ	0.002034999896	0		1	0	https://www.ncbi.nlm.nih.gov/clinvar/variation/357418/
NM_000057.2(BLM):c.3960C>T	concordant	unreportable	unreportable	136519	0		2	SCV000394432;S	Uncertain significance;Beni	Illumina Clinical Se	2016-10-18;2017-1	BLM	chr15	91354520	91354520	http://gnomad.b	0.001446035295	AFR	0.01666449942	3		4	0	https://www.ncbi.nlm.nih.gov/clinvar/variation/136519/
NM_001164277.1(SLC37A4):c.377G>A	concordant	unreportable	unreportable	431869	0		1	SCV000589428	Uncertain significance	GeneDx	11/28/2017	SLC37A4	chr11	118898908	118898908	http://gnomad.b	0.0001564464311	NFE	0.000218924004	0		0	0	https://www.ncbi.nlm.nih.gov/clinvar/variation/431869/
NM_001042432.1(CLN3):c.1022G>A	concordant	unreportable	unreportable	205084	0		1	SCV000240578	Likely benign	GeneDx	11/28/2017	CLN3	chr16	28493460	28493460	http://gnomad.b	0.0002388835804	EAS	0.002507579979	0		0	0	https://www.ncbi.nlm.nih.gov/clinvar/variation/205084/
NM_000018.3(ACADVL):c.1473A>G	concordant	unreportable	unreportable	324994	0		1	SCV000406325	Uncertain significance	Illumina Clinical Se	10/18/2016	ACADVL	chr17	7127503	7127503	http://gnomad.b	0.0006824488871	AMR	0.001369780046	0		0	0	https://www.ncbi.nlm.nih.gov/clinvar/variation/324994/
NM_000518.4(HBB):c.316-45G>C	concordant	unreportable	unreportable	495995	0		1	SCV000697117	Uncertain significance	Laboratory Corpora	1/25/2018	HBB	chr11	5247001	5247001	http://gnomad.b	0.000921407444	EAS	0.01000580005	3		2	0	https://www.ncbi.nlm.nih.gov/clinvar/variation/495995/
NM_000152.3(GAA):c.615G>A	concordant	unreportable	unreportable	196223	0		1	SCV000228796	Uncertain significance	EGL Genetic Diagn	6/30/2017	GAA	chr17	78079616	78079616	http://gnomad.b	7.50E-05	SAS	0.0004223519936	0		0	0	https://www.ncbi.nlm.nih.gov/clinvar/variation/196223/
NM_000227.3(LAMA3):c.794T>C	concordant	unreportable	unreportable	326303	0		2	SCV000535966;S	Uncertain significance;Unc	GeneDx;Illumina C	2017-11-28;2016-1	LAMA3	chr18	21478709	21478709	http://gnomad.b	0.0003331634853	NFE	0.000466174999	0		0	0	https://www.ncbi.nlm.nih.gov/clinvar/variation/326303/
NM_000642.2(AGL):c.1028G>A	concordant	unreportable	unreportable	445557	0		1	SCV000626660	Uncertain significance	Invitae	10/5/2017	AGL	chr1	100340312	100340312	http://gnomad.b	0.000547036791	ASJ	0.0008123479784	0		2	0	https://www.ncbi.nlm.nih.gov/clinvar/variation/445557/
NM_024649.4(BBS1):c.1695+10G>A	concordant	unreportable	unreportable	305471	0		2	SCV000373281;S	Uncertain significance;Beni	Illumina Clinical Se	2016-10-18;2017-0	BBS1	chr11	66299223	66299223	http://gnomad.b	0.002003566851	SAS	0.02719119936	12		14	0	https://www.ncbi.nlm.nih.gov/clinvar/variation/305471/
NM_001360.2(DHCR7):c.729C>T	concordant	unreportable	unreportable	287363	0		1	SCV000341114	Uncertain significance	EGL Genetic Diagn	6/30/2017	DHCR7	chr11	71150027	71150027	http://gnomad.b	0.0002746424263	ASJ	0.001320600044	0		0	0	https://www.ncbi.nlm.nih.gov/clinvar/variation/287363/
NM_000391.3(TPP1):c.1049G>A	concordant	unreportable	unreportable	501669	0		1	SCV000708133	Uncertain significance	EGL Genetic Diagn	6/30/2017	TPP1	chr11	6637572	6637572	http://gnomad.b	5.60E-06	AFR	0.0001307360071	0		0	0	https://www.ncbi.nlm.nih.gov/clinvar/variation/501669/
NM_002485.4(NBN):c.45A>G	concordant	unreportable	unreportable	239199	0		3	SCV000685785;S	Likely benign;Likely benign	Color Genomics, In	2017-10-26;2017-1	NBN	chr8	90995076	90995076	http://gnomad.b	3.35E-05	SAS	0.0004549000005	0		0	0	https://www.ncbi.nlm.nih.gov/clinvar/variation/239199/
NM_000478.4(ALPL):c.1002C>T	concordant	unreportable	unreportable	290031	0		3	SCV000344507;S	Uncertain significance;Unc	EGL Genetic Diagn	2017-06-30;2017-1	ALPL	chr1	21902230	21902230	http://gnomad.b	0.0009578328187	ASJ	0.007818849757	0		0	0	https://www.ncbi.nlm.nih.gov/clinvar/variation/290031/
NM_000383.3(AIRE):c.63C>T	concordant	unreportable	unreportable	495855	0		1	SCV000696655	Likely benign	Laboratory Corpora	1/25/2018	AIRE	chr21	45705952	45705952	http://gnomad.b	0.002436959343	ASJ	0.005658950191	1		3	0	https://www.ncbi.nlm.nih.gov/clinvar/variation/495855/
NM_000383.3(AIRE):c.1476C>T	concordant	unreportable	unreportable	255971	0		1	SCV000629950	Benign	Invitae	10/5/2017	AIRE	chr21	45714359	45714359	http://gnomad.b	0.0008633798816	NFE	0.002058350015	0		1	0	https://www.ncbi.nlm.nih.gov/clinvar/variation/255971/
NM_000152.3(GAA):c.1722C>T	concordant	unreportable	unreportable	193975	0		1	SCV000225487	Uncertain significance	EGL Genetic Diagn	6/30/2017	GAA	chr17	78085867	78085867	http://gnomad.b	2.78E-06	SAS	0.0005847570137	0		0	0	https://www.ncbi.nlm.nih.gov/clinvar/variation/193975/
NM_000051.3(ATM):c.6382T>C	concordant	unreportable	unreportable	186642	0		5	SCV000602576;S	Likely benign;Likely benign	ARUP Laboratories	2017-06-30;2017-1	ATM	chr11	108190715	108190715	http://gnomad.b	0.0001364507218	EAS	0.0004059379862	1		0	0	https://www.ncbi.nlm.nih.gov/clinvar/variation/186642/
NM_000543.4(SMPD1):c.808G>A	concordant	unreportable	unreportable	305199	0		1	SCV000372931	Uncertain significance	Illumina Clinical Se	10/18/2016	SMPD1	chr11	6413103	6413103	http://gnomad.b	0.0004923882017	ASJ	0.004063390195	0		0	0	https://www.ncbi.nlm.nih.gov/clinvar/variation/305199/
NM_015166.3(MLC1):c.627C>T	concordant	unreportable	unreportable	342106	0		1	SCV000439237	Uncertain significance	Illumina Clinical Se	10/18/2016	MLC1	chr22	50512732	50512732	http://gnomad.b	0.001101925293	NFE	0.001345849945	1		0	0	https://www.ncbi.nlm.nih.gov/clinvar/variation/342106/
NM_000520.4(HEXA):c.1397A>G	concordant	unreportable	unreportable	93190	0		1	SCV000393831	Uncertain significance	Illumina Clinical Se	10/18/2016	HEXA	chr15	72638600	72638600	http://gnomad.b	0.0002206893961	ASJ	0.001118800021	0		0	0	https://www.ncbi.nlm.nih.gov/clinvar/variation/93190/
NM_000391.3(TPP1):c.1495C>G	concordant	unreportable	unreportable	502454	0		1	SCV000709191	Uncertain significance	EGL Genetic Diagn	6/30/2017	TPP1	chr11	6636153	6636153	http://gnomad.b	3.92E-05	EAS	5.80E-05	0		0	0	https://www.ncbi.nlm.nih.gov/clinvar/variation/502454/

Supplemental Table 2

NM_018941.3(CLN8):c.708C>T	concordant	unreportable	unreportable	377700	0		1	SCV000512652	Likely benign	GeneDx	11/28/2017	CLN8	chr8	1728580	1728580	http://gnomad.b	2.52E-05	AFR	0.0003267120046	0		0	0	https://www.ncbi.nlm.nih.gov/clinvar/variation/377700/
NM_000018.3(ACADVL):c.308A>G	concordant	unreportable	unreportable	254700	0		1	SCV000654950	Benign	Invitae	10/5/2017	ACADVL	chr17	7124115	7124115	http://gnomad.b	0.001832134564	AFR	0.01293780003	3		9	0	https://www.ncbi.nlm.nih.gov/clinvar/variation/254700/
NM_004543.4(NEB):c.8644G>A	concordant	unreportable	unreportable	198308	0		2	SCV000532338;S(	Uncertain significance;Unc	GeneDx;Invitae	2017-11-28;2017-1	NEB	chr2	152496910	152496910	http://gnomad.b	0.0004595256044	NFE	0.0006812600186	0		0	0	https://www.ncbi.nlm.nih.gov/clinvar/variation/198308/
NM_000098.2(CPT2):c.448A>G	concordant	unreportable	unreportable	374947	0		1	SCV000632611	Uncertain significance	Invitae	10/5/2017	CPT2	chr1	53675794	53675794	http://gnomad.b	0.0001846742738	NFE	0.0003222399973	0		0	0	https://www.ncbi.nlm.nih.gov/clinvar/variation/374947/
NM_000053.3(ATP7B):c.445G>A	concordant	unreportable	unreportable	495422	0		1	SCV000694466	Uncertain significance	Laboratory Corpora	1/25/2018	ATP7B	chr13	52548911	52548911	http://gnomad.b	0.0004264966666	ASJ	0.003557630116	0		0	0	https://www.ncbi.nlm.nih.gov/clinvar/variation/495422/
NM_000271.4(NPC1):c.467T>C	concordant	unreportable	unreportable	285856	0		1	SCV000339055	Uncertain significance	EGL Genetic Diagn	6/30/2017	NPC1	chr18	21141488	21141488	http://gnomad.b	8.29E-05	FIN	0.0004035870079	0		0	0	https://www.ncbi.nlm.nih.gov/clinvar/variation/285856/
NM_000391.3(TPP1):c.796C>T	concordant	unreportable	unreportable	207600	0		4	SCV000340903;S(	Uncertain significance;Unc	EGL Genetic Diagn	2017-06-30;2017-1	TPP1	chr11	6637982	6637982	http://gnomad.b	0.0002070497647	SAS	0.0004872980062	2		0	0	https://www.ncbi.nlm.nih.gov/clinvar/variation/207600/
NM_002485.4(NBN):c.1434C>T	concordant	unreportable	unreportable	411764	0		1	SCV000553082	Uncertain significance	Invitae	3/14/2017	NBN	chr8	90965883	90965883	http://gnomad.b	2.79E-06	ASJ	0.0001018539988	0		0	0	https://www.ncbi.nlm.nih.gov/clinvar/variation/411764/
NM_014363.4(SACS):c.10611A>G	concordant	unreportable	unreportable	240890	0		4	SCV000614921;S(	Likely benign;Likely benign	Athena Diagnostics	2017-08-17;2017-0	SACS	chr13	23907404	23907404	http://gnomad.b	0.001025561984	AFR	0.006714529824	2		0	0	https://www.ncbi.nlm.nih.gov/clinvar/variation/240890/
NM_001360.2(DHCR7):c.1273G>A	concordant	unreportable	unreportable	305953	0		2	SCV000706982;S(	Uncertain significance;Unc	EGL Genetic Diagn	2017-06-30;2016-1	DHCR7	chr11	71146576	71146576	http://gnomad.b	0.0001804023781	SAS	0.001625910052	0		0	0	https://www.ncbi.nlm.nih.gov/clinvar/variation/305953/
NM_014363.4(SACS):c.10821C>A	concordant	unreportable	unreportable	240892	0		2	SCV000614924;S(	Benign;Benign	Athena Diagnostics	2017-08-17;2017-0	SACS	chr13	23907194	23907194	http://gnomad.b	0.001075950868	AFR	0.0147270998	2		5	0	https://www.ncbi.nlm.nih.gov/clinvar/variation/240892/
NM_000642.2(AGL):c.1810T>G	concordant	unreportable	unreportable	456459	0		1	SCV000626679	Likely benign	Invitae	10/5/2017	AGL	chr1	100346262	100346262	http://gnomad.b	0.0002651453834	AFR	0.003528950037	0		0	0	https://www.ncbi.nlm.nih.gov/clinvar/variation/456459/
NM_000487.5(ARSA):c.1334G>T	concordant	unreportable	unreportable	198733	0		1	SCV000439428	Uncertain significance	Illumina Clinical Se	10/18/2016	ARSA	chr22	51063769	51063769	http://gnomad.b	0.000123810435	AMR	0.0002092429932	0		0	0	https://www.ncbi.nlm.nih.gov/clinvar/variation/198733/
NM_017890.4(VPS13B):c.2596G>A	concordant	unreportable	unreportable	235550	0		2	SCV000597881;S(	Uncertain significance;Unc	Genetic Services La	2017-07-05;2016-1	VPS13B	chr8	100286506	100286506	http://gnomad.b	0.0007139057644	NFE	0.001280330005	0		0	0	https://www.ncbi.nlm.nih.gov/clinvar/variation/235550/
NM_000027.3(AGA):c.762C>T	concordant	unreportable	unreportable	290584	0		1	SCV000345161	Uncertain significance	EGL Genetic Diagn	6/30/2017	AGA	chr4	178355580	178355580	http://gnomad.b	0.001170828294	ASJ	0.002335499972	1		0	0	https://www.ncbi.nlm.nih.gov/clinvar/variation/290584/
NM_014363.4(SACS):c.12597A>G	concordant	unreportable	unreportable	240894	0		3	SCV000614933;S(	Benign;Uncertain significar	Athena Diagnostics	2017-08-17;2016-1	SACS	chr13	23905418	23905418	http://gnomad.b	0.001099663284	AFR	0.01483849995	2		5	0	https://www.ncbi.nlm.nih.gov/clinvar/variation/240894/
NM_000051.3(ATM):c.2494C>T	concordant	unreportable	unreportable	140867	0		4	SCV000209704;S(	Uncertain significance;Unc	GeneDx;Genetic Se	2017-02-15;2017-0	ATM	chr11	108137925	108137925	http://gnomad.b	0.0002394849403	AFR	0.0003921570024	0		0	0	https://www.ncbi.nlm.nih.gov/clinvar/variation/140867/
NM_000152.3(GAA):c.576G>C	concordant	unreportable	unreportable	286219	0		1	SCV000339560	Uncertain significance	EGL Genetic Diagn	6/30/2017	GAA	chr17	78079577	78079577	http://gnomad.b	3.34E-05	NFE	6.27E-05	0		0	0	https://www.ncbi.nlm.nih.gov/clinvar/variation/286219/
NM_000352.3(ABCC8):c.4542C>A	concordant	unreportable	unreportable	255932	0		2	SCV000612221;S(	Benign;Likely benign	Athena Diagnostics	2017-08-17;2016-1	ABCC8	chr11	17415816	17415816	http://gnomad.b	0.001447988561	AFR	0.01461909991	5		3	0	https://www.ncbi.nlm.nih.gov/clinvar/variation/255932/
NM_000018.3(ACADVL):c.1839G>A	concordant	unreportable	unreportable	254698	0		2	SCV000523167;S(	Likely benign;Uncertain sig	GeneDx;Illumina C	2017-11-28;2016-1	ACADVL	chr17	7128287	7128287	http://gnomad.b	0.0007192614827	AFR	0.007190479897	0		1	0	https://www.ncbi.nlm.nih.gov/clinvar/variation/254698/
NM_000492.3(CFTR):c.-4G>C	concordant	unreportable	unreportable	53157	0		3	SCV000224229;S(	Likely benign;Likely benign	EGL Genetic Diagn	2017-06-30;2017-1	CFTR	chr7	117120145	117120145	http://gnomad.b	8.28E-05	NFE	0.000170254003	0		0	0	https://www.ncbi.nlm.nih.gov/clinvar/variation/53157/
NM_001360.2(DHCR7):c.1410G>A	concordant	unreportable	unreportable	501850	0		1	SCV000708359	Uncertain significance	EGL Genetic Diagn	6/30/2017	DHCR7	chr11	71146439	71146439	http://gnomad.b	5.65E-05	AFR	0.0002036660007	0		0	0	https://www.ncbi.nlm.nih.gov/clinvar/variation/501850/
NM_000051.3(ATM):c.1272T>C	concordant	unreportable	unreportable	135732	0		4	SCV000537415;S(	Likely benign;Uncertain sig	Color Genomics, In	2017-01-26;2016-1	ATM	chr11	108121464	108121464	http://gnomad.b	0.00054023347	NFE	0.0004319649888	0		1	0	https://www.ncbi.nlm.nih.gov/clinvar/variation/135732/
NM_000057.2(BLM):c.403G>T	concordant	unreportable	unreportable	127508	0		1	SCV000543342	Uncertain significance	Invitae	10/5/2017	BLM	chr15	91292901	91292901	http://gnomad.b	0.0001022689435	ASJ	0.0001015430025	0		0	0	https://www.ncbi.nlm.nih.gov/clinvar/variation/127508/
NM_000051.3(ATM):c.2040C>T	concordant	unreportable	unreportable	136436	0		2	SCV000682022;S(	Likely benign;Likely benign	Color Genomics, In	2017-10-26;2017-1	ATM	chr11	108124682	108124682		4.73E-05					0	0	https://www.ncbi.nlm.nih.gov/clinvar/variation/136436/
NM_138694.3(PKHD1):c.10973T>C	concordant	unreportable	unreportable	406893	0		1	SCV000545852	Uncertain significance	Invitae	3/14/2017	PKHD1	chr6	51523951	51523951	http://gnomad.b	0.0001139788055	FIN	0.0004484709934	0		0	0	https://www.ncbi.nlm.nih.gov/clinvar/variation/406893/
NM_000023.2(SGCA):c.680C>G	concordant	unreportable	unreportable	198033	0		1	SCV000231458	Uncertain significance	EGL Genetic Diagn	6/30/2017	SGCA	chr17	48246548	48246548	http://gnomad.b	0.0003638154728	NFE	0.0004118760116	0		0	0	https://www.ncbi.nlm.nih.gov/clinvar/variation/198033/
NM_000642.2(AGL):c.2657C>T	concordant	unreportable	unreportable	456476	0		1	SCV000626704	Uncertain significance	Invitae	10/5/2017	AGL	chr1	100350235	100350235	http://gnomad.b	0.0001088491574	AMR	0.0003581019992	0		0	0	https://www.ncbi.nlm.nih.gov/clinvar/variation/456476/
NM_000050.4(ASS1):c.1101G>A	concordant	unreportable	unreportable	287511	0		2	SCV000341300;S(	Likely benign;Uncertain sig	EGL Genetic Diagn	2017-06-30;2016-1	ASS1	chr9	133370384	133370384	http://gnomad.b	0.00101098999	FIN	0.002643289976	0		1	0	https://www.ncbi.nlm.nih.gov/clinvar/variation/287511/
NM_000053.3(ATP7B):c.3885C>T	concordant	unreportable	unreportable	312378	0		2	SCV000535345;S(	Likely benign;Uncertain sig	GeneDx;Illumina C	2017-11-28;2016-1	ATP7B	chr13	52511630	52511630	http://gnomad.b	0.0001459067544	ASJ	0.0001015230009	0		0	0	https://www.ncbi.nlm.nih.gov/clinvar/variation/312378/
NM_025136.3(OPA3):c.412G>A	concordant	unreportable	unreportable	280981	0		2	SCV000330981;S(	Benign;Likely benign	EGL Genetic Diagn	2017-06-30;2016-1	OPA3	chr19	46056900	46056900	http://gnomad.b	0.001261957957	AFR	0.01538799983	2		0	0	https://www.ncbi.nlm.nih.gov/clinvar/variation/280981/
NM_000057.2(BLM):c.2638G>C	concordant	unreportable	unreportable	127489	0		1	SCV000623281	Uncertain significance	Invitae	10/5/2017	BLM	chr15	91326134	91326134	http://gnomad.b	6.42E-05	NFE	0.0001075539985	0		0	0	https://www.ncbi.nlm.nih.gov/clinvar/variation/127489/
NM_001079802.1(FKTN):c.167G>A	concordant	unreportable	unreportable	93518	0		4	SCV000320317;S(	Uncertain significance;Likel	Ambry Genetics;EG	2017-11-14;2017-0	FKTN	chr9	108363427	108363427	http://gnomad.b	0.0004715944146	AFR	0.007110579871	0		0	0	https://www.ncbi.nlm.nih.gov/clinvar/variation/93518/
NM_000136.2(FANCC):c.1330-3C>T	concordant	unreportable	unreportable	210976	0		3	SCV000247341;S(	Benign;Benign;Benign	Genetic Services La	2017-10-04;2017-1	FANCC	chr9	97869554	97869554	http://gnomad.b	0.002156945746	AFR	0.02657889947	8		14	0	https://www.ncbi.nlm.nih.gov/clinvar/variation/210976/
NM_000478.4(ALPL):c.757G>A	concordant	unreportable	unreportable	434139	0		1	SCV000593126	Uncertain significance	Genetic Services La	7/5/2017	ALPL	chr1	21894705	21894705	http://gnomad.b	7.04E-05	ASJ	0.0001015230009	0		0	0	https://www.ncbi.nlm.nih.gov/clinvar/variation/434139/
NM_017890.4(VPS13B):c.2356A>G	concordant	unreportable	unreportable	430204	0		1	SCV000582951	Uncertain significance	GeneDx	11/28/2017	VPS13B	chr8	100205126	100205126	http://gnomad.b	0.0003191578712	ASJ	0.0004064209934	0		0	0	https://www.ncbi.nlm.nih.gov/clinvar/variation/430204/
NM_000466.2(PEX1):c.1142C>A	concordant	unreportable	unreportable	235320	0		1	SCV000340768	Benign	EGL Genetic Diagn	6/30/2017	PEX1	chr7	92146687	92146687	http://gnomad.b	0.002191024013	AFR	0.02549020015	3		13	0	https://www.ncbi.nlm.nih.gov/clinvar/variation/235320/
NM_020533.2(MCOLN1):c.1077C>T	concordant	unreportable	unreportable	459178	0		1	SCV000630739	Benign	Invitae	10/5/2017	MCOLN1	chr19	7593799	7593799	http://gnomad.b	0.0005494923737	SAS	0.005815459881	0		1	0	https://www.ncbi.nlm.nih.gov/clinvar/variation/459178/
NM_014363.4(SACS):c.11688G>A	concordant	unreportable	unreportable	240893	0		3	SCV000614929;S(	Benign;Uncertain significar	Athena Diagnostics	2017-08-17;2016-1	SACS	chr13	23906327	23906327	http://gnomad.b	0.001102627336	AFR	0.01477119979	2		5	0	https://www.ncbi.nlm.nih.gov/clinvar/variation/240893/
NM_138694.3(PKHD1):c.11224G>A	concordant	unreportable	unreportable	414168	0		1	SCV000557645	Benign	Invitae	3/14/2017	PKHD1	chr6	51513969	51513969	http://gnomad.b	0.0003419364165	SAS	0.003671220038	1		1	0	https://www.ncbi.nlm.nih.gov/clinvar/variation/414168/
NM_006493.2(CLN5):c.144C>T	concordant	unreportable	unreportable	136793	0		1	SCV000384721	Uncertain significance	Illumina Clinical Se	10/18/2016	CLN5	chr13	77566230	77566230	http://gnomad.b	0.0005454942178	ASJ	0.00471204007	0		0	0	https://www.ncbi.nlm.nih.gov/clinvar/variation/136793/
NM_014363.4(SACS):c.7384C>T	concordant	unreportable	unreportable	448218	0		1	SCV000614976	Likely benign	Athena Diagnostics	8/17/2017	SACS	chr13	23910631	23910631	http://gnomad.b	0.001390140378	AFR	0.01621980034	0		6	0	https://www.ncbi.nlm.nih.gov/clinvar/variation/448218/
NM_000136.2(FANCC):c.875G>A	concordant	unreportable	unreportable	219684	0		2	SCV000278973;S(	Uncertain significance;Unc	GeneDx;Invitae	2017-11-28;2017-1	FANCC	chr9	97888832	97888832	http://gnomad.b	1.43E-05	FIN	4.48E-05	0		0	0	https://www.ncbi.nlm.nih.gov/clinvar/variation/219684/
NM_005562.2(LAMC2):c.2736T>C	concordant	unreportable	unreportable	294012	0		1	SCV000351686	Uncertain significance	Illumina Clinical Se	10/18/2016	LAMC2	chr1	183206621	183206621	http://gnomad.b	0.001268260999	FIN	0.004844789859	2		0	0	https://www.ncbi.nlm.nih.gov/clinvar/variation/294012/
NM_000492.3(CFTR):c.1429C>T	concordant	unreportable	unreportable	455762	0		1	SCV000625726	Uncertain significance	Invitae	10/5/2017	CFTR	chr7	117199554	117199554	http://gnomad.b	3.08E-05	AFR	0.0001308219944	0		0	0	https://www.ncbi.nlm.nih.gov/clinvar/variation/455762/
NM_003060.3(SLC22A5):c.523G>A	concordant	unreportable	unreportable	285602	0		4	SCV000605135;S(	Likely benign;Uncertain sig	ARUP Laboratories	2017-06-30;2017-0	SLC22A5	chr5	131719864	131719864	http://gnomad.b	8.19E-05	EAS	0.0002898889943	0		0	0	https://www.ncbi.nlm.nih.gov/clinvar/variation/285602/
NM_017890.4(VPS13B):c.560G>A	concordant	unreportable	unreportable	437253	0		1	SCV000597911	Uncertain significance	Genetic Services La	7/5/2017	VPS13B	chr8	100115328	100115328	http://gnomad.b	0.0001679778269	SAS	0.0004223250144	0		0	0	https://www.ncbi.nlm.nih.gov/clinvar/variation/437253/
NM_000243.2(MEFV):c.372C>T	concordant	unreportable	unreportable	97519	0		3	SCV000604192;S(	Benign;Uncertain significar	ARUP Laboratories	2017-06-30;2016-1	MEFV	chr16	3304696	3304696	http://gnomad.b	0.0003806325217	NFE	0.00054326799	0		0	0	https://www.ncbi.nlm.nih.gov/clinvar/variation/97519/
NM_001360.2(DHCR7):c.208G>A	concordant	unreportable	unreportable	166989	0		1	SCV000202605	Likely benign	EGL Genetic Diagn	6/30/2017	DHCR7	chr11	71155152	71155152	http://gnomad.b	1.62E-05	AFR	0.004989210051	1		0	0	https://www.ncbi.nlm.nih.gov/clinvar/variation/166989/
NM_017890.4(VPS13B):c.1559A>G	concordant	unreportable	unreportable	193906	0		3	SCV000297277;S(	Uncertain significance;Unc	Division of Genomi	2016-01-06;2017-0	VPS13B	chr8	100147957	100147957	http://gnomad.b	0.0006803101991	AMR	0.001728659961	0		0	0	https://www.ncbi.nlm.nih.gov/clinvar/variation/193906/
NM_000051.3(ATM):c.5144T>C	concordant	unreportable	unreportable	236730	0		3	SCV000682250;S(	Uncertain significance;Unc	Color Genomics, In	2017-10-26;2017-1	ATM	chr11	108170579	108170579	http://gnomad.b	2.78E-06	NFE	8.98E-06	0		0	0	https://www.ncbi.nlm.nih.gov/clinvar/variation/236730/
NM_138694.3(PKHD1):c.5112C>T	concordant	unreportable	unreportable	357437	0		1	SCV000464084	Uncertain significance	Illumina Clinical Se	10/18/2016	PKHD1	chr6	51889496	51889496	http://gnomad.b	0.0007422522212	SAS	0.00526282005	0		6	0	https://www.ncbi.nlm.nih.gov/clinvar/variation/357437/
NM_138694.3(PKHD1):c.2934G>A	concordant	unreportable	unreportable	500387	0		1	SCV000706302	Likely benign	EGL Genetic Diagn	6/30/2017	PKHD1	chr6	51907820	51907820	http://gnomad.b	0.0005142946102	SAS	0.005490220152	1		0	0	https://www.ncbi.nlm.nih.gov/clinvar/variation/500387/
NM_000051.3(ATM):c.3153+20T>C	concordant	unreportable	unreportable	136439	0		1	SCV000682107	Likely benign	Color Genomics, In	10/26/2017	ATM	chr11	108143354	108143354	http://gnomad.b	0.0002673320264	ASJ	0.002846109914	0		0	0	https://www.ncbi.nlm.nih.gov/clinvar/variation/136439/
NM_000391.3(TPP1):c.1497T>C	concordant	unreportable	unreportable	137706	0		2	SCV000345177;S(	Likely benign;Benign	EGL Genetic Diagn	2017-06-30;2017-1	TPP1	chr11	6636151	6636151	http://gnomad.b	0.0004672609555	AFR	0.004639919847	0		1	0	https://www.ncbi.nlm.nih.gov/clinvar/variation/137706/
NM_000303.2(PMM2):c.90C>G	concordant	unreportable	unreportable	285859	0		2	SCV000339058;S(	Uncertain significance;Unc	EGL Genetic Diagn	2017-06-30;2016-1	PMM2	chr16	8895679	8895679	http://gnomad.b	0.0003758540239	SAS	0.003251659917	1		1	0	https://www.ncbi.nlm.nih.gov/clinvar/variation/285859/
NM_000391.3(TPP1):c.42C>A	concordant	unreportable	unreportable	137707	0		1	SCV000559665	Benign	Invitae	10/5/2017	TPP1	chr11	6640474	6640474	http://gnomad.b	0.001038046793	AFR	0.01293780003	2		1	0	https://www.ncbi.nlm.nih.gov/clinvar/variation/137707/
NM_000396.3(CTSK):c.675A>G	concordant	unreportable	unreportable	292583	0		1	SCV000348278	Uncertain significance	Illumina Clinical Se	10/18/2016	CTSK	chr1	150772129	150772129	http://gnomad.b	0.002934503552	NFE	0.003840510035	2		4	0	https://www.ncbi.nlm.nih.gov/clinvar/variation/292583/
NM_004004.5(GJB2):c.-45C>A	concordant	unreportable	unreportable	44720	0		2	SCV000383067;S(	Likely benign;Likely benign	Illumina Clinical Se	2016-10-18;2018-0	GJB2	chr13	20766944	20766944		0.002422450838					1	0	https://www.ncbi.nlm.nih.gov/clinvar/variation/44720/
NM_000543.4(SMPD1):c.719G>A	concordant	unreportable	unreportable	459634	0		2	SCV000704669;S(	Benign;Benign	EGL Genetic Diagn	2017-06-30;2017-1	SMPD1	chr11	6413014	6413014	http://gnomad.b	0.001917221694	AFR	0.02257340029	0		7	0	https://www.ncbi.nlm.nih.gov/clinvar/variation/459634/
NM_000071.2(CBS):c.1059G>A	concordant	unreportable	unreportable	212827	0		2	SCV000317718;S(	Likely benign;Benign	Ambry Genetics;In	2017-11-14;2017-1	CBS	chr21	44480637	44480637	http://gnomad.b	0.0005933332958	AFR	0.005600110162	0		0	0	https://www.ncbi.nlm.nih.gov/clinvar/variation/212827/
NM_000051.3(ATM):c.7313C>T	concordant	unreportable	unreportable	127440	0		4	SCV000611370;S(	Uncertain significance;Unc	Fulgent Genetics,F	2017-05-23;2017-1	ATM	chr11	108200946	108200946	http://gnomad.b	0.0002004990198	AFR	0.001898649964	0		0	0	https://www.ncbi.nlm.nih.gov/clinvar/variation/127440/
NM_014363.4(SACS):c.1033C>T	concordant	unreportable	unreportable	411689	0		2	SCV000619216;S(	Uncertain significance;Unc	GeneDx;Invitae	2017-11-28;2017-0	SACS	chr13	23929718	23929718	http://gnomad.b	7.11E-05	SAS	0.0001949190046	0		0	0	https://www.ncbi.nlm.nih.gov/clinvar/variation/411689/
NM_000152.3(GAA):c.-2C>T	concordant	unreportable	unreportable	283494	0		1	SCV000335646	Likely benign	EGL Genetic Diagn	6/30/2017	GAA	chr17	78078384	78078384	http://gnomad.b	0.0008282333061	SAS	0.007955219597	5		4	0	https://www.ncbi.nlm.nih.gov/clinvar/variation/283494/
NM_000391.3(TPP1):c.866C>A	concordant	unreportable	unreportable	457948	0		1	SCV000628920	Uncertain significance	Invitae	10/5/2017	TPP1	chr11	6637912	6637912		2.80E-06					0	0	https://www.ncbi.nlm.nih.gov/clinvar/variation/457948/
NM_001360.2(DHCR7):c.198C>T	concordant	unreportable	unreportable	290442	0		1	SCV000345001	Uncertain significance	EGL Genetic Diagn	6/30/2017	DHCR7	chr11	71155162	71155162		1.88E-05					0	0	https://www.ncbi.nlm.nih.gov/clinvar/variation/290442/
NM_000023.2(SGCA):c.313-10C>T	concordant	unreportable	unreportable	197095	0		2	SCV000229994;S(	Uncertain significance;Likel	EGL Genetic Diagn	2017-06-30;2017-1	SGCA	chr17	48245298	48245298	http://gnomad.b	1.68E-05	AMR	8.93E-05	0		0	0	https://www.ncbi.nlm.nih.gov/clinvar/variation/197095/
NM_000108.3(DLD):c.788G>A	concordant	unreportable	unreportable	203681	0		1	SCV000238807	Uncertain significance	GeneDx	11/28/2017	DLD	chr7	107556054	107556054	http://gnomad.b	0.0008022306686	AMR	0.001730620046	0		1	0	https://www.ncbi.nlm.nih.gov/clinvar/variation/203681/
NM_000182.4(HADHA):c.2147-8C>G	concordant	unreportable	unreportable	335373	0		1	SCV000429494	Uncertain significance	Illumina Clinical Se	10/18/2016	HADHA	chr2	26414272	26414272	http://gnomad.b	0.0001457922125	NFE	0.0002597220009	0		0	0	https://www.ncbi.nlm.nih.gov/clinvar/variation/335373/
NM_002485.4(NBN):c.663C>T	concordant	unreportable	unreportable	185147	0		3	SCV000685812;S(	Likely benign;Likely benign	Color Genomics, In	2017-10-26;2017-1	NBN	chr8	90983440	90983440	http://gnomad.b	1.12E-05	NFE	1.80E-05	0		0	0	https://www.ncbi.nlm.nih.gov/clinvar/variation/185147/
NM_000310.3(PPT1):c.175G>A	concordant	unreportable	unreportable	206640	0		1	SCV000640454	Uncertain significance	Invitae	10/5/2017	PPT1	chr1	40558129	40558129	http://gnomad.b	3.90E-05	AMR	0.0003871119989	0		0	0	https://www.ncbi.nlm.nih.gov/clinvar/variation/206640/
NM_000152.3(GAA):c.1285C>G	concordant	unreportable	unreportable	291064	0		2	SCV000345748;S(	Benign;Benign	EGL Genetic Diagn	2017-06-30;2017-1	GAA	chr17	78082586	78082586	http://gnomad.b	0.0008921573532	SAS	0.007809319999	5		4	0	https://www.ncbi.nlm.nih.gov/clinvar/variation/291064/
NM_000050.4(ASS1):c.858A>T	concordant	unreportable	unreportable	365259	0		1	SCV000477631	Uncertain significance	Illumina Clinical Se	10/18/2016	ASS1	chr9	133364739	133364739	http://gnomad.b	0.0005319534109	ASJ	0.005583759863	0		1	0	https://www.ncbi.nlm.nih.gov/clinvar/variation/365259/
NM_000159.2(GCDH):c.531C>T	concordant	unreportable	unreportable	385460	0		1	SCV000526718	Likely benign	GeneDx	11/28/2017	GCDH	chr19	13006831	13006831	http://gnomad.b	0.0003795290466	NFE	0.0005911969929	0		0	0	https://www.ncbi.nlm.nih.gov/clinvar/variation/385460/
NM_138694.3(PKHD1):c.7482A>T	concordant	unreportable	unreportable	357429	0		1	SCV000464064	Uncertain significance	Illumina Clinical Se	10/18/2016	PKHD1	chr6	51735306	51735306	http://gnomad.b	0.00115646788	SAS	0.01348629966	2		9	0	https://www.ncbi.nlm.nih.gov/clinvar/variation/357429/
NM_000642.2(AGL):c.2546+10T>C	concordant	unreportable	unreportable	256728	0		2	SCV000525431;S(	Likely benign;Benign	GeneDx;Invitae	2017-11-28;2017-1	AGL	chr1	100350017	100350017	http://gnomad.b	0.0005107537385	AFR	0.007113419939	0		2	0	https://www.ncbi.nlm.nih.gov/clinvar/variation/256728/
NM_002485.4(NBN):c.1354A>C	concordant	unreportable	unreportable	142374	0		3	SCV000211450;S(	Uncertain significance;Unc	GeneDx;Genetic Se	2017-02-15;2017-0	NBN	chr8	90967554	90967554	http://gnomad.b	0.0002565532627	AFR	0.002615399892	0		0	0	https://www.ncbi.nlm.nih.gov/clinvar/variation/142374/
NM_000057.2(BLM):c.2515A>G	concordant	unreportable	unreportable	281221	0		2	SCV000331800;S(	Uncertain significance;Unc	EGL Genetic Diagn	2017-06-30;2017-1	BLM	chr15	91312776	91312776	http://gnomad.b	9.04E-05	EAS	0.002147420077	0		0	0	https://www.ncbi.nlm.nih.gov/clinvar/variation/281221/
NM_000492.3(CFTR):c.4296C>T	concordant	unreportable	unreportable	289600	0		2	SCV000343985;S(	Uncertain significance;Unc	EGL Genetic Diagn	2017-06-30;2016-1	CFTR	chr7	117307015	117307015	http://gnomad.b	0.0001213274377	AMR	0.0008656199789	1		0	0	https://www.ncbi.nlm.nih.gov/clinvar/variation/289600/
NM_000642.2(AGL):c.1885G>A	concordant	unreportable	unreportable	289630	0		2	SCV000344018;S(	Uncertain significance;Likel	EGL Genetic Diagn	2017-06-30;2017-1	AGL	chr1	100346337	100346337	http://gnomad.b	0.0003712035368	AFR	0.005162720103	0		1	0	https://www.ncbi.nlm.nih.gov/clinvar/variation/289630/
NM_033056.3(PCDH15):c.2581G>A	concordant	unreportable	unreportable	300188	0		1	SCV000363189	Uncertain significance	Illumina Clinical Se	10/18/2016	PCDH15	chr10	55780122	55780122	http://gnomad.b	0.0006326184231	AMR	0.001340319985	0		0	0	https://www.ncbi.nlm.nih.gov/clinvar/variation/300188/
NM_000053.3(ATP7B):c.3069T>C	concordant	unreportable	unreportable	210484	0		1	SCV000246745	Likely benign	Genetic Services La	10/4/2017	ATP7B	chr13	52518419	52518419	http://gnomad.b	0.0001992188377	NFE	0.0002908990136	0		0	0	https://www.ncbi.nlm.nih.gov/clinvar/variation/210484/
NM_138694.3(PKHD1):c.5478G>A	concordant	unreportable	unreportable	289814	0		3	SCV000344233;S(	Uncertain significance;Unc	EGL Genetic Diagn	2017-06-30;2016-1	PKHD1	chr6	51882330	51882330	http://gnomad.b	0.0007533721047	NFE	0.001770440023	1		0	0	https://www.ncbi.nlm.nih.gov/clinvar/variation/289814/
NM_000053.3(ATP7B):c.98T>C	concordant	unreportable	unreportable	35736	0		4	SCV000227003;S(	Uncertain significance;Unc	EGL Genetic Diagn	2017-06-30;2017-1	ATP7B	chr13	52549258	52549258	http://gnomad.b	0.00089508182	AMR	0.001191259944	0		2	0	https://www.ncbi.nlm.nih.gov/clinvar/variation/35736/
NM_000136.2(FANCC):c.554G>A	concordant	unreportable	unreportable	182472	0		2	SCV000211038;S(	Uncertain significance;Unc	GeneDx;Invitae	2017-11-28;2017-1	FANCC	chr9	97912337	97912337	http://gnomad.b	8.32E-05	NFE	9.85E-05	0		0	0	https://www.ncbi.nlm.nih.gov/clinvar/variation/182472/
NM_000051.3(ATM):c.334G>A	concordant	unreportable	unreportable	127370	0		5	SCV000611359;S(	Uncertain significance;Unc	Fulgent Genetics,F	2017-05-23;2017-1	ATM	chr11	108106399	108106399	http://gnomad.b	0.0002729014436	AFR	0.002820410067	0		0	0	https://www.ncbi.nlm.nih.gov/clinvar/variation/127370/
NM_000057.2(BLM):c.11T>C	concordant	unreportable	unreportable	127473	0		6	SCV000704456;S(	Uncertain significance;Unc	EGL Genetic Diagn	2017-06-30;2017-0	BLM	chr15	91290633	91290633	http://gnomad.b	0.001234362365	NFE	0.002199860057	0		0	0	https://www.ncbi.nlm.nih.gov/clinvar/variation/127473/
NM_000098.2(CPT2):c.1634A>C	concordant	unreportable	unreportable	166955	0		3	SCV000202564;S(	Uncertain significance;Unc	EGL Genetic Diagn	2017-06-30;2017-1	CPT2	chr1	53676980	53676980	http://gnomad.b	0.0006127828175	FIN	0.002686369931	0		0	0	https://www.ncbi.nlm.nih.gov/clinvar/variation/166955/
NM_000108.3(DLD):c.1395T>C	concordant	unreportable	unreportable	391374	0		1	SCV000534431	Likely benign	GeneDx	11/28/2017	DLD	chr7	107559475	107559475	http://gnomad.b	0.000308949675	EAS	0.003420690075	0		0	0	https://www.ncbi.nlm.nih.gov/clinvar/variation/391374/
NM_000051.3(ATM):c.2932T>C	concordant	unreportable	unreportable	127362	0		4	SCV000343835;S(	Likely benign;Uncertain sig	EGL Genetic Diagn	2017-06-30;2017-0	ATM	chr11	108141988	108141988	http://gnomad.b	0.0008047807877	SAS	0.005003570113	2		1	0	https://www.ncbi.nlm.nih.gov/clinvar/variation/127362/
NM_001042432.1(CLN3):c.461-3C>A	concordant	unreportable	unreportable	418607	0		1	SCV000565798	Uncertain significance	GeneDx	11/28/2017	CLN3	chr16	28497974	28497974	http://gnomad.b	0.0001055532099	AMR	0.0002500469855	0		0	0	https://www.ncbi.nlm.nih.gov/clinvar/variation/418607/
NM_000152.3(GAA):c.1343G>C	concordant	unreportable	unreportable	283498	0		2	SCV000335652;S(	Likely benign;Benign	EGL Genetic Diagn	2017-06-30;2017-1	GAA	chr17	78083760	78083760	http://gnomad.b	0.0009783158515	SAS	0.00796282012	5		4	0	https://www.ncbi.nlm.nih.gov/clinvar/variation/283498/
NM_004937.2(CTNS):c.116C>T	concordant	unreportable	unreportable	322836	0		2	SCV000603242;S(	Uncertain significance;Unc	ARUP Laboratories	2017-06-30;2016-1	CTNS	chr17	3550792	3550792	http://gnomad.b	0.002124494803	AMR	0.002828900004	0		3	0	https://www.ncbi.nlm.nih.gov/clinvar/variation/322836/
NM_000642.2(AGL):c.3884G>A	concordant	unreportable	unreportable	287476	0		1	SCV000341259	Uncertain significance	EGL Genetic Diagn	6/30/2017	AGL	chr1	100378008	100378008	http://gnomad.b	0.0002874734157	ASJ	0.001320600044	0		0	0	https://www.ncbi.nlm.nih.gov/clinvar/variation/287476/
NM_000152.3(GAA):c.368G>A	concordant	unreportable	unreportable	283495	0		2	SCV000335648;S(	Likely benign;Benign	EGL Genetic Diagn	2017-06-30;2017-1	GAA	chr17	78078753	78078753	http://gnomad.b	0.0008421298381	SAS	0.007650739979	5		4	0	https://www.ncbi.nlm.nih.gov/clinvar/variation/283495/
NM_014363.4(SACS):c.7528G>A	concordant	unreportable	unreportable	260399	0		3	SCV000614977;S(	Uncertain significance;Unc	Athena Diagnostics	2017-08-17;2017-1	SACS	chr13	23910487	23910487	http://gnomad.b	0.00136346391	NFE	0.002169430023	0		0	0	https://www.ncbi.nlm.nih.gov/clinvar/variation/260399/
NM_005562.2(LAMC2):c.2892C>T	concordant	unreportable	unreportable	294013	0		1	SCV000351688	Uncertain significance	Illumina Clinical Se	10/18/2016	LAMC2	chr1	183208521	183208521	http://gnomad.b	0.0008399079461	NFE	0.001147430041	0		0	0	https://www.ncbi.nlm.nih.gov/clinvar/variation/294013/

Supplemental Table 2

NM_000232.4(SGCB):c.368A>C	concordant	unreportable	unreportable	92661	0	2	SCV000110258;S	Uncertain significance;Unc	EGL Genetic Diagn	2017-06-30;2016-1	SGCB	chr4	52895905	52895905	http://gnomad.b	0.0001623276668	SAS	0.0009096229915	1		0	0	https://www.ncbi.nlm.nih.gov/clinvar/variation/92661/
NM_000152.3(GAA):c.351G>A	concordant	unreportable	unreportable	283496	0	2	SCV000335649;S	Likely benign;Benign	EGL Genetic Diagn	2017-06-30;2017-1	GAA	chr17	78078736	78078736	http://gnomad.b	0.0008115574677	SAS	0.007718360052	5		4	0	https://www.ncbi.nlm.nih.gov/clinvar/variation/283496/
NM_033056.3(PCDH15):c.343G>A	concordant	unreportable	unreportable	46467	0	2	SCV000343796;S	Likely benign;Benign	EGL Genetic Diagn	2017-06-30;2016-0	PCDH15	chr10	56129011	56129011	http://gnomad.b	0.0005014658232	AFR	0.005952380132	0		1	0	https://www.ncbi.nlm.nih.gov/clinvar/variation/46467/
NM_000243.2(MEFV):c.195C>T	concordant	unreportable	unreportable	97474	0	1	SCV000696059	Uncertain significance	Laboratory Corpora	1/25/2018	MEFV	chr16	3306393	3306393	http://gnomad.b	0.00013434089	SAS	0.0007471899735	0		0	0	https://www.ncbi.nlm.nih.gov/clinvar/variation/97474/
NM_000352.3(ABCC8):c.354C>T	concordant	unreportable	unreportable	255930	0	1	SCV000369413	Likely benign	Illumina Clinical Se	10/18/2016	ABCC8	chr11	17491706	17491706	http://gnomad.b	0.002521719828	FIN	0.02490130067	10		3	0	https://www.ncbi.nlm.nih.gov/clinvar/variation/255930/
NM_003060.3(SLC22A5):c.1441G>A	concordant	unreportable	unreportable	380141	0	2	SCV000517900;S	Likely benign;Likely benign	GeneDx;Invitae	2017-11-28;2017-1	SLC22A5	chr5	131728298	131728298	http://gnomad.b	0.0007879486224	ASJ	0.002843789989	1		0	0	https://www.ncbi.nlm.nih.gov/clinvar/variation/380141/
NM_000049.2(ASPA):c.509T>C	concordant	unreportable	unreportable	322633	0	1	SCV000401982	Uncertain significance	Illumina Clinical Se	10/18/2016	ASPA	chr17	3386869	3386869	http://gnomad.b	0.0005039130147	NFE	0.0008419919759	0		1	0	https://www.ncbi.nlm.nih.gov/clinvar/variation/322633/
NM_001360.2(DHCR7):c.131T>C	concordant	unreportable	unreportable	288193	0	1	SCV000342232	Uncertain significance	EGL Genetic Diagn	6/30/2017	DHCR7	chr11	71155229	71155229	http://gnomad.b	2.69E-05	AFR	0.0002248200035	0		0	0	https://www.ncbi.nlm.nih.gov/clinvar/variation/288193/
NM_001042432.1(CLN3):c.1013G>A	concordant	unreportable	unreportable	373631	0	1	SCV000492244	Uncertain significance	GeneDx	12/21/2016	CLN3	chr16	28493469	28493469	http://gnomad.b	1.94E-05	EAS	0.0002912060008	0		0	0	https://www.ncbi.nlm.nih.gov/clinvar/variation/373631/
NM_000492.3(CFTR):c.2769C>T	concordant	unreportable	unreportable	93150	0	2	SCV000110856;S	Uncertain significance;Unc	EGL Genetic Diagn	2017-06-30;2018-0	CFTR	chr7	117243697	117243697	http://gnomad.b	7.13E-05	NFE	9.85E-05	0		0	0	https://www.ncbi.nlm.nih.gov/clinvar/variation/93150/
NM_000153.3(GALC):c.1788C>T	concordant	unreportable	unreportable	456715	0	1	SCV000627129	Benign	Invitae	10/5/2017	GALC	chr14	88407785	88407785	http://gnomad.b	0.001798520905	AFR	0.0226408001	5		4	0	https://www.ncbi.nlm.nih.gov/clinvar/variation/456715/
NM_014363.4(SACS):c.8127A>C	concordant	unreportable	unreportable	416832	0	2	SCV000614983;S	Likely benign;Benign	Athena Diagnostics	2017-08-17;2017-1	SACS	chr13	23909888	23909888	http://gnomad.b	0.001775467135	AFR	0.02177320048	3		6	0	https://www.ncbi.nlm.nih.gov/clinvar/variation/416832/
NM_004646.3(NPHS1):c.115G>A	concordant	unreportable	unreportable	328883	0	1	SCV000411592	Uncertain significance	Illumina Clinical Se	10/18/2016	NPHS1	chr19	36342518	36342518	http://gnomad.b	0.0008780781517	EAS	0.0009873389499	2		12	0	https://www.ncbi.nlm.nih.gov/clinvar/variation/328883/
NM_003640.3(IKBKAP):c.2025C>T	concordant	unreportable	unreportable	390529	0	1	SCV000533385	Likely benign	GeneDx	11/28/2017	IKBKAP	chr9	111662645	111662645	http://gnomad.b	0.0001372125043	NFE	0.0002687500091	0		0	0	https://www.ncbi.nlm.nih.gov/clinvar/variation/390529/
NM_000053.3(ATP7B):c.2544C>T	concordant	unreportable	unreportable	312387	0	1	SCV000384668	Uncertain significance	Illumina Clinical Se	10/18/2016	ATP7B	chr13	52524439	52524439	http://gnomad.b	0.0005359267324	FIN	0.000942041981	0		1	0	https://www.ncbi.nlm.nih.gov/clinvar/variation/312387/
NM_000271.4(NPC1):c.3321C>A	concordant	unreportable	unreportable	326250	0	1	SCV000407848	Uncertain significance	Illumina Clinical Se	10/18/2016	NPC1	chr18	21115589	21115589	http://gnomad.b	0.0001115088578	ASJ	0.000913706026	0		0	0	https://www.ncbi.nlm.nih.gov/clinvar/variation/326250/
NM_000642.2(AGL):c.4331A>G	concordant	unreportable	unreportable	424906	0	1	SCV000626753	Benign	Invitae	10/5/2017	AGL	chr1	100382037	100382037	http://gnomad.b	0.0006726319726	SAS	0.003941880073	2		0	0	https://www.ncbi.nlm.nih.gov/clinvar/variation/424906/
NM_000271.4(NPC1):c.2428G>T	concordant	unreportable	unreportable	499327	0	1	SCV000704761	Uncertain significance	EGL Genetic Diagn	6/30/2017	NPC1	chr18	21121118	21121118	http://gnomad.b	0.0005032194609	AMR	0.002561050002	0		0	0	https://www.ncbi.nlm.nih.gov/clinvar/variation/499327/
NM_017890.4(VPS13B):c.10624A>G	concordant	unreportable	unreportable	212568	0	1	SCV000577343	Uncertain significance	GeneDx	11/28/2017	VPS13B	chr8	100866166	100866166	http://gnomad.b	0.0001287830006	NFE	0.0001700709981	0		0	0	https://www.ncbi.nlm.nih.gov/clinvar/variation/212568/
NM_004646.3(NPHS1):c.151C>T	concordant	unreportable	unreportable	328882	0	1	SCV000411591	Uncertain significance	Illumina Clinical Se	10/18/2016	NPHS1	chr19	36342482	36342482	http://gnomad.b	0.001416075242	EAS	0.0106271999	4		6	0	https://www.ncbi.nlm.nih.gov/clinvar/variation/328882/
NM_017890.4(VPS13B):c.6706A>C	concordant	unreportable	unreportable	95862	0	1	SCV000597886	Uncertain significance	Genetic Services La	7/5/2017	VPS13B	chr8	100729575	100729575	http://gnomad.b	3.92E-05	NFE	4.49E-05	0		0	0	https://www.ncbi.nlm.nih.gov/clinvar/variation/95862/
NM_000023.2(SGCA):c.366G>A	concordant	unreportable	unreportable	497073	0	1	SCV000701255	Uncertain significance	EGL Genetic Diagn	6/30/2017	SGCA	chr17	48245361	48245361	http://gnomad.b	0.0002518722504	AFR	0.002549020108	0		2	0	https://www.ncbi.nlm.nih.gov/clinvar/variation/497073/
NM_000271.4(NPC1):c.3012G>A	concordant	unreportable	unreportable	326252	0	1	SCV000407851	Uncertain significance	Illumina Clinical Se	10/18/2016	NPC1	chr18	21118535	21118535	http://gnomad.b	0.0001315232682	EAS	0.0005797769991	0		1	0	https://www.ncbi.nlm.nih.gov/clinvar/variation/326252/
NM_000642.2(AGL):c.2088C>T	concordant	unreportable	unreportable	456466	0	1	SCV000626690	Likely benign	Invitae	10/5/2017	AGL	chr1	100346934	100346934	http://gnomad.b	0.0001200131735	AFR	0.0009814179502	0		0	0	https://www.ncbi.nlm.nih.gov/clinvar/variation/456466/
NM_000642.2(AGL):c.3231G>A	concordant	unreportable	unreportable	291340	0	2	SCV000346081;S	Uncertain significance;Likel	Illumina Clinical Se	2016-10-18;2017-1	AGL	chr1	100358135	100358135	http://gnomad.b	0.0001507142179	AMR	0.0003578669857	0		0	0	https://www.ncbi.nlm.nih.gov/clinvar/variation/291340/
NM_000271.4(NPC1):c.1232G>A	concordant	unreportable	unreportable	235359	0	1	SCV000332338	Benign	EGL Genetic Diagn	6/30/2017	NPC1	chr18	21136301	21136301	http://gnomad.b	0.0005861363039	AFR	0.007187659852	2		2	0	https://www.ncbi.nlm.nih.gov/clinvar/variation/235359/
NM_138694.3(PKHD1):c.1676G>A	concordant	unreportable	unreportable	291029	0	2	SCV000345708;S	Uncertain significance;Unc	EGL Genetic Diagn	2017-06-30;2016-1	PKHD1	chr6	51921513	51921513	http://gnomad.b	0.0007200124543	NFE	0.0008358500199	0		1	0	https://www.ncbi.nlm.nih.gov/clinvar/variation/291029/
NM_000642.2(AGL):c.1759C>T	concordant	unreportable	unreportable	291333	0	3	SCV000602454;S	Uncertain significance;Unc	ARUP Laboratories	2017-06-30;2016-1	AGL	chr1	100346211	100346211	http://gnomad.b	0.0007172880372	AMR	0.001162030036	4		0	0	https://www.ncbi.nlm.nih.gov/clinvar/variation/291333/
NM_001876.3(CPT1A):c.1529C>T	concordant	unreportable	unreportable	377754	0	2	SCV000512760;S	Likely benign;Benign	GeneDx;Invitae	2017-02-15;2017-1	CPT1A	chr11	68542830	68542830	http://gnomad.b	0.001999380474	AFR	0.02241240069	3		6	0	https://www.ncbi.nlm.nih.gov/clinvar/variation/377754/
NM_000017.2(ACADS):c.465C>T	concordant	unreportable	unreportable	307436	0	1	SCV000376678	Uncertain significance	Illumina Clinical Se	10/18/2016	ACADS	chr12	121175263	121175263	http://gnomad.b	0.0005069155745	EAS	0.003943860065	0		0	0	https://www.ncbi.nlm.nih.gov/clinvar/variation/307436/
NM_000053.3(ATP7B):c.3211T>C	concordant	unreportable	unreportable	456555	0	1	SCV000626852	Likely benign	Invitae	10/5/2017	ATP7B	chr13	52518277	52518277	http://gnomad.b	5.33E-05			0		2	0	https://www.ncbi.nlm.nih.gov/clinvar/variation/456555/
NM_000057.2(BLM):c.178T>A	concordant	unreportable	unreportable	133710	0	2	SCV000394408;S	Uncertain significance;Beni	Illumina Clinical Se	2016-10-18;2017-1	BLM	chr15	91292676	91292676	http://gnomad.b	0.0003829139514	EAS	0.003421080066	1		0	0	https://www.ncbi.nlm.nih.gov/clinvar/variation/133710/
NM_000053.3(ATP7B):c.3891C>T	concordant	unreportable	unreportable	157954	0	4	SCV000521846;S	Benign;Uncertain significar	GeneDx;Illumina C	2017-11-28;2016-1	ATP7B	chr13	52511624	52511624	http://gnomad.b	0.002702080855	AFR	0.0360131003	11		17	0	https://www.ncbi.nlm.nih.gov/clinvar/variation/157954/
NM_000370.3(TTPA):c.452G>A	concordant	unreportable	unreportable	363561	0	1	SCV000474657	Uncertain significance	Illumina Clinical Se	10/18/2016	TTPA	chr8	63978563	63978563	http://gnomad.b	0.0004323519345	SAS	0.001332290005	0		0	0	https://www.ncbi.nlm.nih.gov/clinvar/variation/363561/
NM_017890.4(VPS13B):c.7413T>C	concordant	unreportable	unreportable	197444	0	1	SCV000630885	Benign	Invitae	10/5/2017	VPS13B	chr8	100789093	100789093	http://gnomad.b	0.001318625941	AFR	0.01450789999	1		3	0	https://www.ncbi.nlm.nih.gov/clinvar/variation/197444/
NM_000352.3(ABCC8):c.793C>T	concordant	unreportable	unreportable	392921	0	1	SCV000536263	Uncertain significance	GeneDx	11/28/2017	ABCC8	chr11	17483159	17483159		7.74E-06					0	0	https://www.ncbi.nlm.nih.gov/clinvar/variation/392921/
NM_000152.3(GAA):c.2478G>A	concordant	unreportable	unreportable	285676	0	4	SCV000338813;S	Likely benign;Likely benign	EGL Genetic Diagn	2017-06-30;2017-1	GAA	chr17	78091545	78091545	http://gnomad.b	0.0009977709963	EAS	0.0120249996	2		2	0	https://www.ncbi.nlm.nih.gov/clinvar/variation/285676/
NM_000352.3(ABCC8):c.-113G>A	concordant	unreportable	unreportable	303792	0	1	SCV000369442	Uncertain significance	Illumina Clinical Se	10/18/2016	ABCC8	chr11	17498436	17498436		0.0003820005472					0	0	https://www.ncbi.nlm.nih.gov/clinvar/variation/303792/
NM_000492.3(CFTR):c.3897A>G	concordant	unreportable	unreportable	93154	0	5	SCV000602975;S	Benign;Likely benign;Benig	ARUP Laboratories	2017-06-30;2017-0	CFTR	chr7	117292919	117292919	http://gnomad.b	0.001402005947	NFE	0.001918380032	1		1	0	https://www.ncbi.nlm.nih.gov/clinvar/variation/93154/
NM_000492.3(CFTR):c.4340T>A	concordant	unreportable	unreportable	455781	0	1	SCV000625752	Uncertain significance	Invitae	10/5/2017	CFTR	chr7	117307059	117307059		1.16E-05					0	0	https://www.ncbi.nlm.nih.gov/clinvar/variation/455781/
NM_018941.3(CLN8):c.290G>A	concordant	unreportable	unreportable	136807	0	2	SCV000227483;S	Benign;Likely benign	EGL Genetic Diagn	2017-06-30;2017-1	CLN8	chr8	1719510	1719510	http://gnomad.b	0.0006548018805	AFR	0.008363829926	0		1	0	https://www.ncbi.nlm.nih.gov/clinvar/variation/136807/
NM_004937.2(CTNS):c.407T>G	concordant	unreportable	unreportable	322839	0	1	SCV000402221	Uncertain significance	Illumina Clinical Se	10/18/2016	CTNS	chr17	3558592	3558592	http://gnomad.b	2.37E-05	NFE	4.48E-05	0		0	0	https://www.ncbi.nlm.nih.gov/clinvar/variation/322839/
NM_000057.2(BLM):c.819_821delGA	concordant	unreportable	unreportable	127516	0	1	SCV000543341	Uncertain significance	Invitae	10/5/2017	BLM	chr15	91295027	91295030	http://gnomad.b	7.14E-05	NFE	5.38E-05	0		0	0	https://www.ncbi.nlm.nih.gov/clinvar/variation/127516/
NM_000382.2(ALDH3A2):c.417G>A	concordant	unreportable	unreportable	322209	0	1	SCV000401251	Uncertain significance	Illumina Clinical Se	10/18/2016	ALDH3A2	chr17	19555891	19555891	http://gnomad.b	0.0003415128459	EAS	0.001507419976	0		0	0	https://www.ncbi.nlm.nih.gov/clinvar/variation/322209/
NM_002485.4(NBN):c.2140C>T	concordant	reportable	reportable	182737	3	0	SCV000611218;S	Likely pathogenic;Pathoger	Fulgent Genetics,F	2017-05-23;2017-1	NBN	chr8	90955525	90955525	http://gnomad.b	3.35E-05	ASJ	0.0001015640009	0		0	0	https://www.ncbi.nlm.nih.gov/clinvar/variation/182737/
NM_000053.3(ATP7B):c.2955C>T	concordant	unreportable	unreportable	285883	0	2	SCV000339094;S	Uncertain significance;Likel	EGL Genetic Diagn	2017-06-30;2017-1	ATP7B	chr13	52520525	52520525	http://gnomad.b	0.0005078677411	AFR	0.007000789978	1		1	0	https://www.ncbi.nlm.nih.gov/clinvar/variation/285883/
NM_000050.4(ASS1):c.99C>T	concordant	unreportable	unreportable	254750	0	1	SCV000530534	Likely benign	GeneDx	11/28/2017	ASS1	chr9	133327714	133327714	http://gnomad.b	0.0002144524222	AFR	0.003398689907	0		0	0	https://www.ncbi.nlm.nih.gov/clinvar/variation/254750/
NM_004543.4(NEB):c.3444G>A	concordant	unreportable	unreportable	331518	0	1	SCV000417016	Uncertain significance	Illumina Clinical Se	10/18/2016	NEB	chr2	152534513	152534513	http://gnomad.b	0.0001807123163	EAS	0.0004060319916	0		0	0	https://www.ncbi.nlm.nih.gov/clinvar/variation/331518/
NM_000053.3(ATP7B):c.1728G>A	concordant	unreportable	unreportable	386031	0	2	SCV000527541;S	Benign;Benign	GeneDx;Laboratory	2017-11-28;2018-0	ATP7B	chr13	52539149	52539149	http://gnomad.b	0.001349637478	AFR	0.01477510016	4		1	0	https://www.ncbi.nlm.nih.gov/clinvar/variation/386031/
NM_000271.4(NPC1):c.1071G>A	concordant	unreportable	unreportable	281049	0	1	SCV000331135	Benign	EGL Genetic Diagn	6/30/2017	NPC1	chr18	21136462	21136462	http://gnomad.b	0.0008120132209	AFR	0.008899359964	1		1	0	https://www.ncbi.nlm.nih.gov/clinvar/variation/281049/
NM_002485.4(NBN):c.456G>A	concordant	unreportable	unreportable	127872	0	5	SCV000537561;S	Uncertain significance;Unc	Color Genomics, In	2017-12-21;2017-0	NBN	chr8	90992986	90992986	http://gnomad.b	0.0002258784161	NFE	0.0002240589965	0		0	0	https://www.ncbi.nlm.nih.gov/clinvar/variation/127872/
NM_033056.3(PCDH15):c.5603C>T	concordant	unreportable	unreportable	46506	0	1	SCV000114273	Benign	EGL Genetic Diagn	6/30/2017	PCDH15	chr10	55581883	55581883		0.0009540708738					2	0	https://www.ncbi.nlm.nih.gov/clinvar/variation/46506/
NM_000051.3(ATM):c.6795C>T	concordant	unreportable	unreportable	135774	0	4	SCV000537473;S	Likely benign;Likely benign	Color Genomics, In	2017-01-26;2017-0	ATM	chr11	108196259	108196259	http://gnomad.b	0.0002812555694	NFE	0.0003708660079	0		0	0	https://www.ncbi.nlm.nih.gov/clinvar/variation/135774/
NM_000520.4(HEXA):c.548T>A	concordant	unreportable	unreportable	496012	0	1	SCV000697171	Uncertain significance	Laboratory Corpora	1/25/2018	HEXA	chr15	72645431	72645431	http://gnomad.b	0.0005399846926	EAS	0.005275969859	0		3	0	https://www.ncbi.nlm.nih.gov/clinvar/variation/496012/
NM_138694.3(PKHD1):c.129T>C	concordant	unreportable	unreportable	281794	0	1	SCV000332776	Uncertain significance	EGL Genetic Diagn	6/30/2017	PKHD1	chr6	51947977	51947977	http://gnomad.b	0.0002752171157	NFE	0.0003943849879	0		0	0	https://www.ncbi.nlm.nih.gov/clinvar/variation/281794/
NM_000112.3(SLC26A2):c.2087A>G	concordant	unreportable	unreportable	284376	0	2	SCV000605141;S	Uncertain significance;Beni	ARUP Laboratories	2017-06-30;2017-0	SLC26A2	chr5	149361243	149361243	http://gnomad.b	0.0006384129444	AFR	0.007530120201	0		0	0	https://www.ncbi.nlm.nih.gov/clinvar/variation/284376/
NM_017890.4(VPS13B):c.11270G>A	concordant	unreportable	unreportable	198013	0	2	SCV000470862;S	Uncertain significance;Beni	Illumina Clinical Se	2016-10-18;2017-1	VPS13B	chr8	100874154	100874154	http://gnomad.b	0.00193454464	SAS	0.003965309821	4		2	0	https://www.ncbi.nlm.nih.gov/clinvar/variation/198013/
NM_000108.3(DLD):c.117G>A	concordant	unreportable	unreportable	358566	0	1	SCV000466212	Uncertain significance	Illumina Clinical Se	10/18/2016	DLD	chr7	107533722	107533722	http://gnomad.b	5.19E-06	AFR	6.55E-05	0		0	0	https://www.ncbi.nlm.nih.gov/clinvar/variation/358566/
NM_000057.2(BLM):c.1044G>A	concordant	unreportable	unreportable	413284	0	2	SCV000555814;S	Likely benign;Uncertain sig	Invitae;Laboratory	2017-10-05;2018-0	BLM	chr15	91298125	91298125	http://gnomad.b	0.0003282119583	EAS	0.003195069963	0		0	0	https://www.ncbi.nlm.nih.gov/clinvar/variation/413284/
NM_000051.3(ATM):c.4465C>T	concordant	unreportable	unreportable	407548	0	4	SCV000687550;S	Uncertain significance;Unc	Color Genomics, In	2017-12-21;2017-1	ATM	chr11	108163374	108163374	http://gnomad.b	1.39E-05	NFE	1.79E-05	0		0	0	https://www.ncbi.nlm.nih.gov/clinvar/variation/407548/
NM_000227.3(LAMA3):c.342T>C	concordant	unreportable	unreportable	326295	0	1	SCV000407912	Uncertain significance	Illumina Clinical Se	10/18/2016	LAMA3	chr18	21461956	21461956	http://gnomad.b	0.0001791803618	NFE	0.0003943289921	0		0	0	https://www.ncbi.nlm.nih.gov/clinvar/variation/326295/
NM_000051.3(ATM):c.95G>A	concordant	unreportable	unreportable	181945	0	2	SCV000209724;S	Uncertain significance;Unc	GeneDx;Invitae	2017-11-28;2017-1	ATM	chr11	108098525	108098525	http://gnomad.b	1.67E-05	SAS	6.50E-05	0		0	0	https://www.ncbi.nlm.nih.gov/clinvar/variation/181945/
NM_138694.3(PKHD1):c.8022G>T	concordant	unreportable	unreportable	262416	0	1	SCV000345798	Uncertain significance	EGL Genetic Diagn	6/30/2017	PKHD1	chr6	51712658	51712658	http://gnomad.b	0.0001473384559	EAS	0.0002319110063	0		0	0	https://www.ncbi.nlm.nih.gov/clinvar/variation/262416/
NM_000136.2(FANCC):c.882T>C	concordant	unreportable	unreportable	456167	0	1	SCV000626257	Likely benign	Invitae	10/5/2017	FANCC	chr9	97888825	97888825	http://gnomad.b	3.57E-05	ASJ	0.0002030869946	0		0	0	https://www.ncbi.nlm.nih.gov/clinvar/variation/456167/
NM_000271.4(NPC1):c.501A>G	concordant	unreportable	unreportable	326278	0	1	SCV000407888	Uncertain significance	Illumina Clinical Se	10/18/2016	NPC1	chr18	21141454	21141454	http://gnomad.b	0.0006318835276	EAS	0.006029679906	0		1	0	https://www.ncbi.nlm.nih.gov/clinvar/variation/326278/
NM_000051.3(ATM):c.1748A>G	concordant	unreportable	unreportable	181989	0	3	SCV000367031;S	Uncertain significance;Unc	Illumina Clinical Se	2016-10-18;2017-1	ATM	chr11	108122704	108122704	http://gnomad.b	1.95E-05	SAS	3.25E-05	0		0	0	https://www.ncbi.nlm.nih.gov/clinvar/variation/181989/
NM_033056.3(PCDH15):c.131T>C	concordant	unreportable	unreportable	450626	0	1	SCV000619232	Uncertain significance	GeneDx	11/28/2017	PCDH15	chr10	56287598	56287598	http://gnomad.b	9.51E-05	SAS	6.52E-05	0		0	0	https://www.ncbi.nlm.nih.gov/clinvar/variation/450626/
NM_000018.3(ACADVL):c.1751+18G>	concordant	unreportable	unreportable	379545	0	1	SCV000516705	Likely benign	GeneDx	11/28/2017	ACADVL	chr17	7128051	7128051	http://gnomad.b	0.0004474146231	SAS	0.001064750017	0		0	0	https://www.ncbi.nlm.nih.gov/clinvar/variation/379545/
NM_000018.3(ACADVL):c.63-31C>T	concordant	unreportable	unreportable	439362	0	1	SCV000602378	Likely benign	ARUP Laboratories	6/30/2017	ACADVL	chr17	7123410	7123410	http://gnomad.b	0.0001245964773	AFR	0.0001615509973	0		0	0	https://www.ncbi.nlm.nih.gov/clinvar/variation/439362/
NM_000051.3(ATM):c.3689A>G	concordant	unreportable	unreportable	142043	0	3	SCV000209782;S	Uncertain significance;Unc	GeneDx;Invitae;Lal	2017-11-28;2017-1	ATM	chr11	108153549	108153549	http://gnomad.b	1.95E-05	NFE	1.80E-05	0		0	0	https://www.ncbi.nlm.nih.gov/clinvar/variation/142043/
NM_000018.3(ACADVL):c.818G>C	concordant	unreportable	unreportable	203574	0	2	SCV000238637;S	Uncertain significance;Likel	GeneDx;Invitae	2017-11-28;2017-1	ACADVL	chr17	7125561	7125561	http://gnomad.b	0.000444582885	AFR	0.003659649985	0		1	0	https://www.ncbi.nlm.nih.gov/clinvar/variation/203574/
NM_000051.3(ATM):c.7375C>T	concordant	unreportable	unreportable	185845	0	3	SCV000682410;S	Uncertain significance;Unc	Color Genomics, In	2017-10-26;2017-1	ATM	chr11	108201008	108201008	http://gnomad.b	4.18E-05	EAS	0.0001161309992	0		0	0	https://www.ncbi.nlm.nih.gov/clinvar/variation/185845/
NM_000051.3(ATM):c.4445G>A	concordant	unreportable	unreportable	185247	0	3	SCV000682208;S	Uncertain significance;Unc	Color Genomics, In	2017-10-26;2017-1	ATM	chr11	108163354	108163354	http://gnomad.b	5.57E-06	NFE	3.59E-05	0		0	0	https://www.ncbi.nlm.nih.gov/clinvar/variation/185247/
NM_017890.4(VPS13B):c.5592G>A	concordant	unreportable	unreportable	212577	0	1	SCV000630879	Benign	Invitae	10/5/2017	VPS13B	chr8	100654335	100654335	http://gnomad.b	0.001928945379	AFR	0.02414290048	7		4	0	https://www.ncbi.nlm.nih.gov/clinvar/variation/212577/
NM_000023.2(SGCA):c.661C>T	concordant	unreportable	unreportable	452720	0	2	SCV000702085;S	Uncertain significance;Unc	EGL Genetic Diagn	2017-06-30;2017-1	SGCA	chr17	48246529	48246529	http://gnomad.b	1.40E-05	SAS	0.0002924170112	0		0	0	https://www.ncbi.nlm.nih.gov/clinvar/variation/452720/
NM_000051.3(ATM):c.998C>T	concordant	unreportable	unreportable	127471	0	4	SCV000602564;S	Likely benign;Likely benign	ARUP Laboratories	2017-06-30;2017-1	ATM	chr11	108117787	108117787	http://gnomad.b	0.001292104794	FIN	0.003633589949	1		2	0	https://www.ncbi.nlm.nih.gov/clinvar/variation/127471/
NM_000152.3(GAA):c.2671C>T	concordant	unreportable	unreportable	449823	0	1	SCV000618250	Uncertain significance	GeneDx	11/28/2017	GAA	chr17	78092476	78092476	http://gnomad.b	7.50E-05	SAS	0.0001949190046	0		0	0	https://www.ncbi.nlm.nih.gov/clinvar/variation/449823/
NM_000051.3(ATM):c.7502A>G	concordant	unreportable	unreportable	232481	0	4	SCV000682418;S	Uncertain significance;Unc	Color Genomics, In	2017-10-26;2017-1	ATM	chr11	108201135	108201135	http://gnomad.b	0.0001475895562	SAS	0.00126696995	0		0	0	https://www.ncbi.nlm.nih.gov/clinvar/variation/232481/
NM_000018.3(ACADVL):c.1567G>A	concordant	unreportable	unreportable	282928	0	4	SCV000602379;S	Uncertain significance;Unc	ARUP Laboratories	2017-06-30;2017-0	ACADVL	chr17	7127674	7127674	http://gnomad.b	0.0005550206717	AFR	0.0009186349926	1		0	0	https://www.ncbi.nlm.nih.gov/clinvar/variation/282928/
NM_000071.2(CBS):c.939G>A	concordant	unreportable	unreportable	212824	0	2	SCV000337245;S	Likely benign;Benign	EGL Genetic Diagn	2017-06-30;2017-1	CBS	chr21	44483078	44483078	http://gnomad.b	0.0005708372467	AFR	0.005948489998	1		0	0	https://www.ncbi.nlm.nih.gov/clinvar/variation/212824/
NM_001079802.1(FKTN):c.1159G>A	concordant	unreportable	unreportable	167070	0	4	SCV000202714;S	Uncertain significance;Unc	EGL Genetic Diagn	2017-06-30;2017-1	FKTN	chr9	108382329	108382329	http://gnomad.b	0.0003758572026	NFE	0.0008250090177	0		0	0	https://www.ncbi.nlm.nih.gov/clinvar/variation/167070/
NM_017890.4(VPS13B):c.5681C>T	concordant	unreportable	unreportable	196918	0	3	SCV000229738;S	Uncertain significance;Unc	EGL Genetic Diagn	2017-06-30;2017-0	VPS13B	chr8	100654424	100654424	http://gnomad.b	0.000929477309	SAS	0.001689300057	0		0	0	https://www.ncbi.nlm.nih.gov/clinvar/variation/196918/
NM_138694.3(PKHD1):c.10859G>A	concordant	unreportable	unreportable	496885	0	1	SCV000700492	Uncertain significance	EGL Genetic Diagn	6/30/2017	PKHD1	chr6	51524065	51524065	http://gnomad.b	0.0002585372905	AFR	0.001503659994	0		0	0	https://www.ncbi.nlm.nih.gov/clinvar/variation/496885/
NM_000228.2(LAMB3):c.2933G>C	concordant	unreportable	unreportable	295075	0	1	SCV000353594	Uncertain significance	Illumina Clinical Se	10/18/2016	LAMB3	chr1	209791370	209791370	http://gnomad.b	0.0006270961529	AMR	0.009648029692	1		3	0	https://www.ncbi.nlm.nih.gov/clinvar/variation/295075/
NM_000136.2(FANCC):c.632C>G	concordant	unreportable	unreportable	127544	0	3	SCV000149266;S	Likely benign;Uncertain sig	GeneDx;Illumina C	2017-11-28;2016-1	FANCC	chr9	97912259	97912259	http://gnomad.b	0.0006301991429	FIN	0.00795506034	1		0	0	https://www.ncbi.nlm.nih.gov/clinvar/variation/127544/
NM_000136.2(FANCC):c.468A>G	concordant	unreportable	unreportable	377840	0	1	SCV000560618	Likely benign	Invitae	3/14/2017	FANCC	chr9	97933414	97933414		1.66E-05					0	0	https://www.ncbi.nlm.nih.gov/clinvar/variation/377840/
NM_005562.2(LAMC2):c.3206C>T	concordant	unreportable	unreportable	195603	0	2	SCV000258025;S	Likely benign;Uncertain sig	Division of Genomi	2015-08-10;2016-1	LAMC2	chr1	183209311	183209311	http://gnomad.b	0.001430643202	SAS	0.009063740261	1		1	0	https://www.ncbi.nlm.nih.gov/clinvar/variation/195603/
NM_000050.4(ASS1):c.1015G>A	concordant	unreportable	unreportable	365262	0	1	SCV000477635	Uncertain significance	Illumina Clinical Se	10/18/2016	ASS1	chr9	133370298	133370298	http://gnomad.b	0.0003258562779	AMR	0.0006551910192	0		0	0	https://www.ncbi.nlm.nih.gov/clinvar/variation/365262/
NM_000057.2(BLM):c.1467G>A	concordant	unreportable	unreportable	236801	0	2	SCV000394414;S	Uncertain significance;Likel	Illumina Clinical Se	2016-10-18;2017-1	BLM	chr15	91304070	91304070	http://gnomad.b	0.0002354564049	AFR	0.001377230044	0		0	0	https://www.ncbi.nlm.nih.gov/clinvar/variation/236801/
NM_000466.2(PEX1):c.3373A>T	concordant	unreportable	unreportable	360915	0	1	SCV000470497	Uncertain significance	Illumina Clinical Se	10/18/2016	PEX1	chr7	92120651	92120651	http://gnomad.b	0.0008625423644	SAS	0.003833410097	2		0	0	https://www.ncbi.nlm.nih.gov/clinvar/variation/360915/
NM_000051.3(ATM):c.8187A>T	concordant	unreportable	unreportable	181986	0	2	SCV000687821;S	Uncertain significance;Unc	Color Genomics, In	2017-12-21;2017-1	ATM	chr11	108206607	108206607		5.57E-06					0	0	https://www.ncbi.nlm.nih.gov/clinvar/variation/181986/
NM_017890.4(VPS13B):c.2704A>G	concordant	unreportable	unreportable	361047	0	2	SCV000597882;S	Uncertain significance;Unc	Genetic Services La	2017-07-05;2016-1	VPS13B	chr8	100287362	100287362	http://gnomad.b	0.0007810968952	NFE	0.001353599946	1		0	0	https://www.ncbi.nlm.nih.gov/clinvar/variation/361047/
NM_017890.4(VPS13B):c.6491A>G	concordant	unreportable	unreportable	196985	0	3	SCV000229838;S	Uncertain significance;Unc	EGL Genetic Diagn	2017-06-30;2017-0	VPS13B	chr8	100712122	100712122	http://gnomad.b	0.0008790839609	SAS	0.001596300048	0		0	0	https://www.ncbi.nlm.nih.gov/clinvar/variation/196985/
NM_001360.2(DHCR7):c.988G>A	concordant	unreportable	unreportable	420113	0	2	SCV000706341;S	Uncertain significance;Unc	EGL Genetic Diagn	2017-06-30;2017-1	DHCR7	chr11	71146861	71146861	http://gnomad.b	0.0003661899018	AMR	0.000600527972	0		0	0	https://www.ncbi.nlm.nih.gov/clinvar/variation/420113/
NM_000243.2(MEFV):c.-15C>G	concordant	unreportable	unreportable	319120	0	2	SCV000513589;S	Likely benign;Uncertain sig	GeneDx;Illumina C	2017-11-28;2016-1	MEFV	chr16	3306602	3306602	http://gnomad.b	0.0009375874615	AFR	0.01176619995	1		0	0	https://www.ncbi.nlm.nih.gov/clinvar/variation/319120/
NM_174878.2(CLRN1):c.183G>A	concordant	unreportable	unreportable	501869	0	1	SCV000708387	Uncertain significance	EGL Genetic Diagn	6/30/2017	CLRN1	chr3	150690313	150690313	http://gnomad.b	0.0002751675436	NFE	0.0002774939931	0		0	0	https://www.ncbi.nlm.nih.gov/clinvar/variation/501869/
NM_033056.3(PCDH15):c.1205G>C	concordant	unreportable	unreportable	281800	0	1	SCV000332789	Uncertain significance	EGL Genetic Diagn	11/3/2016	PCDH15	chr10	55955543	55955543	http://gnomad.b	0.0005246103996	AFR	0.00294041005	0		0	0	https://www.ncbi.nlm.nih.gov/clinvar/variation/281800/

Supplemental Table 2

NM_002435.1(MPI):c.1049C>T	concordant	unreportable	unreportable	94065	0	2	SCV000393951;S(	Uncertain significance;Beni	Illumina Clinical Se	2016-10-18;2017-1	MPI	chr15	75189556	75189556	http://gnomad.b	0.002102204656	EAS	0.01855289936	2	2	0	https://www.ncbi.nlm.nih.gov/clinvar/variation/94065/
NM_012203.1(GRHPR):c.962C>T	concordant	unreportable	unreportable	366861	0	1	SCV000480143	Uncertain significance	Illumina Clinical Se	10/18/2016	GRHPR	chr9	37436754	37436754	http://gnomad.b	0.001318819343	AFR	0.01627030037	2	1	0	https://www.ncbi.nlm.nih.gov/clinvar/variation/366861/
NM_017890.4(VPS13B):c.10388A>G	concordant	unreportable	unreportable	197994	0	1	SCV000231389	Uncertain significance	EGL Genetic Diagn	6/30/2017	VPS13B	chr8	100865930	100865930	http://gnomad.b	0.0001455807833	AFR	0.001698900014	0	0	0	https://www.ncbi.nlm.nih.gov/clinvar/variation/197994/
NM_000136.2(FANCC):c.973G>A	concordant	unreportable	unreportable	127550	0	2	SCV000149273;S(	Likely benign;Benign	GeneDx;Invitae	2017-11-28;2017-1	FANCC	chr9	97887391	97887391	http://gnomad.b	0.001288935606	EAS	0.01020410005	1	4	0	https://www.ncbi.nlm.nih.gov/clinvar/variation/127550/
NM_133647.1(SLC12A6):c.964G>A	concordant	unreportable	unreportable	315620	0	1	SCV000390320	Uncertain significance	Illumina Clinical Se	10/18/2016	SLC12A6	chr15	34546703	34546703	http://gnomad.b	0.0001511308141	EAS	0.002319650026	0	1	0	https://www.ncbi.nlm.nih.gov/clinvar/variation/315620/
NM_138694.3(PKHD1):c.325G>A	concordant	unreportable	unreportable	197870	0	2	SCV000231198;S(	Benign;Uncertain significar	EGL Genetic Diagn	2017-06-30;2016-1	PKHD1	chr6	51944763	51944763	http://gnomad.b	0.001245426948	EAS	0.01095909998	2	5	0	https://www.ncbi.nlm.nih.gov/clinvar/variation/197870/
NM_138694.3(PKHD1):c.7091T>A	concordant	unreportable	unreportable	500968	0	1	SCV000707145	Benign	EGL Genetic Diagn	6/30/2017	PKHD1	chr6	51751949	51751949	http://gnomad.b	0.000455915222	EAS	0.002377359895	0	3	0	https://www.ncbi.nlm.nih.gov/clinvar/variation/500968/
NM_017890.4(VPS13B):c.11972A>T	concordant	unreportable	unreportable	361097	0	1	SCV000470869	Uncertain significance	Illumina Clinical Se	10/18/2016	VPS13B	chr8	100887797	100887797	http://gnomad.b	0.0004535401327	EAS	0.003536639968	0	0	0	https://www.ncbi.nlm.nih.gov/clinvar/variation/361097/
NM_138694.3(PKHD1):c.934C>T	concordant	unreportable	unreportable	502343	0	1	SCV000709035	Uncertain significance	EGL Genetic Diagn	6/30/2017	PKHD1	chr6	51929795	51929795	http://gnomad.b	0.000144558485	SAS	0.0006497299764	0	1	0	https://www.ncbi.nlm.nih.gov/clinvar/variation/502343/
NM_000518.4(HBB):c.316-19T>A	concordant	unreportable	unreportable	495993	0	1	SCV000697114	Uncertain significance	Laboratory Corpor	1/25/2018	HBB	chr11	5246975	5246975	http://gnomad.b	0.0002192716449	NFE	0.0004680299899	0	0	0	https://www.ncbi.nlm.nih.gov/clinvar/variation/495993/
NM_017890.4(VPS13B):c.8085C>T	concordant	unreportable	unreportable	459249	0	1	SCV000630889	Benign	Invitae	10/5/2017	VPS13B	chr8	100821671	100821671	http://gnomad.b	0.0009014810045	SAS	0.009649120271	6	4	0	https://www.ncbi.nlm.nih.gov/clinvar/variation/459249/
NM_000466.2(PEX1):c.2792C>A	concordant	unreportable	unreportable	93108	0	2	SCV000110784;S(	Likely benign;Uncertain sig	EGL Genetic Diagn	2017-06-30;2016-1	PEX1	chr7	92123935	92123935	http://gnomad.b	0.0006212523782	EAS	0.007310280111	0	2	0	https://www.ncbi.nlm.nih.gov/clinvar/variation/93108/
NM_138694.3(PKHD1):c.150T>G	concordant	unreportable	unreportable	357459	0	1	SCV000464134	Uncertain significance	Illumina Clinical Se	10/18/2016	PKHD1	chr6	51947321	51947321	http://gnomad.b	0.000102858922	EAS	0.001159689971	0	0	0	https://www.ncbi.nlm.nih.gov/clinvar/variation/357459/
NM_017890.4(VPS13B):c.3363A>G	concordant	unreportable	unreportable	287832	0	2	SCV000341754;S(	Uncertain significance;Unc	EGL Genetic Diagn	2017-06-30;2016-1	VPS13B	chr8	100454781	100454781	http://gnomad.b	0.0001035863266	EAS	0.0008718899917	0	0	0	https://www.ncbi.nlm.nih.gov/clinvar/variation/287832/
NM_000136.2(FANCC):c.395C>G	concordant	unreportable	unreportable	127541	0	3	SCV000149261;S(	Uncertain significance;Unc	GeneDx;Illumina C	2017-11-28;2016-1	FANCC	chr9	97934380	97934380	http://gnomad.b	6.42E-05	NFE	6.28E-05	0	0	0	https://www.ncbi.nlm.nih.gov/clinvar/variation/127541/
NM_000642.2(AGL):c.854G>A	concordant	unreportable	unreportable	385974	0	2	SCV000527439;S(	Uncertain significance;Unc	GeneDx;Invitae	2017-11-28;2017-1	AGL	chr1	100336321	100336321	http://gnomad.b	0.0003237564682	NFE	0.0004944090033	0	0	0	https://www.ncbi.nlm.nih.gov/clinvar/variation/385974/
NM_001164277.1(SLC37A4):c.492C>T	concordant	unreportable	unreportable	302706	0	1	SCV000367673	Uncertain significance	Illumina Clinical Se	10/18/2016	SLC37A4	chr11	118898472	118898472	http://gnomad.b	0.0001117474508	NFE	0.0001266489999	0	0	0	https://www.ncbi.nlm.nih.gov/clinvar/variation/302706/
NM_000057.2(BLM):c.3592G>A	concordant	unreportable	unreportable	127500	0	1	SCV000555815	Likely benign	Invitae	10/5/2017	BLM	chr15	91347430	91347430	http://gnomad.b	0.0002663749227	AFR	0.003466319991	0	0	0	https://www.ncbi.nlm.nih.gov/clinvar/variation/127500/
NM_000228.2(LAMB3):c.557G>A	concordant	unreportable	unreportable	295137	0	1	SCV000353665	Uncertain significance	Illumina Clinical Se	10/18/2016	LAMB3	chr1	209807799	209807799	http://gnomad.b	3.64E-05	NFE	3.62E-05	0	0	0	https://www.ncbi.nlm.nih.gov/clinvar/variation/295137/
NM_000071.2(CBS):c.636C>T	concordant	unreportable	unreportable	136668	0	3	SCV000317311;S(	Benign;Uncertain significar	Ambry Genetics;Illu	2017-11-14;2016-1	CBS	chr21	44485527	44485527	http://gnomad.b	0.00167595566	EAS	0.01503720041	8	1	0	https://www.ncbi.nlm.nih.gov/clinvar/variation/136668/
NM_014363.4(SACS):c.5419G>A	concordant	unreportable	unreportable	448207	0	1	SCV000614961	Uncertain significance	Athena Diagnostics	8/17/2017	SACS	chr13	23912596	23912596	http://gnomad.b	0.0003082614057	EAS	0.002319110092	0	0	0	https://www.ncbi.nlm.nih.gov/clinvar/variation/448207/
NM_014363.4(SACS):c.2497G>A	concordant	unreportable	unreportable	311555	0	3	SCV000614945;S(	Uncertain significance;Unc	Athena Diagnostics	2017-08-17;2016-1	SACS	chr13	23915518	23915518	http://gnomad.b	0.0001452385469	AFR	0.001899649971	1	0	0	https://www.ncbi.nlm.nih.gov/clinvar/variation/311555/
NM_000271.4(NPC1):c.2973G>A	concordant	unreportable	unreportable	326254	0	1	SCV000407853	Uncertain significance	Illumina Clinical Se	10/18/2016	NPC1	chr18	21118574	21118574	http://gnomad.b	6.58E-05	NFE	0.000134271002	0	0	0	https://www.ncbi.nlm.nih.gov/clinvar/variation/326254/
NM_000520.4(HEXA):c.1216C>T	concordant	unreportable	unreportable	317042	0	1	SCV000393833	Uncertain significance	Illumina Clinical Se	10/18/2016	HEXA	chr15	72638982	72638982	http://gnomad.b	0.0002066028389	NFE	0.0003312090121	0	0	0	https://www.ncbi.nlm.nih.gov/clinvar/variation/317042/
NM_000543.4(SMPD1):c.1599G>A	concordant	unreportable	unreportable	286896	0	2	SCV000340485;S(	Likely benign;Uncertain sig	EGL Genetic Diagn	2017-06-30;2016-1	SMPD1	chr11	6415540	6415540	http://gnomad.b	0.0004352997146	SAS	0.004905459937	2	0	0	https://www.ncbi.nlm.nih.gov/clinvar/variation/286896/
NM_005476.5(GNE):c.1267A>G	concordant	unreportable	unreportable	284451	0	3	SCV000337083;S(	Uncertain significance;Likel	EGL Genetic Diagn	2017-06-30;2016-1	GNE	chr9	36227259	36227259	http://gnomad.b	0.0002073177565	AFR	0.001503270003	0	0	0	https://www.ncbi.nlm.nih.gov/clinvar/variation/284451/
NM_000053.3(ATP7B):c.1426G>A	concordant	unreportable	unreportable	284672	0	1	SCV000337379	Likely benign	EGL Genetic Diagn	6/30/2017	ATP7B	chr13	52544745	52544745	http://gnomad.b	0.000505061842	EAS	0.006899349857	0	0	0	https://www.ncbi.nlm.nih.gov/clinvar/variation/284672/
NM_004543.4(NEB):c.2354A>G	concordant	unreportable	unreportable	331527	0	1	SCV000417031	Uncertain significance	Illumina Clinical Se	10/18/2016	NEB	chr2	152544867	152544867	http://gnomad.b	6.45E-05	NFE	3.58E-05	0	0	0	https://www.ncbi.nlm.nih.gov/clinvar/variation/331527/
NM_000057.2(BLM):c.451A>G	concordant	unreportable	unreportable	236822	0	2	SCV000394410;S(	Uncertain significance;Unc	Illumina Clinical Se	2016-10-18;2017-1	BLM	chr15	91292949	91292949	http://gnomad.b	3.33E-05	SAS	6.50E-05	0	0	0	https://www.ncbi.nlm.nih.gov/clinvar/variation/236822/
NM_000492.3(CFTR):c.1581A>G	concordant	unreportable	unreportable	35826	0	3	SCV000602996;S(	Benign;Likely benign;Benig	ARUP Laboratories	2017-06-30;2016-1	CFTR	chr7	117199706	117199706	http://gnomad.b	0.001586885852	AFR	0.01673859917	1	8	0	https://www.ncbi.nlm.nih.gov/clinvar/variation/35826/
NM_000057.2(BLM):c.3798T>G	concordant	unreportable	unreportable	136518	0	3	SCV000337299;S(	Uncertain significance;Likel	EGL Genetic Diagn	2017-06-30;2017-0	BLM	chr15	91352413	91352413	http://gnomad.b	0.0007372877325	ASJ	0.003350249957	0	0	0	https://www.ncbi.nlm.nih.gov/clinvar/variation/136518/
NM_000360.3(TH):c.115C>G	concordant	unreportable	unreportable	304085	0	1	SCV000369933	Uncertain significance	Illumina Clinical Se	10/18/2016	TH	chr11	2191077	2191077		1.13E-05				0	0	https://www.ncbi.nlm.nih.gov/clinvar/variation/304085/
NM_000137.2(FAH):c.855G>A	concordant	unreportable	unreportable	317215	0	2	SCV000394053;S(	Likely benign;Benign	Illumina Clinical Se	2016-10-18;2017-1	FAH	chr15	80467375	80467375	http://gnomad.b	0.002408020818	AFR	0.02901200019	8	6	0	https://www.ncbi.nlm.nih.gov/clinvar/variation/317215/
NM_138694.3(PKHD1):c.2243C>T	concordant	unreportable	unreportable	262394	0	2	SCV000338551;S(	Uncertain significance;Unc	EGL Genetic Diagn	2017-06-30;2017-0	PKHD1	chr6	51914991	51914991	http://gnomad.b	0.0002057178441	AFR	0.001830299967	0	0	0	https://www.ncbi.nlm.nih.gov/clinvar/variation/262394/
NM_033056.3(PCDH15):c.1339G>A	concordant	unreportable	unreportable	46441	0	1	SCV000604612	Uncertain significance	ARUP Laboratories	6/30/2017	PCDH15	chr10	55944995	55944995	http://gnomad.b	0.0003368821684	NFE	0.0004393040144	0	0	0	https://www.ncbi.nlm.nih.gov/clinvar/variation/46441/
NM_000152.3(GAA):c.1286A>G	concordant	unreportable	unreportable	284497	0	3	SCV000337146;S(	Benign;Likely benign;Benig	EGL Genetic Diagn	2017-06-30;2017-1	GAA	chr17	78082587	78082587	http://gnomad.b	0.001003329609	EAS	0.01046869997	1	3	0	https://www.ncbi.nlm.nih.gov/clinvar/variation/284497/
NM_000151.3(G6PC):c.694G>A	concordant	unreportable	unreportable	214457	0	1	SCV000706732	Uncertain significance	EGL Genetic Diagn	6/30/2017	G6PC	chr17	41063063	41063063	http://gnomad.b	0.0002325735929	NFE	0.000788615027	2	0	0	https://www.ncbi.nlm.nih.gov/clinvar/variation/214457/
NM_000228.2(LAMB3):c.1667G>A	concordant	unreportable	unreportable	295101	0	1	SCV000353625	Uncertain significance	Illumina Clinical Se	10/18/2016	LAMB3	chr1	209799302	209799302	http://gnomad.b	0.0006690891988	SAS	0.008292679675	2	1	0	https://www.ncbi.nlm.nih.gov/clinvar/variation/295101/
NM_017890.4(VPS13B):c.1700G>A	concordant	unreportable	unreportable	95834	0	5	SCV000262849;S(	Uncertain significance;Unc	Ambry Genetics;Fu	2017-11-14;2017-0	VPS13B	chr8	100155250	100155250	http://gnomad.b	0.0005319297853	NFE	0.0007629480097	0	0	0	https://www.ncbi.nlm.nih.gov/clinvar/variation/95834/
NM_000642.2(AGL):c.2917C>T	concordant	unreportable	unreportable	388494	0	2	SCV000530808;S(	Uncertain significance;Unc	GeneDx;Invitae	2017-11-28;2017-1	AGL	chr1	100356880	100356880	http://gnomad.b	8.37E-06	EAS	5.80E-05	0	0	0	https://www.ncbi.nlm.nih.gov/clinvar/variation/388494/
NM_000466.2(PEX1):c.1077G>A	concordant	unreportable	unreportable	497945	0	1	SCV000702722	Uncertain significance	EGL Genetic Diagn	6/30/2017	PEX1	chr7	92146752	92146752	http://gnomad.b	0.0004298844582	AFR	0.005295500159	1	0	0	https://www.ncbi.nlm.nih.gov/clinvar/variation/497945/
NM_000492.3(CFTR):c.3468G>T	concordant	unreportable	unreportable	455775	0	2	SCV000625745;S(	Likely benign;Uncertain sig	Invitae;Laboratory	2017-10-05;2018-0	CFTR	chr7	117254767	117254767	http://gnomad.b	0.0002060640609	EAS	0.001798980054	0	0	0	https://www.ncbi.nlm.nih.gov/clinvar/variation/455775/
NM_000057.2(BLM):c.3427G>A	concordant	unreportable	unreportable	127499	0	1	SCV000543395	Uncertain significance	Invitae	10/5/2017	BLM	chr15	91346819	91346819	http://gnomad.b	0.0001094039861	NFE	0.0001881049975	0	0	0	https://www.ncbi.nlm.nih.gov/clinvar/variation/127499/
NM_000060.2(BTD):c.108C>T	concordant	unreportable	unreportable	343905	0	1	SCV000441822	Uncertain significance	Illumina Clinical Se	10/18/2016	BTD	chr3	15676994	15676994	http://gnomad.b	5.19E-05	NFE	0.000134382004	0	0	0	https://www.ncbi.nlm.nih.gov/clinvar/variation/343905/
NM_017739.3(POMGNT1):c.1510G>A	concordant	unreportable	unreportable	281057	0	5	SCV000331162;S(	Benign;Likely benign;Uncer	EGL Genetic Diagn	2017-06-30;2017-1	POMGNT1	chr1	46657799	46657799	http://gnomad.b	0.0007446850206	AFR	0.008562089875	1	1	0	https://www.ncbi.nlm.nih.gov/clinvar/variation/281057/
NM_138694.3(PKHD1):c.3627G>A	concordant	unreportable	unreportable	501703	0	1	SCV000708180	Uncertain significance	EGL Genetic Diagn	6/30/2017	PKHD1	chr6	51892628	51892628	http://gnomad.b	0.0001695782228	AFR	0.001764480025	0	0	0	https://www.ncbi.nlm.nih.gov/clinvar/variation/501703/
NM_000018.3(ACADVL):c.1066A>G	concordant	unreportable	unreportable	193541	0	2	SCV000511944;S(	Likely benign;Benign	GeneDx;Invitae	2017-11-28;2017-1	ACADVL	chr17	7126173	7126173	http://gnomad.b	0.0007928866738	AFR	0.01052290015	1	1	0	https://www.ncbi.nlm.nih.gov/clinvar/variation/193541/
NM_001042432.1(CLN3):c.240G>A	concordant	unreportable	unreportable	197712	0	1	SCV000230989	Benign	EGL Genetic Diagn	6/30/2017	CLN3	chr16	28499966	28499966	http://gnomad.b	0.0007444279016	SAS	0.007764279842	3	2	0	https://www.ncbi.nlm.nih.gov/clinvar/variation/197712/
NM_000027.3(AGA):c.965C>T	concordant	unreportable	unreportable	387711	0	2	SCV000529834;S(	Likely benign;Benign	GeneDx;Invitae	2017-11-28;2017-1	AGA	chr4	178352938	178352938	http://gnomad.b	0.001081904626	AFR	0.01197019964	2	1	0	https://www.ncbi.nlm.nih.gov/clinvar/variation/387711/
NM_138694.3(PKHD1):c.5193G>A	concordant	unreportable	unreportable	499807	0	1	SCV000705485	Uncertain significance	EGL Genetic Diagn	6/30/2017	PKHD1	chr6	51889415	51889415	http://gnomad.b	0.0001306586307	NFE	0.000152528999	0	0	0	https://www.ncbi.nlm.nih.gov/clinvar/variation/499807/
NM_000152.3(GAA):c.1332T>C	concordant	unreportable	unreportable	286018	0	2	SCV000339272;S(	Benign;Benign	EGL Genetic Diagn	2017-06-30;2017-1	GAA	chr17	78083749	78083749	http://gnomad.b	0.0008032195485	EAS	0.009352849796	1	1	0	https://www.ncbi.nlm.nih.gov/clinvar/variation/286018/
NM_000391.3(TPP1):c.293C>T	concordant	unreportable	unreportable	130609	0	2	SCV000373335;S(	Uncertain significance;Beni	Illumina Clinical Se	2016-10-18;2017-1	TPP1	chr11	6638944	6638944	http://gnomad.b	0.001376601138	AFR	0.003528489964	2	1	0	https://www.ncbi.nlm.nih.gov/clinvar/variation/130609/
NM_005476.5(GNE):c.804G>A	concordant	unreportable	unreportable	285652	0	3	SCV000338780;S(	Uncertain significance;Likel	EGL Genetic Diagn	2017-06-30;2017-1	GNE	chr9	36234095	36234095	http://gnomad.b	0.0001596907043	NFE	0.0003402089933	0	0	0	https://www.ncbi.nlm.nih.gov/clinvar/variation/285652/
NM_000528.3(MAN2B1):c.719G>A	concordant	unreportable	unreportable	328277	0	1	SCV000410807	Uncertain significance	Illumina Clinical Se	10/18/2016	MAN2B1	chr19	12774561	12774561	http://gnomad.b	0.0008000090142	EAS	0.009805059992	1	1	0	https://www.ncbi.nlm.nih.gov/clinvar/variation/328277/
NM_000153.3(GALC):c.28T>C	concordant	unreportable	unreportable	314764	0	1	SCV000389273	Uncertain significance	Illumina Clinical Se	10/18/2016	GALC	chr14	88459481	88459481	http://gnomad.b	0.000123071415	EAS	0.0007433840074	0	0	0	https://www.ncbi.nlm.nih.gov/clinvar/variation/314764/
NM_000053.3(ATP7B):c.-74C>T	concordant	unreportable	unreportable	312404	0	1	SCV000384692	Uncertain significance	Illumina Clinical Se	10/18/2016	ATP7B	chr13	52585547	52585547		0.0001346831579				0	0	https://www.ncbi.nlm.nih.gov/clinvar/variation/312404/
NM_005562.2(LAMC2):c.2681G>A	concordant	unreportable	unreportable	294011	0	1	SCV000351684	Uncertain significance	Illumina Clinical Se	10/18/2016	LAMC2	chr1	183206566	183206566	http://gnomad.b	0.0008035119351	NFE	0.0009140929906	0	1	0	https://www.ncbi.nlm.nih.gov/clinvar/variation/294011/
NM_000136.2(FANCC):c.823T>C	concordant	unreportable	unreportable	219885	0	2	SCV000566691;S(	Uncertain significance;Unc	GeneDx;Invitae	2017-11-28;2016-0	FANCC	chr9	97897648	97897648	http://gnomad.b	2.14E-05	NFE	2.69E-05	0	0	0	https://www.ncbi.nlm.nih.gov/clinvar/variation/219885/
NM_033056.3(PCDH15):c.4974A>C	concordant	unreportable	unreportable	178519	0	1	SCV000204953	Likely benign	Laboratory for Mol	4/26/2016	PCDH15	chr10	55582512	55582512	http://gnomad.b	0.0002134444273	NFE	0.0005373940221	0	0	0	https://www.ncbi.nlm.nih.gov/clinvar/variation/178519/
NM_005476.5(GNE):c.1245C>T	concordant	unreportable	unreportable	284782	0	1	SCV000342858	Likely benign	EGL Genetic Diagn	11/3/2016	GNE	chr9	36227281	36227281	http://gnomad.b	0.0004314450608	AFR	0.001830299967	0	0	0	https://www.ncbi.nlm.nih.gov/clinvar/variation/284782/
NM_000051.3(ATM):c.2561C>A	concordant	unreportable	unreportable	420385	0	1	SCV000569205	Uncertain significance	GeneDx	11/28/2017	ATM	chr11	108137992	108137992	http://gnomad.b	5.57E-06	AMR	5.96E-05	0	0	0	https://www.ncbi.nlm.nih.gov/clinvar/variation/420385/
NM_000050.4(ASS1):c.515C>T	concordant	unreportable	unreportable	365256	0	1	SCV000477625	Uncertain significance	Illumina Clinical Se	10/18/2016	ASS1	chr9	133346240	133346240	http://gnomad.b	4.18E-05	NFE	7.16E-05	0	0	0	https://www.ncbi.nlm.nih.gov/clinvar/variation/365256/
NM_018941.3(CLN8):c.318C>T	concordant	unreportable	unreportable	377699	0	1	SCV000512651	Likely benign	GeneDx	11/28/2017	CLN8	chr8	1719538	1719538	http://gnomad.b	0.0002350570853	AMR	0.0006253349711	0	1	0	https://www.ncbi.nlm.nih.gov/clinvar/variation/377699/
NM_000271.4(NPC1):c.66A>G	concordant	unreportable	unreportable	255701	0	1	SCV000345699	Likely benign	EGL Genetic Diagn	6/30/2017	NPC1	chr18	21153530	21153530	http://gnomad.b	0.0009978613173	AMR	0.002203559969	0	3	0	https://www.ncbi.nlm.nih.gov/clinvar/variation/255701/
NM_002485.4(NBN):c.1882G>A	concordant	unreportable	unreportable	132690	0	4	SCV000685740;S(	Benign;Benign;Likely benig	Color Genomics, In	2017-10-26;2017-1	NBN	chr8	90960084	90960084	http://gnomad.b	0.00131344116	AFR	0.01238180045	0	6	0	https://www.ncbi.nlm.nih.gov/clinvar/variation/132690/
NM_000136.2(FANCC):c.705C>T	concordant	unreportable	unreportable	137276	0	2	SCV000481133;S(	Uncertain significance;Likel	Illumina Clinical Se	2016-10-18;2017-1	FANCC	chr9	97897766	97897766	http://gnomad.b	0.0004613533348	NFE	0.000636360026	0	0	0	https://www.ncbi.nlm.nih.gov/clinvar/variation/137276/
NM_005562.2(LAMC2):c.1491T>G	concordant	unreportable	unreportable	294004	0	1	SCV000351675	Uncertain significance	Illumina Clinical Se	10/18/2016	LAMC2	chr1	183197531	183197531	http://gnomad.b	0.0008119110146	EAS	0.008235700428	0	2	0	https://www.ncbi.nlm.nih.gov/clinvar/variation/294004/
NM_000543.4(SMPD1):c.1165C>T	concordant	unreportable	unreportable	289909	0	1	SCV000344354	Uncertain significance	EGL Genetic Diagn	6/30/2017	SMPD1	chr11	6414519	6414519	http://gnomad.b	0.0002521408183	ASJ	0.002436550101	0	0	0	https://www.ncbi.nlm.nih.gov/clinvar/variation/289909/
NM_000153.3(GALC):c.1236T>A	concordant	unreportable	unreportable	456713	0	1	SCV000627124	Benign	Invitae	10/5/2017	GALC	chr14	88417018	88417018	http://gnomad.b	0.0008055583526	AFR	0.01135919988	0	1	0	https://www.ncbi.nlm.nih.gov/clinvar/variation/456713/
NM_138694.3(PKHD1):c.11800G>A	concordant	unreportable	unreportable	289543	0	2	SCV000343923;S(	Uncertain significance;Beni	EGL Genetic Diagn	2017-06-30;2017-1	PKHD1	chr6	51484304	51484304	http://gnomad.b	0.0005643340858	NFE	0.000828933029	1	0	0	https://www.ncbi.nlm.nih.gov/clinvar/variation/289543/
NM_000153.3(GALC):c.18C>G	concordant	unreportable	unreportable	255372	0	1	SCV000389274	Uncertain significance	Illumina Clinical Se	10/18/2016	GALC	chr14	88459491	88459491	http://gnomad.b	5.03E-05	NFE	6.66E-05	0	0	0	https://www.ncbi.nlm.nih.gov/clinvar/variation/255372/
NM_000016.4(ACADM):c.210T>G	concordant	unreportable	unreportable	384767	0	1	SCV000525791	Likely benign	GeneDx	11/28/2017	ACADM	chr1	76198420	76198420	http://gnomad.b	0.0001006276993	AFR	0.0001308219944	0	0	0	https://www.ncbi.nlm.nih.gov/clinvar/variation/384767/
NM_000152.3(GAA):c.2051C>T	concordant	unreportable	unreportable	285821	0	2	SCV000339004;S(	Uncertain significance;Unc	EGL Genetic Diagn	2017-06-30;2016-1	GAA	chr17	78087027	78087027	http://gnomad.b	9.17E-05	NFE	0.0001410039986	0	0	0	https://www.ncbi.nlm.nih.gov/clinvar/variation/285821/
NM_000051.3(ATM):c.5034G>C	concordant	unreportable	unreportable	453556	0	2	SCV000687602;S(	Likely benign;Likely benign	Color Genomics, In	2017-12-21;2017-1	ATM	chr11	108170469	108170469		2.78E-06				0	0	https://www.ncbi.nlm.nih.gov/clinvar/variation/453556/
NM_000051.3(ATM):c.4665C>T	concordant	unreportable	unreportable	184293	0	4	SCV000682217;S(	Likely benign;Uncertain sig	Color Genomics, In	2017-10-26;2017-0	ATM	chr11	108164093	108164093	http://gnomad.b	4.46E-05	NFE	9.90E-05	0	0	0	https://www.ncbi.nlm.nih.gov/clinvar/variation/184293/
NM_033056.3(PCDH15):c.521A>G	concordant	unreportable	unreportable	290793	0	1	SCV000345423	Uncertain significance	EGL Genetic Diagn	6/30/2017	PCDH15	chr10	56106198	56106198	http://gnomad.b	0.0001517255568	AMR	0.001072069979	0	1	0	https://www.ncbi.nlm.nih.gov/clinvar/variation/290793/
NM_006493.2(CLN5):c.52C>T	concordant	unreportable	unreportable	421520	0	1	SCV000570755	Uncertain significance	GeneDx	11/28/2017	CLN5	chr13	77566138	77566138	http://gnomad.b	5.59E-05	SAS	0.0004802090116	1	1	0	https://www.ncbi.nlm.nih.gov/clinvar/variation/421520/
NM_033056.3(PCDH15):c.2884C>T	concordant	unreportable	unreportable	164914	0	1	SCV000363175	Uncertain significance	Illumina Clinical Se	10/18/2016	PCDH15	chr10	55721637	55721637	http://gnomad.b	0.001275523325	EAS	0.01175099984	0	5	0	https://www.ncbi.nlm.nih.gov/clinvar/variation/164914/
NM_000232.4(SGCB):c.392G>A	concordant	unreportable	unreportable	284821	0	2	SCV000337609;S(	Uncertain significance;Unc	EGL Genetic Diagn	2017-06-30;2016-1	SGCB	chr4	52895881	52895881	http://gnomad.b	8.68E-05	AMR	0.0001191109986	0	0	0	https://www.ncbi.nlm.nih.gov/clinvar/variation/284821/
NM_000051.3(ATM):c.78A>T	concordant	unreportable	unreportable	186439	0	2	SCV000293606;S(	Uncertain significance;Unc	GeneDx;Invitae	2017-11-28;2017-1	ATM	chr11	108098508	108098508	http://gnomad.b	1.95E-05	NFE	8.96E-06	0	0	0	https://www.ncbi.nlm.nih.gov/clinvar/variation/186439/
NM_000352.3(ABCC8):c.3345G>A	concordant	unreportable	unreportable	210075	0	1	SCV000246295	Uncertain significance	Genetic Services La	9/15/2015	ABCC8	chr11	17427095	17427095	http://gnomad.b	0.0002606895626	EAS	0.003884509904	0	0	0	https://www.ncbi.nlm.nih.gov/clinvar/variation/210075/
NM_000057.2(BLM):c.842A>C	concordant	unreportable	unreportable	127517	0	1	SCV000543345	Uncertain significance	Invitae	10/5/2017	BLM	chr15	91295059	91295059	http://gnomad.b	0.0001260524188	NFE	0.0001882289944	0	0	0	https://www.ncbi.nlm.nih.gov/clinvar/variation/127517/
NM_000051.3(ATM):c.4731T>C	concordant	unreportable	unreportable	185156	0	3	SCV000682218;S(	Likely benign;Likely benign	Color Genomics, In	2017-10-26;2017-1	ATM	chr11	108164159	108164159	http://gnomad.b	1.67E-05	AFR	6.54E-05	0	0	0	https://www.ncbi.nlm.nih.gov/clinvar/variation/185156/
NM_000071.2(CBS):c.133C>T	concordant	unreportable	unreportable	212871	0	2	SCV000249718;S(	Uncertain significance;Likel	GeneDx;Invitae	2017-11-28;2017-0	CBS	chr21	44492171	44492171	http://gnomad.b	0.0006411374002	SAS	0.003931120038	1	1	0	https://www.ncbi.nlm.nih.gov/clinvar/variation/212871/
NM_000152.3(GAA):c.676C>G	concordant	unreportable	unreportable	188476	0	3	SCV000228797;S(	Likely benign;Likely benign	EGL Genetic Diagn	2017-06-30;2017-1	GAA	chr17	78079677	78079677	http://gnomad.b	0.0006559163095	AFR	0.006199710071	1	0	0	https://www.ncbi.nlm.nih.gov/clinvar/variation/188476/
NM_017890.4(VPS13B):c.2075G>A	concordant	unreportable	unreportable	194549	0	2	SCV000226341;S(	Uncertain significance;Unc	EGL Genetic Diagn	2017-06-30;2015-0	VPS13B	chr8	100168838	100168838	http://gnomad.b	5.32E-05	NFE	0.0001254550007	0	0	0	https://www.ncbi.nlm.nih.gov/clinvar/variation/194549/
NM_024685.3(BBS10):c.966T>C	concordant	unreportable	unreportable	261758	0	3	SCV000593590;S(	Benign;Uncertain significar	Genetic Services La	2017-07-05;2016-1	BBS10	chr12	76740799	76740799	http://gnomad.b	0.001494040624	AFR	0.01815680042	0	9	0	https://www.ncbi.nlm.nih.gov/clinvar/variation/261758/
NM_017739.3(POMGNT1):c.706G>C	concordant	unreportable	unreportable	285234	0	2	SCV000338171;S(	Uncertain significance;Unc	EGL Genetic Diagn	2017-06-30;2017-1	POMGNT1	chr1	46660270	46660270	http://gnomad.b	3.08E-05	AMR	8.93E-05	0	0	0	https://www.ncbi.nlm.nih.gov/clinvar/variation/285234/
NM_000528.3(MAN2B1):c.2625C>T	concordant	unreportable	unreportable	328256	0	1	SCV000410776	Uncertain significance	Illumina Clinical Se	10/18/2016	MAN2B1	chr19	12759028	12759028	http://gnomad.b	0.0001352127911	AFR	0.001900719944	0	0	0	https://www.ncbi.nlm.nih.gov/clinvar/variation/328256/
NM_000057.2(BLM):c.696C>A	concordant	unreportable	unreportable	127514	0	2	SCV000394411;S(	Uncertain significance;Unc	Illumina Clinical Se	2016-10-18;2017-1	BLM	chr15	91293194	91293194	http://gnomad.b	0.0001094039861	ASJ	0.000813339022	0	0	0	https://www.ncbi.nlm.nih.gov/clinvar/variation/127514/
NM_133647.1(SLC12A6):c.2003G>A	concordant	unreportable	unreportable	426922	0	1	SCV000577497	Uncertain significance	GeneDx	11/28/2017	SLC12A6	chr15	34536214	34536214	http://gnomad.b	2.67E-05	AMR	0.0001191109986	0	0	0	https://www.ncbi.nlm.nih.gov/clinvar/variation/426922/
NM_000383.3(AIRE):c.595G>A	concordant	unreportable	unreportable	128334	0	2	SCV000296910;S(	Uncertain significance;Beni	Division of Genomi	2016-01-06;2017-1	AIRE	chr21	45708284	45708284	http://gnomad.b	0.001495596763	AFR	0.01016530022	0	2	0	https://www.ncbi.nlm.nih.gov/clinvar/variation/128334/
NM_138694.3(PKHD1):c.3804C>T	concordant	unreportable	unreportable	414169	0	1	SCV000557648	Benign	Invitae	3/14/2017	PKHD1	chr6	51890804	51890804	http://gnomad.b	0.0003780760378	EAS	0.003718340071	0	1	0	https://www.ncbi.nlm.nih.gov/clinvar/variation/414169/
NM_005476.5(GNE):c.843C>T	concordant	unreportable	unreportable	283937	0	2	SCV000336319;S(	Uncertain significance;Likel	EGL Genetic Diagn	2017-06-30;2017-1	GNE	chr9	36234056	36234056	http://gnomad.b	0.0001652938869	NFE	0.0003133220016	0	0	0	https://www.ncbi.nlm.nih.gov/clinvar/variation/283937/
NM_000051.3(ATM):c.1595G>A	concordant	unreportable	unreportable	133603	0	4	SCV000611345;S(	Uncertain significance;Unc	Fulgent Genetics,F	2017-05-23;2017-1	ATM	chr11	108121787	108121787	http://gnomad.b	0.0002534084833	AMR	0.0008041940164	0	0	0	https://www.ncbi.nlm.nih.gov/clinvar/variation/133603/
NM_000136.2(FANCC):c.1595G>A	concordant	unreportable	unreportable	216285	0	2	SCV000618137;S(	Uncertain significance;Unc	GeneDx;Invitae	2017-11-28;2017-1	FANCC	chr9	97864071	97864071	http://gnomad.b	4.76E-05	EAS	0.000927858986	0	0	0	https://www.ncbi.nlm.nih.gov/clinvar/variation/216285/
NM_017890.4(VPS13B):c.9720C>T	concordant	unreportable	unreportable	197953	0	2	SCV000231327;S(	Uncertain significance;Unc	EGL Genetic Diagn	2017-06-30;2015-0	VPS13B	chr8	100847455	100847455	http://gnomad.b	5.32E-05	NFE	0.0001257160038	0	0	0	https://www.ncbi.nlm.nih.gov/clinvar/variation/197953/

Supplemental Table 2

NM_000528.3(MAN2B1):c.597C>T	concordant	unreportable	unreportable	328280	0	1	SCV000410810	Uncertain significance	Illumina Clinical Se	10/18/2016	MAN2B1	chr19	12775639	12775639	http://gnomad.b	1.69E-05	NFE	2.71E-05	0		0	0	https://www.ncbi.nlm.nih.gov/clinvar/variation/328280/
NM_000023.2(SGCA):c.80C>T	concordant	unreportable	unreportable	283448	0	1	SCV000335531	Uncertain significance	EGL Genetic Diagn	6/30/2017	SGCA	chr17	48244771	48244771	http://gnomad.b	0.000464564373	SAS	0.007699300069	3		1	0	https://www.ncbi.nlm.nih.gov/clinvar/variation/283448/
NM_000478.4(ALPL):c.1014C>T	concordant	unreportable	unreportable	295547	0	1	SCV000354309	Uncertain significance	Illumina Clinical Se	10/18/2016	ALPL	chr1	21902242	21902242	http://gnomad.b	0.0001042347479	EAS	0.001101579983	0		0	0	https://www.ncbi.nlm.nih.gov/clinvar/variation/295547/
NM_000466.2(PEX1):c.468A>G	concordant	unreportable	unreportable	285002	0	2	SCV000337837;S	Likely benign;Benign	EGL Genetic Diagn	2017-06-30;2017-1	PEX1	chr7	92147459	92147459	http://gnomad.b	0.0006961354774	AFR	0.007579720113	0		0	0	https://www.ncbi.nlm.nih.gov/clinvar/variation/285002/
NM_000071.2(CBS):c.1200_1202delG	concordant	unreportable	unreportable	340082	0	1	SCV000436207	Uncertain significance	Illumina Clinical Se	10/18/2016	CBS	chr21	44479356	44479359	http://gnomad.b	1.12E-05	AMR	0.0001786780049	0		0	0	https://www.ncbi.nlm.nih.gov/clinvar/variation/340082/
NM_000053.3(ATP7B):c.3369G>A	concordant	unreportable	unreportable	92388	0	3	SCV000384657;S	Uncertain significance;Beni	Illumina Clinical Se	2016-10-18;2017-1	ATP7B	chr13	52516565	52516565	http://gnomad.b	0.0009568116007	AFR	0.01164459996	0		4	0	https://www.ncbi.nlm.nih.gov/clinvar/variation/92388/
NM_001360.2(DHCR7):c.122C>T	concordant	unreportable	unreportable	286285	0	1	SCV000339647	Uncertain significance	EGL Genetic Diagn	6/30/2017	DHCR7	chr11	71155238	71155238	http://gnomad.b	3.50E-05	SAS	0.0001082329982	0		0	0	https://www.ncbi.nlm.nih.gov/clinvar/variation/286285/
NM_000057.2(BLM):c.3879A>G	concordant	unreportable	unreportable	236819	0	2	SCV000394429;S	Uncertain significance;Beni	Illumina Clinical Se	2016-10-18;2017-1	BLM	chr15	91354439	91354439	http://gnomad.b	0.0004518860296	AFR	0.003136429936	0		1	0	https://www.ncbi.nlm.nih.gov/clinvar/variation/236819/
NM_017890.4(VPS13B):c.3690T>C	concordant	unreportable	unreportable	195901	0	1	SCV000228258	Uncertain significance	EGL Genetic Diagn	6/30/2017	VPS13B	chr8	100493850	100493850		2.24E-05					0	0	https://www.ncbi.nlm.nih.gov/clinvar/variation/195901/
NM_000492.3(CFTR):c.393T>C	concordant	unreportable	unreportable	416592	0	1	SCV000562303	Likely benign	Invitae	3/14/2017	CFTR	chr7	117171072	117171072	http://gnomad.b	9.63E-06	NFE	1.80E-05	0		0	0	https://www.ncbi.nlm.nih.gov/clinvar/variation/416592/
NM_000057.3(BLM):c.387_389delGAA	concordant	unreportable	unreportable	421741	0	1	SCV000571037	Uncertain significance	GeneDx	11/28/2017	BLM	chr15	91292882	91292885	http://gnomad.b	0.0001355658089	SAS	0.002241570037	0		1	0	https://www.ncbi.nlm.nih.gov/clinvar/variation/421741/
NM_000016.4(ACADM):c.645T>A	concordant	unreportable	unreportable	386718	0	1	SCV000528466	Likely benign	GeneDx	11/28/2017	ACADM	chr1	76211536	76211536	http://gnomad.b	0.0006228038685	AMR	0.0008040500106	0		0	0	https://www.ncbi.nlm.nih.gov/clinvar/variation/386718/
NM_024649.4(BBS1):c.190C>G	concordant	unreportable	unreportable	305457	0	1	SCV000373258	Uncertain significance	Illumina Clinical Se	10/18/2016	BBS1	chr11	66281907	66281907	http://gnomad.b	0.0001337568968	ASJ	0.0009140769835	0		0	0	https://www.ncbi.nlm.nih.gov/clinvar/variation/305457/
NM_000051.3(ATM):c.7983T>C	concordant	unreportable	unreportable	184187	0	3	SCV000537478;S	Likely benign;Likely benign	Color Genomics, In	2017-01-26;2017-1	ATM	chr11	108204668	108204668	http://gnomad.b	5.01E-05	NFE	0.0001883509976	0		0	0	https://www.ncbi.nlm.nih.gov/clinvar/variation/184187/
NM_000071.2(CBS):c.1272C>T	concordant	unreportable	unreportable	236934	0	1	SCV000283381	Likely benign	Invitae	6/10/2016	CBS	chr21	44479030	44479030	http://gnomad.b	9.00E-05	AMR	0.0005843249965	0		0	0	https://www.ncbi.nlm.nih.gov/clinvar/variation/236934/
NM_000383.3(AIRE):c.1483C>T	concordant	unreportable	unreportable	458616	0	1	SCV000629951	Benign	Invitae	10/5/2017	AIRE	chr21	45714366	45714366	http://gnomad.b	0.0007074144836	EAS	0.01059169974	0		1	0	https://www.ncbi.nlm.nih.gov/clinvar/variation/458616/
NM_017890.4(VPS13B):c.2643A>T	concordant	unreportable	unreportable	288192	0	3	SCV000342231;S	Benign;Uncertain significar	EGL Genetic Diagn	2017-06-30;2017-0	VPS13B	chr8	100286553	100286553	http://gnomad.b	0.0006915087208	AFR	0.00686095003	1		0	0	https://www.ncbi.nlm.nih.gov/clinvar/variation/288192/
NM_000152.3(GAA):c.2156C>A	concordant	unreportable	unreportable	194458	0	3	SCV000226203;S	Uncertain significance;Unc	EGL Genetic Diagn	2017-06-30;2017-1	GAA	chr17	78087132	78087132	http://gnomad.b	0.0001639790774	NFE	0.0002773389861	0		0	0	https://www.ncbi.nlm.nih.gov/clinvar/variation/194458/
NM_002485.4(NBN):c.696C>T	concordant	unreportable	unreportable	378235	0	2	SCV000513857;S	Likely benign;Likely benign	GeneDx;Invitae	2017-11-28;2017-1	NBN	chr8	90983407	90983407	http://gnomad.b	2.23E-05	AMR	0.0001793289994	0		0	0	https://www.ncbi.nlm.nih.gov/clinvar/variation/378235/
NM_000152.3(GAA):c.2736G>A	concordant	unreportable	unreportable	384066	0	2	SCV000703073;S	Uncertain significance;Likel	EGL Genetic Diagn	2017-06-30;2017-1	GAA	chr17	78092541	78092541	http://gnomad.b	8.89E-05	AFR	0.000261609006	0		0	0	https://www.ncbi.nlm.nih.gov/clinvar/variation/384066/
NM_000051.3(ATM):c.8592C>T	concordant	unreportable	unreportable	135782	0	3	SCV000537453;S	Likely benign;Likely benign	Color Genomics, In	2017-01-26;2017-1	ATM	chr11	108218013	108218013	http://gnomad.b	0.0005485875958	NFE	0.0009047639905	0		0	0	https://www.ncbi.nlm.nih.gov/clinvar/variation/135782/
NM_000137.2(FAH):c.1205G>A	concordant	unreportable	unreportable	287498	0	1	SCV000341285	Likely benign	EGL Genetic Diagn	6/30/2017	FAH	chr15	80478496	80478496	http://gnomad.b	0.000302389711	AFR	0.002352329902	1		0	0	https://www.ncbi.nlm.nih.gov/clinvar/variation/287498/
NM_000466.2(PEX1):c.1380A>C	concordant	unreportable	unreportable	360928	0	1	SCV000470519	Uncertain significance	Illumina Clinical Se	10/18/2016	PEX1	chr7	92140997	92140997	http://gnomad.b	0.000260704123	SAS	0.004094100092	0		1	0	https://www.ncbi.nlm.nih.gov/clinvar/variation/360928/
NM_000303.2(PMM2):c.93C>T	concordant	unreportable	unreportable	290872	0	3	SCV000345529;S	Likely benign;Likely benign	EGL Genetic Diagn	2017-06-30;2017-1	PMM2	chr16	8895682	8895682	http://gnomad.b	0.0005985823343	AFR	0.006837490015	1		0	0	https://www.ncbi.nlm.nih.gov/clinvar/variation/290872/
NM_000152.3(GAA):c.362A>G	concordant	unreportable	unreportable	284483	0	1	SCV000337127	Uncertain significance	EGL Genetic Diagn	6/30/2017	GAA	chr17	78078747	78078747	http://gnomad.b	3.61E-05	AFR	0.0003334220091	0		0	0	https://www.ncbi.nlm.nih.gov/clinvar/variation/284483/
NM_018941.3(CLN8):c.200C>T	concordant	unreportable	unreportable	195345	0	1	SCV000240715	Uncertain significance	GeneDx	11/28/2017	CLN8	chr8	1719420	1719420	http://gnomad.b	0.0001343183345	AFR	0.0001306850027	0		0	0	https://www.ncbi.nlm.nih.gov/clinvar/variation/195345/
NM_004328.4(BCS1L):c.822G>A	concordant	unreportable	unreportable	334360	0	2	SCV000518682;S	Likely benign;Uncertain sig	GeneDx;Illumina C	2017-11-28;2016-1	BCS1L	chr2	219527335	219527335	http://gnomad.b	0.000150583931	EAS	0.0005219809827	0		0	0	https://www.ncbi.nlm.nih.gov/clinvar/variation/334360/
NM_000051.3(ATM):c.2051A>G	concordant	unreportable	unreportable	186816	0	3	SCV000682023;S	Uncertain significance;Unc	Color Genomics, In	2017-10-26;2017-1	ATM	chr11	108124693	108124693	http://gnomad.b	1.67E-05	AMR	0.0001191190022	0		0	0	https://www.ncbi.nlm.nih.gov/clinvar/variation/186816/
NM_014363.4(SACS):c.3427C>A	concordant	unreportable	unreportable	212112	0	2	SCV000383361;S	Uncertain significance;Unc	Illumina Clinical Se	2016-10-18;2017-0	SACS	chr13	23914588	23914588	http://gnomad.b	0.001221189415	ASJ	0.00447335979	0		1	0	https://www.ncbi.nlm.nih.gov/clinvar/variation/212112/
NM_000152.3(GAA):c.858+10C>T	concordant	unreportable	unreportable	325783	0	3	SCV000703111;S	Uncertain significance;Unc	EGL Genetic Diagn	2017-06-30;2016-1	GAA	chr17	78081531	78081531	http://gnomad.b	0.0001056136431	AMR	2.99E-05	0		0	0	https://www.ncbi.nlm.nih.gov/clinvar/variation/325783/
NM_000152.3(GAA):c.1352C>G	concordant	unreportable	unreportable	281330	0	2	SCV000332073;S	Likely benign;Likely benign	EGL Genetic Diagn	2017-06-30;2017-1	GAA	chr17	78083769	78083769	http://gnomad.b	0.0005058337641	AFR	0.004794429988	0		1	0	https://www.ncbi.nlm.nih.gov/clinvar/variation/281330/
NM_000071.2(CBS):c.1484C>T	concordant	unreportable	unreportable	405370	0	2	SCV000572209;S	Uncertain significance;Unc	GeneDx;Invitae	2017-11-28;2017-1	CBS	chr21	44476981	44476981	http://gnomad.b	0.0001293522825	NFE	0.0001490760042	0		0	0	https://www.ncbi.nlm.nih.gov/clinvar/variation/405370/
NM_017890.4(VPS13B):c.10459C>T	concordant	unreportable	unreportable	361089	0	2	SCV000597920;S	Uncertain significance;Unc	Genetic Services La	2017-07-05;2016-1	VPS13B	chr8	100866001	100866001	http://gnomad.b	3.08E-05	AFR	6.53E-05	0		0	0	https://www.ncbi.nlm.nih.gov/clinvar/variation/361089/
NM_033056.3(PCDH15):c.5353T>C	concordant	unreportable	unreportable	46497	0	2	SCV000363115;S	Uncertain significance;Likel	Illumina Clinical Se	2016-10-18;2016-0	PCDH15	chr10	55582133	55582133	http://gnomad.b	0.0005066090624	AFR	0.00406120019	0		1	0	https://www.ncbi.nlm.nih.gov/clinvar/variation/46497/
NM_000051.3(ATM):c.3925G>A	concordant	unreportable	unreportable	127377	0	5	SCV000602552;S	Uncertain significance;Unc	ARUP Laboratories	2017-06-30;2017-0	ATM	chr11	108155132	108155132	http://gnomad.b	0.0007407324898	NFE	0.001255430048	0		0	0	https://www.ncbi.nlm.nih.gov/clinvar/variation/127377/
NM_000155.3(GALT):c.329-24G>A	concordant	unreportable	unreportable	280995	0	1	SCV000331014	Likely benign	EGL Genetic Diagn	6/30/2017	GALT	chr9	34647630	34647630	http://gnomad.b	0.0004974796198	AFR	0.006534240209	0		1	0	https://www.ncbi.nlm.nih.gov/clinvar/variation/280995/
NM_000152.3(GAA):c.2408A>G	concordant	unreportable	unreportable	496900	0	1	SCV000700554	Uncertain significance	EGL Genetic Diagn	6/30/2017	GAA	chr17	78091475	78091475	http://gnomad.b	0.0001445239326	NFE	7.27E-05	0		0	0	https://www.ncbi.nlm.nih.gov/clinvar/variation/496900/
NM_012203.1(GRHPR):c.468C>T	concordant	unreportable	unreportable	366855	0	1	SCV000480134	Uncertain significance	Illumina Clinical Se	10/18/2016	GRHPR	chr9	37428544	37428544	http://gnomad.b	0.000406710727	SAS	0.0009096229915	0		1	0	https://www.ncbi.nlm.nih.gov/clinvar/variation/366855/
NM_024685.3(BBS10):c.1436C>A	concordant	unreportable	unreportable	499947	0	1	SCV000705687	Uncertain significance	EGL Genetic Diagn	6/30/2017	BBS10	chr12	76740329	76740329	http://gnomad.b	8.95E-05	AFR	0.002091499977	0		0	0	https://www.ncbi.nlm.nih.gov/clinvar/variation/499947/
NM_000035.3(ALDOB):c.906T>C	concordant	unreportable	unreportable	501716	0	1	SCV000708194	Uncertain significance	EGL Genetic Diagn	6/30/2017	ALDOB	chr9	104187218	104187218	http://gnomad.b	0.0003836043009	AFR	0.005424129777	2		0	0	https://www.ncbi.nlm.nih.gov/clinvar/variation/501716/
NM_000271.4(NPC1):c.1431G>A	concordant	unreportable	unreportable	500155	0	1	SCV000705975	Uncertain significance	EGL Genetic Diagn	6/30/2017	NPC1	chr18	21134844	21134844	http://gnomad.b	0.0002315953201	EAS	0.0005797769991	0		0	0	https://www.ncbi.nlm.nih.gov/clinvar/variation/500155/
NM_000051.3(ATM):c.4977A>T	concordant	unreportable	unreportable	302251	0	1	SCV000367057	Uncertain significance	Illumina Clinical Se	10/18/2016	ATM	chr11	108168081	108168081	http://gnomad.b	2.78E-06	SAS	6.50E-05	0		0	0	https://www.ncbi.nlm.nih.gov/clinvar/variation/302251/
NM_000528.3(MAN2B1):c.1822G>A	concordant	unreportable	unreportable	451935	0	1	SCV000620699	Uncertain significance	GeneDx	11/28/2017	MAN2B1	chr19	12766516	12766516	http://gnomad.b	0.0003746521088	FIN	0.008626139723	2		0	0	https://www.ncbi.nlm.nih.gov/clinvar/variation/451935/
NM_000051.3(ATM):c.2805G>C	concordant	unreportable	unreportable	135746	0	3	SCV000537452;S	Likely benign;Likely benign	Color Genomics, In	2017-01-26;2017-1	ATM	chr11	108139303	108139303	http://gnomad.b	0.0004177062912	NFE	0.0004924610257	1		0	0	https://www.ncbi.nlm.nih.gov/clinvar/variation/135746/
NM_000182.4(HADHA):c.1912A>G	concordant	unreportable	unreportable	335375	0	1	SCV000429500	Uncertain significance	Illumina Clinical Se	10/18/2016	HADHA	chr2	26415267	26415267	http://gnomad.b	3.36E-05	AFR	6.53E-05	0		0	0	https://www.ncbi.nlm.nih.gov/clinvar/variation/335375/
NM_000228.2(LAMB3):c.267C>T	concordant	unreportable	unreportable	295144	0	1	SCV000353674	Uncertain significance	Illumina Clinical Se	10/18/2016	LAMB3	chr1	209811910	209811910	http://gnomad.b	0.0002827531761	SAS	0.002209079918	1		0	0	https://www.ncbi.nlm.nih.gov/clinvar/variation/295144/
NM_000382.2(ALDH3A2):c.1108-3C>T	concordant	unreportable	unreportable	198723	0	1	SCV000232540	Uncertain significance	EGL Genetic Diagn	6/30/2017	ALDH3A2	chr17	19568258	19568258	http://gnomad.b	0.001049732108	EAS	0.007537109777	0		5	0	https://www.ncbi.nlm.nih.gov/clinvar/variation/198723/
NM_000492.3(CFTR):c.1364C>T	concordant	unreportable	unreportable	411125	0	1	SCV000552139	Uncertain significance	Invitae	3/14/2017	CFTR	chr7	117188849	117188849		7.90E-05					0	0	https://www.ncbi.nlm.nih.gov/clinvar/variation/411125/
NM_138694.3(PKHD1):c.5358C>T	concordant	unreportable	unreportable	289881	0	2	SCV000344314;S	Likely benign;Benign	EGL Genetic Diagn	2017-06-30;2017-1	PKHD1	chr6	51887621	51887621	http://gnomad.b	0.0003030168244	AFR	0.002942329971	0		0	0	https://www.ncbi.nlm.nih.gov/clinvar/variation/289881/
NM_000051.3(ATM):c.2522A>C	concordant	unreportable	unreportable	184552	0	5	SCV000682062;S	Uncertain significance;Unc	Color Genomics, In	2017-10-26;2017-0	ATM	chr11	108137953	108137953	http://gnomad.b	0.0001448048476	SAS	0.001429499942	1		1	0	https://www.ncbi.nlm.nih.gov/clinvar/variation/184552/
NM_000352.3(ABCC8):c.4090G>A	concordant	unreportable	unreportable	303754	0	1	SCV000369253	Likely benign	Illumina Clinical Se	10/18/2016	ABCC8	chr11	17418492	17418492	http://gnomad.b	0.0001729326801	AFR	0.001633769949	0		0	0	https://www.ncbi.nlm.nih.gov/clinvar/variation/303754/
NM_138694.3(PKHD1):c.4257G>A	concordant	unreportable	unreportable	287100	0	1	SCV000340765	Uncertain significance	EGL Genetic Diagn	6/30/2017	PKHD1	chr6	51890351	51890351		2.22E-05					0	0	https://www.ncbi.nlm.nih.gov/clinvar/variation/287100/
NM_000051.3(ATM):c.3281A>G	concordant	unreportable	unreportable	142339	0	2	SCV000367042;S	Uncertain significance;Unc	Illumina Clinical Se	2016-10-18;2017-1	ATM	chr11	108143576	108143576	http://gnomad.b	1.67E-05	SAS	6.50E-05	0		0	0	https://www.ncbi.nlm.nih.gov/clinvar/variation/142339/
NM_000492.3(CFTR):c.3672T>A	concordant	unreportable	unreportable	495934	0	1	SCV000696981	Uncertain significance	Laboratory Corpor	1/25/2018	CFTR	chr7	117267779	117267779	http://gnomad.b	0.0001348082641	SAS	0.001527359942	0		0	0	https://www.ncbi.nlm.nih.gov/clinvar/variation/495934/
NM_000155.3(GALT):c.414G>T	concordant	unreportable	unreportable	280996	0	2	SCV000331015;S	Benign;Likely benign	EGL Genetic Diagn	2017-06-30;2018-0	GALT	chr9	34647865	34647865	http://gnomad.b	0.0004205214466	AFR	0.006542790215	0		1	0	https://www.ncbi.nlm.nih.gov/clinvar/variation/280996/
NM_000352.3(ABCC8):c.1270G>A	concordant	unreportable	unreportable	389393	0	1	SCV000531909	Uncertain significance	GeneDx	11/28/2017	ABCC8	chr11	17470125	17470125	http://gnomad.b	2.06E-05	AFR	0.0002613700053	0		0	0	https://www.ncbi.nlm.nih.gov/clinvar/variation/389393/
NM_000136.2(FANCC):c.934A>G	concordant	unreportable	unreportable	127548	0	2	SCV000149271;S	Uncertain significance;Likel	GeneDx;Invitae	2017-07-13;2017-1	FANCC	chr9	97887430	97887430	http://gnomad.b	0.0004637314448	FIN	0.00139037997	0		0	0	https://www.ncbi.nlm.nih.gov/clinvar/variation/127548/
NM_000136.2(FANCC):c.840G>A	concordant	unreportable	unreportable	137278	0	1	SCV000260824	Benign	Invitae	10/5/2017	FANCC	chr9	97897631	97897631	http://gnomad.b	0.0005112936443	AFR	0.004839759786	0		2	0	https://www.ncbi.nlm.nih.gov/clinvar/variation/137278/
NM_017890.4(VPS13B):c.10127A>T	concordant	unreportable	unreportable	167835	0	1	SCV000597895	Likely benign	Genetic Services La	7/5/2017	VPS13B	chr8	100861113	100861113	http://gnomad.b	0.0005823231333	AFR	0.007449029945	1		0	0	https://www.ncbi.nlm.nih.gov/clinvar/variation/167835/
NM_138694.3(PKHD1):c.2781A>G	concordant	unreportable	unreportable	357442	0	2	SCV000702827;S	Uncertain significance;Unc	EGL Genetic Diagn	2017-06-30;2016-1	PKHD1	chr6	51908463	51908463	http://gnomad.b	0.0002112777858	AMR	0.0005068570026	0		0	0	https://www.ncbi.nlm.nih.gov/clinvar/variation/357442/
NM_024649.4(BBS1):c.1036G>A	concordant	unreportable	unreportable	305465	0	2	SCV000373271;S	Uncertain significance;Beni	Illumina Clinical Se	2016-10-18;2017-0	BBS1	chr11	66291279	66291279	http://gnomad.b	0.000629772056	SAS	0.00730946986	4		2	0	https://www.ncbi.nlm.nih.gov/clinvar/variation/305465/
NM_000360.3(TH):c.897C>T	concordant	unreportable	unreportable	304074	0	2	SCV000369916;S	Uncertain significance;Likel	Illumina Clinical Se	2016-10-18;2017-0	TH	chr11	2187943	2187943	http://gnomad.b	0.0006084815568	NFE	0.0008198359865	0		0	0	https://www.ncbi.nlm.nih.gov/clinvar/variation/304074/
NM_001042432.1(CLN3):c.318C>T	concordant	unreportable	unreportable	136788	0	2	SCV000231602;S	Likely benign;Benign	EGL Genetic Diagn	2017-06-30;2017-1	CLN3	chr16	28499039	28499039	http://gnomad.b	0.0003194373458	AFR	0.004344389774	1		0	0	https://www.ncbi.nlm.nih.gov/clinvar/variation/136788/
NM_000057.2(BLM):c.3044C>T	concordant	unreportable	unreportable	127494	0	1	SCV000543349	Uncertain significance	Invitae	10/5/2017	BLM	chr15	91337421	91337421	http://gnomad.b	0.0001284307663	NFE	0.0002254410065	0		0	0	https://www.ncbi.nlm.nih.gov/clinvar/variation/127494/
NM_001271208.1(NEB):c.11333T>C	concordant	unreportable	unreportable	435964	0	2	SCV000595953;S	Uncertain significance;Unc	Genetic Services La	2017-07-05;2017-1	NEB	chr2	152471058	152471058	http://gnomad.b	0.0001729675028	NFE	0.000152491004	0		0	0	https://www.ncbi.nlm.nih.gov/clinvar/variation/435964/
NM_000382.2(ALDH3A2):c.1188T>G	concordant	unreportable	unreportable	322217	0	1	SCV000401260	Uncertain significance	Illumina Clinical Se	10/18/2016	ALDH3A2	chr17	19568341	19568341	http://gnomad.b	0.0002323407067	FIN	0.001076519955	0		0	0	https://www.ncbi.nlm.nih.gov/clinvar/variation/322217/
NM_000543.4(SMPD1):c.1474G>A	concordant	unreportable	unreportable	445832	0	1	SCV000702522	Uncertain significance	EGL Genetic Diagn	6/30/2017	SMPD1	chr11	6415259	6415259	http://gnomad.b	0.001087059943	NFE	0.001602820004	0		2	0	https://www.ncbi.nlm.nih.gov/clinvar/variation/445832/
NM_138694.3(PKHD1):c.281+3A>G	concordant	unreportable	unreportable	289883	0	2	SCV000344316;S	Likely benign;Likely benign	EGL Genetic Diagn	2017-06-30;2017-1	PKHD1	chr6	51947187	51947187	http://gnomad.b	0.0003030168244	AFR	0.00294041005	0		0	0	https://www.ncbi.nlm.nih.gov/clinvar/variation/289883/
NM_000391.3(TPP1):c.1181A>G	concordant	unreportable	unreportable	207588	0	1	SCV000548743	Uncertain significance	Invitae	3/14/2017	TPP1	chr11	6636758	6636758		5.60E-06					0	0	https://www.ncbi.nlm.nih.gov/clinvar/variation/207588/
NM_017890.4(VPS13B):c.11694G>C	concordant	unreportable	unreportable	437246	0	2	SCV000703875;S	Benign;Likely benign	EGL Genetic Diagn	2017-06-30;2017-0	VPS13B	chr8	100883799	100883799	http://gnomad.b	0.0008006943084	AFR	0.009409300052	1		0	0	https://www.ncbi.nlm.nih.gov/clinvar/variation/437246/
NM_001360.2(DHCR7):c.1370G>A	concordant	unreportable	unreportable	285508	0	1	SCV000338554	Uncertain significance	EGL Genetic Diagn	6/30/2017	DHCR7	chr11	71146479	71146479	http://gnomad.b	3.50E-05	NFE	2.73E-05	0		0	0	https://www.ncbi.nlm.nih.gov/clinvar/variation/285508/
NM_000642.2(AGL):c.2802A>C	concordant	unreportable	unreportable	256729	0	1	SCV000626708	Benign	Invitae	10/5/2017	AGL	chr1	100353654	100353654	http://gnomad.b	0.002165819132	AFR	0.02640179917	2		7	0	https://www.ncbi.nlm.nih.gov/clinvar/variation/256729/
NM_000466.2(PEX1):c.3044A>C	concordant	unreportable	unreportable	289972	0	1	SCV000344442	Uncertain significance	EGL Genetic Diagn	6/30/2017	PEX1	chr7	92122430	92122430	http://gnomad.b	6.93E-05	AFR	0.0008513429784	0		0	0	https://www.ncbi.nlm.nih.gov/clinvar/variation/289972/
NM_001042432.1(CLN3):c.1159G>A	concordant	unreportable	unreportable	457949	0	1	SCV000628922	Uncertain significance	Invitae	10/5/2017	CLN3	chr16	28489096	28489096	http://gnomad.b	8.33E-06	NFE	2.69E-05	0		0	0	https://www.ncbi.nlm.nih.gov/clinvar/variation/457949/
NM_003060.3(SLC22A5):c.279G>C	concordant	unreportable	unreportable	460406	0	1	SCV000632544	Likely benign	Invitae	10/5/2017	SLC22A5	chr5	131705943	131705943	http://gnomad.b	0.0006947503982	NFE	0.0009802320274	0		0	0	https://www.ncbi.nlm.nih.gov/clinvar/variation/460406/
NM_000543.4(SMPD1):c.551C>T	concordant	unreportable	unreportable	502573	0	1	SCV000709368	Uncertain significance	EGL Genetic Diagn	6/30/2017	SMPD1	chr11	6412846	6412846	http://gnomad.b	1.43E-05	AFR	0.0004132229951	0		0	0	https://www.ncbi.nlm.nih.gov/clinvar/variation/502573/
NM_004646.3(NPHS1):c.128T>C	concordant	unreportable	unreportable	259483	0	1	SCV000698496	Uncertain significance	Laboratory Corpor	1/25/2018	NPHS1	chr19	36342505	36342505	http://gnomad.b	0.0005658725867	AFR	0.006976120174	0		0	0	https://www.ncbi.nlm.nih.gov/clinvar/variation/259483/
NM_000243.2(MEFV):c.1337A>C	concordant	unreportable	unreportable	234502	0	1	SCV000279390	Uncertain significance	GeneDx	11/28/2017	MEFV	chr16	3298928	3298928	http://gnomad.b	6.16E-05	NFE	8.06E-05	0		0	0	https://www.ncbi.nlm.nih.gov/clinvar/variation/234502/
NM_000152.3(GAA):c.2609G>A	concordant	unreportable	unreportable	456411	0	1	SCV000626587	Uncertain significance	Invitae	10/5/2017	GAA	chr17	78092119	78092119	http://gnomad.b	4.72E-05	NFE	4.99E-05	1		0	0	https://www.ncbi.nlm.nih.gov/clinvar/variation/456411/
NM_000053.3(ATP7B):c.133G>A	concordant	unreportable	unreportable	312400	0	1	SCV000384687	Uncertain significance	Illumina Clinical Se	10/18/2016	ATP7B	chr13	52549223	52549223	http://gnomad.b	1.12E-05	AMR	2.98E-05	0		0	0	https://www.ncbi.nlm.nih.gov/clinvar/variation/312400/
NM_017890.4(VPS13B):c.5998_5999d	concordant	reportable	reportable	280018	1	0	SCV000329733	Pathogenic	GeneDx	11/28/2017	VPS13B	chr8	100673593	100673595	http://gnomad.b	5.60E-06	AMR	5.97E-05	0		0	0	https://www.ncbi.nlm.nih.gov/clinvar/variation/280018/
NM_000518.4(HBB):c.151A>T	concordant	unreportable	unreportable	495980	0	1	SCV000697086	Uncertain significance	Laboratory Corpor	1/25/2018	HBB	chr11	5247971	5247971	http://gnomad.b	9.33E-06	AFR	6.53E-05	0		0	0	https://www.ncbi.nlm.nih.gov/clinvar/variation/495980/
NM_000051.3(ATM):c.1380G>C	concordant	unreportable	unreportable	136434	0	4	SCV000681974;S	Likely benign;Likely benign	Color Genomics, In	2017-10-26;2017-0	ATM	chr11	108121572	108121572	http://gnomad.b	0.0002255613973	AFR	0.001894930028	0		0	0	https://www.ncbi.nlm.nih.gov/clinvar/variation/136434/
NM_000051.3(ATM):c.1073A>G	concordant	unreportable	unreportable	127329	0	4	SCV000149042;S	Uncertain significance;Unc	GeneDx;Genetic Se	2017-11-28;2017-0	ATM	chr11	108119667	108119667	http://gnomad.b	0.0002255613973	AFR	0.001846969943	0		0	0	https://www.ncbi.nlm.nih.gov/clinvar/variation/127329/
NM_000528.3(MAN2B1):c.2782G>C	concordant	unreportable	unreportable	287586	0	1	SCV000341394	Likely benign	EGL Genetic Diagn	6/30/2017	MAN2B1	chr19	12758295	12758295	http://gnomad.b	9.86E-05	AMR	0.002590670018	1		0	0	https://www.ncbi.nlm.nih.gov/clinvar/variation/287586/
NM_025136.3(OPA3):c.537A>G	concordant	unreportable	unreportable	386291	0	1	SCV000527867	Likely benign	GeneDx	11/28/2017	OPA3	chr19	46056775	46056775	http://gnomad.b	0.0003760801859	AFR	0.004480899777	0		0	0	https://www.ncbi.nlm.nih.gov/clinvar/variation/386291/
NM_000051.3(ATM):c.4167A>G	concordant	unreportable	unreportable	136445	0	3	SCV000537429;S	Likely benign;Likely benign	Color Genomics, In	2017-01-26;2017-1	ATM	chr11	108159761	108159761	http://gnomad.b	0.0001392354304	AMR	0.0002085690066	0		0	0	https://www.ncbi.nlm.nih.gov/clinvar/variation/136445/
NM_000051.3(ATM):c.8532T>C	concordant	unreportable	unreportable	181843	0	2	SCV000687839;S	Likely benign;Likely benign	Color Genomics, In	2017-12-21;2017-1	ATM	chr11	108216583	108216583	http://gnomad.b	5.29E-05	NFE	5.38E-05	0		0	0	https://www.ncbi.nlm.nih.gov/clinvar/variation/181843/
NM_014363.4(SACS):c.5391G>C	concordant	unreportable	unreportable	448206	0	1	SCV000614960	Uncertain significance	Athena Diagnostics	8/17/2017	SACS	chr13	23912624	23912624	http://gnomad.b	9.19E-05	NFE	0.0001970970043	0		0	0	https://www.ncbi.nlm.nih.gov/clinvar/variation/448206/
NM_015166.3(MLC1):c.561A>C	concordant	unreportable	unreportable	342107	0	1	SCV000439240	Uncertain significance	Illumina Clinical Se	10/18/2016	MLC1	chr22	50515306	50515306	http://gnomad.b	0.0005621497052	ASJ	0.002131979913	0		0	0	https://www.ncbi.nlm.nih.gov/clinvar/variation/342107/
NM_014363.4(SACS):c.8972G>A	concordant	unreportable	unreportable	448227	0	1	SCV000614992	Uncertain significance	Athena Diagnostics	8/17/2017	SACS	chr13	23909043	23909043	http://gnomad.b	0.000130418287	FIN	0.006773729809	0		0	0	https://www.ncbi.nlm.nih.gov/clinvar/variation/448227/
NM_000057.2(BLM):c.4113G>A	concordant	unreportable	unreportable	454149	0	1	SCV000623331	Likely benign	Invitae	10/5/2017	BLM	chr15	91358368	91358368	http://gnomad.b	0.0002711316178	ASJ	0.001928930054	0		1	0	https://www.ncbi.nlm.nih.gov/clinvar/variation/454149/
NM_000352.3(ABCC8):c.2635G>A	concordant	unreportable	unreportable	303771	0	1	SCV000369313	Likely benign	Illumina Clinical Se	10/18/2016	ABCC8	chr11	17432122	17432122	http://gnomad.b	3.61E-05	AFR	0.0003920540039	1		0	0	https://www.ncbi.nlm.nih.gov/clinvar/variation/303771/
NM_000018.3(ACADVL):c.1828-4C>G	concordant	unreportable	unreportable	254697	0	1	SCV000654943	Benign	Invitae	10/5/2017	ACADVL	chr17	7128272	7128272	http://gnomad.b	0.000515376338	AFR	0.00699895015	0		0	0	https://www.ncbi.nlm.nih.gov/clinvar/variation/254697/
NM_138694.3(PKHD1):c.2162C>T	concordant	unreportable	unreportable	501349	0	1	SCV000707678	Uncertain significance	EGL Genetic Diagn	6/30/2017	PKHD1	chr6	51915072	51915072	http://gnomad.b	0.0004503552803	ASJ	0.004880030174	0		0	0	https://www.ncbi.nlm.nih.gov/clinvar/variation/501349/
NM_138694.3(PKHD1):c.11002G>T	concordant	unreportable	unreportable	497601	0	1	SCV000702211	Uncertain significance	EGL Genetic Diagn	6/30/2017	PKHD1	chr6	51523922	51523922	http://gnomad.b	0.0002251776401	AFR	0.00294156	0		0	0	https://www.ncbi.nlm.nih.gov/clinvar/variation/497601/
NM_138694.3(PKHD1):c.2811G>A	concordant	reportable	reportable	458592	1	0	SCV000629911	Pathogenic	Invitae	10/5/2017	PKHD1	chr6	51908433	51908433		1.39E-05					0	0	https://www.ncbi.nlm.nih.gov/clinvar/variation/458592/

Supplemental Table 2

NM_000030.2(AGXT):c.35A>G	concordant	unreportable	unreportable	204019	0		1	SCV000429356	Uncertain significance	Illumina Clinical Se	10/18/2016	AGXT	chr2	241808317	241808317	http://gnomad.b	0.0003372490032	FIN	0.002116549993	1		0	0	https://www.ncbi.nlm.nih.gov/clinvar/variation/204019/
NM_000303.2(PMM2):c.657G>C	concordant	unreportable	unreportable	289152	0		1	SCV000343455	Uncertain significance	EGL Genetic Diagn	6/30/2017	PMM2	chr16	8941598	8941598	http://gnomad.b	0.0001308528824	AFR	0.0008501179982	0		0	0	https://www.ncbi.nlm.nih.gov/clinvar/variation/289152/
NM_004937.2(CTNS):c.694C>T	concordant	unreportable	unreportable	322841	0		1	SCV000402234	Uncertain significance	Illumina Clinical Se	10/18/2016	CTNS	chr17	3561311	3561311	http://gnomad.b	7.41E-05	AFR	0.0002617459977	0		0	0	https://www.ncbi.nlm.nih.gov/clinvar/variation/322841/
NM_017739.3(POMGNT1):c.788G>A	concordant	unreportable	unreportable	286165	0		1	SCV000339490	Uncertain significance	EGL Genetic Diagn	6/30/2017	POMGNT1	chr1	46660037	46660037	http://gnomad.b	3.36E-05	EAS	0.0001159550011	0		0	0	https://www.ncbi.nlm.nih.gov/clinvar/variation/286165/
NM_004646.3(NPHS1):c.3388-14C>T	concordant	unreportable	unreportable	328854	0		1	SCV000411550	Uncertain significance	Illumina Clinical Se	10/18/2016	NPHS1	chr19	36322062	36322062	http://gnomad.b	0.0001728280807	ASJ	0.0001016669994	0		0	0	https://www.ncbi.nlm.nih.gov/clinvar/variation/328854/
NM_000528.3(MAN2B1):c.1110-8C>T	concordant	unreportable	unreportable	457138	0		1	SCV000627788	Likely benign	Invitae	10/5/2017	MAN2B1	chr19	12769166	12769166	http://gnomad.b	0.001135224059	NFE	0.001745839952	0		1	0	https://www.ncbi.nlm.nih.gov/clinvar/variation/457138/
NM_000228.2(LAMB3):c.1807C>T	concordant	unreportable	unreportable	295098	0		1	SCV000353621	Uncertain significance	Illumina Clinical Se	10/18/2016	LAMB3	chr1	209799162	209799162	http://gnomad.b	3.64E-05	NFE	4.49E-05	0		0	0	https://www.ncbi.nlm.nih.gov/clinvar/variation/295098/
NM_004328.4(BCS1L):c.702C>T	concordant	unreportable	unreportable	377559	0		1	SCV000512258	Likely benign	GeneDx	11/28/2017	BCS1L	chr2	219526966	219526966	http://gnomad.b	0.0001143322439	NFE	0.0001880780037	0		0	0	https://www.ncbi.nlm.nih.gov/clinvar/variation/377559/
NM_014363.4(SACS):c.2602A>G	concordant	unreportable	unreportable	448198	0		1	SCV000614946	Uncertain significance	Athena Diagnostics	8/17/2017	SACS	chr13	23915413	23915413	http://gnomad.b	0.0001541307028	AFR	0.00130940997	0		0	0	https://www.ncbi.nlm.nih.gov/clinvar/variation/448198/
NM_000182.4(HADHA):c.79C>T	concordant	unreportable	unreportable	335388	0		1	SCV000429536	Uncertain significance	Illumina Clinical Se	10/18/2016	HADHA	chr2	26462000	26462000	http://gnomad.b	6.17E-05	AFR	0.0009812900098	0		0	0	https://www.ncbi.nlm.nih.gov/clinvar/variation/335388/
NM_000271.4(NPC1):c.541G>A	concordant	unreportable	unreportable	497961	0		1	SCV000702740	Uncertain significance	EGL Genetic Diagn	6/30/2017	NPC1	chr18	21141414	21141414	http://gnomad.b	0.0004345986253	EAS	0.0005797769991	0		0	0	https://www.ncbi.nlm.nih.gov/clinvar/variation/497961/
NM_000051.3(ATM):c.3517T>C	concordant	unreportable	unreportable	136442	0		3	SCV000682135;S(	Likely benign;Likely benign	Color Genomics, In	2017-10-26;2017-0	ATM	chr11	108151836	108151836	http://gnomad.b	0.0002255613973	AFR	0.001895179972	0		0	0	https://www.ncbi.nlm.nih.gov/clinvar/variation/136442/
NM_133647.1(SLC12A6):c.1164C>T	concordant	unreportable	unreportable	379488	0		1	SCV000516602	Likely benign	GeneDx	11/28/2017	SLC12A6	chr15	34544540	34544540	http://gnomad.b	0.0006637902423	NFE	0.0009044830222	0		1	0	https://www.ncbi.nlm.nih.gov/clinvar/variation/379488/
NM_020533.2(MCOLN1):c.1359+13C>	concordant	unreportable	unreportable	330503	0		1	SCV000415621	Uncertain significance	Illumina Clinical Se	10/18/2016	MCOLN1	chr19	7594611	7594611	http://gnomad.b	0.0002140879378	NFE	0.0003227250127	0		0	0	https://www.ncbi.nlm.nih.gov/clinvar/variation/330503/
NM_000051.3(ATM):c.3336T>A	concordant	unreportable	unreportable	215582	0		2	SCV000682120;S(	Likely benign;Likely benign	Color Genomics, In	2017-10-26;2017-1	ATM	chr11	108150269	108150269	http://gnomad.b	3.62E-05	AMR	0.0001490940049	0		0	0	https://www.ncbi.nlm.nih.gov/clinvar/variation/215582/
NM_000152.3(GAA):c.359C>G	concordant	unreportable	unreportable	456421	0		1	SCV000626608	Uncertain significance	Invitae	10/5/2017	GAA	chr17	78078744	78078744	http://gnomad.b	5.00E-05	NFE	0.0001090649967	0		0	0	https://www.ncbi.nlm.nih.gov/clinvar/variation/456421/
NM_000543.4(SMPD1):c.441G>A	concordant	unreportable	unreportable	287033	0		2	SCV000340675;S(	Benign;Uncertain significar	EGL Genetic Diagn	2017-06-30;2016-1	SMPD1	chr11	6412736	6412736	http://gnomad.b	0.0009134157945	AFR	0.005362980068	0		0	0	https://www.ncbi.nlm.nih.gov/clinvar/variation/287033/
NM_002485.4(NBN):c.819A>T	concordant	unreportable	unreportable	136047	0		2	SCV000685830;S(	Likely benign;Likely benign	Color Genomics, In	2017-10-26;2017-1	NBN	chr8	90982669	90982669	http://gnomad.b	5.02E-05	NFE	0.0001254280069	0		0	0	https://www.ncbi.nlm.nih.gov/clinvar/variation/136047/
NM_004646.3(NPHS1):c.1799A>G	concordant	unreportable	unreportable	328867	0		1	SCV000411570	Uncertain significance	Illumina Clinical Se	10/18/2016	NPHS1	chr19	36336401	36336401	http://gnomad.b	0.0002759674191	SAS	0.003098899964	0		0	0	https://www.ncbi.nlm.nih.gov/clinvar/variation/328867/
NM_000441.1(SLC26A4):c.1069G>A	concordant	unreportable	unreportable	43491	0		2	SCV000339669;S(	Likely benign;Uncertain sig	EGL Genetic Diagn	2017-06-30;2016-1	SLC26A4	chr7	107329565	107329565	http://gnomad.b	0.0005779572144	AFR	0.005163399968	0		0	0	https://www.ncbi.nlm.nih.gov/clinvar/variation/43491/
NM_000057.2(BLM):c.3828G>A	concordant	unreportable	unreportable	405304	0		1	SCV000543367	Likely benign	Invitae	10/5/2017	BLM	chr15	91352443	91352443	http://gnomad.b	0.0001308091138	NFE	0.0002417320065	0		0	0	https://www.ncbi.nlm.nih.gov/clinvar/variation/405304/
NM_000017.2(ACADS):c.796-14G>C	concordant	unreportable	unreportable	307440	0		2	SCV000511938;S(	Likely benign;Uncertain sig	GeneDx;Illumina C	2017-11-28;2016-1	ACADS	chr12	121176322	121176322	http://gnomad.b	0.0007901086329	AFR	0.01048479974	1		2	0	https://www.ncbi.nlm.nih.gov/clinvar/variation/307440/
NM_000243.2(MEFV):c.343C>A	concordant	unreportable	unreportable	96982	0		2	SCV000279025;S(	Uncertain significance;Unc	GeneDx;Illumina C	2017-11-28;2016-1	MEFV	chr16	3304725	3304725	http://gnomad.b	0.0003050657711	SAS	0.003121749964	0		2	0	https://www.ncbi.nlm.nih.gov/clinvar/variation/96982/
NM_000303.2(PMM2):c.597C>T	concordant	unreportable	unreportable	321220	0		1	SCV000399679	Uncertain significance	Illumina Clinical Se	10/18/2016	PMM2	chr16	8906921	8906921	http://gnomad.b	1.11E-05	AMR	8.93E-05	0		0	0	https://www.ncbi.nlm.nih.gov/clinvar/variation/321220/
NM_006493.2(CLN5):c.178G>T	concordant	unreportable	unreportable	421564	0		1	SCV000570815	Uncertain significance	GeneDx	11/28/2017	CLN5	chr13	77566264	77566264	http://gnomad.b	0.0001398703122	SAS	0.001606240054	0		0	0	https://www.ncbi.nlm.nih.gov/clinvar/variation/421564/
NM_001876.3(CPT1A):c.2061C>G	concordant	unreportable	unreportable	463966	0		1	SCV000638097	Uncertain significance	Invitae	10/5/2017	CPT1A	chr11	68527774	68527774		5.63E-06					0	0	https://www.ncbi.nlm.nih.gov/clinvar/variation/463966/
NM_006493.2(CLN5):c.134C>T	concordant	unreportable	unreportable	205140	0		1	SCV000549219	Uncertain significance	Invitae	3/14/2017	CLN5	chr13	77566220	77566220	http://gnomad.b	1.68E-05	AMR	9.01E-05	0		0	0	https://www.ncbi.nlm.nih.gov/clinvar/variation/205140/
NM_000543.4(SMPD1):c.349G>A	concordant	unreportable	unreportable	305197	0		1	SCV000372924	Uncertain significance	Illumina Clinical Se	10/18/2016	SMPD1	chr11	6412644	6412644	http://gnomad.b	0.0001213130352	EAS	0.003943399992	1		0	0	https://www.ncbi.nlm.nih.gov/clinvar/variation/305197/
NM_000098.2(CPT2):c.353A>G	concordant	unreportable	unreportable	203666	0		2	SCV000238787;S(	Uncertain significance;Beni	GeneDx;Invitae	2017-11-28;2017-1	CPT2	chr1	53675699	53675699	http://gnomad.b	0.0007191104296	AMR	0.01459200028	5		0	0	https://www.ncbi.nlm.nih.gov/clinvar/variation/203666/
NM_000053.3(ATP7B):c.3405A>G	concordant	unreportable	unreportable	386708	0		3	SCV000705072;S(	Uncertain significance;Likel	EGL Genetic Diagn	2017-06-30;2017-1	ATP7B	chr13	52516529	52516529	http://gnomad.b	0.0005471503289	AMR	0.002620449988	1		0	0	https://www.ncbi.nlm.nih.gov/clinvar/variation/386708/
NM_000018.3(ACADVL):c.829_831del(	concordant	unreportable	unreportable	203589	1		1	SCV000238663;S(	Likely pathogenic;Uncertai	GeneDx;Invitae	2017-11-28;2017-1	ACADVL	chr17	7125569	7125572	http://gnomad.b	0.0002208755734	AFR	0.0002614039986	0		0	0	https://www.ncbi.nlm.nih.gov/clinvar/variation/203589/
NM_033056.3(PCDH15):c.2424G>C	concordant	unreportable	unreportable	46450	0		2	SCV000604605;S(	Uncertain significance;Unc	ARUP Laboratories	2017-06-30;2016-0	PCDH15	chr10	55782754	55782754	http://gnomad.b	0.0001800133724	AMR	0.0007446240052	0		0	0	https://www.ncbi.nlm.nih.gov/clinvar/variation/46450/
NM_000360.3(TH):c.406G>A	concordant	unreportable	unreportable	304078	0		1	SCV000369922	Uncertain significance	Illumina Clinical Se	10/18/2016	TH	chr11	2189802	2189802	http://gnomad.b	0.0001887419644	NFE	0.0002061279956	0		0	0	https://www.ncbi.nlm.nih.gov/clinvar/variation/304078/
NM_024685.3(BBS10):c.1669A>G	concordant	unreportable	unreportable	261755	0		1	SCV000593587	Likely benign	Genetic Services La	7/5/2017	BBS10	chr12	76740096	76740096	http://gnomad.b	0.0004560461082	AFR	0.005230810028	0		1	0	https://www.ncbi.nlm.nih.gov/clinvar/variation/261755/
NM_000370.3(TTPA):c.178G>A	concordant	unreportable	unreportable	448838	0		2	SCV000616203;S(	Uncertain significance;Unc	Athena Diagnostics	2017-08-17;2017-0	TTPA	chr8	63998403	63998403	http://gnomad.b	0.0004143372705	NFE	0.0008700310136	0		0	0	https://www.ncbi.nlm.nih.gov/clinvar/variation/448838/
NM_000051.3(ATM):c.7757A>G	concordant	unreportable	unreportable	133633	0		4	SCV000335111;S(	Uncertain significance;Unc	EGL Genetic Diagn	2017-06-30;2017-1	ATM	chr11	108202733	108202733	http://gnomad.b	0.0001169577615	AMR	0.0003872269881	0		0	0	https://www.ncbi.nlm.nih.gov/clinvar/variation/133633/
NM_000030.2(AGXT):c.732C>A	concordant	unreportable	unreportable	254727	0		1	SCV000429369	Likely benign	Illumina Clinical Se	10/18/2016	AGXT	chr2	241814577	241814577	http://gnomad.b	0.003608265884	AMR	0.06758210063	95		35	0	https://www.ncbi.nlm.nih.gov/clinvar/variation/254727/
NM_000071.2(CBS):c.1479G>A	concordant	unreportable	unreportable	212834	0		2	SCV000319253;S(	Likely benign;Likely benign	Ambry Genetics;In	2017-11-14;2017-1	CBS	chr21	44476986	44476986	http://gnomad.b	0.000317756694	NFE	0.0006630860153	0		0	0	https://www.ncbi.nlm.nih.gov/clinvar/variation/212834/
NM_000152.3(GAA):c.247G>A	concordant	unreportable	unreportable	290327	0		1	SCV000344863	Uncertain significance	EGL Genetic Diagn	6/30/2017	GAA	chr17	78078632	78078632	http://gnomad.b	3.06E-05	EAS	0.0002914090001	0		0	0	https://www.ncbi.nlm.nih.gov/clinvar/variation/290327/
NM_000051.3(ATM):c.610G>A	concordant	unreportable	unreportable	127419	0		3	SCV000537552;S(	Uncertain significance;Unc	Color Genomics, In	2017-12-21;2017-1	ATM	chr11	108114793	108114793	http://gnomad.b	5.85E-05	AMR	0.0002088059991	0		0	0	https://www.ncbi.nlm.nih.gov/clinvar/variation/127419/
NM_005476.5(GNE):c.1242T>C	concordant	unreportable	unreportable	282274	0		1	SCV000341655	Uncertain significance	EGL Genetic Diagn	11/3/2016	GNE	chr9	36227284	36227284	http://gnomad.b	0.0001400795652	SAS	0.001916829962	0		1	0	https://www.ncbi.nlm.nih.gov/clinvar/variation/282274/
NM_000152.3(GAA):c.2155G>T	concordant	unreportable	unreportable	286435	0		1	SCV000339871	Uncertain significance	EGL Genetic Diagn	6/30/2017	GAA	chr17	78087131	78087131	http://gnomad.b	0.0002556961885	ASJ	0.0008810569998	0		0	0	https://www.ncbi.nlm.nih.gov/clinvar/variation/286435/
NM_001876.3(CPT1A):c.1055G>A	concordant	unreportable	unreportable	381411	0		1	SCV000520650	Benign	GeneDx	11/28/2017	CPT1A	chr11	68552391	68552391	http://gnomad.b	0.00174312185	SAS	0.02066140063	9		8	0	https://www.ncbi.nlm.nih.gov/clinvar/variation/381411/
NM_000051.3(ATM):c.8968G>A	concordant	unreportable	unreportable	127466	0		3	SCV000537525;S(	Uncertain significance;Unc	Color Genomics, In	2017-12-21;2017-1	ATM	chr11	108235926	108235926	http://gnomad.b	0.0001475895562	ASJ	0.001015429967	0		0	0	https://www.ncbi.nlm.nih.gov/clinvar/variation/127466/
NM_005476.5(GNE):c.1871C>T	concordant	unreportable	unreportable	285685	0		1	SCV000338823	Uncertain significance	EGL Genetic Diagn	6/30/2017	GNE	chr9	36218242	36218242	http://gnomad.b	7.56E-05	AFR	0.0001306850027	0		0	0	https://www.ncbi.nlm.nih.gov/clinvar/variation/285685/
NM_014363.4(SACS):c.1917A>G	concordant	unreportable	unreportable	287300	0		2	SCV000614944;S(	Likely benign;Uncertain sig	Athena Diagnostics	2017-08-17;2017-0	SACS	chr13	23928834	23928834	http://gnomad.b	0.0002282320023	AFR	0.001568219974	0		0	0	https://www.ncbi.nlm.nih.gov/clinvar/variation/287300/
NM_000152.3(GAA):c.726G>A	concordant	unreportable	unreportable	285366	0		4	SCV000338356;S(	Uncertain significance;Likel	EGL Genetic Diagn	2017-06-30;2017-1	GAA	chr17	78081389	78081389	http://gnomad.b	0.0003196202356	AFR	0.002747969935	0		0	0	https://www.ncbi.nlm.nih.gov/clinvar/variation/285366/
NM_017890.4(VPS13B):c.10018G>A	concordant	unreportable	unreportable	130716	0		4	SCV000258124;S(	Benign;Benign;Uncertain si	Division of Genomi	2015-08-10;2017-1	VPS13B	chr8	100861004	100861004	http://gnomad.b	0.001833757944	AFR	0.02385310084	4		4	0	https://www.ncbi.nlm.nih.gov/clinvar/variation/130716/
NM_138694.3(PKHD1):c.1848A>T	concordant	unreportable	unreportable	357448	0		1	SCV000464113	Uncertain significance	Illumina Clinical Se	10/18/2016	PKHD1	chr6	51918952	51918952	http://gnomad.b	0.0001139788055	NFE	9.87E-05	0		0	0	https://www.ncbi.nlm.nih.gov/clinvar/variation/357448/
NM_017890.4(VPS13B):c.3243A>G	concordant	unreportable	unreportable	361055	0		2	SCV000470791;S(	Uncertain significance;Beni	Illumina Clinical Se	2016-10-18;2017-1	VPS13B	chr8	100454661	100454661	http://gnomad.b	0.0009574736135	AFR	0.0130048003	0		3	0	https://www.ncbi.nlm.nih.gov/clinvar/variation/361055/
NM_000051.3(ATM):c.7522G>A	concordant	unreportable	unreportable	187265	0		3	SCV000682423;S(	Uncertain significance;Unc	Color Genomics, In	2017-10-26;2017-1	ATM	chr11	108202177	108202177	http://gnomad.b	2.78E-05	AFR	0.000130941	0		0	0	https://www.ncbi.nlm.nih.gov/clinvar/variation/187265/
NM_002435.1(MPI):c.472G>A	concordant	unreportable	unreportable	317139	0		1	SCV000393943	Uncertain significance	Illumina Clinical Se	10/18/2016	MPI	chr15	75185128	75185128	http://gnomad.b	4.48E-05	SAS	6.50E-05	0		1	0	https://www.ncbi.nlm.nih.gov/clinvar/variation/317139/
NM_000227.3(LAMA3):c.2718C>T	concordant	unreportable	unreportable	326319	0		1	SCV000407965	Uncertain significance	Illumina Clinical Se	10/18/2016	LAMA3	chr18	21494725	21494725	http://gnomad.b	0.00036396011	SAS	0.0005523429718	0		0	0	https://www.ncbi.nlm.nih.gov/clinvar/variation/326319/
NM_000030.2(AGXT):c.28C>G	concordant	unreportable	unreportable	383367	0		1	SCV000523746	Uncertain significance	GeneDx	11/28/2017	AGXT	chr2	241808310	241808310	http://gnomad.b	5.97E-06	NFE	9.21E-06	0		0	0	https://www.ncbi.nlm.nih.gov/clinvar/variation/383367/
NM_017739.3(POMGNT1):c.38T>C	concordant	unreportable	unreportable	211941	0		3	SCV000706209;S(	Uncertain significance;Unc	EGL Genetic Diagn	2017-06-30;2017-1	POMGNT1	chr1	46663456	46663456	http://gnomad.b	7.56E-05	NFE	0.0002148609929	0		0	0	https://www.ncbi.nlm.nih.gov/clinvar/variation/211941/
NM_000543.4(SMPD1):c.1340+7C>T	concordant	unreportable	unreportable	197159	0		2	SCV000230118;S(	Likely benign;Benign	EGL Genetic Diagn	2017-06-30;2017-1	SMPD1	chr11	6414930	6414930	http://gnomad.b	0.0004376784015	AFR	0.004053350072	0		0	0	https://www.ncbi.nlm.nih.gov/clinvar/variation/197159/
NM_000466.2(PEX1):c.3503A>G	concordant	unreportable	unreportable	282718	0		1	SCV000334308	Uncertain significance	EGL Genetic Diagn	6/30/2017	PEX1	chr7	92119161	92119161	http://gnomad.b	0.0002523837787	AMR	0.000804817013	0		0	0	https://www.ncbi.nlm.nih.gov/clinvar/variation/282718/
NM_001360.2(DHCR7):c.840C>T	concordant	unreportable	unreportable	289501	0		2	SCV000343874;S(	Uncertain significance;Unc	EGL Genetic Diagn	2017-06-30;2016-1	DHCR7	chr11	71148981	71148981	http://gnomad.b	5.65E-05	NFE	0.000125423001	0		0	0	https://www.ncbi.nlm.nih.gov/clinvar/variation/289501/
NM_000057.2(BLM):c.808G>A	concordant	unreportable	unreportable	454165	0		2	SCV000623352;S(	Uncertain significance;Unc	Invitae;Laboratory	2017-10-05;2018-0	BLM	chr15	91295025	91295025	http://gnomad.b	6.66E-05	SAS	0.0002924170112	0		0	0	https://www.ncbi.nlm.nih.gov/clinvar/variation/454165/
NM_000051.3(ATM):c.1010G>A	concordant	unreportable	unreportable	127328	0		3	SCV000681944;S(	Uncertain significance;Unc	Color Genomics, In	2017-10-26;2017-1	ATM	chr11	108117799	108117799	http://gnomad.b	9.47E-05	NFE	0.0001345189958	0		0	0	https://www.ncbi.nlm.nih.gov/clinvar/variation/127328/
NM_000098.2(CPT2):c.1602G>A	concordant	unreportable	unreportable	460425	0		1	SCV000632592	Likely benign	Invitae	10/5/2017	CPT2	chr1	53676948	53676948	http://gnomad.b	0.0001930685589	AMR	0.001116269967	1		1	0	https://www.ncbi.nlm.nih.gov/clinvar/variation/460425/
NM_000137.2(FAH):c.243G>A	concordant	unreportable	unreportable	196216	0		3	SCV000228789;S(	Uncertain significance;Likel	EGL Genetic Diagn	2017-06-30;2017-1	FAH	chr15	80452148	80452148	http://gnomad.b	0.0006131020746	NFE	0.0007161020185	0		0	0	https://www.ncbi.nlm.nih.gov/clinvar/variation/196216/
NM_001079802.1(FKTN):c.668C>T	concordant	unreportable	unreportable	162568	0		6	SCV000319575;S(	Likely benign;Uncertain sig	Ambry Genetics;At	2017-11-14;2017-0	FKTN	chr9	108370120	108370120	http://gnomad.b	0.0005247817546	AFR	0.008691670373	1		0	0	https://www.ncbi.nlm.nih.gov/clinvar/variation/162568/
NM_033056.3(PCDH15):c.5424A>C	concordant	unreportable	unreportable	227842	0		1	SCV000270728	Likely benign	Laboratory for Mol	4/26/2016	PCDH15	chr10	55582062	55582062	http://gnomad.b	3.09E-05	ASJ	0.0004117770004	0		0	0	https://www.ncbi.nlm.nih.gov/clinvar/variation/227842/
NM_000050.4(ASS1):c.765C>T	concordant	unreportable	unreportable	385432	0		2	SCV000707950;S(	Uncertain significance;Likel	EGL Genetic Diagn	2017-06-30;2017-1	ASS1	chr9	133355179	133355179	http://gnomad.b	0.0001671057835	NFE	0.0002417320065	0		0	0	https://www.ncbi.nlm.nih.gov/clinvar/variation/385432/
NM_004937.2(CTNS):c.969C>T	concordant	unreportable	unreportable	284194	0		1	SCV000336716	Uncertain significance	EGL Genetic Diagn	6/30/2017	CTNS	chr17	3563268	3563268	http://gnomad.b	0.000240005689	AFR	0.002945030108	0		0	0	https://www.ncbi.nlm.nih.gov/clinvar/variation/284194/
NM_001042432.1(CLN3):c.1213C>T	concordant	unreportable	unreportable	418137	0		2	SCV000564888;S(	Uncertain significance;Unc	GeneDx;Invitae	2017-11-28;2017-1	CLN3	chr16	28488941	28488941	http://gnomad.b	0.0001166640741	AMR	0.0001191190022	0		0	0	https://www.ncbi.nlm.nih.gov/clinvar/variation/418137/
NM_000182.4(HADHA):c.858G>A	concordant	unreportable	unreportable	335383	0		1	SCV000429523	Uncertain significance	Illumina Clinical Se	10/18/2016	HADHA	chr2	26437372	26437372	http://gnomad.b	0.0001934550511	EAS	0.0001739330037	0		1	0	https://www.ncbi.nlm.nih.gov/clinvar/variation/335383/
NM_003060.3(SLC22A5):c.1643G>A	concordant	unreportable	unreportable	460404	0		1	SCV000632540	Uncertain significance	Invitae	10/5/2017	SLC22A5	chr5	131729933	131729933	http://gnomad.b	0.0001750996939	NFE	0.0004029079864	0		0	0	https://www.ncbi.nlm.nih.gov/clinvar/variation/460404/
NM_000152.3(GAA):c.266G>A	concordant	unreportable	unreportable	283219	0		2	SCV000335191;S(	Uncertain significance;Unc	EGL Genetic Diagn	2017-06-30;2017-1	GAA	chr17	78078651	78078651	http://gnomad.b	0.0002279031245	ASJ	0.001223989995	0		0	0	https://www.ncbi.nlm.nih.gov/clinvar/variation/283219/
NM_000232.4(SGCB):c.92G>T	concordant	unreportable	unreportable	92662	0		1	SCV000642390	Likely benign	Invitae	10/5/2017	SGCB	chr4	52899748	52899748	http://gnomad.b	0.0006213231384	AFR	0.00731929997	0		1	0	https://www.ncbi.nlm.nih.gov/clinvar/variation/92662/
NM_018941.3(CLN8):c.11C>T	concordant	unreportable	unreportable	195348	0		3	SCV000227485;S(	Benign;Likely benign;Likely	EGL Genetic Diagn	2017-06-30;2017-1	CLN8	chr8	1719231	1719231	http://gnomad.b	0.0004673158719	AFR	0.005881580058	0		1	0	https://www.ncbi.nlm.nih.gov/clinvar/variation/195348/
NM_000271.4(NPC1):c.1011G>T	concordant	unreportable	unreportable	288150	0		1	SCV000342173	Uncertain significance	EGL Genetic Diagn	6/30/2017	NPC1	chr18	21136522	21136522	http://gnomad.b	0.0003945698045	NFE	0.0005495889927	0		0	0	https://www.ncbi.nlm.nih.gov/clinvar/variation/288150/
NM_002225.3(IVD):c.243+16C>T	concordant	unreportable	unreportable	386915	0		1	SCV000528729	Likely benign	GeneDx	11/28/2017	IVD	chr15	40699942	40699942	http://gnomad.b	1.12E-05	FIN	0.0005832209717	0		0	0	https://www.ncbi.nlm.nih.gov/clinvar/variation/386915/
NM_000051.3(ATM):c.6234C>T	concordant	unreportable	unreportable	184079	0		3	SCV000682315;S(	Likely benign;Uncertain sig	Color Genomics, In	2017-10-26;2017-0	ATM	chr11	108188135	108188135	http://gnomad.b	3.90E-05	AFR	6.54E-05	0		0	0	https://www.ncbi.nlm.nih.gov/clinvar/variation/184079/
NM_000098.2(CPT2):c.1598T>C	concordant	unreportable	unreportable	203657	0		1	SCV000632591	Likely benign	Invitae	10/5/2017	CPT2	chr1	53676944	53676944	http://gnomad.b	0.0006967256692	AFR	0.00666212989	0		1	0	https://www.ncbi.nlm.nih.gov/clinvar/variation/203657/
NM_001360.2(DHCR7):c.1225G>A	concordant	unreportable	unreportable	500337	0		1	SCV000706233	Uncertain significance	EGL Genetic Diagn	6/30/2017	DHCR7	chr11	71146624	71146624	http://gnomad.b	4.04E-05	SAS	0.0004877730098	0		0	0	https://www.ncbi.nlm.nih.gov/clinvar/variation/500337/
NM_000642.2(AGL):c.2280C>T	concordant	unreportable	unreportable	456469	0		1	SCV000626693	Benign	Invitae	10/5/2017	AGL	chr1	100347219	100347219	http://gnomad.b	0.0004967987184	SAS	0.008349039592	4		1	0	https://www.ncbi.nlm.nih.gov/clinvar/variation/456469/
NM_017890.4(VPS13B):c.8246A>G	concordant	unreportable	unreportable	197529	0		4	SCV000230694;S(	Benign;Benign;Uncertain si	EGL Genetic Diagn	2017-06-30;2017-0	VPS13B	chr8	100829841	100829841	http://gnomad.b	0.001010666592	SAS	0.01403599977	11		6	0	https://www.ncbi.nlm.nih.gov/clinvar/variation/197529/
NM_000051.3(ATM):c.6983C>T	concordant	unreportable	unreportable	186148	0		2	SCV000687747;S(	Uncertain significance;Unc	Color Genomics, In	2017-12-21;2017-0	ATM	chr11	108198379	108198379	http://gnomad.b	1.11E-05	NFE	8.99E-06	0		0	0	https://www.ncbi.nlm.nih.gov/clinvar/variation/186148/
NM_006261.4(PROP1):c.335G>A	concordant	unreportable	unreportable	353014	0		1	SCV000456756	Uncertain significance	Illumina Clinical Se	10/18/2016	PROP1	chr5	177421114	177421114	http://gnomad.b	0.0002704286294	NFE	0.0004591449979	0		0	0	https://www.ncbi.nlm.nih.gov/clinvar/variation/353014/
NM_006493.2(CLN5):c.208C>T	concordant	unreportable	unreportable	205141	0		3	SCV000240641;S(	Uncertain significance;Unc	GeneDx;Illumina C	2017-11-28;2016-1	CLN5	chr13	77566294	77566294	http://gnomad.b	0.0001426677185	SAS	0.0005656480207	0		0	0	https://www.ncbi.nlm.nih.gov/clinvar/variation/205141/
NM_000051.3(ATM):c.8806G>C	concordant	unreportable	unreportable	407465	0		1	SCV000546676	Uncertain significance	Invitae	3/14/2017	ATM	chr11	108225557	108225557		2.78E-06					0	0	https://www.ncbi.nlm.nih.gov/clinvar/variation/407465/
NM_183050.2(BCKDHB):c.447T>C	concordant	unreportable	unreportable	384204	0		2	SCV000524926;S(	Likely benign;Benign	GeneDx;Invitae	2017-11-28;2017-1	BCKDHB	chr6	80877498	80877498	http://gnomad.b	0.0005561421674	AFR	0.006081609987	0		0	0	https://www.ncbi.nlm.nih.gov/clinvar/variation/384204/
NM_138694.3(PKHD1):c.8012G>A	concordant	unreportable	unreportable	496525	0		1	SCV000699878	Uncertain significance	Laboratory Corpora	1/25/2018	PKHD1	chr6	51712668	51712668	http://gnomad.b	1.67E-05	AMR	2.98E-05	0		0	0	https://www.ncbi.nlm.nih.gov/clinvar/variation/496525/
NM_033056.3(PCDH15):c.575A>G	concordant	unreportable	unreportable	300203	0		1	SCV000363241	Uncertain significance	Illumina Clinical Se	10/18/2016	PCDH15	chr10	56106144	56106144	http://gnomad.b	3.86E-05	FIN	0.0001797589939	0		0	0	https://www.ncbi.nlm.nih.gov/clinvar/variation/300203/
NM_033056.3(PCDH15):c.1649T>G	concordant	unreportable	unreportable	300193	0		1	SCV000363205	Uncertain significance	Illumina Clinical Se	10/18/2016	PCDH15	chr10	55912995	55912995	http://gnomad.b	0.000156868796	NFE	0.0003497700091	0		0	0	https://www.ncbi.nlm.nih.gov/clinvar/variation/300193/
NM_000057.2(BLM):c.3117A>G	concordant	unreportable	unreportable	127495	0		1	SCV000555818	Likely benign	Invitae	10/5/2017	BLM	chr15	91337494	91337494	http://gnomad.b	8.56E-05	EAS	0.002946030116	0		0	0	https://www.ncbi.nlm.nih.gov/clinvar/variation/127495/
NM_017890.4(VPS13B):c.886G>A	concordant	unreportable	unreportable	361039	0		1	SCV000470762	Uncertain significance	Illumina Clinical Se	10/18/2016	VPS13B	chr8	100128051	100128051	http://gnomad.b	2.52E-05	AMR	5.96E-05	0		0	0	https://www.ncbi.nlm.nih.gov/clinvar/variation/361039/
NM_000243.2(MEFV):c.1223G>T	concordant	unreportable	unreportable	457995	0		1	SCV000629019	Uncertain significance	Invitae	10/5/2017	MEFV	chr16	3299468	3299468	http://gnomad.b	2.52E-05	AMR	5.96E-05	0		0	0	https://www.ncbi.nlm.nih.gov/clinvar/variation/457995/
NM_000478.4(ALPL):c.436G>A	concordant	unreportable	unreportable	197677	0		1	SCV000230931	Uncertain significance	EGL Genetic Diagn	6/30/2017	ALPL	chr1	21889741	21889741	http://gnomad.b	0.0001436749228	NFE	0.0002342219959	0		0	0	https://www.ncbi.nlm.nih.gov/clinvar/variation/197677/
NM_000071.2(CBS):c.1125C>T	concordant	unreportable	unreportable	391444	0		2	SCV000534519;S(	Likely benign;Likely benign	GeneDx;Invitae	2017-11-28;2017-1	CBS	chr21	44480571	44480571	http://gnomad.b	0.0001827803992	AFR	0.002497039968	0		0	0	https://www.ncbi.nlm.nih.gov/clinvar/variation/391444/
NM_033056.3(PCDH15):c.949T>A	concordant	unreportable	unreportable	96235	0		2	SCV000604606;S(	Uncertain significance;Unc	ARUP Laboratories	2017-06-30;2017-0	PCDH15	chr10	55996619	55996619	http://gnomad.b	0.0001800133724	AFR	0.002679740079	0		0	0	https://www.ncbi.nlm.nih.gov/clinvar/variation/96235/
NM_001164277.1(SLC37A4):c.492C>A	concordant	unreportable	unreportable	496949	0		1	SCV000700826	Uncertain significance	EGL Genetic Diagn	6/30/2017	SLC37A4	chr11	118898472	118898472	http://gnomad.b	0.0003073054896	NFE	0.0003618529881	0		0	0	https://www.ncbi.nlm.nih.gov/clinvar/variation/496949/
NM_017739.3(POMGNT1):c.1596T>C	concordant	unreportable	unreportable	502236	0		1	SCV000708897	Uncertain significance	EGL Genetic Diagn	6/30/2017	POMGNT1	chr1	46656400	46656400	http://gnomad.b	0.0001623749293	SAS	0.00139691995	0		0	0	https://www.ncbi.nlm.nih.gov/clinvar/variation/502236/
NM_000018.3(ACADVL):c.1606-40G>A	concordant	unreportable	unreportable	439354	0		1	SCV000602357	Likely benign	ARUP Laboratories	6/30/2017	ACADVL	chr17	7127759	7127759	http://gnomad.b	5.66E-05	AMR	5.96E-05	0		0	0	https://www.ncbi.nlm.nih.gov/clinvar/variation/439354/
NM_000543.4(SMPD1):c.1561C>T	concordant	unreportable	unreportable	289949	0		1	SCV000344412	Benign	EGL Genetic Diagn	6/30/2017	SMPD1	chr11	6415502	6415502	http://gnomad.b	0.001077545195	AFR	0.01091220044	0		2	0	https://www.ncbi.nlm.nih.gov/clinvar/variation/289949/

Supplemental Table 2

NM_000543.4(SMPD1):c.1589G>C	concordant	unreportable	unreportable	289948	0	2	SCV000344411;S(	Likely benign;Uncertain sig	EGL Genetic Diagn	2017-06-30;2016-1	SMPD1	chr11	6415530	6415530	http://gnomad.b	0.0005114176974	AFR	0.004051759839	0		1	0	https://www.ncbi.nlm.nih.gov/clinvar/variation/289948/
NM_000057.2(BLM):c.2160C>T	concordant	unreportable	unreportable	136513	0	3	SCV000345294;S(	Benign;Uncertain significar	EGL Genetic Diagn	2017-06-30;2016-1	BLM	chr15	91308611	91308611	http://gnomad.b	0.0009299338819	AFR	0.01019470021	1		2	0	https://www.ncbi.nlm.nih.gov/clinvar/variation/136513/
NM_138694.3(PKHD1):c.8120G>A	concordant	unreportable	unreportable	500183	0	1	SCV000706016	Uncertain significance	EGL Genetic Diagn	6/30/2017	PKHD1	chr6	51701255	51701255	http://gnomad.b	7.78E-05	NFE	0.000161377	0		0	0	https://www.ncbi.nlm.nih.gov/clinvar/variation/500183/
NM_000051.3(ATM):c.8938C>A	concordant	unreportable	unreportable	187422	0	1	SCV000622865	Uncertain significance	Invitae	10/5/2017	ATM	chr11	108235896	108235896	http://gnomad.b	2.78E-06	NFE	8.96E-06	0		0	0	https://www.ncbi.nlm.nih.gov/clinvar/variation/187422/
NM_000543.4(SMPD1):c.1534G>A	concordant	unreportable	unreportable	498027	0	1	SCV000702835	Likely benign	EGL Genetic Diagn	6/30/2017	SMPD1	chr11	6415475	6415475	http://gnomad.b	0.0005661274976	AFR	0.006795609836	0		1	0	https://www.ncbi.nlm.nih.gov/clinvar/variation/498027/
NM_000152.3(GAA):c.510C>T	concordant	unreportable	unreportable	280956	0	2	SCV000330941;S(	Uncertain significance;Likel	EGL Genetic Diagn	2017-06-30;2017-1	GAA	chr17	78078895	78078895	http://gnomad.b	0.0001000550303	AMR	8.96E-05	0		0	0	https://www.ncbi.nlm.nih.gov/clinvar/variation/280956/
NM_000051.3(ATM):c.4802G>A	concordant	unreportable	unreportable	142501	0	2	SCV000292997;S(	Uncertain significance;Unc	GeneDx;Invitae	2017-11-28;2017-1	ATM	chr11	108165679	108165679	http://gnomad.b	8.08E-05	AMR	5.96E-05	0		1	0	https://www.ncbi.nlm.nih.gov/clinvar/variation/142501/
NM_000051.3(ATM):c.1773T>C	concordant	unreportable	unreportable	136435	0	3	SCV000537441;S(	Likely benign;Likely benign	Color Genomics, In	2017-01-26;2017-1	ATM	chr11	108122729	108122729	http://gnomad.b	0.0001615130993	NFE	0.0002246670047	0		0	0	https://www.ncbi.nlm.nih.gov/clinvar/variation/136435/
NM_001079802.1(FKTN):c.383G>A	concordant	unreportable	unreportable	286471	0	1	SCV000339912	Uncertain significance	EGL Genetic Diagn	6/30/2017	FKTN	chr9	108366509	108366509	http://gnomad.b	8.51E-05	NFE	0.0001000350021	0		0	0	https://www.ncbi.nlm.nih.gov/clinvar/variation/286471/
NM_000310.3(PPT1):c.329A>G	concordant	unreportable	unreportable	196249	0	1	SCV000242336	Uncertain significance	GeneDx	11/28/2017	PPT1	chr1	40557750	40557750	http://gnomad.b	0.0002563402415	SAS	0.0006497299764	0		0	0	https://www.ncbi.nlm.nih.gov/clinvar/variation/196249/
NM_020533.2(MCOLN1):c.60C>T	concordant	unreportable	unreportable	330486	0	1	SCV000415601	Uncertain significance	Illumina Clinical Se	10/18/2016	MCOLN1	chr19	7589875	7589875	http://gnomad.b	7.14E-05	AFR	0.0001351170067	0		0	0	https://www.ncbi.nlm.nih.gov/clinvar/variation/330486/
NM_000466.2(PEX1):c.627G>A	concordant	unreportable	unreportable	167445	0	1	SCV000470523	Uncertain significance	Illumina Clinical Se	10/18/2016	PEX1	chr7	92147202	92147202	http://gnomad.b	0.0003716420477	ASJ	0.001810139976	0		0	0	https://www.ncbi.nlm.nih.gov/clinvar/variation/167445/
NM_000243.2(MEFV):c.1518C>T	concordant	unreportable	unreportable	36502	0	1	SCV000696050	Uncertain significance	Laboratory Corpor	1/25/2018	MEFV	chr16	3297085	3297085	http://gnomad.b	0.0002574867058	SAS	0.0002274059952	0		0	0	https://www.ncbi.nlm.nih.gov/clinvar/variation/36502/
NM_014363.4(SACS):c.7737G>A	concordant	unreportable	unreportable	448219	0	1	SCV000614978	Likely benign	Athena Diagnostics	8/17/2017	SACS	chr13	23910278	23910278	http://gnomad.b	0.0004268234848	AFR	0.006537660025	0		0	0	https://www.ncbi.nlm.nih.gov/clinvar/variation/448219/
NM_017739.3(POMGNT1):c.421-7C>A	concordant	unreportable	unreportable	198222	0	3	SCV000231752;S(	Uncertain significance;Unc	EGL Genetic Diagn	2017-06-30;2016-1	POMGNT1	chr1	46661603	46661603	http://gnomad.b	0.0005151204654	AMR	0.00169754005	0		0	0	https://www.ncbi.nlm.nih.gov/clinvar/variation/198222/
NM_000642.2(AGL):c.622T>C	concordant	unreportable	unreportable	456512	0	1	SCV000626764	Uncertain significance	Invitae	10/5/2017	AGL	chr1	100330103	100330103	http://gnomad.b	0.0002176983148	NFE	0.0001344880002	0		0	0	https://www.ncbi.nlm.nih.gov/clinvar/variation/456512/
NM_133647.1(SLC12A6):c.186G>A	concordant	unreportable	unreportable	392962	0	1	SCV000536309	Likely benign	GeneDx	11/28/2017	SLC12A6	chr15	34628696	34628696	http://gnomad.b	2.37E-05	NFE	2.69E-05	0		0	0	https://www.ncbi.nlm.nih.gov/clinvar/variation/392962/
NM_000051.3(ATM):c.946T>C	concordant	unreportable	unreportable	220453	0	3	SCV000292194;S(	Uncertain significance;Unc	Color Genomics, In	2017-12-21;2017-1	ATM	chr11	108117735	108117735	http://gnomad.b	3.62E-05	EAS	0.0001160359971	0		0	0	https://www.ncbi.nlm.nih.gov/clinvar/variation/220453/
NM_000228.2(LAMB3):c.777C>T	concordant	unreportable	unreportable	295129	0	1	SCV000353657	Uncertain significance	Illumina Clinical Se	10/18/2016	LAMB3	chr1	209805973	209805973	http://gnomad.b	0.0006074993981	NFE	0.001201319974	0		0	0	https://www.ncbi.nlm.nih.gov/clinvar/variation/295129/
NM_000228.2(LAMB3):c.773G>A	concordant	unreportable	unreportable	295130	0	1	SCV000353658	Uncertain significance	Illumina Clinical Se	10/18/2016	LAMB3	chr1	209805977	209805977	http://gnomad.b	0.0003051494672	ASJ	0.0004063390079	0		0	0	https://www.ncbi.nlm.nih.gov/clinvar/variation/295130/
NM_000136.2(FANCC):c.1493C>T	concordant	unreportable	unreportable	182488	0	2	SCV000211054;S(	Uncertain significance;Unc	GeneDx;Invitae	2017-11-28;2016-0	FANCC	chr9	97869388	97869388	http://gnomad.b	3.09E-05	NFE	3.58E-05	0		0	0	https://www.ncbi.nlm.nih.gov/clinvar/variation/182488/
NM_138694.3(PKHD1):c.10910G>A	concordant	unreportable	unreportable	502771	0	1	SCV000709634	Uncertain significance	EGL Genetic Diagn	6/30/2017	PKHD1	chr6	51524014	51524014	http://gnomad.b	0.000227957611	ASJ	0.001220260048	0		0	0	https://www.ncbi.nlm.nih.gov/clinvar/variation/502771/
NM_000478.4(ALPL):c.1542G>A	concordant	unreportable	unreportable	295555	0	1	SCV000354317	Uncertain significance	Illumina Clinical Se	10/18/2016	ALPL	chr1	21904108	21904108	http://gnomad.b	5.35E-05	AMR	0.0003304099955	0		0	0	https://www.ncbi.nlm.nih.gov/clinvar/variation/295555/
NM_000051.3(ATM):c.8178T>C	concordant	unreportable	unreportable	232558	0	3	SCV000682468;S(	Likely benign;Likely benign	Color Genomics, In	2017-10-26;2017-1	ATM	chr11	108206598	108206598	http://gnomad.b	8.35E-06	NFE	8.98E-06	0		0	0	https://www.ncbi.nlm.nih.gov/clinvar/variation/232558/
NM_000051.3(ATM):c.4917G>A	concordant	unreportable	unreportable	231284	0	2	SCV000516766;S(	Likely benign;Likely benign	GeneDx;Invitae	2017-11-28;2017-1	ATM	chr11	108168021	108168021	http://gnomad.b	1.39E-05	NFE	1.80E-05	0		0	0	https://www.ncbi.nlm.nih.gov/clinvar/variation/231284/
NM_005562.2(LAMC2):c.589C>T	concordant	unreportable	unreportable	293995	0	1	SCV000351662	Uncertain significance	Illumina Clinical Se	10/18/2016	LAMC2	chr1	183190045	183190045	http://gnomad.b	0.0003723591894	EAS	0.005102629773	1		0	0	https://www.ncbi.nlm.nih.gov/clinvar/variation/293995/
NM_138694.3(PKHD1):c.4009G>A	concordant	unreportable	unreportable	288471	0	2	SCV000342583;S(	Uncertain significance;Unc	EGL Genetic Diagn	2017-06-30;2016-1	PKHD1	chr6	51890599	51890599	http://gnomad.b	0.0002974568827	EAS	0.001043839962	0		0	0	https://www.ncbi.nlm.nih.gov/clinvar/variation/288471/
NM_020533.2(MCOLN1):c.16G>T	concordant	unreportable	unreportable	330485	0	1	SCV000415600	Uncertain significance	Illumina Clinical Se	10/18/2016	MCOLN1	chr19	7587652	7587652	http://gnomad.b	0.0006945964204	AFR	0.007604559883	0		1	0	https://www.ncbi.nlm.nih.gov/clinvar/variation/330485/
NM_000051.3(ATM):c.6974C>T	concordant	unreportable	unreportable	127431	0	3	SCV000682378;S(	Uncertain significance;Unc	Color Genomics, In	2017-10-26;2017-1	ATM	chr11	108196951	108196951	http://gnomad.b	2.51E-05	NFE	1.79E-05	0		0	0	https://www.ncbi.nlm.nih.gov/clinvar/variation/127431/
NM_000228.2(LAMB3):c.3204C>T	concordant	unreportable	unreportable	295067	0	1	SCV000353585	Uncertain significance	Illumina Clinical Se	10/18/2016	LAMB3	chr1	209790779	209790779	http://gnomad.b	6.44E-05	SAS	0.0001624330034	0		0	0	https://www.ncbi.nlm.nih.gov/clinvar/variation/295067/
NM_018941.3(CLN8):c.499G>T	concordant	reportable	reportable	205194	1	0	SCV000594158	Pathogenic	Genetic Services La	7/5/2017	CLN8	chr8	1719719	1719719	http://gnomad.b	1.96E-05	NFE	2.69E-05	0		0	0	https://www.ncbi.nlm.nih.gov/clinvar/variation/205194/
NM_000360.3(TH):c.263C>T	concordant	unreportable	unreportable	304083	0	1	SCV000369928	Uncertain significance	Illumina Clinical Se	10/18/2016	TH	chr11	2190929	2190929	http://gnomad.b	3.66E-05	SAS	0.0002274349972	0		1	0	https://www.ncbi.nlm.nih.gov/clinvar/variation/304083/
NM_000352.3(ABCC8):c.1924-10C>T	concordant	unreportable	unreportable	446766	0	1	SCV000612202	Uncertain significance	Athena Diagnostics	8/17/2017	ABCC8	chr11	17449962	17449962	http://gnomad.b	3.10E-05	AMR	0.0001490669965	0		0	0	https://www.ncbi.nlm.nih.gov/clinvar/variation/446766/
NM_000152.3(GAA):c.1417G>A	concordant	unreportable	unreportable	456377	0	1	SCV000626511	Uncertain significance	Invitae	10/5/2017	GAA	chr17	78083834	78083834	http://gnomad.b	2.22E-05	EAS	5.80E-05	0		0	0	https://www.ncbi.nlm.nih.gov/clinvar/variation/456377/
NM_000051.3(ATM):c.9086G>A	concordant	unreportable	unreportable	127468	0	2	SCV000149183;S(	Uncertain significance;Unc	GeneDx;Invitae	2017-11-28;2017-1	ATM	chr11	108236150	108236150	http://gnomad.b	0.000142020139	NFE	0.0002775189932	0		0	0	https://www.ncbi.nlm.nih.gov/clinvar/variation/127468/
NM_017890.4(VPS13B):c.7750G>T	concordant	unreportable	unreportable	361073	0	1	SCV000470824	Uncertain significance	Illumina Clinical Se	10/18/2016	VPS13B	chr8	100791155	100791155	http://gnomad.b	2.24E-05	NFE	6.29E-05	0		0	0	https://www.ncbi.nlm.nih.gov/clinvar/variation/361073/
NM_138694.3(PKHD1):c.9270C>A	concordant	unreportable	unreportable	357419	0	1	SCV000464045	Uncertain significance	Illumina Clinical Se	10/18/2016	PKHD1	chr6	51613144	51613144	http://gnomad.b	7.23E-05	SAS	9.75E-05	0		0	0	https://www.ncbi.nlm.nih.gov/clinvar/variation/357419/
NM_004543.4(NEB):c.8553G>A	concordant	unreportable	unreportable	281363	0	2	SCV000332124;S(	Uncertain significance;Beni	EGL Genetic Diagn	2017-06-30;2017-1	NEB	chr2	152497001	152497001	http://gnomad.b	0.0002943029151	AFR	0.002876949962	0		1	0	https://www.ncbi.nlm.nih.gov/clinvar/variation/281363/
NM_000492.3(CFTR):c.2604A>G	concordant	unreportable	unreportable	455770	0	1	SCV000625738	Likely benign	Invitae	10/5/2017	CFTR	chr7	117235097	117235097	http://gnomad.b	2.89E-05	AMR	2.98E-05	0		0	0	https://www.ncbi.nlm.nih.gov/clinvar/variation/455770/
NM_017890.4(VPS13B):c.1981G>A	concordant	unreportable	unreportable	282709	0	1	SCV000334298	Uncertain significance	EGL Genetic Diagn	6/30/2017	VPS13B	chr8	100160206	100160206	http://gnomad.b	0.0004227441978	NFE	0.0006537699956	0		0	0	https://www.ncbi.nlm.nih.gov/clinvar/variation/282709/
NM_138694.3(PKHD1):c.3851C>T	concordant	unreportable	unreportable	500894	0	1	SCV000707040	Uncertain significance	EGL Genetic Diagn	6/30/2017	PKHD1	chr6	51890757	51890757	http://gnomad.b	0.0001640182811	NFE	0.0001980340021	0		0	0	https://www.ncbi.nlm.nih.gov/clinvar/variation/500894/
NM_000035.3(ALDOB):c.264C>A	concordant	unreportable	unreportable	285374	0	2	SCV000338368;S(	Uncertain significance;Unc	EGL Genetic Diagn	2017-06-30;2017-1	ALDOB	chr9	104192097	104192097	http://gnomad.b	0.0002284702086	SAS	0.003313620109	0		0	0	https://www.ncbi.nlm.nih.gov/clinvar/variation/285374/
NM_000098.2(CPT2):c.1839G>T	concordant	unreportable	unreportable	460429	0	1	SCV000632599	Likely benign	Invitae	10/5/2017	CPT2	chr1	53679129	53679129	http://gnomad.b	4.76E-05	AMR	0.0002084449952	0		0	0	https://www.ncbi.nlm.nih.gov/clinvar/variation/460429/
NM_000271.4(NPC1):c.1224C>T	concordant	unreportable	unreportable	289987	0	1	SCV000344458	Uncertain significance	EGL Genetic Diagn	6/30/2017	NPC1	chr18	21136309	21136309	http://gnomad.b	5.72E-06	EAS	5.80E-05	0		0	0	https://www.ncbi.nlm.nih.gov/clinvar/variation/289987/
NM_000391.3(TPP1):c.83G>A	concordant	unreportable	unreportable	448692	0	1	SCV000615831	Uncertain significance	Athena Diagnostics	8/17/2017	TPP1	chr11	6640433	6640433	http://gnomad.b	6.16E-05	NFE	3.58E-05	0		0	0	https://www.ncbi.nlm.nih.gov/clinvar/variation/448692/
NM_000053.3(ATP7B):c.2806T>G	concordant	unreportable	unreportable	283616	0	1	SCV000335817	Uncertain significance	EGL Genetic Diagn	6/30/2017	ATP7B	chr13	52523857	52523857	http://gnomad.b	7.30E-05	AFR	6.55E-05	0		0	0	https://www.ncbi.nlm.nih.gov/clinvar/variation/283616/
NM_000071.2(CBS):c.1257G>A	concordant	unreportable	unreportable	194318	0	1	SCV000649824	Benign	Invitae	10/5/2017	CBS	chr21	44479045	44479045	http://gnomad.b	0.0003261927124	AFR	0.003613370005	0		0	0	https://www.ncbi.nlm.nih.gov/clinvar/variation/194318/
NM_000137.2(FAH):c.553+10C>T	concordant	unreportable	unreportable	385421	0	1	SCV000526675	Likely benign	GeneDx	11/28/2017	FAH	chr15	80460501	80460501	http://gnomad.b	0.0003634224967	AMR	0.001072190003	1		0	0	https://www.ncbi.nlm.nih.gov/clinvar/variation/385421/
NM_000098.2(CPT2):c.98delA	concordant	reportable	reportable	495549	1	0	SCV000695407	Pathogenic	Laboratory Corpor	1/25/2018	CPT2	chr1	53662712	53662713	http://gnomad.b	1.12E-05	ASJ	0.0001274209935	0		0	0	https://www.ncbi.nlm.nih.gov/clinvar/variation/495549/
NM_000466.2(PEX1):c.2640T>C	concordant	unreportable	unreportable	502747	0	1	SCV000709606	Uncertain significance	EGL Genetic Diagn	6/30/2017	PEX1	chr7	92129096	92129096	http://gnomad.b	0.0001858210238	AMR	0.0008934420184	0		0	0	https://www.ncbi.nlm.nih.gov/clinvar/variation/502747/
NM_000391.3(TPP1):c.14C>A	concordant	unreportable	unreportable	92884	0	4	SCV000615825;S(	Uncertain significance;Unc	Athena Diagnostics	2017-08-17;2017-0	TPP1	chr11	6640618	6640618	http://gnomad.b	0.001046440703	FIN	0.002511210041	0		2	0	https://www.ncbi.nlm.nih.gov/clinvar/variation/92884/
NM_001079802.1(FKTN):c.1337A>T	concordant	unreportable	unreportable	432249	0	1	SCV000589960	Uncertain significance	GeneDx	11/28/2017	FKTN	chr9	108397496	108397496	http://gnomad.b	0.0001347412613	SAS	0.002209230093	0		2	0	https://www.ncbi.nlm.nih.gov/clinvar/variation/432249/
NM_138694.3(PKHD1):c.3242G>A	concordant	unreportable	unreportable	196190	0	1	SCV000228738	Uncertain significance	EGL Genetic Diagn	6/30/2017	PKHD1	chr6	51897950	51897950	http://gnomad.b	3.61E-05	AFR	0.0001307530038	0		0	0	https://www.ncbi.nlm.nih.gov/clinvar/variation/196190/
NM_000057.3(BLM):c.3558+1G>T	concordant	reportable	reportable	220019	1	0	SCV000260243	Pathogenic	Invitae	10/5/2017	BLM	chr15	91346951	91346951	http://gnomad.b	3.81E-05	AFR	6.54E-05	0		0	0	https://www.ncbi.nlm.nih.gov/clinvar/variation/220019/
NM_004543.4(NEB):c.7342C>T	concordant	unreportable	unreportable	331492	0	2	SCV000416967;S(	Uncertain significance;Likel	Illumina Clinical Se	2016-10-18;2017-1	NEB	chr2	152506779	152506779	http://gnomad.b	0.0003097925423	EAS	0.004697829951	1		0	0	https://www.ncbi.nlm.nih.gov/clinvar/variation/331492/
NM_000360.3(TH):c.363G>A	concordant	unreportable	unreportable	304080	0	2	SCV000369924;S(	Uncertain significance;Likel	Illumina Clinical Se	2016-10-18;2017-1	TH	chr11	2189845	2189845	http://gnomad.b	0.0004253736809	NFE	0.0005364710232	0		0	0	https://www.ncbi.nlm.nih.gov/clinvar/variation/304080/
NM_000051.3(ATM):c.7038A>T	concordant	unreportable	unreportable	186841	0	2	SCV000687752;S(	Likely benign;Likely benign	Color Genomics, In	2017-12-21;2017-1	ATM	chr11	108198434	108198434		1.39E-05					0	0	https://www.ncbi.nlm.nih.gov/clinvar/variation/186841/
NM_000136.2(FANCC):c.906C>T	concordant	unreportable	unreportable	456172	0	1	SCV000626263	Likely benign	Invitae	10/5/2017	FANCC	chr9	97887458	97887458		2.38E-06					0	0	https://www.ncbi.nlm.nih.gov/clinvar/variation/456172/
NM_138694.3(PKHD1):c.10684C>A	concordant	unreportable	unreportable	291149	0	2	SCV000345855;S(	Uncertain significance;Unc	EGL Genetic Diagn	2017-06-30;2016-1	PKHD1	chr6	51524240	51524240	http://gnomad.b	0.0001084188638	NFE	0.0001350889943	0		0	0	https://www.ncbi.nlm.nih.gov/clinvar/variation/291149/
NM_000023.2(SGCA):c.408C>T	concordant	unreportable	unreportable	287947	0	1	SCV000341906	Uncertain significance	EGL Genetic Diagn	6/30/2017	SGCA	chr17	48245757	48245757	http://gnomad.b	0.0001315332863	SAS	0.0006538960151	0		1	0	https://www.ncbi.nlm.nih.gov/clinvar/variation/287947/
NM_004543.4(NEB):c.7514C>A	concordant	unreportable	unreportable	95135	0	2	SCV000513916;S(	Likely benign;Uncertain sig	GeneDx;Illumina C	2017-11-28;2016-1	NEB	chr2	152502666	152502666	http://gnomad.b	0.0007460837059	AFR	0.009846200235	1		0	0	https://www.ncbi.nlm.nih.gov/clinvar/variation/95135/
NM_000053.3(ATP7B):c.2976C>A	concordant	unreportable	unreportable	254766	0	1	SCV000709305	Uncertain significance	EGL Genetic Diagn	6/30/2017	ATP7B	chr13	52520504	52520504	http://gnomad.b	4.21E-05	SAS	0.0003585400118	1		0	0	https://www.ncbi.nlm.nih.gov/clinvar/variation/254766/
NM_000543.4(SMPD1):c.107_130del2	concordant	unreportable	unreportable	459633	0	1	SCV000631409	Likely benign	Invitae	10/5/2017	SMPD1	chr11	6411930	6411954	http://gnomad.b	0.001291627022	SAS	0.007240850013	3		1	0	https://www.ncbi.nlm.nih.gov/clinvar/variation/459633/
NM_033056.3(PCDH15):c.5435C>T	concordant	unreportable	unreportable	227011	0	3	SCV000332669;S(	Uncertain significance;Unc	EGL Genetic Diagn	2017-06-30;2017-1	PCDH15	chr10	55582051	55582051	http://gnomad.b	0.0004423185722	EAS	0.001649189973	1		5	0	https://www.ncbi.nlm.nih.gov/clinvar/variation/227011/
NM_000153.3(GALC):c.1717A>G	concordant	unreportable	unreportable	287587	0	1	SCV000341395	Uncertain significance	EGL Genetic Diagn	6/30/2017	GALC	chr14	88407856	88407856	http://gnomad.b	0.0001762158896	AMR	0.001941459952	0		0	0	https://www.ncbi.nlm.nih.gov/clinvar/variation/287587/
NM_000060.2(BTD):c.907C>T	concordant	unreportable	unreportable	343908	0	1	SCV000441827	Uncertain significance	Illumina Clinical Se	10/18/2016	BTD	chr3	15686270	15686270	http://gnomad.b	0.0002566039477	EAS	0.001913710032	1		0	0	https://www.ncbi.nlm.nih.gov/clinvar/variation/343908/
NM_002485.4(NBN):c.2202A>G	concordant	unreportable	unreportable	136042	0	3	SCV000685760;S(	Likely benign;Likely benign	Color Genomics, In	2017-10-26;2017-1	NBN	chr8	90949286	90949286	http://gnomad.b	0.0001115448968	NFE	0.0003333929926	0		0	0	https://www.ncbi.nlm.nih.gov/clinvar/variation/136042/
NM_000288.3(PEX7):c.44C>T	concordant	unreportable	unreportable	447933	0	1	SCV000614439	Uncertain significance	Athena Diagnostics	8/17/2017	PEX7	chr6	137143847	137143847		5.28E-05					0	0	https://www.ncbi.nlm.nih.gov/clinvar/variation/447933/
NM_000151.3(G6PC):c.81G>C	concordant	unreportable	unreportable	323359	0	1	SCV000402974	Uncertain significance	Illumina Clinical Se	10/18/2016	G6PC	chr17	41052974	41052974	http://gnomad.b	0.0001456779648	NFE	0.0002864559938	0		0	0	https://www.ncbi.nlm.nih.gov/clinvar/variation/323359/
NM_000071.2(CBS):c.435G>A	concordant	unreportable	unreportable	92425	0	1	SCV000649839	Likely benign	Invitae	10/5/2017	CBS	chr21	44486369	44486369	http://gnomad.b	1.41E-05	AFR	6.55E-05	0		0	0	https://www.ncbi.nlm.nih.gov/clinvar/variation/92425/
NM_001042432.1(CLN3):c.238A>T	concordant	unreportable	unreportable	418136	0	1	SCV000564887	Uncertain significance	GeneDx	11/28/2017	CLN3	chr16	28499968	28499968	http://gnomad.b	0.0002111064199	AFR	0.002809720114	0		1	0	https://www.ncbi.nlm.nih.gov/clinvar/variation/418136/
NM_000136.2(FANCC):c.1030A>G	concordant	unreportable	unreportable	456149	0	1	SCV000626229	Uncertain significance	Invitae	10/5/2017	FANCC	chr9	97879639	97879639		2.38E-06					0	0	https://www.ncbi.nlm.nih.gov/clinvar/variation/456149/
NM_000051.3(ATM):c.125A>G	concordant	unreportable	unreportable	127335	0	3	SCV000149048;S(	Likely benign;Uncertain sig	GeneDx;Invitae;La	2017-11-28;2017-1	ATM	chr11	108098555	108098555	http://gnomad.b	0.0002534084833	EAS	0.001623379998	0		0	0	https://www.ncbi.nlm.nih.gov/clinvar/variation/127335/
NM_000071.2(CBS):c.1593C>T	concordant	unreportable	unreportable	413354	0	1	SCV000555948	Likely benign	Invitae	3/14/2017	CBS	chr21	44474053	44474053	http://gnomad.b	4.78E-05	NFE	2.71E-05	0		0	0	https://www.ncbi.nlm.nih.gov/clinvar/variation/413354/
NM_002485.4(NBN):c.506G>A	concordant	unreportable	unreportable	186682	0	3	SCV000685793;S(	Uncertain significance;Unc	Color Genomics, In	2017-10-26;2017-1	NBN	chr8	90990526	90990526	http://gnomad.b	1.95E-05	SAS	6.50E-05	0		0	0	https://www.ncbi.nlm.nih.gov/clinvar/variation/186682/
NM_024649.4(BBS1):c.1772C>T	concordant	unreportable	unreportable	391252	0	1	SCV000534280	Uncertain significance	GeneDx	11/28/2017	BBS1	chr11	66299498	66299498		0.0002117817533					0	0	https://www.ncbi.nlm.nih.gov/clinvar/variation/391252/
NM_024649.4(BBS1):c.1719A>G	concordant	unreportable	unreportable	305472	0	2	SCV000373282;S(	Uncertain significance;Beni	Illumina Clinical Se	2016-10-18;2017-1	BBS1	chr11	66299445	66299445	http://gnomad.b	0.0008610600234	AFR	0.007026629988	0		1	0	https://www.ncbi.nlm.nih.gov/clinvar/variation/305472/
NM_001360.2(DHCR7):c.927C>A	concordant	unreportable	unreportable	289153	0	2	SCV000343456;S(	Uncertain significance;Unc	EGL Genetic Diagn	2017-06-30;2016-1	DHCR7	chr11	71148894	71148894	http://gnomad.b	0.0003742676202	NFE	0.0007251829957	0		0	0	https://www.ncbi.nlm.nih.gov/clinvar/variation/289153/
NM_017890.4(VPS13B):c.4697C>T	concordant	unreportable	unreportable	196183	0	1	SCV000228722	Uncertain significance	EGL Genetic Diagn	6/30/2017	VPS13B	chr8	100523729	100523729	http://gnomad.b	3.64E-05	AFR	0.0002638169972	0		0	0	https://www.ncbi.nlm.nih.gov/clinvar/variation/196183/
NM_000466.2(PEX1):c.891A>G	concordant	unreportable	unreportable	287502	0	1	SCV000341291	Uncertain significance	EGL Genetic Diagn	6/30/2017	PEX1	chr7	92146938	92146938	http://gnomad.b	4.99E-05	AFR	0.0005228759837	0		1	0	https://www.ncbi.nlm.nih.gov/clinvar/variation/287502/
NM_000051.3(ATM):c.4321A>C	concordant	unreportable	unreportable	453514	0	2	SCV000687539;S(	Uncertain significance;Unc	Color Genomics, In	2017-12-21;2017-1	ATM	chr11	108160413	108160413	http://gnomad.b	2.78E-06	NFE	2.69E-05	0		0	0	https://www.ncbi.nlm.nih.gov/clinvar/variation/453514/
NM_000051.3(ATM):c.1911A>G	concordant	unreportable	unreportable	229864	0	3	SCV000682015;S(	Likely benign;Likely benign	Color Genomics, In	2017-10-26;2017-1	ATM	chr11	108124553	108124553	http://gnomad.b	1.11E-05	AFR	0.0001965669944	0		0	0	https://www.ncbi.nlm.nih.gov/clinvar/variation/229864/
NM_005562.2(LAMC2):c.3273T>G	concordant	unreportable	unreportable	294018	0	1	SCV000351694	Uncertain significance	Illumina Clinical Se	10/18/2016	LAMC2	chr1	183209471	183209471	http://gnomad.b	6.44E-05	NFE	6.27E-05	0		0	0	https://www.ncbi.nlm.nih.gov/clinvar/variation/294018/
NM_017890.4(VPS13B):c.5816G>A	concordant	unreportable	unreportable	196917	0	1	SCV000470816	Uncertain significance	Illumina Clinical Se	10/18/2016	VPS13B	chr8	100654559	100654559	http://gnomad.b	0.0003891486324	SAS	0.004288219847	1		1	0	https://www.ncbi.nlm.nih.gov/clinvar/variation/196917/
NM_033056.3(PCDH15):c.5313T>G	concordant	unreportable	unreportable	300172	0	1	SCV000363119	Uncertain significance	Illumina Clinical Se	10/18/2016	PCDH15	chr10	55582173	55582173		2.57E-06					0	0	https://www.ncbi.nlm.nih.gov/clinvar/variation/300172/
NM_000227.3(LAMA3):c.128A>C	concordant	unreportable	unreportable	326293	0	1	SCV000407907	Uncertain significance	Illumina Clinical Se	10/18/2016	LAMA3	chr18	21453136	21453136	http://gnomad.b	5.04E-05	NFE	8.06E-05	0		0	0	https://www.ncbi.nlm.nih.gov/clinvar/variation/326293/
NM_138694.3(PKHD1):c.7533A>G	concordant	unreportable	unreportable	290898	0	2	SCV000345565;S(	Uncertain significance;Unc	EGL Genetic Diagn	2017-06-30;2016-1	PKHD1	chr6	51732861	51732861	http://gnomad.b	0.0003057967952	ASJ	0.001119249966	0		0	0	https://www.ncbi.nlm.nih.gov/clinvar/variation/290898/
NM_138694.3(PKHD1):c.935G>A	concordant	unreportable	unreportable	290975	0	1	SCV000345647	Uncertain significance	EGL Genetic Diagn	6/30/2017	PKHD1	chr6	51929794	51929794	http://gnomad.b	0.0002640972323	NFE	0.000252007012	0		0	0	https://www.ncbi.nlm.nih.gov/clinvar/variation/290975/
NM_000228.2(LAMB3):c.1546A>G	concordant	unreportable	unreportable	295106	0	1	SCV000353631	Uncertain significance	Illumina Clinical Se	10/18/2016	LAMB3	chr1	209800263	209800263	http://gnomad.b	0.0001035828467	EAS	0.0002905630099	0		0	0	https://www.ncbi.nlm.nih.gov/clinvar/variation/295106/
NM_000016.4(ACADM):c.443G>A	concordant	reportable	reportable	198025	4	0	SCV000330907;S(	Pathogenic;Pathogenic;Pat	EGL Genetic Diagn	2017-06-30;2017-1	ACADM	chr1	76200531	76200531	http://gnomad.b	5.98E-05	AMR	0.001012569992	0		0	0	https://www.ncbi.nlm.nih.gov/clinvar/variation/198025/
NM_017890.4(VPS13B):c.11862C>T	concordant	unreportable	unreportable	262656	0	1	SCV000470867	Uncertain significance	Illumina Clinical Se	10/18/2016	VPS13B	chr8	100887687	100887687	http://gnomad.b	0.0004199445673	EAS	0.005160020199	0		0	0	https://www.ncbi.nlm.nih.gov/clinvar/variation/262656/
NM_000018.3(ACADVL):c.1533-4T>A	concordant	unreportable	unreportable	474890	0	1	SCV000654937	Likely benign	Invitae	10/5/2017	ACADVL	chr17	7127636	7127636	http://gnomad.b	0.0003822846463	ASJ	0.001727640047	0		0	0	https://www.ncbi.nlm.nih.gov/clinvar/variation/474890/
NM_000227.3(LAMA3):c.695G>A	concordant	unreportable	unreportable	326300	0	1	SCV000407923	Uncertain significance	Illumina Clinical Se	10/18/2016	LAMA3	chr18	21474931	21474931	http://gnomad.b	0.0002127766797	AFR	0.002881840104	0		1	0	https://www.ncbi.nlm.nih.gov/clinvar/variation/326300/
NM_000466.2(PEX1):c.1249G>A	concordant	unreportable	unreportable	498412	0	1	SCV000703405	Uncertain significance	EGL Genetic Diagn	6/30/2017	PEX1	chr7	92143272	92143272	http://gnomad.b	0.000141445854	NFE	0.0002091780043	0		0	0	https://www.ncbi.nlm.nih.gov/clinvar/variation/498412/
NM_014363.4(SACS):c.10274A>G	concordant	unreportable	unreportable	286521	0	3	SCV000614920;S(	Uncertain significance;Likel	Athena Diagnostics	2017-08-17;2017-0	SACS	chr13	23907741	23907741	http://gnomad.b	0.000533529356	AFR	0.004708339926	0		0	0	https://www.ncbi.nlm.nih.gov/clinvar/variation/286521/
NM_005476.5(GNE):c.1509C>G	concordant	unreportable	unreportable	288432	0	2	SCV000342540;S(	Uncertain significance;Likel	EGL Genetic Diagn	2017-06-30;2017-1	GNE	chr9	36222898	36222898	http://gnomad.b	0.0005547150782	SAS	0.002209079918	1		0	0	https://www.ncbi.nlm.nih.gov/clinvar/variation/288432/
NM_000152.3(GAA):c.2395C>T	concordant	unreportable	unreportable	284886	0	1	SCV000337689	Uncertain significance	EGL Genetic Diagn	6/30/2017	GAA	chr17	78091462	78091462	http://gnomad.b	7.23E-05	AFR	0.0003313010093	0		0	0	https://www.ncbi.nlm.nih.gov/clinvar/variation/284886/

Supplemental Table 2

NM_138694.3(PKHD1):c.9925A>G	concordant	unreportable	unreportable	441087	0	1	SCV000703414	Uncertain significance	EGL Genetic Diagn	6/30/2017	PKHD1	chr6	51611592	51611592	http://gnomad.b	0.0002362975236	NFE	0.0003774400102	0		0	0	https://www.ncbi.nlm.nih.gov/clinvar/variation/441087/
NM_004328.4(BCS1L):c.460+11delG	concordant	unreportable	unreportable	420719	0	1	SCV000569669	Likely benign	GeneDx	11/28/2017	BCS1L	chr2	219526277	219526278	http://gnomad.b	1.12E-05	AFR	0.0001962069946	0		0	0	https://www.ncbi.nlm.nih.gov/clinvar/variation/420719/
NM_000466.2(PEX1):c.3198T>C	concordant	unreportable	unreportable	499941	0	1	SCV000705678	Uncertain significance	EGL Genetic Diagn	6/30/2017	PEX1	chr7	92122276	92122276	http://gnomad.b	2.77E-05	AFR	0.0001307019993	0		0	0	https://www.ncbi.nlm.nih.gov/clinvar/variation/499941/
NM_006493.2(CLN5):c.227G>A	concordant	unreportable	unreportable	205128	0	1	SCV000240624	Uncertain significance	GeneDx	11/28/2017	CLN5	chr13	77566313	77566313	http://gnomad.b	1.40E-05	AMR	0.0002134929964	0		0	0	https://www.ncbi.nlm.nih.gov/clinvar/variation/205128/
NM_000518.4(HBB):c.432C>T	concordant	unreportable	unreportable	36329	0	1	SCV000697132	Uncertain significance	Laboratory Corpor	1/25/2018	HBB	chr11	5246840	5246840	http://gnomad.b	0.000475866123	AFR	0.004966670182	1		0	0	https://www.ncbi.nlm.nih.gov/clinvar/variation/36329/
NM_000053.3(ATP7B):c.3105C>T	concordant	unreportable	unreportable	312385	0	1	SCV000384661	Uncertain significance	Illumina Clinical Se	10/18/2016	ATP7B	chr13	52518383	52518383	http://gnomad.b	0.0001487126535	AFR	0.0007252109936	0		0	0	https://www.ncbi.nlm.nih.gov/clinvar/variation/312385/
NM_000642.2(AGL):c.4052A>G	concordant	unreportable	unreportable	456500	0	1	SCV000626747	Uncertain significance	Invitae	10/5/2017	AGL	chr1	100379185	100379185	http://gnomad.b	0.0001088491574	NFE	0.0003050040104	0		0	0	https://www.ncbi.nlm.nih.gov/clinvar/variation/456500/
NM_017739.3(POMGNT1):c.1195G>A	concordant	unreportable	unreportable	502560	0	1	SCV000709351	Uncertain significance	EGL Genetic Diagn	6/30/2017	POMGNT1	chr1	46658588	46658588		8.40E-06					0	0	https://www.ncbi.nlm.nih.gov/clinvar/variation/502560/
NM_000018.3(ACADVL):c.495G>T	concordant	unreportable	unreportable	92285	0	2	SCV000602353;S	Uncertain significance;Unc	ARUP Laboratories	2017-06-30;2017-0	ACADVL	chr17	7124874	7124874	http://gnomad.b	3.11E-05	NFE	9.85E-05	0		0	0	https://www.ncbi.nlm.nih.gov/clinvar/variation/92285/
NM_001042432.1(CLN3):c.975G>A	concordant	unreportable	unreportable	136782	0	1	SCV000628942	Likely benign	Invitae	10/5/2017	CLN3	chr16	28493507	28493507	http://gnomad.b	6.67E-05	NFE	0.0001178669991	0		0	0	https://www.ncbi.nlm.nih.gov/clinvar/variation/136782/
NM_003060.3(SLC22A5):c.1181_1183	concordant	unreportable	unreportable	25410	1	1	SCV000239182;S	Likely pathogenic;Uncertai	GeneDx;Invitae	2017-11-28;2017-1	SLC22A5	chr5	131726501	131726504	http://gnomad.b	8.75E-05	AMR	5.96E-05	0		0	0	https://www.ncbi.nlm.nih.gov/clinvar/variation/25410/
NM_000271.4(NPC1):c.2832C>T	concordant	unreportable	unreportable	326255	0	1	SCV000407854	Uncertain significance	Illumina Clinical Se	10/18/2016	NPC1	chr18	21119398	21119398	http://gnomad.b	3.15E-05	SAS	0.0001969279983	0		0	0	https://www.ncbi.nlm.nih.gov/clinvar/variation/326255/
NM_014363.4(SACS):c.4612A>G	concordant	unreportable	unreportable	448204	0	2	SCV000614957;S	Uncertain significance;Unc	Athena Diagnostics	2017-08-17;2017-1	SACS	chr13	23913403	23913403	http://gnomad.b	2.07E-05	AMR	6.39E-05	0		0	0	https://www.ncbi.nlm.nih.gov/clinvar/variation/448204/
NM_000642.2(AGL):c.2158-18_2158-1	concordant	unreportable	unreportable	422718	0	1	SCV000572255	Likely benign	GeneDx	11/28/2017	AGL	chr1	100347075	100347078	http://gnomad.b	0.0001507142179	NFE	0.000269000011	0		0	0	https://www.ncbi.nlm.nih.gov/clinvar/variation/422718/
NM_000057.3(BLM):c.1270_1272delC	concordant	unreportable	unreportable	454073	0	1	SCV000623232	Uncertain significance	Invitae	10/5/2017	BLM	chr15	91303868	91303871	http://gnomad.b	3.57E-05	NFE	8.99E-06	0		0	0	https://www.ncbi.nlm.nih.gov/clinvar/variation/454073/
NM_000243.2(MEFV):c.97G>T	concordant	unreportable	unreportable	36516	0	2	SCV000329418;S	Uncertain significance;Unc	GeneDx;Laborator	2017-11-28;2018-0	MEFV	chr16	3306491	3306491	http://gnomad.b	0.0005513574027	AFR	0.007775740232	1		1	0	https://www.ncbi.nlm.nih.gov/clinvar/variation/36516/
NM_005476.5(GNE):c.2004C>T	concordant	unreportable	unreportable	284820	0	1	SCV000337608	Uncertain significance	EGL Genetic Diagn	6/30/2017	GNE	chr9	36217527	36217527	http://gnomad.b	0.0001512859304	AFR	0.001437530038	0		0	0	https://www.ncbi.nlm.nih.gov/clinvar/variation/284820/
NM_000391.3(TPP1):c.1490G>A	concordant	unreportable	unreportable	457942	0	1	SCV000628905	Uncertain significance	Invitae	10/5/2017	TPP1	chr11	6636158	6636158	http://gnomad.b	5.32E-05	SAS	0.0002923790016	0		0	0	https://www.ncbi.nlm.nih.gov/clinvar/variation/457942/
NM_000050.4(ASS1):c.539G>T	concordant	unreportable	unreportable	458674	0	1	SCV000630061	Uncertain significance	Invitae	10/5/2017	ASS1	chr9	133346264	133346264	http://gnomad.b	1.95E-05					0	0	https://www.ncbi.nlm.nih.gov/clinvar/variation/458674/
NM_000391.3(TPP1):c.1086G>A	concordant	unreportable	unreportable	378759	0	1	SCV000514962	Likely benign	GeneDx	11/28/2017	TPP1	chr11	6637295	6637295	http://gnomad.b	5.88E-05	AFR	0.0008495619986	0		0	0	https://www.ncbi.nlm.nih.gov/clinvar/variation/378759/
NM_000492.3(CFTR):c.3815T>A	concordant	unreportable	unreportable	195732	0	2	SCV000228014;S	Uncertain significance;Unc	EGL Genetic Diagn	2017-06-30;2018-0	CFTR	chr7	117282589	117282589	http://gnomad.b	9.63E-06	AMR	5.97E-05	0		0	0	https://www.ncbi.nlm.nih.gov/clinvar/variation/195732/
NM_000152.3(GAA):c.1356C>T	concordant	unreportable	unreportable	286268	0	1	SCV000339627	Uncertain significance	EGL Genetic Diagn	6/30/2017	GAA	chr17	78083773	78083773	http://gnomad.b	6.11E-05	AMR	0.0001489599963	0		0	0	https://www.ncbi.nlm.nih.gov/clinvar/variation/286268/
NM_000137.2(FAH):c.715A>T	concordant	unreportable	unreportable	290111	0	1	SCV000344608	Uncertain significance	EGL Genetic Diagn	6/30/2017	FAH	chr15	80465364	80465364	http://gnomad.b	0.0002690991006	AMR	0.006342680193	0		0	0	https://www.ncbi.nlm.nih.gov/clinvar/variation/290111/
NM_174878.2(CLRN1):c.660C>T	concordant	unreportable	unreportable	178318	0	1	SCV000441711	Uncertain significance	Illumina Clinical Se	10/18/2016	CLRN1	chr3	150645762	150645762	http://gnomad.b	0.0006377715029	ASJ	0.003352300031	0		1	0	https://www.ncbi.nlm.nih.gov/clinvar/variation/178318/
NM_006493.2(CLN5):c.299G>A	concordant	unreportable	unreportable	285012	0	2	SCV000337848;S	Uncertain significance;Unc	EGL Genetic Diagn	2017-06-30;2017-1	CLN5	chr13	77566385	77566385		6.15E-05					0	0	https://www.ncbi.nlm.nih.gov/clinvar/variation/285012/
NM_000271.4(NPC1):c.3558C>T	concordant	unreportable	unreportable	502703	0	1	SCV000709548	Uncertain significance	EGL Genetic Diagn	6/30/2017	NPC1	chr18	21114443	21114443	http://gnomad.b	4.86E-05	AFR	0.0002614379919	0		0	0	https://www.ncbi.nlm.nih.gov/clinvar/variation/502703/
NM_000466.2(PEX1):c.2926+8T>C	concordant	unreportable	unreportable	291261	0	1	SCV000345979	Uncertain significance	EGL Genetic Diagn	6/30/2017	PEX1	chr7	92123793	92123793	http://gnomad.b	0.0002052351607	AFR	0.002680090023	0		1	0	https://www.ncbi.nlm.nih.gov/clinvar/variation/291261/
NM_000228.2(LAMB3):c.3124C>T	concordant	unreportable	unreportable	295070	0	1	SCV000353588	Uncertain significance	Illumina Clinical Se	10/18/2016	LAMB3	chr1	209790859	209790859	http://gnomad.b	0.0006466929077	AFR	0.004835340194	0		2	0	https://www.ncbi.nlm.nih.gov/clinvar/variation/295070/
NM_000152.3(GAA):c.1075+9T>C	concordant	unreportable	unreportable	291212	0	1	SCV000345924	Uncertain significance	EGL Genetic Diagn	6/30/2017	GAA	chr17	78082217	78082217	http://gnomad.b	3.89E-05	NFE	4.49E-05	0		0	0	https://www.ncbi.nlm.nih.gov/clinvar/variation/291212/
NM_000642.2(AGL):c.3764A>G	concordant	unreportable	unreportable	456497	0	1	SCV000626739	Likely benign	Invitae	10/5/2017	AGL	chr1	100376331	100376331	http://gnomad.b	0.0004884257063	AFR	0.004837229848	0		0	0	https://www.ncbi.nlm.nih.gov/clinvar/variation/456497/
NM_004646.3(NPHS1):c.1619C>A	concordant	unreportable	unreportable	447874	0	1	SCV000614338	Uncertain significance	Athena Diagnostics	8/17/2017	NPHS1	chr19	36336918	36336918	http://gnomad.b	0.0004543705992	AFR	0.0006711410242	0		0	0	https://www.ncbi.nlm.nih.gov/clinvar/variation/447874/
NM_138694.3(PKHD1):c.6216T>G	concordant	unreportable	unreportable	197050	0	1	SCV000229925	Uncertain significance	EGL Genetic Diagn	6/30/2017	PKHD1	chr6	51777280	51777280	http://gnomad.b	6.12E-05	AFR	0.0009151520208	0		0	0	https://www.ncbi.nlm.nih.gov/clinvar/variation/197050/
NM_000414.3(HSD17B4):c.1685G>A	concordant	unreportable	unreportable	501977	0	1	SCV000708535	Uncertain significance	EGL Genetic Diagn	6/30/2017	HSD17B4	chr5	118862832	118862832	http://gnomad.b	0.0001380522796	AFR	0.0007203670102	0		0	0	https://www.ncbi.nlm.nih.gov/clinvar/variation/501977/
NM_000053.3(ATP7B):c.2174G>A	concordant	unreportable	unreportable	456551	0	1	SCV000626837	Likely benign	Invitae	10/5/2017	ATP7B	chr13	52532628	52532628	http://gnomad.b	0.00015993625	AFR	0.002418929944	0		0	0	https://www.ncbi.nlm.nih.gov/clinvar/variation/456551/
NM_000543.4(SMPD1):c.762G>A	concordant	unreportable	unreportable	289880	0	1	SCV000344313	Uncertain significance	EGL Genetic Diagn	6/30/2017	SMPD1	chr11	6413057	6413057	http://gnomad.b	0.0003092293054	AFR	0.003220289946	0		0	0	https://www.ncbi.nlm.nih.gov/clinvar/variation/289880/
NM_138694.3(PKHD1):c.2269A>G	concordant	unreportable	unreportable	406884	0	1	SCV000545839	Uncertain significance	Invitae	10/5/2017	PKHD1	chr6	51914965	51914965	http://gnomad.b	2.78E-05	NFE	7.18E-05	0		0	0	https://www.ncbi.nlm.nih.gov/clinvar/variation/406884/
NM_000227.3(LAMA3):c.3802C>T	concordant	unreportable	unreportable	326335	0	1	SCV000408003	Uncertain significance	Illumina Clinical Se	10/18/2016	LAMA3	chr18	21512176	21512176	http://gnomad.b	0.0007587168446	AFR	0.008755880408	2		0	0	https://www.ncbi.nlm.nih.gov/clinvar/variation/326335/
NM_001360.2(DHCR7):c.582C>T	concordant	unreportable	unreportable	198131	0	1	SCV000231622	Uncertain significance	EGL Genetic Diagn	6/30/2017	DHCR7	chr11	71152317	71152317	http://gnomad.b	8.89E-05	AFR	0.0009147939854	0		0	0	https://www.ncbi.nlm.nih.gov/clinvar/variation/198131/
NM_000051.3(ATM):c.2770C>T	concordant	unreportable	unreportable	127358	0	4	SCV000682075;S	Uncertain significance;Unc	Color Genomics, In	2017-10-26;2017-1	ATM	chr11	108139268	108139268	http://gnomad.b	7.52E-05	ASJ	0.0005077170208	0		0	0	https://www.ncbi.nlm.nih.gov/clinvar/variation/127358/
NM_000153.3(GALC):c.500A>G	concordant	unreportable	unreportable	498178	0	1	SCV000703060	Uncertain significance	EGL Genetic Diagn	6/30/2017	GALC	chr14	88450820	88450820	http://gnomad.b	4.76E-05	SAS	9.75E-05	0		0	0	https://www.ncbi.nlm.nih.gov/clinvar/variation/498178/
NM_000528.3(MAN2B1):c.1273G>T	concordant	unreportable	unreportable	328271	0	1	SCV000410796	Uncertain significance	Illumina Clinical Se	10/18/2016	MAN2B1	chr19	12768913	12768913	http://gnomad.b	7.61E-05	AMR	0.000178731003	0		0	0	https://www.ncbi.nlm.nih.gov/clinvar/variation/328271/
NM_000152.3(GAA):c.1536C>T	concordant	unreportable	unreportable	284111	0	2	SCV000336595;S	Uncertain significance;Likel	EGL Genetic Diagn	2017-06-30;2017-1	GAA	chr17	78084624	78084624	http://gnomad.b	3.34E-05	AMR	8.93E-05	0		1	0	https://www.ncbi.nlm.nih.gov/clinvar/variation/284111/
NM_138694.3(PKHD1):c.1576A>G	concordant	unreportable	unreportable	284549	0	1	SCV000337211	Uncertain significance	EGL Genetic Diagn	6/30/2017	PKHD1	chr6	51921714	51921714	http://gnomad.b	0.0001334386016	AFR	0.0001307360071	0		0	0	https://www.ncbi.nlm.nih.gov/clinvar/variation/284549/
NM_001360.2(DHCR7):c.70G>T	concordant	unreportable	unreportable	93723	0	2	SCV000594363;S	Likely benign;Uncertain sig	Genetic Services La	2017-07-05;2016-1	DHCR7	chr11	71155929	71155929	http://gnomad.b	0.0006112140272	AFR	0.008102459833	0		0	0	https://www.ncbi.nlm.nih.gov/clinvar/variation/93723/
NM_033056.3(PCDH15):c.3127C>T	concordant	unreportable	unreportable	440027	0	1	SCV000604609	Uncertain significance	ARUP Laboratories	6/30/2017	PCDH15	chr10	55700731	55700731	http://gnomad.b	0.0001851566116	AMR	0.0005070689949	0		0	0	https://www.ncbi.nlm.nih.gov/clinvar/variation/440027/
NM_000492.3(CFTR):c.3204C>T	concordant	unreportable	unreportable	439072	0	2	SCV000625742;S	Likely benign;Uncertain sig	Invitae;Quest Diag	2017-10-05;2017-0	CFTR	chr7	117251699	117251699	http://gnomad.b	2.70E-05	SAS	9.75E-05	0		0	0	https://www.ncbi.nlm.nih.gov/clinvar/variation/439072/
NM_183050.2(BCKDHB):c.63G>T	concordant	unreportable	unreportable	358169	0	1	SCV000465664	Uncertain significance	Illumina Clinical Se	10/18/2016	BCKDHB	chr6	80816473	80816473	http://gnomad.b	0.0004971962234	SAS	0.003099980066	0		0	0	https://www.ncbi.nlm.nih.gov/clinvar/variation/358169/
NM_000108.3(DLD):c.677T>C	concordant	unreportable	unreportable	358568	0	1	SCV000466228	Uncertain significance	Illumina Clinical Se	10/18/2016	DLD	chr7	107546806	107546806	http://gnomad.b	1.56E-05	SAS	0.0001299709984	0		0	0	https://www.ncbi.nlm.nih.gov/clinvar/variation/358568/
NM_006261.4(PROP1):c.52G>A	concordant	unreportable	unreportable	288237	0	1	SCV000342287	Uncertain significance	EGL Genetic Diagn	6/30/2017	PROP1	chr5	177422883	177422883	http://gnomad.b	0.0001126785956	SAS	0.002111620037	2		3	0	https://www.ncbi.nlm.nih.gov/clinvar/variation/288237/
NM_000152.3(GAA):c.2725G>A	concordant	unreportable	unreportable	456414	0	1	SCV000626595	Uncertain significance	Invitae	10/5/2017	GAA	chr17	78092530	78092530	http://gnomad.b	0.0001139515622	SAS	9.75E-05	0		0	0	https://www.ncbi.nlm.nih.gov/clinvar/variation/456414/
NM_000051.3(ATM):c.7209C>T	concordant	unreportable	unreportable	236767	0	1	SCV000283042	Likely benign	Invitae	10/5/2017	ATM	chr11	108199867	108199867	http://gnomad.b	8.35E-06	AMR	8.93E-05	0		0	0	https://www.ncbi.nlm.nih.gov/clinvar/variation/236767/
NM_024649.4(BBS1):c.1181G>A	concordant	unreportable	unreportable	434479	0	2	SCV000702119;S	Uncertain significance;Unc	EGL Genetic Diagn	2017-06-30;2017-0	BBS1	chr11	66294120	66294120	http://gnomad.b	0.0002925932118	AFR	0.00294041005	0		0	0	https://www.ncbi.nlm.nih.gov/clinvar/variation/434479/
NM_000528.3(MAN2B1):c.1435G>T	concordant	unreportable	unreportable	328269	0	1	SCV000410793	Uncertain significance	Illumina Clinical Se	10/18/2016	MAN2B1	chr19	12767855	12767855	http://gnomad.b	3.94E-05	FIN	0.0007217780221	0		0	0	https://www.ncbi.nlm.nih.gov/clinvar/variation/328269/
NM_138694.3(PKHD1):c.1829A>G	concordant	unreportable	unreportable	369725	0	1	SCV000484486	Uncertain significance	EGL Genetic Diagn	6/30/2017	PKHD1	chr6	51920392	51920392	http://gnomad.b	1.67E-05	AFR	6.55E-05	0		0	0	https://www.ncbi.nlm.nih.gov/clinvar/variation/369725/
NM_000050.4(ASS1):c.241G>A	concordant	unreportable	unreportable	365252	0	1	SCV000477619	Uncertain significance	Illumina Clinical Se	10/18/2016	ASS1	chr9	133333854	133333854	http://gnomad.b	0.0002952202176	SAS	0.001234570052	0		0	0	https://www.ncbi.nlm.nih.gov/clinvar/variation/365252/
NM_002225.3(IVD):c.20C>T	concordant	unreportable	unreportable	284237	0	1	SCV000336769	Likely benign	EGL Genetic Diagn	6/30/2017	IVD	chr15	40698039	40698039	http://gnomad.b	0.000344667186	AFR	0.005272869952	0		0	0	https://www.ncbi.nlm.nih.gov/clinvar/variation/284237/
NM_000051.3(ATM):c.2187C>T	concordant	unreportable	unreportable	220365	0	2	SCV000682037;S	Likely benign;Likely benign	Color Genomics, In	2017-10-26;2016-0	ATM	chr11	108127004	108127004	http://gnomad.b	3.62E-05	NFE	3.58E-05	0		0	0	https://www.ncbi.nlm.nih.gov/clinvar/variation/220365/
NM_017739.3(POMGNT1):c.507C>T	concordant	unreportable	unreportable	392733	0	1	SCV000536050	Likely benign	GeneDx	11/28/2017	POMGNT1	chr1	46661510	46661510	http://gnomad.b	5.32E-05	EAS	0.0003584660008	0		0	0	https://www.ncbi.nlm.nih.gov/clinvar/variation/392733/
NM_006493.2(CLN5):c.322C>T	concordant	unreportable	unreportable	451777	0	1	SCV000620522	Uncertain significance	GeneDx	11/28/2017	CLN5	chr13	77569199	77569199	http://gnomad.b	8.39E-06	NFE	8.97E-06	0		0	0	https://www.ncbi.nlm.nih.gov/clinvar/variation/451777/
NM_000518.4(HBB):c.180G>A	concordant	unreportable	unreportable	439139	0	2	SCV000697081;S	Likely benign;Likely benign	Laboratory Corpor	2018-01-25;2017-0	HBB	chr11	5247942	5247942	http://gnomad.b	0.0001003051142	SAS	0.0002274059952	0		0	0	https://www.ncbi.nlm.nih.gov/clinvar/variation/439139/
NM_004328.4(BCS1L):c.201C>T	concordant	unreportable	unreportable	290322	0	2	SCV000344855;S	Uncertain significance;Unc	EGL Genetic Diagn	2017-06-30;2016-1	BCS1L	chr2	219525911	219525911	http://gnomad.b	5.58E-05	NFE	0.0002596380073	0		0	0	https://www.ncbi.nlm.nih.gov/clinvar/variation/290322/
NM_138694.3(PKHD1):c.1717G>T	concordant	unreportable	unreportable	406883	0	1	SCV000545838	Uncertain significance	Invitae	3/14/2017	PKHD1	chr6	51920504	51920504	http://gnomad.b	0.0001056388929	FIN	0.0004039499909	0		0	0	https://www.ncbi.nlm.nih.gov/clinvar/variation/406883/
NM_138694.3(PKHD1):c.12198C>T	concordant	unreportable	unreportable	501329	0	1	SCV000707650	Uncertain significance	EGL Genetic Diagn	6/30/2017	PKHD1	chr6	51483906	51483906	http://gnomad.b	0.0001139788055	AFR	0.001045890036	0		0	0	https://www.ncbi.nlm.nih.gov/clinvar/variation/501329/
NM_001360.2(DHCR7):c.1091C>T	concordant	unreportable	unreportable	305954	0	2	SCV000594361;S	Uncertain significance;Unc	Genetic Services La	2017-07-05;2016-1	DHCR7	chr11	71146758	71146758	http://gnomad.b	0.0002261761158	SAS	0.002762249904	0		1	0	https://www.ncbi.nlm.nih.gov/clinvar/variation/305954/
NM_000396.3(CTSK):c.684C>T	concordant	unreportable	unreportable	292582	0	2	SCV000703541;S	Uncertain significance;Unc	EGL Genetic Diagn	2017-06-30;2016-1	CTSK	chr1	150772120	150772120	http://gnomad.b	0.0004895814676	NFE	0.000599831983	0		0	0	https://www.ncbi.nlm.nih.gov/clinvar/variation/292582/
NM_001042432.1(CLN3):c.771G>A	concordant	unreportable	unreportable	136790	0	1	SCV000628938	Benign	Invitae	10/5/2017	CLN3	chr16	28495346	28495346	http://gnomad.b	0.001038865803	AFR	0.01302439999	3		2	0	https://www.ncbi.nlm.nih.gov/clinvar/variation/136790/
NM_000492.3(CFTR):c.1920T>C	concordant	unreportable	unreportable	256250	0	2	SCV000602986;S	Benign;Uncertain significar	ARUP Laboratories	2017-06-30;2017-0	CFTR	chr7	117232141	117232141	http://gnomad.b	0.0002715423606	AMR	0.0002684640058	1		0	0	https://www.ncbi.nlm.nih.gov/clinvar/variation/256250/
NM_000391.3(TPP1):c.1328A>G	concordant	unreportable	unreportable	457938	0	1	SCV000628898	Uncertain significance	Invitae	10/5/2017	TPP1	chr11	6636499	6636499	http://gnomad.b	5.04E-05	EAS	0.0002898889943	0		0	0	https://www.ncbi.nlm.nih.gov/clinvar/variation/457938/
NM_004543.4(NEB):c.5696C>T	concordant	unreportable	unreportable	285430	0	2	SCV000338441;S	Uncertain significance;Likel	EGL Genetic Diagn	2017-06-30;2017-1	NEB	chr2	152520129	152520129	http://gnomad.b	0.0002013651525	AMR	0.002624829998	0		0	0	https://www.ncbi.nlm.nih.gov/clinvar/variation/285430/
NM_003060.3(SLC22A5):c.1125C>T	concordant	unreportable	unreportable	460390	0	1	SCV000632512	Benign	Invitae	10/5/2017	SLC22A5	chr5	131726454	131726454	http://gnomad.b	0.0006975745868	SAS	0.006010009907	2		5	0	https://www.ncbi.nlm.nih.gov/clinvar/variation/460390/
NM_000136.2(FANCC):c.1425A>G	concordant	unreportable	unreportable	234504	0	1	SCV000279395	Likely benign	GeneDx	11/28/2017	FANCC	chr9	97869456	97869456	http://gnomad.b	7.85E-05	AMR	0.001995240105	0		0	0	https://www.ncbi.nlm.nih.gov/clinvar/variation/234504/
NM_133647.1(SLC12A6):c.338G>A	concordant	unreportable	unreportable	246541	0	2	SCV000605122;S	Uncertain significance;Unc	ARUP Laboratories	2017-06-30;2017-1	SLC12A6	chr15	34553200	34553200	http://gnomad.b	0.0001007538761	NFE	7.18E-05	0		0	0	https://www.ncbi.nlm.nih.gov/clinvar/variation/246541/
NM_000152.3(GAA):c.2157G>A	concordant	unreportable	unreportable	194459	0	1	SCV000534757	Likely benign	GeneDx	11/28/2017	GAA	chr17	78087133	78087133	http://gnomad.b	6.11E-05	AMR	0.0005808330025	0		0	0	https://www.ncbi.nlm.nih.gov/clinvar/variation/194459/
NM_017890.4(VPS13B):c.8854_8856d	concordant	unreportable	unreportable	283889	0	1	SCV000336249	Uncertain significance	EGL Genetic Diagn	6/30/2017	VPS13B	chr8	100831793	100831796	http://gnomad.b	0.0001427811529	SAS	0.000910984003	1		0	0	https://www.ncbi.nlm.nih.gov/clinvar/variation/283889/
NM_001360.2(DHCR7):c.92G>A	concordant	unreportable	unreportable	501845	0	1	SCV000708354	Uncertain significance	EGL Genetic Diagn	6/30/2017	DHCR7	chr11	71155907	71155907	http://gnomad.b	5.92E-05	AFR	6.53E-05	0		0	0	https://www.ncbi.nlm.nih.gov/clinvar/variation/501845/
NM_018941.3(CLN8):c.779C>T	concordant	unreportable	unreportable	205196	0	2	SCV000240705;S	Uncertain significance;Likel	GeneDx;Invitae	2017-11-28;2017-1	CLN8	chr8	1728651	1728651	http://gnomad.b	0.0001734945153	AFR	0.002680090023	0		0	0	https://www.ncbi.nlm.nih.gov/clinvar/variation/205196/
NM_002485.4(NBN):c.1317A>G	concordant	unreportable	unreportable	127858	0	4	SCV000149690;S	Likely benign;Likely benign,	GeneDx;Invitae;La	2017-11-28;2017-1	NBN	chr8	90967591	90967591	http://gnomad.b	0.0003402119353	AFR	0.004314850084	0		1	0	https://www.ncbi.nlm.nih.gov/clinvar/variation/127858/
NM_000051.3(ATM):c.8596C>G	concordant	unreportable	unreportable	186482	0	1	SCV000687845	Uncertain significance	Color Genomics, In	12/21/2017	ATM	chr11	108218017	108218017		1.11E-05					0	0	https://www.ncbi.nlm.nih.gov/clinvar/variation/186482/
NM_000051.3(ATM):c.3919G>A	concordant	unreportable	unreportable	142243	0	1	SCV000282946	Likely benign	Invitae	10/5/2017	ATM	chr11	108155126	108155126	http://gnomad.b	0.0002617626092	SAS	0.002988759894	2		2	0	https://www.ncbi.nlm.nih.gov/clinvar/variation/142243/
NM_000051.3(ATM):c.2836A>G	concordant	unreportable	unreportable	141763	0	3	SCV000611353;S	Uncertain significance;Unc	Fulgent Genetics,F	2017-05-23;2017-1	ATM	chr11	108139334	108139334	http://gnomad.b	2.78E-05	AFR	6.53E-05	0		0	0	https://www.ncbi.nlm.nih.gov/clinvar/variation/141763/
NM_000051.3(ATM):c.1516G>T	concordant	unreportable	unreportable	127339	0	4	SCV000537575;S	Uncertain significance;Unc	Color Genomics, In	2017-01-26;2017-0	ATM	chr11	108121708	108121708	http://gnomad.b	0.0001030342185	NFE	8.07E-05	0		1	0	https://www.ncbi.nlm.nih.gov/clinvar/variation/127339/
NM_004004.5(GJB2):c.*3C>A	concordant	unreportable	unreportable	44716	0	3	SCV000599767;S	Likely benign;Likely benign,	Division of Genomi	2017-05-17;2017-0	GJB2	chr13	20763037	20763037	http://gnomad.b	0.0008633648317	FIN	0.008897280321	0		0	0	https://www.ncbi.nlm.nih.gov/clinvar/variation/44716/
NM_000071.2(CBS):c.1341C>T	concordant	unreportable	unreportable	471358	0	1	SCV000649827	Likely benign	Invitae	10/5/2017	CBS	chr21	44478961	44478961		4.78E-05					0	0	https://www.ncbi.nlm.nih.gov/clinvar/variation/471358/
NM_000391.3(TPP1):c.138G>T	concordant	unreportable	unreportable	207563	0	1	SCV000373336	Uncertain significance	Illumina Clinical Se	10/18/2016	TPP1	chr11	6640098	6640098	http://gnomad.b	2.80E-05	NFE	3.58E-05	0		0	0	https://www.ncbi.nlm.nih.gov/clinvar/variation/207563/
NM_000057.2(BLM):c.1881T>C	concordant	unreportable	unreportable	405301	0	1	SCV000543364	Uncertain significance	Invitae	10/5/2017	BLM	chr15	91304484	91304484	http://gnomad.b	2.85E-05	AFR	0.0002895189973	0		0	0	https://www.ncbi.nlm.nih.gov/clinvar/variation/405301/
NM_000035.3(ALDOB):c.156T>G	concordant	unreportable	unreportable	254731	0	2	SCV000706108;S	Benign;Likely benign	EGL Genetic Diagn	2017-06-30;2017-1	ALDOB	chr9	104192205	104192205	http://gnomad.b	0.0009167014543	AFR	0.009541239589	0		3	0	https://www.ncbi.nlm.nih.gov/clinvar/variation/254731/
NM_000152.3(GAA):c.711G>A	concordant	unreportable	unreportable	456432	0	2	SCV000707954;S	Uncertain significance;Likel	EGL Genetic Diagn	2017-06-30;2017-1	GAA	chr17	78081374	78081374	http://gnomad.b	3.06E-05	AFR	0.0001308729989	0		0	0	https://www.ncbi.nlm.nih.gov/clinvar/variation/456432/
NM_018941.3(CLN8):c.725C>T	concordant	unreportable	unreportable	205195	0	1	SCV000560192	Likely benign	Invitae	3/14/2017	CLN8	chr8	1728597	1728597	http://gnomad.b	0.0004617192747	EAS	0.004928110167	0		0	0	https://www.ncbi.nlm.nih.gov/clinvar/variation/205195/
NM_000050.4(ASS1):c.786C>T	concordant	unreportable	unreportable	392113	0	1	SCV000535327	Likely benign	GeneDx	11/28/2017	ASS1	chr9	133355784	133355784	http://gnomad.b	0.0002228077114	EAS	0.001043720054	0		0	0	https://www.ncbi.nlm.nih.gov/clinvar/variation/392113/
NM_000057.2(BLM):c.4186T>C	concordant	unreportable	unreportable	236820	0	1	SCV000283148	Likely benign	Invitae	10/5/2017	BLM	chr15	91358441	91358441	http://gnomad.b	0.0001212957237	EAS	0.0009276440251	0		0	0	https://www.ncbi.nlm.nih.gov/clinvar/variation/236820/
NM_000528.3(MAN2B1):c.1668C>A	concordant	unreportable	unreportable	328266	0	1	SCV000410789	Uncertain significance	Illumina Clinical Se	10/18/2016	MAN2B1	chr19	12766670	12766670	http://gnomad.b	0.0002394393176	EAS	0.000290091004	0		0	0	https://www.ncbi.nlm.nih.gov/clinvar/variation/328266/
NM_000642.2(AGL):c.1226T>G	concordant	unreportable	unreportable	456451	0	1	SCV000626668	Uncertain significance	Invitae	10/5/2017	AGL	chr1	100340954	100340954	http://gnomad.b	0.00015908723	NFE	0.0001702479931	0		0	0	https://www.ncbi.nlm.nih.gov/clinvar/variation/456451/
NM_020533.2(MCOLN1):c.1179G>A	concordant	unreportable	unreportable	284921	0	1	SCV000337737	Uncertain significance	EGL Genetic Diagn	6/30/2017	MCOLN1	chr19	7594031	7594031	http://gnomad.b	8.80E-05	NFE	0.0001343030017	0		0	0	https://www.ncbi.nlm.nih.gov/clinvar/variation/284921/
NM_017739.3(POMGNT1):c.839G>A	concordant	unreportable	unreportable	162588	0	2	SCV000338697;S	Uncertain significance;Unc	EGL Genetic Diagn	2017-06-30;2017-1	POMGNT1	chr1	46659986	46659986	http://gnomad.b	0.0003499459683	AFR	0.003136429936	0		0	0	https://www.ncbi.nlm.nih.gov/clinvar/variation/162588/
NM_000492.3(CFTR):c.1365G>T	concordant	unreportable	unreportable	193593	0	1	SCV000224842	Uncertain significance	EGL Genetic Diagn	6/30/2017	CFTR	chr7	117188850	117188850		1.93E-06					0	0	https://www.ncbi.nlm.nih.gov/clinvar/variation/193593/
NM_000518.4(HBB):c.316-96G>C	concordant	unreportable	unreportable	36321	0	1	SCV000601285	Uncertain significance	Quest Diagnostics I	8/1/2017	HBB	chr11	5247052	5247052		0.0003125787279					0	0	https://www.ncbi.nlm.nih.gov/clinvar/variation/36321/
NM_000227.3(LAMA3):c.3946G>A	concordant	unreportable	unreportable	326340	0	1	SCV000408016	Uncertain significance	Illumina Clinical Se	10/18/2016	LAMA3	chr18	21513810	21513810	http://gnomad.b	0.0001175871125	AFR	0.001045480021	0		0	0	https://www.ncbi.nlm.nih.gov/clinvar/variation/326340/

Supplemental Table 2

NM_002485.4(NBN):c.1343A>T	concordant	unreportable	unreportable	182720	0	2	SCV000211449;S(	Uncertain significance;Unc	GeneDx;Invitae	2017-07-13;2017-1	NBN	chr8	90967565	90967565	http://gnomad.b	2.79E-05	AFR	0.0005884660059	0		0	0	https://www.ncbi.nlm.nih.gov/clinvar/variation/182720/
NM_138694.3(PKHD1):c.3526G>A	concordant	unreportable	unreportable	288097	0	2	SCV000342102;S(	Uncertain significance;Unc	EGL Genetic Diagn	2017-06-30;2016-1	PKHD1	chr6	51892988	51892988	http://gnomad.b	0.0001223187181	SAS	0.0003248860012	0		0	0	https://www.ncbi.nlm.nih.gov/clinvar/variation/288097/
NM_133647.1(SLC12A6):c.1551G>A	concordant	unreportable	unreportable	382667	0	1	SCV000522669	Likely benign	GeneDx	11/28/2017	SLC12A6	chr15	34542872	34542872	http://gnomad.b	9.48E-05	NFE	0.0001165290014	0		1	0	https://www.ncbi.nlm.nih.gov/clinvar/variation/382667/
NM_000243.2(MEFV):c.1406T>C	concordant	unreportable	unreportable	319113	0	2	SCV000396770;S(	Uncertain significance;Unc	Illumina Clinical Se	2016-10-18;2018-0	MEFV	chr16	3297197	3297197	http://gnomad.b	7.28E-05	NFE	0.0001074129977	0		0	0	https://www.ncbi.nlm.nih.gov/clinvar/variation/319113/
NM_000543.4(SMPD1):c.373C>T	concordant	unreportable	unreportable	291102	0	1	SCV000345800	Uncertain significance	EGL Genetic Diagn	6/30/2017	SMPD1	chr11	6412668	6412668	http://gnomad.b	0.0001784015224	EAS	0.000115969	0		0	0	https://www.ncbi.nlm.nih.gov/clinvar/variation/291102/
NM_000243.2(MEFV):c.198C>T	concordant	unreportable	unreportable	457999	0	2	SCV000629032;S(	Likely benign;Uncertain sig	Invitae;Laboratory	2017-10-05;2018-0	MEFV	chr16	3306390	3306390	http://gnomad.b	5.04E-05	AFR	0.0001960530062	0		1	0	https://www.ncbi.nlm.nih.gov/clinvar/variation/457999/
NM_000051.3(ATM):c.4424A>G	concordant	unreportable	unreportable	127392	0	4	SCV000602575;S(	Likely benign;Uncertain sig	ARUP Laboratories	2017-06-30;2017-1	ATM	chr11	108160516	108160516	http://gnomad.b	0.0005792193905	FIN	0.0009439049754	0		0	0	https://www.ncbi.nlm.nih.gov/clinvar/variation/127392/
NM_000152.3(GAA):c.318C>T	concordant	unreportable	unreportable	195019	0	2	SCV000227029;S(	Uncertain significance;Beni	EGL Genetic Diagn	2017-06-30;2017-1	GAA	chr17	78078703	78078703	http://gnomad.b	8.34E-05	NFE	9.11E-05	2		0	0	https://www.ncbi.nlm.nih.gov/clinvar/variation/195019/
NM_000057.2(BLM):c.2333C>G	concordant	unreportable	unreportable	127484	0	2	SCV000593635;S(	Uncertain significance;Unc	Genetic Services La	2017-07-05;2017-1	BLM	chr15	91312388	91312388	http://gnomad.b	0.0003044284831	ASJ	0.002436550101	0		0	0	https://www.ncbi.nlm.nih.gov/clinvar/variation/127484/
NM_001042432.1(CLN3):c.8G>A	concordant	unreportable	unreportable	409279	0	1	SCV000549227	Uncertain significance	Invitae	3/14/2017	CLN3	chr16	28503073	28503073	http://gnomad.b	1.11E-05	SAS	3.25E-05	0		0	0	https://www.ncbi.nlm.nih.gov/clinvar/variation/409279/
NM_033056.3(PCDH15):c.593C>T	concordant	unreportable	unreportable	46509	0	1	SCV000363239	Uncertain significance	Illumina Clinical Se	10/18/2016	PCDH15	chr10	56106126	56106126	http://gnomad.b	0.0002623051998	NFE	0.0003497319994	0		0	0	https://www.ncbi.nlm.nih.gov/clinvar/variation/46509/
NM_000243.2(MEFV):c.2292G>T	concordant	unreportable	unreportable	378133	0	1	SCV000513594	Uncertain significance	GeneDx	11/28/2017	MEFV	chr16	3293195	3293195	http://gnomad.b	0.0002378953261	NFE	0.0003670550068	0		1	0	https://www.ncbi.nlm.nih.gov/clinvar/variation/378133/
NM_000051.3(ATM):c.4416G>A	concordant	unreportable	unreportable	183820	0	2	SCV000687544;S(	Likely benign;Likely benign	Color Genomics, In	2017-12-21;2017-1	ATM	chr11	108160508	108160508		1.39E-05					0	0	https://www.ncbi.nlm.nih.gov/clinvar/variation/183820/
NM_020533.2(MCOLN1):c.1053C>T	concordant	unreportable	unreportable	288243	0	1	SCV000342295	Uncertain significance	EGL Genetic Diagn	6/30/2017	MCOLN1	chr19	7593775	7593775	http://gnomad.b	4.52E-05	EAS	0.0005218600272	0		0	0	https://www.ncbi.nlm.nih.gov/clinvar/variation/288243/
NM_000243.2(MEFV):c.1459G>C	concordant	unreportable	unreportable	378132	0	2	SCV000513592;S(	Likely benign;Likely benign	GeneDx;Invitae	2017-11-28;2017-1	MEFV	chr16	3297144	3297144	http://gnomad.b	0.0006856982928	AFR	0.007318350021	2		1	0	https://www.ncbi.nlm.nih.gov/clinvar/variation/378132/
NM_001173990.2(TMEM216):c.140T>	concordant	unreportable	unreportable	289981	0	2	SCV000344452;S(	Uncertain significance;Unc	EGL Genetic Diagn	2017-06-30;2016-1	TMEM216	chr11	61161359	61161359	http://gnomad.b	0.0005424917598	AMR	0.001671439968	0		0	0	https://www.ncbi.nlm.nih.gov/clinvar/variation/289981/
NM_000051.3(ATM):c.5753G>C	concordant	unreportable	unreportable	142535	0	4	SCV000682285;S(	Uncertain significance;Unc	Color Genomics, In	2017-10-26;2017-1	ATM	chr11	108178702	108178702	http://gnomad.b	6.96E-05	NFE	0.0001076289991	0		0	0	https://www.ncbi.nlm.nih.gov/clinvar/variation/142535/
NM_005562.2(LAMC2):c.3246G>A	concordant	unreportable	unreportable	294017	0	1	SCV000351693	Uncertain significance	Illumina Clinical Se	10/18/2016	LAMC2	chr1	183209444	183209444	http://gnomad.b	4.76E-05	AFR	0.0006534240092	0		0	0	https://www.ncbi.nlm.nih.gov/clinvar/variation/294017/
NM_033056.3(PCDH15):c.1900G>A	concordant	unreportable	unreportable	300192	0	2	SCV000705648;S(	Uncertain significance;Unc	EGL Genetic Diagn	2017-06-30;2016-1	PCDH15	chr10	55892652	55892652	http://gnomad.b	0.0004886077251	SAS	0.003163109999	0		1	0	https://www.ncbi.nlm.nih.gov/clinvar/variation/300192/
NM_000232.4(SGCB):c.943G>A	concordant	unreportable	unreportable	255606	0	3	SCV000332137;S(	Uncertain significance;Unc	EGL Genetic Diagn	2017-06-30;2017-1	SGCB	chr4	52890137	52890137	http://gnomad.b	0.0002490890059	FIN	0.0009865469765	0		0	0	https://www.ncbi.nlm.nih.gov/clinvar/variation/255606/
NM_133647.1(SLC12A6):c.221C>A	concordant	unreportable	unreportable	440271	0	1	SCV000605123	Uncertain significance	ARUP Laboratories	6/30/2017	SLC12A6	chr15	34628661	34628661	http://gnomad.b	0.0001185339718	FIN	0.0003139300097	0		0	0	https://www.ncbi.nlm.nih.gov/clinvar/variation/440271/
NM_000528.3(MAN2B1):c.2886C>T	concordant	unreportable	unreportable	328252	0	1	SCV000410772	Uncertain significance	Illumina Clinical Se	10/18/2016	MAN2B1	chr19	12758084	12758084	http://gnomad.b	6.76E-05	NFE	7.16E-05	0		0	0	https://www.ncbi.nlm.nih.gov/clinvar/variation/328252/
NM_000271.4(NPC1):c.3528G>A	concordant	unreportable	unreportable	195724	0	1	SCV000228001	Uncertain significance	EGL Genetic Diagn	6/30/2017	NPC1	chr18	21114473	21114473	http://gnomad.b	6.58E-05	SAS	0.0001624849974	0		0	0	https://www.ncbi.nlm.nih.gov/clinvar/variation/195724/
NM_017890.4(VPS13B):c.3017C>G	concordant	unreportable	unreportable	437255	0	1	SCV000597916	Uncertain significance	Genetic Services La	7/5/2017	VPS13B	chr8	100403867	100403867	http://gnomad.b	0.0003079593494	ASJ	0.002234410029	0		0	0	https://www.ncbi.nlm.nih.gov/clinvar/variation/437255/
NM_000051.3(ATM):c.1898+3A>G	concordant	unreportable	unreportable	453392	0	2	SCV000682013;S(	Uncertain significance;Unc	Color Genomics, In	2017-10-26;2017-1	ATM	chr11	108123642	108123642	http://gnomad.b	1.95E-05	AFR	6.57E-05	0		0	0	https://www.ncbi.nlm.nih.gov/clinvar/variation/453392/
NM_033056.3(PCDH15):c.4320G>A	concordant	unreportable	unreportable	288914	0	1	SCV000343160	Uncertain significance	EGL Genetic Diagn	6/30/2017	PCDH15	chr10	55587200	55587200	http://gnomad.b	6.94E-05	AFR	0.001098150038	0		0	0	https://www.ncbi.nlm.nih.gov/clinvar/variation/288914/
NM_000391.3(TPP1):c.101G>A	concordant	unreportable	unreportable	207560	0	2	SCV000243398;S(	Uncertain significance;Unc	GeneDx;Illumina C	2017-11-28;2016-1	TPP1	chr11	6640135	6640135	http://gnomad.b	0.000257413221	NFE	0.0007532149903	0		0	0	https://www.ncbi.nlm.nih.gov/clinvar/variation/207560/
NM_000057.2(BLM):c.780T>C	concordant	unreportable	unreportable	413285	0	1	SCV000555817	Likely benign	Invitae	10/5/2017	BLM	chr15	91293278	91293278	http://gnomad.b	7.85E-05	AFR	0.001315440051	0		0	0	https://www.ncbi.nlm.nih.gov/clinvar/variation/413285/
NM_000152.3(GAA):c.2652G>A	concordant	unreportable	unreportable	289488	0	2	SCV000343860;S(	Uncertain significance;Likel	EGL Genetic Diagn	2017-06-30;2017-1	GAA	chr17	78092457	78092457	http://gnomad.b	0.0002417996565	FIN	0.001384380041	0		0	0	https://www.ncbi.nlm.nih.gov/clinvar/variation/289488/
NM_000051.3(ATM):c.7521C>T	concordant	unreportable	unreportable	183877	0	2	SCV000682422;S(	Likely benign;Likely benign	Color Genomics, In	2017-10-26;2017-1	ATM	chr11	108202176	108202176	http://gnomad.b	5.85E-05	EAS	0.0006404289743	0		0	0	https://www.ncbi.nlm.nih.gov/clinvar/variation/183877/
NM_000288.3(PEX7):c.94C>T	concordant	unreportable	unreportable	355526	0	2	SCV000707915;S(	Uncertain significance;Unc	EGL Genetic Diagn	2017-06-30;2016-1	PEX7	chr6	137143897	137143897	http://gnomad.b	0.000736135649	EAS	0.005211189855	0		0	0	https://www.ncbi.nlm.nih.gov/clinvar/variation/355526/
NM_000159.2(GCDH):c.285G>A	concordant	unreportable	unreportable	387991	0	1	SCV000530193	Likely benign	GeneDx	11/28/2017	GCDH	chr19	13002943	13002943	http://gnomad.b	0.0001461889661	EAS	0.0006957330042	0		0	0	https://www.ncbi.nlm.nih.gov/clinvar/variation/387991/
NM_014363.4(SACS):c.9508C>T	concordant	reportable	reportable	280094	1	0	SCV000329910	Pathogenic	GeneDx	11/28/2017	SACS	chr13	23908507	23908507	http://gnomad.b	3.56E-05	NFE	8.98E-06	0		0	0	https://www.ncbi.nlm.nih.gov/clinvar/variation/280094/
NM_000071.2(CBS):c.1425G>A	concordant	unreportable	unreportable	413353	0	1	SCV000555947	Likely benign	Invitae	3/14/2017	CBS	chr21	44478297	44478297	http://gnomad.b	0.0001968404299	SAS	0.001201999956	2		0	0	https://www.ncbi.nlm.nih.gov/clinvar/variation/413353/
NM_133647.1(SLC12A6):c.2385C>T	concordant	unreportable	unreportable	315609	0	1	SCV000390304	Uncertain significance	Illumina Clinical Se	10/18/2016	SLC12A6	chr15	34532913	34532913	http://gnomad.b	0.0004267222986	SAS	0.001364429947	0		2	0	https://www.ncbi.nlm.nih.gov/clinvar/variation/315609/
NM_000071.2(CBS):c.924C>T	concordant	unreportable	unreportable	340084	0	2	SCV000526472;S(	Likely benign;Uncertain sig	GeneDx;Illumina C	2017-11-28;2016-1	CBS	chr21	44483093	44483093	http://gnomad.b	0.0001912164176	ASJ	0.001218270045	0		0	0	https://www.ncbi.nlm.nih.gov/clinvar/variation/340084/
NM_000051.3(ATM):c.4861C>T	concordant	unreportable	unreportable	490586	0	1	SCV000687577	Likely benign	Color Genomics, In	12/21/2017	ATM	chr11	108165738	108165738	http://gnomad.b	2.78E-06	ASJ	0.000101605001	0		0	0	https://www.ncbi.nlm.nih.gov/clinvar/variation/490586/
NM_000018.3(ACADVL):c.759G>A	concordant	unreportable	unreportable	385582	0	1	SCV000526898	Likely benign	GeneDx	11/28/2017	ACADVL	chr17	7125502	7125502	http://gnomad.b	6.80E-05	AFR	0.0007841089973	0		0	0	https://www.ncbi.nlm.nih.gov/clinvar/variation/385582/
NM_138694.3(PKHD1):c.847T>C	concordant	unreportable	unreportable	497776	0	1	SCV000702484	Uncertain significance	EGL Genetic Diagn	6/30/2017	PKHD1	chr6	51930807	51930807	http://gnomad.b	7.23E-05	EAS	0.0003482699976	0		0	0	https://www.ncbi.nlm.nih.gov/clinvar/variation/497776/
NM_000414.3(HSD17B4):c.1628G>A	concordant	unreportable	unreportable	350468	0	1	SCV000452138	Uncertain significance	Illumina Clinical Se	10/18/2016	HSD17B4	chr5	118861666	118861666	http://gnomad.b	5.07E-05	AMR	0.0001192179989	0		0	0	https://www.ncbi.nlm.nih.gov/clinvar/variation/350468/
NM_138694.3(PKHD1):c.2726G>A	concordant	unreportable	unreportable	215967	0	1	SCV000464101	Uncertain significance	Illumina Clinical Se	10/18/2016	PKHD1	chr6	51908518	51908518	http://gnomad.b	0.0004837149307	SAS	0.006206939928	2		3	0	https://www.ncbi.nlm.nih.gov/clinvar/variation/215967/
NM_000136.2(FANCC):c.1494T>C	concordant	unreportable	unreportable	220345	0	1	SCV000260816	Benign	Invitae	10/5/2017	FANCC	chr9	97869387	97869387	http://gnomad.b	0.0001712239181	EAS	0.00179730996	1		0	0	https://www.ncbi.nlm.nih.gov/clinvar/variation/220345/
NM_000051.3(ATM):c.2338A>T	concordant	unreportable	unreportable	141031	0	2	SCV000687374;S(	Uncertain significance;Unc	Color Genomics, In	2017-12-21;2017-1	ATM	chr11	108128295	108128295	http://gnomad.b	5.57E-06	AFR	6.53E-05	0		0	0	https://www.ncbi.nlm.nih.gov/clinvar/variation/141031/
NM_005562.2(LAMC2):c.2634G>A	concordant	unreportable	unreportable	294010	0	1	SCV000351683	Uncertain significance	Illumina Clinical Se	10/18/2016	LAMC2	chr1	183206519	183206519	http://gnomad.b	0.0003247644058	AFR	0.002091499977	1		0	0	https://www.ncbi.nlm.nih.gov/clinvar/variation/294010/
NM_000050.4(ASS1):c.333C>T	concordant	unreportable	unreportable	365253	0	1	SCV000477620	Uncertain significance	Illumina Clinical Se	10/18/2016	ASS1	chr9	133333946	133333946	http://gnomad.b	0.0003369966635	NFE	0.000442790013	0		0	0	https://www.ncbi.nlm.nih.gov/clinvar/variation/365253/
NM_138694.3(PKHD1):c.2217G>A	concordant	unreportable	unreportable	497775	0	1	SCV000702483	Uncertain significance	EGL Genetic Diagn	6/30/2017	PKHD1	chr6	51915017	51915017	http://gnomad.b	8.90E-05	EAS	0.0004061269865	0		0	0	https://www.ncbi.nlm.nih.gov/clinvar/variation/497775/
NM_138694.3(PKHD1):c.9714A>G	concordant	unreportable	unreportable	357414	0	1	SCV000464038	Uncertain significance	Illumina Clinical Se	10/18/2016	PKHD1	chr6	51612700	51612700	http://gnomad.b	0.0001000789512	NFE	0.0001524340041	0		0	0	https://www.ncbi.nlm.nih.gov/clinvar/variation/357414/
NM_005476.5(GNE):c.624T>G	concordant	unreportable	unreportable	287949	0	2	SCV000341909;S(	Benign;Likely benign	EGL Genetic Diagn	2017-06-30;2017-1	GNE	chr9	36236974	36236974	http://gnomad.b	0.001081414243	AFR	0.0122221997	2		3	0	https://www.ncbi.nlm.nih.gov/clinvar/variation/287949/
NM_001173990.2(TMEM216):c.344G>	concordant	unreportable	unreportable	305079	0	1	SCV000372635	Uncertain significance	Illumina Clinical Se	10/18/2016	TMEM216	chr11	61165360	61165360	http://gnomad.b	6.17E-05	AMR	0.0005062239943	0		0	0	https://www.ncbi.nlm.nih.gov/clinvar/variation/305079/
NM_000071.2(CBS):c.548C>T	concordant	unreportable	unreportable	263808	0	1	SCV000649841	Uncertain significance	Invitae	10/5/2017	CBS	chr21	44485615	44485615	http://gnomad.b	1.69E-05	AFR	6.59E-05	0		0	0	https://www.ncbi.nlm.nih.gov/clinvar/variation/263808/
NM_033056.3(PCDH15):c.2563C>T	concordant	unreportable	unreportable	46453	0	1	SCV000363190	Uncertain significance	Illumina Clinical Se	10/18/2016	PCDH15	chr10	55780140	55780140	http://gnomad.b	0.0004268888546	AFR	0.003333329922	0		0	0	https://www.ncbi.nlm.nih.gov/clinvar/variation/46453/
NM_000018.3(ACADVL):c.68G>A	concordant	unreportable	unreportable	21023	0	3	SCV000602377;S(	Likely benign;Likely benign	ARUP Laboratories	2017-06-30;2017-1	ACADVL	chr17	7123446	7123446	http://gnomad.b	0.0005068811236	AMR	0.01220550016	4		2	0	https://www.ncbi.nlm.nih.gov/clinvar/variation/21023/
NM_138694.3(PKHD1):c.3834C>T	concordant	unreportable	unreportable	502595	0	1	SCV000709398	Uncertain significance	EGL Genetic Diagn	6/30/2017	PKHD1	chr6	51890774	51890774	http://gnomad.b	0.0002918969409	AFR	0.002237430075	0		0	0	https://www.ncbi.nlm.nih.gov/clinvar/variation/502595/
NM_138694.3(PKHD1):c.1307C>G	concordant	unreportable	unreportable	502594	0	1	SCV000709397	Uncertain significance	EGL Genetic Diagn	6/30/2017	PKHD1	chr6	51923326	51923326	http://gnomad.b	0.0002863369992	AFR	0.002614039928	0		1	0	https://www.ncbi.nlm.nih.gov/clinvar/variation/502594/
NM_000642.2(AGL):c.3792A>T	concordant	unreportable	unreportable	381841	0	2	SCV000521515;S(	Likely benign;Benign	GeneDx;Invitae	2017-11-28;2017-1	AGL	chr1	100376359	100376359	http://gnomad.b	0.0004046955852	AFR	0.004319940228	0		0	0	https://www.ncbi.nlm.nih.gov/clinvar/variation/381841/
NM_001042432.1(CLN3):c.49G>A	concordant	unreportable	unreportable	205097	0	2	SCV000240591;S(	Uncertain significance;Unc	GeneDx;Invitae	2017-11-28;2017-1	CLN3	chr16	28502879	28502879	http://gnomad.b	9.17E-05	AMR	0.0002085690066	0		0	0	https://www.ncbi.nlm.nih.gov/clinvar/variation/205097/
NM_000159.2(GCDH):c.1299G>A	concordant	unreportable	unreportable	255395	0	1	SCV000534738	Likely benign	GeneDx	11/28/2017	GCDH	chr19	13010337	13010337	http://gnomad.b	7.31E-05	SAS	0.0006822170108	0		0	0	https://www.ncbi.nlm.nih.gov/clinvar/variation/255395/
NM_000441.1(SLC26A4):c.2291C>T	concordant	unreportable	unreportable	43546	0	1	SCV000466110	Uncertain significance	Illumina Clinical Se	10/18/2016	SLC26A4	chr7	107353039	107353039	http://gnomad.b	0.0001116825535	AFR	0.0001960270019	0		0	0	https://www.ncbi.nlm.nih.gov/clinvar/variation/43546/
NM_000018.3(ACADVL):c.1606-22C>T	concordant	unreportable	unreportable	439359	0	1	SCV000602368	Likely benign	ARUP Laboratories	6/30/2017	ACADVL	chr17	7127777	7127777	http://gnomad.b	0.0003766211701	AFR	0.004859469831	0		0	0	https://www.ncbi.nlm.nih.gov/clinvar/variation/439359/
NM_000151.3(G6PC):c.1068G>A	concordant	unreportable	unreportable	378938	0	1	SCV000515406	Likely benign	GeneDx	11/28/2017	G6PC	chr17	41063437	41063437	http://gnomad.b	7.41E-05	SAS	0.001007150044	1		0	0	https://www.ncbi.nlm.nih.gov/clinvar/variation/378938/
NM_002485.4(NBN):c.441C>T	concordant	unreportable	unreportable	215824	0	4	SCV000685784;S(	Likely benign;Likely benign	Color Genomics, In	2017-10-26;2017-1	NBN	chr8	90993001	90993001	http://gnomad.b	0.0001701059677	AFR	0.001830299967	0		0	0	https://www.ncbi.nlm.nih.gov/clinvar/variation/215824/
NM_000071.2(CBS):c.296T>A	concordant	unreportable	unreportable	405376	0	3	SCV000602919;S(	Uncertain significance;Unc	ARUP Laboratories	2017-06-30;2017-1	CBS	chr21	44488639	44488639	http://gnomad.b	0.0001209162641	AFR	0.001372550032	0		0	0	https://www.ncbi.nlm.nih.gov/clinvar/variation/405376/
NM_000057.2(BLM):c.3613G>A	concordant	unreportable	unreportable	133701	0	1	SCV000283138	Likely benign	Invitae	10/5/2017	BLM	chr15	91347451	91347451	http://gnomad.b	0.000589830186	AFR	0.00849894993	0		2	0	https://www.ncbi.nlm.nih.gov/clinvar/variation/133701/
NM_000050.4(ASS1):c.1134C>T	concordant	unreportable	unreportable	194454	0	1	SCV000526091	Likely benign	GeneDx	11/28/2017	ASS1	chr9	133374898	133374898	http://gnomad.b	0.0003175009887	AFR	0.003501590109	1		0	0	https://www.ncbi.nlm.nih.gov/clinvar/variation/194454/
NM_002485.4(NBN):c.808_809delGT	concordant	reportable	reportable	185833	3	0	SCV000685829;S(	Pathogenic;Pathogenic;Pat	Color Genomics, In	2017-10-26;2017-1	NBN	chr8	90982678	90982680	http://gnomad.b	1.12E-05	NFE	8.96E-06	0		0	0	https://www.ncbi.nlm.nih.gov/clinvar/variation/185833/
NM_003060.3(SLC22A5):c.272A>G	concordant	unreportable	unreportable	460405	0	1	SCV000632543	Uncertain significance	Invitae	10/5/2017	SLC22A5	chr5	131705936	131705936	http://gnomad.b	0.0001129675444	AFR	0.0003445899929	0		0	0	https://www.ncbi.nlm.nih.gov/clinvar/variation/460405/
NM_138694.3(PKHD1):c.9696T>C	concordant	unreportable	unreportable	497783	0	1	SCV000702491	Uncertain significance	EGL Genetic Diagn	6/30/2017	PKHD1	chr6	51612718	51612718		1.39E-05					0	0	https://www.ncbi.nlm.nih.gov/clinvar/variation/497783/
NM_000051.3(ATM):c.2275A>G	concordant	unreportable	unreportable	127346	0	6	SCV000682046;S(	Uncertain significance;Unc	Color Genomics, In	2017-10-26;2017-0	ATM	chr11	108128232	108128232	http://gnomad.b	6.96E-05	NFE	6.27E-05	0		0	0	https://www.ncbi.nlm.nih.gov/clinvar/variation/127346/
NM_000051.3(ATM):c.8199G>A	concordant	unreportable	unreportable	185555	0	1	SCV000558313	Likely benign	Invitae	3/14/2017	ATM	chr11	108206619	108206619	http://gnomad.b	5.57E-06	SAS	6.50E-05	0		0	0	https://www.ncbi.nlm.nih.gov/clinvar/variation/185555/
NM_000023.2(SGCA):c.724G>A	concordant	unreportable	unreportable	497383	0	1	SCV000701861	Uncertain significance	EGL Genetic Diagn	6/30/2017	SGCA	chr17	48246592	48246592	http://gnomad.b	1.68E-05	NFE	3.59E-05	0		0	0	https://www.ncbi.nlm.nih.gov/clinvar/variation/497383/
NM_002485.4(NBN):c.64G>A	concordant	unreportable	unreportable	239202	0	1	SCV000287477	Uncertain significance	Invitae	3/14/2017	NBN	chr8	90995057	90995057	http://gnomad.b	8.37E-06	NFE	1.79E-05	0		0	0	https://www.ncbi.nlm.nih.gov/clinvar/variation/239202/
NM_000228.2(LAMB3):c.1413G>A	concordant	unreportable	unreportable	295109	0	1	SCV000353634	Uncertain significance	Illumina Clinical Se	10/18/2016	LAMB3	chr1	209800800	209800800	http://gnomad.b	0.0003359443676	AFR	0.00373281003	0		1	0	https://www.ncbi.nlm.nih.gov/clinvar/variation/295109/
NM_138694.3(PKHD1):c.4169G>A	concordant	unreportable	unreportable	289823	0	2	SCV000344243;S(	Uncertain significance;Unc	EGL Genetic Diagn	2017-06-30;2016-1	PKHD1	chr6	51890439	51890439	http://gnomad.b	6.67E-05	AFR	0.000326754991	0		0	0	https://www.ncbi.nlm.nih.gov/clinvar/variation/289823/
NM_000071.2(CBS):c.1575T>C	concordant	unreportable	unreportable	413352	0	1	SCV000555945	Likely benign	Invitae	3/14/2017	CBS	chr21	44474071	44474071	http://gnomad.b	8.15E-05	FIN	0.0004036960017	1		0	0	https://www.ncbi.nlm.nih.gov/clinvar/variation/413352/
NM_000441.1(SLC26A4):c.1924T>C	concordant	unreportable	unreportable	43527	0	1	SCV000345424	Likely benign	EGL Genetic Diagn	6/30/2017	SLC26A4	chr7	107342392	107342392	http://gnomad.b	0.0003015428945	AMR	0.005065860227	0		1	0	https://www.ncbi.nlm.nih.gov/clinvar/variation/43527/
NM_017890.4(VPS13B):c.11834G>A	concordant	unreportable	unreportable	290042	0	2	SCV000344525;S(	Uncertain significance;Unc	EGL Genetic Diagn	2017-06-30;2016-1	VPS13B	chr8	100887659	100887659	http://gnomad.b	5.88E-05	NFE	0.0001531260059	0		0	0	https://www.ncbi.nlm.nih.gov/clinvar/variation/290042/
NM_000136.2(FANCC):c.839C>T	concordant	unreportable	unreportable	418188	0	1	SCV000564976	Uncertain significance	GeneDx	11/28/2017	FANCC	chr9	97897632	97897632	http://gnomad.b	2.14E-05	EAS	0.0004638760001	0		0	0	https://www.ncbi.nlm.nih.gov/clinvar/variation/418188/
NM_033056.3(PCDH15):c.4316C>T	concordant	unreportable	unreportable	426841	0	1	SCV000577391	Uncertain significance	GeneDx	11/28/2017	PCDH15	chr10	55587204	55587204	http://gnomad.b	3.86E-05	NFE	1.84E-05	0		0	0	https://www.ncbi.nlm.nih.gov/clinvar/variation/426841/
NM_000136.2(FANCC):c.1366A>G	concordant	unreportable	unreportable	456157	0	1	SCV000626238	Uncertain significance	Invitae	10/5/2017	FANCC	chr9	97869515	97869515		1.66E-05					0	0	https://www.ncbi.nlm.nih.gov/clinvar/variation/456157/
NM_020533.2(MCOLN1):c.1719G>A	concordant	unreportable	unreportable	330506	0	2	SCV000709285;S(	Uncertain significance;Unc	EGL Genetic Diagn	2017-06-30;2016-1	MCOLN1	chr19	7598657	7598657	http://gnomad.b	0.0002069516732	EAS	0.001740140026	0		2	0	https://www.ncbi.nlm.nih.gov/clinvar/variation/330506/
NM_000071.2(CBS):c.1273G>A	concordant	unreportable	unreportable	212866	0	2	SCV000707986;S(	Uncertain significance;Unc	EGL Genetic Diagn	2017-06-30;2017-1	CBS	chr21	44479029	44479029	http://gnomad.b	0.0002165244729	AFR	0.002102039987	0		0	0	https://www.ncbi.nlm.nih.gov/clinvar/variation/212866/
NM_033056.3(PCDH15):c.330C>T	concordant	unreportable	unreportable	46463	0	1	SCV000703913	Uncertain significance	EGL Genetic Diagn	6/30/2017	PCDH15	chr10	56129024	56129024	http://gnomad.b	0.0001002931646	AFR	0.001570679946	0		0	0	https://www.ncbi.nlm.nih.gov/clinvar/variation/46463/
NM_000352.3(ABCC8):c.3000C>T	concordant	unreportable	unreportable	446767	0	1	SCV000612207	Benign	Athena Diagnostics	8/17/2017	ABCC8	chr11	17428597	17428597	http://gnomad.b	0.0003742572929	AMR	0.009812690318	6		1	0	https://www.ncbi.nlm.nih.gov/clinvar/variation/446767/
NM_000642.2(AGL):c.931G>A	concordant	unreportable	unreportable	500887	0	1	SCV000707030	Uncertain significance	EGL Genetic Diagn	6/30/2017	AGL	chr1	100336398	100336398		1.12E-05					0	0	https://www.ncbi.nlm.nih.gov/clinvar/variation/500887/
NM_020533.2(MCOLN1):c.984+13C>T	concordant	unreportable	unreportable	330497	0	1	SCV000415615	Uncertain significance	Illumina Clinical Se	10/18/2016	MCOLN1	chr19	7593602	7593602	http://gnomad.b	1.90E-05	NFE	6.28E-05	0		0	0	https://www.ncbi.nlm.nih.gov/clinvar/variation/330497/
NM_000051.3(ATM):c.3354A>G	concordant	unreportable	unreportable	185554	0	2	SCV000682122;S(	Likely benign;Likely benign	Color Genomics, In	2017-10-26;2017-1	ATM	chr11	108150287	108150287	http://gnomad.b	3.90E-05	NFE	4.49E-05	0		0	0	https://www.ncbi.nlm.nih.gov/clinvar/variation/185554/
NM_133647.1(SLC12A6):c.2565G>A	concordant	unreportable	unreportable	315608	0	1	SCV000390303	Uncertain significance	Illumina Clinical Se	10/18/2016	SLC12A6	chr15	34531233	34531233	http://gnomad.b	0.0003763453606	EAS	0.003246749984	1		0	0	https://www.ncbi.nlm.nih.gov/clinvar/variation/315608/
NM_138694.3(PKHD1):c.2134G>C	concordant	unreportable	unreportable	289824	0	1	SCV000344244	Uncertain significance	EGL Genetic Diagn	6/30/2017	PKHD1	chr6	51917880	51917880	http://gnomad.b	2.78E-05	AFR	0.0002614039986	0		0	0	https://www.ncbi.nlm.nih.gov/clinvar/variation/289824/
NM_033056.3(PCDH15):c.2768C>T	concordant	unreportable	unreportable	300186	0	1	SCV000363182	Uncertain significance	Illumina Clinical Se	10/18/2016	PCDH15	chr10	55755509	55755509	http://gnomad.b	0.0002443038626	EAS	0.002261389978	0		1	0	https://www.ncbi.nlm.nih.gov/clinvar/variation/300186/
NM_001079802.1(FKTN):c.41C>T	concordant	unreportable	unreportable	93520	0	1	SCV000111314	Uncertain significance	EGL Genetic Diagn	6/30/2017	FKTN	chr9	108337354	108337354	http://gnomad.b	4.25E-05	AFR	0.0003922590113	0		0	0	https://www.ncbi.nlm.nih.gov/clinvar/variation/93520/
NM_001876.3(CPT1A):c.192C>T	concordant	unreportable	unreportable	388653	0	1	SCV000531003	Likely benign	GeneDx	11/28/2017	CPT1A	chr11	68579994	68579994	http://gnomad.b	7.60E-05	NFE	0.0002243920026	0		0	0	https://www.ncbi.nlm.nih.gov/clinvar/variation/388653/
NM_000466.2(PEX1):c.1284C>T	concordant	unreportable	unreportable	500641	0	1	SCV000706674	Uncertain significance	EGL Genetic Diagn	6/30/2017	PEX1	chr7	92143237	92143237	http://gnomad.b	2.22E-05	AFR	6.55E-05	0		0	0	https://www.ncbi.nlm.nih.gov/clinvar/variation/500641/
NM_000228.2(LAMB3):c.2831A>G	concordant	unreportable	unreportable	295078	0	1	SCV000353597	Uncertain significance	Illumina Clinical Se	10/18/2016	LAMB3	chr1	209791875	209791875	http://gnomad.b	0.0001371772834	SAS	0.000454811001	0		0	0	https://www.ncbi.nlm.nih.gov/clinvar/variation/295078/
NM_017739.3(POMGNT1):c.266G>A	concordant	unreportable	unreportable	288596	0	1	SCV000342761	Uncertain significance	EGL Genetic Diagn	6/30/2017	POMGNT1	chr1	46662491	46662491	http://gnomad.b	3.64E-05	FIN	0.0005832739989	0		0	0	https://www.ncbi.nlm.nih.gov/clinvar/variation/288596/
NM_000035.3(ALDOB):c.309C>T	concordant	unreportable	unreportable	364315	0	1	SCV000476069	Uncertain significance	Illumina Clinical Se	10/18/2016	ALDOB	chr9	104192052	104192052	http://gnomad.b	1.41E-05	EAS	5.80E-05	0		0	0	https://www.ncbi.nlm.nih.gov/clinvar/variation/364315/
NM_018941.3(CLN8):c.619C>G	concordant	unreportable	unreportable	451285	0	2	SCV000619960;S(	Uncertain significance;Unc	GeneDx;Invitae	2017-11-28;2017-1	CLN8	chr8	1728491	1728491	http://gnomad.b	5.04E-05	AFR	0.0007187660085	0		0	0	https://www.ncbi.nlm.nih.gov/clinvar/variation/451285/
NM_000152.3(GAA):c.1725C>T	concordant	unreportable	unreportable	284233	0	1	SCV000336764	Uncertain significance	EGL Genetic Diagn	6/30/2017	GAA	chr17	78085870	78085870	http://gnomad.b	3.34E-05	AFR	0.0001307019993	0		0	0	https://www.ncbi.nlm.nih.gov/clinvar/variation/284233/

Supplemental Table 2

NM_017890.4(VPS13B):c.5738T>C	concordant	unreportable	unreportable	361068	0	2	SCV000597885;S(	Uncertain significance;Unc	Genetic Services La	2017-07-05;2016-1	VPS13B	chr8	100654481	100654481	http://gnomad.b	4.48E-05	AMR	0.0002384359977	0		0	0	https://www.ncbi.nlm.nih.gov/clinvar/variation/361068/
NM_000027.3(AGA):c.794G>A	concordant	unreportable	unreportable	348230	0	1	SCV000448730	Uncertain significance	Illumina Clinical Se	10/18/2016	AGA	chr4	178355548	178355548	http://gnomad.b	0.0001452419909	SAS	0.00126696995	0		0	0	https://www.ncbi.nlm.nih.gov/clinvar/variation/348230/
NM_138694.3(PKHD1):c.8335T>G	concordant	unreportable	unreportable	502746	0	1	SCV000709605	Uncertain significance	EGL Genetic Diagn	6/30/2017	PKHD1	chr6	51656139	51656139	http://gnomad.b	0.0003141367078	EAS	0.002385389991	0		2	0	https://www.ncbi.nlm.nih.gov/clinvar/variation/502746/
NM_138694.3(PKHD1):c.2981G>A	concordant	unreportable	unreportable	458593	0	1	SCV000629912	Uncertain significance	Invitae	10/5/2017	PKHD1	chr6	51907773	51907773	http://gnomad.b	5.28E-05	AMR	2.98E-05	0		0	0	https://www.ncbi.nlm.nih.gov/clinvar/variation/458593/
NM_000051.3(ATM):c.5693G>A	concordant	unreportable	unreportable	133624	0	2	SCV000209752;S(	Likely benign;Likely benign	GeneDx;Invitae	2017-11-28;2017-1	ATM	chr11	108178642	108178642	http://gnomad.b	0.0001002495099	SAS	0.002209800063	2		0	0	https://www.ncbi.nlm.nih.gov/clinvar/variation/133624/
NM_000051.3(ATM):c.5634G>A	concordant	unreportable	unreportable	378989	0	1	SCV000515508	Likely benign	GeneDx	11/28/2017	ATM	chr11	108175539	108175539	http://gnomad.b	5.85E-05	AMR	0.0003587869869	0		0	0	https://www.ncbi.nlm.nih.gov/clinvar/variation/378989/
NM_001042432.1(CLN3):c.392G>A	concordant	unreportable	unreportable	205109	0	2	SCV000240603;S(	Uncertain significance;Unc	GeneDx;Illumina C	2017-11-28;2016-1	CLN3	chr16	28498845	28498845	http://gnomad.b	0.0002249950001	NFE	0.0005016929936	0		0	0	https://www.ncbi.nlm.nih.gov/clinvar/variation/205109/
NM_017890.4(VPS13B):c.2471C>T	concordant	unreportable	unreportable	290694	0	2	SCV000345300;S(	Uncertain significance;Unc	EGL Genetic Diagn	2017-06-30;2017-0	VPS13B	chr8	100205241	100205241	http://gnomad.b	0.0001987737619	NFE	0.0002151850058	0		0	0	https://www.ncbi.nlm.nih.gov/clinvar/variation/290694/
NM_174878.2(CLRN1):c.226T>C	concordant	unreportable	unreportable	227257	0	1	SCV000345731	Uncertain significance	EGL Genetic Diagn	6/30/2017	CLRN1	chr3	150690270	150690270	http://gnomad.b	0.0002005894243	NFE	0.0003580829944	0		0	0	https://www.ncbi.nlm.nih.gov/clinvar/variation/227257/
NM_138694.3(PKHD1):c.10619A>G	concordant	unreportable	unreportable	290431	0	1	SCV000344990	Uncertain significance	EGL Genetic Diagn	6/30/2017	PKHD1	chr6	51524305	51524305	http://gnomad.b	9.45E-05	NFE	0.0001354209962	0		0	0	https://www.ncbi.nlm.nih.gov/clinvar/variation/290431/
NM_000352.3(ABCC8):c.4198+18C>T	concordant	unreportable	unreportable	446777	0	1	SCV000612219	Likely benign	Athena Diagnostics	8/17/2017	ABCC8	chr11	17417381	17417381	http://gnomad.b	0.0002271354605	AFR	0.003226479981	0		0	0	https://www.ncbi.nlm.nih.gov/clinvar/variation/446777/
NM_000383.3(AIRE):c.1344C>T	concordant	unreportable	unreportable	458614	0	1	SCV000629946	Likely benign	Invitae	10/5/2017	AIRE	chr21	45713737	45713737	http://gnomad.b	0.0001531803016	NFE	0.0003666759876	0		0	0	https://www.ncbi.nlm.nih.gov/clinvar/variation/458614/
NM_000391.3(TPP1):c.-3A>G	concordant	unreportable	unreportable	378752	0	1	SCV000514954	Likely benign	GeneDx	11/28/2017	TPP1	chr11	6640634	6640634	http://gnomad.b	3.64E-05	FIN	4.48E-05	0		0	0	https://www.ncbi.nlm.nih.gov/clinvar/variation/378752/
NM_000152.3(GAA):c.258C>A	concordant	unreportable	unreportable	282138	0	4	SCV000333379;S(	Benign;Likely benign;Benig	EGL Genetic Diagn	2017-06-30;2017-1	GAA	chr17	78078643	78078643	http://gnomad.b	0.0003863235891	AFR	0.005326230079	0		1	0	https://www.ncbi.nlm.nih.gov/clinvar/variation/282138/
NM_000098.2(CPT2):c.1048C>T	concordant	unreportable	unreportable	203662	0	2	SCV000706183;S(	Uncertain significance;Unc	EGL Genetic Diagn	2017-06-30;2017-1	CPT2	chr1	53676394	53676394	http://gnomad.b	0.0002658190304	AFR	0.0007192359772	0		0	0	https://www.ncbi.nlm.nih.gov/clinvar/variation/203662/
NM_138694.3(PKHD1):c.10384A>G	concordant	unreportable	unreportable	496513	0	1	SCV000699841	Uncertain significance	Laboratory Corpora	1/25/2018	PKHD1	chr6	51524540	51524540	http://gnomad.b	5.00E-05	AFR	0.0001961300004	0		0	0	https://www.ncbi.nlm.nih.gov/clinvar/variation/496513/
NM_000152.3(GAA):c.277G>A	concordant	unreportable	unreportable	456417	0	1	SCV000626600	Uncertain significance	Invitae	10/5/2017	GAA	chr17	78078662	78078662	http://gnomad.b	6.39E-05	AMR	0.0002385779953	0		0	0	https://www.ncbi.nlm.nih.gov/clinvar/variation/456417/
NM_138694.3(PKHD1):c.6782A>G	concordant	unreportable	unreportable	496884	0	1	SCV000700491	Uncertain significance	EGL Genetic Diagn	6/30/2017	PKHD1	chr6	51771039	51771039	http://gnomad.b	0.0001278786598	AFR	0.00189665996	0		0	0	https://www.ncbi.nlm.nih.gov/clinvar/variation/496884/
NM_000243.2(MEFV):c.1803T>A	concordant	unreportable	unreportable	319108	0	2	SCV000604186;S(	Likely benign;Uncertain sig	ARUP Laboratories	2017-06-30;2016-1	MEFV	chr16	3293684	3293684	http://gnomad.b	3.36E-05	NFE	4.54E-05	0		0	0	https://www.ncbi.nlm.nih.gov/clinvar/variation/319108/
NM_003060.3(SLC22A5):c.414C>T	concordant	unreportable	unreportable	25377	0	2	SCV000452727;S(	Uncertain significance;Likel	Illumina Clinical Se	2016-10-18;2017-1	SLC22A5	chr5	131714090	131714090	http://gnomad.b	0.0005648377221	NFE	0.0009040459991	0		0	0	https://www.ncbi.nlm.nih.gov/clinvar/variation/25377/
NM_000108.3(DLD):c.860G>A	concordant	unreportable	unreportable	358569	0	1	SCV000466240	Uncertain significance	Illumina Clinical Se	10/18/2016	DLD	chr7	107556126	107556126	http://gnomad.b	0.0001194259248	NFE	0.0001435519953	0		0	0	https://www.ncbi.nlm.nih.gov/clinvar/variation/358569/
NM_000153.3(GALC):c.659G>A	concordant	unreportable	unreportable	211059	0	2	SCV000247447;S(	Uncertain significance;Unc	Genetic Services La	2015-09-15;2016-1	GALC	chr14	88442795	88442795	http://gnomad.b	0.000296490227	AFR	0.002814139938	0		0	0	https://www.ncbi.nlm.nih.gov/clinvar/variation/211059/
NM_000182.4(HADHA):c.1794T>C	concordant	unreportable	unreportable	335377	0	1	SCV000429503	Uncertain significance	Illumina Clinical Se	10/18/2016	HADHA	chr2	26416537	26416537	http://gnomad.b	3.08E-05	AFR	6.53E-05	0		0	0	https://www.ncbi.nlm.nih.gov/clinvar/variation/335377/
NM_000642.2(AGL):c.2575G>A	concordant	unreportable	unreportable	456474	0	1	SCV000626701	Uncertain significance	Invitae	10/5/2017	AGL	chr1	100350153	100350153	http://gnomad.b	3.63E-05	AFR	0.0002615750127	0		0	0	https://www.ncbi.nlm.nih.gov/clinvar/variation/456474/
NM_025136.3(OPA3):c.33G>T	concordant	unreportable	unreportable	284628	0	1	SCV000337321	Uncertain significance	EGL Genetic Diagn	6/30/2017	OPA3	chr19	46087990	46087990	http://gnomad.b	1.39E-05	AFR	0.0005231489777	0		0	0	https://www.ncbi.nlm.nih.gov/clinvar/variation/284628/
NM_033056.3(PCDH15):c.2990A>G	concordant	unreportable	unreportable	46459	0	1	SCV000337988	Likely benign	EGL Genetic Diagn	6/30/2017	PCDH15	chr10	55721531	55721531	http://gnomad.b	0.0003034511135	AFR	0.003736719955	0		1	0	https://www.ncbi.nlm.nih.gov/clinvar/variation/46459/
NM_000098.2(CPT2):c.932A>G	concordant	unreportable	unreportable	447221	0	2	SCV000613024;S(	Uncertain significance;Likel	Athena Diagnostics	2017-08-17;2017-1	CPT2	chr1	53676278	53676278	http://gnomad.b	0.0003553580722	AFR	0.004576960113	2		1	0	https://www.ncbi.nlm.nih.gov/clinvar/variation/447221/
NM_000071.2(CBS):c.1039+3G>A	concordant	unreportable	unreportable	385169	0	1	SCV000526346	Likely benign	GeneDx	11/28/2017	CBS	chr21	44482418	44482418	http://gnomad.b	7.59E-05	AMR	0.00032818201	0		0	0	https://www.ncbi.nlm.nih.gov/clinvar/variation/385169/
NM_017739.3(POMGNT1):c.120+3A>(	concordant	unreportable	unreportable	285722	0	1	SCV000338873	Uncertain significance	EGL Genetic Diagn	6/30/2017	POMGNT1	chr1	46663371	46663371	http://gnomad.b	4.76E-05	AFR	0.0004575159983	0		0	0	https://www.ncbi.nlm.nih.gov/clinvar/variation/285722/
NM_002485.4(NBN):c.804G>A	concordant	unreportable	unreportable	183781	0	2	SCV000685828;S(	Likely benign;Likely benign	Color Genomics, In	2017-10-26;2017-1	NBN	chr8	90982684	90982684	http://gnomad.b	4.46E-05	AFR	0.0004575159983	0		0	0	https://www.ncbi.nlm.nih.gov/clinvar/variation/183781/
NM_017739.3(POMGNT1):c.797G>A	concordant	unreportable	unreportable	501452	0	1	SCV000707815	Uncertain significance	EGL Genetic Diagn	6/30/2017	POMGNT1	chr1	46660028	46660028		2.80E-06					0	0	https://www.ncbi.nlm.nih.gov/clinvar/variation/501452/
NM_000057.2(BLM):c.759G>A	concordant	unreportable	unreportable	254781	0	1	SCV000623350	Likely benign	Invitae	10/5/2017	BLM	chr15	91293257	91293257	http://gnomad.b	0.0001236740713	AFR	0.000917791971	0		0	0	https://www.ncbi.nlm.nih.gov/clinvar/variation/254781/
NM_000352.3(ABCC8):c.4563G>T	concordant	unreportable	unreportable	434043	0	1	SCV000592977	Uncertain significance	Genetic Services La	7/5/2017	ABCC8	chr11	17415289	17415289	http://gnomad.b	0.000314892343	AFR	0.004769990221	0		1	0	https://www.ncbi.nlm.nih.gov/clinvar/variation/434043/
NM_000492.3(CFTR):c.1734A>G	concordant	unreportable	unreportable	416593	0	1	SCV000562304	Likely benign	Invitae	3/14/2017	CFTR	chr7	117230461	117230461	http://gnomad.b	3.27E-05	NFE	1.80E-05	0		0	0	https://www.ncbi.nlm.nih.gov/clinvar/variation/416593/
NM_000360.3(TH):c.5C>T	concordant	unreportable	unreportable	304087	0	1	SCV000369935	Uncertain significance	Illumina Clinical Se	10/18/2016	TH	chr11	2193012	2193012	http://gnomad.b	9.58E-05	SAS	0.002534609986	0		0	0	https://www.ncbi.nlm.nih.gov/clinvar/variation/304087/
NM_000642.2(AGL):c.2248G>T	concordant	unreportable	unreportable	291337	0	2	SCV000524779;S(	Uncertain significance;Unc	GeneDx;Illumina C	2017-11-28;2016-1	AGL	chr1	100347187	100347187	http://gnomad.b	0.0002651453834	NFE	0.000313630997	0		0	0	https://www.ncbi.nlm.nih.gov/clinvar/variation/291337/
NM_138694.3(PKHD1):c.6001G>A	concordant	unreportable	unreportable	197020	0	1	SCV000464076	Uncertain significance	Illumina Clinical Se	10/18/2016	PKHD1	chr6	51799028	51799028	http://gnomad.b	5.84E-05	AFR	0.0001960780064	0		0	0	https://www.ncbi.nlm.nih.gov/clinvar/variation/197020/
NM_000414.3(HSD17B4):c.1417C>T	concordant	unreportable	unreportable	252564	0	2	SCV000297067;S(	Uncertain significance;Unc	Division of Genomi	2016-01-06;2016-1	HSD17B4	chr5	118844919	118844919	http://gnomad.b	0.0002225740834	NFE	0.0002238779998	0		0	0	https://www.ncbi.nlm.nih.gov/clinvar/variation/252564/
NM_017739.3(POMGNT1):c.251G>A	concordant	unreportable	unreportable	451139	0	2	SCV000619798;S(	Uncertain significance;Unc	GeneDx;Invitae	2017-11-28;2017-1	POMGNT1	chr1	46662506	46662506	http://gnomad.b	7.84E-05	AFR	0.0005230130046	0		0	0	https://www.ncbi.nlm.nih.gov/clinvar/variation/451139/
NM_000543.4(SMPD1):c.1340+12A>T	concordant	unreportable	unreportable	305204	0	1	SCV000372940	Uncertain significance	Illumina Clinical Se	10/18/2016	SMPD1	chr11	6414935	6414935	http://gnomad.b	0.0003092293054	AFR	0.002091499977	0		0	0	https://www.ncbi.nlm.nih.gov/clinvar/variation/305204/
NM_000152.3(GAA):c.74C>T	concordant	unreportable	unreportable	497169	0	1	SCV000701493	Uncertain significance	EGL Genetic Diagn	6/30/2017	GAA	chr17	78078459	78078459	http://gnomad.b	2.22E-05	EAS	0.0001160359971	0		0	0	https://www.ncbi.nlm.nih.gov/clinvar/variation/497169/
NM_000243.2(MEFV):c.744T>C	concordant	unreportable	unreportable	282002	0	1	SCV000333134	Uncertain significance	EGL Genetic Diagn	6/30/2017	MEFV	chr16	3304324	3304324	http://gnomad.b	3.36E-05	SAS	0.0003573519934	0		0	0	https://www.ncbi.nlm.nih.gov/clinvar/variation/282002/
NM_000152.3(GAA):c.1194+3G>C	concordant	unreportable	unreportable	198393	0	1	SCV000232012	Uncertain significance	EGL Genetic Diagn	6/30/2017	GAA	chr17	78082409	78082409	http://gnomad.b	8.34E-05	NFE	0.000316930993	0		0	0	https://www.ncbi.nlm.nih.gov/clinvar/variation/198393/
NM_000137.2(FAH):c.870C>T	concordant	unreportable	unreportable	385165	0	1	SCV000526337	Likely benign	GeneDx	11/28/2017	FAH	chr15	80467390	80467390	http://gnomad.b	0.0001248397889	AMR	0.001995119965	1		0	0	https://www.ncbi.nlm.nih.gov/clinvar/variation/385165/
NM_004646.3(NPHS1):c.733A>C	concordant	unreportable	unreportable	328875	0	1	SCV000411581	Uncertain significance	Illumina Clinical Se	10/18/2016	NPHS1	chr19	36340245	36340245	http://gnomad.b	0.0002620296707	ASJ	0.002767530037	0		0	0	https://www.ncbi.nlm.nih.gov/clinvar/variation/328875/
NM_138694.3(PKHD1):c.9868G>A	concordant	unreportable	unreportable	289330	0	1	SCV000343677	Uncertain significance	EGL Genetic Diagn	6/30/2017	PKHD1	chr6	51611649	51611649	http://gnomad.b	5.28E-05	ASJ	0.0001018949988	0		0	0	https://www.ncbi.nlm.nih.gov/clinvar/variation/289330/
NM_017739.3(POMGNT1):c.440G>A	concordant	unreportable	unreportable	436365	0	2	SCV000596516;S(	Uncertain significance;Unc	Genetic Services La	2017-07-05;2017-1	POMGNT1	chr1	46661577	46661577	http://gnomad.b	3.36E-05	AFR	6.55E-05	0		0	0	https://www.ncbi.nlm.nih.gov/clinvar/variation/436365/
NM_017890.4(VPS13B):c.7735G>C	concordant	unreportable	unreportable	448878	0	1	SCV000616275	Uncertain significance	Athena Diagnostics	8/17/2017	VPS13B	chr8	100791140	100791140	http://gnomad.b	0.0002491671099	AMR	0.005635059904	1		1	0	https://www.ncbi.nlm.nih.gov/clinvar/variation/448878/
NM_002485.4(NBN):c.1651dupA	concordant	reportable	reportable	234246	1	0	SCV000552998	Pathogenic	Invitae	10/5/2017	NBN	chr8	90965665	90965665	http://gnomad.b	5.58E-06	NFE	1.79E-05	0		0	0	https://www.ncbi.nlm.nih.gov/clinvar/variation/234246/
NM_000053.3(ATP7B):c.3275C>T	concordant	unreportable	unreportable	312382	0	1	SCV000384658	Uncertain significance	Illumina Clinical Se	10/18/2016	ATP7B	chr13	52516659	52516659	http://gnomad.b	0.0001515185526	AMR	0.0002382230014	0		0	0	https://www.ncbi.nlm.nih.gov/clinvar/variation/312382/
NM_000478.4(ALPL):c.534C>T	concordant	unreportable	unreportable	295544	0	1	SCV000354300	Likely benign	Illumina Clinical Se	10/18/2016	ALPL	chr1	21890595	21890595	http://gnomad.b	0.0005549796038	EAS	0.004580770154	1		0	0	https://www.ncbi.nlm.nih.gov/clinvar/variation/295544/
NM_033056.3(PCDH15):c.679C>T	concordant	unreportable	unreportable	179459	0	1	SCV000342171	Uncertain significance	EGL Genetic Diagn	6/30/2017	PCDH15	chr10	56089382	56089382	http://gnomad.b	6.69E-05	AMR	0.0004177360097	0		0	0	https://www.ncbi.nlm.nih.gov/clinvar/variation/179459/
NM_000159.2(GCDH):c.738G>A	concordant	unreportable	unreportable	391102	0	1	SCV000534087	Likely benign	GeneDx	11/28/2017	GCDH	chr19	13007121	13007121	http://gnomad.b	0.0006241144322	EAS	0.004580770154	0		1	0	https://www.ncbi.nlm.nih.gov/clinvar/variation/391102/
NM_000136.2(FANCC):c.767A>G	concordant	unreportable	unreportable	182476	0	2	SCV000211042;S(	Uncertain significance;Unc	GeneDx;Invitae	2017-11-28;2017-0	FANCC	chr9	97897704	97897704	http://gnomad.b	0.0001236617186	NFE	0.0001522120001	0		0	0	https://www.ncbi.nlm.nih.gov/clinvar/variation/182476/
NM_000017.2(ACADS):c.825C>T	concordant	unreportable	unreportable	307441	0	1	SCV000376685	Uncertain significance	Illumina Clinical Se	10/18/2016	ACADS	chr12	121176365	121176365	http://gnomad.b	0.0001161091539	AMR	0.000238335997	0		0	0	https://www.ncbi.nlm.nih.gov/clinvar/variation/307441/
NM_000051.3(ATM):c.8921C>T	concordant	unreportable	unreportable	135787	0	2	SCV000209668;S(	Uncertain significance;Likel	GeneDx;Invitae	2017-11-28;2017-1	ATM	chr11	108235879	108235879	http://gnomad.b	0.0001086036357	EAS	0.0004638760001	0		0	0	https://www.ncbi.nlm.nih.gov/clinvar/variation/135787/
NM_017890.4(VPS13B):c.4936C>T	concordant	unreportable	unreportable	361064	0	1	SCV000470809	Uncertain significance	Illumina Clinical Se	10/18/2016	VPS13B	chr8	100568793	100568793	http://gnomad.b	0.0002015733923	SAS	0.002697610063	1		0	0	https://www.ncbi.nlm.nih.gov/clinvar/variation/361064/
NM_003060.3(SLC22A5):c.688T>C	concordant	unreportable	unreportable	460412	0	1	SCV000632565	Uncertain significance	Invitae	10/5/2017	SLC22A5	chr5	131721055	131721055	http://gnomad.b	1.13E-05	NFE	1.79E-05	0		0	0	https://www.ncbi.nlm.nih.gov/clinvar/variation/460412/
NM_001079802.1(FKTN):c.1178T>C	concordant	unreportable	unreportable	459218	0	1	SCV000630814	Uncertain significance	Invitae	10/5/2017	FKTN	chr9	108397337	108397337	http://gnomad.b	3.55E-06	NFE	2.69E-05	0		0	0	https://www.ncbi.nlm.nih.gov/clinvar/variation/459218/
NM_000051.3(ATM):c.8265T>C	concordant	unreportable	unreportable	229903	0	2	SCV000682471;S(	Likely benign;Likely benign	Color Genomics, In	2017-10-26;2017-1	ATM	chr11	108206685	108206685	http://gnomad.b	6.40E-05	EAS	0.0005813949974	0		0	0	https://www.ncbi.nlm.nih.gov/clinvar/variation/229903/
NM_000152.3(GAA):c.1083G>A	concordant	unreportable	unreportable	501028	0	1	SCV000707239	Uncertain significance	EGL Genetic Diagn	6/30/2017	GAA	chr17	78082295	78082295	http://gnomad.b	4.17E-05	AMR	0.0001490050054	0		0	0	https://www.ncbi.nlm.nih.gov/clinvar/variation/501028/
NM_000391.3(TPP1):c.665A>G	concordant	unreportable	unreportable	287701	0	1	SCV000341567	Uncertain significance	EGL Genetic Diagn	6/30/2017	TPP1	chr11	6638228	6638228	http://gnomad.b	5.32E-05	EAS	0.0001159550011	0		0	0	https://www.ncbi.nlm.nih.gov/clinvar/variation/287701/
NM_017890.4(VPS13B):c.11233G>A	concordant	unreportable	unreportable	235216	0	1	SCV000597901	Uncertain significance	Genetic Services La	7/5/2017	VPS13B	chr8	100874117	100874117	http://gnomad.b	6.44E-05	AFR	0.003201890038	0		0	0	https://www.ncbi.nlm.nih.gov/clinvar/variation/235216/
NM_138694.3(PKHD1):c.7957C>T	concordant	unreportable	unreportable	500459	0	1	SCV000706420	Uncertain significance	EGL Genetic Diagn	6/30/2017	PKHD1	chr6	51712723	51712723	http://gnomad.b	0.0001139788055	SAS	0.001007340034	0		0	0	https://www.ncbi.nlm.nih.gov/clinvar/variation/500459/
NM_000277.1(PAH):c.71A>G	concordant	unreportable	unreportable	458080	0	1	SCV000629208	Uncertain significance	Invitae	10/5/2017	PAH	chr12	103306666	103306666	http://gnomad.b	9.59E-05	SAS	0.001754499972	0		1	0	https://www.ncbi.nlm.nih.gov/clinvar/variation/458080/
NM_000051.3(ATM):c.1738C>T	concordant	unreportable	unreportable	489513	0	1	SCV000681994	Uncertain significance	Color Genomics, In	10/26/2017	ATM	chr11	108122694	108122694		2.78E-06					0	0	https://www.ncbi.nlm.nih.gov/clinvar/variation/489513/
NM_000414.3(HSD17B4):c.1131C>T	concordant	unreportable	unreportable	350465	0	1	SCV000452124	Uncertain significance	Illumina Clinical Se	10/18/2016	HSD17B4	chr5	118835170	118835170	http://gnomad.b	0.0001944001488	SAS	0.0005523069995	0		0	0	https://www.ncbi.nlm.nih.gov/clinvar/variation/350465/
NM_002485.4(NBN):c.758C>T	concordant	unreportable	unreportable	127879	0	3	SCV000149714;S(	Uncertain significance;Likel	GeneDx;Invitae;Qu	2017-11-28;2017-1	NBN	chr8	90982730	90982730	http://gnomad.b	0.0002091466815	SAS	0.003216170007	1		0	0	https://www.ncbi.nlm.nih.gov/clinvar/variation/127879/
NM_000023.2(SGCA):c.312+19A>C	concordant	unreportable	unreportable	388100	0	1	SCV000530321	Likely benign	GeneDx	11/28/2017	SGCA	chr17	48245116	48245116	http://gnomad.b	0.0001791091558	SAS	0.0008115789969	0		0	0	https://www.ncbi.nlm.nih.gov/clinvar/variation/388100/
NM_002225.3(IVD):c.273C>T	concordant	unreportable	unreportable	385437	0	1	SCV000526692	Likely benign	GeneDx	11/28/2017	IVD	chr15	40700167	40700167	http://gnomad.b	2.80E-05	NFE	6.27E-05	0		0	0	https://www.ncbi.nlm.nih.gov/clinvar/variation/385437/
NM_000051.3(ATM):c.8983C>A	concordant	unreportable	unreportable	184922	0	3	SCV000569077;S(	Uncertain significance;Unc	GeneDx;Illumina C	2017-11-28;2016-1	ATM	chr11	108235941	108235941	http://gnomad.b	1.67E-05	NFE	1.79E-05	0		0	0	https://www.ncbi.nlm.nih.gov/clinvar/variation/184922/
NM_000243.2(MEFV):c.1382G>A	concordant	unreportable	unreportable	234362	0	1	SCV000696046	Uncertain significance	Laboratory Corpora	1/25/2018	MEFV	chr16	3297221	3297221	http://gnomad.b	0.0002099076406	AFR	0.002810090082	0		0	0	https://www.ncbi.nlm.nih.gov/clinvar/variation/234362/
NM_000518.4(HBB):c.207C>T	concordant	unreportable	unreportable	439141	0	1	SCV000601258	Likely benign	Quest Diagnostics	8/1/2017	HBB	chr11	5247915	5247915	http://gnomad.b	0.0002309350303	NFE	0.0004299000138	0		0	0	https://www.ncbi.nlm.nih.gov/clinvar/variation/439141/
NM_000288.3(PEX7):c.-28G>A	concordant	unreportable	unreportable	355524	0	1	SCV000460520	Uncertain significance	Illumina Clinical Se	10/18/2016	PEX7	chr6	137143776	137143776		0.000252786204					2	0	https://www.ncbi.nlm.nih.gov/clinvar/variation/355524/
NM_000441.1(SLC26A4):c.463A>G	concordant	unreportable	unreportable	229259	0	1	SCV000272442	Uncertain significance	Laboratory for Mol	4/26/2016	SLC26A4	chr7	107314656	107314656	http://gnomad.b	0.0001563555749	EAS	0.0001739330037	0		0	0	https://www.ncbi.nlm.nih.gov/clinvar/variation/229259/
NM_000018.3(ACADVL):c.1226C>T	concordant	unreportable	unreportable	21013	0	1	SCV000654921	Uncertain significance	Invitae	10/5/2017	ACADVL	chr17	7127006	7127006	http://gnomad.b	0.0001217647392	EAS	0.0004058440099	0		1	0	https://www.ncbi.nlm.nih.gov/clinvar/variation/21013/
NM_000071.2(CBS):c.1411G>A	concordant	unreportable	unreportable	212882	0	1	SCV000249733	Uncertain significance	GeneDx	11/28/2017	CBS	chr21	44478311	44478311	http://gnomad.b	3.94E-05	AMR	0.0004168899904	0		0	0	https://www.ncbi.nlm.nih.gov/clinvar/variation/212882/
NM_000271.4(NPC1):c.3217G>A	concordant	unreportable	unreportable	255696	0	1	SCV000341094	Likely benign	EGL Genetic Diagn	6/30/2017	NPC1	chr18	21116665	21116665	http://gnomad.b	0.0009235220788	NFE	0.00199705991	1		0	0	https://www.ncbi.nlm.nih.gov/clinvar/variation/255696/
NM_000023.2(SGCA):c.929A>G	concordant	unreportable	unreportable	282511	0	2	SCV000334021;S(	Uncertain significance;Unc	EGL Genetic Diagn	2017-06-30;2017-1	SGCA	chr17	48247685	48247685	http://gnomad.b	0.0001567205114	FIN	0.001929290011	0		0	0	https://www.ncbi.nlm.nih.gov/clinvar/variation/282511/
NM_017739.3(POMGNT1):c.269G>A	concordant	unreportable	unreportable	290280	0	2	SCV000344801;S(	Uncertain significance;Unc	EGL Genetic Diagn	2017-06-30;2017-1	POMGNT1	chr1	46662488	46662488	http://gnomad.b	8.12E-05	AFR	0.0007188600139	0		1	0	https://www.ncbi.nlm.nih.gov/clinvar/variation/290280/
NM_000466.2(PEX1):c.3250A>G	concordant	unreportable	unreportable	360916	0	1	SCV000470498	Uncertain significance	Illumina Clinical Se	10/18/2016	PEX1	chr7	92120774	92120774	http://gnomad.b	0.0001220317172	AFR	0.0004574570048	0		0	0	https://www.ncbi.nlm.nih.gov/clinvar/variation/360916/
NM_000035.3(ALDOB):c.525C>T	concordant	unreportable	unreportable	501416	0	1	SCV000707765	Uncertain significance	EGL Genetic Diagn	6/30/2017	ALDOB	chr9	104189779	104189779	http://gnomad.b	0.0001438516128	AFR	0.001764480025	0		0	0	https://www.ncbi.nlm.nih.gov/clinvar/variation/501416/
NM_000642.2(AGL):c.3635T>C	concordant	unreportable	unreportable	456491	0	1	SCV000626732	Uncertain significance	Invitae	10/5/2017	AGL	chr1	100368285	100368285	http://gnomad.b	3.35E-05	AMR	2.98E-05	0		0	0	https://www.ncbi.nlm.nih.gov/clinvar/variation/456491/
NM_001876.3(CPT1A):c.1163+1G>A	concordant	reportable	reportable	371246	1	0	SCV000638091	Pathogenic	Invitae	10/5/2017	CPT1A	chr11	68552282	68552282	http://gnomad.b	8.17E-05	ASJ	0.0009157509776	0		0	0	https://www.ncbi.nlm.nih.gov/clinvar/variation/371246/
NM_012434.4(SLC17A5):c.1432G>A	concordant	unreportable	unreportable	357920	0	1	SCV000464768	Uncertain significance	Illumina Clinical Se	10/18/2016	SLC17A5	chr6	74304856	74304856	http://gnomad.b	0.0005098048514	ASJ	0.004061739892	0		0	0	https://www.ncbi.nlm.nih.gov/clinvar/variation/357920/
NM_000137.2(FAH):c.831G>A	concordant	unreportable	unreportable	290848	0	1	SCV000345497	Uncertain significance	EGL Genetic Diagn	6/30/2017	FAH	chr15	80465480	80465480	http://gnomad.b	0.0001553561818	FIN	0.001659190049	0		0	0	https://www.ncbi.nlm.nih.gov/clinvar/variation/290848/
NM_000487.5(ARSA):c.659C>T	concordant	unreportable	unreportable	342237	0	1	SCV000439437	Uncertain significance	Illumina Clinical Se	10/18/2016	ARSA	chr22	51065287	51065287	http://gnomad.b	0.0001435075496	FIN	0.02792239934	10		1	0	https://www.ncbi.nlm.nih.gov/clinvar/variation/342237/
NM_002485.4(NBN):c.1035C>T	concordant	unreportable	unreportable	127853	0	4	SCV000149684;S(	Uncertain significance;Beni	GeneDx;Invitae;Lal	2017-07-13;2017-1	NBN	chr8	90971042	90971042	http://gnomad.b	0.0004740658115	EAS	0.00539193023	0		0	0	https://www.ncbi.nlm.nih.gov/clinvar/variation/127853/
NM_000053.3(ATP7B):c.3786C>T	concordant	unreportable	unreportable	288242	0	1	SCV000342294	Uncertain significance	EGL Genetic Diagn	6/30/2017	ATP7B	chr13	52511729	52511729	http://gnomad.b	9.26E-05	EAS	0.0003478660074	0		1	0	https://www.ncbi.nlm.nih.gov/clinvar/variation/288242/
NM_000051.3(ATM):c.6998C>A	concordant	unreportable	unreportable	127434	0	2	SCV000149148;S(	Uncertain significance;Likel	GeneDx;Invitae	2017-11-28;2017-1	ATM	chr11	108198394	108198394	http://gnomad.b	0.0001086036357	AFR	0.001438279985	0		0	0	https://www.ncbi.nlm.nih.gov/clinvar/variation/127434/
NM_006493.2(CLN5):c.712+3_712+4d	concordant	unreportable	unreportable	457967	0	1	SCV000628968	Uncertain significance	Invitae	10/5/2017	CLN5	chr13	77570264	77570264	http://gnomad.b	5.87E-05	AFR	0.0005886200233	0		0	0	https://www.ncbi.nlm.nih.gov/clinvar/variation/457967/
NM_001079802.1(FKTN):c.1249G>A	concordant	unreportable	unreportable	264522	0	2	SCV000320516;S(	Uncertain significance;Unc	Ambry Genetics;EG	2017-11-14;2017-0	FKTN	chr9	108397408	108397408	http://gnomad.b	4.25E-05	NFE	7.17E-05	0		0	0	https://www.ncbi.nlm.nih.gov/clinvar/variation/264522/
NM_017890.4(VPS13B):c.1289G>C	concordant	unreportable	unreportable	361041	0	1	SCV000470765	Uncertain significance	Illumina Clinical Se	10/18/2016	VPS13B	chr8	100146942	100146942	http://gnomad.b	0.0001287830006	NFE	0.0002962829894	0		0	0	https://www.ncbi.nlm.nih.gov/clinvar/variation/361041/
NM_000016.4(ACADM):c.558T>A	concordant	unreportable	unreportable	198379	1	1	SCV000231984;S(	Uncertain significance;Likel	EGL Genetic Diagn	2017-06-30;2017-1	ACADM	chr1	76205754	76205754	http://gnomad.b	2.99E-05	NFE	1.79E-05	0		0	0	https://www.ncbi.nlm.nih.gov/clinvar/variation/198379/
NM_000016.4(ACADM):c.698T>C	concordant	reportable	reportable	203539	2	0	SCV000268440;S(	Likely pathogenic;Likely pa	ARUP Laboratories	2017-06-28;2017-0	ACADM	chr1	76211589	76211589	http://gnomad.b	1.09E-05	AMR	2.98E-05	0		0	0	https://www.ncbi.nlm.nih.gov/clinvar/variation/203539/
NM_000492.3(CFTR):c.2450G>T	concordant	unreportable	unreportable	455766	0	1	SCV000625734	Uncertain significance	Invitae	10/5/2017	CFTR	chr7	117232671	117232671	http://gnomad.b	2.89E-05	NFE	8.92E-05	0		0	0	https://www.ncbi.nlm.nih.gov/clinvar/variation/455766/
NM_000136.2(FANCC):c.535C>T	concordant	reportable	reportable	235535	1	0	SCV000568699	Likely pathogenic	GeneDx	11/28/2017	FANCC	chr9	97912356	97912356	http://gnomad.b	1.66E-05	NFE	1.79E-05	0		0	0	https://www.ncbi.nlm.nih.gov/clinvar/variation/235535/

Supplemental Table 2

NM_000151.3(G6PC):c.969C>A	concordant	reportable	reportable	188777	1	0	SCV000343386	Pathogenic	EGL Genetic Diagn	6/30/2017	G6PC	chr17	41063338	41063338		1.02E-05					0	0	https://www.ncbi.nlm.nih.gov/clinvar/variation/188777/
NM_000152.3(GAA):c.1190C>T	concordant	unreportable	unreportable	456370	0	1	SCV000626497	Uncertain significance	Invitae	10/5/2017	GAA	chr17	78082402	78082402	http://gnomad.b	2.22E-05	NFE	5.43E-05	0		0	0	https://www.ncbi.nlm.nih.gov/clinvar/variation/456370/
NM_000152.3(GAA):c.2297A>C	concordant	reportable	reportable	420102	1	0	SCV000568678	Pathogenic	GeneDx	11/28/2017	GAA	chr17	78090874	78090874	http://gnomad.b	5.56E-06	EAS	5.80E-05	0		0	0	https://www.ncbi.nlm.nih.gov/clinvar/variation/420102/
NM_000152.3(GAA):c.2237G>T	concordant	unreportable	unreportable	284776	0	1	SCV000337526	Uncertain significance	EGL Genetic Diagn	6/30/2017	GAA	chr17	78090814	78090814	http://gnomad.b	6.39E-05	NFE	0.0001077330016	0		0	0	https://www.ncbi.nlm.nih.gov/clinvar/variation/284776/
NM_000153.3(GALC):c.628A>T	concordant	reportable	reportable	369724	3	0	SCV000484485;S	Pathogenic;Pathogenic;Pat	EGL Genetic Diagn	2017-06-30;2017-1	GALC	chr14	88442826	88442826	http://gnomad.b	3.08E-05	NFE	3.60E-05	0		0	0	https://www.ncbi.nlm.nih.gov/clinvar/variation/369724/
NM_000155.3(GALT):c.957C>A	concordant	reportable	reportable	3617	1	0	SCV000479771	Likely pathogenic	Illumina Clinical Se	10/18/2016	GALT	chr9	34649459	34649459	http://gnomad.b	1.10E-05	NFE	8.95E-06	0		0	0	https://www.ncbi.nlm.nih.gov/clinvar/variation/3617/
NM_172250.2(MMAA):c.1075C>T	concordant	reportable	reportable	466216	1	0	SCV000641765	Pathogenic	Invitae	10/5/2017	MMAA	chr4	146576404	146576404	http://gnomad.b	1.60E-05	NFE	8.95E-06	0		0	0	https://www.ncbi.nlm.nih.gov/clinvar/variation/466216/
NM_002485.4(NBN):c.2117C>G	concordant	reportable	reportable	182728	3	0	SCV000537662;S	Pathogenic;Pathogenic;Pat	Color Genomics, In	2017-01-26;2017-1	NBN	chr8	90955548	90955548	http://gnomad.b	1.39E-05	NFE	8.96E-06	0		0	0	https://www.ncbi.nlm.nih.gov/clinvar/variation/182728/
NM_000441.1(SLC26A4):c.2283A>G	concordant	unreportable	unreportable	282912	0	1	SCV000334652	Uncertain significance	EGL Genetic Diagn	6/30/2017	SLC26A4	chr7	107353031	107353031	http://gnomad.b	0.0001284349365	EAS	0.001333490014	0		0	0	https://www.ncbi.nlm.nih.gov/clinvar/variation/282912/
NM_000441.1(SLC26A4):c.1678G>A	concordant	unreportable	unreportable	229254	0	1	SCV000272437	Uncertain significance	Laboratory for Mol	4/26/2016	SLC26A4	chr7	107340591	107340591	http://gnomad.b	1.95E-05	SAS	6.50E-05	0		0	0	https://www.ncbi.nlm.nih.gov/clinvar/variation/229254/
NM_000543.4(SMPD1):c.1624C>T	concordant	reportable	reportable	93318	1	0	SCV000231558	Pathogenic	EGL Genetic Diagn	6/30/2017	SMPD1	chr11	6415565	6415565	http://gnomad.b	1.67E-05	SAS	0.0005197839928	0		0	0	https://www.ncbi.nlm.nih.gov/clinvar/variation/93318/
NM_017890.4(VPS13B):c.7441G>A	concordant	unreportable	unreportable	95865	0	1	SCV000470823	Uncertain significance	Illumina Clinical Se	10/18/2016	VPS13B	chr8	100789121	100789121	http://gnomad.b	0.0002015733923	AFR	0.0006535089924	0		0	0	https://www.ncbi.nlm.nih.gov/clinvar/variation/95865/
NM_000492.3(CFTR):c.1117-1G>A	concordant	reportable	reportable	209059	1	0	SCV000245963	Pathogenic	CFTR2	6/29/2017	CFTR	chr7	117182069	117182069		3.85E-06					0	0	https://www.ncbi.nlm.nih.gov/clinvar/variation/209059/
NM_000155.3(GALT):c.864C>T	concordant	unreportable	unreportable	203732	0	2	SCV000603798;S	Uncertain significance;Unc	ARUP Laboratories	2017-06-30;2017-1	GALT	chr9	34649038	34649038	http://gnomad.b	2.20E-05	AFR	0.0003920540039	0		0	0	https://www.ncbi.nlm.nih.gov/clinvar/variation/203732/
NM_000518.4(HBB):c.*132C>T	concordant	unreportable	unreportable	439130	0	1	SCV000601231	Uncertain significance	Quest Diagnostics I	8/1/2017	HBB	chr11	5246696	5246696		4.67E-06					0	0	https://www.ncbi.nlm.nih.gov/clinvar/variation/439130/
NM_000152.3(GAA):c.1143delC	concordant	reportable	reportable	370263	1	0	SCV000706085	Pathogenic	EGL Genetic Diagn	6/30/2017	GAA	chr17	78082353	78082354	http://gnomad.b	1.11E-05	NFE	9.05E-06	0		0	0	https://www.ncbi.nlm.nih.gov/clinvar/variation/370263/
NM_000277.1(PAH):c.556delA	concordant	reportable	reportable	102733	1	0	SCV000696456	Likely pathogenic	Laboratory Corpora	1/25/2018	PAH	chr12	103249063	103249064		2.74E-06					0	0	https://www.ncbi.nlm.nih.gov/clinvar/variation/102733/
NM_000016.4(ACADM):c.1114dupG	concordant	reportable	reportable	370160	1	0	SCV000693954	Pathogenic	Laboratory Corpora	1/25/2018	ACADM	chr1	76226974	76226974	http://gnomad.b	5.44E-06	NFE	8.97E-06	0		0	0	https://www.ncbi.nlm.nih.gov/clinvar/variation/370160/
NM_001079802.1(FKTN):c.642dupT	concordant	reportable	reportable	93523	2	0	SCV000491171;S	Pathogenic;Pathogenic	GeneDx;Invitae	2016-12-21;2017-0	FKTN	chr9	108366764	108366764	http://gnomad.b	2.84E-05	AMR	0.001013469999	0		0	0	https://www.ncbi.nlm.nih.gov/clinvar/variation/93523/
NM_017890.4(VPS13B):c.9793dupA	concordant	reportable	reportable	279782	1	0	SCV000329308	Pathogenic	GeneDx	11/28/2017	VPS13B	chr8	100847523	100847523	http://gnomad.b	5.60E-06	AMR	2.98E-05	0		0	0	https://www.ncbi.nlm.nih.gov/clinvar/variation/279782/
NM_000053.3(ATP7B):c.628A>G	concordant	unreportable	unreportable	92390	0	2	SCV000626867;S	Likely benign;Uncertain sig	Invitae;Laboratory	2017-10-05;2018-0	ATP7B	chr13	52548728	52548728	http://gnomad.b	0.0004882264473	AFR	0.006737309974	0		1	0	https://www.ncbi.nlm.nih.gov/clinvar/variation/92390/
NM_017890.4(VPS13B):c.9206A>C	concordant	unreportable	unreportable	197914	0	2	SCV000231268;S	Uncertain significance;Unc	EGL Genetic Diagn	2017-06-30;2017-1	VPS13B	chr8	100833658	100833658	http://gnomad.b	0.000111985218	NFE	0.0003049870138	0		0	0	https://www.ncbi.nlm.nih.gov/clinvar/variation/197914/
NM_133647.1(SLC12A6):c.3249G>A	concordant	unreportable	unreportable	289459	0	1	SCV000343828	Uncertain significance	EGL Genetic Diagn	6/30/2017	SLC12A6	chr15	34527494	34527494	http://gnomad.b	0.0001392774169	AFR	0.001960270107	0		0	0	https://www.ncbi.nlm.nih.gov/clinvar/variation/289459/
NM_000441.1(SLC26A4):c.535G>A	concordant	unreportable	unreportable	423822	0	1	SCV000573568	Uncertain significance	GeneDx	11/28/2017	SLC26A4	chr7	107314728	107314728	http://gnomad.b	0.0002875825753	AFR	0.003593829926	0		0	0	https://www.ncbi.nlm.nih.gov/clinvar/variation/423822/
NM_000152.3(GAA):c.868A>G	concordant	unreportable	unreportable	498117	0	1	SCV000702967	Uncertain significance	EGL Genetic Diagn	6/30/2017	GAA	chr17	78081608	78081608	http://gnomad.b	0.0001111722559	AFR	6.74E-05	0		0	0	https://www.ncbi.nlm.nih.gov/clinvar/variation/498117/
NM_017739.3(POMGNT1):c.208G>A	concordant	unreportable	unreportable	282829	0	2	SCV000334493;S	Uncertain significance;Unc	EGL Genetic Diagn	2017-06-30;2017-1	POMGNT1	chr1	46662669	46662669	http://gnomad.b	5.60E-05	AMR	0.0001488889975	0		0	0	https://www.ncbi.nlm.nih.gov/clinvar/variation/282829/
NM_001360.2(DHCR7):c.1018G>A	concordant	unreportable	unreportable	458677	0	1	SCV000630071	Benign	Invitae	10/5/2017	DHCR7	chr11	71146831	71146831	http://gnomad.b	6.19E-05	FIN	0.01012740005	1		0	0	https://www.ncbi.nlm.nih.gov/clinvar/variation/458677/
NM_000642.2(AGL):c.2506G>A	concordant	unreportable	unreportable	456473	0	1	SCV000626697	Uncertain significance	Invitae	10/5/2017	AGL	chr1	100349967	100349967	http://gnomad.b	1.12E-05	EAS	0.0002323960071	0		0	0	https://www.ncbi.nlm.nih.gov/clinvar/variation/456473/
NM_000051.3(ATM):c.7739G>A	concordant	unreportable	unreportable	478937	0	1	SCV000682433	Uncertain significance	Color Genomics, In	10/26/2017	ATM	chr11	108202715	108202715	http://gnomad.b	1.11E-05	EAS	0.0002902930137	0		0	0	https://www.ncbi.nlm.nih.gov/clinvar/variation/478937/
NM_000051.3(ATM):c.5055C>T	concordant	unreportable	unreportable	132750	0	2	SCV000682246;S	Likely benign;Likely benign	Color Genomics, In	2017-10-26;2017-1	ATM	chr11	108170490	108170490	http://gnomad.b	2.23E-05	EAS	0.0005799789797	0		0	0	https://www.ncbi.nlm.nih.gov/clinvar/variation/132750/
NM_000051.3(ATM):c.7044G>A	concordant	unreportable	unreportable	136429	0	2	SCV000687753;S	Likely benign;Likely benign	Color Genomics, In	2017-12-21;2017-1	ATM	chr11	108198440	108198440	http://gnomad.b	6.68E-05	AFR	0.0006536800065	0		0	0	https://www.ncbi.nlm.nih.gov/clinvar/variation/136429/
NM_000271.4(NPC1):c.2269G>A	concordant	unreportable	unreportable	326264	0	3	SCV000703020;S	Uncertain significance;Unc	EGL Genetic Diagn	2017-06-30;2016-1	NPC1	chr18	21121374	21121374	http://gnomad.b	2.29E-05	AFR	6.54E-05	0		0	0	https://www.ncbi.nlm.nih.gov/clinvar/variation/326264/
NM_004646.3(NPHS1):c.597G>A	concordant	unreportable	unreportable	259505	0	1	SCV000411583	Uncertain significance	Illumina Clinical Se	10/18/2016	NPHS1	chr19	36341277	36341277	http://gnomad.b	0.0002564545713	AFR	0.003659649985	0		1	0	https://www.ncbi.nlm.nih.gov/clinvar/variation/259505/
NM_000303.2(PMM2):c.*2G>A	concordant	unreportable	unreportable	384229	0	1	SCV000524952	Likely benign	GeneDx	11/28/2017	PMM2	chr16	8941684	8941684	http://gnomad.b	4.45E-05	EAS	5.81E-05	0		0	0	https://www.ncbi.nlm.nih.gov/clinvar/variation/384229/
NM_000152.3(GAA):c.270C>T	concordant	unreportable	unreportable	456413	0	1	SCV000626593	Likely benign	Invitae	10/5/2017	GAA	chr17	78078655	78078655	http://gnomad.b	5.00E-05	EAS	0.0005818689824	0		0	0	https://www.ncbi.nlm.nih.gov/clinvar/variation/456413/
NM_000023.2(SGCA):c.172G>C	concordant	unreportable	unreportable	497667	0	1	SCV000702306	Uncertain significance	EGL Genetic Diagn	6/30/2017	SGCA	chr17	48244957	48244957	http://gnomad.b	3.64E-05	AFR	0.0003272680042	0		0	0	https://www.ncbi.nlm.nih.gov/clinvar/variation/497667/
NM_000243.2(MEFV):c.1222C>T	concordant	unreportable	unreportable	457994	0	1	SCV000629017	Uncertain significance	Invitae	10/5/2017	MEFV	chr16	3299469	3299469	http://gnomad.b	3.92E-05	NFE	1.80E-05	0		0	0	https://www.ncbi.nlm.nih.gov/clinvar/variation/457994/
NM_001360.2(DHCR7):c.288C>T	concordant	unreportable	unreportable	501852	0	1	SCV000708363	Uncertain significance	EGL Genetic Diagn	6/30/2017	DHCR7	chr11	71155072	71155072	http://gnomad.b	5.39E-05	EAS	5.81E-05	0		0	0	https://www.ncbi.nlm.nih.gov/clinvar/variation/501852/
NM_000492.3(CFTR):c.2820T>G	concordant	unreportable	unreportable	93151	0	3	SCV000110857;S	Likely benign;Benign;Uncer	EGL Genetic Diagn	2017-06-30;2017-1	CFTR	chr7	117243748	117243748	http://gnomad.b	0.0004044247924	AFR	0.004575159866	0		0	0	https://www.ncbi.nlm.nih.gov/clinvar/variation/93151/
NM_000051.3(ATM):c.5868C>T	concordant	unreportable	unreportable	453600	0	2	SCV000687647;S	Likely benign;Likely benign	Color Genomics, In	2017-12-21;2017-1	ATM	chr11	108180992	108180992	http://gnomad.b	2.23E-05	SAS	0.0002924170112	0		0	0	https://www.ncbi.nlm.nih.gov/clinvar/variation/453600/
NM_000360.3(TH):c.-18G>A	concordant	unreportable	unreportable	304088	0	1	SCV000369936	Uncertain significance	Illumina Clinical Se	10/18/2016	TH	chr11	2193034	2193034	http://gnomad.b	6.76E-05	EAS	0.0002333180018	0		0	0	https://www.ncbi.nlm.nih.gov/clinvar/variation/304088/
NM_000051.3(ATM):c.1351C>T	concordant	unreportable	unreportable	185437	0	3	SCV000681971;S	Uncertain significance;Unc	Color Genomics, In	2017-10-26;2017-1	ATM	chr11	108121543	108121543	http://gnomad.b	0.0001364507218	SAS	0.0007471899735	0		1	0	https://www.ncbi.nlm.nih.gov/clinvar/variation/185437/
NM_000108.3(DLD):c.100A>G	concordant	unreportable	unreportable	203672	0	3	SCV000603328;S	Uncertain significance;Unc	ARUP Laboratories	2017-06-30;2017-0	DLD	chr7	107533705	107533705	http://gnomad.b	0.0005815523293	NFE	0.0009858929552	0		0	0	https://www.ncbi.nlm.nih.gov/clinvar/variation/203672/
NM_000642.2(AGL):c.880C>T	concordant	unreportable	unreportable	291328	0	1	SCV000346061	Uncertain significance	Illumina Clinical Se	10/18/2016	AGL	chr1	100336347	100336347		3.07E-05					0	0	https://www.ncbi.nlm.nih.gov/clinvar/variation/291328/
NM_000391.3(TPP1):c.1217A>G	concordant	unreportable	unreportable	207589	0	2	SCV000243433;S	Uncertain significance;Unc	GeneDx;Invitae	2017-11-28;2017-0	TPP1	chr11	6636722	6636722	http://gnomad.b	1.68E-05	AMR	0.0001786880021	0		0	0	https://www.ncbi.nlm.nih.gov/clinvar/variation/207589/
NM_000071.2(CBS):c.670C>T	concordant	unreportable	unreportable	212851	0	3	SCV000344856;S	Uncertain significance;Unc	EGL Genetic Diagn	2017-06-30;2017-1	CBS	chr21	44485379	44485379	http://gnomad.b	0.0002333965097	NFE	0.0003670940059	0		0	0	https://www.ncbi.nlm.nih.gov/clinvar/variation/212851/
NM_000071.2(CBS):c.352G>A	concordant	unreportable	unreportable	340090	0	1	SCV000436229	Uncertain significance	Illumina Clinical Se	10/18/2016	CBS	chr21	44486452	44486452	http://gnomad.b	2.25E-05	FIN	4.52E-05	0		0	0	https://www.ncbi.nlm.nih.gov/clinvar/variation/340090/
NM_000492.3(CFTR):c.360G>A	concordant	unreportable	unreportable	493462	0	2	SCV000700457;S	Uncertain significance;Unc	EGL Genetic Diagn	2017-06-30;2018-0	CFTR	chr7	117171039	117171039	http://gnomad.b	0.0001367340965	AFR	0.0007846209919	0		0	0	https://www.ncbi.nlm.nih.gov/clinvar/variation/493462/
NM_000057.2(BLM):c.274A>G	concordant	unreportable	unreportable	127493	0	2	SCV000257607;S	Uncertain significance;Unc	Division of Genomi	2015-08-10;2017-1	BLM	chr15	91292772	91292772	http://gnomad.b	0.0001831327594	NFE	0.000250860001	0		0	0	https://www.ncbi.nlm.nih.gov/clinvar/variation/127493/
NM_017739.3(POMGNT1):c.1855A>T	concordant	unreportable	unreportable	452605	0	2	SCV000708310;S	Uncertain significance;Unc	EGL Genetic Diagn	2017-06-30;2017-1	POMGNT1	chr1	46655170	46655170	http://gnomad.b	3.36E-05	NFE	9.89E-05	0		0	0	https://www.ncbi.nlm.nih.gov/clinvar/variation/452605/
NM_000051.3(ATM):c.2149C>T	concordant	unreportable	unreportable	140879	0	4	SCV000537605;S	Uncertain significance;Unc	Color Genomics, In	2017-12-21;2017-1	ATM	chr11	108126966	108126966	http://gnomad.b	3.06E-05	AFR	0.0001308729989	0		0	0	https://www.ncbi.nlm.nih.gov/clinvar/variation/140879/
NM_000098.2(CPT2):c.1858T>C	concordant	unreportable	unreportable	460430	0	1	SCV000632600	Uncertain significance	Invitae	10/5/2017	CPT2	chr1	53679148	53679148	http://gnomad.b	2.52E-05	NFE	1.79E-05	0		0	0	https://www.ncbi.nlm.nih.gov/clinvar/variation/460430/
NM_000136.2(FANCC):c.1561G>A	concordant	unreportable	unreportable	234512	0	1	SCV000279409	Uncertain significance	GeneDx	11/28/2017	FANCC	chr9	97864105	97864105	http://gnomad.b	1.19E-05	EAS	5.80E-05	0		0	0	https://www.ncbi.nlm.nih.gov/clinvar/variation/234512/
NM_000492.3(CFTR):c.1210-13_1210-	concordant	unreportable	unreportable	291054	0	1	SCV000345737	Uncertain significance	EGL Genetic Diagn	6/30/2017	CFTR	chr7	117188680	117188684		1.93E-06					0	0	https://www.ncbi.nlm.nih.gov/clinvar/variation/291054/
NM_001360.2(DHCR7):c.855C>T	concordant	unreportable	unreportable	287781	0	1	SCV000341683	Uncertain significance	EGL Genetic Diagn	6/30/2017	DHCR7	chr11	71148966	71148966	http://gnomad.b	7.81E-05	NFE	0.000161168995	0		0	0	https://www.ncbi.nlm.nih.gov/clinvar/variation/287781/
NM_017739.3(POMGNT1):c.1454G>A	concordant	unreportable	unreportable	288113	0	2	SCV000342119;S	Uncertain significance;Unc	EGL Genetic Diagn	2017-06-30;2017-1	POMGNT1	chr1	46657855	46657855	http://gnomad.b	0.0001287801164	SAS	0.0006172439898	0		0	0	https://www.ncbi.nlm.nih.gov/clinvar/variation/288113/
NM_017739.3(POMGNT1):c.1924A>G	concordant	unreportable	unreportable	448109	0	1	SCV000614741	Uncertain significance	Athena Diagnostics	8/17/2017	POMGNT1	chr1	46655001	46655001	http://gnomad.b	3.92E-05	EAS	0.000290158001	0		0	0	https://www.ncbi.nlm.nih.gov/clinvar/variation/448109/
NM_014363.4(SACS):c.8022T>G	concordant	unreportable	unreportable	311521	0	2	SCV000614981;S	Uncertain significance;Unc	Athena Diagnostics	2017-08-17;2016-1	SACS	chr13	23909993	23909993	http://gnomad.b	8.60E-05	EAS	0.001855719951	0		0	0	https://www.ncbi.nlm.nih.gov/clinvar/variation/311521/
NM_138694.3(PKHD1):c.3373A>C	concordant	unreportable	unreportable	282761	0	1	SCV000334377	Uncertain significance	EGL Genetic Diagn	6/30/2017	PKHD1	chr6	51893141	51893141	http://gnomad.b	0.0001334386016	AFR	0.0008495619986	0		0	0	https://www.ncbi.nlm.nih.gov/clinvar/variation/282761/
NM_004543.4(NEB):c.7441A>G	concordant	unreportable	unreportable	218577	0	2	SCV000257877;S	Uncertain significance;Unc	Division of Genomi	2015-08-10;2016-1	NEB	chr2	152502739	152502739	http://gnomad.b	0.0001213354124	NFE	0.0001320629963	0		0	0	https://www.ncbi.nlm.nih.gov/clinvar/variation/218577/
NM_000112.3(SLC26A2):c.1081G>A	concordant	unreportable	unreportable	290033	0	1	SCV000344509	Uncertain significance	EGL Genetic Diagn	6/30/2017	SLC26A2	chr5	149360237	149360237	http://gnomad.b	0.0002927220924	NFE	0.0004308409989	0		0	0	https://www.ncbi.nlm.nih.gov/clinvar/variation/290033/
NM_000051.3(ATM):c.6338C>G	concordant	unreportable	unreportable	233776	0	1	SCV000622656	Uncertain significance	Invitae	10/5/2017	ATM	chr11	108188239	108188239	http://gnomad.b	3.06E-05	SAS	0.0003270329908	0		0	0	https://www.ncbi.nlm.nih.gov/clinvar/variation/233776/
NM_000051.3(ATM):c.4549C>T	concordant	unreportable	unreportable	216219	0	1	SCV000254112	Uncertain significance	Invitae	10/5/2017	ATM	chr11	108163458	108163458	http://gnomad.b	1.39E-05	SAS	6.50E-05	0		0	0	https://www.ncbi.nlm.nih.gov/clinvar/variation/216219/
NM_002485.4(NBN):c.2129C>T	concordant	unreportable	unreportable	411741	0	1	SCV000553044	Uncertain significance	Invitae	3/14/2017	NBN	chr8	90955536	90955536		2.79E-06					0	0	https://www.ncbi.nlm.nih.gov/clinvar/variation/411741/
NM_000023.2(SGCA):c.819G>T	concordant	unreportable	unreportable	281388	0	1	SCV000332157	Uncertain significance	EGL Genetic Diagn	6/30/2017	SGCA	chr17	48247575	48247575	http://gnomad.b	5.04E-05	AFR	0.0005227390211	0		0	0	https://www.ncbi.nlm.nih.gov/clinvar/variation/281388/
NM_004004.5(GJB2):c.*1C>T	concordant	unreportable	unreportable	44715	0	2	SCV000382990;S	Likely benign;Benign	Illumina Clinical Se	2016-10-18;2018-0	GJB2	chr13	20763039	20763039	http://gnomad.b	0.0005169012747	AMR	0.001999049913	0		0	0	https://www.ncbi.nlm.nih.gov/clinvar/variation/44715/
NM_000352.3(ABCC8):c.3975C>T	concordant	unreportable	unreportable	303756	0	3	SCV000612212;S	Likely benign;Likely benign	Athena Diagnostics	2017-08-17;2017-0	ABCC8	chr11	17418753	17418753	http://gnomad.b	0.0003820005472	AFR	0.004901960026	1		0	0	https://www.ncbi.nlm.nih.gov/clinvar/variation/303756/
NM_000227.3(LAMA3):c.3837C>T	concordant	unreportable	unreportable	326338	0	1	SCV000408009	Uncertain significance	Illumina Clinical Se	10/18/2016	LAMA3	chr18	21512211	21512211	http://gnomad.b	4.76E-05	AFR	0.0001306850027	0		0	0	https://www.ncbi.nlm.nih.gov/clinvar/variation/326338/
NM_000051.3(ATM):c.6357A>T	concordant	unreportable	unreportable	414584	0	1	SCV000558399	Likely benign	Invitae	10/5/2017	ATM	chr11	108190690	108190690		2.78E-06					0	0	https://www.ncbi.nlm.nih.gov/clinvar/variation/414584/
NM_024649.4(BBS1):c.1277A>G	concordant	unreportable	unreportable	462952	0	1	SCV000636513	Uncertain significance	Invitae	10/5/2017	BBS1	chr11	66294216	66294216	http://gnomad.b	5.57E-05	SAS	0.0002598919964	0		0	0	https://www.ncbi.nlm.nih.gov/clinvar/variation/462952/
NM_000243.2(MEFV):c.1458C>T	concordant	unreportable	unreportable	439882	0	1	SCV000604180	Likely benign	ARUP Laboratories	6/30/2017	MEFV	chr16	3297145	3297145	http://gnomad.b	3.64E-05	AFR	0.0003267120046	0		0	0	https://www.ncbi.nlm.nih.gov/clinvar/variation/439882/
NM_000414.3(HSD17B4):c.501A>C	concordant	unreportable	unreportable	501432	0	1	SCV000707783	Uncertain significance	EGL Genetic Diagn	6/30/2017	HSD17B4	chr5	118814595	118814595	http://gnomad.b	5.63E-05	SAS	6.50E-05	0		0	0	https://www.ncbi.nlm.nih.gov/clinvar/variation/501432/
NM_017890.4(VPS13B):c.11413G>T	concordant	unreportable	unreportable	361096	0	1	SCV000470863	Uncertain significance	Illumina Clinical Se	10/18/2016	VPS13B	chr8	100880639	100880639	http://gnomad.b	0.0001819759792	NFE	0.000331304007	0		0	0	https://www.ncbi.nlm.nih.gov/clinvar/variation/361096/
NM_000492.3(CFTR):c.927C>G	concordant	unreportable	unreportable	256257	0	1	SCV000625753	Likely benign	Invitae	10/5/2017	CFTR	chr7	117180211	117180211	http://gnomad.b	4.24E-05	SAS	9.75E-05	0		0	0	https://www.ncbi.nlm.nih.gov/clinvar/variation/256257/
NM_000243.2(MEFV):c.1043G>A	concordant	unreportable	unreportable	97428	0	3	SCV000604191;S	Uncertain significance;Unc	ARUP Laboratories	2017-06-30;2017-1	MEFV	chr16	3299648	3299648	http://gnomad.b	0.0001063532046	SAS	0.0003909810039	0		0	0	https://www.ncbi.nlm.nih.gov/clinvar/variation/97428/
NM_000027.3(AGA):c.303A>T	concordant	unreportable	unreportable	254724	0	1	SCV000448737	Uncertain significance	Illumina Clinical Se	10/18/2016	AGA	chr4	178360821	178360821	http://gnomad.b	0.0004001565057	AFR	0.005358779803	0		0	0	https://www.ncbi.nlm.nih.gov/clinvar/variation/254724/
NM_000152.3(GAA):c.909C>T	concordant	unreportable	unreportable	456443	0	1	SCV000626653	Likely benign	Invitae	10/5/2017	GAA	chr17	78081649	78081649	http://gnomad.b	2.50E-05	AFR	0.0002012610057	0		0	0	https://www.ncbi.nlm.nih.gov/clinvar/variation/456443/
NM_000152.3(GAA):c.1842G>A	concordant	unreportable	unreportable	289635	0	1	SCV000344023	Uncertain significance	EGL Genetic Diagn	6/30/2017	GAA	chr17	78086464	78086464	http://gnomad.b	3.61E-05	AFR	0.0001387730008	0		0	0	https://www.ncbi.nlm.nih.gov/clinvar/variation/289635/
NM_001876.3(CPT1A):c.1983C>T	concordant	unreportable	unreportable	387345	0	1	SCV000529341	Likely benign	GeneDx	11/28/2017	CPT1A	chr11	68529048	68529048	http://gnomad.b	0.0001492495283	AFR	0.001176159945	0		0	0	https://www.ncbi.nlm.nih.gov/clinvar/variation/387345/
NM_014363.4(SACS):c.4232T>G	concordant	reportable	reportable	450250	1	0	SCV000618805	Pathogenic	GeneDx	11/28/2017	SACS	chr13	23913783	23913783		2.96E-06					0	0	https://www.ncbi.nlm.nih.gov/clinvar/variation/450250/
NM_000152.3(GAA):c.2215A>T	concordant	unreportable	unreportable	456394	0	1	SCV000626552	Uncertain significance	Invitae	10/5/2017	GAA	chr17	78090792	78090792	http://gnomad.b	8.06E-05	SAS	0.001786760055	1		0	0	https://www.ncbi.nlm.nih.gov/clinvar/variation/456394/
NM_014363.4(SACS):c.6030G>T	concordant	unreportable	unreportable	448211	0	1	SCV000614966	Uncertain significance	Athena Diagnostics	8/17/2017	SACS	chr13	23911985	23911985	http://gnomad.b	2.96E-05	NFE	2.70E-05	0		0	0	https://www.ncbi.nlm.nih.gov/clinvar/variation/448211/
NM_000466.2(PEX1):c.3371A>G	concordant	unreportable	unreportable	498032	0	1	SCV000702842	Uncertain significance	EGL Genetic Diagn	6/30/2017	PEX1	chr7	92120653	92120653	http://gnomad.b	0.0002579306749	AFR	0.002940790029	1		0	0	https://www.ncbi.nlm.nih.gov/clinvar/variation/498032/
NM_000441.1(SLC26A4):c.2018T>G	concordant	unreportable	unreportable	358502	0	1	SCV000466106	Uncertain significance	Illumina Clinical Se	10/18/2016	SLC26A4	chr7	107342486	107342486	http://gnomad.b	2.79E-06	NFE	8.97E-06	0		0	0	https://www.ncbi.nlm.nih.gov/clinvar/variation/358502/
NM_000051.3(ATM):c.4631A>G	concordant	unreportable	unreportable	245791	0	3	SCV000682216;S	Uncertain significance;Unc	Color Genomics, In	2017-10-26;2017-0	ATM	chr11	108164059	108164059	http://gnomad.b	5.85E-05	NFE	7.45E-05	0		0	0	https://www.ncbi.nlm.nih.gov/clinvar/variation/245791/
NM_000520.4(HEXA):c.233G>A	concordant	reportable	reportable	453185	1	0	SCV000622059	Pathogenic	GeneDx	11/28/2017	HEXA	chr15	72668081	72668081	http://gnomad.b	9.39E-06	NFE	8.96E-06	0		0	0	https://www.ncbi.nlm.nih.gov/clinvar/variation/453185/
NM_001042432.1(CLN3):c.319C>T	concordant	unreportable	unreportable	434791	0	1	SCV000594151	Uncertain significance	Genetic Services La	7/5/2017	CLN3	chr16	28499038	28499038	http://gnomad.b	2.22E-05	NFE	1.80E-05	0		0	0	https://www.ncbi.nlm.nih.gov/clinvar/variation/434791/
NM_005476.5(GNE):c.2050C>T	concordant	unreportable	unreportable	283051	0	1	SCV000334871	Uncertain significance	EGL Genetic Diagn	6/30/2017	GNE	chr9	36217481	36217481	http://gnomad.b	2.52E-05	NFE	6.27E-05	0		0	0	https://www.ncbi.nlm.nih.gov/clinvar/variation/283051/
NM_000182.4(HADHA):c.1465A>G	concordant	unreportable	unreportable	495725	0	1	SCV000695939	Uncertain significance	Laboratory Corpora	1/25/2018	HADHA	chr2	26420574	26420574	http://gnomad.b	0.0003756953167	EAS	0.001275509945	0		2	0	https://www.ncbi.nlm.nih.gov/clinvar/variation/495725/
NM_000383.3(AIRE):c.1370G>A	concordant	unreportable	unreportable	387341	0	1	SCV000529334	Uncertain significance	GeneDx	11/28/2017	AIRE	chr21	45713763	45713763	http://gnomad.b	0.0001671057835	ASJ	0.001134249964	0		0	0	https://www.ncbi.nlm.nih.gov/clinvar/variation/387341/
NM_000053.3(ATP7B):c.4213G>A	concordant	unreportable	unreportable	444316	0	1	SCV000694465	Uncertain significance	Laboratory Corpora	1/25/2018	ATP7B	chr13	52509077	52509077	http://gnomad.b	0.0001431008552	AMR	0.0001191330011	0		1	0	https://www.ncbi.nlm.nih.gov/clinvar/variation/444316/
NM_004937.2(CTNS):c.561+4C>T	concordant	unreportable	unreportable	257156	0	2	SCV000343387;S	Uncertain significance;Unc	EGL Genetic Diagn	2017-06-30;2016-1	CTNS	chr17	3559884	3559884	http://gnomad.b	0.0002429687222	FIN	0.001973269973	0		0	0	https://www.ncbi.nlm.nih.gov/clinvar/variation/257156/
NM_000051.3(ATM):c.3353C>T	concordant	unreportable	unreportable	407537	0	1	SCV000546806	Uncertain significance	Invitae	3/14/2017	ATM	chr11	108150286	108150286	http://gnomad.b	5.57E-06	NFE	8.98E-06	0		0	0	https://www.ncbi.nlm.nih.gov/clinvar/variation/407537/
NM_000227.3(LAMA3):c.2294G>A	concordant	unreportable	unreportable	326313	0	1	SCV000407951	Uncertain significance	Illumina Clinical Se	10/18/2016	LAMA3	chr18	21489222	21489222	http://gnomad.b	0.0001651818961	SAS	0.001851849956	0		0	0	https://www.ncbi.nlm.nih.gov/clinvar/variation/326313/
NM_000243.2(MEFV):c.1341G>C	concordant	unreportable	unreportable	279849	0	1	SCV000329421	Uncertain significance	GeneDx	11/28/2017	MEFV	chr16	3298924	3298924	http://gnomad.b	0.0001315421215	NFE	0.0001163670022	0		0	0	https://www.ncbi.nlm.nih.gov/clinvar/variation/279849/
NM_000152.3(GAA):c.861C>T	concordant	unreportable	unreportable	284775	0	2	SCV000337525;S	Uncertain significance;Likel	EGL Genetic Diagn	2017-06-30;2017-1	GAA	chr17	78081601	78081601	http://gnomad.b	9.73E-05	ASJ	0.0002068680042	0		0	0	https://www.ncbi.nlm.nih.gov/clinvar/variation/284775/
NM_000053.3(ATP7B):c.347T>C	concordant	unreportable	unreportable	431956	0	1	SCV000589557	Uncertain significance	GeneDx	11/28/2017	ATP7B	chr13	52549009	52549009	http://gnomad.b	0.0001627421491	AMR	0.0002382230014	0		0	0	https://www.ncbi.nlm.nih.gov/clinvar/variation/431956/

Supplemental Table 2

NM_000152.3(GAA):c.1848C>T	concordant	unreportable	unreportable	284449	0	2	SCV000337081;S	Uncertain significance;Likel	EGL Genetic Diagn	2017-06-30;2017-1	GAA	chr17	78086470	78086470	http://gnomad.b	9.73E-05	AFR	0.0004882130015	0		0	0	https://www.ncbi.nlm.nih.gov/clinvar/variation/284449/
NM_002485.4(NBN):c.1290G>C	concordant	unreportable	unreportable	461500	0	1	SCV000634215	Uncertain significance	Invitae	10/5/2017	NBN	chr8	90967618	90967618		2.79E-06					0	0	https://www.ncbi.nlm.nih.gov/clinvar/variation/461500/
NM_018941.3(CLN8):c.50A>G	concordant	unreportable	unreportable	205201	0	1	SCV000240711	Uncertain significance	GeneDx	11/28/2017	CLN8	chr8	1719270	1719270	http://gnomad.b	5.04E-05	AFR	0.0007187660085	0		0	0	https://www.ncbi.nlm.nih.gov/clinvar/variation/205201/
NM_000023.2(SGCA):c.843C>A	concordant	unreportable	unreportable	282454	0	1	SCV000333944	Uncertain significance	EGL Genetic Diagn	6/30/2017	SGCA	chr17	48247599	48247599	http://gnomad.b	0.0001707134142	AFR	0.002548350021	0		0	0	https://www.ncbi.nlm.nih.gov/clinvar/variation/282454/
NM_000051.3(ATM):c.3806A>G	concordant	unreportable	unreportable	185981	0	3	SCV000564632;S	Uncertain significance;Unc	GeneDx;Invitae;Lal	2017-11-28;2017-1	ATM	chr11	108155013	108155013	http://gnomad.b	3.62E-05	AFR	6.54E-05	0		0	0	https://www.ncbi.nlm.nih.gov/clinvar/variation/185981/
NM_000051.3(ATM):c.3257G>A	concordant	unreportable	unreportable	234265	0	2	SCV000278819;S	Uncertain significance;Unc	GeneDx;Invitae	2017-11-28;2017-1	ATM	chr11	108143552	108143552	http://gnomad.b	1.95E-05	SAS	9.75E-05	0		0	0	https://www.ncbi.nlm.nih.gov/clinvar/variation/234265/
NM_000057.2(BLM):c.1519G>A	concordant	unreportable	unreportable	405326	0	1	SCV000543394	Uncertain significance	Invitae	10/5/2017	BLM	chr15	91304122	91304122	http://gnomad.b	4.76E-05	AMR	5.96E-05	0		0	0	https://www.ncbi.nlm.nih.gov/clinvar/variation/405326/
NM_000051.3(ATM):c.7317A>G	concordant	unreportable	unreportable	236770	0	3	SCV000682404;S	Likely benign;Likely benign	Color Genomics, In	2017-10-26;2017-1	ATM	chr11	108200950	108200950	http://gnomad.b	5.57E-06	NFE	8.98E-06	0		0	0	https://www.ncbi.nlm.nih.gov/clinvar/variation/236770/
NM_000051.3(ATM):c.3055C>G	concordant	unreportable	unreportable	216199	0	3	SCV000687440;S	Uncertain significance;Unc	Color Genomics, In	2017-12-21;2017-1	ATM	chr11	108142111	108142111		5.57E-06					0	0	https://www.ncbi.nlm.nih.gov/clinvar/variation/216199/
NM_012203.1(GRHPR):c.682A>G	concordant	unreportable	unreportable	366858	0	1	SCV000480139	Uncertain significance	Illumina Clinical Se	10/18/2016	GRHPR	chr9	37430591	37430591	http://gnomad.b	0.0001106492419	EAS	0.001739330008	0		0	0	https://www.ncbi.nlm.nih.gov/clinvar/variation/366858/
NM_000051.3(ATM):c.4402G>A	concordant	unreportable	unreportable	127389	0	3	SCV000228684;S	Uncertain significance;Unc	EGL Genetic Diagn	2017-06-30;2017-1	ATM	chr11	108160494	108160494	http://gnomad.b	8.08E-05	AFR	0.0007867820095	0		0	0	https://www.ncbi.nlm.nih.gov/clinvar/variation/127389/
NM_001360.2(DHCR7):c.723G>T	concordant	unreportable	unreportable	501363	0	1	SCV000707693	Uncertain significance	EGL Genetic Diagn	6/30/2017	DHCR7	chr11	71150033	71150033	http://gnomad.b	2.42E-05	NFE	8.06E-05	0		0	0	https://www.ncbi.nlm.nih.gov/clinvar/variation/501363/
NM_000136.2(FANCC):c.1485G>A	concordant	unreportable	unreportable	137285	0	2	SCV000345076;S	Likely benign;Benign	EGL Genetic Diagn	2017-06-30;2017-1	FANCC	chr9	97869396	97869396	http://gnomad.b	0.0005184279742	AFR	0.006078429986	0		0	0	https://www.ncbi.nlm.nih.gov/clinvar/variation/137285/
NM_138694.3(PKHD1):c.9363T>C	concordant	unreportable	unreportable	198015	0	1	SCV000231426	Uncertain significance	EGL Genetic Diagn	6/30/2017	PKHD1	chr6	51613051	51613051	http://gnomad.b	0.0003530563	AMR	0.0007748699863	0		0	0	https://www.ncbi.nlm.nih.gov/clinvar/variation/198015/
NM_002485.4(NBN):c.929T>C	concordant	unreportable	unreportable	239210	0	2	SCV000685835;S	Uncertain significance;Unc	Color Genomics, In	2017-10-26;2016-0	NBN	chr8	90976703	90976703	http://gnomad.b	1.67E-05	NFE	8.97E-06	0		0	0	https://www.ncbi.nlm.nih.gov/clinvar/variation/239210/
NM_000152.3(GAA):c.1320G>T	concordant	unreportable	unreportable	281299	0	1	SCV000332018	Uncertain significance	EGL Genetic Diagn	6/30/2017	GAA	chr17	78082621	78082621		5.28E-05					0	0	https://www.ncbi.nlm.nih.gov/clinvar/variation/281299/
NM_000642.2(AGL):c.3822C>T	concordant	unreportable	unreportable	384995	0	2	SCV000526103;S	Likely benign;Likely benign	GeneDx;Invitae	2017-11-28;2017-1	AGL	chr1	100376389	100376389	http://gnomad.b	0.0001255951816	AFR	0.001834859955	0		0	0	https://www.ncbi.nlm.nih.gov/clinvar/variation/384995/
NM_000053.3(ATP7B):c.1172C>T	concordant	unreportable	unreportable	312394	0	1	SCV000384681	Uncertain significance	Illumina Clinical Se	10/18/2016	ATP7B	chr13	52548184	52548184	http://gnomad.b	5.61E-05	SAS	0.0001624330034	0		0	0	https://www.ncbi.nlm.nih.gov/clinvar/variation/312394/
NM_004543.4(NEB):c.5747T>C	concordant	unreportable	unreportable	423299	0	2	SCV000702981;S	Uncertain significance;Unc	EGL Genetic Diagn	2017-06-30;2017-1	NEB	chr2	152520078	152520078	http://gnomad.b	0.0002323444067	AFR	0.003729880089	0		0	0	https://www.ncbi.nlm.nih.gov/clinvar/variation/423299/
NM_000232.4(SGCB):c.798C>A	concordant	unreportable	unreportable	348882	0	1	SCV000449666	Uncertain significance	Illumina Clinical Se	10/18/2016	SGCB	chr4	52890282	52890282	http://gnomad.b	1.96E-05	EAS	0.0002898889943	0		0	0	https://www.ncbi.nlm.nih.gov/clinvar/variation/348882/
NM_000492.3(CFTR):c.846A>T	concordant	unreportable	unreportable	35891	0	2	SCV000602997;S	Uncertain significance;Unc	ARUP Laboratories	2017-06-30;2017-0	CFTR	chr7	117176704	117176704	http://gnomad.b	6.55E-05	AFR	0.0005953169893	0		0	0	https://www.ncbi.nlm.nih.gov/clinvar/variation/35891/
NM_000136.2(FANCC):c.1374A>C	concordant	unreportable	unreportable	127532	0	1	SCV000149251	Uncertain significance	GeneDx	11/28/2017	FANCC	chr9	97869507	97869507	http://gnomad.b	9.27E-05	AFR	0.0007849290269	0		0	0	https://www.ncbi.nlm.nih.gov/clinvar/variation/127532/
NM_000051.3(ATM):c.7494T>C	concordant	unreportable	unreportable	181839	0	3	SCV000537460;S	Likely benign;Likely benign	Color Genomics, In	2017-01-26;2017-1	ATM	chr11	108201127	108201127	http://gnomad.b	9.19E-05	AFR	0.0001307360071	0		0	0	https://www.ncbi.nlm.nih.gov/clinvar/variation/181839/
NM_001079802.1(FKTN):c.855G>A	concordant	unreportable	unreportable	382767	0	1	SCV000522869	Likely benign	GeneDx	11/28/2017	FKTN	chr9	108377633	108377633	http://gnomad.b	1.06E-05	NFE	1.79E-05	0		0	0	https://www.ncbi.nlm.nih.gov/clinvar/variation/382767/
NM_000492.3(CFTR):c.1365G>A	concordant	unreportable	unreportable	35820	0	2	SCV000700537;S	Uncertain significance;Likel	EGL Genetic Diagn	2017-06-30;2018-0	CFTR	chr7	117188850	117188850		0.0003870923013					1	0	https://www.ncbi.nlm.nih.gov/clinvar/variation/35820/
NM_004937.2(CTNS):c.94G>A	concordant	unreportable	unreportable	322834	0	1	SCV000402208	Uncertain significance	Illumina Clinical Se	10/18/2016	CTNS	chr17	3550770	3550770	http://gnomad.b	0.0001659298591	AFR	0.00137218996	0		0	0	https://www.ncbi.nlm.nih.gov/clinvar/variation/322834/
NM_017890.4(VPS13B):c.9605T>C	concordant	unreportable	unreportable	167833	0	1	SCV000470840	Uncertain significance	Illumina Clinical Se	10/18/2016	VPS13B	chr8	100844796	100844796	http://gnomad.b	0.0001203841093	NFE	8.07E-05	0		0	0	https://www.ncbi.nlm.nih.gov/clinvar/variation/167833/
NM_000466.2(PEX1):c.2927-12T>A	concordant	unreportable	unreportable	360919	0	1	SCV000470502	Uncertain significance	Illumina Clinical Se	10/18/2016	PEX1	chr7	92123722	92123722	http://gnomad.b	7.21E-05	FIN	0.0004485909885	0		0	0	https://www.ncbi.nlm.nih.gov/clinvar/variation/360919/
NM_000051.3(ATM):c.1303T>C	concordant	unreportable	unreportable	185779	0	2	SCV000681965;S	Likely benign;Likely benign	Color Genomics, In	2017-10-26;2017-1	ATM	chr11	108121495	108121495	http://gnomad.b	7.52E-05	SAS	6.50E-05	0		0	0	https://www.ncbi.nlm.nih.gov/clinvar/variation/185779/
NM_000152.3(GAA):c.546+3G>A	concordant	unreportable	unreportable	285456	0	1	SCV000338477	Uncertain significance	EGL Genetic Diagn	6/30/2017	GAA	chr17	78078934	78078934	http://gnomad.b	0.0001056136431	NFE	7.65E-05	0		0	0	https://www.ncbi.nlm.nih.gov/clinvar/variation/285456/
NM_000051.3(ATM):c.591A>T	concordant	unreportable	unreportable	135763	0	2	SCV000682290;S	Likely benign;Likely benign	Color Genomics, In	2017-10-26;2017-1	ATM	chr11	108114774	108114774	http://gnomad.b	7.52E-05	SAS	6.50E-05	0		0	0	https://www.ncbi.nlm.nih.gov/clinvar/variation/135763/
NM_000060.2(BTD):c.1046A>C	concordant	unreportable	unreportable	25107	0	1	SCV000332685	Uncertain significance	EGL Genetic Diagn	6/30/2017	BTD	chr3	15686409	15686409	http://gnomad.b	8.65E-05	NFE	7.17E-05	0		0	0	https://www.ncbi.nlm.nih.gov/clinvar/variation/25107/
NM_000478.4(ALPL):c.1506G>A	concordant	unreportable	unreportable	295554	0	1	SCV000354316	Uncertain significance	Illumina Clinical Se	10/18/2016	ALPL	chr1	21904072	21904072	http://gnomad.b	6.20E-05	EAS	0.0008215960115	0		0	0	https://www.ncbi.nlm.nih.gov/clinvar/variation/295554/
NM_138694.3(PKHD1):c.9544G>A	concordant	unreportable	unreportable	357416	0	1	SCV000464041	Uncertain significance	Illumina Clinical Se	10/18/2016	PKHD1	chr6	51612870	51612870	http://gnomad.b	5.00E-05	SAS	0.0002923980064	0		0	0	https://www.ncbi.nlm.nih.gov/clinvar/variation/357416/
NM_001042432.1(CLN3):c.308C>T	concordant	unreportable	unreportable	318726	0	2	SCV000571272;S	Uncertain significance;Unc	GeneDx;Illumina C	2017-11-28;2016-1	CLN3	chr16	28499049	28499049		5.56E-05					0	0	https://www.ncbi.nlm.nih.gov/clinvar/variation/318726/
NM_000035.3(ALDOB):c.401G>A	concordant	unreportable	unreportable	364313	0	1	SCV000476065	Uncertain significance	Illumina Clinical Se	10/18/2016	ALDOB	chr9	104189903	104189903	http://gnomad.b	5.36E-05	EAS	0.0002898889943	0		0	0	https://www.ncbi.nlm.nih.gov/clinvar/variation/364313/
NM_000051.3(ATM):c.4921G>C	concordant	unreportable	unreportable	143016	0	3	SCV000687584;S	Uncertain significance;Unc	Color Genomics, In	2017-12-21;2017-1	ATM	chr11	108168025	108168025	http://gnomad.b	1.11E-05	NFE	1.80E-05	0		0	0	https://www.ncbi.nlm.nih.gov/clinvar/variation/143016/
NM_000051.3(ATM):c.8860T>C	concordant	unreportable	unreportable	232777	0	2	SCV000687858;S	Uncertain significance;Unc	Color Genomics, In	2017-12-21;2017-1	ATM	chr11	108235818	108235818	http://gnomad.b	5.57E-06	NFE	1.79E-05	0		0	0	https://www.ncbi.nlm.nih.gov/clinvar/variation/232777/
NM_000543.4(SMPD1):c.995C>T	concordant	unreportable	unreportable	501715	0	1	SCV000708193	Uncertain significance	EGL Genetic Diagn	6/30/2017	SMPD1	chr11	6413290	6413290	http://gnomad.b	6.42E-05	AFR	0.001176320016	0		0	0	https://www.ncbi.nlm.nih.gov/clinvar/variation/501715/
NM_000228.2(LAMB3):c.911C>T	concordant	unreportable	unreportable	295124	0	1	SCV000353651	Uncertain significance	Illumina Clinical Se	10/18/2016	LAMB3	chr1	209803992	209803992	http://gnomad.b	0.0001231796015	NFE	0.0006626070244	0		0	0	https://www.ncbi.nlm.nih.gov/clinvar/variation/295124/
NM_000182.4(HADHA):c.1788G>A	concordant	unreportable	unreportable	389019	0	1	SCV000531435	Likely benign	GeneDx	11/28/2017	HADHA	chr2	26416543	26416543	http://gnomad.b	0.0001457922125	EAS	0.0005218000151	0		0	0	https://www.ncbi.nlm.nih.gov/clinvar/variation/389019/
NM_014363.4(SACS):c.13039A>G	concordant	unreportable	unreportable	448191	0	1	SCV000614935	Uncertain significance	Athena Diagnostics	8/17/2017	SACS	chr13	23904976	23904976	http://gnomad.b	0.0001393104429	EAS	0.001043839962	0		0	0	https://www.ncbi.nlm.nih.gov/clinvar/variation/448191/
NM_000528.3(MAN2B1):c.855C>T	concordant	unreportable	unreportable	328275	0	1	SCV000410803	Uncertain significance	Illumina Clinical Se	10/18/2016	MAN2B1	chr19	12774185	12774185	http://gnomad.b	1.13E-05	EAS	5.80E-05	0		0	0	https://www.ncbi.nlm.nih.gov/clinvar/variation/328275/
NM_002225.3(IVD):c.559+1G>A	concordant	reportable	reportable	372389	1	0	SCV000490570	Pathogenic	GeneDx	12/21/2016	IVD	chr15	40703553	40703553	http://gnomad.b	1.12E-05	AFR	6.54E-05	0		0	0	https://www.ncbi.nlm.nih.gov/clinvar/variation/372389/
NM_000051.3(ATM):c.8922G>A	concordant	unreportable	unreportable	181846	0	3	SCV000682526;S	Likely benign;Likely benign	Color Genomics, In	2017-10-26;2017-1	ATM	chr11	108235880	108235880	http://gnomad.b	3.06E-05	SAS	9.75E-05	0		0	0	https://www.ncbi.nlm.nih.gov/clinvar/variation/181846/
NM_000060.2(BTD):c.*57T>A	concordant	unreportable	unreportable	343915	0	1	SCV000441837	Uncertain significance	Illumina Clinical Se	10/18/2016	BTD	chr3	15687052	15687052		0.0001816409967					0	0	https://www.ncbi.nlm.nih.gov/clinvar/variation/343915/
NM_000051.3(ATM):c.7919C>T	concordant	unreportable	unreportable	133634	0	3	SCV000209649;S	Uncertain significance;Unc	GeneDx;Invitae;Lal	2017-11-28;2017-1	ATM	chr11	108203619	108203619	http://gnomad.b	0.0001336660132	NFE	0.0002245500073	0		0	0	https://www.ncbi.nlm.nih.gov/clinvar/variation/133634/
NM_000137.2(FAH):c.841C>A	concordant	unreportable	unreportable	317214	0	1	SCV000394052	Uncertain significance	Illumina Clinical Se	10/18/2016	FAH	chr15	80467361	80467361	http://gnomad.b	0.0002163889675	AMR	0.0001786670036	1		0	0	https://www.ncbi.nlm.nih.gov/clinvar/variation/317214/
NM_017739.3(POMGNT1):c.565G>T	concordant	unreportable	unreportable	392590	0	1	SCV000535881	Uncertain significance	GeneDx	11/28/2017	POMGNT1	chr1	46660603	46660603	http://gnomad.b	2.80E-05	EAS	0.0002899900137	0		0	0	https://www.ncbi.nlm.nih.gov/clinvar/variation/392590/
NM_001164277.1(SLC37A4):c.540C>T	concordant	unreportable	unreportable	388733	0	1	SCV000531094	Likely benign	GeneDx	2/15/2017	SLC37A4	chr11	118898423	118898423	http://gnomad.b	0.0002430507054	ASJ	0.001918300055	0		0	0	https://www.ncbi.nlm.nih.gov/clinvar/variation/388733/
NM_138694.3(PKHD1):c.1256T>C	concordant	unreportable	unreportable	357451	0	1	SCV000464118	Uncertain significance	Illumina Clinical Se	10/18/2016	PKHD1	chr6	51923377	51923377	http://gnomad.b	1.39E-05	AFR	0.0001962579991	0		0	0	https://www.ncbi.nlm.nih.gov/clinvar/variation/357451/
NM_000227.3(LAMA3):c.2900A>G	concordant	unreportable	unreportable	326322	0	1	SCV000407971	Uncertain significance	Illumina Clinical Se	10/18/2016	LAMA3	chr18	21495335	21495335	http://gnomad.b	0.0001987782139	EAS	0.002610509982	0		0	0	https://www.ncbi.nlm.nih.gov/clinvar/variation/326322/
NM_024649.4(BBS1):c.6C>T	concordant	unreportable	unreportable	305456	0	1	SCV000373257	Uncertain significance	Illumina Clinical Se	10/18/2016	BBS1	chr11	66278136	66278136	http://gnomad.b	8.92E-05	FIN	0.0003599709889	0		0	0	https://www.ncbi.nlm.nih.gov/clinvar/variation/305456/
NM_001079802.1(FKTN):c.63G>C	concordant	unreportable	unreportable	285459	0	1	SCV000338481	Uncertain significance	EGL Genetic Diagn	6/30/2017	FKTN	chr9	108337376	108337376	http://gnomad.b	1.06E-05	NFE	3.58E-05	0		0	0	https://www.ncbi.nlm.nih.gov/clinvar/variation/285459/
NM_002485.4(NBN):c.1448T>C	concordant	unreportable	unreportable	230042	0	1	SCV000287452	Uncertain significance	Invitae	6/10/2016	NBN	chr8	90965869	90965869		8.37E-06					0	0	https://www.ncbi.nlm.nih.gov/clinvar/variation/230042/
NM_000227.3(LAMA3):c.1682G>C	concordant	unreportable	unreportable	326309	0	1	SCV000407944	Uncertain significance	Illumina Clinical Se	10/18/2016	LAMA3	chr18	21484552	21484552	http://gnomad.b	3.64E-05	NFE	2.80E-05	0		0	0	https://www.ncbi.nlm.nih.gov/clinvar/variation/326309/
NM_000030.2(AGXT):c.743C>T	concordant	unreportable	unreportable	383368	0	1	SCV000523747	Uncertain significance	GeneDx	11/28/2017	AGXT	chr2	241814588	241814588	http://gnomad.b	0.0001223646826	EAS	0.001275809947	1		0	0	https://www.ncbi.nlm.nih.gov/clinvar/variation/383368/
NM_000051.3(ATM):c.275A>C	concordant	unreportable	unreportable	133625	0	3	SCV000682074;S	Uncertain significance;Unc	Color Genomics, In	2017-10-26;2017-1	ATM	chr11	108099994	108099994	http://gnomad.b	7.52E-05	EAS	0.001045170007	0		0	0	https://www.ncbi.nlm.nih.gov/clinvar/variation/133625/
NM_000152.3(GAA):c.867G>A	concordant	unreportable	unreportable	385251	0	1	SCV000526454	Likely benign	GeneDx	11/28/2017	GAA	chr17	78081607	78081607	http://gnomad.b	3.34E-05	AFR	0.0001353179978	0		0	0	https://www.ncbi.nlm.nih.gov/clinvar/variation/385251/
NM_002485.4(NBN):c.930T>A	concordant	unreportable	unreportable	183861	0	4	SCV000685836;S	Likely benign;Likely benign	Color Genomics, In	2017-10-26;2017-1	NBN	chr8	90976702	90976702	http://gnomad.b	3.07E-05	NFE	7.17E-05	0		0	0	https://www.ncbi.nlm.nih.gov/clinvar/variation/183861/
NM_000391.3(TPP1):c.553C>T	concordant	unreportable	unreportable	207577	0	3	SCV000615828;S	Likely benign;Likely benign	Athena Diagnostics	2017-08-17;2017-0	TPP1	chr11	6638340	6638340	http://gnomad.b	0.0009317239411	AFR	0.01163399965	2		3	0	https://www.ncbi.nlm.nih.gov/clinvar/variation/207577/
NM_000492.3(CFTR):c.1227T>C	concordant	unreportable	unreportable	281883	0	1	SCV000332916	Uncertain significance	EGL Genetic Diagn	6/30/2017	CFTR	chr7	117188712	117188712		1.16E-05					0	0	https://www.ncbi.nlm.nih.gov/clinvar/variation/281883/
NM_003060.3(SLC22A5):c.852C>T	concordant	unreportable	unreportable	390633	0	2	SCV000533519;S	Likely benign;Likely benign	GeneDx;Invitae	2017-11-28;2017-1	SLC22A5	chr5	131722744	131722744	http://gnomad.b	0.0002654737294	NFE	0.0004117730132	0		0	0	https://www.ncbi.nlm.nih.gov/clinvar/variation/390633/
NM_000391.3(TPP1):c.542C>T	concordant	unreportable	unreportable	198075	0	4	SCV000615827;S	Uncertain significance;Unc	Athena Diagnostics	2017-08-17;2017-0	TPP1	chr11	6638351	6638351	http://gnomad.b	0.0002937868283	AFR	0.002942329971	1		0	0	https://www.ncbi.nlm.nih.gov/clinvar/variation/198075/
NM_000136.2(FANCC):c.531C>T	concordant	unreportable	unreportable	182500	0	1	SCV000253081	Likely benign	Invitae	3/14/2017	FANCC	chr9	97912360	97912360	http://gnomad.b	4.99E-05	EAS	0.000289921998	0		0	0	https://www.ncbi.nlm.nih.gov/clinvar/variation/182500/
NM_000051.3(ATM):c.3449G>C	concordant	unreportable	unreportable	127372	0	3	SCV000687480;S	Uncertain significance;Unc	Color Genomics, In	2017-12-21;2017-1	ATM	chr11	108151768	108151768	http://gnomad.b	0.0001531589734	SAS	0.001529149944	1		2	0	https://www.ncbi.nlm.nih.gov/clinvar/variation/127372/
NM_000152.3(GAA):c.32G>A	concordant	unreportable	unreportable	255362	0	2	SCV000331867;S	Uncertain significance;Unc	EGL Genetic Diagn	2017-06-30;2017-1	GAA	chr17	78078417	78078417	http://gnomad.b	0.0001278480942	AFR	0.001250489964	0		0	0	https://www.ncbi.nlm.nih.gov/clinvar/variation/255362/
NM_000182.4(HADHA):c.16G>C	concordant	unreportable	unreportable	335390	0	1	SCV000429540	Uncertain significance	Illumina Clinical Se	10/18/2016	HADHA	chr2	26467449	26467449	http://gnomad.b	0.0001738291764	NFE	0.0002951319912	0		0	0	https://www.ncbi.nlm.nih.gov/clinvar/variation/335390/
NM_000228.2(LAMB3):c.1565G>A	concordant	unreportable	unreportable	295105	0	1	SCV000353630	Uncertain significance	Illumina Clinical Se	10/18/2016	LAMB3	chr1	209800244	209800244	http://gnomad.b	0.0001595735746	AMR	0.0006554640131	0		2	0	https://www.ncbi.nlm.nih.gov/clinvar/variation/295105/
NM_004646.3(NPHS1):c.2916G>T	concordant	unreportable	unreportable	328860	0	1	SCV000411559	Uncertain significance	Illumina Clinical Se	10/18/2016	NPHS1	chr19	36330409	36330409	http://gnomad.b	5.85E-05	NFE	7.16E-05	0		0	0	https://www.ncbi.nlm.nih.gov/clinvar/variation/328860/
NM_004543.4(NEB):c.16617C>T	concordant	unreportable	unreportable	129726	0	4	SCV000614169;S	Benign;Likely benign;Uncer	Athena Diagnostics	2017-08-17;2017-1	NEB	chr2	152384079	152384079		1.29E-05					0	0	https://www.ncbi.nlm.nih.gov/clinvar/variation/129726/
NM_000243.2(MEFV):c.564C>T	concordant	unreportable	unreportable	319116	0	1	SCV000396778	Uncertain significance	Illumina Clinical Se	10/18/2016	MEFV	chr16	3304504	3304504	http://gnomad.b	9.80E-05	SAS	0.0007754230173	0		0	0	https://www.ncbi.nlm.nih.gov/clinvar/variation/319116/
NM_000383.3(AIRE):c.1066C>T	concordant	unreportable	unreportable	285876	0	1	SCV000339087	Uncertain significance	EGL Genetic Diagn	6/30/2017	AIRE	chr21	45712255	45712255	http://gnomad.b	0.0002116673258	NFE	0.000304176996	0		0	0	https://www.ncbi.nlm.nih.gov/clinvar/variation/285876/
NM_033056.3(PCDH15):c.4892C>A	concordant	unreportable	unreportable	300175	0	1	SCV000363133	Uncertain significance	Illumina Clinical Se	10/18/2016	PCDH15	chr10	55582594	55582594	http://gnomad.b	8.74E-05	NFE	0.0001880240015	0		0	0	https://www.ncbi.nlm.nih.gov/clinvar/variation/300175/
NM_002485.4(NBN):c.2071G>A	concordant	unreportable	unreportable	239192	0	1	SCV000287463	Uncertain significance	Invitae	6/10/2016	NBN	chr8	90955594	90955594	http://gnomad.b	2.79E-06	NFE	1.79E-05	0		0	0	https://www.ncbi.nlm.nih.gov/clinvar/variation/239192/
NM_000441.1(SLC26A4):c.147C>G	concordant	unreportable	unreportable	227949	0	2	SCV000343753;S	Uncertain significance;Likel	EGL Genetic Diagn	2017-06-30;2016-0	SLC26A4	chr7	107302233	107302233	http://gnomad.b	0.0002568698731	EAS	0.003976140171	0		0	0	https://www.ncbi.nlm.nih.gov/clinvar/variation/227949/
NM_000228.2(LAMB3):c.3151C>T	concordant	unreportable	unreportable	295069	0	1	SCV000353587	Uncertain significance	Illumina Clinical Se	10/18/2016	LAMB3	chr1	209790832	209790832	http://gnomad.b	3.08E-05	SAS	0.0001624330034	0		0	0	https://www.ncbi.nlm.nih.gov/clinvar/variation/295069/
NM_002485.4(NBN):c.1777C>G	concordant	unreportable	unreportable	127862	0	3	SCV000149694;S	Uncertain significance;Unc	GeneDx;Genetic Se	2017-07-13;2017-0	NBN	chr8	90965540	90965540	http://gnomad.b	0.0001701059677	AFR	0.002550020115	0		0	0	https://www.ncbi.nlm.nih.gov/clinvar/variation/127862/
NM_000492.3(CFTR):c.4027G>A	concordant	unreportable	unreportable	495939	0	1	SCV000697001	Uncertain significance	Laboratory Corpora	1/25/2018	CFTR	chr7	117304805	117304805	http://gnomad.b	2.31E-05	NFE	8.97E-05	0		0	0	https://www.ncbi.nlm.nih.gov/clinvar/variation/495939/
NM_138694.3(PKHD1):c.8554+3A>G	concordant	unreportable	unreportable	499528	0	1	SCV000705053	Uncertain significance	EGL Genetic Diagn	6/30/2017	PKHD1	chr6	51640603	51640603	http://gnomad.b	0.000186258048	AFR	0.0003966680088	0		0	0	https://www.ncbi.nlm.nih.gov/clinvar/variation/499528/
NM_000051.3(ATM):c.2934T>G	concordant	unreportable	unreportable	184551	0	2	SCV000512150;S	Likely benign;Likely benign	GeneDx;Invitae	2017-11-28;2017-0	ATM	chr11	108141990	108141990	http://gnomad.b	1.11E-05	NFE	3.58E-05	0		0	0	https://www.ncbi.nlm.nih.gov/clinvar/variation/184551/
NM_000057.2(BLM):c.1194C>T	concordant	unreportable	unreportable	236800	0	1	SCV000283110	Likely benign	Invitae	10/5/2017	BLM	chr15	91303483	91303483	http://gnomad.b	3.57E-05	EAS	0.0002319380001	0		0	0	https://www.ncbi.nlm.nih.gov/clinvar/variation/236800/
NM_000492.3(CFTR):c.1210-15G>T	concordant	unreportable	unreportable	502598	0	1	SCV000709401	Uncertain significance	EGL Genetic Diagn	6/30/2017	CFTR	chr7	117188680	117188680		5.78E-06					0	0	https://www.ncbi.nlm.nih.gov/clinvar/variation/502598/
NM_002485.4(NBN):c.788T>C	concordant	unreportable	unreportable	127880	0	3	SCV000149715;S	Uncertain significance;Unc	GeneDx;Genetic Se	2017-07-13;2017-0	NBN	chr8	90982700	90982700	http://gnomad.b	0.0001701059677	AFR	0.002548689954	0		0	0	https://www.ncbi.nlm.nih.gov/clinvar/variation/127880/
NM_000642.2(AGL):c.968G>A	concordant	unreportable	unreportable	456516	0	1	SCV000626772	Likely benign	Invitae	10/5/2017	AGL	chr1	100340252	100340252	http://gnomad.b	0.0003963225731	AFR	0.003602310084	0		2	0	https://www.ncbi.nlm.nih.gov/clinvar/variation/456516/
NM_138694.3(PKHD1):c.7301C>T	concordant	unreportable	unreportable	357430	0	1	SCV000464066	Uncertain significance	Illumina Clinical Se	10/18/2016	PKHD1	chr6	51747940	51747940		4.73E-05					0	0	https://www.ncbi.nlm.nih.gov/clinvar/variation/357430/
NM_000051.3(ATM):c.401G>A	concordant	unreportable	unreportable	186081	0	2	SCV000682176;S	Uncertain significance;Unc	Color Genomics, In	2017-10-26;2017-1	ATM	chr11	108106466	108106466	http://gnomad.b	8.35E-06	AFR	6.54E-05	0		0	0	https://www.ncbi.nlm.nih.gov/clinvar/variation/186081/
NM_000051.3(ATM):c.7740A>C	concordant	unreportable	unreportable	127448	0	3	SCV000149163;S	Uncertain significance;Unc	GeneDx;Invitae;Lal	2017-11-28;2017-1	ATM	chr11	108202716	108202716	http://gnomad.b	0.0002172072714	AMR	0.0002978320117	0		1	0	https://www.ncbi.nlm.nih.gov/clinvar/variation/127448/
NM_138694.3(PKHD1):c.4871G>A	concordant	unreportable	unreportable	502043	0	1	SCV000708635	Uncertain significance	EGL Genetic Diagn	6/30/2017	PKHD1	chr6	51889737	51889737	http://gnomad.b	0.0001528983976	AFR	0.002025610069	0		0	0	https://www.ncbi.nlm.nih.gov/clinvar/variation/502043/
NM_001173990.2(TMEM216):c.277G>	concordant	unreportable	unreportable	289040	0	1	SCV000343312	Uncertain significance	EGL Genetic Diagn	6/30/2017	TMEM216	chr11	61165293	61165293	http://gnomad.b	5.91E-05	AMR	0.0003871119989	0		0	0	https://www.ncbi.nlm.nih.gov/clinvar/variation/289040/
NM_000051.3(ATM):c.7593G>A	concordant	unreportable	unreportable	187478	0	1	SCV000292483	Uncertain significance	GeneDx	11/28/2017	ATM	chr11	108202248	108202248		8.35E-06					0	0	https://www.ncbi.nlm.nih.gov/clinvar/variation/187478/
NM_000051.3(ATM):c.3256C>T	concordant	unreportable	unreportable	234020	0	1	SCV000546897	Uncertain significance	Invitae	10/5/2017	ATM	chr11	108143551	108143551	http://gnomad.b	2.51E-05	AFR	6.54E-05	0		0	0	https://www.ncbi.nlm.nih.gov/clinvar/variation/234020/
NM_138694.3(PKHD1):c.400G>A	concordant	unreportable	unreportable	198277	0	1	SCV000464127	Uncertain significance	Illumina Clinical Se	10/18/2016	PKHD1	chr6	51941122	51941122	http://gnomad.b	0.000125098689	SAS	0.0002923980064	0		2	0	https://www.ncbi.nlm.nih.gov/clinvar/variation/198277/
NM_000017.2(ACADS):c.1105G>A	concordant	unreportable	unreportable	203561	0	1	SCV000238616	Uncertain significance	GeneDx	11/28/2017	ACADS	chr12	121177117	121177117	http://gnomad.b	2.83E-05	EAS	0.0001165499998	0		0	0	https://www.ncbi.nlm.nih.gov/clinvar/variation/203561/
NM_000051.3(ATM):c.7516A>G	concordant	unreportable	unreportable	236775	0	2	SCV000292894;S	Uncertain significance;Unc	GeneDx;Invitae	2017-11-28;2017-1	ATM	chr11	108202171	108202171	http://gnomad.b	2.23E-05	AFR	0.0003276540083	0		0	0	https://www.ncbi.nlm.nih.gov/clinvar/variation/236775/
NM_001164277.1(SLC37A4):c.1240C>	concordant	unreportable	unreportable	288044	0	1	SCV000342034	Uncertain significance	EGL Genetic Diagn	6/30/2017	SLC37A4	chr11	118895670	118895670	http://gnomad.b	0.0003296549797	NFE	0.000474630011	0		0	0	https://www.ncbi.nlm.nih.gov/clinvar/variation/288044/
NM_000057.2(BLM):c.3609G>A	concordant	unreportable	unreportable	413287	0	1	SCV000555821	Likely benign	Invitae	3/14/2017	BLM	chr15	91347447	91347447	http://gnomad.b	2.14E-05	AMR	2.98E-05	0		0	0	https://www.ncbi.nlm.nih.gov/clinvar/variation/413287/

Supplemental Table 2

NM_000057.2(BLM):c.1086C>T	concordant	unreportable	unreportable	236799	0	2	SCV000336210;S(	Uncertain significance;Likel	EGL Genetic Diagn(	2017-06-30;2017-1	BLM	chr15	91298167	91298167	http://gnomad.b	0.0001141606812	NFE	0.0001441600034	0		0	0	https://www.ncbi.nlm.nih.gov/clinvar/variation/236799/
NM_001173990.2(TMEM216):c.289T>	concordant	unreportable	unreportable	305078	0	1	SCV000372633	Uncertain significance	Illumina Clinical Se	10/18/2016	TMEM216	chr11	61165305	61165305	http://gnomad.b	0.0004936417909	FIN	0.001210759976	0		0	0	https://www.ncbi.nlm.nih.gov/clinvar/variation/305078/
NM_138694.3(PKHD1):c.4876A>G	concordant	unreportable	unreportable	284552	0	1	SCV000337214	Uncertain significance	EGL Genetic Diagn(	6/30/2017	PKHD1	chr6	51889732	51889732	http://gnomad.b	3.06E-05	AMR	2.98E-05	0		0	0	https://www.ncbi.nlm.nih.gov/clinvar/variation/284552/
NM_000051.3(ATM):c.2944C>T	concordant	unreportable	unreportable	127363	0	3	SCV000682091;S(	Uncertain significance;Unc(	Color Genomics, In	2017-10-26;2017-1	ATM	chr11	108142000	108142000	http://gnomad.b	2.23E-05	SAS	6.51E-05	0		0	0	https://www.ncbi.nlm.nih.gov/clinvar/variation/127363/
NM_014363.4(SACS):c.2926C>A	concordant	unreportable	unreportable	448199	0	1	SCV000614947	Uncertain significance	Athena Diagnostics	8/17/2017	SACS	chr13	23915089	23915089	http://gnomad.b	0.0003349378735	AFR	0.004379080143	0		0	0	https://www.ncbi.nlm.nih.gov/clinvar/variation/448199/
NM_001271208.1(NEB):c.11627G>A	concordant	reportable	reportable	450119	2	0	SCV000618662;S(	Pathogenic;Pathogenic	GeneDx;Invitae	2017-11-28;2017-1	NEB	chr2	152468878	152468878	http://gnomad.b	7.74E-06	AMR	0.000119352997	0		0	0	https://www.ncbi.nlm.nih.gov/clinvar/variation/450119/
NM_000228.2(LAMB3):c.1868G>A	concordant	unreportable	unreportable	295097	0	1	SCV000353620	Uncertain significance	Illumina Clinical Se	10/18/2016	LAMB3	chr1	209799101	209799101	http://gnomad.b	0.0002911517853	NFE	0.0003672390012	0		0	0	https://www.ncbi.nlm.nih.gov/clinvar/variation/295097/
NM_000035.3(ALDOB):c.522C>T	concordant	unreportable	unreportable	390695	0	1	SCV000533593	Likely benign	GeneDx	11/28/2017	ALDOB	chr9	104189782	104189782	http://gnomad.b	2.54E-05	ASJ	0.000101605001	0		0	0	https://www.ncbi.nlm.nih.gov/clinvar/variation/390695/
NM_003640.3(IKBKAP):c.2052T>A	concordant	unreportable	unreportable	455957	0	1	SCV000626009	Uncertain significance	Invitae	10/5/2017	IKBKAP	chr9	111662618	111662618	http://gnomad.b	7.10E-06	AMR	2.98E-05	0		0	0	https://www.ncbi.nlm.nih.gov/clinvar/variation/455957/
NM_033056.3(PCDH15):c.2290C>T	concordant	unreportable	unreportable	164919	0	1	SCV000199857	Uncertain significance	Laboratory for Mol	4/26/2016	PCDH15	chr10	55782888	55782888	http://gnomad.b	0.0004346037134	NFE	0.0006470630178	1		0	0	https://www.ncbi.nlm.nih.gov/clinvar/variation/164919/
NM_000152.3(GAA):c.705G>A	concordant	unreportable	unreportable	282633	0	2	SCV000334193;S(	Uncertain significance;Unc(	EGL Genetic Diagn(	2017-06-30;2016-1	GAA	chr17	78081368	78081368	http://gnomad.b	0.0001389653198	EAS	0.002030869946	0		0	0	https://www.ncbi.nlm.nih.gov/clinvar/variation/282633/
NM_000136.2(FANCC):c.817G>A	concordant	unreportable	unreportable	182480	0	2	SCV000211046;S(	Uncertain significance;Likel	GeneDx;Invitae	2017-11-28;2017-1	FANCC	chr9	97897654	97897654	http://gnomad.b	0.0001355522685	NFE	0.0002239019959	0		0	0	https://www.ncbi.nlm.nih.gov/clinvar/variation/182480/
NM_000352.3(ABCC8):c.2422C>A	concordant	unreportable	unreportable	303773	0	1	SCV000369327	Likely benign	Illumina Clinical Se	10/18/2016	ABCC8	chr11	17434994	17434994	http://gnomad.b	6.97E-05	AMR	0.0001488889975	0		0	0	https://www.ncbi.nlm.nih.gov/clinvar/variation/303773/
NM_000228.2(LAMB3):c.3016C>T	concordant	unreportable	unreportable	295072	0	1	SCV000353590	Uncertain significance	Illumina Clinical Se	10/18/2016	LAMB3	chr1	209791287	209791287	http://gnomad.b	0.0001539745018	FIN	0.0002690579859	0		0	0	https://www.ncbi.nlm.nih.gov/clinvar/variation/295072/
NM_000023.2(SGCA):c.34G>A	concordant	unreportable	unreportable	286013	0	1	SCV000339266	Uncertain significance	EGL Genetic Diagn(	6/30/2017	SGCA	chr17	48243435	48243435	http://gnomad.b	8.68E-05	AMR	0.0005661160103	0		0	0	https://www.ncbi.nlm.nih.gov/clinvar/variation/286013/
NM_000466.2(PEX1):c.2021C>T	concordant	unreportable	unreportable	290970	0	1	SCV000345640	Uncertain significance	EGL Genetic Diagn(	6/30/2017	PEX1	chr7	92134096	92134096	http://gnomad.b	9.98E-05	ASJ	0.0001016469978	0		0	0	https://www.ncbi.nlm.nih.gov/clinvar/variation/290970/
NM_000227.3(LAMA3):c.3829C>T	concordant	unreportable	unreportable	326336	0	1	SCV000408005	Uncertain significance	Illumina Clinical Se	10/18/2016	LAMA3	chr18	21512203	21512203	http://gnomad.b	0.0002491726907	AMR	0.0004467210092	0		0	0	https://www.ncbi.nlm.nih.gov/clinvar/variation/326336/
NM_000360.3(TH):c.1170C>G	concordant	unreportable	unreportable	304070	0	1	SCV000369911	Uncertain significance	Illumina Clinical Se	10/18/2016	TH	chr11	2186928	2186928	http://gnomad.b	0.0002986066899	NFE	0.000513912004	0		0	0	https://www.ncbi.nlm.nih.gov/clinvar/variation/304070/
NM_000098.2(CPT2):c.985G>T	concordant	unreportable	unreportable	460438	0	1	SCV000632618	Uncertain significance	Invitae	10/5/2017	CPT2	chr1	53676331	53676331	http://gnomad.b	5.88E-05	NFE	8.97E-05	0		0	0	https://www.ncbi.nlm.nih.gov/clinvar/variation/460438/
NM_000492.3(CFTR):c.1231A>G	concordant	unreportable	unreportable	502578	0	1	SCV000709377	Uncertain significance	EGL Genetic Diagn(	6/30/2017	CFTR	chr7	117188716	117188716		6.36E-05					0	0	https://www.ncbi.nlm.nih.gov/clinvar/variation/502578/
NM_017739.3(POMGNT1):c.1825C>T	concordant	unreportable	unreportable	286266	0	1	SCV000339624	Uncertain significance	EGL Genetic Diagn(	6/30/2017	POMGNT1	chr1	46655200	46655200	http://gnomad.b	4.20E-05	AMR	5.98E-05	0		0	0	https://www.ncbi.nlm.nih.gov/clinvar/variation/286266/
NM_000182.4(HADHA):c.809C>T	concordant	unreportable	unreportable	468768	0	1	SCV000645776	Uncertain significance	Invitae	10/5/2017	HADHA	chr2	26437421	26437421	http://gnomad.b	0.0002383141934	AFR	0.00137273001	0		0	0	https://www.ncbi.nlm.nih.gov/clinvar/variation/468768/
NM_000182.4(HADHA):c.585T>A	concordant	unreportable	unreportable	335385	0	1	SCV000429528	Uncertain significance	Illumina Clinical Se	10/18/2016	HADHA	chr2	26453151	26453151	http://gnomad.b	0.0002299031043	EAS	0.00179730996	0		0	0	https://www.ncbi.nlm.nih.gov/clinvar/variation/335385/
NM_000051.3(ATM):c.118A>G	concordant	unreportable	unreportable	422792	0	2	SCV000572354;S(	Uncertain significance;Unc(	GeneDx;Invitae	2017-11-28;2017-1	ATM	chr11	108098548	108098548		5.57E-06					0	0	https://www.ncbi.nlm.nih.gov/clinvar/variation/422792/
NM_000051.3(ATM):c.4050G>A	concordant	unreportable	unreportable	186770	0	2	SCV000682181;S(	Likely benign;Likely benign	Color Genomics, In	2017-10-26;2017-1	ATM	chr11	108158383	108158383	http://gnomad.b	4.18E-05	FIN	4.51E-05	0		0	0	https://www.ncbi.nlm.nih.gov/clinvar/variation/186770/
NM_017739.3(POMGNT1):c.355G>A	concordant	unreportable	unreportable	423754	0	1	SCV000573489	Uncertain significance	GeneDx	11/28/2017	POMGNT1	chr1	46661749	46661749	http://gnomad.b	0.0001203814131	NFE	0.0001972600003	0		0	0	https://www.ncbi.nlm.nih.gov/clinvar/variation/423754/
NM_000071.2(CBS):c.134G>A	concordant	unreportable	unreportable	236935	0	1	SCV000283382	Uncertain significance	Invitae	6/10/2016	CBS	chr21	44492170	44492170	http://gnomad.b	5.62E-05	SAS	0.0002274059952	0		0	0	https://www.ncbi.nlm.nih.gov/clinvar/variation/236935/
NM_000642.2(AGL):c.4027G>A	concordant	unreportable	unreportable	456499	0	2	SCV000700584;S(	Likely benign;Benign	EGL Genetic Diagn(	2017-06-30;2017-1	AGL	chr1	100379160	100379160	http://gnomad.b	0.000856838239	EAS	0.006728540175	1		2	0	https://www.ncbi.nlm.nih.gov/clinvar/variation/456499/
NM_000057.2(BLM):c.2263A>G	concordant	unreportable	unreportable	133697	0	2	SCV000593644;S(	Likely benign;Likely benign	Genetic Services La	2017-07-05;2017-1	BLM	chr15	91310209	91310209	http://gnomad.b	0.000439994292	AFR	0.004126819782	0		0	0	https://www.ncbi.nlm.nih.gov/clinvar/variation/133697/
NM_000136.2(FANCC):c.609C>T	concordant	unreportable	unreportable	219714	0	2	SCV000520430;S(	Likely benign;Likely benign	GeneDx;Invitae	2017-11-28;2017-1	FANCC	chr9	97912282	97912282	http://gnomad.b	6.18E-05	SAS	0.0001955159969	0		0	0	https://www.ncbi.nlm.nih.gov/clinvar/variation/219714/
NM_017890.4(VPS13B):c.7226C>T	concordant	unreportable	unreportable	212580	0	1	SCV000249417	Uncertain significance	Genetic Services La	9/15/2015	VPS13B	chr8	100779102	100779102	http://gnomad.b	5.32E-05	NFE	0.0001435799932	0		0	0	https://www.ncbi.nlm.nih.gov/clinvar/variation/212580/
NM_000051.3(ATM):c.7927+13dupT	concordant	unreportable	unreportable	140857	0	2	SCV000682443;S(	Likely benign;Benign	Color Genomics, In	2017-10-26;2017-1	ATM	chr11	108203632	108203632	http://gnomad.b	0.0001364507218	FIN	0.0001974329934	0		0	0	https://www.ncbi.nlm.nih.gov/clinvar/variation/140857/
NM_000492.3(CFTR):c.3429G>A	concordant	unreportable	unreportable	439077	0	1	SCV000601099	Uncertain significance	Quest Diagnostics I	8/1/2017	CFTR	chr7	117254728	117254728	http://gnomad.b	9.05E-05	NFE	0.000179552997	0		0	0	https://www.ncbi.nlm.nih.gov/clinvar/variation/439077/
NM_000051.3(ATM):c.1044G>A	concordant	unreportable	unreportable	481275	0	1	SCV000687283	Likely benign	Color Genomics, In	12/21/2017	ATM	chr11	108117833	108117833	http://gnomad.b	8.35E-06	NFE	8.97E-06	0		0	0	https://www.ncbi.nlm.nih.gov/clinvar/variation/481275/
NM_002485.4(NBN):c.505C>T	concordant	unreportable	unreportable	127873	0	5	SCV000685792;S(	Uncertain significance;Unc(	Color Genomics, In	2017-10-26;2017-1	NBN	chr8	90990527	90990527	http://gnomad.b	0.0001477969883	EAS	0.0004058440099	0		0	0	https://www.ncbi.nlm.nih.gov/clinvar/variation/127873/
NM_000352.3(ABCC8):c.3293G>A	concordant	unreportable	unreportable	388502	0	1	SCV000530817	Uncertain significance	GeneDx	11/28/2017	ABCC8	chr11	17428205	17428205	http://gnomad.b	1.03E-05	EAS	0.000115969	0		0	0	https://www.ncbi.nlm.nih.gov/clinvar/variation/388502/
NM_005562.2(LAMC2):c.69C>T	concordant	unreportable	unreportable	293985	0	1	SCV000351648	Uncertain significance	Illumina Clinical Se	10/18/2016	LAMC2	chr1	183155556	183155556	http://gnomad.b	2.80E-05	NFE	4.71E-05	0		0	0	https://www.ncbi.nlm.nih.gov/clinvar/variation/293985/
NM_005476.5(GNE):c.1651A>G	concordant	unreportable	unreportable	288664	0	1	SCV000342836	Uncertain significance	EGL Genetic Diagn(	11/3/2016	GNE	chr9	36220000	36220000	http://gnomad.b	1.68E-05	AFR	0.0002613700053	0		0	0	https://www.ncbi.nlm.nih.gov/clinvar/variation/288664/
NM_000271.4(NPC1):c.2524T>C	concordant	unreportable	unreportable	499258	0	1	SCV000704660	Uncertain significance	EGL Genetic Diagn(	6/30/2017	NPC1	chr18	21120492	21120492	http://gnomad.b	8.58E-05	AMR	0.000178731003	0		0	0	https://www.ncbi.nlm.nih.gov/clinvar/variation/499258/
NM_004646.3(NPHS1):c.3419G>A	concordant	unreportable	unreportable	328853	0	1	SCV000411549	Uncertain significance	Illumina Clinical Se	10/18/2016	NPHS1	chr19	36322017	36322017	http://gnomad.b	0.00017840318	NFE	0.000331660005	0		0	0	https://www.ncbi.nlm.nih.gov/clinvar/variation/328853/
NM_000153.3(GALC):c.349A>G	concordant	unreportable	unreportable	92504	0	2	SCV000110039;S(	Uncertain significance;Unc(	EGL Genetic Diagn(	2017-06-30;2017-1	GALC	chr14	88452926	88452926	http://gnomad.b	0.0001174772598	AFR	0.00117738999	0		0	0	https://www.ncbi.nlm.nih.gov/clinvar/variation/92504/
NM_000053.3(ATP7B):c.1291T>C	concordant	unreportable	unreportable	495399	0	1	SCV000694397	Uncertain significance	Laboratory Corpor	1/25/2018	ATP7B	chr13	52544880	52544880	http://gnomad.b	0.0001851893421	AFR	0.001112999977	0		0	0	https://www.ncbi.nlm.nih.gov/clinvar/variation/495399/
NM_000414.3(HSD17B4):c.1262G>A	concordant	unreportable	unreportable	502349	0	1	SCV000709042	Uncertain significance	EGL Genetic Diagn(	6/30/2017	HSD17B4	chr5	118842513	118842513	http://gnomad.b	2.54E-05	AFR	0.00032968499	0		0	0	https://www.ncbi.nlm.nih.gov/clinvar/variation/502349/
NM_005562.2(LAMC2):c.509G>T	concordant	unreportable	unreportable	293994	0	1	SCV000351661	Uncertain significance	Illumina Clinical Se	10/18/2016	LAMC2	chr1	183189965	183189965	http://gnomad.b	0.0002603714633	AFR	0.003006540006	0		0	0	https://www.ncbi.nlm.nih.gov/clinvar/variation/293994/
NM_133647.1(SLC12A6):c.1012C>T	concordant	unreportable	unreportable	315618	0	1	SCV000390318	Uncertain significance	Illumina Clinical Se	10/18/2016	SLC12A6	chr15	34546655	34546655	http://gnomad.b	0.0005748897634	EAS	0.00295686	1		1	0	https://www.ncbi.nlm.nih.gov/clinvar/variation/315618/
NM_014363.4(SACS):c.593A>G	concordant	unreportable	unreportable	448209	0	1	SCV000614964	Uncertain significance	Athena Diagnostics	8/17/2017	SACS	chr13	23932485	23932485		1.78E-05					0	0	https://www.ncbi.nlm.nih.gov/clinvar/variation/448209/
NM_000071.2(CBS):c.1072G>A	concordant	unreportable	unreportable	193971	0	2	SCV000249707;S(	Uncertain significance;Unc(	GeneDx;Invitae	2017-11-28;2017-1	CBS	chr21	44480624	44480624	http://gnomad.b	0.0001265402764	NFE	0.0001851169945	0		0	0	https://www.ncbi.nlm.nih.gov/clinvar/variation/193971/
NM_004937.2(CTNS):c.332C>T	concordant	unreportable	unreportable	322837	0	1	SCV000402215	Uncertain significance	Illumina Clinical Se	10/18/2016	CTNS	chr17	3558517	3558517	http://gnomad.b	0.0001244473943	NFE	0.000250631012	0		0	0	https://www.ncbi.nlm.nih.gov/clinvar/variation/322837/
NM_000310.3(PPT1):c.676G>A	concordant	unreportable	unreportable	297245	0	2	SCV000705385;S(	Uncertain significance;Unc(	EGL Genetic Diagn(	2017-06-30;2016-1	PPT1	chr1	40544282	40544282	http://gnomad.b	2.51E-05	AMR	0.0005062239943	0		0	0	https://www.ncbi.nlm.nih.gov/clinvar/variation/297245/
NM_000492.3(CFTR):c.4092G>A	concordant	unreportable	unreportable	35883	0	1	SCV000625749	Likely benign	Invitae	10/5/2017	CFTR	chr7	117304870	117304870	http://gnomad.b	7.51E-05	AFR	0.0001307189959	0		0	0	https://www.ncbi.nlm.nih.gov/clinvar/variation/35883/
NM_000414.3(HSD17B4):c.643C>T	concordant	unreportable	unreportable	228743	0	1	SCV000271842	Uncertain significance	Laboratory for Mol	4/26/2016	HSD17B4	chr5	118824907	118824907	http://gnomad.b	3.38E-05	SAS	0.0002274199942	0		0	0	https://www.ncbi.nlm.nih.gov/clinvar/variation/228743/
NM_000152.3(GAA):c.913G>A	concordant	unreportable	unreportable	288431	0	1	SCV000342539	Uncertain significance	EGL Genetic Diagn(	6/30/2017	GAA	chr17	78081653	78081653	http://gnomad.b	0.0001389653198	FIN	0.000882162014	0		0	0	https://www.ncbi.nlm.nih.gov/clinvar/variation/288431/
NM_000288.3(PEX7):c.188+3A>G	concordant	unreportable	unreportable	355529	0	2	SCV000705198;S(	Uncertain significance;Unc(	EGL Genetic Diagn(	2017-06-30;2016-1	PEX7	chr6	137146412	137146412	http://gnomad.b	0.0002166738891	AMR	0.0003871119989	0		0	0	https://www.ncbi.nlm.nih.gov/clinvar/variation/355529/
NM_017739.3(POMGNT1):c.1539C>T	concordant	unreportable	unreportable	291174	0	1	SCV000345881	Uncertain significance	EGL Genetic Diagn(	6/30/2017	POMGNT1	chr1	46657770	46657770	http://gnomad.b	3.92E-05	AFR	0.0001960780064	0		0	0	https://www.ncbi.nlm.nih.gov/clinvar/variation/291174/
NM_000492.3(CFTR):c.-448A>G	concordant	unreportable	unreportable	439085	0	1	SCV000601123	Uncertain significance	Quest Diagnostics I	8/1/2017	CFTR	chr7	117119701	117119701		0.0001810282404					0	0	https://www.ncbi.nlm.nih.gov/clinvar/variation/439085/
NM_000051.3(ATM):c.3352A>G	concordant	unreportable	unreportable	181948	0	3	SCV000209727;S(	Uncertain significance;Unc(	GeneDx;Illumina C	2017-11-28;2016-1	ATM	chr11	108150285	108150285	http://gnomad.b	9.47E-05	SAS	0.001624749973	0		0	0	https://www.ncbi.nlm.nih.gov/clinvar/variation/181948/
NM_017890.4(VPS13B):c.365C>T	concordant	unreportable	unreportable	361036	0	2	SCV000597871;S(	Uncertain significance;Unc(	Genetic Services La	2017-07-05;2016-1	VPS13B	chr8	100108613	100108613	http://gnomad.b	0.0001035863266	FIN	0.001345289988	1		0	0	https://www.ncbi.nlm.nih.gov/clinvar/variation/361036/
NM_001042432.1(CLN3):c.533+18G>A	concordant	unreportable	unreportable	389859	0	1	SCV000532526	Likely benign	GeneDx	11/28/2017	CLN3	chr16	28497881	28497881	http://gnomad.b	1.39E-05	NFE	9.46E-05	0		0	0	https://www.ncbi.nlm.nih.gov/clinvar/variation/389859/
NM_000053.3(ATP7B):c.1415C>T	concordant	unreportable	unreportable	495400	0	1	SCV000694398	Uncertain significance	Laboratory Corpor	1/25/2018	ATP7B	chr13	52544756	52544756	http://gnomad.b	2.81E-05	AFR	0.0001307700004	0		0	0	https://www.ncbi.nlm.nih.gov/clinvar/variation/495400/
NM_000051.3(ATM):c.646G>T	concordant	unreportable	unreportable	141750	0	3	SCV000682341;S(	Uncertain significance;Unc(	Color Genomics, In	2017-10-26;2017-1	ATM	chr11	108114829	108114829	http://gnomad.b	1.95E-05	AFR	6.54E-05	0		0	0	https://www.ncbi.nlm.nih.gov/clinvar/variation/141750/
NM_017739.3(POMGNT1):c.1813C>T	concordant	unreportable	unreportable	290842	0	1	SCV000345491	Uncertain significance	EGL Genetic Diagn(	6/30/2017	POMGNT1	chr1	46655212	46655212		1.12E-05					0	0	https://www.ncbi.nlm.nih.gov/clinvar/variation/290842/
NM_000227.3(LAMA3):c.4114C>T	concordant	reportable	reportable	449049	1	0	SCV000616762	Pathogenic	GeneDx	11/28/2017	LAMA3	chr18	21519265	21519265	http://gnomad.b	5.60E-06	NFE	2.69E-05	0		0	0	https://www.ncbi.nlm.nih.gov/clinvar/variation/449049/
NM_000071.2(CBS):c.1594G>A	concordant	unreportable	unreportable	501401	0	1	SCV000707749	Uncertain significance	EGL Genetic Diagn(	6/30/2017	CBS	chr21	44474052	44474052	http://gnomad.b	0.0001040442272	EAS	0.001160499989	0		0	0	https://www.ncbi.nlm.nih.gov/clinvar/variation/501401/
NM_002485.4(NBN):c.949A>G	concordant	unreportable	unreportable	142497	0	2	SCV000565990;S(	Uncertain significance;Unc(	GeneDx;Invitae	2017-11-28;2017-1	NBN	chr8	90976683	90976683	http://gnomad.b	7.53E-05	NFE	2.69E-05	0		0	0	https://www.ncbi.nlm.nih.gov/clinvar/variation/142497/
NM_000060.2(BTD):c.1629C>A	concordant	reportable	reportable	25103	1	0	SCV000630332	Pathogenic	Invitae	10/5/2017	BTD	chr3	15686992	15686992	http://gnomad.b	3.75E-05	AFR	0.0007143879775	0		0	0	https://www.ncbi.nlm.nih.gov/clinvar/variation/25103/
NM_138694.3(PKHD1):c.5829C>T	concordant	unreportable	unreportable	357435	0	2	SCV000702743;S(	Uncertain significance;Unc(	EGL Genetic Diagn(	2017-06-30;2016-1	PKHD1	chr6	51824747	51824747	http://gnomad.b	0.0003975358338	EAS	0.000985849998	0		1	0	https://www.ncbi.nlm.nih.gov/clinvar/variation/357435/
NM_000051.3(ATM):c.3402+3A>G	concordant	unreportable	unreportable	187380	0	1	SCV000687471	Uncertain significance	Color Genomics, In	12/21/2017	ATM	chr11	108150338	108150338	http://gnomad.b	1.39E-05	NFE	9.01E-06	0		0	0	https://www.ncbi.nlm.nih.gov/clinvar/variation/187380/
NM_033056.3(PCDH15):c.5864T>C	concordant	unreportable	unreportable	300165	0	1	SCV000363096	Uncertain significance	Illumina Clinical Se	10/18/2016	PCDH15	chr10	55581622	55581622	http://gnomad.b	1.80E-05	NFE	2.69E-05	0		0	0	https://www.ncbi.nlm.nih.gov/clinvar/variation/300165/
NM_000492.3(CFTR):c.1437G>C	concordant	unreportable	unreportable	439478	0	1	SCV000603010	Uncertain significance	ARUP Laboratories	6/30/2017	CFTR	chr7	117199562	117199562	http://gnomad.b	3.27E-05	AMR	0.0003573340073	0		0	0	https://www.ncbi.nlm.nih.gov/clinvar/variation/439478/
NM_133647.1(SLC12A6):c.320_322del	concordant	unreportable	unreportable	432227	0	1	SCV000589931	Uncertain significance	GeneDx	11/28/2017	SLC12A6	chr15	34553215	34553218	http://gnomad.b	4.15E-05	NFE	7.19E-05	0		0	0	https://www.ncbi.nlm.nih.gov/clinvar/variation/432227/
NM_002435.1(MPI):c.132G>A	concordant	unreportable	unreportable	317137	0	1	SCV000393940	Uncertain significance	Illumina Clinical Se	10/18/2016	MPI	chr15	75182983	75182983	http://gnomad.b	3.08E-05	EAS	0.0006378290127	0		0	0	https://www.ncbi.nlm.nih.gov/clinvar/variation/317137/
NM_000466.2(PEX1):c.2645C>T	concordant	unreportable	unreportable	194604	0	1	SCV000226433	Uncertain significance	EGL Genetic Diagn(	6/30/2017	PEX1	chr7	92129091	92129091	http://gnomad.b	0.0001858210238	AFR	0.00248365989	0		0	0	https://www.ncbi.nlm.nih.gov/clinvar/variation/194604/
NM_000136.2(FANCC):c.1095C>T	concordant	unreportable	unreportable	384287	0	1	SCV000525023	Likely benign	GeneDx	11/28/2017	FANCC	chr9	97876970	97876970		2.38E-06					0	0	https://www.ncbi.nlm.nih.gov/clinvar/variation/384287/
NM_000441.1(SLC26A4):c.872G>A	concordant	unreportable	unreportable	358496	0	1	SCV000466086	Uncertain significance	Illumina Clinical Se	10/18/2016	SLC26A4	chr7	107323753	107323753	http://gnomad.b	0.0001144746173	AFR	0.0005883889971	0		0	0	https://www.ncbi.nlm.nih.gov/clinvar/variation/358496/
NM_000071.2(CBS):c.600G>A	concordant	unreportable	unreportable	340087	0	1	SCV000436224	Uncertain significance	Illumina Clinical Se	10/18/2016	CBS	chr21	44485563	44485563	http://gnomad.b	5.06E-05	FIN	0.002512559993	0		0	0	https://www.ncbi.nlm.nih.gov/clinvar/variation/340087/
NM_133647.1(SLC12A6):c.1097_1099(	concordant	unreportable	unreportable	315617	0	2	SCV000617186;S(	Uncertain significance;Unc(	GeneDx;Illumina C	2017-11-28;2016-1	SLC12A6	chr15	34546567	34546570	http://gnomad.b	0.0002103978	NFE	0.0004655740049	0		0	0	https://www.ncbi.nlm.nih.gov/clinvar/variation/315617/
NM_001164277.1(SLC37A4):c.174A>G	concordant	unreportable	unreportable	459622	0	1	SCV000631381	Likely benign	Invitae	10/5/2017	SLC37A4	chr11	118899111	118899111	http://gnomad.b	4.75E-05	AFR	0.0002433479967	0		0	0	https://www.ncbi.nlm.nih.gov/clinvar/variation/459622/
NM_001042432.1(CLN3):c.831G>A	concordant	unreportable	unreportable	136780	0	1	SCV000337486	Uncertain significance	EGL Genetic Diagn(	6/30/2017	CLN3	chr16	28493953	28493953	http://gnomad.b	0.0004333237039	NFE	0.0008149449714	0		0	0	https://www.ncbi.nlm.nih.gov/clinvar/variation/136780/
NM_000370.3(TTPA):c.616G>A	concordant	unreportable	unreportable	363560	0	1	SCV000474656	Uncertain significance	Illumina Clinical Se	10/18/2016	TTPA	chr8	63976812	63976812	http://gnomad.b	3.90E-05	NFE	8.08E-05	0		0	0	https://www.ncbi.nlm.nih.gov/clinvar/variation/363560/
NM_000023.2(SGCA):c.700G>A	concordant	unreportable	unreportable	198031	0	2	SCV000231455;S(	Uncertain significance;Unc(	EGL Genetic Diagn(	2017-06-30;2017-1	SGCA	chr17	48246568	48246568	http://gnomad.b	1.40E-05	AMR	0.000565948023	0		0	0	https://www.ncbi.nlm.nih.gov/clinvar/variation/198031/
NM_024649.4(BBS1):c.803G>A	concordant	unreportable	unreportable	288064	0	2	SCV000342059;S(	Uncertain significance;Unc(	EGL Genetic Diagn(	2017-06-30;2016-1	BBS1	chr11	66288820	66288820	http://gnomad.b	5.57E-06	NFE	5.37E-05	0		0	0	https://www.ncbi.nlm.nih.gov/clinvar/variation/288064/
NM_000391.3(TPP1):c.1307T>C	concordant	unreportable	unreportable	457937	0	1	SCV000628897	Uncertain significance	Invitae	10/5/2017	TPP1	chr11	6636520	6636520	http://gnomad.b	0.0001231106709	AFR	0.0006534240092	0		0	0	https://www.ncbi.nlm.nih.gov/clinvar/variation/457937/
NM_000136.2(FANCC):c.668T>C	concordant	unreportable	unreportable	216290	0	1	SCV000254263	Uncertain significance	Invitae	9/11/2015	FANCC	chr9	97912223	97912223	http://gnomad.b	4.04E-05	AMR	0.0005252099945	0		1	0	https://www.ncbi.nlm.nih.gov/clinvar/variation/216290/
NM_000051.3(ATM):c.6975G>A	concordant	unreportable	unreportable	141676	0	3	SCV000537558;S(	Uncertain significance;Unc(	Color Genomics, In	2017-12-21;2017-1	ATM	chr11	108196952	108196952	http://gnomad.b	1.95E-05	AMR	2.98E-05	0		0	0	https://www.ncbi.nlm.nih.gov/clinvar/variation/141676/
NM_000137.2(FAH):c.133A>G	concordant	unreportable	unreportable	291148	0	1	SCV000345854	Uncertain significance	EGL Genetic Diagn(	6/30/2017	FAH	chr15	80450453	80450453	http://gnomad.b	2.22E-05	NFE	2.69E-05	0		0	0	https://www.ncbi.nlm.nih.gov/clinvar/variation/291148/
NM_000136.2(FANCC):c.1414G>A	concordant	unreportable	unreportable	134299	0	2	SCV000278974;S(	Uncertain significance;Likel	GeneDx;Invitae	2017-11-28;2017-1	FANCC	chr9	97869467	97869467	http://gnomad.b	0.0002354328874	SAS	0.002502110088	0		0	0	https://www.ncbi.nlm.nih.gov/clinvar/variation/134299/
NM_004004.5(GJB2):c.120A>C	concordant	unreportable	unreportable	195206	0	2	SCV000383036;S(	Likely benign;Uncertain sig	Illumina Clinical Se	2016-10-18;2018-0	GJB2	chr13	20763601	20763601	http://gnomad.b	0.0001788199004	NFE	0.0002969549969	0		0	0	https://www.ncbi.nlm.nih.gov/clinvar/variation/195206/
NM_000137.2(FAH):c.975G>C	concordant	unreportable	unreportable	383908	0	1	SCV000524519	Likely benign	GeneDx	11/28/2017	FAH	chr15	80472480	80472480	http://gnomad.b	6.38E-05	NFE	6.28E-05	0		0	0	https://www.ncbi.nlm.nih.gov/clinvar/variation/383908/
NM_000642.2(AGL):c.3584C>G	concordant	unreportable	unreportable	291343	0	1	SCV000346085	Uncertain significance	Illumina Clinical Se	10/18/2016	AGL	chr1	100366413	100366413	http://gnomad.b	0.000153505222	NFE	0.0001969949953	0		0	0	https://www.ncbi.nlm.nih.gov/clinvar/variation/291343/
NM_000051.3(ATM):c.8428A>C	concordant	unreportable	unreportable	181898	0	3	SCV000682488;S(	Uncertain significance;Unc(	Color Genomics, In	2017-10-26;2017-1	ATM	chr11	108216479	108216479	http://gnomad.b	5.57E-05	FIN	0.0001346500067	0		0	0	https://www.ncbi.nlm.nih.gov/clinvar/variation/181898/
NM_000051.3(ATM):c.1392G>A	concordant	unreportable	unreportable	183817	0	3	SCV000681976;S(	Likely benign;Likely benign,	Color Genomics, In	2017-10-26;2017-1	ATM	chr11	108121584	108121584	http://gnomad.b	2.51E-05	NFE	4.48E-05	0		0	0	https://www.ncbi.nlm.nih.gov/clinvar/variation/183817/
NM_000227.3(LAMA3):c.666C>T	concordant	unreportable	unreportable	326299	0	1	SCV000407921	Uncertain significance	Illumina Clinical Se	10/18/2016	LAMA3	chr18	21474902	21474902	http://gnomad.b	0.0001231864988	AMR	0.0001488980051	0		0	0	https://www.ncbi.nlm.nih.gov/clinvar/variation/326299/
NM_001173990.2(TMEM216):c.7C>A	concordant	unreportable	unreportable	452672	0	1	SCV000621494	Uncertain significance	GeneDx	11/28/2017	TMEM216	chr11	61160110	61160110		5.14E-06					0	0	https://www.ncbi.nlm.nih.gov/clinvar/variation/452672/
NM_000016.4(ACADM):c.57T>C	concordant	unreportable	unreportable	298069	0	1	SCV000358934	Uncertain significance	Illumina Clinical Se	10/18/2016	ACADM	chr1	76194112	76194112	http://gnomad.b	8.43E-05	AFR	6.54E-05	0		0	0	https://www.ncbi.nlm.nih.gov/clinvar/variation/298069/
NM_000492.3(CFTR):c.738G>A	concordant	unreportable	unreportable	416597	0	2	SCV000703806;S(	Likely benign;Likely benign	EGL Genetic Diagn(	2017-06-30;2017-0	CFTR	chr7	117175460	117175460	http://gnomad.b	4.43E-05	AFR	0.0004574570048	0		0	0	https://www.ncbi.nlm.nih.gov/clinvar/variation/416597/
NM_000152.3(GAA):c.866C>T	concordant	unreportable	unreportable	285344	0	2	SCV000338319;S(	Uncertain significance;Unc(	EGL Genetic Diagn(	2017-06-30;2017-1	GAA	chr17	78081606	78081606	http://gnomad.b	1.39E-05	AMR	3.01E-05	0		0	0	https://www.ncbi.nlm.nih.gov/clinvar/variation/285344/
NM_006493.2(CLN5):c.283G>T	concordant	unreportable	unreportable	451977	0	1	SCV000620747	Uncertain significance	GeneDx	11/28/2017	CLN5	chr13	77566369	77566369	http://gnomad.b	3.36E-05	NFE	3.36E-05	0		0	0	https://www.ncbi.nlm.nih.gov/clinvar/variation/451977/

Supplemental Table 2

NM_000136.2(FANCC):c.1069C>T	concordant	reportable	reportable	419252	1	0	SCV000566937	Pathogenic	GeneDx	11/28/2017	FANCC	chr9	97879600	97879600	http://gnomad.b	1.43E-05	NFE	8.96E-06	0		0	0	https://www.ncbi.nlm.nih.gov/clinvar/variation/419252/
NM_000071.2(CBS):c.786G>A	concordant	unreportable	unreportable	264505	0	2	SCV000320487;S(	Likely benign;Uncertain sig	Ambry Genetics;Illu	2017-11-14;2016-1	CBS	chr21	44484052	44484052	http://gnomad.b	3.37E-05	SAS	0.0002943289874	0		0	0	https://www.ncbi.nlm.nih.gov/clinvar/variation/264505/
NM_006261.4(PROP1):c.550G>C	concordant	unreportable	unreportable	287105	0	1	SCV000340773	Uncertain significance	EGL Genetic Diagn	6/30/2017	PROP1	chr5	177419841	177419841	http://gnomad.b	0.0001380312796	ASJ	0.001540670055	0		0	0	https://www.ncbi.nlm.nih.gov/clinvar/variation/287105/
NM_002225.3(IVD):c.237C>T	concordant	unreportable	unreportable	488861	0	1	SCV000680798	Uncertain significance	GeneDx	2/7/2018	IVD	chr15	40699920	40699920	http://gnomad.b	0.0002353824685	AFR	0.002352329902	0		0	0	https://www.ncbi.nlm.nih.gov/clinvar/variation/488861/
NM_000228.2(LAMB3):c.404G>A	concordant	unreportable	unreportable	282901	0	1	SCV000334641	Uncertain significance	EGL Genetic Diagn	6/30/2017	LAMB3	chr1	209807952	209807952	http://gnomad.b	5.60E-05	SAS	6.50E-05	0		0	0	https://www.ncbi.nlm.nih.gov/clinvar/variation/282901/
NM_000051.3(ATM):c.6543G>T	concordant	unreportable	unreportable	127425	0	5	SCV000537555;S(	Uncertain significance;Unc	Color Genomics, In	2017-12-21;2017-0	ATM	chr11	108192118	108192118	http://gnomad.b	0.000142020139	AFR	0.002286979929	0		0	0	https://www.ncbi.nlm.nih.gov/clinvar/variation/127425/
NM_000152.3(GAA):c.17C>T	concordant	unreportable	unreportable	499674	0	1	SCV000705295	Uncertain significance	EGL Genetic Diagn	6/30/2017	GAA	chr17	78078402	78078402	http://gnomad.b	3.89E-05	EAS	5.81E-05	0		0	0	https://www.ncbi.nlm.nih.gov/clinvar/variation/499674/
NM_000051.3(ATM):c.2396C>T	concordant	unreportable	unreportable	181928	0	3	SCV000209701;S(	Likely benign;Uncertain sig	GeneDx;Invitae;Lal	2017-11-28;2017-1	ATM	chr11	108129732	108129732	http://gnomad.b	2.78E-05	NFE	4.48E-05	0		0	0	https://www.ncbi.nlm.nih.gov/clinvar/variation/181928/
NM_001079802.1(FKTN):c.333T>C	concordant	unreportable	unreportable	497230	0	1	SCV000701619	Uncertain significance	EGL Genetic Diagn	6/30/2017	FKTN	chr9	108363593	108363593	http://gnomad.b	2.48E-05	AFR	0.0007193299825	0		0	0	https://www.ncbi.nlm.nih.gov/clinvar/variation/497230/
NM_024649.4(BBS1):c.726G>A	concordant	unreportable	unreportable	283883	0	2	SCV000336239;S(	Uncertain significance;Unc	EGL Genetic Diagn	2017-06-30;2017-1	BBS1	chr11	66288743	66288743	http://gnomad.b	2.79E-05	AMR	0.0002680330072	0		0	0	https://www.ncbi.nlm.nih.gov/clinvar/variation/283883/
NM_000152.3(GAA):c.2319C>T	concordant	unreportable	unreportable	325792	0	1	SCV000407296	Uncertain significance	Illumina Clinical Se	10/18/2016	GAA	chr17	78090896	78090896	http://gnomad.b	9.73E-05	NFE	0.0001175619982	0		0	0	https://www.ncbi.nlm.nih.gov/clinvar/variation/325792/
NM_000051.3(ATM):c.2333A>G	concordant	unreportable	unreportable	127348	0	4	SCV000149062;S(	Uncertain significance;Unc	GeneDx;Illumina C	2017-11-28;2016-1	ATM	chr11	108128290	108128290	http://gnomad.b	3.62E-05	NFE	1.79E-05	0		0	0	https://www.ncbi.nlm.nih.gov/clinvar/variation/127348/
NM_000051.3(ATM):c.2085G>A	concordant	unreportable	unreportable	185505	0	3	SCV000682024;S(	Likely benign;Likely benign	Color Genomics, In	2017-10-26;2017-1	ATM	chr11	108124727	108124727		0.000128096596					0	0	https://www.ncbi.nlm.nih.gov/clinvar/variation/185505/
NM_002225.3(IVD):c.925C>G	concordant	unreportable	unreportable	315801	0	1	SCV000390864	Uncertain significance	Illumina Clinical Se	10/18/2016	IVD	chr15	40707637	40707637	http://gnomad.b	0.000240986813	NFE	0.001987860072	2		0	0	https://www.ncbi.nlm.nih.gov/clinvar/variation/315801/
NM_000051.3(ATM):c.1998G>A	concordant	unreportable	unreportable	453399	0	2	SCV000687348;S(	Likely benign;Likely benign	Color Genomics, In	2017-12-21;2017-1	ATM	chr11	108124640	108124640	http://gnomad.b	2.78E-06	NFE	8.96E-06	0		0	0	https://www.ncbi.nlm.nih.gov/clinvar/variation/453399/
NM_000098.2(CPT2):c.1372A>C	concordant	unreportable	unreportable	449681	0	2	SCV000618018;S(	Uncertain significance;Likel	GeneDx;Invitae	2017-11-28;2017-1	CPT2	chr1	53676718	53676718	http://gnomad.b	0.0002210495095	AFR	0.002876190003	0		0	0	https://www.ncbi.nlm.nih.gov/clinvar/variation/449681/
NM_006493.2(CLN5):c.652G>A	concordant	unreportable	unreportable	457966	0	1	SCV000628967	Uncertain significance	Invitae	10/5/2017	CLN5	chr13	77570202	77570202		2.80E-06					0	0	https://www.ncbi.nlm.nih.gov/clinvar/variation/457966/
NM_000152.3(GAA):c.11G>A	concordant	unreportable	unreportable	188475	0	2	SCV000703366;S(	Uncertain significance;Unc	EGL Genetic Diagn	2017-06-30;2016-1	GAA	chr17	78078396	78078396	http://gnomad.b	3.89E-05	NFE	5.43E-05	0		0	0	https://www.ncbi.nlm.nih.gov/clinvar/variation/188475/
NM_004543.4(NEB):c.5656G>A	concordant	unreportable	unreportable	331503	0	1	SCV000416987	Uncertain significance	Illumina Clinical Se	10/18/2016	NEB	chr2	152520169	152520169	http://gnomad.b	2.07E-05	NFE	2.69E-05	0		0	0	https://www.ncbi.nlm.nih.gov/clinvar/variation/331503/
NM_000152.3(GAA):c.2571G>T	concordant	unreportable	unreportable	382786	0	3	SCV000522903;S(	Likely benign;Benign;Benig	GeneDx;Invitae;Lal	2017-11-28;2017-1	GAA	chr17	78092081	78092081	http://gnomad.b	0.0008893780468	AFR	0.01157740038	1		1	0	https://www.ncbi.nlm.nih.gov/clinvar/variation/382786/
NM_000152.3(GAA):c.2092G>A	concordant	unreportable	unreportable	188483	0	2	SCV000337752;S(	Uncertain significance;Unc	EGL Genetic Diagn	2017-06-30;2017-1	GAA	chr17	78087068	78087068	http://gnomad.b	8.34E-05	NFE	8.07E-05	0		0	0	https://www.ncbi.nlm.nih.gov/clinvar/variation/188483/
NM_000137.2(FAH):c.409G>A	concordant	unreportable	unreportable	497627	0	1	SCV000702242	Uncertain significance	EGL Genetic Diagn	6/30/2017	FAH	chr15	80454632	80454632	http://gnomad.b	0.0001026460487	AFR	0.0008494509966	0		0	0	https://www.ncbi.nlm.nih.gov/clinvar/variation/497627/
NM_000053.3(ATP7B):c.4163C>T	concordant	unreportable	unreportable	424208	0	1	SCV000573994	Uncertain significance	GeneDx	11/28/2017	ATP7B	chr13	52509127	52509127	http://gnomad.b	8.14E-05	NFE	8.22E-05	0		0	0	https://www.ncbi.nlm.nih.gov/clinvar/variation/424208/
NM_000071.2(CBS):c.1353G>C	concordant	unreportable	unreportable	212868	0	1	SCV000249715	Uncertain significance	GeneDx	11/28/2017	CBS	chr21	44478949	44478949	http://gnomad.b	1.12E-05	NFE	1.57E-05	0		0	0	https://www.ncbi.nlm.nih.gov/clinvar/variation/212868/
NM_003060.3(SLC22A5):c.35G>A	concordant	unreportable	unreportable	285771	0	1	SCV000338941	Uncertain significance	EGL Genetic Diagn	6/30/2017	SLC22A5	chr5	131705699	131705699		1.69E-05					0	0	https://www.ncbi.nlm.nih.gov/clinvar/variation/285771/
NM_000060.2(BTD):c.1285G>A	concordant	unreportable	unreportable	343912	0	1	SCV000441832	Uncertain significance	Illumina Clinical Se	10/18/2016	BTD	chr3	15686648	15686648	http://gnomad.b	0.0001009116648	SAS	0.0002923790016	1		0	0	https://www.ncbi.nlm.nih.gov/clinvar/variation/343912/
NM_000051.3(ATM):c.4871A>G	concordant	unreportable	unreportable	135757	0	3	SCV000209743;S(	Uncertain significance;Unc	GeneDx;Invitae;Lal	2017-11-28;2017-1	ATM	chr11	108165748	108165748	http://gnomad.b	7.24E-05	AFR	0.0005881579709	0		0	0	https://www.ncbi.nlm.nih.gov/clinvar/variation/135757/
NM_000642.2(AGL):c.1241C>T	concordant	unreportable	unreportable	456452	0	1	SCV000626669	Uncertain significance	Invitae	10/5/2017	AGL	chr1	100340969	100340969	http://gnomad.b	2.23E-05	NFE	3.58E-05	0		0	0	https://www.ncbi.nlm.nih.gov/clinvar/variation/456452/
NM_004328.4(BCS1L):c.985A>G	concordant	unreportable	unreportable	384760	0	1	SCV000525780	Uncertain significance	GeneDx	11/28/2017	BCS1L	chr2	219527701	219527701	http://gnomad.b	8.37E-06	NFE	3.58E-05	0		0	0	https://www.ncbi.nlm.nih.gov/clinvar/variation/384760/
NM_174878.2(CLRN1):c.473A>G	concordant	unreportable	unreportable	343817	0	1	SCV000441713	Uncertain significance	Illumina Clinical Se	10/18/2016	CLRN1	chr3	150645949	150645949	http://gnomad.b	1.54E-05	NFE	6.28E-05	0		0	0	https://www.ncbi.nlm.nih.gov/clinvar/variation/343817/
NM_000051.3(ATM):c.7816A>G	concordant	unreportable	unreportable	187077	0	2	SCV000564661;S(	Uncertain significance;Unc	GeneDx;Invitae	2017-11-28;2017-1	ATM	chr11	108203516	108203516	http://gnomad.b	3.34E-05	NFE	3.60E-05	0		0	0	https://www.ncbi.nlm.nih.gov/clinvar/variation/187077/
NM_017890.4(VPS13B):c.2509A>T	concordant	unreportable	unreportable	289218	0	1	SCV000343537	Uncertain significance	EGL Genetic Diagn	6/30/2017	VPS13B	chr8	100205279	100205279	http://gnomad.b	4.76E-05	AFR	6.54E-05	0		0	0	https://www.ncbi.nlm.nih.gov/clinvar/variation/289218/
NM_000051.3(ATM):c.2281A>T	concordant	unreportable	unreportable	135744	0	3	SCV000209699;S(	Uncertain significance;Unc	GeneDx;Invitae;Lal	2017-11-28;2017-1	ATM	chr11	108128238	108128238	http://gnomad.b	7.24E-05	AFR	0.0005881579709	0		0	0	https://www.ncbi.nlm.nih.gov/clinvar/variation/135744/
NM_000071.2(CBS):c.1205T>C	concordant	unreportable	unreportable	471355	0	1	SCV000649822	Uncertain significance	Invitae	10/5/2017	CBS	chr21	44479354	44479354	http://gnomad.b	7.31E-05	AMR	0.0002680009929	0		0	0	https://www.ncbi.nlm.nih.gov/clinvar/variation/471355/
NM_138694.3(PKHD1):c.3838C>T	concordant	unreportable	unreportable	498678	0	1	SCV000703818	Uncertain significance	EGL Genetic Diagn	6/30/2017	PKHD1	chr6	51890770	51890770	http://gnomad.b	0.0001223187181	AFR	0.0009867120534	0		0	0	https://www.ncbi.nlm.nih.gov/clinvar/variation/498678/
NM_000051.3(ATM):c.2495G>A	concordant	unreportable	unreportable	141644	0	2	SCV000571820;S(	Uncertain significance;Unc	GeneDx;Invitae	2017-11-28;2017-1	ATM	chr11	108137926	108137926	http://gnomad.b	4.18E-05	AFR	6.54E-05	0		0	0	https://www.ncbi.nlm.nih.gov/clinvar/variation/141644/
NM_000288.3(PEX7):c.695G>A	concordant	unreportable	unreportable	500853	0	1	SCV000706975	Uncertain significance	EGL Genetic Diagn	6/30/2017	PEX7	chr6	137191089	137191089	http://gnomad.b	0.0003250108337	EAS	0.003072819905	0		0	0	https://www.ncbi.nlm.nih.gov/clinvar/variation/500853/
NM_002485.4(NBN):c.127C>T	concordant	reportable	reportable	142203	3	0	SCV000685707;S(	Pathogenic;Pathogenic;Pat	Color Genomics, In	2017-10-26;2017-1	NBN	chr8	90994994	90994994	http://gnomad.b	2.79E-05	FIN	0.0001794690033	0		0	0	https://www.ncbi.nlm.nih.gov/clinvar/variation/142203/
NM_000352.3(ABCC8):c.291-3C>T	concordant	unreportable	unreportable	303788	0	2	SCV000592968;S(	Uncertain significance;Unc	Genetic Services La	2017-07-05;2016-1	ABCC8	chr11	17491772	17491772	http://gnomad.b	3.61E-05	SAS	6.50E-05	0		0	0	https://www.ncbi.nlm.nih.gov/clinvar/variation/303788/
NM_000642.2(AGL):c.2607A>G	concordant	unreportable	unreportable	284410	0	1	SCV000337026	Uncertain significance	EGL Genetic Diagn	6/30/2017	AGL	chr1	100350185	100350185	http://gnomad.b	0.0001255951816	NFE	0.0001436089951	0		0	0	https://www.ncbi.nlm.nih.gov/clinvar/variation/284410/
NM_000051.3(ATM):c.3963G>A	concordant	unreportable	unreportable	133618	0	2	SCV000566628;S(	Uncertain significance;Unc	GeneDx;Invitae	2017-11-28;2017-1	ATM	chr11	108155170	108155170	http://gnomad.b	0.0001308813046	EAS	0.00156667002	0		0	0	https://www.ncbi.nlm.nih.gov/clinvar/variation/133618/
NM_138694.3(PKHD1):c.4304G>C	concordant	unreportable	unreportable	96402	0	1	SCV000114594	Uncertain significance	EGL Genetic Diagn	6/30/2017	PKHD1	chr6	51890304	51890304	http://gnomad.b	0.0002529773488	NFE	0.0002425529965	0		0	0	https://www.ncbi.nlm.nih.gov/clinvar/variation/96402/
NM_000487.5(ARSA):c.918G>A	concordant	unreportable	unreportable	288377	0	1	SCV000342472	Uncertain significance	EGL Genetic Diagn	6/30/2017	ARSA	chr22	51064643	51064643	http://gnomad.b	3.66E-05	AFR	0.0001334039989	0		0	0	https://www.ncbi.nlm.nih.gov/clinvar/variation/288377/
NM_033056.3(PCDH15):c.2908G>A	concordant	unreportable	unreportable	286958	0	1	SCV000340570	Uncertain significance	EGL Genetic Diagn	11/3/2016	PCDH15	chr10	55721613	55721613	http://gnomad.b	0.0001080080235	AFR	0.001242320053	0		0	0	https://www.ncbi.nlm.nih.gov/clinvar/variation/286958/
NM_000414.3(HSD17B4):c.2176C>G	concordant	unreportable	unreportable	287598	0	1	SCV000341412	Uncertain significance	EGL Genetic Diagn	6/30/2017	HSD17B4	chr5	118877654	118877654	http://gnomad.b	9.86E-05	AFR	0.001437910018	0		0	0	https://www.ncbi.nlm.nih.gov/clinvar/variation/287598/
NM_033056.3(PCDH15):c.1998C>T	concordant	unreportable	unreportable	286959	0	1	SCV000340571	Uncertain significance	EGL Genetic Diagn	11/3/2016	PCDH15	chr10	55839184	55839184	http://gnomad.b	2.83E-05	AFR	0.0001960530062	0		0	0	https://www.ncbi.nlm.nih.gov/clinvar/variation/286959/
NM_000352.3(ABCC8):c.486C>T	concordant	unreportable	unreportable	303786	0	1	SCV000369405	Likely benign	Illumina Clinical Se	10/18/2016	ABCC8	chr11	17485078	17485078	http://gnomad.b	0.0001187298998	AFR	0.0008780329954	0		0	0	https://www.ncbi.nlm.nih.gov/clinvar/variation/303786/
NM_138694.3(PKHD1):c.633G>A	concordant	unreportable	unreportable	96416	0	2	SCV000114608;S(	Uncertain significance;Unc	EGL Genetic Diagn	2017-06-30;2016-1	PKHD1	chr6	51935838	51935838	http://gnomad.b	0.0004058757464	AMR	0.0003575900046	0		0	0	https://www.ncbi.nlm.nih.gov/clinvar/variation/96416/
NM_000051.3(ATM):c.4082A>G	concordant	unreportable	unreportable	185234	0	3	SCV000682183;S(	Uncertain significance;Unc	Color Genomics, In	2017-10-26;2017-1	ATM	chr11	108158415	108158415	http://gnomad.b	9.19E-05	AFR	0.0006536800065	0		0	0	https://www.ncbi.nlm.nih.gov/clinvar/variation/185234/
NM_000466.2(PEX1):c.1975G>T	concordant	unreportable	unreportable	289197	0	1	SCV000343511	Uncertain significance	EGL Genetic Diagn	6/30/2017	PEX1	chr7	92134142	92134142	http://gnomad.b	7.21E-05	AFR	0.0004575159983	0		0	0	https://www.ncbi.nlm.nih.gov/clinvar/variation/289197/
NM_000152.3(GAA):c.2456G>A	concordant	unreportable	unreportable	456403	0	1	SCV000626572	Uncertain significance	Invitae	10/5/2017	GAA	chr17	78091523	78091523	http://gnomad.b	1.11E-05	AFR	0.0001358139998	0		0	0	https://www.ncbi.nlm.nih.gov/clinvar/variation/456403/
NM_000136.2(FANCC):c.1000C>T	concordant	unreportable	unreportable	216279	0	1	SCV000254248	Uncertain significance	Invitae	10/5/2017	FANCC	chr9	97879669	97879669	http://gnomad.b	8.32E-05	EAS	0.001048580045	1		0	0	https://www.ncbi.nlm.nih.gov/clinvar/variation/216279/
NM_000051.3(ATM):c.1104A>G	concordant	unreportable	unreportable	183806	0	2	SCV000681952;S(	Likely benign;Likely benign	Color Genomics, In	2017-10-26;2017-1	ATM	chr11	108119698	108119698	http://gnomad.b	1.39E-05	EAS	5.80E-05	0		0	0	https://www.ncbi.nlm.nih.gov/clinvar/variation/183806/
NM_138694.3(PKHD1):c.3048T>C	concordant	unreportable	unreportable	96394	0	2	SCV000114586;S(	Uncertain significance;Unc	EGL Genetic Diagn	2017-06-30;2016-1	PKHD1	chr6	51907706	51907706	http://gnomad.b	0.0004058757464	AMR	0.0003578029864	0		0	0	https://www.ncbi.nlm.nih.gov/clinvar/variation/96394/
NM_138694.3(PKHD1):c.9705T>A	concordant	unreportable	unreportable	357415	0	1	SCV000464039	Uncertain significance	Illumina Clinical Se	10/18/2016	PKHD1	chr6	51612709	51612709	http://gnomad.b	4.45E-05	SAS	0.001039569965	1		0	0	https://www.ncbi.nlm.nih.gov/clinvar/variation/357415/
NM_002485.4(NBN):c.1986G>A	concordant	unreportable	unreportable	184121	0	2	SCV000685748;S(	Likely benign;Likely benign	Color Genomics, In	2017-10-26;2017-0	NBN	chr8	90958452	90958452	http://gnomad.b	1.95E-05	AFR	0.0001962069946	0		0	0	https://www.ncbi.nlm.nih.gov/clinvar/variation/184121/
NM_002485.4(NBN):c.1754A>G	concordant	unreportable	unreportable	411789	0	2	SCV000685736;S(	Uncertain significance;Unc	Color Genomics, In	2017-10-26;2017-0	NBN	chr8	90965563	90965563	http://gnomad.b	3.07E-05	AMR	0.0002680649923	0		0	0	https://www.ncbi.nlm.nih.gov/clinvar/variation/411789/
NM_001079802.1(FKTN):c.1228C>A	concordant	unreportable	unreportable	283469	0	1	SCV000335575	Uncertain significance	EGL Genetic Diagn	6/30/2017	FKTN	chr9	108397387	108397387	http://gnomad.b	0.0002269326506	SAS	0.001559350058	0		0	0	https://www.ncbi.nlm.nih.gov/clinvar/variation/283469/
NM_000243.2(MEFV):c.621C>T	concordant	unreportable	unreportable	495756	0	1	SCV000696077	Uncertain significance	Laboratory Corpor	1/25/2018	MEFV	chr16	3304447	3304447	http://gnomad.b	2.24E-05	EAS	5.92E-05	0		0	0	https://www.ncbi.nlm.nih.gov/clinvar/variation/495756/
NM_017890.4(VPS13B):c.1770G>A	concordant	unreportable	unreportable	194257	0	1	SCV000225903	Uncertain significance	EGL Genetic Diagn	6/30/2017	VPS13B	chr8	100155320	100155320	http://gnomad.b	6.44E-05	EAS	0.001217529993	0		0	0	https://www.ncbi.nlm.nih.gov/clinvar/variation/194257/
NM_000035.3(ALDOB):c.523G>A	concordant	unreportable	unreportable	364312	0	1	SCV000476064	Uncertain significance	Illumina Clinical Se	10/18/2016	ALDOB	chr9	104189781	104189781	http://gnomad.b	2.54E-05	SAS	0.0007147499709	0		0	0	https://www.ncbi.nlm.nih.gov/clinvar/variation/364312/
NM_000528.3(MAN2B1):c.1398G>A	concordant	unreportable	unreportable	328270	0	1	SCV000410794	Uncertain significance	Illumina Clinical Se	10/18/2016	MAN2B1	chr19	12768281	12768281	http://gnomad.b	3.66E-05	NFE	6.88E-05	0		0	0	https://www.ncbi.nlm.nih.gov/clinvar/variation/328270/
NM_000051.3(ATM):c.134G>A	concordant	unreportable	unreportable	188157	0	1	SCV000293679	Uncertain significance	GeneDx	11/28/2017	ATM	chr11	108098564	108098564	http://gnomad.b	1.39E-05	AMR	5.96E-05	0		0	0	https://www.ncbi.nlm.nih.gov/clinvar/variation/188157/
NM_000492.3(CFTR):c.3767C>T	concordant	unreportable	unreportable	495935	0	1	SCV000696990	Uncertain significance	Laboratory Corpor	1/25/2018	CFTR	chr7	117282541	117282541	http://gnomad.b	5.78E-06	SAS	0.0002274059952	0		0	0	https://www.ncbi.nlm.nih.gov/clinvar/variation/495935/
NM_000352.3(ABCC8):c.1537G>A	concordant	unreportable	unreportable	303783	0	1	SCV000369382	Uncertain significance	Illumina Clinical Se	10/18/2016	ABCC8	chr11	17464360	17464360	http://gnomad.b	7.23E-05	NFE	8.08E-05	0		0	0	https://www.ncbi.nlm.nih.gov/clinvar/variation/303783/
NM_000543.4(SMPD1):c.1135C>T	concordant	unreportable	unreportable	496881	0	1	SCV000700484	Uncertain significance	EGL Genetic Diagn	6/30/2017	SMPD1	chr11	6414489	6414489	http://gnomad.b	0.0001094196004	NFE	0.0001253289956	0		0	0	https://www.ncbi.nlm.nih.gov/clinvar/variation/496881/
NM_000136.2(FANCC):c.565C>G	concordant	unreportable	unreportable	216289	0	1	SCV000254262	Uncertain significance	Invitae	9/11/2015	FANCC	chr9	97912326	97912326	http://gnomad.b	7.13E-06	AFR	6.54E-05	0		0	0	https://www.ncbi.nlm.nih.gov/clinvar/variation/216289/
NM_183050.2(BCKDHB):c.51A>C	concordant	unreportable	unreportable	281334	0	2	SCV000332078;S(	Uncertain significance;Unc	EGL Genetic Diagn	2017-06-30;2016-1	BCKDHB	chr6	80816461	80816461	http://gnomad.b	0.0001794006992	AFR	0.002437239978	0		0	0	https://www.ncbi.nlm.nih.gov/clinvar/variation/281334/
NM_000051.3(ATM):c.4071T>C	concordant	unreportable	unreportable	230702	0	2	SCV000682182;S(	Likely benign;Likely benign	Color Genomics, In	2017-10-26;2017-1	ATM	chr11	108158404	108158404	http://gnomad.b	1.39E-05	AFR	6.54E-05	0		0	0	https://www.ncbi.nlm.nih.gov/clinvar/variation/230702/
NM_000152.3(GAA):c.1920T>G	concordant	unreportable	unreportable	255358	0	1	SCV000337540	Uncertain significance	EGL Genetic Diagn	6/30/2017	GAA	chr17	78086706	78086706	http://gnomad.b	0.0002056686733	EAS	0.0009380860138	0		1	0	https://www.ncbi.nlm.nih.gov/clinvar/variation/255358/
NM_000051.3(ATM):c.5624G>C	concordant	unreportable	unreportable	453591	0	1	SCV000622606	Uncertain significance	Invitae	10/5/2017	ATM	chr11	108175529	108175529		2.78E-06					0	0	https://www.ncbi.nlm.nih.gov/clinvar/variation/453591/
NM_000152.3(GAA):c.758C>G	concordant	unreportable	unreportable	456437	0	1	SCV000626638	Uncertain significance	Invitae	10/5/2017	GAA	chr17	78081421	78081421	http://gnomad.b	1.95E-05	NFE	8.96E-06	0		0	0	https://www.ncbi.nlm.nih.gov/clinvar/variation/456437/
NM_000518.4(HBB):c.316-128G>C	concordant	unreportable	unreportable	495989	0	1	SCV000697110	Uncertain significance	Laboratory Corpor	1/25/2018	HBB	chr11	5247084	5247084		4.67E-06					0	0	https://www.ncbi.nlm.nih.gov/clinvar/variation/495989/
NM_017890.4(VPS13B):c.1528C>T	concordant	unreportable	unreportable	193908	0	1	SCV000225384	Uncertain significance	EGL Genetic Diagn	6/30/2017	VPS13B	chr8	100147926	100147926	http://gnomad.b	0.0001987737619	AFR	0.0007850319962	1		0	0	https://www.ncbi.nlm.nih.gov/clinvar/variation/193908/
NM_000017.2(ACADS):c.481A>G	concordant	unreportable	unreportable	203553	0	1	SCV000238603	Uncertain significance	GeneDx	11/28/2017	ACADS	chr12	121175648	121175648	http://gnomad.b	1.13E-05	NFE	2.69E-05	0		0	0	https://www.ncbi.nlm.nih.gov/clinvar/variation/203553/
NM_000492.3(CFTR):c.3659C>T	concordant	unreportable	unreportable	7214	0	2	SCV000603027;S(	Uncertain significance;Unc	ARUP Laboratories	2017-06-30;2018-0	CFTR	chr7	117267766	117267766	http://gnomad.b	0.0001502149229	EAS	0.0007557260105	0		0	0	https://www.ncbi.nlm.nih.gov/clinvar/variation/7214/
NM_000518.4(HBB):c.*56A>T	concordant	unreportable	unreportable	305000	0	1	SCV000372525	Uncertain significance	Illumina Clinical Se	10/18/2016	HBB	chr11	5246772	5246772		2.33E-06					0	0	https://www.ncbi.nlm.nih.gov/clinvar/variation/305000/
NM_003060.3(SLC22A5):c.761G>A	concordant	unreportable	unreportable	25389	0	3	SCV000338349;S(	Uncertain significance;Unc	EGL Genetic Diagn	2017-06-30;2016-1	SLC22A5	chr5	131721128	131721128	http://gnomad.b	0.0001440336191	NFE	0.0001790289971	0		0	0	https://www.ncbi.nlm.nih.gov/clinvar/variation/25389/
NM_000098.2(CPT2):c.852delC	concordant	reportable	reportable	372351	1	0	SCV000490509	Pathogenic	GeneDx	12/21/2016	CPT2	chr1	53676193	53676194	http://gnomad.b	1.68E-05	NFE	8.98E-06	0		0	0	https://www.ncbi.nlm.nih.gov/clinvar/variation/372351/
NM_000136.2(FANCC):c.1509G>A	concordant	unreportable	unreportable	182501	0	1	SCV000253076	Likely benign	Invitae	10/5/2017	FANCC	chr9	97869372	97869372	http://gnomad.b	6.42E-05	FIN	0.0005381649826	0		0	0	https://www.ncbi.nlm.nih.gov/clinvar/variation/182501/
NM_000492.3(CFTR):c.2557A>G	concordant	unreportable	unreportable	439487	0	2	SCV000603037;S(	Uncertain significance;Unc	ARUP Laboratories	2017-06-30;2018-0	CFTR	chr7	117235050	117235050	http://gnomad.b	8.67E-05	AFR	6.54E-05	0		0	0	https://www.ncbi.nlm.nih.gov/clinvar/variation/439487/
NM_017890.4(VPS13B):c.5965T>C	concordant	unreportable	unreportable	196921	0	1	SCV000229741	Uncertain significance	EGL Genetic Diagn	6/30/2017	VPS13B	chr8	100654708	100654708	http://gnomad.b	8.40E-05	AFR	0.0009179119952	0		0	0	https://www.ncbi.nlm.nih.gov/clinvar/variation/196921/
NM_024649.4(BBS1):c.1394G>A	concordant	unreportable	unreportable	305470	0	1	SCV000373279	Uncertain significance	Illumina Clinical Se	10/18/2016	BBS1	chr11	66297344	66297344	http://gnomad.b	3.62E-05	EAS	0.0001161039982	0		0	0	https://www.ncbi.nlm.nih.gov/clinvar/variation/305470/
NM_020533.2(MCOLN1):c.815C>G	concordant	unreportable	unreportable	330495	0	1	SCV000415611	Uncertain significance	Illumina Clinical Se	10/18/2016	MCOLN1	chr19	7593081	7593081	http://gnomad.b	0.0002021941635	EAS	0.001507419976	0		2	0	https://www.ncbi.nlm.nih.gov/clinvar/variation/330495/
NM_000051.3(ATM):c.6333T>C	concordant	unreportable	unreportable	135769	0	3	SCV000682326;S(	Likely benign;Likely benign	Color Genomics, In	2017-10-26;2017-1	ATM	chr11	108188234	108188234	http://gnomad.b	0.0001058189271	EAS	0.000348149013	0		0	0	https://www.ncbi.nlm.nih.gov/clinvar/variation/135769/
NM_000228.2(LAMB3):c.927C>T	concordant	unreportable	unreportable	295123	0	1	SCV000353650	Uncertain significance	Illumina Clinical Se	10/18/2016	LAMB3	chr1	209803976	209803976	http://gnomad.b	0.0002575573485	NFE	0.000295698992	0		0	0	https://www.ncbi.nlm.nih.gov/clinvar/variation/295123/
NM_000051.3(ATM):c.8993T>C	concordant	unreportable	unreportable	236795	0	3	SCV000687867;S(	Uncertain significance;Unc	Color Genomics, In	2017-12-21;2017-1	ATM	chr11	108236057	108236057	http://gnomad.b	1.39E-05	AFR	0.0001960270019	0		0	0	https://www.ncbi.nlm.nih.gov/clinvar/variation/236795/
NM_000050.4(ASS1):c.299G>A	concordant	unreportable	unreportable	439417	0	1	SCV000602547	Uncertain significance	ARUP Laboratories	6/30/2017	ASS1	chr9	133333912	133333912	http://gnomad.b	0.0001866014583	AMR	0.0003872269881	0		1	0	https://www.ncbi.nlm.nih.gov/clinvar/variation/439417/
NM_020533.2(MCOLN1):c.1038G>A	concordant	unreportable	unreportable	330500	0	1	SCV000415618	Uncertain significance	Illumina Clinical Se	10/18/2016	MCOLN1	chr19	7593760	7593760	http://gnomad.b	0.0001855428794	EAS	0.001913269982	0		0	0	https://www.ncbi.nlm.nih.gov/clinvar/variation/330500/
NM_017890.4(VPS13B):c.10596C>T	concordant	unreportable	unreportable	287482	0	2	SCV000341266;S(	Uncertain significance;Likel	EGL Genetic Diagn	2017-06-30;2017-0	VPS13B	chr8	100866138	100866138	http://gnomad.b	0.0001007866962	EAS	0.0009276440251	0		0	0	https://www.ncbi.nlm.nih.gov/clinvar/variation/287482/
NM_000018.3(ACADVL):c.678C>A	concordant	unreportable	unreportable	282922	0	1	SCV000334667	Uncertain significance	EGL Genetic Diagn	6/30/2017	ACADVL	chr17	7125326	7125326	http://gnomad.b	0.0001104377867	NFE	5.37E-05	0		0	0	https://www.ncbi.nlm.nih.gov/clinvar/variation/282922/
NM_000152.3(GAA):c.688G>A	concordant	unreportable	unreportable	196222	0	3	SCV000228795;S(	Uncertain significance;Unc	EGL Genetic Diagn	2017-06-30;2015-0	GAA	chr17	78079689	78079689	http://gnomad.b	0.0001889928349	AFR	0.002638519974	0		1	0	https://www.ncbi.nlm.nih.gov/clinvar/variation/196222/
NM_000136.2(FANCC):c.1607T>C	concordant	unreportable	unreportable	127536	0	1	SCV000149255	Uncertain significance	GeneDx	11/28/2017	FANCC	chr9	97864059	97864059	http://gnomad.b	2.38E-05	NFE	3.58E-05	0		0	0	https://www.ncbi.nlm.nih.gov/clinvar/variation/127536/
NM_000051.3(ATM):c.4414T>G	concordant	unreportable	unreportable	127390	0	5	SCV000292154;S(	Uncertain significance;Unc	Color Genomics, In	2017-12-21;2017-0	ATM	chr11	108160506	108160506	http://gnomad.b	6.40E-05	AMR	0.0003579529875	0		1	0	https://www.ncbi.nlm.nih.gov/clinvar/variation/127390/
NM_000051.3(ATM):c.103C>A	concordant	unreportable	unreportable	183805	0	3	SCV000681948;S(	Likely benign;Likely benign	Color Genomics, In	2017-10-26;2017-1	ATM	chr11	108098533	108098533	http://gnomad.b	0.0001364507218	AFR	0.0008504510042	0		0	0	https://www.ncbi.nlm.nih.gov/clinvar/variation/183805/
NM_001360.2(DHCR7):c.1381C>T	concordant	unreportable	unreportable	305952	0	2	SCV000594360;S(	Uncertain significance;Unc	Genetic Services La	2017-07-05;2016-1	DHCR7	chr11	71146468	71146468	http://gnomad.b	0.0003446493193	EAS	0.001689780038	0		0	0	https://www.ncbi.nlm.nih.gov/clinvar/variation/305952/
NM_000136.2(FANCC):c.1249G>A	concordant	unreportable	unreportable	182485	0	2	SCV000211051;S(	Uncertain significance;Unc	GeneDx;Invitae	2017-11-28;2017-0	FANCC	chr9	97873825	97873825	http://gnomad.b	7.37E-05	AFR	0.0002615400008	0		0	0	https://www.ncbi.nlm.nih.gov/clinvar/variation/182485/

Supplemental Table 2

NM_024649.4(BBS1):c.1626A>G	concordant	unreportable	unreportable	261749	0	1	SCV000593584	Uncertain significance	Genetic Services La	7/5/2017	BBS1	chr11	66299144	66299144	http://gnomad.b	3.62E-05	SAS	0.0006497299764	0	0	0	https://www.ncbi.nlm.nih.gov/clinvar/variation/261749/
NM_000051.3(ATM):c.3154-4G>T	concordant	unreportable	unreportable	233588	0	2	SCV000512154;S(	Likely benign;Likely benign	GeneDx;Invitae	2017-11-28;2017-1	ATM	chr11	108143445	108143445	http://gnomad.b	8.35E-06	NFE	2.69E-05	0	0	0	https://www.ncbi.nlm.nih.gov/clinvar/variation/233588/
NM_002485.4(NBN):c.939G>A	concordant	unreportable	unreportable	136048	0	2	SCV000685839;S(	Likely benign;Likely benign	Color Genomics, In	2017-10-26;2017-1	NBN	chr8	90976693	90976693	http://gnomad.b	0.000158951478	AFR	0.001241829945	0	0	0	https://www.ncbi.nlm.nih.gov/clinvar/variation/136048/
NM_000051.3(ATM):c.8113G>A	concordant	unreportable	unreportable	127454	0	3	SCV000687816;S(	Uncertain significance;Unc	Color Genomics, In	2017-12-21;2017-1	ATM	chr11	108205798	108205798	http://gnomad.b	3.62E-05	NFE	8.96E-06	0	0	0	https://www.ncbi.nlm.nih.gov/clinvar/variation/127454/
NM_018941.3(CLN8):c.513C>G	concordant	unreportable	unreportable	386111	0	2	SCV000701918;S(	Uncertain significance;Likel	EGL Genetic Diagn	2017-06-30;2017-1	CLN8	chr8	1719733	1719733	http://gnomad.b	0.000176292814	AFR	0.002157989889	0	1	0	https://www.ncbi.nlm.nih.gov/clinvar/variation/386111/
NM_000243.2(MEFV):c.487G>C	concordant	unreportable	unreportable	234356	0	1	SCV000279034	Uncertain significance	GeneDx	11/28/2017	MEFV	chr16	3304581	3304581		1.40E-05				0	0	https://www.ncbi.nlm.nih.gov/clinvar/variation/234356/
NM_000518.4(HBB):c.316-138G>A	concordant	unreportable	unreportable	495991	0	1	SCV000697112	Uncertain significance	Laboratory Corpora	1/25/2018	HBB	chr11	5247094	5247094		2.80E-05				0	0	https://www.ncbi.nlm.nih.gov/clinvar/variation/495991/
NM_001173990.2(TMEM216):c.343C>	concordant	unreportable	unreportable	500203	0	1	SCV000706039	Uncertain significance	EGL Genetic Diagn	6/30/2017	TMEM216	chr11	61165359	61165359	http://gnomad.b	4.37E-05	EAS	0.0001159550011	0	0	0	https://www.ncbi.nlm.nih.gov/clinvar/variation/500203/
NM_000051.3(ATM):c.4101C>T	concordant	unreportable	unreportable	135753	0	1	SCV000687520	Likely benign	Color Genomics, In	12/21/2017	ATM	chr11	108158434	108158434	http://gnomad.b	5.57E-06	EAS	0.0001162929984	0	0	0	https://www.ncbi.nlm.nih.gov/clinvar/variation/135753/
NM_000051.3(ATM):c.2630G>C	concordant	unreportable	unreportable	127356	0	2	SCV000149070;S(	Uncertain significance;Unc	GeneDx;Invitae	2017-11-28;2017-1	ATM	chr11	108138061	108138061	http://gnomad.b	0.0001058189271	EAS	0.0002320450003	1	0	0	https://www.ncbi.nlm.nih.gov/clinvar/variation/127356/
NM_017739.3(POMGNT1):c.286C>T	concordant	unreportable	unreportable	283338	0	1	SCV000335358	Uncertain significance	EGL Genetic Diagn	6/30/2017	POMGNT1	chr1	46662471	46662471	http://gnomad.b	3.08E-05	EAS	0.0001739330037	0	0	0	https://www.ncbi.nlm.nih.gov/clinvar/variation/283338/
NM_000051.3(ATM):c.4442C>T	concordant	unreportable	unreportable	216216	0	3	SCV000687549;S(	Uncertain significance;Unc	Color Genomics, In	2017-12-21;2017-1	ATM	chr11	108163351	108163351		2.78E-06				0	0	https://www.ncbi.nlm.nih.gov/clinvar/variation/216216/
NM_003060.3(SLC22A5):c.605T>C	concordant	unreportable	unreportable	460410	0	1	SCV000632559	Uncertain significance	Invitae	10/5/2017	SLC22A5	chr5	131719946	131719946	http://gnomad.b	0.0002202867116	AMR	0.0006848910125	1	1	0	https://www.ncbi.nlm.nih.gov/clinvar/variation/460410/
NM_000053.3(ATP7B):c.740A>G	concordant	unreportable	unreportable	312396	0	2	SCV000522449;S(	Uncertain significance;Unc	GeneDx;Illumina C	2017-11-28;2016-1	ATP7B	chr13	52548616	52548616	http://gnomad.b	0.0001655480482	NFE	0.0002427099971	0	0	0	https://www.ncbi.nlm.nih.gov/clinvar/variation/312396/
NM_000228.2(LAMB3):c.3404G>A	concordant	unreportable	unreportable	295062	0	1	SCV000353580	Uncertain significance	Illumina Clinical Se	10/18/2016	LAMB3	chr1	209788731	209788731	http://gnomad.b	0.0002351610573	FIN	0.001827729982	0	0	0	https://www.ncbi.nlm.nih.gov/clinvar/variation/295062/
NM_001042432.1(CLN3):c.616G>A	concordant	unreportable	unreportable	205118	0	1	SCV000240614	Uncertain significance	GeneDx	11/28/2017	CLN3	chr16	28497729	28497729	http://gnomad.b	0.0001222195062	EAS	0.0001744790061	0	0	0	https://www.ncbi.nlm.nih.gov/clinvar/variation/205118/
NM_003060.3(SLC22A5):c.760C>A	concordant	unreportable	unreportable	460415	0	1	SCV000632569	Benign	Invitae	10/5/2017	SLC22A5	chr5	131721127	131721127	http://gnomad.b	6.78E-05	FIN	0.004484300036	1	0	0	https://www.ncbi.nlm.nih.gov/clinvar/variation/460415/
NM_000051.3(ATM):c.3371A>T	concordant	unreportable	unreportable	233576	0	4	SCV000682123;S(	Uncertain significance;Unc	Color Genomics, In	2017-10-26;2017-1	ATM	chr11	108150304	108150304	http://gnomad.b	2.23E-05	AMR	2.98E-05	0	0	0	https://www.ncbi.nlm.nih.gov/clinvar/variation/233576/
NM_006493.2(CLN5):c.412G>A	concordant	unreportable	unreportable	205129	0	2	SCV000704082;S(	Uncertain significance;Unc	EGL Genetic Diagn	2017-06-30;2017-1	CLN5	chr13	77569289	77569289	http://gnomad.b	7.55E-05	AFR	0.0008494509966	0	0	0	https://www.ncbi.nlm.nih.gov/clinvar/variation/205129/
NM_138694.3(PKHD1):c.6687T>C	concordant	unreportable	unreportable	285503	0	1	SCV000338548	Uncertain significance	EGL Genetic Diagn	6/30/2017	PKHD1	chr6	51771134	51771134	http://gnomad.b	7.78E-05	AFR	0.0001309759973	0	0	0	https://www.ncbi.nlm.nih.gov/clinvar/variation/285503/
NM_138694.3(PKHD1):c.55C>T	concordant	unreportable	unreportable	500969	0	1	SCV000707146	Uncertain significance	EGL Genetic Diagn	6/30/2017	PKHD1	chr6	51948051	51948051	http://gnomad.b	8.90E-05	EAS	0.0003483920009	0	1	0	https://www.ncbi.nlm.nih.gov/clinvar/variation/500969/
NM_000352.3(ABCC8):c.4605C>T	concordant	unreportable	unreportable	303749	0	1	SCV000369227	Uncertain significance	Illumina Clinical Se	10/18/2016	ABCC8	chr11	17415247	17415247	http://gnomad.b	2.58E-06	SAS	3.25E-05	0	0	0	https://www.ncbi.nlm.nih.gov/clinvar/variation/303749/
NM_002485.4(NBN):c.105T>G	concordant	unreportable	unreportable	233580	0	2	SCV000685690;S(	Uncertain significance;Unc	Color Genomics, In	2017-10-26;2017-1	NBN	chr8	90995016	90995016	http://gnomad.b	2.23E-05	EAS	0.0005218000151	0	0	0	https://www.ncbi.nlm.nih.gov/clinvar/variation/233580/
NM_000051.3(ATM):c.3175G>A	concordant	unreportable	unreportable	127367	0	5	SCV000244216;S(	Uncertain significance;Unc	Ambry Genetics;Cc	2016-04-08;2017-1	ATM	chr11	108143470	108143470	http://gnomad.b	1.67E-05	NFE	8.96E-06	0	0	0	https://www.ncbi.nlm.nih.gov/clinvar/variation/127367/
NM_000391.3(TPP1):c.1281G>A	concordant	unreportable	unreportable	305507	0	1	SCV000373320	Uncertain significance	Illumina Clinical Se	10/18/2016	TPP1	chr11	6636546	6636546	http://gnomad.b	0.0001175147313	SAS	0.0001299459982	0	0	0	https://www.ncbi.nlm.nih.gov/clinvar/variation/305507/
NM_017890.4(VPS13B):c.406C>T	concordant	unreportable	unreportable	361037	0	1	SCV000470760	Uncertain significance	Illumina Clinical Se	10/18/2016	VPS13B	chr8	100108654	100108654	http://gnomad.b	8.40E-06	NFE	4.48E-05	0	0	0	https://www.ncbi.nlm.nih.gov/clinvar/variation/361037/
NM_000051.3(ATM):c.2610C>T	concordant	unreportable	unreportable	135745	0	3	SCV000682064;S(	Likely benign;Likely benign	Color Genomics, In	2017-10-26;2017-1	ATM	chr11	108138041	108138041	http://gnomad.b	5.57E-05	ASJ	0.0001015230009	0	0	0	https://www.ncbi.nlm.nih.gov/clinvar/variation/135745/
NM_000383.3(AIRE):c.426G>A	concordant	unreportable	unreportable	458619	0	1	SCV000629954	Likely benign	Invitae	10/5/2017	AIRE	chr21	45706979	45706979	http://gnomad.b	7.80E-05	AMR	0.0004178360105	0	0	0	https://www.ncbi.nlm.nih.gov/clinvar/variation/458619/
NM_000053.3(ATP7B):c.1760C>T	concordant	unreportable	unreportable	385010	0	1	SCV000526127	Likely benign	GeneDx	11/28/2017	ATP7B	chr13	52539117	52539117	http://gnomad.b	2.53E-05	AFR	6.54E-05	0	0	0	https://www.ncbi.nlm.nih.gov/clinvar/variation/385010/
NM_000466.2(PEX1):c.645C>G	concordant	unreportable	unreportable	197675	0	1	SCV000230929	Uncertain significance	EGL Genetic Diagn	6/30/2017	PEX1	chr7	92147184	92147184	http://gnomad.b	0.0001553130946	AFR	0.002548560034	0	0	0	https://www.ncbi.nlm.nih.gov/clinvar/variation/197675/
NM_000071.2(CBS):c.301C>G	concordant	unreportable	unreportable	340091	0	1	SCV000436231	Uncertain significance	Illumina Clinical Se	10/18/2016	CBS	chr21	44488634	44488634	http://gnomad.b	4.22E-05	ASJ	0.0003046299971	0	0	0	https://www.ncbi.nlm.nih.gov/clinvar/variation/340091/
NM_001173990.2(TMEM216):c.405G>	concordant	unreportable	unreportable	407338	0	1	SCV000546500	Uncertain significance	Invitae	3/14/2017	TMEM216	chr11	61165421	61165421	http://gnomad.b	5.66E-05	EAS	0.001217529993	0	0	0	https://www.ncbi.nlm.nih.gov/clinvar/variation/407338/
NM_017739.3(POMGNT1):c.1923A>C	concordant	unreportable	unreportable	288627	0	1	SCV000342794	Uncertain significance	EGL Genetic Diagn	6/30/2017	POMGNT1	chr1	46655002	46655002	http://gnomad.b	5.60E-06	NFE	2.71E-05	0	0	0	https://www.ncbi.nlm.nih.gov/clinvar/variation/288627/
NM_000642.2(AGL):c.767G>A	concordant	unreportable	unreportable	439395	0	2	SCV000602453;S(	Uncertain significance;Unc	ARUP Laboratories	2017-06-30;2017-1	AGL	chr1	100336058	100336058	http://gnomad.b	7.26E-05	NFE	8.97E-05	0	0	0	https://www.ncbi.nlm.nih.gov/clinvar/variation/439395/
NM_017890.4(VPS13B):c.5168G>T	concordant	unreportable	unreportable	361065	0	2	SCV000616273;S(	Uncertain significance;Unc	Athena Diagnostics	2017-08-17;2016-1	VPS13B	chr8	100589734	100589734	http://gnomad.b	3.36E-05	FIN	4.49E-05	0	0	0	https://www.ncbi.nlm.nih.gov/clinvar/variation/361065/
NM_000360.3(TH):c.1368C>T	concordant	unreportable	unreportable	304068	0	1	SCV000369909	Uncertain significance	Illumina Clinical Se	10/18/2016	TH	chr11	2185589	2185589	http://gnomad.b	0.000264802159	SAS	0.0008386449772	1	0	0	https://www.ncbi.nlm.nih.gov/clinvar/variation/304068/
NM_020533.2(MCOLN1):c.949G>A	concordant	unreportable	unreportable	330496	0	1	SCV000415612	Uncertain significance	Illumina Clinical Se	10/18/2016	MCOLN1	chr19	7593554	7593554	http://gnomad.b	0.0001260740078	SAS	0.0001624330034	0	0	0	https://www.ncbi.nlm.nih.gov/clinvar/variation/330496/
NM_000152.3(GAA):c.2274C>T	concordant	unreportable	unreportable	456397	0	1	SCV000626557	Likely benign	Invitae	10/5/2017	GAA	chr17	78090851	78090851	http://gnomad.b	4.17E-05	AFR	0.0001964380062	0	0	0	https://www.ncbi.nlm.nih.gov/clinvar/variation/456397/
NM_017739.3(POMGNT1):c.46C>T	concordant	unreportable	unreportable	286147	0	3	SCV000339467;S(	Uncertain significance;Unc	EGL Genetic Diagn	2017-06-30;2016-1	POMGNT1	chr1	46663448	46663448	http://gnomad.b	0.0001315796841	NFE	5.37E-05	0	0	0	https://www.ncbi.nlm.nih.gov/clinvar/variation/286147/
NM_000396.3(CTSK):c.244-10_244-8d	concordant	unreportable	unreportable	292586	0	1	SCV000348281	Uncertain significance	Illumina Clinical Se	10/18/2016	CTSK	chr1	150778499	150778502	http://gnomad.b	0.0004119648934	EAS	0.004464290105	0	0	0	https://www.ncbi.nlm.nih.gov/clinvar/variation/292586/
NM_001042432.1(CLN3):c.734_736de	concordant	unreportable	unreportable	205115	0	2	SCV000240611;S(	Uncertain significance;Unc	GeneDx;Invitae	2017-11-28;2017-1	CLN3	chr16	28495380	28495383	http://gnomad.b	0.0001055532099	AMR	0.0001500329963	0	0	0	https://www.ncbi.nlm.nih.gov/clinvar/variation/205115/
NM_000018.3(ACADVL):c.1005C>A	concordant	unreportable	unreportable	324992	0	1	SCV000406321	Uncertain significance	Illumina Clinical Se	10/18/2016	ACADVL	chr17	7126112	7126112	http://gnomad.b	6.51E-05	NFE	0.000125356004	0	0	0	https://www.ncbi.nlm.nih.gov/clinvar/variation/324992/
NM_003640.3(IKBKAP):c.2065C>T	concordant	unreportable	unreportable	246551	0	1	SCV000294141	Uncertain significance	GeneDx	11/28/2017	IKBKAP	chr9	111662605	111662605	http://gnomad.b	1.42E-05	SAS	3.25E-05	0	0	0	https://www.ncbi.nlm.nih.gov/clinvar/variation/246551/
NM_000023.2(SGCA):c.1063C>T	concordant	unreportable	unreportable	324045	0	1	SCV000403955	Uncertain significance	Illumina Clinical Se	10/18/2016	SGCA	chr17	48252697	48252697	http://gnomad.b	6.44E-05	NFE	6.47E-05	0	0	0	https://www.ncbi.nlm.nih.gov/clinvar/variation/324045/
NM_000051.3(ATM):c.8520G>C	concordant	unreportable	unreportable	221124	0	2	SCV000682492;S(	Uncertain significance;Unc	Color Genomics, In	2017-10-26;2016-0	ATM	chr11	108216571	108216571	http://gnomad.b	3.34E-05			0	0	0	https://www.ncbi.nlm.nih.gov/clinvar/variation/221124/
NM_017890.4(VPS13B):c.4626C>G	concordant	unreportable	unreportable	361061	0	1	SCV000470806	Uncertain significance	Illumina Clinical Se	10/18/2016	VPS13B	chr8	100523658	100523658	http://gnomad.b	0.000162378566	EAS	0.00373395998	1	0	0	https://www.ncbi.nlm.nih.gov/clinvar/variation/361061/
NM_017739.3(POMGNT1):c.1456C>G	concordant	unreportable	unreportable	291060	0	2	SCV000345743;S(	Uncertain significance;Unc	EGL Genetic Diagn	2017-06-30;2017-0	POMGNT1	chr1	46657853	46657853	http://gnomad.b	3.64E-05	NFE	1.79E-05	0	0	0	https://www.ncbi.nlm.nih.gov/clinvar/variation/291060/
NM_000035.3(ALDOB):c.956C>T	concordant	unreportable	unreportable	364308	0	1	SCV000476058	Uncertain significance	Illumina Clinical Se	10/18/2016	ALDOB	chr9	104187168	104187168	http://gnomad.b	3.67E-05	AMR	0.0001191330011	0	0	0	https://www.ncbi.nlm.nih.gov/clinvar/variation/364308/
NM_001360.2(DHCR7):c.522C>T	concordant	unreportable	unreportable	166987	0	1	SCV000202603	Uncertain significance	EGL Genetic Diagn	6/30/2017	DHCR7	chr11	71152377	71152377	http://gnomad.b	2.96E-05	AMR	2.98E-05	0	0	0	https://www.ncbi.nlm.nih.gov/clinvar/variation/166987/
NM_000051.3(ATM):c.8558C>T	concordant	unreportable	unreportable	142249	0	4	SCV000292153;S(	Uncertain significance;Unc	Color Genomics, In	2017-12-21;2017-1	ATM	chr11	108216609	108216609	http://gnomad.b	6.13E-05	EAS	0.000115969	0	0	0	https://www.ncbi.nlm.nih.gov/clinvar/variation/142249/
NM_000518.4(HBB):c.324C>T	concordant	unreportable	unreportable	36322	0	3	SCV000603938;S(	Likely benign;Uncertain sig	ARUP Laboratories	2017-06-30;2018-0	HBB	chr11	5246948	5246948	http://gnomad.b	8.86E-05	AFR	0.0009147939854	0	0	0	https://www.ncbi.nlm.nih.gov/clinvar/variation/36322/
NM_000051.3(ATM):c.3834C>A	concordant	unreportable	unreportable	216205	0	1	SCV000254092	Uncertain significance	Invitae	9/11/2015	ATM	chr11	108155041	108155041	http://gnomad.b	1.67E-05	NFE	8.96E-06	0	0	0	https://www.ncbi.nlm.nih.gov/clinvar/variation/216205/
NM_138694.3(PKHD1):c.3098-3C>T	concordant	unreportable	unreportable	500332	0	1	SCV000706227	Uncertain significance	EGL Genetic Diagn	6/30/2017	PKHD1	chr6	51900522	51900522	http://gnomad.b	4.73E-05	EAS	0.0006379029946	0	0	0	https://www.ncbi.nlm.nih.gov/clinvar/variation/500332/
NM_002485.4(NBN):c.1925A>G	concordant	unreportable	unreportable	141169	0	2	SCV000475289;S(	Uncertain significance;Unc	Illumina Clinical Se	2016-10-18;2016-0	NBN	chr8	90958513	90958513	http://gnomad.b	1.12E-05	AMR	8.93E-05	0	0	0	https://www.ncbi.nlm.nih.gov/clinvar/variation/141169/
NM_017890.4(VPS13B):c.1000T>C	concordant	unreportable	unreportable	437234	0	1	SCV000597872	Uncertain significance	Genetic Services La	7/5/2017	VPS13B	chr8	100133467	100133467	http://gnomad.b	4.48E-05	NFE	0.0002151619992	1	0	0	https://www.ncbi.nlm.nih.gov/clinvar/variation/437234/
NM_000051.3(ATM):c.5185G>C	concordant	unreportable	unreportable	127401	0	3	SCV000149115;S(	Uncertain significance;Unc	GeneDx;Invitae;Lal	2017-11-28;2017-1	ATM	chr11	108172382	108172382	http://gnomad.b	0.0001587283907	AMR	0.0001489069982	0	1	0	https://www.ncbi.nlm.nih.gov/clinvar/variation/127401/
NM_000136.2(FANCC):c.1560C>T	concordant	unreportable	unreportable	383837	0	2	SCV000524421;S(	Likely benign;Likely benign	GeneDx;Invitae	2017-11-28;2017-1	FANCC	chr9	97864106	97864106	http://gnomad.b	6.66E-05	EAS	5.80E-05	0	0	0	https://www.ncbi.nlm.nih.gov/clinvar/variation/383837/
NM_000051.3(ATM):c.6194T>C	concordant	unreportable	unreportable	230510	0	2	SCV000293095;S(	Uncertain significance;Unc	GeneDx;Invitae	2017-11-28;2017-1	ATM	chr11	108186836	108186836	http://gnomad.b	2.78E-05	AFR	0.0002617120044	0	0	0	https://www.ncbi.nlm.nih.gov/clinvar/variation/230510/
NM_000414.3(HSD17B4):c.950C>T	concordant	unreportable	unreportable	228741	0	2	SCV000452122;S(	Uncertain significance;Unc	Illumina Clinical Se	2016-10-18;2016-0	HSD17B4	chr5	118832319	118832319	http://gnomad.b	0.0003944350844	SAS	0.002274059923	3	2	0	https://www.ncbi.nlm.nih.gov/clinvar/variation/228741/
NM_000152.3(GAA):c.2418G>A	concordant	unreportable	unreportable	285734	0	1	SCV000338887	Uncertain significance	EGL Genetic Diagn	6/30/2017	GAA	chr17	78091485	78091485	http://gnomad.b	4.45E-05	SAS	0.0002927969908	0	0	0	https://www.ncbi.nlm.nih.gov/clinvar/variation/285734/
NM_017890.4(VPS13B):c.1529G>A	concordant	unreportable	unreportable	288755	0	2	SCV000342961;S(	Uncertain significance;Unc	EGL Genetic Diagn	2017-06-30;2017-0	VPS13B	chr8	100147927	100147927	http://gnomad.b	0.0001679778269	SAS	0.002080620034	3	0	0	https://www.ncbi.nlm.nih.gov/clinvar/variation/288755/
NM_138694.3(PKHD1):c.9106G>T	concordant	unreportable	unreportable	500858	0	1	SCV000706986	Uncertain significance	EGL Genetic Diagn	6/30/2017	PKHD1	chr6	51613308	51613308	http://gnomad.b	5.56E-06	AFR	0.0001307360071	0	0	0	https://www.ncbi.nlm.nih.gov/clinvar/variation/500858/
NM_000182.4(HADHA):c.955G>A	concordant	unreportable	unreportable	203743	0	1	SCV000238886	Uncertain significance	GeneDx	11/28/2017	HADHA	chr2	26435459	26435459	http://gnomad.b	2.80E-05	EAS	5.80E-05	0	0	0	https://www.ncbi.nlm.nih.gov/clinvar/variation/203743/
NM_000051.3(ATM):c.510G>A	concordant	unreportable	unreportable	186725	0	2	SCV000520798;S(	Likely benign;Likely benign	GeneDx;Invitae	2017-11-28;2017-1	ATM	chr11	108114693	108114693		8.35E-06				0	0	https://www.ncbi.nlm.nih.gov/clinvar/variation/186725/
NM_000057.2(BLM):c.1934A>G	concordant	unreportable	unreportable	405318	0	1	SCV000543382	Uncertain significance	Invitae	10/5/2017	BLM	chr15	91306247	91306247	http://gnomad.b	4.99E-05	AFR	0.0004584149865	0	0	0	https://www.ncbi.nlm.nih.gov/clinvar/variation/405318/
NM_000203.3(IDUA):c.1174C>T	concordant	unreportable	unreportable	350229	0	2	SCV000700304;S(	Benign;Uncertain significar	EGL Genetic Diagn	2017-06-30;2016-1	IDUA	chr4	996258	996258	http://gnomad.b	0.01149840099	NFE	0.0206259992	17	35	0	https://www.ncbi.nlm.nih.gov/clinvar/variation/350229/
NM_138694.3(PKHD1):c.711A>T	concordant	unreportable	unreportable	458605	0	1	SCV000629930	Likely benign	Invitae	10/5/2017	PKHD1	chr6	51934322	51934322	http://gnomad.b	6.95E-05	NFE	8.96E-05	0	0	0	https://www.ncbi.nlm.nih.gov/clinvar/variation/458605/
NM_001042432.1(CLN3):c.67C>T	concordant	unreportable	unreportable	409280	0	1	SCV000549228	Uncertain significance	Invitae	3/14/2017	CLN3	chr16	28502861	28502861	http://gnomad.b	1.94E-05	EAS	0.0002320450003	0	0	0	https://www.ncbi.nlm.nih.gov/clinvar/variation/409280/
NM_001079802.1(FKTN):c.374G>T	concordant	unreportable	unreportable	288016	0	3	SCV000341992;S(	Uncertain significance;Unc	EGL Genetic Diagn	2017-06-30;2016-1	FKTN	chr9	108366500	108366500	http://gnomad.b	5.32E-05	AMR	0.0001489690039	0	0	0	https://www.ncbi.nlm.nih.gov/clinvar/variation/288016/
NM_000414.3(HSD17B4):c.1317A>G	concordant	unreportable	unreportable	500197	0	1	SCV000706032	Uncertain significance	EGL Genetic Diagn	6/30/2017	HSD17B4	chr5	118842568	118842568	http://gnomad.b	9.86E-05	AMR	0.0002682399936	0	0	0	https://www.ncbi.nlm.nih.gov/clinvar/variation/500197/
NM_000203.3(IDUA):c.1345C>A	concordant	unreportable	unreportable	350230	0	2	SCV000700435;S(	Uncertain significance;Unc	EGL Genetic Diagn	2017-06-30;2016-1	IDUA	chr4	996675	996675	http://gnomad.b	0.002900972728	FIN	0.00594564015	0	1	0	https://www.ncbi.nlm.nih.gov/clinvar/variation/350230/
NM_000391.3(TPP1):c.65G>A	concordant	unreportable	unreportable	207559	0	1	SCV000548745	Uncertain significance	Invitae	3/14/2017	TPP1	chr11	6640451	6640451	http://gnomad.b	2.24E-05	NFE	4.48E-05	0	0	0	https://www.ncbi.nlm.nih.gov/clinvar/variation/207559/
NM_001360.2(DHCR7):c.28C>G	concordant	unreportable	unreportable	196325	0	1	SCV000228958	Uncertain significance	EGL Genetic Diagn	6/30/2017	DHCR7	chr11	71155971	71155971	http://gnomad.b	7.27E-05	AFR	0.0009147939854	0	0	0	https://www.ncbi.nlm.nih.gov/clinvar/variation/196325/
NM_004543.4(NEB):c.3399A>G	concordant	unreportable	unreportable	331521	0	1	SCV000417020	Uncertain significance	Illumina Clinical Se	10/18/2016	NEB	chr2	152534558	152534558	http://gnomad.b	0.0002478340338	SAS	0.003119309898	0	0	0	https://www.ncbi.nlm.nih.gov/clinvar/variation/331521/
NM_024685.3(BBS10):c.1974T>C	concordant	unreportable	unreportable	310488	0	1	SCV000381145	Uncertain significance	Illumina Clinical Se	10/18/2016	BBS10	chr12	76739791	76739791	http://gnomad.b	3.08E-05	NFE	2.69E-05	0	0	0	https://www.ncbi.nlm.nih.gov/clinvar/variation/310488/
NM_001042432.1(CLN3):c.516C>T	concordant	unreportable	unreportable	136789	0	1	SCV000628933	Likely benign	Invitae	10/5/2017	CLN3	chr16	28497916	28497916	http://gnomad.b	0.0002027732717	SAS	0.001234570052	0	1	0	https://www.ncbi.nlm.nih.gov/clinvar/variation/136789/
NM_000051.3(ATM):c.921A>T	concordant	unreportable	unreportable	489603	0	1	SCV000682542	Uncertain significance	Color Genomics, In	10/26/2017	ATM	chr11	108117710	108117710		5.57E-06				0	0	https://www.ncbi.nlm.nih.gov/clinvar/variation/489603/
NM_000018.3(ACADVL):c.1273G>A	concordant	unreportable	unreportable	92273	1	1	SCV000109731;S(	Uncertain significance;Likel	EGL Genetic Diagn	2017-06-30;2017-1	ACADVL	chr17	7127135	7127135	http://gnomad.b	3.11E-05	AFR	0.0005227390211	0	0	0	https://www.ncbi.nlm.nih.gov/clinvar/variation/92273/
NM_000051.3(ATM):c.7223C>T	concordant	unreportable	unreportable	181888	0	4	SCV000682396;S(	Uncertain significance;Unc	Color Genomics, In	2017-10-26;2017-1	ATM	chr11	108199881	108199881	http://gnomad.b	2.78E-06	SAS	3.25E-05	0	0	0	https://www.ncbi.nlm.nih.gov/clinvar/variation/181888/
NM_000057.3:c.3397A>G	concordant	unreportable	unreportable	127498	0	1	SCV000623312	Uncertain significance	Invitae	10/5/2017	BLM	chr15	91346789	91346789	http://gnomad.b	3.57E-05	AFR	0.0003267120046	0	0	0	https://www.ncbi.nlm.nih.gov/clinvar/variation/127498/
NM_000051.3(ATM):c.2254C>G	concordant	unreportable	unreportable	185861	0	1	SCV000682044	Uncertain significance	Color Genomics, In	10/26/2017	ATM	chr11	108128211	108128211		8.35E-06				0	0	https://www.ncbi.nlm.nih.gov/clinvar/variation/185861/
NM_000152.3(GAA):c.2064C>T	concordant	unreportable	unreportable	287992	0	2	SCV000341965;S(	Uncertain significance;Unc	EGL Genetic Diagn	2017-06-30;2016-1	GAA	chr17	78087040	78087040		1.95E-05				0	0	https://www.ncbi.nlm.nih.gov/clinvar/variation/287992/
NM_000051.3(ATM):c.2689T>A	concordant	unreportable	unreportable	188232	0	2	SCV000566429;S(	Uncertain significance;Unc	GeneDx;Invitae	2017-11-28;2017-1	ATM	chr11	108139187	108139187	http://gnomad.b	2.51E-05	NFE	6.27E-05	0	0	0	https://www.ncbi.nlm.nih.gov/clinvar/variation/188232/
NM_000050.4(ASS1):c.294C>T	concordant	unreportable	unreportable	495382	0	1	SCV000694163	Uncertain significance	Laboratory Corpora	1/25/2018	ASS1	chr9	133333907	133333907	http://gnomad.b	3.06E-05	EAS	0.0002898889943	0	0	0	https://www.ncbi.nlm.nih.gov/clinvar/variation/495382/
NM_000098.2(CPT2):c.1519G>A	concordant	unreportable	unreportable	429623	0	1	SCV000582237	Uncertain significance	GeneDx	11/28/2017	CPT2	chr1	53676865	53676865	http://gnomad.b	0.0001427028479	EAS	0.001457049977	0	0	0	https://www.ncbi.nlm.nih.gov/clinvar/variation/429623/
NM_001360.2(DHCR7):c.376G>A	concordant	unreportable	unreportable	197725	0	2	SCV000231006;S(	Uncertain significance;Unc	EGL Genetic Diagn	2017-06-30;2016-1	DHCR7	chr11	71153345	71153345	http://gnomad.b	0.0001292434947	AFR	0.001380310045	0	1	0	https://www.ncbi.nlm.nih.gov/clinvar/variation/197725/
NM_014363.4(SACS):c.4900G>C	concordant	unreportable	unreportable	436632	0	2	SCV000596919;S(	Uncertain significance;Unc	Genetic Services La	2017-07-05;2017-1	SACS	chr13	23913115	23913115	http://gnomad.b	4.74E-05	AFR	0.0003270109883	0	0	0	https://www.ncbi.nlm.nih.gov/clinvar/variation/436632/
NM_002485.4(NBN):c.1973A>G	concordant	unreportable	unreportable	141979	0	2	SCV000292182;S(	Uncertain significance;Unc	Color Genomics, In	2017-12-21;2017-1	NBN	chr8	90958465	90958465	http://gnomad.b	1.39E-05	NFE	8.97E-06	0	0	0	https://www.ncbi.nlm.nih.gov/clinvar/variation/141979/
NM_000243.2(MEFV):c.1795A>G	concordant	unreportable	unreportable	97464	0	1	SCV000279053	Uncertain significance	GeneDx	11/28/2017	MEFV	chr16	3293692	3293692	http://gnomad.b	0.0001175482788	AFR	0.0008561640279	1	0	0	https://www.ncbi.nlm.nih.gov/clinvar/variation/97464/
NM_000051.3(ATM):c.3219A>G	concordant	unreportable	unreportable	184040	0	2	SCV000682109;S(	Likely benign;Likely benign	Color Genomics, In	2017-10-26;2017-1	ATM	chr11	108143514	108143514	http://gnomad.b	2.51E-05	NFE	2.69E-05	0	0	0	https://www.ncbi.nlm.nih.gov/clinvar/variation/184040/
NM_000543.4(SMPD1):c.785_807del2	concordant	reportable	reportable	195086	1	0	SCV000227145	Pathogenic	EGL Genetic Diagn	6/30/2017	SMPD1	chr11	6413075	6413098		1.43E-05				0	0	https://www.ncbi.nlm.nih.gov/clinvar/variation/195086/
NM_000153.3(GALC):c.81C>T	concordant	unreportable	unreportable	314761	0	1	SCV000389267	Uncertain significance	Illumina Clinical Se	10/18/2016	GALC	chr14	88459428	88459428	http://gnomad.b	0.0001034918717	SAS	0.0003110420075	0	0	0	https://www.ncbi.nlm.nih.gov/clinvar/variation/314761/
NM_138694.3(PKHD1):c.8181C>G	concordant	unreportable	unreportable	357425	0	1	SCV000464053	Uncertain significance	Illumina Clinical Se	10/18/2016	PKHD1	chr6	51695780	51695780	http://gnomad.b	3.89E-05	NFE	8.98E-06	0	0	0	https://www.ncbi.nlm.nih.gov/clinvar/variation/357425/
NM_000108.3(DLD):c.282C>T	concordant	unreportable	unreportable	377772	0	1	SCV000512804	Likely benign	GeneDx	11/28/2017	DLD	chr7	107543937	107543937	http://gnomad.b	4.41E-05	AFR	0.0004587759904	0	0	0	https://www.ncbi.nlm.nih.gov/clinvar/variation/377772/
NM_000391.3(TPP1):c.947C>A	concordant	unreportable	unreportable	426392	0	1	SCV000576818	Uncertain significance	GeneDx	11/28/2017	TPP1	chr11	6637674	6637674	http://gnomad.b	0.0001398984897	ASJ	0.0001015430025	0	0	0	https://www.ncbi.nlm.nih.gov/clinvar/variation/426392/
NM_000071.2(CBS):c.609C>T	concordant	unreportable	unreportable	264194	0	2	SCV000319942;S(	Likely benign;Likely benign	Ambry Genetics;In	2017-11-14;2017-1	CBS	chr21	44485554	44485554	http://gnomad.b	4.22E-05	NFE	6.34E-05	0	0	0	https://www.ncbi.nlm.nih.gov/clinvar/variation/264194/
NM_138694.3(PKHD1):c.5235C>T	concordant	unreportable	unreportable	458599	0	2	SCV000707899;S(	Uncertain significance;Likel	EGL Genetic Diagn	2017-06-30;2017-1	PKHD1	chr6	51889373	51889373	http://gnomad.b	0.0001612383102	AFR	0.00032968499	0	2	0	https://www.ncbi.nlm.nih.gov/clinvar/variation/458599/

Supplemental Table 2

NM_138694.3(PKHD1):c.1974C>T	concordant	unreportable	unreportable	288693	0		2	SCV000342882;S	Uncertain significance;Unc	EGL Genetic Diagn	2017-06-30;2016-1	PKHD1	chr6	51918040	51918040	http://gnomad.b	2.50E-05	AMR	0.0002085070009	0		0	0	https://www.ncbi.nlm.nih.gov/clinvar/variation/288693/
NM_138694.3(PKHD1):c.1893C>G	concordant	unreportable	unreportable	357447	0		1	SCV000464112	Uncertain significance	Illumina Clinical Se	10/18/2016	PKHD1	chr6	51918907	51918907	http://gnomad.b	5.56E-06	NFE	2.69E-05	0		0	0	https://www.ncbi.nlm.nih.gov/clinvar/variation/357447/
NM_001042432.1(CLN3):c.250C>A	concordant	unreportable	unreportable	197711	0		1	SCV000573068	Uncertain significance	GeneDx	11/28/2017	CLN3	chr16	28499956	28499956	http://gnomad.b	2.50E-05	AFR	0.0006534240092	0		0	0	https://www.ncbi.nlm.nih.gov/clinvar/variation/197711/
NM_000051.3(ATM):c.3242A>G	concordant	unreportable	unreportable	142046	0		4	SCV000611358;S	Uncertain significance;Unc	Fulgent Genetics,F	2017-05-23;2017-1	ATM	chr11	108143537	108143537	http://gnomad.b	0.0001698672251	SAS	0.00133203005	0		0	0	https://www.ncbi.nlm.nih.gov/clinvar/variation/142046/
NM_000391.3(TPP1):c.1280C>T	concordant	unreportable	unreportable	207590	0		1	SCV000243435	Uncertain significance	GeneDx	11/28/2017	TPP1	chr11	6636547	6636547	http://gnomad.b	0.0001063228521	AFR	0.000326754991	0		0	0	https://www.ncbi.nlm.nih.gov/clinvar/variation/207590/
NM_017890.4(VPS13B):c.3656C>G	concordant	unreportable	unreportable	287997	0		1	SCV000341970	Uncertain significance	EGL Genetic Diagn	6/30/2017	VPS13B	chr8	100479852	100479852	http://gnomad.b	0.000218371175	AFR	0.002549020108	1		0	0	https://www.ncbi.nlm.nih.gov/clinvar/variation/287997/
NM_000391.3(TPP1):c.532C>G	concordant	unreportable	unreportable	207552	0		1	SCV000243390	Uncertain significance	GeneDx	11/28/2017	TPP1	chr11	6638361	6638361	http://gnomad.b	2.80E-05	NFE	1.79E-05	0		0	0	https://www.ncbi.nlm.nih.gov/clinvar/variation/207552/
NM_001876.3(CPT1A):c.693C>T	concordant	unreportable	unreportable	386076	0		1	SCV000527594	Likely benign	GeneDx	11/28/2017	CPT1A	chr11	68566686	68566686	http://gnomad.b	1.69E-05	NFE	2.69E-05	0		0	0	https://www.ncbi.nlm.nih.gov/clinvar/variation/386076/
NM_001042432.1(CLN3):c.206C>T	concordant	unreportable	unreportable	205104	0		2	SCV000240598;S	Uncertain significance;Unc	GeneDx;Invitae	2017-11-28;2017-1	CLN3	chr16	28500627	28500627	http://gnomad.b	8.61E-05	AMR	0.0002092429932	0		0	0	https://www.ncbi.nlm.nih.gov/clinvar/variation/205104/
NM_138694.3(PKHD1):c.434C>G	concordant	unreportable	unreportable	290856	0		1	SCV000345506	Uncertain significance	EGL Genetic Diagn	6/30/2017	PKHD1	chr6	51941088	51941088	http://gnomad.b	3.34E-05	NFE	5.38E-05	0		0	0	https://www.ncbi.nlm.nih.gov/clinvar/variation/290856/
NM_000071.2(CBS):c.612G>T	concordant	unreportable	unreportable	340086	0		1	SCV000436223	Uncertain significance	Illumina Clinical Se	10/18/2016	CBS	chr21	44485551	44485551	http://gnomad.b	0.0001124802457	EAS	0.0008708779933	0		0	0	https://www.ncbi.nlm.nih.gov/clinvar/variation/340086/
NM_002485.4(NBN):c.1089C>T	concordant	unreportable	unreportable	182706	0		3	SCV000685692;S	Likely benign;Uncertain sig	Color Genomics, In	2017-10-26;2017-0	NBN	chr8	90970988	90970988	http://gnomad.b	6.13E-05	AFR	0.0002614379919	0		0	0	https://www.ncbi.nlm.nih.gov/clinvar/variation/182706/
NM_000152.3(GAA):c.1504A>G	concordant	unreportable	unreportable	439746	0		3	SCV000603776;S	Uncertain significance;Unc	ARUP Laboratories	2017-06-30;2017-0	GAA	chr17	78084592	78084592	http://gnomad.b	7.50E-05	NFE	9.85E-05	0		0	0	https://www.ncbi.nlm.nih.gov/clinvar/variation/439746/
NM_000108.3(DLD):c.1503G>A	concordant	unreportable	unreportable	358572	0		1	SCV000466251	Uncertain significance	Illumina Clinical Se	10/18/2016	DLD	chr7	107559677	107559677	http://gnomad.b	7.79E-06	AFR	6.58E-05	0		0	0	https://www.ncbi.nlm.nih.gov/clinvar/variation/358572/
NM_000018.3(ACADVL):c.1827+8T>C	concordant	unreportable	unreportable	384235	0		1	SCV000524958	Likely benign	GeneDx	11/28/2017	ACADVL	chr17	7128211	7128211	http://gnomad.b	2.83E-06	NFE	1.79E-05	0		0	0	https://www.ncbi.nlm.nih.gov/clinvar/variation/384235/
NM_000152.3(GAA):c.2330C>T	concordant	unreportable	unreportable	325793	0		2	SCV000407297;S	Uncertain significance;Unc	Illumina Clinical Se	2016-10-18;2017-1	GAA	chr17	78090907	78090907	http://gnomad.b	1.11E-05	AFR	6.58E-05	0		0	0	https://www.ncbi.nlm.nih.gov/clinvar/variation/325793/
NM_000152.3(GAA):c.1347G>A	concordant	unreportable	unreportable	288681	0		1	SCV000342863	Uncertain significance	EGL Genetic Diagn	6/30/2017	GAA	chr17	78083764	78083764	http://gnomad.b	1.95E-05	ASJ	0.000101812002	0		0	0	https://www.ncbi.nlm.nih.gov/clinvar/variation/288681/
NM_000137.2(FAH):c.648C>A	concordant	unreportable	unreportable	502070	0		1	SCV000708667	Uncertain significance	EGL Genetic Diagn	6/30/2017	FAH	chr15	80464532	80464532	http://gnomad.b	0.0001886467922	AMR	0.0002084449952	0		0	0	https://www.ncbi.nlm.nih.gov/clinvar/variation/502070/
NM_000232.4(SGCB):c.369T>C	concordant	unreportable	unreportable	288275	0		1	SCV000342342	Uncertain significance	EGL Genetic Diagn	6/30/2017	SGCB	chr4	52895904	52895904	http://gnomad.b	2.24E-05	NFE	1.79E-05	0		0	0	https://www.ncbi.nlm.nih.gov/clinvar/variation/288275/
NM_138694.3(PKHD1):c.11007G>A	concordant	unreportable	unreportable	287505	0		1	SCV000341294	Uncertain significance	EGL Genetic Diagn	6/30/2017	PKHD1	chr6	51523917	51523917	http://gnomad.b	0.0001834780771	AMR	0.0001493699965	0		0	0	https://www.ncbi.nlm.nih.gov/clinvar/variation/287505/
NM_004543.4(NEB):c.6088C>T	concordant	unreportable	unreportable	465622	0		2	SCV000703689;S	Uncertain significance;Likel	EGL Genetic Diagn	2017-06-30;2017-1	NEB	chr2	152514592	152514592	http://gnomad.b	0.0001781307118	AFR	0.002033089986	0		0	0	https://www.ncbi.nlm.nih.gov/clinvar/variation/465622/
NM_000152.3(GAA):c.2332-1G>C	concordant	reportable	reportable	456400	1		0	SCV000626565	Likely pathogenic	Invitae	10/5/2017	GAA	chr17	78091398	78091398		5.56E-06					0	0	https://www.ncbi.nlm.nih.gov/clinvar/variation/456400/
NM_138694.3(PKHD1):c.3241C>T	concordant	unreportable	unreportable	96395	0		1	SCV000114587	Uncertain significance	EGL Genetic Diagn	6/30/2017	PKHD1	chr6	51897951	51897951	http://gnomad.b	0.0002418574653	EAS	0.0004642530112	0		0	0	https://www.ncbi.nlm.nih.gov/clinvar/variation/96395/
NM_017890.4(VPS13B):c.4832A>G	concordant	unreportable	unreportable	167830	0		1	SCV000630876	Uncertain significance	Invitae	10/5/2017	VPS13B	chr8	100568689	100568689	http://gnomad.b	7.28E-05	AFR	0.001181259984	0		0	0	https://www.ncbi.nlm.nih.gov/clinvar/variation/167830/
NM_001360.2(DHCR7):c.139C>T	concordant	unreportable	unreportable	283587	0		1	SCV000335785	Uncertain significance	EGL Genetic Diagn	6/30/2017	DHCR7	chr11	71155221	71155221	http://gnomad.b	4.85E-05	AFR	0.000869060983	0		0	0	https://www.ncbi.nlm.nih.gov/clinvar/variation/283587/
NM_000441.1(SLC26A4):c.1616T>C	concordant	unreportable	unreportable	229253	0		1	SCV000272436	Uncertain significance	Laboratory for Mol	4/26/2016	SLC26A4	chr7	107340529	107340529	http://gnomad.b	0.0001926524048	AFR	0.002354480093	0		0	0	https://www.ncbi.nlm.nih.gov/clinvar/variation/229253/
NM_003060.3(SLC22A5):c.592G>A	concordant	unreportable	unreportable	460409	0		1	SCV000632557	Uncertain significance	Invitae	10/5/2017	SLC22A5	chr5	131719933	131719933	http://gnomad.b	1.69E-05	SAS	0.0001624330034	0		0	0	https://www.ncbi.nlm.nih.gov/clinvar/variation/460409/
NM_138694.3(PKHD1):c.2093G>A	concordant	unreportable	unreportable	290963	0		1	SCV000345633	Uncertain significance	EGL Genetic Diagn	6/30/2017	PKHD1	chr6	51917921	51917921	http://gnomad.b	0.0001306586307	NFE	0.0001435239974	0		0	0	https://www.ncbi.nlm.nih.gov/clinvar/variation/290963/
NM_000023.2(SGCA):c.1076C>A	concordant	unreportable	unreportable	500012	0		1	SCV000705776	Uncertain significance	EGL Genetic Diagn	6/30/2017	SGCA	chr17	48252710	48252710	http://gnomad.b	6.16E-05	NFE	0.0001456559985	0		0	0	https://www.ncbi.nlm.nih.gov/clinvar/variation/500012/
NM_000492.3(CFTR):c.2392C>T	concordant	unreportable	unreportable	35841	0		2	SCV000700434;S	Uncertain significance;Unc	EGL Genetic Diagn	2017-06-30;2018-0	CFTR	chr7	117232613	117232613	http://gnomad.b	4.24E-05	AMR	4.90E-05	0		0	0	https://www.ncbi.nlm.nih.gov/clinvar/variation/35841/
NM_017890.4(VPS13B):c.11189A>T	concordant	unreportable	unreportable	437258	0		1	SCV000597921	Uncertain significance	Genetic Services La	7/5/2017	VPS13B	chr8	100874073	100874073	http://gnomad.b	5.88E-05	AFR	0.001019450021	0		0	0	https://www.ncbi.nlm.nih.gov/clinvar/variation/437258/
NM_017739.3(POMGNT1):c.1786-6C>	concordant	unreportable	unreportable	497582	0		1	SCV000702181	Uncertain significance	EGL Genetic Diagn	6/30/2017	POMGNT1	chr1	46655245	46655245	http://gnomad.b	7.28E-05	AFR	0.001046319958	0		0	0	https://www.ncbi.nlm.nih.gov/clinvar/variation/497582/
NM_002225.3(IVD):c.507delG	concordant	reportable	reportable	94056	1		0	SCV000631889	Pathogenic	Invitae	10/5/2017	IVD	chr15	40703499	40703500	http://gnomad.b	1.40E-05	NFE	3.58E-05	0		0	0	https://www.ncbi.nlm.nih.gov/clinvar/variation/94056/
NM_000051.3(ATM):c.7358G>A	concordant	unreportable	unreportable	140905	0		2	SCV000209642;S	Uncertain significance;Unc	GeneDx;Invitae	2017-11-28;2017-1	ATM	chr11	108200991	108200991	http://gnomad.b	1.11E-05	SAS	0.0002274059952	0		0	0	https://www.ncbi.nlm.nih.gov/clinvar/variation/140905/
NM_017739.3(POMGNT1):c.1933G>C	concordant	unreportable	unreportable	289586	0		1	SCV000343970	Uncertain significance	EGL Genetic Diagn	6/30/2017	POMGNT1	chr1	46654992	46654992	http://gnomad.b	1.12E-05	NFE	4.53E-05	0		0	0	https://www.ncbi.nlm.nih.gov/clinvar/variation/289586/
NM_000152.3(GAA):c.2069C>T	concordant	unreportable	unreportable	424308	0		2	SCV000574105;S	Uncertain significance;Unc	GeneDx;Invitae	2017-11-28;2017-1	GAA	chr17	78087045	78087045		2.50E-05					0	0	https://www.ncbi.nlm.nih.gov/clinvar/variation/424308/
NM_006493.2(CLN5):c.166A>G	concordant	unreportable	unreportable	409273	0		1	SCV000549218	Uncertain significance	Invitae	10/5/2017	CLN5	chr13	77566252	77566252	http://gnomad.b	3.92E-05	AFR	0.0005793740274	0		0	0	https://www.ncbi.nlm.nih.gov/clinvar/variation/409273/
NM_004543.4(NEB):c.585C>G	concordant	unreportable	unreportable	331540	0		1	SCV000417053	Uncertain significance	Illumina Clinical Se	10/18/2016	NEB	chr2	152580801	152580801	http://gnomad.b	4.13E-05	NFE	6.29E-05	0		0	0	https://www.ncbi.nlm.nih.gov/clinvar/variation/331540/
NM_000391.3(TPP1):c.123G>A	concordant	unreportable	unreportable	378754	0		1	SCV000514956	Likely benign	GeneDx	11/28/2017	TPP1	chr11	6640113	6640113	http://gnomad.b	3.08E-05	AMR	8.93E-05	0		0	0	https://www.ncbi.nlm.nih.gov/clinvar/variation/378754/
NM_138694.3(PKHD1):c.2378G>A	concordant	unreportable	unreportable	498827	0		1	SCV000704039	Uncertain significance	EGL Genetic Diagn	6/30/2017	PKHD1	chr6	51913319	51913319	http://gnomad.b	3.06E-05	SAS	6.50E-05	0		0	0	https://www.ncbi.nlm.nih.gov/clinvar/variation/498827/
NM_000136.2(FANCC):c.800A>G	concordant	unreportable	unreportable	182478	0		2	SCV000211044;S	Uncertain significance;Unc	GeneDx;Invitae	2017-11-28;2017-0	FANCC	chr9	97897671	97897671	http://gnomad.b	2.14E-05	NFE	1.79E-05	0		0	0	https://www.ncbi.nlm.nih.gov/clinvar/variation/182478/
NM_003060.3(SLC22A5):c.1142T>C	concordant	unreportable	unreportable	460391	0		1	SCV000632513	Uncertain significance	Invitae	10/5/2017	SLC22A5	chr5	131726471	131726471	http://gnomad.b	0.0001694513166	AFR	0.002090960043	0		0	0	https://www.ncbi.nlm.nih.gov/clinvar/variation/460391/
NM_000228.2(LAMB3):c.2564C>G	concordant	unreportable	unreportable	295085	0		1	SCV000353607	Uncertain significance	Illumina Clinical Se	10/18/2016	LAMB3	chr1	209796018	209796018	http://gnomad.b	6.72E-05	NFE	8.96E-05	0		0	0	https://www.ncbi.nlm.nih.gov/clinvar/variation/295085/
NM_002225.3(IVD):c.832G>A	concordant	unreportable	unreportable	373346	0		1	SCV000491933	Uncertain significance	GeneDx	12/21/2016	IVD	chr15	40707126	40707126	http://gnomad.b	8.41E-06	NFE	8.96E-06	0		0	0	https://www.ncbi.nlm.nih.gov/clinvar/variation/373346/
NM_000051.3(ATM):c.5734G>A	concordant	unreportable	unreportable	490619	0		1	SCV000687637	Uncertain significance	Color Genomics, In	12/21/2017	ATM	chr11	108178683	108178683	http://gnomad.b	5.57E-06			0		0	0	https://www.ncbi.nlm.nih.gov/clinvar/variation/490619/
NM_000152.3(GAA):c.545C>G	concordant	unreportable	unreportable	499380	0		1	SCV000704834	Uncertain significance	EGL Genetic Diagn	6/30/2017	GAA	chr17	78078930	78078930	http://gnomad.b	5.84E-05	NFE	0.0001048179984	0		0	0	https://www.ncbi.nlm.nih.gov/clinvar/variation/499380/
NM_000271.4(NPC1):c.2820G>A	concordant	unreportable	unreportable	326256	0		2	SCV000709366;S	Uncertain significance;Unc	EGL Genetic Diagn	2017-06-30;2016-1	NPC1	chr18	21119410	21119410	http://gnomad.b	5.43E-05	AMR	8.95E-05	0		0	0	https://www.ncbi.nlm.nih.gov/clinvar/variation/326256/
NM_000057.2(BLM):c.2739C>T	concordant	unreportable	unreportable	317407	0		2	SCV000394421;S	Uncertain significance;Likel	Illumina Clinical Se	2016-10-18;2017-1	BLM	chr15	91328227	91328227	http://gnomad.b	9.28E-05	AMR	0.0007742699818	0		0	0	https://www.ncbi.nlm.nih.gov/clinvar/variation/317407/
NM_000051.3(ATM):c.4980C>T	concordant	unreportable	unreportable	135758	0		3	SCV000537419;S	Likely benign;Likely benign	Color Genomics, In	2017-01-26;2017-1	ATM	chr11	108168084	108168084	http://gnomad.b	0.0002896096952	AMR	0.0004468540137	0		0	0	https://www.ncbi.nlm.nih.gov/clinvar/variation/135758/
NM_000518.4(HBB):c.157G>C	concordant	unreportable	unreportable	15362	0		1	SCV000603922	Uncertain significance	ARUP Laboratories	6/30/2017	HBB	chr11	5247965	5247965		2.33E-06					0	0	https://www.ncbi.nlm.nih.gov/clinvar/variation/15362/
NM_015120.4(ALMS1):c.1868A>G	concordant	unreportable	unreportable	35676	0		1	SCV000431909	Likely benign	Illumina Clinical Se	10/18/2016	ALMS1	chr2	73675519	73675519		8.00E-06					0	0	https://www.ncbi.nlm.nih.gov/clinvar/variation/35676/
NM_000497.3(CYP11B1):c.281C>T	concordant	reportable	reportable	1186	1		0	SCV000613039	Pathogenic	Athena Diagnostics	8/17/2017	CYP11B1	chr8	143960562	143960562	http://gnomad.b	2.42E-05	SAS	3.25E-05	0		0	0	https://www.ncbi.nlm.nih.gov/clinvar/variation/1186/
NM_000169.2(GLA):c.937G>T	concordant	unreportable	unreportable	10738	0		4	SCV000318693;S	Likely benign;Likely benign	Ambry Genetics;EG	2017-11-14;2017-0	GLA	chrX	100653420	100653420	http://gnomad.b	0.003651367083	ASJ	0.007128170226	2	242	1	75	https://www.ncbi.nlm.nih.gov/clinvar/variation/10738/
NM_000169.2(GLA):c.978G>A	concordant	unreportable	unreportable	42466	0		2	SCV000283693;S	Benign;Benign	Invitae;Laboratory	2017-10-05;2016-0	GLA	chrX	100653379	100653379	http://gnomad.b	0.0003099303625	AFR	0.00285807997	0	18	0	5	https://www.ncbi.nlm.nih.gov/clinvar/variation/42466/
NM_000260.3(MYO7A):c.1007G>A	concordant	unreportable	unreportable	43134	0		2	SCV000339775;S	Likely benign;Likely benign	EGL Genetic Diagn	2017-06-30;2016-0	MYO7A	chr11	76870496	76870496	http://gnomad.b	0.001464257709	NFE	0.002183289966	0		0	0	https://www.ncbi.nlm.nih.gov/clinvar/variation/43134/
NM_000260.3(MYO7A):c.2283G>A	concordant	unreportable	unreportable	43179	0		1	SCV000374279	Uncertain significance	Illumina Clinical Se	10/18/2016	MYO7A	chr11	76890091	76890091	http://gnomad.b	0.0005761013938	NFE	0.0009627420222	0		0	0	https://www.ncbi.nlm.nih.gov/clinvar/variation/43179/
NM_000260.3(MYO7A):c.4620G>A	concordant	unreportable	unreportable	43251	0		2	SCV000374385;S	Likely benign;Benign	Illumina Clinical Se	2016-10-18;2016-0	MYO7A	chr11	76910631	76910631	http://gnomad.b	0.00160028165	SAS	0.008054180071	2		0	0	https://www.ncbi.nlm.nih.gov/clinvar/variation/43251/
NM_000260.3(MYO7A):c.4805G>A	concordant	unreportable	unreportable	43260	0		2	SCV000702938;S	Likely benign;Likely benign	EGL Genetic Diagn	2017-06-30;2016-1	MYO7A	chr11	76910816	76910816	http://gnomad.b	0.004184736514	EAS	0.0481753014	26		9	0	https://www.ncbi.nlm.nih.gov/clinvar/variation/43260/
NM_000260.3(MYO7A):c.4983C>T	concordant	unreportable	unreportable	43267	0		1	SCV000059824	Likely benign	Laboratory for Mol	4/26/2016	MYO7A	chr11	76912623	76912623	http://gnomad.b	8.80E-05	NFE	9.85E-05	0		0	0	https://www.ncbi.nlm.nih.gov/clinvar/variation/43267/
NM_000260.3(MYO7A):c.510G>A	concordant	unreportable	unreportable	43272	0		1	SCV000340588	Likely benign	EGL Genetic Diagn	11/3/2016	MYO7A	chr11	76867745	76867745	http://gnomad.b	0.003736657652	ASJ	0.01360409986	6		3	0	https://www.ncbi.nlm.nih.gov/clinvar/variation/43272/
NM_000260.3(MYO7A):c.5904C>T	concordant	unreportable	unreportable	43309	0		1	SCV000374494	Uncertain significance	Illumina Clinical Se	10/18/2016	MYO7A	chr11	76919522	76919522	http://gnomad.b	0.001016178847	SAS	0.001758730039	3		0	0	https://www.ncbi.nlm.nih.gov/clinvar/variation/43309/
NM_000441.1(SLC26A4):c.294_298del	concordant	reportable	reportable	43551	1		0	SCV000060142	Pathogenic	Laboratory for Mol	4/26/2016	SLC26A4	chr7	107303867	107303872		5.58E-06					0	0	https://www.ncbi.nlm.nih.gov/clinvar/variation/43551/
NM_153676.3(USH1C):c.1823C>G	concordant	unreportable	unreportable	5148	0		1	SCV000605534	Uncertain significance	ARUP Laboratories	6/30/2017	USH1C	chr11	17531093	17531093	http://gnomad.b	0.0005840747616	NFE	0.0008223379846	0		0	0	https://www.ncbi.nlm.nih.gov/clinvar/variation/5148/
NM_153676.3(USH1C):c.648G>A	concordant	unreportable	unreportable	48023	0		2	SCV000232690;S	Likely benign;Uncertain sig	EGL Genetic Diagn	2017-06-30;2016-1	USH1C	chr11	17547920	17547920	http://gnomad.b	0.002264289829	EAS	0.01716939919	3		7	0	https://www.ncbi.nlm.nih.gov/clinvar/variation/48023/
NM_206933.2(USH2A):c.11225A>G	concordant	unreportable	unreportable	48368	0		1	SCV000704998	Uncertain significance	EGL Genetic Diagn	6/30/2017	USH2A	chr1	215933008	215933008	http://gnomad.b	0.0001764205867	AFR	0.002221930074	0		0	0	https://www.ncbi.nlm.nih.gov/clinvar/variation/48368/
NM_206933.2(USH2A):c.11404G>A	concordant	unreportable	unreportable	48374	0		1	SCV000065391	Likely benign	Laboratory for Mol	4/26/2016	USH2A	chr1	215916663	215916663	http://gnomad.b	0.001523632339	FIN	0.01274229959	3		1	0	https://www.ncbi.nlm.nih.gov/clinvar/variation/48374/
NM_206933.2(USH2A):c.1179A>G	concordant	unreportable	unreportable	48383	0		2	SCV000354173;S	Uncertain significance;Likel	Illumina Clinical Se	2016-10-18;2016-0	USH2A	chr1	216497659	216497659	http://gnomad.b	0.001114657343	ASJ	0.007552559953	0		0	0	https://www.ncbi.nlm.nih.gov/clinvar/variation/48383/
NM_206933.2(USH2A):c.12343C>T	concordant	unreportable	unreportable	48397	0		2	SCV000707355;S	Uncertain significance;Likel	EGL Genetic Diagn	2017-06-30;2016-0	USH2A	chr1	215848910	215848910	http://gnomad.b	0.0003688794085	AMR	0.0005667579826	0		0	0	https://www.ncbi.nlm.nih.gov/clinvar/variation/48397/
NM_206933.2(USH2A):c.12557T>C	concordant	unreportable	unreportable	48400	0		1	SCV000572702	Uncertain significance	GeneDx	11/28/2017	USH2A	chr1	215848696	215848696	http://gnomad.b	0.000416994114	AFR	0.00690608006	0		1	0	https://www.ncbi.nlm.nih.gov/clinvar/variation/48400/
NM_206933.2(USH2A):c.14516C>T	concordant	unreportable	unreportable	48440	0		1	SCV000065459	Likely benign	Laboratory for Mol	4/26/2016	USH2A	chr1	215821936	215821936	http://gnomad.b	3.21E-05	AFR	0.0004573969927	0		0	0	https://www.ncbi.nlm.nih.gov/clinvar/variation/48440/
NM_206933.2(USH2A):c.14531C>T	concordant	unreportable	unreportable	48443	0		1	SCV000065462	Likely benign	Laboratory for Mol	4/26/2016	USH2A	chr1	215821921	215821921	http://gnomad.b	0.0002566117624	AMR	0.0002680170001	0		2	0	https://www.ncbi.nlm.nih.gov/clinvar/variation/48443/
NM_206933.2(USH2A):c.15102C>T	concordant	unreportable	unreportable	48456	0		1	SCV000065475	Likely benign	Laboratory for Mol	4/26/2016	USH2A	chr1	215807996	215807996	http://gnomad.b	5.61E-05	NFE	4.48E-05	0		0	0	https://www.ncbi.nlm.nih.gov/clinvar/variation/48456/
NM_206933.2(USH2A):c.1663C>G	concordant	unreportable	unreportable	48472	0		2	SCV000341464;S	Likely benign;Likely benign	EGL Genetic Diagn	2017-06-30;2016-0	USH2A	chr1	216465694	216465694	http://gnomad.b	0.001764205867	ASJ	0.002338819904	1		1	0	https://www.ncbi.nlm.nih.gov/clinvar/variation/48472/
NM_206933.2(USH2A):c.2052A>G	concordant	unreportable	unreportable	48482	0		1	SCV000702334	Likely benign	EGL Genetic Diagn	6/30/2017	USH2A	chr1	216424360	216424360	http://gnomad.b	0.0008981411685	SAS	0.006595619954	3		1	0	https://www.ncbi.nlm.nih.gov/clinvar/variation/48482/
NM_206933.2(USH2A):c.2256T>C	concordant	unreportable	unreportable	48486	0		1	SCV000225953	Likely benign	EGL Genetic Diagn	6/30/2017	USH2A	chr1	216420480	216420480	http://gnomad.b	0.0008339882279	AMR	0.001668649958	0		0	0	https://www.ncbi.nlm.nih.gov/clinvar/variation/48486/
NM_206933.2(USH2A):c.3123C>A	concordant	unreportable	unreportable	48495	0		2	SCV000705832;S	Uncertain significance;Likel	EGL Genetic Diagn	2017-06-30;2016-0	USH2A	chr1	216390763	216390763	http://gnomad.b	0.001844397043	SAS	0.002632949967	2		0	0	https://www.ncbi.nlm.nih.gov/clinvar/variation/48495/
NM_206933.2(USH2A):c.3621C>T	concordant	unreportable	unreportable	48505	0		2	SCV000340699;S	Benign;Uncertain significar	EGL Genetic Diagn	2017-06-30;2016-1	USH2A	chr1	216373159	216373159	http://gnomad.b	0.0005452999952	AFR	0.005750880111	0		0	0	https://www.ncbi.nlm.nih.gov/clinvar/variation/48505/
NM_206933.2(USH2A):c.4578G>T	concordant	unreportable	unreportable	48519	0		1	SCV000339667	Uncertain significance	EGL Genetic Diagn	6/30/2017	USH2A	chr1	216348643	216348643	http://gnomad.b	0.0001844397043	AFR	0.002875820035	0		0	0	https://www.ncbi.nlm.nih.gov/clinvar/variation/48519/
NM_206933.2(USH2A):c.4586A>T	concordant	unreportable	unreportable	48520	0		2	SCV000340165;S	Likely benign;Uncertain sig	EGL Genetic Diagn	2017-06-30;2016-1	USH2A	chr1	216348635	216348635	http://gnomad.b	0.0004410514667	AFR	0.006276969798	0		1	0	https://www.ncbi.nlm.nih.gov/clinvar/variation/48520/
NM_206933.2(USH2A):c.478G>A	concordant	unreportable	unreportable	48522	0		1	SCV000515231	Likely benign	GeneDx	11/28/2017	USH2A	chr1	216595201	216595201	http://gnomad.b	0.0004490705843	AFR	0.008300649934	0		0	0	https://www.ncbi.nlm.nih.gov/clinvar/variation/48522/
NM_206933.2(USH2A):c.5612G>A	concordant	unreportable	unreportable	48537	0		1	SCV000345453	Uncertain significance	EGL Genetic Diagn	6/30/2017	USH2A	chr1	216246603	216246603	http://gnomad.b	0.0005693573479	AFR	0.003074310021	0		0	0	https://www.ncbi.nlm.nih.gov/clinvar/variation/48537/
NM_206933.2(USH2A):c.5858C>G	concordant	unreportable	unreportable	48546	0		2	SCV000704147;S	Uncertain significance;Unc	EGL Genetic Diagn	2017-06-30;2016-0	USH2A	chr1	216243634	216243634	http://gnomad.b	0.00129107793	NFE	0.001103610033	0		2	0	https://www.ncbi.nlm.nih.gov/clinvar/variation/48546/
NM_206933.2(USH2A):c.6134A>G	concordant	unreportable	unreportable	48553	0		1	SCV000704178	Uncertain significance	EGL Genetic Diagn	6/30/2017	USH2A	chr1	216221905	216221905	http://gnomad.b	4.01E-05	FIN	0.0001793879928	0		0	0	https://www.ncbi.nlm.nih.gov/clinvar/variation/48553/
NM_206933.2(USH2A):c.6347A>G	concordant	unreportable	unreportable	48559	0		1	SCV000583059	Uncertain significance	GeneDx	11/28/2017	USH2A	chr1	216173883	216173883	http://gnomad.b	0.001178810284	ASJ	0.006401139777	0		1	0	https://www.ncbi.nlm.nih.gov/clinvar/variation/48559/
NM_206933.2(USH2A):c.6711C>A	concordant	unreportable	unreportable	48567	0		1	SCV000065589	Likely benign	Laboratory for Mol	4/26/2016	USH2A	chr1	216166456	216166456	http://gnomad.b	0.0001042485285	AFR	0.001895179972	0		0	0	https://www.ncbi.nlm.nih.gov/clinvar/variation/48567/
NM_206933.2(USH2A):c.8431C>A	concordant	unreportable	unreportable	48601	0		1	SCV000065623	Uncertain significance	Laboratory for Mol	4/26/2016	USH2A	chr1	216052233	216052233	http://gnomad.b	0.0001363249988	SAS	0.002242010087	2		0	0	https://www.ncbi.nlm.nih.gov/clinvar/variation/48601/
NM_004646.3(NPHS1):c.3250dupG	concordant	reportable	reportable	56497	1		0	SCV000568763	Pathogenic	GeneDx	11/28/2017	NPHS1	chr19	36322580	36322580		4.46E-05					0	0	https://www.ncbi.nlm.nih.gov/clinvar/variation/56497/
NM_000053.3(ATP7B):c.3060+16G>T	concordant	unreportable	unreportable	35718	0		2	SCV000602600;S	Benign;Benign	ARUP Laboratories	2017-06-30;2017-1	ATP7B	chr13	52520404	52520404	http://gnomad.b	0.001040988574	AFR	0.03100059927	3		3	0	https://www.ncbi.nlm.nih.gov/clinvar/variation/35718/
NM_000053.3(ATP7B):c.3700G>T	concordant	unreportable	unreportable	35726	0		1	SCV000694453	Uncertain significance	Laboratory Corpor	1/25/2018	ATP7B	chr13	52511815	52511815		8.42E-06					0	0	https://www.ncbi.nlm.nih.gov/clinvar/variation/35726/
NM_000525.3(KCNJ11):c.1154C>G	concordant	unreportable	unreportable	36430	0		1	SCV000369149	Likely benign	Illumina Clinical Se	10/18/2016	KCNJ11	chr11	17408485	17408485	http://gnomad.b	0.006273746049	AFR	0.02429920062	9		7	0	https://www.ncbi.nlm.nih.gov/clinvar/variation/36430/
NM_000260.3(MYO7A):c.1554+8G>A	concordant	unreportable	unreportable	43150	0		2	SCV000225763;S	Uncertain significance;Beni	EGL Genetic Diagn	2017-06-30;2016-0	MYO7A	chr11	76873384	76873384	http://gnomad.b	0.002656467538	FIN	0.00620759977	2		3	0	https://www.ncbi.nlm.nih.gov/clinvar/variation/43150/
NM_000260.3(MYO7A):c.2187+1G>A	concordant	reportable	reportable	43175	1		0	SCV000616804	Likely pathogenic	GeneDx	11/28/2017	MYO7A	chr11	76886511	76886511	http://gnomad.b	4.00E-05	ASJ	0.0003046919883	0		0	0	https://www.ncbi.nlm.nih.gov/clinvar/variation/43175/
NM_000260.3(MYO7A):c.4568+12C>G	concordant	unreportable	unreportable	43246	0		2	SCV000374377;S	Likely benign;Benign	Illumina Clinical Se	2016-10-18;2016-0	MYO7A	chr11	76909678	76909678	http://gnomad.b	0.00420073933	FIN	0.07839749753	59		2	0	https://www.ncbi.nlm.nih.gov/clinvar/variation/43246/
NM_033056.3(PCDH15):c.3718-19C>A	concordant	unreportable	unreportable	46472	0		1	SCV000699760	Benign	Laboratory Corpor	1/25/2018	PCDH15	chr10	55617042	55617042	http://gnomad.b	0.009538137119	SAS	0.04036819935	99		58	0	https://www.ncbi.nlm.nih.gov/clinvar/variation/46472/
NM_000481.3(AMT):c.471+2T>C	concordant	reportable	reportable	56233	1		0	SCV000636426	Pathogenic	Invitae	10/5/2017	AMT	chr3	49457642	49457642	http://gnomad.b	8.00E-06	AMR	2.98E-05	0		0	0	https://www.ncbi.nlm.nih.gov/clinvar/variation/56233/
NM_000337.5(SGCD):c.105G>C	concordant	unreportable	unreportable	36771	0		2	SCV000337618;S	Likely benign;Benign	EGL Genetic Diagn	2016-11-03;2017-1	SGCD	chr5	155771600	155771600	http://gnomad.b	0.0006642338103	AFR	0.007758050226	0		0	0	https://www.ncbi.nlm.nih.gov/clinvar/variation/36771/
NM_000404.2(GLB1):c.176G>A	concordant	reportable	reportable	945	1		0	SCV000490538	Pathogenic	GeneDx	12/21/2016	GLB1	chr3	33114105	33114105	http://gnomad.b	8.00E-06	AMR	8.93E-05	0		0	0	https://www.ncbi.nlm.nih.gov/clinvar/variation/945/

Supplemental Table 2

NM_003494.3(DYSF):c.5371C>T	concordant	unreportable	unreportable	69635	0	3	SCV000341697;S	Uncertain significance;Unc	EGL Genetic Diagn	2017-06-30;2017-1	DYSF	chr2	71895914	71895914	http://gnomad.b	0.0001293347345	AMR	0.0003278880031	0		0	0	https://www.ncbi.nlm.nih.gov/clinvar/variation/69635/
NM_206933.2(USH2A):c.4646G>A	concordant	unreportable	unreportable	73561	0	1	SCV000272897	Uncertain significance	Laboratory for Mol	4/26/2016	USH2A	chr1	216270537	216270537	http://gnomad.b	3.21E-05	FIN	0.0008969409973	0		0	0	https://www.ncbi.nlm.nih.gov/clinvar/variation/73561/
NM_001271208.1(NEB):c.4834C>T	concordant	unreportable	unreportable	74142	0	3	SCV000513914;S	Likely benign;Uncertain sig	GeneDx;Illumina C	2017-11-28;2016-1	NEB	chr2	152522801	152522801	http://gnomad.b	0.0003794958643	AFR	0.002747250022	0		0	0	https://www.ncbi.nlm.nih.gov/clinvar/variation/74142/
NM_000231.2(SGCG):c.787G>A	concordant	reportable	reportable	2009	3	0	SCV000232489;S	Pathogenic;Pathogenic;Pat	EGL Genetic Diagn	2017-06-30;2017-1	SGCG	chr13	23898591	23898591	http://gnomad.b	0.0001360478889	NFE	5.37E-05	0		0	0	https://www.ncbi.nlm.nih.gov/clinvar/variation/2009/
NM_015346.3(ZFYVE26):c.3070C>T	concordant	unreportable	unreportable	79594	0	1	SCV000388048	Uncertain significance	Illumina Clinical Se	10/18/2016	ZFYVE26	chr14	68252900	68252900		8.00E-06					0	0	https://www.ncbi.nlm.nih.gov/clinvar/variation/79594/
NM_000243.2(MEFV):c.941G>A	concordant	unreportable	unreportable	36514	0	1	SCV000052853	Uncertain significance	Laboratory Corpora	11/20/2015	MEFV	chr16	3299750	3299750	http://gnomad.b	1.68E-05	SAS	0.00045507701	0		0	0	https://www.ncbi.nlm.nih.gov/clinvar/variation/36514/
NM_000169.2(GLA):c.714T>C	concordant	unreportable	unreportable	42460	0	2	SCV000622189;S	Likely benign;Likely benign	Invitae;Laboratory	2017-10-05;2016-0	GLA	chrX	100653860	100653860	http://gnomad.b	1.94E-05	FIN	6.08E-05	0	2	0	0	https://www.ncbi.nlm.nih.gov/clinvar/variation/42460/
NM_000169.2(GLA):c.801+14C>T	concordant	unreportable	unreportable	42463	0	1	SCV000535906	Likely benign	GeneDx	11/28/2017	GLA	chrX	100653759	100653759	http://gnomad.b	8.72E-05	EAS	0.0006216010079	0	2	0	0	https://www.ncbi.nlm.nih.gov/clinvar/variation/42463/
NM_000260.3(MYO7A):c.1422G>A	concordant	unreportable	unreportable	43147	0	2	SCV000513832;S	Likely benign;Likely benign	GeneDx;Laboratory	2017-11-28;2016-0	MYO7A	chr11	76873244	76873244	http://gnomad.b	0.0004320760454	NFE	0.0006497040158	0		0	0	https://www.ncbi.nlm.nih.gov/clinvar/variation/43147/
NM_000260.3(MYO7A):c.2617C>T	concordant	unreportable	unreportable	43187	0	1	SCV000059743	Likely benign	Laboratory for Mol	4/26/2016	MYO7A	chr11	76891450	76891450	http://gnomad.b	0.0006881211093	FIN	0.002180630108	1		0	0	https://www.ncbi.nlm.nih.gov/clinvar/variation/43187/
NM_000260.3(MYO7A):c.324C>T	concordant	unreportable	unreportable	43201	0	1	SCV000374193	Likely benign	Illumina Clinical Se	10/18/2016	MYO7A	chr11	76866991	76866991	http://gnomad.b	0.001064187297	EAS	0.0160798002	3		3	0	https://www.ncbi.nlm.nih.gov/clinvar/variation/43201/
NM_000260.3(MYO7A):c.3750+7G>A	concordant	unreportable	unreportable	43221	0	1	SCV000703586	Uncertain significance	EGL Genetic Diagn	6/30/2017	MYO7A	chr11	76901191	76901191	http://gnomad.b	0.0002480436557	SAS	0.0004922579974	0		0	0	https://www.ncbi.nlm.nih.gov/clinvar/variation/43221/
NM_000260.3(MYO7A):c.5108C>T	concordant	unreportable	unreportable	43271	0	1	SCV000374420	Uncertain significance	Illumina Clinical Se	10/18/2016	MYO7A	chr11	76913409	76913409	http://gnomad.b	0.0004800844949	ASJ	0.001638959977	0		0	0	https://www.ncbi.nlm.nih.gov/clinvar/variation/43271/
NM_000260.3(MYO7A):c.5259G>A	concordant	unreportable	unreportable	43277	0	1	SCV000533510	Likely benign	GeneDx	11/28/2017	MYO7A	chr11	76914195	76914195	http://gnomad.b	0.000160028165	AFR	0.003626940073	0		0	0	https://www.ncbi.nlm.nih.gov/clinvar/variation/43277/
NM_033056.3(PCDH15):c.4783A>C	concordant	unreportable	unreportable	46486	0	1	SCV000363139	Uncertain significance	Illumina Clinical Se	10/18/2016	PCDH15	chr10	55582703	55582703	http://gnomad.b	5.40E-05	AMR	0.000774501008	1		0	0	https://www.ncbi.nlm.nih.gov/clinvar/variation/46486/
NM_033056.3(PCDH15):c.5283T>A	concordant	unreportable	unreportable	46491	0	1	SCV000363124	Uncertain significance	Illumina Clinical Se	10/18/2016	PCDH15	chr10	55582203	55582203		1.29E-05					0	0	https://www.ncbi.nlm.nih.gov/clinvar/variation/46491/
NM_153676.3(USH1C):c.403G>A	concordant	unreportable	unreportable	48015	0	3	SCV000113244;S	Likely benign;Uncertain sig	EGL Genetic Diagn	2017-06-30;2016-1	USH1C	chr11	17548863	17548863	http://gnomad.b	0.002528323625	NFE	0.003441229928	2		0	0	https://www.ncbi.nlm.nih.gov/clinvar/variation/48015/
NM_000337.5(SGCD):c.123C>G	concordant	unreportable	unreportable	48115	0	2	SCV000333729;S	Uncertain significance;Likel	EGL Genetic Diagn	2017-06-30;2017-1	SGCD	chr5	155771618	155771618	http://gnomad.b	0.0002240788758	AFR	0.001450420008	0		0	0	https://www.ncbi.nlm.nih.gov/clinvar/variation/48115/
NM_000337.5(SGCD):c.213G>A	concordant	unreportable	unreportable	48116	0	4	SCV000334630;S	Uncertain significance;Likel	EGL Genetic Diagn	2016-11-03;2016-1	SGCD	chr5	155935631	155935631	http://gnomad.b	0.001296456353	NFE	0.001979060005	1		0	0	https://www.ncbi.nlm.nih.gov/clinvar/variation/48116/
NM_000337.5(SGCD):c.393C>T	concordant	unreportable	unreportable	48119	0	1	SCV000526314	Likely benign	GeneDx	11/28/2017	SGCD	chr5	156021952	156021952	http://gnomad.b	1.60E-05	EAS	0.0004173619964	0		0	0	https://www.ncbi.nlm.nih.gov/clinvar/variation/48119/
NM_000337.5(SGCD):c.69C>T	concordant	unreportable	unreportable	48121	0	2	SCV000344191;S	Uncertain significance;Likel	EGL Genetic Diagn	2017-06-30;2017-0	SGCD	chr5	155771564	155771564	http://gnomad.b	6.40E-05	AMR	0.0006059139851	0		0	0	https://www.ncbi.nlm.nih.gov/clinvar/variation/48121/
NM_206933.2(USH2A):c.1036A>C	concordant	reportable	reportable	48347	1	0	SCV000706010	Pathogenic	EGL Genetic Diagn	6/30/2017	USH2A	chr1	216498754	216498754	http://gnomad.b	3.21E-05	NFE	0.0001617079979	0		0	0	https://www.ncbi.nlm.nih.gov/clinvar/variation/48347/
NM_206933.2(USH2A):c.10769C>T	concordant	unreportable	unreportable	48358	0	1	SCV000340203	Uncertain significance	EGL Genetic Diagn	6/30/2017	USH2A	chr1	215953355	215953355	http://gnomad.b	0.0006976632291	NFE	0.001310170046	2		0	0	https://www.ncbi.nlm.nih.gov/clinvar/variation/48358/
NM_206933.2(USH2A):c.11927C>T	concordant	unreportable	unreportable	48385	0	2	SCV000605543;S	Uncertain significance;Unc	ARUP Laboratories	2017-06-30;2017-0	USH2A	chr1	215901511	215901511	http://gnomad.b	0.0007537970522	NFE	0.001057820045	0		0	0	https://www.ncbi.nlm.nih.gov/clinvar/variation/48385/
NM_206933.2(USH2A):c.14517G>A	concordant	unreportable	unreportable	48441	0	1	SCV000065460	Likely benign	Laboratory for Mol	4/26/2016	USH2A	chr1	215821935	215821935	http://gnomad.b	0.0001684014691	AMR	0.00220382004	1		0	0	https://www.ncbi.nlm.nih.gov/clinvar/variation/48441/
NM_206933.2(USH2A):c.1590A>T	concordant	unreportable	unreportable	48470	0	1	SCV000703240	Uncertain significance	EGL Genetic Diagn	6/30/2017	USH2A	chr1	216495279	216495279	http://gnomad.b	4.01E-05	AFR	0.0004574570048	0		0	0	https://www.ncbi.nlm.nih.gov/clinvar/variation/48470/
NM_206933.2(USH2A):c.3700A>G	concordant	unreportable	unreportable	48506	0	1	SCV000354124	Uncertain significance	Illumina Clinical Se	10/18/2016	USH2A	chr1	216373080	216373080	http://gnomad.b	8.02E-06	NFE	2.69E-05	0		0	0	https://www.ncbi.nlm.nih.gov/clinvar/variation/48506/
NM_206933.2(USH2A):c.4335T>C	concordant	unreportable	unreportable	48513	0	1	SCV000065535	Likely benign	Laboratory for Mol	4/26/2016	USH2A	chr1	216363626	216363626	http://gnomad.b	0.0002004779394	AMR	0.003049870022	0		0	0	https://www.ncbi.nlm.nih.gov/clinvar/variation/48513/
NM_206933.2(USH2A):c.7301-6C>T	concordant	unreportable	unreportable	48580	0	2	SCV000229988;S	Uncertain significance;Likel	EGL Genetic Diagn	2017-06-30;2016-0	USH2A	chr1	216074253	216074253	http://gnomad.b	0.0004651088194	NFE	0.0009377649985	0		1	0	https://www.ncbi.nlm.nih.gov/clinvar/variation/48580/
NM_206933.2(USH2A):c.8709C>T	concordant	unreportable	unreportable	48608	0	1	SCV000343277	Uncertain significance	EGL Genetic Diagn	6/30/2017	USH2A	chr1	216040485	216040485		1.60E-05					0	0	https://www.ncbi.nlm.nih.gov/clinvar/variation/48608/
NM_017882.2(CLN6):c.150C>G	concordant	reportable	reportable	68094	1	0	SCV000568366	Likely pathogenic	GeneDx	11/28/2017	CLN6	chr15	68510922	68510922	http://gnomad.b	1.60E-05	NFE	1.79E-05	0		0	0	https://www.ncbi.nlm.nih.gov/clinvar/variation/68094/
NM_001164277.1(SLC37A4):c.1099G>	concordant	unreportable	unreportable	21298	0	1	SCV000331845	Uncertain significance	EGL Genetic Diagn	6/30/2017	SLC37A4	chr11	118895925	118895925	http://gnomad.b	3.07E-05	SAS	0.0001956049964	0		0	0	https://www.ncbi.nlm.nih.gov/clinvar/variation/21298/
NM_000521.3(HEXB):c.1627G>A	concordant	unreportable	unreportable	3881	0	1	SCV000697176	Likely benign	Laboratory Corpora	1/25/2018	HEXB	chr5	74016956	74016956	http://gnomad.b	0.0005280506929	SAS	0.005751609802	3		1	0	https://www.ncbi.nlm.nih.gov/clinvar/variation/3881/
NM_000920.3(PC):c.1892G>A	concordant	reportable	reportable	21222	1	0	SCV000566499	Pathogenic	GeneDx	11/28/2017	PC	chr11	66619351	66619351	http://gnomad.b	2.40E-05	NFE	8.97E-06	0		0	0	https://www.ncbi.nlm.nih.gov/clinvar/variation/21222/
NM_000495.4(COL4A5):c.884G>A	concordant	reportable	reportable	24336	1	0	SCV000613003	Pathogenic	Athena Diagnostics	8/17/2017	COL4A5	chrX	107823961	107823961		9.69E-06					0	0	https://www.ncbi.nlm.nih.gov/clinvar/variation/24336/
NM_000495.4(COL4A5):c.1033-15delT	concordant	unreportable	unreportable	24358	0	1	SCV000603150	Benign	ARUP Laboratories	6/30/2017	COL4A5	chrX	107829820	107829821		0.003961297446					2	81	https://www.ncbi.nlm.nih.gov/clinvar/variation/24358/
NM_000495.4(COL4A5):c.2858G>T	concordant	unreportable	unreportable	24573	0	1	SCV000700762	Benign	EGL Genetic Diagn	6/30/2017	COL4A5	chrX	107865996	107865996	http://gnomad.b	0.003777276293	EAS	0.03632960096	7	223	7	52	https://www.ncbi.nlm.nih.gov/clinvar/variation/24573/
NM_000060.2(BTD):c.-34C>T	concordant	unreportable	unreportable	25104	0	4	SCV000602899;S	Benign;Benign;Likely benig	ARUP Laboratories	2017-06-30;2017-0	BTD	chr3	15643324	15643324	http://gnomad.b	0.002070130724	AFR	0.0543720983	19		13	0	https://www.ncbi.nlm.nih.gov/clinvar/variation/25104/
NM_000155.3(GALT):c.820+13A>G	concordant	reportable	reportable	25269	1	0	SCV000331164	Pathogenic	EGL Genetic Diagn	6/30/2017	GALT	chr9	34648904	34648904		1.10E-05					0	0	https://www.ncbi.nlm.nih.gov/clinvar/variation/25269/
NM_000155.3(GALT):c.1060-1G>A	concordant	reportable	reportable	25329	1	0	SCV000333965	Pathogenic	EGL Genetic Diagn	6/30/2017	GALT	chr9	34650365	34650365		2.75E-06					0	0	https://www.ncbi.nlm.nih.gov/clinvar/variation/25329/
NM_003060.3(SLC22A5):c.137C>T	concordant	unreportable	unreportable	25357	0	1	SCV000707114	Uncertain significance	EGL Genetic Diagn	6/30/2017	SLC22A5	chr5	131705801	131705801	http://gnomad.b	5.65E-06	AFR	6.96E-05	0		0	0	https://www.ncbi.nlm.nih.gov/clinvar/variation/25357/
NM_003060.3(SLC22A5):c.629A>G	concordant	unreportable	unreportable	38276	0	1	SCV000632560	Uncertain significance	Invitae	10/5/2017	SLC22A5	chr5	131719970	131719970	http://gnomad.b	1.13E-05	NFE	4.48E-05	0		0	0	https://www.ncbi.nlm.nih.gov/clinvar/variation/38276/
NM_000352.3(ABCC8):c.3329+6C>T	concordant	unreportable	unreportable	35608	0	2	SCV000192027;S	Likely benign;Likely benign	Genetic Services La	2014-09-11;2016-1	ABCC8	chr11	17428163	17428163	http://gnomad.b	0.004705317551	NFE	0.01766010001	24		14	0	https://www.ncbi.nlm.nih.gov/clinvar/variation/35608/
NM_000352.3(ABCC8):c.4120-19C>T	concordant	unreportable	unreportable	35612	0	1	SCV000192033	Likely benign	Genetic Services La	9/11/2014	ABCC8	chr11	17417496	17417496	http://gnomad.b	0.005386723932	AMR	0.05111360177	60		29	0	https://www.ncbi.nlm.nih.gov/clinvar/variation/35612/
NM_000352.3(ABCC8):c.4412-14C>T	concordant	unreportable	unreportable	35619	0	1	SCV000612220	Uncertain significance	Athena Diagnostics	8/17/2017	ABCC8	chr11	17415960	17415960	http://gnomad.b	3.61E-05	AFR	0.0005227390211	0		0	0	https://www.ncbi.nlm.nih.gov/clinvar/variation/35619/
NM_000383.3(AIRE):c.1567-5C>T	concordant	unreportable	unreportable	35662	0	1	SCV000345077	Benign	EGL Genetic Diagn	6/30/2017	AIRE	chr21	45717534	45717534	http://gnomad.b	0.0005848702424	SAS	0.009456030093	6		0	0	https://www.ncbi.nlm.nih.gov/clinvar/variation/35662/
NM_000383.3(AIRE):c.652+14C>T	concordant	unreportable	unreportable	35667	0	2	SCV000706779;S	Benign;Benign	EGL Genetic Diagn	2017-06-30;2017-1	AIRE	chr21	45708355	45708355	http://gnomad.b	0.006313813521	ASJ	0.02169420011	37		20	0	https://www.ncbi.nlm.nih.gov/clinvar/variation/35667/
NM_000053.3(ATP7B):c.3557-6C>T	concordant	unreportable	unreportable	35722	0	5	SCV000602595;S	Benign;Likely benign;Uncer	ARUP Laboratories	2017-06-30;2017-1	ATP7B	chr13	52513335	52513335	http://gnomad.b	0.001052212171	NFE	0.003982780036	3		0	0	https://www.ncbi.nlm.nih.gov/clinvar/variation/35722/
NM_000053.3(ATP7B):c.*15C>T	concordant	unreportable	unreportable	35733	0	3	SCV000602601;S	Likely benign;Likely benign	ARUP Laboratories	2017-06-30;2017-1	ATP7B	chr13	52508877	52508877	http://gnomad.b	0.0005976565131	AFR	0.01576190069	2		0	0	https://www.ncbi.nlm.nih.gov/clinvar/variation/35733/
NM_000053.3(ATP7B):c.*16G>A	concordant	unreportable	unreportable	35734	0	1	SCV000384643	Uncertain significance	Illumina Clinical Se	10/18/2016	ATP7B	chr13	52508876	52508876	http://gnomad.b	0.0004068553727	ASJ	0.003147850046	1		0	0	https://www.ncbi.nlm.nih.gov/clinvar/variation/35734/
NM_000492.3(CFTR):c.4243-35delT	concordant	unreportable	unreportable	35885	0	1	SCV000603011	Benign	ARUP Laboratories	6/30/2017	CFTR	chr7	117306925	117306926	http://gnomad.b	0.0005045680743	ASJ	0.02005290054	2		1	0	https://www.ncbi.nlm.nih.gov/clinvar/variation/35885/
NM_000497.3(CYP11B1):c.1003A>G	concordant	unreportable	unreportable	35977	0	2	SCV000613031;S	Uncertain significance;Likel	Athena Diagnostics	2017-08-17;2016-1	CYP11B1	chr8	143957246	143957246	http://gnomad.b	0.0007171751358	AFR	0.00812739972	1		0	0	https://www.ncbi.nlm.nih.gov/clinvar/variation/35977/
NM_000497.3(CYP11B1):c.1015G>A	concordant	unreportable	unreportable	35978	0	1	SCV000613032	Uncertain significance	Athena Diagnostics	8/17/2017	CYP11B1	chr8	143957234	143957234	http://gnomad.b	1.61E-05	AFR	0.0002617459977	0		0	0	https://www.ncbi.nlm.nih.gov/clinvar/variation/35978/
NM_000497.3(CYP11B1):c.1120C>A	concordant	unreportable	unreportable	35981	0	2	SCV000472331;S	Likely benign;Benign	Illumina Clinical Se	2016-10-18;2017-0	CYP11B1	chr8	143957129	143957129	http://gnomad.b	0.01755870361	NFE	0.02585509978	55		27	0	https://www.ncbi.nlm.nih.gov/clinvar/variation/35981/
NM_000497.3(CYP11B1):c.1144C>T	concordant	unreportable	unreportable	35985	0	1	SCV000472329	Likely benign	Illumina Clinical Se	10/18/2016	CYP11B1	chr8	143956706	143956706		8.86E-05					0	0	https://www.ncbi.nlm.nih.gov/clinvar/variation/35985/
NM_000497.3(CYP11B1):c.243C>T	concordant	unreportable	unreportable	35987	0	1	SCV000472371	Likely benign	Illumina Clinical Se	10/18/2016	CYP11B1	chr8	143960600	143960600	http://gnomad.b	0.008880078648	ASJ	0.02143440023	5		7	0	https://www.ncbi.nlm.nih.gov/clinvar/variation/35987/
NM_000497.3(CYP11B1):c.799+5G>C	concordant	unreportable	unreportable	35991	0	1	SCV000613042	Uncertain significance	Athena Diagnostics	8/17/2017	CYP11B1	chr8	143958093	143958093	http://gnomad.b	4.83E-05	AFR	0.0006113399868	0		0	0	https://www.ncbi.nlm.nih.gov/clinvar/variation/35991/
NM_001079802.1(FKTN):c.166-4A>G	concordant	unreportable	unreportable	36139	0	5	SCV000613315;S	Uncertain significance;Unc	Athena Diagnostics	2017-08-17;2017-0	FKTN	chr9	108363422	108363422	http://gnomad.b	0.0004432278333	FIN	0.003202490043	0		0	0	https://www.ncbi.nlm.nih.gov/clinvar/variation/36139/
NM_001079802.1(FKTN):c.166-6A>G	concordant	unreportable	unreportable	36140	0	3	SCV000247384;S	Likely benign;Benign;Benig	Genetic Services La	2015-09-15;2017-1	FKTN	chr9	108363420	108363420	http://gnomad.b	0.005676862089	SAS	0.02443809994	29		17	0	https://www.ncbi.nlm.nih.gov/clinvar/variation/36140/
NM_000243.2(MEFV):c.1260+10C>T	concordant	unreportable	unreportable	36498	0	1	SCV000052830	Uncertain significance	Laboratory Corpora	11/20/2015	MEFV	chr16	3299421	3299421	http://gnomad.b	0.0004002239015	ASJ	0.009838080034	1		0	0	https://www.ncbi.nlm.nih.gov/clinvar/variation/36498/
NM_000337.5(SGCD):c.294+8T>C	concordant	unreportable	unreportable	36772	0	4	SCV000333704;S	Benign;Likely benign;Likely	EGL Genetic Diagn	2016-11-03;2014-0	SGCD	chr5	155935720	155935720	http://gnomad.b	0.006274208521	FIN	0.01835950091	6		4	0	https://www.ncbi.nlm.nih.gov/clinvar/variation/36772/
NM_000426.3(LAMA2):c.2049_2050de	concordant	reportable	reportable	38340	2	0	SCV000583109;S	Pathogenic;Pathogenic	GeneDx;Invitae	2017-11-28;2017-1	LAMA2	chr6	129573388	129573390	http://gnomad.b	0.000152038922	AFR	0.0001960530062	0		0	0	https://www.ncbi.nlm.nih.gov/clinvar/variation/38340/
NM_000282.3(PCCA):c.1651G>T	concordant	unreportable	unreportable	38865	0	1	SCV000631899	Benign	Invitae	10/5/2017	PCCA	chr13	101020733	101020733	http://gnomad.b	0.00933578451	NFE	0.01144309994	19		14	0	https://www.ncbi.nlm.nih.gov/clinvar/variation/38865/
NM_000288.3(PEX7):c.340-10A>G	concordant	unreportable	unreportable	7787	0	1	SCV000460534	Uncertain significance	Illumina Clinical Se	10/18/2016	PEX7	chr6	137166743	137166743	http://gnomad.b	5.56E-05	ASJ	0.002133280039	0		0	0	https://www.ncbi.nlm.nih.gov/clinvar/variation/7787/
NM_000532.4(PCCB):c.1490C>T	concordant	unreportable	unreportable	38877	0	1	SCV000521001	Likely benign	GeneDx	11/28/2017	PCCB	chr3	136047691	136047691	http://gnomad.b	0.001814287313	AMR	0.005926500075	1		0	0	https://www.ncbi.nlm.nih.gov/clinvar/variation/38877/
NM_024312.4(GNPTAB):c.1042A>C	concordant	unreportable	unreportable	39020	0	1	SCV000375436	Likely benign	Illumina Clinical Se	10/18/2016	GNPTAB	chr12	102164255	102164255	http://gnomad.b	0.00319795331	AFR	0.04215139896	8		6	0	https://www.ncbi.nlm.nih.gov/clinvar/variation/39020/
NM_024312.4(GNPTAB):c.1123C>T	concordant	reportable	reportable	39022	2	0	SCV000375432;S	Pathogenic;Pathogenic	Illumina Clinical Se	2016-10-18;2018-0	GNPTAB	chr12	102163960	102163960	http://gnomad.b	1.60E-05	NFE	7.17E-05	0		0	0	https://www.ncbi.nlm.nih.gov/clinvar/variation/39022/
NM_024312.4(GNPTAB):c.3410T>A	concordant	reportable	reportable	39071	1	0	SCV000699481	Pathogenic	Laboratory Corpora	1/25/2018	GNPTAB	chr12	102151014	102151014	http://gnomad.b	7.99E-06	AFR	0.0001960530062	0		0	0	https://www.ncbi.nlm.nih.gov/clinvar/variation/39071/
NM_206933.2(USH2A):c.2299delG	concordant	reportable	reportable	2351	2	0	SCV000225952;S	Pathogenic;Pathogenic	EGL Genetic Diagn	2017-06-30;2017-1	USH2A	chr1	216420436	216420437	http://gnomad.b	0.0006415294061	AMR	0.001608869992	0		0	0	https://www.ncbi.nlm.nih.gov/clinvar/variation/2351/
NM_000135.2(FANCA):c.3788_3790de	concordant	reportable	reportable	41003	2	0	SCV000565881;S	Pathogenic;Pathogenic	GeneDx;Invitae	2017-11-28;2017-1	FANCA	chr16	89807249	89807252	http://gnomad.b	0.000103990017	AMR	0.0002086809982	0		0	0	https://www.ncbi.nlm.nih.gov/clinvar/variation/41003/
NM_000169.2(GLA):c.1153A>G	concordant	unreportable	unreportable	42452	0	2	SCV000207836;S	Likely benign;Likely benign	GeneDx;Laboratory	2017-11-28;2016-0	GLA	chrX	100652934	100652934	http://gnomad.b	0.000280874391	SAS	0.005171060096	0	61	1	1	https://www.ncbi.nlm.nih.gov/clinvar/variation/42452/
NM_000169.2(GLA):c.352C>T	concordant	unreportable	unreportable	42454	2	2	SCV000110117;S	Likely pathogenic;Uncertai	EGL Genetic Diagn	2017-06-30;2017-1	GLA	chrX	100658816	100658816	http://gnomad.b	0.000397098277	NFE	0.0003875970142	0	12	0	10	https://www.ncbi.nlm.nih.gov/clinvar/variation/42454/
NM_000169.2(GLA):c.640-854_640-85	concordant	unreportable	unreportable	42458	0	1	SCV000565043	Benign	GeneDx	11/28/2017	GLA	chrX	100654786	100654788		0.01404371955					15	261	https://www.ncbi.nlm.nih.gov/clinvar/variation/42458/
NM_000260.3(MYO7A):c.133-7C>T	concordant	unreportable	unreportable	43141	0	2	SCV000701632;S	Benign;Likely benign	EGL Genetic Diagn	2017-06-30;2016-1	MYO7A	chr11	76858837	76858837	http://gnomad.b	0.00497687593	NFE	0.007675359957	6		3	0	https://www.ncbi.nlm.nih.gov/clinvar/variation/43141/
NM_000260.3(MYO7A):c.1605C>T	concordant	unreportable	unreportable	43153	0	2	SCV000335425;S	Benign;Likely benign	EGL Genetic Diagn	2016-11-03;2016-1	MYO7A	chr11	76873949	76873949	http://gnomad.b	0.003256573157	NFE	0.005890360102	6		1	0	https://www.ncbi.nlm.nih.gov/clinvar/variation/43153/
NM_000260.3(MYO7A):c.1854G>A	concordant	unreportable	unreportable	43161	0	1	SCV000374262	Likely benign	Illumina Clinical Se	10/18/2016	MYO7A	chr11	76883850	76883850	http://gnomad.b	0.00315255485	NFE	0.004755150061	0		1	0	https://www.ncbi.nlm.nih.gov/clinvar/variation/43161/
NM_000260.3(MYO7A):c.2236G>A	concordant	unreportable	unreportable	43177	0	1	SCV000707296	Benign	EGL Genetic Diagn	6/30/2017	MYO7A	chr11	76888643	76888643	http://gnomad.b	0.001624285874	AFR	0.02123939991	5		1	0	https://www.ncbi.nlm.nih.gov/clinvar/variation/43177/
NM_000260.3(MYO7A):c.2293C>A	concordant	unreportable	unreportable	43180	0	3	SCV000704986;S	Uncertain significance;Unc	EGL Genetic Diagn	2017-06-30;2016-1	MYO7A	chr11	76890101	76890101	http://gnomad.b	0.0003040535134	NFE	0.0004506699916	0		0	0	https://www.ncbi.nlm.nih.gov/clinvar/variation/43180/
NM_000260.3(MYO7A):c.2447G>A	concordant	unreportable	unreportable	43183	0	2	SCV000336021;S	Likely benign;Likely benign	EGL Genetic Diagn	2017-06-30;2016-1	MYO7A	chr11	76890860	76890860	http://gnomad.b	0.000968170398	AFR	0.01057889964	1		0	0	https://www.ncbi.nlm.nih.gov/clinvar/variation/43183/
NM_000260.3(MYO7A):c.2527G>A	concordant	unreportable	unreportable	43185	0	2	SCV000702277;S	Uncertain significance;Unc	EGL Genetic Diagn	2017-06-30;2016-1	MYO7A	chr11	76890940	76890940	http://gnomad.b	0.0003200563299	SAS	0.001015719958	0		0	0	https://www.ncbi.nlm.nih.gov/clinvar/variation/43185/
NM_000260.3(MYO7A):c.2754C>T	concordant	unreportable	unreportable	43188	0	1	SCV000374314	Likely benign	Illumina Clinical Se	10/18/2016	MYO7A	chr11	76892485	76892485	http://gnomad.b	0.004312759046	AFR	0.03311410174	8		4	0	https://www.ncbi.nlm.nih.gov/clinvar/variation/43188/
NM_000260.3(MYO7A):c.2618G>A	concordant	unreportable	unreportable	43189	0	1	SCV000374305	Uncertain significance	Illumina Clinical Se	10/18/2016	MYO7A	chr11	76891451	76891451	http://gnomad.b	2.40E-05	AMR	0.0006282160175	0		0	0	https://www.ncbi.nlm.nih.gov/clinvar/variation/43189/
NM_000260.3(MYO7A):c.286-5C>T	concordant	unreportable	unreportable	43192	0	1	SCV000374185	Likely benign	Illumina Clinical Se	10/18/2016	MYO7A	chr11	76866948	76866948	http://gnomad.b	0.004944870297	AFR	0.04544759914	25		9	0	https://www.ncbi.nlm.nih.gov/clinvar/variation/43192/
NM_000260.3(MYO7A):c.2882G>A	concordant	unreportable	unreportable	43193	0	1	SCV000374322	Uncertain significance	Illumina Clinical Se	10/18/2016	MYO7A	chr11	76892613	76892613	http://gnomad.b	0.000128022532	AFR	0.002038940089	0		0	0	https://www.ncbi.nlm.nih.gov/clinvar/variation/43193/
NM_000260.3(MYO7A):c.3042G>T	concordant	unreportable	unreportable	43199	0	2	SCV000520510;S	Likely benign;Likely benign	GeneDx;Illumina C	2017-11-28;2016-1	MYO7A	chr11	76893134	76893134	http://gnomad.b	0.002072364736	AFR	0.02437430061	6		4	0	https://www.ncbi.nlm.nih.gov/clinvar/variation/43199/
NM_000260.3(MYO7A):c.3246G>A	concordant	unreportable	unreportable	43200	0	1	SCV000374334	Likely benign	Illumina Clinical Se	10/18/2016	MYO7A	chr11	76893606	76893606	http://gnomad.b	0.002032357695	AFR	0.02353169955	3		2	0	https://www.ncbi.nlm.nih.gov/clinvar/variation/43200/
NM_000260.3(MYO7A):c.3375+3G>A	concordant	unreportable	unreportable	43203	0	1	SCV000374339	Uncertain significance	Illumina Clinical Se	10/18/2016	MYO7A	chr11	76894205	76894205	http://gnomad.b	2.40E-05	NFE	5.25E-05	0		0	0	https://www.ncbi.nlm.nih.gov/clinvar/variation/43203/
NM_000260.3(MYO7A):c.3404C>A	concordant	unreportable	unreportable	43205	0	1	SCV000338924	Uncertain significance	EGL Genetic Diagn	6/30/2017	MYO7A	chr11	76895661	76895661	http://gnomad.b	0.0002400422474	AMR	0.0007850470138	0		0	0	https://www.ncbi.nlm.nih.gov/clinvar/variation/43205/
NM_000260.3(MYO7A):c.3476G>T	concordant	reportable	reportable	43206	3	0	SCV000705705;S	Pathogenic;Likely pathoger	EGL Genetic Diagn	2017-06-30;2017-1	MYO7A	chr11	76895733	76895733	http://gnomad.b	0.0002880506969	NFE	0.0005198030267	0		1	0	https://www.ncbi.nlm.nih.gov/clinvar/variation/43206/
NM_000260.3(MYO7A):c.3602G>C	concordant	unreportable	unreportable	43215	0	1	SCV000059771	Likely benign	Laboratory for Mol	4/26/2016	MYO7A	chr11	76900487	76900487	http://gnomad.b	0.0003760661876	EAS	0.004522790201	1		0	0	https://www.ncbi.nlm.nih.gov/clinvar/variation/43215/
NM_000260.3(MYO7A):c.3719G>A	concordant	reportable	reportable	43218	1	0	SCV000321919	Pathogenic	GeneDx	11/28/2017	MYO7A	chr11	76901153	76901153	http://gnomad.b	8.80E-05	NFE	0.0001404890063	0		0	0	https://www.ncbi.nlm.nih.gov/clinvar/variation/43218/
NM_000260.3(MYO7A):c.3750+5G>A	concordant	unreportable	unreportable	43220	0	1	SCV000618049	Uncertain significance	GeneDx	11/28/2017	MYO7A	chr11	76901189	76901189	http://gnomad.b	0.0007041239258	NFE	0.001429690048	0		1	0	https://www.ncbi.nlm.nih.gov/clinvar/variation/43220/
NM_000260.3(MYO7A):c.3750+9G>A	concordant	unreportable	unreportable	43222	0	2	SCV000228690;S	Uncertain significance;Likel	EGL Genetic Diagn	2017-06-30;2016-1	MYO7A	chr11	76901193	76901193	http://gnomad.b	0.001840323897	NFE	0.003676709952	1		0	0	https://www.ncbi.nlm.nih.gov/clinvar/variation/43222/
NM_000260.3(MYO7A):c.380T>C	concordant	unreportable	unreportable	43224	0	1	SCV000374199	Uncertain significance	Illumina Clinical Se	10/18/2016	MYO7A	chr11	76867047	76867047	http://gnomad.b	8.80E-05	NFE	0.0001523429964	0		0	0	https://www.ncbi.nlm.nih.gov/clinvar/variation/43224/
NM_000260.3(MYO7A):c.4074C>T	concordant	unreportable	unreportable	43233	0	2	SCV000340410;S	Benign;Likely benign	EGL Genetic Diagn	2017-06-30;2016-1	MYO7A	chr11	76903245	76903245	http://gnomad.b	0.006169085759	ASJ	0.0105584003	5		6	0	https://www.ncbi.nlm.nih.gov/clinvar/variation/43233/
NM_000260.3(MYO7A):c.4461C>T	concordant	unreportable	unreportable	43242	0	1	SCV000374374	Likely benign	Illumina Clinical Se	10/18/2016	MYO7A	chr11	76909559	76909559		0.00470482805					1	0	https://www.ncbi.nlm.nih.gov/clinvar/variation/43242/
NM_000260.3(MYO7A):c.449G>A	concordant	unreportable	unreportable	43244	0	1	SCV000059801	Uncertain significance	Laboratory for Mol	4/26/2016	MYO7A	chr11	76867116	76867116	http://gnomad.b	0.0004480788619	NFE	0.0005465170252	0		0	0	https://www.ncbi.nlm.nih.gov/clinvar/variation/43244/

Supplemental Table 2

NM_000260.3(MYO7A):c.4697C>T	concordant	unreportable	unreportable	43254	0	4	SCV000296901;S(	Benign;Benign;Likely benig	Division of Genomi	2016-01-06;2017-0	MYO7A	chr11	76910708	76910708	http://gnomad.b	0.005737009714	ASJ	0.01300700009	8		3	0	https://www.ncbi.nlm.nih.gov/clinvar/variation/43254/
NM_000260.3(MYO7A):c.4698G>A	concordant	unreportable	unreportable	43255	0	1	SCV000374394	Likely benign	Illumina Clinical Se	10/18/2016	MYO7A	chr11	76910709	76910709	http://gnomad.b	0.001016178847	AFR	0.0109660998	0		0	0	https://www.ncbi.nlm.nih.gov/clinvar/variation/43255/
NM_000260.3(MYO7A):c.494C>T	concordant	reportable	reportable	43264	1	0	SCV000521010	Pathogenic	GeneDx	11/28/2017	MYO7A	chr11	76867729	76867729	http://gnomad.b	1.60E-05	NFE	6.27E-05	0		0	0	https://www.ncbi.nlm.nih.gov/clinvar/variation/43264/
NM_000260.3(MYO7A):c.4992C>T	concordant	unreportable	unreportable	43268	0	2	SCV000336024;S(	Benign;Likely benign	EGL Genetic Diagn	2017-06-30;2016-1	MYO7A	chr11	76912632	76912632	http://gnomad.b	0.000872153499	AFR	0.008691670373	0		0	0	https://www.ncbi.nlm.nih.gov/clinvar/variation/43268/
NM_000260.3(MYO7A):c.5215C>A	concordant	unreportable	unreportable	43276	0	2	SCV000229890;S(	Benign;Likely benign	EGL Genetic Diagn	2017-06-30;2016-1	MYO7A	chr11	76914151	76914151	http://gnomad.b	0.002048360511	AFR	0.02347479947	3		3	0	https://www.ncbi.nlm.nih.gov/clinvar/variation/43276/
NM_000260.3(MYO7A):c.5227C>T	concordant	unreportable	unreportable	43278	0	1	SCV000374437	Likely benign	Illumina Clinical Se	10/18/2016	MYO7A	chr11	76914163	76914163	http://gnomad.b	0.002112371777	ASJ	0.003288669977	2		0	0	https://www.ncbi.nlm.nih.gov/clinvar/variation/43278/
NM_000260.3(MYO7A):c.5392C>T	concordant	reportable	reportable	43282	1	0	SCV000490658	Pathogenic	GeneDx	12/21/2016	MYO7A	chr11	76915186	76915186	http://gnomad.b	4.00E-05	AFR	7.90E-05	0		0	0	https://www.ncbi.nlm.nih.gov/clinvar/variation/43282/
NM_000260.3(MYO7A):c.5481-14G>A	concordant	unreportable	unreportable	43283	0	1	SCV000374448	Likely benign	Illumina Clinical Se	10/18/2016	MYO7A	chr11	76916493	76916493	http://gnomad.b	0.001304229544	AFR	0.01073540002	0		1	0	https://www.ncbi.nlm.nih.gov/clinvar/variation/43283/
NM_000260.3(MYO7A):c.5573T>C	concordant	reportable	reportable	43288	1	0	SCV000059845	Pathogenic	Laboratory for Mol	4/26/2016	MYO7A	chr11	76916599	76916599	http://gnomad.b	4.00E-05	NFE	5.44E-05	0		0	0	https://www.ncbi.nlm.nih.gov/clinvar/variation/43288/
NM_000260.3(MYO7A):c.5598C>T	concordant	unreportable	unreportable	43290	0	2	SCV000520511;S(	Likely benign;Likely benign	GeneDx;Illumina C	2017-11-28;2016-1	MYO7A	chr11	76916624	76916624	http://gnomad.b	0.002184384452	AFR	0.02439190075	7		2	0	https://www.ncbi.nlm.nih.gov/clinvar/variation/43290/
NM_000260.3(MYO7A):c.5619G>A	concordant	unreportable	unreportable	43293	0	1	SCV000374454	Likely benign	Illumina Clinical Se	10/18/2016	MYO7A	chr11	76916645	76916645	http://gnomad.b	0.01629086719	NFE	0.02421619929	38		29	0	https://www.ncbi.nlm.nih.gov/clinvar/variation/43293/
NM_000260.3(MYO7A):c.5648G>A	concordant	reportable	reportable	43294	2	0	SCV000490900;S(	Likely pathogenic;Pathoger	GeneDx;Laborator	2016-12-21;2016-0	MYO7A	chr11	76917153	76917153	http://gnomad.b	2.40E-05	AMR	8.99E-05	0		0	0	https://www.ncbi.nlm.nih.gov/clinvar/variation/43294/
NM_000260.3(MYO7A):c.5824G>T	concordant	reportable	reportable	43300	1	0	SCV000374474	Likely pathogenic	Illumina Clinical Se	10/18/2016	MYO7A	chr11	76918415	76918415	http://gnomad.b	1.60E-05	NFE	2.74E-05	0		0	0	https://www.ncbi.nlm.nih.gov/clinvar/variation/43300/
NM_000260.3(MYO7A):c.5835C>T	concordant	unreportable	unreportable	43302	0	2	SCV000230564;S(	Benign;Likely benign	EGL Genetic Diagn	2017-06-30;2016-1	MYO7A	chr11	76918426	76918426	http://gnomad.b	0.001088191522	AFR	0.01219169982	3		1	0	https://www.ncbi.nlm.nih.gov/clinvar/variation/43302/
NM_000260.3(MYO7A):c.5866G>A	concordant	unreportable	unreportable	43305	0	1	SCV000374488	Likely benign	Illumina Clinical Se	10/18/2016	MYO7A	chr11	76919484	76919484	http://gnomad.b	0.004344764679	ASJ	0.01546919998	6		3	0	https://www.ncbi.nlm.nih.gov/clinvar/variation/43305/
NM_000260.3(MYO7A):c.6025delG	concordant	reportable	reportable	43313	1	0	SCV000059870	Pathogenic	Laboratory for Mol	4/26/2016	MYO7A	chr11	76919820	76919821	http://gnomad.b	2.40E-05	NFE	1.28E-05	0		0	0	https://www.ncbi.nlm.nih.gov/clinvar/variation/43313/
NM_000260.3(MYO7A):c.6240C>T	concordant	unreportable	unreportable	43323	0	2	SCV000604427;S(	Benign;Likely benign	ARUP Laboratories	2017-06-30;2016-1	MYO7A	chr11	76922868	76922868	http://gnomad.b	0.03019731473	NFE	0.04034660012	138		60	0	https://www.ncbi.nlm.nih.gov/clinvar/variation/43323/
NM_000260.3(MYO7A):c.640G>A	concordant	reportable	reportable	43327	1	0	SCV000611217	Pathogenic	Fulgent Genetics,F	5/23/2017	MYO7A	chr11	76867955	76867955	http://gnomad.b	8.00E-06	EAS	5.80E-05	0		0	0	https://www.ncbi.nlm.nih.gov/clinvar/variation/43327/
NM_000260.3(MYO7A):c.6519C>T	concordant	unreportable	unreportable	43331	0	1	SCV000374526	Likely benign	Illumina Clinical Se	10/18/2016	MYO7A	chr11	76924985	76924985	http://gnomad.b	0.01360239402	ASJ	0.03208120167	30		13	0	https://www.ncbi.nlm.nih.gov/clinvar/variation/43331/
NM_000260.3(MYO7A):c.6559-11C>T	concordant	unreportable	unreportable	43334	0	1	SCV000374528	Likely benign	Illumina Clinical Se	10/18/2016	MYO7A	chr11	76925641	76925641	http://gnomad.b	0.01836323193	SAS	0.02528179996	41		38	0	https://www.ncbi.nlm.nih.gov/clinvar/variation/43334/
NM_000260.3(MYO7A):c.905G>A	concordant	unreportable	unreportable	11852	0	1	SCV000374225	Likely benign	Illumina Clinical Se	10/18/2016	MYO7A	chr11	76869378	76869378	http://gnomad.b	0.002104370369	NFE	0.004762160126	3		0	0	https://www.ncbi.nlm.nih.gov/clinvar/variation/11852/
NM_000260.3(MYO7A):c.93C>T	concordant	unreportable	unreportable	43344	0	1	SCV000374156	Likely benign	Illumina Clinical Se	10/18/2016	MYO7A	chr11	76853829	76853829	http://gnomad.b	0.004304757637	AFR	0.05344659835	24		8	0	https://www.ncbi.nlm.nih.gov/clinvar/variation/43344/
NM_000260.3(MYO7A):c.999T>G	concordant	reportable	reportable	43345	1	0	SCV000521009	Pathogenic	GeneDx	11/28/2017	MYO7A	chr11	76869472	76869472		1.60E-05					0	0	https://www.ncbi.nlm.nih.gov/clinvar/variation/43345/
NM_000337.5(SGCD):c.731C>T	concordant	unreportable	unreportable	43356	0	1	SCV000342514	Uncertain significance	EGL Genetic Diagn	6/30/2017	SGCD	chr5	156186259	156186259	http://gnomad.b	4.80E-05	NFE	6.28E-05	0		0	0	https://www.ncbi.nlm.nih.gov/clinvar/variation/43356/
NM_000337.5(SGCD):c.848A>G	concordant	unreportable	unreportable	43358	0	2	SCV000455500;S(	Likely benign;Likely benign	Illumina Clinical Se	2016-10-18;2017-1	SGCD	chr5	156186376	156186376	http://gnomad.b	0.0006882422613	EAS	0.007597730029	0		0	0	https://www.ncbi.nlm.nih.gov/clinvar/variation/43358/
NM_000441.1(SLC26A4):c.1545-5T>G	concordant	unreportable	unreportable	43513	0	2	SCV000605144;S(	Benign;Benign	ARUP Laboratories	2017-06-30;2017-0	SLC26A4	chr7	107338482	107338482	http://gnomad.b	0.001091696961	AFR	0.02862749994	8		7	0	https://www.ncbi.nlm.nih.gov/clinvar/variation/43513/
NM_000441.1(SLC26A4):c.164+2T>C	concordant	reportable	reportable	43518	1	0	SCV000330380	Pathogenic	GeneDx	11/28/2017	SLC26A4	chr7	107302252	107302252		1.40E-05					0	0	https://www.ncbi.nlm.nih.gov/clinvar/variation/43518/
NM_000441.1(SLC26A4):c.416-7T>C	concordant	unreportable	unreportable	43558	0	1	SCV000343166	Likely benign	EGL Genetic Diagn	6/30/2017	SLC26A4	chr7	107314602	107314602	http://gnomad.b	0.00024011749	AFR	0.00686095003	0		0	0	https://www.ncbi.nlm.nih.gov/clinvar/variation/43558/
NM_000492.3(CFTR):c.1210-13_1210-	concordant	unreportable	unreportable	439475	0	2	SCV000602978;S(	Benign;Benign	ARUP Laboratories	2017-06-30;2016-0	CFTR	chr7	117188660	117188662		3.08E-05					0	0	https://www.ncbi.nlm.nih.gov/clinvar/variation/439475/
NM_004004.5(GJB2):c.-6T>A	concordant	unreportable	unreportable	44721	0	3	SCV000331239;S(	Benign;Likely benign;Likely	EGL Genetic Diagn	2017-06-30;2016-1	GJB2	chr13	20763726	20763726	http://gnomad.b	0.0004386675682	AFR	0.01242799964	1		0	0	https://www.ncbi.nlm.nih.gov/clinvar/variation/44721/
NM_004004.5(GJB2):c.31_68del38	concordant	reportable	reportable	44738	1	0	SCV000613511	Pathogenic	Athena Diagnostics	8/17/2017	GJB2	chr13	20763652	20763690	http://gnomad.b	1.40E-05	NFE	4.53E-05	0		0	0	https://www.ncbi.nlm.nih.gov/clinvar/variation/44738/
NM_004004.5(GJB2):c.663G>C	concordant	unreportable	unreportable	44764	0	1	SCV000599766	Uncertain significance	Division of Genomi	5/17/2017	GJB2	chr13	20763058	20763058	http://gnomad.b	5.31E-05	NFE	7.31E-05	0		0	0	https://www.ncbi.nlm.nih.gov/clinvar/variation/44764/
NM_153676.3(USH1C):c.1211-1152G>	concordant	unreportable	unreportable	45425	0	3	SCV000332672;S(	Benign;Benign;Likely benig	EGL Genetic Diagn	2017-06-30;2017-1	USH1C	chr11	17538989	17538989	http://gnomad.b	0.006680855149	EAS	0.0629971996	33		17	0	https://www.ncbi.nlm.nih.gov/clinvar/variation/45425/
NM_153676.3(USH1C):c.1211-1134G>	concordant	unreportable	unreportable	45426	0	2	SCV000515230;S(	Benign;Uncertain significar	GeneDx;Illumina C	2017-11-28;2016-1	USH1C	chr11	17538971	17538971	http://gnomad.b	0.004896626768	AFR	0.05102369934	29		9	0	https://www.ncbi.nlm.nih.gov/clinvar/variation/45426/
NM_153676.3(USH1C):c.1211-1129G>	concordant	unreportable	unreportable	45427	0	1	SCV000369479	Uncertain significance	Illumina Clinical Se	10/18/2016	USH1C	chr11	17538966	17538966	http://gnomad.b	0.001440184344	AFR	0.01681430079	4		1	0	https://www.ncbi.nlm.nih.gov/clinvar/variation/45427/
NM_033056.3(PCDH15):c.3502-14dup	concordant	unreportable	unreportable	46468	0	1	SCV000699759	Benign	Laboratory Corpora	1/25/2018	PCDH15	chr10	55626630	55626630		5.14E-06					0	0	https://www.ncbi.nlm.nih.gov/clinvar/variation/46468/
NM_033056.3(PCDH15):c.3502-8C>T	concordant	unreportable	unreportable	46469	0	1	SCV000363159	Uncertain significance	Illumina Clinical Se	10/18/2016	PCDH15	chr10	55626625	55626625	http://gnomad.b	0.002160160469	ASJ	0.008278150111	4		7	0	https://www.ncbi.nlm.nih.gov/clinvar/variation/46469/
NM_033056.3(PCDH15):c.5726G>A	concordant	unreportable	unreportable	46508	0	1	SCV000063456	Uncertain significance	Laboratory for Mol	4/26/2016	PCDH15	chr10	55581760	55581760	http://gnomad.b	0.0001774417528	AMR	0.0005959119881	0		0	0	https://www.ncbi.nlm.nih.gov/clinvar/variation/46508/
NM_153676.3(USH1C):c.101A>G	concordant	unreportable	unreportable	47971	0	1	SCV000515227	Likely benign	GeneDx	11/28/2017	USH1C	chr11	17554805	17554805	http://gnomad.b	0.00170421814	AFR	0.01858399995	1		1	0	https://www.ncbi.nlm.nih.gov/clinvar/variation/47971/
NM_153676.3(USH1C):c.1430G>A	concordant	unreportable	unreportable	47979	0	1	SCV000064945	Uncertain significance	Laboratory for Mol	4/26/2016	USH1C	chr11	17532052	17532052	http://gnomad.b	0.0003440440376	NFE	0.0004208899918	0		0	0	https://www.ncbi.nlm.nih.gov/clinvar/variation/47979/
NM_153676.3(USH1C):c.1906C>T	concordant	unreportable	unreportable	47986	0	1	SCV000340855	Uncertain significance	EGL Genetic Diagn	6/30/2017	USH1C	chr11	17531010	17531010	http://gnomad.b	0.0004320553031	AFR	0.003661569906	0		0	0	https://www.ncbi.nlm.nih.gov/clinvar/variation/47986/
NM_153676.3(USH1C):c.216G>A	concordant	reportable	reportable	5143	1	0	SCV000064958	Pathogenic	Laboratory for Mol	4/26/2016	USH1C	chr11	17552978	17552978	http://gnomad.b	7.20E-05	NFE	8.07E-05	0		0	0	https://www.ncbi.nlm.nih.gov/clinvar/variation/5143/
NM_153676.3(USH1C):c.225T>C	concordant	unreportable	unreportable	47996	0	3	SCV000332675;S(	Benign;Benign;Uncertain si	EGL Genetic Diagn	2017-06-30;2017-1	USH1C	chr11	17552969	17552969	http://gnomad.b	0.006744863343	EAS	0.05858099833	26		22	0	https://www.ncbi.nlm.nih.gov/clinvar/variation/47996/
NM_005709.3(USH1C):c.1541C>A	concordant	unreportable	unreportable	48002	0	2	SCV000369468;S(	Uncertain significance;Unc	Illumina Clinical Se	2016-10-18;2016-0	USH1C	chr11	17519758	17519758	http://gnomad.b	6.40E-05	NFE	0.0001969669975	0		0	0	https://www.ncbi.nlm.nih.gov/clinvar/variation/48002/
NM_153676.3(USH1C):c.2499C>T	concordant	unreportable	unreportable	48007	0	1	SCV000369462	Uncertain significance	Illumina Clinical Se	10/18/2016	USH1C	chr11	17518352	17518352	http://gnomad.b	0.002184279588	AFR	0.02633300051	6		1	0	https://www.ncbi.nlm.nih.gov/clinvar/variation/48007/
NM_005709.3(USH1C):c.1646+1145G>	concordant	unreportable	unreportable	48009	0	1	SCV000345732	Benign	EGL Genetic Diagn	6/30/2017	USH1C	chr11	17517160	17517160	http://gnomad.b	0.003688472124	NFE	0.005218870007	0		2	0	https://www.ncbi.nlm.nih.gov/clinvar/variation/48009/
NM_153676.3(USH1C):c.388G>A	concordant	unreportable	unreportable	5149	0	1	SCV000332784	Benign	EGL Genetic Diagn	6/30/2017	USH1C	chr11	17548878	17548878	http://gnomad.b	0.003744479293	AFR	0.04406379908	16		14	0	https://www.ncbi.nlm.nih.gov/clinvar/variation/5149/
NM_153676.3(USH1C):c.819+10G>C	concordant	unreportable	unreportable	48025	0	1	SCV000369504	Uncertain significance	Illumina Clinical Se	10/18/2016	USH1C	chr11	17544956	17544956	http://gnomad.b	0.01251360174	ASJ	0.02101520076	24		22	0	https://www.ncbi.nlm.nih.gov/clinvar/variation/48025/
NM_000337.5(SGCD):c.451T>G	concordant	unreportable	unreportable	8176	0	2	SCV000339546;S(	Uncertain significance;Unc	EGL Genetic Diagn	2016-11-03;2017-1	SGCD	chr5	156022010	156022010	http://gnomad.b	0.0001920676078	SAS	0.0006192959845	0		0	0	https://www.ncbi.nlm.nih.gov/clinvar/variation/8176/
NM_000337.5(SGCD):c.507G>A	concordant	unreportable	unreportable	48120	0	2	SCV000455491;S(	Likely benign;Benign	Illumina Clinical Se	2016-10-18;2017-1	SGCD	chr5	156074478	156074478	http://gnomad.b	0.00196869298	AFR	0.02048560046	3		4	0	https://www.ncbi.nlm.nih.gov/clinvar/variation/48120/
NM_000337.5(SGCD):c.699+13_699+1	concordant	unreportable	unreportable	48122	0	2	SCV000331869;S(	Benign;Benign	EGL Genetic Diagn	2017-06-30;2017-1	SGCD	chr5	156184725	156184728	http://gnomad.b	0.001712602836	AFR	0.02216489986	4		2	0	https://www.ncbi.nlm.nih.gov/clinvar/variation/48122/
NM_000337.5(SGCD):c.699+18C>G	concordant	unreportable	unreportable	48123	0	1	SCV000236386	Likely benign	GeneDx	11/28/2017	SGCD	chr5	156184733	156184733	http://gnomad.b	0.0006962450783	EAS	0.01136210002	1		0	0	https://www.ncbi.nlm.nih.gov/clinvar/variation/48123/
NM_000337.5(SGCD):c.699+71G>A	concordant	unreportable	unreportable	48124	0	1	SCV000337546	Benign	EGL Genetic Diagn	6/30/2017	SGCD	chr5	156184786	156184786	http://gnomad.b	0.0006242197253	AFR	0.007126629818	1		0	0	https://www.ncbi.nlm.nih.gov/clinvar/variation/48124/
NM_206933.2(USH2A):c.10190_10191	concordant	reportable	reportable	48343	1	0	SCV000335427	Pathogenic	EGL Genetic Diagn	6/30/2017	USH2A	chr1	215960207	215960209		8.02E-06					0	0	https://www.ncbi.nlm.nih.gov/clinvar/variation/48343/
NM_206933.2(USH2A):c.11411delC	concordant	reportable	reportable	48375	1	0	SCV000065392	Pathogenic	Laboratory for Mol	4/26/2016	USH2A	chr1	215916655	215916656		8.02E-06					0	0	https://www.ncbi.nlm.nih.gov/clinvar/variation/48375/
NM_206933.2(USH2A):c.11677C>A	concordant	unreportable	unreportable	48380	0	1	SCV000707026	Benign	EGL Genetic Diagn	6/30/2017	USH2A	chr1	215914751	215914751	http://gnomad.b	0.01253388077	FIN	0.03194829822	52		15	0	https://www.ncbi.nlm.nih.gov/clinvar/variation/48380/
NM_206933.2(USH2A):c.11864G>A	concordant	reportable	reportable	2357	3	0	SCV000344373;S(	Pathogenic;Pathogenic;Pat	EGL Genetic Diagn	2017-06-30;2016-1	USH2A	chr1	215901574	215901574	http://gnomad.b	0.0001363249988	NFE	0.0001884590019	0		0	0	https://www.ncbi.nlm.nih.gov/clinvar/variation/2357/
NM_206933.2(USH2A):c.12067-2A>G	concordant	reportable	reportable	48390	1	0	SCV000341017	Pathogenic	EGL Genetic Diagn	6/30/2017	USH2A	chr1	215853720	215853720	http://gnomad.b	4.81E-05	AMR	0.0004473610024	0		0	0	https://www.ncbi.nlm.nih.gov/clinvar/variation/48390/
NM_206933.2(USH2A):c.12295-3T>A	concordant	reportable	reportable	48395	2	1	SCV000297410;S(	Uncertain significance;Path	Division of Genomi	2016-01-06;2017-0	USH2A	chr1	215848961	215848961	http://gnomad.b	4.01E-05	NFE	1.81E-05	0		0	0	https://www.ncbi.nlm.nih.gov/clinvar/variation/48395/
NM_206933.2(USH2A):c.12445T>C	concordant	unreportable	unreportable	48399	0	1	SCV000341026	Benign	EGL Genetic Diagn	6/30/2017	USH2A	chr1	215848808	215848808	http://gnomad.b	0.00258215586	AFR	0.03015829995	6		4	0	https://www.ncbi.nlm.nih.gov/clinvar/variation/48399/
NM_206933.2(USH2A):c.1256G>T	concordant	reportable	reportable	2359	2	0	SCV000232370;S(	Pathogenic;Pathogenic	EGL Genetic Diagn	2017-06-30;2017-1	USH2A	chr1	216497582	216497582	http://gnomad.b	5.61E-05	NFE	9.00E-05	0		0	0	https://www.ncbi.nlm.nih.gov/clinvar/variation/2359/
NM_206933.2(USH2A):c.12608A>G	concordant	unreportable	unreportable	48402	0	1	SCV000526228	Likely benign	GeneDx	11/28/2017	USH2A	chr1	215848645	215848645	http://gnomad.b	0.001210886754	FIN	0.02502470091	10		2	0	https://www.ncbi.nlm.nih.gov/clinvar/variation/48402/
NM_206933.2(USH2A):c.12667T>C	concordant	unreportable	unreportable	48404	0	1	SCV000065423	Uncertain significance	Laboratory for Mol	4/26/2016	USH2A	chr1	215848586	215848586	http://gnomad.b	0.0001122676461	AFR	0.0001309929939	0		0	0	https://www.ncbi.nlm.nih.gov/clinvar/variation/48404/
NM_206933.2(USH2A):c.13709G>A	concordant	unreportable	unreportable	48422	0	1	SCV000332495	Likely benign	EGL Genetic Diagn	6/30/2017	USH2A	chr1	215847544	215847544	http://gnomad.b	0.002975092621	ASJ	0.01889480092	3		5	0	https://www.ncbi.nlm.nih.gov/clinvar/variation/48422/
NM_206933.2(USH2A):c.13763C>A	concordant	unreportable	unreportable	48423	0	1	SCV000605539	Benign	ARUP Laboratories	6/30/2017	USH2A	chr1	215847490	215847490	http://gnomad.b	0.002742538211	AFR	0.03038820066	3		2	0	https://www.ncbi.nlm.nih.gov/clinvar/variation/48423/
NM_206933.2(USH2A):c.14074G>A	concordant	unreportable	unreportable	48427	0	1	SCV000515239	Likely benign	GeneDx	11/28/2017	USH2A	chr1	215844373	215844373	http://gnomad.b	0.004739298488	NFE	0.007872389629	6		2	0	https://www.ncbi.nlm.nih.gov/clinvar/variation/48427/
NM_206933.2(USH2A):c.14333C>A	concordant	unreportable	unreportable	48436	0	1	SCV000605553	Benign	ARUP Laboratories	6/30/2017	USH2A	chr1	215823944	215823944	http://gnomad.b	0.002566117624	AFR	0.02934259921	2		4	0	https://www.ncbi.nlm.nih.gov/clinvar/variation/48436/
NM_206933.2(USH2A):c.14519T>C	concordant	unreportable	unreportable	48442	0	1	SCV000617260	Uncertain significance	GeneDx	11/28/2017	USH2A	chr1	215821933	215821933	http://gnomad.b	0.0004811470546	NFE	0.0004393359995	0		0	0	https://www.ncbi.nlm.nih.gov/clinvar/variation/48442/
NM_206933.2(USH2A):c.14960C>T	concordant	unreportable	unreportable	48451	0	1	SCV000605547	Benign	ARUP Laboratories	6/30/2017	USH2A	chr1	215813908	215813908	http://gnomad.b	0.004057673494	AMR	0.04738790169	54		17	0	https://www.ncbi.nlm.nih.gov/clinvar/variation/48451/
NM_206933.2(USH2A):c.15091C>T	concordant	unreportable	unreportable	48455	0	1	SCV000707025	Benign	EGL Genetic Diagn	6/30/2017	USH2A	chr1	215808007	215808007	http://gnomad.b	0.01068146461	FIN	0.05359229818	48		8	0	https://www.ncbi.nlm.nih.gov/clinvar/variation/48455/
NM_206933.2(USH2A):c.15428G>A	concordant	unreportable	unreportable	48463	0	1	SCV000605552	Benign	ARUP Laboratories	6/30/2017	USH2A	chr1	215802247	215802247	http://gnomad.b	0.004490705843	AFR	0.05286199972	28		11	0	https://www.ncbi.nlm.nih.gov/clinvar/variation/48463/
NM_206933.2(USH2A):c.1606T>C	concordant	reportable	reportable	48471	1	0	SCV000490868	Pathogenic	GeneDx	12/21/2016	USH2A	chr1	216495263	216495263	http://gnomad.b	2.41E-05	NFE	3.59E-05	0		0	0	https://www.ncbi.nlm.nih.gov/clinvar/variation/48471/
NM_206933.2(USH2A):c.1841-2A>G	concordant	reportable	reportable	48476	1	0	SCV000354157	Pathogenic	Illumina Clinical Se	10/18/2016	USH2A	chr1	216462754	216462754	http://gnomad.b	1.60E-05	NFE	3.59E-05	0		0	0	https://www.ncbi.nlm.nih.gov/clinvar/variation/48476/
NM_206933.2(USH2A):c.1966G>A	concordant	unreportable	unreportable	48481	0	1	SCV000515232	Uncertain significance	GeneDx	11/28/2017	USH2A	chr1	216462627	216462627	http://gnomad.b	0.000473127937	NFE	0.001084720017	0		0	0	https://www.ncbi.nlm.nih.gov/clinvar/variation/48481/
NM_206933.2(USH2A):c.2209C>T	concordant	reportable	reportable	2361	1	0	SCV000335429	Pathogenic	EGL Genetic Diagn	6/30/2017	USH2A	chr1	216420527	216420527	http://gnomad.b	1.60E-05	SAS	3.25E-05	0		0	0	https://www.ncbi.nlm.nih.gov/clinvar/variation/2361/
NM_206933.2(USH2A):c.2522C>A	concordant	unreportable	unreportable	48487	0	1	SCV000605549	Benign	ARUP Laboratories	6/30/2017	USH2A	chr1	216420214	216420214	http://gnomad.b	0.004915719074	FIN	0.01538390014	10		2	0	https://www.ncbi.nlm.nih.gov/clinvar/variation/48487/
NM_206933.2(USH2A):c.2546G>A	concordant	unreportable	unreportable	48488	0	1	SCV000343168	Benign	EGL Genetic Diagn	6/30/2017	USH2A	chr1	216420190	216420190	http://gnomad.b	0.001371269106	AFR	0.01561889984	3		2	0	https://www.ncbi.nlm.nih.gov/clinvar/variation/48488/
NM_206933.2(USH2A):c.2777G>A	concordant	unreportable	unreportable	48491	0	1	SCV000523476	Likely benign	GeneDx	11/28/2017	USH2A	chr1	216419959	216419959	http://gnomad.b	8.02E-05	AMR	0.0001787630026	0		0	0	https://www.ncbi.nlm.nih.gov/clinvar/variation/48491/
NM_206933.2(USH2A):c.3405G>C	concordant	unreportable	unreportable	48501	0	1	SCV000065523	Uncertain significance	Laboratory for Mol	4/26/2016	USH2A	chr1	216373375	216373375	http://gnomad.b	3.21E-05	ASJ	0.0001016469978	0		0	0	https://www.ncbi.nlm.nih.gov/clinvar/variation/48501/
NM_206933.2(USH2A):c.4106C>T	concordant	unreportable	unreportable	48511	0	1	SCV000702404	Uncertain significance	EGL Genetic Diagn	6/30/2017	USH2A	chr1	216370040	216370040	http://gnomad.b	0.0001363249988	ASJ	0.000814664003	0		0	0	https://www.ncbi.nlm.nih.gov/clinvar/variation/48511/
NM_206933.2(USH2A):c.4338_4339de	concordant	reportable	reportable	2353	2	0	SCV000354106;S(	Pathogenic;Pathogenic	Illumina Clinical Se	2016-10-18;2016-0	USH2A	chr1	216363621	216363623	http://gnomad.b	2.41E-05	NFE	8.99E-06	0		0	0	https://www.ncbi.nlm.nih.gov/clinvar/variation/2353/
NM_206933.2(USH2A):c.4440C>T	concordant	unreportable	unreportable	48516	0	1	SCV000354099	Uncertain significance	Illumina Clinical Se	10/18/2016	USH2A	chr1	216348781	216348781	http://gnomad.b	0.003071322032	AFR	0.0359476991	8		5	0	https://www.ncbi.nlm.nih.gov/clinvar/variation/48516/
NM_206933.2(USH2A):c.4560C>T	concordant	unreportable	unreportable	48518	0	1	SCV000332785	Uncertain significance	EGL Genetic Diagn	6/30/2017	USH2A	chr1	216348661	216348661	http://gnomad.b	0.002750557329	ASJ	0.004276989959	3		1	0	https://www.ncbi.nlm.nih.gov/clinvar/variation/48518/
NM_206933.2(USH2A):c.5975A>G	concordant	unreportable	unreportable	48547	0	2	SCV000605545;S(	Likely benign;Uncertain sig	ARUP Laboratories	2017-06-30;2017-0	USH2A	chr1	216243517	216243517	http://gnomad.b	0.00261423233	FIN	0.01185030025	4		0	0	https://www.ncbi.nlm.nih.gov/clinvar/variation/48547/
NM_206933.2(USH2A):c.6240G>T	concordant	unreportable	unreportable	48555	0	2	SCV000605548;S(	Benign;Benign	ARUP Laboratories	2017-06-30;2017-0	USH2A	chr1	216219858	216219858	http://gnomad.b	0.005428942599	AFR	0.01052150037	10		1	0	https://www.ncbi.nlm.nih.gov/clinvar/variation/48555/
NM_206933.2(USH2A):c.6369C>T	concordant	unreportable	unreportable	48560	0	1	SCV000605551	Benign	ARUP Laboratories	6/30/2017	USH2A	chr1	216173861	216173861	http://gnomad.b	0.004691183782	AFR	0.05020919815	22		14	0	https://www.ncbi.nlm.nih.gov/clinvar/variation/48560/
NM_206933.2(USH2A):c.6713A>C	concordant	unreportable	unreportable	48568	0	1	SCV000605541	Benign	ARUP Laboratories	6/30/2017	USH2A	chr1	216166454	216166454	http://gnomad.b	0.01885294542	FIN	0.04485109821	88		30	0	https://www.ncbi.nlm.nih.gov/clinvar/variation/48568/
NM_206933.2(USH2A):c.7060C>T	concordant	unreportable	unreportable	48575	0	1	SCV000605540	Uncertain significance	ARUP Laboratories	6/30/2017	USH2A	chr1	216138719	216138719	http://gnomad.b	0.0004891661722	AFR	0.004248369951	0		0	0	https://www.ncbi.nlm.nih.gov/clinvar/variation/48575/
NM_206933.2(USH2A):c.8357T>C	concordant	unreportable	unreportable	48599	0	1	SCV000065621	Likely benign	Laboratory for Mol	4/26/2016	USH2A	chr1	216052307	216052307	http://gnomad.b	0.0007698352873	ASJ	0.005284550134	2		0	0	https://www.ncbi.nlm.nih.gov/clinvar/variation/48599/
NM_206933.2(USH2A):c.8559-2A>G	concordant	reportable	reportable	48604	1	0	SCV000700946	Pathogenic	EGL Genetic Diagn	6/30/2017	USH2A	chr1	216051224	216051224	http://gnomad.b	3.21E-05	EAS	0.0004642530112	0		0	0	https://www.ncbi.nlm.nih.gov/clinvar/variation/48604/
NM_206933.2(USH2A):c.9262G>A	concordant	unreportable	unreportable	48618	0	1	SCV000230763	Benign	EGL Genetic Diagn	6/30/2017	USH2A	chr1	216011442	216011442	http://gnomad.b	0.006728039647	FIN	0.01008970011	12		4	0	https://www.ncbi.nlm.nih.gov/clinvar/variation/48618/
NM_206933.2(USH2A):c.949C>A	concordant	reportable	reportable	2358	1	0	SCV000339779	Pathogenic	EGL Genetic Diagn	6/30/2017	USH2A	chr1	216498841	216498841	http://gnomad.b	4.01E-05	NFE	6.29E-05	0		0	0	https://www.ncbi.nlm.nih.gov/clinvar/variation/2358/
NM_017777.3(MKS1):c.1408-34_1408	concordant	reportable	reportable	188400	3	0	SCV000700971;S(	Pathogenic;Pathogenic;Pat	EGL Genetic Diagn	2017-06-30;2017-1	MKS1	chr17	56283913	56283942	http://gnomad.b	0.000247877053	FIN	0.006818859838	0		0	0	https://www.ncbi.nlm.nih.gov/clinvar/variation/188400/
NM_000784.3(CYP27A1):c.1184+1G>A	concordant	reportable	reportable	65833	1	0	SCV000322121	Pathogenic	GeneDx	11/28/2017	CYP27A1	chr2	219678911	219678911	http://gnomad.b	0.0001280266295	SAS	0.0007147499709	0		0	0	https://www.ncbi.nlm.nih.gov/clinvar/variation/65833/
NM_000317.2(PTS):c.155A>G	concordant	reportable	reportable	479	1	0	SCV000636854	Pathogenic	Invitae	10/5/2017	PTS	chr11	112099388	112099388	http://gnomad.b	4.80E-05	EAS	0.001453489996	0		0	0	https://www.ncbi.nlm.nih.gov/clinvar/variation/479/
NM_000404.2(GLB1):c.1445G>A	concordant	reportable	reportable	932	1	0	SCV000696685	Pathogenic	Laboratory Corpora	1/25/2018	GLB1	chr3	33058235	33058235	http://gnomad.b	8.00E-06	EAS	5.80E-05	0		0	0	https://www.ncbi.nlm.nih.gov/clinvar/variation/932/
NM_000404.2(GLB1):c.622C>T	concordant	reportable	reportable	939	1	0	SCV000696688	Pathogenic	Laboratory Corpora	1/25/2018	GLB1	chr3	33099692	33099692	http://gnomad.b	4.00E-05	AFR	0.000196412002	0		0	0	https://www.ncbi.nlm.nih.gov/clinvar/variation/939/

Supplemental Table 2

NM_152618.2(BBS12):c.1063C>T	concordant	reportable	reportable	1147	1	0	SCV000636538	Pathogenic	Invitae	10/5/2017	BBS12	chr4	123664110	123664110	http://gnomad.b	4.00E-05	EAS	5.80E-05	0		0	0	https://www.ncbi.nlm.nih.gov/clinvar/variation/1147/
NM_000263.3(NAGLU):c.889C>T	concordant	reportable	reportable	1562	3	0	SCV000330977;S(	Pathogenic;Pathogenic;Pat	EGL Genetic Diagn(	2017-06-30;2017-1	NAGLU	chr17	40693092	40693092	http://gnomad.b	0.0001040349557	NFE	9.85E-05	0		0	0	https://www.ncbi.nlm.nih.gov/clinvar/variation/1562/
NM_000263.3(NAGLU):c.1693C>T	concordant	reportable	reportable	1567	1	0	SCV000402914	Pathogenic	Illumina Clinical Se	10/18/2016	NAGLU	chr17	40695717	40695717	http://gnomad.b	4.00E-05	EAS	0.0003487970098	0		0	0	https://www.ncbi.nlm.nih.gov/clinvar/variation/1567/
NM_000124.2(ERCC6):c.2203C>T	concordant	reportable	reportable	1701	3	0	SCV000617667;S(	Pathogenic;Pathogenic;Pat	GeneDx;Genetic Se	2017-11-28;2017-0	ERCC6	chr10	50686483	50686483	http://gnomad.b	9.60E-05	AMR	0.0002084699954	0		0	0	https://www.ncbi.nlm.nih.gov/clinvar/variation/1701/
NM_000124.2(ERCC6):c.3284C>G	concordant	unreportable	unreportable	1707	0	1	SCV000362801	Likely benign	Illumina Clinical Se	10/18/2016	ERCC6	chr10	50678722	50678722	http://gnomad.b	0.003152857577	AFR	0.04248369858	15		9	0	https://www.ncbi.nlm.nih.gov/clinvar/variation/1707/
NM_206933.2(USH2A):c.956G>A	concordant	unreportable	unreportable	2355	0	1	SCV000341015	Uncertain significance	EGL Genetic Diagn(	6/30/2017	USH2A	chr1	216498834	216498834	http://gnomad.b	8.02E-06	AMR	0.0004478149931	0		0	0	https://www.ncbi.nlm.nih.gov/clinvar/variation/2355/
NM_024312.4(GNPTAB):c.3503_3504(	concordant	reportable	reportable	2771	4	0	SCV000226964;S(	Pathogenic;Pathogenic;Pat	EGL Genetic Diagn(	2017-06-30;2017-1	GNPTAB	chr12	102147247	102147249	http://gnomad.b	0.0004876878798	SAS	0.0007147029974	0		0	0	https://www.ncbi.nlm.nih.gov/clinvar/variation/2771/
NM_147127.4(EVC2):c.1195C>T	concordant	reportable	reportable	3383	2	0	SCV000680767;S(	Pathogenic;Pathogenic	GeneDx;Invitae	2018-02-07;2017-1	EVC2	chr4	5642516	5642516	http://gnomad.b	4.80E-05	SAS	0.0001299550058	0		0	0	https://www.ncbi.nlm.nih.gov/clinvar/variation/3383/
NM_000135.2(FANCA):c.1115_1118de	concordant	reportable	reportable	3440	2	0	SCV000594661;S(	Pathogenic;Pathogenic	Genetic Services La	2017-07-05;2017-0	FANCA	chr16	89858441	89858445	http://gnomad.b	8.00E-05	NFE	0.000134271002	0		0	0	https://www.ncbi.nlm.nih.gov/clinvar/variation/3440/
NM_000521.3(HEXB):c.1250C>T	concordant	reportable	reportable	3878	2	0	SCV000321768;S(	Pathogenic;Pathogenic	GeneDx;Illumina C	2017-11-28;2016-1	HEXB	chr5	74014629	74014629	http://gnomad.b	0.0007040675905	NFE	0.001102720038	0		0	0	https://www.ncbi.nlm.nih.gov/clinvar/variation/3878/
NM_000521.3(HEXB):c.850C>T	concordant	reportable	reportable	3887	1	0	SCV000680528	Pathogenic	GeneDx	2/7/2018	HEXB	chr5	74009409	74009409	http://gnomad.b	4.80E-05	SAS	0.0002274059952	0		0	0	https://www.ncbi.nlm.nih.gov/clinvar/variation/3887/
NM_015120.4(ALMS1):c.10775delC	concordant	reportable	reportable	3971	1	0	SCV000702798	Pathogenic	EGL Genetic Diagn(	6/30/2017	ALMS1	chr2	73799775	73799776	http://gnomad.b	0.0001120376446	AFR	0.0001309590007	0		0	0	https://www.ncbi.nlm.nih.gov/clinvar/variation/3971/
NM_024301.4(FKRP):c.826C>A	concordant	reportable	reportable	4221	6	0	SCV000613311;S(	Pathogenic;Pathogenic;Pat	Athena Diagnostics	2017-08-17;2017-0	FKRP	chr19	47259533	47259533	http://gnomad.b	0.001224352614	NFE	0.002247239929	0		0	0	https://www.ncbi.nlm.nih.gov/clinvar/variation/4221/
NM_024301.4(FKRP):c.1364C>A	concordant	reportable	reportable	4226	3	0	SCV000255777;S(	Pathogenic;Pathogenic;Pat	Athena Diagnostics	2015-10-06;2017-0	FKRP	chr19	47260071	47260071		1.60E-05					0	0	https://www.ncbi.nlm.nih.gov/clinvar/variation/4226/
NM_024301.4(FKRP):c.235G>A	concordant	unreportable	unreportable	4229	0	4	SCV000230416;S(	Benign;Likely benign;Likely	EGL Genetic Diagn(	2017-06-30;2017-1	FKRP	chr19	47258942	47258942	http://gnomad.b	0.001264364137	AFR	0.01473829988	1		1	0	https://www.ncbi.nlm.nih.gov/clinvar/variation/4229/
NM_000784.3(CYP27A1):c.1183C>T	concordant	reportable	reportable	4255	3	0	SCV000700806;S(	Pathogenic;Pathogenic;Pat	EGL Genetic Diagn(	2017-06-30;2017-1	CYP27A1	chr2	219678909	219678909	http://gnomad.b	0.0002640549234	AMR	0.0005360650248	0		0	0	https://www.ncbi.nlm.nih.gov/clinvar/variation/4255/
NM_000784.3(CYP27A1):c.845-1G>A	concordant	reportable	reportable	4256	1	0	SCV000617407	Pathogenic	GeneDx	11/28/2017	CYP27A1	chr2	219677646	219677646	http://gnomad.b	7.20E-05	ASJ	0.001116749947	0		0	0	https://www.ncbi.nlm.nih.gov/clinvar/variation/4256/
NM_000784.3(CYP27A1):c.1214G>A	concordant	reportable	reportable	4260	1	0	SCV000702839	Likely pathogenic	EGL Genetic Diagn(	6/30/2017	CYP27A1	chr2	219679132	219679132	http://gnomad.b	8.80E-05	EAS	0.0004058440099	0		0	0	https://www.ncbi.nlm.nih.gov/clinvar/variation/4260/
NM_000784.3(CYP27A1):c.1263+1G>A	concordant	reportable	reportable	4262	2	0	SCV000336773;S(	Pathogenic;Pathogenic	EGL Genetic Diagn(	2017-06-30;2017-1	CYP27A1	chr2	219679182	219679182	http://gnomad.b	9.60E-05	EAS	5.80E-05	0		0	0	https://www.ncbi.nlm.nih.gov/clinvar/variation/4262/
NM_000784.3(CYP27A1):c.1016C>T	concordant	reportable	reportable	4266	3	0	SCV000613048;S(	Likely pathogenic;Likely pat	Athena Diagnostics	2017-08-17;2017-0	CYP27A1	chr2	219677818	219677818	http://gnomad.b	9.60E-05	AFR	0.0001313370012	0		0	0	https://www.ncbi.nlm.nih.gov/clinvar/variation/4266/
NM_031885.3(BBS2):c.72C>G	concordant	reportable	reportable	4570	1	0	SCV000699658	Pathogenic	Laboratory Corpora	1/25/2018	BBS2	chr16	56553703	56553703	http://gnomad.b	5.60E-05	NFE	9.93E-05	0		0	0	https://www.ncbi.nlm.nih.gov/clinvar/variation/4570/
NM_031885.3(BBS2):c.175C>T	concordant	reportable	reportable	4571	1	0	SCV000699654	Likely pathogenic	Laboratory Corpora	1/25/2018	BBS2	chr16	56548535	56548535	http://gnomad.b	1.60E-05	NFE	4.48E-05	0		0	0	https://www.ncbi.nlm.nih.gov/clinvar/variation/4571/
NM_031885.3(BBS2):c.823C>T	concordant	reportable	reportable	4572	4	0	SCV000338211;S(	Pathogenic;Pathogenic;Pat	EGL Genetic Diagn(	2017-06-30;2017-1	BBS2	chr16	56536702	56536702	http://gnomad.b	8.80E-05	FIN	0.000762331998	0		0	0	https://www.ncbi.nlm.nih.gov/clinvar/variation/4572/
NM_031885.3(BBS2):c.311A>C	concordant	reportable	reportable	4577	1	0	SCV000699657	Pathogenic	Laboratory Corpora	1/25/2018	BBS2	chr16	56548399	56548399	http://gnomad.b	8.80E-05	ASJ	0.001015229966	0		0	0	https://www.ncbi.nlm.nih.gov/clinvar/variation/4577/
NM_000199.3(SGSH):c.734G>A	concordant	reportable	reportable	5107	4	0	SCV000231496;S(	Pathogenic;Pathogenic;Pat	EGL Genetic Diagn(	2017-06-30;2017-1	SGSH	chr17	78187614	78187614	http://gnomad.b	0.0003200768184	NFE	0.0006790269981	0		0	0	https://www.ncbi.nlm.nih.gov/clinvar/variation/5107/
NM_000199.3(SGSH):c.220C>T	concordant	reportable	reportable	5108	2	0	SCV000227052;S(	Pathogenic;Pathogenic	EGL Genetic Diagn(	2017-06-30;2017-1	SGSH	chr17	78190860	78190860	http://gnomad.b	0.0001200288069	FIN	0.0002526849858	0		0	0	https://www.ncbi.nlm.nih.gov/clinvar/variation/5108/
NM_000199.3(SGSH):c.197C>G	concordant	reportable	reportable	5111	2	0	SCV000321944;S(	Pathogenic;Pathogenic	GeneDx;Laborator	2017-11-28;2018-0	SGSH	chr17	78190883	78190883	http://gnomad.b	0.0001520364888	NFE	0.0002099620033	0		0	0	https://www.ncbi.nlm.nih.gov/clinvar/variation/5111/
NM_000199.3(SGSH):c.1339G>A	concordant	reportable	reportable	5114	2	0	SCV000491269;S(	Likely pathogenic;Likely pat	GeneDx;Illumina C	2016-12-21;2016-1	SGSH	chr17	78184421	78184421	http://gnomad.b	4.00E-05	AMR	0.0003575260052	0		0	0	https://www.ncbi.nlm.nih.gov/clinvar/variation/5114/
NM_005709.3(USH1C):c.91C>T	concordant	reportable	reportable	5146	1	0	SCV000701079	Pathogenic	EGL Genetic Diagn(	6/30/2017	USH1C	chr11	17554815	17554815	http://gnomad.b	8.00E-06	AFR	6.55E-05	0		0	0	https://www.ncbi.nlm.nih.gov/clinvar/variation/5146/
NM_153717.2(EVC):c.1018C>T	concordant	reportable	reportable	5340	1	0	SCV000322261	Pathogenic	GeneDx	11/28/2017	EVC	chr4	5749953	5749953	http://gnomad.b	8.00E-06	EAS	5.80E-05	0		0	0	https://www.ncbi.nlm.nih.gov/clinvar/variation/5340/
NM_003494.3(DYSF):c.3892A>G	concordant	unreportable	unreportable	6667	0	4	SCV000613205;S(	Likely benign;Benign;Likely	Athena Diagnostics	2017-08-17;2016-1	DYSF	chr2	71829924	71829924	http://gnomad.b	0.004453964918	NFE	0.007161689922	7		3	0	https://www.ncbi.nlm.nih.gov/clinvar/variation/6667/
NM_003494.3(DYSF):c.6124C>T	concordant	reportable	reportable	6668	2	0	SCV000333452;S(	Pathogenic;Pathogenic	EGL Genetic Diagn(	2016-11-03;2017-1	DYSF	chr2	71909727	71909727	http://gnomad.b	8.08E-05	NFE	6.27E-05	0		0	0	https://www.ncbi.nlm.nih.gov/clinvar/variation/6668/
NM_003494.3(DYSF):c.3137G>A	concordant	reportable	reportable	6675	1	0	SCV000228714	Pathogenic	EGL Genetic Diagn(	6/30/2017	DYSF	chr2	71797834	71797834	http://gnomad.b	1.62E-05	AMR	0.0001192819982	0		0	0	https://www.ncbi.nlm.nih.gov/clinvar/variation/6675/
NM_003494.3(DYSF):c.5713C>T	concordant	reportable	reportable	6676	3	0	SCV000613217;S(	Pathogenic;Pathogenic;Pat	Athena Diagnostics	2017-08-17;2017-0	DYSF	chr2	71901372	71901372	http://gnomad.b	1.62E-05	AMR	2.98E-05	0		0	0	https://www.ncbi.nlm.nih.gov/clinvar/variation/6676/
NM_003494.3(DYSF):c.895G>A	concordant	reportable	reportable	6681	1	0	SCV000701792	Pathogenic	EGL Genetic Diagn(	6/30/2017	DYSF	chr2	71744158	71744158		8.08E-06					0	0	https://www.ncbi.nlm.nih.gov/clinvar/variation/6681/
NM_000202.5(IDS):c.1402C>T	concordant	reportable	reportable	10497	1	0	SCV000695962	Pathogenic	Laboratory Corpora	1/25/2018	IDS	chrX	148564528	148564528		1.94E-05					0	0	https://www.ncbi.nlm.nih.gov/clinvar/variation/10497/
NM_000169.2(GLA):c.427G>A	concordant	unreportable	unreportable	10748	3	3	SCV000603838;S(	Uncertain significance;Likel	ARUP Laboratories	2017-06-30;2015-0	GLA	chrX	100656740	100656740	http://gnomad.b	0.0005520634582	NFE	0.001010810025	0	28	0	12	https://www.ncbi.nlm.nih.gov/clinvar/variation/10748/
NM_000500.7(CYP21A2):c.844G>T	concordant	reportable	reportable	12151	2	0	SCV000263011;S(	Pathogenic;Pathogenic	Ambry Genetics;Fu	2016-04-08;2017-0	CYP21A2	chr6	32007887	32007887	http://gnomad.b	0.0237497647	ASJ	0.02037730068	1		186	0	https://www.ncbi.nlm.nih.gov/clinvar/variation/12151/
NM_000500.7(CYP21A2):c.293-13C>G	concordant	reportable	reportable	12155	1	0	SCV000330929	Pathogenic	EGL Genetic Diagn(	6/30/2017	CYP21A2	chr6	32006858	32006858	http://gnomad.b	0.0024401637	ASJ	0.003914750181	0		1	0	https://www.ncbi.nlm.nih.gov/clinvar/variation/12155/
NM_000500.7(CYP21A2):c.955C>T	concordant	reportable	reportable	12169	1	0	SCV000330930	Pathogenic	EGL Genetic Diagn(	6/30/2017	CYP21A2	chr6	32008198	32008198	http://gnomad.b	0.002485481026	AMR	0.0002695739968	0		46	0	https://www.ncbi.nlm.nih.gov/clinvar/variation/12169/
NM_000199.3(SGSH):c.892T>C	concordant	reportable	reportable	30459	3	0	SCV000321948;S(	Pathogenic;Pathogenic;Pat	GeneDx;Illumina C	2017-11-28;2016-1	SGSH	chr17	78185927	78185927	http://gnomad.b	0.0001440345683	NFE	0.0002064190048	0		0	0	https://www.ncbi.nlm.nih.gov/clinvar/variation/30459/
NM_000263.3(NAGLU):c.1694G>A	concordant	reportable	reportable	30795	2	0	SCV000521202;S(	Pathogenic;Pathogenic	GeneDx;Laborator	2017-11-28;2018-0	NAGLU	chr17	40695718	40695718	http://gnomad.b	1.60E-05	NFE	9.03E-05	0		0	0	https://www.ncbi.nlm.nih.gov/clinvar/variation/30795/
NM_032957.4(RTEL1):c.1548G>T	concordant	reportable	reportable	42019	1	0	SCV000699742	Likely pathogenic	Laboratory Corpora	1/25/2018	RTEL1	chr20	62319118	62319118	http://gnomad.b	6.40E-05	ASJ	0.0009334890055	0		0	0	https://www.ncbi.nlm.nih.gov/clinvar/variation/42019/
NM_032957.4(RTEL1):c.2992C>T	concordant	reportable	reportable	42020	1	0	SCV000699750	Pathogenic	Laboratory Corpora	1/25/2018	RTEL1	chr20	62324564	62324564	http://gnomad.b	0.000119929002	NFE	5.42E-05	0		0	0	https://www.ncbi.nlm.nih.gov/clinvar/variation/42020/
NM_032957.4(RTEL1):c.2941C>T	concordant	reportable	reportable	42021	1	0	SCV000699749	Likely pathogenic	Laboratory Corpora	1/25/2018	RTEL1	chr20	62324513	62324513	http://gnomad.b	1.60E-05	EAS	0.0001160359971	0		0	0	https://www.ncbi.nlm.nih.gov/clinvar/variation/42021/
NM_032957.4(RTEL1):c.3028C>T	concordant	reportable	reportable	65417	3	0	SCV000329508;S(	Pathogenic;Pathogenic;Like	GeneDx;Genetic Se	2017-11-28;2017-0	RTEL1	chr20	62324600	62324600	http://gnomad.b	0.0001439148024	FIN	0.0003145500086	0		0	0	https://www.ncbi.nlm.nih.gov/clinvar/variation/65417/
NM_031885.3(BBS2):c.612+12C>A	concordant	unreportable	unreportable	35756	0	1	SCV000398069	Likely benign	Illumina Clinical Se	10/18/2016	BBS2	chr16	56543857	56543857	http://gnomad.b	0.004712301587	AFR	0.05803919956	23		13	0	https://www.ncbi.nlm.nih.gov/clinvar/variation/35756/
NM_000492.3(CFTR):c.3469-20T>C	concordant	unreportable	unreportable	7228	0	2	SCV000709545;S(	Benign;Uncertain significar	EGL Genetic Diagn(	2017-06-30;2018-0	CFTR	chr7	117267556	117267556	http://gnomad.b	0.0003620564808	SAS	0.01201639976	9		3	0	https://www.ncbi.nlm.nih.gov/clinvar/variation/7228/
NM_006432.3(NPC2):c.58G>T	concordant	reportable	reportable	8477	1	0	SCV000698700	Pathogenic	Laboratory Corpora	1/25/2018	NPC2	chr14	74959920	74959920	http://gnomad.b	6.39E-05	NFE	0.0001652500068	0		0	0	https://www.ncbi.nlm.nih.gov/clinvar/variation/8477/
NM_000070.2(CAPN3):c.2306G>A	concordant	reportable	reportable	17613	1	0	SCV000331919	Pathogenic	EGL Genetic Diagn(	6/30/2017	CAPN3	chr15	42703124	42703124	http://gnomad.b	3.20E-05	SAS	6.50E-05	0		0	0	https://www.ncbi.nlm.nih.gov/clinvar/variation/17613/
NM_000492.3(CFTR):c.1585-8G>A	concordant	reportable	reportable	35828	2	0	SCV000071567;S(	Pathogenic;Pathogenic	CFTR2;Laboratory (	2017-06-29;2015-1	CFTR	chr7	117227785	117227785		1.93E-06					0	0	https://www.ncbi.nlm.nih.gov/clinvar/variation/35828/
NM_000492.3(CFTR):c.2620-15C>G	concordant	unreportable	unreportable	35844	0	4	SCV000603043;S(	Likely benign;Benign;Benig	ARUP Laboratories	2017-06-30;2017-0	CFTR	chr7	117242865	117242865	http://gnomad.b	0.001669696643	ASJ	0.01969940029	7		10	0	https://www.ncbi.nlm.nih.gov/clinvar/variation/35844/
NM_000492.3(CFTR):c.2932A>T	concordant	reportable	reportable	35855	1	0	SCV000052161	Likely pathogenic	Laboratory Corpora	11/20/2015	CFTR	chr7	117246751	117246751		1.93E-06					0	0	https://www.ncbi.nlm.nih.gov/clinvar/variation/35855/
NM_000492.3(CFTR):c.3139+18C>T	concordant	unreportable	unreportable	35862	0	1	SCV000603053	Benign	ARUP Laboratories	6/30/2017	CFTR	chr7	117250741	117250741	http://gnomad.b	0.000514197236	FIN	0.003235959914	1		0	0	https://www.ncbi.nlm.nih.gov/clinvar/variation/35862/
NM_000492.3(CFTR):c.3893G>C	concordant	unreportable	unreportable	35878	0	1	SCV000052185	Uncertain significance	Laboratory Corpora	11/20/2015	CFTR	chr7	117292915	117292915		3.85E-06					0	0	https://www.ncbi.nlm.nih.gov/clinvar/variation/35878/
NM_000518.4(HBB):c.316-184T>C	concordant	unreportable	unreportable	36315	0	1	SCV000601280	Likely benign	Quest Diagnostics I	8/1/2017	HBB	chr11	5247140	5247140		1.87E-05					0	0	https://www.ncbi.nlm.nih.gov/clinvar/variation/36315/
NM_000518.4(HBB):c.93-22_95del25	concordant	reportable	reportable	15442	1	0	SCV000052664	Pathogenic	Laboratory Corpora	11/20/2015	HBB	chr11	5248026	5248051		7.00E-06					0	0	https://www.ncbi.nlm.nih.gov/clinvar/variation/15442/
NM_004006.2(DMD):c.8762A>G	concordant	unreportable	unreportable	11269	0	5	SCV000603333;S(	Benign;Benign;Benign;Likel	ARUP Laboratories	2017-06-30;2017-1	DMD	chrX	31496398	31496398	http://gnomad.b	0.02491978203	FIN	0.04198360071	37	1768	36	521	https://www.ncbi.nlm.nih.gov/clinvar/variation/11269/
NM_004006.2(DMD):c.10262C>T	concordant	unreportable	unreportable	11276	0	1	SCV000334846	Uncertain significance	EGL Genetic Diagn(	6/30/2017	DMD	chrX	31196049	31196049		1.90E-05					0	2	https://www.ncbi.nlm.nih.gov/clinvar/variation/11276/
NM_000481.3(AMT):c.959G>A	concordant	reportable	reportable	11979	2	0	SCV000636431;S(	Pathogenic;Pathogenic	Invitae;Laboratory	2017-10-05;2018-0	AMT	chr3	49455325	49455325	http://gnomad.b	0.0001760647918	NFE	0.0001614119974	0		0	0	https://www.ncbi.nlm.nih.gov/clinvar/variation/11979/
NM_000532.4(PCCB):c.1304A>G	concordant	reportable	reportable	12018	1	0	SCV000697269	Pathogenic	Laboratory Corpora	1/25/2018	PCCB	chr3	136046480	136046480	http://gnomad.b	7.99E-05	EAS	0.0005802480155	0		0	0	https://www.ncbi.nlm.nih.gov/clinvar/variation/12018/
NM_000359.2(TGM1):c.968G>A	concordant	reportable	reportable	12484	1	0	SCV000617732	Pathogenic	GeneDx	11/28/2017	TGM1	chr14	24728926	24728926	http://gnomad.b	4.01E-05	NFE	7.35E-05	0		0	0	https://www.ncbi.nlm.nih.gov/clinvar/variation/12484/
NM_000359.2(TGM1):c.1187G>T	concordant	reportable	reportable	12487	1	0	SCV000321963	Likely pathogenic	GeneDx	11/28/2017	TGM1	chr14	24727852	24727852	http://gnomad.b	1.60E-05	FIN	0.0006730079767	0		0	0	https://www.ncbi.nlm.nih.gov/clinvar/variation/12487/
NM_000359.2(TGM1):c.1166G>A	concordant	reportable	reportable	12489	1	0	SCV000616892	Pathogenic	GeneDx	11/28/2017	TGM1	chr14	24727873	24727873	http://gnomad.b	8.02E-06	NFE	3.59E-05	0		0	0	https://www.ncbi.nlm.nih.gov/clinvar/variation/12489/
NM_000318.2(PEX2):c.355C>T	concordant	reportable	reportable	13704	1	0	SCV000696558	Pathogenic	Laboratory Corpora	1/25/2018	PEX2	chr8	77896060	77896060	http://gnomad.b	0.0002000512131	ASJ	0.002538070083	0		0	0	https://www.ncbi.nlm.nih.gov/clinvar/variation/13704/
NM_000070.2(CAPN3):c.1715G>A	concordant	reportable	reportable	17614	3	0	SCV000255657;S(	Pathogenic;Pathogenic;Pat	Athena Diagnostics	2015-10-06;2017-0	CAPN3	chr15	42695170	42695170	http://gnomad.b	8.00E-06	SAS	3.25E-05	0		0	0	https://www.ncbi.nlm.nih.gov/clinvar/variation/17614/
NM_000070.2(CAPN3):c.956C>T	concordant	reportable	reportable	17617	1	0	SCV000338158	Likely pathogenic	EGL Genetic Diagn(	6/30/2017	CAPN3	chr15	42684847	42684847	http://gnomad.b	8.00E-06	NFE	3.59E-05	0		0	0	https://www.ncbi.nlm.nih.gov/clinvar/variation/17617/
NM_000070.2(CAPN3):c.550delA	concordant	reportable	reportable	17621	4	0	SCV000255667;S(	Pathogenic;Pathogenic;Pat	Athena Diagnostics	2017-08-17;2017-0	CAPN3	chr15	42680000	42680001	http://gnomad.b	0.000240034565	NFE	0.0004208750033	0		0	0	https://www.ncbi.nlm.nih.gov/clinvar/variation/17621/
NM_000070.2(CAPN3):c.1469G>A	concordant	reportable	reportable	17622	2	0	SCV000331813;S(	Pathogenic;Pathogenic	EGL Genetic Diagn(	2017-06-30;2016-1	CAPN3	chr15	42693953	42693953	http://gnomad.b	1.60E-05	AFR	0.0001307530038	0		0	0	https://www.ncbi.nlm.nih.gov/clinvar/variation/17622/
NM_000260.3(MYO7A):c.652G>A	concordant	reportable	reportable	29924	1	0	SCV000271251	Likely pathogenic	Laboratory for Mol	4/26/2016	MYO7A	chr11	76867967	76867967	http://gnomad.b	4.80E-05	AMR	0.0002084570006	0		0	0	https://www.ncbi.nlm.nih.gov/clinvar/variation/29924/
NM_000359.2(TGM1):c.944G>A	concordant	reportable	reportable	39530	1	0	SCV000616890	Pathogenic	GeneDx	11/28/2017	TGM1	chr14	24728950	24728950	http://gnomad.b	7.22E-05	AMR	8.99E-05	0		0	0	https://www.ncbi.nlm.nih.gov/clinvar/variation/39530/
NM_000478.4(ALPL):c.107C>T	concordant	unreportable	unreportable	295541	0	1	SCV000354295	Uncertain significance	Illumina Clinical Se	10/18/2016	ALPL	chr1	21887164	21887164		2.82E-06					0	0	https://www.ncbi.nlm.nih.gov/clinvar/variation/295541/
NM_000478.4(ALPL):c.997+11C>T	concordant	unreportable	unreportable	295546	0	1	SCV000354307	Likely benign	Illumina Clinical Se	10/18/2016	ALPL	chr1	21900303	21900303	http://gnomad.b	0.0005070879629	EAS	0.01032720041	1		2	0	https://www.ncbi.nlm.nih.gov/clinvar/variation/295546/
NM_000191.2(HMGCL):c.594C>T	concordant	unreportable	unreportable	296852	0	1	SCV000356638	Uncertain significance	Illumina Clinical Se	10/18/2016	HMGCL	chr1	24134781	24134781	http://gnomad.b	0.000151997568	EAS	0.00115955004	1		0	0	https://www.ncbi.nlm.nih.gov/clinvar/variation/296852/
NM_000191.2(HMGCL):c.393A>G	concordant	unreportable	unreportable	382367	0	2	SCV000522277;S(	Likely benign;Benign	GeneDx;Invitae	2017-11-28;2017-1	HMGCL	chr1	24140784	24140784	http://gnomad.b	0.0008959856642	AFR	0.008625200018	1		0	0	https://www.ncbi.nlm.nih.gov/clinvar/variation/382367/
NM_000191.2(HMGCL):c.61-17C>T	concordant	unreportable	unreportable	382366	0	1	SCV000522276	Likely benign	GeneDx	11/28/2017	HMGCL	chr1	24147100	24147100	http://gnomad.b	0.001031983488	AFR	0.01059100032	1		0	0	https://www.ncbi.nlm.nih.gov/clinvar/variation/382366/
NM_000191.2(HMGCL):c.48G>A	concordant	unreportable	unreportable	296855	0	1	SCV000356641	Likely benign	Illumina Clinical Se	10/18/2016	HMGCL	chr1	24151858	24151858	http://gnomad.b	0.004079934721	AFR	0.02978019975	11		5	0	https://www.ncbi.nlm.nih.gov/clinvar/variation/296855/
NM_000310.3(PPT1):c.363-4G>A	concordant	unreportable	unreportable	130021	0	3	SCV000357398;S(	Likely benign;Benign;Benig	Illumina Clinical Se	2016-10-18;2017-1	PPT1	chr1	40557075	40557075	http://gnomad.b	0.002563402415	AMR	0.04831859842	52		15	0	https://www.ncbi.nlm.nih.gov/clinvar/variation/130021/
NM_000310.3(PPT1):c.234+7C>T	concordant	unreportable	unreportable	410224	0	1	SCV000550822	Uncertain significance	Invitae	3/14/2017	PPT1	chr1	40558063	40558063	http://gnomad.b	8.36E-06	AFR	0.0002614720142	0		0	0	https://www.ncbi.nlm.nih.gov/clinvar/variation/410224/
NM_000310.3(PPT1):c.198C>T	concordant	unreportable	unreportable	382693	0	1	SCV000522729	Likely benign	GeneDx	11/28/2017	PPT1	chr1	40558106	40558106	http://gnomad.b	8.36E-06	AFR	6.53E-05	0		0	0	https://www.ncbi.nlm.nih.gov/clinvar/variation/382693/
NM_015506.2(MMACHC):c.67G>T	concordant	unreportable	unreportable	297479	0	1	SCV000357916	Uncertain significance	Illumina Clinical Se	10/18/2016	MMACHC	chr1	45966071	45966071	http://gnomad.b	0.00045097791	NFE	0.0007608719752	0		0	0	https://www.ncbi.nlm.nih.gov/clinvar/variation/297479/
NM_015506.2(MMACHC):c.126G>A	concordant	unreportable	unreportable	465312	0	1	SCV000640281	Likely benign	Invitae	10/5/2017	MMACHC	chr1	45973072	45973072	http://gnomad.b	0.0002452686879	EAS	0.0003478660074	1		0	0	https://www.ncbi.nlm.nih.gov/clinvar/variation/465312/
NM_015506.2(MMACHC):c.276+17T>A	concordant	unreportable	unreportable	387463	0	1	SCV000529517	Likely benign	GeneDx	11/28/2017	MMACHC	chr1	45973239	45973239	http://gnomad.b	0.0005854800937	AFR	0.006422860082	0		0	0	https://www.ncbi.nlm.nih.gov/clinvar/variation/387463/
NM_015506.2(MMACHC):c.316G>A	concordant	unreportable	unreportable	203826	0	2	SCV000238987;S(	Uncertain significance;Unc	GeneDx;Invitae	2017-11-28;2017-1	MMACHC	chr1	45973923	45973923	http://gnomad.b	0.0003639470853	AMR	0.0008636090206	0		0	0	https://www.ncbi.nlm.nih.gov/clinvar/variation/203826/
NM_015506.2(MMACHC):c.334C>T	concordant	unreportable	unreportable	297483	0	1	SCV000357923	Uncertain significance	Illumina Clinical Se	10/18/2016	MMACHC	chr1	45973941	45973941	http://gnomad.b	1.58E-05	EAS	0.0001739330037	0		0	0	https://www.ncbi.nlm.nih.gov/clinvar/variation/297483/
NM_015506.2(MMACHC):c.683C>T	concordant	unreportable	unreportable	288244	0	1	SCV000342296	Uncertain significance	EGL Genetic Diagn(	6/30/2017	MMACHC	chr1	45974721	45974721	http://gnomad.b	0.00015032597	EAS	0.001217529993	0		0	0	https://www.ncbi.nlm.nih.gov/clinvar/variation/288244/
NM_015506.2(MMACHC):c.738C>T	concordant	unreportable	unreportable	138239	0	2	SCV000357930;S(	Likely benign;Benign	Illumina Clinical Se	2016-10-18;2017-1	MMACHC	chr1	45974776	45974776	http://gnomad.b	0.00616336477	AFR	0.04618110135	38		12	0	https://www.ncbi.nlm.nih.gov/clinvar/variation/138239/
NM_015506.2(MMACHC):c.799C>T	concordant	unreportable	unreportable	297489	0	2	SCV000527610;S(	Likely benign;Uncertain sig	GeneDx;Illumina C	2017-11-28;2016-1	MMACHC	chr1	45974837	45974837	http://gnomad.b	0.0004588898032	EAS	0.002842559945	0		1	0	https://www.ncbi.nlm.nih.gov/clinvar/variation/297489/
NM_017739.3(POMGNT1):c.1880C>T	concordant	unreportable	unreportable	471404	0	1	SCV000649963	Uncertain significance	Invitae	10/5/2017	POMGNT1	chr1	46655145	46655145		8.40E-06					0	0	https://www.ncbi.nlm.nih.gov/clinvar/variation/471404/
NM_017739.3(POMGNT1):c.1167T>C	concordant	unreportable	unreportable	471400	0	1	SCV000649952	Likely benign	Invitae	10/5/2017	POMGNT1	chr1	46658616	46658616		1.68E-05					0	0	https://www.ncbi.nlm.nih.gov/clinvar/variation/471400/
NM_017739.3(POMGNT1):c.534+15C>	concordant	unreportable	unreportable	387862	0	1	SCV000530032	Likely benign	GeneDx	11/28/2017	POMGNT1	chr1	46661468	46661468	http://gnomad.b	3.92E-05	AFR	0.001288220054	0		0	0	https://www.ncbi.nlm.nih.gov/clinvar/variation/387862/
NM_017739.3(POMGNT1):c.120+13C>	concordant	unreportable	unreportable	257664	0	2	SCV000527094;S(	Benign;Uncertain significar	GeneDx;Illumina C	2017-11-28;2016-1	POMGNT1	chr1	46663361	46663361	http://gnomad.b	0.002172464571	ASJ	0.02487809956	8		7	0	https://www.ncbi.nlm.nih.gov/clinvar/variation/257664/
NM_000098.2(CPT2):c.912G>T	concordant	unreportable	unreportable	429622	0	1	SCV000582236	Uncertain significance	GeneDx	11/28/2017	CPT2	chr1	53676258	53676258	http://gnomad.b	9.23E-05	AFR	0.0008496730006	0		0	0	https://www.ncbi.nlm.nih.gov/clinvar/variation/429622/
NM_000016.4(ACADM):c.469-9A>G	concordant	unreportable	unreportable	226107	0	2	SCV000526101;S(	Likely benign;Benign	GeneDx;Invitae	2017-11-28;2017-1	ACADM	chr1	76205656	76205656	http://gnomad.b	0.0002230127389	AFR	0.007511740085	1		0	0	https://www.ncbi.nlm.nih.gov/clinvar/variation/226107/
NM_000642.2(AGL):c.1480C>T	concordant	unreportable	unreportable	456454	0	1	SCV000626672	Uncertain significance	Invitae	10/5/2017	AGL	chr1	100343253	100343253	http://gnomad.b	2.23E-05	AFR	0.0001960530062	0		0	0	https://www.ncbi.nlm.nih.gov/clinvar/variation/456454/
NM_000642.2(AGL):c.1979A>C	concordant	unreportable	unreportable	291335	0	1	SCV000346073	Uncertain significance	Illumina Clinical Se	10/18/2016	AGL	chr1	100346711	100346711	http://gnomad.b	1.67E-05	AMR	5.96E-05	0		0	0	https://www.ncbi.nlm.nih.gov/clinvar/variation/291335/
NM_000642.2(AGL):c.2930G>A	concordant	unreportable	unreportable	456478	0	1	SCV000626712	Uncertain significance	Invitae	10/5/2017	AGL	chr1	100356893	100356893	http://gnomad.b	0.0001116401614	EAS	0.0009857360274	0		0	0	https://www.ncbi.nlm.nih.gov/clinvar/variation/456478/

Supplemental Table 2

NM_000642.2(AGL):c.2949+20C>T	concordant	unreportable	unreportable	386919	0		1	SCV000528733	Likely benign	GeneDx	11/28/2017	AGL	chr1	100356932	100356932	http://gnomad.b	0.0008763752672	ASJ	0.01079209987	0		1	0	https://www.ncbi.nlm.nih.gov/clinvar/variation/386919/
NM_000642.2(AGL):c.3084-8T>C	concordant	unreportable	unreportable	456482	0		1	SCV000626716	Benign	Invitae	10/5/2017	AGL	chr1	100357980	100357980	http://gnomad.b	0.0003237564682	EAS	0.007364879828	1		0	0	https://www.ncbi.nlm.nih.gov/clinvar/variation/456482/
NM_000642.2(AGL):c.3260-20T>C	concordant	unreportable	unreportable	256737	0		1	SCV000520633	Likely benign	GeneDx	7/13/2017	AGL	chr1	100361822	100361822	http://gnomad.b	0.0004298146215	AFR	0.01182700042	2		0	0	https://www.ncbi.nlm.nih.gov/clinvar/variation/256737/
NM_000642.2(AGL):c.4162-11G>A	concordant	unreportable	unreportable	291345	0		2	SCV000528923;S(	Benign;Uncertain significar	GeneDx;Illumina C	2017-11-28;2016-1	AGL	chr1	100380934	100380934	http://gnomad.b	0.0006391399242	SAS	0.01861600019	12		3	0	https://www.ncbi.nlm.nih.gov/clinvar/variation/291345/
NM_000396.3(CTSK):c.-1-9C>T	concordant	unreportable	unreportable	283076	0		2	SCV000334955;S(	Uncertain significance;Unc	EGL Genetic Diagn	2016-11-03;2016-1	CTSK	chr1	150779291	150779291	http://gnomad.b	0.0003134515493	NFE	0.001576749957	1		0	0	https://www.ncbi.nlm.nih.gov/clinvar/variation/283076/
NM_014625.2(NPHS2):c.724G>A	concordant	unreportable	unreportable	289749	0		2	SCV000344158;S(	Uncertain significance;Unc	EGL Genetic Diagn	2017-06-30;2016-1	NPHS2	chr1	179526176	179526176	http://gnomad.b	5.35E-05	EAS	0.0001743169996	0		0	0	https://www.ncbi.nlm.nih.gov/clinvar/variation/289749/
NM_014625.2(NPHS2):c.-26G>T	concordant	unreportable	unreportable	293852	0		1	SCV000351510	Uncertain significance	Illumina Clinical Se	10/18/2016	NPHS2	chr1	179545025	179545025	http://gnomad.b	0.001100459885	FIN	0.00709220022	0		2	0	https://www.ncbi.nlm.nih.gov/clinvar/variation/293852/
NM_005562.2(LAMC2):c.80-6C>T	concordant	unreportable	unreportable	293986	0		1	SCV000351649	Uncertain significance	Illumina Clinical Se	10/18/2016	LAMC2	chr1	183177010	183177010	http://gnomad.b	0.0004423515183	NFE	0.001731849974	1		0	0	https://www.ncbi.nlm.nih.gov/clinvar/variation/293986/
NM_005562.2(LAMC2):c.269-15C>T	concordant	unreportable	unreportable	293987	0		1	SCV000351652	Uncertain significance	Illumina Clinical Se	10/18/2016	LAMC2	chr1	183184573	183184573	http://gnomad.b	0.0007391189926	AFR	0.02306590043	4		2	0	https://www.ncbi.nlm.nih.gov/clinvar/variation/293987/
NM_005562.2(LAMC2):c.2754+9T>C	concordant	unreportable	unreportable	290573	0		2	SCV000345148;S(	Benign;Uncertain significar	EGL Genetic Diagn	2017-06-30;2016-1	LAMC2	chr1	183206648	183206648	http://gnomad.b	0.002466529668	ASJ	0.01413930021	8		5	0	https://www.ncbi.nlm.nih.gov/clinvar/variation/290573/
NM_000228.2(LAMB3):c.2702-14G>A	concordant	unreportable	unreportable	295081	0		1	SCV000353602	Likely benign	Illumina Clinical Se	10/18/2016	LAMB3	chr1	209792018	209792018	http://gnomad.b	8.12E-05	AFR	0.06226519868	37		0	0	https://www.ncbi.nlm.nih.gov/clinvar/variation/295081/
NM_000228.2(LAMB3):c.2397A>G	concordant	unreportable	unreportable	295092	0		1	SCV000353615	Uncertain significance	Illumina Clinical Se	10/18/2016	LAMB3	chr1	209796486	209796486	http://gnomad.b	1.96E-05	AFR	6.53E-05	0		0	0	https://www.ncbi.nlm.nih.gov/clinvar/variation/295092/
NM_000228.2(LAMB3):c.2359-15G>A	concordant	unreportable	unreportable	295093	0		1	SCV000353616	Uncertain significance	Illumina Clinical Se	10/18/2016	LAMB3	chr1	209796539	209796539	http://gnomad.b	0.000487119333	AFR	0.01390529983	2		2	0	https://www.ncbi.nlm.nih.gov/clinvar/variation/295093/
NM_000228.2(LAMB3):c.1615C>T	concordant	unreportable	unreportable	295103	0		1	SCV000353627	Uncertain significance	Illumina Clinical Se	10/18/2016	LAMB3	chr1	209799354	209799354	http://gnomad.b	5.88E-05	NFE	0.0001897220063	0		0	0	https://www.ncbi.nlm.nih.gov/clinvar/variation/295103/
NM_000228.2(LAMB3):c.1008G>A	concordant	unreportable	unreportable	295119	0		1	SCV000353646	Uncertain significance	Illumina Clinical Se	10/18/2016	LAMB3	chr1	209803206	209803206	http://gnomad.b	0.0003303452948	EAS	0.003072819905	0		0	0	https://www.ncbi.nlm.nih.gov/clinvar/variation/295119/
NM_000228.2(LAMB3):c.813C>T	concordant	unreportable	unreportable	295128	0		1	SCV000353656	Uncertain significance	Illumina Clinical Se	10/18/2016	LAMB3	chr1	209805937	209805937	http://gnomad.b	3.92E-05	AFR	0.0001325560006	0		0	0	https://www.ncbi.nlm.nih.gov/clinvar/variation/295128/
NM_000228.2(LAMB3):c.582G>T	concordant	unreportable	unreportable	295133	0		1	SCV000353661	Uncertain significance	Illumina Clinical Se	10/18/2016	LAMB3	chr1	209806461	209806461	http://gnomad.b	0.0002883522489	FIN	0.00089686102	0		0	0	https://www.ncbi.nlm.nih.gov/clinvar/variation/295133/
NM_000228.2(LAMB3):c.372+10C>T	concordant	unreportable	unreportable	295142	0		1	SCV000353671	Likely benign	Illumina Clinical Se	10/18/2016	LAMB3	chr1	209811243	209811243	http://gnomad.b	0.001749710248	AMR	0.07197269797	112		13	0	https://www.ncbi.nlm.nih.gov/clinvar/variation/295142/
NM_000182.4(HADHA):c.1690-6G>A	concordant	unreportable	unreportable	203739	0		1	SCV000429506	Uncertain significance	Illumina Clinical Se	10/18/2016	HADHA	chr2	26416647	26416647	http://gnomad.b	0.0004738246905	AFR	0.0139851002	4		1	0	https://www.ncbi.nlm.nih.gov/clinvar/variation/203739/
NM_000182.4(HADHA):c.1690-14C>T	concordant	unreportable	unreportable	335379	0		1	SCV000429510	Uncertain significance	Illumina Clinical Se	10/18/2016	HADHA	chr2	26416655	26416655	http://gnomad.b	0.000157006998	EAS	0.006087659858	0		0	0	https://www.ncbi.nlm.nih.gov/clinvar/variation/335379/
NM_000182.4(HADHA):c.68-10T>G	concordant	unreportable	unreportable	335389	0		3	SCV000513208;S(	Likely benign;Uncertain sig	GeneDx;Illumina C	2017-11-28;2016-1	HADHA	chr2	26462021	26462021	http://gnomad.b	0.0004598062085	AFR	0.01231009979	1		1	0	https://www.ncbi.nlm.nih.gov/clinvar/variation/335389/
NM_004543.4(NEB):c.16947+13C>T	concordant	unreportable	unreportable	331422	0		2	SCV000529167;S(	Likely benign;Uncertain sig	GeneDx;Illumina C	2017-11-28;2016-1	NEB	chr2	152382659	152382659	http://gnomad.b	0.0002116915705	AFR	0.007588639855	0		0	0	https://www.ncbi.nlm.nih.gov/clinvar/variation/331422/
NM_001271208.1(NEB):c.22147G>A	concordant	unreportable	unreportable	465560	0		1	SCV000640702	Uncertain significance	Invitae	10/5/2017	NEB	chr2	152382680	152382680		2.58E-06					0	0	https://www.ncbi.nlm.nih.gov/clinvar/variation/465560/
NM_004543.4(NEB):c.16737+13A>C	concordant	unreportable	unreportable	257790	0		2	SCV000534101;S(	Likely benign;Uncertain sig	GeneDx;Illumina C	2017-11-28;2016-1	NEB	chr2	152383982	152383982	http://gnomad.b	0.0001523146666	AFR	0.00418523006	0		0	0	https://www.ncbi.nlm.nih.gov/clinvar/variation/257790/
NM_004543.4(NEB):c.13999-6C>T	concordant	unreportable	unreportable	129717	0		4	SCV000519543;S(	Benign;Likely benign;Likely	GeneDx;Genetic Se	2017-11-28;2014-0	NEB	chr2	152417628	152417628	http://gnomad.b	0.009714577804	FIN	0.09232489765	157		60	0	https://www.ncbi.nlm.nih.gov/clinvar/variation/129717/
NM_004543.4(NEB):c.5763+15C>T	concordant	unreportable	unreportable	257820	0		1	SCV000416986	Uncertain significance	Illumina Clinical Se	10/18/2016	NEB	chr2	152520047	152520047	http://gnomad.b	0.0003872406778	AMR	0.002017830033	0		0	0	https://www.ncbi.nlm.nih.gov/clinvar/variation/257820/
NM_004543.4(NEB):c.5763+4C>T	concordant	unreportable	unreportable	129750	0		3	SCV000526407;S(	Benign;Likely benign;Benig	GeneDx;Genetic Se	2017-11-28;2014-0	NEB	chr2	152520058	152520058	http://gnomad.b	0.001352760768	AFR	0.03280510008	9		6	0	https://www.ncbi.nlm.nih.gov/clinvar/variation/129750/
NM_004543.4(NEB):c.3987+11A>T	concordant	unreportable	unreportable	331512	0		2	SCV000530922;S(	Benign;Uncertain significar	GeneDx;Illumina C	2017-11-28;2016-1	NEB	chr2	152530980	152530980	http://gnomad.b	0.001298547073	EAS	0.0405735001	11		7	0	https://www.ncbi.nlm.nih.gov/clinvar/variation/331512/
NM_004543.4(NEB):c.612+8T>C	concordant	unreportable	unreportable	129753	0		5	SCV000614182;S(	Benign;Benign;Likely benig	Athena Diagnostics	2017-08-17;2017-1	NEB	chr2	152580766	152580766	http://gnomad.b	0.009650037691	ASJ	0.04700680077	55		53	0	https://www.ncbi.nlm.nih.gov/clinvar/variation/129753/
NM_001875.4(CPS1):c.-29T>G	concordant	unreportable	unreportable	334007	0		2	SCV000518669;S(	Benign;Uncertain significar	GeneDx;Illumina C	2017-11-28;2016-1	CPS1	chr2	211421429	211421429	http://gnomad.b	0.01138018791	ASJ	0.0266915001	28		11	0	https://www.ncbi.nlm.nih.gov/clinvar/variation/334007/
NM_001875.4(CPS1):c.195C>T	concordant	unreportable	unreportable	334011	0		1	SCV000426812	Uncertain significance	Illumina Clinical Se	10/18/2016	CPS1	chr2	211438090	211438090	http://gnomad.b	0.0009763593002	EAS	0.007248059846	0		2	0	https://www.ncbi.nlm.nih.gov/clinvar/variation/334011/
NM_001875.4(CPS1):c.449G>A	concordant	unreportable	unreportable	334013	0		2	SCV000566492;S(	Uncertain significance;Unc	GeneDx;Illumina C	2017-11-28;2016-1	CPS1	chr2	211442212	211442212	http://gnomad.b	0.007530771324	ASJ	0.01096890029	4		7	0	https://www.ncbi.nlm.nih.gov/clinvar/variation/334013/
NM_001875.4(CPS1):c.486T>C	concordant	unreportable	unreportable	203644	0		1	SCV000426815	Uncertain significance	Illumina Clinical Se	10/18/2016	CPS1	chr2	211444452	211444452	http://gnomad.b	0.003137154473	NFE	0.00634089997	7		0	0	https://www.ncbi.nlm.nih.gov/clinvar/variation/203644/
NM_001875.4(CPS1):c.529-4A>T	concordant	unreportable	unreportable	377751	0		2	SCV000705281;S(	Likely benign;Likely benign	EGL Genetic Diagn	2017-06-30;2017-0	CPS1	chr2	211447337	211447337	http://gnomad.b	0.0006402356067	AFR	0.009673199616	0		0	0	https://www.ncbi.nlm.nih.gov/clinvar/variation/377751/
NM_001875.4(CPS1):c.712-16T>C	concordant	unreportable	unreportable	382067	0		1	SCV000521851	Likely benign	GeneDx	11/28/2017	CPS1	chr2	211454814	211454814	http://gnomad.b	0.001232453543	AFR	0.01492150035	1		1	0	https://www.ncbi.nlm.nih.gov/clinvar/variation/382067/
NM_001875.4(CPS1):c.783C>T	concordant	unreportable	unreportable	334016	0		1	SCV000426818	Uncertain significance	Illumina Clinical Se	10/18/2016	CPS1	chr2	211454901	211454901	http://gnomad.b	0.001088400531	FIN	0.002915839897	0		0	0	https://www.ncbi.nlm.nih.gov/clinvar/variation/334016/
NM_001875.4(CPS1):c.1032C>T	concordant	unreportable	unreportable	128850	0		3	SCV000332071;S(	Benign;Likely benign;Benig	EGL Genetic Diagn	2017-06-30;2014-0	CPS1	chr2	211456639	211456639	http://gnomad.b	1.60E-05	AFR	0.6638460159	40326		1	0	https://www.ncbi.nlm.nih.gov/clinvar/variation/128850/
NM_001875.4(CPS1):c.1068C>G	concordant	unreportable	unreportable	334020	0		2	SCV000523932;S(	Benign;Likely benign	GeneDx;Illumina C	2017-11-28;2016-1	CPS1	chr2	211456675	211456675	http://gnomad.b	0.003601325288	AFR	0.0413169004	19		7	0	https://www.ncbi.nlm.nih.gov/clinvar/variation/334020/
NM_001875.4(CPS1):c.1214C>A	concordant	unreportable	unreportable	203645	0		1	SCV000238760	Likely benign	GeneDx	11/28/2017	CPS1	chr2	211459281	211459281	http://gnomad.b	0.001080397586	AFR	0.01276849955	0		0	0	https://www.ncbi.nlm.nih.gov/clinvar/variation/203645/
NM_001875.4(CPS1):c.1359+7G>A	concordant	unreportable	unreportable	334022	0		2	SCV000522398;S(	Benign;Uncertain significar	GeneDx;Illumina C	2017-11-28;2016-1	CPS1	chr2	211460313	211460313	http://gnomad.b	0.002000736271	AFR	0.02812750079	4		1	0	https://www.ncbi.nlm.nih.gov/clinvar/variation/334022/
NM_001875.4(CPS1):c.1437G>A	concordant	unreportable	unreportable	389590	0		2	SCV000532179;S(	Likely benign;Benign	GeneDx;Invitae	2017-11-28;2017-1	CPS1	chr2	211464173	211464173	http://gnomad.b	0.0004161531444	AFR	0.003140540095	0		0	0	https://www.ncbi.nlm.nih.gov/clinvar/variation/389590/
NM_001875.4(CPS1):c.2025C>T	concordant	unreportable	unreportable	382478	0		1	SCV000522425	Likely benign	GeneDx	11/28/2017	CPS1	chr2	211471498	211471498	http://gnomad.b	0.0006882532772	NFE	0.001034450019	0		0	0	https://www.ncbi.nlm.nih.gov/clinvar/variation/382478/
NM_001875.4(CPS1):c.2192+6A>T	concordant	unreportable	unreportable	334025	0		2	SCV000522342;S(	Likely benign;Uncertain sig	GeneDx;Illumina C	2017-11-28;2016-1	CPS1	chr2	211471671	211471671	http://gnomad.b	0.000672247387	NFE	0.001186470035	1		0	0	https://www.ncbi.nlm.nih.gov/clinvar/variation/334025/
NM_001875.4(CPS1):c.2265C>A	concordant	unreportable	unreportable	258478	0		3	SCV000520332;S(	Benign;Likely benign;Benig	GeneDx;Illumina C	2017-11-28;2016-1	CPS1	chr2	211473157	211473157	http://gnomad.b	0.01106807305	FIN	0.03912770003	44		18	0	https://www.ncbi.nlm.nih.gov/clinvar/variation/258478/
NM_001875.4(CPS1):c.2394C>A	concordant	unreportable	unreportable	334027	0		2	SCV000523934;S(	Benign;Likely benign	GeneDx;Illumina C	2017-11-28;2016-1	CPS1	chr2	211476843	211476843	http://gnomad.b	0.002456904141	AFR	0.02728700079	7		2	0	https://www.ncbi.nlm.nih.gov/clinvar/variation/334027/
NM_001875.4(CPS1):c.2448C>T	concordant	unreportable	unreportable	137024	0		2	SCV000426836;S(	Likely benign;Benign	Illumina Clinical Se	2016-10-18;2017-1	CPS1	chr2	211476897	211476897	http://gnomad.b	0.02005538038	NFE	0.02879609913	71		42	0	https://www.ncbi.nlm.nih.gov/clinvar/variation/137024/
NM_001875.4(CPS1):c.3033G>C	concordant	unreportable	unreportable	382785	0		1	SCV000522902	Likely benign	GeneDx	11/28/2017	CPS1	chr2	211507281	211507281	http://gnomad.b	0.0007282680026	AFR	0.006795609836	0		0	0	https://www.ncbi.nlm.nih.gov/clinvar/variation/382785/
NM_001875.4(CPS1):c.3355G>A	concordant	unreportable	unreportable	235274	0		2	SCV000521522;S(	Benign;Likely benign	GeneDx;Illumina C	2017-11-28;2016-1	CPS1	chr2	211513215	211513215	http://gnomad.b	0.004057493157	AFR	0.04882989824	20		10	0	https://www.ncbi.nlm.nih.gov/clinvar/variation/235274/
NM_001875.4(CPS1):c.3481-8C>T	concordant	unreportable	unreportable	258480	0		3	SCV000518505;S(	Likely benign;Uncertain sig	GeneDx;Illumina C	2017-11-28;2016-1	CPS1	chr2	211518741	211518741	http://gnomad.b	0.003993469597	NFE	0.008067119867	2		2	0	https://www.ncbi.nlm.nih.gov/clinvar/variation/258480/
NM_001875.4(CPS1):c.4126G>A	concordant	unreportable	unreportable	258484	0		2	SCV000517746;S(	Benign;Benign	GeneDx;Invitae	2017-11-28;2017-1	CPS1	chr2	211539650	211539650	http://gnomad.b	0.01410118924	FIN	0.03099489957	37		18	0	https://www.ncbi.nlm.nih.gov/clinvar/variation/258484/
NM_001875.4(CPS1):c.4260C>G	concordant	unreportable	unreportable	334035	0		2	SCV000512758;S(	Likely benign;Uncertain sig	GeneDx;Illumina C	2017-11-28;2016-1	CPS1	chr2	211540550	211540550	http://gnomad.b	0.00146453895	AFR	0.01947709918	4		0	0	https://www.ncbi.nlm.nih.gov/clinvar/variation/334035/
NM_001875.4(CPS1):c.4275-10A>G	concordant	unreportable	unreportable	203646	0		2	SCV000426850;S(	Likely benign;Benign	Illumina Clinical Se	2016-10-18;2017-1	CPS1	chr2	211541721	211541721	http://gnomad.b	0.03663748259	NFE	0.04934889823	195		109	0	https://www.ncbi.nlm.nih.gov/clinvar/variation/203646/
NM_001875.4(CPS1):c.4404+4T>A	concordant	unreportable	unreportable	334036	0		1	SCV000426851	Uncertain significance	Illumina Clinical Se	10/18/2016	CPS1	chr2	211541864	211541864	http://gnomad.b	0.001760647918	SAS	0.02319690026	20		1	0	https://www.ncbi.nlm.nih.gov/clinvar/variation/334036/
NM_004328.4(BCS1L):c.999C>T	concordant	unreportable	unreportable	383490	0		1	SCV000523901	Likely benign	GeneDx	11/28/2017	BCS1L	chr2	219527715	219527715	http://gnomad.b	3.35E-05	AFR	0.0001306850027	0		0	0	https://www.ncbi.nlm.nih.gov/clinvar/variation/383490/
NM_000030.2(AGXT):c.*19G>A	concordant	unreportable	unreportable	204062	0		1	SCV000429377	Uncertain significance	Illumina Clinical Se	10/18/2016	AGXT	chr2	241818257	241818257	http://gnomad.b	0.001799656185	NFE	0.007095839828	3		2	0	https://www.ncbi.nlm.nih.gov/clinvar/variation/204062/
NM_000060.2(BTD):c.-45C>T	concordant	unreportable	unreportable	343902	0		1	SCV000441818	Uncertain significance	Illumina Clinical Se	10/18/2016	BTD	chr3	15643313	15643313	http://gnomad.b	0.0003113845657	EAS	0.007425460033	2		0	0	https://www.ncbi.nlm.nih.gov/clinvar/variation/343902/
NM_000060.2(BTD):c.-5G>T	concordant	unreportable	unreportable	343904	0		1	SCV000441821	Uncertain significance	Illumina Clinical Se	10/18/2016	BTD	chr3	15643353	15643353	http://gnomad.b	0.0001643418541	EAS	0.003711439902	0		1	0	https://www.ncbi.nlm.nih.gov/clinvar/variation/343904/
NM_000203.3(IDUA):c.299+6C>T	concordant	unreportable	unreportable	92640	0		1	SCV000451737	Likely benign	Illumina Clinical Se	10/18/2016	IDUA	chr4	981743	981743	http://gnomad.b	0.003422870214	FIN	0.02995420061	19		8	0	https://www.ncbi.nlm.nih.gov/clinvar/variation/92640/
NM_000203.3(IDUA):c.667G>A	concordant	unreportable	unreportable	426817	0		1	SCV000577361	Uncertain significance	GeneDx	11/28/2017	IDUA	chr4	995544	995544	http://gnomad.b	0.0004747046282	AFR	0.005632920191	0		1	0	https://www.ncbi.nlm.nih.gov/clinvar/variation/426817/
NM_000203.3(IDUA):c.1031C>G	concordant	unreportable	unreportable	350228	0		1	SCV000451782	Uncertain significance	Illumina Clinical Se	10/18/2016	IDUA	chr4	996115	996115	http://gnomad.b	0.000297037399	EAS	0.004184099846	0		0	0	https://www.ncbi.nlm.nih.gov/clinvar/variation/350228/
NM_000232.4(SGCB):c.794C>T	concordant	unreportable	unreportable	282121	0		3	SCV000333344;S(	Uncertain significance;Unc	EGL Genetic Diagn	2017-06-30;2017-1	SGCB	chr4	52890286	52890286	http://gnomad.b	0.0001287426323	AFR	0.001176320016	0		0	0	https://www.ncbi.nlm.nih.gov/clinvar/variation/282121/
NM_172250.2(MMAA):c.-35A>T	concordant	unreportable	unreportable	383060	0		1	SCV000523301	Likely benign	GeneDx	2/15/2017	MMAA	chr4	146560257	146560257	http://gnomad.b	0.001063846806	AFR	0.01334299985	2		1	0	https://www.ncbi.nlm.nih.gov/clinvar/variation/383060/
NM_172250.2(MMAA):c.138T>C	concordant	unreportable	unreportable	262806	0		1	SCV000518227	Benign	GeneDx	11/28/2017	MMAA	chr4	146560429	146560429	http://gnomad.b	0.004695323873	AFR	0.03606899828	9		10	0	https://www.ncbi.nlm.nih.gov/clinvar/variation/262806/
NM_172250.2(MMAA):c.494A>G	concordant	unreportable	unreportable	283777	0		1	SCV000336090	Uncertain significance	EGL Genetic Diagn	6/30/2017	MMAA	chr4	146563569	146563569	http://gnomad.b	0.0001679758115	EAS	0.001565400045	0		0	0	https://www.ncbi.nlm.nih.gov/clinvar/variation/283777/
NM_172250.2(MMAA):c.597G>A	concordant	unreportable	unreportable	347600	0		2	SCV000521790;S(	Likely benign;Uncertain sig	GeneDx;Illumina C	2017-11-28;2016-1	MMAA	chr4	146567172	146567172	http://gnomad.b	0.0007518917276	AFR	0.01078149956	3		1	0	https://www.ncbi.nlm.nih.gov/clinvar/variation/347600/
NM_172250.2(MMAA):c.977G>A	concordant	unreportable	unreportable	347605	0		1	SCV000447541	Uncertain significance	Illumina Clinical Se	10/18/2016	MMAA	chr4	146576306	146576306	http://gnomad.b	0.0001039850262	EAS	0.000289921998	0		0	0	https://www.ncbi.nlm.nih.gov/clinvar/variation/347605/
NM_172250.2(MMAA):c.1236A>G	concordant	unreportable	unreportable	382371	0		1	SCV000522285	Likely benign	GeneDx	11/28/2017	MMAA	chr4	146576565	146576565	http://gnomad.b	0.0001679758115	AMR	0.003529839916	0		0	0	https://www.ncbi.nlm.nih.gov/clinvar/variation/382371/
NM_000027.3(AGA):c.*8T>C	concordant	unreportable	unreportable	348227	0		1	SCV000448727	Uncertain significance	Illumina Clinical Se	10/18/2016	AGA	chr4	178352854	178352854	http://gnomad.b	0.0002726992483	AFR	0.008716739714	0		0	0	https://www.ncbi.nlm.nih.gov/clinvar/variation/348227/
NM_000027.3(AGA):c.623-7C>T	concordant	unreportable	unreportable	348233	0		1	SCV000448733	Uncertain significance	Illumina Clinical Se	10/18/2016	AGA	chr4	178357512	178357512		0.0009900168362					1	0	https://www.ncbi.nlm.nih.gov/clinvar/variation/348233/
NM_000027.3(AGA):c.622+13T>C	concordant	unreportable	unreportable	348234	0		1	SCV000448734	Uncertain significance	Illumina Clinical Se	10/18/2016	AGA	chr4	178358546	178358546	http://gnomad.b	0.003826681843	FIN	0.01597129926	16		5	0	https://www.ncbi.nlm.nih.gov/clinvar/variation/348234/
NM_000414.3(HSD17B4):c.281-7A>G	concordant	unreportable	unreportable	500884	0		1	SCV000707027	Benign	EGL Genetic Diagn	6/30/2017	HSD17B4	chr5	118811394	118811394	http://gnomad.b	0.0002056697226	AFR	0.006411930081	0		0	0	https://www.ncbi.nlm.nih.gov/clinvar/variation/500884/
NM_000414.3(HSD17B4):c.715-13C>T	concordant	unreportable	unreportable	226673	0		1	SCV000269168	Benign	Laboratory for Mol	4/26/2016	HSD17B4	chr5	118827782	118827782	http://gnomad.b	0.0002282088703	AFR	0.005228760187	0		1	0	https://www.ncbi.nlm.nih.gov/clinvar/variation/226673/
NM_000414.3(HSD17B4):c.932A>G	concordant	unreportable	unreportable	501525	0		1	SCV000707922	Uncertain significance	EGL Genetic Diagn	6/30/2017	HSD17B4	chr5	118832301	118832301	http://gnomad.b	8.45E-06	NFE	1.79E-05	0		0	0	https://www.ncbi.nlm.nih.gov/clinvar/variation/501525/
NM_000414.3(HSD17B4):c.1767+8T>C	concordant	unreportable	unreportable	195459	0		2	SCV000227621;S(	Benign;Likely benign	EGL Genetic Diagn	2017-06-30;2016-1	HSD17B4	chr5	118862922	118862922	http://gnomad.b	0.001504488108	SAS	0.0432885997	44		13	0	https://www.ncbi.nlm.nih.gov/clinvar/variation/195459/
NM_000414.3(HSD17B4):c.*6A>G	concordant	unreportable	unreportable	226663	0		1	SCV000452156	Uncertain significance	Illumina Clinical Se	10/18/2016	HSD17B4	chr5	118877695	118877695	http://gnomad.b	0.0008395832512	AFR	0.02565779909	7		5	0	https://www.ncbi.nlm.nih.gov/clinvar/variation/226663/
NM_003060.3(SLC22A5):c.394-18C>G	concordant	unreportable	unreportable	385182	0		1	SCV000526362	Likely benign	GeneDx	11/28/2017	SLC22A5	chr5	131714052	131714052	http://gnomad.b	0.0002089899572	AFR	0.0007187660085	0		0	0	https://www.ncbi.nlm.nih.gov/clinvar/variation/385182/
NM_003060.3(SLC22A5):c.572A>G	concordant	unreportable	unreportable	288732	0		1	SCV000342930	Uncertain significance	EGL Genetic Diagn	6/30/2017	SLC22A5	chr5	131719913	131719913	http://gnomad.b	0.0002202867116	EAS	0.002493039938	0		0	0	https://www.ncbi.nlm.nih.gov/clinvar/variation/288732/
NM_003060.3(SLC22A5):c.944C>T	concordant	unreportable	unreportable	460421	0		1	SCV000632580	Uncertain significance	Invitae	10/5/2017	SLC22A5	chr5	131722836	131722836	http://gnomad.b	2.26E-05	EAS	0.0001739330037	0		0	0	https://www.ncbi.nlm.nih.gov/clinvar/variation/460421/
NM_003060.3(SLC22A5):c.1053-3C>T	concordant	unreportable	unreportable	381975	0		1	SCV000521717	Likely benign	GeneDx	11/28/2017	SLC22A5	chr5	131726379	131726379	http://gnomad.b	6.78E-05	AFR	0.0007842109771	0		0	0	https://www.ncbi.nlm.nih.gov/clinvar/variation/381975/
NM_003060.3(SLC22A5):c.1229G>A	concordant	unreportable	unreportable	288728	0		1	SCV000342925	Uncertain significance	EGL Genetic Diagn	6/30/2017	SLC22A5	chr5	131726558	131726558	http://gnomad.b	0.0002259350888	EAS	0.002319650026	0		0	0	https://www.ncbi.nlm.nih.gov/clinvar/variation/288728/
NM_003060.3(SLC22A5):c.1451-16G>A	concordant	unreportable	unreportable	384575	0		1	SCV000525456	Likely benign	GeneDx	11/28/2017	SLC22A5	chr5	131729352	131729352	http://gnomad.b	0.0006128489285	AFR	0.01176320016	1		1	0	https://www.ncbi.nlm.nih.gov/clinvar/variation/384575/
NM_000255.3(MUT):c.1991C>T	concordant	unreportable	unreportable	357254	0		1	SCV000463859	Uncertain significance	Illumina Clinical Se	10/18/2016	MUT	chr6	49403302	49403302	http://gnomad.b	0.0008711776083	ASJ	0.0036607699	2		0	0	https://www.ncbi.nlm.nih.gov/clinvar/variation/357254/
NM_000255.3(MUT):c.1762C>T	concordant	unreportable	unreportable	357256	0		1	SCV000463861	Uncertain significance	Illumina Clinical Se	10/18/2016	MUT	chr6	49409599	49409599	http://gnomad.b	0.0001758340127	AFR	0.00320513011	0		0	0	https://www.ncbi.nlm.nih.gov/clinvar/variation/357256/
NM_000255.3(MUT):c.1629C>T	concordant	unreportable	unreportable	92682	0		2	SCV000463863;S(	Uncertain significance;Beni	Illumina Clinical Se	2016-10-18;2017-1	MUT	chr6	49412399	49412399	http://gnomad.b	0.006625745296	ASJ	0.008125130087	5		6	0	https://www.ncbi.nlm.nih.gov/clinvar/variation/92682/
NM_000255.3(MUT):c.1333-18T>G	concordant	unreportable	unreportable	382974	0		1	SCV000523177	Likely benign	GeneDx	11/28/2017	MUT	chr6	49416658	49416658	http://gnomad.b	0.001846257133	AFR	0.01873940043	3		1	0	https://www.ncbi.nlm.nih.gov/clinvar/variation/382974/
NM_000255.3(MUT):c.1125G>A	concordant	unreportable	unreportable	290321	0		3	SCV000344853;S(	Uncertain significance;Unc	EGL Genetic Diagn	2017-06-30;2016-1	MUT	chr6	49419386	49419386	http://gnomad.b	0.00160648348	NFE	0.002777429996	0		0	0	https://www.ncbi.nlm.nih.gov/clinvar/variation/290321/
NM_000255.3(MUT):c.1115T>C	concordant	unreportable	unreportable	290030	0		3	SCV000344506;S(	Uncertain significance;Unc	EGL Genetic Diagn	2017-06-30;2017-1	MUT	chr6	49419396	49419396	http://gnomad.b	0.0006234114995	ASJ	0.001626349986	0		0	0	https://www.ncbi.nlm.nih.gov/clinvar/variation/290030/
NM_000255.3(MUT):c.1083+20T>C	concordant	unreportable	unreportable	384232	0		1	SCV000524955	Likely benign	GeneDx	2/15/2017	MUT	chr6	49421278	49421278	http://gnomad.b	0.0009111398839	AFR	0.01535580028	4		0	0	https://www.ncbi.nlm.nih.gov/clinvar/variation/384232/
NM_000255.3(MUT):c.711A>G	concordant	unreportable	unreportable	203843	0		2	SCV000700311;S(	Benign;Uncertain significar	EGL Genetic Diagn	2017-06-30;2016-1	MUT	chr6	49425446	49425446	http://gnomad.b	0.012556147	SAS	0.03227720037	37		20	0	https://www.ncbi.nlm.nih.gov/clinvar/variation/203843/
NM_000255.3(MUT):c.461G>A	concordant	unreportable	unreportable	288727	0		2	SCV000342924;S(	Likely benign;Benign	EGL Genetic Diagn	2017-06-30;2017-1	MUT	chr6	49425696	49425696	http://gnomad.b	0.0009111398839	AFR	0.01024209987	2		3	0	https://www.ncbi.nlm.nih.gov/clinvar/variation/288727/
NM_000255.3(MUT):c.393G>A	concordant	unreportable	unreportable	196239	0		2	SCV000520446;S(	Likely benign;Uncertain sig	GeneDx;Illumina C	2017-11-28;2016-1	MUT	chr6	49425764	49425764	http://gnomad.b	0.0004236001215	AFR	0.003200849984	0		0	0	https://www.ncbi.nlm.nih.gov/clinvar/variation/196239/
NM_138694.3(PKHD1):c.11844T>C	concordant	unreportable	unreportable	290952	0		1	SCV000345622	Uncertain significance	EGL Genetic Diagn	6/30/2017	PKHD1	chr6	51484260	51484260	http://gnomad.b	8.34E-06	AMR	0.000119174998	0		0	0	https://www.ncbi.nlm.nih.gov/clinvar/variation/290952/
NM_138694.3(PKHD1):c.11174+11A>C	concordant	unreportable	unreportable	96369	0		1	SCV000114561	Benign	EGL Genetic Diagn	6/30/2017	PKHD1	chr6	51523739	51523739	http://gnomad.b	0.004962247996	AFR	0.02048229985	31		14	0	https://www.ncbi.nlm.nih.gov/clinvar/variation/96369/
NM_138694.3(PKHD1):c.11000G>C	concordant	unreportable	unreportable	357409	0		1	SCV000464031	Uncertain significance	Illumina Clinical Se	10/18/2016	PKHD1	chr6	51523924	51523924	http://gnomad.b	8.34E-06	FIN	8.97E-05	0		0	0	https://www.ncbi.nlm.nih.gov/clinvar/variation/357409/
NM_138694.3(PKHD1):c.9829+10T>G	concordant	unreportable	unreportable	167476	0		2	SCV000291345;S(	Benign;Benign	Invitae;Laboratory	2017-10-05;2018-0	PKHD1	chr6	51612575	51612575	http://gnomad.b	0.0006588530952	AFR	0.0168693997	3		3	0	https://www.ncbi.nlm.nih.gov/clinvar/variation/167476/
NM_138694.3(PKHD1):c.7911+5C>A	concordant	unreportable	unreportable	458606	0		1	SCV000629933	Uncertain significance	Invitae	10/5/2017	PKHD1	chr6	51720686	51720686	http://gnomad.b	1.39E-05	AFR	0.0004573969927	0		0	0	https://www.ncbi.nlm.nih.gov/clinvar/variation/458606/
NM_138694.3(PKHD1):c.7377T>G	concordant	unreportable	unreportable	290859	0		1	SCV000345510	Uncertain significance	EGL Genetic Diagn	6/30/2017	PKHD1	chr6	51735411	51735411	http://gnomad.b	2.50E-05	EAS	0.0004638760001	0		0	0	https://www.ncbi.nlm.nih.gov/clinvar/variation/290859/

Supplemental Table 2

NM_138694.3(PKHD1):c.6795T>C	concordant	unreportable	unreportable	498592	0	1	SCV000703686	Uncertain significance	EGL Genetic Diagn	6/30/2017	PKHD1	chr6	51771026	51771026	http://gnomad.b	4.73E-05	AFR	0.000261609006	0		0	0	https://www.ncbi.nlm.nih.gov/clinvar/variation/498592/
NM_138694.3(PKHD1):c.5752-9G>A	concordant	unreportable	unreportable	498842	0	1	SCV000704057	Uncertain significance	EGL Genetic Diagn	6/30/2017	PKHD1	chr6	51824833	51824833	http://gnomad.b	0.0001195387472	AFR	0.002630189992	0		0	0	https://www.ncbi.nlm.nih.gov/clinvar/variation/498842/
NM_138694.3(PKHD1):c.4540G>C	concordant	unreportable	unreportable	283530	0	1	SCV000335705	Uncertain significance	EGL Genetic Diagn	6/30/2017	PKHD1	chr6	51890068	51890068	http://gnomad.b	3.89E-05	AFR	0.0009156310116	0		0	0	https://www.ncbi.nlm.nih.gov/clinvar/variation/283530/
NM_138694.3(PKHD1):c.4199C>T	concordant	unreportable	unreportable	458597	0	1	SCV000629918	Uncertain significance	Invitae	10/5/2017	PKHD1	chr6	51890409	51890409	http://gnomad.b	2.22E-05	AFR	0.0001960780064	0		0	0	https://www.ncbi.nlm.nih.gov/clinvar/variation/458597/
NM_138694.3(PKHD1):c.4038C>T	concordant	unreportable	unreportable	283528	0	1	SCV000335703	Uncertain significance	EGL Genetic Diagn	6/30/2017	PKHD1	chr6	51890570	51890570	http://gnomad.b	7.23E-05	AFR	0.001177089987	0		0	0	https://www.ncbi.nlm.nih.gov/clinvar/variation/283528/
NM_138694.3(PKHD1):c.3904G>A	concordant	unreportable	unreportable	458595	0	1	SCV000629915	Uncertain significance	Invitae	10/5/2017	PKHD1	chr6	51890704	51890704	http://gnomad.b	2.50E-05	AFR	0.0002626740024	0		0	0	https://www.ncbi.nlm.nih.gov/clinvar/variation/458595/
NM_138694.3(PKHD1):c.2637G>A	concordant	unreportable	unreportable	289979	0	1	SCV000344449	Uncertain significance	EGL Genetic Diagn	6/30/2017	PKHD1	chr6	51909842	51909842	http://gnomad.b	5.56E-05	AFR	0.0004575159983	0		0	0	https://www.ncbi.nlm.nih.gov/clinvar/variation/289979/
NM_138694.3(PKHD1):c.1964+17G>T	concordant	unreportable	unreportable	96382	0	1	SCV000699852	Uncertain significance	Laboratory Corpor	1/25/2018	PKHD1	chr6	51918819	51918819	http://gnomad.b	0.002691011798	ASJ	0.009148200043	6		6	0	https://www.ncbi.nlm.nih.gov/clinvar/variation/96382/
NM_138694.3(PKHD1):c.1960G>A	concordant	unreportable	unreportable	357446	0	1	SCV000464111	Uncertain significance	Illumina Clinical Se	10/18/2016	PKHD1	chr6	51918840	51918840	http://gnomad.b	4.45E-05	EAS	0.0002902589913	0		0	0	https://www.ncbi.nlm.nih.gov/clinvar/variation/357446/
NM_138694.3(PKHD1):c.1234-5C>T	concordant	unreportable	unreportable	241841	0	2	SCV000291326;S(	Benign;Likely benign	Invitae;Laboratory	2017-10-05;2018-0	PKHD1	chr6	51923404	51923404	http://gnomad.b	0.001362185724	AFR	0.03920279816	12		8	0	https://www.ncbi.nlm.nih.gov/clinvar/variation/241841/
NM_183050.2(BCKDHB):c.477+14C>T	concordant	unreportable	unreportable	384205	0	1	SCV000524927	Likely benign	GeneDx	11/28/2017	BCKDHB	chr6	80877542	80877542	http://gnomad.b	0.0001896521677	AFR	0.006111990195	0		0	0	https://www.ncbi.nlm.nih.gov/clinvar/variation/384205/
NM_183050.2(BCKDHB):c.742+7A>T	concordant	unreportable	unreportable	384206	0	2	SCV000524928;S(	Likely benign;Benign	GeneDx;Invitae	2017-11-28;2017-1	BCKDHB	chr6	80881114	80881114	http://gnomad.b	0.0001973407691	AFR	0.006273529958	0		0	0	https://www.ncbi.nlm.nih.gov/clinvar/variation/384206/
NM_183050.2(BCKDHB):c.742+16A>G	concordant	unreportable	unreportable	96606	0	1	SCV000516969	Likely benign	GeneDx	11/28/2017	BCKDHB	chr6	80881123	80881123	http://gnomad.b	0.001086655663	NFE	0.00376779004	5		3	0	https://www.ncbi.nlm.nih.gov/clinvar/variation/96606/
NM_000288.3(PEX7):c.330C>T	concordant	unreportable	unreportable	290908	0	1	SCV000345576	Uncertain significance	EGL Genetic Diagn	6/30/2017	PEX7	chr6	137147598	137147598	http://gnomad.b	0.0001222262964	NFE	0.0003133789869	0		0	0	https://www.ncbi.nlm.nih.gov/clinvar/variation/290908/
NM_000288.3(PEX7):c.418-4G>T	concordant	unreportable	unreportable	355531	0	2	SCV000704014;S(	Uncertain significance;Unc	EGL Genetic Diagn	2017-06-30;2016-1	PEX7	chr6	137167207	137167207	http://gnomad.b	9.17E-05	FIN	0.002513240091	0		0	0	https://www.ncbi.nlm.nih.gov/clinvar/variation/355531/
NM_000466.2(PEX1):c.3810T>A	concordant	unreportable	unreportable	252486	0	1	SCV000296922	Likely benign	Division of Genomi	1/6/2016	PEX1	chr7	92116813	92116813	http://gnomad.b	0.0001469927502	FIN	0.0171341002	6		0	0	https://www.ncbi.nlm.nih.gov/clinvar/variation/252486/
NM_000466.2(PEX1):c.3439-14_3439-	concordant	unreportable	unreportable	256220	0	1	SCV000470496	Likely benign	Illumina Clinical Se	10/18/2016	PEX1	chr7	92119237	92119239	http://gnomad.b	0.01265247031	ASJ	0.04303489998	117		94	0	https://www.ncbi.nlm.nih.gov/clinvar/variation/256220/
NM_000466.2(PEX1):c.3398C>T	concordant	unreportable	unreportable	502339	0	1	SCV000709031	Uncertain significance	EGL Genetic Diagn	6/30/2017	PEX1	chr7	92120626	92120626	http://gnomad.b	5.55E-06	AMR	0.0002085440065	0		0	0	https://www.ncbi.nlm.nih.gov/clinvar/variation/502339/
NM_000466.2(PEX1):c.2060G>A	concordant	unreportable	unreportable	498170	0	1	SCV000703050	Uncertain significance	EGL Genetic Diagn	6/30/2017	PEX1	chr7	92134057	92134057	http://gnomad.b	3.05E-05	AFR	0.0002614039986	0		0	0	https://www.ncbi.nlm.nih.gov/clinvar/variation/498170/
NM_000466.2(PEX1):c.2045C>T	concordant	unreportable	unreportable	497946	0	1	SCV000702723	Uncertain significance	EGL Genetic Diagn	6/30/2017	PEX1	chr7	92134072	92134072	http://gnomad.b	9.98E-05	AFR	0.0008495619986	0		0	0	https://www.ncbi.nlm.nih.gov/clinvar/variation/497946/
NM_000466.2(PEX1):c.1671-7T>C	concordant	unreportable	unreportable	256218	0	1	SCV000470517	Uncertain significance	Illumina Clinical Se	10/18/2016	PEX1	chr7	92136447	92136447	http://gnomad.b	0.005305606248	ASJ	0.01961380057	23		17	0	https://www.ncbi.nlm.nih.gov/clinvar/variation/256218/
NM_000441.1(SLC26A4):c.236G>A	concordant	unreportable	unreportable	502106	0	1	SCV000708714	Uncertain significance	EGL Genetic Diagn	6/30/2017	SLC26A4	chr7	107303812	107303812	http://gnomad.b	1.95E-05	EAS	0.0003478660074	0		0	0	https://www.ncbi.nlm.nih.gov/clinvar/variation/502106/
NM_000441.1(SLC26A4):c.1608C>T	concordant	unreportable	unreportable	227950	0	1	SCV000270850	Likely benign	Laboratory for Mol	4/26/2016	SLC26A4	chr7	107338550	107338550	http://gnomad.b	2.51E-05	NFE	4.49E-05	0		0	0	https://www.ncbi.nlm.nih.gov/clinvar/variation/227950/
NM_000441.1(SLC26A4):c.2008G>A	concordant	unreportable	unreportable	358501	0	1	SCV000466104	Uncertain significance	Illumina Clinical Se	10/18/2016	SLC26A4	chr7	107342476	107342476	http://gnomad.b	8.10E-05	EAS	0.0004059849889	0		0	0	https://www.ncbi.nlm.nih.gov/clinvar/variation/358501/
NM_000441.1(SLC26A4):c.*69C>A	concordant	unreportable	unreportable	358505	0	1	SCV000466113	Uncertain significance	Illumina Clinical Se	10/18/2016	SLC26A4	chr7	107355960	107355960		0.0006421746827					3	0	https://www.ncbi.nlm.nih.gov/clinvar/variation/358505/
NM_000492.3(CFTR):c.164+28A>G	concordant	unreportable	unreportable	235283	0	2	SCV000700265;S(	Benign;Benign	EGL Genetic Diagn	2017-06-30;2016-0	CFTR	chr7	117144445	117144445	http://gnomad.b	0.0008030720878	AFR	0.01986050047	4		1	0	https://www.ncbi.nlm.nih.gov/clinvar/variation/235283/
NM_000492.3(CFTR):c.1393-42G>A	concordant	unreportable	unreportable	237854	0	2	SCV000705742;S(	Benign;Benign	EGL Genetic Diagn	2017-06-30;2016-0	CFTR	chr7	117199476	117199476	http://gnomad.b	0.001022616975	AMR	0.04110730067	39		17	0	https://www.ncbi.nlm.nih.gov/clinvar/variation/237854/
NM_000492.3(CFTR):c.1584+77A>G	concordant	unreportable	unreportable	439057	0	1	SCV000601051	Likely benign	Quest Diagnostics I	8/1/2017	CFTR	chr7	117199786	117199786		0.001194016054					18	0	https://www.ncbi.nlm.nih.gov/clinvar/variation/439057/
NM_000492.3(CFTR):c.1766+86C>A	concordant	unreportable	unreportable	439061	0	1	SCV000601060	Uncertain significance	Quest Diagnostics I	8/1/2017	CFTR	chr7	117230579	117230579		0.0002099157256					0	0	https://www.ncbi.nlm.nih.gov/clinvar/variation/439061/
NM_000492.3(CFTR):c.2042A>T	concordant	unreportable	unreportable	287205	0	1	SCV000340908	Uncertain significance	EGL Genetic Diagn	6/30/2017	CFTR	chr7	117232263	117232263	http://gnomad.b	0.0002484323725	EAS	0.001801279956	0		2	0	https://www.ncbi.nlm.nih.gov/clinvar/variation/287205/
NM_000492.3(CFTR):c.2878C>T	concordant	unreportable	unreportable	495917	0	1	SCV000696931	Uncertain significance	Laboratory Corpor	1/25/2018	CFTR	chr7	117243806	117243806	http://gnomad.b	5.78E-06	AMR	0.0001491290022	0		0	0	https://www.ncbi.nlm.nih.gov/clinvar/variation/495917/
NM_000492.3(CFTR):c.2988+20G>A	concordant	unreportable	unreportable	495922	0	1	SCV000696942	Uncertain significance	Laboratory Corpor	1/25/2018	CFTR	chr7	117246827	117246827	http://gnomad.b	1.16E-05	EAS	0.000173952998	0		0	0	https://www.ncbi.nlm.nih.gov/clinvar/variation/495922/
NM_000492.3(CFTR):c.3469-17T>C	concordant	unreportable	unreportable	53751	0	4	SCV000603031;S(	Likely benign;Uncertain sig	ARUP Laboratories	2017-06-30;2017-0	CFTR	chr7	117267559	117267559	http://gnomad.b	0.0002349515461	SAS	0.003223920008	1		2	0	https://www.ncbi.nlm.nih.gov/clinvar/variation/53751/
NM_000492.3(CFTR):c.3717+45G>A	concordant	unreportable	unreportable	53790	0	1	SCV000709661	Benign	EGL Genetic Diagn	6/30/2017	CFTR	chr7	117267869	117267869	http://gnomad.b	0.0005103455714	EAS	0.01538639981	0		2	0	https://www.ncbi.nlm.nih.gov/clinvar/variation/53790/
NM_000492.3(CFTR):c.3759G>A	concordant	unreportable	unreportable	215752	0	2	SCV000253286;S(	Likely benign;Uncertain sig	Invitae;Laboratory	2015-09-11;2018-0	CFTR	chr7	117282533	117282533	http://gnomad.b	2.12E-05	NFE	4.49E-05	0		0	0	https://www.ncbi.nlm.nih.gov/clinvar/variation/215752/
NM_000492.3(CFTR):c.3983T>C	concordant	unreportable	unreportable	439080	0	2	SCV000700500;S(	Uncertain significance;Unc	EGL Genetic Diagn	2017-06-30;2017-0	CFTR	chr7	117304761	117304761	http://gnomad.b	2.50E-05	SAS	6.50E-05	0		0	0	https://www.ncbi.nlm.nih.gov/clinvar/variation/439080/
NM_000492.3(CFTR):c.4121C>G	concordant	unreportable	unreportable	496886	0	1	SCV000700501	Uncertain significance	EGL Genetic Diagn	6/30/2017	CFTR	chr7	117304899	117304899		1.93E-06					0	0	https://www.ncbi.nlm.nih.gov/clinvar/variation/496886/
NM_000492.3(CFTR):c.4197C>T	concordant	unreportable	unreportable	496897	0	1	SCV000700532	Uncertain significance	EGL Genetic Diagn	6/30/2017	CFTR	chr7	117305573	117305573		7.70E-06					0	0	https://www.ncbi.nlm.nih.gov/clinvar/variation/496897/
NM_000492.3(CFTR):c.4242+10T>C	concordant	unreportable	unreportable	53928	0	2	SCV000075126;S(	Benign;Uncertain significar	Invitae;Laboratory	2017-10-05;2018-0	CFTR	chr7	117305628	117305628	http://gnomad.b	0.000161769917	AFR	0.004515119828	0		0	0	https://www.ncbi.nlm.nih.gov/clinvar/variation/53928/
NM_000492.3(CFTR):c.4242+13A>G	concordant	unreportable	unreportable	93155	0	4	SCV000603039;S(	Benign;Benign;Benign;Likel	ARUP Laboratories	2017-06-30;2017-1	CFTR	chr7	117305631	117305631	http://gnomad.b	0.00160999584	ASJ	0.01579380035	5		2	0	https://www.ncbi.nlm.nih.gov/clinvar/variation/93155/
NM_000492.3(CFTR):c.4243-20A>G	concordant	unreportable	unreportable	439084	0	3	SCV000703817;S(	Uncertain significance;Unc	EGL Genetic Diagn	2017-06-30;2018-0	CFTR	chr7	117306942	117306942	http://gnomad.b	0.0001078466113	AFR	0.002880339976	0		0	0	https://www.ncbi.nlm.nih.gov/clinvar/variation/439084/
NM_000492.3(CFTR):c.4243-5C>T	concordant	unreportable	unreportable	256255	0	4	SCV000603014;S(	Likely benign;Benign;Benig	ARUP Laboratories	2017-06-30;2017-0	CFTR	chr7	117306957	117306957	http://gnomad.b	0.0002099157256	AFR	0.006147000007	0		0	0	https://www.ncbi.nlm.nih.gov/clinvar/variation/256255/
NM_018941.3(CLN8):c.126C>T	concordant	unreportable	unreportable	241287	0	1	SCV000290463	Likely benign	Invitae	6/10/2016	CLN8	chr8	1719346	1719346	http://gnomad.b	1.68E-05	EAS	0.0002319110063	0		0	0	https://www.ncbi.nlm.nih.gov/clinvar/variation/241287/
NM_018941.3(CLN8):c.406G>T	concordant	unreportable	unreportable	205208	0	1	SCV000628990	Uncertain significance	Invitae	10/5/2017	CLN8	chr8	1719626	1719626	http://gnomad.b	3.36E-05	EAS	5.80E-05	0		0	0	https://www.ncbi.nlm.nih.gov/clinvar/variation/205208/
NM_018941.3(CLN8):c.831C>T	concordant	unreportable	unreportable	387535	0	1	SCV000529611	Likely benign	GeneDx	11/28/2017	CLN8	chr8	1728703	1728703	http://gnomad.b	2.24E-05	AMR	0.000119174998	0		0	0	https://www.ncbi.nlm.nih.gov/clinvar/variation/387535/
NM_000370.3(TTPA):c.664-4T>G	concordant	unreportable	unreportable	363557	0	1	SCV000474653	Likely benign	Illumina Clinical Se	10/18/2016	TTPA	chr8	63973988	63973988	http://gnomad.b	0.00166335397	AMR	0.05524099991	58		8	0	https://www.ncbi.nlm.nih.gov/clinvar/variation/363557/
NM_002485.4(NBN):c.2235-18C>T	concordant	unreportable	unreportable	182710	0	1	SCV000685763	Likely benign	Color Genomics, In	10/26/2017	NBN	chr8	90947858	90947858	http://gnomad.b	6.69E-05	EAS	0.001741150045	0		1	0	https://www.ncbi.nlm.nih.gov/clinvar/variation/182710/
NM_002485.4(NBN):c.2060A>C	concordant	unreportable	unreportable	490052	0	1	SCV000685749	Uncertain significance	Color Genomics, In	10/26/2017	NBN	chr8	90958378	90958378	http://gnomad.b	2.23E-05	FIN	9.07E-05	0		0	0	https://www.ncbi.nlm.nih.gov/clinvar/variation/490052/
NM_002485.4(NBN):c.1979G>C	concordant	unreportable	unreportable	216567	0	3	SCV000685746;S(	Uncertain significance;Unc	Color Genomics, In	2017-10-26;2017-1	NBN	chr8	90958459	90958459	http://gnomad.b	2.23E-05	AFR	0.0003922590113	0		0	0	https://www.ncbi.nlm.nih.gov/clinvar/variation/216567/
NM_002485.4(NBN):c.1398-19C>T	concordant	unreportable	unreportable	182707	0	1	SCV000685716	Benign	Color Genomics, In	10/26/2017	NBN	chr8	90965938	90965938		4.46E-05					0	0	https://www.ncbi.nlm.nih.gov/clinvar/variation/182707/
NM_002485.4(NBN):c.1194A>C	concordant	unreportable	unreportable	490044	0	1	SCV000685698	Uncertain significance	Color Genomics, In	10/26/2017	NBN	chr8	90967714	90967714	http://gnomad.b	5.58E-06	NFE	1.79E-05	0		0	0	https://www.ncbi.nlm.nih.gov/clinvar/variation/490044/
NM_002485.4(NBN):c.1124+20G>A	concordant	unreportable	unreportable	379516	0	1	SCV000685693	Likely benign	Color Genomics, In	10/26/2017	NBN	chr8	90970933	90970933	http://gnomad.b	8.37E-06	EAS	0.0001739940053	0		0	0	https://www.ncbi.nlm.nih.gov/clinvar/variation/379516/
NM_002485.4(NBN):c.1124+19C>T	concordant	unreportable	unreportable	492079	0	1	SCV000690638	Benign	Color Genomics, In	12/21/2017	NBN	chr8	90970934	90970934	http://gnomad.b	0.0009174567764	AFR	0.0299346	9		3	0	https://www.ncbi.nlm.nih.gov/clinvar/variation/492079/
NM_002485.4(NBN):c.896+4T>C	concordant	unreportable	unreportable	231622	0	1	SCV000634338	Likely benign	Invitae	10/5/2017	NBN	chr8	90982588	90982588	http://gnomad.b	1.12E-05	FIN	0.006439330056	1		0	0	https://www.ncbi.nlm.nih.gov/clinvar/variation/231622/
NM_002485.4(NBN):c.702+16G>C	concordant	unreportable	unreportable	234384	0	2	SCV000537474;S(	Likely benign;Likely benign	Color Genomics, In	2017-01-26;2017-1	NBN	chr8	90983385	90983385	http://gnomad.b	6.97E-05	EAS	0.00267816009	0		0	0	https://www.ncbi.nlm.nih.gov/clinvar/variation/234384/
NM_002485.4(NBN):c.302T>C	concordant	unreportable	unreportable	185055	0	2	SCV000292180;S(	Uncertain significance;Unc	Color Genomics, In	2017-12-21;2017-1	NBN	chr8	90993621	90993621	http://gnomad.b	2.51E-05	FIN	4.49E-05	0		0	0	https://www.ncbi.nlm.nih.gov/clinvar/variation/185055/
NM_002485.4(NBN):c.37+11A>G	concordant	unreportable	unreportable	490056	0	1	SCV000685777	Benign	Color Genomics, In	10/26/2017	NBN	chr8	90996742	90996742	http://gnomad.b	0.0005493586168	AFR	0.01647759974	2		0	0	https://www.ncbi.nlm.nih.gov/clinvar/variation/490056/
NM_002485.4(NBN):c.37+5G>A	concordant	unreportable	unreportable	140918	0	4	SCV000685778;S(	Benign;Benign;Benign;Likel	Color Genomics, In	2017-10-26;2017-0	NBN	chr8	90996748	90996748	http://gnomad.b	0.001029001673	AFR	0.02326210029	9		4	0	https://www.ncbi.nlm.nih.gov/clinvar/variation/140918/
NM_002485.4(NBN):c.-2C>T	concordant	unreportable	unreportable	142094	0	2	SCV000685686;S(	Likely benign;Likely benign	Color Genomics, In	2017-10-26;2017-1	NBN	chr8	90996791	90996791	http://gnomad.b	0.0001673173452	EAS	0.00245356001	0		0	0	https://www.ncbi.nlm.nih.gov/clinvar/variation/142094/
NM_002485.4(NBN):c.-26G>A	concordant	unreportable	unreportable	182712	0	1	SCV000475304	Uncertain significance	Illumina Clinical Se	10/18/2016	NBN	chr8	90996815	90996815	http://gnomad.b	0.0001115448968	AMR	0.0002100340062	0		0	0	https://www.ncbi.nlm.nih.gov/clinvar/variation/182712/
NM_017890.4(VPS13B):c.2825-4T>A	concordant	unreportable	unreportable	285019	0	1	SCV000337855	Uncertain significance	EGL Genetic Diagn	6/30/2017	VPS13B	chr8	100396432	100396432	http://gnomad.b	7.00E-05	EAS	0.002089140005	0		1	0	https://www.ncbi.nlm.nih.gov/clinvar/variation/285019/
NM_017890.4(VPS13B):c.2934+14A>T	concordant	unreportable	unreportable	361052	0	1	SCV000470786	Likely benign	Illumina Clinical Se	10/18/2016	VPS13B	chr8	100396559	100396559	http://gnomad.b	0.003692712562	SAS	0.03656709939	43		13	0	https://www.ncbi.nlm.nih.gov/clinvar/variation/361052/
NM_017890.4(VPS13B):c.3211-11T>C	concordant	unreportable	unreportable	361054	0	1	SCV000470790	Uncertain significance	Illumina Clinical Se	10/18/2016	VPS13B	chr8	100454618	100454618	http://gnomad.b	0.0006103194378	EAS	0.01796549931	2		5	0	https://www.ncbi.nlm.nih.gov/clinvar/variation/361054/
NM_017890.4(VPS13B):c.4158-18C>T	concordant	unreportable	unreportable	379221	0	1	SCV000515897	Likely benign	GeneDx	11/28/2017	VPS13B	chr8	100519980	100519980	http://gnomad.b	0.0008118928301	ASJ	0.005686429795	4		0	0	https://www.ncbi.nlm.nih.gov/clinvar/variation/379221/
NM_017890.4(VPS13B):c.4300-6G>A	concordant	unreportable	unreportable	286663	0	2	SCV000340192;S(	Uncertain significance;Unc	EGL Genetic Diagn	2017-06-30;2016-1	VPS13B	chr8	100523326	100523326	http://gnomad.b	0.0001035863266	NFE	0.0004430940025	0		0	0	https://www.ncbi.nlm.nih.gov/clinvar/variation/286663/
NM_017890.4(VPS13B):c.7876G>A	concordant	unreportable	unreportable	286894	0	1	SCV000340483	Likely benign	EGL Genetic Diagn	6/30/2017	VPS13B	chr8	100796564	100796564	http://gnomad.b	0.000492734959	EAS	0.00475748023	0		0	0	https://www.ncbi.nlm.nih.gov/clinvar/variation/286894/
NM_017890.4(VPS13B):c.8868-12T>A	concordant	unreportable	unreportable	424235	0	1	SCV000574024	Uncertain significance	GeneDx	11/28/2017	VPS13B	chr8	100832137	100832137	http://gnomad.b	5.88E-05	AFR	0.001437720028	0		0	0	https://www.ncbi.nlm.nih.gov/clinvar/variation/424235/
NM_017890.4(VPS13B):c.9405+9A>G	concordant	unreportable	unreportable	130729	0	2	SCV000332962;S(	Benign;Likely benign	EGL Genetic Diagn	2017-06-30;2017-1	VPS13B	chr8	100836215	100836215	http://gnomad.b	0.000330356393	AFR	0.009363540448	0		0	0	https://www.ncbi.nlm.nih.gov/clinvar/variation/130729/
NM_017890.4(VPS13B):c.9405+10T>A	concordant	unreportable	unreportable	130728	0	3	SCV000616279;S(	Benign;Likely benign;Benig	Athena Diagnostics	2017-08-17;2014-0	VPS13B	chr8	100836216	100836216	http://gnomad.b	0.006310367032	NFE	0.02418600023	44		29	0	https://www.ncbi.nlm.nih.gov/clinvar/variation/130728/
NM_017890.4(VPS13B):c.10315T>C	concordant	unreportable	unreportable	197995	0	2	SCV000231390;S(	Uncertain significance;Likel	EGL Genetic Diagn	2017-06-30;2017-0	VPS13B	chr8	100865857	100865857	http://gnomad.b	0.0001819759792	AFR	0.002025610069	0		0	0	https://www.ncbi.nlm.nih.gov/clinvar/variation/197995/
NM_017890.4(VPS13B):c.11119+13G>	concordant	unreportable	unreportable	361094	0	1	SCV000470858	Likely benign	Illumina Clinical Se	10/18/2016	VPS13B	chr8	100871721	100871721	http://gnomad.b	0.001052661049	AFR	0.03380690143	12		1	0	https://www.ncbi.nlm.nih.gov/clinvar/variation/361094/
NM_017890.4(VPS13B):c.11570+6A>G	concordant	unreportable	unreportable	437245	0	1	SCV000597902	Uncertain significance	Genetic Services La	7/5/2017	VPS13B	chr8	100883121	100883121	http://gnomad.b	5.88E-05	AFR	0.002419879893	0		0	0	https://www.ncbi.nlm.nih.gov/clinvar/variation/437245/
NM_000170.2(GLDC):c.2988G>C	concordant	unreportable	unreportable	367171	0	1	SCV000480489	Uncertain significance	Illumina Clinical Se	10/18/2016	GLDC	chr9	6533092	6533092	http://gnomad.b	0.0003681354739	FIN	0.0107175	0		0	0	https://www.ncbi.nlm.nih.gov/clinvar/variation/367171/
NM_000170.2(GLDC):c.2964G>A	concordant	unreportable	unreportable	462883	0	1	SCV000636402	Likely benign	Invitae	10/5/2017	GLDC	chr9	6533116	6533116	http://gnomad.b	0.001160427037	SAS	0.002339029917	0		0	0	https://www.ncbi.nlm.nih.gov/clinvar/variation/462883/
NM_000170.2(GLDC):c.2919+3A>G	concordant	unreportable	unreportable	367173	0	3	SCV000613529;S(	Benign;Uncertain significar	Athena Diagnostics	2017-08-17;2016-1	GLDC	chr9	6534705	6534705	http://gnomad.b	0.001728636138	AFR	0.01947879978	3		4	0	https://www.ncbi.nlm.nih.gov/clinvar/variation/367173/
NM_000170.2(GLDC):c.2919C>T	concordant	unreportable	unreportable	367174	0	1	SCV000480492	Uncertain significance	Illumina Clinical Se	10/18/2016	GLDC	chr9	6534708	6534708	http://gnomad.b	4.80E-05	AMR	0.000119352997	0		0	0	https://www.ncbi.nlm.nih.gov/clinvar/variation/367174/
NM_000170.2(GLDC):c.2730G>A	concordant	unreportable	unreportable	367176	0	2	SCV000480494;S(	Uncertain significance;Likel	Illumina Clinical Se	2016-10-18;2017-1	GLDC	chr9	6536172	6536172	http://gnomad.b	0.0008723210141	NFE	0.001158279949	0		0	0	https://www.ncbi.nlm.nih.gov/clinvar/variation/367176/
NM_000170.2(GLDC):c.2683A>G	concordant	unreportable	unreportable	367177	0	2	SCV000480495;S(	Uncertain significance;Likel	Illumina Clinical Se	2016-10-18;2017-1	GLDC	chr9	6536219	6536219	http://gnomad.b	0.001088400531	AFR	0.004136569798	2		1	0	https://www.ncbi.nlm.nih.gov/clinvar/variation/367177/
NM_000170.2(GLDC):c.2457+8C>T	concordant	unreportable	unreportable	462872	0	1	SCV000636386	Benign	Invitae	10/5/2017	GLDC	chr9	6553360	6553360	http://gnomad.b	0.0009043327945	AFR	0.01228759997	2		0	0	https://www.ncbi.nlm.nih.gov/clinvar/variation/462872/
NM_000170.2(GLDC):c.2316-6dupA	concordant	unreportable	unreportable	255454	0	1	SCV000480500	Benign	Illumina Clinical Se	10/18/2016	GLDC	chr9	6553514	6553514		8.00E-06					0	0	https://www.ncbi.nlm.nih.gov/clinvar/variation/255454/
NM_000170.2(GLDC):c.2203G>T	concordant	unreportable	unreportable	367181	0	2	SCV000480501;S(	Likely benign;Benign	Illumina Clinical Se	2016-10-18;2017-1	GLDC	chr9	6554781	6554781	http://gnomad.b	0.003185172143	FIN	0.03801650181	24		1	0	https://www.ncbi.nlm.nih.gov/clinvar/variation/367181/
NM_000170.2(GLDC):c.2149A>G	concordant	unreportable	unreportable	367182	0	1	SCV000480502	Uncertain significance	Illumina Clinical Se	10/18/2016	GLDC	chr9	6556206	6556206	http://gnomad.b	0.0001200441763	NFE	0.0001253469964	0		0	0	https://www.ncbi.nlm.nih.gov/clinvar/variation/367182/
NM_000170.2(GLDC):c.2053-5C>G	concordant	unreportable	unreportable	235561	0	1	SCV000636373	Benign	Invitae	10/5/2017	GLDC	chr9	6556307	6556307	http://gnomad.b	0.002777021944	NFE	0.003635840025	3		2	0	https://www.ncbi.nlm.nih.gov/clinvar/variation/235561/
NM_000170.2(GLDC):c.1940C>T	concordant	unreportable	unreportable	254211	0	1	SCV000480504	Uncertain significance	Illumina Clinical Se	10/18/2016	GLDC	chr9	6558671	6558671	http://gnomad.b	5.60E-05	AMR	0.0001488889975	0		0	0	https://www.ncbi.nlm.nih.gov/clinvar/variation/254211/
NM_000170.2(GLDC):c.1927-9A>G	concordant	unreportable	unreportable	367184	0	2	SCV000480505;S(	Uncertain significance;Beni	Illumina Clinical Se	2016-10-18;2017-1	GLDC	chr9	6558693	6558693	http://gnomad.b	0.002040750996	NFE	0.003098970046	2		1	0	https://www.ncbi.nlm.nih.gov/clinvar/variation/367184/
NM_000170.2(GLDC):c.1815C>T	concordant	unreportable	unreportable	367185	0	2	SCV000480506;S(	Likely benign;Benign	Illumina Clinical Se	2016-10-18;2017-1	GLDC	chr9	6587176	6587176	http://gnomad.b	0.003721369464	AFR	0.04292999953	15		7	0	https://www.ncbi.nlm.nih.gov/clinvar/variation/367185/
NM_000170.2(GLDC):c.1707+8G>A	concordant	unreportable	unreportable	367186	0	2	SCV000480507;S(	Uncertain significance;Beni	Illumina Clinical Se	2016-10-18;2017-1	GLDC	chr9	6588393	6588393	http://gnomad.b	0.00478576116	NFE	0.006894949824	6		1	0	https://www.ncbi.nlm.nih.gov/clinvar/variation/367186/
NM_000170.2(GLDC):c.1618A>C	concordant	unreportable	unreportable	462857	0	1	SCV000636361	Likely benign	Invitae	10/5/2017	GLDC	chr9	6588665	6588665	http://gnomad.b	0.0004241560894	AFR	0.004525179975	0		0	0	https://www.ncbi.nlm.nih.gov/clinvar/variation/462857/
NM_000170.2(GLDC):c.1545G>A	concordant	unreportable	unreportable	255453	0	2	SCV000613526;S(	Benign;Benign	Athena Diagnostics	2017-08-17;2017-1	GLDC	chr9	6589230	6589230	http://gnomad.b	0.02392080286	ASJ	0.03258879855	90		38	0	https://www.ncbi.nlm.nih.gov/clinvar/variation/255453/
NM_000170.2(GLDC):c.1331G>A	concordant	unreportable	unreportable	462852	0	1	SCV000636352	Likely benign	Invitae	10/5/2017	GLDC	chr9	6592921	6592921	http://gnomad.b	0.0006402356067	AFR	0.008625200018	1		0	0	https://www.ncbi.nlm.nih.gov/clinvar/variation/462852/
NM_000170.2(GLDC):c.1156-7C>G	concordant	unreportable	unreportable	367195	0	2	SCV000480518;S(	Uncertain significance;Beni	Illumina Clinical Se	2016-10-18;2017-1	GLDC	chr9	6595126	6595126	http://gnomad.b	0.00141652128	FIN	0.00903995987	2		0	0	https://www.ncbi.nlm.nih.gov/clinvar/variation/367195/
NM_000170.2(GLDC):c.936C>T	concordant	unreportable	unreportable	462895	0	1	SCV000636422	Benign	Invitae	10/5/2017	GLDC	chr9	6604710	6604710	http://gnomad.b	0.001472541895	EAS	0.01136760041	2		0	0	https://www.ncbi.nlm.nih.gov/clinvar/variation/462895/
NM_000170.2(GLDC):c.871T>G	concordant	unreportable	unreportable	367197	0	2	SCV000480520;S(	Uncertain significance;Beni	Illumina Clinical Se	2016-10-18;2017-1	GLDC	chr9	6604775	6604775	http://gnomad.b	0.001688621413	AFR	0.02009950019	7		1	0	https://www.ncbi.nlm.nih.gov/clinvar/variation/367197/
NM_000170.2(GLDC):c.678C>T	concordant	unreportable	unreportable	462892	0	1	SCV000636418	Benign	Invitae	10/5/2017	GLDC	chr9	6606627	6606627	http://gnomad.b	0.001320485939	AFR	0.01025880035	2		1	0	https://www.ncbi.nlm.nih.gov/clinvar/variation/462892/
NM_000170.2(GLDC):c.646A>G	concordant	unreportable	unreportable	462891	0	1	SCV000636414	Uncertain significance	Invitae	10/5/2017	GLDC	chr9	6606659	6606659	http://gnomad.b	5.60E-05	AMR	0.0001488889975	0		0	0	https://www.ncbi.nlm.nih.gov/clinvar/variation/462891/
NM_000170.2(GLDC):c.619C>G	concordant	unreportable	unreportable	462888	0	1	SCV000636411	Uncertain significance	Invitae	10/5/2017	GLDC	chr9	6610208	6610208	http://gnomad.b	0.0004001472542	AFR	0.0009309750167	0		0	0	https://www.ncbi.nlm.nih.gov/clinvar/variation/462888/
NM_000170.2(GLDC):c.470+11T>C	concordant	unreportable	unreportable	367203	0	1	SCV000480527	Likely benign	Illumina Clinical Se	10/18/2016	GLDC	chr9	6620173	6620173	http://gnomad.b	0.001656609632	EAS	0.02916279994	9		2	0	https://www.ncbi.nlm.nih.gov/clinvar/variation/367203/

Supplemental Table 2

NM_000170.2(GLDC):c.222C>T	concordant	unreportable	unreportable	462866	0		1	SCV000636377	Benign	Invitae	10/5/2017	GLDC	chr9	6645278	6645278	http://gnomad.b	0.0002801030779	AFR	0.004205469973	0		0	0	https://www.ncbi.nlm.nih.gov/clinvar/variation/462866/
NM_000170.2(GLDC):c.40C>A	concordant	unreportable	unreportable	462885	0		1	SCV000636405	Likely benign	Invitae	10/5/2017	GLDC	chr9	6645460	6645460		0.0005361973206					0	0	https://www.ncbi.nlm.nih.gov/clinvar/variation/462885/
NM_000155.3(GALT):c.268G>C	concordant	unreportable	unreportable	495675	0		1	SCV000695686	Uncertain significance	Laboratory Corpora	1/25/2018	GALT	chr9	34647504	34647504	http://gnomad.b	2.75E-06	AFR	0.0002613700053	0		0	0	https://www.ncbi.nlm.nih.gov/clinvar/variation/495675/
NM_000155.3(GALT):c.687+9G>C	concordant	unreportable	unreportable	366698	0		2	SCV000479764;S(	Uncertain significance;Unc	Illumina Clinical Se	2016-10-18;2018-0	GALT	chr9	34648462	34648462	http://gnomad.b	0.0003902878785	ASJ	0.002030459931	1		0	0	https://www.ncbi.nlm.nih.gov/clinvar/variation/366698/
NM_001128227.2(GNE):c.2206G>A	concordant	unreportable	unreportable	288495	0		1	SCV000342611	Uncertain significance	EGL Genetic Diagn	6/30/2017	GNE	chr9	36217418	36217418	http://gnomad.b	5.60E-06	FIN	0.000134625996	0		0	0	https://www.ncbi.nlm.nih.gov/clinvar/variation/288495/
NM_005476.5(GNE):c.1908G>A	concordant	unreportable	unreportable	284448	0		2	SCV000337079;S(	Uncertain significance;Likel	EGL Genetic Diagn	2017-06-30;2017-1	GNE	chr9	36218205	36218205	http://gnomad.b	4.20E-05	AMR	0.0004466680111	0		0	0	https://www.ncbi.nlm.nih.gov/clinvar/variation/284448/
NM_001128227.2(GNE):c.1727-7G>A	concordant	unreportable	unreportable	286152	0		1	SCV000339475	Uncertain significance	EGL Genetic Diagn	6/30/2017	GNE	chr9	36220024	36220024	http://gnomad.b	6.72E-05	NFE	0.0001432510035	0		0	0	https://www.ncbi.nlm.nih.gov/clinvar/variation/286152/
NM_001128227.2(GNE):c.1505-4G>A	concordant	unreportable	unreportable	500345	0		1	SCV000706243	Uncertain significance	EGL Genetic Diagn	6/30/2017	GNE	chr9	36222999	36222999	http://gnomad.b	7.84E-05	EAS	0.0008697669837	0		0	0	https://www.ncbi.nlm.nih.gov/clinvar/variation/500345/
NM_001128227.2(GNE):c.1163+7A>G	concordant	unreportable	unreportable	289054	0		1	SCV000343328	Uncertain significance	EGL Genetic Diagn	6/30/2017	GNE	chr9	36229011	36229011	http://gnomad.b	1.96E-05	EAS	0.0009276440251	0		0	0	https://www.ncbi.nlm.nih.gov/clinvar/variation/289054/
NM_000136.2(FANCC):c.*5C>T	concordant	unreportable	unreportable	137286	0		1	SCV000260659	Benign	Invitae	1/6/2016	FANCC	chr9	97863984	97863984	http://gnomad.b	0.0004304379052	EAS	0.01166430023	2		2	0	https://www.ncbi.nlm.nih.gov/clinvar/variation/137286/
NM_000136.2(FANCC):c.1603C>T	concordant	unreportable	unreportable	220016	0		1	SCV000260240	Uncertain significance	Invitae	1/6/2016	FANCC	chr9	97864063	97864063	http://gnomad.b	3.80E-05	AFR	0.0001961039961	0		0	0	https://www.ncbi.nlm.nih.gov/clinvar/variation/220016/
NM_000136.2(FANCC):c.1073-4G>A	concordant	unreportable	unreportable	237063	0		1	SCV000283580	Likely benign	Invitae	10/5/2017	FANCC	chr9	97876996	97876996	http://gnomad.b	9.51E-06	EAS	0.0003478660074	0		0	0	https://www.ncbi.nlm.nih.gov/clinvar/variation/237063/
NM_000136.2(FANCC):c.896+6C>T	concordant	unreportable	unreportable	377846	0		2	SCV000512970;S(	Likely benign;Uncertain sig	GeneDx;Invitae	2017-11-28;2017-1	FANCC	chr9	97888805	97888805	http://gnomad.b	9.51E-06	AFR	0.0001960530062	0		0	0	https://www.ncbi.nlm.nih.gov/clinvar/variation/377846/
NM_000136.2(FANCC):c.843+4C>T	concordant	unreportable	unreportable	220920	0		3	SCV000481131;S(	Likely benign;Benign;Benig	Illumina Clinical Se	2016-10-18;2017-1	FANCC	chr9	97897624	97897624	http://gnomad.b	0.001690836191	AFR	0.0460492	16		24	0	https://www.ncbi.nlm.nih.gov/clinvar/variation/220920/
NM_000136.2(FANCC):c.542C>T	concordant	unreportable	unreportable	127543	0		1	SCV000260329	Uncertain significance	Invitae	10/5/2017	FANCC	chr9	97912349	97912349	http://gnomad.b	3.57E-05	SAS	3.25E-05	0		0	0	https://www.ncbi.nlm.nih.gov/clinvar/variation/127543/
NM_000136.2(FANCC):c.457-12T>A	concordant	unreportable	unreportable	381363	0		1	SCV000520576	Likely benign	GeneDx	11/28/2017	FANCC	chr9	97933437	97933437	http://gnomad.b	1.66E-05	AMR	0.000119352997	0		0	0	https://www.ncbi.nlm.nih.gov/clinvar/variation/381363/
NM_000136.2(FANCC):c.233C>A	concordant	unreportable	unreportable	418955	0		2	SCV000566398;S(	Uncertain significance;Unc	GeneDx;Invitae	2017-11-28;2017-1	FANCC	chr9	98009731	98009731	http://gnomad.b	1.19E-05	AFR	6.54E-05	0		0	0	https://www.ncbi.nlm.nih.gov/clinvar/variation/418955/
NM_000136.2(FANCC):c.-29A>C	concordant	unreportable	unreportable	137287	0		1	SCV000481139	Likely benign	Illumina Clinical Se	10/18/2016	FANCC	chr9	98011602	98011602	http://gnomad.b	0.002730070249	EAS	0.05170400068	51		23	0	https://www.ncbi.nlm.nih.gov/clinvar/variation/137287/
NM_000035.3(ALDOB):c.799+11C>T	concordant	unreportable	unreportable	384999	0		1	SCV000526112	Likely benign	GeneDx	11/28/2017	ALDOB	chr9	104187724	104187724	http://gnomad.b	7.62E-05	AFR	0.0021565801	0		0	0	https://www.ncbi.nlm.nih.gov/clinvar/variation/384999/
NM_000035.3(ALDOB):c.799+6G>A	concordant	unreportable	unreportable	254732	0		3	SCV000706102;S(	Likely benign;Likely benign	EGL Genetic Diagn	2017-06-30;2017-1	ALDOB	chr9	104187729	104187729	http://gnomad.b	0.001305946995	ASJ	0.008021930233	1		0	0	https://www.ncbi.nlm.nih.gov/clinvar/variation/254732/
NM_000035.3(ALDOB):c.766G>T	concordant	unreportable	unreportable	502283	0		1	SCV000708955	Uncertain significance	EGL Genetic Diagn	6/30/2017	ALDOB	chr9	104187768	104187768	http://gnomad.b	1.41E-05	AFR	0.0001960530062	0		0	0	https://www.ncbi.nlm.nih.gov/clinvar/variation/502283/
NM_000035.3(ALDOB):c.325-19T>G	concordant	unreportable	unreportable	388143	0		1	SCV000530380	Likely benign	GeneDx	11/28/2017	ALDOB	chr9	104190824	104190824	http://gnomad.b	0.0003892455406	SAS	0.002306540031	2		0	0	https://www.ncbi.nlm.nih.gov/clinvar/variation/388143/
NM_000035.3(ALDOB):c.324+8C>G	concordant	unreportable	unreportable	196201	0		1	SCV000476068	Likely benign	Illumina Clinical Se	10/18/2016	ALDOB	chr9	104192029	104192029	http://gnomad.b	0.001486466666	EAS	0.03507649899	16		12	0	https://www.ncbi.nlm.nih.gov/clinvar/variation/196201/
NM_000035.3(ALDOB):c.243C>T	concordant	unreportable	unreportable	364316	0		1	SCV000476070	Uncertain significance	Illumina Clinical Se	10/18/2016	ALDOB	chr9	104192118	104192118	http://gnomad.b	3.67E-05	SAS	0.0003248649882	1		0	0	https://www.ncbi.nlm.nih.gov/clinvar/variation/364316/
NM_001079802.1(FKTN):c.75G>A	concordant	unreportable	unreportable	414299	0		1	SCV000557838	Likely benign	Invitae	3/14/2017	FKTN	chr9	108337388	108337388	http://gnomad.b	3.19E-05	AMR	5.96E-05	0		0	0	https://www.ncbi.nlm.nih.gov/clinvar/variation/414299/
NM_001079802.1(FKTN):c.102A>C	concordant	unreportable	unreportable	380678	0		1	SCV000630811	Likely benign	Invitae	10/5/2017	FKTN	chr9	108337415	108337415	http://gnomad.b	6.38E-05	AFR	0.0005885430146	0		0	0	https://www.ncbi.nlm.nih.gov/clinvar/variation/380678/
NM_001079802.1(FKTN):c.106-10G>A	concordant	unreportable	unreportable	93513	0		2	SCV000151167;S(	Likely benign;Benign	Genetic Services La	2014-06-27;2017-1	FKTN	chr9	108358869	108358869	http://gnomad.b	0.001414783244	AFR	0.03275790066	7		6	0	https://www.ncbi.nlm.nih.gov/clinvar/variation/93513/
NM_001079802.1(FKTN):c.910+14G>A	concordant	unreportable	unreportable	93525	0		1	SCV000594789	Likely benign	Genetic Services La	7/5/2017	FKTN	chr9	108377702	108377702	http://gnomad.b	0.005637858039	SAS	0.02322790027	29		15	0	https://www.ncbi.nlm.nih.gov/clinvar/variation/93525/
NM_003640.3(IKBKAP):c.3876T>G	concordant	unreportable	unreportable	364551	0		3	SCV000476540;S(	Uncertain significance;Beni	Illumina Clinical Se	2016-10-18;2017-1	IKBKAP	chr9	111637230	111637230	http://gnomad.b	0.007750140761	ASJ	0.05381799862	42		35	0	https://www.ncbi.nlm.nih.gov/clinvar/variation/364551/
NM_003640.3(IKBKAP):c.3513T>C	concordant	unreportable	unreportable	364556	0		1	SCV000476545	Uncertain significance	Illumina Clinical Se	10/18/2016	IKBKAP	chr9	111641785	111641785	http://gnomad.b	5.68E-05	EAS	0.001449440024	0		0	0	https://www.ncbi.nlm.nih.gov/clinvar/variation/364556/
NM_003640.3(IKBKAP):c.3347-15A>G	concordant	unreportable	unreportable	380135	0		1	SCV000517890	Likely benign	GeneDx	11/28/2017	IKBKAP	chr9	111642460	111642460	http://gnomad.b	0.0008658582169	ASJ	0.01056700014	0		0	0	https://www.ncbi.nlm.nih.gov/clinvar/variation/380135/
NM_003640.3(IKBKAP):c.3346+7G>T	concordant	unreportable	unreportable	382378	0		1	SCV000522292	Likely benign	GeneDx	11/28/2017	IKBKAP	chr9	111643978	111643978	http://gnomad.b	3.31E-05	AFR	0.0001311649976	0		0	0	https://www.ncbi.nlm.nih.gov/clinvar/variation/382378/
NM_003640.3(IKBKAP):c.2631C>T	concordant	unreportable	unreportable	381818	0		2	SCV000521484;S(	Likely benign;Benign	GeneDx;Invitae	2017-11-28;2017-1	IKBKAP	chr9	111658881	111658881	http://gnomad.b	0.0009675847287	AFR	0.02875819989	1		3	0	https://www.ncbi.nlm.nih.gov/clinvar/variation/381818/
NM_003640.3(IKBKAP):c.2587+14C>T	concordant	unreportable	unreportable	137580	0		1	SCV000476561	Uncertain significance	Illumina Clinical Se	10/18/2016	IKBKAP	chr9	111659219	111659219	http://gnomad.b	0.00669738965	NFE	0.02770899981	64		36	0	https://www.ncbi.nlm.nih.gov/clinvar/variation/137580/
NM_003640.3(IKBKAP):c.2543C>A	concordant	unreportable	unreportable	245634	0		4	SCV000700566;S(	Likely benign;Likely benign	EGL Genetic Diagn	2017-06-30;2017-1	IKBKAP	chr9	111659277	111659277	http://gnomad.b	0.001111894432	SAS	0.00438567996	3		1	0	https://www.ncbi.nlm.nih.gov/clinvar/variation/245634/
NM_003640.3(IKBKAP):c.2436C>T	concordant	unreportable	unreportable	455960	0		1	SCV000626013	Likely benign	Invitae	10/5/2017	IKBKAP	chr9	111659493	111659493	http://gnomad.b	4.49E-05	AFR	0.001045610057	0		0	0	https://www.ncbi.nlm.nih.gov/clinvar/variation/455960/
NM_003640.3(IKBKAP):c.2217A>G	concordant	unreportable	unreportable	499530	0		1	SCV000705057	Uncertain significance	EGL Genetic Diagn	6/30/2017	IKBKAP	chr9	111661017	111661017	http://gnomad.b	3.08E-05	AFR	0.0009812900098	0		0	0	https://www.ncbi.nlm.nih.gov/clinvar/variation/499530/
NM_003640.3(IKBKAP):c.2204+18G>C	concordant	unreportable	unreportable	382631	0		1	SCV000522622	Likely benign	GeneDx	11/28/2017	IKBKAP	chr9	111662084	111662084	http://gnomad.b	0.0003051795355	AFR	0.008900520392	1		0	0	https://www.ncbi.nlm.nih.gov/clinvar/variation/382631/
NM_003640.3(IKBKAP):c.1886G>A	concordant	unreportable	unreportable	194739	0		4	SCV000226616;S(	Uncertain significance;Unc	EGL Genetic Diagn	2017-06-30;2017-1	IKBKAP	chr9	111663930	111663930	http://gnomad.b	0.001092968569	ASJ	0.004978660028	0		2	0	https://www.ncbi.nlm.nih.gov/clinvar/variation/194739/
NM_003640.3(IKBKAP):c.1878C>T	concordant	unreportable	unreportable	364570	0		2	SCV000476573;S(	Uncertain significance;Likel	Illumina Clinical Se	2016-10-18;2017-1	IKBKAP	chr9	111663938	111663938	http://gnomad.b	0.000212915955	EAS	0.001217809971	0		3	0	https://www.ncbi.nlm.nih.gov/clinvar/variation/364570/
NM_003640.3(IKBKAP):c.1704A>G	concordant	unreportable	unreportable	497746	0		1	SCV000702440	Uncertain significance	EGL Genetic Diagn	6/30/2017	IKBKAP	chr9	111665889	111665889	http://gnomad.b	5.20E-05	ASJ	0.0002031689946	0		0	0	https://www.ncbi.nlm.nih.gov/clinvar/variation/497746/
NM_003640.3(IKBKAP):c.1656G>A	concordant	unreportable	unreportable	455956	0		1	SCV000626006	Likely benign	Invitae	10/5/2017	IKBKAP	chr9	111665937	111665937	http://gnomad.b	7.10E-06	AFR	6.54E-05	0		0	0	https://www.ncbi.nlm.nih.gov/clinvar/variation/455956/
NM_003640.3(IKBKAP):c.1364A>G	concordant	unreportable	unreportable	242315	0		1	SCV000291964	Uncertain significance	GeneDx	11/28/2017	IKBKAP	chr9	111670681	111670681	http://gnomad.b	7.57E-05	AFR	0.00248365989	0		0	0	https://www.ncbi.nlm.nih.gov/clinvar/variation/242315/
NM_003640.3(IKBKAP):c.1230G>A	concordant	unreportable	unreportable	137573	0		3	SCV000338610;S(	Benign;Uncertain significar	EGL Genetic Diagn	2017-06-30;2016-1	IKBKAP	chr9	111673420	111673420	http://gnomad.b	0.002249811924	FIN	0.02919539995	13		3	0	https://www.ncbi.nlm.nih.gov/clinvar/variation/137573/
NM_003640.3(IKBKAP):c.1143G>A	concordant	unreportable	unreportable	380994	0		2	SCV000520023;S(	Likely benign;Benign	GeneDx;Invitae	2017-11-28;2017-1	IKBKAP	chr9	111674590	111674590	http://gnomad.b	0.0002625963445	AFR	0.007383690216	0		1	0	https://www.ncbi.nlm.nih.gov/clinvar/variation/380994/
NM_003640.3(IKBKAP):c.1073G>A	concordant	unreportable	unreportable	364576	0		2	SCV000476581;S(	Uncertain significance;Unc	Illumina Clinical Se	2016-10-18;2017-1	IKBKAP	chr9	111674660	111674660	http://gnomad.b	6.39E-05	EAS	0.0005218000151	0		0	0	https://www.ncbi.nlm.nih.gov/clinvar/variation/364576/
NM_003640.3(IKBKAP):c.959-15C>T	concordant	unreportable	unreportable	137572	0		1	SCV000476582	Uncertain significance	Illumina Clinical Se	10/18/2016	IKBKAP	chr9	111674789	111674789	http://gnomad.b	0.007213119408	NFE	0.02796670049	65		36	0	https://www.ncbi.nlm.nih.gov/clinvar/variation/137572/
NM_003640.3(IKBKAP):c.948G>A	concordant	unreportable	unreportable	455974	0		1	SCV000626033	Likely benign	Invitae	10/5/2017	IKBKAP	chr9	111678494	111678494	http://gnomad.b	0.0004140032458	NFE	0.0008596450207	0		0	0	https://www.ncbi.nlm.nih.gov/clinvar/variation/455974/
NM_003640.3(IKBKAP):c.934G>A	concordant	unreportable	unreportable	137571	0		2	SCV000476584;S(	Uncertain significance;Beni	Illumina Clinical Se	2016-10-18;2017-1	IKBKAP	chr9	111678508	111678508	http://gnomad.b	0.007586905196	NFE	0.02786830068	70		37	0	https://www.ncbi.nlm.nih.gov/clinvar/variation/137571/
NM_003640.3(IKBKAP):c.923T>C	concordant	unreportable	unreportable	364578	0		2	SCV000604025;S(	Uncertain significance;Unc	ARUP Laboratories	2017-06-30;2016-1	IKBKAP	chr9	111678519	111678519	http://gnomad.b	0.0003335683295	AFR	0.007775740232	0		1	0	https://www.ncbi.nlm.nih.gov/clinvar/variation/364578/
NM_003640.3(IKBKAP):c.751A>G	concordant	unreportable	unreportable	137569	0		2	SCV000476587;S(	Uncertain significance;Beni	Illumina Clinical Se	2016-10-18;2017-1	IKBKAP	chr9	111679940	111679940	http://gnomad.b	0.006867722414	NFE	0.02786679938	65		35	0	https://www.ncbi.nlm.nih.gov/clinvar/variation/137569/
NM_003640.3(IKBKAP):c.322G>A	concordant	unreportable	unreportable	364579	0		1	SCV000476591	Uncertain significance	Illumina Clinical Se	10/18/2016	IKBKAP	chr9	111689715	111689715	http://gnomad.b	3.79E-05	EAS	0.0004059849889	0		0	0	https://www.ncbi.nlm.nih.gov/clinvar/variation/364579/
NM_003640.3(IKBKAP):c.286A>G	concordant	unreportable	unreportable	380993	0		2	SCV000520022;S(	Likely benign;Benign	GeneDx;Invitae	2017-11-28;2017-1	IKBKAP	chr9	111692066	111692066	http://gnomad.b	0.0003737857876	AFR	0.01058549993	0		1	0	https://www.ncbi.nlm.nih.gov/clinvar/variation/380993/
NM_003640.3(IKBKAP):c.209G>A	concordant	unreportable	unreportable	364581	0		3	SCV000519956;S(	Likely benign;Uncertain sig	GeneDx;Illumina C	2017-11-28;2016-1	IKBKAP	chr9	111692143	111692143	http://gnomad.b	0.0006387478649	EAS	0.007942950353	1		2	0	https://www.ncbi.nlm.nih.gov/clinvar/variation/364581/
NM_003640.3(IKBKAP):c.208C>T	concordant	unreportable	unreportable	285240	0		2	SCV000338181;S(	Uncertain significance;Unc	EGL Genetic Diagn	2017-06-30;2016-1	IKBKAP	chr9	111692144	111692144	http://gnomad.b	0.0001916243595	EAS	0.003072819905	0		0	0	https://www.ncbi.nlm.nih.gov/clinvar/variation/285240/
NM_003640.3(IKBKAP):c.203G>T	concordant	unreportable	unreportable	364582	0		1	SCV000476595	Uncertain significance	Illumina Clinical Se	10/18/2016	IKBKAP	chr9	111692149	111692149	http://gnomad.b	0.0001040922447	AFR	0.00294041005	0		1	0	https://www.ncbi.nlm.nih.gov/clinvar/variation/364582/
NM_003640.3(IKBKAP):c.189C>T	concordant	unreportable	unreportable	137581	0		2	SCV000476596;S(	Uncertain significance;Beni	Illumina Clinical Se	2016-10-18;2017-1	IKBKAP	chr9	111692163	111692163	http://gnomad.b	0.00715634182	NFE	0.02784940042	65		36	0	https://www.ncbi.nlm.nih.gov/clinvar/variation/137581/
NM_003640.3(IKBKAP):c.-17G>A	concordant	unreportable	unreportable	382635	0		1	SCV000522629	Likely benign	GeneDx	11/28/2017	IKBKAP	chr9	111693443	111693443	http://gnomad.b	0.0001159209088	ASJ	0.002335029887	0		0	0	https://www.ncbi.nlm.nih.gov/clinvar/variation/382635/
NM_000050.4(ASS1):c.106-10T>G	concordant	unreportable	unreportable	197098	0		2	SCV000477616;S(	Uncertain significance;Beni	Illumina Clinical Se	2016-10-18;2017-1	ASS1	chr9	133329682	133329682	http://gnomad.b	0.0006517125558	AFR	0.01874490082	3		1	0	https://www.ncbi.nlm.nih.gov/clinvar/variation/197098/
NM_000050.4(ASS1):c.363+16A>G	concordant	unreportable	unreportable	254744	0		1	SCV000521496	Benign	GeneDx	11/28/2017	ASS1	chr9	133333992	133333992	http://gnomad.b	0.001403688582	EAS	0.03617719933	14		9	0	https://www.ncbi.nlm.nih.gov/clinvar/variation/254744/
NM_000050.4(ASS1):c.364-6T>C	concordant	unreportable	unreportable	254745	0		3	SCV000521834;S(	Benign;Likely benign;Benig	GeneDx;Illumina C	2017-11-28;2016-1	ASS1	chr9	133339492	133339492	http://gnomad.b	0.001200376545	AFR	0.03117240034	7		8	0	https://www.ncbi.nlm.nih.gov/clinvar/variation/254745/
NM_033056.3(PCDH15):c.3983+12T>C	concordant	unreportable	unreportable	138586	0		1	SCV000363153	Uncertain significance	Illumina Clinical Se	10/18/2016	PCDH15	chr10	55600068	55600068	http://gnomad.b	0.001941572803	NFE	0.007836010307	8		5	0	https://www.ncbi.nlm.nih.gov/clinvar/variation/138586/
NM_033056.3(PCDH15):c.3877C>A	concordant	unreportable	unreportable	383744	0		1	SCV000524232	Likely benign	GeneDx	11/28/2017	PCDH15	chr10	55600186	55600186	http://gnomad.b	0.0001800133724	AFR	0.001829829998	0		0	0	https://www.ncbi.nlm.nih.gov/clinvar/variation/383744/
NM_033056.3(PCDH15):c.3717+8G>C	concordant	unreportable	unreportable	196045	0		2	SCV000228483;S(	Benign;Likely benign	EGL Genetic Diagn	2017-06-30;2017-1	PCDH15	chr10	55626394	55626394	http://gnomad.b	0.0002391606234	AFR	0.01034440007	5		0	0	https://www.ncbi.nlm.nih.gov/clinvar/variation/196045/
NM_033056.3(PCDH15):c.3374-16G>A	concordant	unreportable	unreportable	378324	0		1	SCV000514043	Likely benign	GeneDx	11/28/2017	PCDH15	chr10	55663146	55663146	http://gnomad.b	0.0001774417528	NFE	0.0006822629948	0		0	0	https://www.ncbi.nlm.nih.gov/clinvar/variation/378324/
NM_033056.3(PCDH15):c.2138A>G	concordant	unreportable	unreportable	286893	0		2	SCV000340490;S(	Uncertain significance;Unc	EGL Genetic Diagn	2016-11-03;2016-1	PCDH15	chr10	55826599	55826599	http://gnomad.b	5.14E-05	EAS	5.80E-05	0		0	0	https://www.ncbi.nlm.nih.gov/clinvar/variation/286893/
NM_033056.3(PCDH15):c.1548G>A	concordant	unreportable	unreportable	285402	0		1	SCV000338406	Uncertain significance	EGL Genetic Diagn	6/30/2017	PCDH15	chr10	55943246	55943246	http://gnomad.b	3.60E-05	AFR	0.0005228070077	0		0	0	https://www.ncbi.nlm.nih.gov/clinvar/variation/285402/
NM_000543.4(SMPD1):c.236G>A	concordant	unreportable	unreportable	500518	0		1	SCV000706514	Uncertain significance	EGL Genetic Diagn	6/30/2017	SMPD1	chr11	6412064	6412064	http://gnomad.b	2.85E-05	AMR	0.0007444460061	0		0	0	https://www.ncbi.nlm.nih.gov/clinvar/variation/500518/
NM_000543.4(SMPD1):c.319-17C>A	concordant	unreportable	unreportable	286870	0		1	SCV000340449	Uncertain significance	EGL Genetic Diagn	6/30/2017	SMPD1	chr11	6412597	6412597	http://gnomad.b	0.0002806850618	NFE	0.0009478150168	0		0	0	https://www.ncbi.nlm.nih.gov/clinvar/variation/286870/
NM_000543.4(SMPD1):c.657G>A	concordant	unreportable	unreportable	500262	0		1	SCV000706130	Uncertain significance	EGL Genetic Diagn	6/30/2017	SMPD1	chr11	6412952	6412952	http://gnomad.b	3.09E-05	AMR	0.0002392489987	0		0	0	https://www.ncbi.nlm.nih.gov/clinvar/variation/500262/
NM_000543.4(SMPD1):c.1091+9C>T	concordant	unreportable	unreportable	285417	0		2	SCV000338426;S(	Likely benign;Uncertain sig	EGL Genetic Diagn	2017-06-30;2016-1	SMPD1	chr11	6413395	6413395	http://gnomad.b	9.04E-05	AFR	0.002161100041	0		0	0	https://www.ncbi.nlm.nih.gov/clinvar/variation/285417/
NM_000543.4(SMPD1):c.1091+10G>A	concordant	unreportable	unreportable	498584	0		1	SCV000703671	Uncertain significance	EGL Genetic Diagn	6/30/2017	SMPD1	chr11	6413396	6413396	http://gnomad.b	4.04E-05	AFR	0.001244919957	0		0	0	https://www.ncbi.nlm.nih.gov/clinvar/variation/498584/
NM_000543.4(SMPD1):c.1632C>T	concordant	unreportable	unreportable	282907	0		1	SCV000334647	Uncertain significance	EGL Genetic Diagn	6/30/2017	SMPD1	chr11	6415573	6415573	http://gnomad.b	0.000135585157	EAS	0.001913269982	0		0	0	https://www.ncbi.nlm.nih.gov/clinvar/variation/282907/
NM_000391.3(TPP1):c.1076-18A>G	concordant	unreportable	unreportable	495858	0		1	SCV000696661	Benign	Laboratory Corpora	1/25/2018	TPP1	chr11	6637323	6637323	http://gnomad.b	0.003142120078	NFE	0.01296649966	12		7	0	https://www.ncbi.nlm.nih.gov/clinvar/variation/495858/
NM_000391.3(TPP1):c.-40T>C	concordant	unreportable	unreportable	207557	0		1	SCV000373340	Uncertain significance	Illumina Clinical Se	10/18/2016	TPP1	chr11	6640671	6640671	http://gnomad.b	0.0002490193116	NFE	0.0007613620255	0		0	0	https://www.ncbi.nlm.nih.gov/clinvar/variation/207557/
NM_000352.3(ABCC8):c.4119+15C>T	concordant	unreportable	unreportable	303753	0		1	SCV000369247	Likely benign	Illumina Clinical Se	10/18/2016	ABCC8	chr11	17418448	17418448	http://gnomad.b	0.0001987435279	EAS	0.00179730996	0		1	0	https://www.ncbi.nlm.nih.gov/clinvar/variation/303753/
NM_000352.3(ABCC8):c.3552G>A	concordant	unreportable	unreportable	303762	0		2	SCV000592964;S(	Likely benign;Likely benign	Genetic Services La	2017-07-05;2016-1	ABCC8	chr11	17426064	17426064	http://gnomad.b	4.39E-05	NFE	4.48E-05	0		0	0	https://www.ncbi.nlm.nih.gov/clinvar/variation/303762/
NM_000352.3(ABCC8):c.2556+22G>A	concordant	unreportable	unreportable	157693	0		1	SCV000192024	Likely benign	Genetic Services La	9/11/2014	ABCC8	chr11	17434191	17434191	http://gnomad.b	0.001233758524	AFR	0.03368220106	13		12	0	https://www.ncbi.nlm.nih.gov/clinvar/variation/157693/
NM_000352.3(ABCC8):c.2060C>T	concordant	unreportable	unreportable	303777	0		1	SCV000369344	Likely benign	Illumina Clinical Se	10/18/2016	ABCC8	chr11	17449470	17449470	http://gnomad.b	0.0001264731541	EAS	0.002206479898	1		0	0	https://www.ncbi.nlm.nih.gov/clinvar/variation/303777/
NM_000352.3(ABCC8):c.2041-12C>T	concordant	unreportable	unreportable	303778	0		2	SCV000612203;S(	Uncertain significance;Likel	Athena Diagnostics	2017-08-17;2016-1	ABCC8	chr11	17449501	17449501	http://gnomad.b	0.0002658517322	NFE	0.0009561449988	0		0	0	https://www.ncbi.nlm.nih.gov/clinvar/variation/303778/
NM_000352.3(ABCC8):c.1630+10C>T	concordant	unreportable	unreportable	446764	0		1	SCV000612200	Uncertain significance	Athena Diagnostics	8/17/2017	ABCC8	chr11	17464257	17464257	http://gnomad.b	7.74E-05	AFR	0.002223680029	0		0	0	https://www.ncbi.nlm.nih.gov/clinvar/variation/446764/
NM_000352.3(ABCC8):c.822+20C>T	concordant	unreportable	unreportable	255934	0		1	SCV000612224	Likely benign	Athena Diagnostics	8/17/2017	ABCC8	chr11	17483110	17483110	http://gnomad.b	0.0001497029171	AFR	0.004051759839	0		0	0	https://www.ncbi.nlm.nih.gov/clinvar/variation/255934/
NM_001173990.2(TMEM216):c.406G>	concordant	unreportable	unreportable	500240	0		1	SCV000706098	Uncertain significance	EGL Genetic Diagn	6/30/2017	TMEM216	chr11	61165422	61165422	http://gnomad.b	5.91E-05	EAS	0.0007537109777	0		0	0	https://www.ncbi.nlm.nih.gov/clinvar/variation/500240/
NM_024649.4(BBS1):c.831-5C>T	concordant	unreportable	unreportable	96132	0		1	SCV000593580	Likely benign	Genetic Services La	7/5/2017	BBS1	chr11	66290922	66290922	http://gnomad.b	0.000320459232	AFR	0.009147940204	1		0	0	https://www.ncbi.nlm.nih.gov/clinvar/variation/96132/
NM_024649.4(BBS1):c.1595G>A	concordant	unreportable	unreportable	285977	0		1	SCV000339219	Uncertain significance	EGL Genetic Diagn	6/30/2017	BBS1	chr11	66298486	66298486	http://gnomad.b	3.90E-05	AMR	0.0006551130209	0		0	0	https://www.ncbi.nlm.nih.gov/clinvar/variation/285977/
NM_001876.3(CPT1A):c.2142+8C>T	concordant	unreportable	unreportable	282930	0		2	SCV000334676;S(	Likely benign;Likely benign	EGL Genetic Diagn	2017-06-30;2017-1	CPT1A	chr11	68527685	68527685	http://gnomad.b	0.0003970600659	SAS	0.002317219973	0		1	0	https://www.ncbi.nlm.nih.gov/clinvar/variation/282930/
NM_001876.3(CPT1A):c.1163+5G>A	concordant	unreportable	unreportable	463964	0		1	SCV000638092	Likely benign	Invitae	10/5/2017	CPT1A	chr11	68552278	68552278	http://gnomad.b	0.0002759708259	AFR	0.006274770014	0		0	0	https://www.ncbi.nlm.nih.gov/clinvar/variation/463964/
NM_001876.3(CPT1A):c.1092G>A	concordant	unreportable	unreportable	283774	0		1	SCV000336084	Uncertain significance	EGL Genetic Diagn	6/30/2017	CPT1A	chr11	68552354	68552354	http://gnomad.b	4.79E-05	EAS	0.001333949971	0		0	0	https://www.ncbi.nlm.nih.gov/clinvar/variation/283774/
NM_001876.3(CPT1A):c.967+11C>G	concordant	unreportable	unreportable	392119	0		1	SCV000535333	Likely benign	GeneDx	11/28/2017	CPT1A	chr11	68560772	68560772	http://gnomad.b	2.25E-05	EAS	0.0005841800012	0		0	0	https://www.ncbi.nlm.nih.gov/clinvar/variation/392119/
NM_001876.3(CPT1A):c.495C>T	concordant	unreportable	unreportable	287513	0		2	SCV000341302;S(	Uncertain significance;Likel	EGL Genetic Diagn	2017-06-30;2017-1	CPT1A	chr11	68571528	68571528	http://gnomad.b	5.07E-05	SAS	9.75E-05	0		0	0	https://www.ncbi.nlm.nih.gov/clinvar/variation/287513/
NM_001876.3(CPT1A):c.453+9G>A	concordant	unreportable	unreportable	377753	0		1	SCV000512759	Likely benign	GeneDx	11/28/2017	CPT1A	chr11	68574926	68574926	http://gnomad.b	0.0001070090958	AFR	0.002515560016	0		0	0	https://www.ncbi.nlm.nih.gov/clinvar/variation/377753/
NM_001876.3(CPT1A):c.142-16G>A	concordant	unreportable	unreportable	387499	0		1	SCV000529566	Likely benign	GeneDx	11/28/2017	CPT1A	chr11	68580060	68580060	http://gnomad.b	0.0003660837487	EAS	0.01016500033	0		0	0	https://www.ncbi.nlm.nih.gov/clinvar/variation/387499/
NM_001360.2(DHCR7):c.906C>T	concordant	unreportable	unreportable	198772	0		1	SCV000536084	Likely benign	GeneDx	11/28/2017	DHCR7	chr11	71148915	71148915	http://gnomad.b	0.0001777098053	AFR	0.001502880012	0		0	0	https://www.ncbi.nlm.nih.gov/clinvar/variation/198772/
NM_001360.2(DHCR7):c.289G>A	concordant	unreportable	unreportable	305958	0		1	SCV000373924	Uncertain significance	Illumina Clinical Se	10/18/2016	DHCR7	chr11	71155071	71155071	http://gnomad.b	3.50E-05	AFR	6.61E-05	0		0	0	https://www.ncbi.nlm.nih.gov/clinvar/variation/305958/
NM_001360.2(DHCR7):c.99-4G>A	concordant	unreportable	unreportable	93726	0		2	SCV000630079;S(	Benign;Benign	Invitae;Laboratory	2017-10-05;2018-0	DHCR7	chr11	71155265	71155265	http://gnomad.b	0.003955389454	FIN	0.01623759978	17		5	0	https://www.ncbi.nlm.nih.gov/clinvar/variation/93726/
NM_001360.2(DHCR7):c.-6-4G>A	concordant	unreportable	unreportable	305961	0		1	SCV000373931	Uncertain significance	Illumina Clinical Se	10/18/2016	DHCR7	chr11	71156008	71156008	http://gnomad.b	6.46E-05	AFR	0.002025610069	0		0	0	https://www.ncbi.nlm.nih.gov/clinvar/variation/305961/
NM_000051.3(ATM):c.131A>G	concordant	unreportable	unreportable	186471	0		4	SCV000681966;S(	Uncertain significance;Unc	Color Genomics, In	2017-10-26;2017-1	ATM	chr11	108098561	108098561	http://gnomad.b	3.90E-05	AFR	0.0004578160006	0		0	0	https://www.ncbi.nlm.nih.gov/clinvar/variation/186471/

Supplemental Table 2

NM_000051.3(ATM):c.186-17A>G	concordant	unreportable	unreportable	489518	0		1	SCV000682008	Benign	Color Genomics, In	10/26/2017	ATM	chr11	108099888	108099888	http://gnomad.b	0.002233336304	AFR	0.04752320051	10		18	0	https://www.ncbi.nlm.nih.gov/clinvar/variation/489518/
NM_000051.3(ATM):c.186-7C>T	concordant	unreportable	unreportable	135737	0		3	SCV000682009;S(	Benign;Benign;Benign	Color Genomics, In	2017-10-26;2017-1	ATM	chr11	108099898	108099898	http://gnomad.b	0.0005123863839	AFR	0.01798070036	2		5	0	https://www.ncbi.nlm.nih.gov/clinvar/variation/135737/
NM_000051.3(ATM):c.1021G>A	concordant	unreportable	unreportable	133643	0		2	SCV000292850;S(	Uncertain significance;Unc	GeneDx;Invitae	2017-11-28;2017-0	ATM	chr11	108117810	108117810	http://gnomad.b	1.39E-05	AFR	0.000130786997	0		0	0	https://www.ncbi.nlm.nih.gov/clinvar/variation/133643/
NM_000051.3(ATM):c.1511A>G	concordant	unreportable	unreportable	220272	0		2	SCV000570155;S(	Uncertain significance;Unc	GeneDx;Invitae	2017-07-13;2017-0	ATM	chr11	108121703	108121703	http://gnomad.b	7.52E-05	EAS	0.0008116880199	0		0	0	https://www.ncbi.nlm.nih.gov/clinvar/variation/220272/
NM_000051.3(ATM):c.2124+13A>T	concordant	unreportable	unreportable	377505	0		2	SCV000682026;S(	Likely benign;Likely benign	Color Genomics, In	2017-10-26;2017-1	ATM	chr11	108124779	108124779	http://gnomad.b	8.35E-06	EAS	0.0002921239939	0		0	0	https://www.ncbi.nlm.nih.gov/clinvar/variation/377505/
NM_000051.3(ATM):c.2220A>G	concordant	unreportable	unreportable	184290	0		2	SCV000682041;S(	Likely benign;Likely benign	Color Genomics, In	2017-10-26;2017-1	ATM	chr11	108127037	108127037	http://gnomad.b	0.0001392354304	EAS	0.001337519963	1		0	0	https://www.ncbi.nlm.nih.gov/clinvar/variation/184290/
NM_000051.3(ATM):c.2376+20G>C	concordant	unreportable	unreportable	377506	0		1	SCV000682050	Likely benign	Color Genomics, In	10/26/2017	ATM	chr11	108128353	108128353	http://gnomad.b	7.52E-05	EAS	0.002381779952	0		0	0	https://www.ncbi.nlm.nih.gov/clinvar/variation/377506/
NM_000051.3(ATM):c.2804C>G	concordant	unreportable	unreportable	185083	0		1	SCV000622362	Uncertain significance	Invitae	10/5/2017	ATM	chr11	108139302	108139302	http://gnomad.b	5.01E-05	EAS	0.0002319649939	0		0	0	https://www.ncbi.nlm.nih.gov/clinvar/variation/185083/
NM_000051.3(ATM):c.2927T>C	concordant	unreportable	unreportable	187123	0		4	SCV000682090;S(	Uncertain significance;Unc	Color Genomics, In	2017-10-26;2017-1	ATM	chr11	108141983	108141983	http://gnomad.b	5.01E-05	AFR	0.0005883889971	0		0	0	https://www.ncbi.nlm.nih.gov/clinvar/variation/187123/
NM_000051.3(ATM):c.3077+4G>A	concordant	unreportable	unreportable	231928	0		2	SCV000546998;S(	Likely benign;Uncertain sig	Invitae;Laboratory	2017-10-05;2018-0	ATM	chr11	108142137	108142137	http://gnomad.b	2.23E-05	AFR	0.0006576349842	0		0	0	https://www.ncbi.nlm.nih.gov/clinvar/variation/231928/
NM_000051.3(ATM):c.3402+14A>C	concordant	unreportable	unreportable	490526	0		1	SCV000687469	Likely benign	Color Genomics, In	12/21/2017	ATM	chr11	108150349	108150349	http://gnomad.b	2.78E-05	EAS	0.0004065989924	0		0	0	https://www.ncbi.nlm.nih.gov/clinvar/variation/490526/
NM_000051.3(ATM):c.3402+17T>C	concordant	unreportable	unreportable	136440	0		1	SCV000682126	Likely benign	Color Genomics, In	10/26/2017	ATM	chr11	108150352	108150352	http://gnomad.b	0.0002172072714	SAS	0.001203019987	0		0	0	https://www.ncbi.nlm.nih.gov/clinvar/variation/136440/
NM_000051.3(ATM):c.3899A>G	concordant	unreportable	unreportable	181952	0		3	SCV000687506;S(	Uncertain significance;Unc	Color Genomics, In	2017-12-21;2016-1	ATM	chr11	108155106	108155106	http://gnomad.b	7.24E-05	SAS	0.0003898639989	0		1	0	https://www.ncbi.nlm.nih.gov/clinvar/variation/181952/
NM_000051.3(ATM):c.5005+17C>T	concordant	unreportable	unreportable	490599	0		1	SCV000687596	Likely benign	Color Genomics, In	12/21/2017	ATM	chr11	108168126	108168126	http://gnomad.b	1.95E-05	EAS	0.0001165909998	0		0	0	https://www.ncbi.nlm.nih.gov/clinvar/variation/490599/
NM_000051.3(ATM):c.5005+18G>A	concordant	unreportable	unreportable	490600	0		1	SCV000687597	Benign	Color Genomics, In	12/21/2017	ATM	chr11	108168127	108168127	http://gnomad.b	0.0004566922117	AFR	0.01247869991	3		0	0	https://www.ncbi.nlm.nih.gov/clinvar/variation/490600/
NM_000051.3(ATM):c.5063T>C	concordant	unreportable	unreportable	141932	0		3	SCV000687604;S(	Uncertain significance;Unc	Color Genomics, In	2017-12-21;2017-1	ATM	chr11	108170498	108170498	http://gnomad.b	3.90E-05	EAS	0.0008697669837	0		0	0	https://www.ncbi.nlm.nih.gov/clinvar/variation/141932/
NM_000051.3(ATM):c.5156A>G	concordant	unreportable	unreportable	421320	0		3	SCV000682252;S(	Uncertain significance;Unc	Color Genomics, In	2017-10-26;2017-0	ATM	chr11	108170591	108170591	http://gnomad.b	1.67E-05	AMR	2.98E-05	0		0	0	https://www.ncbi.nlm.nih.gov/clinvar/variation/421320/
NM_000051.3(ATM):c.5497-8T>C	concordant	unreportable	unreportable	132706	0		3	SCV000602563;S(	Benign;Benign;Benign	ARUP Laboratories	2017-06-30;2017-0	ATM	chr11	108175394	108175394	http://gnomad.b	0.01009735341	NFE	0.03328479826	88		73	0	https://www.ncbi.nlm.nih.gov/clinvar/variation/132706/
NM_000051.3(ATM):c.6096-14A>G	concordant	unreportable	unreportable	377520	0		2	SCV000682304;S(	Likely benign;Likely benign	Color Genomics, In	2017-10-26;2017-1	ATM	chr11	108186724	108186724	http://gnomad.b	6.13E-05	EAS	0.000174418994	0		0	0	https://www.ncbi.nlm.nih.gov/clinvar/variation/377520/
NM_000051.3(ATM):c.6101G>A	concordant	unreportable	unreportable	127418	0		3	SCV000682307;S(	Uncertain significance;Unc	Color Genomics, In	2017-10-26;2017-1	ATM	chr11	108186743	108186743	http://gnomad.b	7.52E-05	EAS	0.0007551119779	0		0	0	https://www.ncbi.nlm.nih.gov/clinvar/variation/127418/
NM_000051.3(ATM):c.6108T>C	concordant	unreportable	unreportable	185724	0		3	SCV000682308;S(	Likely benign;Benign;Likely	Color Genomics, In	2017-10-26;2017-1	ATM	chr11	108186750	108186750	http://gnomad.b	0.0005068169667	EAS	0.003251649905	0		1	0	https://www.ncbi.nlm.nih.gov/clinvar/variation/185724/
NM_000051.3(ATM):c.6465G>A	concordant	unreportable	unreportable	184230	0		4	SCV000682340;S(	Likely benign;Uncertain sig	Color Genomics, In	2017-10-26;2017-0	ATM	chr11	108192040	108192040	http://gnomad.b	3.34E-05	EAS	0.0001159960011	0		0	0	https://www.ncbi.nlm.nih.gov/clinvar/variation/184230/
NM_000051.3(ATM):c.6572+12G>T	concordant	unreportable	unreportable	140762	0		2	SCV000682353;S(	Benign;Likely benign	Color Genomics, In	2017-10-26;2016-1	ATM	chr11	108192159	108192159	http://gnomad.b	0.001553867403	AFR	0.04645840079	24		11	0	https://www.ncbi.nlm.nih.gov/clinvar/variation/140762/
NM_000051.3(ATM):c.7004C>T	concordant	unreportable	unreportable	186867	0		3	SCV000292479;S(	Uncertain significance;Unc	GeneDx;Invitae;Lal	2017-11-28;2017-1	ATM	chr11	108198400	108198400	http://gnomad.b	6.40E-05	AFR	0.0006535950233	0		0	0	https://www.ncbi.nlm.nih.gov/clinvar/variation/186867/
NM_000051.3(ATM):c.7062G>A	concordant	unreportable	unreportable	184489	0		2	SCV000682386;S(	Likely benign;Likely benign	Color Genomics, In	2017-10-26;2017-1	ATM	chr11	108198458	108198458	http://gnomad.b	0.0001058189271	NFE	0.000188621998	0		0	0	https://www.ncbi.nlm.nih.gov/clinvar/variation/184489/
NM_000051.3(ATM):c.7174C>T	concordant	unreportable	unreportable	186868	0		4	SCV000682393;S(	Uncertain significance;Unc	Color Genomics, In	2017-10-26;2017-1	ATM	chr11	108199832	108199832	http://gnomad.b	7.52E-05	AFR	0.0006535950233	0		0	0	https://www.ncbi.nlm.nih.gov/clinvar/variation/186868/
NM_000051.3(ATM):c.7515+20A>G	concordant	unreportable	unreportable	136430	0		1	SCV000682420	Likely benign	Color Genomics, In	10/26/2017	ATM	chr11	108201168	108201168	http://gnomad.b	1.11E-05	SAS	6.50E-05	0		0	0	https://www.ncbi.nlm.nih.gov/clinvar/variation/136430/
NM_000051.3(ATM):c.7630-17T>C	concordant	unreportable	unreportable	136431	0		1	SCV000682426	Benign	Color Genomics, In	10/26/2017	ATM	chr11	108202589	108202589	http://gnomad.b	0.0003063179469	AFR	0.009293190204	2		0	0	https://www.ncbi.nlm.nih.gov/clinvar/variation/136431/
NM_000051.3(ATM):c.7788+8G>T	concordant	unreportable	unreportable	127450	0		3	SCV000602565;S(	Likely benign;Benign;Benig	ARUP Laboratories	2017-06-30;2017-1	ATM	chr11	108202772	108202772	http://gnomad.b	0.0007518713242	NFE	0.002754719928	0		0	0	https://www.ncbi.nlm.nih.gov/clinvar/variation/127450/
NM_000051.3(ATM):c.7788+19C>T	concordant	unreportable	unreportable	490711	0		1	SCV000687792	Likely benign	Color Genomics, In	12/21/2017	ATM	chr11	108202783	108202783	http://gnomad.b	1.11E-05	NFE	7.22E-05	0		0	0	https://www.ncbi.nlm.nih.gov/clinvar/variation/490711/
NM_000051.3(ATM):c.7928-10T>C	concordant	unreportable	unreportable	127452	0		4	SCV000149167;S(	Uncertain significance;Unc	GeneDx;Genetic Se	2017-11-28;2017-0	ATM	chr11	108204603	108204603	http://gnomad.b	9.47E-05	AFR	0.001895920024	0		0	0	https://www.ncbi.nlm.nih.gov/clinvar/variation/127452/
NM_000051.3(ATM):c.8229G>A	concordant	unreportable	unreportable	184010	0		2	SCV000682469;S(	Likely benign;Likely benign	Color Genomics, In	2017-10-26;2017-1	ATM	chr11	108206649	108206649	http://gnomad.b	3.06E-05	EAS	5.81E-05	0		0	0	https://www.ncbi.nlm.nih.gov/clinvar/variation/184010/
NM_000051.3(ATM):c.8269-14A>T	concordant	unreportable	unreportable	136432	0		1	SCV000682473	Benign	Color Genomics, In	10/26/2017	ATM	chr11	108213935	108213935	http://gnomad.b	0.0002673320264	AFR	0.006600440014	1		0	0	https://www.ncbi.nlm.nih.gov/clinvar/variation/136432/
NM_000051.3(ATM):c.8786+20G>C	concordant	unreportable	unreportable	379249	0		1	SCV000682517	Likely benign	Color Genomics, In	10/26/2017	ATM	chr11	108224627	108224627	http://gnomad.b	3.34E-05	EAS	0.000173952998	0		0	0	https://www.ncbi.nlm.nih.gov/clinvar/variation/379249/
NM_024685.3(BBS10):c.1276A>G	concordant	unreportable	unreportable	406220	0		1	SCV000544763	Uncertain significance	Invitae	10/5/2017	BBS10	chr12	76740489	76740489	http://gnomad.b	6.71E-05	AFR	0.00124214997	0		0	0	https://www.ncbi.nlm.nih.gov/clinvar/variation/406220/
NM_000277.1(PAH):c.442-18G>A	concordant	unreportable	unreportable	439227	0		1	SCV000601713	Uncertain significance	Quest Diagnostics I	8/1/2017	PAH	chr12	103260459	103260459	http://gnomad.b	7.94E-05	AMR	0.0006564030191	0		0	0	https://www.ncbi.nlm.nih.gov/clinvar/variation/439227/
NM_052845.3(MMAB):c.732G>A	concordant	unreportable	unreportable	307075	0		2	SCV000523264;S(	Likely benign;Uncertain sig	GeneDx;Illumina C	2017-11-28;2016-1	MMAB	chr12	109994854	109994854	http://gnomad.b	9.60E-05	AMR	0.0003275559866	0		0	0	https://www.ncbi.nlm.nih.gov/clinvar/variation/307075/
NM_052845.3(MMAB):c.444G>A	concordant	unreportable	unreportable	287810	0		3	SCV000341718;S(	Uncertain significance;Likel	EGL Genetic Diagn	2017-06-30;2017-1	MMAB	chr12	109999300	109999300	http://gnomad.b	0.0006959777287	EAS	0.006635440048	1		0	0	https://www.ncbi.nlm.nih.gov/clinvar/variation/287810/
NM_052845.3(MMAB):c.394T>C	concordant	unreportable	unreportable	378149	0		1	SCV000513638	Likely benign	GeneDx	11/28/2017	MMAB	chr12	109999612	109999612	http://gnomad.b	0.000943969793	NFE	0.00126573001	1		0	0	https://www.ncbi.nlm.nih.gov/clinvar/variation/378149/
NM_052845.3(MMAB):c.185C>T	concordant	unreportable	unreportable	203817	0		2	SCV000238977;S(	Likely benign;Benign	GeneDx;Invitae	2017-11-28;2017-1	MMAB	chr12	110009465	110009465	http://gnomad.b	0.001999936002	AFR	0.02267380059	6		5	0	https://www.ncbi.nlm.nih.gov/clinvar/variation/203817/
NM_000017.2(ACADS):c.1104C>T	concordant	unreportable	unreportable	307442	0		1	SCV000376687	Uncertain significance	Illumina Clinical Se	10/18/2016	ACADS	chr12	121177116	121177116	http://gnomad.b	0.0001670839044	EAS	0.001456880011	0		1	0	https://www.ncbi.nlm.nih.gov/clinvar/variation/307442/
NM_004004.5(GJB2):c.*114T>C	concordant	unreportable	unreportable	311370	0		1	SCV000382966	Likely benign	Illumina Clinical Se	10/18/2016	GJB2	chr13	20762926	20762926		3.91E-05					0	0	https://www.ncbi.nlm.nih.gov/clinvar/variation/311370/
NM_004004.5(GJB2):c.425T>C	concordant	unreportable	unreportable	496220	0		1	SCV000698256	Uncertain significance	Laboratory Corpor	1/25/2018	GJB2	chr13	20763296	20763296	http://gnomad.b	5.03E-05	AFR	0.0004584749986	0		0	0	https://www.ncbi.nlm.nih.gov/clinvar/variation/496220/
NM_004004.5(GJB2):c.-15C>T	concordant	unreportable	unreportable	94388	0		2	SCV000603816;S(	Benign;Likely benign	ARUP Laboratories	2017-06-30;2016-1	GJB2	chr13	20763735	20763735	http://gnomad.b	0.001673642505	AFR	0.05152549967	18		15	0	https://www.ncbi.nlm.nih.gov/clinvar/variation/94388/
NM_004004.5(GJB2):c.-22-6T>C	concordant	unreportable	unreportable	228700	0		2	SCV000698236;S(	Uncertain significance;Unc	Laboratory Corpor	2018-01-25;2016-0	GJB2	chr13	20763748	20763748	http://gnomad.b	0.000198378327	NFE	0.0009610630223	0		0	0	https://www.ncbi.nlm.nih.gov/clinvar/variation/228700/
NM_014363.4(SACS):c.8836A>G	concordant	unreportable	unreportable	448225	0		1	SCV000614990	Uncertain significance	Athena Diagnostics	8/17/2017	SACS	chr13	23909179	23909179	http://gnomad.b	8.89E-06	NFE	8.99E-06	0		0	0	https://www.ncbi.nlm.nih.gov/clinvar/variation/448225/
NM_014363.4(SACS):c.5583G>C	concordant	unreportable	unreportable	448208	0		1	SCV000614962	Uncertain significance	Athena Diagnostics	8/17/2017	SACS	chr13	23912432	23912432	http://gnomad.b	5.04E-05	AFR	0.0007843140047	0		0	0	https://www.ncbi.nlm.nih.gov/clinvar/variation/448208/
NM_000053.3(ATP7B):c.4395C>A	concordant	unreportable	unreportable	456558	0		1	SCV000626866	Likely benign	Invitae	10/5/2017	ATP7B	chr13	52508895	52508895	http://gnomad.b	3.09E-05	AMR	5.96E-05	0		0	0	https://www.ncbi.nlm.nih.gov/clinvar/variation/456558/
NM_000053.3(ATP7B):c.2355+13T>G	concordant	unreportable	unreportable	157938	0		2	SCV000525798;S(	Likely benign;Benign	GeneDx;Laborator	2017-11-28;2018-0	ATP7B	chr13	52532434	52532434	http://gnomad.b	0.001077465263	FIN	0.006008970086	2		1	0	https://www.ncbi.nlm.nih.gov/clinvar/variation/157938/
NM_000053.3(ATP7B):c.1947-4C>T	concordant	unreportable	unreportable	387748	0		1	SCV000529873	Likely benign	GeneDx	11/28/2017	ATP7B	chr13	52534462	52534462	http://gnomad.b	0.0002918135087	EAS	0.008603650145	2		2	0	https://www.ncbi.nlm.nih.gov/clinvar/variation/387748/
NM_000153.3(GALC):c.328+6A>G	concordant	unreportable	unreportable	314757	0		1	SCV000389263	Uncertain significance	Illumina Clinical Se	10/18/2016	GALC	chr14	88454482	88454482	http://gnomad.b	9.79E-05	AMR	0.0001489069982	1		1	0	https://www.ncbi.nlm.nih.gov/clinvar/variation/314757/
NM_000153.3(GALC):c.195+19G>A	concordant	unreportable	unreportable	255373	0		1	SCV000515895	Likely benign	GeneDx	11/28/2017	GALC	chr14	88459295	88459295	http://gnomad.b	0.0009370210005	NFE	0.002904070076	2		1	0	https://www.ncbi.nlm.nih.gov/clinvar/variation/255373/
NM_000153.3(GALC):c.195+10A>G	concordant	unreportable	unreportable	456716	0		1	SCV000627130	Benign	Invitae	10/5/2017	GALC	chr14	88459304	88459304	http://gnomad.b	0.000665704472	AFR	0.01183969993	0		2	0	https://www.ncbi.nlm.nih.gov/clinvar/variation/456716/
NM_133647.1(SLC12A6):c.3042+8A>G	concordant	unreportable	unreportable	195698	0		1	SCV000390299	Uncertain significance	Illumina Clinical Se	10/18/2016	SLC12A6	chr15	34528901	34528901	http://gnomad.b	0.0009986487127	NFE	0.004012539983	0		0	0	https://www.ncbi.nlm.nih.gov/clinvar/variation/195698/
NM_133647.1(SLC12A6):c.2935-7A>G	concordant	unreportable	unreportable	390124	0		2	SCV000605121;S(	Benign;Benign	ARUP Laboratories	2017-06-30;2017-1	SLC12A6	chr15	34529023	34529023	http://gnomad.b	0.0006845336874	AFR	0.01940670051	1		3	0	https://www.ncbi.nlm.nih.gov/clinvar/variation/390124/
NM_133647.1(SLC12A6):c.1944-26C>T	concordant	unreportable	unreportable	159890	0		1	SCV000194916	Likely benign	Genetic Services La	9/11/2014	SLC12A6	chr15	34536299	34536299	http://gnomad.b	0.01488786686	FIN	0.07411900163	236		108	0	https://www.ncbi.nlm.nih.gov/clinvar/variation/159890/
NM_002225.3(IVD):c.560-18C>T	concordant	unreportable	unreportable	380581	0		1	SCV000518796	Likely benign	GeneDx	11/28/2017	IVD	chr15	40703736	40703736	http://gnomad.b	0.001597238179	AMR	0.00711691007	1		3	0	https://www.ncbi.nlm.nih.gov/clinvar/variation/380581/
NM_000520.4(HEXA):c.1421+14G>C	concordant	unreportable	unreportable	317041	0		1	SCV000393830	Uncertain significance	Illumina Clinical Se	10/18/2016	HEXA	chr15	72638562	72638562	http://gnomad.b	6.34E-05	AMR	0.0006584069924	0		0	0	https://www.ncbi.nlm.nih.gov/clinvar/variation/317041/
NM_000520.4(HEXA):c.673-13T>C	concordant	unreportable	unreportable	317045	0		1	SCV000393837	Uncertain significance	Illumina Clinical Se	10/18/2016	HEXA	chr15	72643004	72643004	http://gnomad.b	0.0004554653494	AFR	0.01208829973	1		2	0	https://www.ncbi.nlm.nih.gov/clinvar/variation/317045/
NM_000520.4(HEXA):c.672+30T>G	concordant	unreportable	unreportable	256351	0		1	SCV000539284	Benign	Laboratory for Mol	4/3/2017	HEXA	chr15	72643444	72643444	http://gnomad.b	0.007679521433	SAS	0.04582170025	88		34	0	https://www.ncbi.nlm.nih.gov/clinvar/variation/256351/
NM_000520.4(HEXA):c.346+13C>T	concordant	unreportable	unreportable	317046	0		1	SCV000393838	Uncertain significance	Illumina Clinical Se	10/18/2016	HEXA	chr15	72648853	72648853	http://gnomad.b	0.0002253849152	AFR	0.003925160039	0		0	0	https://www.ncbi.nlm.nih.gov/clinvar/variation/317046/
NM_000520.4(HEXA):c.-59G>A	concordant	unreportable	unreportable	317050	0		1	SCV000393843	Uncertain significance	Illumina Clinical Se	10/18/2016	HEXA	chr15	72668372	72668372		0.0008663232677					1	0	https://www.ncbi.nlm.nih.gov/clinvar/variation/317050/
NM_000520.4(HEXA):c.-167G>A	concordant	unreportable	unreportable	317053	0		1	SCV000393846	Uncertain significance	Illumina Clinical Se	10/18/2016	HEXA	chr15	72668480	72668480		0.003474684109					36	0	https://www.ncbi.nlm.nih.gov/clinvar/variation/317053/
NM_002435.1(MPI):c.345+16C>T	concordant	unreportable	unreportable	94068	0		1	SCV000520644	Benign	GeneDx	11/28/2017	MPI	chr15	75183936	75183936	http://gnomad.b	1.12E-05	FIN	0.02269059978	41		0	0	https://www.ncbi.nlm.nih.gov/clinvar/variation/94068/
NM_002435.1(MPI):c.670+11T>C	concordant	unreportable	unreportable	386069	0		1	SCV000527587	Likely benign	GeneDx	11/28/2017	MPI	chr15	75185672	75185672	http://gnomad.b	0.0001735508504	NFE	0.0008885340067	0		0	0	https://www.ncbi.nlm.nih.gov/clinvar/variation/386069/
NM_002435.1(MPI):c.671-4T>G	concordant	unreportable	unreportable	389367	0		1	SCV000531862	Likely benign	GeneDx	11/28/2017	MPI	chr15	75188489	75188489	http://gnomad.b	0.0001035706688	ASJ	0.0004065039975	0		0	0	https://www.ncbi.nlm.nih.gov/clinvar/variation/389367/
NM_002435.1(MPI):c.1178G>C	concordant	unreportable	unreportable	317143	0		2	SCV000393955;S(	Uncertain significance;Unc	Illumina Clinical Se	2016-10-18;2017-1	MPI	chr15	75189977	75189977	http://gnomad.b	4.48E-05	AMR	0.001429339987	0		0	0	https://www.ncbi.nlm.nih.gov/clinvar/variation/317143/
NM_000137.2(FAH):c.81+8G>A	concordant	unreportable	unreportable	317202	0		1	SCV000394036	Uncertain significance	Illumina Clinical Se	10/18/2016	FAH	chr15	80445485	80445485	http://gnomad.b	1.66E-05	NFE	8.97E-05	0		0	0	https://www.ncbi.nlm.nih.gov/clinvar/variation/317202/
NM_000137.2(FAH):c.82-19G>A	concordant	unreportable	unreportable	380447	0		1	SCV000518527	Benign	GeneDx	11/28/2017	FAH	chr15	80450383	80450383	http://gnomad.b	0.001814338266	AFR	0.02790120058	10		8	0	https://www.ncbi.nlm.nih.gov/clinvar/variation/380447/
NM_000137.2(FAH):c.412G>A	concordant	unreportable	unreportable	290073	0		1	SCV000344566	Uncertain significance	EGL Genetic Diagn	6/30/2017	FAH	chr15	80454635	80454635	http://gnomad.b	2.22E-05	AFR	0.0003267120046	0		0	0	https://www.ncbi.nlm.nih.gov/clinvar/variation/290073/
NM_000137.2(FAH):c.456-20C>T	concordant	unreportable	unreportable	382956	0		1	SCV000523153	Benign	GeneDx	11/28/2017	FAH	chr15	80460374	80460374	http://gnomad.b	0.001733885957	EAS	0.04777370021	25		11	0	https://www.ncbi.nlm.nih.gov/clinvar/variation/382956/
NM_000137.2(FAH):c.1098G>A	concordant	unreportable	unreportable	498024	0		1	SCV000702832	Likely benign	EGL Genetic Diagn	6/30/2017	FAH	chr15	80473419	80473419	http://gnomad.b	0.0004688427629	AFR	0.00411764998	0		0	0	https://www.ncbi.nlm.nih.gov/clinvar/variation/498024/
NM_000057.2(BLM):c.98C>T	concordant	unreportable	unreportable	133703	0		1	SCV000623360	Uncertain significance	Invitae	10/5/2017	BLM	chr15	91290720	91290720	http://gnomad.b	3.09E-05	AFR	7.26E-05	0		0	0	https://www.ncbi.nlm.nih.gov/clinvar/variation/133703/
NM_000057.2(BLM):c.807C>T	concordant	unreportable	unreportable	413294	0		1	SCV000555832	Likely benign	Invitae	10/5/2017	BLM	chr15	91295024	91295024	http://gnomad.b	0.0001236740713	AFR	0.0001961810049	0		0	0	https://www.ncbi.nlm.nih.gov/clinvar/variation/413294/
NM_000057.2(BLM):c.1601A>G	concordant	unreportable	unreportable	127477	0		2	SCV000231997;S(	Uncertain significance;Likel	EGL Genetic Diagn	2017-06-30;2017-1	BLM	chr15	91304204	91304204	http://gnomad.b	0.0003044284831	AFR	0.00438079983	0		0	0	https://www.ncbi.nlm.nih.gov/clinvar/variation/127477/
NM_000057.2(BLM):c.2293G>A	concordant	unreportable	unreportable	454099	0		1	SCV000623265	Likely benign	Invitae	10/5/2017	BLM	chr15	91310239	91310239	http://gnomad.b	0.0003329686534	EAS	0.004046449903	0		1	0	https://www.ncbi.nlm.nih.gov/clinvar/variation/454099/
NM_000057.2(BLM):c.2475G>A	concordant	unreportable	unreportable	127487	0		1	SCV000623272	Uncertain significance	Invitae	10/5/2017	BLM	chr15	91312736	91312736	http://gnomad.b	4.28E-05	AFR	6.53E-05	0		0	0	https://www.ncbi.nlm.nih.gov/clinvar/variation/127487/
NM_000057.2(BLM):c.2868G>A	concordant	unreportable	unreportable	413288	0		1	SCV000555822	Likely benign	Invitae	3/14/2017	BLM	chr15	91333923	91333923	http://gnomad.b	3.81E-05	AMR	0.0004466940009	0		0	0	https://www.ncbi.nlm.nih.gov/clinvar/variation/413288/
NM_000243.2(MEFV):c.1587+33C>G	concordant	unreportable	unreportable	97892	0		2	SCV000604175;S(	Benign;Uncertain significar	ARUP Laboratories	2017-06-30;2017-0	MEFV	chr16	3296983	3296983	http://gnomad.b	0.0009935628324	AFR	0.01462869998	1		2	0	https://www.ncbi.nlm.nih.gov/clinvar/variation/97892/
NM_000243.2(MEFV):c.1587+18C>T	concordant	unreportable	unreportable	445588	0		1	SCV000696051	Likely benign	Laboratory Corpor	1/25/2018	MEFV	chr16	3296998	3296998	http://gnomad.b	0.0009599776099	AFR	0.03027730063	10		3	0	https://www.ncbi.nlm.nih.gov/clinvar/variation/445588/
NM_000243.2(MEFV):c.1356+31G>T	concordant	unreportable	unreportable	439884	0		1	SCV000604188	Likely benign	ARUP Laboratories	6/30/2017	MEFV	chr16	3298878	3298878	http://gnomad.b	0.0005037783375	AFR	0.01431000046	3		0	0	https://www.ncbi.nlm.nih.gov/clinvar/variation/439884/
NM_000243.2(MEFV):c.1261-11T>G	concordant	unreportable	unreportable	97438	0		2	SCV000513591;S(	Likely benign;Uncertain sig	GeneDx;Illumina C	2017-11-28;2016-1	MEFV	chr16	3299015	3299015	http://gnomad.b	0.0009627763784	AFR	0.02830439992	6		5	0	https://www.ncbi.nlm.nih.gov/clinvar/variation/97438/
NM_000303.2(PMM2):c.-15C>G	concordant	unreportable	unreportable	386292	0		1	SCV000527868	Likely benign	GeneDx	11/28/2017	PMM2	chr16	8891725	8891725	http://gnomad.b	3.62E-05	AFR	0.0009845290333	0		0	0	https://www.ncbi.nlm.nih.gov/clinvar/variation/386292/
NM_000303.2(PMM2):c.*10G>A	concordant	unreportable	unreportable	321222	0		2	SCV000533071;S(	Likely benign;Uncertain sig	GeneDx;Illumina C	2017-11-28;2016-1	PMM2	chr16	8941692	8941692	http://gnomad.b	0.0001308528824	NFE	0.0003962040064	0		0	0	https://www.ncbi.nlm.nih.gov/clinvar/variation/321222/
NM_000303.2(PMM2):c.*17G>A	concordant	unreportable	unreportable	321223	0		2	SCV000523179;S(	Likely benign;Uncertain sig	GeneDx;Illumina C	2017-11-28;2016-1	PMM2	chr16	8941699	8941699	http://gnomad.b	0.0009187542806	AFR	0.03149709851	3		3	0	https://www.ncbi.nlm.nih.gov/clinvar/variation/321223/
NM_000303.2(PMM2):c.*18C>T	concordant	unreportable	unreportable	389416	0		1	SCV000531935	Likely benign	GeneDx	11/28/2017	PMM2	chr16	8941700	8941700	http://gnomad.b	8.35E-06	SAS	0.0002277610038	0		0	0	https://www.ncbi.nlm.nih.gov/clinvar/variation/389416/
NM_001042432.1(CLN3):c.1057-11C>T	concordant	unreportable	unreportable	377686	0		1	SCV000512635	Likely benign	GeneDx	11/28/2017	CLN3	chr16	28489209	28489209	http://gnomad.b	0.000111108642	EAS	0.001339699957	0		0	0	https://www.ncbi.nlm.nih.gov/clinvar/variation/377686/
NM_000018.3(ACADVL):c.623-8C>T	concordant	unreportable	unreportable	166642	0		3	SCV000602372;S(	Benign;Uncertain significar	ARUP Laboratories	2017-06-30;2016-1	ACADVL	chr17	7125263	7125263	http://gnomad.b	0.0009627909611	AFR	0.02791940048	7		3	0	https://www.ncbi.nlm.nih.gov/clinvar/variation/166642/
NM_000018.3(ACADVL):c.1182+17C>A	concordant	unreportable	unreportable	380058	0		1	SCV000517741	Likely benign	GeneDx	11/28/2017	ACADVL	chr17	7126573	7126573	http://gnomad.b	4.81E-05	AFR	0.0001307019993	0		0	0	https://www.ncbi.nlm.nih.gov/clinvar/variation/380058/
NM_000018.3(ACADVL):c.1820G>C	concordant	unreportable	unreportable	474892	0		1	SCV000654942	Uncertain significance	Invitae	10/5/2017	ACADVL	chr17	7128196	7128196	http://gnomad.b	3.96E-05	EAS	0.0004058910126	0		0	0	https://www.ncbi.nlm.nih.gov/clinvar/variation/474892/
NM_000382.2(ALDH3A2):c.386-6A>G	concordant	unreportable	unreportable	322208	0		1	SCV000401250	Likely benign	Illumina Clinical Se	10/18/2016	ALDH3A2	chr17	19555854	19555854	http://gnomad.b	0.005606969101	AFR	0.05416880175	31		22	0	https://www.ncbi.nlm.nih.gov/clinvar/variation/322208/
NM_000151.3(G6PC):c.562+10G>A	concordant	unreportable	unreportable	289099	0		1	SCV000343384	Uncertain significance	EGL Genetic Diagn	6/30/2017	G6PC	chr17	41061445	41061445	http://gnomad.b	0.0001226761809	AFR	0.002483010059	0		1	0	https://www.ncbi.nlm.nih.gov/clinvar/variation/289099/
NM_000023.2(SGCA):c.-18G>A	concordant	unreportable	unreportable	324039	0		1	SCV000403941	Uncertain significance	Illumina Clinical Se	10/18/2016	SGCA	chr17	48243384	48243384	http://gnomad.b	5.60E-06	NFE	3.67E-05	0		0	0	https://www.ncbi.nlm.nih.gov/clinvar/variation/324039/
NM_000023.2(SGCA):c.-8G>A	concordant	unreportable	unreportable	288271	0		3	SCV000342336;S(	Benign;Benign;Likely benig	EGL Genetic Diagn	2017-06-30;2017-1	SGCA	chr17	48243394	48243394	http://gnomad.b	0.001161410932	AFR	0.03258160129	8		9	0	https://www.ncbi.nlm.nih.gov/clinvar/variation/288271/
NM_000023.2(SGCA):c.62C>T	concordant	unreportable	unreportable	254720	0		1	SCV000343965	Uncertain significance	EGL Genetic Diagn	6/30/2017	SGCA	chr17	48244753	48244753	http://gnomad.b	4.48E-05	NFE	0.0001702599984	0		0	0	https://www.ncbi.nlm.nih.gov/clinvar/variation/254720/
NM_000023.2(SGCA):c.502G>A	concordant	unreportable	unreportable	498275	0		1	SCV000703211	Uncertain significance	EGL Genetic Diagn	6/30/2017	SGCA	chr17	48245851	48245851		1.68E-05					0	0	https://www.ncbi.nlm.nih.gov/clinvar/variation/498275/
NM_000023.2(SGCA):c.555C>A	concordant	unreportable	unreportable	289773	0		1	SCV000344185	Uncertain significance	EGL Genetic Diagn	6/30/2017	SGCA	chr17	48245904	48245904	http://gnomad.b	8.68E-05	SAS	0.0004086639965	0		0	0	https://www.ncbi.nlm.nih.gov/clinvar/variation/289773/

Supplemental Table 2

NM_000023.2(SGCA):c.582A>C	concordant	unreportable	unreportable	501744	0	1	SCV000708228	Uncertain significance	EGL Genetic Diagn	6/30/2017	SGCA	chr17	48245931	48245931	http://gnomad.b	1.68E-05	AMR	0.0001203759966	0		0	0	https://www.ncbi.nlm.nih.gov/clinvar/variation/501744/
NM_000023.2(SGCA):c.764C>T	concordant	unreportable	unreportable	285931	0	2	SCV000339155;S	Uncertain significance;Unc	EGL Genetic Diagn	2017-06-30;2016-1	SGCA	chr17	48247520	48247520	http://gnomad.b	8.40E-05	AMR	0.00148916	1		0	0	https://www.ncbi.nlm.nih.gov/clinvar/variation/285931/
NM_000023.2(SGCA):c.1155C>T	concordant	unreportable	unreportable	471331	0	1	SCV000649762	Likely benign	Invitae	10/5/2017	SGCA	chr17	48252789	48252789	http://gnomad.b	2.80E-06	AFR	6.74E-05	0		0	0	https://www.ncbi.nlm.nih.gov/clinvar/variation/471331/
NM_000023.2(SGCA):c.*10C>G	concordant	unreportable	unreportable	324046	0	2	SCV000525521;S	Benign;Uncertain significar	GeneDx;Illumina C	2017-11-28;2016-1	SGCA	chr17	48252808	48252808	http://gnomad.b	3.36E-05	AFR	0.01967060007	2		0	0	https://www.ncbi.nlm.nih.gov/clinvar/variation/324046/
NM_000154.1(GALK1):c.1119C>T	concordant	unreportable	unreportable	459629	0	1	SCV000631401	Benign	Invitae	10/5/2017	GALK1	chr17	73754197	73754197	http://gnomad.b	0.003301917035	ASJ	0.01652299985	4		3	0	https://www.ncbi.nlm.nih.gov/clinvar/variation/459629/
NM_000154.1(GALK1):c.1089C>T	concordant	unreportable	unreportable	459628	0	1	SCV000631400	Likely benign	Invitae	10/5/2017	GALK1	chr17	73754309	73754309	http://gnomad.b	8.01E-06	SAS	0.0002044290013	0		0	0	https://www.ncbi.nlm.nih.gov/clinvar/variation/459628/
NM_000154.1(GALK1):c.957G>A	concordant	unreportable	unreportable	459630	0	1	SCV000631403	Benign	Invitae	10/5/2017	GALK1	chr17	73754441	73754441	http://gnomad.b	0.001234211707	AFR	0.01317590009	1		0	0	https://www.ncbi.nlm.nih.gov/clinvar/variation/459630/
NM_000154.1(GALK1):c.611+34G>A	concordant	unreportable	unreportable	496799	0	1	SCV000700291	Benign	EGL Genetic Diagn	6/30/2017	GALK1	chr17	73759061	73759061	http://gnomad.b	0.01989164583	NFE	0.02900220081	73		39	0	https://www.ncbi.nlm.nih.gov/clinvar/variation/496799/
NM_000152.3(GAA):c.197G>A	concordant	unreportable	unreportable	325775	0	2	SCV000407255;S	Uncertain significance;Unc	Illumina Clinical Se	2016-10-18;2017-1	GAA	chr17	78078582	78078582	http://gnomad.b	7.50E-05	EAS	0.0002354049939	0		0	0	https://www.ncbi.nlm.nih.gov/clinvar/variation/325775/
NM_000152.3(GAA):c.546+18G>A	concordant	unreportable	unreportable	281750	0	2	SCV000332713;S	Benign;Likely benign	EGL Genetic Diagn	2017-06-30;2017-1	GAA	chr17	78078949	78078949	http://gnomad.b	0.0004974958449	AFR	0.01601240039	0		0	0	https://www.ncbi.nlm.nih.gov/clinvar/variation/281750/
NM_000152.3(GAA):c.600C>T	concordant	unreportable	unreportable	255364	0	2	SCV000337289;S	Uncertain significance;Likel	EGL Genetic Diagn	2017-06-30;2017-1	GAA	chr17	78079601	78079601	http://gnomad.b	2.78E-06	AFR	0.001375250053	0		0	0	https://www.ncbi.nlm.nih.gov/clinvar/variation/255364/
NM_000152.3(GAA):c.1438-9G>A	concordant	unreportable	unreportable	456379	0	2	SCV000708267;S	Uncertain significance;Likel	EGL Genetic Diagn	2017-06-30;2017-1	GAA	chr17	78084517	78084517	http://gnomad.b	6.39E-05	AFR	0.002360040089	0		0	0	https://www.ncbi.nlm.nih.gov/clinvar/variation/456379/
NM_000152.3(GAA):c.1560C>T	concordant	unreportable	unreportable	290162	0	1	SCV000344666	Uncertain significance	EGL Genetic Diagn	6/30/2017	GAA	chr17	78084748	78084748	http://gnomad.b	2.50E-05	AFR	6.54E-05	0		0	0	https://www.ncbi.nlm.nih.gov/clinvar/variation/290162/
NM_000152.3(GAA):c.1593C>T	concordant	unreportable	unreportable	498567	0	1	SCV000703642	Uncertain significance	EGL Genetic Diagn	6/30/2017	GAA	chr17	78084781	78084781	http://gnomad.b	0.0001806549158	NFE	0.0001702169975	0		0	0	https://www.ncbi.nlm.nih.gov/clinvar/variation/498567/
NM_000152.3(GAA):c.1599C>T	concordant	unreportable	unreportable	255354	0	2	SCV000337287;S	Uncertain significance;Likel	EGL Genetic Diagn	2017-06-30;2017-1	GAA	chr17	78084787	78084787	http://gnomad.b	7.50E-05	AFR	0.0009153920109	0		0	0	https://www.ncbi.nlm.nih.gov/clinvar/variation/255354/
NM_000152.3(GAA):c.1755-18T>C	concordant	unreportable	unreportable	380511	0	1	SCV000518675	Likely benign	GeneDx	11/28/2017	GAA	chr17	78086359	78086359	http://gnomad.b	0.0002834892524	AMR	0.001700980007	1		0	0	https://www.ncbi.nlm.nih.gov/clinvar/variation/380511/
NM_000152.3(GAA):c.1758G>A	concordant	unreportable	unreportable	391582	0	2	SCV000534689;S	Likely benign;Benign	GeneDx;Invitae	2017-11-28;2017-1	GAA	chr17	78086380	78086380	http://gnomad.b	0.0005447440537	EAS	0.004892250057	1		1	0	https://www.ncbi.nlm.nih.gov/clinvar/variation/391582/
NM_000152.3(GAA):c.2245G>T	concordant	unreportable	unreportable	456396	0	1	SCV000626555	Uncertain significance	Invitae	10/5/2017	GAA	chr17	78090822	78090822	http://gnomad.b	3.61E-05	AFR	0.0005236970028	0		0	0	https://www.ncbi.nlm.nih.gov/clinvar/variation/456396/
NM_000152.3(GAA):c.2323C>A	concordant	unreportable	unreportable	281240	0	2	SCV000331863;S	Uncertain significance;Unc	EGL Genetic Diagn	2017-06-30;2017-1	GAA	chr17	78090900	78090900	http://gnomad.b	7.50E-05	AFR	0.000921052997	0		0	0	https://www.ncbi.nlm.nih.gov/clinvar/variation/281240/
NM_000152.3(GAA):c.2332-12A>T	concordant	unreportable	unreportable	92475	0	1	SCV000530270	Likely benign	GeneDx	11/28/2017	GAA	chr17	78091387	78091387	http://gnomad.b	0.0001528618518	AFR	0.00573273981	0		0	0	https://www.ncbi.nlm.nih.gov/clinvar/variation/92475/
NM_000152.3(GAA):c.2647-8C>T	concordant	unreportable	unreportable	282362	0	3	SCV000333790;S	Likely benign;Likely benign	EGL Genetic Diagn	2017-06-30;2017-1	GAA	chr17	78092444	78092444	http://gnomad.b	0.0002167858989	AFR	0.008102459833	0		1	0	https://www.ncbi.nlm.nih.gov/clinvar/variation/282362/
NM_000271.4(NPC1):c.3477+4A>G	concordant	unreportable	unreportable	284996	0	1	SCV000337830	Benign	EGL Genetic Diagn	6/30/2017	NPC1	chr18	21115429	21115429	http://gnomad.b	0.000320230566	AFR	0.009014589712	1		0	0	https://www.ncbi.nlm.nih.gov/clinvar/variation/284996/
NM_000271.4(NPC1):c.3432C>G	concordant	unreportable	unreportable	498610	0	1	SCV000703713	Uncertain significance	EGL Genetic Diagn	6/30/2017	NPC1	chr18	21115478	21115478	http://gnomad.b	8.58E-06	AMR	5.96E-05	0		0	0	https://www.ncbi.nlm.nih.gov/clinvar/variation/498610/
NM_000271.4(NPC1):c.2514+6C>T	concordant	unreportable	unreportable	92707	0	2	SCV000407861;S	Uncertain significance;Benign	Illumina Clinical Se	2016-10-18;2017-1	NPC1	chr18	21121026	21121026	http://gnomad.b	0.001612589636	AFR	0.05404520035	22		10	0	https://www.ncbi.nlm.nih.gov/clinvar/variation/92707/
NM_000271.4(NPC1):c.631+17C>T	concordant	unreportable	unreportable	385613	0	1	SCV000526937	Likely benign	GeneDx	11/28/2017	NPC1	chr18	21141307	21141307	http://gnomad.b	0.0001086496563	AFR	0.00294041005	0		0	0	https://www.ncbi.nlm.nih.gov/clinvar/variation/385613/
NM_000271.4(NPC1):c.544G>A	concordant	unreportable	unreportable	502363	0	1	SCV000709059	Uncertain significance	EGL Genetic Diagn	6/30/2017	NPC1	chr18	21141411	21141411	http://gnomad.b	2.86E-05	AFR	0.0005880820099	0		0	0	https://www.ncbi.nlm.nih.gov/clinvar/variation/502363/
NM_000227.3(LAMA3):c.396-13G>C	concordant	unreportable	unreportable	326296	0	1	SCV000407913	Uncertain significance	Illumina Clinical Se	10/18/2016	LAMA3	chr18	21464724	21464724	http://gnomad.b	0.0002631711564	FIN	0.005605880171	0		1	0	https://www.ncbi.nlm.nih.gov/clinvar/variation/326296/
NM_000227.3(LAMA3):c.2692G>A	concordant	unreportable	unreportable	326318	0	1	SCV000407964	Uncertain significance	Illumina Clinical Se	10/18/2016	LAMA3	chr18	21494699	21494699	http://gnomad.b	4.20E-05	EAS	0.0001742769964	0		0	0	https://www.ncbi.nlm.nih.gov/clinvar/variation/326318/
NM_000227.3(LAMA3):c.3377G>A	concordant	unreportable	unreportable	326328	0	1	SCV000407984	Uncertain significance	Illumina Clinical Se	10/18/2016	LAMA3	chr18	21508113	21508113	http://gnomad.b	1.68E-05	NFE	1.79E-05	0		0	0	https://www.ncbi.nlm.nih.gov/clinvar/variation/326328/
NM_000227.3(LAMA3):c.4816-14C>T	concordant	unreportable	unreportable	326347	0	1	SCV000408035	Likely benign	Illumina Clinical Se	10/18/2016	LAMA3	chr18	21531634	21531634	http://gnomad.b	0.004585897386	SAS	0.03567219898	31		18	0	https://www.ncbi.nlm.nih.gov/clinvar/variation/326347/
NM_020533.2(MCOLN1):c.783G>A	concordant	unreportable	unreportable	288961	0	1	SCV000343222	Uncertain significance	EGL Genetic Diagn	6/30/2017	MCOLN1	chr19	7593049	7593049	http://gnomad.b	0.0001308315176	NFE	0.0001163959969	0		0	0	https://www.ncbi.nlm.nih.gov/clinvar/variation/288961/
NM_020533.2(MCOLN1):c.1359+11C>	concordant	unreportable	unreportable	330502	0	1	SCV000415620	Uncertain significance	Illumina Clinical Se	10/18/2016	MCOLN1	chr19	7594609	7594609	http://gnomad.b	0.0003449194554	AFR	0.002940790029	0		0	0	https://www.ncbi.nlm.nih.gov/clinvar/variation/330502/
NM_000528.3(MAN2B1):c.2785C>T	concordant	unreportable	unreportable	328254	0	1	SCV000410774	Uncertain significance	Illumina Clinical Se	10/18/2016	MAN2B1	chr19	12758292	12758292	http://gnomad.b	3.10E-05	AFR	0.0001306850027	0		0	0	https://www.ncbi.nlm.nih.gov/clinvar/variation/328254/
NM_000528.3(MAN2B1):c.1935C>T	concordant	unreportable	unreportable	328264	0	1	SCV000410787	Likely benign	Illumina Clinical Se	10/18/2016	MAN2B1	chr19	12763078	12763078	http://gnomad.b	0.001211281254	AFR	0.03744120151	15		5	0	https://www.ncbi.nlm.nih.gov/clinvar/variation/328264/
NM_000528.3(MAN2B1):c.1928+10G>	concordant	unreportable	unreportable	286664	0	1	SCV000340193	Uncertain significance	EGL Genetic Diagn	6/30/2017	MAN2B1	chr19	12763167	12763167	http://gnomad.b	9.30E-05	NFE	0.0003043700126	0		0	0	https://www.ncbi.nlm.nih.gov/clinvar/variation/286664/
NM_000159.2(GCDH):c.428T>C	concordant	unreportable	unreportable	92531	0	1	SCV000631937	Uncertain significance	Invitae	10/5/2017	GCDH	chr19	13004390	13004390	http://gnomad.b	2.81E-05	AFR	6.54E-05	0		0	0	https://www.ncbi.nlm.nih.gov/clinvar/variation/92531/
NM_004646.3(NPHS1):c.3595-9G>T	concordant	unreportable	unreportable	259501	0	1	SCV000411548	Likely benign	Illumina Clinical Se	10/18/2016	NPHS1	chr19	36317556	36317556	http://gnomad.b	0.01506949361	FIN	0.1114950031	342		112	0	https://www.ncbi.nlm.nih.gov/clinvar/variation/259501/
NM_004646.3(NPHS1):c.1930+10C>T	concordant	unreportable	unreportable	259485	0	1	SCV000411565	Uncertain significance	Illumina Clinical Se	10/18/2016	NPHS1	chr19	36336260	36336260	http://gnomad.b	0.004638482681	ASJ	0.02163749933	24		16	0	https://www.ncbi.nlm.nih.gov/clinvar/variation/259485/
NM_004646.3(NPHS1):c.1758-11C>G	concordant	unreportable	unreportable	328868	0	2	SCV000411571;S	Uncertain significance;Beni	Illumina Clinical Se	2016-10-18;2018-0	NPHS1	chr19	36336453	36336453	http://gnomad.b	0.001926196834	FIN	0.03669409826	13		4	0	https://www.ncbi.nlm.nih.gov/clinvar/variation/328868/
NM_004646.3(NPHS1):c.1170+8G>A	concordant	unreportable	unreportable	259480	0	1	SCV000411577	Uncertain significance	Illumina Clinical Se	10/18/2016	NPHS1	chr19	36339531	36339531	http://gnomad.b	0.001510851931	NFE	0.007220800035	4		0	0	https://www.ncbi.nlm.nih.gov/clinvar/variation/259480/
NM_004646.3(NPHS1):c.43G>C	concordant	unreportable	unreportable	259502	0	1	SCV000614339	Uncertain significance	Athena Diagnostics	8/17/2017	NPHS1	chr19	36342697	36342697	http://gnomad.b	0.0005435721892	AFR	0.01567639969	2		0	0	https://www.ncbi.nlm.nih.gov/clinvar/variation/259502/
NM_000709.3(BCKDHA):c.-28C>G	concordant	unreportable	unreportable	386052	0	1	SCV000527563	Benign	GeneDx	11/28/2017	BCKDHA	chr19	41903705	41903705	http://gnomad.b	0.001582053189	AFR	0.01778890006	4		0	0	https://www.ncbi.nlm.nih.gov/clinvar/variation/386052/
NM_000709.3(BCKDHA):c.15C>T	concordant	unreportable	unreportable	385661	0	1	SCV000526999	Likely benign	GeneDx	11/28/2017	BCKDHA	chr19	41903747	41903747	http://gnomad.b	0.001368476008	SAS	0.004872980062	1		1	0	https://www.ncbi.nlm.nih.gov/clinvar/variation/385661/
NM_000709.3(BCKDHA):c.34C>A	concordant	unreportable	unreportable	93355	0	2	SCV000150426;S	Likely benign;Benign	Genetic Services La	2014-06-27;2017-1	BCKDHA	chr19	41903766	41903766	http://gnomad.b	0.01097153886	ASJ	0.01686649956	19		16	0	https://www.ncbi.nlm.nih.gov/clinvar/variation/93355/
NM_000709.3(BCKDHA):c.288C>T	concordant	unreportable	unreportable	93353	0	2	SCV000529023;S	Likely benign;Uncertain sig	GeneDx;Illumina C	2017-11-28;2016-1	BCKDHA	chr19	41916721	41916721	http://gnomad.b	0.0004508851588	NFE	0.0005194899859	0		0	0	https://www.ncbi.nlm.nih.gov/clinvar/variation/93353/
NM_000709.3(BCKDHA):c.349C>T	concordant	unreportable	unreportable	457143	0	1	SCV000627800	Uncertain significance	Invitae	10/5/2017	BCKDHA	chr19	41916888	41916888	http://gnomad.b	8.70E-05	AFR	0.0001960270019	0		0	0	https://www.ncbi.nlm.nih.gov/clinvar/variation/457143/
NM_000709.3(BCKDHA):c.484+5G>A	concordant	unreportable	unreportable	93362	0	4	SCV000111114;S	Benign;Benign;Likely benig	EGL Genetic Diagn	2017-06-30;2017-1	BCKDHA	chr19	41920067	41920067	http://gnomad.b	0.003195747441	AMR	0.04548440129	46		4	0	https://www.ncbi.nlm.nih.gov/clinvar/variation/93362/
NM_000709.3(BCKDHA):c.648G>T	concordant	unreportable	unreportable	329364	0	2	SCV000512252;S	Likely benign;Uncertain sig	GeneDx;Illumina C	2017-11-28;2016-1	BCKDHA	chr19	41928070	41928070	http://gnomad.b	0.001044155104	AFR	0.01437660027	2		3	0	https://www.ncbi.nlm.nih.gov/clinvar/variation/329364/
NM_000709.3(BCKDHA):c.708C>T	concordant	unreportable	unreportable	93369	0	2	SCV000527203;S	Likely benign;Uncertain sig	GeneDx;Illumina C	2017-11-28;2016-1	BCKDHA	chr19	41928130	41928130	http://gnomad.b	0.001059976536	NFE	0.001594500034	0		0	0	https://www.ncbi.nlm.nih.gov/clinvar/variation/93369/
NM_000709.3(BCKDHA):c.840C>T	concordant	unreportable	unreportable	381845	0	1	SCV000521521	Likely benign	GeneDx	11/28/2017	BCKDHA	chr19	41928262	41928262	http://gnomad.b	0.0004350646269	AFR	0.003660130082	0		0	0	https://www.ncbi.nlm.nih.gov/clinvar/variation/381845/
NM_000709.3(BCKDHA):c.975C>T	concordant	unreportable	unreportable	93383	0	1	SCV000521820	Likely benign	GeneDx	11/28/2017	BCKDHA	chr19	41928655	41928655	http://gnomad.b	0.003702004461	NFE	0.006161100231	1		4	0	https://www.ncbi.nlm.nih.gov/clinvar/variation/93383/
NM_000709.3(BCKDHA):c.1192G>A	concordant	unreportable	unreportable	329368	0	1	SCV000413358	Uncertain significance	Illumina Clinical Se	10/18/2016	BCKDHA	chr19	41930367	41930367	http://gnomad.b	0.001028334573	EAS	0.0003478660074	0		0	0	https://www.ncbi.nlm.nih.gov/clinvar/variation/329368/
NM_000709.3(BCKDHA):c.1211A>G	concordant	unreportable	unreportable	283770	0	1	SCV000336079	Benign	EGL Genetic Diagn	6/30/2017	BCKDHA	chr19	41930386	41930386	http://gnomad.b	0.001028334573	EAS	0.01113170013	1		1	0	https://www.ncbi.nlm.nih.gov/clinvar/variation/283770/
NM_025136.3(OPA3):c.-38A>G	concordant	unreportable	unreportable	138572	0	2	SCV000700316;S	Benign;Likely benign	EGL Genetic Diagn	2017-06-30;2016-1	OPA3	chr19	46088060	46088060	http://gnomad.b	0.00833505123	FIN	0.05464969948	106		34	0	https://www.ncbi.nlm.nih.gov/clinvar/variation/138572/
NM_000022.2(ADA):c.927G>A	concordant	unreportable	unreportable	338504	0	1	SCV000434052	Uncertain significance	Illumina Clinical Se	10/18/2016	ADA	chr20	43249707	43249707	http://gnomad.b	0.0001840530073	EAS	0.001391469967	0		0	0	https://www.ncbi.nlm.nih.gov/clinvar/variation/338504/
NM_000022.2(ADA):c.834T>C	concordant	unreportable	unreportable	338505	0	2	SCV000434053;S	Uncertain significance;Beni	Illumina Clinical Se	2016-10-18;2017-1	ADA	chr20	43251240	43251240	http://gnomad.b	0.005257514164	EAS	0.02527830005	16		8	0	https://www.ncbi.nlm.nih.gov/clinvar/variation/338505/
NM_000022.2(ADA):c.162G>A	concordant	unreportable	unreportable	338510	0	2	SCV000434061;S	Uncertain significance;Beni	Illumina Clinical Se	2016-10-18;2017-1	ADA	chr20	43257744	43257744	http://gnomad.b	0.002712781281	FIN	0.007714389823	3		1	0	https://www.ncbi.nlm.nih.gov/clinvar/variation/338510/
NM_000022.2(ADA):c.95+9A>G	concordant	unreportable	unreportable	468282	0	1	SCV000644845	Benign	Invitae	10/5/2017	ADA	chr20	43264859	43264859	http://gnomad.b	0.000856246599	AFR	0.01033519953	0		0	0	https://www.ncbi.nlm.nih.gov/clinvar/variation/468282/
NM_000411.6(HLCS):c.2174G>A	concordant	unreportable	unreportable	137547	0	1	SCV000436039	Uncertain significance	Illumina Clinical Se	10/18/2016	HLCS	chr21	38126554	38126554	http://gnomad.b	0.000458317252	SAS	0.0005200550077	0		0	0	https://www.ncbi.nlm.nih.gov/clinvar/variation/137547/
NM_000411.6(HLCS):c.1921G>A	concordant	unreportable	unreportable	203755	0	1	SCV000631923	Benign	Invitae	10/5/2017	HLCS	chr21	38128931	38128931	http://gnomad.b	0.003352952528	FIN	0.01336320024	5		1	0	https://www.ncbi.nlm.nih.gov/clinvar/variation/203755/
NM_000411.6(HLCS):c.1920C>T	concordant	unreportable	unreportable	137546	0	2	SCV000436042;S	Likely benign;Benign	Illumina Clinical Se	2016-10-18;2017-1	HLCS	chr21	38128932	38128932	http://gnomad.b	0.01021163	AFR	0.0505749993	39		30	0	https://www.ncbi.nlm.nih.gov/clinvar/variation/137546/
NM_000411.6(HLCS):c.1710C>T	concordant	unreportable	unreportable	137545	0	1	SCV000631922	Benign	Invitae	10/5/2017	HLCS	chr21	38132113	38132113	http://gnomad.b	0.000546764441	EAS	0.004058440216	0		0	0	https://www.ncbi.nlm.nih.gov/clinvar/variation/137545/
NM_000411.6(HLCS):c.1672G>A	concordant	unreportable	unreportable	203754	0	2	SCV000238902;S	Likely benign;Likely benign	GeneDx;Laborator	2017-07-13;2017-0	HLCS	chr21	38137321	38137321	http://gnomad.b	0.004374115528	ASJ	0.02395940013	8		5	0	https://www.ncbi.nlm.nih.gov/clinvar/variation/203754/
NM_000411.6(HLCS):c.1452-6C>T	concordant	unreportable	unreportable	379867	0	1	SCV000517346	Likely benign	GeneDx	11/28/2017	HLCS	chr21	38139592	38139592	http://gnomad.b	0.0006593335906	AFR	0.009539989755	0		0	0	https://www.ncbi.nlm.nih.gov/clinvar/variation/379867/
NM_000411.6(HLCS):c.1254C>T	concordant	unreportable	unreportable	339963	0	1	SCV000436047	Uncertain significance	Illumina Clinical Se	10/18/2016	HLCS	chr21	38269357	38269357	http://gnomad.b	0.0001366911103	AFR	0.003790349932	0		1	0	https://www.ncbi.nlm.nih.gov/clinvar/variation/339963/
NM_000411.6(HLCS):c.981A>G	concordant	unreportable	unreportable	391042	0	1	SCV000534014	Likely benign	GeneDx	11/28/2017	HLCS	chr21	38308764	38308764	http://gnomad.b	0.0001929756851	AMR	0.001161340042	0		1	0	https://www.ncbi.nlm.nih.gov/clinvar/variation/391042/
NM_000411.6(HLCS):c.901G>A	concordant	unreportable	unreportable	203766	0	1	SCV000238914	Likely benign	GeneDx	11/28/2017	HLCS	chr21	38308844	38308844	http://gnomad.b	0.0004020326772	FIN	0.004036239814	1		0	0	https://www.ncbi.nlm.nih.gov/clinvar/variation/203766/
NM_000411.6(HLCS):c.633G>A	concordant	unreportable	unreportable	459945	0	1	SCV000631926	Benign	Invitae	10/5/2017	HLCS	chr21	38309112	38309112	http://gnomad.b	0.002999163772	NFE	0.003830350004	1		1	0	https://www.ncbi.nlm.nih.gov/clinvar/variation/459945/
NM_000411.6(HLCS):c.473C>T	concordant	unreportable	unreportable	203760	0	1	SCV000238908	Likely benign	GeneDx	11/28/2017	HLCS	chr21	38309272	38309272	http://gnomad.b	0.000723658819	AFR	0.008954250254	1		0	0	https://www.ncbi.nlm.nih.gov/clinvar/variation/203760/
NM_000411.6(HLCS):c.424G>A	concordant	unreportable	unreportable	339966	0	2	SCV000436054;S	Uncertain significance;Unc	Illumina Clinical Se	2016-10-18;2017-0	HLCS	chr21	38309321	38309321	http://gnomad.b	0.003151936189	ASJ	0.02548220009	4		4	0	https://www.ncbi.nlm.nih.gov/clinvar/variation/339966/
NM_000411.6(HLCS):c.285C>T	concordant	unreportable	unreportable	137549	0	1	SCV000631925	Benign	Invitae	10/5/2017	HLCS	chr21	38309460	38309460	http://gnomad.b	0.002331789528	AFR	0.0183636006	4		2	0	https://www.ncbi.nlm.nih.gov/clinvar/variation/137549/
NM_000071.2(CBS):c.1359-14C>T	concordant	unreportable	unreportable	212832	0	1	SCV000436205	Uncertain significance	Illumina Clinical Se	10/18/2016	CBS	chr21	44478377	44478377	http://gnomad.b	0.0001687203685	AFR	0.004787509795	0		1	0	https://www.ncbi.nlm.nih.gov/clinvar/variation/212832/
NM_000071.2(CBS):c.1145+7C>T	concordant	unreportable	unreportable	136674	0	3	SCV000602920;S	Benign;Likely benign;Benig	ARUP Laboratories	2017-06-30;2016-1	CBS	chr21	44480544	44480544	http://gnomad.b	0.001830615998	FIN	0.02806100063	19		6	0	https://www.ncbi.nlm.nih.gov/clinvar/variation/136674/
NM_000071.2(CBS):c.954+8G>A	concordant	unreportable	unreportable	136672	0	2	SCV000436212;S	Uncertain significance;Beni	Illumina Clinical Se	2016-10-18;2017-1	CBS	chr21	44483055	44483055	http://gnomad.b	0.0002446445343	AFR	0.00771543989	0		1	0	https://www.ncbi.nlm.nih.gov/clinvar/variation/136672/
NM_000071.2(CBS):c.829-12C>T	concordant	unreportable	unreportable	136671	0	1	SCV000436217	Uncertain significance	Illumina Clinical Se	10/18/2016	CBS	chr21	44483200	44483200	http://gnomad.b	0.002131500655	EAS	0.0298759006	24		4	0	https://www.ncbi.nlm.nih.gov/clinvar/variation/136671/
NM_000071.2(CBS):c.469G>A	concordant	unreportable	unreportable	263924	0	2	SCV000319464;S	Uncertain significance;Unc	Ambry Genetics;In	2017-11-14;2017-1	CBS	chr21	44485788	44485788		3.66E-05					0	0	https://www.ncbi.nlm.nih.gov/clinvar/variation/263924/
NM_000071.2(CBS):c.400G>A	concordant	unreportable	unreportable	212879	0	2	SCV000249728;S	Uncertain significance;Unc	GeneDx;Invitae	2017-11-28;2017-0	CBS	chr21	44486404	44486404	http://gnomad.b	0.0001040442272	EAS	0.000580316002	0		0	0	https://www.ncbi.nlm.nih.gov/clinvar/variation/212879/
NM_000383.3(AIRE):c.1096-9G>C	concordant	unreportable	unreportable	128330	0	1	SCV000629940	Benign	Invitae	10/5/2017	AIRE	chr21	45712867	45712867	http://gnomad.b	0.001788031884	AFR	0.05344320089	20		11	0	https://www.ncbi.nlm.nih.gov/clinvar/variation/128330/
NM_000383.3(AIRE):c.1566+8C>T	concordant	unreportable	unreportable	446817	0	1	SCV000612308	Uncertain significance	Athena Diagnostics	8/17/2017	AIRE	chr21	45716336	45716336	http://gnomad.b	0.001061121725	NFE	0.003293860005	3		1	0	https://www.ncbi.nlm.nih.gov/clinvar/variation/446817/
NM_015166.3(MLC1):c.1032C>T	concordant	unreportable	unreportable	342103	0	1	SCV000439230	Uncertain significance	Illumina Clinical Se	10/18/2016	MLC1	chr22	50502490	50502490	http://gnomad.b	2.24E-05	AMR	9.04E-05	0		0	0	https://www.ncbi.nlm.nih.gov/clinvar/variation/342103/
NM_015166.3(MLC1):c.598-9C>T	concordant	unreportable	unreportable	284440	0	1	SCV000337070	Uncertain significance	EGL Genetic Diagn	6/30/2017	MLC1	chr22	50512770	50512770	http://gnomad.b	0.0002153508821	ASJ	0.001828519977	0		1	0	https://www.ncbi.nlm.nih.gov/clinvar/variation/284440/
NM_015166.3(MLC1):c.93C>T	concordant	unreportable	unreportable	289952	0	1	SCV000344416	Uncertain significance	EGL Genetic Diagn	6/30/2017	MLC1	chr22	50523239	50523239	http://gnomad.b	4.75E-05	AFR	0.000327354006	0		0	0	https://www.ncbi.nlm.nih.gov/clinvar/variation/289952/
NM_000487.5(ARSA):c.*103C>T	concordant	unreportable	unreportable	342233	0	1	SCV000439424	Uncertain significance	Illumina Clinical Se	10/18/2016	ARSA	chr22	51063470	51063470		0.000253248617					1	0	https://www.ncbi.nlm.nih.gov/clinvar/variation/342233/
NM_000487.5(ARSA):c.225-5C>T	concordant	unreportable	unreportable	342239	0	1	SCV000439443	Uncertain significance	Illumina Clinical Se	10/18/2016	ARSA	chr22	51065839	51065839		1.41E-05					0	0	https://www.ncbi.nlm.nih.gov/clinvar/variation/342239/
NM_000330.3(RS1):c.234G>A	concordant	unreportable	unreportable	285377	0	1	SCV000338372	Uncertain significance	EGL Genetic Diagn	6/30/2017	RS1	chrX	18665403	18665403	http://gnomad.b	0.0001330558872	EAS	0.0005440270179	0	3	0	7	https://www.ncbi.nlm.nih.gov/clinvar/variation/285377/
NM_000206.2(IL2RG):c.858G>A	concordant	unreportable	unreportable	368617	0	1	SCV000482704	Likely benign	Illumina Clinical Se	10/18/2016	IL2RG	chrX	70328193	70328193	http://gnomad.b	0.000145227814	FIN	0.0269710999	6	181	0	2	https://www.ncbi.nlm.nih.gov/clinvar/variation/368617/
NM_000206.2(IL2RG):c.325G>A	concordant	unreportable	unreportable	368619	0	1	SCV000482706	Likely benign	Illumina Clinical Se	10/18/2016	IL2RG	chrX	70330483	70330483	http://gnomad.b	0.000648684236	FIN	0.004439310171	1	54	0	16	https://www.ncbi.nlm.nih.gov/clinvar/variation/368619/
NM_000191.2(HMGCL):c.498-10C>A	concordant	unreportable	unreportable	387964	0	1	SCV000530161	Likely benign	GeneDx	11/28/2017	HMGCL	chr1	24137299	24137299	http://gnomad.b	0.000175997184	NFE	0.0001345189958	0		0	0	https://www.ncbi.nlm.nih.gov/clinvar/variation/387964/
NM_000191.2(HMGCL):c.145-8C>T	concordant	unreportable	unreportable	392129	0	1	SCV000535343	Likely benign	GeneDx	11/28/2017	HMGCL	chr1	24144081	24144081	http://gnomad.b	0.000111998208	AMR	0.0004169640015	0		0	0	https://www.ncbi.nlm.nih.gov/clinvar/variation/392129/
NM_000310.3(PPT1):c.810G>C	concordant	unreportable	unreportable	416009	0	1	SCV000561184	Likely benign	Invitae	3/14/2017	PPT1	chr1	40539844	40539844	http://gnomad.b	2.79E-05	AMR	0.0001489069982	0		0	0	https://www.ncbi.nlm.nih.gov/clinvar/variation/416009/
NM_000310.3(PPT1):c.802C>T	concordant	unreportable	unreportable	449662	0	1	SCV000617991	Uncertain significance	GeneDx	11/28/2017	PPT1	chr1	40539852	40539852	http://gnomad.b	0.0001755373393	NFE	0.0001881450007	0		0	0	https://www.ncbi.nlm.nih.gov/clinvar/variation/449662/
NM_000310.3(PPT1):c.762A>C	concordant	unreportable	unreportable	206639	0	1	SCV000357391	Uncertain significance	Illumina Clinical Se	10/18/2016	PPT1	chr1	40542550	40542550	http://gnomad.b	8.36E-06	NFE	3.58E-05	0		0	0	https://www.ncbi.nlm.nih.gov/clinvar/variation/206639/
NM_000310.3(PPT1):c.627+9C>T	concordant	unreportable	unreportable	416011	0	1	SCV000561187	Likely benign	Invitae	10/5/2017	PPT1	chr1	40546060	40546060	http://gnomad.b	1.11E-05	AFR	0.0003921060124	0		0	0	https://www.ncbi.nlm.nih.gov/clinvar/variation/416011/
NM_000310.3(PPT1):c.536G>A	concordant	unreportable	unreportable	206648	0	2	SCV000242345;S	Uncertain significance;Unc	GeneDx;Invitae	2017-11-28;2017-0	PPT1	chr1	40555082	40555082	http://gnomad.b	3.90E-05	NFE	7.17E-05	0		0	0	https://www.ncbi.nlm.nih.gov/clinvar/variation/206648/
NM_000310.3(PPT1):c.234+18T>C	concordant	unreportable	unreportable	387215	0	1	SCV000529138	Likely benign	GeneDx	11/28/2017	PPT1	chr1	40558052	40558052	http://gnomad.b	5.57E-06	AMR	8.93E-05	0		0	0	https://www.ncbi.nlm.nih.gov/clinvar/variation/387215/
NM_015506.2(MMACHC):c.165G>A	concordant	unreportable	unreportable	287959	0	1	SCV000341926	Benign	EGL Genetic Diagn	6/30/2017	MMACHC	chr1	45973111	45973111	http://gnomad.b	6.33E-05	AMR	0.0003871119989	0		0	0	https://www.ncbi.nlm.nih.gov/clinvar/variation/287959/
NM_015506.2(MMACHC):c.181C>T	concordant	unreportable	unreportable	203824	0	2	SCV000238985;S	Uncertain significance;Unc	GeneDx;Invitae	2017-11-28;2017-1	MMACHC	chr1	45973127	45973127	http://gnomad.b	0.0006883347047	NFE	0.001020989963	0		0	0	https://www.ncbi.nlm.nih.gov/clinvar/variation/203824/

Supplemental Table 2

NM_015506.2(MMACHC):c.277-4C>G	concordant	unreportable	unreportable	297481	0	2	SCV000531461;S(	Likely benign;Uncertain sig	GeneDx;Illumina C	2017-11-28;2016-1	MMACHC	chr1	45973880	45973880	http://gnomad.b	0.00045097791	NFE	0.0006002290174	0		0	0	https://www.ncbi.nlm.nih.gov/clinvar/variation/297481/
NM_015506.2(MMACHC):c.545G>A	concordant	unreportable	unreportable	297487	0	1	SCV000357928	Uncertain significance	Illumina Clinical Se	10/18/2016	MMACHC	chr1	45974583	45974583	http://gnomad.b	4.75E-05	NFE	3.58E-05	0		0	0	https://www.ncbi.nlm.nih.gov/clinvar/variation/297487/
NM_015506.2(MMACHC):c.650A>T	concordant	unreportable	unreportable	465313	0	1	SCV000640289	Uncertain significance	Invitae	10/5/2017	MMACHC	chr1	45974688	45974688	http://gnomad.b	6.33E-05	NFE	0.0001881520002	0		0	0	https://www.ncbi.nlm.nih.gov/clinvar/variation/465313/
NM_015506.2(MMACHC):c.691T>C	concordant	unreportable	unreportable	297488	0	1	SCV000357929	Uncertain significance	Illumina Clinical Se	10/18/2016	MMACHC	chr1	45974729	45974729	http://gnomad.b	0.0001265902905	AFR	0.001635059947	0		0	0	https://www.ncbi.nlm.nih.gov/clinvar/variation/297488/
NM_015506.2(MMACHC):c.*20T>C	concordant	unreportable	unreportable	388271	0	1	SCV000530546	Likely benign	GeneDx	11/28/2017	MMACHC	chr1	45974907	45974907	http://gnomad.b	2.37E-05	AFR	0.0002014229976	0		0	0	https://www.ncbi.nlm.nih.gov/clinvar/variation/388271/
NM_017739.3(POMGNT1):c.1979C>T	concordant	unreportable	unreportable	285069	0	1	SCV000337931	Uncertain significance	EGL Genetic Diagn	6/30/2017	POMGNT1	chr1	46654946	46654946	http://gnomad.b	1.40E-05	AFR	0.0004582350084	0		0	0	https://www.ncbi.nlm.nih.gov/clinvar/variation/285069/
NM_017739.3(POMGNT1):c.1536T>C	concordant	unreportable	unreportable	287396	0	1	SCV000341157	Uncertain significance	EGL Genetic Diagn	6/30/2017	POMGNT1	chr1	46657773	46657773	http://gnomad.b	2.24E-05	AFR	0.0003921060124	0		0	0	https://www.ncbi.nlm.nih.gov/clinvar/variation/287396/
NM_017739.3(POMGNT1):c.1509C>T	concordant	unreportable	unreportable	471402	0	1	SCV000649958	Likely benign	Invitae	10/5/2017	POMGNT1	chr1	46657800	46657800	http://gnomad.b	1.12E-05	EAS	5.80E-05	0		0	0	https://www.ncbi.nlm.nih.gov/clinvar/variation/471402/
NM_017739.3(POMGNT1):c.1476C>A	concordant	unreportable	unreportable	285360	0	1	SCV000338344	Uncertain significance	EGL Genetic Diagn	6/30/2017	POMGNT1	chr1	46657833	46657833	http://gnomad.b	2.80E-05	NFE	2.69E-05	0		0	0	https://www.ncbi.nlm.nih.gov/clinvar/variation/285360/
NM_017739.3(POMGNT1):c.1173T>C	concordant	unreportable	unreportable	390299	0	1	SCV000533091	Likely benign	GeneDx	11/28/2017	POMGNT1	chr1	46658610	46658610		2.80E-06					0	0	https://www.ncbi.nlm.nih.gov/clinvar/variation/390299/
NM_017739.3(POMGNT1):c.1010T>C	concordant	unreportable	unreportable	285192	0	1	SCV000338116	Uncertain significance	EGL Genetic Diagn	6/30/2017	POMGNT1	chr1	46659252	46659252	http://gnomad.b	2.52E-05	AMR	0.0001489069982	0		0	0	https://www.ncbi.nlm.nih.gov/clinvar/variation/285192/
NM_017739.3(POMGNT1):c.959G>T	concordant	unreportable	unreportable	297535	0	1	SCV000357995	Uncertain significance	Illumina Clinical Se	10/18/2016	POMGNT1	chr1	46659303	46659303		1.40E-05					0	0	https://www.ncbi.nlm.nih.gov/clinvar/variation/297535/
NM_017739.3(POMGNT1):c.958C>T	concordant	unreportable	unreportable	286005	0	1	SCV000339255	Uncertain significance	EGL Genetic Diagn	6/30/2017	POMGNT1	chr1	46659304	46659304	http://gnomad.b	4.20E-05	AFR	0.0001962840033	0		0	0	https://www.ncbi.nlm.nih.gov/clinvar/variation/286005/
NM_017739.3(POMGNT1):c.888C>A	concordant	unreportable	unreportable	497835	0	1	SCV000702558	Uncertain significance	EGL Genetic Diagn	6/30/2017	POMGNT1	chr1	46659589	46659589	http://gnomad.b	2.80E-05	NFE	4.48E-05	0		0	0	https://www.ncbi.nlm.nih.gov/clinvar/variation/497835/
NM_017739.3(POMGNT1):c.751+10G	concordant	unreportable	unreportable	471409	0	1	SCV000649975	Likely benign	Invitae	10/5/2017	POMGNT1	chr1	46660215	46660215	http://gnomad.b	0.0001203814131	NFE	8.96E-05	0		0	0	https://www.ncbi.nlm.nih.gov/clinvar/variation/471409/
NM_017739.3(POMGNT1):c.664G>A	concordant	unreportable	unreportable	290168	0	1	SCV000344673	Uncertain significance	EGL Genetic Diagn	6/30/2017	POMGNT1	chr1	46660312	46660312	http://gnomad.b	4.48E-05	EAS	0.0001739330037	0		0	0	https://www.ncbi.nlm.nih.gov/clinvar/variation/290168/
NM_017739.3(POMGNT1):c.652+6G>	concordant	unreportable	unreportable	290446	0	1	SCV000345005	Uncertain significance	EGL Genetic Diagn	6/30/2017	POMGNT1	chr1	46660510	46660510	http://gnomad.b	6.16E-05	AFR	0.0004577559885	0		0	0	https://www.ncbi.nlm.nih.gov/clinvar/variation/290446/
NM_017739.3(POMGNT1):c.444G>A	concordant	unreportable	unreportable	289366	0	1	SCV000343715	Uncertain significance	EGL Genetic Diagn	6/30/2017	POMGNT1	chr1	46661573	46661573	http://gnomad.b	2.24E-05	AFR	6.54E-05	0		0	0	https://www.ncbi.nlm.nih.gov/clinvar/variation/289366/
NM_017739.3(POMGNT1):c.426C>T	concordant	unreportable	unreportable	500537	0	1	SCV000706541	Uncertain significance	EGL Genetic Diagn	6/30/2017	POMGNT1	chr1	46661591	46661591	http://gnomad.b	3.92E-05	AFR	6.54E-05	0		0	0	https://www.ncbi.nlm.nih.gov/clinvar/variation/500537/
NM_017739.3(POMGNT1):c.421-13_4	concordant	unreportable	unreportable	420433	0	1	SCV000569263	Likely benign	GeneDx	11/28/2017	POMGNT1	chr1	46661605	46661605	http://gnomad.b	5.60E-06	AFR	0.0002616429992	0		0	0	https://www.ncbi.nlm.nih.gov/clinvar/variation/420433/
NM_017739.3(POMGNT1):c.287G>C	concordant	unreportable	unreportable	450326	0	1	SCV000618897	Uncertain significance	GeneDx	11/28/2017	POMGNT1	chr1	46662470	46662470	http://gnomad.b	3.64E-05	NFE	4.48E-05	0		0	0	https://www.ncbi.nlm.nih.gov/clinvar/variation/450326/
NM_017739.3(POMGNT1):c.268C>T	concordant	unreportable	unreportable	283329	0	1	SCV000335345	Uncertain significance	EGL Genetic Diagn	6/30/2017	POMGNT1	chr1	46662489	46662489	http://gnomad.b	8.40E-05	AMR	5.96E-05	0		0	0	https://www.ncbi.nlm.nih.gov/clinvar/variation/283329/
NM_017739.3(POMGNT1):c.129C>T	concordant	unreportable	unreportable	297538	0	1	SCV000358009	Uncertain significance	Illumina Clinical Se	10/18/2016	POMGNT1	chr1	46662748	46662748	http://gnomad.b	3.36E-05	AMR	0.0002382230014	0		0	0	https://www.ncbi.nlm.nih.gov/clinvar/variation/297538/
NM_000098.2(CPT2):c.852C>T	concordant	unreportable	unreportable	297605	0	1	SCV000358101	Uncertain significance	Illumina Clinical Se	10/18/2016	CPT2	chr1	53676198	53676198	http://gnomad.b	0.0001147218973	SAS	0.0001949319994	0		0	0	https://www.ncbi.nlm.nih.gov/clinvar/variation/297605/
NM_000098.2(CPT2):c.870C>T	concordant	unreportable	unreportable	392128	0	1	SCV000535342	Likely benign	GeneDx	11/28/2017	CPT2	chr1	53676216	53676216	http://gnomad.b	4.76E-05	NFE	6.27E-05	0		0	0	https://www.ncbi.nlm.nih.gov/clinvar/variation/392128/
NM_000016.4(ACADM):c.-17C>G	concordant	unreportable	unreportable	226052	0	1	SCV000590781	Uncertain significance	GeneDx	11/28/2017	ACADM	chr1	76190456	76190456	http://gnomad.b	1.09E-05	AFR	0.0005719729816	0		0	0	https://www.ncbi.nlm.nih.gov/clinvar/variation/226052/
NM_000016.4(ACADM):c.1194+11A>G	concordant	unreportable	unreportable	384994	0	1	SCV000526102	Likely benign	GeneDx	11/28/2017	ACADM	chr1	76227066	76227066	http://gnomad.b	8.16E-05	AFR	0.003163310001	0		0	0	https://www.ncbi.nlm.nih.gov/clinvar/variation/384994/
NM_000642.2(AGL):c.78A>G	concordant	unreportable	unreportable	456513	0	1	SCV000626768	Likely benign	Invitae	10/5/2017	AGL	chr1	100316676	100316676	http://gnomad.b	2.51E-05	AFR	0.0001960780064	0		0	0	https://www.ncbi.nlm.nih.gov/clinvar/variation/456513/
NM_000642.2(AGL):c.261A>G	concordant	unreportable	unreportable	390684	0	2	SCV000533581;S(	Likely benign;Uncertain sig	GeneDx;Invitae	2017-11-28;2017-1	AGL	chr1	100327237	100327237	http://gnomad.b	3.91E-05	NFE	5.38E-05	0		0	0	https://www.ncbi.nlm.nih.gov/clinvar/variation/390684/
NM_000642.2(AGL):c.318C>T	concordant	unreportable	unreportable	291323	0	1	SCV000346055	Uncertain significance	Illumina Clinical Se	10/18/2016	AGL	chr1	100327837	100327837	http://gnomad.b	2.79E-06	NFE	1.79E-05	0		0	0	https://www.ncbi.nlm.nih.gov/clinvar/variation/291323/
NM_000642.2(AGL):c.846+8T>A	concordant	unreportable	unreportable	456514	0	1	SCV000626769	Uncertain significance	Invitae	10/5/2017	AGL	chr1	100336145	100336145	http://gnomad.b	3.63E-05	NFE	0.0001167200026	0		0	0	https://www.ncbi.nlm.nih.gov/clinvar/variation/456514/
NM_000642.2(AGL):c.846+14G>A	concordant	unreportable	unreportable	389050	0	1	SCV000531473	Likely benign	GeneDx	11/28/2017	AGL	chr1	100336151	100336151	http://gnomad.b	2.79E-05	FIN	0.0002693000133	0		0	0	https://www.ncbi.nlm.nih.gov/clinvar/variation/389050/
NM_000642.2(AGL):c.1050G>A	concordant	unreportable	unreportable	291329	0	1	SCV000346063	Uncertain significance	Illumina Clinical Se	10/18/2016	AGL	chr1	100340334	100340334	http://gnomad.b	1.12E-05	EAS	0.0001739330037	0		0	0	https://www.ncbi.nlm.nih.gov/clinvar/variation/291329/
NM_000642.2(AGL):c.1936A>G	concordant	unreportable	unreportable	456462	0	1	SCV000626685	Uncertain significance	Invitae	10/5/2017	AGL	chr1	100346668	100346668	http://gnomad.b	4.74E-05	SAS	0.0007147029974	0		0	0	https://www.ncbi.nlm.nih.gov/clinvar/variation/456462/
NM_000642.2(AGL):c.2941A>G	concordant	unreportable	unreportable	456479	0	1	SCV000626713	Uncertain significance	Invitae	10/5/2017	AGL	chr1	100356904	100356904	http://gnomad.b	8.37E-05	AFR	0.001961299917	0		0	0	https://www.ncbi.nlm.nih.gov/clinvar/variation/456479/
NM_000642.2(AGL):c.3589-19C>T	concordant	unreportable	unreportable	382407	0	1	SCV000522335	Likely benign	GeneDx	11/28/2017	AGL	chr1	100368220	100368220	http://gnomad.b	0.0001032671493	FIN	0.0005381649826	0		0	0	https://www.ncbi.nlm.nih.gov/clinvar/variation/382407/
NM_000642.2(AGL):c.3697G>A	concordant	unreportable	unreportable	456495	0	1	SCV000626736	Uncertain significance	Invitae	10/5/2017	AGL	chr1	100368347	100368347	http://gnomad.b	5.30E-05	AFR	0.0001989650045	0		0	0	https://www.ncbi.nlm.nih.gov/clinvar/variation/456495/
NM_000642.2(AGL):c.3700+14A>G	concordant	unreportable	unreportable	391083	0	1	SCV000534066	Likely benign	GeneDx	11/28/2017	AGL	chr1	100368364	100368364	http://gnomad.b	3.91E-05	NFE	0.0003463109897	0		0	0	https://www.ncbi.nlm.nih.gov/clinvar/variation/391083/
NM_000642.2(AGL):c.4283A>C	concordant	unreportable	unreportable	424905	0	1	SCV000626751	Uncertain significance	Invitae	10/5/2017	AGL	chr1	100381989	100381989	http://gnomad.b	3.63E-05	NFE	2.70E-05	0		0	0	https://www.ncbi.nlm.nih.gov/clinvar/variation/424905/
NM_014625.2(NPHS2):c.874-13A>G	concordant	unreportable	unreportable	293847	0	1	SCV000351500	Uncertain significance	Illumina Clinical Se	10/18/2016	NPHS2	chr1	179520599	179520599	http://gnomad.b	5.63E-06	FIN	0.0001797430014	0		0	0	https://www.ncbi.nlm.nih.gov/clinvar/variation/293847/
NM_014625.2(NPHS2):c.451+9dupA	concordant	unreportable	unreportable	260427	0	1	SCV000614349	Likely benign	Athena Diagnostics	8/17/2017	NPHS2	chr1	179530414	179530414	http://gnomad.b	0.0002617462131	AFR	0.00771543989	0		0	0	https://www.ncbi.nlm.nih.gov/clinvar/variation/260427/
NM_005562.2(LAMC2):c.503+6T>C	concordant	unreportable	unreportable	293992	0	1	SCV000351659	Uncertain significance	Illumina Clinical Se	10/18/2016	LAMC2	chr1	183187629	183187629	http://gnomad.b	5.04E-05	NFE	0.0002256279986	0		0	0	https://www.ncbi.nlm.nih.gov/clinvar/variation/293992/
NM_005562.2(LAMC2):c.954-15T>C	concordant	unreportable	unreportable	293999	0	1	SCV000351669	Uncertain significance	Illumina Clinical Se	10/18/2016	LAMC2	chr1	183194728	183194728	http://gnomad.b	3.64E-05	NFE	0.0001881049975	0		0	0	https://www.ncbi.nlm.nih.gov/clinvar/variation/293999/
NM_005562.2(LAMC2):c.2081G>A	concordant	unreportable	unreportable	294007	0	1	SCV000351679	Uncertain significance	Illumina Clinical Se	10/18/2016	LAMC2	chr1	183201853	183201853	http://gnomad.b	4.76E-05	NFE	0.0001523630053	0		0	0	https://www.ncbi.nlm.nih.gov/clinvar/variation/294007/
NM_000228.2(LAMB3):c.2646G>A	concordant	unreportable	unreportable	295083	0	1	SCV000353605	Uncertain significance	Illumina Clinical Se	10/18/2016	LAMB3	chr1	209795936	209795936	http://gnomad.b	1.68E-05	NFE	2.69E-05	0		0	0	https://www.ncbi.nlm.nih.gov/clinvar/variation/295083/
NM_000228.2(LAMB3):c.1792C>T	concordant	unreportable	unreportable	218484	0	1	SCV000257743	Uncertain significance	Division of Genomi	8/10/2015	LAMB3	chr1	209799177	209799177	http://gnomad.b	8.40E-05	AFR	0.0005900869728	0		0	0	https://www.ncbi.nlm.nih.gov/clinvar/variation/218484/
NM_000228.2(LAMB3):c.1536C>T	concordant	unreportable	unreportable	295107	0	1	SCV000353632	Uncertain significance	Illumina Clinical Se	10/18/2016	LAMB3	chr1	209800273	209800273	http://gnomad.b	8.40E-06	EAS	5.81E-05	0		0	0	https://www.ncbi.nlm.nih.gov/clinvar/variation/295107/
NM_000228.2(LAMB3):c.1267G>A	concordant	unreportable	unreportable	295111	0	1	SCV000353636	Uncertain significance	Illumina Clinical Se	10/18/2016	LAMB3	chr1	209801401	209801401	http://gnomad.b	1.68E-05	AFR	0.0002201349998	0		0	0	https://www.ncbi.nlm.nih.gov/clinvar/variation/295111/
NM_000228.2(LAMB3):c.271C>A	concordant	unreportable	unreportable	295143	0	1	SCV000353673	Uncertain significance	Illumina Clinical Se	10/18/2016	LAMB3	chr1	209811906	209811906	http://gnomad.b	1.68E-05	NFE	2.69E-05	0		0	0	https://www.ncbi.nlm.nih.gov/clinvar/variation/295143/
NM_033056.3(PCDH15):c.5566G>A	concordant	unreportable	unreportable	300168	0	1	SCV000363104	Uncertain significance	Illumina Clinical Se	10/18/2016	PCDH15	chr10	55581920	55581920	http://gnomad.b	2.57E-06	AFR	6.53E-05	0		0	0	https://www.ncbi.nlm.nih.gov/clinvar/variation/300168/
NM_033056.3(PCDH15):c.4368T>A	concordant	unreportable	unreportable	440029	0	1	SCV000604611	Uncertain significance	ARUP Laboratories	6/30/2017	PCDH15	chr10	55583118	55583118		5.14E-06					0	0	https://www.ncbi.nlm.nih.gov/clinvar/variation/440029/
NM_033056.3(PCDH15):c.4368-13_43	concordant	unreportable	unreportable	227008	0	1	SCV000269624	Benign	Laboratory for Mol	4/26/2016	PCDH15	chr10	55583127	55583127	http://gnomad.b	0.0002263025253	AMR	0.01052660029	1		0	0	https://www.ncbi.nlm.nih.gov/clinvar/variation/227008/
NM_033056.3(PCDH15):c.3122+3A>G	concordant	unreportable	unreportable	497490	0	1	SCV000702021	Uncertain significance	EGL Genetic Diagn	6/30/2017	PCDH15	chr10	55719489	55719489	http://gnomad.b	2.31E-05	NFE	8.97E-06	0		0	0	https://www.ncbi.nlm.nih.gov/clinvar/variation/497490/
NM_033056.3(PCDH15):c.3009+7G>A	concordant	unreportable	unreportable	284434	0	1	SCV000337067	Uncertain significance	EGL Genetic Diagn	11/3/2016	PCDH15	chr10	55721505	55721505	http://gnomad.b	6.94E-05	ASJ	0.001550549991	0		0	0	https://www.ncbi.nlm.nih.gov/clinvar/variation/284434/
NM_033056.3(PCDH15):c.2807C>T	concordant	unreportable	unreportable	300185	0	1	SCV000363179	Uncertain significance	Illumina Clinical Se	10/18/2016	PCDH15	chr10	55755470	55755470	http://gnomad.b	3.60E-05	NFE	8.08E-05	0		0	0	https://www.ncbi.nlm.nih.gov/clinvar/variation/300185/
NM_033056.3(PCDH15):c.2799G>C	concordant	unreportable	unreportable	496490	0	1	SCV000699758	Uncertain significance	Laboratory Corpora	1/25/2018	PCDH15	chr10	55755478	55755478	http://gnomad.b	3.34E-05	NFE	5.39E-05	0		0	0	https://www.ncbi.nlm.nih.gov/clinvar/variation/496490/
NM_033056.3(PCDH15):c.2102C>T	concordant	unreportable	unreportable	164921	0	1	SCV000199859	Uncertain significance	Laboratory for Mol	4/26/2016	PCDH15	chr10	55826635	55826635	http://gnomad.b	0.0001902998508	NFE	0.0002696680021	0		0	0	https://www.ncbi.nlm.nih.gov/clinvar/variation/164921/
NM_033056.3(PCDH15):c.1606G>C	concordant	unreportable	unreportable	229135	0	1	SCV000272296	Uncertain significance	Laboratory for Mol	4/26/2016	PCDH15	chr10	55913038	55913038	http://gnomad.b	4.89E-05	AFR	0.0007190480246	0		0	0	https://www.ncbi.nlm.nih.gov/clinvar/variation/229135/
NM_033056.3(PCDH15):c.594G>A	concordant	unreportable	unreportable	300202	0	1	SCV000363236	Uncertain significance	Illumina Clinical Se	10/18/2016	PCDH15	chr10	56106125	56106125	http://gnomad.b	3.09E-05	AFR	6.54E-05	0		0	0	https://www.ncbi.nlm.nih.gov/clinvar/variation/300202/
NM_000360.3(TH):c.841+12C>T	concordant	unreportable	unreportable	304075	0	1	SCV000369917	Uncertain significance	Illumina Clinical Se	10/18/2016	TH	chr11	2188105	2188105		1.41E-05					0	0	https://www.ncbi.nlm.nih.gov/clinvar/variation/304075/
NM_000360.3(TH):c.398G>A	concordant	unreportable	unreportable	304079	0	2	SCV000701594;S(	Uncertain significance;Unc	EGL Genetic Diagn	2017-06-30;2016-1	TH	chr11	2189810	2189810	http://gnomad.b	8.45E-05	NFE	0.0001031680004	0		0	0	https://www.ncbi.nlm.nih.gov/clinvar/variation/304079/
NM_000543.4(SMPD1):c.291A>G	concordant	unreportable	unreportable	305195	0	1	SCV000372922	Uncertain significance	Illumina Clinical Se	10/18/2016	SMPD1	chr11	6412119	6412119	http://gnomad.b	3.81E-05	NFE	8.96E-06	0		0	0	https://www.ncbi.nlm.nih.gov/clinvar/variation/305195/
NM_000543.4(SMPD1):c.708G>A	concordant	unreportable	unreportable	500493	0	1	SCV000706470	Uncertain significance	EGL Genetic Diagn	6/30/2017	SMPD1	chr11	6413003	6413003	http://gnomad.b	0.0001094196004	FIN	0.000243521994	0		0	0	https://www.ncbi.nlm.nih.gov/clinvar/variation/500493/
NM_000543.4(SMPD1):c.729C>T	concordant	unreportable	unreportable	305198	0	2	SCV000704661;S(	Uncertain significance;Unc	EGL Genetic Diagn	2017-06-30;2016-1	SMPD1	chr11	6413024	6413024	http://gnomad.b	0.000202188392	NFE	0.0002528080076	0		0	0	https://www.ncbi.nlm.nih.gov/clinvar/variation/305198/
NM_000543.4(SMPD1):c.733T>C	concordant	unreportable	unreportable	291201	0	2	SCV000345913;S(	Uncertain significance;Unc	EGL Genetic Diagn	2017-06-30;2017-1	SMPD1	chr11	6413028	6413028	http://gnomad.b	7.61E-05	NFE	7.21E-05	0		0	0	https://www.ncbi.nlm.nih.gov/clinvar/variation/291201/
NM_000543.4(SMPD1):c.1340+8G>A	concordant	unreportable	unreportable	305203	0	1	SCV000372939	Uncertain significance	Illumina Clinical Se	10/18/2016	SMPD1	chr11	6414931	6414931	http://gnomad.b	2.14E-05	AFR	0.0001961550006	0		0	0	https://www.ncbi.nlm.nih.gov/clinvar/variation/305203/
NM_000391.3(TPP1):c.1653C>G	concordant	unreportable	unreportable	305506	0	3	SCV000702022;S(	Uncertain significance;Likel	EGL Genetic Diagn	2017-06-30;2017-1	TPP1	chr11	6635816	6635816	http://gnomad.b	0.0001147167615	NFE	0.0002864559938	0		0	0	https://www.ncbi.nlm.nih.gov/clinvar/variation/305506/
NM_000391.3(TPP1):c.1489C>T	concordant	unreportable	unreportable	457941	0	1	SCV000628904	Uncertain significance	Invitae	10/5/2017	TPP1	chr11	6636159	6636159	http://gnomad.b	1.40E-05	AFR	6.53E-05	0		0	0	https://www.ncbi.nlm.nih.gov/clinvar/variation/457941/
NM_000391.3(TPP1):c.688-4A>G	concordant	unreportable	unreportable	415294	0	1	SCV000559661	Likely benign	Invitae	3/14/2017	TPP1	chr11	6638094	6638094	http://gnomad.b	8.39E-06	AFR	0.0002613700053	0		0	0	https://www.ncbi.nlm.nih.gov/clinvar/variation/415294/
NM_000391.3(TPP1):c.688-7T>A	concordant	unreportable	unreportable	207553	0	2	SCV000708550;S(	Likely benign;Likely benign	EGL Genetic Diagn	2017-06-30;2017-1	TPP1	chr11	6638097	6638097	http://gnomad.b	0.0001846660063	AFR	0.006207529921	1		0	0	https://www.ncbi.nlm.nih.gov/clinvar/variation/207553/
NM_000391.3(TPP1):c.509-5T>C	concordant	unreportable	unreportable	378758	0	1	SCV000514960	Likely benign	GeneDx	11/28/2017	TPP1	chr11	6638389	6638389	http://gnomad.b	5.04E-05	NFE	0.0001433080033	0		0	0	https://www.ncbi.nlm.nih.gov/clinvar/variation/378758/
NM_000391.3(TPP1):c.69G>A	concordant	unreportable	unreportable	379081	0	2	SCV000515652;S(	Likely benign;Likely benign	GeneDx;Invitae	2017-11-28;2017-1	TPP1	chr11	6640447	6640447	http://gnomad.b	2.52E-05	SAS	3.25E-05	0		0	0	https://www.ncbi.nlm.nih.gov/clinvar/variation/379081/
NM_000391.3(TPP1):c.-20C>G	concordant	unreportable	unreportable	381377	0	1	SCV000520597	Likely benign	GeneDx	11/28/2017	TPP1	chr11	6640651	6640651	http://gnomad.b	1.68E-05	AFR	0.000326754991	0		0	0	https://www.ncbi.nlm.nih.gov/clinvar/variation/381377/
NM_000352.3(ABCC8):c.4647C>T	concordant	unreportable	unreportable	303748	0	1	SCV000369224	Likely benign	Illumina Clinical Se	10/18/2016	ABCC8	chr11	17414637	17414637	http://gnomad.b	0.0001135677302	EAS	0.0006378290127	0		0	0	https://www.ncbi.nlm.nih.gov/clinvar/variation/303748/
NM_000352.3(ABCC8):c.3867+7G>A	concordant	unreportable	unreportable	446771	0	1	SCV000612211	Uncertain significance	Athena Diagnostics	8/17/2017	ABCC8	chr11	17419224	17419224	http://gnomad.b	0.0001884191888	NFE	0.0006444120081	0		0	0	https://www.ncbi.nlm.nih.gov/clinvar/variation/446771/
NM_000352.3(ABCC8):c.3858C>T	concordant	unreportable	unreportable	303758	0	1	SCV000369264	Likely benign	Illumina Clinical Se	10/18/2016	ABCC8	chr11	17419240	17419240	http://gnomad.b	7.23E-05	SAS	6.50E-05	0		0	0	https://www.ncbi.nlm.nih.gov/clinvar/variation/303758/
NM_024649.4(BBS1):c.981C>T	concordant	unreportable	unreportable	305464	0	1	SCV000373270	Uncertain significance	Illumina Clinical Se	10/18/2016	BBS1	chr11	66291224	66291224	http://gnomad.b	5.02E-05	AFR	0.0001309759973	0		0	0	https://www.ncbi.nlm.nih.gov/clinvar/variation/305464/
NM_024649.4(BBS1):c.1110+15C>T	concordant	unreportable	unreportable	305466	0	1	SCV000373272	Uncertain significance	Illumina Clinical Se	10/18/2016	BBS1	chr11	66291368	66291368	http://gnomad.b	1.11E-05	AFR	0.0001332620013	0		0	0	https://www.ncbi.nlm.nih.gov/clinvar/variation/305466/
NM_001876.3(CPT1A):c.1557G>A	concordant	unreportable	unreportable	463965	0	1	SCV000638096	Likely benign	Invitae	10/5/2017	CPT1A	chr11	68542802	68542802	http://gnomad.b	0.0001070090958	AFR	0.001568219974	0		0	0	https://www.ncbi.nlm.nih.gov/clinvar/variation/463965/
NM_001876.3(CPT1A):c.694-10C>T	concordant	unreportable	unreportable	389635	0	1	SCV000532237	Likely benign	GeneDx	11/28/2017	CPT1A	chr11	68564411	68564411	http://gnomad.b	0.0001013770381	ASJ	0.003883890109	0		0	0	https://www.ncbi.nlm.nih.gov/clinvar/variation/389635/
NM_001876.3(CPT1A):c.669A>G	concordant	unreportable	unreportable	463968	0	1	SCV000638101	Likely benign	Invitae	10/5/2017	CPT1A	chr11	68566710	68566710	http://gnomad.b	5.91E-05	AFR	0.0006534240092	0		0	0	https://www.ncbi.nlm.nih.gov/clinvar/variation/463968/
NM_001360.2(DHCR7):c.1011C>T	concordant	unreportable	unreportable	384536	0	1	SCV000525412	Likely benign	GeneDx	11/28/2017	DHCR7	chr11	71146838	71146838	http://gnomad.b	2.69E-05	EAS	0.000116782001	0		0	0	https://www.ncbi.nlm.nih.gov/clinvar/variation/384536/
NM_001360.2(DHCR7):c.321+10C>T	concordant	unreportable	unreportable	93717	0	4	SCV000603311;S(	Benign;Likely benign;Likely	ARUP Laboratories	2017-06-30;2017-1	DHCR7	chr11	71155029	71155029	http://gnomad.b	0.0007135317939	NFE	0.003859850112	1		1	0	https://www.ncbi.nlm.nih.gov/clinvar/variation/93717/
NM_001360.2(DHCR7):c.123G>A	concordant	unreportable	unreportable	498417	0	1	SCV000703418	Uncertain significance	EGL Genetic Diagn	6/30/2017	DHCR7	chr11	71155237	71155237	http://gnomad.b	4.31E-05	NFE	8.34E-05	0		0	0	https://www.ncbi.nlm.nih.gov/clinvar/variation/498417/
NM_001360.2(DHCR7):c.99-5C>T	concordant	unreportable	unreportable	305960	0	2	SCV000704664;S(	Uncertain significance;Unc	EGL Genetic Diagn	2017-06-30;2016-1	DHCR7	chr11	71155266	71155266	http://gnomad.b	8.89E-05	NFE	0.000326148991	0		0	0	https://www.ncbi.nlm.nih.gov/clinvar/variation/305960/
NM_000051.3(ATM):c.-18G>A	concordant	unreportable	unreportable	490407	0	1	SCV000687280	Likely benign	Color Genomics, In	12/21/2017	ATM	chr11	108098334	108098334	http://gnomad.b	1.11E-05	NFE	8.06E-05	0		0	0	https://www.ncbi.nlm.nih.gov/clinvar/variation/490407/
NM_000051.3(ATM):c.133C>T	concordant	unreportable	unreportable	140832	0	3	SCV000681970;S(	Uncertain significance;Unc	Color Genomics, In	2017-10-26;2017-1	ATM	chr11	108098563	108098563	http://gnomad.b	6.68E-05	AFR	0.000130786997	0		0	0	https://www.ncbi.nlm.nih.gov/clinvar/variation/140832/
NM_000051.3(ATM):c.332-3T>C	concordant	unreportable	unreportable	186467	0	3	SCV000512132;S(	Likely benign;Uncertain sig	GeneDx;Invitae;Lal	2017-11-28;2017-1	ATM	chr11	108106394	108106394	http://gnomad.b	2.78E-05	AFR	0.0001313029934	0		0	0	https://www.ncbi.nlm.nih.gov/clinvar/variation/186467/
NM_000051.3(ATM):c.518G>T	concordant	unreportable	unreportable	181909	0	2	SCV000209674;S(	Uncertain significance;Unc	GeneDx;Invitae	2017-11-28;2017-1	ATM	chr11	108114701	108114701		1.11E-05					0	0	https://www.ncbi.nlm.nih.gov/clinvar/variation/181909/
NM_000051.3(ATM):c.526C>G	concordant	unreportable	unreportable	407585	0	2	SCV000682259;S(	Uncertain significance;Unc	Color Genomics, In	2017-10-26;2017-0	ATM	chr11	108114709	108114709	http://gnomad.b	2.78E-06	AFR	6.79E-05	0		0	0	https://www.ncbi.nlm.nih.gov/clinvar/variation/407585/
NM_000051.3(ATM):c.659C>T	concordant	unreportable	unreportable	133640	0	2	SCV000682355;S(	Uncertain significance;Unc	Color Genomics, In	2017-10-26;2017-1	ATM	chr11	108114842	108114842	http://gnomad.b	2.23E-05	SAS	0.0002598919964	0		0	0	https://www.ncbi.nlm.nih.gov/clinvar/variation/133640/
NM_000051.3(ATM):c.716T>C	concordant	unreportable	unreportable	187667	0	1	SCV000622717	Uncertain significance	Invitae	10/5/2017	ATM	chr11	108115568	108115568		2.78E-06					0	0	https://www.ncbi.nlm.nih.gov/clinvar/variation/187667/
NM_000051.3(ATM):c.858A>G	concordant	unreportable	unreportable	185146	0	2	SCV000687844;S(	Likely benign;Uncertain sig	Color Genomics, In	2017-12-21;2017-1	ATM	chr11	108115710	108115710	http://gnomad.b	1.11E-05	AFR	6.56E-05	0		0	0	https://www.ncbi.nlm.nih.gov/clinvar/variation/185146/
NM_000051.3(ATM):c.861T>C	concordant	unreportable	unreportable	231408	0	1	SCV000558422	Likely benign	Invitae	10/5/2017	ATM	chr11	108115713	108115713	http://gnomad.b	2.51E-05	AFR	6.56E-05	0		0	0	https://www.ncbi.nlm.nih.gov/clinvar/variation/231408/
NM_000051.3(ATM):c.1049C>T	concordant	unreportable	unreportable	236665	0	1	SCV000282859	Uncertain significance	Invitae	3/14/2017	ATM	chr11	108117838	108117838	http://gnomad.b	5.57E-06	AMR	5.96E-05	0		0	0	https://www.ncbi.nlm.nih.gov/clinvar/variation/236665/
NM_000051.3(ATM):c.1055T>C	concordant	unreportable	unreportable	420755	0	2	SCV000569719;S(	Uncertain significance;Unc	GeneDx;Invitae	2017-11-28;2017-1	ATM	chr11	108117844	108117844	http://gnomad.b	5.57E-06	NFE	1.79E-05	0		0	0	https://www.ncbi.nlm.nih.gov/clinvar/variation/420755/
NM_000051.3(ATM):c.1271C>A	concordant	unreportable	unreportable	185031	0	3	SCV000292200;S(	Uncertain significance;Unc	Color Genomics, In	2017-12-21;2017-1	ATM	chr11	108121463	108121463	http://gnomad.b	2.23E-05	NFE	2.70E-05	0		0	0	https://www.ncbi.nlm.nih.gov/clinvar/variation/185031/

Supplemental Table 2

NM_000051.3(ATM):c.1440A>C	concordant	unreportable	unreportable	186322	0	2	SCV000681978;S	Uncertain significance;Unc	Color Genomics, In	2017-10-26;2017-1	ATM	chr11	108121632	108121632	http://gnomad.b	3.34E-05	AFR	6.54E-05	0		0	0	https://www.ncbi.nlm.nih.gov/clinvar/variation/186322/
NM_000051.3(ATM):c.1464G>T	concordant	unreportable	unreportable	142433	0	3	SCV000611343;S	Uncertain significance;Unc	Fulgent Genetics,F	2017-05-23;2017-1	ATM	chr11	108121656	108121656	http://gnomad.b	4.18E-05	AMR	5.96E-05	0		0	0	https://www.ncbi.nlm.nih.gov/clinvar/variation/142433/
NM_000051.3(ATM):c.1631T>C	concordant	unreportable	unreportable	185076	0	4	SCV000703944;S	Uncertain significance;Unc	EGL Genetic Diagn	2017-06-30;2017-1	ATM	chr11	108122587	108122587	http://gnomad.b	1.67E-05	AFR	0.0001960780064	0		0	0	https://www.ncbi.nlm.nih.gov/clinvar/variation/185076/
NM_000051.3(ATM):c.1685A>G	concordant	unreportable	unreportable	142359	0	3	SCV000687320;S	Uncertain significance;Unc	Color Genomics, In	2017-12-21;2017-1	ATM	chr11	108122641	108122641	http://gnomad.b	1.39E-05	AFR	0.0004575159983	0		0	0	https://www.ncbi.nlm.nih.gov/clinvar/variation/142359/
NM_000051.3(ATM):c.1855A>C	concordant	unreportable	unreportable	142224	0	3	SCV000278811;S	Uncertain significance;Unc	GeneDx;Invitae;Lal	2017-11-28;2017-1	ATM	chr11	108123596	108123596	http://gnomad.b	4.18E-05	NFE	3.59E-05	0		0	0	https://www.ncbi.nlm.nih.gov/clinvar/variation/142224/
NM_000051.3(ATM):c.1888G>A	concordant	unreportable	unreportable	141251	0	3	SCV000278812;S	Uncertain significance;Likel	GeneDx;Invitae;Lal	2017-11-28;2017-1	ATM	chr11	108123629	108123629	http://gnomad.b	6.68E-05	AMR	0.0004480550124	1		0	0	https://www.ncbi.nlm.nih.gov/clinvar/variation/141251/
NM_000051.3(ATM):c.1943T>C	concordant	unreportable	unreportable	216193	0	2	SCV000569225;S	Uncertain significance;Unc	GeneDx;Invitae	2017-11-28;2017-1	ATM	chr11	108124585	108124585	http://gnomad.b	8.35E-06	NFE	3.58E-05	0		0	0	https://www.ncbi.nlm.nih.gov/clinvar/variation/216193/
NM_000051.3(ATM):c.2082T>C	concordant	unreportable	unreportable	231489	0	3	SCV000537486;S	Likely benign;Likely benign	Color Genomics, In	2017-01-26;2017-1	ATM	chr11	108124724	108124724	http://gnomad.b	8.35E-06	AMR	0.0001191470001	0		0	0	https://www.ncbi.nlm.nih.gov/clinvar/variation/231489/
NM_000051.3(ATM):c.2175C>T	concordant	unreportable	unreportable	381009	0	2	SCV000682035;S	Likely benign;Likely benign	Color Genomics, In	2017-10-26;2017-0	ATM	chr11	108126992	108126992	http://gnomad.b	5.57E-06	NFE	3.58E-05	0		0	0	https://www.ncbi.nlm.nih.gov/clinvar/variation/381009/
NM_000051.3(ATM):c.2251-19T>C	concordant	unreportable	unreportable	245926	0	1	SCV000293129	Uncertain significance	GeneDx	11/28/2017	ATM	chr11	108128189	108128189	http://gnomad.b	3.34E-05	AFR	0.001049040002	0		0	0	https://www.ncbi.nlm.nih.gov/clinvar/variation/245926/
NM_000051.3(ATM):c.2386A>C	concordant	unreportable	unreportable	141757	0	1	SCV000564617	Uncertain significance	GeneDx	11/28/2017	ATM	chr11	108129722	108129722	http://gnomad.b	1.11E-05	NFE	8.96E-06	0		0	0	https://www.ncbi.nlm.nih.gov/clinvar/variation/141757/
NM_000051.3(ATM):c.2480A>G	concordant	unreportable	unreportable	216194	0	1	SCV000254069	Uncertain significance	Invitae	10/5/2017	ATM	chr11	108137911	108137911	http://gnomad.b	5.57E-06	AFR	6.54E-05	0		0	0	https://www.ncbi.nlm.nih.gov/clinvar/variation/216194/
NM_000051.3(ATM):c.2606C>G	concordant	unreportable	unreportable	184982	0	2	SCV000570502;S	Uncertain significance;Unc	GeneDx;Invitae	2017-11-28;2017-1	ATM	chr11	108138037	108138037	http://gnomad.b	2.23E-05	AFR	0.0005228070077	0		0	0	https://www.ncbi.nlm.nih.gov/clinvar/variation/184982/
NM_000051.3(ATM):c.2679A>G	concordant	unreportable	unreportable	184455	0	3	SCV000682068;S	Likely benign;Likely benign	Color Genomics, In	2017-10-26;2017-1	ATM	chr11	108139177	108139177	http://gnomad.b	1.67E-05	NFE	2.69E-05	0		0	0	https://www.ncbi.nlm.nih.gov/clinvar/variation/184455/
NM_000051.3(ATM):c.2804C>T	concordant	unreportable	unreportable	142503	0	2	SCV000209713;S	Uncertain significance;Unc	GeneDx;Invitae	2017-11-28;2017-1	ATM	chr11	108139302	108139302	http://gnomad.b	8.08E-05	NFE	9.85E-05	0		0	0	https://www.ncbi.nlm.nih.gov/clinvar/variation/142503/
NM_000051.3(ATM):c.2838+9C>G	concordant	unreportable	unreportable	215578	0	2	SCV000682081;S	Likely benign;Likely benign	Color Genomics, In	2017-10-26;2017-0	ATM	chr11	108139345	108139345	http://gnomad.b	5.57E-06	NFE	1.79E-05	0		0	0	https://www.ncbi.nlm.nih.gov/clinvar/variation/215578/
NM_000051.3(ATM):c.2887A>G	concordant	unreportable	unreportable	142966	0	2	SCV000292902;S	Uncertain significance;Unc	GeneDx;Invitae	2017-11-28;2017-1	ATM	chr11	108141839	108141839	http://gnomad.b	6.13E-05	AFR	0.0005227390211	0		0	0	https://www.ncbi.nlm.nih.gov/clinvar/variation/142966/
NM_000051.3(ATM):c.2967T>C	concordant	unreportable	unreportable	377509	0	1	SCV000682095	Likely benign	Color Genomics, In	10/26/2017	ATM	chr11	108142023	108142023	http://gnomad.b	1.11E-05	AFR	6.54E-05	0		0	0	https://www.ncbi.nlm.nih.gov/clinvar/variation/377509/
NM_000051.3(ATM):c.3016A>G	concordant	unreportable	unreportable	142162	0	2	SCV000278818;S	Uncertain significance;Unc	GeneDx;Invitae	2017-11-28;2017-1	ATM	chr11	108142072	108142072	http://gnomad.b	3.90E-05	NFE	9.86E-05	0		0	0	https://www.ncbi.nlm.nih.gov/clinvar/variation/142162/
NM_000051.3(ATM):c.3284+7G>C	concordant	unreportable	unreportable	386152	0	2	SCV000527686;S	Likely benign;Likely benign	GeneDx;Invitae	2017-11-28;2017-1	ATM	chr11	108143586	108143586	http://gnomad.b	2.78E-06	AFR	0.0001962579991	0		0	0	https://www.ncbi.nlm.nih.gov/clinvar/variation/386152/
NM_000051.3(ATM):c.3284+16T>G	concordant	unreportable	unreportable	490522	0	1	SCV000687462	Likely benign	Color Genomics, In	12/21/2017	ATM	chr11	108143595	108143595	http://gnomad.b	5.57E-06	AFR	0.0003272680042	0		0	0	https://www.ncbi.nlm.nih.gov/clinvar/variation/490522/
NM_000051.3(ATM):c.3304G>A	concordant	unreportable	unreportable	453458	0	1	SCV000622406	Uncertain significance	Invitae	10/5/2017	ATM	chr11	108150237	108150237		8.35E-06					0	0	https://www.ncbi.nlm.nih.gov/clinvar/variation/453458/
NM_000051.3(ATM):c.3328G>A	concordant	unreportable	unreportable	186152	0	2	SCV000682118;S	Uncertain significance;Unc	Color Genomics, In	2017-10-26;2017-0	ATM	chr11	108150261	108150261	http://gnomad.b	2.78E-06	NFE	1.80E-05	0		0	0	https://www.ncbi.nlm.nih.gov/clinvar/variation/186152/
NM_000051.3(ATM):c.3403-13dupA	concordant	unreportable	unreportable	181858	0	4	SCV000687474;S	Benign;Likely benign;Benig	Color Genomics, In	2017-12-21;2015-0	ATM	chr11	108151707	108151707		5.57E-05					0	0	https://www.ncbi.nlm.nih.gov/clinvar/variation/181858/
NM_000051.3(ATM):c.3403-15_3403-	concordant	unreportable	unreportable	490530	0	1	SCV000687475	Likely benign	Color Genomics, In	12/21/2017	ATM	chr11	108151707	108151707		8.35E-05					0	0	https://www.ncbi.nlm.nih.gov/clinvar/variation/490530/
NM_000051.3(ATM):c.3411T>C	concordant	unreportable	unreportable	184330	0	3	SCV000682129;S	Likely benign;Likely benign	Color Genomics, In	2017-10-26;2017-1	ATM	chr11	108151730	108151730	http://gnomad.b	8.35E-06	NFE	1.79E-05	0		0	0	https://www.ncbi.nlm.nih.gov/clinvar/variation/184330/
NM_000051.3(ATM):c.3468G>A	concordant	unreportable	unreportable	183906	0	3	SCV000682130;S	Likely benign;Likely benign	Color Genomics, In	2017-10-26;2017-1	ATM	chr11	108151787	108151787	http://gnomad.b	3.90E-05	NFE	7.17E-05	0		0	0	https://www.ncbi.nlm.nih.gov/clinvar/variation/183906/
NM_000051.3(ATM):c.3544G>C	concordant	unreportable	unreportable	407566	0	3	SCV000682136;S	Uncertain significance;Unc	Color Genomics, In	2017-10-26;2017-1	ATM	chr11	108151863	108151863	http://gnomad.b	5.57E-06	AFR	0.0001961039961	0		0	0	https://www.ncbi.nlm.nih.gov/clinvar/variation/407566/
NM_000051.3(ATM):c.3565C>T	concordant	unreportable	unreportable	232416	0	1	SCV000682139	Uncertain significance	Color Genomics, In	10/26/2017	ATM	chr11	108151884	108151884	http://gnomad.b	8.35E-06	EAS	0.0001159820022	0		0	0	https://www.ncbi.nlm.nih.gov/clinvar/variation/232416/
NM_000051.3(ATM):c.3577-12delT	concordant	unreportable	unreportable	181859	0	1	SCV000682145	Likely benign	Color Genomics, In	10/26/2017	ATM	chr11	108153420	108153421	http://gnomad.b	2.23E-05	NFE	4.50E-05	0		0	0	https://www.ncbi.nlm.nih.gov/clinvar/variation/181859/
NM_000051.3(ATM):c.3588A>G	concordant	unreportable	unreportable	184211	0	2	SCV000682148;S	Likely benign;Likely benign	Color Genomics, In	2017-10-26;2017-1	ATM	chr11	108153448	108153448	http://gnomad.b	4.73E-05	NFE	6.28E-05	0		0	0	https://www.ncbi.nlm.nih.gov/clinvar/variation/184211/
NM_000051.3(ATM):c.3628A>G	concordant	unreportable	unreportable	141117	0	2	SCV000564629;S	Uncertain significance;Unc	GeneDx;Invitae	2017-11-28;2017-1	ATM	chr11	108153488	108153488	http://gnomad.b	5.57E-05	AFR	0.0005886200233	0		0	0	https://www.ncbi.nlm.nih.gov/clinvar/variation/141117/
NM_000051.3(ATM):c.3639T>C	concordant	unreportable	unreportable	236708	0	1	SCV000282936	Likely benign	Invitae	3/14/2017	ATM	chr11	108153499	108153499	http://gnomad.b	1.95E-05	AFR	0.0001308219944	0		0	0	https://www.ncbi.nlm.nih.gov/clinvar/variation/236708/
NM_000051.3(ATM):c.3676G>C	concordant	unreportable	unreportable	232272	0	1	SCV000282938	Uncertain significance	Invitae	10/5/2017	ATM	chr11	108153536	108153536	http://gnomad.b	5.57E-06	NFE	2.69E-05	0		0	0	https://www.ncbi.nlm.nih.gov/clinvar/variation/232272/
NM_000051.3(ATM):c.3695C>G	concordant	unreportable	unreportable	246339	0	2	SCV000293850;S	Uncertain significance;Unc	GeneDx;Invitae	2017-11-28;2017-1	ATM	chr11	108153555	108153555	http://gnomad.b	2.78E-06	NFE	9.05E-06	0		0	0	https://www.ncbi.nlm.nih.gov/clinvar/variation/246339/
NM_000051.3(ATM):c.3918T>C	concordant	unreportable	unreportable	232893	0	1	SCV000558446	Likely benign	Invitae	10/5/2017	ATM	chr11	108155125	108155125	http://gnomad.b	5.57E-06	NFE	8.97E-06	0		0	0	https://www.ncbi.nlm.nih.gov/clinvar/variation/232893/
NM_000051.3(ATM):c.3964C>A	concordant	unreportable	unreportable	142896	0	2	SCV000293003;S	Uncertain significance;Unc	GeneDx;Invitae	2017-11-28;2017-1	ATM	chr11	108155171	108155171	http://gnomad.b	1.95E-05	NFE	5.38E-05	0		0	0	https://www.ncbi.nlm.nih.gov/clinvar/variation/142896/
NM_000051.3(ATM):c.4060C>A	concordant	unreportable	unreportable	127379	0	2	SCV000149093;S	Uncertain significance;Likel	GeneDx;Invitae	2017-11-28;2017-1	ATM	chr11	108158393	108158393	http://gnomad.b	0.0002450543575	NFE	0.0003665619879	0		0	0	https://www.ncbi.nlm.nih.gov/clinvar/variation/127379/
NM_000051.3(ATM):c.4109+6T>C	concordant	unreportable	unreportable	216212	0	3	SCV000278824;S	Uncertain significance;Unc	GeneDx;Invitae;Lal	2017-11-28;2017-1	ATM	chr11	108158448	108158448	http://gnomad.b	1.39E-05	NFE	7.35E-05	0		0	0	https://www.ncbi.nlm.nih.gov/clinvar/variation/216212/
NM_000051.3(ATM):c.4146A>G	concordant	unreportable	unreportable	185040	0	1	SCV000558420	Likely benign	Invitae	3/14/2017	ATM	chr11	108159740	108159740	http://gnomad.b	3.06E-05	AFR	0.0001307360071	0		0	0	https://www.ncbi.nlm.nih.gov/clinvar/variation/185040/
NM_000051.3(ATM):c.4184A>G	concordant	unreportable	unreportable	453506	0	1	SCV000622480	Uncertain significance	Invitae	10/5/2017	ATM	chr11	108159778	108159778		2.78E-06					0	0	https://www.ncbi.nlm.nih.gov/clinvar/variation/453506/
NM_000051.3(ATM):c.4236+12T>C	concordant	unreportable	unreportable	490561	0	1	SCV000687529	Likely benign	Color Genomics, In	12/21/2017	ATM	chr11	108159842	108159842	http://gnomad.b	2.78E-06	NFE	9.14E-06	0		0	0	https://www.ncbi.nlm.nih.gov/clinvar/variation/490561/
NM_000051.3(ATM):c.4375G>A	concordant	unreportable	unreportable	127387	0	5	SCV000682200;S	Uncertain significance;Unc	Color Genomics, In	2017-10-26;2017-0	ATM	chr11	108160467	108160467	http://gnomad.b	9.47E-05	NFE	9.86E-05	0		0	0	https://www.ncbi.nlm.nih.gov/clinvar/variation/127387/
NM_000051.3(ATM):c.4398A>G	concordant	unreportable	unreportable	184024	0	3	SCV000682204;S	Likely benign;Likely benign	Color Genomics, In	2017-10-26;2017-1	ATM	chr11	108160490	108160490		2.78E-06					0	0	https://www.ncbi.nlm.nih.gov/clinvar/variation/184024/
NM_000051.3(ATM):c.4437-7A>G	concordant	unreportable	unreportable	382191	0	3	SCV000682207;S	Likely benign;Likely benign	Color Genomics, In	2017-10-26;2017-1	ATM	chr11	108163339	108163339	http://gnomad.b	1.11E-05	SAS	6.51E-05	0		0	0	https://www.ncbi.nlm.nih.gov/clinvar/variation/382191/
NM_000051.3(ATM):c.4466G>A	concordant	unreportable	unreportable	181959	0	2	SCV000687551;S	Uncertain significance;Unc	Color Genomics, In	2017-12-21;2017-0	ATM	chr11	108163375	108163375	http://gnomad.b	1.67E-05	NFE	1.79E-05	0		0	0	https://www.ncbi.nlm.nih.gov/clinvar/variation/181959/
NM_000051.3(ATM):c.4477C>T	concordant	unreportable	unreportable	143012	0	2	SCV000566354;S	Uncertain significance;Unc	GeneDx;Invitae	2017-11-28;2017-1	ATM	chr11	108163386	108163386	http://gnomad.b	2.23E-05	AFR	0.0002613700053	0		0	0	https://www.ncbi.nlm.nih.gov/clinvar/variation/143012/
NM_000051.3(ATM):c.4703A>G	concordant	unreportable	unreportable	142472	0	2	SCV000566386;S	Uncertain significance;Unc	GeneDx;Invitae	2017-11-28;2017-1	ATM	chr11	108164131	108164131	http://gnomad.b	1.95E-05	NFE	8.98E-05	0		0	0	https://www.ncbi.nlm.nih.gov/clinvar/variation/142472/
NM_000051.3(ATM):c.4792C>A	concordant	unreportable	unreportable	181962	0	4	SCV000682223;S	Uncertain significance;Unc	Color Genomics, In	2017-10-26;2017-0	ATM	chr11	108165669	108165669	http://gnomad.b	1.39E-05	AFR	0.0001960780064	0		0	0	https://www.ncbi.nlm.nih.gov/clinvar/variation/181962/
NM_000051.3(ATM):c.5005+7_5005+	concordant	unreportable	unreportable	135759	0	4	SCV000682238;S	Likely benign;Likely benign	Color Genomics, In	2017-10-26;2017-0	ATM	chr11	108168115	108168117	http://gnomad.b	0.0001336660132	SAS	0.001299800002	1		0	0	https://www.ncbi.nlm.nih.gov/clinvar/variation/135759/
NM_000051.3(ATM):c.5005+14A>T	concordant	unreportable	unreportable	377515	0	2	SCV000682236;S	Likely benign;Likely benign	Color Genomics, In	2017-10-26;2017-1	ATM	chr11	108168123	108168123	http://gnomad.b	1.11E-05	NFE	5.42E-05	0		0	0	https://www.ncbi.nlm.nih.gov/clinvar/variation/377515/
NM_000051.3(ATM):c.5005+15T>A	concordant	unreportable	unreportable	377516	0	1	SCV000682237	Likely benign	Color Genomics, In	10/26/2017	ATM	chr11	108168124	108168124	http://gnomad.b	4.73E-05	ASJ	0.00122224004	0		0	0	https://www.ncbi.nlm.nih.gov/clinvar/variation/377516/
NM_000051.3(ATM):c.5005+17C>G	concordant	unreportable	unreportable	377517	0	2	SCV000687595;S	Likely benign;Likely benign	Color Genomics, In	2017-12-21;2017-1	ATM	chr11	108168126	108168126	http://gnomad.b	2.78E-06	NFE	2.71E-05	0		0	0	https://www.ncbi.nlm.nih.gov/clinvar/variation/377517/
NM_000051.3(ATM):c.5009C>T	concordant	unreportable	unreportable	127397	0	3	SCV000682242;S	Uncertain significance;Unc	Color Genomics, In	2017-10-26;2017-1	ATM	chr11	108170444	108170444	http://gnomad.b	3.06E-05	NFE	2.69E-05	0		0	0	https://www.ncbi.nlm.nih.gov/clinvar/variation/127397/
NM_000051.3(ATM):c.5056A>G	concordant	unreportable	unreportable	142682	0	3	SCV000682247;S	Uncertain significance;Unc	Color Genomics, In	2017-10-26;2017-1	ATM	chr11	108170491	108170491	http://gnomad.b	1.95E-05	NFE	2.69E-05	0		0	0	https://www.ncbi.nlm.nih.gov/clinvar/variation/142682/
NM_000051.3(ATM):c.5189G>A	concordant	unreportable	unreportable	127402	0	3	SCV000687609;S	Uncertain significance;Unc	Color Genomics, In	2017-12-21;2017-1	ATM	chr11	108172386	108172386	http://gnomad.b	9.19E-05	NFE	3.59E-05	0		0	0	https://www.ncbi.nlm.nih.gov/clinvar/variation/127402/
NM_000051.3(ATM):c.5319+8A>G	concordant	unreportable	unreportable	414543	0	1	SCV000558324	Likely benign	Invitae	3/14/2017	ATM	chr11	108172524	108172524	http://gnomad.b	5.57E-06	NFE	1.80E-05	0		0	0	https://www.ncbi.nlm.nih.gov/clinvar/variation/414543/
NM_000051.3(ATM):c.5352C>T	concordant	unreportable	unreportable	183764	0	2	SCV000682264;S	Likely benign;Likely benign	Color Genomics, In	2017-10-26;2017-1	ATM	chr11	108173612	108173612	http://gnomad.b	5.01E-05	AMR	8.94E-05	0		0	0	https://www.ncbi.nlm.nih.gov/clinvar/variation/183764/
NM_000051.3(ATM):c.5489T>C	concordant	unreportable	unreportable	127408	0	3	SCV000682267;S	Uncertain significance;Unc	Color Genomics, In	2017-10-26;2017-1	ATM	chr11	108173749	108173749	http://gnomad.b	4.18E-05	AFR	0.0001962069946	0		0	0	https://www.ncbi.nlm.nih.gov/clinvar/variation/127408/
NM_000051.3(ATM):c.5675-13T>A	concordant	unreportable	unreportable	489562	0	1	SCV000682281	Uncertain significance	Color Genomics, In	10/26/2017	ATM	chr11	108178611	108178611	http://gnomad.b	8.35E-06	AFR	0.0001309929939	0		0	0	https://www.ncbi.nlm.nih.gov/clinvar/variation/489562/
NM_000051.3(ATM):c.5750G>C	concordant	unreportable	unreportable	140925	0	3	SCV000687638;S	Uncertain significance;Unc	Color Genomics, In	2017-12-21;2017-1	ATM	chr11	108178699	108178699	http://gnomad.b	2.78E-06	NFE	2.69E-05	0		0	0	https://www.ncbi.nlm.nih.gov/clinvar/variation/140925/
NM_000051.3(ATM):c.6040G>C	concordant	unreportable	unreportable	185967	0	1	SCV000622633	Uncertain significance	Invitae	10/5/2017	ATM	chr11	108186583	108186583	http://gnomad.b	8.35E-06	AFR	6.53E-05	0		0	0	https://www.ncbi.nlm.nih.gov/clinvar/variation/185967/
NM_000051.3(ATM):c.6042G>A	concordant	unreportable	unreportable	184453	0	3	SCV000687668;S	Likely benign;Likely benign	Color Genomics, In	2017-12-21;2017-1	ATM	chr11	108186585	108186585	http://gnomad.b	8.35E-06	AFR	0.0001306850027	0		0	0	https://www.ncbi.nlm.nih.gov/clinvar/variation/184453/
NM_000051.3(ATM):c.6147T>C	concordant	unreportable	unreportable	187461	0	1	SCV000283008	Likely benign	Invitae	10/5/2017	ATM	chr11	108186789	108186789	http://gnomad.b	1.39E-05	AFR	0.0003267120046	0		0	0	https://www.ncbi.nlm.nih.gov/clinvar/variation/187461/
NM_000051.3(ATM):c.6179G>C	concordant	unreportable	unreportable	230875	0	2	SCV000564649;S	Uncertain significance;Unc	GeneDx;Invitae	2017-11-28;2017-0	ATM	chr11	108186821	108186821	http://gnomad.b	1.67E-05	AFR	0.0001961810049	0		0	0	https://www.ncbi.nlm.nih.gov/clinvar/variation/230875/
NM_000051.3(ATM):c.6396A>G	concordant	unreportable	unreportable	184369	0	4	SCV000682332;S	Likely benign;Likely benign	Color Genomics, In	2017-10-26;2017-1	ATM	chr11	108190729	108190729	http://gnomad.b	3.34E-05	AFR	6.54E-05	0		0	0	https://www.ncbi.nlm.nih.gov/clinvar/variation/184369/
NM_000051.3(ATM):c.6452+17A>G	concordant	unreportable	unreportable	490655	0	1	SCV000687698	Likely benign	Color Genomics, In	12/21/2017	ATM	chr11	108190802	108190802	http://gnomad.b	2.78E-06	AFR	6.57E-05	0		0	0	https://www.ncbi.nlm.nih.gov/clinvar/variation/490655/
NM_000051.3(ATM):c.6475T>G	concordant	unreportable	unreportable	230532	0	4	SCV000687706;S	Uncertain significance;Unc	Color Genomics, In	2017-12-21;2017-1	ATM	chr11	108192050	108192050	http://gnomad.b	1.39E-05	AFR	0.0004573969927	0		0	0	https://www.ncbi.nlm.nih.gov/clinvar/variation/230532/
NM_000051.3(ATM):c.6486C>T	concordant	unreportable	unreportable	387775	0	2	SCV000529905;S	Likely benign;Likely benign	GeneDx;Invitae	2017-11-28;2017-1	ATM	chr11	108192061	108192061	http://gnomad.b	2.78E-06	AFR	0.0001306850027	0		0	0	https://www.ncbi.nlm.nih.gov/clinvar/variation/387775/
NM_000051.3(ATM):c.6537T>G	concordant	unreportable	unreportable	186221	0	5	SCV000682347;S	Uncertain significance;Unc	Color Genomics, In	2017-10-26;2017-0	ATM	chr11	108192112	108192112	http://gnomad.b	3.62E-05	AFR	0.0005880820099	0		0	0	https://www.ncbi.nlm.nih.gov/clinvar/variation/186221/
NM_000051.3(ATM):c.6572+11C>T	concordant	unreportable	unreportable	490663	0	1	SCV000687710	Likely benign	Color Genomics, In	12/21/2017	ATM	chr11	108192158	108192158	http://gnomad.b	1.95E-05	AMR	8.93E-05	0		0	0	https://www.ncbi.nlm.nih.gov/clinvar/variation/490663/
NM_000051.3(ATM):c.6573-11G>A	concordant	unreportable	unreportable	419710	0	1	SCV000567694	Uncertain significance	GeneDx	11/28/2017	ATM	chr11	108196026	108196026	http://gnomad.b	5.57E-06	AFR	6.84E-05	0		0	0	https://www.ncbi.nlm.nih.gov/clinvar/variation/419710/
NM_000051.3(ATM):c.7036G>A	concordant	unreportable	unreportable	220756	0	2	SCV000687751;S	Uncertain significance;Unc	Color Genomics, In	2017-12-21;2016-0	ATM	chr11	108198432	108198432		2.78E-06					0	0	https://www.ncbi.nlm.nih.gov/clinvar/variation/220756/
NM_000051.3(ATM):c.7122A>C	concordant	unreportable	unreportable	181887	0	2	SCV000209637;S	Uncertain significance;Unc	GeneDx;Invitae	2017-11-28;2017-1	ATM	chr11	108199780	108199780		1.39E-05					0	0	https://www.ncbi.nlm.nih.gov/clinvar/variation/181887/
NM_000051.3(ATM):c.7131T>C	concordant	unreportable	unreportable	186547	0	2	SCV000527373;S	Likely benign;Likely benign	GeneDx;Invitae	2017-11-28;2017-1	ATM	chr11	108199789	108199789	http://gnomad.b	5.57E-06	NFE	8.95E-06	0		0	0	https://www.ncbi.nlm.nih.gov/clinvar/variation/186547/
NM_000051.3(ATM):c.7224G>A	concordant	unreportable	unreportable	215593	0	2	SCV000682397;S	Likely benign;Likely benign	Color Genomics, In	2017-10-26;2017-0	ATM	chr11	108199882	108199882	http://gnomad.b	4.73E-05	AFR	0.0007847240195	0		0	0	https://www.ncbi.nlm.nih.gov/clinvar/variation/215593/
NM_000051.3(ATM):c.7244C>G	concordant	unreportable	unreportable	236768	0	3	SCV000682400;S	Uncertain significance;Unc	Color Genomics, In	2017-10-26;2017-1	ATM	chr11	108199902	108199902	http://gnomad.b	3.90E-05	AFR	0.0004577559885	0		0	0	https://www.ncbi.nlm.nih.gov/clinvar/variation/236768/
NM_000051.3(ATM):c.7793G>A	concordant	unreportable	unreportable	229826	0	1	SCV000546708	Uncertain significance	Invitae	10/5/2017	ATM	chr11	108203493	108203493	http://gnomad.b	1.39E-05	AFR	6.55E-05	0		0	0	https://www.ncbi.nlm.nih.gov/clinvar/variation/229826/
NM_000051.3(ATM):c.7808A>G	concordant	unreportable	unreportable	216233	0	1	SCV000254151	Uncertain significance	Invitae	1/6/2016	ATM	chr11	108203508	108203508		8.35E-06					0	0	https://www.ncbi.nlm.nih.gov/clinvar/variation/216233/
NM_000051.3(ATM):c.7902T>G	concordant	unreportable	unreportable	220528	0	2	SCV000682441;S	Uncertain significance;Unc	Color Genomics, In	2017-10-26;2017-0	ATM	chr11	108203602	108203602	http://gnomad.b	1.39E-05	AFR	6.54E-05	0		0	0	https://www.ncbi.nlm.nih.gov/clinvar/variation/220528/
NM_000051.3(ATM):c.7988T>C	concordant	unreportable	unreportable	133635	0	2	SCV000209650;S	Uncertain significance;Unc	GeneDx;Invitae	2017-11-28;2017-1	ATM	chr11	108204673	108204673	http://gnomad.b	3.34E-05	AFR	0.0005883889971	0		0	0	https://www.ncbi.nlm.nih.gov/clinvar/variation/133635/
NM_000051.3(ATM):c.8152-17G>A	concordant	unreportable	unreportable	379552	0	2	SCV000682460;S	Likely benign;Likely benign	Color Genomics, In	2017-10-26;2017-1	ATM	chr11	108206555	108206555	http://gnomad.b	5.57E-06	AMR	8.96E-05	0		0	0	https://www.ncbi.nlm.nih.gov/clinvar/variation/379552/
NM_000051.3(ATM):c.8154C>T	concordant	unreportable	unreportable	185736	0	3	SCV000682462;S	Likely benign;Likely benign	Color Genomics, In	2017-10-26;2017-1	ATM	chr11	108206574	108206574	http://gnomad.b	2.78E-05	AFR	0.000261609006	0		0	0	https://www.ncbi.nlm.nih.gov/clinvar/variation/185736/
NM_000051.3(ATM):c.8155C>T	concordant	unreportable	unreportable	185832	0	2	SCV000682463;S	Uncertain significance;Unc	Color Genomics, In	2017-10-26;2017-1	ATM	chr11	108206575	108206575	http://gnomad.b	1.95E-05	NFE	1.80E-05	0		0	0	https://www.ncbi.nlm.nih.gov/clinvar/variation/185832/
NM_000051.3(ATM):c.8418+13C>T	concordant	unreportable	unreportable	181842	0	1	SCV000682482	Likely benign	Color Genomics, In	10/26/2017	ATM	chr11	108214111	108214111	http://gnomad.b	2.78E-05	AFR	0.0003922079923	0		0	0	https://www.ncbi.nlm.nih.gov/clinvar/variation/181842/
NM_000051.3(ATM):c.8584+10T>C	concordant	unreportable	unreportable	236789	0	2	SCV000682503;S	Likely benign;Likely benign	Color Genomics, In	2017-10-26;2017-1	ATM	chr11	108216645	108216645	http://gnomad.b	2.23E-05	AFR	0.0005886969739	0		0	0	https://www.ncbi.nlm.nih.gov/clinvar/variation/236789/
NM_000051.3(ATM):c.8671+9T>G	concordant	unreportable	unreportable	136433	0	3	SCV000537424;S	Likely benign;Likely benign	Color Genomics, In	2017-01-26;2017-1	ATM	chr11	108218101	108218101	http://gnomad.b	7.52E-05	ASJ	0.0004082469968	0		0	0	https://www.ncbi.nlm.nih.gov/clinvar/variation/136433/
NM_000051.3(ATM):c.8906A>G	concordant	unreportable	unreportable	229994	0	1	SCV000617086	Uncertain significance	GeneDx	11/28/2017	ATM	chr11	108235864	108235864	http://gnomad.b	5.57E-06	AFR	6.53E-05	0		0	0	https://www.ncbi.nlm.nih.gov/clinvar/variation/229994/
NM_000051.3(ATM):c.8965C>G	concordant	unreportable	unreportable	141036	0	3	SCV000682529;S	Uncertain significance;Unc	Color Genomics, In	2017-10-26;2017-1	ATM	chr11	108235923	108235923	http://gnomad.b	3.06E-05	NFE	2.69E-05	0		0	0	https://www.ncbi.nlm.nih.gov/clinvar/variation/141036/
NM_000051.3(ATM):c.9119T>C	concordant	unreportable	unreportable	187317	0	2	SCV000682540;S	Uncertain significance;Unc	Color Genomics, In	2017-10-26;2017-1	ATM	chr11	108236183	108236183	http://gnomad.b	5.57E-06	NFE	2.69E-05	0		0	0	https://www.ncbi.nlm.nih.gov/clinvar/variation/187317/
NM_000051.3(ATM):c.9166G>T	concordant	unreportable	unreportable	181908	0	3	SCV000209673;S	Uncertain significance;Unc	GeneDx;Invitae;Lal	2017-11-28;2016-0	ATM	chr11	108236230	108236230		1.95E-05					0	0	https://www.ncbi.nlm.nih.gov/clinvar/variation/181908/
NM_001164277.1(SLC37A4):c.784+6A	concordant	unreportable	unreportable	386606	0	1	SCV000528339	Likely benign	GeneDx	11/28/2017	SLC37A4	chr11	118897641	118897641	http://gnomad.b	6.98E-05	AFR	0.001913690008	0		0	0	https://www.ncbi.nlm.nih.gov/clinvar/variation/386606/
NM_024685.3(BBS10):c.393G>A	concordant	unreportable	unreportable	310496	0	1	SCV000381156	Uncertain significance	Illumina Clinical Se	10/18/2016	BBS10	chr12	76741372	76741372	http://gnomad.b	1.12E-05	FIN	9.00E-05	0		0	0	https://www.ncbi.nlm.nih.gov/clinvar/variation/310496/
NM_024685.3(BBS10):c.42G>A	concordant	unreportable	unreportable	310499	0	2	SCV000381161;S	Uncertain significance;Likel	Illumina Clinical Se	2016-10-18;2017-0	BBS10	chr12	76742097	76742097	http://gnomad.b	4.20E-05	NFE	7.65E-05	0		0	0	https://www.ncbi.nlm.nih.gov/clinvar/variation/310499/
NM_000277.1(PAH):c.694C>T	concordant	reportable	reportable	102788	1	0	SCV000601715	Pathogenic	Quest Diagnostics	8/1/2017	PAH	chr12	103248926	103248926	http://gnomad.b	2.74E-06	AFR	6.54E-05	0		0	0	https://www.ncbi.nlm.nih.gov/clinvar/variation/102788/
NM_000277.1(PAH):c.442-5C>G	concordant	reportable	reportable	102677	1	0	SCV000700534	Pathogenic	EGL Genetic Diagn	6/30/2017	PAH	chr12	103260446	103260446	http://gnomad.b	5.48E-06	NFE	5.38E-05	0		0	0	https://www.ncbi.nlm.nih.gov/clinvar/variation/102677/
NM_000277.1(PAH):c.441+5G>T	concordant	reportable	reportable	92742	3	0	SCV000611225;S	Likely pathogenic;Pathoger	Fulgent Genetics,F	2017-05-23;2017-1	PAH	chr12	103271235	103271235	http://gnomad.b	1.64E-05	AMR	0.0001191109986	0		0	0	https://www.ncbi.nlm.nih.gov/clinvar/variation/92742/

Supplemental Table 2

NM_000277.1(PAH):c.2T>C	concordant	reportable	reportable	203873	1	0	SCV000239097	Pathogenic	GeneDx	11/28/2017	PAH	chr12	103310907	103310907	http://gnomad.b	5.48E-06	AFR	0.0001306850027	0		0	0	https://www.ncbi.nlm.nih.gov/clinvar/variation/203873/
NM_052845.3(MMAB):c.624C>T	concordant	unreportable	unreportable	387984	0	1	SCV000530184	Likely benign	GeneDx	11/28/2017	MMAB	chr12	109996921	109996921	http://gnomad.b	0.0002799910403	AFR	0.003659649985	0		0	0	https://www.ncbi.nlm.nih.gov/clinvar/variation/387984/
NM_052845.3(MMAB):c.561C>T	concordant	unreportable	unreportable	307079	0	1	SCV000375742	Uncertain significance	Illumina Clinical Se	10/18/2016	MMAB	chr12	109998868	109998868	http://gnomad.b	4.00E-05	SAS	9.75E-05	0		0	0	https://www.ncbi.nlm.nih.gov/clinvar/variation/307079/
NM_052845.3(MMAB):c.79C>T	concordant	unreportable	unreportable	284358	0	1	SCV000336949	Uncertain significance	EGL Genetic Diagn	6/30/2017	MMAB	chr12	110011207	110011207	http://gnomad.b	3.20E-05	AFR	0.0002119540004	0		0	0	https://www.ncbi.nlm.nih.gov/clinvar/variation/284358/
NM_052845.3(MMAB):c.-4G>C	concordant	unreportable	unreportable	307082	0	1	SCV000375750	Uncertain significance	Illumina Clinical Se	10/18/2016	MMAB	chr12	110011289	110011289	http://gnomad.b	0.001679946242	NFE	0.002289080061	0		0	0	https://www.ncbi.nlm.nih.gov/clinvar/variation/307082/
NM_000017.2(ACADS):c.624+8C>T	concordant	unreportable	unreportable	307438	0	3	SCV000531456;S	Likely benign;Uncertain sig	GeneDx;Illumina C	2017-11-28;2016-1	ACADS	chr12	121175799	121175799	http://gnomad.b	0.0002435460302	ASJ	0.0009142620256	0		0	0	https://www.ncbi.nlm.nih.gov/clinvar/variation/307438/
NM_014363.4(SACS):c.13738_13739d	concordant	unreportable	unreportable	504206	0	1	SCV000710518	Uncertain significance	GeneDx	3/26/2018	SACS	chr13	23904275	23904277		2.96E-05					0	0	https://www.ncbi.nlm.nih.gov/clinvar/variation/504206/
NM_014363.4(SACS):c.13538G>A	concordant	unreportable	unreportable	373337	0	1	SCV000491924	Uncertain significance	GeneDx	12/21/2016	SACS	chr13	23904477	23904477	http://gnomad.b	4.74E-05	NFE	5.37E-05	0		0	0	https://www.ncbi.nlm.nih.gov/clinvar/variation/373337/
NM_014363.4(SACS):c.9550C>T	concordant	unreportable	unreportable	448229	0	2	SCV000614994;S	Uncertain significance;Unc	Athena Diagnostics	2017-08-17;2017-1	SACS	chr13	23908465	23908465	http://gnomad.b	7.11E-05	AFR	0.0002629499941	0		0	0	https://www.ncbi.nlm.nih.gov/clinvar/variation/448229/
NM_014363.4(SACS):c.7273C>T	concordant	reportable	reportable	370415	1	0	SCV000710509	Pathogenic	GeneDx	3/26/2018	SACS	chr13	23910742	23910742	http://gnomad.b	8.89E-06	AFR	6.55E-05	0		0	0	https://www.ncbi.nlm.nih.gov/clinvar/variation/370415/
NM_014363.4(SACS):c.6485A>G	concordant	unreportable	unreportable	451915	0	1	SCV000620676	Uncertain significance	GeneDx	11/28/2017	SACS	chr13	23911530	23911530	http://gnomad.b	2.96E-06	NFE	9.01E-06	0		0	0	https://www.ncbi.nlm.nih.gov/clinvar/variation/451915/
NM_014363.4(SACS):c.6266C>T	concordant	unreportable	unreportable	448214	0	1	SCV000614970	Uncertain significance	Athena Diagnostics	8/17/2017	SACS	chr13	23911749	23911749	http://gnomad.b	6.82E-05	SAS	9.76E-05	0		0	0	https://www.ncbi.nlm.nih.gov/clinvar/variation/448214/
NM_014363.4(SACS):c.6084A>T	concordant	unreportable	unreportable	448213	0	1	SCV000614969	Uncertain significance	Athena Diagnostics	8/17/2017	SACS	chr13	23911931	23911931	http://gnomad.b	2.96E-05	AFR	0.0005916379741	0		0	0	https://www.ncbi.nlm.nih.gov/clinvar/variation/448213/
NM_014363.4(SACS):c.5732C>T	concordant	unreportable	unreportable	444312	0	1	SCV000614963	Uncertain significance	Athena Diagnostics	8/17/2017	SACS	chr13	23912283	23912283	http://gnomad.b	6.52E-05	SAS	9.75E-05	0		0	0	https://www.ncbi.nlm.nih.gov/clinvar/variation/444312/
NM_000053.3(ATP7B):c.4022-19C>T	concordant	unreportable	unreportable	495418	0	1	SCV000694459	Uncertain significance	Laboratory Corpor	1/25/2018	ATP7B	chr13	52509850	52509850	http://gnomad.b	3.09E-05	NFE	0.0002811389859	0		0	0	https://www.ncbi.nlm.nih.gov/clinvar/variation/495418/
NM_000053.3(ATP7B):c.3583G>A	concordant	unreportable	unreportable	444317	0	1	SCV000694452	Uncertain significance	Laboratory Corpor	1/25/2018	ATP7B	chr13	52513303	52513303	http://gnomad.b	0.0001851893421	NFE	0.0003583270009	0		0	0	https://www.ncbi.nlm.nih.gov/clinvar/variation/444317/
NM_000053.3(ATP7B):c.3243+5G>A	concordant	unreportable	unreportable	377539	0	1	SCV000512211	Uncertain significance	GeneDx	11/28/2017	ATP7B	chr13	52518240	52518240	http://gnomad.b	8.70E-05	SAS	0.002209230093	0		0	0	https://www.ncbi.nlm.nih.gov/clinvar/variation/377539/
NM_000053.3(ATP7B):c.1839C>T	concordant	unreportable	unreportable	392131	0	2	SCV000535346;S	Likely benign;Uncertain sig	GeneDx;Laborator	2017-11-28;2018-0	ATP7B	chr13	52539038	52539038	http://gnomad.b	0.0001010123684	EAS	0.0001159550011	0		0	0	https://www.ncbi.nlm.nih.gov/clinvar/variation/392131/
NM_000053.3(ATP7B):c.1829C>T	concordant	unreportable	unreportable	439430	0	1	SCV000602610	Uncertain significance	ARUP Laboratories	6/30/2017	ATP7B	chr13	52539048	52539048	http://gnomad.b	7.58E-05	AMR	0.0003573340073	0		0	0	https://www.ncbi.nlm.nih.gov/clinvar/variation/439430/
NM_000053.3(ATP7B):c.1543+13C>T	concordant	unreportable	unreportable	312390	0	2	SCV000522340;S	Likely benign;Uncertain sig	GeneDx;Illumina C	2017-11-28;2016-1	ATP7B	chr13	52544615	52544615	http://gnomad.b	0.0002721722149	NFE	0.001297289971	1		0	0	https://www.ncbi.nlm.nih.gov/clinvar/variation/312390/
NM_000053.3(ATP7B):c.999G>T	concordant	unreportable	unreportable	500187	0	1	SCV000706021	Uncertain significance	EGL Genetic Diagn	6/30/2017	ATP7B	chr13	52548357	52548357	http://gnomad.b	2.81E-06	AFR	6.54E-05	0		0	0	https://www.ncbi.nlm.nih.gov/clinvar/variation/500187/
NM_000053.3(ATP7B):c.51+4A>T	concordant	reportable	reportable	312401	1	0	SCV000384689	Likely pathogenic	Illumina Clinical Se	10/18/2016	ATP7B	chr13	52585419	52585419	http://gnomad.b	1.12E-05	AMR	2.98E-05	0		0	0	https://www.ncbi.nlm.nih.gov/clinvar/variation/312401/
NM_006493.2(CLN5):c.231G>A	concordant	reportable	reportable	451600	1	0	SCV000620322	Likely pathogenic	GeneDx	11/28/2017	CLN5	chr13	77566317	77566317	http://gnomad.b	2.80E-06	AFR	7.64E-05	0		0	0	https://www.ncbi.nlm.nih.gov/clinvar/variation/451600/
NM_006493.2(CLN5):c.673A>G	concordant	unreportable	unreportable	205146	0	1	SCV000240647	Uncertain significance	GeneDx	11/28/2017	CLN5	chr13	77570223	77570223	http://gnomad.b	6.71E-05	NFE	8.06E-05	0		0	0	https://www.ncbi.nlm.nih.gov/clinvar/variation/205146/
NM_006493.2(CLN5):c.803A>C	concordant	unreportable	unreportable	409274	0	2	SCV000589476;S	Uncertain significance;Unc	GeneDx;Invitae	2017-11-28;2017-1	CLN5	chr13	77574683	77574683	http://gnomad.b	5.04E-05	AFR	0.0001961039961	0		0	0	https://www.ncbi.nlm.nih.gov/clinvar/variation/409274/
NM_006493.2(CLN5):c.920G>A	concordant	unreportable	unreportable	423209	0	1	SCV000572871	Uncertain significance	GeneDx	11/28/2017	CLN5	chr13	77574800	77574800	http://gnomad.b	8.39E-06	AFR	6.54E-05	0		0	0	https://www.ncbi.nlm.nih.gov/clinvar/variation/423209/
NM_006493.2(CLN5):c.1192C>T	concordant	unreportable	unreportable	128780	0	3	SCV000240631;S	Uncertain significance;Unc	GeneDx;Illumina C	2017-11-28;2016-1	CLN5	chr13	77575072	77575072	http://gnomad.b	0.000148262531	NFE	0.0001865600061	0		0	0	https://www.ncbi.nlm.nih.gov/clinvar/variation/128780/
NM_000153.3(GALC):c.1567C>A	concordant	unreportable	unreportable	286665	0	1	SCV000340196	Uncertain significance	EGL Genetic Diagn	6/30/2017	GALC	chr14	88412000	88412000	http://gnomad.b	4.20E-05	NFE	8.99E-06	0		0	0	https://www.ncbi.nlm.nih.gov/clinvar/variation/286665/
NM_000153.3(GALC):c.1418G>A	concordant	unreportable	unreportable	314748	0	1	SCV000389243	Uncertain significance	Illumina Clinical Se	10/18/2016	GALC	chr14	88414143	88414143	http://gnomad.b	1.96E-05	AFR	6.55E-05	0		0	0	https://www.ncbi.nlm.nih.gov/clinvar/variation/314748/
NM_000153.3(GALC):c.329-10_329-8d	concordant	unreportable	unreportable	314756	0	1	SCV000389262	Uncertain significance	Illumina Clinical Se	10/18/2016	GALC	chr14	88452953	88452956	http://gnomad.b	0.0004615178062	NFE	0.001912739943	0		0	0	https://www.ncbi.nlm.nih.gov/clinvar/variation/314756/
NM_133647.1(SLC12A6):c.3228-15T>	concordant	unreportable	unreportable	315607	0	1	SCV000390298	Uncertain significance	Illumina Clinical Se	10/18/2016	SLC12A6	chr15	34527530	34527530	http://gnomad.b	5.93E-06	AMR	5.96E-05	0		0	0	https://www.ncbi.nlm.nih.gov/clinvar/variation/315607/
NM_133647.1(SLC12A6):c.2163-14del	concordant	unreportable	unreportable	315610	0	1	SCV000390305	Uncertain significance	Illumina Clinical Se	10/18/2016	SLC12A6	chr15	34533551	34533552	http://gnomad.b	1.78E-05	NFE	0.0002871289907	0		0	0	https://www.ncbi.nlm.nih.gov/clinvar/variation/315610/
NM_133647.1(SLC12A6):c.1943+15T>	concordant	unreportable	unreportable	315612	0	1	SCV000390307	Uncertain significance	Illumina Clinical Se	10/18/2016	SLC12A6	chr15	34537471	34537471	http://gnomad.b	0.0001185339718	NFE	0.000277618994	0		0	0	https://www.ncbi.nlm.nih.gov/clinvar/variation/315612/
NM_133647.1(SLC12A6):c.543+8C>G	concordant	unreportable	unreportable	387888	0	1	SCV000530068	Likely benign	GeneDx	11/28/2017	SLC12A6	chr15	34551006	34551006	http://gnomad.b	1.19E-05	AFR	0.0005880820099	0		0	0	https://www.ncbi.nlm.nih.gov/clinvar/variation/387888/
NM_002225.3(IVD):c.793+13G>A	concordant	unreportable	unreportable	315799	0	2	SCV000531657;S	Likely benign;Uncertain sig	GeneDx;Illumina C	2017-11-28;2016-1	IVD	chr15	40705299	40705299	http://gnomad.b	4.48E-05	EAS	0.0004641989944	0		0	0	https://www.ncbi.nlm.nih.gov/clinvar/variation/315799/
NM_002225.3(IVD):c.1010T>C	concordant	unreportable	unreportable	315802	0	1	SCV000390866	Uncertain significance	Illumina Clinical Se	10/18/2016	IVD	chr15	40708317	40708317	http://gnomad.b	6.73E-05	EAS	0.0003478660074	0		0	0	https://www.ncbi.nlm.nih.gov/clinvar/variation/315802/
NM_000137.2(FAH):c.81+12G>A	concordant	unreportable	unreportable	317203	0	1	SCV000394037	Uncertain significance	Illumina Clinical Se	10/18/2016	FAH	chr15	80445489	80445489	http://gnomad.b	0.000432777935	ASJ	0.00701077003	0		0	0	https://www.ncbi.nlm.nih.gov/clinvar/variation/317203/
NM_000137.2(FAH):c.249G>A	concordant	unreportable	unreportable	317209	0	1	SCV000394045	Uncertain significance	Illumina Clinical Se	10/18/2016	FAH	chr15	80452154	80452154	http://gnomad.b	0.0001581303993	SAS	0.001624329947	0		0	0	https://www.ncbi.nlm.nih.gov/clinvar/variation/317209/
NM_000137.2(FAH):c.706+9T>C	concordant	unreportable	unreportable	386297	0	1	SCV000527874	Likely benign	GeneDx	11/28/2017	FAH	chr15	80464599	80464599	http://gnomad.b	4.99E-05	AFR	0.001373089966	0		0	0	https://www.ncbi.nlm.nih.gov/clinvar/variation/386297/
NM_000137.2(FAH):c.707-7_707-5del	concordant	unreportable	unreportable	500962	0	1	SCV000707139	Uncertain significance	EGL Genetic Diagn	6/30/2017	FAH	chr15	80465343	80465346	http://gnomad.b	3.61E-05	AFR	0.001176159945	0		0	0	https://www.ncbi.nlm.nih.gov/clinvar/variation/500962/
NM_000137.2(FAH):c.747A>G	concordant	unreportable	unreportable	317213	0	2	SCV000704032;S	Uncertain significance;Unc	EGL Genetic Diagn	2017-06-30;2016-1	FAH	chr15	80465396	80465396	http://gnomad.b	5.55E-05	AFR	0.001176159945	0		0	0	https://www.ncbi.nlm.nih.gov/clinvar/variation/317213/
NM_000137.2(FAH):c.934G>A	concordant	unreportable	unreportable	499798	0	1	SCV000705475	Uncertain significance	EGL Genetic Diagn	6/30/2017	FAH	chr15	80469899	80469899	http://gnomad.b	1.11E-05	NFE	8.95E-06	0		0	0	https://www.ncbi.nlm.nih.gov/clinvar/variation/499798/
NM_000137.2(FAH):c.961-17G>A	concordant	unreportable	unreportable	382107	0	1	SCV000521902	Likely benign	GeneDx	11/28/2017	FAH	chr15	80472449	80472449	http://gnomad.b	0.0001664530519	AFR	0.004003149923	2		0	0	https://www.ncbi.nlm.nih.gov/clinvar/variation/382107/
NM_000137.2(FAH):c.961-15G>A	concordant	unreportable	unreportable	388746	0	1	SCV000531107	Likely benign	GeneDx	11/28/2017	FAH	chr15	80472451	80472451	http://gnomad.b	7.49E-05	SAS	0.0001625379955	0		0	0	https://www.ncbi.nlm.nih.gov/clinvar/variation/388746/
NM_000057.2(BLM):c.368A>G	concordant	unreportable	unreportable	127503	0	1	SCV000543326	Uncertain significance	Invitae	10/5/2017	BLM	chr15	91292866	91292866	http://gnomad.b	5.23E-05	ASJ	0.0001015849994	0		0	0	https://www.ncbi.nlm.nih.gov/clinvar/variation/127503/
NM_000057.2(BLM):c.1936A>G	concordant	unreportable	unreportable	405311	0	1	SCV000543374	Uncertain significance	Invitae	10/5/2017	BLM	chr15	91306249	91306249	http://gnomad.b	2.14E-05	AFR	0.0001964889962	0		0	0	https://www.ncbi.nlm.nih.gov/clinvar/variation/405311/
NM_000243.2(MEFV):c.1957C>T	concordant	unreportable	unreportable	289866	0	2	SCV000344296;S	Uncertain significance;Unc	EGL Genetic Diagn	2017-06-30;2018-0	MEFV	chr16	3293530	3293530	http://gnomad.b	1.68E-05	AFR	0.0001960270019	0		0	0	https://www.ncbi.nlm.nih.gov/clinvar/variation/289866/
NM_000243.2(MEFV):c.1587+7G>A	concordant	unreportable	unreportable	495751	0	1	SCV000696052	Uncertain significance	Laboratory Corpor	1/25/2018	MEFV	chr16	3297009	3297009	http://gnomad.b	1.12E-05	NFE	6.31E-05	0		0	0	https://www.ncbi.nlm.nih.gov/clinvar/variation/495751/
NM_000243.2(MEFV):c.116G>A	concordant	unreportable	unreportable	495750	0	1	SCV000696041	Uncertain significance	Laboratory Corpor	1/25/2018	MEFV	chr16	3306472	3306472	http://gnomad.b	5.60E-06	AFR	0.0001960530062	0		0	0	https://www.ncbi.nlm.nih.gov/clinvar/variation/495750/
NM_000303.2(PMM2):c.339G>A	concordant	unreportable	unreportable	286312	0	1	SCV000339687	Uncertain significance	EGL Genetic Diagn	6/30/2017	PMM2	chr16	8900256	8900256	http://gnomad.b	5.57E-06	AFR	0.0001960270019	0		0	0	https://www.ncbi.nlm.nih.gov/clinvar/variation/286312/
NM_000303.2(PMM2):c.640-12G>A	concordant	unreportable	unreportable	321221	0	1	SCV000399680	Uncertain significance	Illumina Clinical Se	10/18/2016	PMM2	chr16	8941569	8941569	http://gnomad.b	3.90E-05	NFE	8.08E-05	0		0	0	https://www.ncbi.nlm.nih.gov/clinvar/variation/321221/
NM_000303.2(PMM2):c.*19G>A	concordant	unreportable	unreportable	381857	0	1	SCV000521537	Likely benign	GeneDx	11/28/2017	PMM2	chr16	8941701	8941701	http://gnomad.b	9.47E-05	SAS	0.001951980055	2		0	0	https://www.ncbi.nlm.nih.gov/clinvar/variation/381857/
NM_001042432.1(CLN3):c.1087G>A	concordant	unreportable	unreportable	502109	0	1	SCV000708721	Uncertain significance	EGL Genetic Diagn	6/30/2017	CLN3	chr16	28489168	28489168	http://gnomad.b	7.22E-05	AFR	0.0001317519927	0		0	0	https://www.ncbi.nlm.nih.gov/clinvar/variation/502109/
NM_001042432.1(CLN3):c.1033A>T	concordant	unreportable	unreportable	318718	0	3	SCV000567921;S	Uncertain significance;Unc	GeneDx;Illumina C	2017-11-28;2016-1	CLN3	chr16	28493449	28493449	http://gnomad.b	2.50E-05	NFE	5.44E-05	0		0	0	https://www.ncbi.nlm.nih.gov/clinvar/variation/318718/
NM_001042432.1(CLN3):c.677+8G>A	concordant	unreportable	unreportable	318722	0	1	SCV000396328	Uncertain significance	Illumina Clinical Se	10/18/2016	CLN3	chr16	28497660	28497660	http://gnomad.b	8.89E-05	NFE	0.0002119109995	0		0	0	https://www.ncbi.nlm.nih.gov/clinvar/variation/318722/
NM_001042432.1(CLN3):c.436C>T	concordant	unreportable	unreportable	318724	0	1	SCV000396331	Uncertain significance	Illumina Clinical Se	10/18/2016	CLN3	chr16	28498801	28498801	http://gnomad.b	2.78E-06	NFE	1.79E-05	0		0	0	https://www.ncbi.nlm.nih.gov/clinvar/variation/318724/
NM_001042432.1(CLN3):c.418G>A	concordant	unreportable	unreportable	205088	0	2	SCV000240582;S	Uncertain significance;Unc	GeneDx;Illumina C	2017-11-28;2016-1	CLN3	chr16	28498819	28498819	http://gnomad.b	4.72E-05	AMR	0.0001786780049	0		0	0	https://www.ncbi.nlm.nih.gov/clinvar/variation/205088/
NM_001042432.1(CLN3):c.290C>T	concordant	unreportable	unreportable	434792	0	1	SCV000594152	Uncertain significance	Genetic Services La	7/5/2017	CLN3	chr16	28499916	28499916	http://gnomad.b	3.06E-05	AFR	0.0001306850027	0		0	0	https://www.ncbi.nlm.nih.gov/clinvar/variation/434792/
NM_001042432.1(CLN3):c.222T>C	concordant	unreportable	unreportable	457951	0	1	SCV000628928	Uncertain significance	Invitae	10/5/2017	CLN3	chr16	28500611	28500611	http://gnomad.b	6.39E-05	FIN	0.0002279150067	0		0	0	https://www.ncbi.nlm.nih.gov/clinvar/variation/457951/
NM_001042432.1(CLN3):c.180C>T	concordant	unreportable	unreportable	377684	0	1	SCV000512632	Likely benign	GeneDx	11/28/2017	CLN3	chr16	28500653	28500653	http://gnomad.b	2.78E-05	AFR	6.59E-05	0		0	0	https://www.ncbi.nlm.nih.gov/clinvar/variation/377684/
NM_000049.2(ASPA):c.906G>A	concordant	unreportable	unreportable	322669	0	1	SCV000402022	Uncertain significance	Illumina Clinical Se	10/18/2016	ASPA	chr17	3402346	3402346	http://gnomad.b	1.89E-05	AMR	0.0002084699954	0		0	0	https://www.ncbi.nlm.nih.gov/clinvar/variation/322669/
NM_004937.2(CTNS):c.480C>T	concordant	unreportable	unreportable	496275	0	1	SCV000698524	Uncertain significance	Laboratory Corpor	1/25/2018	CTNS	chr17	3559799	3559799	http://gnomad.b	0.0001392625603	NFE	0.000226203003	0		0	0	https://www.ncbi.nlm.nih.gov/clinvar/variation/496275/
NM_004937.2(CTNS):c.853-15G>A	concordant	unreportable	unreportable	322843	0	1	SCV000402240	Uncertain significance	Illumina Clinical Se	10/18/2016	CTNS	chr17	3563137	3563137	http://gnomad.b	0.000210375357	FIN	0.0008992810035	0		0	0	https://www.ncbi.nlm.nih.gov/clinvar/variation/322843/
NM_000018.3(ACADVL):c.-36A>G	concordant	unreportable	unreportable	324981	0	2	SCV000517063;S	Likely benign;Uncertain sig	GeneDx;Illumina C	2017-11-28;2016-1	ACADVL	chr17	7123268	7123268	http://gnomad.b	0.0006654584584	NFE	0.0051746401	0		0	0	https://www.ncbi.nlm.nih.gov/clinvar/variation/324981/
NM_000018.3(ACADVL):c.369C>T	concordant	unreportable	unreportable	389353	0	1	SCV000531844	Likely benign	GeneDx	11/28/2017	ACADVL	chr17	7124269	7124269	http://gnomad.b	2.55E-05	NFE	8.95E-05	0		0	0	https://www.ncbi.nlm.nih.gov/clinvar/variation/389353/
NM_000018.3(ACADVL):c.477+17G>A	concordant	unreportable	unreportable	386274	0	1	SCV000527848	Likely benign	GeneDx	11/28/2017	ACADVL	chr17	7124394	7124394	http://gnomad.b	1.98E-05	AFR	6.56E-05	0		0	0	https://www.ncbi.nlm.nih.gov/clinvar/variation/386274/
NM_000018.3(ACADVL):c.756T>C	concordant	unreportable	unreportable	254704	0	1	SCV000523205	Likely benign	GeneDx	11/28/2017	ACADVL	chr17	7125499	7125499	http://gnomad.b	3.40E-05	AFR	0.0001306850027	0		0	0	https://www.ncbi.nlm.nih.gov/clinvar/variation/254704/
NM_000018.3(ACADVL):c.1552G>A	concordant	unreportable	unreportable	439356	0	1	SCV000602364	Uncertain significance	ARUP Laboratories	6/30/2017	ACADVL	chr17	7127659	7127659	http://gnomad.b	2.27E-05	SAS	0.0002275389998	0		0	0	https://www.ncbi.nlm.nih.gov/clinvar/variation/439356/
NM_000018.3(ACADVL):c.1754C>T	concordant	unreportable	unreportable	287514	0	2	SCV000341303;S	Uncertain significance;Unc	EGL Genetic Diagn	2017-06-30;2016-1	ACADVL	chr17	7128130	7128130	http://gnomad.b	0.0001245964773	NFE	0.0001432999998	0		0	0	https://www.ncbi.nlm.nih.gov/clinvar/variation/287514/
NM_000018.3(ACADVL):c.1835C>G	concordant	unreportable	unreportable	474893	0	1	SCV000654944	Uncertain significance	Invitae	10/5/2017	ACADVL	chr17	7128283	7128283	http://gnomad.b	8.50E-06	NFE	2.69E-05	0		0	0	https://www.ncbi.nlm.nih.gov/clinvar/variation/474893/
NM_000382.2(ALDH3A2):c.1021C>G	concordant	unreportable	unreportable	322216	0	1	SCV000401259	Uncertain significance	Illumina Clinical Se	10/18/2016	ALDH3A2	chr17	19566726	19566726	http://gnomad.b	8.40E-06	NFE	2.69E-05	0		0	0	https://www.ncbi.nlm.nih.gov/clinvar/variation/322216/
NM_000382.2(ALDH3A2):c.1267C>T	concordant	unreportable	unreportable	500063	0	1	SCV000705845	Uncertain significance	EGL Genetic Diagn	6/30/2017	ALDH3A2	chr17	19575093	19575093	http://gnomad.b	1.68E-05	EAS	5.80E-05	0		0	0	https://www.ncbi.nlm.nih.gov/clinvar/variation/500063/
NM_000151.3(G6PC):c.-45G>A	concordant	unreportable	unreportable	137405	0	1	SCV000402973	Uncertain significance	Illumina Clinical Se	10/18/2016	G6PC	chr17	41052849	41052849	http://gnomad.b	0.0005903791205	NFE	0.002061010106	0		0	0	https://www.ncbi.nlm.nih.gov/clinvar/variation/137405/
NM_000151.3(G6PC):c.258G>A	concordant	reportable	reportable	502134	1	0	SCV000708758	Pathogenic	EGL Genetic Diagn	6/30/2017	G6PC	chr17	41055975	41055975		2.56E-06					0	0	https://www.ncbi.nlm.nih.gov/clinvar/variation/502134/
NM_000151.3(G6PC):c.340+10C>A	concordant	unreportable	unreportable	285532	0	3	SCV000338595;S	Uncertain significance;Likel	EGL Genetic Diagn	2017-06-30;2017-1	G6PC	chr17	41056067	41056067	http://gnomad.b	0.0004804817085	NFE	0.00176470005	1		0	0	https://www.ncbi.nlm.nih.gov/clinvar/variation/285532/
NM_000023.2(SGCA):c.155T>G	concordant	unreportable	unreportable	281286	0	1	SCV000331992	Uncertain significance	EGL Genetic Diagn	6/30/2017	SGCA	chr17	48244846	48244846	http://gnomad.b	0.0001119432224	AFR	6.54E-05	0		0	0	https://www.ncbi.nlm.nih.gov/clinvar/variation/281286/
NM_000023.2(SGCA):c.189C>T	concordant	unreportable	unreportable	497842	0	1	SCV000702568	Uncertain significance	EGL Genetic Diagn	6/30/2017	SGCA	chr17	48244974	48244974	http://gnomad.b	6.44E-05	AFR	0.0008513429784	0		0	0	https://www.ncbi.nlm.nih.gov/clinvar/variation/497842/
NM_000023.2(SGCA):c.312+6G>A	concordant	unreportable	unreportable	500172	0	1	SCV000705996	Uncertain significance	EGL Genetic Diagn	6/30/2017	SGCA	chr17	48245103	48245103	http://gnomad.b	1.96E-05	SAS	0.0001001670025	0		0	0	https://www.ncbi.nlm.nih.gov/clinvar/variation/500172/
NM_000023.2(SGCA):c.981C>T	concordant	unreportable	unreportable	373223	0	1	SCV000491801	Uncertain significance	GeneDx	12/21/2016	SGCA	chr17	48248025	48248025	http://gnomad.b	5.32E-05	AFR	0.0004539260117	0		0	0	https://www.ncbi.nlm.nih.gov/clinvar/variation/373223/
NM_000023.2(SGCA):c.984-10G>A	concordant	unreportable	unreportable	289445	0	1	SCV000343813	Uncertain significance	EGL Genetic Diagn	6/30/2017	SGCA	chr17	48252608	48252608	http://gnomad.b	4.48E-05	AFR	0.001016580034	0		0	0	https://www.ncbi.nlm.nih.gov/clinvar/variation/289445/
NM_000023.2(SGCA):c.1108C>T	concordant	unreportable	unreportable	497816	0	1	SCV000702537	Uncertain significance	EGL Genetic Diagn	6/30/2017	SGCA	chr17	48252742	48252742	http://gnomad.b	9.52E-05	AFR	6.67E-05	0		0	0	https://www.ncbi.nlm.nih.gov/clinvar/variation/497816/
NM_000154.1(GALK1):c.1044C>T	concordant	unreportable	unreportable	325225	0	1	SCV000406616	Uncertain significance	Illumina Clinical Se	10/18/2016	GALK1	chr17	73754354	73754354	http://gnomad.b	8.01E-05	NFE	0.0001009400003	0		0	0	https://www.ncbi.nlm.nih.gov/clinvar/variation/325225/
NM_000154.1(GALK1):c.840C>T	concordant	unreportable	unreportable	325229	0	1	SCV000406620	Uncertain significance	Illumina Clinical Se	10/18/2016	GALK1	chr17	73754634	73754634	http://gnomad.b	0.001538757453	SAS	0.02179309912	9		2	0	https://www.ncbi.nlm.nih.gov/clinvar/variation/325229/
NM_000154.1(GALK1):c.746C>T	concordant	unreportable	unreportable	325232	0	2	SCV000406623;S	Uncertain significance;Unc	Illumina Clinical Se	2016-10-18;2017-1	GALK1	chr17	73758832	73758832	http://gnomad.b	0.0008735654292	ASJ	0.004877060186	0		0	0	https://www.ncbi.nlm.nih.gov/clinvar/variation/325232/
NM_000152.3(GAA):c.70G>A	concordant	unreportable	unreportable	290440	0	1	SCV000344999	Uncertain significance	EGL Genetic Diagn	6/30/2017	GAA	chr17	78078455	78078455	http://gnomad.b	1.11E-05	AMR	8.94E-05	0		0	0	https://www.ncbi.nlm.nih.gov/clinvar/variation/290440/
NM_000152.3(GAA):c.249C>T	concordant	unreportable	unreportable	290271	0	2	SCV000344792;S	Uncertain significance;Likel	EGL Genetic Diagn	2017-06-30;2017-1	GAA	chr17	78078634	78078634	http://gnomad.b	3.89E-05	NFE	8.20E-05	0		0	0	https://www.ncbi.nlm.nih.gov/clinvar/variation/290271/
NM_000152.3(GAA):c.546+6C>T	concordant	unreportable	unreportable	284357	0	1	SCV000336948	Uncertain significance	EGL Genetic Diagn	6/30/2017	GAA	chr17	78078937	78078937	http://gnomad.b	5.56E-06	AFR	0.0003574489965	0		0	0	https://www.ncbi.nlm.nih.gov/clinvar/variation/284357/
NM_000152.3(GAA):c.663C>T	concordant	unreportable	unreportable	456426	0	2	SCV000708091;S	Uncertain significance;Likel	EGL Genetic Diagn	2017-06-30;2017-1	GAA	chr17	78079664	78079664	http://gnomad.b	8.62E-05	FIN	0.000591575983	0		0	0	https://www.ncbi.nlm.nih.gov/clinvar/variation/456426/
NM_000152.3(GAA):c.692+9T>C	concordant	unreportable	unreportable	288402	0	2	SCV000342505;S	Uncertain significance;Likel	EGL Genetic Diagn	2017-06-30;2017-1	GAA	chr17	78079702	78079702	http://gnomad.b	8.34E-06	NFE	1.84E-05	0		0	0	https://www.ncbi.nlm.nih.gov/clinvar/variation/288402/
NM_000152.3(GAA):c.693-4G>T	concordant	unreportable	unreportable	281097	0	3	SCV000331313;S	Uncertain significance;Likel	EGL Genetic Diagn	2017-06-30;2017-1	GAA	chr17	78081352	78081352	http://gnomad.b	0.0001667583838	AMR	0.001310460037	0		0	0	https://www.ncbi.nlm.nih.gov/clinvar/variation/281097/
NM_000152.3(GAA):c.851C>G	concordant	unreportable	unreportable	456440	0	1	SCV000626644	Uncertain significance	Invitae	10/5/2017	GAA	chr17	78081514	78081514	http://gnomad.b	2.78E-05	AFR	0.0002696510055	0		0	0	https://www.ncbi.nlm.nih.gov/clinvar/variation/456440/
NM_000152.3(GAA):c.910G>A	concordant	unreportable	unreportable	285649	0	1	SCV000338777	Uncertain significance	EGL Genetic Diagn	6/30/2017	GAA	chr17	78081650	78081650	http://gnomad.b	2.22E-05	AMR	0.0002392199967	0		1	0	https://www.ncbi.nlm.nih.gov/clinvar/variation/285649/
NM_000152.3(GAA):c.922C>T	concordant	unreportable	unreportable	456444	0	1	SCV000626656	Uncertain significance	Invitae	10/5/2017	GAA	chr17	78081662	78081662	http://gnomad.b	4.17E-05	AFR	0.0004060089996	0		0	0	https://www.ncbi.nlm.nih.gov/clinvar/variation/456444/
NM_000152.3(GAA):c.1019A>G	concordant	unreportable	unreportable	289356	0	2	SCV000343704;S	Uncertain significance;Unc	EGL Genetic Diagn	2017-06-30;2017-1	GAA	chr17	78082152	78082152	http://gnomad.b	6.11E-05	AFR	0.0004581150133	0		0	0	https://www.ncbi.nlm.nih.gov/clinvar/variation/289356/
NM_000152.3(GAA):c.1075+12T>G	concordant	unreportable	unreportable	325785	0	1	SCV000407276	Uncertain significance	Illumina Clinical Se	10/18/2016	GAA	chr17	78082220	78082220	http://gnomad.b	1.67E-05	AFR	6.57E-05	0		0	0	https://www.ncbi.nlm.nih.gov/clinvar/variation/325785/
NM_000152.3(GAA):c.1192C>T	concordant	unreportable	unreportable	286605	0	1	SCV000340101	Uncertain significance	EGL Genetic Diagn	6/30/2017	GAA	chr17	78082404	78082404	http://gnomad.b	2.78E-05	NFE	6.33E-05	0		0	0	https://www.ncbi.nlm.nih.gov/clinvar/variation/286605/
NM_000152.3(GAA):c.1288G>A	concordant	unreportable	unreportable	501777	0	1	SCV000708268	Uncertain significance	EGL Genetic Diagn	6/30/2017	GAA	chr17	78082589	78082589	http://gnomad.b	2.50E-05	AFR	0.0005169869983	0		0	0	https://www.ncbi.nlm.nih.gov/clinvar/variation/501777/

Supplemental Table 2

NM_000152.3(GAA):c.1310G>A	concordant	unreportable	unreportable	456374	0	1	SCV000626504	Uncertain significance	Invitae	10/5/2017	GAA	chr17	78082611	78082611	http://gnomad.b	8.62E-05	AFR	0.0004109810106	0	0	0	https://www.ncbi.nlm.nih.gov/clinvar/variation/456374/
NM_000152.3(GAA):c.1392G>C	concordant	unreportable	unreportable	432217	0	1	SCV000589915	Uncertain significance	GeneDx	11/28/2017	GAA	chr17	78083809	78083809	http://gnomad.b	5.84E-05	NFE	0.0001077639972	0	0	0	https://www.ncbi.nlm.nih.gov/clinvar/variation/432217/
NM_000152.3(GAA):c.1416C>T	concordant	unreportable	unreportable	456376	0	2	SCV000708270;S(	Uncertain significance;Likel	EGL Genetic Diagn	2017-06-30;2017-1	GAA	chr17	78083833	78083833	http://gnomad.b	4.17E-05	AFR	6.69E-05	0	0	0	https://www.ncbi.nlm.nih.gov/clinvar/variation/456376/
NM_000152.3(GAA):c.1478C>T	concordant	reportable	reportable	379593	1	0	SCV000516792	Likely pathogenic	GeneDx	7/13/2017	GAA	chr17	78084566	78084566	http://gnomad.b	3.34E-05	NFE	2.69E-05	0	0	0	https://www.ncbi.nlm.nih.gov/clinvar/variation/379593/
NM_000152.3(GAA):c.1607A>G	concordant	unreportable	unreportable	287414	0	1	SCV000341177	Uncertain significance	EGL Genetic Diagn	6/30/2017	GAA	chr17	78084795	78084795	http://gnomad.b	2.22E-05	NFE	1.79E-05	0	0	0	https://www.ncbi.nlm.nih.gov/clinvar/variation/287414/
NM_000152.3(GAA):c.1803G>A	concordant	unreportable	unreportable	456384	0	1	SCV000626525	Likely benign	Invitae	10/5/2017	GAA	chr17	78086425	78086425	http://gnomad.b	2.50E-05	NFE	4.54E-05	0	0	0	https://www.ncbi.nlm.nih.gov/clinvar/variation/456384/
NM_000152.3(GAA):c.1823G>A	concordant	unreportable	unreportable	290266	0	2	SCV000344787;S(	Uncertain significance;Unc	EGL Genetic Diagn	2017-06-30;2017-1	GAA	chr17	78086445	78086445	http://gnomad.b	2.78E-05	AMR	0.0004782399919	0	0	0	https://www.ncbi.nlm.nih.gov/clinvar/variation/290266/
NM_000152.3(GAA):c.1828G>A	concordant	unreportable	unreportable	282242	0	1	SCV000333598	Uncertain significance	EGL Genetic Diagn	6/30/2017	GAA	chr17	78086450	78086450	http://gnomad.b	7.78E-05	AMR	5.98E-05	0	0	0	https://www.ncbi.nlm.nih.gov/clinvar/variation/282242/
NM_000152.3(GAA):c.1860C>T	concordant	unreportable	unreportable	499822	0	1	SCV000705517	Uncertain significance	EGL Genetic Diagn	6/30/2017	GAA	chr17	78086482	78086482	http://gnomad.b	5.00E-05	AFR	0.0007811390096	0	0	0	https://www.ncbi.nlm.nih.gov/clinvar/variation/499822/
NM_000152.3(GAA):c.1888+10_1888+	concordant	unreportable	unreportable	422879	0	2	SCV000709496;S(	Uncertain significance;Likel	EGL Genetic Diagn	2017-06-30;2017-1	GAA	chr17	78086520	78086520	http://gnomad.b	2.50E-05	AFR	0.0009650959983	0	0	0	https://www.ncbi.nlm.nih.gov/clinvar/variation/422879/
NM_000152.3(GAA):c.2122C>T	concordant	unreportable	unreportable	282642	0	1	SCV000334202	Uncertain significance	EGL Genetic Diagn	6/30/2017	GAA	chr17	78087098	78087098	http://gnomad.b	2.50E-05	AFR	0.0002394640032	0	0	0	https://www.ncbi.nlm.nih.gov/clinvar/variation/282642/
NM_000152.3(GAA):c.2161G>C	concordant	unreportable	unreportable	284866	0	1	SCV000337662	Uncertain significance	EGL Genetic Diagn	6/30/2017	GAA	chr17	78087137	78087137		3.06E-05				0	0	https://www.ncbi.nlm.nih.gov/clinvar/variation/284866/
NM_000152.3(GAA):c.2275G>A	concordant	unreportable	unreportable	281241	0	2	SCV000331868;S(	Uncertain significance;Unc	EGL Genetic Diagn	2017-06-30;2017-1	GAA	chr17	78090852	78090852	http://gnomad.b	0.0001083929495	AFR	0.0009820610285	0	0	0	https://www.ncbi.nlm.nih.gov/clinvar/variation/281241/
NM_000152.3(GAA):c.2332-10C>G	concordant	unreportable	unreportable	286317	0	2	SCV000339693;S(	Uncertain significance;Likel	EGL Genetic Diagn	2017-06-30;2017-1	GAA	chr17	78091389	78091389	http://gnomad.b	5.00E-05	AFR	0.001317520044	0	0	0	https://www.ncbi.nlm.nih.gov/clinvar/variation/286317/
NM_000152.3(GAA):c.2800-11C>G	concordant	unreportable	unreportable	325796	0	2	SCV000522821;S(	Likely benign;Uncertain sig	GeneDx;Illumina C	2017-11-28;2016-1	GAA	chr17	78093060	78093060	http://gnomad.b	3.06E-05	AMR	0.0008933360223	1	0	0	https://www.ncbi.nlm.nih.gov/clinvar/variation/325796/
NM_000271.4(NPC1):c.3556C>T	concordant	unreportable	unreportable	286652	0	1	SCV000340176	Uncertain significance	EGL Genetic Diagn	6/30/2017	NPC1	chr18	21114445	21114445	http://gnomad.b	5.43E-05	SAS	3.25E-05	0	0	0	https://www.ncbi.nlm.nih.gov/clinvar/variation/286652/
NM_000271.4(NPC1):c.3364T>C	concordant	unreportable	unreportable	498583	0	1	SCV000703670	Uncertain significance	EGL Genetic Diagn	6/30/2017	NPC1	chr18	21115546	21115546	http://gnomad.b	5.72E-06	AFR	6.54E-05	0	0	0	https://www.ncbi.nlm.nih.gov/clinvar/variation/498583/
NM_000271.4(NPC1):c.3059G>C	concordant	unreportable	unreportable	452564	0	2	SCV000704655;S(	Uncertain significance;Unc	EGL Genetic Diagn	2017-06-30;2017-1	NPC1	chr18	21116823	21116823	http://gnomad.b	1.43E-05	AFR	0.0003268400033	0	0	0	https://www.ncbi.nlm.nih.gov/clinvar/variation/452564/
NM_000271.4(NPC1):c.1542G>A	concordant	unreportable	unreportable	289739	0	1	SCV000344145	Uncertain significance	EGL Genetic Diagn	6/30/2017	NPC1	chr18	21134733	21134733	http://gnomad.b	1.14E-05	AFR	0.0001961300004	0	0	0	https://www.ncbi.nlm.nih.gov/clinvar/variation/289739/
NM_000271.4(NPC1):c.1010G>A	concordant	unreportable	unreportable	502348	0	1	SCV000709041	Uncertain significance	EGL Genetic Diagn	6/30/2017	NPC1	chr18	21136523	21136523	http://gnomad.b	1.14E-05	AFR	6.56E-05	0	0	0	https://www.ncbi.nlm.nih.gov/clinvar/variation/502348/
NM_000271.4(NPC1):c.834G>A	concordant	unreportable	unreportable	286569	0	1	SCV000340053	Uncertain significance	EGL Genetic Diagn	6/30/2017	NPC1	chr18	21140242	21140242	http://gnomad.b	2.57E-05	SAS	0.000130047003	0	0	0	https://www.ncbi.nlm.nih.gov/clinvar/variation/286569/
NM_000271.4(NPC1):c.481C>T	concordant	unreportable	unreportable	181454	0	1	SCV000345703	Uncertain significance	EGL Genetic Diagn	6/30/2017	NPC1	chr18	21141474	21141474	http://gnomad.b	2.86E-05	NFE	4.48E-05	0	0	0	https://www.ncbi.nlm.nih.gov/clinvar/variation/181454/
NM_000271.4(NPC1):c.233G>A	concordant	unreportable	unreportable	326279	0	1	SCV000407890	Uncertain significance	Illumina Clinical Se	10/18/2016	NPC1	chr18	21152092	21152092	http://gnomad.b	4.86E-05	AFR	0.0005883119884	0	0	0	https://www.ncbi.nlm.nih.gov/clinvar/variation/326279/
NM_000271.4(NPC1):c.181-4A>C	concordant	unreportable	unreportable	440009	0	1	SCV000604564	Likely benign	ARUP Laboratories	6/30/2017	NPC1	chr18	21152148	21152148	http://gnomad.b	2.00E-05	NFE	3.60E-05	0	0	0	https://www.ncbi.nlm.nih.gov/clinvar/variation/440009/
NM_000227.3(LAMA3):c.304G>A	concordant	unreportable	unreportable	326294	0	1	SCV000407909	Uncertain significance	Illumina Clinical Se	10/18/2016	LAMA3	chr18	21461918	21461918	http://gnomad.b	4.76E-05	AFR	0.0002621230087	0	0	0	https://www.ncbi.nlm.nih.gov/clinvar/variation/326294/
NM_000227.3(LAMA3):c.477-4G>A	concordant	unreportable	unreportable	326297	0	1	SCV000407915	Uncertain significance	Illumina Clinical Se	10/18/2016	LAMA3	chr18	21469971	21469971	http://gnomad.b	1.96E-05	NFE	0.0001076080007	0	0	0	https://www.ncbi.nlm.nih.gov/clinvar/variation/326297/
NM_000227.3(LAMA3):c.1186C>T	concordant	unreportable	unreportable	326307	0	1	SCV000407937	Uncertain significance	Illumina Clinical Se	10/18/2016	LAMA3	chr18	21481099	21481099	http://gnomad.b	2.52E-05	AMR	0.0001191539995	0	0	0	https://www.ncbi.nlm.nih.gov/clinvar/variation/326307/
NM_000227.3(LAMA3):c.2638C>T	concordant	unreportable	unreportable	326316	0	1	SCV000407958	Uncertain significance	Illumina Clinical Se	10/18/2016	LAMA3	chr18	21494509	21494509	http://gnomad.b	0.0001959785208	ASJ	0.0002031280019	0	0	0	https://www.ncbi.nlm.nih.gov/clinvar/variation/326316/
NM_000227.3(LAMA3):c.3881+4G>A	concordant	unreportable	unreportable	326339	0	1	SCV000408011	Uncertain significance	Illumina Clinical Se	10/18/2016	LAMA3	chr18	21512259	21512259	http://gnomad.b	0.0002351742249	NFE	0.001235560048	0	0	0	https://www.ncbi.nlm.nih.gov/clinvar/variation/326339/
NM_000227.3(LAMA3):c.4200-14G>A	concordant	unreportable	unreportable	326342	0	1	SCV000408021	Uncertain significance	Illumina Clinical Se	10/18/2016	LAMA3	chr18	21523738	21523738	http://gnomad.b	9.24E-05	SAS	0.0002598919964	1	0	0	https://www.ncbi.nlm.nih.gov/clinvar/variation/326342/
NM_020533.2(MCOLN1):c.177G>A	concordant	unreportable	unreportable	330487	0	1	SCV000415602	Uncertain significance	Illumina Clinical Se	10/18/2016	MCOLN1	chr19	7589992	7589992	http://gnomad.b	8.56E-05	NFE	0.0001611979969	0	0	0	https://www.ncbi.nlm.nih.gov/clinvar/variation/330487/
NM_020533.2(MCOLN1):c.1013G>A	concordant	unreportable	unreportable	459177	0	1	SCV000630738	Uncertain significance	Invitae	10/5/2017	MCOLN1	chr19	7593735	7593735	http://gnomad.b	1.67E-05	SAS	0.0002599259897	1	0	0	https://www.ncbi.nlm.nih.gov/clinvar/variation/459177/
NM_020533.2(MCOLN1):c.1360-16G>.	concordant	unreportable	unreportable	496445	0	1	SCV000699451	Uncertain significance	Laboratory Corpora	1/25/2018	MCOLN1	chr19	7595156	7595156	http://gnomad.b	2.85E-05	AFR	0.0003923620097	0	0	0	https://www.ncbi.nlm.nih.gov/clinvar/variation/496445/
NM_000528.3(MAN2B1):c.2619G>A	concordant	unreportable	unreportable	328257	0	1	SCV000410777	Uncertain significance	Illumina Clinical Se	10/18/2016	MAN2B1	chr19	12759034	12759034	http://gnomad.b	5.92E-05	AMR	0.0001036629983	0	0	0	https://www.ncbi.nlm.nih.gov/clinvar/variation/328257/
NM_000528.3(MAN2B1):c.2267+8dup	concordant	unreportable	unreportable	290029	0	2	SCV000344505;S(	Likely benign;Uncertain sig	EGL Genetic Diagn	2016-11-03;2016-1	MAN2B1	chr19	12760718	12760718	http://gnomad.b	0.002143686126	NFE	0.007535569835	6	3	0	https://www.ncbi.nlm.nih.gov/clinvar/variation/290029/
NM_000528.3(MAN2B1):c.2161G>A	concordant	unreportable	unreportable	439875	0	1	SCV000604141	Uncertain significance	ARUP Laboratories	6/30/2017	MAN2B1	chr19	12760922	12760922	http://gnomad.b	0.0001464805237	NFE	0.0001790289971	0	0	0	https://www.ncbi.nlm.nih.gov/clinvar/variation/439875/
NM_000528.3(MAN2B1):c.2047-5C>T	concordant	unreportable	unreportable	328263	0	1	SCV000410785	Uncertain significance	Illumina Clinical Se	10/18/2016	MAN2B1	chr19	12761041	12761041	http://gnomad.b	1.97E-05	EAS	0.0001739330037	0	0	0	https://www.ncbi.nlm.nih.gov/clinvar/variation/328263/
NM_000528.3(MAN2B1):c.1845G>A	concordant	unreportable	unreportable	328265	0	1	SCV000410788	Uncertain significance	Illumina Clinical Se	10/18/2016	MAN2B1	chr19	12763260	12763260	http://gnomad.b	3.10E-05	NFE	3.58E-05	0	0	0	https://www.ncbi.nlm.nih.gov/clinvar/variation/328265/
NM_000528.3(MAN2B1):c.630+12G>C	concordant	unreportable	unreportable	328279	0	1	SCV000410809	Uncertain significance	Illumina Clinical Se	10/18/2016	MAN2B1	chr19	12775594	12775594	http://gnomad.b	0.0002704255823	SAS	0.003475439968	1	0	0	https://www.ncbi.nlm.nih.gov/clinvar/variation/328279/
NM_004646.3(NPHS1):c.3456G>T	concordant	unreportable	unreportable	282923	0	1	SCV000334668	Uncertain significance	EGL Genetic Diagn	6/30/2017	NPHS1	chr19	36321980	36321980	http://gnomad.b	8.08E-05	AFR	0.0007199899992	0	0	0	https://www.ncbi.nlm.nih.gov/clinvar/variation/282923/
NM_004646.3(NPHS1):c.3311+14T>C	concordant	unreportable	unreportable	328856	0	1	SCV000411553	Uncertain significance	Illumina Clinical Se	10/18/2016	NPHS1	chr19	36322364	36322364	http://gnomad.b	2.23E-05	NFE	8.06E-05	0	0	0	https://www.ncbi.nlm.nih.gov/clinvar/variation/328856/
NM_004646.3(NPHS1):c.2469G>A	concordant	unreportable	unreportable	328863	0	1	SCV000411562	Uncertain significance	Illumina Clinical Se	10/18/2016	NPHS1	chr19	36333318	36333318	http://gnomad.b	2.79E-05	AMR	8.93E-05	0	0	0	https://www.ncbi.nlm.nih.gov/clinvar/variation/328863/
NM_004646.3(NPHS1):c.485C>G	concordant	unreportable	unreportable	328878	0	1	SCV000411585	Uncertain significance	Illumina Clinical Se	10/18/2016	NPHS1	chr19	36341904	36341904	http://gnomad.b	1.95E-05	NFE	2.69E-05	0	0	0	https://www.ncbi.nlm.nih.gov/clinvar/variation/328878/
NM_000709.3(BCKDHA):c.-25G>C	concordant	unreportable	unreportable	329356	0	1	SCV000413336	Uncertain significance	Illumina Clinical Se	10/18/2016	BCKDHA	chr19	41903708	41903708	http://gnomad.b	0.001012514041	ASJ	0.01038059965	2	1	0	https://www.ncbi.nlm.nih.gov/clinvar/variation/329356/
NM_000709.3(BCKDHA):c.63C>T	concordant	unreportable	unreportable	329357	0	1	SCV000413337	Uncertain significance	Illumina Clinical Se	10/18/2016	BCKDHA	chr19	41903795	41903795	http://gnomad.b	0.0001502950529	AFR	0.002119770041	0	0	0	https://www.ncbi.nlm.nih.gov/clinvar/variation/329357/
NM_000709.3(BCKDHA):c.213C>T	concordant	unreportable	unreportable	392392	0	1	SCV000535657	Likely benign	GeneDx	11/28/2017	BCKDHA	chr19	41916646	41916646	http://gnomad.b	0.0001819361167	NFE	0.0002416759962	0	0	0	https://www.ncbi.nlm.nih.gov/clinvar/variation/392392/
NM_000709.3(BCKDHA):c.288+10G>A	concordant	unreportable	unreportable	329360	0	1	SCV000413343	Uncertain significance	Illumina Clinical Se	10/18/2016	BCKDHA	chr19	41916731	41916731	http://gnomad.b	0.0007277444668	SAS	0.01016960014	5	1	0	https://www.ncbi.nlm.nih.gov/clinvar/variation/329360/
NM_000709.3(BCKDHA):c.336T>G	concordant	unreportable	unreportable	389804	0	1	SCV000532458	Likely benign	GeneDx	11/28/2017	BCKDHA	chr19	41916875	41916875	http://gnomad.b	0.0001740258507	NFE	0.0002506579913	0	0	0	https://www.ncbi.nlm.nih.gov/clinvar/variation/389804/
NM_000709.3(BCKDHA):c.376-4C>T	concordant	unreportable	unreportable	93359	0	3	SCV000111111;S(	Benign;Benign;Benign	EGL Genetic Diagn	2017-06-30;2017-1	BCKDHA	chr19	41919950	41919950	http://gnomad.b	0.002816054676	SAS	0.02092269994	10	0	0	https://www.ncbi.nlm.nih.gov/clinvar/variation/93359/
NM_000709.3(BCKDHA):c.435C>T	concordant	unreportable	unreportable	329361	0	1	SCV000413345	Uncertain significance	Illumina Clinical Se	10/18/2016	BCKDHA	chr19	41920013	41920013	http://gnomad.b	2.37E-05	SAS	6.50E-05	0	0	0	https://www.ncbi.nlm.nih.gov/clinvar/variation/329361/
NM_000709.3(BCKDHA):c.436G>A	concordant	unreportable	unreportable	329362	0	1	SCV000413346	Uncertain significance	Illumina Clinical Se	10/18/2016	BCKDHA	chr19	41920014	41920014	http://gnomad.b	7.91E-06	SAS	6.50E-05	0	0	0	https://www.ncbi.nlm.nih.gov/clinvar/variation/329362/
NM_000709.3(BCKDHA):c.448A>G	concordant	unreportable	unreportable	329363	0	1	SCV000413347	Uncertain significance	Illumina Clinical Se	10/18/2016	BCKDHA	chr19	41920026	41920026	http://gnomad.b	0.000134474521	NFE	0.0001886420068	0	0	0	https://www.ncbi.nlm.nih.gov/clinvar/variation/329363/
NM_000709.3(BCKDHA):c.1080C>T	concordant	unreportable	unreportable	198748	0	2	SCV000232575;S(	Uncertain significance;Likel	EGL Genetic Diagn	2017-06-30;2017-1	BCKDHA	chr19	41928987	41928987	http://gnomad.b	7.91E-05	AFR	9.22E-05	0	0	0	https://www.ncbi.nlm.nih.gov/clinvar/variation/198748/
NM_000709.3(BCKDHA):c.1081A>G	concordant	unreportable	unreportable	457140	0	1	SCV000627796	Likely benign	Invitae	10/5/2017	BCKDHA	chr19	41928988	41928988	http://gnomad.b	0.001645335316	ASJ	0.01543349959	1	0	0	https://www.ncbi.nlm.nih.gov/clinvar/variation/457140/
NM_000709.3(BCKDHA):c.1191C>T	concordant	unreportable	unreportable	329367	0	2	SCV000527521;S(	Likely benign;Uncertain sig	GeneDx;Illumina C	2017-11-28;2016-1	BCKDHA	chr19	41930366	41930366	http://gnomad.b	0.000553718616	NFE	0.0007409440004	0	0	0	https://www.ncbi.nlm.nih.gov/clinvar/variation/329367/
NM_000182.4(HADHA):c.1829G>A	concordant	unreportable	unreportable	335376	0	1	SCV000429502	Uncertain significance	Illumina Clinical Se	10/18/2016	HADHA	chr2	26416502	26416502	http://gnomad.b	1.96E-05	AFR	6.53E-05	0	0	0	https://www.ncbi.nlm.nih.gov/clinvar/variation/335376/
NM_000182.4(HADHA):c.1690-13G>A	concordant	unreportable	unreportable	335378	0	1	SCV000429508	Uncertain significance	Illumina Clinical Se	10/18/2016	HADHA	chr2	26416654	26416654	http://gnomad.b	0.0001121478557	NFE	0.0003314049973	0	0	0	https://www.ncbi.nlm.nih.gov/clinvar/variation/335378/
NM_000182.4(HADHA):c.1621-19A>G	concordant	unreportable	unreportable	387142	0	1	SCV000529043	Likely benign	GeneDx	11/28/2017	HADHA	chr2	26417526	26417526	http://gnomad.b	1.40E-05	AFR	0.0005886200233	0	0	0	https://www.ncbi.nlm.nih.gov/clinvar/variation/387142/
NM_000182.4(HADHA):c.918+19T>C	concordant	unreportable	unreportable	383913	0	1	SCV000524528	Likely benign	GeneDx	11/28/2017	HADHA	chr2	26437293	26437293	http://gnomad.b	2.24E-05	EAS	5.80E-05	0	0	0	https://www.ncbi.nlm.nih.gov/clinvar/variation/383913/
NM_000182.4(HADHA):c.560G>C	concordant	unreportable	unreportable	335386	0	1	SCV000429529	Uncertain significance	Illumina Clinical Se	10/18/2016	HADHA	chr2	26455041	26455041	http://gnomad.b	9.25E-05	ASJ	0.001116979984	0	0	0	https://www.ncbi.nlm.nih.gov/clinvar/variation/335386/
NM_000182.4(HADHA):c.-20G>T	concordant	unreportable	unreportable	384221	0	1	SCV000524943	Likely benign	GeneDx	11/28/2017	HADHA	chr2	26467484	26467484		8.41E-06				0	0	https://www.ncbi.nlm.nih.gov/clinvar/variation/384221/
NM_004543.4(NEB):c.18673C>T	concordant	unreportable	unreportable	194312	0	3	SCV000225982;S(	Likely benign;Uncertain sig	EGL Genetic Diagn	2017-06-30;2017-1	NEB	chr2	152359886	152359886	http://gnomad.b	6.97E-05	NFE	0.0004033269943	0	0	0	https://www.ncbi.nlm.nih.gov/clinvar/variation/194312/
NM_004543.4(NEB):c.15803A>T	concordant	unreportable	unreportable	331428	0	1	SCV000416856	Uncertain significance	Illumina Clinical Se	10/18/2016	NEB	chr2	152394745	152394745	http://gnomad.b	5.16E-06	NFE	8.98E-06	0	0	0	https://www.ncbi.nlm.nih.gov/clinvar/variation/331428/
NM_001271208.1(NEB):c.19944+14A>	concordant	unreportable	unreportable	383697	0	1	SCV000524163	Likely benign	GeneDx	11/28/2017	NEB	chr2	152408238	152408238	http://gnomad.b	9.04E-05	NFE	0.0001980980014	0	0	0	https://www.ncbi.nlm.nih.gov/clinvar/variation/383697/
NM_004543.4(NEB):c.3468C>T	concordant	unreportable	unreportable	435965	0	1	SCV000595954	Uncertain significance	Genetic Services La	7/5/2017	NEB	chr2	152534489	152534489	http://gnomad.b	0.0001858755254	AFR	0.001636130037	0	1	0	https://www.ncbi.nlm.nih.gov/clinvar/variation/435965/
NM_004543.4(NEB):c.403G>A	concordant	unreportable	unreportable	285101	0	2	SCV000337974;S(	Uncertain significance;Unc	EGL Genetic Diagn	2017-06-30;2017-1	NEB	chr2	152581475	152581475	http://gnomad.b	2.84E-05	AFR	0.0001308729989	0	0	0	https://www.ncbi.nlm.nih.gov/clinvar/variation/285101/
NM_001875.4(CPS1):c.5C>T	concordant	unreportable	unreportable	334009	0	2	SCV000619805;S(	Uncertain significance;Unc	GeneDx;Illumina C	2017-11-28;2016-1	CPS1	chr2	211421462	211421462	http://gnomad.b	0.0003041119132	ASJ	0.0009164970252	0	0	0	https://www.ncbi.nlm.nih.gov/clinvar/variation/334009/
NM_001875.4(CPS1):c.295C>A	concordant	unreportable	unreportable	334012	0	1	SCV000426813	Uncertain significance	Illumina Clinical Se	10/18/2016	CPS1	chr2	211441128	211441128	http://gnomad.b	8.00E-05	NFE	8.07E-05	0	0	0	https://www.ncbi.nlm.nih.gov/clinvar/variation/334012/
NM_001875.4(CPS1):c.381+17C>T	concordant	unreportable	unreportable	383903	0	1	SCV000524511	Likely benign	GeneDx	11/28/2017	CPS1	chr2	211441231	211441231	http://gnomad.b	0.0001760647918	AFR	0.003006929997	1	0	0	https://www.ncbi.nlm.nih.gov/clinvar/variation/383903/
NM_001875.4(CPS1):c.528+9A>G	concordant	unreportable	unreportable	334014	0	1	SCV000426816	Uncertain significance	Illumina Clinical Se	10/18/2016	CPS1	chr2	211444503	211444503	http://gnomad.b	4.80E-05	NFE	8.96E-05	0	0	0	https://www.ncbi.nlm.nih.gov/clinvar/variation/334014/
NM_001875.4(CPS1):c.637G>A	concordant	unreportable	unreportable	334015	0	1	SCV000426817	Uncertain significance	Illumina Clinical Se	10/18/2016	CPS1	chr2	211452797	211452797	http://gnomad.b	0.0006162267715	FIN	0.004798640031	0	0	0	https://www.ncbi.nlm.nih.gov/clinvar/variation/334015/
NM_001875.4(CPS1):c.1087-7C>T	concordant	unreportable	unreportable	334021	0	1	SCV000426827	Uncertain significance	Illumina Clinical Se	10/18/2016	CPS1	chr2	211457596	211457596	http://gnomad.b	5.60E-05	NFE	0.0001439649932	0	0	0	https://www.ncbi.nlm.nih.gov/clinvar/variation/334021/
NM_001875.4(CPS1):c.1116C>T	concordant	unreportable	unreportable	384375	0	1	SCV000525151	Likely benign	GeneDx	11/28/2017	CPS1	chr2	211457632	211457632	http://gnomad.b	0.0008483121789	ASJ	0.008133389987	0	1	0	https://www.ncbi.nlm.nih.gov/clinvar/variation/384375/
NM_001875.4(CPS1):c.1131C>T	concordant	unreportable	unreportable	389021	0	1	SCV000531437	Likely benign	GeneDx	11/28/2017	CPS1	chr2	211457647	211457647	http://gnomad.b	7.20E-05	NFE	8.08E-05	0	0	0	https://www.ncbi.nlm.nih.gov/clinvar/variation/389021/
NM_001875.4(CPS1):c.2391+20C>T	concordant	unreportable	unreportable	385016	0	1	SCV000526133	Likely benign	GeneDx	11/28/2017	CPS1	chr2	211473303	211473303		0.0001520559566				0	0	https://www.ncbi.nlm.nih.gov/clinvar/variation/385016/
NM_001875.4(CPS1):c.2544C>T	concordant	unreportable	unreportable	387992	0	1	SCV000530194	Likely benign	GeneDx	11/28/2017	CPS1	chr2	211476993	211476993	http://gnomad.b	4.00E-05	NFE	0.0002428059961	0	0	0	https://www.ncbi.nlm.nih.gov/clinvar/variation/387992/
NM_001875.4(CPS1):c.3057T>C	concordant	unreportable	unreportable	385662	0	1	SCV000527000	Likely benign	GeneDx	11/28/2017	CPS1	chr2	211507305	211507305	http://gnomad.b	7.20E-05	NFE	0.0002059599938	0	0	0	https://www.ncbi.nlm.nih.gov/clinvar/variation/385662/
NM_001875.4(CPS1):c.3141+15delA	concordant	unreportable	unreportable	334030	0	3	SCV000569051;S(	Likely benign;Uncertain sig	GeneDx;Illumina C	2017-11-28;2016-1	CPS1	chr2	211507399	211507400		0.007626806665				4	0	https://www.ncbi.nlm.nih.gov/clinvar/variation/334030/
NM_001875.4(CPS1):c.3404+9_3404+	concordant	unreportable	unreportable	421252	0	1	SCV000570394	Likely benign	GeneDx	11/28/2017	CPS1	chr2	211513271	211513273	http://gnomad.b	0.000664244442	SAS	0.007212480064	3	1	0	https://www.ncbi.nlm.nih.gov/clinvar/variation/421252/
NM_001875.4(CPS1):c.4252C>T	concordant	unreportable	unreportable	196974	0	2	SCV000229821;S(	Uncertain significance;Unc	EGL Genetic Diagn	2017-06-30;2016-1	CPS1	chr2	211540542	211540542	http://gnomad.b	0.0007442738928	ASJ	0.001930500031	0	0	0	https://www.ncbi.nlm.nih.gov/clinvar/variation/196974/
NM_004328.4(BCS1L):c.-14G>A	concordant	unreportable	unreportable	334355	0	1	SCV000427422	Uncertain significance	Illumina Clinical Se	10/18/2016	BCS1L	chr2	219525697	219525697	http://gnomad.b	0.0001115436526	AFR	0.003082609968	0	1	0	https://www.ncbi.nlm.nih.gov/clinvar/variation/334355/
NM_004328.4(BCS1L):c.771G>A	concordant	unreportable	unreportable	384871	0	1	SCV000525934	Likely benign	GeneDx	11/28/2017	BCS1L	chr2	219527284	219527284	http://gnomad.b	0.0001199094266	ASJ	0.0002031689946	0	0	0	https://www.ncbi.nlm.nih.gov/clinvar/variation/384871/
NM_000030.2(AGXT):c.-14G>A	concordant	unreportable	unreportable	335293	0	1	SCV000429354	Uncertain significance	Illumina Clinical Se	10/18/2016	AGXT	chr2	241808269	241808269	http://gnomad.b	1.19E-05	NFE	0.0001096530032	0	0	0	https://www.ncbi.nlm.nih.gov/clinvar/variation/335293/
NM_000030.2(AGXT):c.484G>A	concordant	unreportable	unreportable	335295	0	1	SCV000429361	Uncertain significance	Illumina Clinical Se	10/18/2016	AGXT	chr2	241810826	241810826	http://gnomad.b	5.07E-05	AFR	0.0001534210023	0	0	0	https://www.ncbi.nlm.nih.gov/clinvar/variation/335295/
NM_000030.2(AGXT):c.1071+12C>T	concordant	unreportable	unreportable	335301	0	1	SCV000429375	Uncertain significance	Illumina Clinical Se	10/18/2016	AGXT	chr2	241817579	241817579	http://gnomad.b	0.0002059308072	EAS	0.0003485129855	0	0	0	https://www.ncbi.nlm.nih.gov/clinvar/variation/335301/
NM_000022.2(ADA):c.606+10G>A	concordant	unreportable	unreportable	434080	0	1	SCV000593031	Uncertain significance	Genetic Services La	7/5/2017	ADA	chr20	43252833	43252833	http://gnomad.b	0.0008642489037	ASJ	0.004101810046	0	0	0	https://www.ncbi.nlm.nih.gov/clinvar/variation/434080/
NM_000022.2(ADA):c.591T>A	concordant	unreportable	unreportable	338509	0	1	SCV000434057	Uncertain significance	Illumina Clinical Se	10/18/2016	ADA	chr20	43252858	43252858	http://gnomad.b	0.0004801382798	ASJ	0.005322420038	0	0	0	https://www.ncbi.nlm.nih.gov/clinvar/variation/338509/
NM_000022.2(ADA):c.508A>G	concordant	unreportable	unreportable	373095	0	1	SCV000491658	Uncertain significance	GeneDx	12/21/2016	ADA	chr20	43252941	43252941	http://gnomad.b	6.40E-05	SAS	0.0001299550058	0	0	0	https://www.ncbi.nlm.nih.gov/clinvar/variation/373095/
NM_000411.6(HLCS):c.1975G>A	concordant	unreportable	unreportable	459944	0	1	SCV000631924	Uncertain significance	Invitae	10/5/2017	HLCS	chr21	38128877	38128877	http://gnomad.b	0.0001125691496	NFE	0.0001969279983	0	0	0	https://www.ncbi.nlm.nih.gov/clinvar/variation/459944/
NM_000411.6(HLCS):c.1911C>T	concordant	unreportable	unreportable	339959	0	1	SCV000436043	Uncertain significance	Illumina Clinical Se	10/18/2016	HLCS	chr21	38128941	38128941	http://gnomad.b	1.61E-05	SAS	0.0001299459982	0	0	0	https://www.ncbi.nlm.nih.gov/clinvar/variation/339959/
NM_000411.6(HLCS):c.1180-12T>C	concordant	unreportable	unreportable	339964	0	1	SCV000436048	Uncertain significance	Illumina Clinical Se	10/18/2016	HLCS	chr21	38269443	38269443	http://gnomad.b	0.0002331789528	FIN	0.001437040046	0	0	0	https://www.ncbi.nlm.nih.gov/clinvar/variation/339964/
NM_000411.6(HLCS):c.949G>T	concordant	unreportable	unreportable	418250	0	1	SCV000565063	Uncertain significance	GeneDx	11/28/2017	HLCS	chr21	38308796	38308796	http://gnomad.b	0.0001286504567	AFR	0.001894930028	0	0	0	https://www.ncbi.nlm.nih.gov/clinvar/variation/418250/
NM_000411.6(HLCS):c.890G>A	concordant	unreportable	unreportable	203765	0	1	SCV000436050	Uncertain significance	Illumina Clinical Se	10/18/2016	HLCS	chr21	38308855	38308855	http://gnomad.b	0.0009568377718	ASJ	0.004670999944	0	0	0	https://www.ncbi.nlm.nih.gov/clinvar/variation/203765/
NM_000411.6(HLCS):c.889C>T	concordant	unreportable	unreportable	203764	0	1	SCV000238912	Uncertain significance	GeneDx	11/28/2017	HLCS	chr21	38308856	38308856	http://gnomad.b	3.22E-05	AFR	0.0003920540039	0	0	0	https://www.ncbi.nlm.nih.gov/clinvar/variation/203764/
NM_000411.6(HLCS):c.632C>T	concordant	unreportable	unreportable	203762	0	1	SCV000436052	Uncertain significance	Illumina Clinical Se	10/18/2016	HLCS	chr21	38309113	38309113	http://gnomad.b	0.0002010163386	FIN	0.002107620006	0	0	0	https://www.ncbi.nlm.nih.gov/clinvar/variation/203762/
NM_000411.6(HLCS):c.400G>A	concordant	unreportable	unreportable	339967	0	1	SCV000436055	Uncertain significance	Illumina Clinical Se	10/18/2016	HLCS	chr21	38309345	38309345	http://gnomad.b	2.41E-05	NFE	2.69E-05	0	0	0	https://www.ncbi.nlm.nih.gov/clinvar/variation/339967/
NM_000411.6(HLCS):c.53-9A>G	concordant	unreportable	unreportable	339971	0	1	SCV000436061	Uncertain significance	Illumina Clinical Se	10/18/2016	HLCS	chr21	38309701	38309701	http://gnomad.b	4.02E-05	NFE	0.0001053439992	0	0	0	https://www.ncbi.nlm.nih.gov/clinvar/variation/339971/

Supplemental Table 2

NM_000411.6(HLCS):c.53-17_53-16de	concordant	unreportable	unreportable	339972	0	2	SCV000565061;S(	Likely benign;Uncertain sig	GeneDx;Illumina C	2017-11-28;2016-1	HLCS	chr21	38309707	38309709	http://gnomad.b	0.0007879840473	NFE	0.001270059962	2		1	0	https://www.ncbi.nlm.nih.gov/clinvar/variation/339972/
NM_000071.2(CBS):c.*28G>A	concordant	unreportable	unreportable	392677	0	1	SCV000535984	Uncertain significance	GeneDx	11/28/2017	CBS	chr21	44473962	44473962	http://gnomad.b	8.44E-06	AMR	9.41E-05	0		0	0	https://www.ncbi.nlm.nih.gov/clinvar/variation/392677/
NM_000071.2(CBS):c.1485G>A	concordant	unreportable	unreportable	471360	0	1	SCV000649829	Likely benign	Invitae	10/5/2017	CBS	chr21	44476980	44476980		2.25E-05					0	0	https://www.ncbi.nlm.nih.gov/clinvar/variation/471360/
NM_000071.2(CBS):c.1161C>T	concordant	unreportable	unreportable	263900	0	3	SCV000516872;S(	Likely benign;Uncertain sig	GeneDx;Illumina C	2017-11-28;2016-1	CBS	chr21	44479398	44479398	http://gnomad.b	8.15E-05	NFE	0.0001075269975	0		0	0	https://www.ncbi.nlm.nih.gov/clinvar/variation/263900/
NM_000071.2(CBS):c.1146-7C>T	concordant	unreportable	unreportable	471354	0	1	SCV000649820	Likely benign	Invitae	10/5/2017	CBS	chr21	44479420	44479420	http://gnomad.b	1.12E-05	AMR	5.96E-05	0		0	0	https://www.ncbi.nlm.nih.gov/clinvar/variation/471354/
NM_000071.2(CBS):c.1009A>G	concordant	unreportable	unreportable	212859	0	1	SCV000249702	Uncertain significance	GeneDx	11/28/2017	CBS	chr21	44482451	44482451	http://gnomad.b	2.25E-05	NFE	8.98E-06	0		0	0	https://www.ncbi.nlm.nih.gov/clinvar/variation/212859/
NM_000071.2(CBS):c.829-13G>A	concordant	unreportable	unreportable	136670	0	1	SCV000436219	Uncertain significance	Illumina Clinical Se	10/18/2016	CBS	chr21	44483201	44483201	http://gnomad.b	0.0001827803992	NFE	0.0009233690216	0		0	0	https://www.ncbi.nlm.nih.gov/clinvar/variation/136670/
NM_000071.2(CBS):c.675C>T	concordant	unreportable	unreportable	212821	0	1	SCV000649846	Likely benign	Invitae	10/5/2017	CBS	chr21	44485374	44485374	http://gnomad.b	8.15E-05	AFR	0.0001960780064	0		0	0	https://www.ncbi.nlm.nih.gov/clinvar/variation/212821/
NM_000071.2(CBS):c.667-10_667-7de	concordant	unreportable	unreportable	405377	0	2	SCV000572399;S(	Likely benign;Uncertain sig	GeneDx;Invitae	2017-04-07;2017-1	CBS	chr21	44485388	44485392	http://gnomad.b	0.0001884044115	AFR	0.001961299917	1		0	0	https://www.ncbi.nlm.nih.gov/clinvar/variation/405377/
NM_000071.2(CBS):c.616G>A	concordant	unreportable	unreportable	212849	0	1	SCV000249687	Uncertain significance	GeneDx	11/28/2017	CBS	chr21	44485547	44485547	http://gnomad.b	2.25E-05	NFE	3.62E-05	0		0	0	https://www.ncbi.nlm.nih.gov/clinvar/variation/212849/
NM_000071.2(CBS):c.532G>A	concordant	unreportable	unreportable	340088	0	1	SCV000436225	Uncertain significance	Illumina Clinical Se	10/18/2016	CBS	chr21	44485631	44485631	http://gnomad.b	5.62E-06	NFE	4.55E-05	0		0	0	https://www.ncbi.nlm.nih.gov/clinvar/variation/340088/
NM_000071.2(CBS):c.404C>T	concordant	unreportable	unreportable	471364	0	1	SCV000649836	Uncertain significance	Invitae	10/5/2017	CBS	chr21	44486400	44486400	http://gnomad.b	3.37E-05	NFE	6.27E-05	0		0	0	https://www.ncbi.nlm.nih.gov/clinvar/variation/471364/
NM_000071.2(CBS):c.152G>A	concordant	unreportable	unreportable	420469	0	1	SCV000569322	Uncertain significance	GeneDx	11/28/2017	CBS	chr21	44492152	44492152	http://gnomad.b	1.12E-05	AFR	0.0001307700004	0		0	0	https://www.ncbi.nlm.nih.gov/clinvar/variation/420469/
NM_000383.3(AIRE):c.226G>A	concordant	unreportable	unreportable	458617	0	1	SCV000629952	Uncertain significance	Invitae	10/5/2017	AIRE	chr21	45706533	45706533	http://gnomad.b	5.57E-05	AFR	0.0008510079933	0		0	0	https://www.ncbi.nlm.nih.gov/clinvar/variation/458617/
NM_000383.3(AIRE):c.538+6C>T	concordant	unreportable	unreportable	384652	0	1	SCV000525576	Likely benign	GeneDx	11/28/2017	AIRE	chr21	45707480	45707480	http://gnomad.b	3.34E-05	AFR	0.0003828479967	0		0	0	https://www.ncbi.nlm.nih.gov/clinvar/variation/384652/
NM_000383.3(AIRE):c.655G>A	concordant	unreportable	unreportable	285376	0	1	SCV000338370	Uncertain significance	EGL Genetic Diagn	6/30/2017	AIRE	chr21	45709542	45709542	http://gnomad.b	1.39E-05	SAS	0.0001299709984	1		0	0	https://www.ncbi.nlm.nih.gov/clinvar/variation/285376/
NM_000383.3(AIRE):c.1401-11C>T	concordant	unreportable	unreportable	446816	0	1	SCV000612307	Benign	Athena Diagnostics	8/17/2017	AIRE	chr21	45714273	45714273	http://gnomad.b	0.0003091456995	FIN	0.03306220099	4		0	0	https://www.ncbi.nlm.nih.gov/clinvar/variation/446816/
NM_000487.5(ARSA):c.1258A>C	concordant	unreportable	unreportable	342234	0	1	SCV000439429	Uncertain significance	Illumina Clinical Se	10/18/2016	ARSA	chr22	51063845	51063845	http://gnomad.b	5.06E-05	AFR	0.0003347620077	0		0	0	https://www.ncbi.nlm.nih.gov/clinvar/variation/342234/
NM_000487.5(ARSA):c.1211-15T>C	concordant	unreportable	unreportable	342235	0	1	SCV000439430	Uncertain significance	Illumina Clinical Se	10/18/2016	ARSA	chr22	51063907	51063907	http://gnomad.b	0.0001603907908	EAS	0.0005811249721	0		0	0	https://www.ncbi.nlm.nih.gov/clinvar/variation/342235/
NM_000487.5(ARSA):c.640G>A	concordant	unreportable	unreportable	196275	0	1	SCV000228888	Uncertain significance	EGL Genetic Diagn	6/30/2017	ARSA	chr22	51065306	51065306	http://gnomad.b	1.69E-05	SAS	3.25E-05	0		0	0	https://www.ncbi.nlm.nih.gov/clinvar/variation/196275/
NM_000060.2(BTD):c.460-8dupT	concordant	unreportable	unreportable	25010	0	1	SCV000230004	Uncertain significance	EGL Genetic Diagn	11/3/2016	BTD	chr3	15685808	15685808	http://gnomad.b	0.0002018233296	AFR	0.005873300135	0		0	0	https://www.ncbi.nlm.nih.gov/clinvar/variation/25010/
NM_000060.2(BTD):c.1172C>T	concordant	unreportable	unreportable	343910	0	1	SCV000441830	Uncertain significance	Illumina Clinical Se	10/18/2016	BTD	chr3	15686535	15686535	http://gnomad.b	2.88E-06	NFE	8.96E-06	0		0	0	https://www.ncbi.nlm.nih.gov/clinvar/variation/343910/
NM_000203.3(IDUA):c.248G>A	concordant	unreportable	unreportable	350190	0	1	SCV000451735	Uncertain significance	Illumina Clinical Se	10/18/2016	IDUA	chr4	981686	981686		1.39E-05					0	0	https://www.ncbi.nlm.nih.gov/clinvar/variation/350190/
NM_000203.3(IDUA):c.630C>T	concordant	unreportable	unreportable	350222	0	1	SCV000451774	Uncertain significance	Illumina Clinical Se	10/18/2016	IDUA	chr4	995507	995507	http://gnomad.b	0.0004691525273	ASJ	0.002321159933	0		0	0	https://www.ncbi.nlm.nih.gov/clinvar/variation/350222/
NM_000203.3(IDUA):c.663C>G	concordant	unreportable	unreportable	350223	0	1	SCV000451775	Uncertain significance	Illumina Clinical Se	10/18/2016	IDUA	chr4	995540	995540	http://gnomad.b	0.0002193079861	AMR	0.0002781730145	0		0	0	https://www.ncbi.nlm.nih.gov/clinvar/variation/350223/
NM_000203.3(IDUA):c.793-9C>T	concordant	unreportable	unreportable	350225	0	2	SCV000451777;S(	Uncertain significance;Likel	Illumina Clinical Se	2016-10-18;2017-1	IDUA	chr4	995761	995761	http://gnomad.b	0.0002442924403	NFE	0.0007922489895	0		0	0	https://www.ncbi.nlm.nih.gov/clinvar/variation/350225/
NM_000203.3(IDUA):c.1002G>A	concordant	unreportable	unreportable	350227	0	1	SCV000451781	Uncertain significance	Illumina Clinical Se	10/18/2016	IDUA	chr4	996086	996086	http://gnomad.b	0.0002359642889	NFE	0.0004105840053	0		0	0	https://www.ncbi.nlm.nih.gov/clinvar/variation/350227/
NM_000203.3(IDUA):c.1118A>C	concordant	unreportable	unreportable	373483	0	1	SCV000492075	Uncertain significance	GeneDx	12/21/2016	IDUA	chr4	996202	996202	http://gnomad.b	0.0001388025229	AFR	0.002936699893	0		1	0	https://www.ncbi.nlm.nih.gov/clinvar/variation/373483/
NM_000203.3(IDUA):c.1479C>A	concordant	unreportable	unreportable	350231	0	1	SCV000451793	Uncertain significance	Illumina Clinical Se	10/18/2016	IDUA	chr4	996900	996900	http://gnomad.b	0.0003470063072	ASJ	0.00299634994	1		1	0	https://www.ncbi.nlm.nih.gov/clinvar/variation/350231/
NM_000203.3(IDUA):c.1728-9C>T	concordant	unreportable	unreportable	350232	0	1	SCV000451794	Uncertain significance	Illumina Clinical Se	10/18/2016	IDUA	chr4	997791	997791	http://gnomad.b	3.33E-05	NFE	7.18E-05	0		0	0	https://www.ncbi.nlm.nih.gov/clinvar/variation/350232/
NM_000232.4(SGCB):c.789C>T	concordant	unreportable	unreportable	497563	0	1	SCV000702155	Uncertain significance	EGL Genetic Diagn	6/30/2017	SGCB	chr4	52890291	52890291	http://gnomad.b	5.04E-05	NFE	9.85E-05	0		0	0	https://www.ncbi.nlm.nih.gov/clinvar/variation/497563/
NM_000232.4(SGCB):c.752C>T	concordant	unreportable	unreportable	498982	0	1	SCV000704263	Uncertain significance	EGL Genetic Diagn	6/30/2017	SGCB	chr4	52894135	52894135	http://gnomad.b	1.96E-05	EAS	0.0001739330037	0		0	0	https://www.ncbi.nlm.nih.gov/clinvar/variation/498982/
NM_000232.4(SGCB):c.251T>C	concordant	unreportable	unreportable	498262	0	1	SCV000703193	Uncertain significance	EGL Genetic Diagn	6/30/2017	SGCB	chr4	52896022	52896022	http://gnomad.b	1.68E-05	NFE	4.48E-05	0		0	0	https://www.ncbi.nlm.nih.gov/clinvar/variation/498262/
NM_000232.4(SGCB):c.244A>G	concordant	unreportable	unreportable	450609	0	1	SCV000619211	Uncertain significance	GeneDx	11/28/2017	SGCB	chr4	52896029	52896029	http://gnomad.b	1.68E-05	AFR	6.54E-05	0		0	0	https://www.ncbi.nlm.nih.gov/clinvar/variation/450609/
NM_000232.4(SGCB):c.188A>G	concordant	unreportable	unreportable	285180	0	1	SCV000338096	Uncertain significance	EGL Genetic Diagn	6/30/2017	SGCB	chr4	52899652	52899652	http://gnomad.b	6.16E-05	AFR	0.0001960270019	0		0	0	https://www.ncbi.nlm.nih.gov/clinvar/variation/285180/
NM_172250.2(MMAA):c.630A>G	concordant	unreportable	unreportable	347601	0	2	SCV000532816;S(	Likely benign;Uncertain sig	GeneDx;Illumina C	2017-11-28;2016-1	MMAA	chr4	146567205	146567205	http://gnomad.b	0.0005999136124	ASJ	0.004771570209	0		0	0	https://www.ncbi.nlm.nih.gov/clinvar/variation/347601/
NM_172250.2(MMAA):c.800C>T	concordant	unreportable	unreportable	466218	0	1	SCV000641769	Uncertain significance	Invitae	10/5/2017	MMAA	chr4	146572280	146572280	http://gnomad.b	0.0001999712041	AMR	0.0002977790136	0		0	0	https://www.ncbi.nlm.nih.gov/clinvar/variation/466218/
NM_172250.2(MMAA):c.1078C>T	concordant	unreportable	unreportable	347606	0	1	SCV000447542	Uncertain significance	Illumina Clinical Se	10/18/2016	MMAA	chr4	146576407	146576407	http://gnomad.b	4.80E-05	AMR	0.0003573340073	0		0	0	https://www.ncbi.nlm.nih.gov/clinvar/variation/347606/
NM_172250.2(MMAA):c.1177G>A	concordant	unreportable	unreportable	347608	0	1	SCV000447545	Uncertain significance	Illumina Clinical Se	10/18/2016	MMAA	chr4	146576506	146576506	http://gnomad.b	0.0002799596858	NFE	0.0004298150016	0		0	0	https://www.ncbi.nlm.nih.gov/clinvar/variation/347608/
NM_000414.3(HSD17B4):c.56C>G	concordant	unreportable	unreportable	227431	0	1	SCV000270272	Likely benign	Laboratory for Mol	4/26/2016	HSD17B4	chr5	118788326	118788326	http://gnomad.b	5.63E-06	AFR	0.0001963090035	0		0	0	https://www.ncbi.nlm.nih.gov/clinvar/variation/227431/
NM_000414.3(HSD17B4):c.936_937de	concordant	reportable	reportable	504023	1	0	SCV000710327	Likely pathogenic	GeneDx	3/26/2018	HSD17B4	chr5	118832303	118832305	http://gnomad.b	2.54E-05	AFR	0.0003921060124	0		0	0	https://www.ncbi.nlm.nih.gov/clinvar/variation/504023/
NM_000414.3(HSD17B4):c.943C>T	concordant	unreportable	unreportable	350464	0	1	SCV000452119	Uncertain significance	Illumina Clinical Se	10/18/2016	HSD17B4	chr5	118832312	118832312	http://gnomad.b	1.13E-05	AMR	8.93E-05	0		0	0	https://www.ncbi.nlm.nih.gov/clinvar/variation/350464/
NM_000414.3(HSD17B4):c.1210-8T>C	concordant	unreportable	unreportable	227432	0	1	SCV000270273	Likely benign	Laboratory for Mol	4/26/2016	HSD17B4	chr5	118837728	118837728	http://gnomad.b	0.0003718959368	NFE	0.002289050026	0		0	0	https://www.ncbi.nlm.nih.gov/clinvar/variation/227432/
NM_000414.3(HSD17B4):c.1357C>T	concordant	unreportable	unreportable	350466	0	1	SCV000452126	Uncertain significance	Illumina Clinical Se	10/18/2016	HSD17B4	chr5	118844859	118844859	http://gnomad.b	3.10E-05	NFE	8.96E-05	0		0	0	https://www.ncbi.nlm.nih.gov/clinvar/variation/350466/
NM_000414.3(HSD17B4):c.1452A>G	concordant	unreportable	unreportable	228742	0	1	SCV000271841	Uncertain significance	Laboratory for Mol	4/26/2016	HSD17B4	chr5	118850690	118850690	http://gnomad.b	1.69E-05	AFR	0.0001307530038	0		0	0	https://www.ncbi.nlm.nih.gov/clinvar/variation/228742/
NM_003060.3(SLC22A5):c.93C>T	concordant	unreportable	unreportable	460420	0	1	SCV000632579	Likely benign	Invitae	10/5/2017	SLC22A5	chr5	131705757	131705757	http://gnomad.b	1.98E-05	AFR	0.0002658870071	0		0	0	https://www.ncbi.nlm.nih.gov/clinvar/variation/460420/
NM_003060.3(SLC22A5):c.952-14T>C	concordant	unreportable	unreportable	350814	0	1	SCV000452734	Uncertain significance	Illumina Clinical Se	10/18/2016	SLC22A5	chr5	131724599	131724599	http://gnomad.b	3.11E-05	NFE	9.85E-05	0		0	0	https://www.ncbi.nlm.nih.gov/clinvar/variation/350814/
NM_003060.3(SLC22A5):c.1043T>A	concordant	unreportable	unreportable	426359	0	1	SCV000576777	Uncertain significance	GeneDx	11/28/2017	SLC22A5	chr5	131724704	131724704	http://gnomad.b	2.82E-06	NFE	8.96E-06	0		0	0	https://www.ncbi.nlm.nih.gov/clinvar/variation/426359/
NM_000112.3(SLC26A2):c.1744C>T	concordant	unreportable	unreportable	352028	0	1	SCV000454827	Uncertain significance	Illumina Clinical Se	10/18/2016	SLC26A2	chr5	149360900	149360900	http://gnomad.b	8.64E-05	EAS	0.000289921998	0		0	0	https://www.ncbi.nlm.nih.gov/clinvar/variation/352028/
NM_000112.3(SLC26A2):c.2129A>G	concordant	unreportable	unreportable	352031	0	1	SCV000454844	Uncertain significance	Illumina Clinical Se	10/18/2016	SLC26A2	chr5	149361285	149361285	http://gnomad.b	4.74E-05	NFE	0.0001085480035	0		0	0	https://www.ncbi.nlm.nih.gov/clinvar/variation/352031/
NM_000255.3(MUT):c.1808+15T>C	concordant	unreportable	unreportable	357255	0	2	SCV000524963;S(	Likely benign;Uncertain sig	GeneDx;Illumina C	2017-11-28;2016-1	MUT	chr6	49409538	49409538	http://gnomad.b	0.0004795473073	ASJ	0.002543749986	0		0	0	https://www.ncbi.nlm.nih.gov/clinvar/variation/357255/
NM_000255.3(MUT):c.1332+3A>C	concordant	unreportable	unreportable	357261	0	2	SCV000576617;S(	Uncertain significance;Unc	GeneDx;Illumina C	2017-11-28;2016-1	MUT	chr6	49419176	49419176	http://gnomad.b	3.20E-05	AFR	0.0007196129882	0		0	0	https://www.ncbi.nlm.nih.gov/clinvar/variation/357261/
NM_138694.3(PKHD1):c.12038G>A	concordant	unreportable	unreportable	357402	0	1	SCV000464021	Uncertain significance	Illumina Clinical Se	10/18/2016	PKHD1	chr6	51484066	51484066	http://gnomad.b	2.78E-05	AFR	6.54E-05	0		0	0	https://www.ncbi.nlm.nih.gov/clinvar/variation/357402/
NM_138694.3(PKHD1):c.11836T>C	concordant	unreportable	unreportable	287519	0	1	SCV000341308	Uncertain significance	EGL Genetic Diagn	6/30/2017	PKHD1	chr6	51484268	51484268	http://gnomad.b	4.17E-05	NFE	0.0002159709984	0		0	0	https://www.ncbi.nlm.nih.gov/clinvar/variation/287519/
NM_138694.3(PKHD1):c.10940A>C	concordant	unreportable	unreportable	501330	0	1	SCV000707651	Uncertain significance	EGL Genetic Diagn	6/30/2017	PKHD1	chr6	51523984	51523984	http://gnomad.b	5.28E-05	AFR	0.0001307360071	0		0	0	https://www.ncbi.nlm.nih.gov/clinvar/variation/501330/
NM_138694.3(PKHD1):c.8440+10G>C	concordant	unreportable	unreportable	288152	0	1	SCV000342175	Uncertain significance	EGL Genetic Diagn	6/30/2017	PKHD1	chr6	51656024	51656024	http://gnomad.b	3.89E-05	NFE	0.0002062970016	0		0	0	https://www.ncbi.nlm.nih.gov/clinvar/variation/288152/
NM_138694.3(PKHD1):c.8174-10dupT	concordant	unreportable	unreportable	197942	0	2	SCV000464054;S(	Uncertain significance;Beni	Illumina Clinical Se	2016-10-18;2016-0	PKHD1	chr6	51695796	51695796	http://gnomad.b	0.0003836359795	AFR	0.01071900036	2		0	0	https://www.ncbi.nlm.nih.gov/clinvar/variation/197942/
NM_138694.3(PKHD1):c.8173+12C>T	concordant	unreportable	unreportable	262418	0	1	SCV000464055	Uncertain significance	Illumina Clinical Se	10/18/2016	PKHD1	chr6	51701190	51701190	http://gnomad.b	3.34E-05	NFE	0.0001075399996	0		0	0	https://www.ncbi.nlm.nih.gov/clinvar/variation/262418/
NM_138694.3(PKHD1):c.8156T>C	concordant	unreportable	unreportable	357426	0	1	SCV000464056	Uncertain significance	Illumina Clinical Se	10/18/2016	PKHD1	chr6	51701219	51701219	http://gnomad.b	1.95E-05	NFE	8.96E-06	0		0	0	https://www.ncbi.nlm.nih.gov/clinvar/variation/357426/
NM_138694.3(PKHD1):c.8107+8T>G	concordant	unreportable	unreportable	502032	0	1	SCV000708624	Uncertain significance	EGL Genetic Diagn	6/30/2017	PKHD1	chr6	51712565	51712565	http://gnomad.b	6.95E-05	NFE	0.0002512469946	0		0	0	https://www.ncbi.nlm.nih.gov/clinvar/variation/502032/
NM_138694.3(PKHD1):c.7912-5T>G	concordant	unreportable	unreportable	96425	0	3	SCV000114617;S(	Uncertain significance;Unc	EGL Genetic Diagn	2017-06-30;2016-1	PKHD1	chr6	51712773	51712773	http://gnomad.b	0.0005698940275	SAS	0.001786989975	0		2	0	https://www.ncbi.nlm.nih.gov/clinvar/variation/96425/
NM_138694.3(PKHD1):c.7887C>T	concordant	unreportable	unreportable	499612	0	1	SCV000705197	Uncertain significance	EGL Genetic Diagn	6/30/2017	PKHD1	chr6	51720715	51720715	http://gnomad.b	3.34E-05	AFR	0.0003920540039	0		0	0	https://www.ncbi.nlm.nih.gov/clinvar/variation/499612/
NM_138694.3(PKHD1):c.7734-6C>A	concordant	unreportable	unreportable	502737	0	1	SCV000709595	Uncertain significance	EGL Genetic Diagn	6/30/2017	PKHD1	chr6	51720874	51720874	http://gnomad.b	1.39E-05	AFR	0.0008497840026	0		0	0	https://www.ncbi.nlm.nih.gov/clinvar/variation/502737/
NM_138694.3(PKHD1):c.6774A>C	concordant	unreportable	unreportable	262410	0	2	SCV000335693;S(	Uncertain significance;Likel	EGL Genetic Diagn	2017-06-30;2017-0	PKHD1	chr6	51771047	51771047	http://gnomad.b	0.000144558485	AFR	0.001831019996	1		1	0	https://www.ncbi.nlm.nih.gov/clinvar/variation/262410/
NM_138694.3(PKHD1):c.6565G>A	concordant	unreportable	unreportable	501080	0	1	SCV000707304	Uncertain significance	EGL Genetic Diagn	6/30/2017	PKHD1	chr6	51774198	51774198	http://gnomad.b	7.78E-05	SAS	0.0002923790016	0		0	0	https://www.ncbi.nlm.nih.gov/clinvar/variation/501080/
NM_138694.3(PKHD1):c.6042C>T	concordant	unreportable	unreportable	500569	0	1	SCV000706576	Uncertain significance	EGL Genetic Diagn	6/30/2017	PKHD1	chr6	51798987	51798987	http://gnomad.b	8.34E-06	EAS	0.0001159550011	0		0	0	https://www.ncbi.nlm.nih.gov/clinvar/variation/500569/
NM_138694.3(PKHD1):c.5324G>A	concordant	unreportable	unreportable	290385	0	1	SCV000344931	Uncertain significance	EGL Genetic Diagn	6/30/2017	PKHD1	chr6	51887655	51887655	http://gnomad.b	5.56E-05	NFE	4.48E-05	0		0	0	https://www.ncbi.nlm.nih.gov/clinvar/variation/290385/
NM_138694.3(PKHD1):c.4606G>A	concordant	unreportable	unreportable	500568	0	1	SCV000706575	Uncertain significance	EGL Genetic Diagn	6/30/2017	PKHD1	chr6	51890002	51890002	http://gnomad.b	2.78E-05	EAS	0.000174033994	0		0	0	https://www.ncbi.nlm.nih.gov/clinvar/variation/500568/
NM_138694.3(PKHD1):c.4447G>A	concordant	unreportable	unreportable	289885	0	1	SCV000344319	Uncertain significance	EGL Genetic Diagn	6/30/2017	PKHD1	chr6	51890161	51890161	http://gnomad.b	5.56E-05	AFR	0.0005892370245	0		0	0	https://www.ncbi.nlm.nih.gov/clinvar/variation/289885/
NM_138694.3(PKHD1):c.4380C>T	concordant	unreportable	unreportable	500250	0	1	SCV000706111	Uncertain significance	EGL Genetic Diagn	6/30/2017	PKHD1	chr6	51890228	51890228	http://gnomad.b	2.78E-05	SAS	0.0002025660069	0		0	0	https://www.ncbi.nlm.nih.gov/clinvar/variation/500250/
NM_138694.3(PKHD1):c.2788A>G	concordant	unreportable	unreportable	498399	0	1	SCV000703390	Uncertain significance	EGL Genetic Diagn	6/30/2017	PKHD1	chr6	51908456	51908456	http://gnomad.b	5.56E-06	NFE	1.79E-05	0		0	0	https://www.ncbi.nlm.nih.gov/clinvar/variation/498399/
NM_138694.3(PKHD1):c.2715+7delG	concordant	unreportable	unreportable	195911	0	3	SCV000228271;S(	Benign;Uncertain significar	EGL Genetic Diagn	2017-06-30;2016-1	PKHD1	chr6	51909756	51909757	http://gnomad.b	0.0003391564456	AFR	0.00908378046	1		1	0	https://www.ncbi.nlm.nih.gov/clinvar/variation/195911/
NM_138694.3(PKHD1):c.2280-9G>A	concordant	unreportable	unreportable	289887	0	1	SCV000344321	Uncertain significance	EGL Genetic Diagn	6/30/2017	PKHD1	chr6	51913426	51913426	http://gnomad.b	1.39E-05	AFR	0.0001960780064	0		0	0	https://www.ncbi.nlm.nih.gov/clinvar/variation/289887/
NM_138694.3(PKHD1):c.2090C>T	concordant	unreportable	unreportable	497809	0	1	SCV000702528	Uncertain significance	EGL Genetic Diagn	6/30/2017	PKHD1	chr6	51917924	51917924	http://gnomad.b	3.34E-05	AFR	6.53E-05	0		0	0	https://www.ncbi.nlm.nih.gov/clinvar/variation/497809/
NM_138694.3(PKHD1):c.1602+4G>A	concordant	unreportable	unreportable	501166	0	1	SCV000707421	Uncertain significance	EGL Genetic Diagn	6/30/2017	PKHD1	chr6	51921684	51921684	http://gnomad.b	1.67E-05	AFR	0.0001962069946	0		0	0	https://www.ncbi.nlm.nih.gov/clinvar/variation/501166/
NM_138694.3(PKHD1):c.930delC	concordant	reportable	reportable	96442	1	0	SCV000260958	Pathogenic	Invitae	1/6/2016	PKHD1	chr6	51929798	51929799	http://gnomad.b	3.34E-05	AFR	0.0001307189959	0		0	0	https://www.ncbi.nlm.nih.gov/clinvar/variation/96442/
NM_138694.3(PKHD1):c.779-12delT	concordant	unreportable	unreportable	357455	0	1	SCV000464124	Uncertain significance	Illumina Clinical Se	10/18/2016	PKHD1	chr6	51930886	51930887		2.78E-06					0	0	https://www.ncbi.nlm.nih.gov/clinvar/variation/357455/
NM_138694.3(PKHD1):c.708-5C>T	concordant	unreportable	unreportable	502781	0	1	SCV000709644	Uncertain significance	EGL Genetic Diagn	6/30/2017	PKHD1	chr6	51934330	51934330	http://gnomad.b	5.28E-05	NFE	7.17E-05	0		0	0	https://www.ncbi.nlm.nih.gov/clinvar/variation/502781/
NM_138694.3(PKHD1):c.449-9_449-7d	concordant	unreportable	unreportable	198616	0	1	SCV000291334	Likely benign	Invitae	10/5/2017	PKHD1	chr6	51938345	51938348	http://gnomad.b	0.0003780760378	AFR	0.0107942	2		0	0	https://www.ncbi.nlm.nih.gov/clinvar/variation/198616/
NM_138694.3(PKHD1):c.192C>T	concordant	unreportable	unreportable	357458	0	1	SCV000464132	Uncertain significance	Illumina Clinical Se	10/18/2016	PKHD1	chr6	51947279	51947279	http://gnomad.b	2.78E-05	AMR	5.96E-05	0		0	0	https://www.ncbi.nlm.nih.gov/clinvar/variation/357458/
NM_138694.3(PKHD1):c.130+9G>A	concordant	unreportable	unreportable	262388	0	1	SCV000709028	Uncertain significance	EGL Genetic Diagn	6/30/2017	PKHD1	chr6	51947967	51947967	http://gnomad.b	3.06E-05	AFR	0.0006535950233	0		0	0	https://www.ncbi.nlm.nih.gov/clinvar/variation/262388/
NM_012434.4(SLC17A5):c.1272C>A	concordant	unreportable	unreportable	357922	0	1	SCV000464772	Uncertain significance	Illumina Clinical Se	10/18/2016	SLC17A5	chr6	74310152	74310152	http://gnomad.b	1.19E-05	NFE	4.48E-05	0		0	0	https://www.ncbi.nlm.nih.gov/clinvar/variation/357922/
NM_183050.2(BCKDHB):c.245A>G	concordant	unreportable	unreportable	358170	0	1	SCV000465666	Uncertain significance	Illumina Clinical Se	10/18/2016	BCKDHB	chr6	80837312	80837312	http://gnomad.b	3.59E-05	NFE	2.69E-05	0		0	0	https://www.ncbi.nlm.nih.gov/clinvar/variation/358170/
NM_000288.3(PEX7):c.339+10A>G	concordant	unreportable	unreportable	291202	0	1	SCV000345914	Uncertain significance	EGL Genetic Diagn	6/30/2017	PEX7	chr6	137147617	137147617	http://gnomad.b	6.94E-05	AFR	0.00313683995	0		0	0	https://www.ncbi.nlm.nih.gov/clinvar/variation/291202/
NM_000288.3(PEX7):c.804-5C>T	concordant	unreportable	unreportable	355535	0	1	SCV000460546	Uncertain significance	Illumina Clinical Se	10/18/2016	PEX7	chr6	137219275	137219275	http://gnomad.b	1.11E-05	NFE	6.28E-05	0		0	0	https://www.ncbi.nlm.nih.gov/clinvar/variation/355535/
NM_000288.3(PEX7):c.941A>G	concordant	unreportable	unreportable	355536	0	2	SCV000703705;S(	Uncertain significance;Unc	EGL Genetic Diagn	2017-06-30;2016-1	PEX7	chr6	137234633	137234633	http://gnomad.b	6.39E-05	NFE	9.85E-05	0		0	0	https://www.ncbi.nlm.nih.gov/clinvar/variation/355536/
NM_000466.2(PEX1):c.3419A>G	concordant	unreportable	unreportable	252586	0	1	SCV000297098	Uncertain significance	Division of Genomi	1/6/2016	PEX1	chr7	92120605	92120605	http://gnomad.b	2.77E-06	NFE	1.79E-05	0		0	0	https://www.ncbi.nlm.nih.gov/clinvar/variation/252586/
NM_000466.2(PEX1):c.2750C>T	concordant	unreportable	unreportable	288721	0	1	SCV000342913	Uncertain significance	EGL Genetic Diagn	6/30/2017	PEX1	chr7	92126060	92126060	http://gnomad.b	1.11E-05	AFR	0.0002616769925	0		0	0	https://www.ncbi.nlm.nih.gov/clinvar/variation/288721/
NM_000466.2(PEX1):c.2509C>T	concordant	unreportable	unreportable	497947	0	1	SCV000702724	Uncertain significance	EGL Genetic Diagn	6/30/2017	PEX1	chr7	92130895	92130895	http://gnomad.b	3.05E-05	AFR	0.0003922590113	0		0	0	https://www.ncbi.nlm.nih.gov/clinvar/variation/497947/
NM_000466.2(PEX1):c.2072C>T	concordant	unreportable	unreportable	360925	0	1	SCV000470515	Uncertain significance	Illumina Clinical Se	10/18/2016	PEX1	chr7	92132509	92132509	http://gnomad.b	5.55E-06	NFE	3.59E-05	0		0	0	https://www.ncbi.nlm.nih.gov/clinvar/variation/360925/
NM_000466.2(PEX1):c.1587+5T>C	concordant	unreportable	unreportable	426135	0	1	SCV000576503	Uncertain significance	GeneDx	11/28/2017	PEX1	chr7	92140253	92140253	http://gnomad.b	0.0001358989577	ASJ	0.002900349908	0		0	0	https://www.ncbi.nlm.nih.gov/clinvar/variation/426135/
NM_000466.2(PEX1):c.1360-4G>T	concordant	unreportable	unreportable	288457	0	2	SCV000342566;S(	Uncertain significance;Unc	EGL Genetic Diagn	2017-06-30;2016-1	PEX1	chr7	92141021	92141021	http://gnomad.b	0.000119258269	NFE	0.0007354000118	0		0	0	https://www.ncbi.nlm.nih.gov/clinvar/variation/288457/
NM_000466.2(PEX1):c.1360-7C>T	concordant	unreportable	unreportable	286647	0	1	SCV000340170	Uncertain significance	EGL Genetic Diagn	6/30/2017	PEX1	chr7	92141024	92141024	http://gnomad.b	0.0001525396464	ASJ	0.002238499932	0		0	0	https://www.ncbi.nlm.nih.gov/clinvar/variation/286647/
NM_000466.2(PEX1):c.1271T>C	concordant	unreportable	unreportable	499334	0	1	SCV000704773	Uncertain significance	EGL Genetic Diagn	6/30/2017	PEX1	chr7	92143250	92143250	http://gnomad.b	6.38E-05	AFR	0.0001311300002	0		0	0	https://www.ncbi.nlm.nih.gov/clinvar/variation/499334/
NM_000466.2(PEX1):c.453G>A	concordant	unreportable	unreportable	288024	0	1	SCV000342004	Uncertain significance	EGL Genetic Diagn	6/30/2017	PEX1	chr7	92147474	92147474	http://gnomad.b	3.33E-05	SAS	3.25E-05	0		0	0	https://www.ncbi.nlm.nih.gov/clinvar/variation/288024/
NM_000466.2(PEX1):c.293C>T	concordant	unreportable	unreportable	288355	0	1	SCV000342441	Uncertain significance	EGL Genetic Diagn	6/30/2017	PEX1	chr7	92148373	92148373	http://gnomad.b	3.88E-05	AFR	0.0005883889971	0		0	0	https://www.ncbi.nlm.nih.gov/clinvar/variation/288355/
NM_000466.2(PEX1):c.130-9T>C	concordant	unreportable	unreportable	195076	0	2	SCV000227130;S(	Likely benign;Uncertain sig	EGL Genetic Diagn	2017-06-30;2016-1	PEX1	chr7	92151568	92151568	http://gnomad.b	0.0001275786134	AFR	0.005495939869	0		1	0	https://www.ncbi.nlm.nih.gov/clinvar/variation/195076/

Supplemental Table 2

NM_000466.2(PEX1):c.-19A>G	concordant	unreportable	unreportable	360933	0		1	SCV000470530	Uncertain significance	Illumina Clinical Se	10/18/2016	PEX1	chr7	92157768	92157768	http://gnomad.b	0.0001220317172	NFE	0.0004810820101	0		0	0	https://www.ncbi.nlm.nih.gov/clinvar/variation/360933/
NM_000441.1(SLC26A4):c.1544+9C>T	concordant	unreportable	unreportable	165255	0		1	SCV000708710	Benign	EGL Genetic Diagn	6/30/2017	SLC26A4	chr7	107336493	107336493	http://gnomad.b	0.0004383540225	SAS	0.01305960026	4		1	0	https://www.ncbi.nlm.nih.gov/clinvar/variation/165255/
NM_000108.3(DLD):c.-8G>T	concordant	unreportable	unreportable	358563	0		1	SCV000466197	Uncertain significance	Illumina Clinical Se	10/18/2016	DLD	chr7	107531688	107531688	http://gnomad.b	0.000106444846	ASJ	0.002845529933	0		0	0	https://www.ncbi.nlm.nih.gov/clinvar/variation/358563/
NM_000492.3(CFTR):c.274-6T>C	concordant	unreportable	unreportable	237855	0		5	SCV000603006;S(	Likely benign;Uncertain sig	ARUP Laboratories	2017-06-30;2017-0	CFTR	chr7	117170947	117170947	http://gnomad.b	0.0002638390312	NFE	0.0008182720048	0		0	0	https://www.ncbi.nlm.nih.gov/clinvar/variation/237855/
NM_000492.3(CFTR):c.744-9_744-6du	concordant	unreportable	unreportable	35889	0		1	SCV000601131	Likely benign	Quest Diagnostics I	8/1/2017	CFTR	chr7	117176568	117176568	http://gnomad.b	0.0004776064215	AFR	0.004648069851	1		0	0	https://www.ncbi.nlm.nih.gov/clinvar/variation/35889/
NM_000492.3(CFTR):c.900C>A	concordant	unreportable	unreportable	439493	0		1	SCV000603062	Likely benign	ARUP Laboratories	6/30/2017	CFTR	chr7	117180184	117180184	http://gnomad.b	3.85E-06	AFR	6.54E-05	0		0	0	https://www.ncbi.nlm.nih.gov/clinvar/variation/439493/
NM_000492.3(CFTR):c.972C>G	concordant	unreportable	unreportable	495965	0		1	SCV000697063	Uncertain significance	Laboratory Corpor	1/25/2018	CFTR	chr7	117180256	117180256	http://gnomad.b	1.73E-05	NFE	2.69E-05	0		0	0	https://www.ncbi.nlm.nih.gov/clinvar/variation/495965/
NM_000492.3(CFTR):c.1059A>G	concordant	unreportable	unreportable	495886	0		1	SCV000696820	Uncertain significance	Laboratory Corpor	1/25/2018	CFTR	chr7	117180343	117180343	http://gnomad.b	5.78E-06	EAS	5.80E-05	0		0	0	https://www.ncbi.nlm.nih.gov/clinvar/variation/495886/
NM_000492.3(CFTR):c.1679+18G>A	concordant	unreportable	unreportable	53334	0		1	SCV000696862	Uncertain significance	Laboratory Corpor	1/25/2018	CFTR	chr7	117227905	117227905	http://gnomad.b	1.35E-05	NFE	0.0001630350016	0		0	0	https://www.ncbi.nlm.nih.gov/clinvar/variation/53334/
NM_000492.3(CFTR):c.2490+19G>C	concordant	unreportable	unreportable	495911	0		1	SCV000696901	Uncertain significance	Laboratory Corpor	1/25/2018	CFTR	chr7	117232730	117232730	http://gnomad.b	7.70E-06	SAS	6.65E-05	0		0	0	https://www.ncbi.nlm.nih.gov/clinvar/variation/495911/
NM_000492.3(CFTR):c.2620-26A>G	concordant	unreportable	unreportable	53529	0		2	SCV000074651;S(	Likely benign;Uncertain sig	Invitae;Laboratory	2017-10-05;2018-0	CFTR	chr7	117242854	117242854	http://gnomad.b	0.0009744711664	ASJ	0.02609669976	3		5	0	https://www.ncbi.nlm.nih.gov/clinvar/variation/53529/
NM_000492.3(CFTR):c.2620-6T>C	concordant	unreportable	unreportable	53530	0		2	SCV000342915;S(	Uncertain significance;Unc	EGL Genetic Diagn	2017-06-30;2018-0	CFTR	chr7	117242874	117242874	http://gnomad.b	4.81E-05	FIN	0.00381165999	1		0	0	https://www.ncbi.nlm.nih.gov/clinvar/variation/53530/
NM_000492.3(CFTR):c.3140-33A>G	concordant	unreportable	unreportable	439483	0		1	SCV000603028	Likely benign	ARUP Laboratories	6/30/2017	CFTR	chr7	117251602	117251602	http://gnomad.b	3.27E-05	NFE	0.000112613001	0		0	0	https://www.ncbi.nlm.nih.gov/clinvar/variation/439483/
NM_000492.3(CFTR):c.3517G>A	concordant	unreportable	unreportable	497599	0		1	SCV000702209	Uncertain significance	EGL Genetic Diagn	6/30/2017	CFTR	chr7	117267624	117267624	http://gnomad.b	5.78E-05	AFR	0.0003924129996	0		0	0	https://www.ncbi.nlm.nih.gov/clinvar/variation/497599/
NM_000492.3(CFTR):c.3718-24G>A	concordant	unreportable	unreportable	499621	0		1	SCV000705210	Uncertain significance	EGL Genetic Diagn	6/30/2017	CFTR	chr7	117282468	117282468	http://gnomad.b	0.0002426548754	SAS	0.006920979824	3		1	0	https://www.ncbi.nlm.nih.gov/clinvar/variation/499621/
NM_000492.3(CFTR):c.4197C>G	concordant	unreportable	unreportable	289973	0		2	SCV000344443;S(	Uncertain significance;Unc	EGL Genetic Diagn	2017-06-30;2018-0	CFTR	chr7	117305573	117305573	http://gnomad.b	8.86E-05	AFR	0.001176779973	0		0	0	https://www.ncbi.nlm.nih.gov/clinvar/variation/289973/
NM_018941.3(CLN8):c.112G>A	concordant	unreportable	unreportable	420346	0		1	SCV000569153	Uncertain significance	GeneDx	11/28/2017	CLN8	chr8	1719332	1719332	http://gnomad.b	0.0001790911126	AFR	0.0006534240092	0		1	0	https://www.ncbi.nlm.nih.gov/clinvar/variation/420346/
NM_018941.3(CLN8):c.207G>A	concordant	unreportable	unreportable	261062	0		2	SCV000342666;S(	Uncertain significance;Likel	EGL Genetic Diagn	2017-06-30;2017-1	CLN8	chr8	1719427	1719427	http://gnomad.b	0.0001287217372	FIN	0.0008973440272	0		0	0	https://www.ncbi.nlm.nih.gov/clinvar/variation/261062/
NM_018941.3(CLN8):c.385C>T	concordant	unreportable	unreportable	195344	0		2	SCV000620010;S(	Uncertain significance;Unc	GeneDx;Invitae	2017-11-28;2017-1	CLN8	chr8	1719605	1719605	http://gnomad.b	6.16E-05	SAS	0.0001624330034	0		0	0	https://www.ncbi.nlm.nih.gov/clinvar/variation/195344/
NM_018941.3(CLN8):c.599T>C	concordant	unreportable	unreportable	205192	0		1	SCV000240700	Uncertain significance	GeneDx	11/28/2017	CLN8	chr8	1728471	1728471	http://gnomad.b	2.80E-05	AFR	0.0005880820099	0		0	0	https://www.ncbi.nlm.nih.gov/clinvar/variation/205192/
NM_000370.3(TTPA):c.663+10A>G	concordant	unreportable	unreportable	363559	0		1	SCV000474655	Uncertain significance	Illumina Clinical Se	10/18/2016	TTPA	chr8	63976755	63976755	http://gnomad.b	9.01E-06	NFE	1.84E-05	0		0	0	https://www.ncbi.nlm.nih.gov/clinvar/variation/363559/
NM_002485.4(NBN):c.2235-8C>T	concordant	unreportable	unreportable	416883	0		1	SCV000562940	Likely benign	Invitae	3/14/2017	NBN	chr8	90947848	90947848	http://gnomad.b	2.79E-06	NFE	2.71E-05	0		0	0	https://www.ncbi.nlm.nih.gov/clinvar/variation/416883/
NM_002485.4(NBN):c.2234+2T>G	concordant	reportable	reportable	185026	2		0	SCV000618013;S(	Likely pathogenic;Likely pat	GeneDx;Quest Dia	2017-11-28;2017-0	NBN	chr8	90949252	90949252	http://gnomad.b	1.12E-05	NFE	9.01E-06	0		0	0	https://www.ncbi.nlm.nih.gov/clinvar/variation/185026/
NM_002485.4(NBN):c.2215C>G	concordant	unreportable	unreportable	182730	0		4	SCV000690700;S(	Uncertain significance;Unc	Color Genomics, In	2017-12-21;2017-0	NBN	chr8	90949273	90949273	http://gnomad.b	3.90E-05	NFE	6.30E-05	0		0	0	https://www.ncbi.nlm.nih.gov/clinvar/variation/182730/
NM_002485.4(NBN):c.2036A>G	concordant	unreportable	unreportable	461536	0		1	SCV000634271	Uncertain significance	Invitae	10/5/2017	NBN	chr8	90958402	90958402	http://gnomad.b	2.79E-06	AFR	6.54E-05	0		0	0	https://www.ncbi.nlm.nih.gov/clinvar/variation/461536/
NM_002485.4(NBN):c.1915-8C>T	concordant	unreportable	unreportable	488774	0		1	SCV000680637	Uncertain significance	GeneDx	2/7/2018	NBN	chr8	90958531	90958531	http://gnomad.b	2.79E-06	AFR	6.54E-05	1		0	0	https://www.ncbi.nlm.nih.gov/clinvar/variation/488774/
NM_002485.4(NBN):c.1888T>C	concordant	unreportable	unreportable	211575	0		2	SCV000248134;S(	Uncertain significance;Unc	Genetic Services La	2015-09-15;2017-1	NBN	chr8	90960078	90960078		5.58E-06					0	0	https://www.ncbi.nlm.nih.gov/clinvar/variation/211575/
NM_002485.4(NBN):c.1873G>A	concordant	unreportable	unreportable	411774	0		1	SCV000553094	Uncertain significance	Invitae	10/5/2017	NBN	chr8	90960093	90960093		2.79E-06					0	0	https://www.ncbi.nlm.nih.gov/clinvar/variation/411774/
NM_002485.4(NBN):c.1825C>A	concordant	unreportable	unreportable	220517	0		1	SCV000261120	Uncertain significance	Invitae	3/14/2017	NBN	chr8	90965492	90965492	http://gnomad.b	8.37E-06	NFE	8.98E-06	0		0	0	https://www.ncbi.nlm.nih.gov/clinvar/variation/220517/
NM_002485.4(NBN):c.1405G>T	concordant	unreportable	unreportable	134873	0		4	SCV000685717;S(	Uncertain significance;Unc	Color Genomics, In	2017-10-26;2017-0	NBN	chr8	90965912	90965912	http://gnomad.b	5.58E-05	AFR	0.0004055149911	0		0	0	https://www.ncbi.nlm.nih.gov/clinvar/variation/134873/
NM_002485.4(NBN):c.1382C>T	concordant	unreportable	unreportable	461508	0		2	SCV000685713;S(	Uncertain significance;Unc	Color Genomics, In	2017-10-26;2017-1	NBN	chr8	90967526	90967526	http://gnomad.b	2.79E-05	AFR	0.0003923109907	0		0	0	https://www.ncbi.nlm.nih.gov/clinvar/variation/461508/
NM_002485.4(NBN):c.1306T>C	concordant	unreportable	unreportable	219677	0		1	SCV000259684	Likely benign	Invitae	10/5/2017	NBN	chr8	90967602	90967602	http://gnomad.b	1.95E-05	NFE	1.79E-05	0		0	0	https://www.ncbi.nlm.nih.gov/clinvar/variation/219677/
NM_002485.4(NBN):c.1124+6G>T	concordant	unreportable	unreportable	138426	0		3	SCV000690639;S(	Likely benign;Uncertain sig	Color Genomics, In	2017-12-21;2017-1	NBN	chr8	90970947	90970947	http://gnomad.b	0.0001031790296	ASJ	0.001726589981	0		0	0	https://www.ncbi.nlm.nih.gov/clinvar/variation/138426/
NM_002485.4(NBN):c.935T>C	concordant	unreportable	unreportable	142859	0		1	SCV000634343	Uncertain significance	Invitae	10/5/2017	NBN	chr8	90976697	90976697	http://gnomad.b	1.12E-05	AFR	0.0001960780064	0		0	0	https://www.ncbi.nlm.nih.gov/clinvar/variation/142859/
NM_002485.4(NBN):c.896+13T>G	concordant	unreportable	unreportable	492134	0		1	SCV000690747	Likely benign	Color Genomics, In	12/21/2017	NBN	chr8	90982579	90982579	http://gnomad.b	3.07E-05	AMR	0.0002692679991	0		0	0	https://www.ncbi.nlm.nih.gov/clinvar/variation/492134/
NM_002485.4(NBN):c.798G>A	concordant	unreportable	unreportable	382019	0		3	SCV000690741;S(	Likely benign;Likely benign	Color Genomics, In	2017-12-21;2017-1	NBN	chr8	90982690	90982690	http://gnomad.b	2.79E-05	SAS	6.50E-05	0		0	0	https://www.ncbi.nlm.nih.gov/clinvar/variation/382019/
NM_002485.4(NBN):c.786C>A	concordant	unreportable	unreportable	185571	0		2	SCV000279303;S(	Uncertain significance;Unc	GeneDx;Invitae	2017-11-28;2017-1	NBN	chr8	90982702	90982702	http://gnomad.b	3.90E-05	AFR	0.0005228070077	0		0	0	https://www.ncbi.nlm.nih.gov/clinvar/variation/185571/
NM_002485.4(NBN):c.783T>G	concordant	unreportable	unreportable	234702	0		1	SCV000634329	Uncertain significance	Invitae	10/5/2017	NBN	chr8	90982705	90982705	http://gnomad.b	2.79E-06	NFE	1.79E-05	0		0	0	https://www.ncbi.nlm.nih.gov/clinvar/variation/234702/
NM_002485.4(NBN):c.481-18_481-17(	concordant	unreportable	unreportable	418372	0		2	SCV000685789;S(	Likely benign;Likely benign	Color Genomics, In	2017-10-26;2017-1	NBN	chr8	90990567	90990569	http://gnomad.b	5.58E-06	SAS	6.52E-05	0		0	0	https://www.ncbi.nlm.nih.gov/clinvar/variation/418372/
NM_002485.4(NBN):c.393T>G	concordant	unreportable	unreportable	184328	0		2	SCV000526924;S(	Likely benign;Likely benign	GeneDx;Invitae	2017-11-28;2017-1	NBN	chr8	90993049	90993049		2.79E-06					0	0	https://www.ncbi.nlm.nih.gov/clinvar/variation/184328/
NM_002485.4(NBN):c.333G>A	concordant	unreportable	unreportable	183765	0		3	SCV000685773;S(	Likely benign;Likely benign	Color Genomics, In	2017-10-26;2017-1	NBN	chr8	90993109	90993109	http://gnomad.b	1.39E-05	NFE	2.69E-05	0		0	0	https://www.ncbi.nlm.nih.gov/clinvar/variation/183765/
NM_002485.4(NBN):c.211_212insGA	concordant	reportable	reportable	216086	1		0	SCV000253873	Pathogenic	Invitae	3/14/2017	NBN	chr8	90993711	90993711		1.12E-05					0	0	https://www.ncbi.nlm.nih.gov/clinvar/variation/216086/
NM_002485.4(NBN):c.38-20dupT	concordant	unreportable	unreportable	422261	0		2	SCV000685780;S(	Likely benign;Likely benign	Color Genomics, In	2017-10-26;2017-1	NBN	chr8	90995102	90995102	http://gnomad.b	1.95E-05	AFR	0.0003972979903	0		0	0	https://www.ncbi.nlm.nih.gov/clinvar/variation/422261/
NM_002485.4(NBN):c.37+10G>C	concordant	unreportable	unreportable	215822	0		3	SCV000685776;S(	Likely benign;Uncertain sig	Color Genomics, In	2017-10-26;2016-1	NBN	chr8	90996743	90996743	http://gnomad.b	7.25E-05	ASJ	0.001638339949	0		0	0	https://www.ncbi.nlm.nih.gov/clinvar/variation/215822/
NM_002485.4(NBN):c.37+7G>A	concordant	unreportable	unreportable	239197	0		2	SCV000690715;S(	Likely benign;Likely benign	Color Genomics, In	2017-12-21;2017-1	NBN	chr8	90996746	90996746	http://gnomad.b	5.58E-06	NFE	2.78E-05	0		0	0	https://www.ncbi.nlm.nih.gov/clinvar/variation/239197/
NM_002485.4(NBN):c.21C>T	concordant	unreportable	unreportable	389032	0		1	SCV000531449	Likely benign	GeneDx	11/28/2017	NBN	chr8	90996769	90996769	http://gnomad.b	2.79E-06	NFE	2.78E-05	0		0	0	https://www.ncbi.nlm.nih.gov/clinvar/variation/389032/
NM_002485.4(NBN):c.-15T>A	concordant	unreportable	unreportable	388901	0		1	SCV000531296	Likely benign	GeneDx	11/28/2017	NBN	chr8	90996804	90996804		8.37E-06					0	0	https://www.ncbi.nlm.nih.gov/clinvar/variation/388901/
NM_017890.4(VPS13B):c.1594C>T	concordant	unreportable	unreportable	448874	0		1	SCV000616266	Uncertain significance	Athena Diagnostics	8/17/2017	VPS13B	chr8	100148923	100148923	http://gnomad.b	5.60E-05	NFE	0.0001164459973	0		0	0	https://www.ncbi.nlm.nih.gov/clinvar/variation/448874/
NM_017890.4(VPS13B):c.1843+10T>C	concordant	unreportable	unreportable	361043	0		1	SCV000470772	Uncertain significance	Illumina Clinical Se	10/18/2016	VPS13B	chr8	100155403	100155403	http://gnomad.b	5.04E-05	NFE	0.0001705169998	0		0	0	https://www.ncbi.nlm.nih.gov/clinvar/variation/361043/
NM_017890.4(VPS13B):c.2473G>A	concordant	unreportable	unreportable	194773	0		2	SCV000226662;S(	Uncertain significance;Unc	EGL Genetic Diagn	2017-06-30;2017-0	VPS13B	chr8	100205243	100205243	http://gnomad.b	0.000162378566	AFR	0.0002614720142	0		1	0	https://www.ncbi.nlm.nih.gov/clinvar/variation/194773/
NM_017890.4(VPS13B):c.3083-9C>T	concordant	unreportable	unreportable	361053	0		1	SCV000470788	Uncertain significance	Illumina Clinical Se	10/18/2016	VPS13B	chr8	100443756	100443756	http://gnomad.b	5.04E-05	NFE	0.0002779370116	0		0	0	https://www.ncbi.nlm.nih.gov/clinvar/variation/361053/
NM_017890.4(VPS13B):c.4337A>G	concordant	unreportable	unreportable	95854	0		2	SCV000597884;S(	Uncertain significance;Unc	Genetic Services La	2017-07-05;2016-1	VPS13B	chr8	100523369	100523369	http://gnomad.b	8.96E-05	NFE	0.000134818998	0		0	0	https://www.ncbi.nlm.nih.gov/clinvar/variation/95854/
NM_017890.4(VPS13B):c.5025-9A>G	concordant	unreportable	unreportable	196790	0		1	SCV000616272	Uncertain significance	Athena Diagnostics	8/17/2017	VPS13B	chr8	100587877	100587877	http://gnomad.b	1.68E-05	NFE	5.37E-05	0		0	0	https://www.ncbi.nlm.nih.gov/clinvar/variation/196790/
NM_017890.4(VPS13B):c.8234T>C	concordant	unreportable	unreportable	448880	0		1	SCV000616277	Uncertain significance	Athena Diagnostics	8/17/2017	VPS13B	chr8	100829829	100829829	http://gnomad.b	5.60E-06	NFE	8.99E-06	0		0	0	https://www.ncbi.nlm.nih.gov/clinvar/variation/448880/
NM_017890.4(VPS13B):c.8276G>A	concordant	unreportable	unreportable	286967	0		1	SCV000340583	Uncertain significance	EGL Genetic Diagn	6/30/2017	VPS13B	chr8	100829871	100829871	http://gnomad.b	5.60E-05	SAS	0.0003898639989	0		0	0	https://www.ncbi.nlm.nih.gov/clinvar/variation/286967/
NM_017890.4(VPS13B):c.9258+6A>G	concordant	unreportable	unreportable	288892	0		1	SCV000343135	Uncertain significance	EGL Genetic Diagn	6/30/2017	VPS13B	chr8	100833716	100833716	http://gnomad.b	5.60E-06	NFE	8.97E-06	0		0	0	https://www.ncbi.nlm.nih.gov/clinvar/variation/288892/
NM_017890.4(VPS13B):c.10701G>A	concordant	unreportable	unreportable	437250	0		1	SCV000597908	Uncertain significance	Genetic Services La	7/5/2017	VPS13B	chr8	100866243	100866243	http://gnomad.b	6.72E-05	AMR	0.0001786670036	0		0	0	https://www.ncbi.nlm.nih.gov/clinvar/variation/437250/
NM_017890.4(VPS13B):c.10830C>T	concordant	unreportable	unreportable	361092	0		1	SCV000470856	Uncertain significance	Illumina Clinical Se	10/18/2016	VPS13B	chr8	100866372	100866372	http://gnomad.b	7.00E-05	NFE	0.0001253509981	0		0	0	https://www.ncbi.nlm.nih.gov/clinvar/variation/361092/
NM_000170.2(GLDC):c.2487C>T	concordant	unreportable	unreportable	462873	0		1	SCV000636387	Likely benign	Invitae	10/5/2017	GLDC	chr9	6550885	6550885	http://gnomad.b	0.00193671271	NFE	0.002891259966	2		0	0	https://www.ncbi.nlm.nih.gov/clinvar/variation/462873/
NM_000170.2(GLDC):c.2316-6delA	concordant	unreportable	unreportable	462870	0		1	SCV000636383	Benign	Invitae	10/5/2017	GLDC	chr9	6553514	6553515	http://gnomad.b	4.00E-05	SAS	0.0002929120092	0		0	0	https://www.ncbi.nlm.nih.gov/clinvar/variation/462870/
NM_000170.2(GLDC):c.2244G>A	concordant	unreportable	unreportable	462867	0		1	SCV000636378	Likely benign	Invitae	10/5/2017	GLDC	chr9	6554740	6554740	http://gnomad.b	0.0002320854074	EAS	0.0005230130046	0		0	0	https://www.ncbi.nlm.nih.gov/clinvar/variation/462867/
NM_000170.2(GLDC):c.2147T>A	concordant	unreportable	unreportable	367183	0		1	SCV000480503	Uncertain significance	Illumina Clinical Se	10/18/2016	GLDC	chr9	6556208	6556208	http://gnomad.b	0.0001520559566	NFE	0.0004745449987	0		0	0	https://www.ncbi.nlm.nih.gov/clinvar/variation/367183/
NM_000170.2(GLDC):c.1932C>T	concordant	unreportable	unreportable	462863	0		1	SCV000636371	Likely benign	Invitae	10/5/2017	GLDC	chr9	6558679	6558679	http://gnomad.b	9.60E-05	AFR	0.0004573969927	0		0	0	https://www.ncbi.nlm.nih.gov/clinvar/variation/462863/
NM_000170.2(GLDC):c.1752T>C	concordant	unreportable	unreportable	462859	0		1	SCV000636365	Likely benign	Invitae	10/5/2017	GLDC	chr9	6587239	6587239	http://gnomad.b	0.0001680618468	NFE	0.0001969419973	0		0	0	https://www.ncbi.nlm.nih.gov/clinvar/variation/462859/
NM_000170.2(GLDC):c.1483-15G>C	concordant	unreportable	unreportable	367191	0		1	SCV000480512	Uncertain significance	Illumina Clinical Se	10/18/2016	GLDC	chr9	6589307	6589307	http://gnomad.b	0.0002240824623	SAS	0.0007806399954	0		0	0	https://www.ncbi.nlm.nih.gov/clinvar/variation/367191/
NM_000170.2(GLDC):c.1000T>C	concordant	unreportable	unreportable	367196	0		1	SCV000480519	Uncertain significance	Illumina Clinical Se	10/18/2016	GLDC	chr9	6604646	6604646	http://gnomad.b	0.0003201178034	NFE	0.0006359270192	0		0	0	https://www.ncbi.nlm.nih.gov/clinvar/variation/367196/
NM_000170.2(GLDC):c.698T>C	concordant	unreportable	unreportable	367199	0		1	SCV000480522	Uncertain significance	Illumina Clinical Se	10/18/2016	GLDC	chr9	6606607	6606607	http://gnomad.b	0.0002160795173	NFE	0.0003580639896	0		0	0	https://www.ncbi.nlm.nih.gov/clinvar/variation/367199/
NM_000170.2(GLDC):c.498T>C	concordant	unreportable	unreportable	255458	0		1	SCV000636406	Benign	Invitae	10/5/2017	GLDC	chr9	6610329	6610329	http://gnomad.b	0.001992733326	ASJ	0.008741609752	0		0	0	https://www.ncbi.nlm.nih.gov/clinvar/variation/255458/
NM_000155.3(GALT):c.405G>A	concordant	unreportable	unreportable	281069	0		1	SCV000331211	Uncertain significance	EGL Genetic Diagn	6/30/2017	GALT	chr9	34647856	34647856		5.50E-06					0	0	https://www.ncbi.nlm.nih.gov/clinvar/variation/281069/
NM_000155.3(GALT):c.688-4C>T	concordant	unreportable	unreportable	366699	0		1	SCV000479765	Uncertain significance	Illumina Clinical Se	10/18/2016	GALT	chr9	34648755	34648755	http://gnomad.b	6.60E-05	NFE	0.0001438750041	0		0	0	https://www.ncbi.nlm.nih.gov/clinvar/variation/366699/
NM_000155.3(GALT):c.843G>A	concordant	unreportable	unreportable	366700	0		1	SCV000479766	Uncertain significance	Illumina Clinical Se	10/18/2016	GALT	chr9	34649017	34649017		5.50E-06					0	0	https://www.ncbi.nlm.nih.gov/clinvar/variation/366700/
NM_000155.3(GALT):c.875C>T	concordant	unreportable	unreportable	281084	0		1	SCV000331262	Uncertain significance	EGL Genetic Diagn	6/30/2017	GALT	chr9	34649049	34649049	http://gnomad.b	3.02E-05	AFR	6.53E-05	0		0	0	https://www.ncbi.nlm.nih.gov/clinvar/variation/281084/
NM_000155.3(GALT):c.1006A>G	concordant	unreportable	unreportable	280997	0		1	SCV000331017	Uncertain significance	EGL Genetic Diagn	6/30/2017	GALT	chr9	34649508	34649508	http://gnomad.b	8.25E-06	EAS	0.0001739330037	0		0	0	https://www.ncbi.nlm.nih.gov/clinvar/variation/280997/
NM_000155.3(GALT):c.*8G>A	concordant	unreportable	unreportable	439751	0		2	SCV000603801;S(	Uncertain significance;Unc	ARUP Laboratories	2017-06-30;2018-0	GALT	chr9	34650454	34650454	http://gnomad.b	0.0002913416558	ASJ	0.006504070014	0		1	0	https://www.ncbi.nlm.nih.gov/clinvar/variation/439751/
NM_001128227.2(GNE):c.1505-5C>T	concordant	unreportable	unreportable	282272	0		1	SCV000341643	Uncertain significance	EGL Genetic Diagn	11/3/2016	GNE	chr9	36223000	36223000	http://gnomad.b	6.44E-05	SAS	0.001916700043	0		0	0	https://www.ncbi.nlm.nih.gov/clinvar/variation/282272/
NM_005476.5(GNE):c.1234G>A	concordant	unreportable	unreportable	290219	0		1	SCV000344729	Uncertain significance	EGL Genetic Diagn	6/30/2017	GNE	chr9	36227292	36227292	http://gnomad.b	6.72E-05	EAS	0.0001739330037	0		0	0	https://www.ncbi.nlm.nih.gov/clinvar/variation/290219/
NM_001128227.2(GNE):c.258-18A>G	concordant	unreportable	unreportable	257525	0		1	SCV000525544	Likely benign	GeneDx	11/28/2017	GNE	chr9	36246497	36246497	http://gnomad.b	0.0001765002521	NFE	0.0004756039998	0		0	0	https://www.ncbi.nlm.nih.gov/clinvar/variation/257525/
NM_000136.2(FANCC):c.*7C>T	concordant	unreportable	unreportable	377849	0		1	SCV000594677	Uncertain significance	Genetic Services La	7/5/2017	FANCC	chr9	97863982	97863982	http://gnomad.b	9.51E-06	AFR	6.56E-05	0		0	0	https://www.ncbi.nlm.nih.gov/clinvar/variation/377849/
NM_000136.2(FANCC):c.1623T>G	concordant	unreportable	unreportable	215637	0		1	SCV000253078	Likely benign	Invitae	9/11/2015	FANCC	chr9	97864043	97864043	http://gnomad.b	2.38E-06	AFR	0.0004578160006	0		0	0	https://www.ncbi.nlm.nih.gov/clinvar/variation/215637/
NM_000136.2(FANCC):c.1297C>T	concordant	unreportable	unreportable	127531	0		2	SCV000149250;S(	Uncertain significance;Unc	GeneDx;Invitae	2017-11-28;2016-0	FANCC	chr9	97873777	97873777	http://gnomad.b	2.38E-06	ASJ	0.0001016469978	0		0	0	https://www.ncbi.nlm.nih.gov/clinvar/variation/127531/
NM_000136.2(FANCC):c.996+1G>T	concordant	reportable	reportable	265137	3		0	SCV000321625;S(	Likely pathogenic;Likely pat	GeneDx;Invitae;Lal	2017-11-28;2017-0	FANCC	chr9	97887367	97887367	http://gnomad.b	3.09E-05	AMR	2.98E-05	0		0	0	https://www.ncbi.nlm.nih.gov/clinvar/variation/265137/
NM_000136.2(FANCC):c.974C>T	concordant	unreportable	unreportable	127551	0		1	SCV000149274	Uncertain significance	GeneDx	11/28/2017	FANCC	chr9	97887390	97887390	http://gnomad.b	1.43E-05	AFR	0.0003267120046	0		0	0	https://www.ncbi.nlm.nih.gov/clinvar/variation/127551/
NM_000136.2(FANCC):c.844-10_844-8	concordant	unreportable	unreportable	219407	0		1	SCV000259259	Likely benign	Invitae	1/6/2016	FANCC	chr9	97888870	97888873		4.76E-06					0	0	https://www.ncbi.nlm.nih.gov/clinvar/variation/219407/
NM_000136.2(FANCC):c.843+5G>A	concordant	unreportable	unreportable	216291	0		2	SCV000566528;S(	Uncertain significance;Unc	GeneDx;Invitae	2017-11-28;2016-0	FANCC	chr9	97897623	97897623	http://gnomad.b	1.19E-05	AFR	0.0001308390056	0		0	0	https://www.ncbi.nlm.nih.gov/clinvar/variation/216291/
NM_000136.2(FANCC):c.826A>G	concordant	unreportable	unreportable	409662	0		1	SCV000549971	Uncertain significance	Invitae	10/5/2017	FANCC	chr9	97897645	97897645	http://gnomad.b	3.80E-05	NFE	5.37E-05	0		0	0	https://www.ncbi.nlm.nih.gov/clinvar/variation/409662/
NM_000136.2(FANCC):c.692_694delA(	concordant	unreportable	unreportable	182466	0		2	SCV000211032;S(	Uncertain significance;Unc	GeneDx;Invitae	2017-11-28;2017-0	FANCC	chr9	97897776	97897779	http://gnomad.b	2.14E-05	NFE	3.59E-05	0		0	0	https://www.ncbi.nlm.nih.gov/clinvar/variation/182466/
NM_000136.2(FANCC):c.672C>T	concordant	unreportable	unreportable	137275	0		1	SCV000283596	Likely benign	Invitae	6/10/2016	FANCC	chr9	97912219	97912219	http://gnomad.b	0.0001165273887	AFR	0.001491480041	0		0	0	https://www.ncbi.nlm.nih.gov/clinvar/variation/137275/
NM_000136.2(FANCC):c.522-4A>G	concordant	unreportable	unreportable	182499	0		1	SCV000253080	Likely benign	Invitae	10/5/2017	FANCC	chr9	97912373	97912373	http://gnomad.b	1.66E-05	AFR	0.0003926189966	0		0	0	https://www.ncbi.nlm.nih.gov/clinvar/variation/182499/
NM_000136.2(FANCC):c.487_490delG	concordant	reportable	reportable	182465	2		0	SCV000626250;S(	Pathogenic;Likely pathoger	Invitae;Laboratory	2017-10-05;2018-0	FANCC	chr9	97933391	97933395	http://gnomad.b	1.43E-05	AMR	0.0001490139985	0		0	0	https://www.ncbi.nlm.nih.gov/clinvar/variation/182465/
NM_000136.2(FANCC):c.345+8_345+9	concordant	unreportable	unreportable	237071	0		2	SCV000707951;S(	Uncertain significance;Likel	EGL Genetic Diagn	2017-06-30;2017-1	FANCC	chr9	98002921	98002923	http://gnomad.b	2.62E-05	NFE	0.0001616960071	0		0	0	https://www.ncbi.nlm.nih.gov/clinvar/variation/237071/
NM_000136.2(FANCC):c.345+6A>T	concordant	unreportable	unreportable	127539	0		2	SCV000149258;S(	Likely benign;Uncertain sig	GeneDx;Invitae	2017-11-28;2017-1	FANCC	chr9	98002925	98002925	http://gnomad.b	4.04E-05	NFE	0.0001794849959	0		0	0	https://www.ncbi.nlm.nih.gov/clinvar/variation/127539/
NM_000136.2(FANCC):c.250+16G>A	concordant	unreportable	unreportable	383579	0		1	SCV000524007	Likely benign	GeneDx	11/28/2017	FANCC	chr9	98009698	98009698	http://gnomad.b	9.51E-06	AFR	0.0003929270024	0		0	0	https://www.ncbi.nlm.nih.gov/clinvar/variation/383579/
NM_000136.2(FANCC):c.248A>G	concordant	unreportable	unreportable	220742	0		1	SCV000261532	Uncertain significance	Invitae	1/6/2016	FANCC	chr9	98009716	98009716	http://gnomad.b	1.90E-05	AFR	0.0002617120044	0		0	0	https://www.ncbi.nlm.nih.gov/clinvar/variation/220742/
NM_000136.2(FANCC):c.166-7T>C	concordant	unreportable	unreportable	219433	0		1	SCV000259298	Likely benign	Invitae	1/6/2016	FANCC	chr9	98009805	98009805	http://gnomad.b	3.09E-05	AFR	0.001051519997	0		0	0	https://www.ncbi.nlm.nih.gov/clinvar/variation/219433/
NM_000136.2(FANCC):c.6T>C	concordant	unreportable	unreportable	386410	0		1	SCV000528079	Likely benign	GeneDx	11/28/2017	FANCC	chr9	98011568	98011568	http://gnomad.b	2.38E-06	NFE	9.03E-06	0		0	0	https://www.ncbi.nlm.nih.gov/clinvar/variation/386410/
NM_000035.3(ALDOB):c.*12C>T	concordant	unreportable	unreportable	364307	0		2	SCV000518537;S(	Likely benign;Uncertain sig	GeneDx;Illumina C	2017-11-28;2016-1	ALDOB	chr9	104184079	104184079	http://gnomad.b	0.001469542947	ASJ	0.03253529966	11		3	0	https://www.ncbi.nlm.nih.gov/clinvar/variation/364307/
NM_000035.3(ALDOB):c.590A>G	concordant	unreportable	unreportable	500747	0		1	SCV000706819	Uncertain significance	EGL Genetic Diagn	6/30/2017	ALDOB	chr9	104188871	104188871	http://gnomad.b	0.0001466722327	AMR	0.0006253719912	0		0	0	https://www.ncbi.nlm.nih.gov/clinvar/variation/500747/

Supplemental Table 2

NM_001079802.1(FKTN):c.293C>G	concordant	unreportable	unreportable	459221	0		1	SCV000630819	Uncertain significance	Invitae	10/5/2017	FKTN	chr9	108363553	108363553	http://gnomad.b	1.77E-05	AFR	0.0003267969878	0		0	0	https://www.ncbi.nlm.nih.gov/clinvar/variation/459221/
NM_001079802.1(FKTN):c.342A>G	concordant	unreportable	unreportable	499924	0		1	SCV000705656	Uncertain significance	EGL Genetic Diagn	6/30/2017	FKTN	chr9	108363602	108363602	http://gnomad.b	3.19E-05	AFR	0.0003924129996	0		0	0	https://www.ncbi.nlm.nih.gov/clinvar/variation/499924/
NM_001079802.1(FKTN):c.647+12G>A	concordant	unreportable	unreportable	388171	0		1	SCV000530411	Likely benign	GeneDx	11/28/2017	FKTN	chr9	108366785	108366785	http://gnomad.b	4.96E-05	NFE	0.0001726329938	0		0	0	https://www.ncbi.nlm.nih.gov/clinvar/variation/388171/
NM_001079802.1(FKTN):c.703C>A	concordant	unreportable	unreportable	290215	0		2	SCV000344724;S	Uncertain significance;Unc	EGL Genetic Diagn	2017-06-30;2017-1	FKTN	chr9	108370155	108370155	http://gnomad.b	0.0001560161973	SAS	0.0005523780128	1		0	0	https://www.ncbi.nlm.nih.gov/clinvar/variation/290215/
NM_001079802.1(FKTN):c.781-9T>C	concordant	unreportable	unreportable	449025	0		1	SCV000616721	Uncertain significance	GeneDx	11/28/2017	FKTN	chr9	108377550	108377550	http://gnomad.b	1.42E-05	AFR	6.54E-05	0		0	0	https://www.ncbi.nlm.nih.gov/clinvar/variation/449025/
NM_001079802.1(FKTN):c.910+13C>T	concordant	unreportable	unreportable	257334	0		1	SCV000520381	Likely benign	GeneDx	11/28/2017	FKTN	chr9	108377701	108377701	http://gnomad.b	0.0001134663253	AMR	0.001935560023	1		0	0	https://www.ncbi.nlm.nih.gov/clinvar/variation/257334/
NM_001079802.1(FKTN):c.1056C>G	concordant	unreportable	unreportable	459217	0		1	SCV000630812	Uncertain significance	Invitae	10/5/2017	FKTN	chr9	108382226	108382226	http://gnomad.b	1.06E-05	NFE	1.79E-05	0		0	0	https://www.ncbi.nlm.nih.gov/clinvar/variation/459217/
NM_003640.3(IKBKAP):c.3869A>G	concordant	unreportable	unreportable	364552	0		2	SCV000618672;S	Uncertain significance;Unc	GeneDx;Illumina C	2017-11-28;2016-1	IKBKAP	chr9	111637237	111637237	http://gnomad.b	0.0001064579775	AFR	0.002875440056	0		0	0	https://www.ncbi.nlm.nih.gov/clinvar/variation/364552/
NM_003640.3(IKBKAP):c.3474C>T	concordant	unreportable	unreportable	364558	0		2	SCV000513274;S	Likely benign;Uncertain sig	GeneDx;Illumina C	2017-11-28;2016-1	IKBKAP	chr9	111641824	111641824	http://gnomad.b	0.0001774299625	SAS	0.001364610041	0		0	0	https://www.ncbi.nlm.nih.gov/clinvar/variation/364558/
NM_003640.3(IKBKAP):c.3417G>C	concordant	unreportable	unreportable	455965	0		1	SCV000626023	Uncertain significance	Invitae	10/5/2017	IKBKAP	chr9	111642375	111642375	http://gnomad.b	4.97E-05	NFE	0.0005821029772	0		0	0	https://www.ncbi.nlm.nih.gov/clinvar/variation/455965/
NM_003640.3(IKBKAP):c.3398G>A	concordant	unreportable	unreportable	426637	0		1	SCV000577133	Uncertain significance	GeneDx	11/28/2017	IKBKAP	chr9	111642394	111642394	http://gnomad.b	2.37E-06	AFR	6.54E-05	0		0	0	https://www.ncbi.nlm.nih.gov/clinvar/variation/426637/
NM_003640.3(IKBKAP):c.3280A>G	concordant	unreportable	unreportable	242294	0		3	SCV000705489;S	Uncertain significance;Unc	EGL Genetic Diagn	2017-06-30;2017-1	IKBKAP	chr9	111644410	111644410	http://gnomad.b	0.0003761515205	NFE	0.001695859944	2		1	0	https://www.ncbi.nlm.nih.gov/clinvar/variation/242294/
NM_003640.3(IKBKAP):c.3259G>A	concordant	unreportable	unreportable	364561	0		2	SCV000476552;S	Uncertain significance;Unc	Illumina Clinical Se	2016-10-18;2017-1	IKBKAP	chr9	111644431	111644431	http://gnomad.b	3.08E-05	NFE	7.18E-05	0		0	0	https://www.ncbi.nlm.nih.gov/clinvar/variation/364561/
NM_003640.3(IKBKAP):c.3222+14C>T	concordant	unreportable	unreportable	364562	0		2	SCV000528667;S	Likely benign;Uncertain sig	GeneDx;Illumina C	2017-11-28;2016-1	IKBKAP	chr9	111651598	111651598	http://gnomad.b	0.000170332764	SAS	0.003086420009	0		0	0	https://www.ncbi.nlm.nih.gov/clinvar/variation/364562/
NM_003640.3(IKBKAP):c.2803A>T	concordant	unreportable	unreportable	364564	0		1	SCV000476558	Uncertain significance	Illumina Clinical Se	10/18/2016	IKBKAP	chr9	111656280	111656280	http://gnomad.b	7.33E-05	NFE	8.08E-05	0		0	0	https://www.ncbi.nlm.nih.gov/clinvar/variation/364564/
NM_003640.3(IKBKAP):c.2189G>A	concordant	unreportable	unreportable	289151	0		1	SCV000343454	Uncertain significance	EGL Genetic Diagn	6/30/2017	IKBKAP	chr9	111662117	111662117	http://gnomad.b	1.42E-05	AFR	6.54E-05	0		0	0	https://www.ncbi.nlm.nih.gov/clinvar/variation/289151/
NM_003640.3(IKBKAP):c.2008T>C	concordant	unreportable	unreportable	364568	0		1	SCV000476568	Uncertain significance	Illumina Clinical Se	10/18/2016	IKBKAP	chr9	111663711	111663711	http://gnomad.b	1.18E-05	NFE	3.60E-05	0		0	0	https://www.ncbi.nlm.nih.gov/clinvar/variation/364568/
NM_003640.3(IKBKAP):c.1911T>C	concordant	unreportable	unreportable	364569	0		2	SCV000476571;S	Uncertain significance;Likel	Illumina Clinical Se	2016-10-18;2017-1	IKBKAP	chr9	111663808	111663808	http://gnomad.b	0.0001395782372	FIN	0.001793719945	1		1	0	https://www.ncbi.nlm.nih.gov/clinvar/variation/364569/
NM_003640.3(IKBKAP):c.1439_1441de	concordant	unreportable	unreportable	364575	0		1	SCV000476579	Uncertain significance	Illumina Clinical Se	10/18/2016	IKBKAP	chr9	111670603	111670606	http://gnomad.b	8.04E-05	NFE	0.0001971180027	0		0	0	https://www.ncbi.nlm.nih.gov/clinvar/variation/364575/
NM_003640.3(IKBKAP):c.1224G>A	concordant	unreportable	unreportable	455953	0		1	SCV000626003	Likely benign	Invitae	10/5/2017	IKBKAP	chr9	111673426	111673426	http://gnomad.b	2.37E-06			0		0	0	https://www.ncbi.nlm.nih.gov/clinvar/variation/455953/
NM_003640.3(IKBKAP):c.1214G>A	concordant	unreportable	unreportable	455952	0		1	SCV000626002	Uncertain significance	Invitae	10/5/2017	IKBKAP	chr9	111673436	111673436	http://gnomad.b	2.37E-06	NFE	4.48E-05	0		0	0	https://www.ncbi.nlm.nih.gov/clinvar/variation/455952/
NM_003640.3(IKBKAP):c.947C>T	concordant	unreportable	unreportable	364577	0		2	SCV000583006;S	Uncertain significance;Unc	GeneDx;Illumina C	2017-11-28;2016-1	IKBKAP	chr9	111678495	111678495	http://gnomad.b	5.91E-05	AFR	0.0008494509966	0		0	0	https://www.ncbi.nlm.nih.gov/clinvar/variation/364577/
NM_003640.3(IKBKAP):c.650-7C>T	concordant	unreportable	unreportable	455972	0		1	SCV000626031	Likely benign	Invitae	10/5/2017	IKBKAP	chr9	111681188	111681188	http://gnomad.b	4.97E-05	AMR	0.0004467740073	0		0	0	https://www.ncbi.nlm.nih.gov/clinvar/variation/455972/
NM_003640.3(IKBKAP):c.304-5C>T	concordant	unreportable	unreportable	364580	0		1	SCV000476592	Uncertain significance	Illumina Clinical Se	10/18/2016	IKBKAP	chr9	111689738	111689738	http://gnomad.b	1.66E-05	NFE	9.87E-05	0		0	0	https://www.ncbi.nlm.nih.gov/clinvar/variation/364580/
NM_003640.3(IKBKAP):c.154A>G	concordant	unreportable	unreportable	455955	0		1	SCV000626005	Uncertain significance	Invitae	10/5/2017	IKBKAP	chr9	111692198	111692198	http://gnomad.b	4.73E-06	NFE	0.0002150340006	0		0	0	https://www.ncbi.nlm.nih.gov/clinvar/variation/455955/
NM_003640.3(IKBKAP):c.80G>A	concordant	unreportable	unreportable	246586	0		1	SCV000294182	Uncertain significance	GeneDx	11/28/2017	IKBKAP	chr9	111693347	111693347	http://gnomad.b	1.18E-05	NFE	8.06E-05	0		0	0	https://www.ncbi.nlm.nih.gov/clinvar/variation/246586/
NM_003640.3(IKBKAP):c.22C>T	concordant	unreportable	unreportable	455958	0		1	SCV000626011	Uncertain significance	Invitae	10/5/2017	IKBKAP	chr9	111693405	111693405	http://gnomad.b	4.73E-06	NFE	8.95E-06	0		0	0	https://www.ncbi.nlm.nih.gov/clinvar/variation/455958/
NM_000050.4(ASS1):c.174+4C>A	concordant	unreportable	unreportable	365250	0		1	SCV000477617	Uncertain significance	Illumina Clinical Se	10/18/2016	ASS1	chr9	133329764	133329764	http://gnomad.b	7.24E-05	AMR	0.0002986140025	0		0	0	https://www.ncbi.nlm.nih.gov/clinvar/variation/365250/
NM_000050.4(ASS1):c.420+13T>G	concordant	unreportable	unreportable	365254	0		2	SCV000518688;S	Likely benign;Uncertain sig	GeneDx;Illumina C	2017-11-28;2016-1	ASS1	chr9	133339567	133339567	http://gnomad.b	0.000504102447	NFE	0.001378890011	0		0	0	https://www.ncbi.nlm.nih.gov/clinvar/variation/365254/
NM_000050.4(ASS1):c.458G>A	concordant	unreportable	unreportable	365255	0		1	SCV000477623	Uncertain significance	Illumina Clinical Se	10/18/2016	ASS1	chr9	133342149	133342149	http://gnomad.b	4.73E-05	AFR	0.0002615400008	0		0	0	https://www.ncbi.nlm.nih.gov/clinvar/variation/365255/
NM_000050.4(ASS1):c.773+45C>T	concordant	unreportable	unreportable	382470	0		1	SCV000522416	Likely benign	GeneDx	11/28/2017	ASS1	chr9	133355232	133355232	http://gnomad.b	8.36E-06	AFR	0.0001310270018	0		0	0	https://www.ncbi.nlm.nih.gov/clinvar/variation/382470/
NM_000531.5(OTC):c.429T>C	concordant	unreportable	unreportable	256370	0		1	SCV000523914	Benign	GeneDx	11/28/2017	OTC	chrX	38260570	38260570	http://gnomad.b	0.001016998402	AFR	0.01068380009	0	42	0	16	https://www.ncbi.nlm.nih.gov/clinvar/variation/256370/
NM_000531.5(OTC):c.626C>G	concordant	unreportable	unreportable	426468	0		1	SCV000576918	Uncertain significance	GeneDx	11/28/2017	OTC	chrX	38262956	38262956	http://gnomad.b	5.81E-05	ASJ	0.0001374569983	0	3	0	2	https://www.ncbi.nlm.nih.gov/clinvar/variation/426468/
NM_000206.2(IL2RG):c.855-8C>T	concordant	unreportable	unreportable	463386	0		1	SCV000637253	Likely benign	Invitae	10/5/2017	IL2RG	chrX	70328204	70328204	http://gnomad.b	2.90E-05	AFR	0.0007356550195	0	2	0	0	https://www.ncbi.nlm.nih.gov/clinvar/variation/463386/
NM_033056.3(PCDH15):c.1172A>G	concordant	unreportable	unreportable	300197	0		1	SCV000363221	Uncertain significance	Illumina Clinical Se	10/18/2016	PCDH15	chr10	55955576	55955576	http://gnomad.b	1.29E-05	NFE	8.95E-06	0		0	0	https://www.ncbi.nlm.nih.gov/clinvar/variation/300197/
NM_000492.3(CFTR):c.1500C>A	concordant	unreportable	unreportable	495899	0		1	SCV000696847	Uncertain significance	Laboratory Corpora	1/25/2018	CFTR	chr7	117199625	117199625	http://gnomad.b	1.35E-05	EAS	0.0006957330042	0		0	0	https://www.ncbi.nlm.nih.gov/clinvar/variation/495899/
NM_000352.3(ABCC8):c.3224T>C	concordant	unreportable	unreportable	303765	0		1	SCV000369293	Uncertain significance	Illumina Clinical Se	10/18/2016	ABCC8	chr11	17428274	17428274	http://gnomad.b	5.16E-06	NFE	1.79E-05	0		0	0	https://www.ncbi.nlm.nih.gov/clinvar/variation/303765/
NM_017739.3(POMGNT1):c.960C>G	concordant	unreportable	unreportable	284940	0		2	SCV000337762;S	Uncertain significance;Unc	EGL Genetic Diagn	2017-06-30;2016-1	POMGNT1	chr1	46659302	46659302	http://gnomad.b	0.0001511766583	FIN	0.0002243159979	0		0	0	https://www.ncbi.nlm.nih.gov/clinvar/variation/284940/
NM_017739.3(POMGNT1):c.1927T>G	concordant	unreportable	unreportable	289590	0		2	SCV000343975;S	Uncertain significance;Unc	EGL Genetic Diagn	2017-06-30;2016-1	POMGNT1	chr1	46654998	46654998	http://gnomad.b	0.0001091831421	ASJ	0.0002030460018	0		0	0	https://www.ncbi.nlm.nih.gov/clinvar/variation/289590/
NM_024685.3(BBS10):c.1918A>G	concordant	unreportable	unreportable	462955	0		1	SCV000636519	Uncertain significance	Invitae	10/5/2017	BBS10	chr12	76739847	76739847	http://gnomad.b	8.39E-06	SAS	3.26E-05	0		0	0	https://www.ncbi.nlm.nih.gov/clinvar/variation/462955/
NM_000136.2(FANCC):c.916G>A	concordant	unreportable	unreportable	456173	0		1	SCV000626264	Uncertain significance	Invitae	10/5/2017	FANCC	chr9	97887448	97887448	http://gnomad.b	9.51E-06	NFE	8.95E-06	0		0	0	https://www.ncbi.nlm.nih.gov/clinvar/variation/456173/
NM_000152.3(GAA):c.1488G>A	concordant	unreportable	unreportable	456381	0		1	SCV000626517	Likely benign	Invitae	10/5/2017	GAA	chr17	78084576	78084576	http://gnomad.b	1.67E-05	AFR	0.000130786997	0		0	0	https://www.ncbi.nlm.nih.gov/clinvar/variation/456381/
NM_000051.3(ATM):c.7566A>G	concordant	unreportable	unreportable	220378	0		2	SCV000565740;S	Uncertain significance;Unc	GeneDx;Invitae	2017-11-28;2017-0	ATM	chr11	108202221	108202221	http://gnomad.b	5.01E-05	EAS	0.0001741760061	0		0	0	https://www.ncbi.nlm.nih.gov/clinvar/variation/220378/
NM_000152.3(GAA):c.1274C>T	concordant	unreportable	unreportable	456372	0		1	SCV000626500	Uncertain significance	Invitae	10/5/2017	GAA	chr17	78082575	78082575		8.34E-06					0	0	https://www.ncbi.nlm.nih.gov/clinvar/variation/456372/
NM_002485.4(NBN):c.227C>A	concordant	unreportable	unreportable	140980	0		2	SCV000690705;S	Uncertain significance;Unc	Color Genomics, In	2017-12-21;2017-1	NBN	chr8	90993696	90993696	http://gnomad.b	2.23E-05	NFE	1.79E-05	0		0	0	https://www.ncbi.nlm.nih.gov/clinvar/variation/140980/
NM_000153.3(GALC):c.226G>A	concordant	unreportable	unreportable	381334	0		1	SCV000520538	Uncertain significance	GeneDx	11/28/2017	GALC	chr14	88454837	88454837	http://gnomad.b	5.59E-05	NFE	8.08E-05	0		0	0	https://www.ncbi.nlm.nih.gov/clinvar/variation/381334/
NM_000053.3(ATP7B):c.1230A>G	concordant	unreportable	unreportable	312393	0		1	SCV000384679	Uncertain significance	Illumina Clinical Se	10/18/2016	ATP7B	chr13	52548126	52548126	http://gnomad.b	5.05E-05	NFE	0.0001164389978	0		0	0	https://www.ncbi.nlm.nih.gov/clinvar/variation/312393/
NM_000159.2(GCDH):c.892G>A	concordant	reportable	reportable	429706	2		0	SCV000582339;S	Pathogenic;Pathogenic	GeneDx;Laborator	2017-11-28;2018-0	GCDH	chr19	13007763	13007763	http://gnomad.b	1.97E-05	AFR	0.000130941	0		0	0	https://www.ncbi.nlm.nih.gov/clinvar/variation/429706/
NM_000136.2(FANCC):c.997-3C>T	concordant	unreportable	unreportable	237079	0		1	SCV000283601	Uncertain significance	Invitae	6/10/2016	FANCC	chr9	97879675	97879675		1.19E-05					0	0	https://www.ncbi.nlm.nih.gov/clinvar/variation/237079/
NM_000018.3(ACADVL):c.1803G>A	concordant	unreportable	unreportable	373437	0		1	SCV000492029	Uncertain significance	GeneDx	12/21/2016	ACADVL	chr17	7128179	7128179	http://gnomad.b	7.36E-05	FIN	0.0005382130039	0		0	0	https://www.ncbi.nlm.nih.gov/clinvar/variation/373437/
NM_000071.2(CBS):c.1332C>T	concordant	unreportable	unreportable	413355	0		1	SCV000555949	Likely benign	Invitae	3/14/2017	CBS	chr21	44478970	44478970		2.25E-05					0	0	https://www.ncbi.nlm.nih.gov/clinvar/variation/413355/
NM_002485.4(NBN):c.1122A>G	concordant	unreportable	unreportable	234206	0		2	SCV000534120;S	Likely benign;Likely benign	GeneDx;Invitae	2017-11-28;2017-1	NBN	chr8	90970955	90970955		1.12E-05					0	0	https://www.ncbi.nlm.nih.gov/clinvar/variation/234206/
NM_000136.2(FANCC):c.626G>A	concordant	unreportable	unreportable	188350	0		2	SCV000567486;S	Uncertain significance;Unc	GeneDx;Invitae	2017-11-28;2016-0	FANCC	chr9	97912265	97912265	http://gnomad.b	5.23E-05	SAS	0.0002958970144	0		0	0	https://www.ncbi.nlm.nih.gov/clinvar/variation/188350/
NM_000152.3(GAA):c.1298A>G	concordant	unreportable	unreportable	456373	0		1	SCV000626503	Uncertain significance	Invitae	10/5/2017	GAA	chr17	78082599	78082599		2.50E-05					0	0	https://www.ncbi.nlm.nih.gov/clinvar/variation/456373/
NM_000018.3(ACADVL):c.1678+4A>T	concordant	unreportable	unreportable	373435	0		1	SCV000492027	Uncertain significance	GeneDx	12/21/2016	ACADVL	chr17	7127875	7127875	http://gnomad.b	1.70E-05	NFE	8.98E-06	0		0	0	https://www.ncbi.nlm.nih.gov/clinvar/variation/373435/
NM_000518.4(HBB):c.33C>T	concordant	unreportable	unreportable	439156	0		1	SCV000601293	Likely benign	Quest Diagnostics I	8/1/2017	HBB	chr11	5248219	5248219	http://gnomad.b	2.80E-05	ASJ	0.0002032519988	0		0	0	https://www.ncbi.nlm.nih.gov/clinvar/variation/439156/
NM_000391.3(TPP1):c.523C>T	concordant	unreportable	unreportable	198076	0		1	SCV000617039	Uncertain significance	GeneDx	11/28/2017	TPP1	chr11	6638370	6638370	http://gnomad.b	4.20E-05	AFR	6.54E-05	0		0	0	https://www.ncbi.nlm.nih.gov/clinvar/variation/198076/
NM_000152.3(GAA):c.317G>A	concordant	unreportable	unreportable	286481	0		1	SCV000339923	Uncertain significance	EGL Genetic Diagn	6/30/2017	GAA	chr17	78078702	78078702	http://gnomad.b	3.34E-05	EAS	0.0001166319998	0		0	0	https://www.ncbi.nlm.nih.gov/clinvar/variation/286481/
NM_000051.3(ATM):c.6604T>G	concordant	unreportable	unreportable	181882	0		4	SCV000682356;S	Uncertain significance;Unc	Color Genomics, In	2017-10-26;2017-1	ATM	chr11	108196068	108196068	http://gnomad.b	1.95E-05	AFR	6.54E-05	0		0	0	https://www.ncbi.nlm.nih.gov/clinvar/variation/181882/
NM_000035.3(ALDOB):c.488C>T	concordant	unreportable	unreportable	389159	1		2	SCV000707930;S	Uncertain significance;Likel	EGL Genetic Diagn	2017-06-30;2017-1	ALDOB	chr9	104189816	104189816	http://gnomad.b	5.36E-05	FIN	0.0004934950266	0		0	0	https://www.ncbi.nlm.nih.gov/clinvar/variation/389159/
NM_005476.5(GNE):c.23G>A	concordant	unreportable	unreportable	286466	0		1	SCV000339907	Uncertain significance	EGL Genetic Diagn	6/30/2017	GNE	chr9	36249330	36249330	http://gnomad.b	3.08E-05	NFE	7.17E-05	0		0	0	https://www.ncbi.nlm.nih.gov/clinvar/variation/286466/
NM_000370.3(TTPA):c.814A>C	concordant	unreportable	unreportable	289110	0		1	SCV000343404	Uncertain significance	EGL Genetic Diagn	6/30/2017	TTPA	chr8	63973834	63973834	http://gnomad.b	0.0001471197555	SAS	0.0003898889991	0		1	0	https://www.ncbi.nlm.nih.gov/clinvar/variation/289110/
NM_002485.4(NBN):c.1454C>T	concordant	unreportable	unreportable	232337	0		1	SCV000553101	Uncertain significance	Invitae	3/14/2017	NBN	chr8	90965863	90965863	http://gnomad.b	2.79E-05	AMR	0.000208893005	0		0	0	https://www.ncbi.nlm.nih.gov/clinvar/variation/232337/
NM_002485.4(NBN):c.254A>G	concordant	unreportable	unreportable	127868	0		1	SCV000287467	Uncertain significance	Invitae	10/5/2017	NBN	chr8	90993669	90993669	http://gnomad.b	7.53E-05	SAS	0.0007797270082	0		0	0	https://www.ncbi.nlm.nih.gov/clinvar/variation/127868/
NM_001360.2(DHCR7):c.241C>T	concordant	unreportable	unreportable	305959	0		1	SCV000373925	Uncertain significance	Illumina Clinical Se	10/18/2016	DHCR7	chr11	71155119	71155119	http://gnomad.b	4.85E-05	AMR	0.0003286130086	0		0	0	https://www.ncbi.nlm.nih.gov/clinvar/variation/305959/
NM_000136.2(FANCC):c.1604G>A	concordant	unreportable	unreportable	127535	0		2	SCV000149254;S	Likely benign;Uncertain sig	GeneDx;Invitae	2017-11-28;2017-1	FANCC	chr9	97864062	97864062	http://gnomad.b	2.38E-05	NFE	3.58E-05	0		0	0	https://www.ncbi.nlm.nih.gov/clinvar/variation/127535/
NM_014363.4(SACS):c.110G>A	concordant	unreportable	unreportable	450612	0		1	SCV000619215	Uncertain significance	GeneDx	11/28/2017	SACS	chr13	23949319	23949319		2.37E-05					0	0	https://www.ncbi.nlm.nih.gov/clinvar/variation/450612/
NM_001042432.1(CLN3):c.1012C>T	concordant	unreportable	unreportable	421563	0		1	SCV000570814	Uncertain significance	GeneDx	11/28/2017	CLN3	chr16	28493470	28493470	http://gnomad.b	2.78E-05	EAS	0.0003494060074	0		0	0	https://www.ncbi.nlm.nih.gov/clinvar/variation/421563/
NM_000051.3(ATM):c.8450A>G	concordant	unreportable	unreportable	220111	0		1	SCV000260404	Uncertain significance	Invitae	10/5/2017	ATM	chr11	108216501	108216501	http://gnomad.b	2.23E-05	EAS	0.0005221019965	0		0	0	https://www.ncbi.nlm.nih.gov/clinvar/variation/220111/
NM_000051.3(ATM):c.6443A>G	concordant	unreportable	unreportable	231615	0		2	SCV000332198;S	Uncertain significance;Unc	EGL Genetic Diagn	2017-06-30;2017-1	ATM	chr11	108190776	108190776	http://gnomad.b	1.39E-05	AFR	0.0002617120044	0		0	0	https://www.ncbi.nlm.nih.gov/clinvar/variation/231615/
NM_001042432.1(CLN3):c.963-16C>A	concordant	unreportable	unreportable	381985	0		1	SCV000521729	Likely benign	GeneDx	11/28/2017	CLN3	chr16	28493535	28493535	http://gnomad.b	3.89E-05	AMR	0.0005591100198	0		0	0	https://www.ncbi.nlm.nih.gov/clinvar/variation/381985/
NM_004646.3(NPHS1):c.3024A>G	concordant	unreportable	unreportable	429811	0		1	SCV000582470	Uncertain significance	GeneDx	11/28/2017	NPHS1	chr19	36330224	36330224		2.79E-06					0	0	https://www.ncbi.nlm.nih.gov/clinvar/variation/429811/
NM_001173990.2(TMEM216):c.123A>	concordant	unreportable	unreportable	498870	0		1	SCV000704097	Uncertain significance	EGL Genetic Diagn	6/30/2017	TMEM216	chr11	61160791	61160791	http://gnomad.b	5.91E-05	AMR	0.0004210170009	0		0	0	https://www.ncbi.nlm.nih.gov/clinvar/variation/498870/
NM_024649.4(BBS1):c.858C>T	concordant	unreportable	unreportable	501683	0		1	SCV000708155	Uncertain significance	EGL Genetic Diagn	6/30/2017	BBS1	chr11	66290954	66290954	http://gnomad.b	3.90E-05	AFR	0.0002614039986	0		0	0	https://www.ncbi.nlm.nih.gov/clinvar/variation/501683/
NM_002485.4(NBN):c.940G>A	concordant	unreportable	unreportable	141727	0		2	SCV000537534;S	Uncertain significance;Unc	Color Genomics, In	2017-12-21;2017-1	NBN	chr8	90976692	90976692	http://gnomad.b	3.07E-05	NFE	6.28E-05	0		0	0	https://www.ncbi.nlm.nih.gov/clinvar/variation/141727/
NM_000051.3(ATM):c.4674G>A	concordant	unreportable	unreportable	230265	0		3	SCV000537446;S	Likely benign;Likely benign	Color Genomics, In	2017-01-26;2017-1	ATM	chr11	108164102	108164102	http://gnomad.b	3.34E-05	AFR	6.55E-05	0		0	0	https://www.ncbi.nlm.nih.gov/clinvar/variation/230265/
NM_000051.3(ATM):c.6424A>G	concordant	unreportable	unreportable	478935	0		1	SCV000682335	Uncertain significance	Color Genomics, In	10/26/2017	ATM	chr11	108190757	108190757	http://gnomad.b	1.39E-05	NFE	9.10E-06	0		0	0	https://www.ncbi.nlm.nih.gov/clinvar/variation/478935/
NM_002485.4(NBN):c.1301C>T	concordant	unreportable	unreportable	185019	0		2	SCV000685708;S	Uncertain significance;Unc	Color Genomics, In	2017-10-26;2017-0	NBN	chr8	90967607	90967607	http://gnomad.b	5.30E-05	SAS	6.50E-05	0		0	0	https://www.ncbi.nlm.nih.gov/clinvar/variation/185019/
NM_001079802.1(FKTN):c.820C>T	concordant	unreportable	unreportable	290479	0		2	SCV000345042;S	Uncertain significance;Unc	EGL Genetic Diagn	2017-06-30;2017-1	FKTN	chr9	108377598	108377598	http://gnomad.b	6.03E-05	SAS	0.0003898380091	0		0	0	https://www.ncbi.nlm.nih.gov/clinvar/variation/290479/
NM_000051.3(ATM):c.6477T>G	concordant	unreportable	unreportable	141490	0		1	SCV000546956	Uncertain significance	Invitae	10/5/2017	ATM	chr11	108192052	108192052		5.57E-06					0	0	https://www.ncbi.nlm.nih.gov/clinvar/variation/141490/
NM_000391.3(TPP1):c.554G>A	concordant	unreportable	unreportable	457945	0		1	SCV000628912	Uncertain significance	Invitae	10/5/2017	TPP1	chr11	6638339	6638339	http://gnomad.b	2.24E-05	SAS	0.0001299629948	0		0	0	https://www.ncbi.nlm.nih.gov/clinvar/variation/457945/
NM_001079802.1(FKTN):c.7A>G	concordant	unreportable	unreportable	407111	0		1	SCV000546102	Uncertain significance	Invitae	3/14/2017	FKTN	chr9	108337320	108337320		3.55E-06					0	0	https://www.ncbi.nlm.nih.gov/clinvar/variation/407111/
NM_000152.3(GAA):c.1092G>A	concordant	unreportable	unreportable	388892	0		1	SCV000531286	Likely benign	GeneDx	11/28/2017	GAA	chr17	78082304	78082304	http://gnomad.b	2.78E-06	AMR	2.98E-05	0		0	0	https://www.ncbi.nlm.nih.gov/clinvar/variation/388892/
NM_000232.4(SGCB):c.498C>T	concordant	unreportable	unreportable	287905	0		1	SCV000341849	Uncertain significance	EGL Genetic Diagn	6/30/2017	SGCB	chr4	52895019	52895019	http://gnomad.b	4.48E-05	EAS	0.0001159550011	0		0	0	https://www.ncbi.nlm.nih.gov/clinvar/variation/287905/
NM_138694.3(PKHD1):c.8494G>T	concordant	unreportable	unreportable	357424	0		1	SCV000464051	Uncertain significance	Illumina Clinical Se	10/18/2016	PKHD1	chr6	51640666	51640666	http://gnomad.b	5.84E-05	NFE	8.11E-05	0		0	0	https://www.ncbi.nlm.nih.gov/clinvar/variation/357424/
NM_000391.3(TPP1):c.638C>T	concordant	unreportable	unreportable	207578	0		1	SCV000243418	Uncertain significance	GeneDx	11/28/2017	TPP1	chr11	6638255	6638255	http://gnomad.b	0.0001986558553	SAS	0.001721899956	1		0	0	https://www.ncbi.nlm.nih.gov/clinvar/variation/207578/
NM_000243.2(MEFV):c.1171G>A	concordant	unreportable	unreportable	450568	0		1	SCV000619168	Uncertain significance	GeneDx	11/28/2017	MEFV	chr16	3299520	3299520	http://gnomad.b	3.36E-05	AFR	0.0003269260051	0		0	0	https://www.ncbi.nlm.nih.gov/clinvar/variation/450568/
NM_000017.2(ACADS):c.336C>T	concordant	unreportable	unreportable	307435	0		1	SCV000376675	Uncertain significance	Illumina Clinical Se	10/18/2016	ACADS	chr12	121174914	121174914		1.13E-05					0	0	https://www.ncbi.nlm.nih.gov/clinvar/variation/307435/
NM_000057.2(BLM):c.1097T>C	concordant	unreportable	unreportable	127472	0		1	SCV000543398	Uncertain significance	Invitae	10/5/2017	BLM	chr15	91303386	91303386	http://gnomad.b	0.0001355658089	AMR	0.0003574620059	0		1	0	https://www.ncbi.nlm.nih.gov/clinvar/variation/127472/
NM_000271.4(NPC1):c.2747A>G	concordant	unreportable	unreportable	374343	0		1	SCV000705541	Uncertain significance	EGL Genetic Diagn	6/30/2017	NPC1	chr18	21119823	21119823	http://gnomad.b	8.58E-05	AMR	0.0002382230014	0		0	0	https://www.ncbi.nlm.nih.gov/clinvar/variation/374343/
NM_006493.2(CLN5):c.65G>T	concordant	unreportable	unreportable	409276	0		1	SCV000549223	Uncertain significance	Invitae	3/14/2017	CLN5	chr13	77566151	77566151	http://gnomad.b	3.08E-05	FIN	0.0003141030029	0		0	0	https://www.ncbi.nlm.nih.gov/clinvar/variation/409276/
NM_000051.3(ATM):c.5953A>G	concordant	unreportable	unreportable	246420	0		1	SCV000293967	Uncertain significance	GeneDx	11/28/2017	ATM	chr11	108183172	108183172	http://gnomad.b	2.78E-06	EAS	5.80E-05	0		0	0	https://www.ncbi.nlm.nih.gov/clinvar/variation/246420/
NM_000051.3(ATM):c.2720G>T	concordant	unreportable	unreportable	185390	0		2	SCV000682072;S	Uncertain significance;Unc	Color Genomics, In	2017-10-26;2017-1	ATM	chr11	108139218	108139218	http://gnomad.b	8.35E-06	EAS	0.0003478660074	0		0	0	https://www.ncbi.nlm.nih.gov/clinvar/variation/185390/
NM_000051.3(ATM):c.6504G>A	concordant	unreportable	unreportable	187165	0		2	SCV000682344;S	Uncertain significance;Likely benign	Color Genomics, In	2017-10-26;2017-1	ATM	chr11	108192079	108192079		2.78E-06					0	0	https://www.ncbi.nlm.nih.gov/clinvar/variation/187165/
NM_000391.3(TPP1):c.7C>A	concordant	unreportable	unreportable	207558	0		3	SCV000343531;S	Uncertain significance;Unc	EGL Genetic Diagn	2017-06-30;2016-0	TPP1	chr11	6640625	6640625	http://gnomad.b	7.55E-05	AFR	0.0006534240092	0		0	0	https://www.ncbi.nlm.nih.gov/clinvar/variation/207558/
NM_000232.4(SGCB):c.249A>G	concordant	unreportable	unreportable	284933	0		1	SCV000337751	Uncertain significance	EGL Genetic Diagn	6/30/2017	SGCB	chr4	52896024	52896024	http://gnomad.b	2.52E-05	SAS	0.0005522710271	0		0	0	https://www.ncbi.nlm.nih.gov/clinvar/variation/284933/
NM_000051.3(ATM):c.210A>T	concordant	unreportable	unreportable	418022	0		1	SCV000622307	Uncertain significance	Invitae	10/5/2017	ATM	chr11	108099929	108099929		8.35E-06					0	0	https://www.ncbi.nlm.nih.gov/clinvar/variation/418022/

Supplemental Table 2

NM_000071.2(CBS):c.612G>C	concordant	unreportable	unreportable	264320	0	2	SCV000320179;S	Likely benign;Likely benign	Ambry Genetics;In	2017-11-14;2017-1	CBS	chr21	44485551	44485551	http://gnomad.b	2.53E-05	NFE	5.43E-05	0		0	0	https://www.ncbi.nlm.nih.gov/clinvar/variation/264320/
NM_000051.3(ATM):c.8427A>G	concordant	unreportable	unreportable	219709	0	2	SCV000682487;S	Likely benign;Likely benign	Color Genomics, In	2017-10-26;2017-1	ATM	chr11	108216478	108216478	http://gnomad.b	6.40E-05	EAS	0.0009316410287	0		0	0	https://www.ncbi.nlm.nih.gov/clinvar/variation/219709/
NM_002485.4(NBN):c.120G>T	concordant	unreportable	unreportable	184454	0	2	SCV000685702;S	Likely benign;Likely benign	Color Genomics, In	2017-10-26;2017-1	NBN	chr8	90995001	90995001	http://gnomad.b	1.67E-05	AMR	8.93E-05	0		0	0	https://www.ncbi.nlm.nih.gov/clinvar/variation/184454/
NM_000642.2(AGL):c.1911G>A	concordant	unreportable	unreportable	456461	0	1	SCV000626684	Likely benign	Invitae	10/5/2017	AGL	chr1	100346643	100346643	http://gnomad.b	1.40E-05	SAS	6.50E-05	0		0	0	https://www.ncbi.nlm.nih.gov/clinvar/variation/456461/
NM_004328.4(BCS1L):c.258T>C	concordant	unreportable	unreportable	334357	0	1	SCV000427433	Uncertain significance	Illumina Clinical Se	10/18/2016	BCS1L	chr2	219525968	219525968		3.07E-05					0	0	https://www.ncbi.nlm.nih.gov/clinvar/variation/334357/
NM_000051.3(ATM):c.-218T>A	concordant	unreportable	unreportable	302238	0	1	SCV000367014	Uncertain significance	Illumina Clinical Se	10/18/2016	ATM	chr11	108093726	108093726		8.35E-06					0	0	https://www.ncbi.nlm.nih.gov/clinvar/variation/302238/
NM_000136.2(FANCC):c.1242G>A	concordant	unreportable	unreportable	137281	0	1	SCV000260469	Likely benign	Invitae	3/14/2017	FANCC	chr9	97873832	97873832	http://gnomad.b	7.13E-05	ASJ	0.0003045689955	0		0	0	https://www.ncbi.nlm.nih.gov/clinvar/variation/137281/
NM_000051.3(ATM):c.2269G>A	concordant	unreportable	unreportable	127345	0	3	SCV000682045;S	Uncertain significance;Unc	Color Genomics, In	2017-10-26;2017-1	ATM	chr11	108128226	108128226	http://gnomad.b	1.11E-05	NFE	2.69E-05	0		0	0	https://www.ncbi.nlm.nih.gov/clinvar/variation/127345/
NM_000051.3(ATM):c.4149G>A	concordant	unreportable	unreportable	183939	0	2	SCV000515428;S	Likely benign;Likely benign	GeneDx;Invitae	2017-11-28;2017-1	ATM	chr11	108159743	108159743		1.95E-05					0	0	https://www.ncbi.nlm.nih.gov/clinvar/variation/183939/
NM_001079802.1(FKTN):c.1021C>T	concordant	unreportable	unreportable	290218	0	1	SCV000344728	Uncertain significance	EGL Genetic Diagn	6/30/2017	FKTN	chr9	108380350	108380350		3.55E-05					0	0	https://www.ncbi.nlm.nih.gov/clinvar/variation/290218/
NM_002485.4(NBN):c.897-2A>T	concordant	reportable	reportable	219440	2	0	SCV000259306;S	Likely pathogenic;Likely pa	Invitae;Laboratory	2016-01-06;2018-0	NBN	chr8	90976737	90976737		8.37E-06					0	0	https://www.ncbi.nlm.nih.gov/clinvar/variation/219440/
NM_000023.2(SGCA):c.1015G>A	concordant	unreportable	unreportable	282864	0	1	SCV000334565	Uncertain significance	EGL Genetic Diagn	6/30/2017	SGCA	chr17	48252649	48252649	http://gnomad.b	0.0001091446418	EAS	0.0005352050066	0		0	0	https://www.ncbi.nlm.nih.gov/clinvar/variation/282864/
NM_033056.3(PCDH15):c.4719G>A	concordant	unreportable	unreportable	284546	0	1	SCV000337208	Uncertain significance	EGL Genetic Diagn	6/30/2017	PCDH15	chr10	55582767	55582767	http://gnomad.b	0.0002237309057	SAS	0.002956470009	1		1	0	https://www.ncbi.nlm.nih.gov/clinvar/variation/284546/
NM_000360.3(TH):c.1470C>G	concordant	unreportable	unreportable	304065	0	1	SCV000369906	Uncertain significance	Illumina Clinical Se	10/18/2016	TH	chr11	2185487	2185487	http://gnomad.b	1.97E-05	NFE	5.07E-05	0		0	0	https://www.ncbi.nlm.nih.gov/clinvar/variation/304065/
NM_024649.4(BBS1):c.744C>T	concordant	unreportable	unreportable	434480	0	1	SCV000593582	Uncertain significance	Genetic Services La	7/5/2017	BBS1	chr11	66288761	66288761	http://gnomad.b	7.25E-05	AMR	0.0002977790136	0		0	0	https://www.ncbi.nlm.nih.gov/clinvar/variation/434480/
NM_000152.3(GAA):c.265C>T	concordant	unreportable	unreportable	456412	0	1	SCV000626590	Uncertain significance	Invitae	10/5/2017	GAA	chr17	78078650	78078650	http://gnomad.b	0.0001222894814	SAS	0.001919819973	0		0	0	https://www.ncbi.nlm.nih.gov/clinvar/variation/456412/
NM_017739.3(POMGNT1):c.1284+9G>	concordant	unreportable	unreportable	290485	0	3	SCV000345049;S	Uncertain significance;Unc	EGL Genetic Diagn	2017-06-30;2017-0	POMGNT1	chr1	46658181	46658181	http://gnomad.b	6.16E-05	AFR	0.000326754991	0		0	0	https://www.ncbi.nlm.nih.gov/clinvar/variation/290485/
NM_017890.4(VPS13B):c.11162G>A	concordant	unreportable	unreportable	361095	0	2	SCV000616265;S	Uncertain significance;Unc	Athena Diagnostics	2017-08-17;2016-1	VPS13B	chr8	100874046	100874046		2.24E-05					0	0	https://www.ncbi.nlm.nih.gov/clinvar/variation/361095/
NM_000136.2(FANCC):c.1544C>G	concordant	unreportable	unreportable	367609	0	2	SCV000618177;S	Uncertain significance;Unc	GeneDx;Illumina C	2017-11-28;2016-1	FANCC	chr9	97864122	97864122	http://gnomad.b	3.57E-05	FIN	8.97E-05	0		0	0	https://www.ncbi.nlm.nih.gov/clinvar/variation/367609/
NM_002485.4(NBN):c.1667T>A	concordant	unreportable	unreportable	127859	0	3	SCV000685730;S	Uncertain significance;Unc	Color Genomics, In	2017-10-26;2017-0	NBN	chr8	90965650	90965650	http://gnomad.b	5.58E-05	NFE	4.49E-05	0		0	0	https://www.ncbi.nlm.nih.gov/clinvar/variation/127859/
NM_000492.3(CFTR):c.954G>A	concordant	unreportable	unreportable	455785	0	1	SCV000625758	Likely benign	Invitae	10/5/2017	CFTR	chr7	117180238	117180238	http://gnomad.b	1.93E-05	SAS	0.000259908993	0		0	0	https://www.ncbi.nlm.nih.gov/clinvar/variation/455785/
NM_002485.4(NBN):c.2196A>G	concordant	unreportable	unreportable	136041	0	3	SCV000685759;S	Likely benign;Likely benign	Color Genomics, In	2017-10-26;2017-1	NBN	chr8	90949292	90949292	http://gnomad.b	0.0001477969883	EAS	0.0002321530046	0		0	0	https://www.ncbi.nlm.nih.gov/clinvar/variation/136041/
NM_000051.3(ATM):c.7736G>C	concordant	unreportable	unreportable	246325	0	1	SCV000293832	Uncertain significance	GeneDx	11/28/2017	ATM	chr11	108202712	108202712		2.78E-06					0	0	https://www.ncbi.nlm.nih.gov/clinvar/variation/246325/
NM_002435.1(MPI):c.378G>A	concordant	unreportable	unreportable	317138	0	1	SCV000393942	Uncertain significance	Illumina Clinical Se	10/18/2016	MPI	chr15	75185034	75185034	http://gnomad.b	6.16E-05	EAS	0.0001739330037	0		0	0	https://www.ncbi.nlm.nih.gov/clinvar/variation/317138/
NM_000051.3(ATM):c.507T>C	concordant	unreportable	unreportable	184009	0	2	SCV000593475;S	Likely benign;Likely benign	Genetic Services La	2017-07-05;2017-1	ATM	chr11	108114690	108114690	http://gnomad.b	2.51E-05	NFE	4.42E-05	0		0	0	https://www.ncbi.nlm.nih.gov/clinvar/variation/184009/
NM_000051.3(ATM):c.3102T>C	concordant	unreportable	unreportable	231127	0	2	SCV000519984;S	Likely benign;Likely benign	GeneDx;Invitae	2017-11-28;2017-0	ATM	chr11	108143283	108143283	http://gnomad.b	1.11E-05	AFR	6.54E-05	0		0	0	https://www.ncbi.nlm.nih.gov/clinvar/variation/231127/
NM_000520.4(HEXA):c.581C>T	concordant	unreportable	unreportable	458318	0	1	SCV000629535	Uncertain significance	Invitae	10/5/2017	HEXA	chr15	72643565	72643565	http://gnomad.b	2.82E-05	AFR	0.0004573969927	0		0	0	https://www.ncbi.nlm.nih.gov/clinvar/variation/458318/
NM_000051.3(ATM):c.1953A>G	concordant	unreportable	unreportable	181849	0	3	SCV000682016;S	Likely benign;Likely benign	Color Genomics, In	2017-10-26;2017-1	ATM	chr11	108124595	108124595	http://gnomad.b	9.19E-05	AMR	0.0004169399908	0		0	0	https://www.ncbi.nlm.nih.gov/clinvar/variation/181849/
NM_017739.3(POMGNT1):c.16C>T	concordant	unreportable	unreportable	500451	0	1	SCV000706409	Uncertain significance	EGL Genetic Diagn	6/30/2017	POMGNT1	chr1	46663478	46663478		4.20E-05					0	0	https://www.ncbi.nlm.nih.gov/clinvar/variation/500451/
NM_002485.4(NBN):c.16C>T	concordant	unreportable	unreportable	182731	0	2	SCV000211463;S	Uncertain significance;Unc	GeneDx;Invitae	2017-11-28;2017-1	NBN	chr8	90996774	90996774	http://gnomad.b	1.95E-05	AFR	0.0001373999985	0		0	0	https://www.ncbi.nlm.nih.gov/clinvar/variation/182731/
NM_000277.1(PAH):c.592_613del22	concordant	reportable	reportable	102746	1	0	SCV000696457	Pathogenic	Laboratory Corpora	1/25/2018	PAH	chr12	103249006	103249028	http://gnomad.b	1.37E-05	AMR	0.0001191679985	0		0	0	https://www.ncbi.nlm.nih.gov/clinvar/variation/102746/
NM_000051.3(ATM):c.5583G>A	concordant	unreportable	unreportable	232961	0	2	SCV000682274;S	Likely benign;Likely benign	Color Genomics, In	2017-10-26;2017-1	ATM	chr11	108175488	108175488	http://gnomad.b	2.78E-05	NFE	4.49E-05	0		0	0	https://www.ncbi.nlm.nih.gov/clinvar/variation/232961/
NM_133647.1(SLC12A6):c.1707A>G	concordant	unreportable	unreportable	383689	0	1	SCV000524152	Likely benign	GeneDx	11/28/2017	SLC12A6	chr15	34538011	34538011	http://gnomad.b	5.93E-05	EAS	0.00144960999	0		0	0	https://www.ncbi.nlm.nih.gov/clinvar/variation/383689/
NM_017739.3(POMGNT1):c.1305G>A	concordant	unreportable	unreportable	471401	0	1	SCV000649956	Likely benign	Invitae	10/5/2017	POMGNT1	chr1	46658088	46658088	http://gnomad.b	1.96E-05	AMR	2.98E-05	0		0	0	https://www.ncbi.nlm.nih.gov/clinvar/variation/471401/
NM_033056.3(PCDH15):c.5318C>G	concordant	unreportable	unreportable	300171	0	1	SCV000363116	Uncertain significance	Illumina Clinical Se	10/18/2016	PCDH15	chr10	55582168	55582168	http://gnomad.b	0.0001234377411	NFE	0.0001474059973	0		0	0	https://www.ncbi.nlm.nih.gov/clinvar/variation/300171/
NM_000492.3(CFTR):c.697C>G	concordant	unreportable	unreportable	389270	0	1	SCV000531739	Uncertain significance	GeneDx	11/28/2017	CFTR	chr7	117175419	117175419	http://gnomad.b	2.89E-05	SAS	0.0001949190046	0		0	0	https://www.ncbi.nlm.nih.gov/clinvar/variation/389270/
NM_000642.2(AGL):c.2966A>G	concordant	unreportable	unreportable	291339	0	1	SCV000346079	Uncertain significance	Illumina Clinical Se	10/18/2016	AGL	chr1	100357178	100357178	http://gnomad.b	4.47E-05	SAS	0.0003249070141	0		0	0	https://www.ncbi.nlm.nih.gov/clinvar/variation/291339/
NM_000271.4(NPC1):c.3559G>A	concordant	unreportable	unreportable	499348	0	1	SCV000704787	Uncertain significance	EGL Genetic Diagn	6/30/2017	NPC1	chr18	21114442	21114442	http://gnomad.b	5.72E-05	AMR	0.0001786990033	0		0	0	https://www.ncbi.nlm.nih.gov/clinvar/variation/499348/
NM_004646.3(NPHS1):c.427G>C	concordant	unreportable	unreportable	328880	0	1	SCV000411587	Uncertain significance	Illumina Clinical Se	10/18/2016	NPHS1	chr19	36341962	36341962	http://gnomad.b	9.20E-05	NFE	0.0001432610006	0		0	0	https://www.ncbi.nlm.nih.gov/clinvar/variation/328880/
NM_000018.3(ACADVL):c.538G>A	concordant	unreportable	unreportable	166641	1	1	SCV000602371;S	Likely pathogenic;Uncertai	ARUP Laboratories	2017-06-30;2017-0	ACADVL	chr17	7124917	7124917	http://gnomad.b	1.42E-05	AMR	2.98E-05	0		0	0	https://www.ncbi.nlm.nih.gov/clinvar/variation/166641/
NM_000051.3(ATM):c.5645G>A	concordant	unreportable	unreportable	142104	0	1	SCV000622610	Uncertain significance	Invitae	10/5/2017	ATM	chr11	108175550	108175550	http://gnomad.b	5.57E-06	AFR	6.55E-05	0		0	0	https://www.ncbi.nlm.nih.gov/clinvar/variation/142104/
NM_006493.2(CLN5):c.834C>T	concordant	unreportable	unreportable	377692	0	1	SCV000512642	Likely benign	GeneDx	11/28/2017	CLN5	chr13	77574714	77574714	http://gnomad.b	5.04E-05	AFR	0.0001307360071	0		0	0	https://www.ncbi.nlm.nih.gov/clinvar/variation/377692/
NM_002485.4(NBN):c.683T>G	concordant	unreportable	unreportable	186350	0	4	SCV000685815;S	Uncertain significance;Unc	Color Genomics, In	2017-10-26;2017-0	NBN	chr8	90983420	90983420	http://gnomad.b	4.74E-05	FIN	0.0001348439982	0		0	0	https://www.ncbi.nlm.nih.gov/clinvar/variation/186350/
NM_000492.3(CFTR):c.4142A>G	concordant	unreportable	unreportable	411115	0	1	SCV000552128	Uncertain significance	Invitae	3/14/2017	CFTR	chr7	117305518	117305518	http://gnomad.b	1.54E-05	NFE	9.07E-06	0		0	0	https://www.ncbi.nlm.nih.gov/clinvar/variation/411115/
NM_000492.3(CFTR):c.1210-6delT	concordant	unreportable	unreportable	495892	0	1	SCV000696829	Uncertain significance	Laboratory Corpora	1/25/2018	CFTR	chr7	117188682	117188683		1.93E-06					0	0	https://www.ncbi.nlm.nih.gov/clinvar/variation/495892/
NM_033056.3(PCDH15):c.4272A>G	concordant	unreportable	unreportable	300177	0	1	SCV000363147	Uncertain significance	Illumina Clinical Se	10/18/2016	PCDH15	chr10	55587248	55587248	http://gnomad.b	1.80E-05	AFR	0.000130786997	0		0	0	https://www.ncbi.nlm.nih.gov/clinvar/variation/300177/
NM_000152.3(GAA):c.1482A>G	concordant	unreportable	unreportable	286163	0	3	SCV000339488;S	Uncertain significance;Likel	EGL Genetic Diagn	2017-06-30;2017-1	GAA	chr17	78084570	78084570	http://gnomad.b	0.0001028343367	NFE	0.0001254209928	0		0	0	https://www.ncbi.nlm.nih.gov/clinvar/variation/286163/
NM_001360.2(DHCR7):c.375C>T	concordant	unreportable	unreportable	286416	0	1	SCV000339844	Uncertain significance	EGL Genetic Diagn	6/30/2017	DHCR7	chr11	71153346	71153346	http://gnomad.b	2.96E-05	AMR	0.0002682399936	0		0	0	https://www.ncbi.nlm.nih.gov/clinvar/variation/286416/
NM_000071.2(CBS):c.395G>A	concordant	unreportable	unreportable	405373	0	1	SCV000543505	Uncertain significance	Invitae	3/14/2017	CBS	chr21	44486409	44486409	http://gnomad.b	1.97E-05	NFE	1.79E-05	0		0	0	https://www.ncbi.nlm.nih.gov/clinvar/variation/405373/
NM_000051.3(ATM):c.3291C>G	concordant	unreportable	unreportable	230299	0	2	SCV000570191;S	Uncertain significance;Unc	GeneDx;Invitae	2017-11-28;2017-1	ATM	chr11	108150224	108150224		2.78E-06					0	0	https://www.ncbi.nlm.nih.gov/clinvar/variation/230299/
NM_000051.3(ATM):c.7542T>C	concordant	unreportable	unreportable	187253	0	2	SCV000526560;S	Likely benign;Likely benign	GeneDx;Invitae	2017-11-28;2017-1	ATM	chr11	108202197	108202197	http://gnomad.b	1.39E-05	EAS	0.0001162249973	0		0	0	https://www.ncbi.nlm.nih.gov/clinvar/variation/187253/
NM_017890.4(VPS13B):c.10643A>G	concordant	unreportable	unreportable	95823	0	1	SCV000470855	Uncertain significance	Illumina Clinical Se	10/18/2016	VPS13B	chr8	100866185	100866185	http://gnomad.b	0.0001875752401	NFE	0.0003312090121	0		0	0	https://www.ncbi.nlm.nih.gov/clinvar/variation/95823/
NM_000057.2(BLM):c.542G>T	concordant	unreportable	unreportable	127512	0	1	SCV000543388	Uncertain significance	Invitae	10/5/2017	BLM	chr15	91293040	91293040	http://gnomad.b	4.28E-05	NFE	8.26E-05	0		0	0	https://www.ncbi.nlm.nih.gov/clinvar/variation/127512/
NM_000152.3(GAA):c.2509C>T	concordant	unreportable	unreportable	456408	0	1	SCV000626579	Uncertain significance	Invitae	10/5/2017	GAA	chr17	78092019	78092019	http://gnomad.b	2.78E-05	NFE	4.50E-05	0		0	0	https://www.ncbi.nlm.nih.gov/clinvar/variation/456408/
NM_000052.5(ATP7A):c.610+8G>A	concordant	unreportable	unreportable	92385	0	1	SCV000640000	Benign	Invitae	10/5/2017	ATP7A	chrX	77244235	77244235	http://gnomad.b	0.001239745465	AFR	0.01765500009	3	57	1	16	https://www.ncbi.nlm.nih.gov/clinvar/variation/92385/
NM_000070.2(CAPN3):c.2332G>A	concordant	unreportable	unreportable	92413	0	3	SCV000523001;S	Benign;Likely benign;Benig	GeneDx;Genetic Se	2017-11-28;2014-0	CAPN3	chr15	42703150	42703150	http://gnomad.b	0.002336336432	AFR	0.03156450018	9		6	0	https://www.ncbi.nlm.nih.gov/clinvar/variation/92413/
NM_000070.2(CAPN3):c.318C>T	concordant	unreportable	unreportable	92416	0	2	SCV000525495;S	Likely benign;Benign	GeneDx;Invitae	2017-11-28;2017-1	CAPN3	chr15	42676689	42676689	http://gnomad.b	0.005304763886	NFE	0.007298550103	4		2	0	https://www.ncbi.nlm.nih.gov/clinvar/variation/92416/
NM_000070.2(CAPN3):c.479C>G	concordant	unreportable	unreportable	92417	0	4	SCV000520877;S	Benign;Likely benign;Likely	GeneDx;Genetic Se	2017-11-28;2014-0	CAPN3	chr15	42678464	42678464	http://gnomad.b	0.003800547279	AFR	0.05156860128	16		10	0	https://www.ncbi.nlm.nih.gov/clinvar/variation/92417/
NM_000070.2(CAPN3):c.62G>A	concordant	unreportable	unreportable	92419	0	4	SCV000523830;S	Benign;Likely benign;Uncer	GeneDx;Genetic Se	2017-11-28;2014-0	CAPN3	chr15	42652065	42652065	http://gnomad.b	0.003888559953	AFR	0.04009160027	10		12	0	https://www.ncbi.nlm.nih.gov/clinvar/variation/92419/
NM_000202.5(IDS):c.641C>T	concordant	unreportable	unreportable	92622	0	1	SCV000695964	Benign	Laboratory Corpora	1/25/2018	IDS	chrX	148579705	148579705	http://gnomad.b	0.004397222195	AFR	0.05007350072	14	175	8	74	https://www.ncbi.nlm.nih.gov/clinvar/variation/92622/
NM_000263.3(NAGLU):c.2209C>A	concordant	unreportable	unreportable	92692	0	1	SCV000402918	Uncertain significance	Illumina Clinical Se	10/18/2016	NAGLU	chr17	40696233	40696233	http://gnomad.b	0.01571728101	SAS	0.0358420983	66		20	0	https://www.ncbi.nlm.nih.gov/clinvar/variation/92692/
NM_000286.2(PEX12):c.102A>T	concordant	unreportable	unreportable	92773	0	1	SCV000540016	Likely benign	Laboratory for Mol	4/3/2017	PEX12	chr17	33904939	33904939	http://gnomad.b	0.005377548734	FIN	0.01331960037	14		1	0	https://www.ncbi.nlm.nih.gov/clinvar/variation/92773/
NM_000286.2(PEX12):c.451C>T	concordant	unreportable	unreportable	92774	0	1	SCV000296903	Likely benign	Division of Genomi	1/6/2016	PEX12	chr17	33904286	33904286	http://gnomad.b	0.002008578471	NFE	0.003277159994	0		1	0	https://www.ncbi.nlm.nih.gov/clinvar/variation/92774/
NM_000287.3(PEX6):c.2816C>A	concordant	unreportable	unreportable	92787	0	3	SCV000110430;S	Benign;Benign;Benign	EGL Genetic Diagn	2017-06-30;2016-1	PEX6	chr6	42932200	42932200	http://gnomad.b	5.60E-05	NFE	0.4483070076	19261		0	0	https://www.ncbi.nlm.nih.gov/clinvar/variation/92787/
NM_000404.2(GLB1):c.1306C>T	concordant	unreportable	unreportable	92895	0	2	SCV000603839;S	Benign;Benign	ARUP Laboratories	2017-06-30;2017-0	GLB1	chr3	33059981	33059981	http://gnomad.b	0.01242718136	ASJ	0.03634519875	23		13	0	https://www.ncbi.nlm.nih.gov/clinvar/variation/92895/
NM_000709.3(BCKDHA):c.114C>G	concordant	unreportable	unreportable	93337	0	1	SCV000527564	Benign	GeneDx	11/28/2017	BCKDHA	chr19	41916547	41916547	http://gnomad.b	0.001479219731	AFR	0.01699570008	4		0	0	https://www.ncbi.nlm.nih.gov/clinvar/variation/93337/
NM_001918.3(DBT):c.724T>C	concordant	unreportable	unreportable	94011	0	1	SCV000627813	Benign	Invitae	10/5/2017	DBT	chr1	100681587	100681587	http://gnomad.b	0.008889527275	NFE	0.0138226999	16		5	0	https://www.ncbi.nlm.nih.gov/clinvar/variation/94011/
NM_001918.3(DBT):c.753C>T	concordant	unreportable	unreportable	94012	0	1	SCV000527582	Likely benign	GeneDx	11/28/2017	DBT	chr1	100681558	100681558	http://gnomad.b	0.001763541766	NFE	0.002310380107	1		0	0	https://www.ncbi.nlm.nih.gov/clinvar/variation/94012/
NM_003494.3(DYSF):c.2162+16C>T	concordant	unreportable	unreportable	94284	0	1	SCV000519668	Benign	GeneDx	11/28/2017	DYSF	chr2	71783217	71783217	http://gnomad.b	0.01364481449	ASJ	0.02864770032	20		16	0	https://www.ncbi.nlm.nih.gov/clinvar/variation/94284/
NM_003494.3(DYSF):c.383G>A	concordant	unreportable	unreportable	94311	0	4	SCV000331938;S	Benign;Benign;Benign;Beni	EGL Genetic Diagn	2016-11-03;2017-1	DYSF	chr2	71738977	71738977	http://gnomad.b	0.009635437717	ASJ	0.05962190032	20		16	0	https://www.ncbi.nlm.nih.gov/clinvar/variation/94311/
NM_003494.3(DYSF):c.457+17G>C	concordant	unreportable	unreportable	94326	0	2	SCV000112187;S	Benign;Benign	EGL Genetic Diagn	2017-06-30;2017-1	DYSF	chr2	71739068	71739068	http://gnomad.b	0.02711987713	FIN	0.08919899911	126		63	0	https://www.ncbi.nlm.nih.gov/clinvar/variation/94326/
NM_003494.3(DYSF):c.4731G>A	concordant	unreportable	unreportable	94328	0	4	SCV000519971;S	Benign;Likely benign;Uncer	GeneDx;Genetic Se	2017-11-28;2014-0	DYSF	chr2	71886100	71886100	http://gnomad.b	0.02118664619	ASJ	0.04020300135	54		34	0	https://www.ncbi.nlm.nih.gov/clinvar/variation/94328/
NM_013339.3(ALG6):c.1323T>C	concordant	unreportable	unreportable	95528	0	3	SCV000520328;S	Benign;Likely benign;Uncer	GeneDx;Genetic Se	2017-11-28;2014-0	ALG6	chr1	63894794	63894794	http://gnomad.b	0.008306525184	NFE	0.01545260008	12		5	0	https://www.ncbi.nlm.nih.gov/clinvar/variation/95528/
NM_024301.4(FKRP):c.1440C>T	concordant	unreportable	unreportable	96106	0	3	SCV000525535;S	Likely benign;Likely benign	GeneDx;Genetic Se	2017-11-28;2017-0	FKRP	chr19	47260147	47260147	http://gnomad.b	0.0004161198425	AFR	0.008000560105	1		0	0	https://www.ncbi.nlm.nih.gov/clinvar/variation/96106/
NM_024312.4(GNPTAB):c.3602+8C>A	concordant	unreportable	unreportable	96122	0	3	SCV000603853;S	Benign;Benign;Likely benig	ARUP Laboratories	2017-06-30;2017-0	GNPTAB	chr12	102147142	102147142	http://gnomad.b	0.03165174288	NFE	0.0506592989	207		90	0	https://www.ncbi.nlm.nih.gov/clinvar/variation/96122/
NM_031885.3(BBS2):c.1413A>C	concordant	unreportable	unreportable	96206	0	2	SCV000398058;S	Likely benign;Benign	Illumina Clinical Se	2016-10-18;2017-1	BBS2	chr16	56533804	56533804	http://gnomad.b	0.01244879672	FIN	0.03973089904	42		14	0	https://www.ncbi.nlm.nih.gov/clinvar/variation/96206/
NM_152618.2(BBS12):c.116T>C	concordant	unreportable	unreportable	96504	0	2	SCV000246780;S	Likely benign;Benign	Genetic Services La	2015-09-15;2017-1	BBS12	chr4	123663163	123663163	http://gnomad.b	0.007689599437	ASJ	0.01249490026	7		1	0	https://www.ncbi.nlm.nih.gov/clinvar/variation/96504/
NM_000426.3(LAMA2):c.1634T>A	concordant	unreportable	unreportable	92940	0	2	SCV000196841;S	Likely benign;Likely benign	GeneDx;Invitae	2017-11-28;2017-1	LAMA2	chr6	129513850	129513850	http://gnomad.b	0.004433134883	ASJ	0.01198210008	1		2	0	https://www.ncbi.nlm.nih.gov/clinvar/variation/92940/
NM_000426.3(LAMA2):c.1798G>A	concordant	unreportable	unreportable	92941	0	2	SCV000151644;S	Likely benign;Benign	Genetic Services La	2014-06-27;2017-1	LAMA2	chr6	129571272	129571272	http://gnomad.b	0.0154519557	ASJ	0.02813900076	29		19	0	https://www.ncbi.nlm.nih.gov/clinvar/variation/92941/
NM_000426.3(LAMA2):c.3556-15T>G	concordant	unreportable	unreportable	92953	0	2	SCV000524135;S	Benign;Uncertain significar	GeneDx;Illumina C	2017-11-28;2016-1	LAMA2	chr6	129636606	129636606	http://gnomad.b	0.005633442161	SAS	0.02628160082	23		7	0	https://www.ncbi.nlm.nih.gov/clinvar/variation/92953/
NM_000426.3(LAMA2):c.4437-5T>A	concordant	unreportable	unreportable	92958	0	3	SCV000613968;S	Likely benign;Benign;Benig	Athena Diagnostics	2017-08-17;2017-1	LAMA2	chr6	129670438	129670438	http://gnomad.b	0.007409896934	ASJ	0.01525009982	4		6	0	https://www.ncbi.nlm.nih.gov/clinvar/variation/92958/
NM_000426.3(LAMA2):c.4470C>T	concordant	unreportable	unreportable	92959	0	2	SCV000516592;S	Likely benign;Benign	GeneDx;Invitae	2017-11-28;2017-1	LAMA2	chr6	129670476	129670476	http://gnomad.b	0.00710581909	ASJ	0.01514230017	4		5	0	https://www.ncbi.nlm.nih.gov/clinvar/variation/92959/
NM_000426.3(LAMA2):c.4750G>A	concordant	unreportable	unreportable	92962	0	3	SCV000519478;S	Benign;Likely benign;Benig	GeneDx;Genetic Se	2017-11-28;2014-0	LAMA2	chr6	129687396	129687396	http://gnomad.b	0.02218167851	NFE	0.03009900078	71		36	0	https://www.ncbi.nlm.nih.gov/clinvar/variation/92962/
NM_000426.3(LAMA2):c.5530C>A	concordant	unreportable	unreportable	92968	0	3	SCV000521018;S	Likely benign;Likely benign	GeneDx;Genetic Se	2017-11-28;2014-0	LAMA2	chr6	129722453	129722453	http://gnomad.b	0.01089078804	NFE	0.01840149984	27		16	0	https://www.ncbi.nlm.nih.gov/clinvar/variation/92968/
NM_000426.3(LAMA2):c.5633C>T	concordant	unreportable	unreportable	92969	0	2	SCV000529949;S	Likely benign;Uncertain sig	GeneDx;Genetic Se	2017-11-28;2017-0	LAMA2	chr6	129723539	129723539	http://gnomad.b	0.0007922028039	AFR	0.008168869652	0		1	0	https://www.ncbi.nlm.nih.gov/clinvar/variation/92969/
NM_000426.3(LAMA2):c.6150T>C	concordant	unreportable	unreportable	92974	0	3	SCV000613975;S	Benign;Likely benign;Benig	Athena Diagnostics	2017-08-17;2017-1	LAMA2	chr6	129762025	129762025	http://gnomad.b	0.00113629089	AFR	0.01183009986	0		2	0	https://www.ncbi.nlm.nih.gov/clinvar/variation/92974/
NM_004006.2(DMD):c.-128771C>T	concordant	unreportable	unreportable	94402	0	1	SCV000112287	Benign	EGL Genetic Diagn	6/30/2017	DMD	chrX	33358200	33358200		0.009151493288					6	189	https://www.ncbi.nlm.nih.gov/clinvar/variation/94402/
NM_004006.2(DMD):c.10087-20C>T	concordant	unreportable	unreportable	94423	0	1	SCV000112308	Benign	EGL Genetic Diagn	6/30/2017	DMD	chrX	31196942	31196942	http://gnomad.b	0.00189865006	NFE	0.003656399902	0	126	0	40	https://www.ncbi.nlm.nih.gov/clinvar/variation/94423/
NM_004006.2(DMD):c.10789C>T	concordant	unreportable	unreportable	94446	0	5	SCV000603358;S	Benign;Benign;Uncertain si	ARUP Laboratories	2017-06-30;2017-1	DMD	chrX	31165400	31165400	http://gnomad.b	0.005259260666	FIN	0.009869020432	3	309	3	107	https://www.ncbi.nlm.nih.gov/clinvar/variation/94446/
NM_004006.2(DMD):c.1225A>T	concordant	unreportable	unreportable	94453	0	4	SCV000603338;S	Benign;Benign;Likely benig	ARUP Laboratories	2017-06-30;2017-0	DMD	chrX	32662355	32662355	http://gnomad.b	0.003275171353	AFR	0.03782369941	2	119	1	50	https://www.ncbi.nlm.nih.gov/clinvar/variation/94453/
NM_004006.2(DMD):c.1483-7C>G	concordant	unreportable	unreportable	94470	0	3	SCV000603361;S	Benign;Benign;Benign	ARUP Laboratories	2017-06-30;2017-1	DMD	chrX	32614000	32614000	http://gnomad.b	0.0005601017676	AFR	0.00671746023	0	31	0	11	https://www.ncbi.nlm.nih.gov/clinvar/variation/94470/
NM_004006.2(DMD):c.1513G>C	concordant	unreportable	unreportable	94471	0	2	SCV000235827;S	Likely benign;Likely benign	GeneDx;Invitae	2017-11-28;2017-1	DMD	chrX	32613963	32613963	http://gnomad.b	0.001186656287	FIN	0.002798390109	0	61	0	21	https://www.ncbi.nlm.nih.gov/clinvar/variation/94471/
NM_004006.2(DMD):c.1554T>A	concordant	unreportable	unreportable	94473	0	2	SCV000319874;S	Benign;Benign	Ambry Genetics;In	2017-11-14;2017-1	DMD	chrX	32613922	32613922	http://gnomad.b	0.002904934592	AFR	0.03759710118	9	150	3	43	https://www.ncbi.nlm.nih.gov/clinvar/variation/94473/
NM_004006.2(DMD):c.1997C>T	concordant	unreportable	unreportable	94487	0	3	SCV000603366;S	Benign;Benign;Benign	ARUP Laboratories	2017-06-30;2017-1	DMD	chrX	32563447	32563447	http://gnomad.b	0.001338548292	AFR	0.01397520024	0	49	0	17	https://www.ncbi.nlm.nih.gov/clinvar/variation/94487/
NM_004006.2(DMD):c.2143A>T	concordant	unreportable	unreportable	94494	0	3	SCV000603348;S	Benign;Benign;Benign	ARUP Laboratories	2017-06-30;2017-1	DMD	chrX	32563301	32563301	http://gnomad.b	0.001727771554	AFR	0.02002779953	3	79	2	28	https://www.ncbi.nlm.nih.gov/clinvar/variation/94494/
NM_004006.2(DMD):c.2386G>A	concordant	unreportable	unreportable	94509	0	1	SCV000625868	Benign	Invitae	10/5/2017	DMD	chrX	32509630	32509630	http://gnomad.b	0.0003037840096	ASJ	0.001249479945	0	17	0	2	https://www.ncbi.nlm.nih.gov/clinvar/variation/94509/
NM_004006.2(DMD):c.2391T>G	concordant	unreportable	unreportable	94510	0	4	SCV000318674;S	Benign;Benign;Benign;Beni	Ambry Genetics;EG	2017-11-14;2017-0	DMD	chrX	32509625	32509625	http://gnomad.b	0.007091457973	NFE	0.01101600006	8	406	4	140	https://www.ncbi.nlm.nih.gov/clinvar/variation/94510/

Supplemental Table 2

NM_004006.2(DMD):c.3021G>A	concordant	unreportable	unreportable	94548	0	3	SCV000603354;S	Benign;Benign;Benign	ARUP Laboratories	2017-06-30;2017-1	DMD	chrX	32486756	32486756	http://gnomad.b	0.01362281418	NFE	0.02018369921	11	894	9	275	https://www.ncbi.nlm.nih.gov/clinvar/variation/94548/
NM_004006.2(DMD):c.3276+17A>T	concordant	unreportable	unreportable	94582	0	1	SCV000112473	Benign	EGL Genetic Diagn	6/30/2017	DMD	chrX	32482686	32482686	http://gnomad.b	0.000920845279	AFR	0.01096470002	2	38	0	13	https://www.ncbi.nlm.nih.gov/clinvar/variation/94582/
NM_004006.2(DMD):c.3406A>T	concordant	unreportable	unreportable	94587	0	4	SCV000318913;S	Benign;Likely benign;Benig	Ambry Genetics;Illu	2017-11-14;2016-1	DMD	chrX	32481582	32481582	http://gnomad.b	0.006873113217	SAS	0.05015030131	31	886	18	136	https://www.ncbi.nlm.nih.gov/clinvar/variation/94587/
NM_004006.2(DMD):c.3603+8A>G	concordant	unreportable	unreportable	94600	0	3	SCV000603367;S	Benign;Uncertain significar	ARUP Laboratories	2017-06-30;2016-1	DMD	chrX	32472771	32472771		0.002098008316					0	34	https://www.ncbi.nlm.nih.gov/clinvar/variation/94600/
NM_004006.2(DMD):c.3734C>T	concordant	unreportable	unreportable	94604	0	4	SCV000318573;S	Benign;Benign;Likely benig	Ambry Genetics;EG	2017-11-14;2017-0	DMD	chrX	32466625	32466625	http://gnomad.b	0.01330953692	AMR	0.08937940001	112	969	23	265	https://www.ncbi.nlm.nih.gov/clinvar/variation/94604/
NM_004006.2(DMD):c.3787-18T>C	concordant	unreportable	unreportable	94607	0	1	SCV000112498	Benign	EGL Genetic Diagn	6/30/2017	DMD	chrX	32459449	32459449	http://gnomad.b	0.0063604777	ASJ	0.01106599998	5	441	5	136	https://www.ncbi.nlm.nih.gov/clinvar/variation/94607/
NM_004006.2(DMD):c.5182C>T	concordant	unreportable	unreportable	94656	0	2	SCV000112548;S	Likely benign;Benign	EGL Genetic Diagn	2017-06-30;2017-1	DMD	chrX	32381048	32381048	http://gnomad.b	0.000825912776	AFR	0.009853029624	0	24	0	11	https://www.ncbi.nlm.nih.gov/clinvar/variation/94656/
NM_004006.2(DMD):c.5984A>T	concordant	unreportable	unreportable	94687	0	2	SCV000235845;S	Likely benign;Benign	GeneDx;Invitae	2017-11-28;2017-0	DMD	chrX	32328332	32328332	http://gnomad.b	0.0002658110084	AFR	0.003116030013	0	15	0	4	https://www.ncbi.nlm.nih.gov/clinvar/variation/94687/
NM_004006.2(DMD):c.6322C>T	concordant	unreportable	unreportable	94703	0	3	SCV000317913;S	Benign;Likely benign;Benig	Ambry Genetics;Illu	2017-11-14;2016-1	DMD	chrX	32235149	32235149	http://gnomad.b	0.002999867094	AFR	0.03648189828	4	137	6	44	https://www.ncbi.nlm.nih.gov/clinvar/variation/94703/
NM_004006.2(DMD):c.6463C>T	concordant	unreportable	unreportable	94741	0	5	SCV000603340;S	Benign;Benign;Benign;Likel	ARUP Laboratories	2017-06-30;2017-1	DMD	chrX	31986607	31986607	http://gnomad.b	0.02803356813	NFE	0.0410484001	69	1780	48	601	https://www.ncbi.nlm.nih.gov/clinvar/variation/94741/
NM_004006.2(DMD):c.6828C>T	concordant	unreportable	unreportable	94747	0	2	SCV000112642;S	Benign;Benign	EGL Genetic Diagn	2017-06-30;2017-1	DMD	chrX	31947797	31947797	http://gnomad.b	0.0004556760144	FIN	0.0009797320236	0	35	0	9	https://www.ncbi.nlm.nih.gov/clinvar/variation/94747/
NM_004006.2(DMD):c.7183G>A	concordant	unreportable	unreportable	94757	0	5	SCV000603350;S	Benign;Likely benign;Benig	ARUP Laboratories	2017-06-30;2017-1	DMD	chrX	31854852	31854852	http://gnomad.b	0.001072737284	NFE	0.001611190033	0	54	1	20	https://www.ncbi.nlm.nih.gov/clinvar/variation/94757/
NM_004006.2(DMD):c.7476T>C	concordant	unreportable	unreportable	94765	0	1	SCV000288069	Benign	Invitae	10/5/2017	DMD	chrX	31792143	31792143	http://gnomad.b	0.001756251305	AFR	0.0207464993	3	59	2	15	https://www.ncbi.nlm.nih.gov/clinvar/variation/94765/
NM_004006.2(DMD):c.802T>C	concordant	unreportable	unreportable	94784	0	5	SCV000319100;S	Likely benign;Benign;Likely	Ambry Genetics;EG	2017-11-14;2017-0	DMD	chrX	32717258	32717258	http://gnomad.b	0.004575746644	AMR	0.04375680163	20	435	6	66	https://www.ncbi.nlm.nih.gov/clinvar/variation/94784/
NM_004006.2(DMD):c.8571T>C	concordant	unreportable	unreportable	94809	0	2	SCV000482226;S	Uncertain significance;Beni	Illumina Clinical Se	2016-10-18;2017-1	DMD	chrX	31497197	31497197	http://gnomad.b	0.001955609562	ASJ	0.006597939879	1	103	0	37	https://www.ncbi.nlm.nih.gov/clinvar/variation/94809/
NM_004543.4(NEB):c.14352G>A	concordant	unreportable	unreportable	95110	0	3	SCV000533973;S	Likely benign;Uncertain sig	GeneDx;Illumina C	2017-11-28;2016-1	NEB	chr2	152410513	152410513	http://gnomad.b	0.001277894237	FIN	0.01318390016	9		2	0	https://www.ncbi.nlm.nih.gov/clinvar/variation/95110/
NM_000070.2(CAPN3):c.1435A>G	concordant	reportable	reportable	92405	3	0	SCV000331499;S	Pathogenic;Likely pathoger	EGL Genetic Diagn	2017-06-30;2016-1	CAPN3	chr15	42693919	42693919	http://gnomad.b	7.20E-05	NFE	0.0001074480024	0		0	0	https://www.ncbi.nlm.nih.gov/clinvar/variation/92405/
NM_000070.2(CAPN3):c.1746-20C>G	concordant	unreportable	unreportable	92408	0	3	SCV000109927;S	Likely benign;Uncertain sig	EGL Genetic Diagn	2017-06-30;2017-1	CAPN3	chr15	42695919	42695919	http://gnomad.b	0.002968427454	FIN	0.01000269968	3		1	0	https://www.ncbi.nlm.nih.gov/clinvar/variation/92408/
NM_000070.2(CAPN3):c.2393C>A	concordant	reportable	reportable	92414	4	0	SCV000255664;S	Pathogenic;Pathogenic;Pat	Athena Diagnostics	2015-10-06;2017-0	CAPN3	chr15	42703497	42703497	http://gnomad.b	0.0001040149782	NFE	0.0001970580051	0		0	0	https://www.ncbi.nlm.nih.gov/clinvar/variation/92414/
NM_000318.2(PEX2):c.722G>T	concordant	unreportable	unreportable	92837	0	1	SCV000110487	Uncertain significance	EGL Genetic Diagn	6/30/2017	PEX2	chr8	77895693	77895693	http://gnomad.b	0.0002080532616	AFR	0.001830070047	0		0	0	https://www.ncbi.nlm.nih.gov/clinvar/variation/92837/
NM_000318.2(PEX2):c.91C>G	concordant	unreportable	unreportable	92838	0	2	SCV000110488;S	Benign;Uncertain significar	EGL Genetic Diagn	2017-06-30;2016-1	PEX2	chr8	77896324	77896324	http://gnomad.b	0.0008162089495	FIN	0.01618280075	8		0	0	https://www.ncbi.nlm.nih.gov/clinvar/variation/92838/
NM_000404.2(GLB1):c.1426C>T	concordant	unreportable	unreportable	92897	0	1	SCV000443171	Uncertain significance	Illumina Clinical Se	10/18/2016	GLB1	chr3	33058254	33058254	http://gnomad.b	0.0007761987069	AMR	0.001756889978	0		0	0	https://www.ncbi.nlm.nih.gov/clinvar/variation/92897/
NM_000426.3(LAMA2):c.1621A>G	concordant	unreportable	unreportable	92939	0	5	SCV000613961;S	Uncertain significance;Unc	Athena Diagnostics	2017-08-17;2017-0	LAMA2	chr6	129513837	129513837	http://gnomad.b	0.002416618654	NFE	0.002777429996	1		0	0	https://www.ncbi.nlm.nih.gov/clinvar/variation/92939/
NM_000426.3(LAMA2):c.2115T>G	concordant	unreportable	unreportable	92945	0	3	SCV000527742;S	Likely benign;Likely benign	GeneDx;Genetic Se	2017-11-28;2017-0	LAMA2	chr6	129581874	129581874	http://gnomad.b	0.0006961782216	NFE	0.001192420023	1		0	0	https://www.ncbi.nlm.nih.gov/clinvar/variation/92945/
NM_000426.3(LAMA2):c.6322C>T	concordant	unreportable	unreportable	92976	0	1	SCV000110647	Uncertain significance	EGL Genetic Diagn	6/30/2017	LAMA2	chr6	129766859	129766859	http://gnomad.b	9.60E-05	SAS	0.0003249289875	0		0	0	https://www.ncbi.nlm.nih.gov/clinvar/variation/92976/
NM_000426.3(LAMA2):c.7111T>G	concordant	unreportable	unreportable	92981	0	2	SCV000620505;S	Uncertain significance;Likel	GeneDx;Invitae	2017-11-28;2017-1	LAMA2	chr6	129785553	129785553	http://gnomad.b	0.0003600921836	AFR	0.005559190176	0		0	0	https://www.ncbi.nlm.nih.gov/clinvar/variation/92981/
NM_000426.3(LAMA2):c.8692A>C	concordant	unreportable	unreportable	92993	0	2	SCV000110666;S	Uncertain significance;Likel	EGL Genetic Diagn	2017-06-30;2017-1	LAMA2	chr6	129826489	129826489	http://gnomad.b	0.0002880737469	AMR	0.002591599943	0		0	0	https://www.ncbi.nlm.nih.gov/clinvar/variation/92993/
NM_001918.3(DBT):c.1127G>A	concordant	unreportable	unreportable	93982	0	1	SCV000346204	Uncertain significance	Illumina Clinical Se	10/18/2016	DBT	chr1	100672083	100672083	http://gnomad.b	3.99E-05	AFR	0.0002614039986	0		0	0	https://www.ncbi.nlm.nih.gov/clinvar/variation/93982/
NM_001918.3(DBT):c.1418A>G	concordant	unreportable	unreportable	93993	0	1	SCV000346196	Uncertain significance	Illumina Clinical Se	10/18/2016	DBT	chr1	100661842	100661842	http://gnomad.b	0.002130613808	NFE	0.003759329906	3		1	0	https://www.ncbi.nlm.nih.gov/clinvar/variation/93993/
NM_001918.3(DBT):c.670G>T	concordant	reportable	reportable	94009	1	0	SCV000680731	Pathogenic	GeneDx	2/7/2018	DBT	chr1	100681641	100681641	http://gnomad.b	6.38E-05	AFR	0.001045480021	0		0	0	https://www.ncbi.nlm.nih.gov/clinvar/variation/94009/
NM_001918.3(DBT):c.901C>T	concordant	reportable	reportable	94016	1	0	SCV000238793	Pathogenic	GeneDx	11/28/2017	DBT	chr1	100680411	100680411	http://gnomad.b	0.0002553544639	NFE	0.001038720016	2		0	0	https://www.ncbi.nlm.nih.gov/clinvar/variation/94016/
NM_003494.3(DYSF):c.1120G>C	concordant	unreportable	unreportable	94264	0	3	SCV000342092;S	Benign;Benign;Benign	EGL Genetic Diagn	2016-11-03;2017-1	DYSF	chr2	71753416	71753416	http://gnomad.b	0.005803896209	FIN	0.009058300406	8		10	0	https://www.ncbi.nlm.nih.gov/clinvar/variation/94264/
NM_001130987.1(DYSF):c.431C>A	concordant	unreportable	unreportable	94322	0	1	SCV000431677	Uncertain significance	Illumina Clinical Se	10/18/2016	DYSF	chr2	71739022	71739022	http://gnomad.b	0.0001050844717	AFR	0.001043020049	0		0	0	https://www.ncbi.nlm.nih.gov/clinvar/variation/94322/
NM_003494.3(DYSF):c.4577A>C	concordant	unreportable	unreportable	94327	0	3	SCV000337924;S	Likely benign;Likely benign	EGL Genetic Diagn	2016-11-03;2017-1	DYSF	chr2	71883359	71883359	http://gnomad.b	0.0001859186808	AFR	0.001698900014	0		0	0	https://www.ncbi.nlm.nih.gov/clinvar/variation/94327/
NM_003494.3(DYSF):c.4742G>A	concordant	unreportable	unreportable	94329	0	3	SCV000112190;S	Uncertain significance;Unc	EGL Genetic Diagn	2017-06-30;2017-0	DYSF	chr2	71886111	71886111	http://gnomad.b	0.0008487591949	EAS	0.003537049983	0		0	0	https://www.ncbi.nlm.nih.gov/clinvar/variation/94329/
NM_003494.3(DYSF):c.4876G>A	concordant	unreportable	unreportable	94332	0	1	SCV000112193	Likely benign	EGL Genetic Diagn	6/30/2017	DYSF	chr2	71887771	71887771	http://gnomad.b	0.0009538436666	EAS	0.009570769966	0		2	0	https://www.ncbi.nlm.nih.gov/clinvar/variation/94332/
NM_004006.2(DMD):c.-1128G>A	concordant	unreportable	unreportable	94395	0	1	SCV000112280	Benign	EGL Genetic Diagn	6/30/2017	DMD	chrX	33230557	33230557		0.00571493668					1	111	https://www.ncbi.nlm.nih.gov/clinvar/variation/94395/
NM_004006.2(DMD):c.10262+1G>A	concordant	unreportable	unreportable	94433	0	3	SCV000112320;S	Uncertain significance;Unc	EGL Genetic Diagn	2017-06-30;2017-1	DMD	chrX	31196048	31196048	http://gnomad.b	0.0001993582563	AFR	0.00159597001	0	9	0	1	https://www.ncbi.nlm.nih.gov/clinvar/variation/94433/
NM_004006.2(DMD):c.10643C>T	concordant	unreportable	unreportable	94444	0	1	SCV000530430	Likely benign	GeneDx	11/28/2017	DMD	chrX	31165546	31165546	http://gnomad.b	2.85E-05	AFR	0.0001636130037	0	1	0	1	https://www.ncbi.nlm.nih.gov/clinvar/variation/94444/
NM_004006.2(DMD):c.1337A>G	concordant	unreportable	unreportable	94461	0	2	SCV000320017;S	Uncertain significance;Likel	Ambry Genetics;Ge	2017-11-14;2017-1	DMD	chrX	32632565	32632565	http://gnomad.b	9.49E-05	FIN	0.001625200035	0	23	0	2	https://www.ncbi.nlm.nih.gov/clinvar/variation/94461/
NM_004006.2(DMD):c.1731A>T	concordant	unreportable	unreportable	94478	0	2	SCV000112366;S	Uncertain significance;Likel	EGL Genetic Diagn	2017-06-30;2017-1	DMD	chrX	32591728	32591728	http://gnomad.b	0.0002753042587	AFR	0.001715549966	0	10	0	5	https://www.ncbi.nlm.nih.gov/clinvar/variation/94478/
NM_004006.2(DMD):c.2457A>C	concordant	unreportable	unreportable	94513	0	2	SCV000112403;S	Uncertain significance;Beni	EGL Genetic Diagn	2017-06-30;2017-1	DMD	chrX	32509559	32509559	http://gnomad.b	0.0002942907593	NFE	0.0005132119986	0	17	0	5	https://www.ncbi.nlm.nih.gov/clinvar/variation/94513/
NM_004006.2(DMD):c.2490C>T	concordant	unreportable	unreportable	94516	0	3	SCV000112406;S	Benign;Benign;Likely benig	EGL Genetic Diagn	2017-06-30;2017-1	DMD	chrX	32509526	32509526	http://gnomad.b	0.0005316220167	NFE	0.0008610790246	0	39	0	15	https://www.ncbi.nlm.nih.gov/clinvar/variation/94516/
NM_004006.2(DMD):c.2827C>T	concordant	unreportable	unreportable	94539	0	3	SCV000112429;S	Benign;Likely benign;Benig	EGL Genetic Diagn	2017-06-30;2017-1	DMD	chrX	32490403	32490403	http://gnomad.b	0.0002468245078	NFE	0.0003757700033	0	13	0	4	https://www.ncbi.nlm.nih.gov/clinvar/variation/94539/
NM_004006.2(DMD):c.3432+2036A>C	concordant	unreportable	unreportable	94590	0	2	SCV000112481;S	Likely benign;Uncertain sig	EGL Genetic Diagn	2017-06-30;2016-0	DMD	chrX	32479520	32479520		0.004252976134					2	68	https://www.ncbi.nlm.nih.gov/clinvar/variation/94590/
NM_004006.2(DMD):c.3970C>T	concordant	unreportable	unreportable	94608	0	6	SCV000320674;S	Uncertain significance;Beni	Ambry Genetics;At	2017-11-14;2017-0	DMD	chrX	32456459	32456459	http://gnomad.b	0.0004366895138	AFR	0.006946150213	0	18	0	9	https://www.ncbi.nlm.nih.gov/clinvar/variation/94608/
NM_004006.2(DMD):c.4072-245C>T	concordant	unreportable	unreportable	94610	0	1	SCV000271676	Uncertain significance	Laboratory for Mol	4/26/2016	DMD	chrX	32430275	32430275	http://gnomad.b	0.0002563177581	AFR	0.001672239974	1	10	1	5	https://www.ncbi.nlm.nih.gov/clinvar/variation/94610/
NM_004006.2(DMD):c.4093C>T	concordant	unreportable	unreportable	94611	0	3	SCV000112502;S	Likely benign;Likely benign	EGL Genetic Diagn	2017-06-30;2017-1	DMD	chrX	32430009	32430009	http://gnomad.b	0.0002088515066	AFR	0.002127310028	0	3	0	4	https://www.ncbi.nlm.nih.gov/clinvar/variation/94611/
NM_004006.2(DMD):c.4233+2C>T	concordant	unreportable	unreportable	94616	0	4	SCV000319745;S	Likely benign;Benign;Likely	Ambry Genetics;At	2017-11-14;2017-0	DMD	chrX	32429867	32429867	http://gnomad.b	0.0005316220167	AMR	0.001921189949	1	38	0	12	https://www.ncbi.nlm.nih.gov/clinvar/variation/94616/
NM_004006.2(DMD):c.4529A>G	concordant	unreportable	unreportable	94632	0	3	SCV000112524;S	Benign;Likely benign;Benig	EGL Genetic Diagn	2017-06-30;2016-1	DMD	chrX	32404572	32404572	http://gnomad.b	0.00188915681	FIN	0.04277610034	15	334	0	31	https://www.ncbi.nlm.nih.gov/clinvar/variation/94632/
NM_004006.2(DMD):c.5181A>T	concordant	unreportable	unreportable	94655	0	2	SCV000112547;S	Uncertain significance;Beni	EGL Genetic Diagn	2017-06-30;2017-0	DMD	chrX	32381049	32381049	http://gnomad.b	0.0001423987545	AMR	0.0005288210232	0	16	0	5	https://www.ncbi.nlm.nih.gov/clinvar/variation/94655/
NM_004006.2(DMD):c.5586+18A>G	concordant	unreportable	unreportable	94672	0	1	SCV000112564	Benign	EGL Genetic Diagn	6/30/2017	DMD	chrX	32364042	32364042	http://gnomad.b	0.0009493250299	EAS	0.007284979802	0	30	2	15	https://www.ncbi.nlm.nih.gov/clinvar/variation/94672/
NM_004006.2(DMD):c.5620G>A	concordant	unreportable	unreportable	94675	0	4	SCV000235874;S	Likely benign;Uncertain sig	GeneDx;Illumina C	2017-11-28;2016-1	DMD	chrX	32361370	32361370	http://gnomad.b	0.0002563177581	AFR	0.003106100019	0	8	0	6	https://www.ncbi.nlm.nih.gov/clinvar/variation/94675/
NM_004006.2(DMD):c.606C>T	concordant	unreportable	unreportable	94690	0	2	SCV000112583;S	Uncertain significance;Beni	EGL Genetic Diagn	2017-06-30;2017-1	DMD	chrX	32827653	32827653	http://gnomad.b	6.65E-05	AMR	0.000376534008	0	4	0	1	https://www.ncbi.nlm.nih.gov/clinvar/variation/94690/
NM_004006.2(DMD):c.6320G>A	concordant	unreportable	unreportable	94702	0	3	SCV000112596;S	Uncertain significance;Unc	EGL Genetic Diagn	2017-06-30;2017-0	DMD	chrX	32235151	32235151	http://gnomad.b	0.0003037840096	AFR	0.0009050520021	0	15	0	8	https://www.ncbi.nlm.nih.gov/clinvar/variation/94702/
NM_004006.2(DMD):c.7151C>A	concordant	unreportable	unreportable	94756	0	3	SCV000112651;S	Benign;Uncertain significar	EGL Genetic Diagn	2017-06-30;2016-1	DMD	chrX	31854884	31854884	http://gnomad.b	0.0005695950179	EAS	0.004584299866	1	23	0	6	https://www.ncbi.nlm.nih.gov/clinvar/variation/94756/
NM_004006.2(DMD):c.8767G>T	concordant	unreportable	unreportable	94816	0	2	SCV000112715;S	Uncertain significance;Likel	EGL Genetic Diagn	2017-06-30;2017-1	DMD	chrX	31496393	31496393	http://gnomad.b	0.0001803717557	AFR	0.001143319998	0	6	0	5	https://www.ncbi.nlm.nih.gov/clinvar/variation/94816/
NM_004006.2(DMD):c.9G>A	concordant	reportable	reportable	29962	3	0	SCV000224422;S	Pathogenic;Pathogenic;Pat	EGL Genetic Diagn	2017-06-30;2017-1	DMD	chrX	33229421	33229421		9.49E-06					0	0	https://www.ncbi.nlm.nih.gov/clinvar/variation/29962/
NM_024301.4(FKRP):c.520A>T	concordant	unreportable	unreportable	96110	0	4	SCV000613309;S	Likely benign;Benign;Benig	Athena Diagnostics	2017-08-17;2017-0	FKRP	chr19	47259227	47259227	http://gnomad.b	0.00226465222	SAS	0.01440440025	7		0	0	https://www.ncbi.nlm.nih.gov/clinvar/variation/96110/
NM_024301.4(FKRP):c.606G>A	concordant	unreportable	unreportable	96113	0	3	SCV000114126;S	Uncertain significance;Likel	EGL Genetic Diagn	2017-06-30;2017-1	FKRP	chr19	47259313	47259313	http://gnomad.b	0.0001440414839	AFR	0.002231149934	0		0	0	https://www.ncbi.nlm.nih.gov/clinvar/variation/96113/
NM_000492.3(CFTR):c.-812T>G	concordant	unreportable	unreportable	51029	0	3	SCV000705550;S	Likely benign;Likely benign	EGL Genetic Diagn	2017-06-30;2016-0	CFTR	chr7	117119337	117119337		0.002547876192					12	0	https://www.ncbi.nlm.nih.gov/clinvar/variation/51029/
NM_000492.3(CFTR):c.3717+40A>G	concordant	reportable	reportable	53789	1	0	SCV000677605	Pathogenic	CFTR2	6/29/2017	CFTR	chr7	117267864	117267864		1.93E-06					0	0	https://www.ncbi.nlm.nih.gov/clinvar/variation/53789/
NM_000492.3(CFTR):c.3935A>G	concordant	unreportable	unreportable	53857	0	1	SCV000601112	Uncertain significance	Quest Diagnostics I	8/1/2017	CFTR	chr7	117292957	117292957	http://gnomad.b	3.66E-05	NFE	9.02E-06	0		0	0	https://www.ncbi.nlm.nih.gov/clinvar/variation/53857/
NM_000016.4(ACADM):c.388-12_388-	concordant	unreportable	unreportable	92264	0	1	SCV000109717	Uncertain significance	EGL Genetic Diagn	6/30/2017	ACADM	chr1	76200462	76200465		2.72E-06					0	0	https://www.ncbi.nlm.nih.gov/clinvar/variation/92264/
NM_000018.3(ACADVL):c.1001T>G	concordant	unreportable	unreportable	92270	1	1	SCV000109727;S	Uncertain significance;Likel	EGL Genetic Diagn	2017-06-30;2017-1	ACADVL	chr17	7126108	7126108	http://gnomad.b	2.27E-05	NFE	3.58E-05	0		0	0	https://www.ncbi.nlm.nih.gov/clinvar/variation/92270/
NM_000018.3(ACADVL):c.1825G>A	concordant	unreportable	unreportable	92281	0	1	SCV000109741	Uncertain significance	EGL Genetic Diagn	6/30/2017	ACADVL	chr17	7128201	7128201	http://gnomad.b	2.83E-06	SAS	3.25E-05	0		0	0	https://www.ncbi.nlm.nih.gov/clinvar/variation/92281/
NM_000018.3(ACADVL):c.605T>C	concordant	unreportable	unreportable	92288	0	1	SCV000602360	Uncertain significance	ARUP Laboratories	6/30/2017	ACADVL	chr17	7124984	7124984	http://gnomad.b	2.83E-06			0		0	0	https://www.ncbi.nlm.nih.gov/clinvar/variation/92288/
NM_000155.3(GALT):c.291C>T	concordant	unreportable	unreportable	92516	0	1	SCV000110056	Uncertain significance	EGL Genetic Diagn	6/30/2017	GALT	chr9	34647527	34647527		1.10E-05					0	0	https://www.ncbi.nlm.nih.gov/clinvar/variation/92516/
NM_000155.3(GALT):c.820+4A>C	concordant	unreportable	unreportable	92523	0	1	SCV000110075	Uncertain significance	EGL Genetic Diagn	6/30/2017	GALT	chr9	34648895	34648895	http://gnomad.b	2.75E-06	AFR	6.54E-05	0		0	0	https://www.ncbi.nlm.nih.gov/clinvar/variation/92523/
NM_000286.2(PEX12):c.888_889delCT	concordant	reportable	reportable	92776	2	0	SCV000110419;S	Pathogenic;Pathogenic	EGL Genetic Diagn	2017-06-30;2018-0	PEX12	chr17	33902991	33902993	http://gnomad.b	0.00012003457	AFR	0.0003267120046	0		0	0	https://www.ncbi.nlm.nih.gov/clinvar/variation/92776/
NM_000287.3(PEX6):c.1480-5C>T	concordant	unreportable	unreportable	92780	0	1	SCV000110423	Benign	EGL Genetic Diagn	6/30/2017	PEX6	chr6	42936241	42936241	http://gnomad.b	0.003672881492	ASJ	0.01650829986	4		2	0	https://www.ncbi.nlm.nih.gov/clinvar/variation/92780/
NM_000287.3(PEX6):c.210G>A	concordant	unreportable	unreportable	92783	0	1	SCV000110426	Benign	EGL Genetic Diagn	6/30/2017	PEX6	chr6	42946679	42946679	http://gnomad.b	0.007801872449	NFE	0.0125892004	4		2	0	https://www.ncbi.nlm.nih.gov/clinvar/variation/92783/
NM_000426.3(LAMA2):c.3630delT	concordant	reportable	reportable	92954	2	0	SCV000228198;S	Pathogenic;Pathogenic	EGL Genetic Diagn	2017-06-30;2017-1	LAMA2	chr6	129636693	129636694	http://gnomad.b	2.40E-05	NFE	5.37E-05	0		0	0	https://www.ncbi.nlm.nih.gov/clinvar/variation/92954/
NM_000426.3(LAMA2):c.3976C>T	concordant	reportable	reportable	92956	1	0	SCV000617517	Pathogenic	GeneDx	11/28/2017	LAMA2	chr6	129637234	129637234	http://gnomad.b	2.40E-05	NFE	0.0001165770009	0		0	0	https://www.ncbi.nlm.nih.gov/clinvar/variation/92956/
NM_000426.3(LAMA2):c.5072-6delC	concordant	unreportable	unreportable	92965	0	3	SCV000110636;S	Benign;Likely benign;Benig	EGL Genetic Diagn	2017-06-30;2016-1	LAMA2	chr6	129712625	129712626	http://gnomad.b	0.002968760003	AMR	0.05035800114	51		7	0	https://www.ncbi.nlm.nih.gov/clinvar/variation/92965/
NM_000426.3(LAMA2):c.5914C>T	concordant	reportable	reportable	92971	1	0	SCV000658727	Pathogenic	Invitae	10/5/2017	LAMA2	chr6	129748945	129748945		2.40E-05					0	0	https://www.ncbi.nlm.nih.gov/clinvar/variation/92971/
NM_000426.3(LAMA2):c.6563G>A	concordant	unreportable	unreportable	92978	0	2	SCV000460067;S	Uncertain significance;Unc	Illumina Clinical Se	2016-10-18;2017-1	LAMA2	chr6	129774266	129774266	http://gnomad.b	0.0001040266308	NFE	0.0002427099971	0		0	0	https://www.ncbi.nlm.nih.gov/clinvar/variation/92978/
NM_000426.3(LAMA2):c.6955C>T	concordant	reportable	reportable	92980	1	0	SCV000230812	Pathogenic	EGL Genetic Diagn	6/30/2017	LAMA2	chr6	129781432	129781432	http://gnomad.b	6.40E-05	NFE	3.60E-05	0		0	0	https://www.ncbi.nlm.nih.gov/clinvar/variation/92980/
NM_000487.5(ARSA):c.1211-10C>T	concordant	unreportable	unreportable	93119	0	1	SCV000110800	Uncertain significance	EGL Genetic Diagn	6/30/2017	ARSA	chr22	51063902	51063902	http://gnomad.b	2.81E-05	SAS	0.0002651289979	0		0	0	https://www.ncbi.nlm.nih.gov/clinvar/variation/93119/
NM_000521.3(HEXB):c.1614-14C>A	concordant	unreportable	unreportable	93199	0	1	SCV000513237	Likely benign	GeneDx	11/28/2017	HEXB	chr5	74016929	74016929	http://gnomad.b	0.0002800268826	NFE	0.0003053869877	0		0	0	https://www.ncbi.nlm.nih.gov/clinvar/variation/93199/
NM_000709.3(BCKDHA):c.978C>T	concordant	unreportable	unreportable	93384	0	1	SCV000536415	Likely benign	GeneDx	11/28/2017	BCKDHA	chr19	41928658	41928658	http://gnomad.b	3.96E-05	EAS	0.0002900570107	0		0	0	https://www.ncbi.nlm.nih.gov/clinvar/variation/93384/
NM_001918.3(DBT):c.76T>C	concordant	unreportable	unreportable	94013	0	1	SCV000512789	Likely benign	GeneDx	11/28/2017	DBT	chr1	100706416	100706416	http://gnomad.b	0.0008139423537	AFR	0.009023150429	0		0	0	https://www.ncbi.nlm.nih.gov/clinvar/variation/94013/
NM_003494.3(DYSF):c.1053+1G>A	concordant	reportable	reportable	94262	1	0	SCV000331460	Pathogenic	EGL Genetic Diagn	6/30/2017	DYSF	chr2	71748035	71748035		1.62E-05					0	0	https://www.ncbi.nlm.nih.gov/clinvar/variation/94262/
NM_003494.3(DYSF):c.1663C>T	concordant	reportable	reportable	94278	3	0	SCV000613184;S	Pathogenic;Pathogenic;Pat	Athena Diagnostics	2017-08-17;2017-0	DYSF	chr2	71778761	71778761	http://gnomad.b	3.23E-05	SAS	0.0001033630033	0		0	0	https://www.ncbi.nlm.nih.gov/clinvar/variation/94278/
NM_003494.3(DYSF):c.3032-17G>T	concordant	unreportable	unreportable	94298	0	1	SCV000112158	Uncertain significance	EGL Genetic Diagn	6/30/2017	DYSF	chr2	71797712	71797712	http://gnomad.b	2.43E-05	NFE	4.57E-05	0		0	0	https://www.ncbi.nlm.nih.gov/clinvar/variation/94298/
NM_003494.3(DYSF):c.3444_3445delT	concordant	reportable	reportable	94305	2	0	SCV000331273;S	Pathogenic;Pathogenic	EGL Genetic Diagn	2017-06-30;2017-1	DYSF	chr2	71817341	71817343		2.43E-05					0	0	https://www.ncbi.nlm.nih.gov/clinvar/variation/94305/
NM_003494.3(DYSF):c.4756C>T	concordant	reportable	reportable	94330	1	0	SCV000230620	Pathogenic	EGL Genetic Diagn	6/30/2017	DYSF	chr2	71886125	71886125	http://gnomad.b	4.85E-05	SAS	6.50E-05	0		0	0	https://www.ncbi.nlm.nih.gov/clinvar/variation/94330/
NM_001130987.1(DYSF):c.606G>A	concordant	unreportable	unreportable	94335	0	1	SCV000112196	Uncertain significance	EGL Genetic Diagn	6/30/2017	DYSF	chr2	71740898	71740898	http://gnomad.b	0.0001293347345	SAS	0.001104759984	0		0	0	https://www.ncbi.nlm.nih.gov/clinvar/variation/94335/
NM_003494.3(DYSF):c.5836_5839delC	concordant	reportable	reportable	94347	2	0	SCV000255774;S	Pathogenic;Pathogenic	Athena Diagnostics	2015-10-06;2017-0	DYSF	chr2	71906253	71906257		2.43E-05					0	0	https://www.ncbi.nlm.nih.gov/clinvar/variation/94347/
NM_003494.3(DYSF):c.610C>T	concordant	reportable	reportable	94352	1	0	SCV000331137	Pathogenic	EGL Genetic Diagn	6/30/2017	DYSF	chr2	71740998	71740998	http://gnomad.b	4.04E-05	AMR	2.98E-05	0		0	0	https://www.ncbi.nlm.nih.gov/clinvar/variation/94352/
NM_003494.3(DYSF):c.663+1G>C	concordant	reportable	reportable	94355	1	0	SCV000331410	Pathogenic	EGL Genetic Diagn	6/30/2017	DYSF	chr2	71741052	71741052		8.08E-06					0	0	https://www.ncbi.nlm.nih.gov/clinvar/variation/94355/
NM_004006.2(DMD):c.1812C>T	concordant	unreportable	unreportable	94480	0	3	SCV000112368;S	Uncertain significance;Likel	EGL Genetic Diagn	2017-06-30;2017-1	DMD	chrX	32591647	32591647	http://gnomad.b	0.000284797509	AFR	0.002615659963	0	18	0	5	https://www.ncbi.nlm.nih.gov/clinvar/variation/94480/
NM_004006.2(DMD):c.1848C>A	concordant	unreportable	unreportable	94481	0	3	SCV000112369;S	Uncertain significance;Likel	EGL Genetic Diagn	2017-06-30;2017-1	DMD	chrX	32583963	32583963	http://gnomad.b	6.65E-05	AMR	0.001960640075	0	6	0	0	https://www.ncbi.nlm.nih.gov/clinvar/variation/94481/
NM_004006.2(DMD):c.2173G>T	concordant	unreportable	unreportable	94498	0	1	SCV000112387	Benign	EGL Genetic Diagn	6/30/2017	DMD	chrX	32536244	32536244	http://gnomad.b	0.0001803717557	SAS	0.002328410046	0	26	0	0	https://www.ncbi.nlm.nih.gov/clinvar/variation/94498/
NM_004006.2(DMD):c.31+68A>G	concordant	unreportable	unreportable	94560	0	1	SCV000112450	Uncertain significance	EGL Genetic Diagn	6/30/2017	DMD	chrX	33229331	33229331		0.0002753042587					0	3	https://www.ncbi.nlm.nih.gov/clinvar/variation/94560/
NM_004006.2(DMD):c.3217G>A	concordant	unreportable	unreportable	94579	0	2	SCV000112470;S	Uncertain significance;Beni	EGL Genetic Diagn	2017-06-30;2017-1	DMD	chrX	32482762	32482762	http://gnomad.b	0.0001518920048	AMR	0.002523160074	1	17	0	2	https://www.ncbi.nlm.nih.gov/clinvar/variation/94579/
NM_004006.2(DMD):c.3419A>G	concordant	unreportable	unreportable	94588	0	3	SCV000603376;S	Benign;Uncertain significar	ARUP Laboratories	2017-06-30;2017-0	DMD	chrX	32481569	32481569	http://gnomad.b	6.65E-05	AMR	0.001871049986	0	15	0	1	https://www.ncbi.nlm.nih.gov/clinvar/variation/94588/

Supplemental Table 2

NM_004006.2(DMD):c.4125T>C	concordant	unreportable	unreportable	94613	0	3	SCV000319947;S(	Likely benign;Likely benign,	Ambry Genetics;EG	2017-11-14;2017-0	DMD	chrX	32429977	32429977	http://gnomad.b	0.0002753042587	AFR	0.002943820087	0	11	0	3	https://www.ncbi.nlm.nih.gov/clinvar/variation/94613/
NM_004006.2(DMD):c.4421G>C	concordant	unreportable	unreportable	94626	0	1	SCV000603375	Uncertain significance	ARUP Laboratories	6/30/2017	DMD	chrX	32407715	32407715	http://gnomad.b	9.49E-06	AMR	0.0002258019958	0	2	0	0	https://www.ncbi.nlm.nih.gov/clinvar/variation/94626/
NM_004006.2(DMD):c.4744G>A	concordant	unreportable	unreportable	94637	0	3	SCV000603364;S(	Likely benign;Likely benign,	ARUP Laboratories	2017-06-30;2017-1	DMD	chrX	32398728	32398728	http://gnomad.b	8.54E-05	SAS	0.001254310017	0	14	0	2	https://www.ncbi.nlm.nih.gov/clinvar/variation/94637/
NM_004006.2(DMD):c.4775T>C	concordant	unreportable	unreportable	94638	0	1	SCV000625906	Uncertain significance	Invitae	10/5/2017	DMD	chrX	32398697	32398697	http://gnomad.b	3.80E-05	AMR	0.0001130160017	0	1	0	0	https://www.ncbi.nlm.nih.gov/clinvar/variation/94638/
NM_004006.2(DMD):c.5586+9G>A	concordant	unreportable	unreportable	94674	0	4	SCV000112566;S(	Likely benign;Benign;Uncer	EGL Genetic Diagn(	2017-06-30;2017-1	DMD	chrX	32364051	32364051	http://gnomad.b	0.0004366895138	NFE	0.0006550109829	0	18	0	8	https://www.ncbi.nlm.nih.gov/clinvar/variation/94674/
NM_004006.2(DMD):c.6438+61441C>	concordant	unreportable	unreportable	94740	0	1	SCV000112635	Uncertain significance	EGL Genetic Diagn(	6/30/2017	DMD	chrX	32173592	32173592		0.0001044257533					0	4	https://www.ncbi.nlm.nih.gov/clinvar/variation/94740/
NM_004006.2(DMD):c.6471T>A	concordant	unreportable	unreportable	94742	0	1	SCV000482233	Uncertain significance	Illumina Clinical Se	10/18/2016	DMD	chrX	31986599	31986599	http://gnomad.b	0.0002088515066	EAS	0.00109203998	0	9	0	1	https://www.ncbi.nlm.nih.gov/clinvar/variation/94742/
NM_004006.2(DMD):c.7016A>G	concordant	unreportable	unreportable	94755	0	3	SCV000112650;S(	Likely benign;Likely benign,	EGL Genetic Diagn(	2017-06-30;2017-1	DMD	chrX	31893387	31893387	http://gnomad.b	0.0001044257533	NFE	1.30E-05	0	0	0	1	https://www.ncbi.nlm.nih.gov/clinvar/variation/94755/
NM_004006.2(DMD):c.7323T>C	concordant	unreportable	unreportable	94764	0	1	SCV000112659	Uncertain significance	EGL Genetic Diagn(	6/30/2017	DMD	chrX	31792296	31792296	http://gnomad.b	7.59E-05	NFE	0.0001509110007	0	8	0	1	https://www.ncbi.nlm.nih.gov/clinvar/variation/94764/
NM_004006.2(DMD):c.7542+10A>G	concordant	unreportable	unreportable	94766	0	1	SCV000112661	Uncertain significance	EGL Genetic Diagn(	6/30/2017	DMD	chrX	31792067	31792067	http://gnomad.b	9.49E-06	AMR	0.0001132800026	0	1	0	0	https://www.ncbi.nlm.nih.gov/clinvar/variation/94766/
NM_004006.2(DMD):c.7653G>A	concordant	unreportable	unreportable	94771	0	3	SCV000516843;S(	Likely benign;Benign;Likely	GeneDx;Invitae;Lal	2017-11-28;2016-0	DMD	chrX	31747755	31747755	http://gnomad.b	0.0003417570108	SAS	0.00135806005	0	24	0	12	https://www.ncbi.nlm.nih.gov/clinvar/variation/94771/
NM_004006.2(DMD):c.8255A>G	concordant	unreportable	unreportable	94797	0	2	SCV000112692;S(	Uncertain significance;Beni	EGL Genetic Diagn(	2017-06-30;2017-1	DMD	chrX	31525533	31525533	http://gnomad.b	0.0001423987545	NFE	0.0003385859891	0	4	0	6	https://www.ncbi.nlm.nih.gov/clinvar/variation/94797/
NM_004006.2(DMD):c.8852G>A	concordant	unreportable	unreportable	94817	0	2	SCV000112716;S(	Uncertain significance;Beni	EGL Genetic Diagn(	2017-06-30;2017-1	DMD	chrX	31496308	31496308	http://gnomad.b	0.0001139190036	AMR	0.0001133440019	0	2	0	0	https://www.ncbi.nlm.nih.gov/clinvar/variation/94817/
NM_004006.2(DMD):c.9804A>G	concordant	unreportable	unreportable	94861	0	3	SCV000319515;S(	Likely benign;Benign;Benig	Ambry Genetics;EG	2017-11-14;2017-0	DMD	chrX	31222081	31222081	http://gnomad.b	0.0001613852551	EAS	0.002883190056	0	16	0	6	https://www.ncbi.nlm.nih.gov/clinvar/variation/94861/
NM_017890.4(VPS13B):c.1864A>G	concordant	unreportable	unreportable	95836	0	1	SCV000470773	Uncertain significance	Illumina Clinical Se	10/18/2016	VPS13B	chr8	100160089	100160089	http://gnomad.b	4.76E-05	AFR	6.84E-05	0		1	0	https://www.ncbi.nlm.nih.gov/clinvar/variation/95836/
NM_017890.4(VPS13B):c.7997G>A	concordant	unreportable	unreportable	95870	0	1	SCV000470828	Uncertain significance	Illumina Clinical Se	10/18/2016	VPS13B	chr8	100796685	100796685	http://gnomad.b	1.12E-05	EAS	0.0002323689987	0		0	0	https://www.ncbi.nlm.nih.gov/clinvar/variation/95870/
NM_206933.2(USH2A):c.12234_12235	concordant	reportable	reportable	96665	1	0	SCV000331482	Pathogenic	EGL Genetic Diagn(	6/30/2017	USH2A	chr1	215853549	215853551	http://gnomad.b	8.02E-06	NFE	1.79E-05	0		0	0	https://www.ncbi.nlm.nih.gov/clinvar/variation/96665/
NM_000441.1(SLC26A4):c.1233C>T	concordant	unreportable	unreportable	287878	0	1	SCV000341809	Uncertain significance	EGL Genetic Diagn(	6/30/2017	SLC26A4	chr7	107330652	107330652	http://gnomad.b	0.0001144746173	AMR	0.0001788700029	0		0	0	https://www.ncbi.nlm.nih.gov/clinvar/variation/287878/
NM_000152.3(GAA):c.1075+4G>A	concordant	unreportable	unreportable	198047	0	1	SCV000231481	Uncertain significance	EGL Genetic Diagn(	6/30/2017	GAA	chr17	78082212	78082212	http://gnomad.b	8.34E-06	NFE	8.97E-06	0		0	0	https://www.ncbi.nlm.nih.gov/clinvar/variation/198047/
NM_000136.2(FANCC):c.1161C>T	concordant	unreportable	unreportable	255274	0	2	SCV000524801;S(	Likely benign;Likely benign	GeneDx;Invitae	2017-11-28;2017-0	FANCC	chr9	97873913	97873913	http://gnomad.b	8.32E-05	NFE	3.58E-05	0		0	0	https://www.ncbi.nlm.nih.gov/clinvar/variation/255274/
NM_000057.2(BLM):c.3934G>A	concordant	unreportable	unreportable	127506	0	1	SCV000623322	Uncertain significance	Invitae	10/5/2017	BLM	chr15	91354494	91354494	http://gnomad.b	1.66E-05	AFR	6.53E-05	0		0	0	https://www.ncbi.nlm.nih.gov/clinvar/variation/127506/
NM_001079802.1(FKTN):c.277A>G	concordant	unreportable	unreportable	364487	0	1	SCV000476417	Uncertain significance	Illumina Clinical Se	10/18/2016	FKTN	chr9	108363537	108363537	http://gnomad.b	1.42E-05	NFE	2.69E-05	0		0	0	https://www.ncbi.nlm.nih.gov/clinvar/variation/364487/
NM_014363.4(SACS):c.10675C>G	concordant	unreportable	unreportable	448186	0	1	SCV000614923	Uncertain significance	Athena Diagnostics	8/17/2017	SACS	chr13	23907340	23907340	http://gnomad.b	2.37E-05	NFE	9.47E-06	0		0	0	https://www.ncbi.nlm.nih.gov/clinvar/variation/448186/
NM_000016.4(ACADM):c.661G>A	concordant	unreportable	unreportable	226081	0	1	SCV000589302	Uncertain significance	GeneDx	11/28/2017	ACADM	chr1	76211552	76211552	http://gnomad.b	2.72E-06	AMR	8.93E-05	0		0	0	https://www.ncbi.nlm.nih.gov/clinvar/variation/226081/
NM_000203.3(IDUA):c.367G>A	concordant	unreportable	unreportable	196235	0	1	SCV000451771	Uncertain significance	Illumina Clinical Se	10/18/2016	IDUA	chr4	994467	994467	http://gnomad.b	0.0001027138669	SAS	0.000844869006	0		0	0	https://www.ncbi.nlm.nih.gov/clinvar/variation/196235/
NM_002485.4(NBN):c.1177T>C	concordant	unreportable	unreportable	411739	0	1	SCV000553040	Uncertain significance	Invitae	3/14/2017	NBN	chr8	90967731	90967731		1.67E-05					0	0	https://www.ncbi.nlm.nih.gov/clinvar/variation/411739/
NM_000528.3(MAN2B1):c.1838G>A	concordant	unreportable	unreportable	450771	0	1	SCV000619386	Uncertain significance	GeneDx	11/28/2017	MAN2B1	chr19	12763267	12763267	http://gnomad.b	0.0002422562508	SAS	0.001982710091	0		1	0	https://www.ncbi.nlm.nih.gov/clinvar/variation/450771/
NM_002485.4(NBN):c.1419A>G	concordant	unreportable	unreportable	132701	0	2	SCV000685719;S(	Likely benign;Likely benign	Color Genomics, In	2017-10-26;2017-1	NBN	chr8	90965898	90965898	http://gnomad.b	4.74E-05	NFE	9.10E-05	0		0	0	https://www.ncbi.nlm.nih.gov/clinvar/variation/132701/
NM_000051.3(ATM):c.5639C>T	concordant	unreportable	unreportable	135761	0	3	SCV000682276;S(	Uncertain significance;Unc(	Color Genomics, In	2017-10-26;2017-1	ATM	chr11	108175544	108175544		1.67E-05					0	0	https://www.ncbi.nlm.nih.gov/clinvar/variation/135761/
NM_000152.3(GAA):c.131G>T	concordant	unreportable	unreportable	325774	0	2	SCV000407254;S(	Uncertain significance;Unc(	Illumina Clinical Se	2016-10-18;2017-1	GAA	chr17	78078516	78078516	http://gnomad.b	0.0001528618518	NFE	0.000255032006	0		0	0	https://www.ncbi.nlm.nih.gov/clinvar/variation/325774/
NM_000478.4(ALPL):c.1329G>A	concordant	unreportable	unreportable	295551	0	1	SCV000354313	Uncertain significance	Illumina Clinical Se	10/18/2016	ALPL	chr1	21903895	21903895	http://gnomad.b	2.82E-06	SAS	3.26E-05	0		0	0	https://www.ncbi.nlm.nih.gov/clinvar/variation/295551/
NM_000051.3(ATM):c.5624G>A	concordant	unreportable	unreportable	216222	0	2	SCV000682275;S(	Uncertain significance;Unc(	Color Genomics, In	2017-10-26;2017-1	ATM	chr11	108175529	108175529	http://gnomad.b	1.67E-05	EAS	5.84E-05	0		0	0	https://www.ncbi.nlm.nih.gov/clinvar/variation/216222/
NM_000466.2(PEX1):c.3283G>A	concordant	unreportable	unreportable	502697	0	1	SCV000709537	Uncertain significance	EGL Genetic Diagn(	6/30/2017	PEX1	chr7	92120741	92120741	http://gnomad.b	0.000141445854	SAS	0.001104829949	0		0	0	https://www.ncbi.nlm.nih.gov/clinvar/variation/502697/
NM_000487.5(ARSA):c.224+8G>A	concordant	unreportable	unreportable	456717	0	1	SCV000627139	Likely benign	Invitae	10/5/2017	ARSA	chr22	51065976	51065976	http://gnomad.b	5.35E-05	EAS	0.0006110339891	0		0	0	https://www.ncbi.nlm.nih.gov/clinvar/variation/456717/
NM_001042432.1(CLN3):c.243G>A	concordant	unreportable	unreportable	377685	0	1	SCV000512633	Likely benign	GeneDx	11/28/2017	CLN3	chr16	28499963	28499963	http://gnomad.b	4.72E-05	EAS	0.0004058440099	0		0	0	https://www.ncbi.nlm.nih.gov/clinvar/variation/377685/
NM_002485.4(NBN):c.1202C>A	concordant	unreportable	unreportable	219698	0	2	SCV000569350;S(	Uncertain significance;Unc(	GeneDx;Invitae	2017-11-28;2017-0	NBN	chr8	90967706	90967706		5.58E-06					0	0	https://www.ncbi.nlm.nih.gov/clinvar/variation/219698/
NM_000155.3(GALT):c.328+7T>C	concordant	unreportable	unreportable	495676	0	1	SCV000695688	Uncertain significance	Laboratory Corpor	1/25/2018	GALT	chr9	34647571	34647571	http://gnomad.b	4.40E-05	ASJ	0.0001015230009	0		0	0	https://www.ncbi.nlm.nih.gov/clinvar/variation/495676/
NM_000159.2(GCDH):c.1209C>T	concordant	unreportable	unreportable	387159	0	1	SCV000529064	Likely benign	GeneDx	11/28/2017	GCDH	chr19	13008643	13008643	http://gnomad.b	7.87E-05	EAS	0.000173952998	0		0	0	https://www.ncbi.nlm.nih.gov/clinvar/variation/387159/
NM_000203.3(IDUA):c.814A>C	concordant	unreportable	unreportable	350226	0	1	SCV000451778	Uncertain significance	Illumina Clinical Se	10/18/2016	IDUA	chr4	995791	995791		1.11E-05					0	0	https://www.ncbi.nlm.nih.gov/clinvar/variation/350226/
NM_000023.2(SGCA):c.1035G>A	concordant	unreportable	unreportable	389132	0	1	SCV000531577	Likely benign	GeneDx	11/28/2017	SGCA	chr17	48252669	48252669	http://gnomad.b	2.80E-05	EAS	0.0002368830028	0		0	0	https://www.ncbi.nlm.nih.gov/clinvar/variation/389132/
NM_020533.2(MCOLN1):c.490G>A	concordant	unreportable	unreportable	330490	0	1	SCV000415605	Uncertain significance	Illumina Clinical Se	10/18/2016	MCOLN1	chr19	7591731	7591731	http://gnomad.b	0.0001189377432	NFE	0.0001432379941	0		0	0	https://www.ncbi.nlm.nih.gov/clinvar/variation/330490/
NM_000391.3(TPP1):c.890G>A	concordant	unreportable	unreportable	207584	0	1	SCV000243425	Uncertain significance	GeneDx	11/28/2017	TPP1	chr11	6637731	6637731		4.20E-05					0	0	https://www.ncbi.nlm.nih.gov/clinvar/variation/207584/
NM_002485.4(NBN):c.588T>C	concordant	unreportable	unreportable	186444	0	2	SCV000685799;S(	Likely benign;Likely benign	Color Genomics, In	2017-10-26;2017-1	NBN	chr8	90983515	90983515	http://gnomad.b	2.79E-06	NFE	1.80E-05	0		0	0	https://www.ncbi.nlm.nih.gov/clinvar/variation/186444/
NM_000051.3(ATM):c.7135C>G	concordant	unreportable	unreportable	186475	0	3	SCV000682389;S(	Uncertain significance;Unc(	Color Genomics, In	2017-10-26;2017-1	ATM	chr11	108199793	108199793	http://gnomad.b	3.34E-05	AMR	0.0003573769936	0		0	0	https://www.ncbi.nlm.nih.gov/clinvar/variation/186475/
NM_000051.3(ATM):c.512A>G	concordant	unreportable	unreportable	407583	0	2	SCV000564609;S(	Uncertain significance;Unc(	GeneDx;Invitae	2017-11-28;2017-0	ATM	chr11	108114695	108114695		2.78E-06					0	0	https://www.ncbi.nlm.nih.gov/clinvar/variation/407583/
NM_000098.2(CPT2):c.853G>A	concordant	unreportable	unreportable	290583	0	1	SCV000345160	Uncertain significance	EGL Genetic Diagn(	6/30/2017	CPT2	chr1	53676199	53676199	http://gnomad.b	5.32E-05	NFE	6.28E-05	0		0	0	https://www.ncbi.nlm.nih.gov/clinvar/variation/290583/
NM_000441.1(SLC26A4):c.1969A>T	concordant	unreportable	unreportable	229256	0	1	SCV000272439	Uncertain significance	Laboratory for Mol	4/26/2016	SLC26A4	chr7	107342437	107342437		8.38E-06					0	0	https://www.ncbi.nlm.nih.gov/clinvar/variation/229256/
NM_000023.2(SGCA):c.402C>T	concordant	unreportable	unreportable	285226	0	1	SCV000338162	Uncertain significance	EGL Genetic Diagn(	6/30/2017	SGCA	chr17	48245751	48245751		2.80E-06					0	0	https://www.ncbi.nlm.nih.gov/clinvar/variation/285226/
NM_000441.1(SLC26A4):c.1068C>T	concordant	unreportable	unreportable	358499	0	1	SCV000466094	Uncertain significance	Illumina Clinical Se	10/18/2016	SLC26A4	chr7	107329564	107329564		1.12E-05					0	0	https://www.ncbi.nlm.nih.gov/clinvar/variation/358499/
NM_017739.3(POMGNT1):c.107G>A	concordant	unreportable	unreportable	450727	0	2	SCV000703190;S(	Uncertain significance;Unc(	EGL Genetic Diagn(	2017-06-30;2017-1	POMGNT1	chr1	46663387	46663387	http://gnomad.b	5.60E-06	EAS	5.80E-05	0		0	0	https://www.ncbi.nlm.nih.gov/clinvar/variation/450727/
NM_000051.3(ATM):c.4304A>C	concordant	unreportable	unreportable	188194	0	3	SCV000682193;S(	Uncertain significance;Unc(	Color Genomics, In	2017-10-26;2017-1	ATM	chr11	108160396	108160396	http://gnomad.b	8.35E-06	SAS	3.27E-05	0		0	0	https://www.ncbi.nlm.nih.gov/clinvar/variation/188194/
NM_000051.3(ATM):c.728T>C	concordant	unreportable	unreportable	185337	0	1	SCV000546853	Uncertain significance	Invitae	3/14/2017	ATM	chr11	108115580	108115580		2.78E-06					0	0	https://www.ncbi.nlm.nih.gov/clinvar/variation/185337/
NM_000271.4(NPC1):c.3794A>T	concordant	unreportable	unreportable	291193	0	1	SCV000345904	Uncertain significance	EGL Genetic Diagn(	6/30/2017	NPC1	chr18	21112209	21112209	http://gnomad.b	0.0001229456637	EAS	0.0001739330037	0		0	0	https://www.ncbi.nlm.nih.gov/clinvar/variation/291193/
NM_001079802.1(FKTN):c.445G>A	concordant	unreportable	unreportable	501810	0	1	SCV000708314	Uncertain significance	EGL Genetic Diagn(	6/30/2017	FKTN	chr9	108366571	108366571	http://gnomad.b	0.0001099205027	SAS	6.50E-05	0		0	0	https://www.ncbi.nlm.nih.gov/clinvar/variation/501810/
NM_000271.4(NPC1):c.3492C>T	concordant	unreportable	unreportable	500946	0	1	SCV000707121	Uncertain significance	EGL Genetic Diagn(	6/30/2017	NPC1	chr18	21114509	21114509	http://gnomad.b	3.72E-05	FIN	0.0003616639879	0		0	0	https://www.ncbi.nlm.nih.gov/clinvar/variation/500946/
NM_000018.3(ACADVL):c.1103A>C	concordant	unreportable	unreportable	439360	1	2	SCV000602369;S(	Likely pathogenic;Uncertai	ARUP Laboratories	2017-06-30;2017-0	ACADVL	chr17	7126477	7126477	http://gnomad.b	5.66E-06	NFE	8.95E-06	0		0	0	https://www.ncbi.nlm.nih.gov/clinvar/variation/439360/
NM_000051.3(ATM):c.2476A>C	concordant	unreportable	unreportable	142345	0	2	SCV000566330;S(	Uncertain significance;Unc(	GeneDx;Invitae	2017-11-28;2017-1	ATM	chr11	108137907	108137907	http://gnomad.b	4.46E-05	NFE	9.85E-05	0		0	0	https://www.ncbi.nlm.nih.gov/clinvar/variation/142345/
NM_000136.2(FANCC):c.9A>T	concordant	unreportable	unreportable	449791	0	1	SCV000618194	Uncertain significance	GeneDx	11/28/2017	FANCC	chr9	98011565	98011565	http://gnomad.b	2.38E-05	NFE	4.51E-05	0		0	0	https://www.ncbi.nlm.nih.gov/clinvar/variation/449791/
NM_000642.2(AGL):c.4436T>C	concordant	unreportable	unreportable	456507	0	1	SCV000626758	Uncertain significance	Invitae	10/5/2017	AGL	chr1	100382242	100382242	http://gnomad.b	2.51E-05	NFE	2.69E-05	0		0	0	https://www.ncbi.nlm.nih.gov/clinvar/variation/456507/
NM_000051.3(ATM):c.8360C>T	concordant	unreportable	unreportable	243110	0	2	SCV000292485;S(	Uncertain significance;Unc(	GeneDx;Invitae	2017-11-28;2017-0	ATM	chr11	108214040	108214040	http://gnomad.b	2.78E-06	NFE	8.97E-06	0		0	0	https://www.ncbi.nlm.nih.gov/clinvar/variation/243110/
NM_001042432.1(CLN3):c.987C>T	concordant	unreportable	unreportable	415537	0	1	SCV000560162	Likely benign	Invitae	3/14/2017	CLN3	chr16	28493495	28493495	http://gnomad.b	2.78E-05	AFR	6.64E-05	0		0	0	https://www.ncbi.nlm.nih.gov/clinvar/variation/415537/
NM_001079802.1(FKTN):c.929A>G	concordant	unreportable	unreportable	238269	0	1	SCV000285822	Uncertain significance	Invitae	10/5/2017	FKTN	chr9	108380258	108380258	http://gnomad.b	7.45E-05	FIN	0.0001347100042	0		0	0	https://www.ncbi.nlm.nih.gov/clinvar/variation/238269/
NM_000271.4(NPC1):c.2196dupT	concordant	reportable	reportable	92706	1	0	SCV000596046	Pathogenic	Genetic Services La	7/5/2017	NPC1	chr18	21123467	21123467	http://gnomad.b	2.86E-05	FIN	4.52E-05	0		0	0	https://www.ncbi.nlm.nih.gov/clinvar/variation/92706/
NM_004543.4(NEB):c.8152A>T	concordant	unreportable	unreportable	331488	0	1	SCV000416960	Uncertain significance	Illumina Clinical Se	10/18/2016	NEB	chr2	152499672	152499672	http://gnomad.b	3.36E-05	NFE	1.80E-05	0		0	0	https://www.ncbi.nlm.nih.gov/clinvar/variation/331488/
NM_017739.3(POMGNT1):c.1014C>T	concordant	unreportable	unreportable	471398	0	1	SCV000649950	Likely benign	Invitae	10/5/2017	POMGNT1	chr1	46659248	46659248	http://gnomad.b	5.60E-06	SAS	6.50E-05	0		0	0	https://www.ncbi.nlm.nih.gov/clinvar/variation/471398/
NM_000018.3(ACADVL):c.109C>T	concordant	unreportable	unreportable	324982	0	1	SCV000406305	Uncertain significance	Illumina Clinical Se	10/18/2016	ACADVL	chr17	7123487	7123487		2.55E-05					0	0	https://www.ncbi.nlm.nih.gov/clinvar/variation/324982/
NM_006493.2(CLN5):c.127G>A	concordant	unreportable	unreportable	205139	0	1	SCV000240639	Uncertain significance	GeneDx	11/28/2017	CLN5	chr13	77566213	77566213	http://gnomad.b	6.43E-05	NFE	5.00E-05	0		0	0	https://www.ncbi.nlm.nih.gov/clinvar/variation/205139/
NM_138694.3(PKHD1):c.10651G>A	concordant	unreportable	unreportable	501942	0	1	SCV000708476	Uncertain significance	EGL Genetic Diagn(	6/30/2017	PKHD1	chr6	51524273	51524273	http://gnomad.b	3.61E-05	AMR	0.0003591309942	0		0	0	https://www.ncbi.nlm.nih.gov/clinvar/variation/501942/
NM_000018.3(ACADVL):c.1569A>G	concordant	unreportable	unreportable	388745	0	1	SCV000531106	Likely benign	GeneDx	11/28/2017	ACADVL	chr17	7127676	7127676	http://gnomad.b	2.83E-06	EAS	0.0001159820022	0		0	0	https://www.ncbi.nlm.nih.gov/clinvar/variation/388745/
NM_000051.3(ATM):c.79G>A	concordant	unreportable	unreportable	185887	0	2	SCV000294083;S(	Uncertain significance;Unc(	GeneDx;Invitae	2017-11-28;2017-0	ATM	chr11	108098509	108098509	http://gnomad.b	1.95E-05	NFE	8.96E-06	0		0	0	https://www.ncbi.nlm.nih.gov/clinvar/variation/185887/
NM_000051.3(ATM):c.7370A>C	concordant	unreportable	unreportable	231363	0	2	SCV000687768;S(	Uncertain significance;Unc(	Color Genomics, In	2017-12-21;2017-1	ATM	chr11	108201003	108201003	http://gnomad.b	3.34E-05	SAS	0.0003573519934	0		0	0	https://www.ncbi.nlm.nih.gov/clinvar/variation/231363/
NM_002485.4(NBN):c.37+1G>A	concordant	reportable	reportable	186314	2	0	SCV000537621;S(	Likely pathogenic;Likely pat	Color Genomics, In	2017-01-26;2017-0	NBN	chr8	90996752	90996752	http://gnomad.b	1.12E-05	AFR	6.83E-05	0		0	0	https://www.ncbi.nlm.nih.gov/clinvar/variation/186314/
NM_000232.4(SGCB):c.282A>G	concordant	unreportable	unreportable	499302	0	1	SCV000704733	Uncertain significance	EGL Genetic Diagn(	6/30/2017	SGCB	chr4	52895991	52895991	http://gnomad.b	4.48E-05	EAS	0.0002319110063	0		0	0	https://www.ncbi.nlm.nih.gov/clinvar/variation/499302/
NM_000051.3(ATM):c.7354C>G	concordant	unreportable	unreportable	127442	0	3	SCV000682407;S(	Uncertain significance;Unc(	Color Genomics, In	2017-10-26;2017-1	ATM	chr11	108200987	108200987	http://gnomad.b	5.57E-06	NFE	1.79E-05	0		0	0	https://www.ncbi.nlm.nih.gov/clinvar/variation/127442/
NM_006493.2(CLN5):c.562T>C	concordant	unreportable	unreportable	196438	0	1	SCV000240652	Uncertain significance	GeneDx	11/28/2017	CLN5	chr13	77570112	77570112		8.39E-06					0	0	https://www.ncbi.nlm.nih.gov/clinvar/variation/196438/
NM_138694.3(PKHD1):c.8887G>A	concordant	unreportable	unreportable	357421	0	2	SCV000706839;S(	Uncertain significance;Unc(	EGL Genetic Diagn(	2017-06-30;2016-1	PKHD1	chr6	51618062	51618062	http://gnomad.b	6.39E-05	AMR	0.0002385499974	0		1	0	https://www.ncbi.nlm.nih.gov/clinvar/variation/357421/
NM_001042432.1(CLN3):c.461-3C>G	concordant	unreportable	unreportable	422288	0	1	SCV000571714	Uncertain significance	GeneDx	11/28/2017	CLN3	chr16	28497974	28497974	http://gnomad.b	5.56E-06	NFE	9.76E-06	0		0	0	https://www.ncbi.nlm.nih.gov/clinvar/variation/422288/
NM_000060.2(BTD):c.1485C>A	concordant	unreportable	unreportable	343913	0	1	SCV000441835	Uncertain significance	Illumina Clinical Se	10/18/2016	BTD	chr3	15686848	15686848	http://gnomad.b	3.46E-05	EAS	0.0001739330037	0		0	0	https://www.ncbi.nlm.nih.gov/clinvar/variation/343913/
NM_003640.3(IKBKAP):c.2799G>A	concordant	unreportable	unreportable	364565	0	1	SCV000476559	Uncertain significance	Illumina Clinical Se	10/18/2016	IKBKAP	chr9	111656284	111656284	http://gnomad.b	9.46E-05	EAS	0.002437039977	0		0	0	https://www.ncbi.nlm.nih.gov/clinvar/variation/364565/
NM_000051.3(ATM):c.8629T>C	concordant	unreportable	unreportable	181844	0	2	SCV000682506;S(	Likely benign;Likely benign	Color Genomics, In	2017-10-26;2017-1	ATM	chr11	108218050	108218050	http://gnomad.b	3.62E-05	AMR	0.0002983830054	0		0	0	https://www.ncbi.nlm.nih.gov/clinvar/variation/181844/
NM_000152.3(GAA):c.1886C>T	concordant	unreportable	unreportable	290659	0	1	SCV000345254	Uncertain significance	EGL Genetic Diagn(	6/30/2017	GAA	chr17	78086508	78086508	http://gnomad.b	1.95E-05	EAS	6.15E-05	0		0	0	https://www.ncbi.nlm.nih.gov/clinvar/variation/290659/
NM_017890.4(VPS13B):c.7163A>G	concordant	unreportable	unreportable	361072	0	1	SCV000470822	Uncertain significance	Illumina Clinical Se	10/18/2016	VPS13B	chr8	100779039	100779039	http://gnomad.b	4.76E-05	EAS	0.0002325849928	0		0	0	https://www.ncbi.nlm.nih.gov/clinvar/variation/361072/
NM_000642.2(AGL):c.1076C>T	concordant	unreportable	unreportable	456449	0	1	SCV000626662	Uncertain significance	Invitae	10/5/2017	AGL	chr1	100340360	100340360	http://gnomad.b	5.02E-05	AMR	0.000119160999	0		0	0	https://www.ncbi.nlm.nih.gov/clinvar/variation/456449/
NM_002485.4(NBN):c.1180A>G	concordant	unreportable	unreportable	411759	0	1	SCV000553074	Uncertain significance	Invitae	3/14/2017	NBN	chr8	90967728	90967728		2.79E-06					0	0	https://www.ncbi.nlm.nih.gov/clinvar/variation/411759/
NM_000051.3(ATM):c.5764C>T	concordant	unreportable	unreportable	141596	0	2	SCV000569917;S(	Uncertain significance;Unc(	GeneDx;Invitae	2017-11-28;2017-0	ATM	chr11	108180888	108180888	http://gnomad.b	5.57E-06	NFE	3.59E-05	0		0	0	https://www.ncbi.nlm.nih.gov/clinvar/variation/141596/
NM_000492.3(CFTR):c.3969G>A	concordant	unreportable	unreportable	358735	0	1	SCV000466526	Uncertain significance	Illumina Clinical Se	10/18/2016	CFTR	chr7	117304747	117304747	http://gnomad.b	1.16E-05	NFE	8.98E-06	0		0	0	https://www.ncbi.nlm.nih.gov/clinvar/variation/358735/
NM_000352.3(ABCC8):c.4239G>T	concordant	unreportable	unreportable	303751	0	1	SCV000369239	Uncertain significance	Illumina Clinical Se	10/18/2016	ABCC8	chr11	17417225	17417225	http://gnomad.b	6.97E-05	NFE	0.0001223800064	0		0	0	https://www.ncbi.nlm.nih.gov/clinvar/variation/303751/
NM_017890.4(VPS13B):c.9668G>A	concordant	unreportable	unreportable	212585	0	1	SCV000249429	Uncertain significance	Genetic Services La	9/15/2015	VPS13B	chr8	100844859	100844859	http://gnomad.b	3.36E-05	AFR	6.56E-05	0		0	0	https://www.ncbi.nlm.nih.gov/clinvar/variation/212585/
NM_000051.3(ATM):c.5345A>G	concordant	unreportable	unreportable	236736	0	1	SCV000282989	Uncertain significance	Invitae	6/10/2016	ATM	chr11	108173605	108173605		8.35E-06					0	0	https://www.ncbi.nlm.nih.gov/clinvar/variation/236736/
NM_000051.3(ATM):c.2735A>G	concordant	unreportable	unreportable	181936	0	2	SCV000209712;S(	Uncertain significance;Unc(	GeneDx;Invitae	2017-02-15;2017-1	ATM	chr11	108139233	108139233		8.35E-06					0	0	https://www.ncbi.nlm.nih.gov/clinvar/variation/181936/
NM_001042432.1(CLN3):c.32T>C	concordant	unreportable	unreportable	424077	0	1	SCV000573854	Uncertain significance	GeneDx	11/28/2017	CLN3	chr16	28503049	28503049	http://gnomad.b	1.67E-05	AFR	6.59E-05	0		0	0	https://www.ncbi.nlm.nih.gov/clinvar/variation/424077/
NM_000288.3(PEX7):c.576C>T	concordant	unreportable	unreportable	198066	0	1	SCV000460539	Uncertain significance	Illumina Clinical Se	10/18/2016	PEX7	chr6	137187814	137187814	http://gnomad.b	0.0001111148149	AMR	0.0002084570006	0		0	0	https://www.ncbi.nlm.nih.gov/clinvar/variation/198066/
NM_002485.4(NBN):c.775G>A	concordant	unreportable	unreportable	182716	0	2	SCV000211443;S(	Uncertain significance;Unc(	GeneDx;Invitae	2017-11-28;2017-1	NBN	chr8	90982713	90982713	http://gnomad.b	2.79E-05	NFE	8.96E-06	0		0	0	https://www.ncbi.nlm.nih.gov/clinvar/variation/182716/
NM_000027.3(AGA):c.571G>C	concordant	unreportable	unreportable	348235	0	1	SCV000448735	Uncertain significance	Illumina Clinical Se	10/18/2016	AGA	chr4	178358610	178358610	http://gnomad.b	1.78E-05	EAS	5.80E-05	0		0	0	https://www.ncbi.nlm.nih.gov/clinvar/variation/348235/
NM_002485.4(NBN):c.119C>T	concordant	unreportable	unreportable	141146	0	4	SCV000685700;S(	Uncertain significance;Unc(	Color Genomics, In	2017-10-26;2017-1	NBN	chr8	90995002	90995002	http://gnomad.b	5.58E-06	NFE	2.69E-05	0		0	0	https://www.ncbi.nlm.nih.gov/clinvar/variation/141146/
NM_000228.2(LAMB3):c.1016A>G	concordant	unreportable	unreportable	295117	0	1	SCV000353644	Uncertain significance	Illumina Clinical Se	10/18/2016	LAMB3	chr1	209803198	209803198	http://gnomad.b	1.68E-05	EAS	0.0001739330037	0		0	0	https://www.ncbi.nlm.nih.gov/clinvar/variation/295117/
NM_000051.3(ATM):c.1020C>A	concordant	unreportable	unreportable	135730	0	3	SCV000681945;S(	Likely benign;Likely benign,	Color Genomics, In	2017-10-26;2017-1	ATM	chr11	108117809	108117809	http://gnomad.b	0.0001865754767	SAS	0.001461889944	2		0	0	https://www.ncbi.nlm.nih.gov/clinvar/variation/135730/

Supplemental Table 2

NM_000053.3(ATP7B):c.3221C>T	concordant	unreportable	unreportable	495413	0	1	SCV000694442	Uncertain significance	Laboratory Corpora	1/25/2018	ATP7B	chr13	52518267	52518267	http://gnomad.b	8.42E-06	NFE	8.96E-06	0		0	0	https://www.ncbi.nlm.nih.gov/clinvar/variation/495413/
NM_000051.3(ATM):c.9006C>T	concordant	unreportable	unreportable	183808	0	2	SCV000682530;S	Benign;Likely benign	Color Genomics, In	2017-10-26;2017-1	ATM	chr11	108236070	108236070	http://gnomad.b	0.0001141730529	SAS	0.002306540031	1		0	0	https://www.ncbi.nlm.nih.gov/clinvar/variation/183808/
NM_000051.3(ATM):c.2606C>T	concordant	unreportable	unreportable	186608	0	2	SCV000564619;S	Uncertain significance;Unc	GeneDx;Invitae	2017-11-28;2017-1	ATM	chr11	108138037	108138037	http://gnomad.b	1.67E-05	NFE	2.69E-05	0		0	0	https://www.ncbi.nlm.nih.gov/clinvar/variation/186608/
NM_000051.3(ATM):c.2578G>A	concordant	unreportable	unreportable	127354	0	2	SCV000682063;S	Uncertain significance;Unc	Color Genomics, In	2017-10-26;2017-1	ATM	chr11	108138009	108138009	http://gnomad.b	5.57E-06	EAS	0.000290091004	0		0	0	https://www.ncbi.nlm.nih.gov/clinvar/variation/127354/
NM_000528.3(MAN2B1):c.2865G>A	concordant	unreportable	unreportable	328253	0	1	SCV000410773	Uncertain significance	Illumina Clinical Se	10/18/2016	MAN2B1	chr19	12758105	12758105	http://gnomad.b	2.54E-05	NFE	3.58E-05	0		0	0	https://www.ncbi.nlm.nih.gov/clinvar/variation/328253/
NM_000243.2(MEFV):c.663G>C	concordant	unreportable	unreportable	97535	0	1	SCV000629049	Likely benign	Invitae	10/5/2017	MEFV	chr16	3304405	3304405	http://gnomad.b	8.40E-05	AMR	0.0001790620008	0		0	0	https://www.ncbi.nlm.nih.gov/clinvar/variation/97535/
NM_000391.3(TPP1):c.118C>T	concordant	unreportable	unreportable	196263	0	1	SCV000535836	Uncertain significance	GeneDx	11/28/2017	TPP1	chr11	6640118	6640118	http://gnomad.b	1.12E-05	NFE	8.96E-06	0		0	0	https://www.ncbi.nlm.nih.gov/clinvar/variation/196263/
NM_000112.3(SLC26A2):c.844G>C	concordant	unreportable	unreportable	284353	0	1	SCV000336943	Uncertain significance	EGL Genetic Diagn	6/30/2017	SLC26A2	chr5	149360000	149360000	http://gnomad.b	2.23E-05	EAS	0.000289921998	0		0	0	https://www.ncbi.nlm.nih.gov/clinvar/variation/284353/
NM_000051.3(ATM):c.3059C>T	concordant	unreportable	unreportable	220080	0	4	SCV000682099;S	Uncertain significance;Unc	Color Genomics, In	2017-10-26;2017-1	ATM	chr11	108142115	108142115	http://gnomad.b	5.57E-06	EAS	0.0001160090033	0		0	0	https://www.ncbi.nlm.nih.gov/clinvar/variation/220080/
NM_000051.3(ATM):c.2942G>A	concordant	unreportable	unreportable	230737	0	4	SCV000687435;S	Uncertain significance;Unc	Color Genomics, In	2017-12-21;2017-1	ATM	chr11	108141998	108141998	http://gnomad.b	3.34E-05	SAS	0.0001303269964	0		1	0	https://www.ncbi.nlm.nih.gov/clinvar/variation/230737/
NM_000441.1(SLC26A4):c.2227T>G	concordant	unreportable	unreportable	501589	0	1	SCV000708013	Uncertain significance	EGL Genetic Diagn	6/30/2017	SLC26A4	chr7	107350636	107350636	http://gnomad.b	3.91E-05	AFR	0.0001307019993	0		0	0	https://www.ncbi.nlm.nih.gov/clinvar/variation/501589/
NM_000051.3(ATM):c.1295T>A	concordant	unreportable	unreportable	423251	0	2	SCV000681964;S	Uncertain significance;Unc	Color Genomics, In	2017-10-26;2017-1	ATM	chr11	108121487	108121487	http://gnomad.b	3.90E-05	SAS	6.50E-05	0		0	0	https://www.ncbi.nlm.nih.gov/clinvar/variation/423251/
NM_000152.3(GAA):c.702G>A	concordant	unreportable	unreportable	288515	0	1	SCV000342643	Uncertain significance	EGL Genetic Diagn	6/30/2017	GAA	chr17	78081365	78081365	http://gnomad.b	5.00E-05	EAS	0.0004062679945	0		0	0	https://www.ncbi.nlm.nih.gov/clinvar/variation/288515/
NM_024649.4(BBS1):c.447C>T	concordant	unreportable	unreportable	305460	0	1	SCV000373263	Uncertain significance	Illumina Clinical Se	10/18/2016	BBS1	chr11	66283025	66283025	http://gnomad.b	2.79E-05	NFE	1.79E-05	0		0	0	https://www.ncbi.nlm.nih.gov/clinvar/variation/305460/
NM_017739.3(POMGNT1):c.251G>T	concordant	unreportable	unreportable	471405	0	1	SCV000649965	Uncertain significance	Invitae	10/5/2017	POMGNT1	chr1	46662506	46662506	http://gnomad.b	2.24E-05	EAS	5.80E-05	0		0	0	https://www.ncbi.nlm.nih.gov/clinvar/variation/471405/
NM_000391.3(TPP1):c.318C>T	concordant	unreportable	unreportable	378755	0	1	SCV000514957	Likely benign	GeneDx	11/28/2017	TPP1	chr11	6638919	6638919	http://gnomad.b	9.23E-05	AMR	0.0008040019893	0		0	0	https://www.ncbi.nlm.nih.gov/clinvar/variation/378755/
NM_000051.3(ATM):c.4307A>G	concordant	unreportable	unreportable	453512	0	1	SCV000622488	Uncertain significance	Invitae	10/5/2017	ATM	chr11	108160399	108160399	http://gnomad.b	5.57E-06	AFR	6.55E-05	0		0	0	https://www.ncbi.nlm.nih.gov/clinvar/variation/453512/
NM_000051.3(ATM):c.4779A>G	concordant	unreportable	unreportable	133621	0	1	SCV000682220	Likely benign	Color Genomics, In	10/26/2017	ATM	chr11	108165656	108165656	http://gnomad.b	1.39E-05	NFE	1.79E-05	0		0	0	https://www.ncbi.nlm.nih.gov/clinvar/variation/133621/
NM_002485.4(NBN):c.1843T>C	concordant	unreportable	unreportable	411782	0	2	SCV000569009;S	Uncertain significance;Unc	GeneDx;Invitae	2017-11-28;2017-1	NBN	chr8	90965474	90965474	http://gnomad.b	1.39E-05	NFE	4.49E-05	0		0	0	https://www.ncbi.nlm.nih.gov/clinvar/variation/411782/
NM_000051.3(ATM):c.121A>G	concordant	unreportable	unreportable	407674	0	1	SCV000547056	Uncertain significance	Invitae	3/14/2017	ATM	chr11	108098551	108098551		5.57E-06					0	0	https://www.ncbi.nlm.nih.gov/clinvar/variation/407674/
NM_138694.3(PKHD1):c.11818A>T	concordant	unreportable	unreportable	290109	0	1	SCV000344606	Uncertain significance	EGL Genetic Diagn	6/30/2017	PKHD1	chr6	51484286	51484286	http://gnomad.b	7.51E-05	EAS	0.001043959986	0		0	0	https://www.ncbi.nlm.nih.gov/clinvar/variation/290109/
NM_000051.3(ATM):c.986G>A	concordant	unreportable	unreportable	236798	0	1	SCV000283107	Uncertain significance	Invitae	6/10/2016	ATM	chr11	108117775	108117775	http://gnomad.b	2.78E-05	SAS	0.0004872980062	0		0	0	https://www.ncbi.nlm.nih.gov/clinvar/variation/236798/
NM_001164277.1(SLC37A4):c.742C>T	concordant	reportable	reportable	188762	2	0	SCV000330663;S	Pathogenic;Pathogenic	GeneDx;Laborator	2017-11-28;2018-0	SLC37A4	chr11	118897689	118897689	http://gnomad.b	2.79E-05	AMR	8.94E-05	0		0	0	https://www.ncbi.nlm.nih.gov/clinvar/variation/188762/
NM_004004.5(GJB2):c.587T>C	concordant	unreportable	unreportable	447447	0	1	SCV000613519	Uncertain significance	Athena Diagnostics	8/17/2017	GJB2	chr13	20763134	20763134	http://gnomad.b	3.63E-05	EAS	0.000115969	0		0	0	https://www.ncbi.nlm.nih.gov/clinvar/variation/447447/
NM_000071.2(CBS):c.1208C>T	concordant	unreportable	unreportable	282049	0	1	SCV000333222	Uncertain significance	EGL Genetic Diagn	6/30/2017	CBS	chr21	44479351	44479351	http://gnomad.b	1.12E-05	SAS	3.25E-05	0		0	0	https://www.ncbi.nlm.nih.gov/clinvar/variation/282049/
NM_000492.3(CFTR):c.66A>G	concordant	unreportable	unreportable	416596	0	1	SCV000562312	Likely benign	Invitae	3/14/2017	CFTR	chr7	117144319	117144319	http://gnomad.b	9.63E-06	AFR	0.0001961039961	0		0	0	https://www.ncbi.nlm.nih.gov/clinvar/variation/416596/
NM_000518.4(HBB):c.*56A>G	concordant	unreportable	unreportable	495968	0	1	SCV000697067	Uncertain significance	Laboratory Corpora	1/25/2018	HBB	chr11	5246772	5246772		0.0001376279473					0	0	https://www.ncbi.nlm.nih.gov/clinvar/variation/495968/
NM_017890.4(VPS13B):c.8826G>A	concordant	unreportable	unreportable	285278	0	2	SCV000338228;S	Uncertain significance;Likel	EGL Genetic Diagn	2017-06-30;2017-0	VPS13B	chr8	100831769	100831769	http://gnomad.b	8.40E-05	EAS	0.0006980799953	0		0	0	https://www.ncbi.nlm.nih.gov/clinvar/variation/285278/
NM_000051.3(ATM):c.4358T>C	concordant	unreportable	unreportable	422614	0	1	SCV000572123	Uncertain significance	GeneDx	11/28/2017	ATM	chr11	108160450	108160450		5.57E-06					0	0	https://www.ncbi.nlm.nih.gov/clinvar/variation/422614/
NM_004543.4(NEB):c.19460G>A	concordant	unreportable	unreportable	331405	0	1	SCV000416814	Uncertain significance	Illumina Clinical Se	10/18/2016	NEB	chr2	152348283	152348283		2.32E-05					0	0	https://www.ncbi.nlm.nih.gov/clinvar/variation/331405/
NM_000050.4(ASS1):c.846C>T	concordant	unreportable	unreportable	365258	0	1	SCV000477630	Uncertain significance	Illumina Clinical Se	10/18/2016	ASS1	chr9	133364727	133364727	http://gnomad.b	3.34E-05	EAS	0.0002319110063	0		0	0	https://www.ncbi.nlm.nih.gov/clinvar/variation/365258/
NM_000478.4(ALPL):c.1131G>A	concordant	unreportable	unreportable	295549	0	1	SCV000354311	Uncertain significance	Illumina Clinical Se	10/18/2016	ALPL	chr1	21902359	21902359	http://gnomad.b	3.10E-05	ASJ	0.0001015230009	0		0	0	https://www.ncbi.nlm.nih.gov/clinvar/variation/295549/
NM_000492.3(CFTR):c.1718C>T	concordant	unreportable	unreportable	502512	0	1	SCV000709286	Uncertain significance	EGL Genetic Diagn	6/30/2017	CFTR	chr7	117230445	117230445	http://gnomad.b	3.47E-05	NFE	3.60E-05	0		0	0	https://www.ncbi.nlm.nih.gov/clinvar/variation/502512/
NM_000152.3(GAA):c.2510G>A	concordant	unreportable	unreportable	284232	0	1	SCV000336763	Uncertain significance	EGL Genetic Diagn	6/30/2017	GAA	chr17	78092020	78092020	http://gnomad.b	2.22E-05	AFR	0.0001964380062	0		0	0	https://www.ncbi.nlm.nih.gov/clinvar/variation/284232/
NM_017890.4(VPS13B):c.7123C>T	concordant	reportable	reportable	504191	1	0	SCV000710501	Likely pathogenic	GeneDx	3/26/2018	VPS13B	chr8	100733273	100733273	http://gnomad.b	2.80E-06	AMR	2.98E-05	0		0	0	https://www.ncbi.nlm.nih.gov/clinvar/variation/504191/
NM_000051.3(ATM):c.6966C>T	concordant	unreportable	unreportable	220592	0	1	SCV000261258	Likely benign	Invitae	1/6/2016	ATM	chr11	108196943	108196943		5.57E-06					0	0	https://www.ncbi.nlm.nih.gov/clinvar/variation/220592/
NM_000492.3(CFTR):c.2629T>G	concordant	unreportable	unreportable	455771	0	1	SCV000625739	Uncertain significance	Invitae	10/5/2017	CFTR	chr7	117242889	117242889	http://gnomad.b	2.31E-05	SAS	6.50E-05	0		0	0	https://www.ncbi.nlm.nih.gov/clinvar/variation/455771/
NM_000271.4(NPC1):c.2510T>C	concordant	unreportable	unreportable	498832	0	1	SCV000704044	Uncertain significance	EGL Genetic Diagn	6/30/2017	NPC1	chr18	21121036	21121036	http://gnomad.b	5.15E-05	NFE	7.16E-05	0		0	0	https://www.ncbi.nlm.nih.gov/clinvar/variation/498832/
NM_000152.3(GAA):c.69C>T	concordant	unreportable	unreportable	456431	0	1	SCV000626630	Likely benign	Invitae	10/5/2017	GAA	chr17	78078454	78078454	http://gnomad.b	4.45E-05	EAS	0.0001740750013	0		0	0	https://www.ncbi.nlm.nih.gov/clinvar/variation/456431/
NM_017890.4(VPS13B):c.7987T>C	concordant	unreportable	unreportable	448879	0	1	SCV000616276	Uncertain significance	Athena Diagnostics	8/17/2017	VPS13B	chr8	100796675	100796675	http://gnomad.b	8.40E-06	NFE	2.70E-05	0		0	0	https://www.ncbi.nlm.nih.gov/clinvar/variation/448879/
NM_000271.4(NPC1):c.916G>A	concordant	unreportable	unreportable	502701	0	1	SCV000709544	Uncertain significance	EGL Genetic Diagn	6/30/2017	NPC1	chr18	21137120	21137120		2.00E-05					0	0	https://www.ncbi.nlm.nih.gov/clinvar/variation/502701/
NM_000152.3(GAA):c.2052G>A	concordant	unreportable	unreportable	387758	0	3	SCV000702586;S	Uncertain significance;Likel	EGL Genetic Diagn	2017-06-30;2017-1	GAA	chr17	78087028	78087028		0.0001167308686					0	0	https://www.ncbi.nlm.nih.gov/clinvar/variation/387758/
NM_000051.3(ATM):c.2638+3A>G	concordant	unreportable	unreportable	233657	0	2	SCV000517875;S	Likely benign;Uncertain sig	GeneDx;Invitae	2017-11-28;2017-1	ATM	chr11	108138072	108138072	http://gnomad.b	5.57E-06	EAS	0.0001160359971	0		0	0	https://www.ncbi.nlm.nih.gov/clinvar/variation/233657/
NM_000051.3(ATM):c.3751T>C	concordant	unreportable	unreportable	453491	0	1	SCV000622453	Uncertain significance	Invitae	10/5/2017	ATM	chr11	108154958	108154958	http://gnomad.b	8.35E-06	SAS	3.30E-05	0		0	0	https://www.ncbi.nlm.nih.gov/clinvar/variation/453491/
NM_000051.3(ATM):c.6313A>G	concordant	unreportable	unreportable	245884	0	2	SCV000293063;S	Uncertain significance;Unc	GeneDx;Invitae	2017-11-28;2017-0	ATM	chr11	108188214	108188214		8.35E-06					0	0	https://www.ncbi.nlm.nih.gov/clinvar/variation/245884/
NM_000391.3(TPP1):c.645C>T	concordant	unreportable	unreportable	384149	0	1	SCV000524852	Likely benign	GeneDx	11/28/2017	TPP1	chr11	6638248	6638248	http://gnomad.b	4.20E-05	AFR	0.0001307189959	0		0	0	https://www.ncbi.nlm.nih.gov/clinvar/variation/384149/
NM_017739.3(POMGNT1):c.1077T>C	concordant	unreportable	unreportable	281837	0	1	SCV000332845	Uncertain significance	EGL Genetic Diagn	6/30/2017	POMGNT1	chr1	46659010	46659010	http://gnomad.b	5.60E-06	SAS	3.27E-05	0		0	0	https://www.ncbi.nlm.nih.gov/clinvar/variation/281837/
NM_000053.3(ATP7B):c.2484C>T	concordant	unreportable	unreportable	254765	0	1	SCV000702264	Likely benign	EGL Genetic Diagn	6/30/2017	ATP7B	chr13	52524499	52524499	http://gnomad.b	0.0001767716447	SAS	0.0026638899	0		0	0	https://www.ncbi.nlm.nih.gov/clinvar/variation/254765/
NM_000053.3(ATP7B):c.1145C>G	concordant	unreportable	unreportable	426499	0	1	SCV000576955	Uncertain significance	GeneDx	11/28/2017	ATP7B	chr13	52548211	52548211	http://gnomad.b	6.17E-05	NFE	9.85E-05	0		0	0	https://www.ncbi.nlm.nih.gov/clinvar/variation/426499/
NM_000018.3(ACADVL):c.969G>A	concordant	unreportable	unreportable	324990	0	1	SCV000406319	Uncertain significance	Illumina Clinical Se	10/18/2016	ACADVL	chr17	7126076	7126076	http://gnomad.b	8.50E-06	SAS	6.50E-05	0		0	0	https://www.ncbi.nlm.nih.gov/clinvar/variation/324990/
NM_000051.3(ATM):c.8987+3G>A	concordant	unreportable	unreportable	184495	0	3	SCV000340486;S	Uncertain significance;Likel	EGL Genetic Diagn	2017-06-30;2017-1	ATM	chr11	108235948	108235948	http://gnomad.b	0.0001002495099	EAS	0.001565580023	0		0	0	https://www.ncbi.nlm.nih.gov/clinvar/variation/184495/
NM_000051.3(ATM):c.4759C>G	concordant	unreportable	unreportable	185808	0	1	SCV000622527	Uncertain significance	Invitae	10/5/2017	ATM	chr11	108164187	108164187		1.11E-05					0	0	https://www.ncbi.nlm.nih.gov/clinvar/variation/185808/
NM_000152.3(GAA):c.1265G>A	concordant	unreportable	unreportable	284246	0	2	SCV000336788;S	Uncertain significance;Likel	EGL Genetic Diagn	2017-06-30;2017-1	GAA	chr17	78082566	78082566	http://gnomad.b	0.0001139515622	EAS	0.00322310999	0		0	0	https://www.ncbi.nlm.nih.gov/clinvar/variation/284246/
NM_000051.3(ATM):c.8187A>C	concordant	unreportable	unreportable	141700	0	1	SCV000622806	Uncertain significance	Invitae	10/5/2017	ATM	chr11	108206607	108206607	http://gnomad.b	8.35E-06	NFE	3.59E-05	0		0	0	https://www.ncbi.nlm.nih.gov/clinvar/variation/141700/
NM_000051.3(ATM):c.6681C>T	concordant	unreportable	unreportable	231520	0	2	SCV000682358;S	Likely benign;Likely benign	Color Genomics, In	2017-10-26;2017-1	ATM	chr11	108196145	108196145	http://gnomad.b	4.18E-05	SAS	0.0001624330034	0		0	0	https://www.ncbi.nlm.nih.gov/clinvar/variation/231520/
NM_000243.2(MEFV):c.1527C>T	concordant	unreportable	unreportable	319110	0	1	SCV000396765	Uncertain significance	Illumina Clinical Se	10/18/2016	MEFV	chr16	3297076	3297076	http://gnomad.b	8.40E-06	SAS	3.25E-05	0		0	0	https://www.ncbi.nlm.nih.gov/clinvar/variation/319110/
NM_002485.4(NBN):c.1471G>A	concordant	unreportable	unreportable	419397	0	2	SCV000567168;S	Uncertain significance;Unc	GeneDx;Invitae	2017-11-28;2017-1	NBN	chr8	90965846	90965846	http://gnomad.b	8.37E-06	AFR	6.55E-05	0		0	0	https://www.ncbi.nlm.nih.gov/clinvar/variation/419397/
NM_000108.3(DLD):c.763A>C	concordant	unreportable	unreportable	203680	0	1	SCV000466236	Uncertain significance	Illumina Clinical Se	10/18/2016	DLD	chr7	107556029	107556029	http://gnomad.b	0.0001531767296	SAS	0.00237166998	0		0	0	https://www.ncbi.nlm.nih.gov/clinvar/variation/203680/
NM_000227.3(LAMA3):c.3366G>C	concordant	unreportable	unreportable	326327	0	1	SCV000407981	Uncertain significance	Illumina Clinical Se	10/18/2016	LAMA3	chr18	21508102	21508102	http://gnomad.b	6.72E-05	EAS	0.001043720054	0		0	0	https://www.ncbi.nlm.nih.gov/clinvar/variation/326327/
NM_000243.2(MEFV):c.657C>T	concordant	unreportable	unreportable	97534	0	1	SCV000696078	Uncertain significance	Laboratory Corpora	1/25/2018	MEFV	chr16	3304411	3304411	http://gnomad.b	0.0001259445844	ASJ	0.0002116849937	0		0	0	https://www.ncbi.nlm.nih.gov/clinvar/variation/97534/
NM_000152.3(GAA):c.1143C>T	concordant	unreportable	unreportable	284480	0	2	SCV000337122;S	Uncertain significance;Likel	EGL Genetic Diagn	2017-06-30;2017-1	GAA	chr17	78082355	78082355	http://gnomad.b	4.17E-05	AMR	5.98E-05	0		0	0	https://www.ncbi.nlm.nih.gov/clinvar/variation/284480/
NM_002485.4(NBN):c.648G>A	concordant	unreportable	unreportable	185038	0	1	SCV000685807	Likely benign	Color Genomics, In	10/26/2017	NBN	chr8	90983455	90983455		2.79E-06					0	0	https://www.ncbi.nlm.nih.gov/clinvar/variation/185038/
NM_138694.3(PKHD1):c.7192T>G	concordant	unreportable	unreportable	357432	0	1	SCV000464068	Uncertain significance	Illumina Clinical Se	10/18/2016	PKHD1	chr6	51750688	51750688	http://gnomad.b	8.06E-05	NFE	4.50E-05	0		0	0	https://www.ncbi.nlm.nih.gov/clinvar/variation/357432/
NM_002485.4(NBN):c.644G>A	concordant	unreportable	unreportable	141888	0	4	SCV000685806;S	Uncertain significance;Unc	Color Genomics, In	2017-10-26;2017-1	NBN	chr8	90983459	90983459	http://gnomad.b	4.46E-05	NFE	4.49E-05	0		0	0	https://www.ncbi.nlm.nih.gov/clinvar/variation/141888/
NM_002485.4(NBN):c.73G>A	concordant	unreportable	unreportable	141440	0	3	SCV000211446;S	Uncertain significance;Unc	GeneDx;Invitae;Qu	2017-11-28;2017-1	NBN	chr8	90995048	90995048	http://gnomad.b	3.63E-05	SAS	0.0001299550058	0		1	0	https://www.ncbi.nlm.nih.gov/clinvar/variation/141440/
NM_000051.3(ATM):c.1380G>A	concordant	unreportable	unreportable	231597	0	2	SCV000681973;S	Likely benign;Likely benign	Color Genomics, In	2017-10-26;2017-1	ATM	chr11	108121572	108121572	http://gnomad.b	2.51E-05	AMR	2.98E-05	0		0	0	https://www.ncbi.nlm.nih.gov/clinvar/variation/231597/
NM_000016.4(ACADM):c.773C>T	concordant	unreportable	unreportable	431984	0	1	SCV000589621	Uncertain significance	GeneDx	11/28/2017	ACADM	chr1	76215168	76215168	http://gnomad.b	8.16E-06	NFE	8.95E-06	0		0	0	https://www.ncbi.nlm.nih.gov/clinvar/variation/431984/
NM_000051.3(ATM):c.3300G>A	concordant	unreportable	unreportable	135750	0	2	SCV000682116;S	Likely benign;Likely benign	Color Genomics, In	2017-10-26;2017-1	ATM	chr11	108150233	108150233	http://gnomad.b	7.52E-05	NFE	7.36E-05	0		0	0	https://www.ncbi.nlm.nih.gov/clinvar/variation/135750/
NM_000391.3(TPP1):c.319G>A	concordant	unreportable	unreportable	207568	0	1	SCV000243406	Uncertain significance	GeneDx	11/28/2017	TPP1	chr11	6638918	6638918	http://gnomad.b	7.55E-05	AFR	0.0007187660085	1		0	0	https://www.ncbi.nlm.nih.gov/clinvar/variation/207568/
NM_000051.3(ATM):c.7000T>C	concordant	unreportable	unreportable	418584	0	2	SCV000687748;S	Uncertain significance;Unc	Color Genomics, In	2017-12-21;2017-1	ATM	chr11	108198396	108198396		2.78E-06					0	0	https://www.ncbi.nlm.nih.gov/clinvar/variation/418584/
NM_000018.3(ACADVL):c.1434G>A	concordant	unreportable	unreportable	429246	0	1	SCV000581766	Uncertain significance	GeneDx	11/28/2017	ACADVL	chr17	7127388	7127388	http://gnomad.b	9.06E-05	EAS	0.001971469959	0		0	0	https://www.ncbi.nlm.nih.gov/clinvar/variation/429246/
NM_000112.3(SLC26A2):c.993A>C	concordant	unreportable	unreportable	352024	0	1	SCV000454784	Uncertain significance	Illumina Clinical Se	10/18/2016	SLC26A2	chr5	149360149	149360149	http://gnomad.b	5.58E-05	NFE	9.87E-05	0		0	0	https://www.ncbi.nlm.nih.gov/clinvar/variation/352024/
NM_001360.2(DHCR7):c.1099C>T	concordant	unreportable	unreportable	281496	0	1	SCV000332301	Uncertain significance	EGL Genetic Diagn	6/30/2017	DHCR7	chr11	71146750	71146750	http://gnomad.b	0.0001561692228	EAS	0.001919719973	0		0	0	https://www.ncbi.nlm.nih.gov/clinvar/variation/281496/
NM_018941.3(CLN8):c.151G>A	concordant	unreportable	unreportable	499266	0	1	SCV000704683	Uncertain significance	EGL Genetic Diagn	6/30/2017	CLN8	chr8	1719371	1719371	http://gnomad.b	9.51E-05	SAS	0.0006497299764	0		0	0	https://www.ncbi.nlm.nih.gov/clinvar/variation/499266/
NM_017739.3(POMGNT1):c.289C>T	concordant	unreportable	unreportable	290011	0	1	SCV000344487	Likely benign	EGL Genetic Diagn	6/30/2017	POMGNT1	chr1	46662468	46662468	http://gnomad.b	0.0001455775228	SAS	0.002306540031	2		0	0	https://www.ncbi.nlm.nih.gov/clinvar/variation/290011/
NM_017890.4(VPS13B):c.3436C>G	concordant	unreportable	unreportable	361057	0	1	SCV000470794	Uncertain significance	Illumina Clinical Se	10/18/2016	VPS13B	chr8	100454854	100454854	http://gnomad.b	8.40E-06	FIN	0.001077200053	1		0	0	https://www.ncbi.nlm.nih.gov/clinvar/variation/361057/
NM_000018.3(ACADVL):c.864C>T	concordant	unreportable	unreportable	474904	0	1	SCV000654972	Likely benign	Invitae	10/5/2017	ACADVL	chr17	7125607	7125607	http://gnomad.b	1.13E-05	SAS	6.50E-05	0		0	0	https://www.ncbi.nlm.nih.gov/clinvar/variation/474904/
NM_000051.3(ATM):c.3432G>A	concordant	unreportable	unreportable	407599	0	1	SCV000546919	Likely benign	Invitae	10/5/2017	ATM	chr11	108151751	108151751		2.78E-06					0	0	https://www.ncbi.nlm.nih.gov/clinvar/variation/407599/
NM_024649.4(BBS1):c.772G>C	concordant	unreportable	unreportable	289320	0	1	SCV000343666	Uncertain significance	EGL Genetic Diagn	6/30/2017	BBS1	chr11	66288789	66288789	http://gnomad.b	5.85E-05	EAS	0.0008697669837	0		0	0	https://www.ncbi.nlm.nih.gov/clinvar/variation/289320/
NM_000051.3(ATM):c.6636C>T	concordant	unreportable	unreportable	232114	0	1	SCV000516572	Likely benign	GeneDx	11/28/2017	ATM	chr11	108196100	108196100		5.57E-06					0	0	https://www.ncbi.nlm.nih.gov/clinvar/variation/232114/
NM_000391.3(TPP1):c.75C>T	concordant	unreportable	unreportable	378753	0	1	SCV000514955	Likely benign	GeneDx	11/28/2017	TPP1	chr11	6640441	6640441	http://gnomad.b	4.76E-05	SAS	0.0001299709984	0		0	0	https://www.ncbi.nlm.nih.gov/clinvar/variation/378753/
NM_000051.3(ATM):c.5776A>G	concordant	unreportable	unreportable	236743	0	1	SCV000283000	Uncertain significance	Invitae	6/10/2016	ATM	chr11	108180900	108180900	http://gnomad.b	2.78E-06	NFE	8.97E-06	0		0	0	https://www.ncbi.nlm.nih.gov/clinvar/variation/236743/
NM_000051.3(ATM):c.8391T>C	concordant	unreportable	unreportable	236787	0	3	SCV000682480;S	Likely benign;Uncertain sig	Color Genomics, In	2017-10-26;2016-1	ATM	chr11	108214071	108214071	http://gnomad.b	0.0001448048476	EAS	0.00179794	0		0	0	https://www.ncbi.nlm.nih.gov/clinvar/variation/236787/
NM_033056.3(PCDH15):c.466G>C	concordant	unreportable	unreportable	300204	0	1	SCV000363247	Uncertain significance	Illumina Clinical Se	10/18/2016	PCDH15	chr10	56128888	56128888	http://gnomad.b	2.83E-05	NFE	7.19E-05	0		0	0	https://www.ncbi.nlm.nih.gov/clinvar/variation/300204/
NM_138694.3(PKHD1):c.658G>A	concordant	unreportable	unreportable	498018	0	1	SCV000702825	Uncertain significance	EGL Genetic Diagn	6/30/2017	PKHD1	chr6	51935813	51935813	http://gnomad.b	0.0001334386016	FIN	0.001435109996	0		0	0	https://www.ncbi.nlm.nih.gov/clinvar/variation/498018/
NM_000051.3(ATM):c.7140A>G	concordant	unreportable	unreportable	236765	0	3	SCV000682390;S	Likely benign;Likely benign	Color Genomics, In	2017-10-26;2017-1	ATM	chr11	108199798	108199798	http://gnomad.b	8.63E-05	SAS	0.000844648981	0		0	0	https://www.ncbi.nlm.nih.gov/clinvar/variation/236765/
NM_000051.3(ATM):c.8140C>G	concordant	unreportable	unreportable	407710	0	3	SCV000682457;S	Uncertain significance;Unc	Color Genomics, In	2017-10-26;2017-1	ATM	chr11	108205825	108205825		8.35E-06					0	0	https://www.ncbi.nlm.nih.gov/clinvar/variation/407710/
NM_000391.3(TPP1):c.497A>T	concordant	unreportable	unreportable	207573	0	1	SCV000628910	Uncertain significance	Invitae	10/5/2017	TPP1	chr11	6638543	6638543		1.12E-05					0	0	https://www.ncbi.nlm.nih.gov/clinvar/variation/207573/
NM_001042432.1(CLN3):c.1086C>G	concordant	unreportable	unreportable	205098	0	2	SCV000396321;S	Uncertain significance;Unc	Illumina Clinical Se	2016-10-18;2016-0	CLN3	chr16	28489169	28489169	http://gnomad.b	6.94E-05	EAS	0.0004644140136	0		0	0	https://www.ncbi.nlm.nih.gov/clinvar/variation/205098/
NM_000352.3(ABCC8):c.2937C>T	concordant	unreportable	unreportable	303769	0	1	SCV000369307	Uncertain significance	Illumina Clinical Se	10/18/2016	ABCC8	chr11	17428660	17428660	http://gnomad.b	3.36E-05	EAS	0.000292022014	0		0	0	https://www.ncbi.nlm.nih.gov/clinvar/variation/303769/
NM_000136.2(FANCC):c.993G>A	concordant	unreportable	unreportable	220261	0	1	SCV000260676	Likely benign	Invitae	1/6/2016	FANCC	chr9	97887371	97887371	http://gnomad.b	2.62E-05	EAS	0.0001159550011	0		0	0	https://www.ncbi.nlm.nih.gov/clinvar/variation/220261/
NM_002485.4(NBN):c.353_355delCTT	concordant	unreportable	unreportable	182704	0	4	SCV000685775;S	Uncertain significance;Unc	Color Genomics, In	2017-10-26;2017-0	NBN	chr8	90993086	90993089	http://gnomad.b	4.46E-05	AFR	0.0001307360071	0		0	0	https://www.ncbi.nlm.nih.gov/clinvar/variation/182704/
NM_000051.3(ATM):c.76G>C	concordant	unreportable	unreportable	181944	0	4	SCV000602572;S	Uncertain significance;Unc	ARUP Laboratories	2017-06-30;2017-1	ATM	chr11	108098506	108098506		1.39E-05					0	0	https://www.ncbi.nlm.nih.gov/clinvar/variation/181944/
NM_000152.3(GAA):c.1932C>T	concordant	unreportable	unreportable	387757	0	2	SCV000529884;S	Likely benign;Likely benign	GeneDx;Invitae	2017-11-28;2017-1	GAA	chr17	78086718	78086718	http://gnomad.b	6.67E-05	NFE	7.43E-05	0		0	0	https://www.ncbi.nlm.nih.gov/clinvar/variation/387757/
NM_000051.3(ATM):c.38G>A	concordant	unreportable	unreportable	188171	0	1	SCV000322154	Uncertain significance	GeneDx	11/28/2017	ATM	chr11	108098389	108098389	http://gnomad.b	1.39E-05	SAS	3.25E-05	0		0	0	https://www.ncbi.nlm.nih.gov/clinvar/variation/188171/
NM_000071.2(CBS):c.887C>T	concordant	unreportable	unreportable	471368	0	1	SCV000649851	Uncertain significance	Invitae	10/5/2017	CBS	chr21	44483130	44483130	http://gnomad.b	0.0001209162641	SAS	0.001721789944	0		0	0	https://www.ncbi.nlm.nih.gov/clinvar/variation/471368/
NM_000051.3(ATM):c.4753A>G	concordant	unreportable	unreportable	414544	0	1	SCV000558325	Benign	Invitae	10/5/2017	ATM	chr11	108164181	108164181	http://gnomad.b	1.11E-05	SAS	0.0002276269952	0		0	0	https://www.ncbi.nlm.nih.gov/clinvar/variation/414544/

Supplemental Table 2

NM_000051.3(ATM):c.712A>G	concordant	unreportable	unreportable	186210	0	2	SCV000293178;S	Uncertain significance;Unc	GeneDx;Invitae	2017-11-28;2017-1	ATM	chr11	108115564	108115564	http://gnomad.b	1.67E-05	NFE	2.69E-05	0		0	0	https://www.ncbi.nlm.nih.gov/clinvar/variation/186210/
NM_018941.3(CLN8):c.796G>A	concordant	unreportable	unreportable	424331	0	1	SCV000574129	Uncertain significance	GeneDx	11/28/2017	CLN8	chr8	1728668	1728668	http://gnomad.b	7.84E-05	EAS	0.0008117820253	0		0	0	https://www.ncbi.nlm.nih.gov/clinvar/variation/424331/
NM_006493.2(CLN5):c.789A>T	concordant	unreportable	unreportable	312428	0	1	SCV000384729	Uncertain significance	Illumina Clinical Se	10/18/2016	CLN5	chr13	77574669	77574669	http://gnomad.b	0.0001035040311	NFE	0.000116449999	0		0	0	https://www.ncbi.nlm.nih.gov/clinvar/variation/312428/
NM_000051.3(ATM):c.2449G>C	concordant	unreportable	unreportable	133608	0	3	SCV000292218;S	Uncertain significance;Unc	Color Genomics, In	2017-12-21;2017-1	ATM	chr11	108129785	108129785	http://gnomad.b	6.13E-05	AMR	0.001966040116	0		0	0	https://www.ncbi.nlm.nih.gov/clinvar/variation/133608/
NM_001042432.1(CLN3):c.1198-5C>T	concordant	unreportable	unreportable	287485	0	1	SCV000341269	Uncertain significance	EGL Genetic Diagn	6/30/2017	CLN3	chr16	28488961	28488961	http://gnomad.b	1.00E-04	EAS	0.001217529993	0		0	0	https://www.ncbi.nlm.nih.gov/clinvar/variation/287485/
NM_003060.3(SLC22A5):c.1250dupT	concordant	reportable	reportable	503649	1	0	SCV000709849	Likely pathogenic	GeneDx	3/26/2018	SLC22A5	chr5	131726578	131726578	http://gnomad.b	2.82E-06	NFE	8.96E-06	0		0	0	https://www.ncbi.nlm.nih.gov/clinvar/variation/503649/
NM_002485.4(NBN):c.2240A>G	concordant	unreportable	unreportable	461549	0	1	SCV000634287	Uncertain significance	Invitae	10/5/2017	NBN	chr8	90947835	90947835		2.79E-06					0	0	https://www.ncbi.nlm.nih.gov/clinvar/variation/461549/
NM_000383.3(AIRE):c.1404G>C	concordant	unreportable	unreportable	458615	0	1	SCV000629948	Benign	Invitae	10/5/2017	AIRE	chr21	45714287	45714287	http://gnomad.b	7.80E-05	AMR	0.001586750033	0		0	0	https://www.ncbi.nlm.nih.gov/clinvar/variation/458615/
NM_000227.3(LAMA3):c.5021G>A	concordant	unreportable	unreportable	326349	0	1	SCV000408039	Uncertain significance	Illumina Clinical Se	10/18/2016	LAMA3	chr18	21533065	21533065	http://gnomad.b	1.96E-05	NFE	2.69E-05	0		0	0	https://www.ncbi.nlm.nih.gov/clinvar/variation/326349/
NM_000520.4(HEXA):c.-2564_253+51	concordant	reportable	reportable	3891	1	0	SCV000697167	Pathogenic	Laboratory Corpora	1/25/2018	HEXA	chr15	72662932	72670877		1.17E-05					0	0	https://www.ncbi.nlm.nih.gov/clinvar/variation/3891/
NM_000492.3(CFTR):c.1820_1903del8	concordant	reportable	reportable	53396	1	0	SCV000696875	Pathogenic	Laboratory Corpora	1/25/2018	CFTR	chr7	117232037	117232121		7.70E-06					0	0	https://www.ncbi.nlm.nih.gov/clinvar/variation/53396/
NM_001876.3(CPT1A):c.517C>T	concordant	unreportable	unreportable	463967	0	1	SCV000638100	Uncertain significance	Invitae	10/5/2017	CPT1A	chr11	68571506	68571506	http://gnomad.b	1.69E-05	AMR	8.93E-05	0		0	0	https://www.ncbi.nlm.nih.gov/clinvar/variation/463967/
NM_002485.4(NBN):c.266G>A	concordant	unreportable	unreportable	221045	0	1	SCV000262164	Uncertain significance	Invitae	10/5/2017	NBN	chr8	90993657	90993657	http://gnomad.b	2.51E-05	AFR	6.54E-05	0		0	0	https://www.ncbi.nlm.nih.gov/clinvar/variation/221045/
NM_001042432.1(CLN3):c.754C>T	concordant	unreportable	unreportable	193615	0	1	SCV000568999	Uncertain significance	GeneDx	11/28/2017	CLN3	chr16	28495363	28495363	http://gnomad.b	0.0001055532099	NFE	0.0001189079994	0		0	0	https://www.ncbi.nlm.nih.gov/clinvar/variation/193615/
NM_000642.2(AGL):c.1602C>T	concordant	unreportable	unreportable	456456	0	1	SCV000626675	Benign	Invitae	10/5/2017	AGL	chr1	100343375	100343375	http://gnomad.b	0.0002260713269	AMR	0.003547579981	0		1	0	https://www.ncbi.nlm.nih.gov/clinvar/variation/456456/
NM_000053.3(ATP7B):c.618T>A	concordant	unreportable	unreportable	312399	0	1	SCV000384686	Uncertain significance	Illumina Clinical Se	10/18/2016	ATP7B	chr13	52548738	52548738		2.81E-06					0	0	https://www.ncbi.nlm.nih.gov/clinvar/variation/312399/
NM_000227.3(LAMA3):c.3830G>A	concordant	unreportable	unreportable	326337	0	1	SCV000408008	Uncertain significance	Illumina Clinical Se	10/18/2016	LAMA3	chr18	21512204	21512204	http://gnomad.b	4.20E-05	AFR	6.53E-05	0		0	0	https://www.ncbi.nlm.nih.gov/clinvar/variation/326337/
NM_002485.4(NBN):c.720C>T	concordant	unreportable	unreportable	183990	0	3	SCV000685820;S	Likely benign;Likely benign	Color Genomics, In	2017-10-26;2017-1	NBN	chr8	90982768	90982768	http://gnomad.b	4.18E-05	AFR	6.55E-05	0		0	0	https://www.ncbi.nlm.nih.gov/clinvar/variation/183990/
NM_000051.3(ATM):c.6868G>A	concordant	unreportable	unreportable	482670	0	1	SCV000682370	Uncertain significance	Color Genomics, In	10/26/2017	ATM	chr11	108196845	108196845		2.78E-06					0	0	https://www.ncbi.nlm.nih.gov/clinvar/variation/482670/
NM_000152.3(GAA):c.425G>A	concordant	unreportable	unreportable	456422	0	1	SCV000626612	Uncertain significance	Invitae	10/5/2017	GAA	chr17	78078810	78078810	http://gnomad.b	8.34E-06	EAS	5.82E-05	0		0	0	https://www.ncbi.nlm.nih.gov/clinvar/variation/456422/
NM_000027.3(AGA):c.657T>C	concordant	unreportable	unreportable	348232	0	1	SCV000448732	Uncertain significance	Illumina Clinical Se	10/18/2016	AGA	chr4	178357471	178357471	http://gnomad.b	8.89E-06	NFE	1.79E-05	0		0	0	https://www.ncbi.nlm.nih.gov/clinvar/variation/348232/
NM_002485.4(NBN):c.1034G>T	concordant	unreportable	unreportable	127852	0	2	SCV000149683;S	Uncertain significance;Unc	GeneDx;Invitae	2017-11-28;2017-1	NBN	chr8	90971043	90971043	http://gnomad.b	8.37E-06	AMR	5.96E-05	0		0	0	https://www.ncbi.nlm.nih.gov/clinvar/variation/127852/
NM_001360.2(DHCR7):c.1365C>T	concordant	unreportable	unreportable	290040	0	1	SCV000344522	Uncertain significance	EGL Genetic Diagn	6/30/2017	DHCR7	chr11	71146484	71146484	http://gnomad.b	8.35E-05	AFR	0.0004683530133	0		0	0	https://www.ncbi.nlm.nih.gov/clinvar/variation/290040/
NM_000271.4(NPC1):c.699G>A	concordant	unreportable	unreportable	326277	0	1	SCV000407885	Uncertain significance	Illumina Clinical Se	10/18/2016	NPC1	chr18	21140377	21140377	http://gnomad.b	1.43E-05	SAS	9.75E-05	0		0	0	https://www.ncbi.nlm.nih.gov/clinvar/variation/326277/
NM_000136.2(FANCC):c.748C>A	concordant	unreportable	unreportable	234480	0	1	SCV000279322	Uncertain significance	GeneDx	11/28/2017	FANCC	chr9	97897723	97897723	http://gnomad.b	2.14E-05	NFE	1.79E-05	0		0	0	https://www.ncbi.nlm.nih.gov/clinvar/variation/234480/
NM_000051.3(ATM):c.4611+9C>G	concordant	unreportable	unreportable	377513	0	3	SCV000512162;S	Likely benign;Likely benign	GeneDx;Invitae;La	2017-11-28;2017-1	ATM	chr11	108163529	108163529	http://gnomad.b	2.78E-06	NFE	2.69E-05	0		0	0	https://www.ncbi.nlm.nih.gov/clinvar/variation/377513/
NM_004328.4(BCS1L):c.768C>G	concordant	unreportable	unreportable	334359	0	1	SCV000427442	Uncertain significance	Illumina Clinical Se	10/18/2016	BCS1L	chr2	219527281	219527281	http://gnomad.b	2.51E-05	ASJ	0.000203211006	0		0	0	https://www.ncbi.nlm.nih.gov/clinvar/variation/334359/
NM_002485.4(NBN):c.1729G>T	concordant	unreportable	unreportable	141617	0	4	SCV000685734;S	Uncertain significance;Unc	Color Genomics, In	2017-10-26;2017-0	NBN	chr8	90965588	90965588	http://gnomad.b	5.86E-05	NFE	4.48E-05	0		0	0	https://www.ncbi.nlm.nih.gov/clinvar/variation/141617/
NM_001042432.1(CLN3):c.771G>C	concordant	unreportable	unreportable	497479	0	1	SCV000702002	Uncertain significance	EGL Genetic Diagn	6/30/2017	CLN3	chr16	28495346	28495346		1.11E-05					0	0	https://www.ncbi.nlm.nih.gov/clinvar/variation/497479/
NM_138694.3(PKHD1):c.5301T>C	concordant	unreportable	unreportable	357436	0	1	SCV000464082	Uncertain significance	Illumina Clinical Se	10/18/2016	PKHD1	chr6	51887678	51887678	http://gnomad.b	2.50E-05	SAS	9.75E-05	0		0	0	https://www.ncbi.nlm.nih.gov/clinvar/variation/357436/
NM_000518.4(HBB):c.45G>A	concordant	unreportable	unreportable	440467	0	1	SCV000605838	Uncertain significance	Quest Diagnostics I	8/1/2017	HBB	chr11	5248207	5248207	http://gnomad.b	2.33E-05	SAS	0.0001299550058	0		0	0	https://www.ncbi.nlm.nih.gov/clinvar/variation/440467/
NM_033056.3(PCDH15):c.4463A>G	concordant	unreportable	unreportable	227840	0	1	SCV000270726	Likely benign	Laboratory for Mol	4/26/2016	PCDH15	chr10	55583023	55583023	http://gnomad.b	5.91E-05	EAS	0.001159689971	0		0	0	https://www.ncbi.nlm.nih.gov/clinvar/variation/227840/
NM_000492.3(CFTR):c.41A>T	concordant	unreportable	unreportable	495946	0	1	SCV000697010	Uncertain significance	Laboratory Corpora	1/25/2018	CFTR	chr7	117120189	117120189	http://gnomad.b	5.58E-05	NFE	8.10E-05	0		0	0	https://www.ncbi.nlm.nih.gov/clinvar/variation/495946/
NM_000071.2(CBS):c.1500G>A	concordant	unreportable	unreportable	387779	0	1	SCV000529911	Likely benign	GeneDx	11/28/2017	CBS	chr21	44476965	44476965		1.97E-05					0	0	https://www.ncbi.nlm.nih.gov/clinvar/variation/387779/
NM_017890.4(VPS13B):c.8736A>G	concordant	unreportable	unreportable	437242	0	1	SCV000597892	Likely benign	Genetic Services La	7/5/2017	VPS13B	chr8	100831679	100831679	http://gnomad.b	0.0001483804138	SAS	0.00146208005	2		0	0	https://www.ncbi.nlm.nih.gov/clinvar/variation/437242/
NM_000051.3(ATM):c.4018T>C	concordant	unreportable	unreportable	385319	0	3	SCV000682175;S	Likely benign;Likely benign	Color Genomics, In	2017-10-26;2017-1	ATM	chr11	108158351	108158351		5.57E-06					0	0	https://www.ncbi.nlm.nih.gov/clinvar/variation/385319/
NM_000136.2(FANCC):c.792T>G	concordant	unreportable	unreportable	182477	0	3	SCV000211043;S	Uncertain significance;Unc	GeneDx;Invitae;La	2017-11-28;2016-0	FANCC	chr9	97897679	97897679	http://gnomad.b	5.95E-05	NFE	8.06E-05	0		0	0	https://www.ncbi.nlm.nih.gov/clinvar/variation/182477/
NM_000543.4(SMPD1):c.1341-9T>C	concordant	unreportable	unreportable	497294	0	1	SCV000701722	Uncertain significance	EGL Genetic Diagn	6/30/2017	SMPD1	chr11	6415117	6415117	http://gnomad.b	6.18E-05	EAS	0.0006957330042	0		0	0	https://www.ncbi.nlm.nih.gov/clinvar/variation/497294/
NM_017890.4(VPS13B):c.9859G>A	concordant	unreportable	unreportable	361084	0	1	SCV000470843	Uncertain significance	Illumina Clinical Se	10/18/2016	VPS13B	chr8	100847808	100847808	http://gnomad.b	7.28E-05	SAS	0.0006822170108	0		0	0	https://www.ncbi.nlm.nih.gov/clinvar/variation/361084/
NM_000057.2(BLM):c.4126A>G	concordant	unreportable	unreportable	127511	0	1	SCV000623333	Uncertain significance	Invitae	10/5/2017	BLM	chr15	91358381	91358381	http://gnomad.b	1.43E-05	NFE	8.95E-06	0		0	0	https://www.ncbi.nlm.nih.gov/clinvar/variation/127511/
NM_017890.4(VPS13B):c.5836A>G	concordant	unreportable	unreportable	196920	0	1	SCV000229740	Uncertain significance	EGL Genetic Diagn	6/30/2017	VPS13B	chr8	100654579	100654579	http://gnomad.b	3.08E-05	SAS	3.25E-05	0		0	0	https://www.ncbi.nlm.nih.gov/clinvar/variation/196920/
NM_014363.4(SACS):c.789C>T	concordant	unreportable	unreportable	448221	0	1	SCV000614980	Likely benign	Athena Diagnostics	8/17/2017	SACS	chr13	23929962	23929962	http://gnomad.b	1.48E-05	AMR	8.93E-05	0		0	0	https://www.ncbi.nlm.nih.gov/clinvar/variation/448221/
NM_001079802.1(FKTN):c.706A>G	concordant	unreportable	unreportable	281831	0	2	SCV000332832;S	Uncertain significance;Unc	EGL Genetic Diagn	2017-06-30;2017-1	FKTN	chr9	108370158	108370158	http://gnomad.b	2.48E-05	NFE	8.96E-06	0		0	0	https://www.ncbi.nlm.nih.gov/clinvar/variation/281831/
NM_000051.3(ATM):c.5145G>A	concordant	unreportable	unreportable	186194	0	1	SCV000259444	Likely benign	Invitae	1/6/2016	ATM	chr11	108170580	108170580	http://gnomad.b	4.18E-05			0		0	0	https://www.ncbi.nlm.nih.gov/clinvar/variation/186194/
NM_000051.3(ATM):c.5658T>C	concordant	unreportable	unreportable	414598	0	2	SCV000682278;S	Likely benign;Likely benign	Color Genomics, In	2017-10-26;2017-1	ATM	chr11	108175563	108175563	http://gnomad.b	1.11E-05	AFR	0.0001309929939	0		0	0	https://www.ncbi.nlm.nih.gov/clinvar/variation/414598/
NM_002485.4(NBN):c.1008_1010dup	concordant	unreportable	unreportable	186477	0	2	SCV000571923;S	Uncertain significance;Unc	GeneDx;Invitae	2017-11-28;2017-1	NBN	chr8	90971066	90971066		2.79E-06					0	0	https://www.ncbi.nlm.nih.gov/clinvar/variation/186477/
NM_001079802.1(FKTN):c.357A>G	concordant	unreportable	unreportable	501555	0	1	SCV000707961	Uncertain significance	EGL Genetic Diagn	6/30/2017	FKTN	chr9	108363617	108363617	http://gnomad.b	1.42E-05	AMR	0.001072769985	0		0	0	https://www.ncbi.nlm.nih.gov/clinvar/variation/501555/
NM_014363.4(SACS):c.12216T>A	concordant	unreportable	unreportable	311502	0	2	SCV000614931;S	Likely benign;Uncertain sig	Athena Diagnostics	2017-08-17;2016-1	SACS	chr13	23905799	23905799	http://gnomad.b	7.71E-05	EAS	0.00295755011	0		0	0	https://www.ncbi.nlm.nih.gov/clinvar/variation/311502/
NM_000152.3(GAA):c.1388G>A	concordant	unreportable	unreportable	325788	0	1	SCV000407281	Uncertain significance	Illumina Clinical Se	10/18/2016	GAA	chr17	78083805	78083805	http://gnomad.b	8.34E-06	SAS	6.50E-05	0		0	0	https://www.ncbi.nlm.nih.gov/clinvar/variation/325788/
NM_017739.3(POMGNT1):c.698C>T	concordant	unreportable	unreportable	373719	0	1	SCV000492339	Uncertain significance	GeneDx	12/21/2016	POMGNT1	chr1	46660278	46660278	http://gnomad.b	9.80E-05	SAS	0.002176600043	1		0	0	https://www.ncbi.nlm.nih.gov/clinvar/variation/373719/
NM_000466.2(PEX1):c.3106G>T	concordant	unreportable	unreportable	284663	0	1	SCV000337369	Uncertain significance	EGL Genetic Diagn	6/30/2017	PEX1	chr7	92122368	92122368	http://gnomad.b	5.82E-05	AFR	0.0008496730006	0		0	0	https://www.ncbi.nlm.nih.gov/clinvar/variation/284663/
NM_000492.3(CFTR):c.29G>A	concordant	unreportable	unreportable	439071	0	1	SCV000601085	Uncertain significance	Quest Diagnostics I	8/1/2017	CFTR	chr7	117120177	117120177	http://gnomad.b	4.04E-05	AMR	2.98E-05	0		0	0	https://www.ncbi.nlm.nih.gov/clinvar/variation/439071/
NM_000071.2(CBS):c.147G>A	concordant	unreportable	unreportable	236936	0	1	SCV000283383	Likely benign	Invitae	6/10/2016	CBS	chr21	44492157	44492157	http://gnomad.b	8.15E-05	SAS	0.0004873609869	0		0	0	https://www.ncbi.nlm.nih.gov/clinvar/variation/236936/
NM_018941.3(CLN8):c.648C>T	concordant	unreportable	unreportable	282074	0	2	SCV000333264;S	Uncertain significance;Likel	EGL Genetic Diagn	2017-06-30;2017-1	CLN8	chr8	1728520	1728520	http://gnomad.b	0.0001007387508	SAS	0.001299459953	1		0	0	https://www.ncbi.nlm.nih.gov/clinvar/variation/282074/
NM_000642.2(AGL):c.3346C>T	concordant	unreportable	unreportable	456488	0	1	SCV000626725	Uncertain significance	Invitae	10/5/2017	AGL	chr1	100361928	100361928	http://gnomad.b	1.95E-05	NFE	4.48E-05	0		0	0	https://www.ncbi.nlm.nih.gov/clinvar/variation/456488/
NM_002485.4(NBN):c.332A>G	concordant	unreportable	unreportable	418680	0	1	SCV000634297	Uncertain significance	Invitae	10/5/2017	NBN	chr8	90993110	90993110		2.79E-06					0	0	https://www.ncbi.nlm.nih.gov/clinvar/variation/418680/
NM_014363.4(SACS):c.8315G>C	concordant	unreportable	unreportable	448223	0	1	SCV000614984	Uncertain significance	Athena Diagnostics	8/17/2017	SACS	chr13	23909700	23909700	http://gnomad.b	6.22E-05	NFE	9.87E-05	0		0	0	https://www.ncbi.nlm.nih.gov/clinvar/variation/448223/
NM_017890.4(VPS13B):c.9444C>T	concordant	unreportable	unreportable	361081	0	1	SCV000470838	Uncertain significance	Illumina Clinical Se	10/18/2016	VPS13B	chr8	100844635	100844635	http://gnomad.b	0.0001203841093	AMR	0.000844780996	0		1	0	https://www.ncbi.nlm.nih.gov/clinvar/variation/361081/
NM_000051.3(ATM):c.7648A>G	concordant	unreportable	unreportable	236777	0	2	SCV000687785;S	Uncertain significance;Unc	Color Genomics, In	2017-12-21;2016-0	ATM	chr11	108202624	108202624		5.57E-06					0	0	https://www.ncbi.nlm.nih.gov/clinvar/variation/236777/
NM_000051.3(ATM):c.6700C>T	concordant	unreportable	unreportable	186856	0	3	SCV000682361;S	Likely benign;Likely benign	Color Genomics, In	2017-10-26;2017-1	ATM	chr11	108196164	108196164	http://gnomad.b	1.11E-05	NFE	2.69E-05	0		0	0	https://www.ncbi.nlm.nih.gov/clinvar/variation/186856/
NM_000152.3(GAA):c.634G>A	concordant	unreportable	unreportable	456425	0	1	SCV000626620	Uncertain significance	Invitae	10/5/2017	GAA	chr17	78079635	78079635	http://gnomad.b	4.45E-05	EAS	0.0005223450135	0		0	0	https://www.ncbi.nlm.nih.gov/clinvar/variation/456425/
NM_018941.3(CLN8):c.256C>T	concordant	unreportable	unreportable	283772	0	1	SCV000336082	Uncertain significance	EGL Genetic Diagn	6/30/2017	CLN8	chr8	1719476	1719476	http://gnomad.b	3.08E-05	EAS	0.0002898889943	0		0	0	https://www.ncbi.nlm.nih.gov/clinvar/variation/283772/
NM_024685.3(BBS10):c.483A>G	concordant	unreportable	unreportable	287666	0	1	SCV000341506	Uncertain significance	EGL Genetic Diagn	6/30/2017	BBS10	chr12	76741282	76741282	http://gnomad.b	4.76E-05	EAS	0.0001162120025	0		0	0	https://www.ncbi.nlm.nih.gov/clinvar/variation/287666/
NM_000137.2(FAH):c.1026G>A	concordant	unreportable	unreportable	387640	0	1	SCV000529738	Likely benign	GeneDx	11/28/2017	FAH	chr15	80472531	80472531	http://gnomad.b	4.44E-05	NFE	9.87E-05	0		0	0	https://www.ncbi.nlm.nih.gov/clinvar/variation/387640/
NM_000051.3(ATM):c.3369A>G	concordant	unreportable	unreportable	132725	0	2	SCV000687468;S	Likely benign;Likely benign	Color Genomics, In	2017-12-21;2017-1	ATM	chr11	108150302	108150302	http://gnomad.b	1.39E-05	NFE	1.80E-05	0		0	0	https://www.ncbi.nlm.nih.gov/clinvar/variation/132725/
NM_000051.3(ATM):c.2284C>G	concordant	unreportable	unreportable	407700	0	1	SCV000547098	Uncertain significance	Invitae	3/14/2017	ATM	chr11	108128241	108128241		1.11E-05					0	0	https://www.ncbi.nlm.nih.gov/clinvar/variation/407700/
NM_017890.4(VPS13B):c.2785A>G	concordant	unreportable	unreportable	459246	0	1	SCV000630866	Uncertain significance	Invitae	10/5/2017	VPS13B	chr8	100287443	100287443		1.12E-05					0	0	https://www.ncbi.nlm.nih.gov/clinvar/variation/459246/
NM_001042432.1(CLN3):c.460+3G>A	concordant	unreportable	unreportable	409262	0	1	SCV000549197	Uncertain significance	Invitae	10/5/2017	CLN3	chr16	28498774	28498774	http://gnomad.b	5.28E-05	EAS	0.0005219210288	0		0	0	https://www.ncbi.nlm.nih.gov/clinvar/variation/409262/
NM_024649.4(BBS1):c.1594C>T	concordant	unreportable	unreportable	393063	0	1	SCV000536421	Uncertain significance	GeneDx	11/28/2017	BBS1	chr11	66298485	66298485	http://gnomad.b	4.18E-05	AFR	0.0001307189959	0		0	0	https://www.ncbi.nlm.nih.gov/clinvar/variation/393063/
NM_020533.2(MCOLN1):c.1021G>A	concordant	unreportable	unreportable	330499	0	1	SCV000415617	Uncertain significance	Illumina Clinical Se	10/18/2016	MCOLN1	chr19	7593743	7593743	http://gnomad.b	2.62E-05	AFR	0.0002617120044	0		0	0	https://www.ncbi.nlm.nih.gov/clinvar/variation/330499/
NM_138694.3(PKHD1):c.217C>T	concordant	unreportable	unreportable	499252	0	1	SCV000704651	Uncertain significance	EGL Genetic Diagn	6/30/2017	PKHD1	chr6	51947254	51947254	http://gnomad.b	4.17E-05	AFR	0.0001307019993	0		0	0	https://www.ncbi.nlm.nih.gov/clinvar/variation/499252/
NM_000051.3(ATM):c.2577C>T	concordant	unreportable	unreportable	181853	0	2	SCV000252949;S	Likely benign;Uncertain sig	Invitae;Laboratory	2017-10-05;2018-0	ATM	chr11	108138008	108138008	http://gnomad.b	2.23E-05	AFR	6.53E-05	0		0	0	https://www.ncbi.nlm.nih.gov/clinvar/variation/181853/
NM_000155.3(GALT):c.833T>C	concordant	unreportable	unreportable	500367	0	1	SCV000706277	Uncertain significance	EGL Genetic Diagn	6/30/2017	GALT	chr9	34649007	34649007	http://gnomad.b	0.0001044432351	SAS	0.0005522710271	0		0	0	https://www.ncbi.nlm.nih.gov/clinvar/variation/500367/
NM_002485.4(NBN):c.1370A>G	concordant	unreportable	unreportable	231710	0	3	SCV000685711;S	Uncertain significance;Unc	Color Genomics, In	2017-10-26;2017-0	NBN	chr8	90967538	90967538		8.37E-06					0	0	https://www.ncbi.nlm.nih.gov/clinvar/variation/231710/
NM_002485.4(NBN):c.1238A>G	concordant	unreportable	unreportable	127857	0	3	SCV000149688;S	Uncertain significance;Likel	GeneDx;Invitae;La	2017-11-28;2017-1	NBN	chr8	90967670	90967670	http://gnomad.b	5.30E-05	SAS	0.001202240004	2		0	0	https://www.ncbi.nlm.nih.gov/clinvar/variation/127857/
NM_000023.2(SGCA):c.1052G>A	concordant	unreportable	unreportable	289248	0	1	SCV000343575	Uncertain significance	EGL Genetic Diagn	6/30/2017	SGCA	chr17	48252686	48252686	http://gnomad.b	8.40E-06	NFE	8.48E-05	0		0	0	https://www.ncbi.nlm.nih.gov/clinvar/variation/289248/
NM_002485.4(NBN):c.803C>T	concordant	unreportable	unreportable	182734	0	3	SCV000685827;S	Uncertain significance;Unc	Color Genomics, In	2017-10-26;2017-0	NBN	chr8	90982685	90982685	http://gnomad.b	5.02E-05	NFE	7.17E-05	0		0	0	https://www.ncbi.nlm.nih.gov/clinvar/variation/182734/
NM_000051.3(ATM):c.3613C>T	concordant	unreportable	unreportable	216202	0	2	SCV000572291;S	Uncertain significance;Unc	GeneDx;Invitae	2017-11-28;2016-0	ATM	chr11	108153473	108153473	http://gnomad.b	1.95E-05	EAS	5.80E-05	0		0	0	https://www.ncbi.nlm.nih.gov/clinvar/variation/216202/
NM_033056.3(PCDH15):c.128T>C	concordant	unreportable	unreportable	229132	0	1	SCV000272293	Uncertain significance	Laboratory for Mol	4/26/2016	PCDH15	chr10	56287601	56287601	http://gnomad.b	2.83E-05	NFE	2.69E-05	0		0	0	https://www.ncbi.nlm.nih.gov/clinvar/variation/229132/
NM_000136.2(FANCC):c.15A>G	concordant	unreportable	unreportable	415746	0	1	SCV000560624	Likely benign	Invitae	10/5/2017	FANCC	chr9	98011559	98011559	http://gnomad.b	4.04E-05	SAS	3.25E-05	0		0	0	https://www.ncbi.nlm.nih.gov/clinvar/variation/415746/
NM_000492.3(CFTR):c.811delT	concordant	reportable	reportable	495959	1	0	SCV000697046	Likely pathogenic	Laboratory Corpora	1/25/2018	CFTR	chr7	117176668	117176669		3.85E-06					0	0	https://www.ncbi.nlm.nih.gov/clinvar/variation/495959/
NM_002485.4(NBN):c.1283A>T	concordant	unreportable	unreportable	461499	0	1	SCV000634214	Uncertain significance	Invitae	10/5/2017	NBN	chr8	90967625	90967625		5.58E-06					0	0	https://www.ncbi.nlm.nih.gov/clinvar/variation/461499/
NM_000051.3(ATM):c.1977G>T	concordant	unreportable	unreportable	407501	0	1	SCV000546741	Uncertain significance	Invitae	10/5/2017	ATM	chr11	108124619	108124619	http://gnomad.b	8.35E-06	NFE	8.96E-06	0		0	0	https://www.ncbi.nlm.nih.gov/clinvar/variation/407501/
NM_000071.2(CBS):c.1338G>A	concordant	unreportable	unreportable	413350	0	1	SCV000555942	Likely benign	Invitae	3/14/2017	CBS	chr21	44478964	44478964	http://gnomad.b	0.0001462243194	SAS	0.0002550370118	0		0	0	https://www.ncbi.nlm.nih.gov/clinvar/variation/413350/
NM_001079802.1(FKTN):c.21C>T	concordant	unreportable	unreportable	286899	0	1	SCV000340491	Uncertain significance	EGL Genetic Diagn	6/30/2017	FKTN	chr9	108337334	108337334	http://gnomad.b	0.000138287084	SAS	0.0008772499859	0		0	0	https://www.ncbi.nlm.nih.gov/clinvar/variation/286899/
NM_000057.2(BLM):c.3071A>G	concordant	unreportable	unreportable	317408	0	2	SCV000394422;S	Uncertain significance;Unc	Illumina Clinical Se	2016-10-18;2017-1	BLM	chr15	91337448	91337448	http://gnomad.b	3.09E-05	NFE	5.41E-05	0		0	0	https://www.ncbi.nlm.nih.gov/clinvar/variation/317408/
NM_000136.2(FANCC):c.1035T>G	concordant	unreportable	unreportable	385617	0	1	SCV000526942	Likely benign	GeneDx	11/28/2017	FANCC	chr9	97879634	97879634	http://gnomad.b	7.13E-06	NFE	8.96E-06	0		0	0	https://www.ncbi.nlm.nih.gov/clinvar/variation/385617/
NM_000391.3(TPP1):c.425T>G	concordant	unreportable	unreportable	207571	0	1	SCV000548739	Uncertain significance	Invitae	3/14/2017	TPP1	chr11	6638615	6638615	http://gnomad.b	1.96E-05	AMR	0.0005360330106	0		0	0	https://www.ncbi.nlm.nih.gov/clinvar/variation/207571/
NM_000243.2(MEFV):c.1061G>A	concordant	unreportable	unreportable	495749	0	1	SCV000696039	Uncertain significance	Laboratory Corpora	1/25/2018	MEFV	chr16	3299630	3299630	http://gnomad.b	1.12E-05	SAS	0.0003906759957	0		0	0	https://www.ncbi.nlm.nih.gov/clinvar/variation/495749/
NM_000152.3(GAA):c.2151C>T	concordant	unreportable	unreportable	325790	0	2	SCV000407292;S	Uncertain significance;Likel	Illumina Clinical Se	2016-10-18;2017-1	GAA	chr17	78087127	78087127		3.34E-05					0	0	https://www.ncbi.nlm.nih.gov/clinvar/variation/325790/
NM_005476.5(GNE):c.207C>T	concordant	unreportable	unreportable	288617	0	1	SCV000342784	Uncertain significance	EGL Genetic Diagn	11/3/2016	GNE	chr9	36246437	36246437	http://gnomad.b	2.80E-05	NFE	2.72E-05	0		0	0	https://www.ncbi.nlm.nih.gov/clinvar/variation/288617/
NM_002485.4(NBN):c.1659G>A	concordant	unreportable	unreportable	232738	0	1	SCV000634243	Uncertain significance	Invitae	10/5/2017	NBN	chr8	90965658	90965658	http://gnomad.b	1.95E-05	NFE	1.79E-05	0		0	0	https://www.ncbi.nlm.nih.gov/clinvar/variation/232738/
NM_001079802.1(FKTN):c.526T>G	concordant	unreportable	unreportable	288846	0	1	SCV000343079	Uncertain significance	EGL Genetic Diagn	6/30/2017	FKTN	chr9	108366652	108366652	http://gnomad.b	1.77E-05	EAS	0.0001159550011	0		0	0	https://www.ncbi.nlm.nih.gov/clinvar/variation/288846/
NM_002485.4(NBN):c.45A>C	concordant	unreportable	unreportable	461565	0	1	SCV000634309	Likely benign	Invitae	10/5/2017	NBN	chr8	90995076	90995076		1.12E-05					0	0	https://www.ncbi.nlm.nih.gov/clinvar/variation/461565/
NM_000466.2(PEX1):c.1369A>G	concordant	unreportable	unreportable	360929	0	1	SCV000470520	Uncertain significance	Illumina Clinical Se	10/18/2016	PEX1	chr7	92141008	92141008	http://gnomad.b	5.82E-05	FIN	9.24E-05	0		0	0	https://www.ncbi.nlm.nih.gov/clinvar/variation/360929/
NM_018941.3(CLN8):c.666G>A	concordant	unreportable	unreportable	457978	0	1	SCV000628993	Likely benign	Invitae	10/5/2017	CLN8	chr8	1728538	1728538	http://gnomad.b	1.96E-05	AMR	5.96E-05	0		0	0	https://www.ncbi.nlm.nih.gov/clinvar/variation/457978/
NM_002485.4(NBN):c.1902T>G	concordant	unreportable	unreportable	232887	0	1	SCV000562954	Likely benign	Invitae	3/14/2017	NBN	chr8	90960064	90960064		5.58E-06					0	0	https://www.ncbi.nlm.nih.gov/clinvar/variation/232887/

Supplemental Table 2

NM_000051.3(ATM):c.8355T>C	concordant	unreportable	unreportable	234230	0	3	SCV000537469;S	Likely benign;Likely benign	Color Genomics, In	2017-01-26;2017-1	ATM	chr11	108214035	108214035	http://gnomad.b	3.62E-05	EAS	0.0002319380001	0	0	0	https://www.ncbi.nlm.nih.gov/clinvar/variation/234230/
NM_000492.3(CFTR):c.3628A>G	concordant	unreportable	unreportable	439482	0	1	SCV000603025	Uncertain significance	ARUP Laboratories	6/30/2017	CFTR	chr7	117267735	117267735		3.85E-06				0	0	https://www.ncbi.nlm.nih.gov/clinvar/variation/439482/
NM_000030.2(AGXT):c.601G>A	concordant	unreportable	unreportable	335297	0	1	SCV000429366	Uncertain significance	Illumina Clinical Se	10/18/2016	AGXT	chr2	241813400	241813400	http://gnomad.b	1.79E-05	NFE	8.97E-06	0	0	0	https://www.ncbi.nlm.nih.gov/clinvar/variation/335297/
NM_000518.4(HBB):c.223G>A	concordant	unreportable	unreportable	495982	0	1	SCV000697096	Uncertain significance	Laboratory Corpora	1/25/2018	HBB	chr11	5247899	5247899	http://gnomad.b	1.87E-05	NFE	1.79E-05	0	0	0	https://www.ncbi.nlm.nih.gov/clinvar/variation/495982/
NM_017739.3(POMGNT1):c.1457G>A	concordant	unreportable	unreportable	286190	0	1	SCV000339521	Uncertain significance	EGL Genetic Diagn	6/30/2017	POMGNT1	chr1	46657852	46657852	http://gnomad.b	1.40E-05	EAS	5.80E-05	0	0	0	https://www.ncbi.nlm.nih.gov/clinvar/variation/286190/
NM_000492.3(CFTR):c.2552G>A	concordant	unreportable	unreportable	411119	0	1	SCV000552132	Uncertain significance	Invitae	3/14/2017	CFTR	chr7	117235045	117235045	http://gnomad.b	3.66E-05	EAS	0.000869867974	0	0	0	https://www.ncbi.nlm.nih.gov/clinvar/variation/411119/
NM_001079802.1(FKTN):c.766C>A	concordant	unreportable	unreportable	390531	0	2	SCV000708686;S	Uncertain significance;Likel	EGL Genetic Diagn	2017-06-30;2017-1	FKTN	chr9	108370218	108370218	http://gnomad.b	3.55E-05	NFE	4.48E-05	0	0	0	https://www.ncbi.nlm.nih.gov/clinvar/variation/390531/
NM_017739.3(POMGNT1):c.550C>T	concordant	unreportable	unreportable	471407	0	1	SCV000649972	Uncertain significance	Invitae	10/5/2017	POMGNT1	chr1	46660618	46660618	http://gnomad.b	5.04E-05	NFE	8.07E-05	0	0	0	https://www.ncbi.nlm.nih.gov/clinvar/variation/471407/
NM_000152.3(GAA):c.2481+128A>G	concordant	unreportable	unreportable	495666	0	1	SCV000695652	Uncertain significance	Laboratory Corpora	1/25/2018	GAA	chr17	78091676	78091676		2.50E-05				0	0	https://www.ncbi.nlm.nih.gov/clinvar/variation/495666/
NM_002485.4(NBN):c.80G>T	concordant	unreportable	unreportable	239208	0	1	SCV000287486	Uncertain significance	Invitae	6/10/2016	NBN	chr8	90995041	90995041	http://gnomad.b	8.37E-06	AFR	0.0001307019993	0	0	0	https://www.ncbi.nlm.nih.gov/clinvar/variation/239208/
NM_000136.2(FANCC):c.1444_1446de	concordant	unreportable	unreportable	422023	0	1	SCV000571381	Uncertain significance	GeneDx	11/28/2017	FANCC	chr9	97869434	97869437	http://gnomad.b	1.19E-05	AMR	5.96E-05	0	0	0	https://www.ncbi.nlm.nih.gov/clinvar/variation/422023/
NM_000051.3(ATM):c.846A>T	concordant	unreportable	unreportable	481079	0	1	SCV000687834	Uncertain significance	Color Genomics, In	12/21/2017	ATM	chr11	108115698	108115698	http://gnomad.b	5.57E-06	SAS	0.0003574450093	1	0	0	https://www.ncbi.nlm.nih.gov/clinvar/variation/481079/
NM_000136.2(FANCC):c.1589T>C	concordant	unreportable	unreportable	216284	0	2	SCV000279385;S	Uncertain significance;Unc	GeneDx;Invitae	2017-11-28;2017-1	FANCC	chr9	97864077	97864077	http://gnomad.b	2.85E-05	AFR	6.54E-05	0	0	0	https://www.ncbi.nlm.nih.gov/clinvar/variation/216284/
NM_000035.3(ALDOB):c.487G>A	concordant	unreportable	unreportable	502333	0	1	SCV000709024	Uncertain significance	EGL Genetic Diagn	6/30/2017	ALDOB	chr9	104189817	104189817	http://gnomad.b	2.26E-05	SAS	9.75E-05	0	0	0	https://www.ncbi.nlm.nih.gov/clinvar/variation/502333/
NM_138694.3(PKHD1):c.1980C>G	concordant	unreportable	unreportable	496516	0	1	SCV000699853	Uncertain significance	Laboratory Corpora	1/25/2018	PKHD1	chr6	51918034	51918034		1.39E-05				0	0	https://www.ncbi.nlm.nih.gov/clinvar/variation/496516/
NM_017739.3(POMGNT1):c.1050G>T	concordant	unreportable	unreportable	285324	0	1	SCV000338291	Uncertain significance	EGL Genetic Diagn	6/30/2017	POMGNT1	chr1	46659037	46659037		1.12E-05				0	0	https://www.ncbi.nlm.nih.gov/clinvar/variation/285324/
NM_002485.4(NBN):c.1147G>A	concordant	unreportable	unreportable	461491	0	1	SCV000634200	Uncertain significance	Invitae	10/5/2017	NBN	chr8	90967761	90967761	http://gnomad.b	5.58E-06	SAS	9.76E-05	0	0	0	https://www.ncbi.nlm.nih.gov/clinvar/variation/461491/
NM_001042432.1(CLN3):c.1028G>A	concordant	unreportable	unreportable	205096	0	1	SCV000240590	Uncertain significance	GeneDx	11/28/2017	CLN3	chr16	28493454	28493454		3.89E-05				1	0	https://www.ncbi.nlm.nih.gov/clinvar/variation/205096/
NM_000136.2(FANCC):c.598G>C	concordant	unreportable	unreportable	419678	0	1	SCV000567646	Uncertain significance	GeneDx	11/28/2017	FANCC	chr9	97912293	97912293	http://gnomad.b	1.90E-05	NFE	4.48E-05	0	0	0	https://www.ncbi.nlm.nih.gov/clinvar/variation/419678/
NM_000518.4(HBB):c.*67G>T	concordant	unreportable	unreportable	495970	0	1	SCV000697069	Uncertain significance	Laboratory Corpora	1/25/2018	HBB	chr11	5246761	5246761		1.17E-05				0	0	https://www.ncbi.nlm.nih.gov/clinvar/variation/495970/
NM_000051.3(ATM):c.372C>A	concordant	unreportable	unreportable	185849	0	2	SCV000682158;S	Likely benign;Likely benign	Color Genomics, In	2017-10-26;2017-1	ATM	chr11	108106437	108106437	http://gnomad.b	2.78E-05	NFE	6.28E-05	0	0	0	https://www.ncbi.nlm.nih.gov/clinvar/variation/185849/
NM_000391.3(TPP1):c.1116C>T	concordant	unreportable	unreportable	305508	0	1	SCV000373323	Uncertain significance	Illumina Clinical Se	10/18/2016	TPP1	chr11	6637265	6637265		5.60E-06				0	0	https://www.ncbi.nlm.nih.gov/clinvar/variation/305508/
NM_005476.5(GNE):c.1276A>C	concordant	unreportable	unreportable	284972	0	1	SCV000337799	Uncertain significance	EGL Genetic Diagn	6/30/2017	GNE	chr9	36227250	36227250	http://gnomad.b	8.40E-06	EAS	0.0002898889943	0	0	0	https://www.ncbi.nlm.nih.gov/clinvar/variation/284972/
NM_000492.3(CFTR):c.1210G>C	concordant	unreportable	unreportable	495893	0	1	SCV000696830	Uncertain significance	Laboratory Corpora	1/25/2018	CFTR	chr7	117188695	117188695		5.39E-05				0	0	https://www.ncbi.nlm.nih.gov/clinvar/variation/495893/
NM_024685.3(BBS10):c.1028G>A	concordant	unreportable	unreportable	310493	0	2	SCV000708828;S	Uncertain significance;Unc	EGL Genetic Diagn	2017-06-30;2016-1	BBS10	chr12	76740737	76740737	http://gnomad.b	9.51E-05	NFE	0.0001523929968	0	0	0	https://www.ncbi.nlm.nih.gov/clinvar/variation/310493/
NM_018941.3(CLN8):c.545C>T	concordant	unreportable	unreportable	450759	0	1	SCV000619373	Uncertain significance	GeneDx	11/28/2017	CLN8	chr8	1728417	1728417	http://gnomad.b	3.08E-05	AFR	6.53E-05	0	0	0	https://www.ncbi.nlm.nih.gov/clinvar/variation/450759/
NM_000023.2(SGCA):c.600G>A	concordant	unreportable	unreportable	390366	0	1	SCV000533178	Likely benign	GeneDx	11/28/2017	SGCA	chr17	48246468	48246468	http://gnomad.b	2.80E-06	AMR	5.96E-05	0	0	0	https://www.ncbi.nlm.nih.gov/clinvar/variation/390366/
NM_000051.3(ATM):c.3802G>A	concordant	unreportable	unreportable	216204	0	2	SCV000568179;S	Uncertain significance;Unc	GeneDx;Invitae	2017-11-28;2017-0	ATM	chr11	108155009	108155009	http://gnomad.b	5.57E-06	NFE	1.79E-05	0	0	0	https://www.ncbi.nlm.nih.gov/clinvar/variation/216204/
NM_000018.3(ACADVL):c.1220G>C	concordant	unreportable	unreportable	474878	0	1	SCV000654920	Uncertain significance	Invitae	10/5/2017	ACADVL	chr17	7127000	7127000	http://gnomad.b	3.40E-05	AMR	2.98E-05	0	0	0	https://www.ncbi.nlm.nih.gov/clinvar/variation/474878/
NM_002435.1(MPI):c.1175G>A	concordant	unreportable	unreportable	317142	0	1	SCV000393954	Uncertain significance	Illumina Clinical Se	10/18/2016	MPI	chr15	75189974	75189974	http://gnomad.b	8.96E-05	ASJ	0.0003045689955	0	0	0	https://www.ncbi.nlm.nih.gov/clinvar/variation/317142/
NM_017890.4(VPS13B):c.9906G>A	concordant	unreportable	unreportable	361085	0	1	SCV000470844	Uncertain significance	Illumina Clinical Se	10/18/2016	VPS13B	chr8	100847855	100847855	http://gnomad.b	1.68E-05	SAS	6.50E-05	0	0	0	https://www.ncbi.nlm.nih.gov/clinvar/variation/361085/
NM_138694.3(PKHD1):c.2372A>G	concordant	unreportable	unreportable	499345	0	1	SCV000704784	Uncertain significance	EGL Genetic Diagn	6/30/2017	PKHD1	chr6	51913325	51913325	http://gnomad.b	1.39E-05	AMR	0.0003871119989	0	0	0	https://www.ncbi.nlm.nih.gov/clinvar/variation/499345/
NM_000136.2(FANCC):c.262A>C	concordant	unreportable	unreportable	409661	0	2	SCV000594679;S	Uncertain significance;Unc	Genetic Services La	2017-07-05;2017-1	FANCC	chr9	98003014	98003014	http://gnomad.b	2.38E-06	NFE	8.99E-06	0	0	0	https://www.ncbi.nlm.nih.gov/clinvar/variation/409661/
NM_000051.3(ATM):c.4916C>T	concordant	unreportable	unreportable	482528	0	1	SCV000682230	Uncertain significance	Color Genomics, In	10/26/2017	ATM	chr11	108168020	108168020	http://gnomad.b	2.23E-05	EAS	0.0001743580069	0	0	0	https://www.ncbi.nlm.nih.gov/clinvar/variation/482528/
NM_000051.3(ATM):c.4358T>G	concordant	unreportable	unreportable	141941	0	2	SCV000682198;S	Uncertain significance;Unc	Color Genomics, In	2017-10-26;2017-1	ATM	chr11	108160450	108160450	http://gnomad.b	1.67E-05	EAS	5.80E-05	0	0	0	https://www.ncbi.nlm.nih.gov/clinvar/variation/141941/
NM_017890.4(VPS13B):c.3865A>G	concordant	unreportable	unreportable	361060	0	1	SCV000470801	Uncertain significance	Illumina Clinical Se	10/18/2016	VPS13B	chr8	100494025	100494025	http://gnomad.b	2.24E-05	EAS	0.00122106995	0	0	0	https://www.ncbi.nlm.nih.gov/clinvar/variation/361060/
NM_000035.3(ALDOB):c.226G>A	concordant	unreportable	unreportable	500239	0	1	SCV000706097	Uncertain significance	EGL Genetic Diagn	6/30/2017	ALDOB	chr9	104192135	104192135	http://gnomad.b	0.0001382103731	EAS	0.00115955004	0	0	0	https://www.ncbi.nlm.nih.gov/clinvar/variation/500239/
NM_000152.3(GAA):c.1410C>T	concordant	unreportable	unreportable	285294	0	1	SCV000338252	Uncertain significance	EGL Genetic Diagn	6/30/2017	GAA	chr17	78083827	78083827	http://gnomad.b	4.17E-05	EAS	0.0002321259963	0	0	0	https://www.ncbi.nlm.nih.gov/clinvar/variation/285294/
NM_017890.4(VPS13B):c.1954A>C	concordant	unreportable	unreportable	361044	0	1	SCV000470774	Uncertain significance	Illumina Clinical Se	10/18/2016	VPS13B	chr8	100160179	100160179		8.40E-06				0	0	https://www.ncbi.nlm.nih.gov/clinvar/variation/361044/
NM_000642.2(AGL):c.2591G>A	concordant	unreportable	unreportable	456475	0	1	SCV000626702	Uncertain significance	Invitae	10/5/2017	AGL	chr1	100350169	100350169	http://gnomad.b	2.79E-05	EAS	0.000232018996	0	0	0	https://www.ncbi.nlm.nih.gov/clinvar/variation/456475/
NM_000441.1(SLC26A4):c.1110A>G	concordant	unreportable	unreportable	358500	0	1	SCV000466099	Uncertain significance	Illumina Clinical Se	10/18/2016	SLC26A4	chr7	107329606	107329606	http://gnomad.b	2.79E-05	AMR	2.98E-05	0	0	0	https://www.ncbi.nlm.nih.gov/clinvar/variation/358500/
NM_000051.3(ATM):c.6762C>T	concordant	unreportable	unreportable	414582	0	1	SCV000558397	Likely benign	Invitae	3/14/2017	ATM	chr11	108196226	108196226	http://gnomad.b	5.57E-06	SAS	0.0002274500002	0	0	0	https://www.ncbi.nlm.nih.gov/clinvar/variation/414582/
NM_018941.3(CLN8):c.510G>A	concordant	unreportable	unreportable	499264	0	1	SCV000704681	Uncertain significance	EGL Genetic Diagn	6/30/2017	CLN8	chr8	1719730	1719730	http://gnomad.b	2.80E-05	SAS	9.75E-05	0	0	0	https://www.ncbi.nlm.nih.gov/clinvar/variation/499264/
NM_020533.2(MCOLN1):c.1707-14C>	concordant	unreportable	unreportable	330505	0	1	SCV000415623	Uncertain significance	Illumina Clinical Se	10/18/2016	MCOLN1	chr19	7598631	7598631	http://gnomad.b	3.09E-05	SAS	0.0004548699944	0	0	0	https://www.ncbi.nlm.nih.gov/clinvar/variation/330505/
NM_138694.3(PKHD1):c.5199C>T	concordant	unreportable	unreportable	289886	0	1	SCV000344320	Uncertain significance	EGL Genetic Diagn	6/30/2017	PKHD1	chr6	51889409	51889409	http://gnomad.b	7.23E-05	AFR	0.0007195189828	0	0	0	https://www.ncbi.nlm.nih.gov/clinvar/variation/289886/
NM_000152.3(GAA):c.310G>A	concordant	unreportable	unreportable	498906	0	1	SCV000704155	Uncertain significance	EGL Genetic Diagn	6/30/2017	GAA	chr17	78078695	78078695	http://gnomad.b	2.22E-05	AFR	6.67E-05	0	0	0	https://www.ncbi.nlm.nih.gov/clinvar/variation/498906/
NM_000487.5(ARSA):c.369C>T	concordant	unreportable	unreportable	342238	0	1	SCV000439441	Uncertain significance	Illumina Clinical Se	10/18/2016	ARSA	chr22	51065690	51065690		1.13E-05				0	0	https://www.ncbi.nlm.nih.gov/clinvar/variation/342238/
NM_000152.3(GAA):c.783C>T	concordant	unreportable	unreportable	456439	0	1	SCV000626641	Likely benign	Invitae	10/5/2017	GAA	chr17	78081446	78081446	http://gnomad.b	1.39E-05	NFE	5.38E-05	0	0	0	https://www.ncbi.nlm.nih.gov/clinvar/variation/456439/
NM_001079802.1(FKTN):c.722_724de	concordant	unreportable	unreportable	459223	0	1	SCV000630821	Uncertain significance	Invitae	10/5/2017	FKTN	chr9	108370168	108370171		1.42E-05				0	0	https://www.ncbi.nlm.nih.gov/clinvar/variation/459223/
NM_000018.3(ACADVL):c.1019G>T	concordant	unreportable	unreportable	426177	1	1	SCV000576556;S	Likely pathogenic;Uncertai	GeneDx;Invitae	2017-11-28;2017-1	ACADVL	chr17	7126126	7126126		5.66E-06				0	0	https://www.ncbi.nlm.nih.gov/clinvar/variation/426177/
NM_000232.4(SGCB):c.543C>T	concordant	unreportable	unreportable	286223	0	1	SCV000339564	Uncertain significance	EGL Genetic Diagn	6/30/2017	SGCB	chr4	52894974	52894974	http://gnomad.b	1.12E-05	AFR	6.54E-05	0	0	0	https://www.ncbi.nlm.nih.gov/clinvar/variation/286223/
NM_001042432.1(CLN3):c.-1G>T	concordant	unreportable	unreportable	420263	0	2	SCV000706034;S	Uncertain significance;Unc	EGL Genetic Diagn	2017-06-30;2017-1	CLN3	chr16	28503081	28503081	http://gnomad.b	3.33E-05	AMR	0.0006852169754	0	0	0	https://www.ncbi.nlm.nih.gov/clinvar/variation/420263/
NM_138694.3(PKHD1):c.10150A>C	concordant	unreportable	unreportable	500246	0	1	SCV000706106	Uncertain significance	EGL Genetic Diagn	6/30/2017	PKHD1	chr6	51609189	51609189	http://gnomad.b	1.67E-05	AMR	2.98E-05	0	0	0	https://www.ncbi.nlm.nih.gov/clinvar/variation/500246/
NM_000152.3(GAA):c.1053G>T	concordant	unreportable	unreportable	286713	0	1	SCV000340259	Uncertain significance	EGL Genetic Diagn	6/30/2017	GAA	chr17	78082186	78082186	http://gnomad.b	2.78E-05	SAS	0.0001624330034	0	0	0	https://www.ncbi.nlm.nih.gov/clinvar/variation/286713/
NM_000051.3(ATM):c.2189G>A	concordant	unreportable	unreportable	141238	0	3	SCV000682038;S	Uncertain significance;Unc	Color Genomics, In	2017-10-26;2017-0	ATM	chr11	108127006	108127006		1.11E-05				0	0	https://www.ncbi.nlm.nih.gov/clinvar/variation/141238/
NM_138694.3(PKHD1):c.2079G>T	concordant	unreportable	unreportable	357444	0	2	SCV000708190;S	Uncertain significance;Unc	EGL Genetic Diagn	2017-06-30;2016-1	PKHD1	chr6	51917935	51917935	http://gnomad.b	0.0001195387472	AMR	8.93E-05	0	0	0	https://www.ncbi.nlm.nih.gov/clinvar/variation/357444/
NM_000051.3(ATM):c.6246A>G	concordant	unreportable	unreportable	232881	0	2	SCV000682318;S	Likely benign;Uncertain sig	Color Genomics, In	2017-10-26;2017-1	ATM	chr11	108188147	108188147	http://gnomad.b	2.78E-05	SAS	0.0001299459982	0	0	0	https://www.ncbi.nlm.nih.gov/clinvar/variation/232881/
NM_000051.3(ATM):c.2150G>A	concordant	unreportable	unreportable	420250	0	1	SCV000568987	Uncertain significance	GeneDx	11/28/2017	ATM	chr11	108126967	108126967	http://gnomad.b	2.51E-05	SAS	6.52E-05	0	0	0	https://www.ncbi.nlm.nih.gov/clinvar/variation/420250/
NM_000051.3(ATM):c.5633C>T	concordant	unreportable	unreportable	426534	0	3	SCV000687631;S	Uncertain significance;Unc	Color Genomics, In	2017-12-21;2017-1	ATM	chr11	108175538	108175538	http://gnomad.b	5.57E-06	SAS	0.000162686003	1	0	0	https://www.ncbi.nlm.nih.gov/clinvar/variation/426534/
NM_000051.3(ATM):c.741T>C	concordant	unreportable	unreportable	231311	0	2	SCV000682413;S	Likely benign;Likely benign	Color Genomics, In	2017-10-26;2017-0	ATM	chr11	108115593	108115593		5.57E-06				0	0	https://www.ncbi.nlm.nih.gov/clinvar/variation/231311/
NM_000492.3(CFTR):c.1186A>T	concordant	unreportable	unreportable	411126	0	1	SCV000552140	Uncertain significance	Invitae	3/14/2017	CFTR	chr7	117182139	117182139	http://gnomad.b	9.63E-06	SAS	0.0001625069999	0	0	0	https://www.ncbi.nlm.nih.gov/clinvar/variation/411126/
NM_000492.3(CFTR):c.662C>T	concordant	unreportable	unreportable	495955	0	1	SCV000697041	Uncertain significance	Laboratory Corpora	1/25/2018	CFTR	chr7	117175384	117175384	http://gnomad.b	1.35E-05	NFE	1.79E-05	0	0	0	https://www.ncbi.nlm.nih.gov/clinvar/variation/495955/
NM_001042432.1(CLN3):c.837+5G>A	concordant	unreportable	unreportable	318720	0	1	SCV000396325	Uncertain significance	Illumina Clinical Se	10/18/2016	CLN3	chr16	28493942	28493942	http://gnomad.b	5.28E-05	SAS	0.000259908993	0	0	0	https://www.ncbi.nlm.nih.gov/clinvar/variation/318720/
NM_000049.2(ASPA):c.905C>T	concordant	unreportable	unreportable	322668	0	1	SCV000402021	Uncertain significance	Illumina Clinical Se	10/18/2016	ASPA	chr17	3402345	3402345	http://gnomad.b	9.46E-06	NFE	8.96E-06	0	0	0	https://www.ncbi.nlm.nih.gov/clinvar/variation/322668/
NM_138694.3(PKHD1):c.4130A>C	concordant	unreportable	unreportable	286356	0	1	SCV000339758	Uncertain significance	EGL Genetic Diagn	6/30/2017	PKHD1	chr6	51890478	51890478	http://gnomad.b	3.89E-05	NFE	1.79E-05	0	0	0	https://www.ncbi.nlm.nih.gov/clinvar/variation/286356/
NM_000057.2(BLM):c.3949G>A	concordant	unreportable	unreportable	180588	0	1	SCV000623323	Uncertain significance	Invitae	10/5/2017	BLM	chr15	91354509	91354509	http://gnomad.b	6.42E-05	AMR	0.001369860023	0	0	0	https://www.ncbi.nlm.nih.gov/clinvar/variation/180588/
NM_002485.4(NBN):c.123delC	concordant	reportable	reportable	141631	3	0	SCV000685705;S	Pathogenic;Pathogenic;Pat	Color Genomics, In	2017-10-26;2017-1	NBN	chr8	90994997	90994998	http://gnomad.b	1.12E-05	NFE	8.95E-06	0	0	0	https://www.ncbi.nlm.nih.gov/clinvar/variation/141631/
NM_000051.3(ATM):c.5076A>C	concordant	unreportable	unreportable	246410	0	2	SCV000293954;S	Uncertain significance;Unc	GeneDx;Invitae	2017-11-28;2017-1	ATM	chr11	108170511	108170511	http://gnomad.b	1.11E-05	NFE	1.79E-05	0	0	0	https://www.ncbi.nlm.nih.gov/clinvar/variation/246410/
NM_000136.2(FANCC):c.109C>G	concordant	unreportable	unreportable	219747	0	1	SCV000259793	Uncertain significance	Invitae	1/6/2016	FANCC	chr9	98011465	98011465		4.76E-06				0	0	https://www.ncbi.nlm.nih.gov/clinvar/variation/219747/
NM_000441.1(SLC26A4):c.1947C>T	concordant	unreportable	unreportable	227951	0	1	SCV000270851	Likely benign	Laboratory for Mol	4/26/2016	SLC26A4	chr7	107342415	107342415	http://gnomad.b	3.63E-05	AMR	0.0004769290099	0	0	0	https://www.ncbi.nlm.nih.gov/clinvar/variation/227951/
NM_000271.4(NPC1):c.2349G>C	concordant	unreportable	unreportable	326262	0	1	SCV000407864	Uncertain significance	Illumina Clinical Se	10/18/2016	NPC1	chr18	21121294	21121294	http://gnomad.b	2.86E-05	SAS	0.0008121629944	0	0	0	https://www.ncbi.nlm.nih.gov/clinvar/variation/326262/
NM_000051.3(ATM):c.2418G>T	concordant	unreportable	unreportable	140813	0	2	SCV000682053;S	Uncertain significance;Unc	Color Genomics, In	2017-10-26;2017-1	ATM	chr11	108129754	108129754	http://gnomad.b	3.06E-05	NFE	1.79E-05	0	0	0	https://www.ncbi.nlm.nih.gov/clinvar/variation/140813/
NM_000051.3(ATM):c.8138G>A	concordant	unreportable	unreportable	231772	0	1	SCV000682456	Uncertain significance	Color Genomics, In	10/26/2017	ATM	chr11	108205823	108205823		8.35E-06				0	0	https://www.ncbi.nlm.nih.gov/clinvar/variation/231772/
NM_002485.4(NBN):c.1066G>A	concordant	unreportable	unreportable	230223	0	2	SCV000690631;S	Uncertain significance;Unc	Color Genomics, In	2017-12-21;2017-1	NBN	chr8	90971011	90971011	http://gnomad.b	1.39E-05	NFE	3.58E-05	0	0	0	https://www.ncbi.nlm.nih.gov/clinvar/variation/230223/
NM_024649.4(BBS1):c.1147C>T	concordant	unreportable	unreportable	387598	0	1	SCV000529690	Uncertain significance	GeneDx	11/28/2017	BBS1	chr11	66293630	66293630	http://gnomad.b	0.0001337568968	SAS	0.001007150044	0	0	0	https://www.ncbi.nlm.nih.gov/clinvar/variation/387598/
NM_000391.3(TPP1):c.18-10G>T	concordant	unreportable	unreportable	383237	0	1	SCV000523560	Likely benign	GeneDx	11/28/2017	TPP1	chr11	6640508	6640508	http://gnomad.b	2.52E-05	AMR	0.0001191109986	0	0	0	https://www.ncbi.nlm.nih.gov/clinvar/variation/383237/
NM_000182.4(HADHA):c.199G>C	concordant	unreportable	unreportable	501894	0	1	SCV000708417	Uncertain significance	EGL Genetic Diagn	6/30/2017	HADHA	chr2	26459838	26459838	http://gnomad.b	1.12E-05	AMR	0.0001191190022	0	0	0	https://www.ncbi.nlm.nih.gov/clinvar/variation/501894/
NM_004646.3(NPHS1):c.428A>T	concordant	unreportable	unreportable	328879	0	1	SCV000411586	Uncertain significance	Illumina Clinical Se	10/18/2016	NPHS1	chr19	36341961	36341961	http://gnomad.b	4.46E-05	NFE	3.58E-05	0	0	0	https://www.ncbi.nlm.nih.gov/clinvar/variation/328879/
NM_000152.3(GAA):c.2154C>T	concordant	unreportable	unreportable	456392	0	1	SCV000626548	Likely benign	Invitae	10/5/2017	GAA	chr17	78087130	78087130		1.11E-05				0	0	https://www.ncbi.nlm.nih.gov/clinvar/variation/456392/
NM_000492.3(CFTR):c.1950C>T	concordant	unreportable	unreportable	495906	0	1	SCV000696880	Uncertain significance	Laboratory Corpora	1/25/2018	CFTR	chr7	117232171	117232171	http://gnomad.b	3.66E-05	ASJ	0.0001016469978	0	0	0	https://www.ncbi.nlm.nih.gov/clinvar/variation/495906/
NM_004328.4(BCS1L):c.321-12G>A	concordant	unreportable	unreportable	334358	0	1	SCV000427436	Uncertain significance	Illumina Clinical Se	10/18/2016	BCS1L	chr2	219526117	219526117	http://gnomad.b	7.81E-05	EAS	0.0002319110063	0	0	0	https://www.ncbi.nlm.nih.gov/clinvar/variation/334358/
NM_000051.3(ATM):c.4878T>C	concordant	unreportable	unreportable	187337	0	1	SCV000622538	Likely benign	Invitae	10/5/2017	ATM	chr11	108165755	108165755	http://gnomad.b	1.67E-05	AMR	0.0001191330011	0	0	0	https://www.ncbi.nlm.nih.gov/clinvar/variation/187337/
NM_000136.2(FANCC):c.1290C>T	concordant	unreportable	unreportable	220495	0	1	SCV000261080	Likely benign	Invitae	10/5/2017	FANCC	chr9	97873784	97873784	http://gnomad.b	3.33E-05	FIN	0.0003605230013	0	0	0	https://www.ncbi.nlm.nih.gov/clinvar/variation/220495/
NM_138694.3(PKHD1):c.904G>A	concordant	unreportable	unreportable	357453	0	1	SCV000464122	Uncertain significance	Illumina Clinical Se	10/18/2016	PKHD1	chr6	51929825	51929825	http://gnomad.b	5.56E-06	SAS	0.0001299550058	1	0	0	https://www.ncbi.nlm.nih.gov/clinvar/variation/357453/
NM_000136.2(FANCC):c.1508C>T	concordant	unreportable	unreportable	234312	0	1	SCV000278975	Uncertain significance	GeneDx	11/28/2017	FANCC	chr9	97869373	97869373	http://gnomad.b	3.57E-05	NFE	1.79E-05	0	0	0	https://www.ncbi.nlm.nih.gov/clinvar/variation/234312/
NM_000414.3(HSD17B4):c.1280C>T	concordant	unreportable	unreportable	439793	0	3	SCV000603970;S	Uncertain significance;Unc	ARUP Laboratories	2017-06-30;2017-0	HSD17B4	chr5	118842531	118842531	http://gnomad.b	9.58E-05	AMR	0.002354690107	0	0	0	https://www.ncbi.nlm.nih.gov/clinvar/variation/439793/
NM_000642.2(AGL):c.4341A>G	concordant	unreportable	unreportable	456504	0	1	SCV000626754	Uncertain significance	Invitae	10/5/2017	AGL	chr1	100382047	100382047	http://gnomad.b	6.14E-05	EAS	0.0001741549931	0	0	0	https://www.ncbi.nlm.nih.gov/clinvar/variation/456504/
NM_000051.3(ATM):c.283C>A	concordant	unreportable	unreportable	181957	0	3	SCV000537548;S	Uncertain significance;Unc	Color Genomics, In	2017-12-21;2017-0	ATM	chr11	108100002	108100002	http://gnomad.b	9.19E-05	EAS	0.0001742159948	1	0	0	https://www.ncbi.nlm.nih.gov/clinvar/variation/181957/
NM_000396.3(CTSK):c.551A>G	concordant	unreportable	unreportable	292584	0	1	SCV000348279	Uncertain significance	Illumina Clinical Se	10/18/2016	CTSK	chr1	150776564	150776564	http://gnomad.b	8.96E-06	NFE	8.95E-06	0	0	0	https://www.ncbi.nlm.nih.gov/clinvar/variation/292584/
NM_000642.2(AGL):c.1895T>C	concordant	unreportable	unreportable	456460	0	1	SCV000626682	Uncertain significance	Invitae	10/5/2017	AGL	chr1	100346347	100346347	http://gnomad.b	6.42E-05	AFR	0.0005881579709	0	0	0	https://www.ncbi.nlm.nih.gov/clinvar/variation/456460/
NM_024685.3(BBS10):c.389C>T	concordant	unreportable	unreportable	434486	0	1	SCV000593591	Uncertain significance	Genetic Services La	7/5/2017	BBS10	chr12	76741376	76741376	http://gnomad.b	1.68E-05	FIN	4.50E-05	0	0	0	https://www.ncbi.nlm.nih.gov/clinvar/variation/434486/
NM_002485.4(NBN):c.83G>A	concordant	unreportable	unreportable	230463	0	1	SCV000685832	Uncertain significance	Color Genomics, In	10/26/2017	NBN	chr8	90995038	90995038		8.37E-06				0	0	https://www.ncbi.nlm.nih.gov/clinvar/variation/230463/
NM_000152.3(GAA):c.685C>T	concordant	unreportable	unreportable	286250	0	1	SCV000339602	Uncertain significance	EGL Genetic Diagn	6/30/2017	GAA	chr17	78079686	78079686	http://gnomad.b	3.06E-05	SAS	3.26E-05	0	0	0	https://www.ncbi.nlm.nih.gov/clinvar/variation/286250/
NM_000051.3(ATM):c.1364T>C	concordant	unreportable	unreportable	230788	0	2	SCV000622251;S	Uncertain significance;Unc	Invitae;Laboratory	2017-10-05;2018-0	ATM	chr11	108121556	108121556		1.39E-05				0	0	https://www.ncbi.nlm.nih.gov/clinvar/variation/230788/
NM_000051.3(ATM):c.7375C>G	concordant	unreportable	unreportable	181982	0	3	SCV000682409;S	Uncertain significance;Unc	Color Genomics, In	2017-10-26;2017-1	ATM	chr11	108201008	108201008	http://gnomad.b	3.34E-05	AMR	5.96E-05	0	0	0	https://www.ncbi.nlm.nih.gov/clinvar/variation/181982/
NM_000414.3(HSD17B4):c.1596T>C	concordant	unreportable	unreportable	350467	0	1	SCV000452135	Uncertain significance	Illumina Clinical Se	10/18/2016	HSD17B4	chr5	118861634	118861634	http://gnomad.b	2.82E-05				0	0	https://www.ncbi.nlm.nih.gov/clinvar/variation/350467/
NM_000018.3(ACADVL):c.1333-7G>T	concordant	unreportable	unreportable	474883	0	1	SCV000654928	Likely benign	Invitae	10/5/2017	ACADVL	chr17	7127280	7127280	http://gnomad.b	2.83E-06	AMR	8.93E-05	0	0	0	https://www.ncbi.nlm.nih.gov/clinvar/variation/474883/

Supplemental Table 2

NM_000051.3(ATM):c.1363G>A	concordant	unreportable	unreportable	229981	0		1	SCV000687304	Uncertain significance	Color Genomics, In	12/21/2017	ATM	chr11	108121555	108121555	http://gnomad.b	1.95E-05	SAS	6.50E-05	0		0	0	https://www.ncbi.nlm.nih.gov/clinvar/variation/229981/
NM_000051.3(ATM):c.2922-8T>C	concordant	unreportable	unreportable	490505	0		1	SCV000687434	Likely benign	Color Genomics, In	12/21/2017	ATM	chr11	108141970	108141970	http://gnomad.b	5.57E-06	EAS	0.0001746829948	0		0	0	https://www.ncbi.nlm.nih.gov/clinvar/variation/490505/
NM_000243.2(MEFV):c.1513G>T	concordant	unreportable	unreportable	319112	0		1	SCV000396767	Uncertain significance	Illumina Clinical Se	10/18/2016	MEFV	chr16	3297090	3297090	http://gnomad.b	4.48E-05	NFE	1.79E-05	0		0	0	https://www.ncbi.nlm.nih.gov/clinvar/variation/319112/
NM_033056.3(PCDH15):c.5726G>T	concordant	unreportable	unreportable	300166	0		1	SCV000363099	Uncertain significance	Illumina Clinical Se	10/18/2016	PCDH15	chr10	55581760	55581760	http://gnomad.b	4.11E-05	EAS	0.00104360003	0		0	0	https://www.ncbi.nlm.nih.gov/clinvar/variation/300166/
NM_000051.3(ATM):c.2519A>T	concordant	unreportable	unreportable	185978	0		2	SCV000687393;S(	Uncertain significance;Unc	Color Genomics, In	2017-12-21;2017-1	ATM	chr11	108137950	108137950	http://gnomad.b	1.39E-05	NFE	8.95E-06	0		0	0	https://www.ncbi.nlm.nih.gov/clinvar/variation/185978/
NM_000051.3(ATM):c.3153+20dupT	concordant	unreportable	unreportable	419885	0		2	SCV000682106;S(	Likely benign;Likely benign	Color Genomics, In	2017-10-26;2017-1	ATM	chr11	108143347	108143347	http://gnomad.b	5.01E-05	AMR	0.0002085570013	0		0	0	https://www.ncbi.nlm.nih.gov/clinvar/variation/419885/
NM_138694.3(PKHD1):c.2244G>A	concordant	unreportable	unreportable	285114	0		1	SCV000337993	Uncertain significance	EGL Genetic Diagn	6/30/2017	PKHD1	chr6	51914990	51914990		3.06E-05					0	0	https://www.ncbi.nlm.nih.gov/clinvar/variation/285114/
NM_000391.3(TPP1):c.1496C>G	concordant	unreportable	unreportable	457943	0		1	SCV000628906	Uncertain significance	Invitae	10/5/2017	TPP1	chr11	6636152	6636152	http://gnomad.b	3.64E-05	AMR	0.0001786670036	0		0	0	https://www.ncbi.nlm.nih.gov/clinvar/variation/457943/
NM_000023.2(SGCA):c.132G>A	concordant	unreportable	unreportable	288901	0		1	SCV000343147	Uncertain significance	EGL Genetic Diagn	6/30/2017	SGCA	chr17	48244823	48244823	http://gnomad.b	3.08E-05	SAS	3.25E-05	0		0	0	https://www.ncbi.nlm.nih.gov/clinvar/variation/288901/
NM_000051.3(ATM):c.2919A>G	concordant	unreportable	unreportable	127361	0		2	SCV000149075;S(	Uncertain significance;Likel	GeneDx;Invitae	2017-11-28;2017-1	ATM	chr11	108141871	108141871	http://gnomad.b	1.67E-05	SAS	6.50E-05	0		0	0	https://www.ncbi.nlm.nih.gov/clinvar/variation/127361/
NM_000051.3(ATM):c.3154-5C>T	concordant	unreportable	unreportable	141292	0		1	SCV000558392	Likely benign	Invitae	10/5/2017	ATM	chr11	108143444	108143444	http://gnomad.b	5.29E-05	EAS	0.0001741760061	0		0	0	https://www.ncbi.nlm.nih.gov/clinvar/variation/141292/
NM_000051.3(ATM):c.4606A>G	concordant	unreportable	unreportable	127393	0		2	SCV000149107;S(	Uncertain significance;Unc	GeneDx;Invitae	2017-11-28;2017-1	ATM	chr11	108163515	108163515	http://gnomad.b	8.91E-05	AMR	0.001489339978	0		0	0	https://www.ncbi.nlm.nih.gov/clinvar/variation/127393/
NM_000027.3(AGA):c.508-2A>G	concordant	reportable	reportable	373678	1		0	SCV000492293	Likely pathogenic	GeneDx	12/21/2016	AGA	chr4	178358675	178358675		2.07E-05					0	0	https://www.ncbi.nlm.nih.gov/clinvar/variation/373678/
NM_000152.3(GAA):c.2455C>T	concordant	unreportable	unreportable	456402	0		1	SCV000626571	Uncertain significance	Invitae	10/5/2017	GAA	chr17	78091522	78091522	http://gnomad.b	4.45E-05	AMR	9.03E-05	0		0	0	https://www.ncbi.nlm.nih.gov/clinvar/variation/456402/
NM_000228.2(LAMB3):c.2419C>A	concordant	unreportable	unreportable	295091	0		1	SCV000353614	Uncertain significance	Illumina Clinical Se	10/18/2016	LAMB3	chr1	209796464	209796464	http://gnomad.b	2.24E-05	NFE	0.0001075330001	0		0	0	https://www.ncbi.nlm.nih.gov/clinvar/variation/295091/
NM_000051.3(ATM):c.3504C>T	concordant	unreportable	unreportable	230071	0		2	SCV000512159;S(	Likely benign;Likely benign	GeneDx;Invitae	2017-11-28;2016-0	ATM	chr11	108151823	108151823	http://gnomad.b	8.35E-06	NFE	8.96E-06	0		0	0	https://www.ncbi.nlm.nih.gov/clinvar/variation/230071/
NM_000051.3(ATM):c.6497T>C	concordant	unreportable	unreportable	489574	0		1	SCV000682342	Uncertain significance	Color Genomics, In	10/26/2017	ATM	chr11	108192072	108192072	http://gnomad.b	2.78E-05	FIN	0.0003139009932	0		0	0	https://www.ncbi.nlm.nih.gov/clinvar/variation/489574/
NM_000051.3(ATM):c.3236C>G	concordant	unreportable	unreportable	407615	0		1	SCV000546939	Uncertain significance	Invitae	3/14/2017	ATM	chr11	108143531	108143531	http://gnomad.b	5.57E-06	NFE	8.97E-06	0		0	0	https://www.ncbi.nlm.nih.gov/clinvar/variation/407615/
NM_000227.3(LAMA3):c.4762C>T	concordant	unreportable	unreportable	326346	0		1	SCV000408033	Uncertain significance	Illumina Clinical Se	10/18/2016	LAMA3	chr18	21530070	21530070	http://gnomad.b	5.32E-05	EAS	0.0005798449856	0		0	0	https://www.ncbi.nlm.nih.gov/clinvar/variation/326346/
NM_000051.3(ATM):c.6507C>G	concordant	unreportable	unreportable	215589	0		3	SCV000687708;S(	Likely benign;Likely benign	Color Genomics, In	2017-12-21;2017-1	ATM	chr11	108192082	108192082	http://gnomad.b	1.95E-05	NFE	8.95E-06	0		0	0	https://www.ncbi.nlm.nih.gov/clinvar/variation/215589/
NM_138694.3(PKHD1):c.8755C>T	concordant	unreportable	unreportable	283340	0		1	SCV000335360	Uncertain significance	EGL Genetic Diagn	6/30/2017	PKHD1	chr6	51619624	51619624		8.34E-06					0	0	https://www.ncbi.nlm.nih.gov/clinvar/variation/283340/
NM_000492.3(CFTR):c.3789T>C	concordant	unreportable	unreportable	439484	0		2	SCV000603033;S(	Likely benign;Uncertain sig	ARUP Laboratories	2017-06-30;2018-0	CFTR	chr7	117282563	117282563	http://gnomad.b	2.31E-05	AMR	0.0006564810174	0		0	0	https://www.ncbi.nlm.nih.gov/clinvar/variation/439484/
NM_000352.3(ABCC8):c.2256-3C>T	concordant	unreportable	unreportable	303774	0		1	SCV000369333	Uncertain significance	Illumina Clinical Se	10/18/2016	ABCC8	chr11	17436889	17436889		1.03E-05					0	0	https://www.ncbi.nlm.nih.gov/clinvar/variation/303774/
NM_017890.4(VPS13B):c.9175G>A	concordant	unreportable	unreportable	361078	0		1	SCV000470834	Uncertain significance	Illumina Clinical Se	10/18/2016	VPS13B	chr8	100833627	100833627	http://gnomad.b	4.48E-05	NFE	8.97E-05	0		0	0	https://www.ncbi.nlm.nih.gov/clinvar/variation/361078/
NM_138694.3(PKHD1):c.2639G>A	concordant	unreportable	unreportable	282054	0		2	SCV000333228;S(	Uncertain significance;Unc	EGL Genetic Diagn	2017-06-30;2017-1	PKHD1	chr6	51909840	51909840	http://gnomad.b	5.00E-05	EAS	0.0001161039982	0		0	0	https://www.ncbi.nlm.nih.gov/clinvar/variation/282054/
NM_000051.3(ATM):c.9068G>A	concordant	unreportable	unreportable	236797	0		1	SCV000283104	Uncertain significance	Invitae	3/14/2017	ATM	chr11	108236132	108236132	http://gnomad.b	2.78E-06	EAS	5.80E-05	0		0	0	https://www.ncbi.nlm.nih.gov/clinvar/variation/236797/
NM_000478.4(ALPL):c.1171dupC	concordant	reportable	reportable	504232	1		0	SCV000710544	Likely pathogenic	GeneDx	3/26/2018	ALPL	chr1	21902393	21902393	http://gnomad.b	2.82E-06	AFR	6.53E-05	0		0	0	https://www.ncbi.nlm.nih.gov/clinvar/variation/504232/
NM_000051.3(ATM):c.6645T>G	concordant	unreportable	unreportable	420454	0		1	SCV000569295	Uncertain significance	GeneDx	11/28/2017	ATM	chr11	108196109	108196109		5.57E-06					0	0	https://www.ncbi.nlm.nih.gov/clinvar/variation/420454/
NM_017890.4(VPS13B):c.2823C>T	concordant	unreportable	unreportable	361050	0		1	SCV000470784	Uncertain significance	Illumina Clinical Se	10/18/2016	VPS13B	chr8	100287481	100287481	http://gnomad.b	5.04E-05	AFR	6.55E-05	0		0	0	https://www.ncbi.nlm.nih.gov/clinvar/variation/361050/
NM_002485.4(NBN):c.1845+3A>G	concordant	unreportable	unreportable	136038	0		1	SCV000166514	Uncertain significance	Invitae	10/5/2017	NBN	chr8	90965469	90965469	http://gnomad.b	2.79E-06	AFR	6.54E-05	0		0	0	https://www.ncbi.nlm.nih.gov/clinvar/variation/136038/
NM_000152.3(GAA):c.1375G>C	concordant	unreportable	unreportable	497673	0		1	SCV000702313	Uncertain significance	EGL Genetic Diagn	6/30/2017	GAA	chr17	78083792	78083792	http://gnomad.b	2.22E-05	AFR	6.56E-05	0		0	0	https://www.ncbi.nlm.nih.gov/clinvar/variation/497673/
NM_000051.3(ATM):c.4910A>G	concordant	unreportable	unreportable	407499	0		1	SCV000546736	Uncertain significance	Invitae	10/5/2017	ATM	chr11	108168014	108168014	http://gnomad.b	8.91E-05	SAS	0.0008449240122	0		0	0	https://www.ncbi.nlm.nih.gov/clinvar/variation/407499/
NM_000030.2(AGXT):c.352C>T	concordant	unreportable	unreportable	204095	0		1	SCV000338371	Uncertain significance	EGL Genetic Diagn	6/30/2017	AGXT	chr2	241808773	241808773	http://gnomad.b	3.88E-05	SAS	0.0001174540012	0		0	0	https://www.ncbi.nlm.nih.gov/clinvar/variation/204095/
NM_024649.4(BBS1):c.1240G>T	concordant	reportable	reportable	412287	1		0	SCV000553782	Pathogenic	Invitae	10/5/2017	BBS1	chr11	66294179	66294179		5.57E-06					0	0	https://www.ncbi.nlm.nih.gov/clinvar/variation/412287/
NM_033056.3(PCDH15):c.1631T>G	concordant	unreportable	unreportable	300194	0		1	SCV000363207	Uncertain significance	Illumina Clinical Se	10/18/2016	PCDH15	chr10	55913013	55913013	http://gnomad.b	1.29E-05	AMR	0.0001192249983	0		0	0	https://www.ncbi.nlm.nih.gov/clinvar/variation/300194/
NM_000053.3(ATP7B):c.2191G>A	concordant	unreportable	unreportable	289085	0		1	SCV000343370	Uncertain significance	EGL Genetic Diagn	6/30/2017	ATP7B	chr13	52532611	52532611	http://gnomad.b	1.12E-05	AFR	0.0001307360071	0		0	0	https://www.ncbi.nlm.nih.gov/clinvar/variation/289085/
NM_000051.3(ATM):c.6144A>G	concordant	unreportable	unreportable	453615	0		1	SCV000622643	Uncertain significance	Invitae	10/5/2017	ATM	chr11	108186786	108186786	http://gnomad.b	1.39E-05	NFE	8.96E-06	0		0	0	https://www.ncbi.nlm.nih.gov/clinvar/variation/453615/
NM_000051.3(ATM):c.2922-7dupA	concordant	unreportable	unreportable	453440	0		1	SCV000622377	Likely benign	Invitae	10/5/2017	ATM	chr11	108141970	108141970	http://gnomad.b	5.57E-06	SAS	3.26E-05	0		0	0	https://www.ncbi.nlm.nih.gov/clinvar/variation/453440/
NM_000051.3(ATM):c.2922-8T>A	concordant	unreportable	unreportable	434431	0		3	SCV000705844;S(	Uncertain significance;Likel	EGL Genetic Diagn	2017-06-30;2017-0	ATM	chr11	108141970	108141970		2.78E-06					0	0	https://www.ncbi.nlm.nih.gov/clinvar/variation/434431/
NM_000136.2(FANCC):c.802T>A	concordant	unreportable	unreportable	182479	0		2	SCV000211045;S(	Uncertain significance;Unc	GeneDx;Invitae	2017-11-28;2017-0	FANCC	chr9	97897669	97897669	http://gnomad.b	1.19E-05	NFE	8.95E-06	0		0	0	https://www.ncbi.nlm.nih.gov/clinvar/variation/182479/
NM_000051.3(ATM):c.1727T>C	concordant	unreportable	unreportable	181920	0		3	SCV000209691;S(	Uncertain significance;Unc	GeneDx;Invitae;Lal	2017-11-28;2017-1	ATM	chr11	108122683	108122683	http://gnomad.b	1.67E-05	FIN	0.0003591630084	0		0	0	https://www.ncbi.nlm.nih.gov/clinvar/variation/181920/
NM_002225.3(IVD):c.1075-10C>T	concordant	unreportable	unreportable	390425	0		1	SCV000533247	Likely benign	GeneDx	11/28/2017	IVD	chr15	40708479	40708479	http://gnomad.b	1.12E-05	AMR	0.0002084449952	0		0	0	https://www.ncbi.nlm.nih.gov/clinvar/variation/390425/
NM_138694.3(PKHD1):c.11476C>A	concordant	unreportable	unreportable	357406	0		1	SCV000464028	Uncertain significance	Illumina Clinical Se	10/18/2016	PKHD1	chr6	51503677	51503677	http://gnomad.b	5.28E-05	NFE	4.48E-05	0		0	0	https://www.ncbi.nlm.nih.gov/clinvar/variation/357406/
NM_000051.3(ATM):c.2127T>G	concordant	unreportable	unreportable	220354	0		1	SCV000260836	Uncertain significance	Invitae	10/5/2017	ATM	chr11	108126944	108126944		1.11E-05					0	0	https://www.ncbi.nlm.nih.gov/clinvar/variation/220354/
NM_000016.4(ACADM):c.131A>G	concordant	unreportable	unreportable	265023	0		1	SCV000321372	Uncertain significance	GeneDx	11/28/2017	ACADM	chr1	76198341	76198341	http://gnomad.b	1.09E-05	AFR	0.0004579350061	0		0	0	https://www.ncbi.nlm.nih.gov/clinvar/variation/265023/
NM_000051.3(ATM):c.2168T>C	concordant	unreportable	unreportable	233481	0		1	SCV000622312	Uncertain significance	Invitae	10/5/2017	ATM	chr11	108126985	108126985	http://gnomad.b	5.57E-06	AMR	0.0002682720078	0		0	0	https://www.ncbi.nlm.nih.gov/clinvar/variation/233481/
NM_000108.3(DLD):c.105C>G	concordant	reportable	reportable	421142	1		0	SCV000570256	Likely pathogenic	GeneDx	11/28/2017	DLD	chr7	107533710	107533710		7.79E-06					0	0	https://www.ncbi.nlm.nih.gov/clinvar/variation/421142/
NM_000023.2(SGCA):c.541C>A	concordant	unreportable	unreportable	288595	1		1	SCV000342760;S(	Uncertain significance;Likel	EGL Genetic Diagn	2017-06-30;2017-1	SGCA	chr17	48245890	48245890		2.52E-05					0	0	https://www.ncbi.nlm.nih.gov/clinvar/variation/288595/
NM_002485.4(NBN):c.540C>T	concordant	unreportable	unreportable	416885	0		1	SCV000562943	Likely benign	Invitae	3/14/2017	NBN	chr8	90990492	90990492		5.58E-06					0	0	https://www.ncbi.nlm.nih.gov/clinvar/variation/416885/
NM_000271.4(NPC1):c.302T>G	concordant	unreportable	unreportable	500461	0		1	SCV000706422	Uncertain significance	EGL Genetic Diagn	6/30/2017	NPC1	chr18	21148948	21148948	http://gnomad.b	4.86E-05	SAS	0.0001949319994	0		0	0	https://www.ncbi.nlm.nih.gov/clinvar/variation/500461/
NM_000642.2(AGL):c.310G>A	concordant	unreportable	unreportable	456484	0		1	SCV000626718	Uncertain significance	Invitae	10/5/2017	AGL	chr1	100327829	100327829	http://gnomad.b	2.23E-05	SAS	0.0001949190046	0		0	0	https://www.ncbi.nlm.nih.gov/clinvar/variation/456484/
NM_000271.4(NPC1):c.398T>G	concordant	unreportable	unreportable	501360	0		1	SCV000707689	Uncertain significance	EGL Genetic Diagn	6/30/2017	NPC1	chr18	21148852	21148852	http://gnomad.b	2.86E-05	AFR	0.0002614039986	0		0	0	https://www.ncbi.nlm.nih.gov/clinvar/variation/501360/
NM_000051.3(ATM):c.8246A>T	concordant	unreportable	unreportable	230416	0		2	SCV000367068;S(	Uncertain significance;Unc	Illumina Clinical Se	2016-10-18;2017-1	ATM	chr11	108206666	108206666	http://gnomad.b	3.90E-05	EAS	0.0004065510002	0		0	0	https://www.ncbi.nlm.nih.gov/clinvar/variation/230416/
NM_000136.2(FANCC):c.948G>C	concordant	unreportable	unreportable	449864	0		1	SCV000618309	Uncertain significance	GeneDx	11/28/2017	FANCC	chr9	97887416	97887416		7.13E-06					0	0	https://www.ncbi.nlm.nih.gov/clinvar/variation/449864/
NM_001079802.1(FKTN):c.411C>A	concordant	reportable	reportable	167069	1		0	SCV000613317	Likely pathogenic	Athena Diagnostics	8/17/2017	FKTN	chr9	108366537	108366537	http://gnomad.b	4.25E-05	SAS	0.0001624330034	0		0	0	https://www.ncbi.nlm.nih.gov/clinvar/variation/167069/
NM_000151.3(G6PC):c.993G>C	concordant	unreportable	unreportable	323364	0		1	SCV000402985	Uncertain significance	Illumina Clinical Se	10/18/2016	G6PC	chr17	41063362	41063362	http://gnomad.b	1.53E-05	EAS	5.80E-05	0		0	0	https://www.ncbi.nlm.nih.gov/clinvar/variation/323364/
NM_000051.3(ATM):c.754T>A	concordant	unreportable	unreportable	216231	0		1	SCV000254147	Uncertain significance	Invitae	9/11/2015	ATM	chr11	108115606	108115606		5.57E-06					0	0	https://www.ncbi.nlm.nih.gov/clinvar/variation/216231/
NM_000352.3(ABCC8):c.794G>A	concordant	unreportable	unreportable	453073	0		1	SCV000621929	Uncertain significance	GeneDx	11/28/2017	ABCC8	chr11	17483158	17483158	http://gnomad.b	1.29E-05	SAS	3.25E-05	0		0	0	https://www.ncbi.nlm.nih.gov/clinvar/variation/453073/
NM_018941.3(CLN8):c.77C>T	concordant	unreportable	unreportable	409294	0		1	SCV000549257	Uncertain significance	Invitae	3/14/2017	CLN8	chr8	1719297	1719297	http://gnomad.b	2.52E-05	AMR	0.0002977790136	0		0	0	https://www.ncbi.nlm.nih.gov/clinvar/variation/409294/
NM_000152.3(GAA):c.212A>G	concordant	unreportable	unreportable	500671	0		1	SCV000706711	Uncertain significance	EGL Genetic Diagn	6/30/2017	GAA	chr17	78078597	78078597	http://gnomad.b	1.95E-05	NFE	9.17E-06	0		0	0	https://www.ncbi.nlm.nih.gov/clinvar/variation/500671/
NM_000051.3(ATM):c.3538G>A	concordant	unreportable	unreportable	407457	0		1	SCV000546662	Uncertain significance	Invitae	3/14/2017	ATM	chr11	108151857	108151857		2.78E-06					0	0	https://www.ncbi.nlm.nih.gov/clinvar/variation/407457/
NM_000518.4(HBB):c.327C>T	concordant	unreportable	unreportable	495997	0		1	SCV000697121	Uncertain significance	Laboratory Corpor	1/25/2018	HBB	chr11	5246945	5246945	http://gnomad.b	2.80E-05	EAS	0.0001161170003	0		0	0	https://www.ncbi.nlm.nih.gov/clinvar/variation/495997/
NM_001042432.1(CLN3):c.1234A>G	concordant	unreportable	unreportable	421549	0		1	SCV000570793	Uncertain significance	GeneDx	11/28/2017	CLN3	chr16	28488920	28488920		2.22E-05					0	0	https://www.ncbi.nlm.nih.gov/clinvar/variation/421549/
NM_000414.3(HSD17B4):c.811G>A	concordant	unreportable	unreportable	350463	0		1	SCV000452115	Uncertain significance	Illumina Clinical Se	10/18/2016	HSD17B4	chr5	118829584	118829584	http://gnomad.b	8.45E-05	SAS	0.0007798279985	0		1	0	https://www.ncbi.nlm.nih.gov/clinvar/variation/350463/
NM_000642.2(AGL):c.2084A>G	concordant	unreportable	unreportable	291336	0		1	SCV000346075	Uncertain significance	Illumina Clinical Se	10/18/2016	AGL	chr1	100346930	100346930	http://gnomad.b	5.58E-06	EAS	5.80E-05	0		0	0	https://www.ncbi.nlm.nih.gov/clinvar/variation/291336/
NM_000051.3(ATM):c.2873A>G	concordant	unreportable	unreportable	133611	0		3	SCV000682085;S(	Uncertain significance;Unc	Color Genomics, In	2017-10-26;2017-1	ATM	chr11	108141825	108141825	http://gnomad.b	9.19E-05	SAS	0.0007147499709	0		0	0	https://www.ncbi.nlm.nih.gov/clinvar/variation/133611/
NM_000051.3(ATM):c.4658A>C	concordant	unreportable	unreportable	133620	0		2	SCV000292470;S(	Uncertain significance;Unc	GeneDx;Invitae	2017-11-28;2016-0	ATM	chr11	108164086	108164086	http://gnomad.b	8.35E-06	AFR	6.56E-05	0		0	0	https://www.ncbi.nlm.nih.gov/clinvar/variation/133620/
NM_000051.3(ATM):c.6178C>T	concordant	unreportable	unreportable	127422	0		3	SCV000687676;S(	Uncertain significance;Unc	Color Genomics, In	2017-12-21;2017-1	ATM	chr11	108186820	108186820	http://gnomad.b	3.34E-05	SAS	6.50E-05	0		0	0	https://www.ncbi.nlm.nih.gov/clinvar/variation/127422/
NM_000051.3(ATM):c.400G>A	concordant	unreportable	unreportable	133632	0		1	SCV000546747	Uncertain significance	Invitae	10/5/2017	ATM	chr11	108106465	108106465	http://gnomad.b	1.11E-05	AMR	0.0001192319978	0		0	0	https://www.ncbi.nlm.nih.gov/clinvar/variation/133632/
NM_000051.3(ATM):c.8495G>A	concordant	unreportable	unreportable	133638	0		3	SCV000292186;S(	Uncertain significance;Unc	Color Genomics, In	2017-12-21;2017-1	ATM	chr11	108216546	108216546	http://gnomad.b	0.0001002495099	SAS	0.0005199189764	0		0	0	https://www.ncbi.nlm.nih.gov/clinvar/variation/133638/
NM_000051.3(ATM):c.649A>G	concordant	unreportable	unreportable	133639	0		3	SCV000687707;S(	Uncertain significance;Unc	Color Genomics, In	2017-12-21;2017-1	ATM	chr11	108114832	108114832	http://gnomad.b	3.34E-05	SAS	6.50E-05	0		0	0	https://www.ncbi.nlm.nih.gov/clinvar/variation/133639/
NM_002485.4(NBN):c.872A>G	concordant	unreportable	unreportable	134877	0		1	SCV000690744	Uncertain significance	Color Genomics, In	12/21/2017	NBN	chr8	90982616	90982616		2.79E-06					0	0	https://www.ncbi.nlm.nih.gov/clinvar/variation/134877/
NM_003494.3(DYSF):c.1392dupA	concordant	reportable	reportable	94271	2		0	SCV000331281;S(	Pathogenic;Pathogenic	EGL Genetic Diagn	2016-11-03;2018-0	DYSF	chr2	71762435	71762435	http://gnomad.b	1.62E-05	NFE	8.95E-06	0		0	0	https://www.ncbi.nlm.nih.gov/clinvar/variation/94271/
NM_003494.3(DYSF):c.4200dupC	concordant	reportable	reportable	94320	2		0	SCV000331300;S(	Pathogenic;Pathogenic	EGL Genetic Diagn	2016-11-03;2017-1	DYSF	chr2	71839796	71839796	http://gnomad.b	8.08E-06	SAS	6.50E-05	0		0	0	https://www.ncbi.nlm.nih.gov/clinvar/variation/94320/
NM_003494.3(DYSF):c.5979dupA	concordant	reportable	reportable	94351	3		0	SCV000333039;S(	Pathogenic;Pathogenic;Pat	EGL Genetic Diagn	2016-11-03;2017-1	DYSF	chr2	71908162	71908162	http://gnomad.b	4.85E-05	AMR	0.0002084570006	0		0	0	https://www.ncbi.nlm.nih.gov/clinvar/variation/94351/
NM_000243.2(MEFV):c.1776C>T	concordant	unreportable	unreportable	97459	0		1	SCV000341487	Uncertain significance	EGL Genetic Diagn	6/30/2017	MEFV	chr16	3293876	3293876	http://gnomad.b	2.24E-05	SAS	0.0001300559961	0		0	0	https://www.ncbi.nlm.nih.gov/clinvar/variation/97459/
NM_000277.1(PAH):c.168+5G>C	concordant	reportable	reportable	102606	2		0	SCV000330988;S(	Pathogenic;Pathogenic	EGL Genetic Diagn	2017-06-30;2017-1	PAH	chr12	103306564	103306564	http://gnomad.b	8.22E-06	NFE	7.17E-05	0		0	0	https://www.ncbi.nlm.nih.gov/clinvar/variation/102606/
NM_000277.1(PAH):c.60+5G>T	concordant	reportable	reportable	102751	2		0	SCV000239043;S(	Pathogenic;Pathogenic	GeneDx;Invitae	2017-11-28;2017-1	PAH	chr12	103310844	103310844	http://gnomad.b	2.74E-05	AMR	0.0002084449952	0		0	0	https://www.ncbi.nlm.nih.gov/clinvar/variation/102751/
NM_002485.4(NBN):c.*401C>G	concordant	unreportable	unreportable	127007	0		1	SCV000475281	Uncertain significance	Illumina Clinical Se	10/18/2016	NBN	chr8	90947409	90947409		0.001639709983					0	0	https://www.ncbi.nlm.nih.gov/clinvar/variation/127007/
NM_002485.4(NBN):c.1202C>G	concordant	unreportable	unreportable	127010	0		3	SCV000685701;S(	Uncertain significance;Unc	Color Genomics, In	2017-10-26;2017-1	NBN	chr8	90967706	90967706	http://gnomad.b	8.37E-06	FIN	0.0006731289905	0		0	0	https://www.ncbi.nlm.nih.gov/clinvar/variation/127010/
NM_000051.3(ATM):c.1138T>A	concordant	unreportable	unreportable	127331	0		4	SCV000701958;S(	Uncertain significance;Unc	EGL Genetic Diagn	2017-06-30;2017-1	ATM	chr11	108119732	108119732	http://gnomad.b	3.34E-05	NFE	2.69E-05	0		0	0	https://www.ncbi.nlm.nih.gov/clinvar/variation/127331/
NM_000051.3(ATM):c.202A>G	concordant	unreportable	unreportable	127344	0		4	SCV000611348;S(	Uncertain significance;Unc	Fulgent Genetics,F	2017-05-23;2017-1	ATM	chr11	108099921	108099921	http://gnomad.b	5.85E-05	NFE	6.32E-05	0		0	0	https://www.ncbi.nlm.nih.gov/clinvar/variation/127344/
NM_000051.3(ATM):c.2638+2T>C	concordant	reportable	reportable	127357	3		0	SCV000682067;S(	Likely pathogenic;Pathogen	Color Genomics, In	2017-10-26;2017-1	ATM	chr11	108138071	108138071	http://gnomad.b	8.35E-06	NFE	8.96E-06	0		0	0	https://www.ncbi.nlm.nih.gov/clinvar/variation/127357/
NM_000051.3(ATM):c.382G>A	concordant	unreportable	unreportable	127375	0		1	SCV000546770	Uncertain significance	Invitae	3/14/2017	ATM	chr11	108106447	108106447		2.78E-06					0	0	https://www.ncbi.nlm.nih.gov/clinvar/variation/127375/
NM_000051.3(ATM):c.4201T>A	concordant	unreportable	unreportable	127383	0		2	SCV000149097;S(	Uncertain significance;Unc	GeneDx;Invitae	2017-11-28;2017-0	ATM	chr11	108159795	108159795	http://gnomad.b	2.78E-06	NFE	3.59E-05	0		0	0	https://www.ncbi.nlm.nih.gov/clinvar/variation/127383/
NM_000051.3(ATM):c.4420C>G	concordant	unreportable	unreportable	127391	0		2	SCV000149105;S(	Uncertain significance;Unc	GeneDx;Invitae	2017-11-28;2017-1	ATM	chr11	108160512	108160512	http://gnomad.b	2.23E-05	NFE	1.80E-05	0		0	0	https://www.ncbi.nlm.nih.gov/clinvar/variation/127391/
NM_000051.3(ATM):c.5250G>T	concordant	unreportable	unreportable	127404	0		1	SCV000682257	Uncertain significance	Color Genomics, In	10/26/2017	ATM	chr11	108172447	108172447		2.78E-06					0	0	https://www.ncbi.nlm.nih.gov/clinvar/variation/127404/
NM_000051.3(ATM):c.6160G>T	concordant	unreportable	unreportable	127420	0		2	SCV000149134;S(	Uncertain significance;Unc	GeneDx;Invitae	2017-11-28;2017-1	ATM	chr11	108186802	108186802	http://gnomad.b	5.57E-06	AFR	6.54E-05	0		0	0	https://www.ncbi.nlm.nih.gov/clinvar/variation/127420/
NM_000051.3(ATM):c.6232T>C	concordant	unreportable	unreportable	127423	0		2	SCV000149137;S(	Uncertain significance;Unc	GeneDx;Invitae	2017-11-28;2017-1	ATM	chr11	108188133	108188133	http://gnomad.b	2.78E-06	AFR	6.54E-05	0		0	0	https://www.ncbi.nlm.nih.gov/clinvar/variation/127423/
NM_000051.3(ATM):c.6239A>G	concordant	unreportable	unreportable	127424	0		1	SCV000682317	Uncertain significance	Color Genomics, In	10/26/2017	ATM	chr11	108188140	108188140		2.78E-06					0	0	https://www.ncbi.nlm.nih.gov/clinvar/variation/127424/
NM_000051.3(ATM):c.68G>A	concordant	unreportable	unreportable	127429	0		2	SCV000682372;S(	Uncertain significance;Unc	Color Genomics, In	2017-10-26;2017-1	ATM	chr11	108098419	108098419		2.78E-06					0	0	https://www.ncbi.nlm.nih.gov/clinvar/variation/127429/
NM_000051.3(ATM):c.6998C>T	concordant	unreportable	unreportable	127435	0		3	SCV000149149;S(	Uncertain significance;Unc	GeneDx;Invitae;Lal	2017-11-28;2017-1	ATM	chr11	108198394	108198394	http://gnomad.b	5.01E-05	AFR	0.0007845190121	0		0	0	https://www.ncbi.nlm.nih.gov/clinvar/variation/127435/
NM_000051.3(ATM):c.7078T>C	concordant	unreportable	unreportable	127436	0		2	SCV000687754;S(	Uncertain significance;Unc	Color Genomics, In	2017-12-21;2017-1	ATM	chr11	108198474	108198474		2.78E-06					0	0	https://www.ncbi.nlm.nih.gov/clinvar/variation/127436/
NM_000051.3(ATM):c.7998dupT	concordant	reportable	reportable	127453	1		0	SCV000682448	Pathogenic	Color Genomics, In	10/26/2017	ATM	chr11	108204682	108204682		5.57E-06					0	0	https://www.ncbi.nlm.nih.gov/clinvar/variation/127453/
NM_000051.3(ATM):c.8113G>T	concordant	unreportable	unreportable	127455	0		1	SCV000546757	Uncertain significance	Invitae	3/14/2017	ATM	chr11	108205798	108205798	http://gnomad.b	8.35E-06	NFE	8.96E-06	0		0	0	https://www.ncbi.nlm.nih.gov/clinvar/variation/127455/
NM_000051.3(ATM):c.8797A>G	concordant	unreportable	unreportable	127464	0		1	SCV000149179	Uncertain significance	GeneDx	11/28/2017	ATM	chr11	108225548	108225548	http://gnomad.b	2.23E-05	EAS	0.0002898889943	0		0	0	https://www.ncbi.nlm.nih.gov/clinvar/variation/127464/
NM_000051.3(ATM):c.8895G>C	concordant	unreportable	unreportable	127465	0		2	SCV000149180;S(	Uncertain significance;Unc	GeneDx;Invitae	2017-11-28;2017-1	ATM	chr11	108235853	108235853	http://gnomad.b	8.35E-06	NFE	8.96E-06	0		0	0	https://www.ncbi.nlm.nih.gov/clinvar/variation/127465/

Supplemental Table 2

NM_000051.3(ATM):c.9094G>C	concordant	unreportable	unreportable	127469	0	1	SCV000622885	Uncertain significance	Invitae	10/5/2017	ATM	chr11	108236158	108236158	http://gnomad.b	1.11E-05	FIN	8.97E-05	0		0	0	https://www.ncbi.nlm.nih.gov/clinvar/variation/127469/
NM_000136.2(FANCC):c.-79+5G>A	concordant	unreportable	unreportable	127528	0	1	SCV000149247	Likely benign	GeneDx	11/28/2017	FANCC	chr9	98079803	98079803		0.0004661095548					1	0	https://www.ncbi.nlm.nih.gov/clinvar/variation/127528/
NM_000136.2(FANCC):c.1009C>T	concordant	unreportable	unreportable	127530	0	2	SCV000149249;S	Uncertain significance;Unc	GeneDx;Invitae	2017-11-28;2015-0	FANCC	chr9	97879660	97879660	http://gnomad.b	4.76E-06	NFE	8.99E-06	0		0	0	https://www.ncbi.nlm.nih.gov/clinvar/variation/127530/
NM_000136.2(FANCC):c.1575T>G	concordant	unreportable	unreportable	127534	0	2	SCV000149253;S	Uncertain significance;Unc	GeneDx;Invitae	2017-11-28;2015-0	FANCC	chr9	97864091	97864091	http://gnomad.b	1.19E-05	AFR	6.54E-05	0		0	0	https://www.ncbi.nlm.nih.gov/clinvar/variation/127534/
NM_000136.2(FANCC):c.397C>T	concordant	unreportable	unreportable	127542	0	1	SCV000549949	Uncertain significance	Invitae	3/14/2017	FANCC	chr9	97934378	97934378		2.38E-06					0	0	https://www.ncbi.nlm.nih.gov/clinvar/variation/127542/
NM_000136.2(FANCC):c.843+1G>A	concordant	reportable	reportable	127547	1	0	SCV000260307	Likely pathogenic	Invitae	1/6/2016	FANCC	chr9	97897627	97897627	http://gnomad.b	9.51E-06	NFE	1.79E-05	0		0	0	https://www.ncbi.nlm.nih.gov/clinvar/variation/127547/
NM_002485.4(NBN):c.2068A>C	concordant	unreportable	unreportable	127864	0	1	SCV000261356	Uncertain significance	Invitae	6/10/2016	NBN	chr8	90958370	90958370		2.79E-06					0	0	https://www.ncbi.nlm.nih.gov/clinvar/variation/127864/
NM_002485.4(NBN):c.2149A>T	concordant	unreportable	unreportable	127865	0	3	SCV000611487;S	Uncertain significance;Unc	Fulgent Genetics,F	2017-05-23;2017-1	NBN	chr8	90955516	90955516		5.58E-06					0	0	https://www.ncbi.nlm.nih.gov/clinvar/variation/127865/
NM_002485.4(NBN):c.220T>C	concordant	unreportable	unreportable	127867	0	2	SCV000149699;S	Uncertain significance;Unc	GeneDx;Invitae	2017-11-28;2017-1	NBN	chr8	90993703	90993703	http://gnomad.b	1.12E-05	NFE	8.96E-06	0		0	0	https://www.ncbi.nlm.nih.gov/clinvar/variation/127867/
NM_002485.4(NBN):c.602A>T	concordant	unreportable	unreportable	127875	0	2	SCV000685802;S	Uncertain significance;Unc	Color Genomics, In	2017-10-26;2017-1	NBN	chr8	90983501	90983501	http://gnomad.b	5.58E-06	NFE	8.97E-06	0		0	0	https://www.ncbi.nlm.nih.gov/clinvar/variation/127875/
NM_002485.4(NBN):c.925G>A	concordant	unreportable	unreportable	127881	0	2	SCV000149716;S	Uncertain significance;Unc	GeneDx;Laboratory	2017-11-28;2018-0	NBN	chr8	90976707	90976707		8.37E-06					0	0	https://www.ncbi.nlm.nih.gov/clinvar/variation/127881/
NM_001875.4(CPS1):c.937A>G	concordant	unreportable	unreportable	128853	0	1	SCV000426819	Uncertain significance	Illumina Clinical Se	10/18/2016	CPS1	chr2	211455620	211455620	http://gnomad.b	4.00E-05	SAS	3.25E-05	0		0	0	https://www.ncbi.nlm.nih.gov/clinvar/variation/128853/
NM_001130987.1(DYSF):c.1380+6G>C	concordant	unreportable	unreportable	128945	0	4	SCV000512912;S	Benign;Likely benign;Uncer	GeneDx;Genetic Se	2017-11-28;2014-0	DYSF	chr2	71755537	71755537	http://gnomad.b	0.001972354701	AFR	0.02510239929	6		4	0	https://www.ncbi.nlm.nih.gov/clinvar/variation/128945/
NM_003494.3(DYSF):c.1422C>T	concordant	unreportable	unreportable	128946	0	3	SCV000527063;S	Benign;Likely benign;Benig	GeneDx;Genetic Se	2017-11-28;2014-0	DYSF	chr2	71766311	71766311	http://gnomad.b	0.002910031525	AFR	0.03417409956	6		3	0	https://www.ncbi.nlm.nih.gov/clinvar/variation/128946/
NM_003494.3(DYSF):c.3287G>A	concordant	unreportable	unreportable	128947	0	3	SCV000525220;S	Benign;Likely benign;Benig	GeneDx;Genetic Se	2017-11-28;2014-0	DYSF	chr2	71801440	71801440	http://gnomad.b	0.001657101285	AFR	0.02058820054	6		2	0	https://www.ncbi.nlm.nih.gov/clinvar/variation/128947/
NM_003494.3(DYSF):c.4323G>A	concordant	unreportable	unreportable	128948	0	3	SCV000525308;S	Benign;Likely benign;Benig	GeneDx;Genetic Se	2017-11-28;2014-0	DYSF	chr2	71839926	71839926	http://gnomad.b	0.002610944952	AFR	0.0343561992	6		5	0	https://www.ncbi.nlm.nih.gov/clinvar/variation/128948/
NM_001130987.1(DYSF):c.4430A>G	concordant	unreportable	unreportable	128949	0	3	SCV000523004;S	Benign;Likely benign;Benig	GeneDx;Genetic Se	2017-11-28;2014-0	DYSF	chr2	71840506	71840506	http://gnomad.b	0.001948104438	AFR	0.02504939958	5		1	0	https://www.ncbi.nlm.nih.gov/clinvar/variation/128949/
NM_024301.4(FKRP):c.192C>T	concordant	unreportable	unreportable	129056	0	2	SCV000677299;S	Benign;Benign	Athena Diagnostics	2017-07-17;2017-1	FKRP	chr19	47258899	47258899	http://gnomad.b	0.00440926987	AFR	0.04667060077	11		10	0	https://www.ncbi.nlm.nih.gov/clinvar/variation/129056/
NM_024301.4(FKRP):c.427C>A	concordant	unreportable	unreportable	129057	0	3	SCV000603720;S	Likely benign;Likely benign	ARUP Laboratories	2017-06-30;2017-1	FKRP	chr19	47259134	47259134	http://gnomad.b	0.004481290612	NFE	0.006825449876	5		0	0	https://www.ncbi.nlm.nih.gov/clinvar/variation/129057/
NM_024301.4(FKRP):c.822C>G	concordant	unreportable	unreportable	129058	0	4	SCV000337230;S	Benign;Likely benign;Likely	EGL Genetic Diagn	2017-06-30;2017-1	FKRP	chr19	47259529	47259529	http://gnomad.b	0.000976281169	AFR	0.009518859908	0		0	0	https://www.ncbi.nlm.nih.gov/clinvar/variation/129058/
NM_004543.4(NEB):c.14208C>T	concordant	unreportable	unreportable	129718	0	4	SCV000525742;S	Benign;Likely benign;Uncer	GeneDx;Genetic Se	2017-11-28;2014-0	NEB	chr2	152417109	152417109	http://gnomad.b	0.001110089943	AFR	0.03008539975	5		5	0	https://www.ncbi.nlm.nih.gov/clinvar/variation/129718/
NM_004543.4(NEB):c.14975C>T	concordant	unreportable	unreportable	129720	0	4	SCV000341975;S	Benign;Benign;Benign;Beni	EGL Genetic Diagn	2017-06-30;2017-1	NEB	chr2	152404901	152404901	http://gnomad.b	0.003260566507	NFE	0.01163769979	11		5	0	https://www.ncbi.nlm.nih.gov/clinvar/variation/129720/
NM_004543.4(NEB):c.16753G>A	concordant	unreportable	unreportable	129728	0	4	SCV000522736;S	Benign;Likely benign;Likely	GeneDx;Genetic Se	2017-11-28;2014-0	NEB	chr2	152383521	152383521	http://gnomad.b	0.005875731885	FIN	0.05085970089	38		20	0	https://www.ncbi.nlm.nih.gov/clinvar/variation/129728/
NM_004543.4(NEB):c.3147+5G>A	concordant	unreportable	unreportable	129739	0	3	SCV000614175;S	Benign;Benign;Benign	Athena Diagnostics	2017-08-17;2017-1	NEB	chr2	152536427	152536427	http://gnomad.b	0.007904873037	ASJ	0.04711620137	44		44	0	https://www.ncbi.nlm.nih.gov/clinvar/variation/129739/
NM_004543.4(NEB):c.3775-6T>C	concordant	unreportable	unreportable	129742	0	5	SCV000614178;S	Benign;Benign;Likely benig	Athena Diagnostics	2017-08-17;2017-1	NEB	chr2	152531911	152531911	http://gnomad.b	0.001463769762	AFR	0.03918249905	8		6	0	https://www.ncbi.nlm.nih.gov/clinvar/variation/129742/
NM_004543.4(NEB):c.4407G>C	concordant	unreportable	unreportable	129743	0	4	SCV000522709;S	Benign;Likely benign;Likely	GeneDx;Genetic Se	2017-11-28;2014-0	NEB	chr2	152527636	152527636	http://gnomad.b	0.009859147657	AFR	0.05589700118	78		62	0	https://www.ncbi.nlm.nih.gov/clinvar/variation/129743/
NM_004543.4(NEB):c.8318G>A	concordant	unreportable	unreportable	129758	0	6	SCV000614184;S	Benign;Benign;Likely benig	Athena Diagnostics	2017-08-17;2017-1	NEB	chr2	152499143	152499143	http://gnomad.b	0.0111396235	ASJ	0.04988820106	94		74	0	https://www.ncbi.nlm.nih.gov/clinvar/variation/129758/
NM_002485.4(NBN):c.1373A>G	concordant	unreportable	unreportable	132755	0	3	SCV000685712;S	Uncertain significance;Unc	Color Genomics, In	2017-10-26;2017-0	NBN	chr8	90967535	90967535	http://gnomad.b	1.67E-05	NFE	8.97E-06	0		0	0	https://www.ncbi.nlm.nih.gov/clinvar/variation/132755/
NM_002485.4(NBN):c.1398-10dupT	concordant	unreportable	unreportable	132766	0	4	SCV000685715;S	Likely benign;Likely benign	Color Genomics, In	2017-10-26;2017-0	NBN	chr8	90965928	90965928		0.0003262688232					0	0	https://www.ncbi.nlm.nih.gov/clinvar/variation/132766/
NM_000243.2(MEFV):c.818C>T	concordant	unreportable	unreportable	132874	0	1	SCV000618611	Uncertain significance	GeneDx	11/28/2017	MEFV	chr16	3304250	3304250		5.60E-06					0	0	https://www.ncbi.nlm.nih.gov/clinvar/variation/132874/
NM_000051.3(ATM):c.146C>T	concordant	unreportable	unreportable	135733	0	1	SCV000687307	Uncertain significance	Color Genomics, In	12/21/2017	ATM	chr11	108098576	108098576		2.78E-06					0	0	https://www.ncbi.nlm.nih.gov/clinvar/variation/135733/
NM_000051.3(ATM):c.1846A>G	concordant	unreportable	unreportable	135736	0	1	SCV000166082	Uncertain significance	Invitae	10/5/2017	ATM	chr11	108123587	108123587		1.11E-05					0	0	https://www.ncbi.nlm.nih.gov/clinvar/variation/135736/
NM_000051.3(ATM):c.192A>G	concordant	unreportable	unreportable	135738	0	2	SCV000524004;S	Likely benign;Likely benign	GeneDx;Invitae	2017-11-28;2016-0	ATM	chr11	108099911	108099911	http://gnomad.b	3.90E-05	EAS	0.0008763729711	0		0	0	https://www.ncbi.nlm.nih.gov/clinvar/variation/135738/
NM_000051.3(ATM):c.482A>C	concordant	unreportable	unreportable	135756	0	1	SCV000522015	Likely benign	GeneDx	11/28/2017	ATM	chr11	108106547	108106547		5.57E-06					0	0	https://www.ncbi.nlm.nih.gov/clinvar/variation/135756/
NM_000051.3(ATM):c.6179G>A	concordant	unreportable	unreportable	135765	0	3	SCV000687677;S	Uncertain significance;Unc	Color Genomics, In	2017-12-21;2017-1	ATM	chr11	108186821	108186821		1.39E-05					0	0	https://www.ncbi.nlm.nih.gov/clinvar/variation/135765/
NM_000051.3(ATM):c.6293T>C	concordant	unreportable	unreportable	135766	0	3	SCV000682321;S	Uncertain significance;Unc	Color Genomics, In	2017-10-26;2017-1	ATM	chr11	108188194	108188194	http://gnomad.b	2.23E-05	NFE	8.96E-06	0		0	0	https://www.ncbi.nlm.nih.gov/clinvar/variation/135766/
NM_000051.3(ATM):c.6317A>T	concordant	unreportable	unreportable	135768	0	2	SCV000682322;S	Uncertain significance;Unc	Color Genomics, In	2017-10-26;2017-1	ATM	chr11	108188218	108188218	http://gnomad.b	2.23E-05			0		0	0	https://www.ncbi.nlm.nih.gov/clinvar/variation/135768/
NM_000051.3(ATM):c.6343G>A	concordant	unreportable	unreportable	135770	0	3	SCV000292178;S	Uncertain significance;Unc	Color Genomics, In	2017-12-21;2017-1	ATM	chr11	108188244	108188244	http://gnomad.b	4.18E-05	AFR	0.0001983340044	0		0	0	https://www.ncbi.nlm.nih.gov/clinvar/variation/135770/
NM_000051.3(ATM):c.6420C>A	concordant	unreportable	unreportable	135771	0	3	SCV000687695;S	Uncertain significance;Unc	Color Genomics, In	2017-12-21;2017-1	ATM	chr11	108190753	108190753	http://gnomad.b	1.11E-05	AMR	8.94E-05	0		0	0	https://www.ncbi.nlm.nih.gov/clinvar/variation/135771/
NM_000051.3(ATM):c.6572+4T>C	concordant	unreportable	unreportable	135772	0	2	SCV000167099;S	Likely benign;Uncertain sig	GeneDx;Invitae	2017-11-28;2017-0	ATM	chr11	108192151	108192151	http://gnomad.b	2.23E-05	NFE	6.27E-05	0		0	0	https://www.ncbi.nlm.nih.gov/clinvar/variation/135772/
NM_000051.3(ATM):c.6765T>C	concordant	unreportable	unreportable	135773	0	2	SCV000687725;S	Likely benign;Likely benign	Color Genomics, In	2017-12-21;2017-1	ATM	chr11	108196229	108196229	http://gnomad.b	2.51E-05	EAS	5.80E-05	0		0	0	https://www.ncbi.nlm.nih.gov/clinvar/variation/135773/
NM_000051.3(ATM):c.7785T>C	concordant	unreportable	unreportable	135777	0	3	SCV000687789;S	Likely benign;Likely benign	Color Genomics, In	2017-12-21;2017-1	ATM	chr11	108202761	108202761	http://gnomad.b	1.67E-05	NFE	8.99E-06	0		0	0	https://www.ncbi.nlm.nih.gov/clinvar/variation/135777/
NM_000051.3(ATM):c.8036_8051del1	concordant	reportable	reportable	185795	1	0	SCV000166143	Pathogenic	Invitae	8/15/2016	ATM	chr11	108205719	108205735		2.78E-06					0	0	https://www.ncbi.nlm.nih.gov/clinvar/variation/185795/
NM_000051.3(ATM):c.8269-10_8269-	concordant	unreportable	unreportable	127458	0	2	SCV000149173;S	Uncertain significance;Likel	GeneDx;Invitae	2017-11-28;2017-1	ATM	chr11	108213937	108213939		5.57E-06					0	0	https://www.ncbi.nlm.nih.gov/clinvar/variation/127458/
NM_002485.4(NBN):c.104T>C	concordant	unreportable	unreportable	136034	0	3	SCV000690629;S	Uncertain significance;Unc	Color Genomics, In	2017-12-21;2017-1	NBN	chr8	90995017	90995017	http://gnomad.b	8.37E-06	EAS	0.001275509945	0		0	0	https://www.ncbi.nlm.nih.gov/clinvar/variation/136034/
NM_002485.4(NBN):c.1361C>A	concordant	unreportable	unreportable	136035	0	2	SCV000685710;S	Uncertain significance;Unc	Color Genomics, In	2017-10-26;2017-1	NBN	chr8	90967547	90967547		2.51E-05					0	0	https://www.ncbi.nlm.nih.gov/clinvar/variation/136035/
NM_002485.4(NBN):c.384A>G	concordant	unreportable	unreportable	136045	0	3	SCV000685782;S	Likely benign;Likely benign	Color Genomics, In	2017-10-26;2017-1	NBN	chr8	90993058	90993058	http://gnomad.b	5.58E-06	NFE	3.58E-05	0		0	0	https://www.ncbi.nlm.nih.gov/clinvar/variation/136045/
NM_000051.3(ATM):c.2466+7A>G	concordant	unreportable	unreportable	136437	0	2	SCV000682058;S	Likely benign;Benign	Color Genomics, In	2017-10-26;2017-1	ATM	chr11	108129809	108129809	http://gnomad.b	7.80E-05	EAS	0.004713689908	0		0	0	https://www.ncbi.nlm.nih.gov/clinvar/variation/136437/
NM_000051.3(ATM):c.3577-13T>C	concordant	unreportable	unreportable	136443	0	2	SCV000682143;S	Likely benign;Uncertain sig	Color Genomics, In	2017-10-26;2016-1	ATM	chr11	108153424	108153424	http://gnomad.b	3.34E-05	NFE	5.40E-05	0		0	0	https://www.ncbi.nlm.nih.gov/clinvar/variation/136443/
NM_001042432.1(CLN3):c.1197+8T>C	concordant	unreportable	unreportable	136784	0	1	SCV000560161	Likely benign	Invitae	3/14/2017	CLN3	chr16	28489050	28489050		2.78E-06					0	0	https://www.ncbi.nlm.nih.gov/clinvar/variation/136784/
NM_024301.4(FKRP):c.1177G>A	concordant	unreportable	unreportable	137380	0	4	SCV000613306;S	Likely benign;Likely benign	Athena Diagnostics	2017-08-17;2017-0	FKRP	chr19	47259884	47259884	http://gnomad.b	0.0004801382798	AFR	0.007128450088	0		1	0	https://www.ncbi.nlm.nih.gov/clinvar/variation/137380/
NM_000411.6(HLCS):c.-393+11G>T	concordant	unreportable	unreportable	137553	0	1	SCV000436072	Likely benign	Illumina Clinical Se	10/18/2016	HLCS	chr21	38362447	38362447		0.02942879197					66	0	https://www.ncbi.nlm.nih.gov/clinvar/variation/137553/
NM_133259.3(LRPPRC):c.2325A>G	concordant	unreportable	unreportable	138145	0	1	SCV000430609	Likely benign	Illumina Clinical Se	10/18/2016	LRPPRC	chr2	44171005	44171005	http://gnomad.b	0.002926595234	AMR	0.05372269824	56		4	0	https://www.ncbi.nlm.nih.gov/clinvar/variation/138145/
NM_133259.3(LRPPRC):c.-11A>G	concordant	unreportable	unreportable	138147	0	1	SCV000430645	Uncertain significance	Illumina Clinical Se	10/18/2016	LRPPRC	chr2	44223097	44223097	http://gnomad.b	0.003262434032	EAS	0.02835240029	3		5	0	https://www.ncbi.nlm.nih.gov/clinvar/variation/138147/
NM_133259.3(LRPPRC):c.58C>T	concordant	unreportable	unreportable	138149	0	1	SCV000430642	Uncertain significance	Illumina Clinical Se	10/18/2016	LRPPRC	chr2	44223029	44223029	http://gnomad.b	0.001655205501	AFR	0.02058229968	0		0	0	https://www.ncbi.nlm.nih.gov/clinvar/variation/138149/
NM_133259.3(LRPPRC):c.64C>G	concordant	unreportable	unreportable	138150	0	3	SCV000604135;S	Uncertain significance;Unc	ARUP Laboratories	2017-06-30;2017-0	LRPPRC	chr2	44223023	44223023	http://gnomad.b	0.00143131297	NFE	0.002553079976	1		0	0	https://www.ncbi.nlm.nih.gov/clinvar/variation/138150/
NM_002485.4(NBN):c.38-10T>A	concordant	unreportable	unreportable	138425	0	3	SCV000685779;S	Likely benign;Likely benign	Color Genomics, In	2017-10-26;2017-1	NBN	chr8	90995093	90995093	http://gnomad.b	5.58E-05	NFE	0.0002164930047	0		0	0	https://www.ncbi.nlm.nih.gov/clinvar/variation/138425/
NM_002485.4(NBN):c.1914+10G>A	concordant	unreportable	unreportable	138428	0	2	SCV000685742;S	Likely benign;Benign	Color Genomics, In	2017-10-26;2017-1	NBN	chr8	90960042	90960042	http://gnomad.b	9.48E-05	SAS	0.001259539975	1		0	0	https://www.ncbi.nlm.nih.gov/clinvar/variation/138428/
NM_002485.4(NBN):c.481-18A>G	concordant	unreportable	unreportable	138429	0	1	SCV000685790	Likely benign	Color Genomics, In	10/26/2017	NBN	chr8	90990569	90990569	http://gnomad.b	3.90E-05	AMR	0.001193030039	2		0	0	https://www.ncbi.nlm.nih.gov/clinvar/variation/138429/
NM_000337.5(SGCD):c.144G>C	concordant	unreportable	unreportable	139099	0	2	SCV000338989;S	Likely benign;Benign	EGL Genetic Diagn	2016-11-03;2017-1	SGCD	chr5	155771639	155771639	http://gnomad.b	0.0003201126797	AFR	0.002906590002	0		1	0	https://www.ncbi.nlm.nih.gov/clinvar/variation/139099/
NM_000337.5(SGCD):c.3+20G>A	concordant	unreportable	unreportable	139100	0	1	SCV000171621	Benign	GeneDx	11/28/2017	SGCD	chr5	155756609	155756609	http://gnomad.b	0.0005441915554	EAS	0.005187079776	2		0	0	https://www.ncbi.nlm.nih.gov/clinvar/variation/139100/
NM_000071.2(CBS):c.1114G>T	concordant	unreportable	unreportable	212864	0	1	SCV000249710	Uncertain significance	GeneDx	11/28/2017	CBS	chr21	44480582	44480582	http://gnomad.b	2.81E-06	AMR	2.98E-05	0		0	0	https://www.ncbi.nlm.nih.gov/clinvar/variation/212864/
NM_000154.1(GALK1):c.1096C>T	concordant	unreportable	unreportable	325224	0	1	SCV000406615	Uncertain significance	Illumina Clinical Se	10/18/2016	GALK1	chr17	73754302	73754302	http://gnomad.b	8.01E-05	AFR	0.0001522069942	0		0	0	https://www.ncbi.nlm.nih.gov/clinvar/variation/325224/
NM_000051.3(ATM):c.6196C>G	concordant	unreportable	unreportable	453616	0	1	SCV000622645	Uncertain significance	Invitae	10/5/2017	ATM	chr11	108186838	108186838		2.78E-06					0	0	https://www.ncbi.nlm.nih.gov/clinvar/variation/453616/
NM_000053.3(ATP7B):c.3498T>C	concordant	unreportable	unreportable	157951	0	1	SCV000384652	Uncertain significance	Illumina Clinical Se	10/18/2016	ATP7B	chr13	52515275	52515275	http://gnomad.b	3.09E-05	NFE	1.79E-05	0		0	0	https://www.ncbi.nlm.nih.gov/clinvar/variation/157951/
NM_000051.3(ATM):c.8327T>C	concordant	unreportable	unreportable	229723	0	2	SCV000564668;S	Uncertain significance;Unc	GeneDx;Invitae	2017-11-28;2017-1	ATM	chr11	108214007	108214007	http://gnomad.b	2.51E-05	NFE	4.48E-05	0		0	0	https://www.ncbi.nlm.nih.gov/clinvar/variation/229723/
NM_000051.3(ATM):c.8562C>T	concordant	unreportable	unreportable	236790	0	2	SCV000527586;S	Likely benign;Likely benign	GeneDx;Invitae	2017-11-28;2016-0	ATM	chr11	108216613	108216613	http://gnomad.b	2.78E-06	NFE	8.98E-06	0		0	0	https://www.ncbi.nlm.nih.gov/clinvar/variation/236790/
NM_000466.2(PEX1):c.830A>G	concordant	unreportable	unreportable	360930	0	1	SCV000470522	Uncertain significance	Illumina Clinical Se	10/18/2016	PEX1	chr7	92146999	92146999		2.77E-06					0	0	https://www.ncbi.nlm.nih.gov/clinvar/variation/360930/
NM_000051.3(ATM):c.1379C>T	concordant	unreportable	unreportable	141561	0	2	SCV000565853;S	Uncertain significance;Unc	GeneDx;Invitae	2017-11-28;2017-1	ATM	chr11	108121571	108121571		8.35E-06					0	0	https://www.ncbi.nlm.nih.gov/clinvar/variation/141561/
NM_033056.3(PCDH15):c.2897G>C	concordant	unreportable	unreportable	450627	0	1	SCV000619233	Uncertain significance	GeneDx	11/28/2017	PCDH15	chr10	55721624	55721624	http://gnomad.b	0.0001131512627	SAS	0.001495740027	0		0	0	https://www.ncbi.nlm.nih.gov/clinvar/variation/450627/
NM_000411.6(HLCS):c.59A>G	concordant	unreportable	unreportable	339970	0	1	SCV000436060	Uncertain significance	Illumina Clinical Se	10/18/2016	HLCS	chr21	38309686	38309686	http://gnomad.b	1.61E-05	NFE	2.85E-05	0		0	0	https://www.ncbi.nlm.nih.gov/clinvar/variation/339970/
NM_001128227.2(GNE):c.2122C>T	concordant	unreportable	unreportable	286153	0	2	SCV000339476;S	Uncertain significance;Unc	EGL Genetic Diagn	2017-06-30;2017-1	GNE	chr9	36217502	36217502	http://gnomad.b	8.40E-06	SAS	3.25E-05	0		0	0	https://www.ncbi.nlm.nih.gov/clinvar/variation/286153/
NM_000152.3(GAA):c.2842C>T	concordant	unreportable	unreportable	325798	0	1	SCV000407309	Uncertain significance	Illumina Clinical Se	10/18/2016	GAA	chr17	78093113	78093113	http://gnomad.b	1.11E-05	NFE	1.79E-05	0		0	0	https://www.ncbi.nlm.nih.gov/clinvar/variation/325798/
NM_000271.4(NPC1):c.2692G>A	concordant	unreportable	unreportable	440008	0	1	SCV000604563	Uncertain significance	ARUP Laboratories	6/30/2017	NPC1	chr18	21119878	21119878	http://gnomad.b	3.72E-05	AFR	6.53E-05	0		0	0	https://www.ncbi.nlm.nih.gov/clinvar/variation/440008/
NM_000057.2(BLM):c.1433G>C	concordant	unreportable	unreportable	454076	0	1	SCV000623237	Uncertain significance	Invitae	10/5/2017	BLM	chr15	91304036	91304036	http://gnomad.b	4.52E-05	SAS	3.25E-05	0		0	0	https://www.ncbi.nlm.nih.gov/clinvar/variation/454076/
NM_033056.3(PCDH15):c.665A>G	concordant	unreportable	unreportable	300201	0	1	SCV000363235	Uncertain significance	Illumina Clinical Se	10/18/2016	PCDH15	chr10	56089396	56089396	http://gnomad.b	2.83E-05	SAS	0.0004875829909	0		0	0	https://www.ncbi.nlm.nih.gov/clinvar/variation/300201/
NM_000136.2(FANCC):c.1634A>G	concordant	unreportable	unreportable	234672	0	1	SCV000626240	Uncertain significance	Invitae	10/5/2017	FANCC	chr9	97864032	97864032	http://gnomad.b	2.85E-05	SAS	0.0003574209986	0		0	0	https://www.ncbi.nlm.nih.gov/clinvar/variation/234672/
NM_033056.3(PCDH15):c.851C>T	concordant	unreportable	unreportable	300199	0	1	SCV000363229	Uncertain significance	Illumina Clinical Se	10/18/2016	PCDH15	chr10	56077056	56077056	http://gnomad.b	1.80E-05	SAS	0.0003248649882	0		0	0	https://www.ncbi.nlm.nih.gov/clinvar/variation/300199/
NM_000023.2(SGCA):c.737A>G	concordant	unreportable	unreportable	284388	0	1	SCV000336989	Uncertain significance	EGL Genetic Diagn	6/30/2017	SGCA	chr17	48246605	48246605	http://gnomad.b	8.40E-06	SAS	3.25E-05	0		0	0	https://www.ncbi.nlm.nih.gov/clinvar/variation/284388/
NM_017890.4(VPS13B):c.162A>G	concordant	unreportable	unreportable	437233	0	1	SCV000597870	Likely benign	Genetic Services La	7/5/2017	VPS13B	chr8	100050665	100050665	http://gnomad.b	1.12E-05	SAS	0.0001633450011	0		0	0	https://www.ncbi.nlm.nih.gov/clinvar/variation/437233/
NM_002485.4(NBN):c.266G>C	concordant	unreportable	unreportable	184749	0	3	SCV000685767;S	Uncertain significance;Unc	Color Genomics, In	2017-10-26;2017-1	NBN	chr8	90993657	90993657	http://gnomad.b	1.95E-05	AMR	0.0002977790136	0		0	0	https://www.ncbi.nlm.nih.gov/clinvar/variation/184749/
NM_003060.3(SLC22A5):c.1120G>A	concordant	unreportable	unreportable	350816	0	1	SCV000452737	Uncertain significance	Illumina Clinical Se	10/18/2016	SLC22A5	chr5	131726449	131726449		8.47E-06					0	0	https://www.ncbi.nlm.nih.gov/clinvar/variation/350816/
NM_000492.3(CFTR):c.2490+5G>T	concordant	unreportable	unreportable	288151	0	1	SCV000342174	Uncertain significance	EGL Genetic Diagn	6/30/2017	CFTR	chr7	117232716	117232716	http://gnomad.b	1.93E-05	NFE	2.21E-05	0		0	0	https://www.ncbi.nlm.nih.gov/clinvar/variation/288151/
NM_000051.3(ATM):c.8082A>C	concordant	unreportable	unreportable	377521	0	2	SCV000512179;S	Likely benign;Likely benign	GeneDx;Invitae	2017-11-28;2017-1	ATM	chr11	108205767	108205767	http://gnomad.b	5.29E-05	SAS	0.0009096229915	0		0	0	https://www.ncbi.nlm.nih.gov/clinvar/variation/377521/
NM_000057.2(BLM):c.700G>A	concordant	unreportable	unreportable	236824	0	1	SCV000283154	Uncertain significance	Invitae	10/5/2017	BLM	chr15	91293198	91293198		1.66E-05					0	0	https://www.ncbi.nlm.nih.gov/clinvar/variation/236824/
NM_000051.3(ATM):c.3085A>T	concordant	unreportable	unreportable	181938	0	2	SCV000209716;S	Uncertain significance;Unc	GeneDx;Invitae	2017-11-28;2016-0	ATM	chr11	108143266	108143266		5.57E-06					0	0	https://www.ncbi.nlm.nih.gov/clinvar/variation/181938/
NM_000051.3(ATM):c.8288G>A	concordant	unreportable	unreportable	453727	0	1	SCV000622815	Uncertain significance	Invitae	10/5/2017	ATM	chr11	108213968	108213968	http://gnomad.b	8.35E-06	AFR	6.54E-05	0		0	0	https://www.ncbi.nlm.nih.gov/clinvar/variation/453727/
NM_000382.2(ALDH3A2):c.86C>T	concordant	unreportable	unreportable	322207	0	1	SCV000401249	Uncertain significance	Illumina Clinical Se	10/18/2016	ALDH3A2	chr17	19552370	19552370		1.96E-05					0	0	https://www.ncbi.nlm.nih.gov/clinvar/variation/322207/
NM_000098.2(CPT2):c.1559C>T	concordant	unreportable	unreportable	287806	0	1	SCV000341714	Uncertain significance	EGL Genetic Diagn	6/30/2017	CPT2	chr1	53676905	53676905	http://gnomad.b	8.39E-05	EAS	0.0004086399858	0		0	0	https://www.ncbi.nlm.nih.gov/clinvar/variation/287806/
NM_006493.2(CLN5):c.1051A>G	concordant	unreportable	unreportable	409275	0	2	SCV000570757;S	Uncertain significance;Unc	GeneDx;Invitae	2017-11-28;2017-0	CLN5	chr13	77574931	77574931		5.59E-06					0	0	https://www.ncbi.nlm.nih.gov/clinvar/variation/409275/
NM_000051.3(ATM):c.1902A>G	concordant	unreportable	unreportable	407610	0	2	SCV000687343;S	Likely benign;Uncertain sig	Color Genomics, In	2017-12-21;2017-0	ATM	chr11	108124544	108124544		1.67E-05					0	0	https://www.ncbi.nlm.nih.gov/clinvar/variation/407610/
NM_000051.3(ATM):c.1037T>A	concordant	unreportable	unreportable	407452	0	1	SCV000546655	Uncertain significance	Invitae	10/5/2017	ATM	chr11	108117826	108117826		2.78E-06					0	0	https://www.ncbi.nlm.nih.gov/clinvar/variation/407452/
NM_000051.3(ATM):c.876G>A	concordant	unreportable	unreportable	230005	0	2	SCV000682513;S	Likely benign;Likely benign	Color Genomics, In	2017-10-26;2017-1	ATM	chr11	108115728	108115728	http://gnomad.b	8.35E-05	ASJ	0.000203500007	0		0	0	https://www.ncbi.nlm.nih.gov/clinvar/variation/230005/
NM_002485.4(NBN):c.1162A>C	concordant	unreportable	unreportable	492083	0	1	SCV000690644	Uncertain significance	Color Genomics, In	12/21/2017	NBN	chr8	90967746	90967746	http://gnomad.b	5.58E-06	SAS	3.25E-05	0		0	0	https://www.ncbi.nlm.nih.gov/clinvar/variation/492083/
NM_000136.2(FANCC):c.472G>C	concordant	unreportable	unreportable	234311	0	2	SCV000278972;S	Uncertain significance;Unc	GeneDx;Invitae	2017-11-28;2017-1	FANCC	chr9	97933410	97933410	http://gnomad.b	0.0001022587289	EAS	5.81E-05	0		0	0	https://www.ncbi.nlm.nih.gov/clinvar/variation/234311/
NM_000152.3(GAA):c.614C>T	concordant	unreportable	unreportable	287137	0	1	SCV000340821	Uncertain significance	EGL Genetic Diagn	6/30/2017	GAA	chr17	78079615	78079615	http://gnomad.b	1.67E-05	NFE	8.08E-05	0		0	0	https://www.ncbi.nlm.nih.gov/clinvar/variation/287137/
NM_017739.3(POMGNT1):c.70A>G	concordant	unreportable	unreportable	471408	0	1	SCV000649974	Uncertain significance	Invitae	10/5/2017	POMGNT1	chr1	46663424	46663424		1.12E-05					0	0	https://www.ncbi.nlm.nih.gov/clinvar/variation/471408/

Supplemental Table 2

NM_000642.2(AGL):c.1065G>A	concordant	unreportable	unreportable	291330	0		1	SCV000346064	Uncertain significance	Illumina Clinical Se	10/18/2016	AGL	chr1	100340349	100340349	http://gnomad.b	5.30E-05	AMR	0.0005958059919	0		0	0	https://www.ncbi.nlm.nih.gov/clinvar/variation/291330/
NM_000228.2(LAMB3):c.2496G>A	concordant	unreportable	unreportable	295088	0		1	SCV000353611	Uncertain significance	Illumina Clinical Se	10/18/2016	LAMB3	chr1	209796387	209796387	http://gnomad.b	2.80E-05	AMR	2.98E-05	0		0	0	https://www.ncbi.nlm.nih.gov/clinvar/variation/295088/
NM_017890.4(VPS13B):c.1108G>C	concordant	unreportable	unreportable	361040	0		1	SCV000470764	Uncertain significance	Illumina Clinical Se	10/18/2016	VPS13B	chr8	100133575	100133575	http://gnomad.b	2.52E-05	SAS	0.0002274059952	0		0	0	https://www.ncbi.nlm.nih.gov/clinvar/variation/361040/
NM_000051.3(ATM):c.3478G>C	concordant	unreportable	unreportable	230990	0		3	SCV000682133;S(	Uncertain significance;Unc	Color Genomics, In	2017-10-26;2017-1	ATM	chr11	108151797	108151797	http://gnomad.b	2.23E-05	NFE	1.79E-05	0		0	0	https://www.ncbi.nlm.nih.gov/clinvar/variation/230990/
NM_000051.3(ATM):c.5157T>C	concordant	unreportable	unreportable	186831	0		2	SCV000682253;S(	Likely benign;Likely benign	Color Genomics, In	2017-10-26;2016-0	ATM	chr11	108170592	108170592		5.57E-06					0	0	https://www.ncbi.nlm.nih.gov/clinvar/variation/186831/
NM_000018.3(ACADVL):c.54G>A	concordant	unreportable	unreportable	474899	0		1	SCV000654956	Likely benign	Invitae	10/5/2017	ACADVL	chr17	7123357	7123357		5.66E-06					0	0	https://www.ncbi.nlm.nih.gov/clinvar/variation/474899/
NM_000271.4(NPC1):c.546C>T	concordant	unreportable	unreportable	501573	0		1	SCV000707987	Uncertain significance	EGL Genetic Diagn	6/30/2017	NPC1	chr18	21141409	21141409	http://gnomad.b	3.72E-05	AFR	0.0001306850027	0		0	0	https://www.ncbi.nlm.nih.gov/clinvar/variation/501573/
NM_000642.2(AGL):c.535_538delTTA(	concordant	reportable	reportable	197686	1		0	SCV000230948	Pathogenic	EGL Genetic Diagn	6/30/2017	AGL	chr1	100330013	100330017	http://gnomad.b	1.67E-05	NFE	1.79E-05	0		0	0	https://www.ncbi.nlm.nih.gov/clinvar/variation/197686/
NM_002485.4(NBN):c.1761C>T	concordant	unreportable	unreportable	239188	0		3	SCV000685737;S(	Likely benign;Likely benign	Color Genomics, In	2017-10-26;2017-1	NBN	chr8	90965556	90965556		1.67E-05					0	0	https://www.ncbi.nlm.nih.gov/clinvar/variation/239188/
NM_000051.3(ATM):c.2636T>C	concordant	unreportable	unreportable	246364	0		2	SCV000293885;S(	Uncertain significance;Unc	GeneDx;Invitae	2017-11-28;2017-1	ATM	chr11	108138067	108138067	http://gnomad.b	1.39E-05	SAS	9.75E-05	0		0	0	https://www.ncbi.nlm.nih.gov/clinvar/variation/246364/
NM_004937.2(CTNS):c.771_793del23	concordant	reportable	reportable	496276	1		0	SCV000698526	Pathogenic	Laboratory Corpor	1/25/2018	CTNS	chr17	3561375	3561398	http://gnomad.b	2.96E-06	SAS	0.0001624330034	0		0	0	https://www.ncbi.nlm.nih.gov/clinvar/variation/496276/
NM_000049.2(ASPA):c.807G>A	concordant	unreportable	unreportable	322667	0		1	SCV000402020	Uncertain significance	Illumina Clinical Se	10/18/2016	ASPA	chr17	3402247	3402247	http://gnomad.b	1.89E-05	NFE	1.79E-05	0		0	0	https://www.ncbi.nlm.nih.gov/clinvar/variation/322667/
NM_000492.3(CFTR):c.1582G>A	concordant	unreportable	unreportable	502030	0		1	SCV000708622	Uncertain significance	EGL Genetic Diagn	6/30/2017	CFTR	chr7	117199707	117199707	http://gnomad.b	3.08E-05	SAS	6.50E-05	0		0	0	https://www.ncbi.nlm.nih.gov/clinvar/variation/502030/
NM_000051.3(ATM):c.7357C>T	concordant	unreportable	unreportable	230366	0		3	SCV000682408;S(	Uncertain significance;Unc	Color Genomics, In	2017-10-26;2017-1	ATM	chr11	108200990	108200990	http://gnomad.b	5.57E-06	EAS	5.81E-05	0		0	0	https://www.ncbi.nlm.nih.gov/clinvar/variation/230366/
NM_000051.3(ATM):c.931A>G	concordant	unreportable	unreportable	229990	0		2	SCV000547030;S(	Uncertain significance;Unc	Invitae;Laboratory	2017-10-05;2018-0	ATM	chr11	108117720	108117720	http://gnomad.b	1.11E-05	AMR	5.96E-05	0		0	0	https://www.ncbi.nlm.nih.gov/clinvar/variation/229990/
NM_000051.3(ATM):c.8942A>G	concordant	unreportable	unreportable	187627	0		3	SCV000682528;S(	Uncertain significance;Unc	Color Genomics, In	2017-10-26;2017-1	ATM	chr11	108235900	108235900	http://gnomad.b	1.67E-05	NFE	8.96E-06	0		0	0	https://www.ncbi.nlm.nih.gov/clinvar/variation/187627/
NM_000051.3(ATM):c.5456C>T	concordant	unreportable	unreportable	231669	0		1	SCV000622591	Uncertain significance	Invitae	10/5/2017	ATM	chr11	108173716	108173716	http://gnomad.b	2.78E-06	NFE	8.98E-06	0		0	0	https://www.ncbi.nlm.nih.gov/clinvar/variation/231669/
NM_001079802.1(FKTN):c.443A>G	concordant	unreportable	unreportable	286032	0		1	SCV000339287	Uncertain significance	EGL Genetic Diagn	6/30/2017	FKTN	chr9	108366569	108366569	http://gnomad.b	4.25E-05	AMR	0.0006257449859	0		0	0	https://www.ncbi.nlm.nih.gov/clinvar/variation/286032/
NM_000642.2(AGL):c.3345A>T	concordant	unreportable	unreportable	291341	0		2	SCV000346083;S(	Uncertain significance;Likel	Illumina Clinical Se	2016-10-18;2017-1	AGL	chr1	100361927	100361927	http://gnomad.b	2.51E-05	NFE	8.06E-05	0		0	0	https://www.ncbi.nlm.nih.gov/clinvar/variation/291341/
NM_000136.2(FANCC):c.745A>G	concordant	unreportable	unreportable	420182	0		1	SCV000568875	Uncertain significance	GeneDx	11/28/2017	FANCC	chr9	97897726	97897726	http://gnomad.b	1.43E-05	AFR	0.0002615750127	0		0	0	https://www.ncbi.nlm.nih.gov/clinvar/variation/420182/
NM_002485.4(NBN):c.1763A>G	concordant	unreportable	unreportable	230644	0		1	SCV000634250	Uncertain significance	Invitae	10/5/2017	NBN	chr8	90965554	90965554		1.12E-05					0	0	https://www.ncbi.nlm.nih.gov/clinvar/variation/230644/
NM_001042432.1(CLN3):c.537C>G	concordant	unreportable	unreportable	288316	0		1	SCV000342393	Uncertain significance	EGL Genetic Diagn	6/30/2017	CLN3	chr16	28497808	28497808		1.11E-05					0	0	https://www.ncbi.nlm.nih.gov/clinvar/variation/288316/
NM_001079802.1(FKTN):c.397A>G	concordant	unreportable	unreportable	364488	0		1	SCV000476422	Uncertain significance	Illumina Clinical Se	10/18/2016	FKTN	chr9	108366523	108366523	http://gnomad.b	5.67E-05	EAS	0.001217529993	0		0	0	https://www.ncbi.nlm.nih.gov/clinvar/variation/364488/
NM_000228.2(LAMB3):c.858C>T	concordant	unreportable	unreportable	295127	0		1	SCV000353654	Uncertain significance	Illumina Clinical Se	10/18/2016	LAMB3	chr1	209804045	209804045	http://gnomad.b	7.28E-05	AMR	0.0002084449952	0		0	0	https://www.ncbi.nlm.nih.gov/clinvar/variation/295127/
NM_001042432.1(CLN3):c.941G>A	concordant	unreportable	unreportable	318719	0		1	SCV000396324	Uncertain significance	Illumina Clinical Se	10/18/2016	CLN3	chr16	28493669	28493669	http://gnomad.b	1.94E-05	NFE	1.79E-05	0		0	0	https://www.ncbi.nlm.nih.gov/clinvar/variation/318719/
NM_000152.3(GAA):c.2132_2133delC	concordant	unreportable	unreportable	456391	0		1	SCV000626545	Uncertain significance	Invitae	10/5/2017	GAA	chr17	78087107	78087109		0.0001083929495					1	0	https://www.ncbi.nlm.nih.gov/clinvar/variation/456391/
NM_000051.3(ATM):c.5362G>A	concordant	unreportable	unreportable	181965	0		2	SCV000209746;S(	Uncertain significance;Unc	GeneDx;Invitae	2017-11-28;2017-1	ATM	chr11	108173622	108173622	http://gnomad.b	2.78E-05	EAS	0.000173952998	0		0	0	https://www.ncbi.nlm.nih.gov/clinvar/variation/181965/
NM_000441.1(SLC26A4):c.2007C>T	concordant	unreportable	unreportable	227952	0		1	SCV000270852	Likely benign	Laboratory for Mol	4/26/2016	SLC26A4	chr7	107342475	107342475	http://gnomad.b	1.40E-05	SAS	0.0001624849974	0		0	0	https://www.ncbi.nlm.nih.gov/clinvar/variation/227952/
NM_000051.3(ATM):c.6841T>C	concordant	unreportable	unreportable	490677	0		1	SCV000687733	Uncertain significance	Color Genomics, In	12/21/2017	ATM	chr11	108196818	108196818		2.78E-06					0	0	https://www.ncbi.nlm.nih.gov/clinvar/variation/490677/
NM_000051.3(ATM):c.4492T>C	concordant	unreportable	unreportable	184493	0		2	SCV000682211;S(	Likely benign;Likely benign	Color Genomics, In	2017-10-26;2017-0	ATM	chr11	108163401	108163401	http://gnomad.b	2.78E-05	NFE	1.79E-05	0		0	0	https://www.ncbi.nlm.nih.gov/clinvar/variation/184493/
NM_000023.2(SGCA):c.657C>T	concordant	unreportable	unreportable	290709	0		1	SCV000345321	Uncertain significance	EGL Genetic Diagn	6/30/2017	SGCA	chr17	48246525	48246525	http://gnomad.b	3.08E-05	AFR	6.53E-05	0		0	0	https://www.ncbi.nlm.nih.gov/clinvar/variation/290709/
NM_000152.3(GAA):c.1757C>T	concordant	unreportable	unreportable	500702	0		1	SCV000706759	Uncertain significance	EGL Genetic Diagn	6/30/2017	GAA	chr17	78086379	78086379	http://gnomad.b	2.50E-05	EAS	0.0002329639974	0		0	0	https://www.ncbi.nlm.nih.gov/clinvar/variation/500702/
NM_000071.2(CBS):c.1563C>T	concordant	unreportable	unreportable	263389	0		2	SCV000317679;S(	Likely benign;Likely benign	Ambry Genetics;Ge	2017-11-14;2017-1	CBS	chr21	44474083	44474083	http://gnomad.b	5.62E-06	EAS	5.80E-05	0		0	0	https://www.ncbi.nlm.nih.gov/clinvar/variation/263389/
NM_000018.3(ACADVL):c.1328T>G	concordant	unreportable	unreportable	285852	0		1	SCV000339046	Uncertain significance	EGL Genetic Diagn	6/30/2017	ACADVL	chr17	7127190	7127190		2.83E-06					0	0	https://www.ncbi.nlm.nih.gov/clinvar/variation/285852/
NM_000487.5(ARSA):c.474C>A	concordant	reportable	reportable	265461	1		0	SCV000322395	Pathogenic	GeneDx	11/28/2017	ARSA	chr22	51065472	51065472	http://gnomad.b	2.81E-06	AMR	0.0002700760087	0		0	0	https://www.ncbi.nlm.nih.gov/clinvar/variation/265461/
NM_000051.3(ATM):c.5825C>T	concordant	unreportable	unreportable	181999	0		2	SCV000209789;S(	Uncertain significance;Unc	GeneDx;Invitae	2017-11-28;2017-0	ATM	chr11	108180949	108180949		2.78E-06					0	0	https://www.ncbi.nlm.nih.gov/clinvar/variation/181999/
NM_000053.3(ATP7B):c.3061A>G	concordant	unreportable	unreportable	430276	0		1	SCV000583047	Uncertain significance	GeneDx	11/28/2017	ATP7B	chr13	52518427	52518427	http://gnomad.b	1.12E-05	NFE	2.74E-05	0		0	0	https://www.ncbi.nlm.nih.gov/clinvar/variation/430276/
NM_000492.3(CFTR):c.1934T>A	concordant	unreportable	unreportable	495905	0		1	SCV000696879	Uncertain significance	Laboratory Corpor	1/25/2018	CFTR	chr7	117232155	117232155	http://gnomad.b	1.93E-05	AFR	6.54E-05	0		0	0	https://www.ncbi.nlm.nih.gov/clinvar/variation/495905/
NM_000492.3(CFTR):c.94C>A	concordant	unreportable	unreportable	502597	0		1	SCV000709400	Uncertain significance	EGL Genetic Diagn	6/30/2017	CFTR	chr7	117144347	117144347	http://gnomad.b	1.93E-06	SAS	3.25E-05	0		0	0	https://www.ncbi.nlm.nih.gov/clinvar/variation/502597/
NM_000492.3(CFTR):c.1571G>A	concordant	unreportable	unreportable	495901	0		1	SCV000696851	Uncertain significance	Laboratory Corpor	1/25/2018	CFTR	chr7	117199696	117199696		1.93E-06					0	0	https://www.ncbi.nlm.nih.gov/clinvar/variation/495901/
NM_001128227.2(GNE):c.841C>A	concordant	unreportable	unreportable	283249	0		1	SCV000335233	Uncertain significance	EGL Genetic Diagn	6/30/2017	GNE	chr9	36236850	36236850	http://gnomad.b	2.80E-06	NFE	8.95E-06	0		0	0	https://www.ncbi.nlm.nih.gov/clinvar/variation/283249/
NM_001128227.2(GNE):c.2098G>A	concordant	unreportable	unreportable	290224	0		2	SCV000701431;S(	Uncertain significance;Unc	EGL Genetic Diagn	2017-06-30;2017-1	GNE	chr9	36217526	36217526	http://gnomad.b	8.40E-06	ASJ	0.0001015230009	0		0	0	https://www.ncbi.nlm.nih.gov/clinvar/variation/290224/
NM_001128227.2(GNE):c.1394A>G	concordant	unreportable	unreportable	499709	0		1	SCV000705342	Uncertain significance	EGL Genetic Diagn	6/30/2017	GNE	chr9	36223480	36223480	http://gnomad.b	2.80E-06	FIN	4.49E-05	0		0	0	https://www.ncbi.nlm.nih.gov/clinvar/variation/499709/
NM_000352.3(ABCC8):c.2695-1G>C	concordant	reportable	reportable	371626	1		0	SCV000696586	Pathogenic	Laboratory Corpor	1/25/2018	ABCC8	chr11	17430065	17430065		1.03E-05					0	0	https://www.ncbi.nlm.nih.gov/clinvar/variation/371626/
NM_000352.3(ABCC8):c.4119+1G>A	concordant	reportable	reportable	210078	1		0	SCV000246299	Pathogenic	Genetic Services La	9/15/2015	ABCC8	chr11	17418462	17418462		7.74E-06					0	0	https://www.ncbi.nlm.nih.gov/clinvar/variation/210078/
NM_000352.3(ABCC8):c.4308-2A>G	concordant	reportable	reportable	280114	1		0	SCV000329971	Pathogenic	GeneDx	11/28/2017	ABCC8	chr11	17416824	17416824	http://gnomad.b	2.58E-06	NFE	3.72E-05	0		0	0	https://www.ncbi.nlm.nih.gov/clinvar/variation/280114/
NM_000492.3(CFTR):c.870-2A>G	concordant	reportable	reportable	495962	1		0	SCV000697052	Likely pathogenic	Laboratory Corpor	1/25/2018	CFTR	chr7	117180152	117180152	http://gnomad.b	3.85E-06	AMR	8.96E-05	0		0	0	https://www.ncbi.nlm.nih.gov/clinvar/variation/495962/
NM_000271.4(NPC1):c.3246-2A>G	concordant	reportable	reportable	281939	2		0	SCV000332997;S(	Pathogenic;Pathogenic	EGL Genetic Diagn	2017-06-30;2017-0	NPC1	chr18	21115666	21115666	http://gnomad.b	5.72E-06	AMR	2.98E-05	0		0	0	https://www.ncbi.nlm.nih.gov/clinvar/variation/281939/
NM_000492.3(CFTR):c.340_342delAA(	concordant	unreportable	unreportable	439075	0		1	SCV000601097	Uncertain significance	Quest Diagnostics I	8/1/2017	CFTR	chr7	117171018	117171021		1.93E-06					0	0	https://www.ncbi.nlm.nih.gov/clinvar/variation/439075/
NM_000487.5(ARSA):c.930C>T	concordant	unreportable	unreportable	285844	0		1	SCV000339037	Uncertain significance	EGL Genetic Diagn	6/30/2017	ARSA	chr22	51064631	51064631		3.10E-05					0	0	https://www.ncbi.nlm.nih.gov/clinvar/variation/285844/
NM_004937.2(CTNS):c.1008T>G	concordant	unreportable	unreportable	322845	0		1	SCV000402246	Uncertain significance	Illumina Clinical Se	10/18/2016	CTNS	chr17	3563567	3563567	http://gnomad.b	4.15E-05	ASJ	0.0005076139933	0		0	0	https://www.ncbi.nlm.nih.gov/clinvar/variation/322845/
NM_012203.1(GRHPR):c.812C>T	concordant	unreportable	unreportable	366860	0		1	SCV000480142	Uncertain significance	Illumina Clinical Se	10/18/2016	GRHPR	chr9	37432082	37432082	http://gnomad.b	7.48E-05	SAS	0.0001624330034	0		0	0	https://www.ncbi.nlm.nih.gov/clinvar/variation/366860/
NM_018941.3(CLN8):c.59C>G	concordant	unreportable	unreportable	205203	0		2	SCV000240713;S(	Uncertain significance;Unc	GeneDx;Invitae	2017-11-28;2017-1	CLN8	chr8	1719279	1719279	http://gnomad.b	7.84E-05	AMR	0.0002977790136	0		0	0	https://www.ncbi.nlm.nih.gov/clinvar/variation/205203/
NM_006261.4(PROP1):c.592T>C	concordant	unreportable	unreportable	286901	0		2	SCV000340493;S(	Uncertain significance;Unc	EGL Genetic Diagn	2017-06-30;2016-1	PROP1	chr5	177419799	177419799	http://gnomad.b	4.79E-05	SAS	0.0004381829931	0		0	0	https://www.ncbi.nlm.nih.gov/clinvar/variation/286901/
NM_000051.3(ATM):c.4545C>T	concordant	unreportable	unreportable	229709	0		1	SCV000558434	Likely benign	Invitae	3/14/2017	ATM	chr11	108163454	108163454	http://gnomad.b	5.57E-06	NFE	8.96E-06	0		0	0	https://www.ncbi.nlm.nih.gov/clinvar/variation/229709/
NM_000071.2(CBS):c.1209G>A	concordant	unreportable	unreportable	471356	0		1	SCV000649823	Likely benign	Invitae	10/5/2017	CBS	chr21	44479350	44479350	http://gnomad.b	3.94E-05	SAS	6.50E-05	0		0	0	https://www.ncbi.nlm.nih.gov/clinvar/variation/471356/
NM_000051.3(ATM):c.2728A>G	concordant	unreportable	unreportable	453431	0		1	SCV000622355	Uncertain significance	Invitae	10/5/2017	ATM	chr11	108139226	108139226	http://gnomad.b	1.39E-05	EAS	0.0001739330037	0		0	0	https://www.ncbi.nlm.nih.gov/clinvar/variation/453431/
NM_000310.3(PPT1):c.367G>A	concordant	unreportable	unreportable	206644	0		1	SCV000357397	Uncertain significance	Illumina Clinical Se	10/18/2016	PPT1	chr1	40557067	40557067	http://gnomad.b	1.67E-05	SAS	3.26E-05	0		0	0	https://www.ncbi.nlm.nih.gov/clinvar/variation/206644/
NM_000051.3(ATM):c.1370G>A	concordant	unreportable	unreportable	453362	0		1	SCV000622252	Uncertain significance	Invitae	10/5/2017	ATM	chr11	108121562	108121562	http://gnomad.b	1.39E-05	SAS	9.75E-05	0		0	0	https://www.ncbi.nlm.nih.gov/clinvar/variation/453362/
NM_000071.2(CBS):c.1498_1499delT(	concordant	reportable	reportable	499845	1		0	SCV000705553	Pathogenic	EGL Genetic Diagn	6/30/2017	CBS	chr21	44476965	44476967		2.81E-06					0	0	https://www.ncbi.nlm.nih.gov/clinvar/variation/499845/
NM_002485.4(NBN):c.2139T>C	concordant	unreportable	unreportable	184232	0		3	SCV000685754;S(	Likely benign;Likely benign	Color Genomics, In	2017-10-26;2017-1	NBN	chr8	90955526	90955526	http://gnomad.b	1.67E-05	NFE	3.59E-05	0		0	0	https://www.ncbi.nlm.nih.gov/clinvar/variation/184232/
NM_000492.3(CFTR):c.2659A>C	concordant	unreportable	unreportable	288458	0		1	SCV000342567	Uncertain significance	EGL Genetic Diagn	6/30/2017	CFTR	chr7	117243587	117243587	http://gnomad.b	1.16E-05	AMR	0.000209054997	0		0	0	https://www.ncbi.nlm.nih.gov/clinvar/variation/288458/
NM_005562.2(LAMC2):c.1675G>A	concordant	unreportable	unreportable	294006	0		1	SCV000351677	Uncertain significance	Illumina Clinical Se	10/18/2016	LAMC2	chr1	183197715	183197715	http://gnomad.b	1.68E-05	SAS	9.75E-05	0		0	0	https://www.ncbi.nlm.nih.gov/clinvar/variation/294006/
NM_000520.4(HEXA):c.574G>A	concordant	unreportable	unreportable	496013	0		1	SCV000697172	Uncertain significance	Laboratory Corpor	1/25/2018	HEXA	chr15	72643572	72643572	http://gnomad.b	1.17E-05	NFE	8.95E-06	0		0	0	https://www.ncbi.nlm.nih.gov/clinvar/variation/496013/
NM_002485.4(NBN):c.156_157delTT	concordant	reportable	reportable	371121	5		0	SCV000690667;S(	Pathogenic;Likely pathoger	Color Genomics, In	2017-12-21;2017-1	NBN	chr8	90994963	90994965	http://gnomad.b	1.12E-05	NFE	3.58E-05	0		0	0	https://www.ncbi.nlm.nih.gov/clinvar/variation/371121/
NM_018941.3(CLN8):c.488C>T	concordant	unreportable	unreportable	205209	0		1	SCV000240720	Uncertain significance	GeneDx	11/28/2017	CLN8	chr8	1719708	1719708	http://gnomad.b	2.80E-06	NFE	8.95E-06	0		0	0	https://www.ncbi.nlm.nih.gov/clinvar/variation/205209/
NM_000051.3(ATM):c.7904C>G	concordant	unreportable	unreportable	453709	0		1	SCV000622786	Uncertain significance	Invitae	10/5/2017	ATM	chr11	108203604	108203604		2.78E-06					0	0	https://www.ncbi.nlm.nih.gov/clinvar/variation/453709/
NM_000051.3(ATM):c.2346A>G	concordant	unreportable	unreportable	181852	0		3	SCV000687375;S(	Likely benign;Uncertain sig	Color Genomics, In	2017-12-21;2017-0	ATM	chr11	108128303	108128303	http://gnomad.b	4.46E-05	AMR	0.0004764460027	0		1	0	https://www.ncbi.nlm.nih.gov/clinvar/variation/181852/
NM_000051.3(ATM):c.7511T>C	concordant	unreportable	unreportable	407635	0		2	SCV000682419;S(	Uncertain significance;Unc	Color Genomics, In	2017-10-26;2017-0	ATM	chr11	108201144	108201144		2.78E-06					0	0	https://www.ncbi.nlm.nih.gov/clinvar/variation/407635/
NM_000271.4(NPC1):c.2373+14A>G	concordant	unreportable	unreportable	326261	0		1	SCV000407863	Uncertain significance	Illumina Clinical Se	10/18/2016	NPC1	chr18	21121256	21121256	http://gnomad.b	6.58E-05	ASJ	0.0004061739892	0		0	0	https://www.ncbi.nlm.nih.gov/clinvar/variation/326261/
NM_000136.2(FANCC):c.1513G>A	concordant	unreportable	unreportable	220138	0		2	SCV000618178;S(	Uncertain significance;Unc	GeneDx;Invitae	2017-11-28;2017-1	FANCC	chr9	97869368	97869368	http://gnomad.b	4.28E-05	EAS	0.0001739330037	0		0	0	https://www.ncbi.nlm.nih.gov/clinvar/variation/220138/
NM_000152.3(GAA):c.607C>T	concordant	unreportable	unreportable	386960	0		1	SCV000528796	Uncertain significance	GeneDx	11/28/2017	GAA	chr17	78079608	78079608	http://gnomad.b	1.95E-05	AMR	0.0001191260017	0		0	0	https://www.ncbi.nlm.nih.gov/clinvar/variation/386960/
NM_000492.3(CFTR):c.1211G>T	concordant	unreportable	unreportable	455760	0		1	SCV000625724	Uncertain significance	Invitae	10/5/2017	CFTR	chr7	117188696	117188696		1.93E-06					0	0	https://www.ncbi.nlm.nih.gov/clinvar/variation/455760/
NM_000051.3(ATM):c.6057T>C	concordant	unreportable	unreportable	490639	0		1	SCV000687669	Likely benign	Color Genomics, In	12/21/2017	ATM	chr11	108186600	108186600	http://gnomad.b	1.95E-05	EAS	0.0001160629981	0		0	0	https://www.ncbi.nlm.nih.gov/clinvar/variation/490639/
NM_000023.2(SGCA):c.766G>A	concordant	unreportable	unreportable	500930	0		1	SCV000707096	Uncertain significance	EGL Genetic Diagn	6/30/2017	SGCA	chr17	48247522	48247522	http://gnomad.b	4.76E-05	AMR	0.0001786670036	0		0	0	https://www.ncbi.nlm.nih.gov/clinvar/variation/500930/
NM_138694.3(PKHD1):c.1796C>T	concordant	unreportable	unreportable	282369	0		1	SCV000333817	Uncertain significance	EGL Genetic Diagn	6/30/2017	PKHD1	chr6	51920425	51920425	http://gnomad.b	4.73E-05	NFE	9.87E-05	0		0	0	https://www.ncbi.nlm.nih.gov/clinvar/variation/282369/
NM_000071.2(CBS):c.456C>T	concordant	unreportable	unreportable	385090	0		1	SCV000526227	Likely benign	GeneDx	11/28/2017	CBS	chr21	44485801	44485801		1.12E-05					0	0	https://www.ncbi.nlm.nih.gov/clinvar/variation/385090/
NM_002485.4(NBN):c.867G>T	concordant	unreportable	unreportable	411768	0		1	SCV000553087	Uncertain significance	Invitae	3/14/2017	NBN	chr8	90982621	90982621		2.79E-06					0	0	https://www.ncbi.nlm.nih.gov/clinvar/variation/411768/
NM_024649.4(BBS1):c.1088T>C	concordant	unreportable	unreportable	462951	0		1	SCV000636511	Uncertain significance	Invitae	10/5/2017	BBS1	chr11	66291331	66291331	http://gnomad.b	7.52E-05	SAS	0.0007471899735	0		0	0	https://www.ncbi.nlm.nih.gov/clinvar/variation/462951/
NM_014625.2(NPHS2):c.148C>T	concordant	unreportable	unreportable	293851	0		1	SCV000351507	Uncertain significance	Illumina Clinical Se	10/18/2016	NPHS2	chr1	179544852	179544852	http://gnomad.b	1.97E-05	EAS	0.0002674749994	0		0	0	https://www.ncbi.nlm.nih.gov/clinvar/variation/293851/
NM_001042432.1(CLN3):c.1210C>T	concordant	unreportable	unreportable	421929	0		1	SCV000571271	Uncertain significance	GeneDx	11/28/2017	CLN3	chr16	28488944	28488944	http://gnomad.b	5.56E-06	SAS	0.0001299459982	0		0	0	https://www.ncbi.nlm.nih.gov/clinvar/variation/421929/
NM_000051.3(ATM):c.497-3A>G	concordant	unreportable	unreportable	490596	0		1	SCV000687591	Uncertain significance	Color Genomics, In	12/21/2017	ATM	chr11	108114677	108114677		2.78E-06					0	0	https://www.ncbi.nlm.nih.gov/clinvar/variation/490596/
NM_000271.4(NPC1):c.1671C>T	concordant	unreportable	unreportable	500944	0		1	SCV000707118	Uncertain significance	EGL Genetic Diagn	6/30/2017	NPC1	chr18	21128056	21128056	http://gnomad.b	2.00E-05	AFR	0.0001306850027	0		0	0	https://www.ncbi.nlm.nih.gov/clinvar/variation/500944/
NM_000051.3(ATM):c.5473C>A	concordant	unreportable	unreportable	481296	0		1	SCV000687620	Uncertain significance	Color Genomics, In	12/21/2017	ATM	chr11	108173733	108173733		5.57E-06					0	0	https://www.ncbi.nlm.nih.gov/clinvar/variation/481296/
NM_000051.3(ATM):c.1834C>T	concordant	unreportable	unreportable	453387	0		1	SCV000622286	Uncertain significance	Invitae	10/5/2017	ATM	chr11	108123575	108123575	http://gnomad.b	1.39E-05	SAS	0.0001301489974	0		0	0	https://www.ncbi.nlm.nih.gov/clinvar/variation/453387/
NM_000243.2(MEFV):c.1736G>A	concordant	unreportable	unreportable	319109	0		1	SCV000396763	Uncertain significance	Illumina Clinical Se	10/18/2016	MEFV	chr16	3294277	3294277	http://gnomad.b	1.96E-05	AFR	0.0003920540039	0		0	0	https://www.ncbi.nlm.nih.gov/clinvar/variation/319109/
NM_000243.2(MEFV):c.66G>A	concordant	unreportable	unreportable	319119	0		1	SCV000396788	Uncertain significance	Illumina Clinical Se	10/18/2016	MEFV	chr16	3306522	3306522	http://gnomad.b	6.72E-05	SAS	0.0009096229915	0		0	0	https://www.ncbi.nlm.nih.gov/clinvar/variation/319119/
NM_001079802.1(FKTN):c.382C>T	concordant	unreportable	unreportable	264196	0		2	SCV000319948;S(	Uncertain significance;Unc	Ambry Genetics;EG	2017-11-14;2017-0	FKTN	chr9	108366508	108366508	http://gnomad.b	4.25E-05	AFR	6.54E-05	0		0	0	https://www.ncbi.nlm.nih.gov/clinvar/variation/264196/
NM_004937.2(CTNS):c.1083G>A	concordant	unreportable	unreportable	322846	0		1	SCV000402248	Uncertain significance	Illumina Clinical Se	10/18/2016	CTNS	chr17	3563642	3563642	http://gnomad.b	2.67E-05	AMR	8.93E-05	0		0	0	https://www.ncbi.nlm.nih.gov/clinvar/variation/322846/
NM_002485.4(NBN):c.34G>A	concordant	unreportable	unreportable	239195	0		1	SCV000287469	Uncertain significance	Invitae	6/10/2016	NBN	chr8	90996756	90996756		2.79E-06					0	0	https://www.ncbi.nlm.nih.gov/clinvar/variation/239195/
NM_000478.4(ALPL):c.648C>T	concordant	unreportable	unreportable	295545	0		1	SCV000354301	Uncertain significance	Illumina Clinical Se	10/18/2016	ALPL	chr1	21890709	21890709	http://gnomad.b	1.69E-05	NFE	0.0002069350012	0		0	0	https://www.ncbi.nlm.nih.gov/clinvar/variation/295545/
NM_138694.3(PKHD1):c.4265G>C	concordant	unreportable	unreportable	285782	0		1	SCV000338953	Uncertain significance	EGL Genetic Diagn	6/30/2017	PKHD1	chr6	51890343	51890343		2.78E-06					0	0	https://www.ncbi.nlm.nih.gov/clinvar/variation/285782/
NM_002225.3(IVD):c.627delT	concordant	reportable	reportable	459930	2		0	SCV000710512;S(	Pathogenic;Pathogenic	GeneDx;Invitae	2018-03-26;2017-1	IVD	chr15	40703819	40703820	http://gnomad.b	4.48E-05	NFE	5.37E-05	0		0	0	https://www.ncbi.nlm.nih.gov/clinvar/variation/459930/
NM_138694.3(PKHD1):c.4017G>C	concordant	unreportable	unreportable	285781	0		1	SCV000338952	Uncertain significance	EGL Genetic Diagn	6/30/2017	PKHD1	chr6	51890591	51890591		5.56E-06					0	0	https://www.ncbi.nlm.nih.gov/clinvar/variation/285781/
NM_138694.3(PKHD1):c.3731A>C	concordant	unreportable	unreportable	285780	0		1	SCV000338951	Uncertain significance	EGL Genetic Diagn	6/30/2017	PKHD1	chr6	51890877	51890877		2.78E-06					0	0	https://www.ncbi.nlm.nih.gov/clinvar/variation/285780/
NM_000136.2(FANCC):c.166-4_166-1(	concordant	unreportable	unreportable	420828	0		1	SCV000569812	Uncertain significance	GeneDx	11/28/2017	FANCC	chr9	98009798	98009798	http://gnomad.b	2.38E-06	NFE	9.01E-06	0		0	0	https://www.ncbi.nlm.nih.gov/clinvar/variation/420828/
NM_000051.3(ATM):c.246A>G	concordant	unreportable	unreportable	230836	0		1	SCV000521701	Likely benign	GeneDx	11/28/2017	ATM	chr11	108099965	108099965	http://gnomad.b	1.67E-05	NFE	1.79E-05	0		0	0	https://www.ncbi.nlm.nih.gov/clinvar/variation/230836/

Supplemental Table 2

NM_001079802.1(FKTN):c.1248C>T	concordant	unreportable	unreportable	386541	0	2	SCV000613313;S(	Likely benign;Likely benign	Athena Diagnostics	2017-08-17;2017-1	FKTN	chr9	108397407	108397407	http://gnomad.b	1.42E-05	AFR	6.54E-05	0		0	0	https://www.ncbi.nlm.nih.gov/clinvar/variation/386541/
NM_000051.3(ATM):c.3998A>T	concordant	unreportable	unreportable	231126	0	2	SCV000546837;S(	Uncertain significance;Unc	Invitae;Laboratory	2017-03-14;2018-0	ATM	chr11	108158331	108158331	http://gnomad.b	2.78E-06	AFR	6.54E-05	0		0	0	https://www.ncbi.nlm.nih.gov/clinvar/variation/231126/
NM_000051.3(ATM):c.5550A>G	concordant	unreportable	unreportable	184456	0	2	SCV000682270;S(	Likely benign;Likely benign	Color Genomics, In	2017-10-26;2017-1	ATM	chr11	108175455	108175455	http://gnomad.b	1.11E-05	NFE	8.98E-06	0		0	0	https://www.ncbi.nlm.nih.gov/clinvar/variation/184456/
NM_018941.3(CLN8):c.806A>G	concordant	unreportable	unreportable	205197	0	1	SCV000240706	Uncertain significance	GeneDx	11/28/2017	CLN8	chr8	1728678	1728678	http://gnomad.b	1.12E-05	AFR	0.0002617120044	0		0	0	https://www.ncbi.nlm.nih.gov/clinvar/variation/205197/
NM_033056.3(PCDH15):c.4049G>A	concordant	unreportable	unreportable	499167	0	1	SCV000704515	Uncertain significance	EGL Genetic Diagn	6/30/2017	PCDH15	chr10	55591228	55591228	http://gnomad.b	6.94E-05	EAS	0.001391469967	1		0	0	https://www.ncbi.nlm.nih.gov/clinvar/variation/499167/
NM_000051.3(ATM):c.2322T>C	concordant	unreportable	unreportable	231158	0	1	SCV000558456	Likely benign	Invitae	10/5/2017	ATM	chr11	108128279	108128279	http://gnomad.b	1.39E-05	AMR	0.0001786670036	0		0	0	https://www.ncbi.nlm.nih.gov/clinvar/variation/231158/
NM_000051.3(ATM):c.9045G>A	concordant	unreportable	unreportable	186935	0	3	SCV000687875;S(	Likely benign;Likely benign,	Color Genomics, In	2017-12-21;2017-1	ATM	chr11	108236109	108236109		1.67E-05					0	0	https://www.ncbi.nlm.nih.gov/clinvar/variation/186935/
NM_004543.4(NEB):c.475C>T	concordant	unreportable	unreportable	331541	0	1	SCV000417055	Uncertain significance	Illumina Clinical Se	10/18/2016	NEB	chr2	152581403	152581403	http://gnomad.b	2.32E-05	NFE	4.48E-05	0		0	0	https://www.ncbi.nlm.nih.gov/clinvar/variation/331541/
NM_001042432.1(CLN3):c.447G>T	concordant	unreportable	unreportable	457953	0	1	SCV000628931	Uncertain significance	Invitae	10/5/2017	CLN3	chr16	28498790	28498790	http://gnomad.b	1.67E-05	AMR	5.96E-05	0		0	0	https://www.ncbi.nlm.nih.gov/clinvar/variation/457953/
NM_000051.3(ATM):c.903T>G	concordant	unreportable	unreportable	231747	0	1	SCV000622879	Likely benign	Invitae	10/5/2017	ATM	chr11	108117692	108117692		2.78E-06					0	0	https://www.ncbi.nlm.nih.gov/clinvar/variation/231747/
NM_000051.3(ATM):c.3514G>C	concordant	unreportable	unreportable	481072	0	1	SCV000682134	Uncertain significance	Color Genomics, In	10/26/2017	ATM	chr11	108151833	108151833		5.57E-06					0	0	https://www.ncbi.nlm.nih.gov/clinvar/variation/481072/
NM_000051.3(ATM):c.5707A>G	concordant	unreportable	unreportable	229836	0	1	SCV000546955	Uncertain significance	Invitae	3/14/2017	ATM	chr11	108178656	108178656	http://gnomad.b	5.57E-06	AFR	6.54E-05	0		0	0	https://www.ncbi.nlm.nih.gov/clinvar/variation/229836/
NM_000136.2(FANCC):c.790A>G	concordant	unreportable	unreportable	220568	0	1	SCV000261209	Uncertain significance	Invitae	1/6/2016	FANCC	chr9	97897681	97897681	http://gnomad.b	7.13E-06			0		0	0	https://www.ncbi.nlm.nih.gov/clinvar/variation/220568/
NM_138694.3(PKHD1):c.1737G>A	concordant	unreportable	unreportable	498711	0	1	SCV000703855	Uncertain significance	EGL Genetic Diagn	6/30/2017	PKHD1	chr6	51920484	51920484	http://gnomad.b	4.45E-05	NFE	7.18E-05	0		0	0	https://www.ncbi.nlm.nih.gov/clinvar/variation/498711/
NM_000051.3(ATM):c.1370G>T	concordant	unreportable	unreportable	219530	0	2	SCV000571106;S(	Uncertain significance;Unc	GeneDx;Invitae	2017-11-28;2017-1	ATM	chr11	108121562	108121562	http://gnomad.b	5.57E-06	SAS	3.25E-05	0		0	0	https://www.ncbi.nlm.nih.gov/clinvar/variation/219530/
NM_000051.3(ATM):c.7927+13delT	concordant	unreportable	unreportable	422794	0	2	SCV000682444;S(	Likely benign;Likely benign	Color Genomics, In	2017-10-26;2017-1	ATM	chr11	108203632	108203633	http://gnomad.b	5.57E-06	EAS	5.83E-05	0		0	0	https://www.ncbi.nlm.nih.gov/clinvar/variation/422794/
NM_000051.3(ATM):c.7116T>C	concordant	unreportable	unreportable	187526	0	2	SCV000682388;S(	Likely benign;Likely benign	Color Genomics, In	2017-10-26;2017-1	ATM	chr11	108199774	108199774	http://gnomad.b	2.23E-05	AFR	6.54E-05	0		0	0	https://www.ncbi.nlm.nih.gov/clinvar/variation/187526/
NM_000227.3(LAMA3):c.3500G>A	concordant	unreportable	unreportable	326333	0	1	SCV000407994	Uncertain significance	Illumina Clinical Se	10/18/2016	LAMA3	chr18	21508620	21508620		5.60E-06					0	0	https://www.ncbi.nlm.nih.gov/clinvar/variation/326333/
NM_000057.2(BLM):c.2598A>T	concordant	unreportable	unreportable	413297	0	1	SCV000555836	Likely benign	Invitae	3/14/2017	BLM	chr15	91326094	91326094	http://gnomad.b	3.33E-05	AFR	0.0001307360071	0		0	0	https://www.ncbi.nlm.nih.gov/clinvar/variation/413297/
NM_000382.2(ALDH3A2):c.566T>C	concordant	unreportable	unreportable	322211	0	1	SCV000401254	Uncertain significance	Illumina Clinical Se	10/18/2016	ALDH3A2	chr17	19559773	19559773	http://gnomad.b	3.08E-05	AFR	6.53E-05	0		0	0	https://www.ncbi.nlm.nih.gov/clinvar/variation/322211/
NM_001360.2(DHCR7):c.778C>G	concordant	unreportable	unreportable	281795	0	1	SCV000332777	Uncertain significance	EGL Genetic Diagn	6/30/2017	DHCR7	chr11	71149978	71149978	http://gnomad.b	5.39E-06	NFE	1.79E-05	0		0	0	https://www.ncbi.nlm.nih.gov/clinvar/variation/281795/
NM_000136.2(FANCC):c.957G>A	concordant	unreportable	unreportable	415744	0	1	SCV000560622	Likely benign	Invitae	3/14/2017	FANCC	chr9	97887407	97887407		1.66E-05					0	0	https://www.ncbi.nlm.nih.gov/clinvar/variation/415744/
NM_000071.2(CBS):c.708C>T	concordant	unreportable	unreportable	413356	0	1	SCV000555952	Likely benign	Invitae	3/14/2017	CBS	chr21	44485341	44485341	http://gnomad.b	1.97E-05	NFE	2.69E-05	0		0	0	https://www.ncbi.nlm.nih.gov/clinvar/variation/413356/
NM_000051.3(ATM):c.2150G>C	concordant	unreportable	unreportable	420303	0	2	SCV000569088;S(	Uncertain significance;Unc	GeneDx;Invitae	2017-11-28;2017-1	ATM	chr11	108126967	108126967	http://gnomad.b	2.23E-05	ASJ	0.0003048159997	0		0	0	https://www.ncbi.nlm.nih.gov/clinvar/variation/420303/
NM_000051.3(ATM):c.8066A>G	concordant	unreportable	unreportable	489594	0	1	SCV000682452	Uncertain significance	Color Genomics, In	10/26/2017	ATM	chr11	108205751	108205751	http://gnomad.b	1.11E-05	NFE	8.97E-06	0		0	0	https://www.ncbi.nlm.nih.gov/clinvar/variation/489594/
NM_000271.4(NPC1):c.1153A>G	concordant	unreportable	unreportable	500645	0	1	SCV000706679	Uncertain significance	EGL Genetic Diagn	6/30/2017	NPC1	chr18	21136380	21136380	http://gnomad.b	2.29E-05	AMR	0.000238321998	0		0	0	https://www.ncbi.nlm.nih.gov/clinvar/variation/500645/
NM_000051.3(ATM):c.6141A>G	concordant	unreportable	unreportable	453614	0	1	SCV000622642	Likely benign	Invitae	10/5/2017	ATM	chr11	108186783	108186783		2.78E-06					0	0	https://www.ncbi.nlm.nih.gov/clinvar/variation/453614/
NM_017739.3(POMGNT1):c.172A>G	concordant	unreportable	unreportable	288926	0	2	SCV000343178;S(	Uncertain significance;Unc	EGL Genetic Diagn	2017-06-30;2017-1	POMGNT1	chr1	46662705	46662705	http://gnomad.b	1.40E-05	NFE	2.69E-05	0		0	0	https://www.ncbi.nlm.nih.gov/clinvar/variation/288926/
NM_000136.2(FANCC):c.440C>T	concordant	unreportable	unreportable	182469	0	2	SCV000211035;S(	Uncertain significance;Unc	GeneDx;Invitae	2017-11-28;2016-0	FANCC	chr9	97934335	97934335	http://gnomad.b	2.14E-05	NFE	1.79E-05	0		0	0	https://www.ncbi.nlm.nih.gov/clinvar/variation/182469/
NM_000051.3(ATM):c.6330C>T	concordant	unreportable	unreportable	215588	0	2	SCV000682324;S(	Likely benign;Likely benign	Color Genomics, In	2017-10-26;2017-1	ATM	chr11	108188231	108188231	http://gnomad.b	1.11E-05	EAS	0.0004060319916	0		0	0	https://www.ncbi.nlm.nih.gov/clinvar/variation/215588/
NM_000152.3(GAA):c.2391C>T	concordant	unreportable	unreportable	282388	0	1	SCV000333841	Uncertain significance	EGL Genetic Diagn	6/30/2017	GAA	chr17	78091458	78091458	http://gnomad.b	1.11E-05	EAS	5.81E-05	0		0	0	https://www.ncbi.nlm.nih.gov/clinvar/variation/282388/
NM_000152.3(GAA):c.1194+17G>T	concordant	unreportable	unreportable	383995	0	1	SCV000524634	Likely benign	GeneDx	11/28/2017	GAA	chr17	78082423	78082423	http://gnomad.b	0.0001417446262	AMR	0.0002089799964	0		0	0	https://www.ncbi.nlm.nih.gov/clinvar/variation/383995/
NM_000520.4(HEXA):c.1033G>A	concordant	unreportable	unreportable	317044	0	1	SCV000393836	Uncertain significance	Illumina Clinical Se	10/18/2016	HEXA	chr15	72640429	72640429	http://gnomad.b	1.88E-05	NFE	1.79E-05	0		0	0	https://www.ncbi.nlm.nih.gov/clinvar/variation/317044/
NM_005476.5(GNE):c.487A>G	concordant	unreportable	unreportable	283110	0	1	SCV000335007	Uncertain significance	EGL Genetic Diagn	6/30/2017	GNE	chr9	36246157	36246157	http://gnomad.b	2.80E-05	SAS	0.0001949190046	0		0	0	https://www.ncbi.nlm.nih.gov/clinvar/variation/283110/
NM_000057.2(BLM):c.191A>C	concordant	unreportable	unreportable	287232	0	2	SCV000340939;S(	Uncertain significance;Unc	EGL Genetic Diagn	2017-06-30;2017-1	BLM	chr15	91292689	91292689	http://gnomad.b	2.38E-06	NFE	8.97E-06	0		0	0	https://www.ncbi.nlm.nih.gov/clinvar/variation/287232/
NM_000098.2(CPT2):c.1408G>T	concordant	unreportable	unreportable	460424	0	1	SCV000632588	Uncertain significance	Invitae	10/5/2017	CPT2	chr1	53676754	53676754	http://gnomad.b	6.44E-05	EAS	0.0004059379862	0		0	0	https://www.ncbi.nlm.nih.gov/clinvar/variation/460424/
NM_000159.2(GCDH):c.1305G>A	concordant	unreportable	unreportable	386429	0	1	SCV000528101	Likely benign	GeneDx	11/28/2017	GCDH	chr19	13010343	13010343	http://gnomad.b	7.59E-05	SAS	0.0004223250144	0		0	0	https://www.ncbi.nlm.nih.gov/clinvar/variation/386429/
NM_000136.2(FANCC):c.1485G>C	concordant	unreportable	unreportable	377848	0	1	SCV000512972	Likely benign	GeneDx	11/28/2017	FANCC	chr9	97869396	97869396	http://gnomad.b	2.38E-06	AFR	0.0001960780064	0		0	0	https://www.ncbi.nlm.nih.gov/clinvar/variation/377848/
NM_000382.2(ALDH3A2):c.1144G>A	concordant	unreportable	unreportable	390465	0	1	SCV000533303	Uncertain significance	GeneDx	11/28/2017	ALDH3A2	chr17	19568297	19568297	http://gnomad.b	2.52E-05	SAS	0.0003573519934	0		0	0	https://www.ncbi.nlm.nih.gov/clinvar/variation/390465/
NM_000017.2(ACADS):c.988_990delC(	concordant	reportable	reportable	203569	1	0	SCV000238629	Pathogenic	GeneDx	11/28/2017	ACADS	chr12	121176676	121176679		0.0001614200433					0	0	https://www.ncbi.nlm.nih.gov/clinvar/variation/203569/
NM_000071.2(CBS):c.210-3C>T	concordant	unreportable	unreportable	264609	0	1	SCV000320641	Uncertain significance	Ambry Genetics	11/14/2017	CBS	chr21	44488728	44488728	http://gnomad.b	1.12E-05	NFE	2.75E-05	0		0	0	https://www.ncbi.nlm.nih.gov/clinvar/variation/264609/
NM_000071.2(CBS):c.18C>T	concordant	unreportable	unreportable	413358	0	1	SCV000555955	Likely benign	Invitae	3/14/2017	CBS	chr21	44492286	44492286	http://gnomad.b	3.94E-05	SAS	0.0003264349943	0		0	0	https://www.ncbi.nlm.nih.gov/clinvar/variation/413358/
NM_000152.3(GAA):c.794G>A	concordant	unreportable	unreportable	498281	0	1	SCV000703218	Uncertain significance	EGL Genetic Diagn	6/30/2017	GAA	chr17	78081457	78081457	http://gnomad.b	6.11E-05	SAS	0.0001949190046	0		0	0	https://www.ncbi.nlm.nih.gov/clinvar/variation/498281/
NM_017890.4(VPS13B):c.1646G>A	concordant	unreportable	unreportable	361042	0	1	SCV000470769	Uncertain significance	Illumina Clinical Se	10/18/2016	VPS13B	chr8	100148975	100148975		1.40E-05					0	0	https://www.ncbi.nlm.nih.gov/clinvar/variation/361042/
NM_000227.3(LAMA3):c.3268G>A	concordant	unreportable	unreportable	326325	0	1	SCV000407978	Uncertain significance	Illumina Clinical Se	10/18/2016	LAMA3	chr18	21501467	21501467		5.60E-06					0	0	https://www.ncbi.nlm.nih.gov/clinvar/variation/326325/
NM_000051.3(ATM):c.3843T>G	concordant	unreportable	unreportable	216206	0	1	SCV000254093	Uncertain significance	Invitae	10/5/2017	ATM	chr11	108155050	108155050		1.11E-05					0	0	https://www.ncbi.nlm.nih.gov/clinvar/variation/216206/
NM_024649.4(BBS1):c.636C>T	concordant	unreportable	unreportable	305461	0	1	SCV000373264	Uncertain significance	Illumina Clinical Se	10/18/2016	BBS1	chr11	66287132	66287132	http://gnomad.b	8.36E-06	SAS	3.25E-05	0		0	0	https://www.ncbi.nlm.nih.gov/clinvar/variation/305461/
NM_000051.3(ATM):c.2921+1G>C	concordant	reportable	reportable	188097	3	0	SCV000682088;S(	Likely pathogenic;Pathoger	Color Genomics, In	2017-10-26;2017-1	ATM	chr11	108141874	108141874	http://gnomad.b	2.78E-06	NFE	8.96E-06	0		0	0	https://www.ncbi.nlm.nih.gov/clinvar/variation/188097/
NM_000051.3(ATM):c.6891A>G	concordant	unreportable	unreportable	236759	0	1	SCV000283027	Uncertain significance	Invitae	10/5/2017	ATM	chr11	108196868	108196868	http://gnomad.b	2.78E-06	NFE	8.96E-06	0		0	0	https://www.ncbi.nlm.nih.gov/clinvar/variation/236759/
NM_000051.3(ATM):c.2579A>T	concordant	unreportable	unreportable	236692	0	2	SCV000567485;S(	Uncertain significance;Unc	GeneDx;Invitae	2017-11-28;2017-1	ATM	chr11	108138010	108138010	http://gnomad.b	8.35E-06	NFE	8.95E-06	0		0	0	https://www.ncbi.nlm.nih.gov/clinvar/variation/236692/
NM_000152.3(GAA):c.1264C>T	concordant	unreportable	unreportable	456371	0	1	SCV000626498	Uncertain significance	Invitae	10/5/2017	GAA	chr17	78082565	78082565	http://gnomad.b	8.34E-06	EAS	5.96E-05	0		0	0	https://www.ncbi.nlm.nih.gov/clinvar/variation/456371/
NM_000051.3(ATM):c.7382G>A	concordant	unreportable	unreportable	216230	0	2	SCV000682412;S(	Uncertain significance;Unc	Color Genomics, In	2017-10-26;2017-1	ATM	chr11	108201015	108201015	http://gnomad.b	2.51E-05	AMR	0.0002382649982	0		0	0	https://www.ncbi.nlm.nih.gov/clinvar/variation/216230/
NM_000232.4(SGCB):c.680G>A	concordant	unreportable	unreportable	290601	0	1	SCV000345187	Uncertain significance	EGL Genetic Diagn	6/30/2017	SGCB	chr4	52894207	52894207	http://gnomad.b	3.64E-05	AFR	0.0001307019993	0		0	0	https://www.ncbi.nlm.nih.gov/clinvar/variation/290601/
NM_000023.2(SGCA):c.37+3A>G	concordant	unreportable	unreportable	449763	0	1	SCV000618143	Uncertain significance	GeneDx	11/28/2017	SGCA	chr17	48243441	48243441	http://gnomad.b	8.40E-06	NFE	9.14E-06	0		0	0	https://www.ncbi.nlm.nih.gov/clinvar/variation/449763/
NM_001164277.1(SLC37A4):c.878T>C	concordant	unreportable	unreportable	282064	0	1	SCV000333247	Uncertain significance	EGL Genetic Diagn	6/30/2017	SLC37A4	chr11	118896783	118896783		1.12E-05					0	0	https://www.ncbi.nlm.nih.gov/clinvar/variation/282064/
NM_000152.3(GAA):c.1661C>T	concordant	unreportable	unreportable	498953	0	1	SCV000704224	Uncertain significance	EGL Genetic Diagn	6/30/2017	GAA	chr17	78085806	78085806	http://gnomad.b	1.39E-05	EAS	0.0001159820022	0		0	0	https://www.ncbi.nlm.nih.gov/clinvar/variation/498953/
NM_000051.3(ATM):c.2170G>A	concordant	unreportable	unreportable	453406	0	3	SCV000682034;S(	Uncertain significance;Unc	Color Genomics, In	2017-10-26;2017-1	ATM	chr11	108126987	108126987		5.57E-06					0	0	https://www.ncbi.nlm.nih.gov/clinvar/variation/453406/
NM_000098.2(CPT2):c.674G>A	concordant	unreportable	unreportable	197107	0	1	SCV000230027	Uncertain significance	EGL Genetic Diagn	6/30/2017	CPT2	chr1	53676020	53676020		2.80E-06					0	0	https://www.ncbi.nlm.nih.gov/clinvar/variation/197107/
NM_002485.4(NBN):c.1125-3C>T	concordant	unreportable	unreportable	140859	0	3	SCV000527317;S(	Likely benign;Uncertain sig	GeneDx;Illumina C	2017-11-28;2016-1	NBN	chr8	90967786	90967786		1.39E-05					0	0	https://www.ncbi.nlm.nih.gov/clinvar/variation/140859/
NM_014363.4(SACS):c.134C>T	concordant	unreportable	unreportable	448193	0	1	SCV000614937	Uncertain significance	Athena Diagnostics	8/17/2017	SACS	chr13	23949295	23949295		3.26E-05					0	0	https://www.ncbi.nlm.nih.gov/clinvar/variation/448193/
NM_000053.3(ATP7B):c.4185G>C	concordant	unreportable	unreportable	312377	0	1	SCV000384645	Uncertain significance	Illumina Clinical Se	10/18/2016	ATP7B	chr13	52509105	52509105	http://gnomad.b	8.42E-06	NFE	9.11E-06	0		0	0	https://www.ncbi.nlm.nih.gov/clinvar/variation/312377/
NM_000051.3(ATM):c.6823A>G	concordant	unreportable	unreportable	181885	0	3	SCV000209634;S(	Uncertain significance;Unc	GeneDx;Invitae;Lal	2017-11-28;2017-1	ATM	chr11	108196800	108196800	http://gnomad.b	2.23E-05	NFE	8.98E-06	0		0	0	https://www.ncbi.nlm.nih.gov/clinvar/variation/181885/
NM_000441.1(SLC26A4):c.371T>A	concordant	unreportable	unreportable	358494	0	1	SCV000466079	Uncertain significance	Illumina Clinical Se	10/18/2016	SLC26A4	chr7	107312649	107312649	http://gnomad.b	1.40E-05	NFE	1.79E-05	0		0	0	https://www.ncbi.nlm.nih.gov/clinvar/variation/358494/
NM_000441.1(SLC26A4):c.1050G>A	concordant	unreportable	unreportable	179617	0	1	SCV000466093	Uncertain significance	Illumina Clinical Se	10/18/2016	SLC26A4	chr7	107329546	107329546	http://gnomad.b	5.86E-05	NFE	8.97E-06	0		0	0	https://www.ncbi.nlm.nih.gov/clinvar/variation/179617/
NM_000051.3(ATM):c.8805G>A	concordant	unreportable	unreportable	407626	0	2	SCV000570412;S(	Uncertain significance;Unc	GeneDx;Invitae	2017-11-28;2017-1	ATM	chr11	108225556	108225556	http://gnomad.b	2.78E-05	EAS	0.0004638220125	0		0	0	https://www.ncbi.nlm.nih.gov/clinvar/variation/407626/
NM_000152.3(GAA):c.1849G>A	concordant	unreportable	unreportable	499878	0	1	SCV000705598	Uncertain significance	EGL Genetic Diagn	6/30/2017	GAA	chr17	78086471	78086471	http://gnomad.b	8.34E-06	AFR	0.0001396449952	0		0	0	https://www.ncbi.nlm.nih.gov/clinvar/variation/499878/
NM_018941.3(CLN8):c.88G>T	concordant	unreportable	unreportable	423135	0	1	SCV000572789	Uncertain significance	GeneDx	11/28/2017	CLN8	chr8	1719308	1719308	http://gnomad.b	1.96E-05	SAS	0.0001949190046	0		0	0	https://www.ncbi.nlm.nih.gov/clinvar/variation/423135/
NM_000051.3(ATM):c.6257A>T	concordant	unreportable	unreportable	181977	0	3	SCV000682319;S(	Uncertain significance;Unc	Color Genomics, In	2017-10-26;2017-1	ATM	chr11	108188158	108188158	http://gnomad.b	1.39E-05	NFE	1.79E-05	0		0	0	https://www.ncbi.nlm.nih.gov/clinvar/variation/181977/
NM_000136.2(FANCC):c.329T>C	concordant	unreportable	unreportable	456161	0	1	SCV000626246	Uncertain significance	Invitae	10/5/2017	FANCC	chr9	98002947	98002947	http://gnomad.b	1.66E-05	AMR	5.96E-05	0		0	0	https://www.ncbi.nlm.nih.gov/clinvar/variation/456161/
NM_000051.3(ATM):c.3665T>C	concordant	unreportable	unreportable	216203	0	1	SCV000254089	Uncertain significance	Invitae	9/11/2015	ATM	chr11	108153525	108153525		2.78E-06					0	0	https://www.ncbi.nlm.nih.gov/clinvar/variation/216203/
NM_000152.3(GAA):c.2166C>T	concordant	unreportable	unreportable	382801	0	1	SCV000522919	Likely benign	GeneDx	11/28/2017	GAA	chr17	78087142	78087142	http://gnomad.b	2.22E-05	EAS	0.0004014990118	0		0	0	https://www.ncbi.nlm.nih.gov/clinvar/variation/382801/
NM_000227.3(LAMA3):c.2509C>T	concordant	unreportable	unreportable	326315	0	1	SCV000407956	Uncertain significance	Illumina Clinical Se	10/18/2016	LAMA3	chr18	21494380	21494380	http://gnomad.b	3.08E-05	AMR	2.98E-05	0		0	0	https://www.ncbi.nlm.nih.gov/clinvar/variation/326315/
NM_138694.3(PKHD1):c.3119G>A	concordant	unreportable	unreportable	287204	0	1	SCV000340907	Uncertain significance	EGL Genetic Diagn	6/30/2017	PKHD1	chr6	51900498	51900498	http://gnomad.b	2.22E-05	AMR	0.00026832	0		0	0	https://www.ncbi.nlm.nih.gov/clinvar/variation/287204/
NM_000153.3(GALC):c.155delG	concordant	reportable	reportable	418220	1	0	SCV000565021	Likely pathogenic	GeneDx	11/28/2017	GALC	chr14	88459353	88459354		1.96E-05					0	0	https://www.ncbi.nlm.nih.gov/clinvar/variation/418220/
NM_000051.3(ATM):c.6411C>G	concordant	unreportable	unreportable	233658	0	3	SCV000682333;S(	Uncertain significance;Unc	Color Genomics, In	2017-10-26;2017-1	ATM	chr11	108190744	108190744	http://gnomad.b	5.57E-06	NFE	9.09E-06	0		0	0	https://www.ncbi.nlm.nih.gov/clinvar/variation/233658/
NM_000051.3(ATM):c.4666T>C	concordant	unreportable	unreportable	140851	0	3	SCV000569103;S(	Uncertain significance;Unc	GeneDx;Invitae;Lal	2017-11-28;2017-1	ATM	chr11	108164094	108164094	http://gnomad.b	1.11E-05	NFE	2.70E-05	0		0	0	https://www.ncbi.nlm.nih.gov/clinvar/variation/140851/
NM_000051.3(ATM):c.1641C>A	concordant	unreportable	unreportable	219526	0	2	SCV000681990;S(	Likely benign;Likely benign	Color Genomics, In	2017-10-26;2017-1	ATM	chr11	108122597	108122597	http://gnomad.b	2.23E-05	AMR	2.98E-05	0		0	0	https://www.ncbi.nlm.nih.gov/clinvar/variation/219526/
NM_006261.4(PROP1):c.193C>T	concordant	unreportable	unreportable	286955	0	1	SCV000340561	Uncertain significance	EGL Genetic Diagn	6/30/2017	PROP1	chr5	177421256	177421256	http://gnomad.b	2.25E-05	AFR	6.57E-05	0		0	0	https://www.ncbi.nlm.nih.gov/clinvar/variation/286955/
NM_000391.3(TPP1):c.1543C>T	concordant	unreportable	unreportable	207595	0	1	SCV000243442	Uncertain significance	GeneDx	11/28/2017	TPP1	chr11	6636105	6636105	http://gnomad.b	0.0001063228521	SAS	0.0007471899735	0		0	0	https://www.ncbi.nlm.nih.gov/clinvar/variation/207595/
NM_017890.4(VPS13B):c.2852G>T	concordant	unreportable	unreportable	195520	0	1	SCV000227703	Uncertain significance	EGL Genetic Diagn	6/30/2017	VPS13B	chr8	100396463	100396463	http://gnomad.b	5.60E-06	AMR	2.98E-05	0		0	0	https://www.ncbi.nlm.nih.gov/clinvar/variation/195520/
NM_000152.3(GAA):c.395G>C	concordant	unreportable	unreportable	325777	0	2	SCV000407258;S(	Uncertain significance;Unc	Illumina Clinical Se	2016-10-18;2017-1	GAA	chr17	78078780	78078780	http://gnomad.b	2.50E-05	AMR	5.96E-05	0		0	0	https://www.ncbi.nlm.nih.gov/clinvar/variation/325777/
NM_000051.3(ATM):c.2054A>G	concordant	unreportable	unreportable	453401	0	1	SCV000622304	Uncertain significance	Invitae	10/5/2017	ATM	chr11	108124696	108124696		2.78E-06					0	0	https://www.ncbi.nlm.nih.gov/clinvar/variation/453401/
NM_000051.3(ATM):c.1368A>G	concordant	unreportable	unreportable	233615	0	3	SCV000681972;S(	Likely benign;Likely benign,	Color Genomics, In	2017-10-26;2017-1	ATM	chr11	108121560	108121560	http://gnomad.b	2.78E-05	EAS	0.0004058440099	0		0	0	https://www.ncbi.nlm.nih.gov/clinvar/variation/233615/
NM_000051.3(ATM):c.6554T>C	concordant	unreportable	unreportable	246421	0	1	SCV000293970	Uncertain significance	GeneDx	11/28/2017	ATM	chr11	108192129	108192129	http://gnomad.b	1.39E-05	AFR	0.0001306850027	0		0	0	https://www.ncbi.nlm.nih.gov/clinvar/variation/246421/
NM_000153.3(GALC):c.2001A>C	concordant	unreportable	unreportable	314744	0	1	SCV000389233	Uncertain significance	Illumina Clinical Se	10/18/2016	GALC	chr14	88401133	88401133	http://gnomad.b	5.59E-06	NFE	1.80E-05	0		0	0	https://www.ncbi.nlm.nih.gov/clinvar/variation/314744/
NM_000232.4(SGCB):c.67C>T	concordant	unreportable	unreportable	289128	0	1	SCV000343422	Uncertain significance	EGL Genetic Diagn	6/30/2017	SGCB	chr4	52899773	52899773	http://gnomad.b	2.52E-05	AMR	5.96E-05	0		0	0	https://www.ncbi.nlm.nih.gov/clinvar/variation/289128/
NM_001042432.1(CLN3):c.207G>A	concordant	unreportable	unreportable	386233	0	1	SCV000527797	Likely benign	GeneDx	11/28/2017	CLN3	chr16	28500626	28500626	http://gnomad.b	3.61E-05	SAS	0.0001973290055	0		0	0	https://www.ncbi.nlm.nih.gov/clinvar/variation/386233/
NM_001042432.1(CLN3):c.266G>A	concordant	unreportable	unreportable	205106	0	1	SCV000240600	Uncertain significance	GeneDx	11/28/2017	CLN3	chr16	28499940	28499940	http://gnomad.b	2.78E-06	AMR	8.93E-05	0		0	0	https://www.ncbi.nlm.nih.gov/clinvar/variation/205106/
NM_024649.4(BBS1):c.1139G>A	concordant	unreportable	unreportable	216740	0	2	SCV000536422;S(	Uncertain significance;Unc	GeneDx;Invitae	2017-11-28;2015-0	BBS1	chr11	66293622	66293622	http://gnomad.b	2.51E-05	EAS	0.0002319110063	0		0	0	https://www.ncbi.nlm.nih.gov/clinvar/variation/216740/
NM_017890.4(VPS13B):c.10086C>G	concordant	unreportable	unreportable	361087	0	1	SCV000470847	Uncertain significance	Illumina Clinical Se	10/18/2016	VPS13B	chr8	100861072	100861072	http://gnomad.b	8.12E-05	SAS	0.001007079962	1		0	0	https://www.ncbi.nlm.nih.gov/clinvar/variation/361087/
NM_000051.3(ATM):c.562G>T	concordant	unreportable	unreportable	453592	0	1	SCV000622607	Uncertain significance	Invitae	10/5/2017	ATM	chr11	108114745	108114745		2.78E-06					0	0	https://www.ncbi.nlm.nih.gov/clinvar/variation/453592/
NM_000112.3(SLC26A2):c.2021A>G	concordant	unreportable	unreportable	352030	0	1	SCV000454833	Uncertain significance	Illumina Clinical Se	10/18/2016	SLC26A2	chr5	149361177	149361177	http://gnomad.b	8.36E-06	NFE	8.97E-06	0		0	0	https://www.ncbi.nlm.nih.gov/clinvar/variation/352030/
NM_000051.3(ATM):c.3772C>A	concordant	unreportable	unreportable	142817	0	2	SCV000687501;S(	Uncertain significance;Unc	Color Genomics, In	2017-12-21;2017-1	ATM	chr11	108154979	108154979		1.39E-05					0	0	https://www.ncbi.nlm.nih.gov/clinvar/variation/142817/
NM_000391.3(TPP1):c.1116C>G	concordant	unreportable	unreportable	419889	0	1	SCV000568067	Uncertain significance	GeneDx	11/28/2017	TPP1	chr11	6637265	6637265	http://gnomad.b	3.92E-05	EAS	0.0003478660074	0		0	0	https://www.ncbi.nlm.nih.gov/clinvar/variation/419889/
NM_138694.3(PKHD1):c.4973C>T	concordant	unreportable	unreportable	458600	0	1	SCV000629921	Uncertain significance	Invitae	10/5/2017	PKHD1	chr6	51889635	51889635		5.56E-06					0	0	https://www.ncbi.nlm.nih.gov/clinvar/variation/458600/
NM_002485.4(NBN):c.1591A>G	concordant	unreportable	unreportable	142242	0	2	SCV000685729;S(	Uncertain significance;Unc	Color Genomics, In	2017-10-26;2017-1	NBN	chr8	90965726	90965726	http://gnomad.b	5.58E-06	SAS	6.50E-05	0		0	0	https://www.ncbi.nlm.nih.gov/clinvar/variation/142242/
NM_000053.3(ATP7B):c.352G>A	concordant	unreportable	unreportable	439427	0	1	SCV000602603	Uncertain significance	ARUP Laboratories	6/30/2017	ATP7B	chr13	52549004	52549004	http://gnomad.b	0.000115041864	AMR	0.002203559969	0		0	0	https://www.ncbi.nlm.nih.gov/clinvar/variation/439427/

Supplemental Table 2

NM_033056.3(PCDH15):c.3782A>C	concordant	unreportable	unreportable	300179	0		1	SCV000363156	Uncertain significance	Illumina Clinical Se	10/18/2016	PCDH15	chr10	55616959	55616959	http://gnomad.b		2.06E-05	SAS	3.25E-05	0		0	0	https://www.ncbi.nlm.nih.gov/clinvar/variation/300179/
NM_033056.3(PCDH15):c.4294G>C	concordant	unreportable	unreportable	286960	0		1	SCV000340574	Uncertain significance	EGL Genetic Diagn	11/3/2016	PCDH15	chr10	55587226	55587226			2.57E-06					0	0	https://www.ncbi.nlm.nih.gov/clinvar/variation/286960/
NM_000051.3(ATM):c.6678A>G	concordant	unreportable	unreportable	185367	0		2	SCV000526459;S(	Likely benign;Likely benign	GeneDx;Invitae	2017-11-28;2017-1	ATM	chr11	108196142	108196142	http://gnomad.b		2.78E-06	EAS	0.0002320450003	0		0	0	https://www.ncbi.nlm.nih.gov/clinvar/variation/185367/
NM_000136.2(FANCC):c.1248C>T	concordant	unreportable	unreportable	380272	0		1	SCV000518137	Likely benign	GeneDx	11/28/2017	FANCC	chr9	97873826	97873826	http://gnomad.b		1.43E-05	EAS	0.0001739330037	0		0	0	https://www.ncbi.nlm.nih.gov/clinvar/variation/380272/
NM_000155.3(GALT):c.377+7A>G	concordant	unreportable	unreportable	495677	0		1	SCV000695690	Uncertain significance	Laboratory Corpora	1/25/2018	GALT	chr9	34647709	34647709	http://gnomad.b		8.25E-06	NFE	8.96E-06	0		0	0	https://www.ncbi.nlm.nih.gov/clinvar/variation/495677/
NM_000642.2(AGL):c.4413G>A	concordant	unreportable	unreportable	456506	0		1	SCV000626756	Likely benign	Invitae	10/5/2017	AGL	chr1	100382219	100382219	http://gnomad.b		1.95E-05	SAS	3.25E-05	0		0	0	https://www.ncbi.nlm.nih.gov/clinvar/variation/456506/
NM_000071.2(CBS):c.65A>G	concordant	unreportable	unreportable	212870	0		1	SCV000249717	Uncertain significance	GeneDx	11/28/2017	CBS	chr21	44492239	44492239	http://gnomad.b		4.50E-05	ASJ	0.0001025430029	0		0	0	https://www.ncbi.nlm.nih.gov/clinvar/variation/212870/
NM_000051.3(ATM):c.5002C>T	concordant	unreportable	unreportable	220386	0		3	SCV000682235;S(	Likely benign;Likely benign	Color Genomics, In	2017-10-26;2017-1	ATM	chr11	108168106	108168106	http://gnomad.b		4.18E-05	ASJ	0.0004068349954	0		0	0	https://www.ncbi.nlm.nih.gov/clinvar/variation/220386/
NM_000243.2(MEFV):c.2271C>T	concordant	unreportable	unreportable	495753	0		1	SCV000696063	Uncertain significance	Laboratory Corpora	1/25/2018	MEFV	chr16	3293216	3293216	http://gnomad.b		1.68E-05	NFE	8.95E-06	0		0	0	https://www.ncbi.nlm.nih.gov/clinvar/variation/495753/
NM_000152.3(GAA):c.297C>T	concordant	unreportable	unreportable	498124	0		1	SCV000702978	Uncertain significance	EGL Genetic Diagn	6/30/2017	GAA	chr17	78078682	78078682	http://gnomad.b		2.22E-05	AMR	0.0006261550006	0		0	0	https://www.ncbi.nlm.nih.gov/clinvar/variation/498124/
NM_017890.4(VPS13B):c.11430C>T	concordant	unreportable	unreportable	437249	0		1	SCV000597907	Likely benign	Genetic Services La	7/5/2017	VPS13B	chr8	100880656	100880656	http://gnomad.b		1.12E-05			0		0	0	https://www.ncbi.nlm.nih.gov/clinvar/variation/437249/
NM_000152.3(GAA):c.444C>T	concordant	unreportable	unreportable	285476	0		1	SCV000338503	Uncertain significance	EGL Genetic Diagn	6/30/2017	GAA	chr17	78078829	78078829			2.78E-06					0	0	https://www.ncbi.nlm.nih.gov/clinvar/variation/285476/
NM_006493.2(CLN5):c.1016G>T	concordant	unreportable	unreportable	422251	0		1	SCV000571671	Uncertain significance	GeneDx	11/28/2017	CLN5	chr13	77574896	77574896	http://gnomad.b		2.80E-05	NFE	5.38E-05	0		0	0	https://www.ncbi.nlm.nih.gov/clinvar/variation/422251/
NM_000030.2(AGXT):c.930C>T	concordant	unreportable	unreportable	335299	0		1	SCV000429372	Uncertain significance	Illumina Clinical Se	10/18/2016	AGXT	chr2	241817037	241817037	http://gnomad.b		5.67E-05	AMR	0.0001254709932	0		0	0	https://www.ncbi.nlm.nih.gov/clinvar/variation/335299/
NM_000383.3(AIRE):c.925A>G	concordant	unreportable	unreportable	458620	0		1	SCV000629959	Uncertain significance	Invitae	10/5/2017	AIRE	chr21	45711023	45711023	http://gnomad.b		2.79E-06	NFE	9.04E-06	0		0	0	https://www.ncbi.nlm.nih.gov/clinvar/variation/458620/
NM_017890.4(VPS13B):c.3706G>A	concordant	unreportable	unreportable	448877	0		1	SCV000616271	Uncertain significance	Athena Diagnostics	8/17/2017	VPS13B	chr8	100493866	100493866	http://gnomad.b		1.68E-05	NFE	8.97E-06	0		0	0	https://www.ncbi.nlm.nih.gov/clinvar/variation/448877/
NM_138694.3(PKHD1):c.7641A>G	concordant	unreportable	unreportable	288474	0		1	SCV000342587	Uncertain significance	EGL Genetic Diagn	6/30/2017	PKHD1	chr6	51732753	51732753	http://gnomad.b		1.67E-05	EAS	0.0005225270288	0		0	0	https://www.ncbi.nlm.nih.gov/clinvar/variation/288474/
NM_000151.3(G6PC):c.638C>T	concordant	unreportable	unreportable	323362	0		1	SCV000402982	Uncertain significance	Illumina Clinical Se	10/18/2016	G6PC	chr17	41063007	41063007	http://gnomad.b		1.53E-05	NFE	8.97E-06	0		0	0	https://www.ncbi.nlm.nih.gov/clinvar/variation/323362/
NM_138694.3(PKHD1):c.11210A>G	concordant	unreportable	unreportable	498746	0		1	SCV000703908	Uncertain significance	EGL Genetic Diagn	6/30/2017	PKHD1	chr6	51513983	51513983	http://gnomad.b		2.78E-06	AMR	8.94E-05	0		0	0	https://www.ncbi.nlm.nih.gov/clinvar/variation/498746/
NM_001042432.1(CLN3):c.327C>T	concordant	unreportable	unreportable	318725	0		1	SCV000396334	Uncertain significance	Illumina Clinical Se	10/18/2016	CLN3	chr16	28499030	28499030	http://gnomad.b		1.67E-05	AFR	6.57E-05	0		0	0	https://www.ncbi.nlm.nih.gov/clinvar/variation/318725/
NM_024685.3(BBS10):c.55G>C	concordant	unreportable	unreportable	462956	0		1	SCV000636521	Uncertain significance	Invitae	10/5/2017	BBS10	chr12	76742084	76742084			2.80E-06					0	0	https://www.ncbi.nlm.nih.gov/clinvar/variation/462956/
NM_000137.2(FAH):c.300C>T	concordant	unreportable	unreportable	500770	0		1	SCV000706847	Uncertain significance	EGL Genetic Diagn	6/30/2017	FAH	chr15	80452205	80452205	http://gnomad.b		3.33E-05	EAS	0.000115969	0		0	0	https://www.ncbi.nlm.nih.gov/clinvar/variation/500770/
NM_138694.3(PKHD1):c.3536A>G	concordant	unreportable	unreportable	406890	0		1	SCV000545849	Uncertain significance	Invitae	3/14/2017	PKHD1	chr6	51892978	51892978	http://gnomad.b		5.56E-06	SAS	3.25E-05	0		0	0	https://www.ncbi.nlm.nih.gov/clinvar/variation/406890/
NM_002485.4(NBN):c.772G>C	concordant	unreportable	unreportable	411788	0		2	SCV000690740;S(	Uncertain significance;Unc	Color Genomics, In	2017-12-21;2017-1	NBN	chr8	90982716	90982716			1.12E-05					0	0	https://www.ncbi.nlm.nih.gov/clinvar/variation/411788/
NM_000152.3(GAA):c.31C>T	concordant	unreportable	unreportable	456419	0		1	SCV000626603	Uncertain significance	Invitae	10/5/2017	GAA	chr17	78078416	78078416	http://gnomad.b		2.50E-05	SAS	0.0002604679903	0		0	0	https://www.ncbi.nlm.nih.gov/clinvar/variation/456419/
NM_000303.2(PMM2):c.593A>C	concordant	unreportable	unreportable	321219	0		1	SCV000399678	Uncertain significance	Illumina Clinical Se	10/18/2016	PMM2	chr16	8906917	8906917	http://gnomad.b		1.11E-05	NFE	1.79E-05	0		0	0	https://www.ncbi.nlm.nih.gov/clinvar/variation/321219/
NM_000642.2(AGL):c.4262A>G	concordant	unreportable	unreportable	456502	0		1	SCV000626750	Uncertain significance	Invitae	10/5/2017	AGL	chr1	100381968	100381968	http://gnomad.b		3.35E-05	AFR	0.0003279120137	0		0	0	https://www.ncbi.nlm.nih.gov/clinvar/variation/456502/
NM_183050.2(BCKDHB):c.1031C>T	concordant	unreportable	unreportable	358172	0		1	SCV000465670	Uncertain significance	Illumina Clinical Se	10/18/2016	BCKDHB	chr6	80982931	80982931	http://gnomad.b		3.08E-05	FIN	4.49E-05	0		0	0	https://www.ncbi.nlm.nih.gov/clinvar/variation/358172/
NM_000228.2(LAMB3):c.2157C>T	concordant	unreportable	unreportable	295094	0		1	SCV000353617	Uncertain significance	Illumina Clinical Se	10/18/2016	LAMB3	chr1	209797051	209797051	http://gnomad.b		1.68E-05	NFE	1.81E-05	0		0	0	https://www.ncbi.nlm.nih.gov/clinvar/variation/295094/
NM_033056.3(PCDH15):c.3699C>T	concordant	unreportable	unreportable	497542	0		1	SCV000702123	Uncertain significance	EGL Genetic Diagn	6/30/2017	PCDH15	chr10	55626420	55626420	http://gnomad.b		3.86E-05	NFE	2.69E-05	0		0	0	https://www.ncbi.nlm.nih.gov/clinvar/variation/497542/
NM_000271.4(NPC1):c.2131A>G	concordant	unreportable	unreportable	326265	0		1	SCV000407867	Uncertain significance	Illumina Clinical Se	10/18/2016	NPC1	chr18	21123533	21123533			3.72E-05					0	0	https://www.ncbi.nlm.nih.gov/clinvar/variation/326265/
NM_002485.4(NBN):c.1816G>A	concordant	unreportable	unreportable	186796	0		1	SCV000697956	Uncertain significance	Laboratory Corpora	1/25/2018	NBN	chr8	90965501	90965501	http://gnomad.b		8.37E-06	AMR	2.98E-05	0		0	0	https://www.ncbi.nlm.nih.gov/clinvar/variation/186796/
NM_000391.3(TPP1):c.206C>G	concordant	unreportable	unreportable	424229	0		1	SCV000574018	Uncertain significance	GeneDx	11/28/2017	TPP1	chr11	6640030	6640030			2.80E-06					0	0	https://www.ncbi.nlm.nih.gov/clinvar/variation/424229/
NM_000136.2(FANCC):c.246A>G	concordant	unreportable	unreportable	456158	0		1	SCV000626243	Uncertain significance	Invitae	10/5/2017	FANCC	chr9	98009718	98009718			4.76E-06					0	0	https://www.ncbi.nlm.nih.gov/clinvar/variation/456158/
NM_004937.2(CTNS):c.355C>T	concordant	unreportable	unreportable	290319	0		1	SCV000344851	Uncertain significance	EGL Genetic Diagn	6/30/2017	CTNS	chr17	3558540	3558540	http://gnomad.b		1.48E-05	NFE	8.95E-06	0		0	0	https://www.ncbi.nlm.nih.gov/clinvar/variation/290319/
NM_000051.3(ATM):c.6585T>C	concordant	unreportable	unreportable	187010	0		1	SCV000527918	Likely benign	GeneDx	11/28/2017	ATM	chr11	108196049	108196049			2.78E-06					0	0	https://www.ncbi.nlm.nih.gov/clinvar/variation/187010/
NM_002225.3(IVD):c.242G>C	concordant	unreportable	unreportable	459927	0		1	SCV000631884	Uncertain significance	Invitae	10/5/2017	IVD	chr15	40699925	40699925	http://gnomad.b		1.12E-05	NFE	1.79E-05	0		0	0	https://www.ncbi.nlm.nih.gov/clinvar/variation/459927/
NM_004937.2(CTNS):c.363C>T	concordant	unreportable	unreportable	322838	0		1	SCV000402220	Uncertain significance	Illumina Clinical Se	10/18/2016	CTNS	chr17	3558548	3558548	http://gnomad.b		1.19E-05	SAS	3.25E-05	0		0	0	https://www.ncbi.nlm.nih.gov/clinvar/variation/322838/
NM_002225.3(IVD):c.390delT	concordant	reportable	reportable	459924	1		0	SCV000631877	Pathogenic	Invitae	10/5/2017	IVD	chr15	40702920	40702921			8.41E-06					0	0	https://www.ncbi.nlm.nih.gov/clinvar/variation/459924/
NM_002485.4(NBN):c.340G>T	concordant	unreportable	unreportable	219644	0		4	SCV000685774;S(	Uncertain significance;Unc	Color Genomics, In	2017-10-26;2017-1	NBN	chr8	90993102	90993102			2.23E-05					0	0	https://www.ncbi.nlm.nih.gov/clinvar/variation/219644/
NM_000051.3(ATM):c.5267C>G	concordant	unreportable	unreportable	186974	0		1	SCV000546735	Uncertain significance	Invitae	3/14/2017	ATM	chr11	108172464	108172464			2.78E-06					0	0	https://www.ncbi.nlm.nih.gov/clinvar/variation/186974/
NM_000370.3(TTPA):c.752C>T	concordant	unreportable	unreportable	448840	0		1	SCV000616206	Uncertain significance	Athena Diagnostics	8/17/2017	TTPA	chr8	63973896	63973896	http://gnomad.b		3.60E-05	AMR	0.00032757601	0		0	0	https://www.ncbi.nlm.nih.gov/clinvar/variation/448840/
NM_002225.3(IVD):c.1169A>G	concordant	unreportable	unreportable	459926	0		1	SCV000631882	Uncertain significance	Invitae	10/5/2017	IVD	chr15	40710350	40710350	http://gnomad.b		2.80E-06	NFE	1.79E-05	0		0	0	https://www.ncbi.nlm.nih.gov/clinvar/variation/459926/
NM_003060.3(SLC22A5):c.1621A>T	concordant	unreportable	unreportable	286533	0		1	SCV000340008	Uncertain significance	EGL Genetic Diagn	6/30/2017	SLC22A5	chr5	131729911	131729911	http://gnomad.b		1.13E-05	AMR	5.96E-05	0		0	0	https://www.ncbi.nlm.nih.gov/clinvar/variation/286533/
NM_017890.4(VPS13B):c.584C>G	concordant	unreportable	unreportable	361038	0		1	SCV000470761	Uncertain significance	Illumina Clinical Se	10/18/2016	VPS13B	chr8	100123329	100123329	http://gnomad.b		3.92E-05	NFE	9.12E-06	0		1	0	https://www.ncbi.nlm.nih.gov/clinvar/variation/361038/
NM_017739.3(POMGNT1):c.6C>T	concordant	unreportable	unreportable	499877	0		1	SCV000705597	Uncertain significance	EGL Genetic Diagn	6/30/2017	POMGNT1	chr1	46663488	46663488			5.60E-06					0	0	https://www.ncbi.nlm.nih.gov/clinvar/variation/499877/
NM_000051.3(ATM):c.7620C>A	concordant	unreportable	unreportable	215594	0		1	SCV000252982	Likely benign	Invitae	10/5/2017	ATM	chr11	108202275	108202275	http://gnomad.b		5.57E-06	NFE	1.79E-05	0		0	0	https://www.ncbi.nlm.nih.gov/clinvar/variation/215594/
NM_000017.2(ACADS):c.1054G>A	concordant	unreportable	unreportable	432218	0		1	SCV000589916	Uncertain significance	GeneDx	11/28/2017	ACADS	chr12	121176967	121176967	http://gnomad.b		1.98E-05	NFE	3.68E-05	0		0	0	https://www.ncbi.nlm.nih.gov/clinvar/variation/432218/
NM_017890.4(VPS13B):c.6751C>T	concordant	unreportable	unreportable	361071	0		1	SCV000470820	Uncertain significance	Illumina Clinical Se	10/18/2016	VPS13B	chr8	100732591	100732591	http://gnomad.b		1.12E-05	NFE	1.79E-05	0		0	0	https://www.ncbi.nlm.nih.gov/clinvar/variation/361071/
NM_000051.3(ATM):c.1556T>C	concordant	unreportable	unreportable	407704	0		1	SCV000547105	Uncertain significance	Invitae	3/14/2017	ATM	chr11	108121748	108121748	http://gnomad.b		3.06E-05	SAS	0.000259908993	0		0	0	https://www.ncbi.nlm.nih.gov/clinvar/variation/407704/
NM_000051.3(ATM):c.7235A>C	concordant	unreportable	unreportable	407621	0		2	SCV000682398;S(	Uncertain significance;Unc	Color Genomics, In	2017-10-26;2017-1	ATM	chr11	108199893	108199893			5.57E-06					0	0	https://www.ncbi.nlm.nih.gov/clinvar/variation/407621/
NM_000136.2(FANCC):c.652G>C	concordant	unreportable	unreportable	456165	0		1	SCV000626255	Uncertain significance	Invitae	10/5/2017	FANCC	chr9	97912239	97912239	http://gnomad.b		9.51E-06	EAS	0.0002947770117	0		0	0	https://www.ncbi.nlm.nih.gov/clinvar/variation/456165/
NM_000051.3(ATM):c.4821A>G	concordant	unreportable	unreportable	187216	0		1	SCV000558371	Likely benign	Invitae	10/5/2017	ATM	chr11	108165698	108165698			1.67E-05					0	0	https://www.ncbi.nlm.nih.gov/clinvar/variation/187216/
NM_000051.3(ATM):c.6114C>T	concordant	unreportable	unreportable	187634	0		2	SCV000687675;S(	Likely benign;Likely benign	Color Genomics, In	2017-12-21;2017-1	ATM	chr11	108186756	108186756	http://gnomad.b		3.34E-05	SAS	6.50E-05	0		0	0	https://www.ncbi.nlm.nih.gov/clinvar/variation/187634/
NM_000136.2(FANCC):c.889A>T	concordant	unreportable	unreportable	182481	0		1	SCV000211047	Uncertain significance	GeneDx	11/28/2017	FANCC	chr9	97888818	97888818			1.66E-05					0	0	https://www.ncbi.nlm.nih.gov/clinvar/variation/182481/
NM_000232.4(SGCB):c.708C>T	concordant	unreportable	unreportable	497341	0		1	SCV000701798	Uncertain significance	EGL Genetic Diagn	6/30/2017	SGCB	chr4	52894179	52894179	http://gnomad.b		3.64E-05	AMR	0.00032757601	0		0	0	https://www.ncbi.nlm.nih.gov/clinvar/variation/497341/
NM_000051.3(ATM):c.8902T>C	concordant	unreportable	unreportable	389086	0		2	SCV000531517;S(	Likely benign;Likely benign	GeneDx;Invitae	2017-11-28;2017-0	ATM	chr11	108235860	108235860	http://gnomad.b		2.78E-06	NFE	1.79E-05	0		0	0	https://www.ncbi.nlm.nih.gov/clinvar/variation/389086/
NM_000051.3(ATM):c.5725A>G	concordant	unreportable	unreportable	232769	0		1	SCV000567877	Uncertain significance	GeneDx	11/28/2017	ATM	chr11	108178674	108178674	http://gnomad.b		1.11E-05	AMR	2.98E-05	0		0	0	https://www.ncbi.nlm.nih.gov/clinvar/variation/232769/
NM_138694.3(PKHD1):c.642G>A	concordant	unreportable	unreportable	290945	0		1	SCV000345615	Uncertain significance	EGL Genetic Diagn	6/30/2017	PKHD1	chr6	51935829	51935829	http://gnomad.b		2.78E-05	AFR	6.54E-05	0		0	0	https://www.ncbi.nlm.nih.gov/clinvar/variation/290945/
NM_000391.3(TPP1):c.78C>A	concordant	unreportable	unreportable	457947	0		1	SCV000628915	Uncertain significance	Invitae	10/5/2017	TPP1	chr11	6640438	6640438	http://gnomad.b		1.40E-05	AFR	0.0001307019993	0		0	0	https://www.ncbi.nlm.nih.gov/clinvar/variation/457947/
NM_000642.2(AGL):c.4207A>T	concordant	unreportable	unreportable	291346	0		1	SCV000346090	Uncertain significance	Illumina Clinical Se	10/18/2016	AGL	chr1	100380990	100380990	http://gnomad.b		5.30E-05	SAS	0.0002274650033	0		0	0	https://www.ncbi.nlm.nih.gov/clinvar/variation/291346/
NM_001042432.1(CLN3):c.1135C>T	concordant	unreportable	unreportable	377687	0		1	SCV000512636	Likely benign	GeneDx	11/28/2017	CLN3	chr16	28489120	28489120			1.39E-05					0	0	https://www.ncbi.nlm.nih.gov/clinvar/variation/377687/
NM_000051.3(ATM):c.9002G>A	concordant	unreportable	unreportable	231903	0		2	SCV000687869;S(	Uncertain significance;Unc	Color Genomics, In	2017-12-21;2017-1	ATM	chr11	108236066	108236066			1.67E-05					0	0	https://www.ncbi.nlm.nih.gov/clinvar/variation/231903/
NM_000152.3(GAA):c.2753C>T	concordant	unreportable	unreportable	325795	0		1	SCV000407305	Uncertain significance	Illumina Clinical Se	10/18/2016	GAA	chr17	78092558	78092558	http://gnomad.b		3.61E-05	SAS	0.000454811001	0		0	0	https://www.ncbi.nlm.nih.gov/clinvar/variation/325795/
NM_000051.3(ATM):c.3961A>G	concordant	unreportable	unreportable	181953	0		1	SCV000209732	Uncertain significance	GeneDx	11/28/2017	ATM	chr11	108155168	108155168	http://gnomad.b		8.35E-06	SAS	3.25E-05	0		0	0	https://www.ncbi.nlm.nih.gov/clinvar/variation/181953/
NM_014363.4(SACS):c.8971C>T	concordant	unreportable	unreportable	448226	0		1	SCV000614991	Uncertain significance	Athena Diagnostics	8/17/2017	SACS	chr13	23909044	23909044	http://gnomad.b		1.78E-05	NFE	3.61E-05	0		0	0	https://www.ncbi.nlm.nih.gov/clinvar/variation/448226/
NM_000051.3(ATM):c.652C>T	concordant	reportable	reportable	451807	1		0	SCV000620558	Likely pathogenic	GeneDx	11/28/2017	ATM	chr11	108114835	108114835			2.78E-06					0	0	https://www.ncbi.nlm.nih.gov/clinvar/variation/451807/
NM_017890.4(VPS13B):c.5452C>T	concordant	unreportable	unreportable	212576	0		1	SCV000249410	Uncertain significance	Genetic Services La	9/15/2015	VPS13B	chr8	100654195	100654195	http://gnomad.b		8.40E-06	AMR	0.0001192959971	0		0	0	https://www.ncbi.nlm.nih.gov/clinvar/variation/212576/
NM_000152.3(GAA):c.1188C>G	concordant	unreportable	unreportable	290186	0		1	SCV000344693	Uncertain significance	EGL Genetic Diagn	6/30/2017	GAA	chr17	78082400	78082400	http://gnomad.b		2.22E-05	NFE	8.14E-05	0		0	0	https://www.ncbi.nlm.nih.gov/clinvar/variation/290186/
NM_017739.3(POMGNT1):c.1110+9T>	concordant	unreportable	unreportable	471399	0		1	SCV000649951	Uncertain significance	Invitae	10/5/2017	POMGNT1	chr1	46658968	46658968			2.80E-06					0	0	https://www.ncbi.nlm.nih.gov/clinvar/variation/471399/
NM_000051.3(ATM):c.3108C>T	concordant	unreportable	unreportable	230144	0		2	SCV000682102;S(	Likely benign;Likely benign	Color Genomics, In	2017-10-26;2017-0	ATM	chr11	108143289	108143289			2.51E-05					0	0	https://www.ncbi.nlm.nih.gov/clinvar/variation/230144/
NM_002485.4(NBN):c.966C>T	concordant	unreportable	unreportable	184004	0		3	SCV000685840;S(	Likely benign;Likely benign	Color Genomics, In	2017-10-26;2017-1	NBN	chr8	90976666	90976666	http://gnomad.b		8.37E-06	NFE	3.59E-05	0		0	0	https://www.ncbi.nlm.nih.gov/clinvar/variation/184004/
NM_000051.3(ATM):c.2023C>T	concordant	reportable	reportable	231933	1		0	SCV000322534	Likely pathogenic	GeneDx	11/28/2017	ATM	chr11	108124665	108124665	http://gnomad.b		5.57E-06	NFE	2.69E-05	0		0	0	https://www.ncbi.nlm.nih.gov/clinvar/variation/231933/
NM_138694.3(PKHD1):c.11539G>T	concordant	unreportable	unreportable	499367	0		1	SCV000704818	Uncertain significance	EGL Genetic Diagn	6/30/2017	PKHD1	chr6	51497489	51497489	http://gnomad.b		5.56E-06	AFR	6.54E-05	0		0	0	https://www.ncbi.nlm.nih.gov/clinvar/variation/499367/
NM_138694.3(PKHD1):c.8997A>G	concordant	unreportable	unreportable	287478	0		1	SCV000341261	Uncertain significance	EGL Genetic Diagn	6/30/2017	PKHD1	chr6	51613417	51613417	http://gnomad.b		1.11E-05	NFE	1.85E-05	0		0	0	https://www.ncbi.nlm.nih.gov/clinvar/variation/287478/
NM_000271.4(NPC1):c.72C>T	concordant	unreportable	unreportable	471945	0		1	SCV000650852	Likely benign	Invitae	10/5/2017	NPC1	chr18	21153524	21153524	http://gnomad.b		1.14E-05	EAS	0.0002319110063	0		0	0	https://www.ncbi.nlm.nih.gov/clinvar/variation/471945/
NM_000051.3(ATM):c.1759G>A	concordant	unreportable	unreportable	246372	0		1	SCV000293896	Uncertain significance	GeneDx	11/28/2017	ATM	chr11	108122715	108122715			2.78E-06					0	0	https://www.ncbi.nlm.nih.gov/clinvar/variation/246372/
NM_000051.3(ATM):c.5640G>A	concordant	unreportable	unreportable	414529	0		1	SCV000558294	Likely benign	Invitae	3/14/2017	ATM	chr11	108175545	108175545	http://gnomad.b		2.78E-06	AMR	0.0001213740034	0		0	0	https://www.ncbi.nlm.nih.gov/clinvar/variation/414529/
NM_001164277.1(SLC37A4):c.700C>T	concordant	unreportable	unreportable	452731	0		1	SCV000621562	Uncertain significance	GeneDx	11/28/2017	SLC37A4	chr11	118897731	118897731	http://gnomad.b		1.96E-05	NFE	1.79E-05	0		0	0	https://www.ncbi.nlm.nih.gov/clinvar/variation/452731/
NM_004646.3(NPHS1):c.1861G>A	concordant	unreportable	unreportable	328866	0		1	SCV000411568	Uncertain significance	Illumina Clinical Se	10/18/2016	NPHS1	chr19	36336339	36336339	http://gnomad.b		8.36E-06	SAS	3.25E-05	0		0	0	https://www.ncbi.nlm.nih.gov/clinvar/variation/328866/
NM_000136.2(FANCC):c.1159T>C	concordant	unreportable	unreportable	419389	0		1	SCV000567154	Uncertain significance	GeneDx	11/28/2017	FANCC	chr9	97873915	97873915	http://gnomad.b		7.13E-06	NFE	8.96E-06	0		0	0	https://www.ncbi.nlm.nih.gov/clinvar/variation/419389/
NM_017739.3(POMGNT1):c.1645C>T	concordant	unreportable	unreportable	286892	0		1	SCV000340480	Uncertain significance	EGL Genetic Diagn	6/30/2017	POMGNT1	chr1	46656149	46656149	http://gnomad.b		2.24E-05	EAS	0.0001739330037	0		0	0	https://www.ncbi.nlm.nih.gov/clinvar/variation/286892/
NM_005562.2(LAMC2):c.1268C>A	concordant	unreportable	unreportable	294003	0		1	SCV000351673	Uncertain significance	Illumina Clinical Se	10/18/2016	LAMC2	chr1	183196034	183196034	http://gnomad.b		2.80E-06	NFE	8.97E-06	0		0	0	https://www.ncbi.nlm.nih.gov/clinvar/variation/294003/
NM_000051.3(ATM):c.1726A>G	concordant	unreportable	unreportable	421483	0		2	SCV000687322;S(	Uncertain significance;Unc	Color Genomics, In	2017-12-21;2017-1	ATM	chr11	108122682	108122682	http://gnomad.b		2.78E-05	ASJ	0.0003046919883	0		0	0	https://www.ncbi.nlm.nih.gov/clinvar/variation/421483/
NM_000051.3(ATM):c.1841G>C	concordant	unreportable	unreportable	407689	0		2	SCV000567546;S(	Uncertain significance;Unc	GeneDx;Invitae	2017-11-28;2017-1	ATM	chr11	108123582	108123582	http://gnomad.b		1.39E-05	NFE	1.80E-05	0		0	0	https://www.ncbi.nlm.nih.gov/clinvar/variation/407689/
NM_000071.2(CBS):c.1070C>G	concordant	unreportable	unreportable	212880	0		2	SCV000249730;S(	Uncertain significance;Unc	GeneDx;Invitae	2017-11-28;2017-1	CBS	chr21	44480626	44480626	http://gnomad.b		2.53E-05	AMR	2.99E-05	0		0	0	https://www.ncbi.nlm.nih.gov/clinvar/variation/212880/
NM_000051.3(ATM):c.5417T>C	concordant	unreportable	unreportable	186433	0		1	SCV000282991	Uncertain significance	Invitae	10/5/2017	ATM	chr11	108173677	108173677			1.11E-05					0	0	https://www.ncbi.nlm.nih.gov/clinvar/variation/186433/
NM_138694.3(PKHD1):c.8036T>C	concordant	unreportable	unreportable	496526	0		1	SCV000699879	Uncertain significance	Laboratory Corpora	1/25/2018	PKHD1	chr6	51712644	51712644	http://gnomad.b		4.17E-05	ASJ	0.0004065039975	0		0	0	https://www.ncbi.nlm.nih.gov/clinvar/variation/496526/
NM_004328.4(BCS1L):c.209A>G	concordant	unreportable	unreportable	283781	0		1	SCV000336094	Uncertain significance	EGL Genetic Diagn	6/30/2017	BCS1L	chr2	219525919	219525919	http://gnomad.b		1.67E-05	EAS	0.0002319110063	0		0	0	https://www.ncbi.nlm.nih.gov/clinvar/variation/283781/
NM_006493.2(CLN5):c.1032C>G	concordant	unreportable	unreportable	205132	0		1	SCV000240628	Uncertain significance	GeneDx	11/28/2017	CLN5	chr13	77574912	77574912	http://gnomad.b		5.59E-06	NFE	8.97E-06	0		0	0	https://www.ncbi.nlm.nih.gov/clinvar/variation/205132/
NM_000049.2(ASPA):c.927C>T	concordant	unreportable	unreportable	413758	0		1	SCV000556695	Likely benign	Invitae	3/14/2017	ASPA	chr17	3402367	3402367	http://gnomad.b		5.20E-05	AMR	0.0002682560007	0		0	0	https://www.ncbi.nlm.nih.gov/clinvar/variation/413758/
NM_000051.3(ATM):c.7314A>C	concordant	unreportable	unreportable	380111	0		2	SCV000517844;S(	Likely benign;Likely benign	GeneDx;Invitae	2017-11-28;2017-1	ATM	chr11	108200947	108200947	http://gnomad.b		3.06E-05	NFE	4.49E-05	0		0	0	https://www.ncbi.nlm.nih.gov/clinvar/variation/380111/
NM_000642.2(AGL):c.30A>T	concordant	unreportable	unreportable	456483	0		1	SCV000626717	Uncertain significance	Invitae	10/5/2017	AGL	chr1	100316628	100316628	http://gnomad.b		5.58E-06	AFR	0.0001306850027	0		0	0	https://www.ncbi.nlm.nih.gov/clinvar/variation/456483/
NM_033056.3(PCDH15):c.1427C>T	concordant	unreportable	unreportable	423939	0		1	SCV000573704	Uncertain significance	GeneDx	11/28/2017	PCDH15	chr10	55944907	55944907	http://gnomad.b		2.06E-05	AMR	0.0001489429997	0		0	0	https://www.ncbi.nlm.nih.gov/clinvar/variation/423939/
NM_000051.3(ATM):c.7235A>G	concordant	unreportable	unreportable	186904	0		3	SCV000682399;S(	Uncertain significance;Unc	Color Genomics, In	2017-10-26;2017-1	ATM	chr11	108199893	108199893	http://gnomad.b		1.95E-05	SAS	3.25E-05	0		0	0	https://www.ncbi.nlm.nih.gov/clinvar/variation/186904/
NM_000050.4(ASS1):c.711C>T	concordant	unreportable	unreportable	387469	0		1	SCV000529523	Likely benign	GeneDx	11/28/2017	ASS1	chr9	133355125	133355125	http://gnomad.b		3.90E-05	AMR	0.0002382370003	0		1	0	https://www.ncbi.nlm.nih.gov/clinvar/variation/387469/
NM_000487.5(ARSA):c.25C>T	concordant	unreportable	unreportable	342240	0		1	SCV000439444	Uncertain significance	Illumina Clinical Se	10/18/2016	ARSA	chr22	51066183	51066183	http://gnomad.b		1.41E-05	EAS	9.77E-05	0		0	0	https://www.ncbi.nlm.nih.gov/clinvar/variation/342240/

Supplemental Table 2

NM_000051.3(ATM):c.6025T>C	concordant	unreportable	unreportable	181973	0	3	SCV000682299;S	Uncertain significance;Unc	Color Genomics, In	2017-10-26;2017-1	ATM	chr11	108186568	108186568	http://gnomad.b	1.67E-05	NFE	1.79E-05	0		0	0	https://www.ncbi.nlm.nih.gov/clinvar/variation/181973/
NM_000051.3(ATM):c.1332C>A	concordant	unreportable	unreportable	184641	0	3	SCV000681968;S	Likely benign;Likely benign	Color Genomics, In	2017-10-26;2017-1	ATM	chr11	108121524	108121524	http://gnomad.b	3.34E-05	AMR	2.98E-05	0		0	0	https://www.ncbi.nlm.nih.gov/clinvar/variation/184641/
NM_000051.3(ATM):c.2264G>A	concordant	unreportable	unreportable	479068	0	1	SCV000687372	Uncertain significance	Color Genomics, In	12/21/2017	ATM	chr11	108128221	108128221		2.78E-06					0	0	https://www.ncbi.nlm.nih.gov/clinvar/variation/479068/
NM_000051.3(ATM):c.1340G>A	concordant	unreportable	unreportable	230430	0	2	SCV000687301;S	Uncertain significance;Unc	Color Genomics, In	2017-12-21;2017-1	ATM	chr11	108121532	108121532	http://gnomad.b	5.57E-06	AFR	0.0001307019993	0		0	0	https://www.ncbi.nlm.nih.gov/clinvar/variation/230430/
NM_000051.3(ATM):c.4058A>G	concordant	unreportable	unreportable	418032	0	3	SCV000687519;S	Uncertain significance;Unc	Color Genomics, In	2017-12-21;2017-1	ATM	chr11	108158391	108158391		5.57E-06					0	0	https://www.ncbi.nlm.nih.gov/clinvar/variation/418032/
NM_002485.4(NBN):c.902G>A	concordant	unreportable	unreportable	490063	0	1	SCV000685834	Uncertain significance	Color Genomics, In	10/26/2017	NBN	chr8	90976730	90976730	http://gnomad.b	5.58E-06	EAS	0.0001739739964	0		0	0	https://www.ncbi.nlm.nih.gov/clinvar/variation/490063/
NM_000152.3(GAA):c.906G>A	concordant	unreportable	unreportable	500089	0	1	SCV000705879	Uncertain significance	EGL Genetic Diagn	6/30/2017	GAA	chr17	78081646	78081646		5.56E-06					0	0	https://www.ncbi.nlm.nih.gov/clinvar/variation/500089/
NM_000051.3(ATM):c.2386A>T	concordant	unreportable	unreportable	219746	0	1	SCV000259791	Uncertain significance	Invitae	6/10/2016	ATM	chr11	108129722	108129722	http://gnomad.b	2.78E-06	NFE	8.96E-06	0		0	0	https://www.ncbi.nlm.nih.gov/clinvar/variation/219746/
NM_033056.3(PCDH15):c.466G>A	concordant	unreportable	unreportable	300205	0	1	SCV000363249	Uncertain significance	Illumina Clinical Se	10/18/2016	PCDH15	chr10	56128888	56128888	http://gnomad.b	3.86E-05	FIN	0.0007632219931	0		0	0	https://www.ncbi.nlm.nih.gov/clinvar/variation/300205/
NM_024649.4(BBS1):c.437G>A	concordant	unreportable	unreportable	412286	0	1	SCV000553780	Uncertain significance	Invitae	3/14/2017	BBS1	chr11	66283015	66283015	http://gnomad.b	4.18E-05	EAS	0.0002319110063	0		0	0	https://www.ncbi.nlm.nih.gov/clinvar/variation/412286/
NM_000051.3(ATM):c.6536T>C	concordant	unreportable	unreportable	236754	0	2	SCV000682346;S	Uncertain significance;Unc	Color Genomics, In	2017-10-26;2017-0	ATM	chr11	108192111	108192111	http://gnomad.b	1.11E-05	NFE	8.95E-06	0		0	0	https://www.ncbi.nlm.nih.gov/clinvar/variation/236754/
NM_000051.3(ATM):c.2908C>T	concordant	unreportable	unreportable	414526	0	1	SCV000558289	Likely benign	Invitae	3/14/2017	ATM	chr11	108141860	108141860		1.11E-05					0	0	https://www.ncbi.nlm.nih.gov/clinvar/variation/414526/
NM_000492.3(CFTR):c.316A>G	concordant	unreportable	unreportable	497781	0	1	SCV000702489	Uncertain significance	EGL Genetic Diagn	6/30/2017	CFTR	chr7	117170995	117170995		9.63E-06					0	0	https://www.ncbi.nlm.nih.gov/clinvar/variation/497781/
NM_000543.4(SMPD1):c.1037C>T	concordant	unreportable	unreportable	291288	0	1	SCV000346008	Uncertain significance	EGL Genetic Diagn	6/30/2017	SMPD1	chr11	6413332	6413332	http://gnomad.b	3.33E-05	AFR	0.0001307019993	0		0	0	https://www.ncbi.nlm.nih.gov/clinvar/variation/291288/
NM_014363.4(SACS):c.8414C>T	concordant	unreportable	unreportable	193710	0	1	SCV000614989	Uncertain significance	Athena Diagnostics	8/17/2017	SACS	chr13	23909601	23909601	http://gnomad.b	8.89E-06	NFE	1.81E-05	0		0	0	https://www.ncbi.nlm.nih.gov/clinvar/variation/193710/
NM_000137.2(FAH):c.963C>A	concordant	reportable	reportable	291075	1	0	SCV000345761	Pathogenic	EGL Genetic Diagn	6/30/2017	FAH	chr15	80472468	80472468		1.11E-05					0	0	https://www.ncbi.nlm.nih.gov/clinvar/variation/291075/
NM_000108.3(DLD):c.1382G>A	concordant	unreportable	unreportable	203685	0	1	SCV000238812	Uncertain significance	GeneDx	11/28/2017	DLD	chr7	107559462	107559462	http://gnomad.b	4.15E-05	SAS	0.0002274199942	0		0	0	https://www.ncbi.nlm.nih.gov/clinvar/variation/203685/
NM_000414.3(HSD17B4):c.1450A>G	concordant	unreportable	unreportable	227434	0	1	SCV000270275	Likely benign	Laboratory for Mol	4/26/2016	HSD17B4	chr5	118850688	118850688	http://gnomad.b	6.48E-05	NFE	8.97E-06	0		0	0	https://www.ncbi.nlm.nih.gov/clinvar/variation/227434/
NM_002485.4(NBN):c.1247T>C	concordant	unreportable	unreportable	216563	0	1	SCV000254762	Uncertain significance	Invitae	9/11/2015	NBN	chr8	90967661	90967661		5.58E-06					0	0	https://www.ncbi.nlm.nih.gov/clinvar/variation/216563/
NM_138694.3(PKHD1):c.193G>A	concordant	unreportable	unreportable	288717	0	1	SCV000342908	Uncertain significance	EGL Genetic Diagn	6/30/2017	PKHD1	chr6	51947278	51947278	http://gnomad.b	3.06E-05	SAS	0.0002924170112	0		0	0	https://www.ncbi.nlm.nih.gov/clinvar/variation/288717/
NM_000051.3(ATM):c.5626C>T	concordant	unreportable	unreportable	407503	0	2	SCV000687629;S	Uncertain significance;Unc	Color Genomics, In	2017-12-21;2017-1	ATM	chr11	108175531	108175531		8.35E-06					0	0	https://www.ncbi.nlm.nih.gov/clinvar/variation/407503/
NM_002485.4(NBN):c.224G>C	concordant	unreportable	unreportable	142014	0	3	SCV000685765;S	Uncertain significance;Unc	Color Genomics, In	2017-10-26;2017-1	NBN	chr8	90993699	90993699	http://gnomad.b	1.95E-05	NFE	1.79E-05	0		0	0	https://www.ncbi.nlm.nih.gov/clinvar/variation/142014/
NM_000152.3(GAA):c.-32-4G>A	concordant	unreportable	unreportable	382800	0	1	SCV000522918	Likely benign	GeneDx	11/28/2017	GAA	chr17	78078350	78078350	http://gnomad.b	3.06E-05	NFE	1.95E-05	0		1	0	https://www.ncbi.nlm.nih.gov/clinvar/variation/382800/
NM_000051.3(ATM):c.2012T>A	concordant	unreportable	unreportable	185852	0	1	SCV000254066	Uncertain significance	Invitae	10/5/2017	ATM	chr11	108124654	108124654	http://gnomad.b	8.35E-06	AMR	2.98E-05	0		0	0	https://www.ncbi.nlm.nih.gov/clinvar/variation/185852/
NM_000051.3(ATM):c.6482G>A	concordant	unreportable	unreportable	185236	0	2	SCV000293556;S	Uncertain significance;Unc	GeneDx;Invitae	2017-11-28;2017-0	ATM	chr11	108192057	108192057	http://gnomad.b	2.23E-05	AFR	6.54E-05	0		0	0	https://www.ncbi.nlm.nih.gov/clinvar/variation/185236/
NM_000018.3(ACADVL):c.1605+6_160	concordant	unreportable	unreportable	422276	0	1	SCV000571700	Likely benign	GeneDx	11/28/2017	ACADVL	chr17	7127717	7127719		0.0002180438353					1	0	https://www.ncbi.nlm.nih.gov/clinvar/variation/422276/
NM_000051.3(ATM):c.8959G>T	concordant	unreportable	unreportable	453755	0	1	SCV000622867	Uncertain significance	Invitae	10/5/2017	ATM	chr11	108235917	108235917	http://gnomad.b	5.57E-06	SAS	3.25E-05	0		0	0	https://www.ncbi.nlm.nih.gov/clinvar/variation/453755/
NM_017739.3(POMGNT1):c.652+8C>T	concordant	unreportable	unreportable	286911	0	1	SCV000340506	Uncertain significance	EGL Genetic Diagn	6/30/2017	POMGNT1	chr1	46660508	46660508	http://gnomad.b	5.60E-06	AFR	0.0001307530038	0		0	0	https://www.ncbi.nlm.nih.gov/clinvar/variation/286911/
NM_000151.3(G6PC):c.922G>A	concordant	unreportable	unreportable	323363	0	1	SCV000402983	Uncertain significance	Illumina Clinical Se	10/18/2016	G6PC	chr17	41063291	41063291	http://gnomad.b	2.30E-05	AFR	6.53E-05	0		0	0	https://www.ncbi.nlm.nih.gov/clinvar/variation/323363/
NM_001164277.1(SLC37A4):c.607C>A	concordant	unreportable	unreportable	459623	0	1	SCV000631384	Uncertain significance	Invitae	10/5/2017	SLC37A4	chr11	118898356	118898356	http://gnomad.b	6.70E-05	SAS	0.000910510018	0		1	0	https://www.ncbi.nlm.nih.gov/clinvar/variation/459623/
NM_000051.3(ATM):c.411C>T	concordant	unreportable	unreportable	183922	0	3	SCV000687521;S	Likely benign;Likely benign	Color Genomics, In	2017-12-21;2017-1	ATM	chr11	108106476	108106476	http://gnomad.b	2.23E-05	EAS	0.0002319110063	0		0	0	https://www.ncbi.nlm.nih.gov/clinvar/variation/183922/
NM_002485.4(NBN):c.1619A>G	concordant	unreportable	unreportable	182723	0	2	SCV000211453;S	Uncertain significance;Unc	GeneDx;Invitae	2017-11-28;2017-0	NBN	chr8	90965698	90965698	http://gnomad.b	1.12E-05	NFE	8.98E-06	0		0	0	https://www.ncbi.nlm.nih.gov/clinvar/variation/182723/
NM_000136.2(FANCC):c.521G>A	concordant	unreportable	unreportable	409653	0	1	SCV000549955	Uncertain significance	Invitae	3/14/2017	FANCC	chr9	97933361	97933361	http://gnomad.b	3.09E-05	AMR	5.96E-05	0		0	0	https://www.ncbi.nlm.nih.gov/clinvar/variation/409653/
NM_024685.3(BBS10):c.1510_1511de	concordant	reportable	reportable	288255	1	0	SCV000342310	Pathogenic	EGL Genetic Diagn	6/30/2017	BBS10	chr12	76740253	76740255		5.60E-06					0	0	https://www.ncbi.nlm.nih.gov/clinvar/variation/288255/
NM_000051.3(ATM):c.4534G>A	concordant	unreportable	unreportable	407648	0	1	SCV000546999	Uncertain significance	Invitae	3/14/2017	ATM	chr11	108163443	108163443		8.35E-06					0	0	https://www.ncbi.nlm.nih.gov/clinvar/variation/407648/
NM_000051.3(ATM):c.6400T>C	concordant	unreportable	unreportable	490652	0	1	SCV000687693	Uncertain significance	Color Genomics, In	12/21/2017	ATM	chr11	108190733	108190733	http://gnomad.b	1.95E-05	SAS	3.25E-05	0		0	0	https://www.ncbi.nlm.nih.gov/clinvar/variation/490652/
NM_138694.3(PKHD1):c.12201G>A	concordant	unreportable	unreportable	500795	0	1	SCV000706888	Uncertain significance	EGL Genetic Diagn	6/30/2017	PKHD1	chr6	51483903	51483903	http://gnomad.b	1.67E-05	SAS	3.25E-05	0		0	0	https://www.ncbi.nlm.nih.gov/clinvar/variation/500795/
NM_000051.3(ATM):c.8120C>G	concordant	unreportable	unreportable	220257	0	3	SCV000537544;S	Uncertain significance;Unc	Color Genomics, In	2017-12-21;2017-1	ATM	chr11	108205805	108205805	http://gnomad.b	2.78E-05	EAS	0.0002321799984	0		0	0	https://www.ncbi.nlm.nih.gov/clinvar/variation/220257/
NM_000391.3(TPP1):c.1466A>G	concordant	unreportable	unreportable	408909	0	1	SCV000548738	Uncertain significance	Invitae	3/14/2017	TPP1	chr11	6636182	6636182	http://gnomad.b	1.96E-05	FIN	0.000224235002	0		0	0	https://www.ncbi.nlm.nih.gov/clinvar/variation/408909/
NM_014363.4(SACS):c.11348A>G	concordant	unreportable	unreportable	448188	0	1	SCV000614928	Uncertain significance	Athena Diagnostics	8/17/2017	SACS	chr13	23906667	23906667		2.96E-06					0	0	https://www.ncbi.nlm.nih.gov/clinvar/variation/448188/
NM_000271.4(NPC1):c.2598G>T	concordant	unreportable	unreportable	498823	0	1	SCV000704034	Uncertain significance	EGL Genetic Diagn	6/30/2017	NPC1	chr18	21120418	21120418		8.58E-06					0	0	https://www.ncbi.nlm.nih.gov/clinvar/variation/498823/
NM_000051.3(ATM):c.4332G>A	concordant	unreportable	unreportable	184635	0	3	SCV000682196;S	Likely benign;Likely benign	Color Genomics, In	2017-10-26;2017-1	ATM	chr11	108160424	108160424	http://gnomad.b	2.78E-06	AFR	6.55E-05	0		0	0	https://www.ncbi.nlm.nih.gov/clinvar/variation/184635/
NM_000051.3(ATM):c.200A>G	concordant	unreportable	unreportable	231354	0	2	SCV000593493;S	Uncertain significance;Unc	Genetic Services La	2017-07-05;2017-1	ATM	chr11	108099919	108099919	http://gnomad.b	1.39E-05	AMR	2.99E-05	0		0	0	https://www.ncbi.nlm.nih.gov/clinvar/variation/231354/
NM_006493.2(CLN5):c.101C>G	concordant	unreportable	unreportable	409277	0	1	SCV000549224	Uncertain significance	Invitae	3/14/2017	CLN5	chr13	77566187	77566187	http://gnomad.b	1.68E-05	AFR	7.69E-05	0		0	0	https://www.ncbi.nlm.nih.gov/clinvar/variation/409277/
NM_000051.3(ATM):c.5650A>G	concordant	unreportable	unreportable	453594	0	1	SCV000622612	Uncertain significance	Invitae	10/5/2017	ATM	chr11	108175555	108175555		2.78E-06					0	0	https://www.ncbi.nlm.nih.gov/clinvar/variation/453594/
NM_002485.4(NBN):c.2201C>T	concordant	unreportable	unreportable	483988	0	1	SCV000690699	Uncertain significance	Color Genomics, In	12/21/2017	NBN	chr8	90949287	90949287		8.37E-06					0	0	https://www.ncbi.nlm.nih.gov/clinvar/variation/483988/
NM_000492.3(CFTR):c.4273G>A	concordant	unreportable	unreportable	495950	0	1	SCV000697021	Uncertain significance	Laboratory Corpor	1/25/2018	CFTR	chr7	117306992	117306992		3.85E-06					0	0	https://www.ncbi.nlm.nih.gov/clinvar/variation/495950/
NM_000543.4(SMPD1):c.529A>T	concordant	unreportable	unreportable	286802	0	1	SCV000340367	Uncertain significance	EGL Genetic Diagn	6/30/2017	SMPD1	chr11	6412824	6412824	http://gnomad.b	7.14E-05	EAS	0.0001201490013	0		0	0	https://www.ncbi.nlm.nih.gov/clinvar/variation/286802/
NM_001079802.1(FKTN):c.531T>A	concordant	unreportable	unreportable	285381	0	1	SCV000338377	Uncertain significance	EGL Genetic Diagn	6/30/2017	FKTN	chr9	108366657	108366657	http://gnomad.b	2.84E-05	SAS	0.0005522710271	1		0	0	https://www.ncbi.nlm.nih.gov/clinvar/variation/285381/
NM_000051.3(ATM):c.1251C>T	concordant	unreportable	unreportable	490417	0	1	SCV000687293	Likely benign	Color Genomics, In	12/21/2017	ATM	chr11	108121443	108121443		2.78E-06					0	0	https://www.ncbi.nlm.nih.gov/clinvar/variation/490417/
NM_000053.3(ATP7B):c.676C>T	concordant	unreportable	unreportable	312397	0	1	SCV000384684	Uncertain significance	Illumina Clinical Se	10/18/2016	ATP7B	chr13	52548680	52548680	http://gnomad.b	1.96E-05	EAS	0.0003478660074	0		0	0	https://www.ncbi.nlm.nih.gov/clinvar/variation/312397/
NM_001164277.1(SLC37A4):c.80A>G	concordant	unreportable	unreportable	459624	0	1	SCV000631385	Uncertain significance	Invitae	10/5/2017	SLC37A4	chr11	118900000	118900000		3.63E-05					0	0	https://www.ncbi.nlm.nih.gov/clinvar/variation/459624/
[truncated: 1,898,702 more chars]
