# Supplementary material for: Inter‐lab concordance of variant classifications establishes clinical validity of expanded carrier screening
Source: Clin Genet. 2019 Jul 1;96(3):236–45. doi: 10.1111/cge.13582 (PMC6852020; doi:10.1111/cge.13582)
Supplement: Supplementary file 3 — TABLE S3 Details of the 106 variants excluded from the clinical variant classification performance analysis [file CGE-96-236-s003.pdf]

Supplemental Table 3

standard_name	category	MWH	ClinVar	clinvar_id	n_reportable_cv	n_unreportable_cv	accession	raw_clinical_significance	submitter	submission_date	gene	chrom	start	stop	gnomad_link	allele_frequency	gnomad_POPMAX	gnomad_AF_POPMAX	gnomad_Hom	gnomad_Hemi	MWH_Hom	MWH_Hemi	clinvar_link	reason_for_exclusion
NM_000016.4(ACADM):c.503A>C	discordant	unreportable	reportable	226072	1	0	SCV000511930	Likely pathogenic	GeneDx	11/28/2017	ACADM	chr1	76205699	76205699	http://gnomad	1.09E-05	NFE	1.79E-05	0		0	0	https://www.ncbi.nlm.nih.gov/clinvar/variation/226072/	Unclear submitter classification
NM_000017.2(ACADS):c.815G>A	discordant	unreportable	reportable	203556	1	0	SCV000238609	Likely pathogenic	GeneDx	11/28/2017	ACADS	chr12	121176355	121176355	http://gnomad	6.23E-05	AFR	0.000131234	0		0	0	https://www.ncbi.nlm.nih.gov/clinvar/variation/203556/	Unclear submitter classification
NM_000383.3(AIRE):c.274C>T	discordant	reportable	unreportable	379319	0	1	SCV000696653	Uncertain significance	Laboratory Co	1/25/2018	AIRE	chr21	45706581	45706581		1.39E-05					0	0	https://www.ncbi.nlm.nih.gov/clinvar/variation/379319/	Unclear submitter classification
NM_000487.5(ARSA):c.938G>A	discordant	unreportable	reportable	68165	1	0	SCV000521136	Likely pathogenic	GeneDx	11/28/2017	ARSA	chr22	51064623	51064623	http://gnomad	1.13E-05	SAS	3.33E-05	0		0	0	https://www.ncbi.nlm.nih.gov/clinvar/variation/68165/	Unclear submitter classification
NM_000053.3(ATP7B):c.122A>G	discordant	unreportable	reportable	157928	4	0	SCV000331624;S	Likely pathogenic;Likely pathog	EGL Genetic D	2017-06-30;2017-11-28;2	ATP7B	chr13	52549234	52549234	http://gnomad	0.00026656	NFE	0.000430208	0		0	0	https://www.ncbi.nlm.nih.gov/clinvar/variation/157928/	Other reason, not ECS-appropriate
NM_000053.3(ATP7B):c.2755C>T	discordant	reportable	unreportable	312386	1	1	SCV000384667;S	Uncertain significance;Likely pa	Illumina Clini	2016-10-18;2018-01-25	ATP7B	chr13	52523908	52523908	http://gnomad	8.14E-05	AFR	0.000197602	0		1	0	https://www.ncbi.nlm.nih.gov/clinvar/variation/312386/	Unclear submitter classification
NM_004328.4(BCS1L):c.550C>T	discordant	unreportable	reportable	6171	1	0	SCV000329628	Pathogenic	GeneDx	11/28/2017	BCS1L	chr2	219526571	219526571	http://gnomad	0.000276071	ASJ	0.00294536	1		0	0	https://www.ncbi.nlm.nih.gov/clinvar/variation/6171/	Homozygotes observed in the population
NM_000060.2(BTD):c.443G>A	discordant	unreportable	reportable	25005	1	0	SCV000576611	Likely pathogenic	GeneDx	11/28/2017	BTD	chr3	15683548	15683548	http://gnomad	8.94E-05	AFR	0.000196155	0		0	0	https://www.ncbi.nlm.nih.gov/clinvar/variation/25005/	No published cases
NM_000492.3(CFTR):c.2939T>A	discordant	unreportable	reportable	53604	1	0	SCV000696937	Likely pathogenic	Laboratory Co	1/25/2018	CFTR	chr7	117246758	117246758	http://gnomad	1.16E-05	AMR	2.98E-05	0		0	0	https://www.ncbi.nlm.nih.gov/clinvar/variation/53604/	Other reason, not ECS-appropriate
NM_000492.3(CFTR):c.2856G>C	discordant	unreportable	reportable	53580	1	0	SCV000696930	Pathogenic	Laboratory Co	1/25/2018	CFTR	chr7	117243784	117243784	http://gnomad	0.000111698	NFE	0.000107498	0		0	0	https://www.ncbi.nlm.nih.gov/clinvar/variation/53580/	Other reason, not ECS-appropriate
NM_000492.3(CFTR):c.14C>T	discordant	reportable	unreportable	35824	1	1	SCV000074336;S	Uncertain significance;Pathoge	Invitae;Labor	2017-03-14;2015-11-20	CFTR	chr7	117120162	117120162	http://gnomad	6.36E-05	NFE	5.38E-05	0		0	0	https://www.ncbi.nlm.nih.gov/clinvar/variation/35824/	Other reason, not ECS-appropriate
NM_001876.3(CPT1A):c.1436C>T	discordant	unreportable	reportable	65644	2	0	SCV000321521;S	Pathogenic;Pathogenic	GeneDx;Invita	2017-11-28;2017-10-05	CPT1A	chr11	68548130	68548130	http://gnomad	6.48E-05	AMR	5.96E-05	0		2	0	https://www.ncbi.nlm.nih.gov/clinvar/variation/65644/	Homozygotes observed in the population
NM_000153.3(GALC):c.1468T>A	discordant	unreportable	reportable	431995	1	0	SCV000589634	Likely pathogenic	GeneDx	11/28/2017	GALC	chr14	88414093	88414093		5.59E-06					0	0	https://www.ncbi.nlm.nih.gov/clinvar/variation/431995/	Unclear submitter classification
NM_000153.3(GALC):c.1901T>C	discordant	reportable	unreportable	225368	1	1	SCV000389235;S	Uncertain significance;Pathoge	Illumina Clini	2016-10-18;2018-01-25	GALC	chr14	88406259	88406259	http://gnomad	0.000738428	EAS	0.00875768	0		2	0	https://www.ncbi.nlm.nih.gov/clinvar/variation/225368/	Homozygotes observed in the population
NM_001128227.2(GNE):c.2179G>A	discordant	unreportable	reportable	6028	3	0	SCV000331242;S	Pathogenic;Pathogenic;Likely p	EGL Genetic D	2017-06-30;2017-11-28;2	GNE	chr9	36217445	36217445	http://gnomad	0.001092621	SAS	0.0135794	2		5	0	https://www.ncbi.nlm.nih.gov/clinvar/variation/6028/	Other reason, not ECS-appropriate
NM_000520.4(HEXA):c.748G>A	discordant	unreportable	reportable	381668	1	0	SCV000521180	Pathogenic	GeneDx	11/28/2017	HEXA	chr15	72642916	72642916		2.35E-06					0	0	https://www.ncbi.nlm.nih.gov/clinvar/variation/381668/	No published cases
NM_000243.2(MEFV):c.2084A>G	discordant	unreportable	reportable	2547	4	1	SCV000604187;S	Pathogenic;Likely pathogenic;P	ARUP Laborat	2017-06-30;2017-06-30;2	MEFV	chr16	3293403	3293403	http://gnomad	0.005012594	FIN	0.0165934	8		12	0	https://www.ncbi.nlm.nih.gov/clinvar/variation/2547/	Other reason, not ECS-appropriate
NM_000243.2(MEFV):c.2230G>T	discordant	unreportable	reportable	2548	6	0	SCV000604174;S	Pathogenic;Pathogenic;Pathoge	ARUP Laborat	2017-06-30;2017-06-30;2	MEFV	chr16	3293257	3293257	http://gnomad	0.00345368	ASJ	0.0126904	2		5	0	https://www.ncbi.nlm.nih.gov/clinvar/variation/2548/	Other reason, not ECS-appropriate
NM_000277.1(PAH):c.601C>T	discordant	reportable	unreportable	102752	1	1	SCV000629203;S	Uncertain significance;Pathoge	Invitae;Labor	2017-10-05;2018-01-25	PAH	chr12	103249019	103249019	http://gnomad	2.47E-05	NFE	8.96E-06	0		0	0	https://www.ncbi.nlm.nih.gov/clinvar/variation/102752/	Other reason, not ECS-appropriate
NM_000277.1(PAH):c.506G>A	discordant	unreportable	reportable	102706	3	1	SCV000239055;S	Likely pathogenic;Likely pathog	GeneDx;Gene	2017-11-28;2017-07-05;2	PAH	chr12	103260377	103260377	http://gnomad	0.000682166	ASJ	0.0063655	0		0	0	https://www.ncbi.nlm.nih.gov/clinvar/variation/102706/	Unclear submitter classification
NM_000277.1(PAH):c.212G>A	discordant	unreportable	reportable	102633	1	0	SCV000239046	Likely pathogenic	GeneDx	11/28/2017	PAH	chr12	103288653	103288653		1.37E-05					0	0	https://www.ncbi.nlm.nih.gov/clinvar/variation/102633/	Unclear submitter classification
NM_000303.2(PMM2):c.647A>G	discordant	unreportable	reportable	198713	1	0	SCV000568194	Likely pathogenic	GeneDx	11/28/2017	PMM2	chr16	8941588	8941588	http://gnomad	4.45E-05	NFE	3.58E-05	0		0	0	https://www.ncbi.nlm.nih.gov/clinvar/variation/198713/	Unclear submitter classification
NM_000023.2(SGCA):c.402C>G	discordant	reportable	unreportable	284945	1	1	SCV000337768;S	Pathogenic;Uncertain significar	EGL Genetic D	2017-06-30;2016-10-18	SGCA	chr17	48245751	48245751		1.68E-05					0	0	https://www.ncbi.nlm.nih.gov/clinvar/variation/284945/	Unclear submitter classification
NM_003060.3(SLC22A5):c.1463G>A	discordant	unreportable	reportable	38794	2	1	SCV000111946;S	Pathogenic;Pathogenic;Likely b	EGL Genetic D	2017-06-30;2017-11-28;2	SLC22A5	chr5	131729380	131729380	http://gnomad	0.00327041	NFE	0.00471064	6		1	0	https://www.ncbi.nlm.nih.gov/clinvar/variation/38794/	Other reason, not ECS-appropriate
NM_000441.1(SLC26A4):c.2153T>C	discordant	unreportable	reportable	381687	1	0	SCV000521214	Likely pathogenic	GeneDx	11/28/2017	SLC26A4	chr7	107350562	107350562	http://gnomad	3.07E-05	NFE	6.27E-05	0		0	0	https://www.ncbi.nlm.nih.gov/clinvar/variation/381687/	Unclear submitter classification
NM_199292.2(TH):c.1196C>T	discordant	unreportable	reportable	378729	1	0	SCV000514900	Likely pathogenic	GeneDx	11/28/2017	TH	chr11	2187233	2187233		1.41E-05					0	0	https://www.ncbi.nlm.nih.gov/clinvar/variation/378729/	Unclear submitter classification
NM_000049.2(ASPA):c.237-2A>T	discordant	reportable	unreportable	189114	0	1	SCV000401981	Uncertain significance	Illumina Clini	10/18/2016	ASPA	chr17	3384895	3384895		7.10E-06					0	0	https://www.ncbi.nlm.nih.gov/clinvar/variation/189114/	Unclear submitter classification
NM_017739.3(POMGNT1):c.1285-2A>G	discordant	reportable	unreportable	56578	0	1	SCV000357989	Uncertain significance	Illumina Clini	10/18/2016	POMGNT1	chr1	46658110	46658110	http://gnomad	1.12E-05	NFE	8.97E-06	0		0	0	https://www.ncbi.nlm.nih.gov/clinvar/variation/56578/	Unclear submitter classification
NM_000153.3(GALC):c.956A>G	discordant	unreportable	reportable	265349	3	0	SCV000603777;S	Pathogenic;Pathogenic;Pathoge	ARUP Laborat	2017-06-30;2017-11-28;2	GALC	chr14	88431926	88431926	http://gnomad	0.00102373	SAS	0.00984469	4		1	0	https://www.ncbi.nlm.nih.gov/clinvar/variation/265349/	Homozygotes observed in the population
NM_000017.2(ACADS):c.1192C>T	discordant	unreportable	reportable	377423	1	0	SCV000511940	Likely pathogenic	GeneDx	11/28/2017	ACADS	chr12	121177204	121177204	http://gnomad	5.95E-05	NFE	8.27E-05	0		0	0	https://www.ncbi.nlm.nih.gov/clinvar/variation/377423/	Unclear submitter classification
NM_000153.3(GALC):c.266C>T	discordant	unreportable	reportable	449966	1	0	SCV000618462	Pathogenic	GeneDx	11/28/2017	GALC	chr14	88454550	88454550	http://gnomad	0.000151042	ASJ	0.00030463	0		0	0	https://www.ncbi.nlm.nih.gov/clinvar/variation/449966/	No published cases
NM_000352.3(ABCC8):c.4615G>A	discordant	unreportable	reportable	35623	1	0	SCV000592975	Likely pathogenic	Genetic Servi	7/5/2017	ABCC8	chr11	17414669	17414669		2.58E-06					0	0	https://www.ncbi.nlm.nih.gov/clinvar/variation/35623/	No published cases
NM_000017.2(ACADS):c.322G>A	discordant	unreportable	reportable	377421	1	0	SCV000511936	Likely pathogenic	GeneDx	11/28/2017	ACADS	chr12	121174900	121174900		7.65E-05					0	0	https://www.ncbi.nlm.nih.gov/clinvar/variation/377421/	Unclear submitter classification
NM_000049.2(ASPA):c.770C>G	discordant	unreportable	reportable	430157	1	0	SCV000582889	Likely pathogenic	GeneDx	11/28/2017	ASPA	chr17	3402210	3402210	http://gnomad	2.60E-05	FIN	4.51E-05	0		0	0	https://www.ncbi.nlm.nih.gov/clinvar/variation/430157/	Unclear submitter classification
NM_000155.3(GALT):c.1014C>G	discordant	unreportable	reportable	25316	1	0	SCV000331181	Likely pathogenic	EGL Genetic D	6/30/2017	GALT	chr9	34649516	34649516	http://gnomad	1.37E-05	NFE	2.69E-05	0		0	0	https://www.ncbi.nlm.nih.gov/clinvar/variation/25316/	No published cases
NM_000159.2(GCDH):c.281G>A	discordant	unreportable	reportable	418224	1	0	SCV000565028	Likely pathogenic	GeneDx	11/28/2017	GCDH	chr19	13002939	13002939	http://gnomad	3.37E-05	SAS	0.00100708	0		0	0	https://www.ncbi.nlm.nih.gov/clinvar/variation/418224/	Unclear submitter classification
NM_000543.4(SMPD1):c.1427G>A	discordant	unreportable	reportable	385606	1	0	SCV000526930	Likely pathogenic	GeneDx	11/28/2017	SMPD1	chr11	6415212	6415212	http://gnomad	4.52E-05	SAS	0.000194919	0		0	0	https://www.ncbi.nlm.nih.gov/clinvar/variation/385606/	Unclear submitter classification
NM_005709.3(USH1C):c.1285-2684C>T	discordant	unreportable	reportable	47990	1	0	SCV000340206	Likely pathogenic	EGL Genetic D	6/30/2017	USH1C	chr11	17526211	17526211	http://gnomad	0.000176023	AFR	0.00215658	0		0	0	https://www.ncbi.nlm.nih.gov/clinvar/variation/47990/	No published cases
NM_000082.3(ERCC8):c.479C>T	discordant	unreportable	reportable	1717	1	0	SCV000521089	Likely pathogenic	GeneDx	11/28/2017	ERCC8	chr5	60200621	60200621	http://gnomad	8.00E-06	NFE	1.79E-05	0		0	0	https://www.ncbi.nlm.nih.gov/clinvar/variation/1717/	Unclear submitter classification
NM_033380.2(COL4A5):c.1871G>A	discordant	unreportable	reportable	24455	1	0	SCV000329310	Pathogenic	GeneDx	11/28/2017	COL4A5	chrX	107842023	107842023	http://gnomad	5.81E-05	NFE	0.00020015	0	4	0	3	https://www.ncbi.nlm.nih.gov/clinvar/variation/24455/	Homozygotes observed in the population
NM_206933.2(USH2A):c.5857+2T>C	discordant	reportable	unreportable	48544	0	1	SCV000354057	Uncertain significance	Illumina Clini	10/18/2016	USH2A	chr1	216246229	216246229		1.60E-05					0	0	https://www.ncbi.nlm.nih.gov/clinvar/variation/48544/	Unclear submitter classification
NM_000404.2(GLB1):c.601C>T	discordant	reportable	unreportable	925	1	1	SCV000321710;S	Pathogenic;Uncertain significar	GeneDx;Illum	2017-11-28;2016-10-18	GLB1	chr3	33099713	33099713	http://gnomad	3.20E-05	NFE	8.99E-05	0		0	0	https://www.ncbi.nlm.nih.gov/clinvar/variation/925/	Unclear submitter classification
NM_000124.2(ERCC6):c.2047C>T	discordant	reportable	unreportable	1711	0	1	SCV000362900	Uncertain significance	Illumina Clini	10/18/2016	ERCC6	chr10	50690855	50690855	http://gnomad	8.00E-06	EAS	5.80E-05	0		0	0	https://www.ncbi.nlm.nih.gov/clinvar/variation/1711/	Unclear submitter classification
NM_000495.4(COL4A5):c.5030G>A	discordant	unreportable	reportable	10467	1	0	SCV000612999	Likely pathogenic	Athena Diagn	8/17/2017	COL4A5	chrX	107939580	107939580	http://gnomad	1.94E-05	ASJ	0.000411466	0	0	0	1	https://www.ncbi.nlm.nih.gov/clinvar/variation/10467/	Homozygotes observed in the population
NM_004004.5(GJB2):c.-22-2A>C	discordant	unreportable	reportable	375406	3	0	SCV000603828;S	Pathogenic;Pathogenic;Pathoge	ARUP Laborat	2017-06-30;2018-02-07;2	GJB2	chr13	20763744	20763744	http://gnomad	0.000360434	ASJ	0.00457689	0		0	0	https://www.ncbi.nlm.nih.gov/clinvar/variation/375406/	Other reason, not ECS-appropriate
NM_000023.2(SGCA):c.307A>G	discordant	unreportable	reportable	284685	1	0	SCV000337402	Likely pathogenic	EGL Genetic D	6/30/2017	SGCA	chr17	48245092	48245092		1.68E-05					0	0	https://www.ncbi.nlm.nih.gov/clinvar/variation/284685/	No published cases
NM_000182.4(HADHA):c.1637A>G	discordant	unreportable	reportable	429528	1	0	SCV000582118	Likely pathogenic	GeneDx	11/28/2017	HADHA	chr2	26417491	26417491	http://gnomad	4.49E-05	AFR	0.000326797	0		0	0	https://www.ncbi.nlm.nih.gov/clinvar/variation/429528/	Unclear submitter classification
NM_000060.2(BTD):c.442C>T	discordant	unreportable	reportable	373539	1	0	SCV000492140	Likely pathogenic	GeneDx	12/21/2016	BTD	chr3	15683547	15683547	http://gnomad	4.90E-05	NFE	0.000313609	1		0	0	https://www.ncbi.nlm.nih.gov/clinvar/variation/373539/	Unclear submitter classification
NR_003051.3(RMRP):n.146C>T	discordant	unreportable	reportable	383542	1	0	SCV000523960	Likely pathogenic	GeneDx	2/15/2017	RMRP	chr9	35657870	35657870	http://gnomad	0.000172837	SAS	0.000178221	0		0	0	https://www.ncbi.nlm.nih.gov/clinvar/variation/383542/	Unclear submitter classification
NM_000543.4(SMPD1):c.1299T>G	discordant	unreportable	reportable	93313	1	0	SCV000230119	Likely pathogenic	EGL Genetic D	6/30/2017	SMPD1	chr11	6414882	6414882		4.76E-06					0	0	https://www.ncbi.nlm.nih.gov/clinvar/variation/93313/	No published cases
NM_183050.2(BCKDHB):c.974T>G	discordant	unreportable	reportable	96622	1	0	SCV000331066	Likely pathogenic	EGL Genetic D	6/30/2017	BCKDHB	chr6	80982874	80982874		1.28E-05					0	0	https://www.ncbi.nlm.nih.gov/clinvar/variation/96622/	No published cases
NM_000159.2(GCDH):c.641C>T	discordant	unreportable	reportable	430567	1	0	SCV000583398	Likely pathogenic	GeneDx	11/28/2017	GCDH	chr19	13007024	13007024	http://gnomad	1.12E-05	AMR	5.96E-05	0		0	0	https://www.ncbi.nlm.nih.gov/clinvar/variation/430567/	Unclear submitter classification
NM_004328.4(BCS1L):c.269G>A	discordant	unreportable	reportable	214165	1	0	SCV000251199	Likely pathogenic	GeneDx	11/28/2017	BCS1L	chr2	219525979	219525979	http://gnomad	1.39E-05	AFR	0.000130685	0		0	0	https://www.ncbi.nlm.nih.gov/clinvar/variation/214165/	No published cases
NM_000228.2(LAMB3):c.225_226delCA	discordant	reportable	unreportable	295145	0	1	SCV000353675	Uncertain significance	Illumina Clini	10/18/2016	LAMB3	chr1	209811950	209811952		8.40E-06					0	0	https://www.ncbi.nlm.nih.gov/clinvar/variation/295145/	Unclear submitter classification
NM_000017.2(ACADS):c.328G>A	discordant	unreportable	reportable	432099	1	0	SCV000589781	Likely pathogenic	GeneDx	11/28/2017	ACADS	chr12	121174906	121174906		2.27E-05					0	0	https://www.ncbi.nlm.nih.gov/clinvar/variation/432099/	Unclear submitter classification
NM_000017.2(ACADS):c.989_990delGCinsAT	discordant	unreportable	reportable	431801	1	0	SCV000589305	Likely pathogenic	GeneDx	11/28/2017	ACADS	chr12	121176677	121176679		1.98E-05					0	0	https://www.ncbi.nlm.nih.gov/clinvar/variation/431801/	No published cases
NM_000271.4(NPC1):c.2849T>G	discordant	unreportable	reportable	372774	1	0	SCV000491261	Likely pathogenic	GeneDx	12/21/2016	NPC1	chr18	21119381	21119381	http://gnomad	2.57E-05	NFE	1.79E-05	0		0	0	https://www.ncbi.nlm.nih.gov/clinvar/variation/372774/	Unclear submitter classification
NM_000018.3(ACADVL):c.1748C>G	discordant	unreportable	reportable	426482	1	0	SCV000576934	Likely pathogenic	GeneDx	11/28/2017	ACADVL	chr17	7128030	7128030		8.50E-06					0	0	https://www.ncbi.nlm.nih.gov/clinvar/variation/426482/	Unclear submitter classification
NM_000053.3(ATP7B):c.2002A>G	discordant	unreportable	reportable	157934	1	0	SCV000535376	Likely pathogenic	GeneDx	11/28/2017	ATP7B	chr13	52534403	52534403	http://gnomad	5.61E-06	NFE	8.98E-06	0		0	0	https://www.ncbi.nlm.nih.gov/clinvar/variation/157934/	Unclear submitter classification
NM_152419.2(HGSNAT):c.1345dupG	discordant	reportable	unreportable	1231	0	1	SCV000474016	Uncertain significance	Illumina Clini	10/18/2016	HGSNAT	chr8	43047540	43047540	http://gnomad	1.60E-05	NFE	8.98E-06	0		0	0	https://www.ncbi.nlm.nih.gov/clinvar/variation/1231/	Unclear submitter classification
NM_206933.2(USH2A):c.12575G>A	discordant	unreportable	reportable	166434	2	0	SCV000331629;S	Pathogenic;Likely pathogenic	EGL Genetic D	2017-06-30;2017-11-28	USH2A	chr1	215848678	215848678	http://gnomad	0.001122676	ASJ	0.00829408	1		0	0	https://www.ncbi.nlm.nih.gov/clinvar/variation/166434/	Unclear submitter classification
NM_206933.2(USH2A):c.5012G>A	discordant	reportable	unreportable	179773	0	1	SCV000337203	Uncertain significance	EGL Genetic D	6/30/2017	USH2A	chr1	216258195	216258195	http://gnomad	6.42E-05	SAS	0.000389838	0		1	0	https://www.ncbi.nlm.nih.gov/clinvar/variation/179773/	Homozygotes observed in the population
NM_017739.3(POMGNT1):c.1463G>A	discordant	unreportable	reportable	422459	1	0	SCV000571942	Likely pathogenic	GeneDx	11/28/2017	POMGNT1	chr1	46657846	46657846	http://gnomad	1.40E-05	NFE	4.48E-05	0		0	0	https://www.ncbi.nlm.nih.gov/clinvar/variation/422459/	No published cases
NM_003494.3(DYSF):c.701G>A	discordant	unreportable	reportable	198495	1	0	SCV000332010	Likely pathogenic	EGL Genetic D	6/30/2017	DYSF	chr2	71742790	71742790	http://gnomad	3.23E-05	AMR	0.000148889	0		0	0	https://www.ncbi.nlm.nih.gov/clinvar/variation/198495/	Other reason, not ECS-appropriate
NM_000199.3(SGSH):c.235A>C	discordant	unreportable	reportable	372782	1	0	SCV000491272	Likely pathogenic	GeneDx	12/21/2016	SGSH	chr17	78190845	78190845		2.40E-05					0	0	https://www.ncbi.nlm.nih.gov/clinvar/variation/372782/	Unclear submitter classification
NM_000199.3(SGSH):c.1063G>A	discordant	reportable	unreportable	325835	1	1	SCV000491270;S	Likely pathogenic;Uncertain sig	GeneDx;Illum	2016-12-21;2016-10-18	SGSH	chr17	78184697	78184697	http://gnomad	3.20E-05	EAS	0.000116347	0		0	0	https://www.ncbi.nlm.nih.gov/clinvar/variation/325835/	Unclear submitter classification
NM_000391.3(TPP1):c.887-18A>G	discordant	unreportable	reportable	381560	1	0	SCV000520944	Likely pathogenic	GeneDx	11/28/2017	TPP1	chr11	6637752	6637752		2.80E-06					0	0	https://www.ncbi.nlm.nih.gov/clinvar/variation/381560/	Unclear submitter classification
NM_000282.3(PCCA):c.923dupT	discordant	reportable	unreportable	310846	0	1	SCV000382013	Uncertain significance	Illumina Clini	10/18/2016	PCCA	chr13	100925451	100925451	http://gnomad	2.40E-05	ASJ	0.000101771	0		0	0	https://www.ncbi.nlm.nih.gov/clinvar/variation/310846/	Unclear submitter classification
NM_138413.3(HOGA1):c.107C>T	discordant	unreportable	reportable	204265	1	0	SCV000366314	Likely pathogenic	Illumina Clini	10/18/2016	HOGA1	chr10	99344567	99344567	http://gnomad	0.000370627	ASJ	0.00284495	0		1	0	https://www.ncbi.nlm.nih.gov/clinvar/variation/204265/	Homozygotes observed in the population
NM_001128227.2(GNE):c.79C>T	discordant	unreportable	reportable	195244	1	0	SCV000701243	Pathogenic	EGL Genetic D	6/30/2017	GNE	chr9	36249367	36249367	http://gnomad	5.60E-06	SAS	3.26E-05	0		0	0	https://www.ncbi.nlm.nih.gov/clinvar/variation/195244/	No published cases
NM_000070.2(CAPN3):c.1303G>A	discordant	unreportable	reportable	282173	2	0	SCV000333467;S	Pathogenic;Likely pathogenic	EGL Genetic D	2017-06-30;2017-11-28	CAPN3	chr15	42691799	42691799	http://gnomad	8.00E-05	AMR	0.00026848	0		0	0	https://www.ncbi.nlm.nih.gov/clinvar/variation/282173/	Unclear submitter classification
NM_000920.3(PC):c.796T>G	discordant	unreportable	reportable	381581	1	0	SCV000520993	Likely pathogenic	GeneDx	11/28/2017	PC	chr11	66637880	66637880		8.00E-06					0	0	https://www.ncbi.nlm.nih.gov/clinvar/variation/381581/	Unclear submitter classification
NM_006019.3(TCIRG1):c.1297C>T	discordant	reportable	unreportable	305804	0	1	SCV000373721	Uncertain significance	Illumina Clini	10/18/2016	TCIRG1	chr11	67815031	67815031	http://gnomad	8.00E-06	FIN	0.000225428	0		0	0	https://www.ncbi.nlm.nih.gov/clinvar/variation/305804/	Unclear submitter classification
NM_152618.2(BBS12):c.1092delA	discordant	reportable	unreportable	347497	0	1	SCV000447409	Uncertain significance	Illumina Clini	10/18/2016	BBS12	chr4	123664138	123664139	http://gnomad	2.40E-05	NFE	3.58E-05	0		0	0	https://www.ncbi.nlm.nih.gov/clinvar/variation/347497/	Unclear submitter classification
NM_000053.3(ATP7B):c.-676A>G	discordant	reportable	unreportable	393099	0	1	SCV000536461	Uncertain significance	GeneDx	11/28/2017	ATP7B	chr13	52586149	52586149		8.42E-05					0	0	https://www.ncbi.nlm.nih.gov/clinvar/variation/393099/	Unclear submitter classification
NM_000920.3(PC):c.1185+5_1185+8delGCGG	discordant	unreportable	reportable	426656	1	0	SCV000577158	Likely pathogenic	GeneDx	11/28/2017	PC	chr11	66633649	66633653	http://gnomad	0.000200074	SAS	0.000424171	0		0	0	https://www.ncbi.nlm.nih.gov/clinvar/variation/426656/	Unclear submitter classification
NM_000070.2(CAPN3):c.1636C>T	discordant	unreportable	reportable	284465	1	0	SCV000700977	Likely pathogenic	EGL Genetic D	6/30/2017	CAPN3	chr15	42695091	42695091	http://gnomad	3.20E-05	AMR	0.000148889	0		0	0	https://www.ncbi.nlm.nih.gov/clinvar/variation/284465/	No published cases
NM_000135.2(FANCA):c.3482C>T	discordant	unreportable	reportable	435126	1	0	SCV000594653	Likely pathogenic	Genetic Servi	7/5/2017	FANCA	chr16	89813023	89813023	http://gnomad	2.40E-05	SAS	6.50E-05	0		0	0	https://www.ncbi.nlm.nih.gov/clinvar/variation/435126/	No published cases
NM_032957.4(RTEL1):c.1720C>T	discordant	unreportable	reportable	422356	1	0	SCV000571807	Likely pathogenic	GeneDx	11/28/2017	RTEL1	chr20	62319665	62319665	http://gnomad	3.20E-05	EAS	0.000116104	0		0	0	https://www.ncbi.nlm.nih.gov/clinvar/variation/422356/	No published cases
NM_000108.3(DLD):c.161A>G	discordant	unreportable	reportable	424265	1	0	SCV000574056	Likely pathogenic	GeneDx	11/28/2017	DLD	chr7	107542225	107542225		1.04E-05					0	0	https://www.ncbi.nlm.nih.gov/clinvar/variation/424265/	No published cases
NM_000277.1(PAH):c.757G>A	discordant	unreportable	reportable	422049	1	0	SCV000571414	Likely pathogenic	GeneDx	11/28/2017	PAH	chr12	103246678	103246678	http://gnomad	5.48E-06	NFE	8.97E-06	0		0	0	https://www.ncbi.nlm.nih.gov/clinvar/variation/422049/	No published cases
NR_003051.3(RMRP):n.148C>T	discordant	unreportable	reportable	426728	1	0	SCV000577254	Likely pathogenic	GeneDx	11/28/2017	RMRP	chr9	35657868	35657868		5.58E-06					0	0	https://www.ncbi.nlm.nih.gov/clinvar/variation/426728/	Unclear submitter classification
NM_017777.3(MKS1):c.1208C>T	discordant	unreportable	reportable	217672	1	0	SCV000571461	Likely pathogenic	GeneDx	11/28/2017	MKS1	chr17	56285320	56285320	http://gnomad	8.00E-06	AFR	6.54E-05	0		0	0	https://www.ncbi.nlm.nih.gov/clinvar/variation/217672/	Unclear submitter classification
NM_000050.4(ASS1):c.1173C>A	discordant	unreportable	reportable	432044	1	0	SCV000589715	Likely pathogenic	GeneDx	11/28/2017	ASS1	chr9	133374937	133374937		2.79E-06					0	0	https://www.ncbi.nlm.nih.gov/clinvar/variation/432044/	Unclear submitter classification
NM_003060.3(SLC22A5):c.842C>T	discordant	unreportable	reportable	418493	1	0	SCV000565569	Likely pathogenic	GeneDx	11/28/2017	SLC22A5	chr5	131722734	131722734	http://gnomad	5.65E-06	NFE	8.95E-06	0		0	0	https://www.ncbi.nlm.nih.gov/clinvar/variation/418493/	Unclear submitter classification
NM_183050.2(BCKDHB):c.637G>T	discordant	unreportable	reportable	390609	1	0	SCV000533487	Likely pathogenic	GeneDx	11/28/2017	BCKDHB	chr6	80881002	80881002		7.69E-06					0	0	https://www.ncbi.nlm.nih.gov/clinvar/variation/390609/	Unclear submitter classification
NM_000260.3(MYO7A):c.2904G>A	discordant	unreportable	reportable	228280	1	0	SCV000271248	Likely pathogenic	Laboratory fo	4/26/2016	MYO7A	chr11	76892635	76892635	http://gnomad	8.00E-06	EAS	5.81E-05	0		0	0	https://www.ncbi.nlm.nih.gov/clinvar/variation/228280/	No published cases
NM_002485.4(NBN):c.2184+1G>T	discordant	reportable	unreportable	233424	1	1	SCV000571724;S	Uncertain significance;Likely pa	GeneDx;Invita	2017-07-13;2017-03-14	NBN	chr8	90955480	90955480	http://gnomad	1.12E-05	AFR	0.000130736	0		0	0	https://www.ncbi.nlm.nih.gov/clinvar/variation/233424/	Unclear submitter classification
NM_000303.2(PMM2):c.337C>A	discordant	unreportable	reportable	420784	1	0	SCV000569753	Likely pathogenic	GeneDx	11/28/2017	PMM2	chr16	8900254	8900254	http://gnomad	5.57E-06	AMR	2.98E-05	0		0	0	https://www.ncbi.nlm.nih.gov/clinvar/variation/420784/	Unclear submitter classification
NM_001271208.1(NEB):c.24094C>T	discordant	unreportable	reportable	373977	1	0	SCV000571535	Likely pathogenic	GeneDx	11/28/2017	NEB	chr2	152357937	152357937	http://gnomad	0.000139407	NFE	0.000509442	0		0	0	https://www.ncbi.nlm.nih.gov/clinvar/variation/373977/	Unclear submitter classification
NM_052845.3(MMAB):c.377C>T	discordant	unreportable	reportable	432132	1	0	SCV000589819	Likely pathogenic	GeneDx	11/28/2017	MMAB	chr12	109999629	109999629		1.60E-05					0	0	https://www.ncbi.nlm.nih.gov/clinvar/variation/432132/	Unclear submitter classification
NM_000426.3(LAMA2):c.4523G>A	discordant	unreportable	reportable	422393	1	0	SCV000571860	Likely pathogenic	GeneDx	11/28/2017	LAMA2	chr6	129670529	129670529	http://gnomad	1.60E-05	AMR	2.98E-05	0		0	0	https://www.ncbi.nlm.nih.gov/clinvar/variation/422393/	Unclear submitter classification
NM_000317.2(PTS):c.248C>A	discordant	unreportable	reportable	432083	1	0	SCV000589759	Likely pathogenic	GeneDx	11/28/2017	PTS	chr11	112103890	112103890		1.60E-05					0	0	https://www.ncbi.nlm.nih.gov/clinvar/variation/432083/	Unclear submitter classification
NM_031885.3(BBS2):c.118G>T	discordant	unreportable	reportable	285263	1	0	SCV000338210	Likely pathogenic	EGL Genetic D	6/30/2017	BBS2	chr16	56548592	56548592		2.40E-05					0	0	https://www.ncbi.nlm.nih.gov/clinvar/variation/285263/	No published cases
NM_000521.3(HEXB):c.1417G>A	discordant	unreportable	reportable	372685	1	0	SCV000491081	Likely pathogenic	GeneDx	12/21/2016	HEXB	chr5	74014796	74014796	http://gnomad	1.60E-05	NFE	8.95E-06	0		0	0	https://www.ncbi.nlm.nih.gov/clinvar/variation/372685/	No published cases
NM_001918.3(DBT):c.401C>T	discordant	unreportable	reportable	429606	1	0	SCV000582217	Likely pathogenic	GeneDx	11/28/2017	DBT	chr1	100696321	100696321	http://gnomad	7.98E-06	NFE	1.80E-05	0		0	0	https://www.ncbi.nlm.nih.gov/clinvar/variation/429606/	Unclear submitter classification
NM_000091.4(COL4A3):c.520G>A	discordant	unreportable	reportable	447175	1	0	SCV000612953	Pathogenic	Athena Diagn	8/17/2017	COL4A3	chr2	228113210	228113210	http://gnomad	1.60E-05	AMR	2.98E-05	0		0	0	https://www.ncbi.nlm.nih.gov/clinvar/variation/447175/	No published cases
NM_138694.3(PKHD1):c.3474G>A	discordant	reportable	unreportable	357440	0	1	SCV000464094	Uncertain significance	Illumina Clini	10/18/2016	PKHD1	chr6	51893040	51893040		2.78E-06					0	0	https://www.ncbi.nlm.nih.gov/clinvar/variation/357440/	Unclear submitter classification
NM_000023.2(SGCA):c.614C>T	discordant	unreportable	reportable	423721	1	0	SCV000573450	Likely pathogenic	GeneDx	11/28/2017	SGCA	chr17	48246482	48246482	http://gnomad	2.80E-06	SAS	3.25E-05	0		0	0	https://www.ncbi.nlm.nih.gov/clinvar/variation/423721/	No published cases
NM_004937.2(CTNS):c.944A>G	discordant	unreportable	reportable	422148	1	0	SCV000571544	Likely pathogenic	GeneDx	11/28/2017	CTNS	chr17	3563243	3563243	http://gnomad	2.96E-06	NFE	8.96E-06	0		0	0	https://www.ncbi.nlm.nih.gov/clinvar/variation/422148/	Unclear submitter classification
NM_000277.1(PAH):c.637C>T	discordant	unreportable	reportable	430401	1	0	SCV000583195	Likely pathogenic	GeneDx	11/28/2017	PAH	chr12	103248983	103248983		5.48E-06					0	0	https://www.ncbi.nlm.nih.gov/clinvar/variation/430401/	No published cases
NM_000353.2(TAT):c.340G>A	discordant	unreportable	reportable	432087	1	0	SCV000589763	Likely pathogenic	GeneDx	7/13/2017	TAT	chr16	71609825	71609825	http://gnomad	1.60E-05	AMR	2.98E-05	0		0	0	https://www.ncbi.nlm.nih.gov/clinvar/variation/432087/	Unclear submitter classification
NM_000092.4(COL4A4):c.3734G>T	discordant	unreportable	reportable	447188	1	0	SCV000612969	Likely pathogenic	Athena Diagn	8/17/2017	COL4A4	chr2	227896744	227896744	http://gnomad	8.00E-06	NFE	8.96E-06	0		0	0	https://www.ncbi.nlm.nih.gov/clinvar/variation/447188/	No published cases
NM_000495.4(COL4A5):c.687+5G>A	discordant	unreportable	reportable	432038	1	0	SCV000589709	Likely pathogenic	GeneDx	11/28/2017	COL4A5	chrX	107821354	107821354		9.69E-06					0	0	https://www.ncbi.nlm.nih.gov/clinvar/variation/432038/	Unclear submitter classification
NM_000018.3(ACADVL):c.62+6T>C	discordant	unreportable	reportable	432072	1	0	SCV000589747	Likely pathogenic	GeneDx	11/28/2017	ACADVL	chr17	7123371	7123371		2.83E-06					0	0	https://www.ncbi.nlm.nih.gov/clinvar/variation/432072/	No published cases
NM_000170.2(GLDC):c.1926+5G>A	discordant	unreportable	reportable	462862	1	0	SCV000636369	Likely pathogenic	Invitae	10/5/2017	GLDC	chr9	6565349	6565349		8.00E-06					0	0	https://www.ncbi.nlm.nih.gov/clinvar/variation/462862/	Unclear submitter classification
