# Supplementary material for: Inter‐lab concordance of variant classifications establishes clinical validity of expanded carrier screening
Source: Clin Genet. 2019 Jul 1;96(3):236–45. doi: 10.1111/cge.13582 (PMC6852020; doi:10.1111/cge.13582)
Supplement: Supplementary file 4 — TABLE S4 Per‐gene clinical variant classification performance [file CGE-96-236-s004.pdf]

Supplemental Table 4

gene	disease	Classification sensitivity	Classification specificity	Classification PPV	Classification NPV	MWH positive rate	ClinVar positive rate
Combined ECS	*Combined ECS*	0.98086666	0.999863224	0.994976622	0.999450313	0.317986257	0.321652864
CFTR	cystic fibrosis	0.999038934	0.999521805	0.999230984	0.99940234	0.107313328	0.107333958
ATM	ataxia-telangiectasia	0.981500743	1	1	0.99990418	0.002064143	0.002103048
PAH	phenylalanine hydroxylase deficiency	0.970247786	0.999053986	0.99745079	0.988766247	0.012732412	0.013089393
CAPN3	calpainopathy	0.882533101	0.998847335	0.955952347	0.996677563	0.00325933	0.003530478
ATP7B	Wilson disease	0.981824376	0.998533043	0.955323629	0.999418793	0.005994302	0.005832507
DYSF	dysferlinopathy	0.970023619	0.999961608	0.989804034	0.999884833	0.001583121	0.001615403
USH2A	USH2A-related disorders	0.989716213	0.999869752	0.989716213	0.999869752	0.004657074	0.004657074
GJB2	GJB2-related DFNB1 nonsyndromic hearing loss and deafness	0.983974662	1	1	0.98477833	0.023125368	0.023501995
HBB	Hb beta chain-related hemoglobinopathy	0.99667385	1	1	0.999060872	0.005560733	0.00557929
NBN	Nijmegen breakage syndrome	0.996034878	1	1	0.999903647	0.001399037	0.001404606
GAA	Pompe disease	0.63964578	0.999777779	0.978076032	0.994444734	0.001771691	0.002709074
PKHD1	PKHD1-related autosomal recessive polycystic kidney disease	0.994283743	0.999940915	0.988632492	0.999970456	0.002926135	0.002909504
VPS13B	Cohen syndrome	0.990479115	1	1	0.99998607	0.000582164	0.00058776
SLC22A5	primary carnitine deficiency	0.676979899	1	1	0.96526856	0.002702276	0.003991664
LAMA2	LAMA2-related muscular dystrophy	1	0.999969882	0.987350046	1	0.001263567	0.001247583
NEB	NEB-related nemaline myopathy	0.991938185	1	1	0.999992748	0.000634885	0.000640045
NPC1	Niemann-Pick disease type C	0.986310516	1	1	0.9998695	0.001234478	0.001251612
ACADVL	very long chain acyl-CoA dehydrogenase deficiency	0.962190306	0.999294111	0.988348285	0.997650995	0.002907976	0.002987032
SLC26A4	Pendred syndrome	0.914162718	0.994554045	0.966655857	0.985313281	0.003005618	0.003178207
GALT	galactosemia	0.88665668	0.998344277	0.971766299	0.992755903	0.000972554	0.001065909
DHCR7	Smith-Lemli-Opitz syndrome	0.99545843	1	1	0.999111341	0.011665681	0.011718903
GCDH	glutaric acidemia type 1	0.968784542	1	1	0.994894971	0.002089585	0.002156914
FANCC	Fanconi anemia type C	0.965809835	1	1	0.999084114	0.001877401	0.001943862
ABCC8	ABCC8-related hyperinsulinism	0.960544526	1	1	0.999372356	0.002006678	0.002089105
AGL	glycogen storage disease type III	0.984968261	0.999740421	0.93573865	0.999942303	0.000781196	0.000742151
ACADS	short chain acyl-CoA dehydrogenase deficiency	0.96032014	1	1	0.995848606	0.006535841	0.006805898
SMPD1	Niemann-Pick disease, SMPD1-associated	0.949565679	1	1	0.999481176	0.001431045	0.001507052
GNE	inclusion body myopathy 2	1	0.999703768	0.98815124	1	0.001416684	0.001399898
MYO7A	MYO7A-related disorders	1	0.999688036	0.904323695	1	0.001503239	0.001359415
ARSA	metachromatic leukodystrophy	0.967335868	0.998762909	0.95486012	0.999116049	0.000871979	0.000860733
BTD	biotinidase deficiency	0.831046389	1	1	0.998639806	0.000680229	0.000818521
CBS	homocystinuria caused by cystathionine beta-synthase deficiency	0.991437757	1	1	0.999811924	0.001949826	0.001966665
TGM1	TGM1-related autosomal recessive congenital ichthyosis	0.985516384	1	1	0.999132782	0.001090489	0.001106515
BBS10	Bardet-Biedl syndrome, BBS10-related	1	1	1	1	0.000805484	0.000805484
BLM	Bloom syndrome	1	1	1	1	0.000722789	0.000722789
HEXA	hexosaminidase A deficiency	1	1	1	1	0.003410237	0.003410237
PMM2	congenital disorder of glycosylation type Ia	1	1	1	1	0.001813738	0.001813738
SGSH	mucopolysaccharidosis type IIIA	0.991080147	1	1	0.99937057	0.001775015	0.00179099
FANCA	Fanconi anemia complementation group A	0.96668313	1	1	0.999887588	0.000927517	0.000959484
ACADM	medium chain acyl-CoA dehydrogenase deficiency	0.826139291	0.996193004	0.897670122	0.992994354	0.000690571	0.000750364
GLB1	GLB1-related disorders	1	1	1	1	0.000591992	0.000591992
MUT	MUT-related methylmalonic acidemia	1	0.999737682	0.96875885	1	0.000511407	0.00049543
IVD	isovaleric acidemia	0.991135267	1	1	0.999258458	0.001876273	0.001893054
GALC	Krabbe disease	0.989133558	1	1	0.999959472	0.000508956	0.000514547
G6PC	glycogen storage disease type Ia	1	1	1	1	0.002864766	0.002864766
HEXB	Sandhoff disease	1	1	1	1	0.001695224	0.001695224
NPHS1	congenital Finnish nephrosis	1	1	1	1	0.000952927	0.000952927
SGCA	alpha-sarcoglycanopathy	1	1	1	1	0.001387272	0.001387272

ASS1	citrullinemia type 1	0.816130171	1	1	0.99666578	0.000395416	0.000484501
CPT2	carnitine palmitoyltransferase II deficiency	1	0.995241555	0.965839481	1	0.005863839	0.005663527
PEX1	PEX1-related Zellweger syndrome spectrum	1	1	1	1	0.000133117	0.000133117
SACS	ARSACS	1	1	1	1	0.000177829	0.000177829
ALPL	hypophosphatasia, autosomal recessive	0.990994105	1	1	0.999915662	0.000619605	0.000625236
CYP27A1	cerebrotendinous xanthomatosis	0.970322159	1	1	0.998184187	0.001567216	0.00161515
AIRE	polyglandular autoimmune syndrome type 1	1	1	1	1	0.000723934	0.000723934
ALMS1	Alstrom syndrome	1	1	1	1	0.000400069	0.000400069
FAH	tyrosinemia type I	1	1	1	1	0.002017911	0.002017911
PPT1	PPT1-related neuronal ceroid lipofuscinosis	1	1	1	1	0.000373305	0.000373305
TPP1	TPP1-related neuronal ceroid lipofuscinosis	1	1	1	1	0.001068473	0.001068473
LAMB3	Herlitz junctional epidermolysis bullosa, LAMB3-related	0.995172838	1	1	0.999929399	0.001152978	0.001158571
MEFV	familial Mediterranean fever	0.793466971	1	1	0.995133851	0.001460055	0.001840096
BBS2	Bardet-Biedl syndrome, BBS2-related	0.53742438	1	1	0.994111084	0.000575891	0.001071575
ASPA	Canavan disease	1	1	1	1	0.003086157	0.003086157
BBS1	Bardet-Biedl syndrome, BBS1-related	1	1	1	1	0.00361361	0.00361361
ERCC6	ERCC6-related disorders	1	1	1	1	0.000528022	0.000528022
GLDC	GLDC-related glycine encephalopathy	1	1	1	1	0.000368077	0.000368077
HADHA	HADHA-related disorders	1	1	1	1	0.000218667	0.000218667
BCKDHB	maple syrup urine disease type 1B	0.894746554	1	1	0.998374977	0.000174262	0.000194761
CLN5	CLN5-related neuronal ceroid lipofuscinosis	0.720953934	1	1	0.997394017	0.000173427	0.000240552
DMD	dystrophinopathy (including Duchenne/Becker muscular dystrophy)	0.714298307	1	1	0.999923927	9.49E-05	0.000132897
FKTN	FKTN-related disorders	1	1	1	1	0.000234	0.000234
MMACHC	methylmalonic aciduria and homocystinuria, cblC type	1	1	1	1	0.003587506	0.003587506
POMGNT1	muscle-eye-brain disease	1	0.997417434	0.746558785	1	0.000794816	0.000593377
SGCB	beta-sarcoglycanopathy	1	1	1	1	0.000470113	0.000470113
SGCG	gamma-sarcoglycanopathy	1	1	1	1	0.000560066	0.000560066
RTEL1	RTEL1-related disorders	0.977281725	1	1	0.999942925	0.000687396	0.000703376
FKRP	FKRP-related disorders	0.930759691	1	1	0.99570104	0.002574923	0.002766475
BCKDHA	maple syrup urine disease type Ia	0.833354354	1	1	0.999544317	0.000237285	0.000284735
PCCB	PCCB-related propionic acidemia	0.764755606	1	1	0.99065064	0.000415537	0.000543359
GLA	Fabry disease	0.666684602	1	1	0.999073186	0.000116218	0.000174322
CLN3	CLN3-related neuronal ceroid lipofuscinosis	1	1	1	1	2.78E-05	2.78E-05
COL4A3	COL4A3-related Alport syndrome	1	0.999415021	0.939433897	1	0.00105575	0.000991807
CYP21A2	21-hydroxylase-deficient congenital adrenal hyperplasia	1		1		0.07575562	0.07575562
EVC2	EVC2-related Ellis-van Creveld syndrome	1	1	1	1	0.000272047	0.000272047
GBA	Gaucher disease	1		1		0.001212238	0.001212238
GNPTAB	GNPTAB-related disorders	1	1	1	1	0.001039033	0.001039033
HSD17B4	D-bifunctional protein deficiency	1	1	1	1	0.000625326	0.000625326
NAGLU	mucopolysaccharidosis type IIIB	1	1	1	1	0.000352069	0.000352069
NPHS2	nephrotic syndrome, NPHS2-related	1	1	1	1	0.000168857	0.000168857
SLC17A5	Salla disease	1	1	1	1	0.000468215	0.000468215
USH1C	USH1C-related disorders	1	1	1	1	0.000943822	0.000943822
ZFYVE26	spastic paraplegia type 15	1	1	1	1	0.000160038	0.000160038
IDUA	mucopolysaccharidosis type I	0.984134329	1	1	0.999734831	0.000688308	0.000699405
MMAB	methylmalonic acidemia, cblB type	0.962973453	1	1	0.99879812	0.000415934	0.000431927
PTS	6-pyruvoyl-tetrahydropterin synthase deficiency	0.909099368	1	1	0.964295515	0.000159913	0.000175903
COL4A4	COL4A4-related Alport syndrome	0.846169612	1	1	0.999887643	0.00017602	0.00020802
ALDH3A2	Sjogren-Larsson syndrome	1	1	1	1	6.72E-05	6.72E-05
BCS1L	GRACILE syndrome	1	1	1	1	0.00018961	0.00018961

CTNS	cystinosis	1	1	1	1	0.000183696	0.000183696
DLD	lipoamide dehydrogenase deficiency	1	1	1	1	0.000565885	0.000565885
EVC	EVC-related Ellis-van Creveld syndrome	1	1	1	1	4.80E-05	4.80E-05
GRHPR	primary hyperoxaluria type 2	1	1	1	1	0.000890896	0.000890896
HGSNAT	mucopolysaccharidosis type IIIC	1	1	1	1	0.000176054	0.000176054
HLCS	holocarboxylase synthetase deficiency	1	1	1	1	0.000144724	0.000144724
PEX10	peroxisome biogenesis disorder type 6	1	1	1	1	0.000160039	0.000160039
PEX6	peroxisome biogenesis disorder type 4	1	1	1	1	0.000128024	0.000128024
PEX7	rhizomelic chondrodysplasia punctata type 1	1	1	1	1	0.000372186	0.000372186
RS1	X-linked juvenile retinoschisis	1	1	1	1	2.96E-05	2.96E-05
SLC26A2	sulfate transporter-related osteochondrodysplasia	1	0.998025029	0.978171688	1	0.002802013	0.002740849
TH	Segawa syndrome	1	1	1	1	0.000366165	0.000366165
XPC	xeroderma pigmentosum group C	1	1	1	1	0.000336068	0.000336068
AGA	aspartylglycosaminuria	0.923080203	1	1	0.999841726	7.11E-05	7.71E-05
ADA	adenosine deaminase deficiency	0.894754952	1	1	0.998486045	0.000272054	0.000304055
CLN8	Northern epilepsy	0.555567232	0.999676122	0.909094008	0.99741486	6.16E-05	0.000100735
PCCA	PCCA-related propionic acidemia	0.545479714	1	1	0.999215449	9.59E-05	0.000175833
AMT	AMT-related glycine encephalopathy	1	0.996548434	0.741995311	1	0.000496093	0.000368099
BBS12	Bardet-Biedl syndrome, BBS12-related	1	1	1	1	0.000144022	0.000144022
CYP11B1	11-beta-hydroxylase-deficient congenital adrenal hyperplasia	1	1	1	1	9.67E-05	9.67E-05
DBT	maple syrup urine disease type II	1	1	1	1	0.000638252	0.000638252
ERCC8	ERCC8-related disorders	1	1	1	1	8.00E-05	8.00E-05
IKBKAP	familial dysautonomia	1	0.999907902	0.625004695	1	3.79E-05	2.37E-05
MAN2B1	alpha-mannosidosis	1	1	1	1	4.51E-05	4.51E-05
MCOLN1	mucolipidosis IV	1	1	1	1	0.000837137	0.000837137
MKS1	MKS1-related disorders	1	1	1	1	0.000591595	0.000591595
MMAA	methylmalonic acidemia, cblA type	1	1	1	1	4.80E-05	4.80E-05
MPI	congenital disorder of glycosylation type Ib	1	1	1	1	0.000263103	0.000263103
PCDH15	PCDH15-related disorders	1	1	1	1	3.09E-05	3.09E-05
PEX12	peroxisome biogenesis disorder type 3	1	1	1	1	0.000432055	0.000432055
SLC37A4	glycogen storage disease type Ib	1	0.999896573	0.973687697	1	0.000212304	0.000206718
TTPA	ataxia with vitamin E deficiency	1	1	1	1	8.41E-05	8.41E-05
HMGCL	HMG-CoA lyase deficiency	0.833342222	1	1	0.998852123	8.00E-05	9.60E-05
ABCD1	X-linked adrenoleukodystrophy	1	1	1	1	1.94E-05	1.94E-05
AGXT	primary hyperoxaluria type 1	1	1	1	1	0.000113408	0.000113408
ALDOB	hereditary fructose intolerance	1	1	1	1	2.26E-05	2.26E-05
ALG6	congenital disorder of glycosylation type Ic	1	1	1	1	6.40E-05	6.40E-05
ARG1	argininemia	1	1	1	1	6.40E-05	6.40E-05
CLN6	CLN6-related neuronal ceroid lipofuscinosis	1	0.999826395	0.666672882	1	4.79E-05	3.20E-05
COL4A5	X-linked Alport syndrome	1	1	1	1	1.94E-05	1.94E-05
CPT1A	carnitine palmitoyltransferase IA deficiency	1	1	1	1	0.000163323	0.000163323
GNPTG	mucolipidosis III gamma	1	1	1	1	3.20E-05	3.20E-05
IDS	mucopolysaccharidosis type II	1	1	1	1	3.87E-05	3.87E-05
IL2RG	X-linked severe combined immunodeficiency	1	1	1	1	1.94E-05	1.94E-05
KCNJ11	KCNJ11-related familial hyperinsulinism	1	1	1	1	1.60E-05	1.60E-05
LAMA3	Herlitz junctional epidermolysis bullosa, LAMA3-related	1	1	1	1	1.12E-05	1.12E-05
LIPA	lysosomal acid lipase deficiency	1	1	1	1	0.001748048	0.001748048
NPC2	Niemann-Pick disease type C2	1	1	1	1	0.000127745	0.000127745
PC	pyruvate carboxylase deficiency	1	1	1	1	4.80E-05	4.80E-05
PEX2	peroxisome biogenesis disorder type 5	1	1	1	1	0.000400062	0.000400062

PROP1	PROP1-related combined pituitary hormone deficiency	1	1	1	1	0.000642165	0.000642165
RMRP	cartilage-hair hypoplasia	1		1		7.81E-05	7.81E-05
SLC12A6	Andermann syndrome	1	1	1	1	5.93E-06	5.93E-06
ATP7A	ATP7A-related disorders		1		1	0	0
CLRN1	Usher syndrome type 3		1		1	0	0
CPS1	carbamoylphosphate synthetase I deficiency		1		1	0	0
CTSK	pycnodysostosis		1		1	0	0
GALK1	galactokinase deficiency		1		1	0	0
HOGA1	primary hyperoxaluria type 3		1		1	0	0
HYLS1	hydrolethalus syndrome		1		1	0	0
LAMC2	Herlitz junctional epidermolysis bullosa, LAMC2-related		1		1	0	0
LRPPRC	Leigh syndrome, French-Canadian type		1		1	0	0
MESP2	spondylothoracic dysostosis		1		1	0	0
MLC1	megalencephalic leukoencephalopathy with subcortical cysts		1		1	0	0
MTM1	X-linked myotubular myopathy		1		1	0	0
NR0B1	X-linked congenital adrenal hypoplasia		1		1	0	0
OPA3	Costeff optic atrophy syndrome		1		1	0	0
OTC	ornithine transcarbamylase deficiency		1		1	0	0
SGCD	delta-sarcoglycanopathy		1		1	0	0
STAR	lipoid congenital adrenal hyperplasia		1		1	0	0
TAT	tyrosinemia type II		1		1	0	0
TCIRG1	autosomal recessive osteopetrosis type 1		1		1	0	0
TMEM216	Joubert syndrome 2		1		1	0	0
XPA	xeroderma pigmentosum group A		1		1	0	0
