# Supplementary material for: Inter‐lab concordance of variant classifications establishes clinical validity of expanded carrier screening
Source: Clin Genet. 2019 Jul 1;96(3):236–45. doi: 10.1111/cge.13582 (PMC6852020; doi:10.1111/cge.13582)
Supplement: Supplementary file 1 — TABLE S1 Details of sensitivity, specificity, positive predictive value, and negative predictive value calculations [file CGE-96-236-s001.pdf]

Supplemental Table 1. Calculation of test performance. "TRUE" indicates that an individuals carrying a combination of variants of the given type was used in the numerator or denominator of the metric in questi

		Sensitivity		Specificity		PPV		NPV		MWH carrier	ClinVar carrier
	Complete description of variants	*Numerator*	*Denominator*	*Numerator*	*Denominator*	*Numerator*	*Denominator*	*Numerator*	*Denominator*		
1	TP	TRUE	TRUE			TRUE	TRUE			TRUE	TRUE
2	TN			TRUE	TRUE			TRUE	TRUE		
3	FP				TRUE		TRUE			TRUE	
4	FN		TRUE						TRUE		TRUE
5	TP & TN	TRUE	TRUE			TRUE	TRUE			TRUE	TRUE
6	TP & FP	TRUE	TRUE			TRUE	TRUE			TRUE	TRUE
7	TP & FN	TRUE	TRUE			TRUE	TRUE			TRUE	TRUE
8	TN & FP				TRUE		TRUE			TRUE	
9	TN & FN		TRUE						TRUE		TRUE
10	FP & FN		TRUE				TRUE			TRUE	TRUE
11	TP & TN & FP	TRUE	TRUE			TRUE	TRUE			TRUE	TRUE
12	TP & TN & FN	TRUE	TRUE			TRUE	TRUE			TRUE	TRUE
13	TP & FP & FN	TRUE	TRUE			TRUE	TRUE			TRUE	TRUE
14	TN & FP & FN		TRUE				TRUE			TRUE	TRUE
15	TP & TN & FP & FN	TRUE	TRUE			TRUE	TRUE			TRUE	TRUE

Implicit in the categorization is that individuals do not carry other types of variants.

TP: true positive
TN: true negative
FP: false positive
FN: false negative
